# XXIV World Allergy Congress 2015: Seoul, Korea. 14-17 October 2015

**DOI:** 10.1186/s40413-016-0096-1

**Published:** 2016-04-19

**Authors:** Heung-Man Lee, Il-Ho Park, Jae-Min Shin, Hyun-Sun Yoon, Gyeong Yul Park, Margit Zeher, Katsuhiko Matsui, Saki Tamai, Reiko Ikeda, Drsushil Suri, Dranu Suri, Marzieh Heidarzadeh Arani, Azwin Lubis, Anang Endaryanto, Shinichiro Koga, Lee Ju Suk, Yasunobu Tsuzuki, Seo Hyeong Kim, Jung U. Shin, Ji Yeon Noh, Shan Jin, Shan Jin, Hemin Lee, Jungsoo Lee, Chang Ook Park, Kwang Hoon Lee, Kwang Hoon Lee, Fatma Merve Tepetam, Chun Wook Park, Jee Hee Son, Soo Ick Cho, Yong Se Cho, Yun Sun Byun, Yoon Seok Yang, Bo Young Chung, Hye One Kim, Hee Jin Cho, Yoshinori Katada, Toshio Tanaka, Akihiko Nakabayashi, Koji Nishida, Kenichi Aoyagi, Yuki Tsukamoto, Kazushi Konma, Motoo Matsuura, Jung-Won Park, Yoshinori Harada, Kyoung Yong Jeong, Akiko Yura, Maiko Yoshimura, Tae-Suk Kyung, Young Hyo Kim, Chang-Shin Park, Tae Young Jang, Min-Jeong Heo, Ah-Yeoun Jung, Seung-Chan Yang, Hye One Kim, Yong Se Cho, Yun Sun Byun, Yoon Seok Yang, Bo Young Chung, Jee Hee Son, Chun Wook Park, Hee Jin Cho, Oliver Pfaar, Angelika Sager, Amit Agarwal, Meenu Singh, Bishnupda Chatterjee, Anil Chauhan, Ilja Striz, Eva Cecrdlova, Katerina Petrickova, Libor Kolesar, Alena Sekerkova, Veronika Svachova, Miroslav Petricek, Hyuck Hoon Kwon, Kyu Han Kim, Suman Kumar, Lou Ver Leigh Arciaga Manzon, Pilar Agnes Gonzalez Andaya, Bark-Lynn Lew, Youngjun Oh, Dongwoo Suh, Woo-Young Sim, Kyoung Yong Jeong, Myung-Hee Yi, Mina Son, Dongpyo Lyu, Jae-Hyun Lee, Tai-Soon Yong, Chein-Soo Hong, Jung-Won Park, Kyoung Yong Jeong, Myung-Hee Yi, Mina Son, Dongpyo Lyu, Jae-Hyun Lee, Tai-Soon Yong, Chein-Soo Hong, Jung-Won Park, Mohammadreza Siavashi, Hey Suk Yun, Ha-Na Kang, Jae-Won Oh, Young Jin Choi, Jae-Won Oh, Young Jin Choi, Ha-Na Kang, Tatiana Sentsova, Ilya Vorozhko, Olga Chernyak, Vera Revyakina, Anna Timopheeva, Andrey Donnikov, Tatiana Sentsova, Ilya Vorozhko, Olga Chernyak, Vera Revyakina, Anna Timopheeva, Ji Hyun Lee, Young Min Park, Sang Soo Choi, Kyung Do Han, Han Mi Jung, Young Hoon Youn, Jun Young Lee, Yong Gyu Park, Seung-Hwan Lee, Jing Li, Mulin Feng, Marjut Roponen, Bianca Schaub, Gary W. K. Wong, Zhaowei Yang, Young Jin Choi, Ha-Na Kang, Jae-Won Oh, Baoqing Sun, Peiyan Zheng, Yoon-Sung Park, Sang Wook Son, Sukran Kose, Kemal Kiraz, Arzu Didem Yalcin, Sehyo Yune, Jae-Won Paeng, Mi-Jung Oh, Byung-Jae Lee, Dong-Chull Choi, Young Hee Lim, Kyoung Won Ha, Jin-Young Lee, Kiwako Yamamoto-Hanada, Masami Narita, Masaki Futamura, Yukihiro Ohya, Jihyun Kim, Jinwha Choi, Kwanghoon Kim, Jaehee Choi, Kangmo Ahn, Sun-Ho/Brian Chang, Lisha Li, Yu-Ichi Adachi, Kumiko Tsuji Kanatani, Shigeyuki Narabayashi, Ikuo Okafuji, Yuya Tanaka, Satoru Tsuruta, Nobue Takamatsu, Soo Whan Kim, Do Hyun Kim, Jong-Seo Yoon, Jin Tack Kim, Hwan Soo Kim, Yoon Hong Chun, Hyun Hee Kim, Sul Mui Won, Kam Lun E. Hon, Chung Mo Chow, Ting Fan Leung, Do Hyun Kim, Soo Whan Kim, Gemmalyn Esguerra, Emily Resurreccion, Kristine Elisa Kionisala, Jenni Rose Dela Cruz, Muhammad Imran, Yun Seon Choe, Kyu Han Kim, Mira Choi, Byung Soo Kim, Hyun-Joo Lee, Jeong-Min Kim, Jeong-Min Kim, Gun-Wook Kim, Je-Ho Mun, Je-Ho Mun, Hoon-Soo Kim, Margaret Song, Hyun-Chang Ko, Hyun-Chang Ko, Moon-Bum Kim, Sun-Young Yoon, Amit Kandhare, Noriko Yahiro, Amit Agarwal, Meenu Singh, Jasleen Kaur, Ruby Pawankar, Pankaj Pant, Sukhmanjeet Singh, Hwan Soo Kim, Jong-Seo Yoon, Sul Mui Won, Yoon Hong Chun, Jin Tack Kim, Hyun Hee Kim, Hwan Soo Kim, Sul Mui Won, Yoon Hong Chun, Jong-Seo Yoon, Hyun Hee Kim, Jin Tack Kim, Thatchai Kampitak, So Min Kim, Hyun Joo Lee, Hei Sung Kim, Jeong Deuk Lee, Sang Hyun Cho, Kiran Godse, Juwita Soekarno, Sarie Ratnasari, E. Alwi Datau, Eko Surachmanto, JC Matheos, Ting Fan Leung, Jamie Sui-Lam Kwok, Christine Kit-Ching Tung, Man Fung Tang, Stephen Kwok-Wing Tsui, Gary WK Wong, Kam Lun Ellis Hon, Wing Hung Tam, Hing Yee Sy, Sohee Lee, Hyun-Woo Shin, Mingyu Lee, Dae Woo Kim, Roza Khalmuratova, Mi Yeoung Kim, Jaewon Jeong, Chansun Park, Christine Yee Yan Wai, Patrick S. C. Leung, Nicki Y. H. Leung, Ka Hou Chu, Hee Seon Lee, Kyung Eun Lee, Jung Yeon Hong, Mi Na Kim, Min Jung Kim, Yoon Hee Kim, In Suk Sol, Seo Hee Yoon, Kyung Won Kim, Myung Hyun Sohn, Kyu-Earn Kim, Ji Hye Kim, Hae-Sim Park, Yoo Seob Shin, Young Min Ye, Daehong Seo, Moon Gyeong Yoon, Young Mok Lee, Daehong Seo, Ji Hye Kim, Young-Mok Lee, Young Min Ye, Hae-Sim Park, Ga Young Ban, Kumsun Cho, Seung-Hyun Kim, Yong Eun Kwon, Moon Gyeong Yoon, Ji Hye Kim, Yoo Seob Shin, Young Min Ye, Dong-Ho Nahm, Hae-Sim Park, Pilar Agnes Gonzalez Andaya, Pilar Agnes Gonzalez Andaya, Ga Young Ban, Chang Gyu Jung, Seung-Ihm Lee, Duy Le Pham, Dong-Hyeon Suh, Eun-Mi Yang, Young Min Ye, Yoo Seob Shin, Hae-Sim Park, Wan Jun Wang, MO Xian, Yan Qing Xie, Jing Ping Zheng, Jing Li, Emma Merike Savilahti, Outi Mäkitie, Anna Kaarina Kukkonen, Sture Andersson, Heli Viljakainen, Erkki Savilahti, Mikael Kuitunen, Kiran Godse, Duy Le Pham, Ga Young Ban, Seung-Hyun Kim, Eun-Mi Yang, Hae-Sim Park, Ji-Ho Lee, Yong-Joon Chwae, Li-Ming Chin, Chi-Chang Shieh, Ji Hye Kim, Hye-Soo Yoo, Moon Gyeong Yoon, Ga Young Ban, Ga Young Ban, Yoo Seob Shin, Young Min Ye, Hae-Sim Park, Myong Soon Sung, Jin Uck Choi, Sung Won Kim, Yong Jin Hwang, Arum Park, Eun Lee, Song-I Yang, Hyun-Ju Cho, Jinho Yu, Dong Hun Lee, Eun Ju Kim, Yeon Kyung Kim, Eun Jin Doh, Hee Chul Eun, Jin Ho Chung, Young Mee Lee, Seon Pil Jin, Xingnan Li, Naftali Kaminski, Sally Wenzel, Eugene Bleecker, Deborah Meyers, Zeng Huasong, Ji-Hun Mo, Ramachandran Samivel, Eun-Hee Kim, Ji-Hye Kim, Jun-Sang Bae, Young-Jun Chung, Dae Woo Kim, Eun Lee, Si Hyeon Lee, Young-Ho Kim, Hyun-Ju Cho, Ho-Sung Yu, Mi-Jin Kang, Song-I Yang, Young-Ho Jung, Hyung Young Kim, Ju-Hee Seo, Byoung-Ju Kim, Hyo-Bin Kim, So-Yeon Lee, Ho-Jang Kwon, Soo-Jong Hong, Sailesh Palikhe, Hae-Sim Park, Seung-Hyun Kim, Ji Hye Kim, Eun-Mi Yang, Suda Sibunruang, Jettanong Klaewsongkram, Tatiana Sentsova, Ilya Vorozhko, Anna Timopheeva, Olga Chernyak, Vera Revyakina, Andrey Sokolnikov, Makoto Nisihino, Yu Okada, Noriyuki Yanagida, Motohiro Ebisawa, Sakura Sato, Kiyotake Ogura, Tomoyuki Asaumi, Kenichi Nagakura, Tetsuharu Manabe, Hirotoshi Unno, Pedro M. Rodrigues, Denise Schrama, Gadija Mohamed, Lizex Hüsselmann, Lizex Hüsselmann, Bongani Ndimba, Jae-Uoong Shim, Young Il Koh, Joon Haeng Rhee, Ji-Ung Jeong, Dinh Van Nguyen, Hieu Chi Chu, Mui Thi Tran, Christopher Vidal, Suran Fernando, Sheryl Van Nunen, Sy Van Than, Mulin Feng, Jing Li, Dong-Kyu Kim, Seung-No Hong, Kyoung Mi Eun, Hong Ryul Jin, Dae Woo Kim, Jun Bao, Yi-Xiao Bao, Sanjoy Podder, Goutam Kumar, Shampa Dutta, Amlan Ghosh, Goutam Kumar Saha, Sanjoy Podder, Salil Kumar Gupta, Tu/Hoang Kim Trinh, Yoo Seob Shin, Hae-Sim Park, Jing-Nan Liu, Duy Le Pham, Hyun-Chang Ko, Byung Soo Kim, Moon-Bum Kim, Ömer Özbudak, Fatih Üzer, Mona Al-Ahmad, Maryam Alowayesh, Norman Carroll, Anand Bahadur Singh, Yordanis Pérez-Llano, María Del Carmen Luzardo Lorenzo, Wendy Ramírez González, Carlos Calcines Cruz, Rady Laborde Quintana, Alain Morejón, Virgilio Bourg, Marilé Hechavarría Stoker, Jun-Sang Bae, Ramachandran Samivel, Eun-Hee Kim, Ji-Hye Kim, Ji-Hun Mo, Keiko Hanaoka, Michihiro Hide, Akio Tanaka, Makiko Hiragun, Mikio Kawai, Kwanghoon Kim, Hye-Young Kim, Jihyun Kim, Kangmo Ahn, Youngshin Han, Gun-Wook Kim, Hyun-Chang Ko, Byung Soo Kim, Moon-Bum Kim, Margaret Song, Takeshi Matsubara, Hiroshi Iwamoto, Yuki Nakazato, Kazuyoshi Namba, Yasuhiro Takeda, Jae Hoon Lee, Woo Yong Bae, Els De Schryver, Lien Calus, Philippe Gevaert, Thibaut Van Zele, Claus Bachert, Akio Mori, Satoshi Kouyama, Miyako Yamaguchi, Yo Iijima, Akemi Abe-Ohtomo, Hiroaki Hayashi, Kentaroh Watai, Chihiro Mitsui, Chiyako Oshikata, Kiyoshi Sekiya, Takahiro Tsuburai, Mamoru Ohtomo, Yuma Fukutomi, Masami Taniguchi, Ju Wan Kang, Jeong Hong Kim, Jeong Hong Kim, Keun-Hwa Lee, Hye-Sook Lee, Seong-Chul Hong, Jaechun Lee, Ji Won Seo, Jae Hoon Lee, Woo Yong Bae, Ivans Sergejs Kuznecovs, Galina Kuznecova, Karl-Christian Bergmann, Torsten Zuberbier, Joseph Salame, Torsten Sehlinger, Georg Bölke, Yoo Suk Kim, Jung Hyun Chang, Jeong Hong Kim, Ju Wan Kang, Seung-No Hong, Doo Hee Han, Chae-Seo Rhee, Young-Joo Ko, Young Hyo Kim, Dae-Young Kim, Tae Young Jang, Tomoharu Yokooji, Taiki Hirano, Hiroaki Matsuo, Galina Kuznecova, Ivans Sergejs Kuznecovs, Roohi Rasool Wani, Shafia Alam Syed, Ghulam Hassan, Ayaz Gul, Saniya Nissar, Zaffar Amin Shah, Marilyn Urrutia Pereira, Carmen Fernandez, Dirceu Sole, Herberto Jose Chong Neto, Veronica Acosta, Alfonso Mario Cepeda, Mirta Alvarez Castello, Claudia Almendarez, Jose Santos Lozano Saenz, Juan C. Sisul, Nelson Rosario Filho, Antonio Castillo, Marylin Valentin Rostan, Jennifer Avila, Hector Badellino, Maria Carolina Manotas, Raúl Lázaro Castro Almarales, Mayda González León, Woo Kyung Kim, Hae-Sun Yoon, Takehito Kobayashi, Tooru Noguchi, Tomoyuki Soma, Kazuyuki Nakagome, Hidetomo Nakamoto, Hirohito Kita, Makoto Nagata, Marilyn Urrutia Pereira, Dirceu Sole, Herberto Jose Chong Neto, Alfonso Mario Cepeda, Raúl Lázaro Castro Almarales, Juan C. Sisul, Marylin Valentin Rostan, Hector Badellino, Miguel Alejandro Medina Avalos, Antonio Castillo, Claudia Almendarez, Nelson Rosario Filho, Caridad Sanchez Silot, Jennifer Avila, Felicia Berroa Rodriguez, Jose Santos Lozano Saenz, Mirta Alvarez Castello, Carmen Fernandez, Wai Tuck Soh, Alain Jacquet, Kiat Ruxrungtham, Emmanuel Nony, Maxime Le Mignon, Mary Lee-Wong, Suzanne McClelland, Suzanne McClelland, Nanette B. Silverberg, Christian E. Song, Zhaowei Yang, Jiukai Zhang, Wentao Zheng, Nanshan Zhong, Jing Li, Jung Ho Bae, Young Joo Cho, Joo Yeon Kim, Dimitrios Vourdas, Christos Grigoreas, Konstantinos Petalas, Yuan Zhang, Seung-Heon Shin, Mi-Kyung Ye, Jeong-Kyu Kim, Yong Feng, Yunxiao Shang, Sungchul Seo, Ji Tae Choung, Dohyeong Kim, Young Yoo, Hyunwook Lim, Wenjing Zhu, Chuanhe Liu, Li Sha, Li Chang, Min Zhao, Linqing Zhao, Yuan Qian, Yuzhi Chen, Min-Hye Kim, Young Joo Cho, Mina Rho, Jung-Won Kim, Yeon-Mi Kang, Kyung-Eun Yum, Hyeon-Il Choi, Jun-Pyo Choi, Han-Ki Park, Taek-Ki Min, Bok-Yang Pyun, Yoon-Keun Kim, Xueyan Wang, Woo Kyung Kim, Yu Ran Nam, Joo Hyun Nam, Jung-Won Kim, Min-Hye Kim, Mina Rho, Yeon-Mi Kang, Kyung-Eun Yum, Hyeon-Il Choi, Jun-Pyo Choi, Han-Ki Park, Taek-Ki Min, Young Joo Cho, Bok-Yang Pyun, Yoon-Keun Kim, Hiroshi Narita, Junko Hirose, Kumiko Kizu, Ayu Matsunaga, Jingyun Li, Yuan Zhang, Luo Zhang, Yean Jung Choi, Hye Lim Shin, Song-I Yang, So-Yeon Lee, Sung-Ok Kwon, Young-Ho Jung, Ji-Won Kwon, Hyung Young Kim, Ju-Hee Seo, Byoung-Ju Kim, Hyo-Bin Kim, Se-Young Oh, Ho-Jang Kwon, Eun Lee, Mi-Jin Kang, Soo-Jong Hong, Yun-Jeong Lee, Joonil Kim, Joon Young Choi, Ji Young Kang, Seok Chan Kim, Sei Won Kim, Seung Joon Kim, Young Kyoon Kim, Chin Kook Rhee, Hea Yon Lee, Hwa Young Lee, Sook Young Lee, Tae Kyung Koh, Sung Wan Kim, Kun Hee Lee, Chul Kwon, Joong-Saeng Jo, Sung-Hwa Dong, Young Seok Byun, Charles Song, Ji Young Kang, Hwa Young Lee, In Kyoung Kim, Sei Won Kim, Chin Kook Rhee, Seung Joon Kim, Seok Chan Kim, Sook Young Lee, Young Kyoon Kim, Soon Seog Kwon, Joon Young Choi, Pona Park, Hong Ryul Jin, Dong-Kyu Kim, Dae Woo Kim, Astrid Hogenkamp, Leon Knippels, Betty C.a.m. Van Esch, Jolanda Van Bilsen, Prescilla V. Jeurink, Marjan Gros, Johan Garssen, Joost J. Smit, Raymond H. H. Pieters, Boo-Young Kim, Soo Whan Kim, Ramon Lleonart, Christophe Lay, Kaouther Benamor, Chua Mei Chen, Jan Knol, Charmaine Chew, Voranush Chongsrisawat, Anne Goh, Wen Chin Chiang, Rajeshwar Rao, Surasith Chaithongwongwatthana, Nipon Khemapech, Ji Young Yhi, Sang-Heon Kim, Dong Won Park, Ji-Yong Moon, Tae Hyung Kim, Jang Won Sohn, Dong Ho Shin, Ho Joo Yoon, Seok Hyun Cho, Sang-Heon Kim, Ji-Yong Moon, Jae-Hyun Lee, Ga Young Ban, Sujeong Kim, Mi-Ae Kim, Joo-Hee Kim, Min-Hye Kim, Chan-Sun Park, Hyouk-Soo Kwon, Jae-Woo Kwon, Jae Woo Jung, Hye-Ryun Kang, Jong-Sook Park, Tae-Bum Kim, Heung Woo Park, You Sook Cho, Kwang-Ha Yoo, Yeon-Mok Oh, Sang-Rok Lee, Kaja Julge, Maire Vasar, Maire Vasar, Tiia Voor, Tiina Rebane, Yu Jin Kim, Sang Min Lee, Shin Myung Kang, Sojeong Kim, Sun Young Kyung, Sung Hwan Jeong, Jeong-Woong Park, Hyunjung Hwang, Yong Han Seon, Sanghui Park, Sang Pyo Lee, Marius Iordache, Yeongsang Jeong, Sohee Eun, Byung Min Choi, Ji Tae Choung, Wonhee Seo, Liang Zhang, Ruby Pawankar, Manabu Nonaka, Miyuki Hayashi, Shingo Yamanishi, Harumi Suzaki, Yasuhiko Itoh, So Watanabe, Hitome Kobayashi, Zsuzsanna Zotter, Henriette Farkas, Lilian Varga, Nora Veszeli, Eva Imreh, Gabor Kovacs, Marsel Nallbani, Semen Zheleznov, Galina Urzhumtseva, Natalia Petrova, Zhanna Sarsaniia, Nikolai Didkovskii, Torsten Zuberbier, Nader Dashti Gerdabi, Ali Khodadadi, Zahra Abdoli, Mehri Ghafourian, Mohammad Ali Assarehzadegan, Khodayar Ghorban, Hyo-Bin Kim, Hui Zhou, Jeong Hee Kim, Rima Habre, Theresa Bastain, Frank Gilliland, Jong-Wook Bae, Kyu-Hyung Han, Young-Koo Jee, Misoo Choi, Seung-Phil Hong, Seung-Hyun Kim, Hee-Kyoo Kim, Gil-Soon Choi, Jeonghoon Heo, Young-Ho Kim, Eun-Kee Park, Takashi Inoue, Kiyotake Ogura, Noriyuki Yanagida, Hirotoshi Unno, Kenichi Nagakura, Tetsuharu Manabe, Tomoyuki Asaumi, Sakura Sato, Yu Okada, Motohiro Ebisawa, Min-Gu Kim, You Sook Cho, Tae-Bum Kim, Hee-Bom Moon, Jung-Hyun Kim, Hyo-Jung Kim, So-Young Park, Bomi Seo, Hyouk-Soo Kwon, Jaemoon Lee, Taehoon Lee, Hye-Soo Yoo, Jieun Kim, Inok Kim, Haejin Kim, Younhee Chang, Hae-Sim Park, Sooyoung Lee, Sooyoung Lee, Kazuyo Kuzume, Munemitsu Koizumi, Koji Nishimura, Michiko Okamoto, Seung-Hyun Kim, Young Min Ye, Gyu Young Hur, Hae-Sim Park, Sang-Heon Kim, Young-Koo Jee, Seung-Hyun Kim, Hyunna Choi, Young Min Ye, Hae-Sim Park, Bui Van Khanh, Hieu Chi Chu, Nguyen Nhu Nguyet, Nguyen Hoang Phuong, Joo Young Roh, Hyun Jeong Kim, Jung Eun Kim, Bark-Lynn Lew, Kyung Ho Lee, Seung-Phil Hong, Yong Hyun Jang, Kui Young Park, Seong Jun Seo, Jung Min Bae, Eung Ho Choi, Ki Beom Suhr, Seung Chul Lee, Hyun-Chang Ko, Young Lip Park, Sang Wook Son, Young Jun Seo, Yang Won Lee, Sang Hyun Cho, Chun Wook Park, Kun Hee Lee, Sung Wan Kim, Chia-Yu Chu, Derrick Aw, Young-Min Ye, Giovanni Bader, Fabrizio Dolfi, Nathalie Oliveira, Jae Chol Choi, Jae Woo Jung, Hye-Ryun Kang, Kijeong Kim, Byoung Whui Choi, Jong Wook Shin, Jae Woo Jung, Jae Chol Choi, In Won Park, Byoung Whui Choi, Jae Yeol Kim, Jin-Young Lee, Kyoung Won Ha, Yun-Jin Jeung, Sehyo Yune, Byung-Jae Lee, Dong-Chull Choi, Mi-Jung Oh, Young Hee Lim, Eui Jun Lee, Dongin Suh, Dongin Suh, Sung-Il Woo, Sung-Il Woo, Hwa Jin Cho, Hwa Jin Cho, Eun Hee Chung, Eun Hee Chung, Soo Youn Chung, Ashok Shah, Kamal Gera, Kyoung Won Ha, Mi-Jung Oh, Young Hee Lim, Sehyo Yune, Jae-Won Paeng, Mi-Jin Jang, Byung-Jae Lee, Dong-Chull Choi, Jin-Young Lee, Mi-Jin Jang, Jae-Won Paeng, Yun-Jin Jeung, Young Hee Lim, Mi-Jung Oh, Kyoung Won Ha, Byung-Jae Lee, Dong-Chull Choi, Sehyo Yune, Jin-Young Lee, Takahiro Ito, Jin-Young Lee, Sehyo Yune, Byung-Jae Lee, Dong-Chull Choi, Mi-Jin Jang, Jae-Won Paeng, Young Eun Kim, Young Nam Kim, Yongseok Lee, Jihye Kim, Hwa Young Lee, Sook Young Lee, Soon Seog Kwon, Young Kyoon Kim, Chin Kook Rhee, Sei Won Kim, Hea Yon Lee, Joon Young Choi, In Kyoung Kim, Maria Ascension Aranzabal, Alejandro Joral, Susana Lizarza, Miguel Echenagusia, Eva Maria Lasa, Jose Antonio Navarro, Eun-Jung Jo, Sun-Mi Jang, Seung-Eon Song, Hae-Jung Na, Chang-Hoon Kim, Woo-Seop Lee, Hye-Kyung Park, Sachiko Miyauchi, Yoshitaka Uchida, Tomoyuki Soma, Susumu Yamazaki, Toru Noguchi, Takehito Kobayashi, Kazuyuki Nakagome, Makoto Nagata, Hea Lin Oh, Do Kyun Kim, Dongin Suh, Young Yull Koh, Jin-Young Lee, Byung-Jae Lee, Dong-Chull Choi, Jae-Won Paeng, Mi-Jin Jang, Jihye Kim, Young Nam Kim, Sehyo Yune, Sang-Hoon Kim, Jang Won Sohn, Ho Joo Yoon, Dong Ho Shin, Jae Hyung Lee, Byoung Hoon Lee, Youn-Seup Kim, Jae-Seuk Park, Young-Koo Jee, Sang-Heon Kim, Jin-Young Lee, Jae-Won Paeng, Mi-Jin Jang, Dong-Chull Choi, Byung-Jae Lee, Yongseok Lee, Young Eun Kim, Sehyo Yune, Jisun Yoon, Li Zhang, Xuxu Cai, Yong Feng, Jong-Seo Yoon, Kyunguk Jeong, Hye-Soo Yoo, Sooyoung Lee, Sooyoung Lee, Hae-Jin Lee, Noo Ri Lee, Bo-Kyung Kim, Minyoung Jung, Dong Hye Kim, Catharina S. Moniaga, Kenji Kabashima, Eung Ho Choi, Noriyuki Yanagida, Sakura Sato, Chizuko Sugizaki, Motohiro Ebisawa, Jamie Kiehm, Punita Ponda, Sherry Farzan, Jared Weiss, Claudia Elera, Catherine Destio, Cristina Sison, Annette Lee, Soo Hyun Ri, Chang Hoon Lim, Iñaki Izquierdo Pulido, Jorg Taubel, Georg Ferber, Eva Santamaria Masdeu, Alireza Khayatzadeh, Masoud Movahedi, Motohiro Ebisawa, Mohammad Gharagozlou, Ulus Atasoy, Patsharaporn Techasintana, Matt Gubin, Jacqueline Glascock, Suzanne Ridenhour, Joseph Magee, Nelson Rosario Filho, Herberto Jose Chong Neto, Gustavo Falbo Wandalsen, Ana Caroline Dela Bianca, Carolina Aranda, Dirceu Sole, Javier Mallol, Luis Garcia-Marcos, Luis Garcia-Marcos, Jennifer Toh, Yoomie Lee, Joyce Huang, Elina Jerschow, Jenny Shliozberg, Pattraporn Satitsuksanoa, Narissara Suratannon, Jongkonnee Wongpiyabovorn, Pantipa Chatchatee, Kiat Ruxrungtham, Alain Jacquet, Min Suk Yang, Jin Yong Lee, Ja Yeun Kim, Han-Ki Park, Ju-Young Kim, Woo-Jung Song, Hye-Ryun Kang, Heung Woo Park, Yoon-Seok Chang, Sang-Heon Cho, Kyung-up Min, Chang-Han Park, Suk-Il Chang, Sook-Hee Song, Si-Heon Kim, Gil-Soon Choi, Su-Chin Kim, Ji Hye Kim, Ga Young Ban, Yoo Seob Shin, Hae-Sim Park, Young Min Ye, Yoon Ha Hwang, Da Woon Sim, Kyung Hee Park, Kyung Hee Park, Hye Jung Park, Hye Jung Park, Jung-Won Park, Jung-Won Park, Jae-Hyun Lee, Jae-Hyun Lee, Katrina Allen, Cara Beck, Jennifer Koplin, Melanie Matheson, Mimi Tang, Anne-Louise Ponsonby, Lyle Gurrin, Shyamali Dharmage, Melissa Wake, Vicki Mcwilliam, Xiaoying Liu, Jing Wang, Li Xiang, Qun Wang, Ji-Eun Lee, Dong-Young Kim, Chae-Seo Rhee, Chae-Seo Rhee, Francisco Vazquez-Nava, Sang-Heon Cho, Jaewon Jeong, Diahn-Warng Perng, David Price, Glenn Neira, Jiangtao Lin, Olga Semernik, Ha-Su Kim, Jin-a Jung, Ji-in Jung, Qing Miao, Li Xiang, Sang-Heon Cho, Jaewon Jeong, Diahn-Warng Perng, Jiangtao Lin, David Price, Glenn Neira, Hyun Young Lee, Hae-Sim Park, Young Min Ye, Su Chin Kim, Shokrollah Farrokhi, Mohammadkazem Gheiby, Sang-Heon Kim, Heung Woo Park, Sang-Hoon Kim, Young-Koo Jee, Sunjoo Park, Keun Ai Moon, Hyouk-Soo Kwon, Tae-Bum Kim, You Sook Cho, Hee-Bom Moon, Kyoung Young Lee, Gyong Hwa Hong, Eun Hee Ha, Heejae Han, Hye Jung Park, Yoon Hee Park, Yoon-Jo Kim, Kangtaek Lee, Jung-Won Park, Jae-Hyun Lee, Tatiana Slavyanskaya, Vladislava Derkach, Hye Jung Park, Chein-Soo Hong, Jae-Hyun Lee, Sungryeol Kim, Kyung Hee Park, Choong-Kun Lee, Beodeul Kang, Seung-Hoon Beom, Sang Joon Shin, Minku Jung, Jung-Won Park, Eun-Hee Kim, Ji-Hye Kim, Ji-Hun Mo, Young-Jun Chung, Mi-Ae Kim, Hae-Sim Park, Moon Gyeong Yoon, Young-Soo Lee, Ji Hye Kim, Ga Young Ban, Hye-Soo Yoo, Yoo Seob Shin, Young Min Ye, Dong-Ho Nahm, Qing Miao, Li Xiang, Ying-Ji Li, Takako Shimizu, Hirofumi Inagaki, Yukiyo Hirata, Hajime Takizawa, Arata Azuma, Masayuki Yamamoto, Tomoyuki Kawada, Min-Gu Kim, Gyong Hwa Hong, Kyoung Young Lee, Eun Hee Ha, Keun Ai Moon, Sunjoo Park, Hyouk-Soo Kwon, Tae-Bum Kim, Hee-Bom Moon, You Sook Cho, Jung-Hyun Kim, Hyo-Jung Kim, So-Young Park, Bomi Seo, Ji-Hye Kim, Ramachandran Samivel, Eun-Hee Kim, Young-Jun Chung, Ji-Hun Mo, M. S. Soumya, G. Inbaraj, R. Chellaa, Ruby Pawankar, Wonsun Choi, Hae-Sim Park, Young Min Ye, Ji Hye Kim, Ga Young Ban, Yoo-Seob Shin, Saskia Braber, Kim Verheijden, Aletta Kraneveld, Johan Garssen, Gert Folkerts, Linette Willemsen, Stephanie Eichhorn, Fatima Ferreira, Isabel Pablos, Bianca Kastner, Bettina Schweidler, Sabrina Wildner, Peter Briza, Jung-Won Park, Naveen Arora, Stefan Vieths, Gabriele Gadermaier, Sung-Min Park, Won-Ku Lee, Jeong-Min Kim, Gun-Wook Kim, Je-Ho Mun, Hoon-Soo Kim, Margaret Song, Hyun-Chang Ko, Moon-Bum Kim, Byung Soo Kim, Young Nam Kim, Sehyo Yune, Jin-Young Lee, Jihye Kim, Young Eun Kim, Jae-Won Paeng, Mi-Jin Jang, Dong-Chull Choi, Byung-Jae Lee, Yongseok Lee, Si Hui Goh, Bee Wah Lee, Jian Yi Soh, Hyungoo Kang, Hyunhee Kim, Hye-Yung Yum, Young Min Ye, Hae-Sim Park, Ga-Young Ban, Ji Hye Kim, Yoo Seob Shin, Takumi Takizawa, Masahiko Tabata, Akira Aizawa, Hisako Yagi, Yutaka Nishida, Hirokazu Arakawa, Akihiro Morikawa, Solongo Orosoo, Saskia Braber, Marianne Bol-Schoenmakers, Peyman Akbari, Prescilla V. Jeurink, Prescilla V. Jeurink, Priscilla De Graaff, Joost J. Smit, Betty C. A. M. Van Esch, Johan Garssen, Johan Garssen, Johanna Fink-Gremmels, Raymond H. H. Pieters, Gun-Woo Kim, Eun-Jung Jo, Sujeong Kim, Woo-Jung Song, Yoon-Seok Chang, Shoaib Faruqi, Ju-Young Kim, Mingyu Kang, Min-Hye Kim, Jana Plevkova, Heung Woo Park, Sang-Heon Cho, Alyn Morice, So-Hee Lee, Sun-Sin Kim, Seoung-Eun Lee, Bilun Gemicioglu, Zeynep Misirligil, Arif Hikmet Cimrin, Hakan Gunen, Tevfik Ozlu, Aykut Cilli, Levent Akyildiz, Hasan Bayram, Esra Uzaslan, Oznur Abadoglu, Mecit Suerdem, Keigo Kainuma, Hyun-a Kim, Ha-Su Kim, Woo Yong Bae, Jin-a Jung, Rose Kamenwa, William Macharia, Nusrat Said, Vidya Nerurkar, Meenal Patel, Simi Bhatia, Inseon S. Choi, Soo-Jeong Kim, Joo-Min Won, Myeong-Soo Park, Mizuho Nagao, Dong Won Park, Jang Won Sohn, Ji Young Yhi, Ji-Yong Moon, Sang-Heon Kim, Tae Hyung Kim, Dong Ho Shin, Ho Joo Yoon, Yukiyoshi Hyo, Jaechun Lee, Su Hee Kim, Eunkyoung Lee, Hahn Jin Jung, Jaehyun Lim, Seung-No Hong, Doo Hee Han, Chae-Seo Rhee, Kun Song Lee, Jaechun Lee, Sun Young Yang, Mi Young Ahn, Jong Hoo Lee, Jasmina Golez, Hui-Qin Tian, Lei Cheng, Xin-Yuan Chen, Ji-Yong Moon, Sang-Heon Kim, Tae Hyung Kim, Ji Young Yhi, Ho Joo Yoon, Jang Won Sohn, Dong Ho Shin, Dong Won Park, Won Im Cho, Jong Sub Choi, Dongin Suh, Gyeong Hoon Kang, Jin Soo Moon, Jae Sung Ko, Kyung Jae Lee, Shin Jie Choi, Wenting Luo, Baoqing Sun, Qi Gao, Li Xiang, Kunling Shen, Yong Hyun Jang, Karl-Christian Bergmann, Torsten Sehlinger, Georg Bölke, Uwe Berger, Torsten Zuberbier, Joanna Kolodziejczyk, Milena Wojciechowska, Anna Hnatyszyn-Dzikowska, Micha Chojnacki, Zbigniew Bartuzi, Katsunori Masaki, Koichi Fukunaga, Takashi Kamatani, Kengo Ohtsuka, Takae Tanosaki, Masako Matsusaka, Takao Mochimaru, Hiroki Kabata, Soichiro Ueda, Yusuke Suzuki, Katsuhiko Kamei, Koichiro Asano, Tomoko Betsuyaku, Karlee Trafford, Ismet Bulut, Zeynep Ferhan Ozseker, Kenta Horimukai, Hideaki Morita, Masami Narita, Hironori Niizeki, Kenji Matsumoto, Yukihiro Ohya, Hirohisa Saito, Shigenori Kabashima, Mai Kondo, Eisuke Inoue, Robert Siebers, Francis F. S. Wu, Robert Siebers, Francis F. S. Wu, Ming-Hui Ting, Hung-En Laio, Tsung-Huai Kuo, Pei-Yuan Lee, Daniel Eugene Maddox, Gwanghui Ryu, Hyo Yeol Kim, Hun-Jong Dhong, Sang Duk Hong, Seung-Kyu Chung, Osamu Higuchi, Yu-Ichi Adachi, Toshiko Itazawa, Yoko Adachi, Miki Hamamichi, Motokazu Nakabayashi, Yasunori Ito, Takuya Wada, Gyoukei Murakami, Miki Takao, Junko Yamamoto, Hyun Jung Jin, Moon Gyeong Yoon, Young Min Ye, Yoo-Seob Shin, Seung-Hyun Kim, Hae-Sim Park, Taek-Ki Min, Bok-Yang Pyun, So-Yeon Lee, Hyun Hee Kim, Gwang-Cheon Jang, Jinho Yu, Dongin Suh, Sooyoung Lee, Yong Mean Park, Jeong Hee Kim, Hye-Yung Yum, Kyung Won Kim, Hyeon-Jong Yang, Kangmo Ahn, Ji-Won Kwon, Myung Hyun Sohn, Hae Ran Lee, Jung Hyun Kwon, Kyu-Earn Kim, Soo-Jong Hong, Su-Mi Cho, Dong-Ho Nahm, Myoung-Eun Kim, Jin Hwa Lee, Chin Kook Rhee, Hye Yun Park, Woo Jin Kim, Yong Bum Park, Kwang-Ha Yoo, Heejeong Kang, Hyeon-Jong Yang, Taek-Ki Min, Bok-Yang Pyun, Lee Ju Suk, Cheol Hong Kim, Jung Hyun Kwon, Sang Hyun Lee, Wonhee Seo, Kang-in Kim, Young Cheon Park, Hyeon-Jong Yang, Taek-Ki Min, Bok-Yang Pyun, Sujeong Kim, Sun Jin, Jong-Myung Lee, Hye-Jin Jung, Jung-Wha Park, Hyo-Jung Kim, Tae-Bum Kim, You Sook Cho, Hee-Bom Moon, Hyouk-Soo Kwon, So Young Park, So-Young Park, Jung-Hyun Kim, Bomi Seo, Min-Gu Kim, Youn Yee Kim, Yena Lee, Taek-Ki Min, Hyeon-Jong Yang, Bok-Yang Pyun, Suk Hee Han, Suyeon Park, Jeongho Lee, Won-Ho Hahn, Youhoon Jeon, Joo-Hee Kim, Tae-Rim Shin, Cheol-Hong Kim, In-Gyu Hyun, Jeong-Hee Choi, Sun-Mi Jang, Hae-Jung Na, Seung-Eon Song, Hye-Kyung Park, Eun-Jung Jo, Dong Hun Lee, Jin-Young Lee, Yang Park, Jae-Won Oh, Mi Hee Lee, Soo-Jong Hong, Soo-Jong Hong, So-Yeon Lee, Joon Soo Park, Dong-Ho Nahm, Hye-Yung Yum, Hye-Yung Yum, Kyu Young Choi, Dong-Young Kim, Sirinoot Palapinyo, Jettanong Klaewsongkram, Xing Chen, Yuting Jin, Xiaoming Hou, Fengqin Liu, Chunyan Guo, Yulin Wang, Ikuo Okafuji, Yuya Tanaka, Shegeyuki Narabayashi, Satoru Tsuruta, Yong Hyun Jang, Jun-Hong Ahn, Dong-Won Lee, Jin Hong Chung, Hyun Jung Jin, Min-Su Sohn, Young a Park, Kyunguk Jeong, Yoon Hee Kim, In Suk Sol, Seo Hee Yoon, Kyung Won Kim, Myung Hyun Sohn, Kyu-Earn Kim, Sooyoung Lee, Ho Kim, Ja Yeun Kim, So-Yeon Lee, Taek-Ki Min, Tae-Won Song, Kangmo Ahn, Jihyun Kim, Gwang-Cheon Jang, Hyeon-Jong Yang, Bok-Yang Pyun, Ji-Won Kwon, Myung Hyun Sohn, Kyu-Earn Kim, Jinho Yu, Soo-Jong Hong, Jung-Hyun Kwon, Sung-Won Kim, Sooyoung Lee, Woo Kyung Kim, Hyung Young Kim, Hye-Young Kim, Youhoon Jeon, Chang Hoon Lim, Yeongsang Jeong, Su Jung Kim, Hun Soo Chang, Jeong-Seok Heo, Da-Jeong Bae, Jong-Uk Lee, Ji-Na Kim, Chang-Gi Min, Hyun Ji Song, Jong-Sook Park, Soo Hyun Kim, Choon-Sik Park, Jing-Nan Liu, Youngwoo Choi, Yoo Seob Shin, Hae-Sim Park, Nima Rezaei, Sedigheh Bahrami Mahneh, Arezou Rezaei, Maryam Sadr, Masoud Movahedi, Jun Seak Gang, Joon Soo Park, Seung Soo Kim, Hyun Ho Bang, Kyeong Bae Park, Hye Sun Kim, Tae Ho Kim, Young Hwangbo, Hyun Jung Lee, Gyeong Hee Yoo, Young Chang Kim, Sakura Sato, Noriyuki Yanagida, Motohiro Ebisawa, Sailesh Palikhe, Hae-Sim Park, Seung-Hyun Kim, Ri-Yeon Kim, Eun-Mi Yang, Li Yuan Gabriella Nadine Lee, Marion Aw, Marion Aw, Bee Wah Lee, Bee Wah Lee, Evelyn Xiu Ling Loo, Yiong Huak Chan, Lynette Shek, Lynette Shek, I-Chun Kuo, I-Chun Kuo, Phaik Ling Quah, Phaik Ling Quah, Genevieve Llanora, Gerez Irvin, Joo Hyun Jung, Il Gyu Kang, Seon Tae Kim, Hyoungmin Park, Seon Tae Kim, Joo Hyun Jung, Il Gyu Kang, Hyoungmin Park, Kwang-Pil Ko, Jungsoo Lee, Howard Chu, Hemin Lee, Jung U. Shin, Chang Ook Park, Kwang Hoon Lee, Kwang Hoon Lee, Hong Kyu Kang, Dong Chang Lee, Geun Jeon Kim, Jae Hyung Hwang, Jin Bu Ha, Su Hee Jeong, Ho Kim, Shinha Hwang, Whahee Lee, Enkhbayar Bazarsad, Logii Narantsetseg, Munkhbayarlakh Sonomjamts, Gwang-Cheon Jang, Hyun-Hee Lee, Chang-Jong Lee, Huynsun Lim, Ji-Eun Soh, Dae-Jin Song, Ji-Won Kwon, Hyung Young Kim, Ju-Hee Seo, Byoung-Ju Kim, Hyo-Bin Kim, So-Yeon Lee, Gwang-Cheon Jang, Woo Kyung Kim, Young-Ho Jung, Soo-Jong Hong, Jung Yeon Shim, Enkhbayar Bazarsad, Logii Narantsetseg, Munkhbayarlakh Sonomjamts, Prabhakarrao Pv, Ranjitha Nadendla, Juan Fang, Jing Zhao, Dae-Jin Song, Sungchul Seo, Young Yoo, Yu-Ri Kim, Ji Tae Choung, Jee Hoo Lee, Margot Berings, Natalie De Ruyck, Claus Bachert, Philippe Gevaert, Gabriële Holtappels, Pureun-Haneul Lee, Byeong-Gon Kim, Choon-Sik Park, George D. Leikauf, An-Soo Jang, Byeong-Gon Kimc, Pureun-Haneul Lee, Choon-Sik Park, An-Soo Jang, Yang Park, Min-Su Sohn, Hyun Jung Jin, Dong-Won Lee, Jun-Hong Ahn, Jin Hong Chung, Sae-in Kim, Han-Ki Park, Do-Yeon Kim, Mina Rho, Jun-Pyo Choi, Yoon-Keun Kim, Wasu Kamchaisatian, Thitikul Hiranras, Surinda Wongpun, Phornthip Chiraphorn, Anupan Tantachun, Wannipa Wongrassamee, Planee Vatanasurkitt, Naratip Somboonkul, Nattipat Juthacharoenwong, Surangkana Techapaitoon, Montri Tuchinda, Sejin An, Jae Ho Lee, Ji-Hyeon Shin, Soo Whan Kim, Si Won Kim, Jun Myung Kang, Boo-Young Kim, Byung-Guk Kim, Ji-Won Kwon, Woo Kyung Kim, Hyung Young Kim, Hyo-Bin Kim, Ju-Hee Seo, So-Yeon Lee, Gwang-Cheon Jang, Young-Ho Jung, Soo-Jong Hong, Byoung-Ju Kim, Dae-Jin Song, Jung Yeon Shim, Jung-Won Lee, Kyung Ho Kim, Young Yoo, Won Suck Yoon, Sungchul Seo, In Soon Kang, Jae Won Choi, Hye-Young Lim, Ji Tae Choung, Michelle Buela, Koji Nishimura, Sang Chul Park, Hyo Jin Chung, Chang-Hoon Kim, Ju Wan Kang, Seong-Chul Hong, Keun-Hwa Lee, Jaechun Lee, Hye-Sook Lee, Jeong Hong Kim, Narantsetseg Logii, N. Nyamdavaa, B. Enkhbayar, B. Oyuntsatsral, S. Munkhbayarlakh, Enkhbayar Bazarsad, Munkhbayarlakh Sonomjamts, Bashir Omarjee, Shuo Li, Miyuki Hayashi, Ruby Pawankar, Shingo Yamanishi, Toru Igarashi, Yasuhiko Itoh, B. Gantulga, B. Enkhbayar, S. Munkhbayarlakh, L. Narantsetseg, Oyuntsatsral Batsaikhan, Pei-Chi Chen, Jiu-Yao Wang, Nicki Y. H. Leung, Christine Yee Yan Wai, Patrick S. C. Leung, Ka Hou Chu, Eun Jin Doh, Dong Hun Lee, Mira Choi, Hyun-Sun Yoon, Kyu Han Kim, Ji Soo Lim, Ji Hyeon Baek, Man Yong Han, Seung Jin Lee, Youhoon Jeon, Kyung Suk Lee, Young-Ho Jung, Hye Mi Jee, Youn Ho Shin, Yi Jiang, Miao Liu, Chaw Su Naing, Tze Chin Tan, Yong Yeow Chong, Young-Ho Kim, Eun Lee, Song-I Yang, Hyun-Ju Cho, Hyung Young Kim, Ji-Won Kwon, Young-Ho Jung, Byoung-Ju Kim, Ju-Hee Seo, Ho-Jang Kwon, Hyo-Bin Kim, So-Yeon Lee, Soo-Jong Hong, Soo Hyun Kim, Jacqueline Elizabeth Joseph, M. S. Soumya, Ruby Pawankar, Harshitha Kumar, Sohyoung Yang, Sung-Il Woo, Nima Rezaei, Sukran Kose, Basak Gol Serin, Arzu Didem Yalcin, Süheyla Serin Senger, Mehmet Erden, Ertan Serin, Ting Fan Leung, Agnes Sze-Yin Leung, Harshitha Kumar, M. S. Soumya, Jacqueline Elizabeth Joseph, Ruby Pawankar, Eun Hee Chung, Eunji Kim, Young Yoo, Young Yoo, Ji Tae Choung, Ji Tae Choung, Sungchul Seo, Sungchul Seo, In Soon Kang, Jue Seong Lee, Ji Hyen Hwang, Sang Min Lee, Joo Hyun Jung, Seung Joon Choi, Eugene Joe, Hyunjung Hwang, Shin Myung Kang, Yu Jin Kim, Sun Young Kyung, Jeong-Woong Park, Sung Hwan Jeong, Sang Pyo Lee, Alina Gaisina, Igor Shilovskiy, Aleksandra Nikonova, Oleg Kamyshnikov, Musa Khaitov, Alexander Mitin, Komogorova Viktoriya, Marina Litvina, Nina Sharova, M. J. Faiah, Intan H. Ismail, E. A. Miles, Faizah Mohamed Jamli, Jun-Pyo Choi, Han-Byul Choi, Yoon-Keun Kim, Hyeon-Il Choi, Da-Il Yoon, Mingyu Kang, Mi Yeoung Kim, Sujeong Kim, Eun-Jung Jo, Seoung-Eun Lee, Woo-Jung Song, Sang Min Lee, Chansun Park, Yoon-Seok Chang, Jaechun Lee, Young-Koo Jee, Inseon S. Choi, Kyung-up Min, Sang-Heon Cho, Sang-Heon Cho, Anton Laskin, Oleg Kamyshnikov, Alexander Babakhin, Valentina Berzhets, Musa Khaitov, Jae Ho Lee, Sejin An, Yoon-Seok Chang, Kyung-up Min, Sang-Heon Cho, Sae-Hoon Kim, Yong Eun Kwon, Young-Koo Jee, Tae-Bum Kim, Hee-Bom Moon, Hye-Kyung Park, Sung-Yoon Kang, Jun-Pyo Choi, Han-Ki Park, Ji-Hyun Lee, Yoon-Keun Kim, Sang-Yoon Kim, Hyunjung Hwang, Eugene Joe, Sang Min Lee, Seung Joon Choi, Joo Hyun Jung, Yong Han Seon, Shin Myung Kang, Yu Jin Kim, Sun Young Kyung, Jeong-Woong Park, Sung Hwan Jeong, Sang Pyo Lee, Natalya Khramykhoverchenko, Asma Sultana, Pnhrc, Rabih Halwani, Ahmed Bahammam, Saleh Al Muhsen, Sun Kyung Kim, Kwang Il Nam, Hyung Chae Yang, Jeong-Eun Kim, Ju Suk Lee, Ji Hyun Lee, Kyung Woo Kang, Je-Kyung Kim, Youn-Soo Hahn, Jae-Yub Jung, Yosuke Baba, Sususmu Yamazaki, Eisuke Inage, Mari Mori, Yoshikazu Ohtsuka, Masato Kantake, Toshiaki Shimizu, Asuka Honjoh, Tomoaki Yokokura, Shaza Ali Mohammed Elhassan, Caroline Beck, Mehdi Adeli, Heysung Baek, Seung Jin Lee, Ji Hyeon Baek, Jungwon Yoon, Sun Hee Choi, Young-Ho Jung, Youn Ho Shin, Man Yong Han, Min Sun Na, Enrico Compalati, Maurizio Marogna, Huimin Huang, Baoqing Sun, Mingyu Bai, Yiting Huo, Peiyan Zheng, Nili Wei, Wenting Luo, Anand Andiappan, Rosalba Minisini, Olaf Rötzschke, Elena Boggio, Luca Gigliotti, Nausicaa Clemente, Annalisa Chiocchetti, Umberto Dianzani, Mario Pirisi, Elisa Villa, Fumiya Yamaide, Syuji Yonekura, Naoki Shimojo, Yuzaburo Inoue, Yoshitaka Okamoto, Retno Asih Setyoningrum, Landia Setiawati, Sri Sumei, Deddy Iskandar, Indriyani Sang Ayu Kompiyang, Tsuyoshi Oguma, Jun Tanaka, Katsuyoshi Tomomatsu, Koichiro Asano, Keisuke Uno, Yoshinori Matsuwaki, Kazuhiro Omura, Eika Hayashi, Norifumi Tatsumi, Hirohito Kita, Nobuyoshi Otori, Hiromi Kojima, Mohammadreza Fatemi Khorasgani, Dukhee/Betty Lew, Kim/S. Lemessurier, Joseph/a Moore, Jeoung-Eun Park, Ae-Kyung Yi, Chi/Young Song, Kafait/U Malik, Ja Kyoung Kim, Hyeon-Jong Yang, Bong-Seong Kim, Youn Ho Shin, So-Yeon Lee, Geunhwa Park, Woo Kyung Kim, Hyo-Bin Kim, Heysung Baek, Dae Hyun Lim, Dae Hyun Lim, Jin Tack Kim, Dongin Suh, Aimee Lou Manalo Nano, Baoqing Sun, Wenting Luo, Hikmet Tekin Nacaroglu, Semiha Bahceci Erdem, Ozlem Sumer, Sait Karaman, Canan Sule Unsal Karkiner, Suna Asilsoy, Ilker Gunay, Demet Can, Danielle Kiers, Jiu-Yao Wang, Hsu Han Yin, Allen Kaplan, Kusumam Joseph, Baby G. Tholanikunnel, Radim Dudek, Gulden Bilgin, Hatice Surer, Aytun Sadan Kilinc, Dogan Yucel, Ji Young Lee, Jihyun Kim, Hea-Kyoung Yang, Minji Kim, Sang-Il Lee, Kangmo Ahn, Sung Do Moon, Byung-Keun Kim, Sang-Heon Cho, Kyung-up Min, Yoon-Seok Chang, Heung Woo Park, Hye-Ryun Kang, Woo-Jung Song, Min-Koo Kang, Ju-Young Kim, Kyonghee Sohn, Ha Kyung Won, Seoung-Eun Lee, Kyung-Mook Kim, Claus Bachert, Miao-Hsi Hsieh, Jiu-Yao Wang, Helen Smith, Clare Brown, Christina Jones, Mark Davies, Won Suck Yoon, Hemin Lee, Howard Chu, Jungsoo Lee, Jung U. Shin, Chang Ook Park, Kwang Hoon Lee, Kwang Hoon Lee, Seo Hyeong Kim, Seo Hyeong Kim, Ji Yeon Noh, Ji Hye Kim, Ji Hye Kim, Won Suck Yoon, Jean-Pierre L’huillier, Jean-Eric Autegarden, Catherine Bertrand, Dominique Tardy, Intan Hakimah Ismail, Mimi Tang, Paul Licciardi, Frances Oppedisano, Robert Boyle, Roy Robins-Browne, Hisako Yagi, Harumi Koyama, Yutaka Nishida, Takumi Takizawa, Hirokazu Arakawa, Hong Kyu Kang, Hemin Lee, Jungsoo Lee, Jung U. Shin, Kwang Hoon Lee, Kwang Hoon Lee, Howard Chu, Chang Ook Park, Na Young Yoon, Hyeyoung Lee, Seong Jun Seo, Eunhee Choi, Hye-Young Wang, Minyoung Jung, Eung Ho Choi, Dong Hye Kim, Joo-Hee Kim, Young-Sook Jang, Jeong-Hee Choi, Sunghoon Park, Young Il Hwang, Seung Hun Jang, Ki-Suck Jung, Mi-Jin Kang, Dongin Suh, Eun Lee, Kil Yong Choi, Young-Ho Jung, Song-I Yang, Bong-Soo Kim, Ha-Jung Kim, Juneyoung Koh, Hyun-Jin Kim, Kangmo Ahn, Youn Ho Shin, Hyun-Ju Cho, Byoung-Ju Kim, Young-Ho Kim, Yean Jung, Mizuko Mamura, Jeong-Hwan Yoon, Susumu Nakae, Inkyu Lee, Isao Matsumoto, Takayuki Sumida, Jin Soo Han, Katsuko Sudo, Ji Hyeon Ju, Gaik Chin Yap, Wen Tso Liu, Seungdae Oh, Pei Ying Hong, Chiung Hui Huang, Marion Aw, Lynette Shek, Bee Wah Lee, Young Seok Byun, Sung Wan Kim, Tae Kyung Koh, Joong-Saeng Jo, Kun Hee Lee, Chul Kwon, Sung-Hwa Dong, Myung Shin Kim, Chansun Park, Han Seok Cho, Min-Ju Kim, Min Ji Kim, Young Ok Park, Hye Yeong Lee, Hee Seong Kim, Eun Lee, Hyun-Ju Cho, Jinho Yu, Soo-Jong Hong, Keum Hee Hwang, Jung-Hyun Kim, You Sook Cho, Sae-Hoon Kim, Hyouk-Soo Kwon, Mira Yoo, Hyo-Jung Kim, So-Young Park, Bomi Shin, So Young Park, Bomi Seo, Min-Gu Kim, Hee-Bom Moon, Jin-Ah Park, Tae-Bum Kim, Jaemoon Lee, Jin Hyeok Jeong, Tae Wook Kang, Han Seok Yoo, Yong Hee Cho, Seok Hyun Cho, Kyung Rae Kim, Jue Seong Lee, Sun-Ho Kee, Sewon Kim, Young Yoo, Heung Sik Na, Seung Keun Back, Seung Jin Lee, Bo Seon Seo, Ji Hyeon Baek, Kyung Suk Lee, Young-Ho Jung, Hye Mi Jee, Youn Ho Shin, Man Yong Han, Mi-Ae Kim, Young-Hee Nam, Dong Sub Jeon, Soo-Keol Lee, Jisun Park, Seung Hyun Moon, Rong Jun Lin, Ren Zheng Guan, Gyeong Yul Park, Hyun-Sun Yoon, Woo-Hyeok Choi, Heysung Baek, Jin-Sung Park, Eunmi Kwon, Zac Callaway, Chang-Keun Kim, Takao Fujisawa, Qingling Zhang, Rihuang Qiu, Naijian Li, Zhaowei Yang, Jing Li, Kian Fan Chung, Nanshan Zhong, Kam Lun E. Hon, Yin Ching K. Tsang, Ting Fan Leung, Yoon Young Jang, Hai Lee Chung, Seung Gook Lee, Ji Hyun Na, Jong Hoon Lee, Young-Hee Nam, Dong Sub Jeon, Soo-Keol Lee, Mikita Yamamoto, Sakura Sato, Noriyuki Yanagida, Ayako Ogawa, Kanako Ogura, Kyohei Takahashi, Kenichi Nagakura, Shigehito Emura, Tomoyuki Asaumi, Katsuhito Iikura, Motohiro Ebisawa, Yu Okada, Jiaying Luo, Xiao Lan, Baoqing Sun, Zhao Chen, Guiyuan Sun, Shimin Li, Jiaqing Hu, Woo-Hyeok Choi, Heysung Baek, Young-Hee Nam, Dong Sub Jeon, Hee-Joo Nam, Yeo Myeong Noh, Sang Hee Kim Kim, Ye Suel Park, Soo-Keol Lee, Yean Jung Choi, Si Hyeon Lee, Young-Ho Kim, Mi-Jin Kang, Hyun-Ju Cho, Eun Lee, Song-I Yang, Youn Ho Shin, Kangmo Ahn, Kyung Won Kim, Yoon Hee Kim, So-Yeon Lee, Hyoung Yoon Chang, In Ae Choi, Kyung-Sook Lee, Yee-Jin Shin, Yoon Hee Kim, Min Jung Kim, In Suk Sol, Seo Hee Yoon, Young a Park, Kyung Won Kim, Myung Hyun Sohn, Kyu-Earn Kim, Yong Ju Lee, In Suk Sol, Kyu-Earn Kim, Yoon Hee Kim, Min Jung Kim, Seo Hee Yoon, Yong Ju Lee, Kyung Won Kim, Young a Park, Myung Hyun Sohn, Sung Joo Park, Ji-Won Kwon, Woo Kyung Kim, Hyung Young Kim, Hyo-Bin Kim, Ju-Hee Seo, So-Yeon Lee, Gwang-Cheon Jang, Young-Ho Jung, Soo-Jong Hong, Byoung-Ju Kim, Dae-Jin Song, Yun Seok Yang, Jung Yeon Shim, Yoon Young Jang, Hai Lee Chung, Ji Hye Kim, Hyun Seok Lee, Chang Ho Lee, Changbum Cho, Yun-Kyu Lim, Kyu Rang Kim, Mijin Kim, Baek-Jo Kim, Young-Min Kim, Youngshin Han, Jihyun Kim, Hae-Kwan Cheong, Byoung-Hak Jeon, Kangmo Ahn, Chuang/Yao Ming, Jiu-Yao Wang, Ye/Yi Ling, Huimin Huang, Baoqing Sun, Yun Chen, Peiyan Zheng, Nili Wei, Wenting Luo, Do Hyeong Lee, Gil-Soon Choi, Hee-Kyoo Kim, Han Su Park, So-Young Park, Hyo-Jung Kim, Bomi Seo, Jung-Hyun Kim, Min-Gu Kim, Hyouk-Soo Kwon, You Sook Cho, Hee-Bom Moon, Tae-Bum Kim, Yoon Su Lee, Eun-Soon Shin, Akio Tanaka, Satoshi Morioke, Yukihiro Ohya, Naoki Shimojo, Akira Akasawa, Michihiro Hide, Hiroko Shizukawa, Naoto Watanabe, Meeyong Shin, Myeong Sun Jang, Young-Hee Nam, Yeo Myeong Noh, Dong Sub Jeon, Hee-Joo Nam, Sang Hee Kim Kim, Ye Suel Park, Soo-Keol Lee, Ji-in Jung, Ha-Su Kim, Hyun-a Kim, Jin-a Jung, Anne Goh, Rajeshwar Rao, Bindu Nandanan, Ruurd Van Elburg, Chua Mei Chien, Juandy Jo, Johan Garssen, Johan Garssen, Leon Knippels, Elena Sandalova, Wen Chin Chiang, Young-Hee Nam, Ji Young Juong, Soo Jin Kim, Eun Young Kim, Su Mi Lee, Young Ki Son, Hee-Joo Nam, Ki-Ho Kim, Soo-Keol Lee, Da-Eun Park, Hye-Ryun Kang, Heung Woo Park, Hyun Seung Lee, Yoon-Seok Chang, Jung-Won Park, Sang-Heon Cho, Kyung-up Min, Woo-Jung Song, Hyunwook Lim, Sungchul Seo, Ji Tae Choung, Young Yoo, Jun-Sik Park, Byung Kwan Kim, Ha Kyeong Won, Hye-Ryun Kang, Byung-Keun Kim, Sung Do Moon, Ju-Young Kim, So-Hee Lee, Woo-Jung Song, Heung Woo Park, Min-Koo Kang, Sun-Sin Kim, Sang-Heon Cho, Kyung-up Min, Yoon-Seok Chang, Kyoung Hee Sohn, Kyung-Mook Kim, Ki-Woong Kim, Hak Chul Jang, Young-Hee Nam, Dong Sub Jeon, Hee-Joo Nam, Yeo Myeong Noh, Sang Hee Kim Kim, Ye Suel Park, Soo-Keol Lee, Ting Fan Leung, Man Fung Tang, Hing Yee Sy, Wa Cheong Chan, Wilson Wai San Tam, Seung Kyu Chung, Sujin Kim, Sang Duk Hong, Hyo Yeol Kim Hyo Yeol Kim, Hun-Jong Dhong, Jong in Jeong, Young-Hee Nam, Dong Sub Jeon, Soo-Keol Lee, Ji Won Lee, Mingyu Kang, Soon-Hee Kim, Boram Bae, Sujeong Kim, Hye-Ryun Kang, Yoon-Seok Chang, Woo-Jung Song, Da-Eun Park, Hyun Seung Lee, Heung Woo Park, Han-Ki Park, Jung-Won Park, Young-Hee Nam, Dong Sub Jeon, Hee-Joo Nam, Yeo Myeong Noh, Sang Hee Kim Kim, Ye Suel Park, Soo-Keol Lee, Yung-I Hou, Jiu-Yao Wang, Ja Hyeong Kim, Seol Jae Hee, Eun-Hee Ha, Hyesook Park, Mina Ha, Yun-Chul Hong, Yangho Kim, Namsoo Chang, Yuta Soma, So Watanabe, Ruby Pawankar, Harumi Suzaki, Hitome Kobayashi, Young-Hee Nam, Chansun Park, Soo-Keol Lee, Jongrok Lee, Jooyoung Roh, Haryeong Ryu, Cheol-Woo Kim, Jae Hwa Cho, Mi Ra Eom, Ji Young Kang, Hye Gyeung Lee, Hae Young Choi, Hye Jin Lee, Ju Yun Woo, Ji Yeon Byun, You Won Choi, Ja Hyeong Kim, Eun-Hee Ha, Hyesook Park, Mina Ha, Yun-Chul Hong, Yangho Kim, Namsoo Chang, Jae-Woo Kwon, Hun Soo Chang, Jeong-Seok Heo, Jong-Uk Lee, Jong-Sook Park, Eusom Kim, Soo Hyun Kim, Choon-Sik Park, Hae Young Choi, Ji Yeon Byun, Ju Yun Woo, You Won Choi, Hyun-Ju Jin, Jin-Hwa Son, Jeong-Min Kim, Gun-Wook Kim, Je-Ho Mun, Margaret Song, Hyun-Chang Ko, Moon-Bum Kim, Hoon-Soo Kim, Byung Soo Kim, Sheryl Van Nunen, Dinh Van Nguyen, Anthony Elias, Susannah Olivia Lauer, Seung-Chul Lee, Ho-June Lee, Jung Min Bae, Emi Ono, Sung-Hwa Dong, Tae Kyung Koh, Young Seok Byun, Sung Wan Kim, Joong-Saeng Jo, Chul Kwon, Kun Hee Lee, Li-Fan Liu, Sunghee Lee, Wei-Leng Chen, Jiu-Yao Wang, Youin Bae, Gyeong-Hun Park, Suk Yeon Kim, Hyun Seung Lee, Woo-Jung Song, Mingyu Kang, Han-Ki Park, Da-Eun Park, Hye-Ryun Kang, Heung Woo Park, Yoon-Seok Chang, Hye-Young Kim, Kyung-up Min, Sang-Heon Cho, Ji-Won Lee, Boram Bae, Jung-Won Park, Yasuhiro Suzuki, Ismet Bulut, Zeynep Ferhan Ozseker, Intan Hakimah Ismail, Robert Boyle, Paul Licciardi, Frances Oppedisano, Roy Robins-Browne, Mimi Tang, Ji Won Lee, Hyun Seung Lee, Mingyu Kang, Da-Eun Park, Han-Ki Park, Soon-Hee Kim, Woo-Jung Song, Hye-Ryun Kang, Heung Woo Park, Yoon-Seok Chang, Chang-Han Park, Suk-Il Chang, Sook-Hee Song, Kyung-up Min, Sang-Heon Cho, Boram Bae, Grace Shieh, Min Jung Kim, Jung Yeon Hong, Seo Hee Yoon, Doo Hee Shim, In Suk Sol, Yoon Hee Kim, Mi Na Kim, Kyung Eun Lee, Kyung Won Kim, Myung Hyun Sohn, Kyu-Earn Kim, Jae Myun Lee, Hiok Hee Chng, Dong Chan Kim, Song-I Yang, Hae Ran Lee, An Deok Seo, So Yeon Lee, Maria Cristina Artesani, Paola Francalanci, Lamia Dahdah, Thomas Schreiner, Alessandro Fiocchi, Kaushik Chakraborty, Ha Kyeong Won, Ju-Young Kim, Eun-Jung Jo, Kyoung Hee Sohn, Kyung-Mook Kim, Heung Woo Park, Yoon-Seok Chang, Sang-Heon Cho, Woo-Jung Song, Byung-Keun Kim, Syuji Yonekura, Yoshitaka Okamoto, Gyu Young Hur, Young Min Ye, Joo-Hee Kim, Ki-Suck Jung, Junga Kim, Jae Jeong Shim, Hae-Sim Park, Kazuhiro Sekimoto, Kazuko Sugai, Keiji Tsuchimoto, Hiromi Uehara, Masanori Ikeda, Euncho Chung, Kang Seo Park, Yean Jung Choi, Jeewon Park, Soo-Jong Hong, So Yeon Lee, Alain Jacquet, Arun Buaklin, Nat Malainual, S. Roopashree, Kyu Rang Kim, Mijin Kim, Changbum Cho, Baek-Jo Kim, Jae-Won Oh, Mae Ja Han, Hyun-Ju Cho, Youn Ho Shin, Eun Lee, Young-Ho Kim, Darae Lee, Mi-Jin Kang, Song-I Yang, Kangmo Ahn, Kyung Won Kim, Yoon Hee Kim, Hye-Sung Won, Soo Hyun Kim, Suk-Joo Choi, Young Han Kim, Jong Kwan Jun, Eun-Jin Kim, Jeom Gyu Lee, So-Yeon Lee, Soo-Jong Hong, Dongin Suh, Yosuke Amagai, Akane Tanaka, Hiroshi Matsuda, Ralph Mösges, Pauline Dieterich, Anatoli Astvatsatourov, Christoph Hüser, Jaswinder Singh, Kija Shah-Hosseini, Silke Allekotte, Enrico Compalati, Kyoung Hee Sohn, Woo-Jung Song, Byung-Keun Kim, Ju-Young Kim, Min Suk Yang, So-Hee Lee, Sae-Hoon Kim, Hye-Ryun Kang, Heung Woo Park, Sun-Sin Kim, Kyung-up Min, Sang-Heon Cho, Yoon-Seok Chang, Woo-Sung Chang, Ji-Hye Do, Yeon-Seop Kim, Dankyu Yoon, Hye-Sun Lim, Jeom-Kyu Lee, Eun-Jin Kim, Bernard Thong, Yew Kuang Cheng, Jinfeng Hou, Khai Pang Leong, Justina Tan, Faith Chia, Grace Chan, Sze-Chin Tan, Teck Choon Tan, Chwee Ying Tang, Hiok Hee Chng, Chan-Sun Park, Mi Yeoung Kim, Eun-Young Kim, Jae-Gook Shin, Jae-Hyeog Choi, Saegwang Park, Yeonye Kim, Kyung-Hwan Lim, Jae Woo Jung, Mingyu Kang, Ju-Young Kim, Ju-Young Kim, Hyun Jeong Kim, Yeon-Ju Woo, Soo-Youn Jung, Hye-Ryun Kang, Hye-Ryun Kang, Thierry Porée, Nabile Boukhettala, Emeline Furon, Alan David Bullimore, Matthew Heath, Simon Hewings, Murray Skinner, Marcin Kurowski, Hubert Krysztofiak, Aleksandra Wardzynska, Marzanna Jarzebska, Janusz Jurczyk, Marek L. Kowalski, So Ri Kim, Yong Chul Lee, Dong Im Kim, Yang Keun Rhee, Heung Bum Lee, Seoung Ju Park, Yeong Hun Choe Choe, Seung Yong Park, Joo-Hee Kim, Sunghoon Park, Young Il Hwang, Seung Hun Jang, Ki-Suck Jung, Jiang Min, Nong Guang-Min, Nalin Nag, Wahyuni Indawati, Alan David Bullimore, Matthew Heath, Murray Skinner, Seong Jun Seo, Won Jong Oh, Alan David Bullimore, Murray Skinner, Matthew Heath, Andrew Bell, Hournaz Hasanzadeh, Salman Sadeghzade, Nima Rezaei, Alireza Zarebidoki, Kyu-Sup Cho, Moo-Young Oh, Sung-Woo Kim, Munemitsu Koizumi, Kazuyo Kuzume, Kui Young Park, Won Jong Oh, Delara Babaie, Mohammad Nabavi, Fariborz Zandieh, Mehrdad Amir Moini, Zahra Chavoshzadeh, Hamideh Seifi, Mitra Sahragard, Mehrnaz Mesdaghi, Mohammad Hassan Bemanian, Sun Young Choi, Yeon a No, Dorota Kiedik, Agnieszka Muszynska, Iwona Pirogowicz, Andrzej M. Fal, Chikako Motomura, Masatoshi Wakatsuki, Yuko Akamine, Mihoko Iwata, Hiroshi Matsuzaki, Naohiko Taba, Yoko Murakami, Hiroshi Odajima, Reni Ghrahani, Yuri Takaoka, Jong-Uk Lee, Jeong-Seok Heo, Da-Jeong Bae, Hyun Ji Song, Choon-Sik Park, Jong-Sook Park, Jae Hoon Cho, Ji Ho Choi, Fiska Febriana, Reni Ghrahani, Gartika Sapartini, Budi Setiabudiawan, Young-Hee Nam, Mi Yeoung Kim, Gil-Soon Choi, Chan-Sun Park, Chiung-Hui Huang, Jian Yi Soh, Lynette Shek, Lynette Shek, Dianne J. Delsing, Bee Wah Lee, Bee Wah Lee, Si Hui Goh, Wen Chin Chiang, Wenyin Loh, Hea-Kyoung Yang, Ji Young Lee, Minji Kim, Kangmo Ahn, Jihyun Kim, Young-Min Kim, Hye-Young Kim, Yong Mean Park, Woo Kyung Kim, So-Yeon Lee, Jongin Jeong, Sang Duk Hong, Seung Kyu Chung, Hun-Jong Dhong, Hyo Yeol Kim, Sujin Kim, Hana Bak, Hye-Rim Son, Si-Eun Lee, Kwang-Jin Kim, Young-Wook Lim, Ho-Hyun Kim, Yong-Won Lee, Man Yong Han, Young-Ho Jung, Hye Mi Jee, Seung Jin Lee, Kyung Suk Lee, Mi-Ae Kim, Jaechun Lee, Eunkyoung Lee, Jasmina Golez, Da-Jeong Bae, Chang-Gi Min, Jong-Uk Lee, Jong-Sook Park, Hun Soo Chang, Choon-Sik Park, An-Soo Jang, Ha-Jung Kim, Young-Joon Kim, Bok Kyoung Jung, Seung-Hwa Lee, Mi-Jin Kang, Sekyoo Jeong, Eun Lee, Hyun-Ju Cho, Young-Ho Kim, Song-I Yang, Seo Hee Kim, Soo-Jong Hong, Rabih Halwani, Saleh Al Muhsen, Asma Sultana, Achraf Al-Faraj, Rosan Kanana, Sibtain Afzal, Roaa Al Kufaidi, Hee-Kyoo Kim, Chul-Ho Oak, Gil-Soon Choi, Ye-Jin Moon, Eun-Kee Park, Mina Abrari, Ali Akbar Amirzargar, Alireza Zarebidoki, Mina Ha, Soo-Jong Hong, Ju-Hee Seo, Mingyu Kang, Byung-Ha Cho, Han-Ki Park, Han-Ki Park, Kyung-Mook Kim, Chang-Han Park, Heung Woo Park, Heung Woo Park, Yoon-Seok Chang, Yoon-Seok Chang, Yoon-Seok Chang, Sook-Hee Song, Mi-Kyeong Kim, Mi-Kyeong Kim, Sang-Heon Cho, Suk-Il Chang, Kyung-up Min, Kyung-up Min, Alyn Morice, Jungi Choi, Yusok Han, Jin-Sung Park, Eunmi Kwon, Chang-Keun Kim, Gartika Sapartini, Ji-Na Kim, Seungwoo Shin, Hun Soo Chang, Eun-Young Shim, Ji Ah Jun, Hyeonju Lee, Jong-Sook Park, Choon-Sik Park, Revaz Sepiashvili, Darejan Khachapuridze, Sofio Gamkrelidze, Manana Chikhladze, Seung-Eun Lee, Yun-Seong Kim, Doo-Soo Jeon, Woo-Hyun Cho, Hye-Ju Yeo, Seong-Hoon Yoon, Seung-Hyun Kim, Taehyeong Lee, Hyun Ji Song, Choon-Sik Park, Ji Ah Jun, Jong-Sook Park, Dankyu Yoon, Yeon-Seop Kim, Woo-Sung Chang, Mi-Jin Kang, Soo-Jong Hong, Jeom-Kyu Lee, Eun-Jin Kim, Minkee Park, Nanju Alice Lee, Johanna Rost, Sridevi Muralidharan, Dianne Campbell, Sam Mehr, Seung-Hwa Lee, Seon-Joo Yoon, Ha-Jung Kim, Eun Lee, Song-I Yang, Young-Ho Jung, Ho-Sung Yu, Hee-Suk Kim, Yeon Hee Park, So-Yeon Lee, Jun-Sung Park, Hyun Ok Jun, Ha Kyeong Won, Min-Koo Kang, Sung Do Moon, Byung-Keun Kim, Ju-Young Kim, Sang-Heon Cho, Hye-Ryun Kang, Ji-Su Shim, Soo Jie Chung, Jaehee Choi, Kangmo Ahn, Kwanghoon Kim, Jihyun Kim, Jiyoung Lee, Bo Bae Park, In Young Nho, Chang-Han Park, Jang Min Kim, Suk-Il Chang, Sun Kyung Kim, Hyung Chae Yang, Kwang Il Nam, Jeongmin Lee, Sooyoung Lee, Kyunguk Jeong, Se-Ah Jeon, Midori Fujiwara, Shoko Shindo, Hiroyuki Murota, Mayuko Tahara, Aya Takahashi, Ichiro Katayama, Jae Woo Jung, Hyun Ji Song, Taehyeong Lee, An-Soo Jang, Jong-Sook Park, Hun Soo Chang, Choon-Sik Park, Byoung Whui Choi, Min-Hye Kim, Da-Jeong Bae, Hyun Ji Song, Taehyeong Lee, Ji Ah Jun, Jong-Sook Park, An-Soo Jang, Hun Soo Chang, Young Joo Cho, Choon-Sik Park, Sue Jean Mun, Etsushi Kuroda, Koji Ozasa, Ken Ishii, Sunmi Kim, Gyeong-Hun Park, Hyun Ji Song, Taehyeong Lee, Ji Ah Jun, Hun Soo Chang, Jong-Sook Park, Choon-Sik Park, Mi-Ae Kim, Seungwoo Shin, Jong-Sook Park, Hun Soo Chang, You Sook Cho, Hae-Sim Park, Choon-Sik Park, Zhang Min, Seo Hee Yoon, In Suk Sol, Young a Park, Yoon Hee Kim, Min Jung Kim, Kyung Won Kim, Myung Hyun Sohn, Kyu-Earn Kim, Wu Shiquan, Yongwon Lee, Hana Bak, Maricar Wisco Ching, John Donnie Ramos, Kyunguk Jeong, Sooyoung Lee, Kangmo Ahn, Myung Hyun Sohn, Kyung Won Kim, So-Yeon Lee, Tae Won Song, Youhoon Jeon, Jihyun Kim, Taek Ki Min, Kyu-Earn Kim, Bok-Yang Pyun, Hyeon-Jong Yang, Hae Ran Lee, Youngmin Ahn, Ji-Won Kwon, Dae Hyun Lim, Jeong Hee Kim, Dongin Suh, Hyung Young Ki, Kyunguk Jeong, Byeong Sub Park, Sooyoung Lee, Se-Ah Jeon, Kyu Jung Park, Song-I Yang, Eun Lee, Hyun-Ju Cho, Young-Ho Kim, Mi-Jin Kang, Yean Jung Choi, Kil Yong Choi, Youn Ho Shin, Kangmo Ahn, Kyung Won Kim, Byoung-Ju Kim, So-Yeon Lee, Eun-Jin K, Roccatello Dario, Jing Liao, Yong Feng, Yunxiao Shang, Yongwon Lee, Hana Bak, Hyung Young Kim, Byoung-Ju Kim, Ji-Won Kwon, Ju-Hee Seo, Eun Lee, So-Yeon Lee, Song-I Yang, Young-Ho Jung, Hyo-Bin Kim, Ho-Jang Kwon, Hee Ju Park, Zhang Min, Nong Guang-Min, Jiang Min, Gyu Young Hur, Eun Jung Sim, Sora Yoon, Juwhan Choi, Junga Kim, Jae Keom Sim, Jee Youn Oh, Kyunguk Jeong, Byeong Sub Park, Jeong-Min Lee, Sooyoung Lee, Eunjae Cheon, Youngjoo Na, Kyu Jung Park, Eunjoo Lee, William Yang, Suzanne Kelly, Rob Perrins, Jimmy Yang, William Yang, Suzanne Kelly, Rob Perrins, Jacob Karsh, Jimmy Yang, Hector Badellino, Alvaro Teijeiro, Mabel Cuello, Marilyn Urrutia Pereira, Gustavo Egues, Ayse Aktas, Jin-Kyong Chun, Hilda Angela Mujuru, Elopy N. Sibanda, Andreea Ioana Popescu, Raluca Greblescu, Suheyla Rahman, Ayse Aktas, Nataly Tataurshchikova, Eishika Dissanayake, Yuzaburo Inoue, Naoki Shimojo, Taiji Nakano, Conny Tanjung, Erika Jensen-Jarolim, Judit Fazekas, Josef Singer, Anna Lukschal, Reinhard Horvat, Gertrude Achatz-Straussberger, Gernot Achatz, Yuji Fujita, Shuji Ikegami, Yoshitaka Nakamura, Yuzaburo Inoue, Naoki Shimojo, Yoichi Kohno, Shuichi Suzuki, Naoko Ozawa, Takayuki Kubota, Ken Nonaka, Osamu Ohara, Kentaro Masuda, Chin Kook Rhee, Sook Young Lee, Hwa Young Lee, Hea Yon Lee, Ji Young Kang, Sei Won Kim, Soon Seog Kwon, Young Kyoon Kim, Gun-Woo Kim, Ju-Young Kim, Sang-Heon Cho, Sang-Heon Cho, Hye-Ryun Kang, Hye-Ryun Kang, Hyo-Soo Kim, Jung Gyu Han, Jin Lee, Ji Young Lee, Ji Young Go, So Jung Park, Julie Kim-Chang, Cassandra Love, Patricia Lugar, Endah Citraresmi, Nastiti Kaswandani, Cynthia Utami, Mardjanis Said, Young-Ho Jung, Song-I Yang, Byoung-Ju Kim, Ji-Won Kwon, Hwan-Cheol Kim, Jong-Han Leem, Ju-Hee Seo, Hyung Young Kim, So-Yeon Lee, Ho-Jang Kwon, Hyo-Bin Kim, Hyun-Ju Cho, Susumu Yamazaki, Nobuhiro Nakano, Asuka Honjoh, Eisuke Inage, Yosuke Baba, Yoshikazu Ohtsuka, Toshiaki Shimizu, Ishaq M., Sameera M. I. Khan, Imran Khan, Sabeen Khan, Evelyn Xiu Ling Loo, Anne Goh, Oon Hoe Teoh, Yiong Huak Chan, Seang Mei Saw, Kenneth Kwek, Peter D Gluckman, Keith M Godfrey, Hugo Van Bever, Yap Seng Chong, Bee Wah Lee, Lynette Shek, Alison Joanne Lee, Daniela Rossi, Agnes Nemeth, Torsten Sehlinger, Karl-Christian Bergmann, Frank Goergen, Mohammad S. Ehlayel, Abdul Bari Bener, Hieu Chi Chu, Nga Thi Quynh Do, Dinh Van Nguyen, Ha Thi Thu Nguyen, Huong Thi Minh Le, Sheryl Van Nunen, Christopher Vidal, Suran Fernando, Fotis Psarros, Ekaterini Syrigou, Ekaterini Politi, Spyridon Chrysoulakis, Dimitrios Vourdas, Konstantinos Petalas, Mouli Saha, Kashinath Bhattacharya, Subir Jain, Xiaolian Song, Haiyan Lin, Kyohei Nishikawa, Takashi Shimada, Hiroshi Yasueda, Tadao Enomoto, Daisuke Aizawa, Takayoshi Kobayashi, Chellaa R., Subir Jain, Subir Jain, Marina Yudina, Ishaq M., Sameera M. I. Khan, Imran Khan, Sabeen Khan, Mayako Saito, Nurul Iman Nilam Sari, Jae Young Kim, Jaechul Song, Inah Kim, Kyeong Joon Lee, Soo Jin Park, Soo Yong Roh, Somxay Billamay, Muchammad Fahrul Udin, Mae Ja Han, Jae-Won Oh, Kyu Rang Kim, Baek-Jo Kim, Jorge A. Mazza, Jason Kangeun Ko, David J. T. Huang, Kanae Furuya, Keigo Kainuma, Takahiro Ito, Mizuho Nagao, Takao Fujisawa, Junya Hirayama, Yu Kuwahara, Rosanna Qualizza, Cristoforo Incorvaia, Anna Maraschini, Syed Mohammed Hasnain, Abdulrahman Al-Frayh, Anita Hartog, Jacqueline Bastiaans, Reinilde Loonstra, Lieke Rutten, Lucien Harthoorn, Jeroen Van Bergenhenegouwen, Johan Garssen, Kwang-Ha Yoo, Sang-Heon Cho, AG Ghoshal, Abdul Razak Bin Abdul Muttalif, Horng- Chyuan Lin, Sanguansak Thanaviratananich, Shalini Bagga, Rab Faruqi, Santwona Baidya, Colman Taylor, De Yun Wang, Hae-Ryun Ahn, Soon-Kwan Hong, Jong-Woong Kim, Gui-Hyun Nam, Mee-Ja Kim, Jae-Kyoung Park, Myung-Hee Yi, Kyoung Yong Jeong, Ju-Yeong Kim, Tai-Soon Yong, Bum Joon Kim, Hs Joo, Kj Lim, Jae-Hyun Lee, Jung-Won Park, Kh Yoon, DS Choi, Hanseung Joo, Bum Joon Kim, Kj Lim, MJ Kim, DS Choi, Kh Yoon, Bum Joon Kim, Hanseung Joo, Woo Sang Jung, Kj Lim, DS Choi, Ji-Hoon Lee, Soon-Chan Kwon, Soo-Jin Lee, Soo Yong Roh, Hogil Kim, Kyeong Joon Lee, Aurora Van De Loo, Amanda Fernstrand, Johan Garssen, Joris Verster, Aurora Van De Loo, Johan Garssen, Joris Verster, Jasmina Golez, Koji Ozasa, Etsushi Kuroda, Ken Ishii, Muhammad Imran, Selina Gierer, John Martinez, Lydia Wong, Bee Wah Lee, Gaik Chin Yap, Genevieve Llanora, Bernard Thong, Lynette Shek, Mohammad S. Ehlayel, Ashraf Soliman, Pedro Martins, João Marques, Joana Gomes-Belo, Teresa Palmeiro, Iolanda Caires, Joana Belo, Maria Amália Botelho, Paula Leiria-Pinto, Nuno Neuparth, Narissara Suratannon, Jaichat Mekaroonkamol, Jarungchit Ngamphaiboon, Piyawadee Lertchanaruengrith, Pantipa Chatchatee, Gholamali Kardar, Ahmad Majd, Youcef Shahali, Farrokh Ghahremaninejad, Zahra Pourpak, Fateme Mousavi, Mahnaz Sadeghi-Shabestari, K. Van Bilsen, O. Manusama, W.a. Dik, M. Van Der Burg, V. H. J. Van Der Velden, V.a.S. H. Dalm, P. M. Van Hagen, Yongping Liu, Yi Yeong Jeong, Ji Hye Kim, Moon Gyeong Yoon, Young Min Ye, Yoo Seob Shin, Ga Young Ban, Hae-Sim Park, Hye Min Jung, Soo Yong Roh, Jaechul Song, Ji-Hoon Lee, Hogil Kim, Jae Young Kim, Kyeong Joon Lee, Lydia Wong, Mohana Rajakulendran, Haripriya Santhanam, Lynette Shek, Tow Keang Lim, Hogil Kim, Soo-Jin Lee, Ji-Hoon Lee, Soo Yong Roh, Soon-Chan Kwon, Ani Wistiani, Galuh H, Gholam Ali Kardar, Maryam Sharifshoushtari, Ahmad Majd, Taher Nejadsattari, Zahra Pourpak, Mostafa Moin, Ji Hye Kim, Daehong Seo, Young Min Ye, Hae-Sim Park, Jung-Won Park, Jae-Hyun Lee, Yoo Seob Shin, Marek L. Kowalski, Aleksandra Wardzynska, Marcin Kurowski, Malgorzata/Ewa Pawelczyk, Adam Wysokinski, Iwona Kloszewska, Janina Grzegorczyk, Wojciech Piotrowski, Joanna Makowska, Soon-Chan Kwon, Jaechul Song, Yong-Kyu Kim, Ilknur Bostanci, Zeynep Sengul Emeksiz, Aysegul Ertugrul, Serap Ozmen, Soner Sahin, Sang-Ha Kim, Myoung Kyu Lee, Won Yeon Lee, Suk Joong Yong, Seok Jeong Lee, Ye-Ryung Jung, Myung Shin Kim, Jong-Sook Park, An-Soo Jang, Choon-Sik Park, Diah Asri Wulandari, Cissy Kartasasmita, Finny Fitry Yani, Rizanda Machmud, Dhina Lydia Lestari, Rusdi Rusdi, Yurmalina Yurmalina, Eryati Darwin, Ilknur Bostanci, Gulin Karacan, Nazli Ercan, Asuman Colak, Murat Alisik, Gulay Basarir, Ozcan Erel, Ilknur Bostanci, Yasemin Keskin, Andreas/Ludwig Lopata, Kyall Zenger, Roni Nugraha, Sandip Kamath, Leonard Bielory, Panos Georgopoulos, Yong Zhang, Wheat Mi, Ting Cai, MO Xian, Jing Li, Mulin Feng

**Affiliations:** Korea University College of Medicine, Guro Hospital, Seoul, South Korea; College of Medicine, Korea University, Seoul, South Korea; Korea University Guro Hospital, Seoul, South Korea; Seoul National University Boramae Hospital, Seoul, South Korea; Division of Clinical Immunology, Faculty of Medicine, University of Debrecen, Debrecen, Hungary; Meiji Pharmaceutical University, Kiyose, Tokyo, Japan; Suri Medical Foundation Hospital, Lucknow District, Uttar Pradesh State, India; Shahid Beheshti University, Tehran, Iran; Airlangga University Teaching Hospital, Surabaya, Indonesia; Tokyo Metropolitan Police Hospital, Tokyo, Japan; Shinichiro KogaSamsung Changwon Hospital, Seoul, South Korea; Sapporo Tokushukai Hospital, Sapporo, Japan; Department of Dermatology, Yonsei University College of Medicine, Seoul, South Korea; Brain Korea 21 PLUS Project for Medical Science, Yonsei University College of Medicine, Seoul, South Korea; Sureyyapasa Chest Diseases and Chest Surgery Training Hospital, Istanbul, Turkey; Kangnam Sacred Heart Hospital, Seoul, South Korea; Sakai City Medical Center, Sakai, Japan; Osaka University, Suita, Japan; Yonsei University College of Medicine, Seoul, South Korea; Osaka-Minami Medical Center, Kawachinagano, Japan; Inha University, College of Medicine, Incheon, South Korea; Inha University Hospital, Incheon, South Korea; Kangnam Sacred Heart Hospital, Seoul, South Korea; Chuncheon Sacred Heart Hospital, Chuncheon, South Korea; Center for Rhinology and Allergology Wiesbaden, Wiesbaden, Germany; Leti Pharma Gmbh, Witten, Germany; Postgraduate Institute of Medical Education and Research, Chandigarh, India; West Bengal Technical University, Kolkata, India; Institute of Microbiology, Academy of Sciences of the Czech Republic, Prague, Czech Republic; Institute of Microbiology, Academy of Sciences of the Czech Republic, Prague, Czech Republic; Seoul National University Hospital, Seoul, South Korea; Ent and Allergy Centre(INDIA), Panchkula, India; University of Santo Tomas Hospital, Manila, Philippines; University of Santo Tomas Faculty of Medicine, Manila, Philippines; Kyung Hee University Hospital at Gang-Dong, Seoul, South Korea; Yonsei University College of Medicine, Seoul, South Korea; Sangji University, Wonju, South Korea; Division of Allergy and Immunology, Department of Internal Medicine, Yonsei University College of Medicine, Seoul, South Korea; Yonsei University College of Medicine, Seoul, South Korea; Sangji University, Wonju, South Korea; Division of Allergy and Immunology, Department of Internal Medicine, Yonsei University College of Medicine, Seoul, South Korea; Pasteur Institute of Iran, Tehran, Iran; Hanyang University Medical Center, Seoul, South Korea; Hanyang University Seoul Hospital, Seoul, South Korea; Hanyang University Kuri Hospital, Guri, South Korea; Hanyang University Kuri Hospital, Guri, South Korea; Hanyang University Seoul Hospital, Seoul, South Korea; Institute of Nutrition, Moscow, Russia; DNA-Technology, JSC, Moscow, Russia; Institute of Nutrition, Moscow, Russia; Seoul St. Mary’s Hospital, Seoul, South Korea; College of Medicine, the Catholic University of Korea, Seoul, South Korea; State Key Laboratory of Respiratory Disease, the First Affiliated Hospital of Guangzhou Medical College, Guangzhou, China; Guangzhou Institute of Respiratory Disease, the First Affiliated Hospital of Guangzhou Medical College, Guangzhou, China; University of Eastern Finland, Kuopio, Finland; University Children’s Hospital Munich, Munich, Germany; Prince of Wales Hospital, Hong Kong, Hong Kong; The First Affiliated Hospital, Guangzhou Medical University, Guangzhou, China; Hanyang University Medical Center, Seoul, South Korea; Hanyang University Seoul Hospital, Seoul, South Korea; Hanyang University Kuri Hospital, Guri, South Korea; First Affiliated Hospital Guangzhou Medical University, Guangzhou, China; Inha University Hospital, Incheon, South Korea; Korea University, Seoul, South Korea; Tepecik Training and Research Hospital, Izmir, Turkey; Antalya Education and Research Hospital, Antalya, Turkey; Antalya Training and Research Hospital, Antalya, Turkey; Samsung Medical Center, Seoul, South Korea; Samsung Biomedical Research Institute, Seoul, South Korea; Bundang Jaesang Hospital, Bundang, South Korea; Samsung Medical Center, Sungkyunkwan University School of Medicine, Seoul, South Korea; Ansan Sarang General Hospital, Ansan, South Korea; Seoul Samsung Medical Clinic, Incheon, South Korea; Health Promotion Center, Samsung Medical Center, Seoul, South Korea; National Center for Child Health and Development, Tokyo, Japan; Samsung Medical Center, Seoul, South Korea; Sahmyook Medical Center, Seoul, South Korea; King Sejong ORL-Hns Clinic, Sejong, South Korea; Peking Union Medical College Hospital, Beijing, China; Toyama University School of Med, Toyama, Japan; Kyoto University, Kyoto, Japan; Kakogawa City West Hospital, Hyogo, Japan; Kobe City Medical Center General Hospital, Kobe, Japan; Beppu University, Beppu, Japan; College of Medicine, the Catholic University of Korea, Seoul, South Korea; The Catholic University of Korea, Seoul, South Korea; The Catholic University of Korea, Seoul, South Korea; Prince of Wales Hospital, Hong Kong, Hong Kong; The Catholic University of Korea, Seoul, South Korea; College of Medicine, the Catholic University of Korea, Seoul, South Korea; Philippine Children’s Medical Center, Quezon, Philippines; Kansas University Medical Center, Kansas City, USA; Seoul National University Hospital, Seoul, South Korea; Pusan National University, Pusan, South Korea; Pusan National University Yangsan Hospital, Busan, South Korea; Asan Medical Center, University of Ulsan College of Medicine, Seoul, South Korea; Bharati Vidyapeeth Deemed University, Pune, India; Yashiro Dermatology Clinic, Hirakata, Japan; Postgraduate Institute of Medical Education and Research, Chandigarh, Japan; Postgraduate Institute of Medical Education and Research, Chandigarh, Japan; Nippon Medical School, Tokyo, Japan; The Catholic University of Korea, Seoul, South Korea; The Catholic University of Korea, Seoul, South Korea; Samitivej Sukhumvit Hospital, Bangkok, Thailand; Incheon St. Mary’s Hospital, Incheon, South Korea; D.Y.Patil Medical College and Hospital, Navi Mumbai, India; Medical Faculty of Univesity of Sam Ratulangi, Manado, Indonesia; Samratulangi University Manado, Manado, Indonesia; Prince of Wales Hospital, Hong Kong, Hong Kong; The Chinese University of Hong Kong, Hong Kong, Hong Kong; Ajou University School of Medicine, Suwon, South Korea; Seoul National University College of Medicine, Seoul, South Korea; Seoul National University Hospital, Seoul, South Korea; Boramae Medical Center, Seoul, South Korea; Busan Paik Hospital, Inje University College of Medicine, Busan, South Korea; Ilsan Paik Hospital, Inje University College of Medicine, Goyang, South Korea; Haeundae Paik Hospital, Inje University College of Medicine, Busan, South Korea; The Chinese University of Hong Kong, Hong Kong, Hong Kong; The Chinese University of Hong Kong, Hong Kong, Hong Kong; University of California, Davis, USA; Yonsei University College of Medicine, Seoul, South Korea; Ajou University School of Medicine, Suwon, South Korea; Choongmoo Hospital, Cheonan, South Korea; GF Clinic, Seoul, South Korea; Cheonan Chungmu Hospital, Cheonan, South Korea; Ajou University School of Medicine, Suwon, South Korea; GF Allergy Clinic, Seoul, South Korea; Ajou University School of Medicine, Suwon, South Korea; Seoul National University College of Medicine, Seoul, South Korea; Chosun University College of Medicine, Gwangju, South Korea; University of Santo Tomas Faculty of Medicine, Manila, Philippines; University of Santo Tomas Faculty of Medicine, Manila, Philippines; Ajou University School of Medicine, Suwon, South Korea; Uiseong Public Health Center, Uiseong, South Korea; State Key Laboratory of Respiratory Disease, the First Affiliated Hospital of Guangzhou Medical University, Guangzhou, China; Children’s Hospital, Helsinki, Finland; Hospital for Allergic and Skin Diseases, Helsinki, Finland; D.Y.Patil Medical College and Hospital, Navi Mumbai, India; Ajou University School of Medicine, Suwon, South Korea; The Armed Forces Chun Cheon Hospital, Chun Cheon, South Korea; National Cheng Kung University, Tainan, Taiwan; Ajou University School of Medicine, Suwon, South Korea; Suwon Center for Environmental Disease and Atopy, Suwon, South Korea; Gumi CHA University Hospital, Gumi, South Korea; Busan St. Mary’s Hospital, Busan, South Korea; Asan Medical Center Seoul Korea, Seoul, South Korea; Hallym University Sacred Heart Hospital, Anyang, South Korea; Seoul National University Hospital, Seoul, South Korea; Wake Forest School of Medicine, Winston-Salem, USA; Yale School of Medicine, New Haven, USA; Division of Pulmonary, Allergy, and Critical Care Medicine, Pittsburgh, USA; Guangzhou Women and Children’s Medical Center, Guangzhou, China; Dankook University, Cheonan, South Korea; Seoul National University, Seoul, South Korea; Asan Medical Center, Seoul, South Korea; Asan Institute for Life Sciences, University of Ulsan College of Medicine, Seoul, South Korea; Asan Medical Center, Seoul, South Korea; Institute for Life Science, Asan Medical Center, Seoul, South Korea; Hallym University Sacred Heart Hospital, Anyang, South Korea; Bundang CHA Medical Center, CHA University School of Medicine, Seongnam, South Korea; Pusan National University Yangsan Hospital, Busan, South Korea; Korea Cancer Center Hospital, Seoul, South Korea; University of Cincinnati College of Medicine, Cincinnati, USA; Inje University Sanggye Paik Hospital, Seoul, South Korea; Hallym University Sacred Heart Hospital, Anyang, South Korea; Dankook University College of Medicine, Cheonan, South Korea; Childhood Asthma Atopy Center, Environmental Health Center, Asan Medical Center, University of Ulsan College of Medicine, Seoul, South Korea; Ajou University School of Medicine, Suwon, South Korea; Chulalongkorn University, Bangkok, Thailand; Allergy and Clinical Immunology Research Group, Chulalongkorn University, Bangkok, Thailand; Institute of Nutrition, Moscow, Russia; Sagamihara National Hospital, Sagamihara, Japan; Ccmar, Universidade Do Algarve, Faro, Portugal; University of the Western Cape, Naprsu, National Agricultural Proteomics Research and Services Unit, Bellville, South Korea; Agricultural Research Council, Infruitec, Proteomics Unit, Bellville, South Korea; Chonnam National University, Gwangju, South Korea; Chonnam National University Hospital, Gwangju, South Korea; Sydney Medical School - Northern, University of Sydney, Sydney, Australia; Hanoi Medical University, Hanoi, Vietnam; Bach Mai Hospital, Hanoi, Vietnam; Hanoi Medical University, Hanoi, Vietnam; Royal North Shore Hospital, Sydney, Australia; Sydney Medical School, Sydney, Australia; Guangzhou Medical University, Guangzhou, China; Chuncheon Sacred Heart Hospital, Chuncheon, South Korea; Seoul National University Hospital, Seoul, South Korea; Boramae Medical Center, Seoul, South Korea; Xinhua Hospital, Shanghai, China; Barasat Government College, Kolkata, India; Calcutta University, Kolkata, India; Presidency University, Kolkata, India; Calcutta University, Kolkata, India; Barasat Government College, Kolkata, India; Ajou University School of Medicine, Suwon, South Korea; Pusan National University Yangsan Hospital, Busan, South Korea; Pusan National University, Pusan, South Korea; Akdeniz University Faculty of Medicine, Antalya, Turkey; Al-Rashed Allergy Center, Kuwait, Kuwait; Faculty of Pharmacy, Kuwait, Kuwait; School of Pharmacy, Virginia, USA; Institute of Genomics and Integrative Biology, Delhi, India; University of Havana, Havana, Cuba; National Center of Bioproducts, Mayabeque, Cuba; National Center of Bioproducts, Bejucal, Mayabeque Cuba; Dankook University, Cheonan, South Korea; Hiroshima University, Hiroshima, Japan; Medical Research Institute of Pusan National University Hospital, Busan, South Korea; Samsung Medical Center, Seoul, South Korea; Pusan National University, Pusan, South Korea; Pusan National University Yangsan Hospital, Busan, South Korea; Morinaga Milk Industry Co., Ltd, Zama, Japan; Department of Otorhinolaryngology-Head and Neck Surgery, Dong-a University College of Medicine, Busan, South Korea; Ghent University, Ghent, Belgium; National Hospital Organization, Sagamihara National Hospital, Sagamihara, Japan; Jeju National University Hospital, Jeju, South Korea; Jeju National University, Jeju, South Korea; Jeju National University School of Medicine, Jeju, South Korea; Department of Otorhinolaryngology-Head and Neck Surgery, Dong-a University College of Medicine, Busan, South Korea; Preventive Medicine Institute, Riga, Latvia; Allergy-Centre-Charité, Berlin, Germany; Charité-Universitaetsmedizin Berlin, Berlin, Germany; Bluestone Technology Gmbh, Woerrstadt, Germany; Ajou University School of Medicine, Suwon, South Korea; National Health Insurance Corporation Ilsan Hospital, Goyang, South Korea; Jeju National University, Jeju, South Korea; Seoul National University Hospital, Seoul, South Korea; Seoul National University Bundang Hospital, Seoul, South Korea; Inha University, College of Medicine, Incheon, South Korea; Hiroshima University, Hiroshima, Japan; Hiroshima University Hospital, Hiroshima, Japan; Preventive Medicine Institute, Riga, Latvia; Sheri-Kashmir Institute of Medical Sciences(SKIMS), Srinagar, India; Skims, Srinagar, India; Centro Pediátrico Paidos – Universidad Nacional Del Este, Asunción, Paraguay; Brazilian Society, Sao Paulo, Brazil; Universidade Federal Do Paraná, Curitiba, Brazil; Hospital Dr Avelino L Castelán, Resistencia, Chaco Argentina; Hospital Universitario Metropolitano, Barranquilla, Colombia; Hospital Universitário General Calixto Garcia, Havana, Cuba; Centro De Asma y Alergia, Tagucigalpa, Honduras; Centro medica San Angel, Guadalupe, Mexico; Juan De Salazar, Asunción, Paraguay; Fellow of the American College of Allergy, Asthma & Immunology, Barbados, Cuba; State Director Gard/ARIA, ᅟ, Paraguay; University of Parana, Curitiba, Brazil; Centro De Medicina Avanzada, Santo Domingo, Dominican Republic; Rafael Barradas 1671, Montevideo, Uruguay; Pediatric Program of Asthma Prevention (PIPA), Uruguaiana, Brazil; Clínica Regional Del Este, San Francisco, Argentina; Hospital Universitario Metropolitano, Barranquilla, Colombia; National Center of Bioproducts, Mayabeque, Cuba; Cuban Society of Integr, Havana, Cuba; Brazilian Sociaty, Uruguaina, Brazil; Seoul Paik Hospital/Inje University College of Medicine, Seoul, South Korea; Seoul Paik Hospital/Inje University, Seoul, South Korea; Saitama Medical University, Saitama, Japan; Mayo Clinic College of Medicine, Rochester, USA; Brazilian Society, Sao Paulo, Brazil; Universidade Federal Do Paraná, Curitiba, Brazil; Hospital Universitario Metropolitano, Barranquilla, Colombia; National Center of Bioproducts, Mayabeque, Cuba; Juan De Salazar, Asunción, Paraguay; Fellow of the American College of Allergy, Asthma & Immunology, Barbados, Cuba; State Director Gard/ARIA, Asunción, Paraguay; Rafael Barradas 1671, Montevideo, Uruguay; Clínica Regional Del Este, San Francisco, Argentina; Servicio De Alergologia Del Hospital Issste, Veracruz, Mexico; Centro De Medicina Avanzada Dr. Abel González, Santo Domingo, Dominican Republic; Centro De Asma y Alergia, Tagucigalpa, Honduras; University of Parana, Curitiba, Brazil; University Hospital Infantil Sur, Santiago de Cuba, Cuba; Pediatric Program of Asthma Prevention (PIPA), Uruguaiana, Brazil; Centro Medica San Angel, Guadalupe, Mexico; University Hospital Calixto García, Havana, Cuba; Centro Pediátr, Asunción, Paraguay; Chulalongkorn University, Bangkok, Thailand; Stallergenes, Antony, France; Mount Sinai Beth Israel, New York, USA; Comprehensive Pharmacy Services, Memphis, USA; Skagit Valley Hospital, Mt. Vernon, USA; Mount Sinai St. Luke’s, New York, USA; Mount Sinai Roosevelt, New York, USA; Massachusetts Eye and Ear, Boston, USA; Agro-Product Safety Research Center, Beijing, China; Department of Allergy and Clinical Immunology, Guangzhou, China; State Key Laboratory of Respiratory Disease, Guangzhou, China; Guangzhou Medical University, Guangzhou, China; The First Affiliated Hospital, Guangzhou Medical University, Guangzhou, China; Ewha Womans University, Seoul, South Korea; 251 General Air Force Hospital, Athens, Greece; Hellenic Air Force, Athens, Greece; Beijing Institute of Otolaryngology, Beijing, China; Daegu Catholic University Hospital, Daegu, South Korea; Shengjing Hospital of China Medical University, Shenyang, China; Korea University, Seoul, South Korea; The University of Texas at Dallas, Richardson, USA; Korea University Medical Center, Seoul, South Korea; Capital Institute of Pediatrics, Beijing, China; Capital of Pediatric Research for Asthma Center, Beijing, China; Ewha Womans University, School of Medicine, Seoul, South Korea; Department of Computer Engineering, Hanyang University, Seoul, South Korea; Ewha Institute of Convergence Medicine, Ewha Womans University Medical Center, Seoul, South Korea; Soonchunhyang University Hospital, Asan, South Korea; Soonchunhyang University, Asan, South Korea; Beijing Shijitan Hospital, Beijing, China; Dongguk University Graduate School of Medicine, Seoul, South Korea; Dongguk University College of Medicine, Seoul, South Korea; Ewha Womans University, School of Medicine, Seoul, South Korea; Department of Computer Engineering, Hanyang University, Seoul, South Korea; Ewha Institute of Convergence Medicine, Ewha Womans University Medical Center, Seoul, South Korea; Soonchunhyang University Hospital, Asan, South Korea; Soonchunhyang University, Asan, South Korea; Department of Food and Nutrition, Kyoto, Japan; Department of Food Science and Nutrition, Kyoto, Japan; Department of Life and Living, Osaka, Japan; Beijing Institute of Otolaryngology, Beijing, China; Asan Institute for Life Sciences, Asan, South Korea; Hallym University Sacred Heart Hospital, Anyang, South Korea; Kyung Hee University, Seoul, South Korea; Bundang CHA Medical Center, CHA University School of Medicine, Seongnam, South Korea; Seoul National University Bundang Hospital, Seoul, South Korea; Pusan National University Yangsan Hospital, Busan, South Korea; Korea Cancer Center Hospital, Seoul, South Korea; University of Cincinnati College of Medicine, Cincinnati, USA; Inje University Sanggye Paik Hospital, Seoul, South Korea; Dankook University College of Medicine, Cheonan, South Korea; Asan Medical Center, University of Ulsan College of Medicine, Seoul, South Korea; Asan Institute for Life Science, Asan Medical Center, Seoul, South Korea; Childhood Asthma Atopy Center, Environmental Health Center, Asan Medical Center, University of Ulsan College of Medicine, Seoul, South Korea; Asan Medical Center, University of Ulsan College of Medicine, Seoul, South Korea; Asan Medical Center, Univers, Seoul, South Korea; St. Mary’s Hospital, Seoul, South Korea; Kyung Hee Medical Center, Seoul, South Korea; Kyung Hee University Hospital at Gangdong, Seoul, South Korea; Harbor-UCLA, Torrance, USA; Seoul St. Mary’s Hospital, Seoul, South Korea; Pucheon St.Mary’s Hospital, Seoul, South Korea; Boramae Medical Center, Seoul, South Korea; Chuncheon Sacred Heart Hospital, Chuncheon, South Korea; Utrecht University, Utrecht, Netherlands; Utrecht University, Utrecht, Netherlands; Senior Scientist, Utrecht, Netherlands; Nutricia Research, Utrecht, Netherlands; Tno, Utrecht, Netherlands; Friesland Campina, Utrecht, Netherlands; Institute for Risk Assessment Sciences, Utrecht, Netherlands; College of Medicine, the Catholic University of Korea, Seoul, South Korea; Hospital Universitari De Bellvitge, Barcelona, Spain; Danone Nutricia Research, Utrecht, Netherlands; Kk Hospital, Singapore, Singapore; King Chulalongkorn Memorial Hospital, Bangkok, Thailand; Kk Women’s and Children’s Hospital, Singapore, Singapore; Hanyang University College of Medicine, Seoul, South Korea; Hanyang University College of Medicine, Seoul, South Korea; Yonsei University College of Medicine, Seoul, South Korea; Ajou University School of Medicine, Suwon, South Korea; Asan Medical Center, University of Ulsan College of Medicine, Seoul, South Korea; Bundang Medical Center, CHA University, Seoul, South Korea; Hallym University College of Medicine, Seoul, South Korea; Ewha Womans University, School of Medicine, Seoul, South Korea; Inje University College of Medicine, Seoul, South Korea; Kangwon National University College of Medicine, Chuncheon, South Korea; Chung-Ang University Hospital, Seoul, South Korea; Seoul National University Hospital, Seoul, South Korea; Soonchunhyang University Bucheon Hospital, Asan, South Korea; University of Ulsan College of Medicine, Seoul, South Korea; Konkuk University College of Medicine, Seoul, South Korea; Cheongju St Mary’s Hospital, Seoul, South Korea; Children’s Clinic of Tartu University Hospital, Tartu, Estonia; Department of Pediatrics of University of Tartu, Tartu, Estonia; Gachon University Gil Medical Center Incheon, Seoul, South Korea; Ewha Womans University School of Medicine, Seoul, South Korea; St. Spiridon Emergency Hospital, Lasi, Romania; Korea University, Seoul, South Korea; Nippon Medical School, Tokyo, Japan; The First Hospital Affiliated to China Medical University, Shenyang, China; Tokyo Women’s Medical University, Tokyo, Japan; Tokyo General Hospital, Tokyo, Japan; Showa University School of Medicine, Tokyo, Japan; Semmelweis University, Budapest, Hungary; Hungarian Defence Forces, Medical Center, Budapest, Hungary; Scientific Research Institute of Physical-Chemical Medicine, Moscow, Russia; City Clinical Hospital N7, Moscow, Russia; Charité-Universitaetsmedizin Berlin, Berlin, Germany; School of Medicine, Ahvaz Jundishapur University of Medical Sciences, Ahvaz, Iran; School of Medicine, Aja University of Medical Sciences, Tehran, Iran; Sanggye Paik Hospital, Seoul, South Korea; Keck School of Medicine, Los Angeles, CA USA; Inha University Hospital, Incheon, South Korea; Dankook University College of Medicine, Cheonan, South Korea; Ajou University School of Medicine, Suwon, South Korea; Kosin University Gospel Hospital, Busan, South Korea; College of Medicine, Kosin University, Busan, South Korea; College of Medicine, Kosin University, Busan, South Korea; Sagamihara National Hospital, Sagamihara, Japan; Asan Medical Center, University of Ulsan College of Medicine, Seoul, South Korea; Asan Medical Center, Seoul, South Korea; Ulsan University Hospital, Ulsan, South Korea; Suwon Center for Environmental Disease and Atopy, Suwon, South Korea; Ajou University School of Medicine, Suwon, South Korea; Ehime Graduated School of Medicine, Matsuyama, Japan; Ajou University School of Medicine, Suwon, South Korea; Korea University College of Medicine, Seoul, South Korea; Hanyang University College of Medicine, Seoul, South Korea; Dankook University College of Medicine, Cheonan, South Korea; Ajou University School of Medicine, Suwon, South Korea; Ajou University Hospital, Suwon, South Korea; Ajou University School of Medicine, Suwon, South Korea; Bach Mai Hospital, Hanoi, Vietnam; Hanoi Medical University, Hanoi, Vietnam; Gil Medical Center, Incheon, South Korea; Seoul Medical Center, Seoul, South Korea; St. Paul’s Hospital, Seoul, South Korea; Kyung Hee University Hospital at Gang-Dong, Seoul, South Korea; Bucheon St. Mary’s Hospital, Bucheon, South Korea; Dankook University College of Medicine, Cheonan, South Korea; Kyungpook National University, Daegu, South Korea; Chung-Ang University Hospital, Seoul, South Korea; St. Vincent’s Hospital, Suwon, South Korea; Yonsei University, Wonju College of Medicine, Seoul, South Korea; SA Dermatology Clinic, Daejeon, South Korea; Chonnam National University Hospital, Gwangju, South Korea; Pusan National University Yangsan Hospital, Busan, South Korea; Soonchunhyang University Hospital, Asan, South Korea; Korea University, Seoul, South Korea; Chungnam National University Hospital, Daejeon, South Korea; Konkuk University Hospital, Seoul, South Korea; Incheon St.Mary’s Hospital, Incheon, South Korea; Kangnam Sacred Heart Hospital, Seoul, South Korea; Kyung Hee University Hospital at Gangdong, Seoul, South Korea; Kyung Hee Medical Center, Seoul, South Korea; National Taiwan University Hospital, Taipei City, Taiwan; National University Hospital, Singapore, Singapore; Ajou University School of Medicine, Suwon, South Korea; Novartis Pharma AG, Basel, Switzerland; Chung-Ang University Hospital, Seoul, South Korea; Seoul National University Hospital, Seoul, South Korea; Department of Mircobiology, Seoul, South Korea; Chung Ang University, Seoul, South Korea; Chung-Ang University Hospital, Seoul, South Korea; Chung Ang University, Seoul, South Korea; Health Promotion Center, Samsung Medical Center, Seoul, South Korea; Seoul Samsung Medical Clinic, Seoul, South Korea; Clean Medicine Allergy Clinic, Seoul, South Korea; Samsung Medical Center, Seoul, South Korea; Samsung Medical Center, Sungkyunkwan University School of Medicine, Seoul, South Korea; Bundang Jesaeng Hospital, Bundang, South Korea; Ansan Sarang General Hospital, Ansan, South Korea; Seoul National University Hospital, Seoul, South Korea; Pediatic SIG, Korea Institute of Drug Safety and Risk Management, Seoul, South Korea; Chungbuk National University Hispital, Cheongju, South Korea; Chonnam National University Hospital, Gwangju, South Korea; National Medical Center, Seoul, South Korea; Korea Institute of Drug Safety and Risk Management, Gyeonggi-do, South Korea; Respiratory Medicine, Delhi, India; Vallabhbhai Patel Chest Institute, University of Delhi, Delhi, India; Seoul Samsung Medical Clinic, Incheon, South Korea; Bundang Jesaeng Hospital, Gyeonggi-do, South Korea; Ansan Sarang General Hospital, Ansan, South Korea; Samsung Medical Center, Seoul, South Korea; Samsung Biomedical Research Institute, Seoul, South Korea; Samsung Medical Center, Sungkyunkwan University School of Medicine, Seoul, South Korea; Health Promotion Center, Samsung Medical Center, Seoul, South Korea; Samsung Biomedical Research Institute, Seoul, South Korea; Clean Medicine Allergy Clinic, Seoul, South Korea; Sarang General Hospital, Ansan, South Korea; Bundang Jesaeng Hospital, Bundang, South Korea; Seoul Samsung Medical Clinic, Incheon, South Korea; Samsung Medical Center, Seoul, South Korea; Samsung Medical Center, Sungkyunkwan University School of Medicine, Seoul, South Korea; Health Promotion Center, Samsung Medical Center, Seoul, South Korea; Allergy Center and Department of Clinical Research, Mie National Hospital, Tsu, Japan; Health Promotion Center, Samsung Medical Center, Seoul, South Korea; Samsung Medical Center, Seoul, South Korea; Samsung Medical Center, Sungkyunkwan University School of Medicine, Seoul, South Korea; Samsung Biomedical Research Institute, Seoul, South Korea; Seoul St. Mary’s Hospital, Seoul, South Korea; Pucheon St.Mary’s Hospital, Seoul, South Korea; St. Mary’s Hospital, Seoul, South Korea; Hospital De Zumarraga, Gipuzkoa, Spain; Hospital Universitario Donostia, Gipuzkoa, Spain; Hospital De Mendaro, Gipuzkoa, Spain; Pusan National University Hospital, Busan, South Korea; APEC Climate Center, Busan, South Korea; Saitama Medical University, Saitama, Japan; Seoul National University College of Medicine, Seoul, South Korea; Seoul National University Hospital, Seoul, South Korea; Health Promotion Center, Samsung Medical Center, Seoul, South Korea; Samsung Medical Center, Sungkyunkwan University School of Medicine, Seoul, South Korea; Samsung Biomedical Research Institute, Seoul, South Korea; Samsung Medical Center, Seoul, South Korea; Eulji University School of Medicine, Daejeon, South Korea; Hanyang University College of Medicine, Seoul, South Korea; Dankook University College of Medicine, Cheonan, South Korea; Health Promotion Center, Samsung Medical Center, Seoul, South Korea; Samsung Biomedical Research Institute, Seoul, South Korea; Samsung Medical Center, Sungkyunkwan University School of Medicine, Seoul, South Korea; Samsung Medical Center, Seoul, South Korea; Childhood Asthma Atopy Center, Environmental Health Center, Asan Medical Center, University of Ulsan College of Medicine, Seoul, South Korea; Research Center for Standardization of Allergic Diseases, University of Ulsan College of Medicine, Seoul, South Korea; Shengjing Hospital of China Medical University, Shenyang, China; Ajou University School of Medicine, Suwon, South Korea; Suwon Center for Environmental Disease and Atopy, Suwon, South Korea; Yonsei University, Wonju College of Medicine, Seoul, South Korea; Kyoto University Graduate School of Medicine, Kyoto, Japan; Sagamihara National Hospital, Sagamihara, Japan; North Shore Long Island Jewish Health System, Great Neck, USA; Korea University Medical Center, Seoul, South Korea; Richmond Pharmacology, London, England; Statistik Georg Ferber Gmbh, Riehen, Switzerland; J. Uriach y Compañía, S.a, Barcelona, Spain; Children’s Medical Center, Tehran, Iran; Sagamihara National Hospital, Sagamihara, Japan; University of Missouri-Columbia, Columbia, USA; University of Missouri, Columbia, USA; Washington University, Missouri, USA; Federal University of Parana, Curitiba, Brazil; Federal University of Sao Paulo, Sao Paulo, Brazil; Universidade Faderal De Pernambuco, Recife, Brazil; WAO Junior Member, São Paulo, Brazil; Brazilian Society, Sao Paulo, Brazil; University of Santiago De Chile (USACH), Santiago, Chile; Imib-Arrixaca Research Institute, Murcia, Spain; Arrixaca University Children’s Hospital, University of Murcia, Murcia, Spain; University of Parana, Curitiba, Brazil; Children’s Hospital at Montefiore, Bronx, USA; Montefiore Medical Center, Bronx, USA; Faculty of Medicine, Bangkok, Thailand; Chulalongkorn University, Bangkok, Thailand; Smg-Snu Boramae Medical Center, Seoul, South Korea; Seoul National University, Seoul, South Korea; Ewha Institute of Convergence Medicine, Ewha Womans University Medical Center, Seoul, South Korea; Seoul National University Hospital, Seoul, South Korea; Seoul National University Bundang Hospital, Seoul, South Korea; Sung-Ae General Hospital, Seoul, South Korea; Seoul Medical Center, Seoul, South Korea; Ajou University School of Medicine, Suwon, South Korea; Kosin University Gospel Hospital, Busan, South Korea; Busan St.Mary’s Hospital, Busan, South Korea; Yonsei University College of Medicine, Seoul, South Korea; Severance Hospital Regional Pharmacovigilance Centre, Seoul, South Korea; University of Melbourne, Royal Children’s Hospital, Melbourne, Australia; University of Manchester, Manchester, UK; Murdoch Childrens Research Institute, Parkville, Australia; University of Melbourne, Melbourne, Australia; Beijing Children’s Hospital, Beijing, China; Seoul National University, Seoul, South Korea; Seoul National University Bundang Hospital, Seoul, South Korea; Chosun University, Gwangju, South Korea; Department of Research, Faculty of Medicine, Tampico, Mexico; Ilsan Paik Hospital, Inje University College of Medicine, Goyang, South Korea; Department of Chest Medicine, Taipei Veterans General Hospital, Taipei, Taiwan; Research in Real Life, Singapore, Singapore; Mundipharma Pte Ltd, Singapore, Singapore; China-Japan Friendship Hospital, Beijing, China; Seoul National University Hospital, Seoul, South Korea; Rostov State Medical University, Rostov-on-Don, Russia; Dong-a University Hospital, Busan, South Korea; Beijing Children’s Hospital, Beijing, China; Ilsan Paik Hospital, Inje University College of Medicine, Goyang, South Korea; Department of Chest Medicine, Taipei Veterans General Hospital, Taipei, Taiwan; China-Japan Friendship Hospital, Beijing, China; Research in Real Life, Singapore, Singapore; Mundipharma Pte Ltd, Singapore, Singapore; Seoul National University Hospital, Seoul, South Korea; Ajou University Medical Center, Suwon, South Korea; Ajou University School of Medicine, Suwon, South Korea; Bushehr University of Medical Sciences, Bushehr, Iran; Department of Immunology, Asthma and Allergy, Ahvaz, Iran; Seoul National University Hospital, Seoul, South Korea; Eulji University School of Medicine, Daejeon, South Korea; Dankook University College of Medicine, Cheonan, South Korea; Hanyang University College of Medicine, Seoul, South Korea; Asan Institute for Life Sciences, Seoul, South Korea; Asan Medical Center, University of Ulsan College of Medicine, Seoul, South Korea; Yonsei University College of Medicine, Seoul, South Korea; Yonsei University, Seoul, South Korea; People’s Friendship University of Russia, Moscow, Russia; Institute of Immunophysiology, Moscow, Russia; Pacific State Medical University, Vladivostok, Russia; Severance Hospital Regional Pharmacovigilance Centre, Seoul, South Korea; Yonsei University College of Medicine, Seoul, South Korea; Dankook University, Cheonan, South Korea; Ajou University School of Medicine, Suwon, South Korea; Department of Allergy and Clinical Immunology, Ajou University School of Medicine, Suwon, South Korea; Bundang Medical Center, CHA University, Seongnam, South Korea; Beijing Children’s Hospital, Beijing, China; Nippon Medical School, Tokyo, Japan; Kyorin University Hospital, Tokyo, Japan; Tohoku University Graduate School of Medicine, Sendai, Japan; Asan Medical Center, University of Ulsan College of Medicine, Seoul, South Korea; Asan Institute for Life Sciences, Seoul, South Korea; Dankook University, Cheonan, South Korea; Department of ENT, St Johns Medical College Hospital, Bangalore, India; Department of Pediatrics, Nippon Medical School, Tokyo, Japan; Ajou University School of Medicine, Suwon, South Korea; Utrecht University, Utrecht, Netherlands; University of Salzburg, Salzburg, Austria; Christian Doppler Laboratory for Innovative Tools for the Characterization of Biosimilars, Salzburg, Austria; Division of Allergy and Immunology, Department of Internal Medicine, Yonsei University College of Medicine, Seoul, South Korea; Csir-Institute of Genomic and Integrative Biology, Delhi, India; Paul-Ehrlich-Institut, Langen, Germany; Pusan National University Yangsan Hospital, Busan, South Korea; Pusan National University, Pusan, South Korea; Samsung Medical Center, Seoul, South Korea; Health Promotion Center, Samsung Medical Center, Seoul, South Korea; Samsung Biomedical Research Institute, Seoul, South Korea; Samsung Medical Center, Sungkyunkwan University School of Medicine, Seoul, South Korea; National University of Singapore, Singapore, Singapore; National University Hospital, Singapore, Singapore; Kandang Kerbau Women’s and Children’s Hospital, Singapore, Singapore; Seoul Medical Center, Seoul, South Korea; Ajou University School of Medicine, Suwon, South Korea; Gunma University Graduate School of Medicine, Maebashi, Japan; Donguri-Kodomo Clinic, Honjo, Japan; Kitakanto Allergy Institute, Midori, Japan; Utrecht University, Utrecht, Netherlands; Nutricia Research, Utrecht, Netherlands; Seoul National University Hospital, Seoul, South Korea; Pusan National University Hospital, Busan, South Korea; Asan Medical Center, University of Ulsan College of Medicine, Seoul, South Korea; Seoul National University Bundang Hospital, Seoul, South Korea; Castle Hill Hospital, Yorkshire, UK; Chungbuk National University Hospital, Cheongju, South Korea; Ewha Womans University, School of Medicine, Seoul, South Korea; Comenius University, Bratislava, Slovakia; Ankara University Faculty of Medicine, Ankara, Turkey; Dokuz Eylul University Faculty of Medicine, Izmir, Turkey; Sureyyapasa Pulmonary Diseases Hospital and Research Center, Istanbul, Turkey; Karadeniz Technical University Faculty of Medicine, Trabzon, Turkey; Akdeniz University Hospital, Antalya, Turkey; Mardin Medical Park Hospital, Mardin, Turkey; Gaziantep University Faculty of Medicine, Gaziantep, Turkey; Uludag University Faculty of Medicine, Bursa, Turkey; Cumhuriyet University Faculty of Medicine, Sivas, Turkey; Selcuk University Faculty of Medicine, Konya, Turkey; Istanbul University Cerrahpasa Faculty of Medicine, Istanbul, Turkey; Mie National Hospital, Tsu, Japan; Dong-a University Hospital, Busan, South Korea; Department of Otorhinolaryngology-Head and Neck Surgery, Dong-a University College of Medicine, Busan, South Korea; Aga Khan University Hospital, Nairobi, Kenya; Srl Ltd, New Delhi, India; Chonnam National University Medical School, Gwangju, South Korea; Allergy Center and Department of Clinical Research, Mie National Hospital, Tsu, Japan; Hanyang University College of Medicine, Seoul, South Korea; Kawasaki Medical School, Kurashiki, Japan; Jeju National University Hospital, Jeju, South Korea; Jeju National University School of Medicine, Jeju, South Korea; Seoul National University Hospital, Seoul, South Korea; Seoul National University Bundang Hospital, Seoul, South Korea; Dankook University Hospital, Cheonan, South Korea; Jeju National University Hospital, Jeju, South Korea; Ambulanta Meznar, Celje, Slovenia; Jeju National University School of Medicine, Jeju, South Korea; The First Affiliated Hospital of Nanjing Medical University, Nanjing, China; Hanyang University College of Medicine, Seoul, South Korea; Seoul National University Hospital, Seoul, South Korea; First Affiliated Hospital of Guangzhou Medical University, Guangzhou Institute of Respiratory Diseases, Guangzhou, China; First Affiliated Hospital Guangzhou Medical University, Guangzhou, China; Beijing Children’s Hospital, Beijing, China; Kyungpook National University School of Medicine, Daegu, South Korea; Allergy-Centre-Charité, Berlin, Germany; Bluestone Technology Gmbh, Woerrstadt, Germany; Charite - Universtaetsmedizin Berlin, Berlin, Germany; Medical University of Vienna, Wien, Austria; Nicolaus Copernicus University in Torun, Ludwik Rydygier Medical College in Bydgoszcz, Bydgoszcz, Poland; Keio University Hospital, Tokyo, Japan; Chiba University, Chiba, Japan; Tokai University School of Medicine, Tokyo, Japan; Toronto Allerrgy Immunology Clinic, Toronto, Canada; Süreyyapasa Chest Disease and Chest Surgery Training and Research Hospital, Istanbul, Turkey; Jikei University Katsushika Medical Center, Tokyo, Japan; National Center for Child Health and Development, Tokyo, Japan; National Research Institute for Child Health & Development, Tokyo, Japan; University of Otago, Dunedin, New Zealand; Chung Chou University of Science and Technology, Yuanlin City, Taiwan; University of Otago, Dunedin, New Zealand; Chung Chou University of Science and Technology, Yuanlin City, Taiwan; Asia University, Taichung City, Taiwan; Show Chwan Memorial Hospital, Changhua, Taiwan; Mayo Clinic, Rochester, USA; Samsung Medical Center, Seoul, South Korea; Koseiren Takaoka Hospital, Takaoka, Japan; University of Toyama, Toyama, Japan; Toyama University School of Medicine, Toyama, Japan; University of Toyama, Toyama, Japan; Murakami Allergy and Pediatric, Toyama, Japan; Takashige Memorial Hospital, Toyama, Japan; Saiseikai Takaoka Hospital, Toyama, Japan; College of Medicine, Yeungnam University, Gyeongsan, South Korea; Ajou University School of Medicine, Suwon, South Korea; Soonchunhyang University College of Medicine, Asan, South Korea; Hallym University Sacred Heart Hospital, Anyang, South Korea; The Catholic University of Korea, Seoul, South Korea; NHIS Ilsan Hospital, Ilsan, South Korea; Asan Medical Center Seoul Korea, Seoul, South Korea; Seoul National University Hospital, Seoul, South Korea; Ajou University School of Medicine, Suwon, South Korea; Konkuk University School of Medicine, Seoul, South Korea; Inha University Hospital, Incheon, South Korea; Seoul Medical Center, Seoul, South Korea; Yonsei University College of Medicine, Seoul, South Korea; Samsung Medical Center, Seoul, South Korea; Seoul National University Bundang Hospital, Seoul, South Korea; Hallym University Medical Center, Chuncheon, South Korea; Department of Pediatrics, Korea University College of Medicine, Seoul, South Korea; Research Center for Standardization of Allergic Diseases, University, Seoul, South Korea; Ajou University Hospital, Gyeonggi-do, South Korea; Ewha Womans University School of Medicine, Seoul, South Korea; Seoul St. Mary’s Hospital, Seoul, South Korea; Sungkyunkwan University School of Medicine, Seoul, South Korea; Environmental Health Center, Kangwon National University Hospital, Chuncheon, South Korea; Hallym University Kangdong Sacred Heart Hospital, Seoul, South Korea; Konkuk University College of Medicine, Seoul, South Korea; Soonchunhyang University College of Medicine, Asan, South Korea; Samsung Changwon Hospital, Changwon, South Korea; International St. Marry’s Hospital, Incheon, South Korea; Department of Pediatrics, Korea University College of Medicine, Seoul, South Korea; Soonchunhyang University College of Medicine, Asan, South Korea; Kyungpook National University School of Medicine, Daegu, South Korea; Asan Medical Center, University of Ulsan College of Medicine, Seoul, South Korea; Seoul Metropolitan Dong-Bu Hospital, Seoul, South Korea; Soonchunhyang University College of Medicine, Asan, South Korea; Soonchunhyang Medical Center, Seoul, South Korea; Dongtan Sacred Heart Hospital, College of Medicine, Hallym University, Chuncheon, South Korea; Hallym University, College of Medicine, Chuncheon, South Korea; Pusan National University Hospital, Busan, South Korea; Seoul National University College of Medicine, Seoul, South Korea; Seoul National University, Seoul, South Korea; Health Promotion Center, Samsung Medical Center, Sungkyunkwan University School of Medicine, Seoul, South Korea; Wonkwang University Sanbon Medical Center, Iksan, South Korea; Hanyang University Kuri Hospital, Guri, South Korea; Seoul Women’s Hospital, Seoul, South Korea; Research Center for Standardization of Allergic Diseases, University of Ulsan College of Medicine, Ulsan, South Korea; Childhood Asthma Atopy Center, Environmental Health Center, Asan Medical Center, University of Ulsan College of Medicine, Seoul, South Korea; Hallym University Sacred Heart Hospital, Anyang, South Korea; Soonchunhyang University College of Medicine, Asan, South Korea; Ajou University School of Medicine, Suwon, South Korea; Seoul Medical Center, Seoul, South Korea; The Korean Academy of Asthma, Allergy and Clinical Immunology Work Group on Severe/Recalcitrant Atopic Dermatitis, Seoul, South Korea; Hallym University College of Medicine, Anyang, South Korea; Seoul National University, Seoul, South Korea; Chulalongkorn University, Bangkok, Thailand; Allergy and Clinical Immunology Research Group, Chulalongkorn University, Bangkok, Thailand; Shandong Provincial Hospital Affiliated to Shandong University, Jinan, China; Kobe City Medical Center General Hospital, Kobe, Japan; Kakogawa City West Hospital, Hyogo, Japan; Kyungpook National University School of Medicine, Daegu, South Korea; Yeungnam University College of Medicine, Daegu, South Korea; Andong Sungso Hospital, Andong, South Korea; College of Medicine, Yeungnam University, Goyang-si, South Korea; Yonsei University College of Medicine, Seoul, South Korea; Ajou University School of Medicine, Suwon, South Korea; Seoul National University, Seoul, South Korea; Hallym University Sacred Heart Hospital, Anyang, South Korea; Soonchunhyang University Hospital, Asan, South Korea; Ilsan Paik Hospital, Goyang-si, South Korea; Samsung Medical Center, Seoul, South Korea; NHIS Ilsan Hospital, Goyang-si, South Korea; Soonchunhyang University College of Medicine, Asan, South Korea; Soonchunhyang University, Asan, South Korea; Seoul National University Bundang Hospital, Seoul, South Korea; Yonsei University College of Medicine, Seoul, South Korea; Asan Medical Center Seoul Korea, Seoul, South Korea; Research Center for Standardization of Allergic Diseases, University of Ulsan College of Medicine, Ulsan, South Korea; Ewha Womans University Hospital, Seoul, South Korea; Pusan Saint Maria Hospital, Pusan, South Korea; Ajou University School of Medicine, Suwon, South Korea; Seoul Paik Hospital, Seoul, South Korea; Pusan National University Yangsan Hospital, Busan, South Korea; Medical Research Institute of Pusan National University Hospital, Busan, South Korea; Dongtan Sacred Heart Hospital, College of Medicine, Hallym University, Hwaseong, South Korea; Korea University Medical Center, Seoul, South Korea; Department of Pediatrics, Korea University College of Medicine, Seoul, South Korea; Kepco Medical Center, Seoul, South Korea; Soonchunhyang University, Asan, South Korea; Soonchunhyang University Bucheon Hospital, Asan, South Korea; Konkuk University, Seoul, South Korea; Ajou University School of Medicine, Suwon, South Korea; Pohang University of Science & Technology (POSTECH), Gyeongsangbuk-do, South Korea; Tehran University of Medical Sciences, Tehran, Iran; Usern, Tehran, Iran; Soonchunhyang University Hospital, Asan, South Korea; Soonchunhyang University, Asan, South Korea; Sagamihara National Hospital, Sagamihara, Japan; Ajou University School of Medicine, Suwon, South Korea; National University of Singapore, Singapore, Singapore; National University Health System, Singapore, Singapore; Agency of Science, Technology and Research, Singapore, Singapore; Gachon University Gil Medical Center, Incheon, South Korea; Gil Hospital, Incheon, South Korea; Gil Medical Center, Gachon Medical School, Incheon, South Korea; Gil Medical Center, Gachon Medical School, Incheon, South Korea; Gachon University Gil Medical Center, Incheon, South Korea; Gil Hospital, Incheon, South Korea; Yonsei University College of Medicine, Department of Dermatology, Seoul, South Korea; Brain Korea 21 PLUS Project for Medical Science, Yonsei University College of Medicine, Seoul, South Korea; Dae-Jeon St. Mary’s Hospital, Daejeon, South Korea; Seoul National University, Seoul, South Korea; Ach Medical University, Ulaanbaatar, Mongolia; Department of Cellular Biology and Biochemistry, School of Pharmacy and Bio-Medicine, Mongolian National University of Medical Sciences, Ulaanbaatar, Mongolia; School of Medicine, Mongolian National University of Medical Sciences, Ulaanbaatar, Mongolia; NHIS Ilsan Hospital, Ilsan, South Korea; Catholic Kwandong University College of Medicine, Ilsan, South Korea; National Health Insurance Service, Seoul, South Korea; Kangbuk Samsung Hospital, Seoul, South Korea; Guro Hospital, Seoul, South Korea; Seoul National University Bundang Hospital, Seoul, South Korea; Pusan National University Yangsan Hospital, Busan, South Korea; Korea Cancer Center Hospital, Seoul, South Korea; University of Cincinnati College of Medicine, Cincinnati, USA; Inje University Sanggye Paik Hospital, Seoul, South Korea; Hallym University Sacred Heart Hospital, Anyang, South Korea; National Health Corporation Ilsan Hospital, Seoul, South Korea; Seoul Paik Hospital, Seoul, South Korea; Bundang CHA Medical Center, CHA University School of Medicine, Seongnam, South Korea; Research Center for Standardization of Allergic Diseases, University of Ulsan College of Medicine, Seoul, South Korea; Kangbuk Samsung Hospital/Sungkyunkwan University School of Medicine, Seoul, South Korea; Ach Medical University, Ulaanbaatar, Mongolia; Department of Cellular Biology and Biochemistry, School of Pharmacy and Bio-Medicine, Mongolian National University of Medical Sciences, Ulaanbaatar, Mongolia; School of Medicine, Mongolian National University of Medical Sciences, Ulaanbaatar, Mongolia; Mnr Medical College, Hyderabad, India; Sai Hospital, Guntur, India; Capital Institute of Pediatrics, Beijing, China; Affiliated Children Hospital of Capital Institute of Pediatrics, Beijing, China; Guro Hospital, Seoul, South Korea; Korea University, Seoul, South Korea; Korea University Medical Center, Seoul, South Korea; Korea University Guro Hospital, Seoul, South Korea; Ghent University, Ghent, Belgium; Soonchunhyang University Bucheon Hospital, Asan, South Korea; University of Pittsburgh, Pittsburgh, USA; Soonchunhyang University Bucheon Hospital, Asan, South Korea; Wonkwang University Sanbon Medical Center, Iksan, South Korea; Yeungnam University College of Medicine, Daegu, South Korea; College of Medicine, Yeungnam University, Daegu, South Korea; Andong Sungso Hospital, Andong, South Korea; Ewha Womans University, School of Medicine, Seoul, South Korea; Ewha Institute of Convergence Medicine, Ewha Womans University Medical Center, Seoul, South Korea; Department of Computer Engineering, Hanyang University, Seoul, South Korea; Samitivej International Children’s Hospital, Bangkok Hospital Group, Bangkok, Thailand; Chungnam National University Hospital, Daejeon, South Korea; Chungnam National University School of Medicine, Daejeon, South Korea; College of Medicine, the Catholic University of Korea, Seoul, South Korea; Seoul National University Bundang Hospital, Seoul, South Korea; Seoul Paik Hospital, Seoul, South Korea; Pusan National University Yangsan Hospital, Busan, South Korea; Inje University Sanggye Paik Hospital, Seoul, South Korea; Korea Cancer Center Hospital, Seoul, South Korea; Hallym University Sacred Heart Hospital, Anyang, South Korea; National Health Corporation Ilsan Hospital, Seoul, South Korea; Bundang CHA Medical Center, CHA University School of Medicine, Seongnam, South Korea; Research Center for Standardization of Allergic Diseases, University of Ulsan College of Medicine, Seoul, South Korea; University of Cincinnati College of Medicine, Cincinnati, USA; Guro Hospital, Seoul, South Korea; Kangbuk Samsung Hospital/Sungkyunkwan University School of Medicine, Seoul, South Korea; Kangbuk Samsung Hospital, Seoul, South Korea; Derpartment of Dermatology, National Medical Center, Seoul, South Korea; Korea University, Seoul, South Korea; Philippine General Hospital, Manila, Philippines; Ehime Prefectural Niihama Hospital, Ehime, Japan; Yonsei University College of Medicine, Seoul, South Korea; Jeju National University, Jeju, South Korea; Jeju National University School of Medicine, Jeju, South Korea; Mongolian National University of Medical Sciences, Ulaanbaatar, Mongolia; Department of Cellular Biology and Biochemistry, School of Pharmacy and Bio-Medicine, Mongolian National University of Medical Sciences, Ulaanbaatar, Mongolia; Department of Physiology and Molecular Biology, Ach Medical University, Ulaanbaatar, Mongolia; School of Medicine, Mongolian National University of Medical Sciences, Ulaanbaatar, Mongolia; Ach Medical University, Ulaanbaatar, Mongolia; Mnums, Ulaanbaatar, Mongolia; D’allergologie Et Exploration Du Sommeil, Saint Denis, Reunion; Capital Institute of Pediatrics, Beijing, China; Nippon Medical School, Tokyo, Japan; School of Medicine, Mongolian National University of Medical Sciences, Ulaanbaatar, Mongolia; Department of Physiology and Molecular Biology, Ach Medical University, Ulaanbaatar, Mongolia; Department of Cellular Biology and Biochemistry, School of Pharmacy and Bio-Medicine, Mongolian National University of Medical Sciences, Ulaanbaatar, Mongolia; Mnums, Ulaanbaatar, Mongolia; National Cheng Kung University, Tainan, Taiwan; National Cheng Kung University Hospital, Tainan, Taiwan; The Chinese University of Hong Kong, Hong Kong, Hong Kong; University of California, Davis, USA; Seoul National University Hospital, Seoul, South Korea; Seoul National University, Seoul, South Korea; Seoul National University Boramae Hospital, Seoul, South Korea; Dongtan Sacred Heart Hospital, College of Medicine, Hallym University, Chuncheon, South Korea; CHA University School of Medicine, Gyeonggi-do, South Korea; Bundang CHA Medical Center, CHA University School of Medicine, Seongnam, South Korea; Renmin Hospital, Wuhan University, Wuhan, China; Singapore General Hospital, Singapore, Singapore; Asan Medical Center, Seoul, South Korea; Hallym University Sacred Heart Hospital, Anyang, South Korea; Pusan National University Yangsan Hospital, Busan, South Korea; Seoul National University Bundang Hospital, Seoul, South Korea; Bundang CHA Medical Center, CHA University School of Medicine, Seongnam, South Korea; University of Cincinnati College of Medicine, Ohio, USA; Korea Cancer Center Hospital, Seoul, South Korea; Dankook University College of Medicine, Cheonan, South Korea; Sanggye Paik Hospital, Seoul, South Korea; Research Center for Standardization of Allergic Diseases, University of Ulsan College of Medicine, Dong-gu, South Korea; St. John’s Research Institute, Bengaluru, India; St. John’s Medical College Hospital, Bangalore, India; Nippon Medical School, Tokyo, Japan; Chungbuk National University Hostpital, Jeonju-si, South Korea; Pediatic SIG, Korea Institute of Drug Safety and Risk Management, Seoul, South Korea; Tehran University of Medical Sciences, Tehran, Iran; Tepecik Training and Research Hospital, Izmir, Turkey; Tepecik Training and Research Hospital, Allergy and Clinical Immunology, Izmir, Turkey; Near East University, Nicosia, Cyprus; Prince of Wales Hospital, Hong Kong, Hong Kong; The Chinese University of Hong Kong, Hong Kong, Hong Kong; St. John’s Research Institute, Bangalore, India; St. John’s Medical College Hospital, Bangalore, India; Nippon Medical School, Tokyo, Japan; National Medical Center, Seoul, South Korea; Korea University, Seoul, South Korea; Korea University Anam Hospital, Seoul, South Korea; Gachon University Gil Medical Center South Korea, Incheon, South Korea; National Research Center - Institute of Immunology, Moscow, Russia; Metchnikov’s Research Institute for Vaccines and Sera, Moscow, Russia; Department of Paediatrics, Hospital Serdang, Jalan Puchong, Kajang, 43000 Malaysia; Department of Paediatrics, Faculty of Medicine and Health Sciences, Universiti Putra Malaysia, 43400 Serdang, Malaysia; Human Development and Health Unit, Faculty of Medicine, University of Southampton, Southampton, SO166YD UK; Hospital Serdang, Kajang, Selangor Malaysia; Ewha Institute of Convergence Medicine, Ewha Womans University Medical Center, Seoul, South Korea; Ewha Womans University Mokdong Hospital, Seoul, South Korea; Ewha Womans University Mokdong Hospital South Korea, Seoul, South Korea; Chungbuk National University Hospital South Korea, Cheongju, South Korea; Hospital, Busan, South Korea; Kyungpook National University School of Medicine, Daegu, South Korea; Pusan National University Hospital, Busan, South Korea; Yangsan Pusan National University Hospital, Yangsan, South Korea; Seoul National University Hospital, Seoul, South Korea; Gachon University Gil Medical Center, Incheon, South Korea; Haeundae Paik Hospital, Inje University College of Medicine, Busan, South Korea; Seoul National University Bundang Hospital, Seoul, South Korea; Jeju National University School of Medicine, Jeju, South Korea; Dankook University College of Medicine, Cheonan, South Korea; Chonnam National University Medical School, Gwangju, South Korea; Seoul National University College of Medicine, Seoul, South Korea; National Research Center - Institute of Immunology of Federal Medico-Biology Agency of Russia, Moscow, Russia; Metchnikov’s Research Institute for Vaccines and Sera, Russian Academy of Medical Scienses, Moscow, Russia; Chungnam National University School of Medicine, Daejeon, South Korea; Chungnam National University Hospital South Korea, Daejeon, South Korea; Seoul National University Bundang Hospital, Seoul, South Korea; Seoul National University Hospital South Korea, Seoul, South Korea; Chosun University College of Medicine, Gwangju, South Korea; Dankook University College of Medicine, Cheonan, South Korea; Asan Medical Center, University of Ulsan College of Medicine, Seoul, South Korea; Pusan National University Hospital, Busan, South Korea; Ewha Institute of Convergence Medicine, Ewha Womans University Medical Center, Seoul, South Korea; Ewha Womans University Mokdong Hospital, Seoul, South Korea; Gachon University Gil Medical Center, Incheon, South Korea; Semey State Medical University, Semey, Kazakhstan; Prince Naif Center for Immunology Research, College of Medicine, King Saud University, Riyadh, Saudi Arabia; King Saud University, Riyadh, Saudi Arabia; Prince Naif Center for Immunology Research and Asthma Research Chair, Department of Pediatrics, College of Medicine, King Saud University Saudi Arabia, Riyadh, Saudi Arabia; University Sleep Disorders Center, College of Medicine, King Saud University, Riyadh, Saudi Arabia; Gwangju Veterans Hospital, Gwangju, South Korea; Chonnam National University Medical School, Gwangju, South Korea; Chonnam National University Hospital, Gwangju, South Korea; Samsung Changwon Hospital, Sungkyunkwan University School of Medicine, Changwon, South Korea; Chungbuk National University Hospital, Cheongju, South Korea; Juntendo University Faculty of Medicine, Tokyo, Japan; Juntendo University Shizuoka Hospital, Tokyo, Japan; Hamad General Hospital, Doha, Qatar; Hamad Medical Corporation Qatar, Doha, Qatar; Kangdong Sacred Heart Hospital, Seoul, South Korea; CHA University School of Medicine, Seongnam, South Korea; Dongtan Sacred Heart Hospital, College of Medicine, Hallym University, Hwaseong, South Korea; Myongji General Hospital, Goyang, South Korea; Kyunghee University Hospital at Gangdong, Seoul, South Korea; Lofarma S.p.a, Milan, Italy; Ospedale Di Cuasso Al Monte, Varese, Italy; Allergy and Respiratory Diseases Clinic, Genoa, Italy; The First Affiliated Hospital of Guangzhou Medical University, Guangzhou, China; First Affiliated Hospital of Guangzhou Medical University, Guangzhou Institute of Respiratory Diseases, Guangzhou, China; Agency for Science, Technology and Research (A*STAR), Singapore, Singapore; University of Eastern Piedmont, Novara, Italy; University of Eastern Piedmont “Amedeo Avogadro”, Novara, Italy; Chiba University, Chiba, Japan; Graduate School of Medicine, Chiba University, Chiba, Japan; Unknown, Chiba, Japan; Airlangga University/Dr Soetomo Hospital, Surabaya, Indonesia; West Nusa Tenggara General Hospital Indonesia, Mataram, Indonesia; Tokai University School of Medicine, Kanagawa, Japan; Jikei University, Tokyo, Japan; Mayo Clinic, Rochester, USA; Islamic Azad University- Najafabad Branch, Isfahan, Iran; University of Tennessee/Le Bonheur Children’s Hospital’s, Memphis, USA; Department of Pediatrics, Kangwon National University, Chuncheon, South Korea; Soonchunhyang University College of Medicine, Asan, South Korea; Gangneung Asan Hospital, Gangneung, South Korea; Gangnam CHA Hospitatl, Seoul, South Korea; Hallym University Sacred Heart Hospital, Anyang, South Korea; Busan St. Mary’s Medical Center, Busan, South Korea; Seoul Paik Hospital, Seoul, South Korea; Sanggye Paik Hospital, Seoul, South Korea; Kangdong Sacred Heart Hospital, Seoul, South Korea; Inha University Hospital, Incheon, South Korea; Environmental Health Center for Allergic Rhinitis, Incheon, South Korea; The Catholic University of Korea, Seoul, South Korea; Seoul National University Hospital South Korea, Seoul, South Korea; University of the Philippines Philippine General Hospital, Manila, Philippines; First Affiliated Hospital Guangzhou Medical University, Guangzhou, China; First Affiliated Hospital of Guangzhou Medical University, Guangzhou Institute of Respiratory Diseases China, Guangzhou, China; Dr Behcet Uz Children’s Hospital Turkey, Izmir, Turkey; Dokuz Eylul University Hospital, Izmir, Turkey; Toronto Allergists, Toronto, Canada; National Cheng Kung University Hospital, Tainan, Taiwan; National Cheng Kung University, Tainan, Taiwan; Medical University of South Carolina, Charleston, USA; Medical Professional Institute, Metylovice, Czech Republic; Ankara Training and Research Hospital, Ankara, Turkey; Samsung Medical Center, Seoul, South Korea; Seoul National University Hospital, Seoul, South Korea; Seoul National University Bundang Hospital, Seoul, South Korea; Yangsan Pusan National University Hospital, Busan, South Korea; Pogunhan Mom Hospital, Seoul, South Korea; University of Ghent, Ghent, Belgium; The Institute of Basic Medical Sciences of National Cheng Kung University, Tainan, Taiwan; National Cheng Kung University Hospital, Tainan, Taiwan; Brighton and Sussex Medical School, Brighton, UK; Korea University, Seoul, South Korea; Department of Dermatology, Yonsei University College of Medicine, Seoul, South Korea; Brain Korea 21 PLUS Project for Medical Science, Yonsei University College of Medicine, Seoul, South Korea; Korea University, Seoul, South Korea; Hôpital Tenon, Paris, France; Hôpital Henri Mondor, Créteil, France; Hôpital Saint-Camille, Bry-sur-marne, France; Cabinet De Pneumologie France, La Varenne Saint-Hilaire, France; Murdoch Childrens Research Institute, Parkville, Australia; Imperial College London, London, UK; Universiti Putra Malaysia (UPM), Serdang, Malaysia; Gunma University Graduate School of Medicine, Maebashi, Japan; Yonsei University College of Medicine, Department of Dermatology, Seoul, South Korea; Brain Korea 21 PLUS Project for Medical Science, Yonsei University College of Medicine, Seoul, South Korea; College of Health Sciences, Wonju, South Korea; Chung-Ang University Hospital, Seoul, South Korea; Institute of Lifestyle Medicine, Wonju, South Korea; M&d, Inc, Wonju, South Korea; Yonsei University Wonju College of Medicine, Wonju, South Korea; Hallym University Sacred Heart Hospital, Anyang, South Korea; Hallym University Dongtan Sacred Heart Hospital, Chuncheon, South Korea; Seoul National University Hospital, Seoul, South Korea; Department of Pediatrics, Childhood Asthma Atopy Center, Environmental Health Center, Asan Medical Center, University of Ulsan College of Medicine, Seoul, South Korea; Asan Institute for Life Science, University of Ulsan College of Medicine, Ulsan, South Korea; Bundang CHA Medical Center, CHA University School of Medicine, Seongnam, South Korea; Asan Medical Center, Seoul, South Korea; Department of Life Sciences, Hallym University, Chuncheon, South Korea; Asan Institute for Life Science, Asan Medical Center, Seoul, South Korea; Asan Medical Center, University of Ulsan College of Medicine, Seoul, South Korea; Samsung, Seoul, South Korea; CHA University School of Medicine, Seongnam, South Korea; University of Cincinnati College of Medicine, Cincinnati, USA; Department of Molecular Pathology, Tokyo Medical University, Tokyo, Japan; Department of Internal Medicine, Kyungpook National University School of Medicine, Daegu, South Korea; Department of Experimental Pathology, Graduate School of Comprehensive Human Science, University of Tsukuba, Tsukuba, Japan; The Institute of Medical Science, Tokyo, Japan; Division of Clinical Immunology, Major of Advanced Biomedical Applications, Graduate School of Comprehensive Human Science, University of Tsukuba, Tsukuba, Japan; Institute for the 3Rs, Department of Laboratory Animal Medicine, College of Veterinary Medicine, Konkuk University, Seoul, South Korea; Animal Research Center, Tokyo Medical University, Tokyo, Japan; Department of Internal Medicine, Catholic University, Seoul, South Korea; University of Illinois at Urbana-Champaign, Urbana-Champaign, USA; King Abdullah University of Science and Technology, Thuwal, Saudi Arabia; National University Health System, Singapore, Singapore; National University of Singapore, Singapore, Singapore; Kyung Hee Medical Center, Seoul, South Korea; Kyung Hee University Hospital at Gangdong, Seoul, South Korea; Soonchunhyang University Gumi Hospital South Korea, Gyeongsangbuk-do, South Korea; Haeundae Paik Hospital, Inje University College of Medicine, Busan, South Korea; Asan Medical Center, Seoul, South Korea; Asan Medical Center, University of Ulsan College of Medicine, Seoul, South Korea; Harvard T.H. Chan School of Public Health, Boston, USA; Asan Institute for Life Sciences, Seoul, South Korea; Asan Medical Center, Seoul, South Korea; Hanyang University College of Medicine, Soeul, South Korea; Korea University, Seoul, South Korea; CHA Bundang Medical Center, CHA University, Bundang-gu, South Korea; Dongtan Sacred Heart Hospital, College of Medicine, Hallym University, Hwaseong, South Korea; Bundang CHA Medical Center, CHA University School of Medicine, Seongnam, South Korea; CHA University School of Medicine, Seongnam, South Korea; Bundang Medical Center, CHA University, Seongnam, South Korea; Dong-a University School of Medicine, Busan, South Korea; Inha University Hospital, Incheon, South Korea; Inha University Hospital, Incheon, South Korea; The Affiliated Hospital of Qingdao University, Qingdao, China; Seoul National University Boramae Hospital, Seoul, South Korea; Kangdong Sacred Heart Hospital, Seoul, South Korea; Sanggye Paik Hospital, Seoul, South Korea; Ulsan University, Ulsan, South Korea; Inje University Sanggye Paik Hospital, Seoul, South Korea; Mie National Hospital, Tsu, Japan; Guangzhou Institute of Respiratory Disease, Guangzhou, China; Department of Allergy and Clinical Immunology, State Key Laboratory of Respiratory Disease, Guangzhou, China; State Key Laboratory of Respiratory Disease, the First Affiliated Hospital, Guangzhou Medical University, Guangzhou, China; Imperial College of Science, Tech. & Medicine, London, UK; State Key Laboratory of Respiratory Disease, Guangzhou, China; The Chinese Unbiversity of Hong Kong, Hong Kong, Hong Kong; Prince of Wales Hospital, Hong Kong, Hong Kong; Catholic University Hospital of Daegu, Daegu, South Korea; Dong-a University School of Medicine, Busan, South Korea; Sagamihara National Hospital, Sagamihara, Japan; Guangzhou Institute of Respiratory Disease, Guangzhou, China; Guangzhou Medical University, Guangzhou, China; First Affiliated Hospital Guangzhou Medical University, Guangzhou, China; Kangdong Sacred Heart Hospital, Seoul, South Korea; Dong-a University School of Medicine, Busan, South Korea; Dong-a University Hospital Regional Pharmacovigilance Center, Busan, South Korea; Asan Institute for Life Sciences, University of Ulsan College of Medicine, Seoul, South Korea; Asan Medical Center, Seoul, South Korea; Department of Pediatrics, Childhood Asthma Atopy Center, Environmental Health Center, Asan Medical Center, University of Ulsan College of Medicine, Ulsan, South Korea; Hallym University Sacred Heart Hospital, Anyang, South Korea; CHA University School of Medicine, Seoul, South Korea; Samsung Medical Center, Seoul, South Korea; Yonsei University College of Medicine, Seoul, South Korea; Hallym University College of Medicine, Anyang, South Korea; Ajou University College of Medicine, Suwon, South Korea; Sewon Infant Child Development Center, Seoul, South Korea; Hanshin University, Osan, South Korea; University of Ulsan College of Medicine, Seoul, South Korea; Yonsei University College of Medicine, Seoul, South Korea; Hallym Sacred Heart Kangnam Hospital, Seoul, South Korea; Yonsei University College of Medicine, Seoul, South Korea; Hallym Sacred Heart Kangnam Hospital, Seoul, South Korea; Kangbuk Samsung Hospital/Sungkyunkwan University School of Medicine, Seoul, South Korea; Seoul National University Bundang Hospital, Seoul, South Korea; Seoul Paik Hospital, Seoul, South Korea; Pusan National University Yangsan Hospital, Busan, South Korea; Inje University Sanggye Paik Hospital, Seoul, South Korea; Korea Cancer Center Hospital, Seoul, South Korea; Hallym University Sacred Heart Hospital, Anyang, South Korea; National Health Corporation Ilsan Hospital, Seoul, South Korea; Bundang CHA Medical Center, CHA University School of Medicine, Seongnam, South Korea; Asan Medical Center, Seoul, South Korea; University of Cincinnati College of Medicine, Cincinnati, USA; Guro Hospital, Seoul, South Korea; Kangbuk Samsung Hospital, Seoul, South Korea; Catholic University Hospital of Daegu, Daegu, South Korea; Korea Meteorological Agency, Jeju, South Korea; Samsung Medical Center, Seoul, South Korea; Sungkyunkwan University School of Medicine, Suwon, South Korea; National Formosa University, Yunlin, Taiwan; National Cheng Kung University Hospital, Tainan, Taiwan; The First Affiliated Hospital of Guangzhou Medical University, Guangzhou, China; First Affiliated Hospital Guangzhou Medical University, Guangzhou, China; Guangzhou Yuexiu District Maternal and Child Health Care Hospital, Guangzhou, China; First Affiliated Hospital of Guangzhou Medical University, Guangzhou Institute of Respiratory Diseases, Guangzhou, China; Kosin University Gospel Hospital, Busan, South Korea; Asan Medical Center, University of Ulsan College of Medicine, Seoul, South Korea; G-SAM Medical Center, Gunpo, South Korea; DNA Link, Seoul, South Korea; Hiroshima University, Hiroshima, Japan; National Center for Child Health and Development, Tokyo, Japan; Graduate School of Medicine, Chiba University, Chiba, Japan; Tokyo Metropolitan Children’s Hospital, Tokyo, Japan; Tokyo Allergy and Respiratory Disease Research Institute, Tokyo, Japan; Soonchunhyang University, Asan, South Korea; Dong-a University School of Medicine, Busan, South Korea; Dong-a University Hospital Regional Pharmacovigilance Center, Busan, South Korea; Dong-a University Hospital, Busan, South Korea; Kk Women’s and Children’s Hospital, Singapore, Singapore; Danone Nutricia, Singapore, Singapore; Nutricia Research, Utrecht, Netherlands; Utrecht University, Utrecht, Netherlands; Dong-a University School of Medicine, Busan, South Korea; Dong-a University Hospital Regional Pharmacovigilance Center, Busan, South Korea; Institute of Allergy and Clinical Immunology, Seoul National University Medical Research Center, Seoul, South Korea; Seoul National University Hospital, Seoul, South Korea; Seoul National University College of Medicine, Seoul, South Korea; Division of Allergy and Immunology, Department of Internal Medicine, Yonsei University College of Medicine, Seoul, South Korea; Korea University Medical Center, Seoul, South Korea; Korea University, Seoul, South Korea; Korea University Anam Hospital, Seoul, South Korea; Korea University Guro Hospital, Seoul, South Korea; Seoul National University Hospital, Seoul, South Korea; Seoul National University Bundang Hospital, Seoul, South Korea; Seoul National University College of Medicine, Seoul, South Korea; Seoul National University Medical Research Center, Seoul, South Korea; Pogunhan Mom Hospital, Seoul, South Korea; Dong-a University School of Medicine, Busan, South Korea; Dong-a University Hospital Regional Pharmacovigilance Center, Busan, South Korea; Prince of Wales Hospital, Hong Kong, Hong Kong; Yong Loo Lin School of Medicine, Singapore, Singapore; Samsung Medical Center, Seoul, South Korea; Samsung Seoul Hospital, Seoul, South Korea; Dong-a University School of Medicine, Busan, South Korea; Seoul National University Medical Research Center, Seoul, South Korea; Chungbuk National University Hospital, Cheongju, South Korea; Seoul National University Bundang, Seoul, South Korea; Institute of Allergy and Clinical Immunology, Seoul National University Medical Research Center, Seoul, South Korea; Seoul National University Hospital, Seoul, South Korea; Seoul National University College of Medicine, Seoul, South Korea; Division of Allergy and Immunology, Department of Internal Medicine, Yonsei University College of Medicine, Seoul, South Korea; Dong-a University School of Medicine, Busan, South Korea; Dong-a University Hospital Regional Pharmacovigilance Center, Busan, South Korea; National Cheng Kung University, Tainan, Taiwan; National Cheng Kung University Hospital, Tainan, Taiwan; Ulsan University Hospital, Ulsan, South Korea; Ewha Womans University, Seoul, South Korea; Dankook University College of Medicine, Cheonan, South Korea; Seoul National University College of Medicine, Seoul, South Korea; Showa University School of Medicine, Tokyo, Japan; Nippon Medical School, Tokyo, Japan; Tokyo General Hospital, Tokyo, Japan; Dong-a University School of Medicine, Busan, South Korea; Haeundae Paik Hospital, Inje University College of Medicine, Busan, South Korea; Gilhospital, Incheon, South Korea; Inha University Hospital, Incheon, South Korea; Ewha Womans Mokdong Hospital, Seoul, South Korea; Ewha Womans University Mokdong Hospital, Seoul, South Korea; Ulsan University Hospital, Ulsan, South Korea; Ewha Womans University, Seoul, South Korea; Dankook University College of Medicine, Cheonan, South Korea; Seoul National University College of Medicine, Seoul, South Korea; Soonchunhyang University Bucheon Hospital, Asan, South Korea; Soonchunhyang University, Asan, South Korea; Konkuk University, Seoul, South Korea; Kangwon National University College of Medicine, Chuncheon, South Korea; Ewha Womans Mokdong Hospital, Seoul, South Korea; Ewha Womans University Mokdong Hospital, Seoul, South Korea; Pusan National University, Pusan, South Korea; Pusan National University Yangsan Hospital, Busan, South Korea; Sydney Medical School - Northern, University of Sydney, Sydney, Australia; The University of Sydney, Sydney, Australia; Royal North Shore Hospital, Sydney, Australia; Griffith University, Nathan, Australia; Chonnam National University Medical School, Gwangju, South Korea; St. Vincent’s Hospital, Suwon, South Korea; Osaka University, Suita, Japan; Kyung Hee Medical Center, Seoul, South Korea; Kyung Hee University Hospital at Gangdong, Seoul, South Korea; College of Medicine, National Cheng Kung University, Taiwan, Taiwan; Department of Pediatrics, Childhood Asthma Atopy Center, Environmental Health Center, Asan Medical Center, University of Ulsan College of Medicine, Seoul, South Korea; National Cheng Kung University, Tainan, Taiwan; National Cheng Kung University Hospital, Tainan, Taiwan; Hallym University Dongtan Sacred Heart Hospital, Chuncheon, South Korea; Hallym University Sacred Heart Hospital, Chuncheon, South Korea; Institute of Allergy and Clinical Immunology, Seoul National University Medical Research Center, Seoul, South Korea; Seoul National University College of Medicine, Seoul, South Korea; Chungbuk National University Hospital, Cheongju, South Korea; Seoul National University Hospital, Seoul, South Korea; Medical Research Institute of Pusan National University Hospital, Busan, South Korea; Seoul National University Medical Research Center, Seoul, South Korea; Division of Allergy and Immunology, Department of Internal Medicine, Seoul, South Korea; Tokyo Medical and Dental Unibersity, Tokyo, Japan; Sureyyapasa Chest Diseases and Chest Surgery Training Hospital, Istanbul, Turkey; Süreyyapa°a Chest Disease and Chest Surgery Training and Research Hospital, Istanbul, Turkey; Imperial College London, London, UK; Murdoch Childrens Research Institute, Parkville, Australia; Universiti Putra Malaysia (UPM), Serdang, Malaysia; Seoul National University Medical Research Center, Seoul, South Korea; Institute of Allergy and Clinical Immunology, Seoul National University Medical Research Center, Seoul, South Korea; Chungbuk National University Hospital, Cheongju, South Korea; Seoul National University Bundang Hospital, Seoul, South Korea; Seoul National University College of Medicine, Seoul, South Korea; Seoul National University Hospital, Seoul, South Korea; Sung-Ae General Hospital, Seoul, South Korea; Seoul Medical Center, Seoul, South Korea; UST Hospital, Manila, Philippines; Yonsei University College of Medicine, Seoul, South Korea; Tan Tock Seng Hospital, Singapore, Singapore; Hallym University Sacred Heart Hospital, Anyang, South Korea; The Bambino Gesù Children’s Research Hospital, Vatican City, Italy; Miltenyi Biotech, Bergisch Gladbach, Germany; Ospedale Pediatrico Bambino Gesù, Vatican City, Italy; Barrackpore Population Health Research Foundation, Kolkata, India; Seoul National University Hospital, Seoul, South Korea; Pusan National University Hospital, Busan, South Korea; Seoul National University College of Medicine, Seoul, South Korea; Pogunhan Mom Hospital, Seoul, South Korea; Seoul National University Medical Research Center, Seoul, South Korea; Seoul National University Bundang Hospital, Seoul, South Korea; Chiba University, Chiba, Japan; Korea University College of Medicine, Seoul, South Korea; Ajou University School of Medicine, Suwon, South Korea; Hallym University, College of Medicine, Chuncheon, South Korea; Hallym University Sacred Heart Hospital, Anyang, South Korea; National Hospital Organization Fukuyama Medical Center, Fukuyama, Japan; Okayama University Graduate School of Medicine, Dentistry and Pharmaceutical Sciences, Okayama, Japan; Presbyterian Medical Center, Jeonju, South Korea; Pediatrics, Presbyterian Medical Center, Jeonju, South Korea; Asan Medical Center, Seoul, South Korea; Hallym University Sacred Heart Hospital, Anyang, South Korea; Chulalongkorn University, Bangkok, Thailand; Mahidol University, Salaya, Thailand; University, Bangalore, India; Korea Meteorological Agency, Seoul, South Korea; Hanyang University Kuri Hospital, Guri, South Korea; Korea Meteorological Administration, Seoul, South Korea; Department of Pediatrics, Childhood Asthma Atopy Center, Environmental Health Center, Asan Medical Center, University of Ulsan College of Medicine, Ulsan, South Korea; CHA Medical Center, Seoul, South Korea; Asan Medical Center, Seoul, South Korea; Asan Institute for Life Science, Asan Medical Center, Seoul, South Korea; Hallym University Sacred Heart Hospital, Anyang, South Korea; Samsung Medical Center, Seoul, South Korea; Yonsei University College of Medicine, Seoul, South Korea; Seoul National University College of Medicine, Seoul, South Korea; Korea National Institute of Health, Seoul, South Korea; Seoul National University Hospital, Seoul, South Korea; Tokyo University of Agriculture and Technology, Tokyo, Japan; Institute of Medical Statistics, Informatics and Epidemiology, University Hospital of Cologne, Cologne, Germany; Lofarma S.p.a., Milan, Italy; Seoul National University College of Medicine, Seoul, South Korea; Seoul National University Hospital, Seoul, South Korea; Seoul National University Bundang Hospital, Seoul, South Korea; Smg-Snu Boramae Medical Center, Seoul, South Korea; Harvard T.H. Chan School of Public Health, Boston, USA; Korea National Institute of Health, Korea Center for Disease Control and Prevention, Seoul, South Korea; Tan Tock Seng Hospital, Singapore, Singapore; Inje University College of Medicine, Gyeongsangnam-do, South Korea; Busan Paik Hospital, Inje University College of Medicine, Busan, South Korea; Seoul National University Medical Research Center, Seoul, South Korea; The Armed Forces Capital Hospital, Bundang-gu, South Korea; Chung-Ang University Hospital, Seoul, South Korea; Chungbuk National University Hospital, Cheongju, South Korea; Seoul National University Hospital, Seoul, South Korea; Korea Institute of Drug Safety and Risk Management, Seoul, South Korea; Laboratoire Protec’som, Valognes, France; Allergy Therapeutics, Worthing, UK; Medical University of Lodz, Lodz, Poland; National Centre for Sports Medicine, Warsaw, Poland; Chonbuk National University Medical School/Hospital, Jeonju, South Korea; Hallym University Sacred Heart Hospital, Anyang, South Korea; The First Affiliated Hospital of Guangxi Medical University, Nanning, China; Department of Pediatrics, the First Affiliated Hospital of Guangxi Medical University, Nanning, China; Indraprastha Apollo Hospitals, New Delhi, India; Cipto Mangunkusumo Hospital, Nanning, Indonesia; Allergy Therapeutics, Worthing, UK; Chung-Ang University Hospital, Seoul, South Korea; Allergy Therapeutics, Worthing, UK; Center for Research and Training in Skin Diseases and Leprosy, Tehran University of Medical Sciences, Tehran, Iran; Department of Cell and Molecular Biology, Azad University of Ashkezar Branch, Tehran, Iran; Department of Immunology, Tehran University of Medical Sciences, Tehran, Iran; Molecular Immunology Research Center (MIRC), Tehran University of Medical Sciences, Tehran, Iran; Pusan National University, Pusan, South Korea; Busan Paik Hospital, Inje Universit College of Medicine, Busan, South Korea; Department of Pediatrics, Busan Paik Hospital, Inje Universit College of Medicine, Busan, Korea; Ehime Graduated School of Medicine, Matsuyama, Japan; Chung-Ang University Hospital, Seoul, South Korea; Mofid Children’s Hospital, Shahid Beheshti University of Medical Sciences, Tehran, Iran; Hazrate Rasool Hospital, Iran University of Medical Scienses, Tehran, Iran; Bahrami Childrens’ Hospital, Tehran University of Medical Sciences, Tehran, Iran; Hazrate Rasool Hospital, Iran University of Medical Sciences, Tehran, Iran; Chung-Ang University Hospital, Seoul, South Korea; Wroclaw Medical University, Wroclaw, Poland; Wroclaw Medical University, National Institute of Public Health, Wroclaw, Poland; Fukuoka National Hospital, Fukuoka, Japan; Faculty of Medicine Universitas Padjadjaran/Dr. Hasan Sadikin General Hospital, Bandung, Indonesia; Osaka Prefectural Hospital Organization Osaka Prefectural Medical Center for Respiratory and Allergic Diseases, Osaka, Japan; Soonchunhyang University, Asan, South Korea; Soonchunhyang University Bucheon Hospital, Asan, South Korea; Konkuk University Hospital, Seoul, South Korea; Korea University Hospital, Seoul, South Korea; Faculty of Medicine Universitas Padjadjaran/Dr. Hasan Sadikin General Hospital, Bandung, Indonesia; Dr. Hasan Sadikin General Hospital, Bandung, Indonesia; Dong-a University School of Medicine, Busan, South Korea; Busan Paik Hospital, Inje University College of Medicine, Gimhae, South Korea; Kosin University Gospel Hospital, Busan, South Korea; Inje University College of Medicine, Gimhae, South Korea; National University of Singapore, Singapore, Singapore; National University Health System, Singapore, Singapore; National University Hospital, Singapore, Singapore; Frieslandcampina, Amersfoort, Netherlands; Kk woman’s and Children’s Hospital, Singapore, Singapore; Samsung Medical Center, Seoul, South Korea; Samsung, Seoul, South Korea; Medical Research Institute of Pusan National University Hospital, Busan, South Korea; Konkuk University School of Medicine, Seoul, South Korea; Seoul Paik Hospital/Inje University College of Medicine, Seoul, South Korea; Department of Pediatrics, Hallym Sacred Heart Hospital, Hallym University College of Medicine, Chuncheon, South Korea; Samsung Medical Center, Seoul, South Korea; Samsung Seoul Hospital, Seoul, South Korea; Cheongdam Hana Dermatology Clinic, Seoul, South Korea; Institute for Environmental Research, College of Medicine, Yonsei University, Seoul, South Korea; National Institute of Horticultural & Herbal Science, Rural Development Administration, Wanju, South Korea; Catholic Kwandong University International St. Mary’s Hospital, College of Medicine, Incheon, South Korea; Bundang CHA Medical Center, CHA University School of Medicine, Seongnam, South Korea; Bundang Medical Center, CHA University, Seongnam, South Korea; Jeju National University School of Medicine, Jeju, South Korea; Jeju National University Hospital, Jeju, South Korea; Ambulanta Meznar, Celje, Slovenia; Soonchunhyang University Bucheon Hospital, Asan, South Korea; Soonchunhyang University, Asan, South Korea; Asan Institute for Life Sciences, Seoul, South Korea; Neopharm Co., Ltd., Seoul, South Korea; Asan Medical Center, Seoul, South Korea; Hallym University Sacred Heart Hospital, Anyang, South Korea; Prince Naif Center for Immunology Research and Asthma Research Chair, Department of Pediatrics, College of Medicine, King Saud University, Riyadh, Saudi Arabia; Prince Naif Center for Immunology Research, College of Medicine, King Saud University, Riyadh, Saudi Arabia; Pnhrc, King Saud University, Riyadh, Saudi Arabia; Department of Radiological Sciences, College of Applied Medical Sciences, King Saud University, Riyadh, Saudi Arabia; Kosin University Gospel Hospital, Busan, South Korea; Kosin University, Busan, South Korea; College of Medicine, Kosin University, Busan, South Korea; Department of Cell and Molecular Biology, University of Tehran, Tehran, Iran; Department of Immunology, Tehran University of Medical Sciences, Tehran, Iran; Molecular Immunology Research Center (MIRC), Tehran University of Medical Sciences, Tehran, Iran; Dankook University College of Medicine, Cheonan, South Korea; Department of Pediatrics, Childhood Asthma Atopy Center, Environmental Health Center, Asan Medical Center, University of Ulsan College of Medicine, Ulsan, South Korea; Department of Pediatrics, Korea Cancer Center Hospital, Seoul, South Korea; Chungbuk National University Hospital, Cheongju, South Korea; Seoul National University Medical Research Center, Seoul, South Korea; Institute of Allergy and Clinical Immunology, Seoul National University Medical Research Center, Seoul, South Korea; Ewha Institute of Convergence Medicine, Ewha Womans University Medical Center, Seoul, South Korea; Pogunhan Mom Hospital, Seoul, South Korea; Sung-Ae General Hospital, Seoul, South Korea; Seoul National University College of Medicine, Seoul, South Korea; Seoul National University Bundang Hospital, Seoul, South Korea; Seoul Medical Center, Seoul, South Korea; Chungbuk National University, Seoul, South Korea; Seoul National University Hospital, Seoul, South Korea; Sanggye Paik Hospital, Seoul, South Korea; Inje University Sanggye Paik Hospital, Seoul, South Korea; Faculty of Medicine, Universitas Padjadjaran, Bandung, Indonesia; Dr. Hasan Sadikin General Hospital, Jawa Barat, Indonesia; Soonchunhyang University, Asan, South Korea; Soonchunhyang University Bucheon Hospital, Asan, South Korea; Institute of Allergy Asthma and Clinical Immunology, Tsqaltubo, Georgia; Peopels Friendship University of Russia, Moscow, Russia; Yangsan Pusan National University Hospital, Busan, South Korea; Soonchunhyang University, Asan, South Korea; Soonchunhyang University Bucheon Hospital, Asan, South Korea; Korea National Institute of Health, Korea Center for Disease Control and Prevention, Cheongju, South Korea; Asan Institute for Life Sciences, Seoul, South Korea; Department of Pediatrics, Childhood Asthma Atopy Center, Environmental Health Center, Asan Medical Center, University of Ulsan College of Medicine, Ulsan, South Korea; Dankook University College of Medicine, Cheonan, South Korea; University of New South Wales, New South Wales, Australia; Children’s Hospital Westmead, Sydney, Australia; Asan Institute for Life Sciences, Seoul, South Korea; Neopharm Co., Ltd., Seoul, South Korea; Department of Pediatrics, Childhood Asthma Atopy Center, Environmental Health Center, Asan Medical Center, University of Ulsan College of Medicine, Ulsan, South Korea; Hallym University Sacred Heart Hospital, Anyang, South Korea; Bundang CHA Medical Center, CHA University School of Medicine, Seongnam, South Korea; Asan Institute for Life Science, Asan Medical Center, Seoul, South Korea; Department of Pediatrics, Hallym Sacred Heart Hospital, Hallym University College of Medicine, Chuncheon, South Korea; Asan Medical Center, Seoul, South Korea; Seoul National University Hospital, Seoul, South Korea; Seoul National University Bundang Hospital, Seoul, South Korea; Seoul National University Medical Research Center, Seoul, South Korea; Samsung Medical Center, Seoul, South Korea; Medical Research Institute of Pusan National University Hospital, Busan, South Korea; Sahmyook Medical Center, Seoul, South Korea; Sung-Ae General Hospital, Seoul, South Korea; Chonnam National University Medical School and Chonnam National University Hospital, Gwangju, South Korea; Chonnam National University Medical School, Gwangju, South Korea; Gwangju Veterans Hospital, Gwangju, South Korea; Ajou University School of Medicine, Suwon, South Korea; Maeil Dairies, Co. Ltd, Seoul, South Korea; Osaka University Graduate School of Medicine, Suita, Japan; Soonchunhyang University Bucheon Hospital, Asan, South Korea; Soonchunhyang University Hospital, Asan, South Korea; Chung Ang University, Seoul, South Korea; Chung-Ang University Hospital, Seoul, South Korea; Ewha Womans University, School of Medicine, Seoul, South Korea; Soonchunhyang University Bucheon Hospital, Asan, South Korea; Pusan National University Yangsan Hospital, Busan, South Korea; National Institute of Biomedical Innovation, Health and Nutrition, Osaka, Japan; Yokohama City University Graduate School of Medicine, Yokohama, Japan; WPI Immunology Frontier Research Center, Osaka University, Suita, Japan; Kangwon National University Hospital, Kangwon-do, South Korea; Dongtan Sacred Heart Hospital, Hallym University College of Medicine, Hwaseong, South Korea; Soonchunhyang University, Asan, South Korea; Soonchunhyang University Bucheon Hospital, Asan, South Korea; Soonchunhyang University Bucheon Hospital, Asan, South Korea; Asan Medical Center, University of Ulsan College of Medicine, Seoul, South Korea; Ajou University School of Medicine, Suwon, South Korea; Bundang Medical Center, CHA University, Seoul, South Korea; The First Affiliated Hospital of Guangxi Medical University Pediatrics, Nanning, China; Yonsei University College of Medicine, Seoul, South Korea; The First Affiliated Hospital of Guangzhou Medical University, Guangzhou, China; Catholic Kwandong University International St. Mary’s Hospital, College of Medicine, Incheon, South Korea; Cheongdam-Hana Dermatology, Seoul, South Korea; Centro Escolar University, Manila, Philippines; University of Santo Tomas, Manila, Philippines; Ajou University School of Medicine, Suwon, South Korea; Samsung Medical Center, Sungkyunkwan University, Seoul, South Korea; Yonsei University College of Medicine, Seoul, South Korea; Hallym University Sacred Heart Hospital, Anyang, South Korea; Ilsan Paik Hospital, Inje University College of Medicine, Goyang, South Korea; Dongtan Sacred Heart Hospital, College of Medicine, Hallym University, Hwaseong, South Korea; Soonchunhyang University College of Medicine, Asan, South Korea; Eulji University Hospital, Daejeon, South Korea; Seoul National University Bundang Hospital, Seoul, South Korea; Inha University Hospital, Incheon, South Korea; Seoul National University Hospital, Seoul, South Korea; Ajou University School of Medicine, Suwon, South Korea; Department of Pediatrics, Childhood Asthma Atopy Center, Environmental Health Center, Asan Medical Center, University of Ulsan College of Medicine, Ulsan, South Korea; Asan Institute for Life Science, University of Ulsan College of Medicine, Ulsan, South Korea; Asan Medical Center, Seoul, South Korea; Gangnam CHA Hospital, Seoul, South Korea; Samsung Medical Center, Seoul, South Korea; Department of Pediatrics, Yonsei University College of Medicine, Seoul, South Korea; University of Cincinnati College of Medicine, Cincinnati, USA; Department of Pediatrics, Hallym Sacred Heart Hospital, Hallym University College of Medicine, Chuncheon, South Korea; Cmid, G.Bosco Hospital, Turin, Italy; The First Affiliated Hospital of Guangxi Medical University, Nanning, China; Shengjing Hospital of China Medical University, Shenyang, China; Catholic Kwandong University International St. Mary’s Hospital, College of Medicine, Incheon, South Korea; Cheongdam-Hana Dermatology, Seoul, South Korea; University of Cincinnati College of Medicine, Cincinnati, USA; Seoul National University Bundang Hospital, Seoul, South Korea; Department of Pediatrics, Korea Cancer Center Hospital, Seoul, South Korea; Asan Medical Center, Seoul, South Korea; Department of Pediatrics, Hallym Sacred Heart Hospital, Hallym University College of Medicine, Gangwon-do, South Korea; Hallym University Sacred Heart Hospital, Anyang, South Korea; Bundang CHA Medical Center, CHA University School of Medicine, Seongnam, South Korea; Inje University Sanggye Paik Hospital, Seoul, South Korea; Department of Preventive Medicine, College of Medicine, Dankook University, Yongin, South Korea; Pusan National University Children’s Hospital, Busan, South Korea; Department of Pediatrics, the First Affiliated Hospital of Guangxi Medical University, Nanning, China; The First Affiliated Hospital of Guangxi Medical University, Nanning, China; The First Affiliated Hosital of Guangxi Medical University Pediatrics, Nanning, China; Regional Pharmacovigilance Center, Korea University Guro Hospital, Seoul, South Korea; Korea University College of Medicine, Seoul, South Korea; Ajou University School of Medicine, Suwon, South Korea; Red Maple Trials Inc., Ottawa, Canada; Red Maple Trials Inc., Ottawa, Canada; Pediatric Hospital, Buenos Aires, Argentina; Brazilian Sociaty, Uruguaina, Brazil; Hospital Santa Caterina, Girona, Spain; Clínica Regional Del Este, San Francisco, Argentina; Celal Bayar University School of Medicine, Manisa, Turkey; University of Zimbabwe, Harare, Zimbabwe; Asthma Allergy and Immune Dysfunction Clinic, Harare, Zimbabwe; Pop De Basesti Medical Centre, Bucharest, Romania; Medas Clinic, Bucharest, Romania; Celal Bayar University School of Medicine, Manisa, Turkey; Peoples’ Friendship University of Russia, Moscow, Russia; Graduate School of Medicine, Chiba University, Chiba, Japan; Chiba University, Chiba, Japan; Pantai Indah Kapuk Hospital, Jakarta, Indonesia; Messerli Research Institute of the University of Veterinary Medicine Vienna, Medical University Vienna and University Vienna, Wien, Austria; Institute of Pathophysiology and Allergy Research, Center of Pathophysiology, Infectiology and Immunology, Medical University of Vienna, Wien, Austria; Medical University of Vienna, Wien, Austria; University of Salzburg, Salzburg, Austria; Meiji Co Limited, Tokyo, Japan; Chiba University, Chiba, Japan; National Shimoshizu Hospital, Yotsukaido, Japan; Kazusa DNA Research Institute, Chiba, Japan; Masuda Maternity Clinic, Chiba, Japan; Seoul St. Mary’s Hospital, Seoul, South Korea; St. Mary’s Hospital, Seoul, South Korea; Pucheon St.Mary’s Hospital, Seoul, South Korea; Seoul National University Hospital, Seoul, South Korea; Seoul National University Medical Research Center, Seoul, South Korea; Duke University Medical Center, Durham, USA; Harapan Kita Women & Children Hospital, Jakarta, Indonesia; Faculty of Medicine University of Indonesia, Jakarta, Indonesia; Hallym University Sacred Heart Hospital, Anyang, South Korea; University of Cincinnati College of Medicine, Cincinnati, USA; Seoul National University Bundang Hospital, Seoul, South Korea; Inha University Hospital, Incheon, South Korea; Department of Pediatrics, Korea Cancer Center Hospital, Seoul, South Korea; Pusan National University Yangsan Hospital, Busan, South Korea; Dankook University College of Medicine, Cheonan, South Korea; Inje University Sanggye Paik Hospital, Seoul, South Korea; Department of Pediatrics, Childhood Asthma Atopy Center, Environmental Health Center, Asan Medical Center, University of Ulsan College of Medicine, Ulsan, South Korea; Bundang CHA Medical Center, CHA University School of Medicine, Seongnam, South Korea; Atopy (Allergy) Research Center, Tokyo, Japan; Juntendo University Faculty of Medicine, Tokyo, Japan; Juntendo University Shizuoka Hospital, Tokyo, Japan; Juntendo University, Tokyo, Japan; Consultant Inova, Nowshera, Pakistan; Cardiac Care Center, Karachi, Pakistan; George Washington Hospital, Washington, DC, USA; Al-Junaid Medical Center Kpk, Nowshera, Pakistan; Yong, Singapore, Singapore; Kk Women’s and Children’s Hospital, Singapore, Singapore; National University of Singapore, Singapore, Singapore; Department of Maternal Fetal Medicine, Kk Women’s and Children’s Hospital, Singapore, Singapore; Growth, Development and Metabolism Programme, Singapore Institute for Clinical Sciences (SICS), Agency for Science, Technology and Research (A*STAR), Singapore, Singapore; Nihr Southampton Biomedical Research Centre, University of Southampton and University Hospital Southampton NHS Foundation Trust, SO16 6YD, Southampton, UK; Department of Paediatrics, Yong Loo Lin School of Medicine, National University of Singapore, Singapore, Singapore; Department of Obstetrics & Gynaecology, Yong Loo Lin School of Medicine, National University of Singapore, Singapore, Singapore; National University Health System, Singapore, Singapore; Agency of Science, Technology and Research, Singapore, Singapore; G. Bosco Hospital, Turin, Italy; Semmelweis University, Faculty of Medicine, Budapest, Hungary; Charité-Universitätsmedizin Berlin, Berlin, Germany; Bluestone Technology Gmbh, Wörrstadt, Germany; Hamad Med Corp, Doha, Qatar; Istanbul University, Istanbul, Turkey; National Institute of Hygiene and Epidemiology, Hanoi, Vietnam; Hanoi Medical University, Hanoi, Vietnam; Vietnam National Hospital of Paediatrics, Hanoi, Vietnam; Sydney Medical School - Northern, University of Sydney, Sydney, Australia; Royal North Shore Hospital, Sydney, Australia; Bach Mai Hospital, Hanoi, Vietnam; “Sotiria” General Hospital, Athens, Greece; Araiteion Hospital Greece, Athens, Greece; Athens Naval Hospital, Athens, Greece; Hellenic Air Force, Athens, Greece; 251 General Air Force Hospital Greece, Athens, Greece; Visva Bharati, Santiniketan, Santiniketan, India; Ent Centre, New Delhi, India; Shanghai Tenth People’s Hospital, Tongji University, Shanghai, China; Nichinichi Pharmaceutical Co., Ltd., Tokyo, Japan; Jpn Health Promotion Supporting Network, Tokyo, Japan; Kanto Chemical Co., Inc., Tokyo, Japan; St.Johns National Academy of Health Sciences, Bengaluru, India; Ent Centre, Bengaluru, India; Ent Centre, Bengaluru, India; European Medical Center, Moscow, Russia; Consultant Inova, Nowshera, Pakistan; Cardiac Care Center, Karachi, Pakistan; George Washington Hospital, Washington, USA; Al-Junaid Medical Center Kpk, Nowshera, Pakistan; National Center for Child Health and Development, Tokyo, Japan; Harapan Kita Mother and Children Hospital, Jakarta, Indonesia; Hanyang University College of Medicine, Seoul, South Korea; Hanyang University, Seoul, South Korea; Children’s Hospital, Luang Prabang, Laos; Saiful Anwar Hospital, Malang, Indonesia; Hanyang University Kuri Hospital, Gyeonggi-do, South Korea; Korea Meteorological Agency, Seoul, South Korea; Korea Meteorological Administration, Seoul, South Korea; University of Western Ontario, London, Canada; Schulich School of Medicine & Dentistry, London, Canada; Allergy Center and Department of Clinical Research, Mie National Hospital, Tsu, Japan; Istituti Clinici Di Perfezionamento, Milan, Italy; Irccs Fondazione Ca’ Granda Ospedale Maggiore, Milan, Italy; King Khalid University Hospital, Riyadh, Saudi Arabia; King Faisal Specialist Hospital and Research Centre, Riyadh, Saudi Arabia; Nutricia Research, Utrecht, Netherlands; Utrecht University, Utrecht, Netherlands; Seoul National University Hospital, Seoul, South Korea; National Allergy Asthma Bronchitis Institute, Kolkata, India; Institute of Respiratory Medicine, Kuala Lumpur, Malaysia; Chang Gung Memorial Hospital, Taipei, Taiwan; Khon Kaen University, Nai-Muang, Thailand; Merck & Co., Inc., New Jersey, USA; Merck & Co., Inc. (retired), New Jersey, USA; Optum, Melbourne, Australia; National University of Singapore, Singapore, Singapore; Dami IM Clinic, Seoul, South Korea; Coco ENT Clinic, Seoul, South Korea; Jong-Woong Kim IM Clinic, Seoul, South Korea; Hamchun Medical Clinic, Seoul, South Korea; Myung ENT Clinic, Seoul, South Korea; Seoulbom IM Clinic, Seoul, South Korea; Konkuk University Medical Center, Seoul, South Korea; Yonsei University College of Medicine, Seoul, South Korea; Proteometech Inc., Seoul, South Korea; Division of Allergy and Immunology, Department of Internal Medicine, Yonsei University College of Medicine, Seoul, South Korea; Proteometech Inc., Seoul, South Korea; Proteometech Inc., Seoul, South Korea; Kyung Hee University, Seoul, South Korea; Hanyang University College of Medicine, Seoul, South Korea; Utrecht University, Utrecht, Netherlands; Utrecht University, Utrecht, Netherlands; Ambulanta Meznar, Gregorciceva 5, Celje, Slovenia; Yokohama City University Graduate School of Medicine, Yokohama, Japan; National Institute of Biomedical Innovation, Health and Nutrition, Osaka, Japan; WPI Immunology Frontier Research Center, Osaka University, Osaka, Japan; Kansas University Medical Center, Kansas, USA; National University Health System, Singapore, Singapore; National University of Singapore, Singapore, Singapore; Tan Tock Seng Hospital, Singapore, Singapore; Hamad Medical Corporation, Doha, Qatar; Weill-Cornell Medical College, Doha, Qatar; Section of Pediatric Allergy Immunology, Hamad Medical Corporation, Doha, Qatar; Section of Pediatric Endocrinology, Alexandria University, Alexandria, Egypt; Cedoc, NOVA Medical School / Faculdade De Ciências Médicas, Universidade Nova De Lisboa, Lisbon, Portugal; Serviço De Imunoalergologia, Hospital De Dona Estefânia, Centro Hospitalar De Lisboa Central, Epe, Portugal; Escola Superior De Tecnologia Da Saúde De Lisboa, Instituto Politécnico De Lisbo0a, (ESTeSL-IPL), Lisbon, Portugal; Serviço De Imuno, Porto, Portugal; Chulalongkorn University, Bangkok, Thailand; Chulalongkorn University Hospital, Bangkok, Thailand; Tehran University of Medical Sciences, Tehran, Iran; Islamic Azad University, North Tehran Branch, Tehran, Iran; Armand Trousseau Hospital, Paris, France; Kharazmi University, Tehran, Iran; Children Hospital, Immunology Research Center, Tabriz University of Medical Science, Tabriz, Iran; Erasmus MC, Rotterdam, Netherlands; The Second Affiliated Hospital, Guangzhou Medical University, Guangdong, China; Gyeongsang National University School of Medicine, Jinju, South Korea; Ajou University School of Medicine, Suwon-si, South Korea; Hanyang University College of Medicine, Seoul, South Korea; National University Health System, Singapore, Singapore; Hanyang University College of Medicine, Seoul, South Korea; Allergy and Immunology Division, Department of Child Health Faculty of Medicine Diponegoro University/dr. Kariadi Hospital, Semarang, Indonesia; Dr. Kariadi Hospital, Jawa Tengah, Indonesia; Immunology, Asthma and Allergy Research Institute, Children’s Medical Center, Tehran University of Medical Sciences, Tehran, Iran; Science and Research Branch, Islamic Azad University, Tehran, Iran; Islamic Azad University, North Tehran Branch, Tehran, Iran; Ajou University School of Medicine, Suwon, South Korea; Chungmu Hospital, Cheonan, South Korea; Division of Allergy and Immunology, Department of Internal Medicine, Yonsei University College of Medicine, Seoul, South Korea; Medical University of Lodz, Lodz, Poland; Hanyang University College of Medicine, Seoul, South Korea; Daejeon Sun Hospital, Daejeon, South Korea; Dr. Sami Ulus Obstetrics, Children’s Health and Diseases Training and Research Hospital, Ankara, Turkey; Yonsei University Wonju College of Medicine, Seoul, South Korea; Soonchunhyang University Gumi Hospital, Seoul, South Korea; Soonchunhyang University Bucheon Hospital, Asan, South Korea; Soonchunhyang University Hospital, Asan, South Korea; Hasan Sadikin General Hospital, Jawa Barat, Indonesia; Dr. M. Djamil Hospital, Padang, Indonesia; Faculty of Medicine Andalas University, Padang, Indonesia; Rsup Dr.M.Djamil/Faculty of Medicine Andalas University, Padang, Indonesia; Dr. Sami Ulus Obstetrics, Children’s Health and Diseases Training and Research Hospital, Ankara, Turkey; Dr Sami Ulus Children Hospital, Ankara, Turkey; Ataturk Hospital, Ankara, Turkey; Mimar Sinan University, Istanbul, Turkey; Dr. Sami Ulus Obstetrics, Children’s Health and Diseases Training and Research Hospital, Ankara, Turkey; Faculty of Dendistry, Ankara University, Ankara, Turkey; James Cook University, Townsville, Australia; Rutgers University, Springfield, USA; State Key Laboratory of Respiratory Disease, the First Affiliated Hospital, Guangzhou Medical University, Guangzhou, China; Guangzhou Medical University, Guangzhou, China

## Abstract

A1 Pirfenidone inhibits TGF-b1-induced extracellular matrix production in nasal polyp-derived fibroblasts

Jae-Min Shin, Heung-Man Lee, Il-Ho Park

A2 The efficacy of a 2-week course of oral steroid in the treatment of chronic spontaneous urticaria refractory to antihistamines

Hyun-Sun Yoon, Gyeong Yul Park

A3 The altered distribution of follicular t helper cells may predict a more pronounced clinical course of primary sjögren’s syndrome

Margit Zeher

A4 Betamethasone suppresses Th2 cell development induced by langerhans cell like dendritic cells

Katsuhiko Matsui, Saki Tamai, Reiko Ikeda

A5 An evaluation of variousallergens in cases of allergic bronchial asthma at lucknow and neighbouring districts by intradermal skintest

Drsushil Suri, Dranu Suri

A6 Evaluation ferqency of ADHD in childhood asthma

Marzieh Heidarzadeh Arani

A7 Steven johnson syndrome caused by typhoid fever in a child

Azwin Lubis, Anang Endaryanto

A8 Chronic Bronchitis with Radio Contrast Media Hypersensitivity: A Case with Hypothesized GINA Step 1 Asthma

Shinichiro Koga

A9 The association between asthma and depression in Korean adult : An analysis of the fifth korea national health and nutrition examination survey (2010-2012)

Lee Ju Suk

A10 Management of allergic disease exacerbations in pregnancy

Yasunobu Tsuzuki

A11 Subcutaneous immunotherapy mouse model for atopic dermatitis

Seo Hyeong Kim, Jung U Shin, Ji Yeon Noh, Shan Jin, Shan Jin, Hemin Lee, Jungsoo Lee, Chang Ook Park, Kwang Hoon Lee, Kwang Hoon Lee

A12 Atopic disease and/or atopy are risk factors for local anesthetic allergy in patients with history of hypersensitivity reactions to drugs?

Fatma Merve Tepetam

A13 Food hypersensitivity in patients with atopic dermatitis in Korea

Chun Wook Park, Jee Hee Son, Soo Ick Cho, Yong Se Cho, Yun Sun Byun, Yoon Seok Yang, Bo Young Chung, Hye One Kim, Hee Jin Cho

A14 Anaphylaxis caused by an ant (Brachyponera chinensis) in Japan

Yoshinori Katada, Toshio Tanaka, Akihiko Nakabayashi, Koji Nishida, Kenichi Aoyagi, Yuki Tsukamoto, Kazushi Konma, Motoo Matsuura, Jung-Won Park, Yoshinori Harada, Kyoung Yong Jeong, Akiko Yura, Maiko Yoshimura

A15 Anti-allergic effect of anti-IL-33 by suppression of immunoglobulin light chain and inducible nitric oxide synthase

Tae-Suk Kyung, Young Hyo Kim, Chang-Shin Park, Tae Young Jang, Min-Jeong Heo, Ah-Yeoun Jung, Seung-Chan Yang

A16 Food hypersensitivity in patients with chronic urticaria in Korea

Hye One Kim, Yong Se Cho, Yun Sun Byun, Yoon Seok Yang, Bo Young Chung, Jee Hee Son, Chun Wook Park, Hee Jin Cho

A17 Dose optimizing study of a depigmented polymerized allergen extract of phleum pollen by means of conjunctival provocation test (CPT)

Angelika Sager, Oliver Pfaar

A18 Correlation of cutaneous sensitivity and cytokine response in children with asthma

Amit Agarwal, Meenu Singh, Bishnupda Chatterjee, Anil Chauhan

A19 Colabomycin E, a Streptomycete-Derived Secondary Metabolite, Inhibits Proinflammatory Cytokines in Human Monocytes/Macrophages

Ilja Striz, Eva Cecrdlova, Katerina Petrickova, Libor Kolesar, Alena Sekerkova, Veronika Svachova, Miroslav Petricek

A20 Intravenous immunoglobluin treatment in a child with resistant atopic dermatitis: A brief review on this therapeutic regimen

Hyuck Hoon Kwon, Kyu Han Kim

A21 Wheat allergy is difficult to diagnose then other food allergens

Suman Kumar

A22 The effects of spirulina (Arthrospira platensis) dietary supplement as an adjunct therapy for children aged 7 to 14 years old with asthma: A randomized - double blind placebo controlled clinical trial

Lou Ver Leigh Arciaga Manzon, Pilar Agnes Gonzalez Andaya

A23 The study about cause and clinicopathological findings of injection induced dermatitis

Bark-Lynn Lew, Youngjun Oh, Dongwoo Suh, Woo-Young Sim

A24 IgE reactivity of recombinant allergen pac c 3 of the Asian needle ant pachycondyla chinensis

Kyoung Yong Jeong, Myung-Hee Yi, Mina Son, Dongpyo Lyu, Jae-Hyun Lee, Tai-Soon Yong, Chein-Soo Hong, Jung-Won Park

A25 Characterization of specific IgE antibody related to antigen 5 of echinococcus granulosus

Mohammadreza Siavashi

A26 Development of binary forecast model of asthma exacerbation: Asthma index

Hey Suk Yun, Ha-Na Kang, Jae-Won Oh, Young Jin Choi

A27 Different levels in rantes, IL-5 and TNF-á between the nasal polyps of adolescents with allergic, local allergic and non-allergic rhinitis

Ha-Na Kang, Jae-Won Oh, Young Jin Choi

A28 Tgfβ1 level is associated with VDR gene polymorphism in children with allergy diseases

Tatiana Sentsova, Ilya Vorozhko, Olga Chernyak, Vera Revyakina, Anna Timopheeva, Andrey Donnikov

A29 Dynamics of immunological biomarkers in children with food allergy fed goat milk formula

Tatiana Sentsova, Ilya Vorozhko, Olga Chernyak, Vera Revyakina, Anna Timopheeva

A30 Association between obesity, abdominal obesity and adiposity and the prevalence of atopic dermatitis in young Korean adults: The korea national health and nutrition examination survey, 2008–2010

Ji Hyun Lee, Young Min Park, Sang Soo Choi, Kyung Do Han, Han Mi Jung, Young Hoon Youn, Jun Young Lee, Yong Gyu Park, Seung-Hwan Lee

A31 Associations of natural history and environmental factors with asthma among children in rural and urban areas of guangdong, China

Zhaowei Yang, Jing Li, Mulin Feng, Marjut Roponen, Bianca Schaub, Gary WK Wong

A32 The effect of CO2-enriched atmospheres to producing of allergenic pollen by ragweed

Young Jin Choi, Ha-Na Kang, Jae-Won Oh

A33 Application evaluation of house dust mite and components specific-IgE and IgG4 in specific immunotherapy with allergic diseases

Baoqing Sun, Peiyan Zheng

A34 Effect of Asian dust events on asthma according to the socioeconomic status using claim data in KOREA

Yoon-Sung Park

A35 TSLP downregulates human â-defensin 2 through STAT3-dependent pathway in keratinocytes

Sang Wook Son

A36 Effects of anti-IgE on IL-4, IL-5, IL-17, and CD19,20,200 in a case of netherton syndrome (SPINK5 mutation)

Arzu Didem Yalcin, Sukran Kose, Kemal Kiraz

A37 Augmentation of arginase 1 expression exacerbates airway inflammation in murine asthma models

Jin-Young Lee, Sehyo Yune, Jae-Won Paeng, Mi-Jung Oh, Byung-Jae Lee, Dong-Chull Choi, Young Hee Lim, Kyoung Won Ha

A38 Caregivers of children with no food allergy – their experiences and perception of the condition

Kiwako Yamamoto-Hanada, Masami Narita, Masaki Futamura, Yukihiro Ohya

A39 Evaluation of Drug Provocation Tests in Korean Children: A Single Center Experience

Jihyun Kim, Jinwha Choi, Kwanghoon Kim, Jaehee Choi, Kangmo Ahn

A40 Danyoung classification 2015 update by digital HD endoscopic evaluation

SUN-HO/Brian Chang

A41 Effect on quality of life of the mixed house dust mite/weed pollen extract immunotherapy in polysensitized patients

Lisha Li

A42 Ambient desert dust and allergic symptoms: A time series analysis from a national birth cohort (JECS)

Kumiko Tsuji Kanatani, Yu-Ichi Adachi

A43 Individuals Allergic to Cow’s Milk Should be Vigilant When Consuming Beef Because It May be Injected Beef

Shigeyuki Narabayashi, Ikuo Okafuji, Yuya Tanaka, Satoru Tsuruta, Nobue Takamatsu

A44 Quality of life of chronic rhinosinusitis patients with or without nasal polyps in Korea

Soo Whan Kim, Do Hyun Kim

A45 House dust mite sensitization and exacerbation of asthma in the fall in children

Jong-Seo Yoon, Jin Tack Kim, Hwan Soo Kim, Yoon Hong Chun, Hyun Hee Kim, Sul Mui Won

A46 Evidence-based health advice for childhood eczema and household pets

Kam Lun E. Hon, Chung Mo Chow, Ting Fan Leung

A47 Relationship between allergic rhinitis and mental health in korea

Do Hyun Kim, Soo Whan Kim

A48 Oscillometric bronchodilator response in 3 to 5 years old healthy and asthmatic Filipino children

Gemmalyn Esguerra, Emily Resurreccion, Kristine Elisa Kionisala, Jenni Rose Dela Cruz

A49 The use of aeroallergen immunotherapy to treat eosinophilic esophagitis

Muhammad Imran

A50 A study of the eczema herpeticum in Korean

Yun Seon Choe, Kyu Han Kim, Mira Choi

A51 Specific sublingual immunotherapy in Korean patients with atopic dermatitis

Byung Soo Kim, Hyun-Joo Lee, Jeong-Min Kim, Jeong-Min Kim, Gun-Wook Kim, Je-Ho Mun, Je-Ho Mun, Hoon-Soo Kim, Margaret Song, Hyun-Chang Ko, Hyun-Chang Ko, Moon-Bum Kim

A52 Association between polymorphisms in bitter taste receptors genes and clinical features in Korean asthmatics

Sun-Young Yoon

A53 Effect of glycosides based standardized fenugreek seed extract in bleomycin-induced pulmonary fibrosis in rats

Amit Kandhare

A54 A kampo formula, ogi-kenchu-to, decreases side-effects of steroid ointment for infantile atopic dermatitis: Three cases report

Noriko Yahiro

A55 To test use of jet nebulizers NE-C802 as a drug delivery system in the children with asthma

Amit Agarwal, Meenu Singh, Jasleen Kaur, Ruby Pawankar, Pankaj Pant, Sukhmanjeet Singh

A56 Immunoglobulin e to allergen components of house dust mite in Korean children with allergic disease

Hwan Soo Kim, Jong-Seo Yoon, Sul Mui Won, Yoon Hong Chun, Jin Tack Kim, Hyun Hee Kim

A57 Effectiveness of premedication and rapid desensitization in hypersensitivity to l-asparaginase

Hwan Soo Kim, Sul Mui Won, Yoon Hong Chun, Jong-Seo Yoon, Hyun Hee Kim, Jin Tack Kim

A58 Angioedema with Eosinophilia: The First Report from Thailand

Thatchai Kampitak

A59 Evaluation of anti-pruritic and anti-inflammatory effects of Korean red ginseng extract on atopic dermatitis murine model

So Min Kim, Hyun Joo Lee, Hei Sung Kim, Jeong Deuk Lee, Sang Hyun Cho

A60 Subcutaneous autologous serum therapy in chronic urticaria

Kiran Godse

A61 Allergic bronchopulmonary aspergillosis in asthma and lung tuberculosis

Juwita Soekarno, Sarie Ratnasari, E. Alwi Datau, Eko Surachmanto, JC Matheos

A62 Infantile eczema is associated with campylobacter and roseburia subpopulations but not microbial diversity in stool samples of Chinese newborns

Ting Fan Leung, Jamie Sui-Lam Kwok, Christine Kit-Ching Tung, Man Fung Tang, Stephen Kwok-Wing Tsui, Gary WK Wong, Kam Lun Ellis Hon, Wing Hung Tam, Hing Yee Sy

A63 Association between serum chitinase level and toll-like receptor polymorphisms in bakery workers

Sohee Lee

A64 IFN-gamma contributes to nasal polypogenesis by inducing epithelial-to-mesenchymal transition via non-smad pathway

Hyun-Woo Shin, Mingyu Lee, Dae Woo Kim, Roza Khalmuratova

A65 Management and education status of anaphylaxis patients who visit our emergency room (ER)

Mi Yeoung Kim, Jaewon Jeong, Chansun Park

A66 Hypoallergen-Encoding DNA Plasmids As Immunoprophylactic Vaccines of Shrimp Tropomyosin Hypersensitivity

Christine Yee Yan Wai, Patrick S.C. Leung, Nicki Y.H. Leung, Ka Hou Chu

A67 The relationship between sputum pentraxin 3 levels and childhood asthma

Hee Seon Lee, Kyung Eun Lee, Jung Yeon Hong, Mi Na Kim, Min Jung Kim, Yoon Hee Kim, In Suk Sol, Seo Hee Yoon, Kyung Won Kim, Myung Hyun Sohn, Kyu-Earn Kim

A68 The role of local antibody responses in the nasal inflammation of allergic rhinitis (AR) patients

Ji Hye Kim, Hae-Sim Park, Yoo Seob Shin, Young Min Ye, Daehong Seo, Moon Gyeong Yoon, Young Mok Lee

A69 A case of ofloxacin-induced anaphylaxis by non-IgE, but specific IgG4-mediated responses

Daehong Seo, Ji Hye Kim, Young-Mok Lee, Young Min Ye, Hae-Sim Park

A70 Serum LTE4 metabolite as a biomarker for aspirin exacerbated respiratory disease

Ga Young Ban, Kumsun Cho, Seung-Hyun Kim, Yong Eun Kwon, Moon Gyeong Yoon, Ji Hye Kim, Yoo Seob Shin, Young Min Ye, Dong-Ho Nahm, Hae-Sim Park

A71 Local and systemic reactions of dust mite subcutaneous immunotherapy (SCIT) among children in a tertiary care hospital

Pilar Agnes Gonzalez Andaya

A72 Effects of carboxymethyl glucan (CM-glucan) in children with allergic rhinitis and asthma: A randomized controlled trial

Pilar Agnes Gonzalez Andaya

A73 Autophagy mechanisms in patients with severe asthma: A new therapeutic target

Ga Young Ban, Chang Gyu Jung, Seung-Ihm Lee, Duy Le Pham, Dong-Hyeon Suh, Eun-Mi Yang, Young Min Ye, Yoo Seob Shin, Hae-Sim Park

A74 Aggravation of airway inflammation and hypperresponsiveness following nasal challenge with dermatophagoides pteronyssinus in perennial allergic rhinitis patients without symptoms of asthma

Wan Jun Wang, MO Xian, Yan Qing Xie, Jing Ping Zheng, Jing Li

A75 Serum 25-hydroxyvitamin d in early childhood is non-linearly associated with allergy

Emma Merike Savilahti, Outi Mäkitie, Anna Kaarina Kukkonen, Sture Andersson, Heli Viljakainen, Erkki Savilahti, Mikael Kuitunen

A76 Fric test in dermographism

Kiran Godse

A77 Neutrophil autophagy and extracellular trap could contribute to asthma severity

Duy Le Pham, Ga Young Ban, Seung-Hyun Kim, Eun-Mi Yang, Hae-Sim Park, Ji-Ho Lee, Yong-Joon Chwae

A78 Redox Modulation for the Treatment of Toluene Diisocyanates-Induced Lung Inflammation

Li-Ming Chin, Chi-Chang Shieh

A79 A case of occupational asthma and rhinitis with anaphylaxis to Korean ginseng and sanyak

Ji Hye Kim, Hye-Soo Yoo, Moon Gyeong Yoon, Ga Young Ban, Ga Young Ban, Yoo Seob Shin, Young Min Ye, Hae-Sim Park

A80 Factors of influencing epidermal permeability barrier defects in atopic dermatitis children

Myong Soon Sung, Jin Uck Choi, Sung Won Kim, Yong Jin Hwang

A81 Innate type 2 response to aspergillusfumigatus in a murine model of atopic dermatitis-like skin inflammation

Arum Park, Eun Lee, Song-I Yang, Hyun-Ju Cho, Jinho Yu

A82 Activin a receptor 1C may implicate in the development of sensitive skin

Dong Hun Lee, Eun Ju Kim, Yeon Kyung Kim, Eun Jin Doh, Hee Chul Eun, Jin Ho Chung, Young Mee Lee, Seon Pil Jin

A83 Genetic association and eQTL analyses of genes associated with allergy in atopic/non-atopic asthma

Xingnan Li, Naftali Kaminski, Sally Wenzel, Eugene Bleecker, Deborah Meyers

A84 Gastroscope feature and clinical characteristics in 172 cases of children with henoch-schonlein purpura

Zeng Huasong

A85 The role of TRPV1 in CD4+ t cell mediated inflammatory response of allergic rhinitis

Ji-Hun Mo, Ramachandran Samivel, Eun-Hee Kim, Ji-Hye Kim, Jun-Sang Bae, Young-Jun Chung, Dae Woo Kim

A86 A Phenotype of Rhinitis from School Children Is Associated with the Development of Bronchial Hyperresponsiveness

Eun Lee, Si Hyeon Lee, Young-Ho Kim, Hyun-Ju Cho, Ho-Sung Yu, Mi-Jin Kang, Song-I Yang, Young-Ho Jung, Hyung Young Kim, Ju-Hee Seo, Byoung-Ju Kim, Hyo-Bin Kim, So-Yeon Lee, Ho-Jang Kwon, Soo-Jong Hong

A87 Increased basal activation status was noted in adult anaphylaxis patients

Sailesh Palikhe, Hae-Sim Park, Seung-Hyun Kim, Ji Hye Kim, Eun-Mi Yang

A88 Clinical values of interferon-gamma enzyme-linked immunospot assays for management of antibiotic hypersensitivity in hospitalized patients

Suda Sibunruang, Jettanong Klaewsongkram

A89 VDR gene polymorphism and 25-hydroxy vitamin d levels in children with food allergy

Tatiana Sentsova, Ilya Vorozhko, Anna Timopheeva, Olga Chernyak, Vera Revyakina, Andrey Sokolnikov

A90 An analysis of 145 oral almond challenge tests

Makoto Nisihino, Yu Okada, Noriyuki Yanagida, Motohiro Ebisawa, Sakura Sato, Kiyotake Ogura, Tomoyuki Asaumi, Kenichi Nagakura, Tetsuharu Manabe, Hirotoshi Unno

A91 Effect of creatine supplementation in fish allergenic potential; A proteomics study

Pedro M Rodrigues, Denise Schrama, Gadija Mohamed, Lizex Hüsselmann, Lizex Hüsselmann, Bongani Ndimba

A92 Flagellin modulates the function of invariant NKT cells via dendritic cells in asthma patients

Jae-Uoong Shim, Young Il Koh, Joon Haeng Rhee, Ji-Ung Jeong

A93 Clinical and subclinical manifestations of allopurinol – induced severe cutaneous adverse reactions in Vietnam

Dinh Van Nguyen, Hieu Chi Chu, Mui Thi Tran, Christopher Vidal, Suran Fernando, Sheryl Van Nunen, Sy Van Than

A94 Time course of serum inhibitory activity for facilitated allergen-IgE binding during house dust mite immunotherapy

Mulin Feng, Jing Li

A95 Periostin is a novel biomarker in eosinophilic nasal polyps of chronic rhinosinusitis

Dong-Kyu Kim, Seung-No Hong, Kyoung Mi Eun, Hong Ryul Jin, Dae Woo Kim

A96 Dominance of Th1-response in children with refractory mycoplasma pneumoniae pneumonia

Jun Bao, Yi-Xiao Bao

A97 Studies on the role of CD14 polymorphism among pollen and mold induced asthmatics of kolkata, India

Sanjoy Podder, Goutam Kumar, Shampa Dutta, Amlan Ghosh

A98 House dust mite allergy – Indian perspective

Goutam Kumar Saha, Sanjoy Podder, Salil Kumar Gupta

A99 Increased expression of purinergic (P2Y12) receptor and cysteinyl leukotriene receptors in the lung tissue of a mouse model of allergic asthma

Tu/Hoang Kim Trinh, Yoo Seob Shin, Hae-Sim Park, Jing-Nan Liu, Duy Le Pham

A100 Autologous serum skin test in chronic idiopathic urticaria - relationship with autoimmune markers and disease severity

Hyun-Chang Ko, Byung Soo Kim, Moon-Bum Kim

A101 Anxiety and depression levels in severe asthma patients treated with omalizumab

Ömer Özbudak, Fatih Üzer

A102 Economic burden of refractory chronic spontaneous urticaria on Kuwait health system

Mona Al-Ahmad, Maryam Alowayesh, Norman Carroll

A103 IgE-mediated maize allergy in India: A 28 kd protein responsible for food-induced allergic reaction

Anand Bahadur Singh

A104 Liposomal encapsulation of house dust mite allergens and dexamethasone modulates allergic response in a murine model of asthma

Yordanis Pérez-Llano, María Del Carmen Luzardo Lorenzo, Wendy Ramírez González, Carlos Calcines Cruz, Rady Laborde Quintana, Alain Morejón, Virgilio Bourg, Marilé Hechavarría Stoker

A105 Immune Suppressive Effects of Tonsil-Derived Mesenchymal Stem Cells for Eosinophilic Rhinosinusitis with Nasal Polyps in a Mouse Model

Jun-Sang Bae, Ramachandran Samivel, Eun-Hee Kim, Ji-Hye Kim, Ji-Hun Mo

A106 Second line treatments of dermographic urticaria refractory to antihistamines

Keiko Hanaoka, Michihiro Hide, Akio Tanaka, Makiko Hiragun, Mikio Kawai

A107 Diagnostic Value of Specific IgE to Peanut and Ara h 2 in Korean Children with Peanut Allergy

Kwanghoon Kim, Kwanghoon Kim, Hye-Young Kim, Jihyun Kim, Kangmo Ahn, Youngshin Han

A108 Inappropriate amounts of topical tacrolimus applied on Korean patients with eczema

Gun-Wook Kim, Hyun-Chang Ko, Byung Soo Kim, Moon-Bum Kim, Margaret Song

A109 Identification of an IgG1-mediated anaphylaxis marker and its application in evaluating the antigenicity of infant formulas

Takeshi Matsubara, Hiroshi Iwamoto, Yuki Nakazato, Kazuyoshi Namba, Yasuhiro Takeda

A110 Nitric oxide as a screening tool for evaluation of postoperative state of chronic rhinosinusitis

Jae Hoon Lee, Woo Yong Bae

A111 Comparison of different medical treatment options for crswnp: Doxycycline, methylprednisolone, mepolizumab, omalizumab

Els De Schryver, Lien Calus, Philippe Gevaert, Thibaut Van Zele, Claus Bachert

A112 Successful treatment of steroid resistant asthma model by blocking CD28 signal

Akio Mori, Satoshi Kouyama, Miyako Yamaguchi, Yo Iijima, Akemi Abe-Ohtomo, Hiroaki Hayashi, Kentaroh Watai, Chihiro Mitsui, Chiyako Oshikata, Kiyoshi Sekiya, Takahiro Tsuburai, Mamoru Ohtomo, Yuma Fukutomi, Masami Taniguchi

A113 Serum periostin levels was not associated with allergic rhinitis and allergic sensitization in Korean children

Ju Wan Kang, Jeong Hong Kim, Jeong Hong Kim, Keun-Hwa Lee, Hye-Sook Lee, Seong-Chul Hong, Jaechun Lee

A114 Roles of ADAM10 and ADAM17 in allergic rhinitis

Ji Won Seo, Jae Hoon Lee, Woo Yong Bae

A115 Mechanism of oral and topical polyprenol action in atopic dermatitis

Ivans Sergejs Kuznecovs, Galina Kuznecova

A116 Technical and clinical validation of a mobile chamber for allergen exposure tests

Karl-Christian Bergmann, Torsten Zuberbier, Joseph Salame, Torsten Sehlinger, Georg Bölke

A117 The association between serum lead level and total immunoglobulin e according to allergic sensitization

Yoo Suk Kim, Jung Hyun Chang, Jeong Hong Kim, Ju Wan Kang

A118 Clinical and laboratory characteristics of nasal obstruction dominant allergic sensitization

Seung-No Hong, Doo Hee Han, Chae-Seo Rhee

A119 Nasal provocation test is useful for the diagnoses of allergic, non- allergic, and local allergic rhinitis

Young-Joo Ko, Young Hyo Kim, Dae-Young Kim, Tae Young Jang

A120 Aspirin facilitates the intestinal absorption and oral sensitization of food allergens in rats

Tomoharu Yokooji, Taiki Hirano, Hiroaki Matsuo

A121 Gestational Secondhand Smoke Exposure Could Affect Maternal n-Glycosylation and Cause Filaggrin Loss in Children with Atopic Dermatitis

Galina Kuznecova, Ivans Sergejs Kuznecovs

A122 Allergen specific immunotherapy in the treatment of allergic rhinitis and asthma--a randomized prospective study from kashmir valley-north of India

Roohi Rasool Wani, Shafia Alam Syed, Ghulam Hassan, Ayaz Gul, Saniya Nissar, Zaffar Amin Shah

A123 Sleep disorders in latin-American children with asthma and/or allergic rhinitis and normal controls

Marilyn Urrutia Pereira, Carmen Fernandez, Dirceu Sole, Herberto Jose Chong Neto, Veronica Acosta, Alfonso Mario Cepeda, Mirta Alvarez Castello, Claudia Almendarez, Jose Santos Lozano Saenz, Juan C. Sisul, Nelson Rosario Filho, Antonio Castillo, Marylin Valentin Rostan, Jennifer Avila, Hector Badellino, Maria Carolina Manotas, Raúl Lázaro Castro Almarales, Mayda González León

A124 Association between respiratory symptoms and exhaled nitric oxide in Afghanistan

Woo Kyung Kim, Hae-Sun Yoon

A125 ATP, a danger signal, activates human eosinophils via P2 purinergic receptors

Takehito Kobayashi, Tooru Noguchi, Tomoyuki Soma, Kazuyuki Nakagome, Hidetomo Nakamoto, Hirohito Kita, Makoto Nagata

A126 Atopic dermatitis and sleep disorders in latin American children

Marilyn Urrutia Pereira, Dirceu Sole, Herberto Jose Chong Neto, Alfonso Mario Cepeda, Raúl Lázaro Castro Almarales, Juan C. Sisul, Marylin Valentin Rostan, Hector Badellino, Miguel Alejandro Medina Avalos, Antonio Castillo, Claudia Almendarez, Nelson Rosario Filho, Caridad Sanchez Silot, Jennifer Avila, Felicia Berroa Rodriguez, Jose Santos Lozano Saenz, Mirta Alvarez Castello, Carmen Fernandez

A127 Der p 23: A Major House Dust Mite Allergen in Spite of Limited Release from Fecal Pellets and Prominent Protease Sensitivity

Wai Tuck Soh, Alain Jacquet, Kiat Ruxrungtham, Emmanuel Nony, Maxime Le Mignon

A128 Anaphylactic Reaction After Inhalation of Budesonide

Mary Lee-Wong, Suzanne McClelland, Suzanne McClelland, Nanette B. Silverberg, Christian E. Song

A129 Lipidomic analysis of mattress dust from urban and rural schoolchildren in China

Zhaowei Yang, Jiukai Zhang, Wentao Zheng, Nanshan Zhong, Jing Li

A130 Improvements in quality of life in children with allergic rhinitis after adenotonsillectomy

Jung Ho Bae, Young Joo Cho, Joo Yeon Kim

A131 The seasonal variation of asthma exacerbations in patients allergic to pollens in Greece

Konstantinos Petalas, Dimitrios Vourdas, Christos Grigoreas

A132 Whole-genome sequencing study in allergic rhinitis nuclear families

Yuan Zhang

A133 Effect of the production of extracellular matrix from nasal fibroblasts by eosinophils activated with airborne fungi

Seung-Heon Shin, Mi-Kyung Ye, Jeong-Kyu Kim

A134 The study of clinical characteristics, lung function and bronchodilator responsiveness in infants with RSV bronchiolitis

Yong Feng, Yunxiao Shang

A135 GIS-based association between PM10 and allergic diseases in seoul: Implication for health and environmental policy

Sungchul Seo, Ji Tae Choung, Dohyeong Kim, Young Yoo, Hyunwook Lim

A136 The relationship between rhinovirus and recurrent wheezing

Wenjing Zhu, Chuanhe Liu, Li Sha, Li Chang, Min Zhao, Linqing Zhao, Yuan Qian, Yuzhi Chen

A137 Dominancy of Staphylcoccus Aureus in the Skin of Atopic Dermatitis Patients Compared to Healthy Subjects through Metagenomic Analysis

Min-Hye Kim, Young Joo Cho, Mina Rho, Jung-Won Kim, Yeon-Mi Kang, Kyung-Eun Yum, Hyeon-Il Choi, Jun-Pyo Choi, Han-Ki Park, Taek-Ki Min, Bok-Yang Pyun, Yoon-Keun Kim

A138 Micronized Cellulose Powder Reduces the Dose of Locally Applied Glucocorticoids in Patients with Allergic Rhinitis

Xueyan Wang

A139 New strategy for atopic dermatitis therapy with modulation of calcium ion channels

Woo Kyung Kim, Yu Ran Nam, Joo Hyun Nam

A140 Difference in the Systemic Bacterial Composition of Atopic Dermatitis Patients Compared to Healthy Subjects through Metagenomic Analysis of Urine

Jung-Won Kim, Min-Hye Kim, Mina Rho, Yeon-Mi Kang, Kyung-Eun Yum, Hyeon-Il Choi, Jun-Pyo Choi, Han-Ki Park, Taek-Ki Min, Young Joo Cho, Bok-Yang Pyun, Yoon-Keun Kim

A141 Occurrence and physiological function of immune complexes of food proteins and IgA in human saliva

Hiroshi Narita, Junko Hirose, Kumiko Kizu, Ayu Matsunaga

A142 Association between DNA hypomethylation at IL13 gene and allergic rhinitis in house dust mite-sensitized subjects

Jingyun Li, Yuan Zhang, Luo Zhang

A143 Effect of dietary methyl donors on asthma and atopy is modified by MTHFR polymorphism

Yean Jung Choi, Hye Lim Shin, Song-I Yang, So-Yeon Lee, Sung-Ok Kwon, Young-Ho Jung, Ji-Won Kwon, Hyung Young Kim, Ju-Hee Seo, Byoung-Ju Kim, Hyo-Bin Kim, Se-Young Oh, Ho-Jang Kwon, Eun Lee, Mi-Jin Kang, Soo-Jong Hong, Yun-Jeong Lee, Joonil Kim

A144 The effect of TSLP in a murine model of allergic asthma

Joon Young Choi, Ji Young Kang, Seok Chan Kim, Sei Won Kim, Seung Joon Kim, Young Kyoon Kim, Chin Kook Rhee, Hea Yon Lee, Hwa Young Lee, Sook Young Lee

A145 Evaluation of Aspirin Hypersensitivity in Chronic Rhinosinusitis Patients

Tae Kyung Koh, Sung Wan Kim, Kun Hee Lee, Chul Kwon, Joong-Saeng Jo, Sung-Hwa Dong, Young Seok Byun

A146 Chronic cough without wheezing in young children as a manifestation of chronic sinusitis

Charles Song

A147 Expression of muscarinic receptors and effect of tiotropium bromide on chronic asthma according to age in a murine model

Ji Young Kang, Hwa Young Lee, In Kyoung Kim, Sei Won Kim, Chin Kook Rhee, Seung Joon Kim, Seok Chan Kim, Sook Young Lee, Young Kyoon Kim, Soon Seog Kwon, Joon Young Choi

A148 Discrimination between non-eosinophilic and eosinophilic chronic rhinosinusitis with nasal polyps

Pona Park, Hong Ryul Jin, Dong-Kyu Kim, Dae Woo Kim

A149 Significant reduction in allergic features in the offspring of mice supplemented with specific non-digestible oligosaccharides during lactation

Astrid Hogenkamp

A150 Allergenicity assessment of hydrolysed infant formula; A multicenter comparison of a mouse model and a Guinea pig model for cow’s milk allergy

Leon Knippels, Betty C.a.m. Van Esch, Jolanda Van Bilsen, Prescilla V. Jeurink; Marjan Gros, Johan Garssen, Joost J Smit, Raymond H.H. Pieters

A151 Clinical significance between the allergic test and serum eosinophil cationic protein

Boo-Young Kim, Soo Whan Kim

A152 Hydroclorothiazide-induced acute non-cardiogenic pulmonary edema

Ramon Lleonart

A153 A Synbiotic Mixture of Scgos/Lcfos and *Bifidobacterium Breve* M-16V Is Able to Restore the Delayed Colonization of *Bifidobacterium* Observed in C-Section Delivered Infants

Christophe Lay, Kaouther Benamor, Chua Mei Chen, Jan Knol, Charmaine Chew, Voranush Chongsrisawat, Anne Goh, Wen Chin Chiang, Rajeshwar Rao, Surasith Chaithongwongwatthana, Nipon Khemapech

A154 Atopic characteristics of patients with asthma-COPD overlap syndrome

Ji Young Yhi, Sang-Heon Kim, Dong Won Park, Ji-Yong Moon, Tae Hyung Kim, Jang Won Sohn, Dong Ho Shin, Ho Joo Yoon, Seok Hyun Cho

A155 Perceptions and practices of severe asthma and asthma-COPD overlap syndrome among specialists: A questionnaire survey

Sang-Heon Kim, Ji-Yong Moon, Jae-Hyun Lee, Ga Young Ban, Sujeong Kim, Mi-Ae Kim, Joo-Hee Kim, Min-Hye Kim, Chan-Sun Park, Hyouk-Soo Kwon, Jae-Woo Kwon, Jae Woo Jung, Hye-Ryun Kang, Jong-Sook Park, Tae-Bum Kim, Heung Woo Park, You Sook Cho, Kwang-Ha Yoo, Yeon-Mok Oh

A156 A case of surgical diagnosed eosinophilic enteritis with intussusception in adult patient

Sang-Rok Lee

A157 Reference values of total IgE in estonian children

Kaja Julge, Maire Vasar, Tiia Voor, Tiina Rebane

A158 A case of eosinophilic granulomatosis with polyangiitis accompanied by rapidly progressive glomerulonephritis

Yu Jin Kim, Sang Min Lee, Shin Myung Kang, Sojeong Kim, Sun Young Kyung, Sung Hwan Jeong, Jeong-Woong Park, Hyunjung Hwang, Yong Han Seon, Sanghui Park, Sang Pyo Lee

A159 Associations Between Infectious Diseases and Urticaria

Marius Iordache

A160 Sleep in infants in korea – finding of bisq survey

Yeongsang Jeong, Sohee Eun, Byung Min Choi, Ji Tae Choung, Wonhee Seo

A161 Increased Expression of Filaggrin, TSLP, Periostin, IL13 and IL-33 in Nasal Polyps

Liang Zhang, Ruby Pawankar, Manabu Nonaka, Miyuki Hayashi, Shingo Yamanishi, Harumi Suzaki, Yasuhiko Itoh, So Watanabe, Hitome Kobayashi

A162 Asymptomatic bacteruria increases the risk of edematous attacks in patients with hereditary angioedema due to C1 inhibitor deficency (C1-INH-HAE)

Zsuzsanna Zotter, Henriette Farkas, Lilian Varga, Nora Veszeli, Eva Imreh, Gabor Kovacs, Marsel Nallbani

A163 Gastric Erosions Cause Spontaneous Urticaria Independent of Helicobacter Pylori

Semen Zheleznov, Galina Urzhumtseva, Natalia Petrova, Zhanna Sarsaniia, Nikolai Didkovskii, Torsten Zuberbier

A164 The Effect of G2 Vaccine on the Gene Expression NKG2D and Receptor Presenting on the Surface of NK Cells in Peripheral Blood

Nader Dashti Gerdabi, Ali Khodadadi, Zahra Abdoli, Mehri Ghafourian, Mohammad Ali Assarehzadegan, Khodayar Ghorban

A165 Ethnic differences in lifetime prevalence and indoor environmental factors for childhood eczema

Hyo-Bin Kim, Hui Zhou, Jeong Hee Kim, Rima Habre, Theresa Bastain, Frank Gilliland

A166 A case of methazolamide-induced toxic epidermal necrolysis

Jong-Wook Bae, Kyu-Hyung Han, Young-Koo Jee, Misoo Choi, Seung-Phil Hong, Seung-Hyun Kim

A167 Inflammatory responses of human adipose-tissue derived stem cells to LPS and nanoparticles

Hee-Kyoo Kim, Gil-Soon Choi, Jeonghoon Heo, Young-Ho Kim, Eun-Kee Park

A168 Analysis of 71 Cashew Nut Oral Challenge Tests

Takashi Inoue, Kiyotake Ogura, Noriyuki Yanagida, Hirotoshi Unno, Kenichi Nagakura, Tetsuharu Manabe, Tomoyuki Asaumi, Sakura Sato, Yu Okada, Motohiro Ebisawa

A169 Fungal sensitization is associated with asthma exacerbation

Min-Gu Kim, You Sook Cho, Tae-Bum Kim, Hee-Bom Moon, Jung-Hyun Kim, Hyo-Jung Kim, So-Young Park, Bomi Seo, Hyouk-Soo Kwon, Jaemoon Lee, Taehoon Lee

A170 Individual therapeutic patient education and consultation in children with atopic dermatitis

Hye-Soo Yoo, Jieun Kim, Inok Kim, Haejin Kim, Younhee Chang, Hae-Sim Park, Sooyoung Lee

A171 Utility of Alpha-Lactalbumin Specific IgE Levels Using Immulite 2000 3gAllergy in Predicting Clinical Severity of Milk Allergy

Kazuyo Kuzume, Munemitsu Koizumi, Koji Nishimura, Michiko Okamoto

A172 Isoniazid/rifampicin-specific t-cell responses in patients with anti-tuberculosis –induced dress syndrome

Seung-Hyun Kim, Young Min Ye, Gyu Young Hur, Hae-Sim Park, Sang-Heon Kim, Young-Koo Jee

A173 Genetic biomarkers associated with aspirin-exacerbated respiratory disease (AERD) phenotype based on genome-wide association study

Seung-Hyun Kim, Hyunna Choi, Young Min Ye, Hae-Sim Park

A174 Assessment of ORAL drug provocation test in the diagnosis of NON-steroidal ANTI-inflammatory drugs hypersensitivity

Bui VAN Khanh, Hieu Chi Chu, Nguyen Nhu Nguyet, Nguyen Hoang Phuong

A175 Korean treatment guideline of atopic dermatitis

Joo Young Roh, Hyun Jeong Kim, Jung Eun Kim, Bark-Lynn Lew, Kyung Ho Lee, Seung-Phil Hong, Yong Hyun Jang, Kui Young Park, Seong Jun Seo, Jung Min Bae, Eung Ho Choi, Ki Beom Suhr, Seung Chul Lee, Hyun-Chang Ko, Young Lip Park, Sang Wook Son, Young Jun Seo, Yang Won Lee, Sang Hyun Cho, Chun Wook Park

A176 Systemic side reaction of subcutaneous immunotherapy(SCIT) for perennial allergic rhinitis

Kun Hee Lee, Sung Wan Kim

A177 Clinical baseline characteristics of Asian patients suffering from refractory chronic spontaneous urticaria (CSU) in three phase 3 omalizumab clinical trials

Chia-Yu CHU, Derrick Aw, Young-Min Ye, Giovanni Bader, Fabrizio Dolfi, Nathalie Oliveira

A178 A metagenomic approach through t-RFLP to the microbiome of asthma

Jae Chol Choi, Jae Woo Jung, Hye-Ryun Kang, Kijeong Kim, Byoung Whui Choi

A179 Clinical characteristics and ten-year trend of peripheral blood eosinophilia among health screening program recipients at a tertiary hospital of South Korea

Jong Wook Shin, Jae Woo Jung, Jae Chol Choi, In Won Park, Byoung Whui Choi, Jae Yeol Kim

A180 The prevalence of toxocariasis and diagnostic value of serologic tests in asymptomatic Korean adults

Jin-Young Lee, Kyoung Won Ha, Yun-Jin Jeung, Sehyo Yune, Byung-Jae Lee, Dong-Chull Choi, Mi-Jung Oh, Young Hee Lim

A181 Cutaneous Drug Hypersensitivity Reaction in Korean Children: An Analysis of KAERS Database on 2012-2013

Eui Jun Lee, Dongin Suh, Sung-Il Woo, Hwa Jin Cho, Eun Hee Chung, Soo Youn Chung

A182 Comparison of clinical characteristics, quality of life and sleep in patients with allergic rhinitis when categorised as “sneezers and runners” and “blockers”

Kamal Gera, Ashok Shah

A183 Role of s-nitrosoglutathione reductase (GSNOR) in the murine strain differences of airway hyperresponsiveness

Jin-Young Lee, Kyoung Won Ha, Mi-Jung Oh, Young Hee Lim, Sehyo Yune, Jae-Won Paeng, Mi-Jin Jang, Byung-Jae Lee, Dong-Chull Choi

A184 Protection from airway bronchoconstriction by gsno

Jin-Young Lee, Mi-Jin Jang, Jae-Won Paeng, Yun-Jin Jeung, Young Hee Lim, Mi-Jung Oh, Kyoung Won Ha, Byung-Jae Lee, Dong-Chull Choi, Sehyo Yune

A185 Does EIA-targeted asthma treatment improve daily physical activity of children?

Takahiro Ito

A186 Wheezing as a clue to the diagnosis of cough variant asthma and nonasthmatic eosinophilic bronchitis

Jihye Kim, Jin-Young Lee, Sehyo Yune, Byung-Jae Lee, Dong-Chull Choi, Mi-Jin Jang, Jae-Won Paeng, Young Eun Kim, Young Nam Kim, Yongseok Lee

A187 Antagonism of microRNA-21 suppressed the airway inflammation in a mouse model of bronchial asthma

Hwa Young Lee, Sook Young Lee, Soon Seog Kwon, Young Kyoon Kim, Chin Kook Rhee, Sei Won Kim, Hea Yon Lee, Joon Young Choi, In Kyoung Kim

A188 Chlorhexidine anaphylaxis: A report of two cases

Jose Antonio Navarro, Maria Ascension Aranzabal, Alejandro Joral, Susana Lizarza, Miguel Echenagusia, EVA Maria Lasa

A189 Effects of Particulate Matter on Respiratory Allergic Diseases Considering Meteorological Factors in Busan, Korea

Eun-Jung Jo, Sun-Mi Jang, Seung-Eon Song, Hae-Jung Na, Chang-Hoon Kim, Woo-Seop Lee, Hye-Kyung Park

A190 Clinical characteristics of neutrophilic asthma

Sachiko Miyauchi, Yoshitaka Uchida, Tomoyuki Soma, Susumu Yamazaki, Toru Noguchi, Takehito Kobayashi, Kazuyuki Nakagome, Makoto Nagata

A191 Current Practice of Infants and Children with Acute Urticaria at a Single Wide Regional Emergency Medical Center

Hea Lin Oh, Do Kyun Kim, Dongin Suh, Young Yull Koh

A192 Discordance between sputum eosinophilia and exhaled nitric oxide

Sehyo Yune, Jin-Young Lee, Byung-Jae Lee, Dong-Chull Choi, Jae-Won Paeng, Mi-Jin Jang, Jihye Kim, Young Nam Kim

A193 Association between genetic polymorphisms of costimulatory molecules and antituberculosis drugs induced hepatitis

Sang-Heon Kim, Sang-Hoon Kim, Jang Won Sohn, Ho Joo Yoon, Dong Ho Shin, Jae Hyung Lee, Byoung Hoon Lee, Youn-Seup Kim, Jae-Seuk Park, Young-Koo Jee

A194 The prevalence of gastroesophageal reflux disease in chronic unexplained cough

Sehyo Yune, Jin-Young Lee, Jae-Won Paeng, Mi-Jin Jang, Dong-Chull Choi, Byung-Jae Lee, Yongseok Lee, Young Eun Kim

A195 Risk Factors of Allergic Rhinitis in Preschool Children and Clinical Utility of Feno

Jisun Yoon

A196 Relationship between serum 25-hydroxyvitamin d and asthma exacerbation severity in children

Yong Feng, Li Zhang, Xuxu Cai

A197 Usefulness of Specific IgE Antibody Levels to Wheat, Gluten and Ï-5 Gliadin for Wheat Allergy in Korean Children

Jong-Seo Yoon, Kyunguk Jeong, Hye-Soo Yoo, Sooyoung Lee, Sooyoung Lee

A198 Neutralization of stratum corneum accelerates the progress from atopic dermatitis to asthma-like lesion in flaky tail mice treated by house dust mite allergen

Hae-Jin Lee, Noo Ri Lee, Bo-Kyung Kim, Minyoung Jung, Dong Hye Kim, Catharina S. Moniaga, Kenji Kabashima, Eung Ho Choi

A199 Trends in oral food challenges in Japan: A six-year prospective study

Noriyuki Yanagida, Sakura Sato, Chizuko Sugizaki, Motohiro Ebisawa

A200 The Gut Microbiome in the Food Allergic Host

Jamie Kiehm, Punita Ponda, Sherry Farzan, Jared Weiss, Claudia Elera, Catherine Destio, Cristina Sison, Annette Lee

A201 Cord blood cytokines and maternal environmental exposure during pregnancy

Soo Hyun Ri, Chang Hoon Lim

A202 Rupatadine pharmacokinetics in Japanese healthy volunteers after single and repeated oral doses of 10, 20 and 40 mg

Iñaki Izquierdo Pulido, Jorg Taubel, Georg Ferber, Eva Santamaria Masdeu

A203 A safe and effective method to desensitize patients with wheat allergy

Alireza Khayatzadeh, Masoud Movahedi, Motohiro Ebisawa, Mohammad Gharagozlou

A204 RNA Binding Protein Hur Regulates CD4+ T Cell Differentiation and Is Required for Allergic Airway Inflammation and Normal IL-2 Homeostasis

Ulus Atasoy, Patsharaporn Techasintana, Matt Gubin, Jacqueline Glascock, Suzanne Ridenhour, Joseph Magee

A205 Time Trends in the Epidemiology of Recurrent Wheezing in Infants from South America

Nelson Rosario Filho; Herberto Jose Chong Neto, Gustavo Falbo Wandalsen, Ana Caroline Dela Bianca, Carolina Aranda, Dirceu Sole, Javier Mallol, Luis Garcia-Marcos, Luis Garcia-Marcos

A206 Successful Cyclophosphamide Desensitization in a Pediatric Patient with Systemic Lupus Erythematosus

Jennifer Toh, Yoomie Lee, Joyce Huang, Elina Jerschow, Jenny Shliozberg

A207 The Fatty Acid Binding Protein Der p 13 Is a Minor House Dust Mite Allergen Able to Activate Innate Immunity

Pattraporn Satitsuksanoa, Narissara Suratannon, Jongkonnee Wongpiyabovorn, Pantipa Chatchatee, Kiat Ruxrungtham, Alain Jacquet

A208 Epidemiology of Stevens-Johnson Syndrome and Toxic Epidemal Necrolysis: An Administrative Database Study

Min Suk Yang; Jin Yong Lee, Ja Yeun Kim, Han-Ki Park, Ju-Young Kim, Woo-Jung Song, Hye-Ryun Kang, Heung Woo Park, Yoon-Seok Chang, Sang-Heon Cho, Kyung-up Min, Chang-Han Park, Suk-Il Chang, Sook-Hee Song

A209 Regional Differences of Vitamin D and Food-Induced Anaphylaxis in Korea

Si-Heon Kim, Gil-Soon Choi, Su-Chin Kim, Ji Hye Kim, Ga Young Ban, Yoo Seob Shin, Hae-Sim Park, Young Min Ye

A210 Triggering Factors of Atopic Dermatitis By Severity

Yoon Ha Hwang

A211 Clinical Features of Adverse Drug Reactions of Monoclonal Antibodies in Korea

Da Woon Sim, Kyung Hee Park, Kyung Hee Park, Hye Jung Park, Hye Jung Park, Jung-Won Park, Jung-Won Park, Jae-Hyun Lee, Jae-Hyun Lee

A212 Food Allergy with Eczema Is Associated with Reduced Growth in the First Four Years of Life

Katrina Allen, Cara Beck, Jennifer Koplin, Melanie Matheson, Mimi Tang, Anne-Louise Ponsonby, Lyle Gurrin, Shyamali Dharmage, Melissa Wake, Vicki Mcwilliam

A213 The Preliminary Study on Clinical Efficacy and Impact Factors of One Year’s Dust Mite Specific Immunotherapy in Allergic Asthma and Rhinitis Children Sensitized to Dust Mite

Xiaoying Liu, Jing Wang, Li Xiang, Qun Wang

A214 Lipopolysaccharide Signaling through Toll- like Receptor 4 Could be Augmented By Dermatophagoides Farinae in the Human Middle Ear Epithelial Cell

Ji-Eun Lee, Dong-Young Kim, Chae-Seo Rhee, Chae-Seo Rhee

A215 Drug Allergy in Pregnant Adolescents: Relation with Familial and Personal Atopy, and Substances Use

Francisco Vazquez-Nava

A216 Patients and Physicians Concept of Well-Controlled Asthma: Findings from Realise Asia

Sang-Heon Cho, Jaewon Jeong, Diahn-Warng Perng, David Price, Glenn Neira, Jiangtao Lin

A217 The Role of Vasoactive Intestinal Peptide in the Pathophysiology of Acute Asthma

Olga Semernik

A218 Comparison of Serum Cytokine Levels According to the Severity in Atopic Dermatitis

Ha-Su Kim, Jin-a Jung, Ji-in Jung

A219 The Different Influence on the Regulatory T Cell Response Between Subcutanous Immnuotherapy(SCIT) and Sublingual Immunotherapy(SLIT) in Children with Asthma

Qing Miao, Li Xiang

A220 Asthma State of Affairs in Asia: Seeing through Physicians’ and Patients’ Lenses

Sang-Heon Cho, Jaewon Jeong, Diahn-Warng Perng, Jiangtao Lin, David Price

A221 Identification of Aspirin Exacerbated Respiratory Disease (AERD) Phenotypes Using Two Step Cluster Analysis

Hyun Young Lee, Hae-Sim Park, Young Min Ye, Su Chin Kim

A222 Dusty Air Pollution Are Associated with an Increase Risk of Allergic Diseases in General Population

Shokrollah Farrokhi, Mohammadkazem Gheiby

A223 A Genome-Wide Association Study of Antituberculosis Drugs-Induced Hepatitis

Sang-Heon Kim; Heung Woo Park, Sang-Hoon Kim, Young-Koo Jee

A224 The Role of peroxiredoxin6 of Bronchial Epithelial Cells in Regulating Mitochondrial Function Under Oxidative Stress By Translocation to Outside Mitochondrial Membrane

Sunjoo Park, Keun Ai Moon, Hyouk-Soo Kwon, Tae-Bum Kim, You Sook Cho, Hee-Bom Moon, Kyoung Young Lee, Gyong Hwa Hong, Eun Hee Ha

A225 Toxic and Adjuvant Effects of 3 Types of Silica Nanoparticles on Airway System

Heejae Han, Hye Jung Park, Yoon Hee Park, Yoon-Jo Kim, Kangtaek Lee, Jung-Won Park, Jae-Hyun Lee

A226 Procedure for Diagnostic and Selection of Immunotherapy Method for Children with Different Immunopathogenetic Phenotypes of Atopic Dermatitis

Tatiana Slavyanskaya, Vladislava Derkach

A227 Prediction of the Success of Our Desensitization Protocol with Symptoms and Results of a Skin Prick Test in Patients with Hypersensitivity to Platinum-Based Chemotherapy

Hye Jung Park, Chein-Soo Hong, Jae-Hyun Lee, Jae-Hyun Lee, Sungryeol KIM, Sungryeol KIM, Kyung Hee Park, Kyung Hee Park, Choong-Kun Lee, Beodeul Kang, Seung-Hoon Beom, Sang Joon Shin, Minku Jung, Jung-Won Park, Jung-Won Park

A228 Anti-Allergic Effect of Intralymphatic Injection of OVA-Flagelin Mixture in Mouse Model of Allergic Rhinitis

Eun-Hee Kim, Ji-Hye Kim, Ji-Hun Mo, Young-Jun Chung

A229 Serum Periostin Level Is Higher in Respiratory Type of NSAID Hypersensitivity Than Cutaneous Type

Mi-Ae Kim, Hae-Sim Park, Moon Gyeong Yoon, Young-Soo Lee, Ji Hye Kim, Ga Young Ban, Hye-Soo Yoo, Yoo Seob Shin, Young Min Ye, Dong-Ho Nahm

A230 A Retrospective Analysis of Allergy Blood Testing in Beijing Children’s Hospital in the Year of 2013: A Single-Center Research

Qing Miao, Li Xiang

A231 Role of Nrf2 in the Allergic Airway Inflammation Differ Between BALB/c and C57BL/6 Mice

Ying-Ji Li, Takako Shimizu, Hirofumi Inagaki, Yukiyo Hirata, Hajime Takizawa, Arata Azuma, Masayuki Yamamoto, Tomoyuki Kawada

A232 Effect of Human Mesenchymal Stem Cell on Neutrophilic Asthma Model

Min-Gu Kim, Gyong Hwa Hong, Kyoung Young Lee, Eun Hee Ha, Keun Ai Moon, Sunjoo Park, Hyouk-Soo Kwon, Tae-Bum Kim, Hee-Bom Moon, You Sook Cho, Jung-Hyun Kim, Hyo-Jung Kim, So-Young Park, Bomi Seo

A233 Immunomodulatory Effect of Tonsil Derived Mesenchymal Stem Cells in a Mouse Model of Allergic Rhinitis

Ji-Hye Kim, Ramachandran Samivel, Eun-Hee Kim, Young-Jun Chung, Ji-Hun Mo

A234 Alternative Therapy Such As Yoga May be a Low Cost Tool for Improving the Quality of Life of Patient’s with Allergic Rhinitis and Asthma

Soumya M. S., G. Inbaraj, R. Chellaa, Ruby Pawankar

A235 Substantial Impairment of the Quality of Life in Adult Patients with Chronic Urticaria

Wonsun Choi, Hae-Sim Park, Young Min Ye, Ji Hye Kim, Ga Young Ban, Yoo-Seob Shin

A236 Dietary Galacto-Oligosaccharides Reduce Airway Eosinophilia and Enhance the Th2 Suppressive Effect of Budesonide in House Dust Mite-Induced Asthma in Mice

Saskia Braber, Kim Verheijden, Aletta Kraneveld, Johan Garssen, Linette Willemsen, Gert Folkerts

A237 Production and Characterization of Recombinant Periplaneta americana Allergens for Component Resolved Diagnosis

Stephanie Eichhorn, Fatima Ferreira, Isabel Pablos, Bianca Kastner, Bettina Schweidler, Sabrina Wildner, Peter Briza, Jung-Won Park, Naveen Arora, Stefan Vieths, Gabriele Gadermaier

A238 Assessment of Characteristics of Itch in Patients with Hand Eczema

Sung-Min Park, Won-Ku Lee, Jeong-Min Kim, Gun-Wook Kim, Je-Ho Mun, Hoon-Soo Kim, Margaret Song, Hyun-Chang Ko, Moon-Bum Kim, Byung Soo Kim

A239 The Hidden Culprit: A Case of Repeated Anaphylaxis from Cremophor Hypersensitivity.

Young Nam Kim, Sehyo Yune, Jin-Young Lee, Jihye Kim, Young Eun Kim, Jae-Won Paeng, Mi-Jin Jang, Dong-Chull Choi, Byung-Jae Lee, Yongseok Lee

A240 Spectrum of Anaphylaxis in Children and Adults at Emergency Departments in Singapore

Si Hui Goh, Bee Wah Lee, Jian Yi Soh

A241 Improved Quality of Life through an Integrated Health Care Service for Children with Atopic Dermatitis

Hyungoo Kang; Hyunhee Kim; Hye-Yung Yum

A242 Criteria Combining Autologous Serum Skin Test and Clusterin for Predicting Antihistamine-Refractoriness in Patients with Chronic Spontaneous Urticaria

Young Min Ye; Hae-Sim Park; Ga-Young Ban; Ji Hye Kim; Yoo Seob Shin

A243 Urinary Leukotriene E4 Levels in Wheezing Infants

Takumi Takizawa, Masahiko Tabata, Akira Aizawa, Hisako Yagi, Yutaka Nishida, Hirokazu Arakawa, Akihiro Morikawa, Solongo Orosoo

A244 Allergic Sensitization to Whey in Mice Is Facilitated By the Mycotoxin Deoxynivalenol (DON)

Saskia Braber, Marianne Bol-Schoenmakers, Peyman Akbari, Prescilla V. Jeurink, Prescilla V. Jeurink, Priscilla De Graaff, Joost J. Smit, Betty C. A. M. Van Esch, Johan Garssen, Johan Garssen, Johanna Fink-Gremmels, Raymond H. H. Pieters

A245 How to Define Chronic Cough: Based on a Systematic Review of the Epidemiological Literature

Gun-Woo Kim, Eun-Jung Jo, Sujeong Kim, Woo-Jung Song, Yoon-Seok Chang, Shoaib Faruqi, Ju-Young Kim, Mingyu Kang, Min-Hye Kim, Jana Plevkova, Heung Woo Park, Sang-Heon Cho, Alyn Morice, So-Hee Lee, Sun-Sin Kim, Seoung-Eun Lee

A246 Asko Study: Comparison of Behavior and Habits in Diagnosis and Treatment of Adult Asthma and COPD Patients

Bilun Gemicioglu, Zeynep Misirligil, Arif Hikmet Cimrin, Hakan Gunen, Tevfik Ozlu, Aykut Cilli, Levent Akyildiz, Hasan Bayram, Esra Uzaslan, Oznur Abadoglu, Mecit Suerdem

A247 Changes in Pulmonary Function in the Treatment of Obesity in Children

Keigo Kainuma

A248 Changes of Feno and Nasal Feno Levels after Treatment in Pediatric Allergic Rhinitis

Hyun-a Kim, Ha-Su Kim, Woo Yong Bae, Jin-a Jung

A249 Prevalence of Vitamin D Deficiency in Exclusively Breastfed Infants in Kenya

Rose Kamenwa, William Macharia, Nusrat Said

A250 In-Vitro Screening of Atopy in the Indian Population: Are Current Methods Adequate, Keeping Local IgE Seroprevalence for Common Food & Inhalant Allergens in Mind?

Vidya Nerurkar, Meenal Patel, Simi Bhatia

A251 Usefulness of House Dust Mites Nasal Provocation Test in Asthma

Inseon S Choi, Soo-Jeong Kim, Joo-Min Won, Myeong-Soo Park

A252 Biomarker-Based Treatment Option for Preschool Children with Recurrent Wheeze

Mizuho Nagao

A253 Anti-Tuberculosis Drugs-Induced Liver Injury in Patients with Connective Tissue Diseases

Dong Won Park, Jang Won Sohn, Ji Young Yhi, Ji-Yong Moon, Sang-Heon Kim, Tae Hyung Kim, Dong Ho Shin, Ho Joo Yoon

A254 Ocular Symptoms of Cedar Pollinosis in Otolaryngology Patients

Yukiyoshi Hyo

A255 The Clinical Characteristics of Adverse Drug Reactions Reported in a Regional University Hospital for 6 Years and the Suggestions for the Reporting System

Jaechun Lee, Su Hee Kim, Eunkyoung Lee

A256 Changes in Skin Prick Test Results over 3 Years in School-Aged Children

Hahn Jin Jung, Jaehyun Lim, Seung-No Hong, Doo Hee Han, Chae-Seo Rhee

A257 The Analysis of Risk Factors and Features of Food Allergy in Korean Children: Nationwide Cross-Sectional Survey

Kun Song Lee

A258 A Sequential Indirect-Direct Bronchial Provocation Test for Diagnosis of Asthma: A Pilot Study

Jaechun Lee, Sun Young Yang, Mi Young Ahn, Jong Hoo Lee, Jasmina Golez

A259 Association of *VDR* and *CYP2R1* Polymorphisms with Persistent Allergic Rhinitis in a Han Chinese Population

Hui-Qin Tian, Lei Cheng, Xin-Yuan Chen

A260 Associations of Metabolic Syndrome with Asthma and Atopy in Korean Adults

Ji-Yong Moon, Sang-Heon Kim, Tae Hyung Kim, Ji Young Yhi, Ho Joo Yoon, Jang Won Sohn, Dong Ho Shin, Dong Won Park

A261 Clinical Manifestation and Treatment Outcome of Eosinophilic Gastroenteritis in Korean Children

Won Im Cho, Jong Sub Choi, Dongin Suh, Gyeong Hoon Kang, Jin Soo Moon, Jae Sung Ko, Kyung Jae Lee, Shin Jie Choi

A262 The Sensitization Model and Correlation of Bermuda and Timothy Grass Pollen Allergen in Allergic Patients in Southern China

Wenting Luo, Baoqing Sun

A263 A Pilot Study on the Outcomes of Respiratory Allergic Diseases at Pre-School Age in Chinese Infants with Atopic Dermatitis

Qi Gao, Li Xiang, Kunling Shen

A264 Activation of Toll like Receptor 1 and 6 By House Dust Mite Enhances the Expression of Tight Junction Protein in Epidermal Keratinocytes

Yong Hyun Jang

A265 Pollen Exposure in a Mobile Exposure Chamber: Comparing Real-Life Symptoms with Exposure Symptoms

Karl-Christian Bergmann, Torsten Sehlinger, Georg Bölke, Uwe Berger, Torsten Zuberbier

A266 Retrospective Analysis of the Incidence of Allergy in Patients with Contact Eczema

Joanna Kolodziejczyk, Milena Wojciechowska, Anna Hnatyszyn-Dzikowska, Micha Chojnacki, Zbigniew Bartuzi

A267 Effect of Fungal Sensitization in Patients with Severe Asthma

Katsunori Masaki, Koichi Fukunaga, Takashi Kamatani, Kengo Ohtsuka, Takae Tanosaki, Masako Matsusaka, Takao Mochimaru, Hiroki Kabata, Soichiro Ueda, Yusuke Suzuki, Katsuhiko Kamei, Koichiro Asano, Tomoko Betsuyaku

A268 SCIg Patient Preference Pump Versus Push

Karlee Trafford

A269 Fixed Drug Eruption Induced By Ornidazole and Diclofenac

Ismet Bulut, Zeynep Ferhan Ozseker

A270 Transepidermal Water Loss Measurement during Infancy Can Predict the Subsequent Development of Atopic Dermatitis

Kenta Horimukai, Hideaki Morita, Masami Narita, Hironori Niizeki, Kenji Matsumoto, Yukihiro Ohya, Hirohisa Saito, Shigenori Kabashima, Mai Kondo, Eisuke Inoue

A271 Inhalant Allergens on Soft Toys: A Literature Review

Robert Siebers, Francis FS Wu

A272 Fractional Exhaled Nitric Oxide in Elderly Asthmatics

Robert Siebers, Francis FS Wu, Ming-Hui Ting, Hung-En Laio, Tsung-Huai Kuo, Pei-Yuan Lee

A273 Dye and Preservative Challenge in Meal-Associated Urticaria and Angioedema: A Low-Yield Diagnostic Maneuver

Daniel Eugene Maddox

A274 The Changes of Allergic Sensitization with Age in Children with Allergic Rhinitis

Gwanghui RyuHyo Yeol Kim, Hun-Jong Dhong, Sang Duk Hong, Seung-Kyu Chung

A275 Component-Specific IgE and IgG4 Levels in Milk Allergy Children Tolerated Baked Milk Products

Osamu Higuchi, Yu-Ichi Adachi, Toshiko Itazawa, Yoko Adachi, Miki Hamamichi, Motokazu Nakabayashi, Yasunori Ito, Takuya Wada, Gyoukei Murakami, Miki Takao, Junko Yamamoto

A276 Serum Surfactant Protein(SP)-D Level: A Potential Biomarker for Aspirin-Exacerbated Respiratory Disease

Hyun Jung Jin, Moon Gyeong Yoon, Young Min Ye, Yoo-Seob Shin, Seung-Hyun Kim, Hae-Sim Park

A277 Clinical Characteristics of Anaphylaxis in Korean Children

Taek-Ki Min, Bok-Yang Pyun, So-Yeon Lee, Hyun Hee Kim, Gwang-Cheon Jang, Jinho Yu, Dongin Suh, Sooyoung Lee, Yong Mean Park, Jeong Hee Kim, Hye-Yung Yum, Kyung Won Kim, Hyeon-Jong Yang, Kangmo Ahn, Ji-Won Kwon, Myung Hyun Sohn, Hae Ran Lee, Jung Hyun Kwon, Kyu-Earn Kim, Soo-Jong Hong

A278 Immunological Changes Induced By Intramuscular Injections of Autolologous Immunoglobulin in Patients with Severe Atopic Dermatitis

Su-Mi Cho

A279 Identification of Subtypes in Subjects with Mild to Moderate Airflow Limitation and Their Clinical and Socioeconomic Implications

Jin Hwa Lee, Chin Kook Rhee, Hye Yun Park, Woo Jin Kim, Yong Bum Park, Kwang-Ha Yoo

A280 Cephalosporin-Induced Dress (Drug Rash with Eosinophilia and Systemic Symptoms) Syndrome in a 7-Year-Old Boy

Heejeong Kang, Hyeon-Jong Yang, Taek-Ki Min, Bok-Yang Pyun

A281 Maternal Depression Is Associated with Children’s Asthma : An Analysis of the Fifth Korea National Health and Nutrition Examination Survey (2010-2012)

Lee Ju Suk, Cheol Hong Kim

A282 Increased Length of Hospitalization Associated with Infiltration on Chest Radiography in Pediatric Asthma Patients

Jung Hyun Kwon, Sang Hyun Lee, Wonhee Seo

A283 A Case of 16-Year-Old Boy with Smoking-Induced Acute Eosinophilic Pneumonia

Kang-in Kim, Young Cheon Park, Hyeon-Jong Yang, Taek-Ki Min; Bok-Yang Pyun

A284 A Case of Pranlukast Induced Anaphylactic Shock

Sujeong Kim, Sun Jin, Jong-Myung Lee, Hye-Jin Jung, Jung-Wha Park

A285 Comparison of Asthma-Related Outcomes Between Metabolically Healthy Obese and Metabolically Unhealthy Obese Asthma Patients

Hyo-Jung Kim, Tae-Bum Kim, You Sook Cho, Hee-Bom Moon, Hyouk-Soo Kwon, So Young Park, So-Young Park, Jung-Hyun Kim, Bomi Seo, Min-Gu Kim, Youn Yee Kim

A286 Rick Factors Associated with Longer Length of Stay in Infants Hospitalized with Respiratory Syncytial Virus Bronchiolitis

Yena Lee, Taek-Ki Min, Hyeon-Jong Yang, Bok-Yang Pyun, Suk Hee Han, Suyeon Park, Jeongho Lee, Won-Ho Hahn

A287 Urinary Excretion of 9Î±, 11Î^2^-Prostaglandin F_2_ and Leukotriene E_4_ in Patients with Exercise-Induced Bronchoconstriction

Youhoon Jeon, Joo-Hee Kim, Tae-Rim Shin, Cheol-Hong Kim, In-Gyu Hyun, Jeong-Hee Choi

A288 The Aeroallergen Sensitization Pattern and Effect on Airway Hyperresponsiveness in Busan, Korea

Sun-Mi Jang, Hae-Jung Na, Seung-Eon Song, Hye-Kyung Park, Eun-Jung Jo

A289 Multicenter Questionnaires on Current Management of Atopic Dermatitis Among Korean Patients and Caregivers

Dong Hun Lee, Jin-Young Lee, Yang Park, Jae-Won Oh, Mi Hee Lee, Soo-Jong Hong, Soo-Jong Hong, So-Yeon Lee, Joon Soo Park, Dong-Ho Nahm, Hye-Yung Yum, Hye-Yung Yum

A290 Der p 1, Der p 2 and Der p 10 IgE Reactivities in Allergic Rhinitis Patients in Korea

Kyu Young, Dong-Young Kim

A291 De-Labeling Beta-Lactam Hypersensitivity: An Experience from a Tertiary Care Hospital in Thailand

Sirinoot Palapinyo; Jettanong Klaewsongkram

A292 Sonic Hedgehog Signaling: Evidence for Its Protective Role in Endotoxin Induced Acute Lung Injury Mouse Model

Xing Chen, Yuting Jin, Xiaoming Hou, Fengqin Liu, Chunyan Guo, Yulin Wang

A293 Analyses of the Factors behind the Negative Attitudes Toward the Administration of Adrenaline Auto-Injectors in School Settings

Ikuo Okafuji, Yuya Tanaka, Shegeyuki Narabayashi, Satoru Tsuruta

A294 Low Vitamin D Levels Are Related to High House Dust Mite Sensitization in Patients with Severe Atopic Dermatitis

Yong Hyun Jang

A295 Appendicular Skeletal Muscle Mass Index: A Potential Predictor of Skeletal Muscle Abnormality According to the Severity Airflow Limitation of COPD

Jun-Hong Ahn, Dong-Won Lee, Jin Hong Chung, Hyun Jung Jin, Min-Su Sohn

A296 Etiology and Clinical Feature of Oral Allergy Syndrome in Children

Young a Park, Kyunguk Jeong, Yoon Hee Kim, In Suk Sol, Seo Hee Yoon, Kyung Won Kim, Myung Hyun Sohn, Kyu-Earn Kim, Sooyoung Lee

A297 Traffic-Related Pollution Levels and Poorly Controlled Asthma in Adults

Ho Kim, Ja Yeun Kim

A298 Anaphylaxis in Korean Children, 2009-2013 : Triggers of Anaphylaxis By Age Groups

So-Yeon Lee, Taek-Ki Min, Tae-Won Song, Kangmo Ahn, Jihyun Kim, Gwang-Cheon Jang, Hyeon-Jong Yang, Bok-Yang Pyun, Ji-Won Kwon, Myung Hyun Sohn, Kyu-Earn Kim, Jinho Yu, Soo-Jong Hong, Jung-Hyun Kwon, Sung-Won Kim, Sooyoung Lee, Woo Kyung Kim, Hyung Young Kim, Hye-Young Kim, Youhoon Jeon

A299 Maternal Allergy Is Associated with Acute Bronchiolitis Severity in Infant

Chang Hoon Lim, Yeongsang Jeong, Su Jung Kim

A300 Evaluation of Inflammatory Mediator Profiles in Sputum of Asthmatics As an Endotype for Refractory Asthma

Hun Soo Chang, Jeong-Seok Heo, Da-Jeong Bae, Jong-Uk Lee, Ji-Na Kim, Chang-Gi Min, Hyun Ji Song, Jong-Sook Park, Soo Hyun Kim, Choon-Sik Park

A301 Autophagy Is Associated with the Severity of Asthma in an Ovalbumin-Specific Mouse Model of Allergic Asthma

Jing-Nan Liu, Youngwoo Choi, Yoo Seob Shin, Hae-Sim Park

A302 Interleukin-9 and Interleukin-33 Levels in Children with Asthma

Nima Rezaei, Sedigheh Bahrami Mahneh, Arezou Rezaei, Maryam Sadr, Masoud Movahedi

A303 Pediatric Anaphylaxis at a University Hospital in Cheonan, Korea, 2013~2014

Jun Seak Gang, Joon Soo Park, Seung Soo Kim, Hyun Ho Bang, Kyeong Bae Park, Hye Sun Kim, Tae Ho Kim, Young Hwangbo, Hyun Jung Lee, Gyeong Hee Yoo, Young Chang Kim

A304 Initial Antigen-Specific IgE Levels Predict Clinical Outcome of Rush Oral Immunotherapy for Food Anaphylaxis

Sakura Sato, Noriyuki Yanagida, Motohiro Ebisawa

A305 *ABCC4* gene Polymorphism Is Associated with High Periostin Levels in Asthmatic Patients

Sailesh Palikhe, Hae-Sim Park, Seung-Hyun Kim, Ri-Yeon Kim, Eun-Mi Yang

A306 The Role of Clinical Phenotype and Allergen Sensitization at 2 Years As Predictors of Atopic Disorders at 5 Years

Li Yuan Gabriella Nadine Lee, Marion Aw, Marion Aw, Bee Wah Lee, Bee Wah Lee, Evelyn Xiu Ling Loo, Yiong Huak Chan, Lynette Shek, Lynette Shek, I-Chun Kuo, I-Chun Kuo, Phaik Ling Quah, Phaik Ling Quah, Genevieve Llanora, Gerez Irvin

A307 The Effect of Korean Red Ginseng (KRG) on Rhinovirus Infection in Human Nasal Epithelial Cells

Joo Hyun Jung, Il Gyu Kang, Seon Tae Kim, Hyoungmin Park

A308 The Effect of Korean Red Ginseng on the Symptoms and Allergic Inflammation in Patients with Allergic Rhinitis

Seon Tae Kim, Joo Hyun Jung, Il Gyu Kang, Hyoungmin Park, Kwang-Pil Ko

A309 Validation of the Newly Developed Multiple Allergen Simultaneous Test in Korea

Jungsoo Lee, Howard Chu, Hemin Lee, Jung U Shin, Chang Ook Park, Kwang Hoon Lee, Kwang Hoon Lee, Hong Kyu Kang

A310 Assessment of Symptoms Severities of Allergic Rhinitis Patients Sensitive to Multiple Allergens in Skin Prick Test

Dong Chang Lee, Geun Jeon Kim, Jae Hyung Hwang, Jin Bu Ha, Su Hee Jeong

A311 Diurnal Temperature Range and Emergency Department Visits for Asthma in Korea 6 Cities

Ho Kim, Shinha Hwang, Whahee Lee

A312 Mannan-Binding Lectin Serum Levels in Atopic Mongolian Adults

Enkhbayar Bazarsad, Logii Narantsetseg, Munkhbayarlakh Sonomjamts

A313 Prevalence of Doctor Diagnosed Atopic Eczema, during 2003-2014 in KOREA ; Using Big Data of 48.1 Million South Korean Health-Care Records

Gwang-Cheon Jang, Hyun-Hee Lee, Chang-Jong Lee, Huynsun Lim

A314 Association of Recurrent Wheeze with Lung Function and Airway Inflammation in Preschool Children

Ji-Eun Soh, Dae-Jin Song, Ji-Won Kwon, Hyung Young Kim, Ju-Hee Seo, Byoung-Ju Kim, Hyo-Bin Kim, So-Yeon Lee, Gwang-Cheon Jang, Woo Kyung Kim, Young-Ho Jung, Soo-Jong Hong, Jung Yeon Shim

A315 Mannan-Binding Lectin Serum Levels in Healthy Mongolian Adults

Enkhbayar Bazarsad, Logii Narantsetseg, Munkhbayarlakh Sonomjamts

A316 Rotanebuliser

Prabhakarrao Pv, Ranjitha Nadendla

A317 The Level of Serum Interleukin 13 and Interleukin 17A and Its Effect Factors in Children with Asthma

Juan Fang, Jing Zhao

A318 Is Vitamin D Insufficiency Also Involved in Childhood Asthma in South Korea?

Dae-Jin Song, Sungchul Seo, Young Yoo, Yu-Ri Kim, Ji Tae Choung, Jee Hoo Lee

A319 Collection of Nasal Secretions for Measurement of Local IgE: A Quest for the Best Method

Margot Berings, Natalie De Ruyck, Claus Bachert, Philippe Gevaert, Gabriële Holtappels

A320 The Role of Claudin 5 in a Murine Model of Asthma

Pureun-Haneul Lee, Byeong-Gon Kim, Choon-Sik Park, George D Leikauf, An-Soo Jang

A321 Claudin-4 in a Murine Model of Asthma: Modulation By Acrolein, a Highly Reactive Unsaturated Aldehyde

Byeong-Gon Kim, Pureun-Haneul Lee, Choon-Sik Park, An-Soo Jang

A322 Efficacy and Safety of Sublingual Immunotherapy in House Dust Mite Sensitized Children with Allergic Rhinitis

Yang Park

A323 The Association of Vitamine D Deficiency and Skeletal Muscle Dysfunction in Chronic Airway Disease

Min-Su Sohn, Hyun Jung Jin, Dong-Won Lee, Jun-Hong Ahn, Jin Hong Chung

A324 Bacteria Derived Extracellular Vesicles in Indoor Dust Is Closely Associated with Airway Disease and Lung Cancer: Analysis of Indoor Dustâ€™s Microbiome and IgG Sensitization of Indoor Bacteria Derived Extracellular Vesicles

Sae-in Kim, Han-Ki Park, Do-Yeon Kim, Mina Rho, Jun-Pyo Choi, Yoon-Keun Kim

A325 Clinical Care Program for Childhood Asthma (CCP-Childhood Asthma); A Multidisciplinary Team Care at Samitivej International Children’s Hospital

Wasu Kamchaisatian, Thitikul Hiranras, Surinda Wongpun, Phornthip Chiraphorn, Anupan Tantachun, Wannipa Wongrassamee, Planee Vatanasurkitt, Naratip Somboonkul, Nattipat Juthacharoenwong, Surangkana Techapaitoon, Montri Tuchinda

A326 Continuous B Cell Stimulation with CD40 Ligand Induce IgE Isotype Switching

Jae Ho Lee, Sejin An

A327 Effects of Interleukin-9 on Allergen-Specific Immunotherapy in a Mouse Model of Allergic Rhinitis

Ji-Hyeon Shin, Soo Whan Kim, Si Won Kim, Jun Myung Kang, Boo-Young Kim, Byung-Guk Kim

A328 Usefulness of Exhaled Nitric Oxide for Evaluating Wheeze and Airway Hyperresponsiveness in Preschool Children

Jung-Won Lee, Ji-Won Kwon, Woo Kyung Kim, Hyung Young Kim, Hyo-Bin Kim, Ju-Hee Seo, So-Yeon Lee, Gwang-Cheon Jang, Young-Ho Jung, Soo-Jong Hong, Byoung-Ju Kim, Dae-Jin Song, Jung Yeon Shim

A329 Systemic Cyclosporine Treatment in Hand Eczema Patients

Kyung Ho Kim

A330 Lipid Profiles and Adipokines in Korean Children with Atopic Dermatitis

Young Yoo, Won Suck Yoon, Sungchul Seo, In Soon Kang, Jae Won Choi, Hye-Young Lim, Ji Tae Choung

A331 Validation of Montelukast and Levocetirizine Combination Tablet Versus Individual Tablets in the Treatment of Moderate to Severe Persistent Allergic Rhinitis Among Adult Filipinos Seen at the Philippine General Hospital-Outpatient Department

Michelle Buela

A332 Efficacy of Makyokansekito on Treatment of Wheezing Lower Respiratory Tract Infection in Children: A Retrospective Study of 68 Patients

Koji Nishimura

A333 Serum Eosinophilia and Total IgE Are Associated with the Risk of Allergic Sensitization and Allergic Symptoms in Two Years Follow-up, Respectively

Sang Chul Park, Hyo Jin Chung, Chang-Hoon Kim, Ju Wan Kang, Seong-Chul Hong, Keun-Hwa Lee, Jaechun Lee, Hye-Sook Lee, Jeong Hong Kim

A334 The Sensitization to *Russian Thistle* on Mongolian Patients

Narantsetseg Logii

A335 The Association Between Air Pollution, Allergic Sensitization to Inhalant Allergens and Airway Hyperresponsiveness in Ulaanbaatar, Mongolia

Munkhbayarlakh Sonomjamts, Enkhbayar Bazarsad

A336 Pre-Coseasonal Treatment with a 5-Grass Pollen Sublingual Tablet in Adults Demonstrated a Reduction on Asthma Symptoms in Réunion Island

Bashir Omarjee

A337 Peak Expiratory Flow Rate Reference Values for Children Aged 5-14 Years Old in Beijing Urban Area

Shuo LI

A338 Soybean Storage Proteins As the Main Allergen in a Patient with Food-Dependent Exercise-Induced Anaphylaxis Due to Tofu

Miyuki Hayashi, Ruby Pawankar, Shingo Yamanishi, Toru Igarashi, Yasuhiko Itoh

A339 A Study of Allergy Skin Prick Test with Weed Pollen

Oyuntsatsral Batsaikhan, B. Gantulga, B. Enkhbayar, S. Munkhbayarlakh, L.Narantsetseg

A340 The Role of Neurotrophin in a Murine Model of House Dust Mite Induced Allergic Rhinitis

Pei-Chi Chen, Jiu-Yao Wang

A341 Mimotopes of the Major Shellfish Allergen Tropomyosin Suppress Splenocyte Proliferation and Local Cytokine Expression in a Mouse Model of Shellfish Allergy

Nicki Y. H. Leung, Christine Yee Yan Wai, Patrick S.C. Leung, Ka Hou Chu

A342 A Questionnaire Survey on Understanding of Atopic Dermatitis Among Korean Patients and Caregivers

Eun Jin Doh, Dong Hun Lee, Mira Choi, Hyun-Sun Yoon, Kyu Han Kim, Ji Soo Lim

A343 Comparison of the Dosage of Bronchodilators in the Bronchodilator Response Test in Children

Ji Hyeon Baek, Man Yong Han, Seung Jin Lee, Youhoon Jeon, Kyung Suk Lee, Young-Ho Jung, Hye Mi Jee, Youn Ho Shin

A344 The Expression and Effect of Natural Killer T Lymphocytes in Chidren with Asthma

Yi Jiang, Miao Liu

A345 Oral Provocation Test in Non-Steroidal Anti-Inflammatory Drug Hypersensitive Patients Referred to Singapore General Hospital

Chaw Su Naing, Tze Chin Tan, Yong Yeow Chong

A346 Different Phenotypes of Bhr (bronchial hyperresponsiveness) By Natural Course in Children and It’s Characteristics

Young-Ho Kim, Eun Lee, Song-I Yang, Hyun-Ju Cho, Hyung Young Kim, Ji-Won Kwon, Young-Ho Jung, Byoung-Ju Kim, Ju-Hee Seo, Ho-Jang Kwon, Hyo-Bin Kim, So-Yeon Lee, Soo-Jong Hong, Soo Hyun Kim

A347 Spectrum of Allergens Causing Allergic Rhinitis and Asthma in Urban Bangalore, India − a Study of 120 Patients

Jacqueline Elizabeth Joseph, Soumya M. S, Ruby Pawankar, Harshitha Kumar

A348 High Prevalence of Wheezing Illness and Risk Factor of Atopic Asthma Progression in Korean Preschool Children

Sohyoung Yang, Sung-Il Woo

A349 Clinical and Laboratory Screening of Primary Immunodeficiency Diseases: International Effects

Nima Rezaei

A350 The Effect of *Helicobacter Pylori* Infection in Atopic Individuals

Sukran Kose, Basak Gol Serin, Arzu Didem Yalcin, Süheyla Serin Senger, Mehmet Erden, Ertan Serin

A351 Clinical Spectrum and Natural History of Chronic Urticaria in Hong Kong Children

Agnes Sze-Yin Leung, Ting Fan Leung

A352 Skin Prick Test Reactivity to Common Pollen Aeroallergens in Patients with Allergic Rhinitis − in Urban Bangalore, India

Harshitha Kumar, Soumya M.S., Jacqueline Elizabeth Joseph, Ruby Pawankar

A353 Seasonal Patterns of Asthma-Related ED Visits and Admissions in Children and Adolescents Who Visited Emergency Rooms of Korea in 2007-2012

Eun Hee Chung

A354 Prevalence of Atopic Dermatitis and Its Associated Risk Factors in Elementary School Children: A Cross-Sectional Study in Gyeonggi-Do, South Korea

Eunji Kim, Young Yoo, Ji Tae Choung, Sungchul Seo, In Soon Kang, Jue Seong Lee, Ji Hyen Hwang

A355 Intralymphatic Immunotherapy for *Dermatophagoides Farinae*, *Dermatophagoides Pteronyssinus*, Cat, and/or Dog Allergy in Patients with Allergic Rhinitis: 1 Year Follow-up

Sang Min Lee, Joo Hyun Jung, Seung Joon Choi, Eugene Joe, Hyunjung Hwang, Shin Myung Kang, Yu Jin Kim, Sun Young Kyung, Jeong-Woong Park, Sung Hwan Jeong, Sang Pyo Lee

A356 Respiratory Syncytial Virus Regulates IL-33 Expression in Bronchoalveolar Cells and Lung Tissue in Vivo

Alina Gaisina, Igor Shilovskiy, Aleksandra Nikonova, Oleg Kamyshnikov, Musa Khaitov, Alexander Mitin, Komogorova Viktoriya, Marina Litvina, Nina Sharova

A357 The Prevalence of Parent-Perceived Food Hypersensitivity in Pre-School Children Attending a Tertiary Care Hospital in Malaysia

Faizah Mohamed Jamli

A358 Th2 Dominant Airway Inflammation Induced By House Dust Mite Chitin Is Dependent on TNF-a and NKT Cell

Da-Il Yoon, Jun-Pyo Choi, Han-Byul Choi, Yoon-Keun Kim, Hyeon-Il Choi

A359 Geographic Variations in the Patterns of Sensitization to Aeroallergens in Korean Adults: A Multi-Center Study

Mingyu Kang, Mi Yeoung Kim, Sujeong Kim, Eun-Jung Jo, Seoung-Eun Lee, Woo-Jung Song, Sang Min Lee, Chansun Park, Yoon-Seok Chang, Jaechun Lee, Young-Koo Jee, Inseon S Choi, Kyung-up Min, Sang-Heon Cho

A360 Experimental Mouse Model of Asthma Induced By Dust Mite *Dermatophagoides Pteronyssinus a*llergenic Extract

Anton Laskin, Oleg Kamyshnikov, Alexander Babakhin, Valentina Berzhets, Musa Khaitov

A361 Severe Refractory Pulmonary Complications in Children with *Mycoplasma Pneumoniae* Pneumonia

Sejin An, Jae Ho Lee

A362 Usefulness of Interactive e-Learning Education Program for Asthma Guideline

Sung-Yoon Kang, Yoon-Seok Chang, Kyung-up Min, Sang-Heon Cho, Sae-Hoon Kim, Yong Eun Kwon, Young-Koo Jee, Tae-Bum Kim, Hee-Bom Moon, Hye-Kyung Park

A363 Airway Inflammation Induced By House Dust Mite Derived Vesicles Is Mainly Induced By LPS Derived from Gram Negative Bacteria in Dust Mite.

Sang-Yoon Kim, Jun-Pyo Choi, Han-Ki Park, Ji-Hyun Lee, Yoon-Keun Kim

A364 Changes in the Recognition of Causal Allergen, Its Avoidance, and Allergen Specific Immunotherapy after Skin Prick Test / Intradermal Test, Nasal Provocation Test, and Intralymphatic Immunotherapy in Patients with Allergic Rhinitis: 1 Year Follow-up

Hyunjung Hwang, Eugene Joe, Sang Min Lee, Seung Joon Choi, Joo Hyun Jung, Yong Han Seon, Shin Myung Kang, Yu Jin Kim, Sun Young Kyung, Jeong-Woong Park, Sung Hwan Jeong, Sang Pyo Lee

A365 Laboratory Diagnostic of Staphylococcal Sensitization

Natalya Khramykhoverchenko

A366 Th-17 Regulatory Cytokines Enhance Neutrophil Production of IL-17 during Asthma

Saleh Al Muhsen, Asma Sultana, Rabih Halwani, Ahmed Bahammam

A367 Diagnostic Value of Serum Total IgE and Prediction of Cut-Off Value to Recommend Mast in Allergic Rhinitis

Hyung Chae Yang, Sun Kyung Kim, Kwang Il Nam

A368 Diagnostic Value of an Increase in FEV1 and/or FVC >12% and >200 mL from Baseline after Bronchodilators for Diagnosis of Asthma

Jeong-Eun Kim, Ju Suk Lee, Ji Hyun Lee, Kyung Woo Kang

A369 Combined Use of Fractional Exhaled Nitric Oxide and Bronchodilator Response in Predicting Future Loss of Asthma Control Among Children with Atopic Asthma

Je-Kyung Kim, Youn-Soo Hahn, Jae-Yub Jung

A370 Antigen-Specific IgA Plays an Important Role in Mucosal Immune Response in Allergic Children : Measurement of Secretory IgA and Antigen-Specific IgA

Yosuke Baba, Sususmu Yamazaki, Eisuke Inage, Mari Mori, Yoshikazu Ohtsuka, Masato Kantake, Toshiaki Shimizu, Asuka Honjoh, Tomoaki Yokokura

A371 Why Teaching Pediatrics Trainees about Anaphylaxis and Its Acute Management Is Essential: Cross Sectional Survey.

Mehdi Adeli, Shaza Ali Mohammed Elhassan, Caroline Beck

A372 Prevalence and Clinical Characteristics of Local Allergic Rhinitis in Children

Min Sun Na, Heysung Baek, Seung Jin Lee, Ji Hyeon Baek, Jungwon Yoon, Sun Hee Choi, Young-Ho Jung, Youn Ho Shin, Man Yong Han

A373 House Dust Mites Sublingual Immunotherapy Can Influence the Long-Term Evolution of Severe Atopic Dermatitis and the Progression to Respiratory Allergy.

Enrico Compalati, Maurizio Marogna

A374 The Positive Distribution Characteristics of 90 Food Specific IgG in Patients with Allergic Diseases

Huimin Huang, Baoqing Sun, Mingyu BAI, Yiting Huo, Peiyan Zheng, Nili Wei, Wenting Luo

A375 Evaluation of Serum Levels of Osteopontin As a Potential Biomarker of Immune Activation in Patients with Allergic Diseases

Elisa Villa, Anand Andiappan, Rosalba Minisini, Olaf Rötzschke, Elena Boggio, Luca Gigliotti, Nausicaa Clemente, Annalisa Chiocchetti, Umberto Dianzani, Mario Pirisi

A376 Prevalence of Allergic Rhinitis in 3-6-Year-Old (preschool) Children in Chiba City (urban area), Japan

Fumiya Yamaide, Syuji Yonekura, Naoki Shimojo, Yuzaburo Inoue, Yoshitaka Okamoto

A377 Comparative Efficacy of Combination Nebulized Salbutamol and Fluticasone Propionate and Nebulized Salbutamol in Children with Mild Moderate Asthma Attack

Retno Asih Setyoningrum, Landia Setiawati, Sri Sumei, Deddy Iskandar

A378 Characteristics of Children Hospitalized with Asthma in West Nusa Tenggara General Hospital Mataram Indonesia

Indriyani Sang Ayu Kompiyang

A379 Identification of Phenotypes in Allergic Bronchopulmonary Aspergillosis Using Cluster Analysis

Tsuyoshi Oguma, Jun Tanaka, Katsuyoshi Tomomatsu, Koichiro Asano

A380 The Roles of Type 2 Innate Lymphoid Cells (ILC2) in Chronic Rhinosinusitis (CRS)

Keisuke Uno, Yoshinori Matsuwaki, Kazuhiro Omura, Eika Hayashi, Norifumi Tatsumi, Hirohito Kita, Nobuyoshi Otori, Hiromi Kojima

A381 Respiratory Symptoms, Signs and Spirometry Indexes Comparision in 7-12 Years Old Girls in Esfahan Metropolis and Its Far Suburb

Mohammadreza Fatemi Khorasgani

A382 Induction of Kruppel-like Transcription Factor (KLF4&5) By Baker’s Yeast Mannan in Human Bronchial Epithelial and Smooth Muscle Cells

Dukhee/Betty Lew, Kim/S. Lemessurier, Joseph/a Moore, Jeoung-Eun Park, Ae-Kyung Yi, Chi/Young Song, Kafait/U Malik

A383 Korean Profile in Childhood Asthma Severity Classification

Dongin Suh, Ja Kyoung Kim, Hyeon-Jong Yang, Bong-Seong Kim, Youn Ho Shin, So-Yeon Lee, Geunhwa Park, Woo Kyung Kim, Hyo-Bin Kim, Heysung Baek, Dae Hyun Lim, Dae Hyun Lim, Jin Tack Kim

A384 Prevalence of Food Sensitization, IgE-Mediated and Non-IgE-Mediated Food Allergy Among Pediatric Patients Diagnosed with Autism Spectrum Disorders

Aimee Lou Manalo Nano

A385 Component-Resolved Diagnostic Study of *Dermatophagoides Pteronyssinus* Major Allergen Molecules in a Southern China

Wenting Luo, Baoqing Sun

A386 Risk Factors for Systemic and Local Reactions to Subcutaneous Allergen Immunotherapy

Hikmet Tekin Nacaroglu, Semiha Bahceci Erdem, Ozlem Sumer, Sait Karaman, Canan Sule Unsal Karkiner, Suna Asilsoy, Ilker Gunay, Demet Can

A387 Literature Review and Current Treatment Options for Cyclical Anaphylaxis

Danielle Kiers

A388 The Effect of Surfactant Protein D in Acute Lung Injury and Pulmonary Fibrosis Induced By Bleomycin

Hsu Han Yin, Jiu-Yao Wang

A389 Activation of Endothelial Cells to Release Hsp90, an Activator of the Prekallikrein-High Molecular Weight Kininogen (HK) Complex

Allen Kaplan, Kusumam Joseph, Baby G. Tholanikunnel

A390 The Effect of Climatic Treatment in 51 Asthmatic Children from Areas Severely Polluted Environment of Northern Moravia, Czech Republic

Radim Dudek

A391 Comparison of Some Vitamin Groups in Asthmatic Patients

Gulden Bilgin, Hatice Surer, Aytun Sadan Kilinc, Dogan Yucel

A392 Sensitization in Children with Atopic Dermatitis: A Single Center Study

Ji Young Lee, Jihyun Kim, Hea-Kyoung Yang, Minji Kim, Sang-Il Lee, Kangmo Ahn

A393 Staphylococcal Enterotoxin IgE Sensitization: A Risk Factor for COPD Overlap in the Elderly Asthma?

Sung Do Moon, Byung-Keun Kim, Sang-Heon Cho, Kyung-up Min, Yoon-Seok Chang, Heung Woo Park, Hye-Ryun Kang, Woo-Jung Song, Min-Koo Kang, Ju-Young Kim, Kyonghee Sohn, Ha Kyung Won, Seoung-Eun Lee, Kyung-Mook Kim, Claus Bachert

A394 The Effects of Probiotics and PparÎ^3^ on the Murine Model of Allergic Asthma

Miao-Hsi Hsieh, Jiu-Yao Wang

A395 Adult Patients’ Views on the Design of Adrenaline Autoinjectors

Helen Smith, Clare Brown, Christina Jones, Mark Davies

A396 CCL22 miRNA modulated Th1 responses and induced therapeutic effects on OVA-induced mouse model of asthma

Won Suck Yoon

A397 Clinical, Histological, and Skin Microbiome Characteristics of Head and Neck Dermatitis in Atopic Dermatitis

Hemin Lee, Howard Chu, Jungsoo Lee, Jung U Shin, Chang Ook Park, Kwang Hoon Lee, Seo Hyeong Kim, Ji Yeon Noh, Ji Hye Kim

A398 MicroRNA-432 modulates Th1 responses and induced therapeutic effects in atopic like murine model.

Won Suck Yoon

A399 Case Report of Near-Fatal Asthma Due to Snail Allergy in a House Dust Mite-Allergic Adult

Jean-Pierre L’huillier, Jean-Eric Autegarden, Catherine Bertrand, Dominique Tardy

A400 Relationship Between Gut Microbiota in the First 3 Months of Life and Infant Immune Function at Age 12 Months

Intan Hakimah Ismail, Mimi Tang, Paul Licciardi, Frances Oppedisano, Robert Boyle, Roy Robins-Browne

A401 A Pediatric Case of Food-Dependent Exercise-Induced Anaphylaxis Due to Spice Allergy

Hisako Yagi, Harumi Koyama, Yutaka Nishida, Takumi Takizawa, Hirokazu Arakawa

A402 Correlations Between Objective Severity Score and Each of the Subjective Severity Intensity in Atopic Dermatitis

Hong Kyu Kang, Hemin Lee, Jungsoo Lee, Jung U Shin, Kwang Hoon Lee, Kwang Hoon Lee, Howard Chu, Chang Ook Park

A403 Barrier Related Gene Mutations in Atopic Dermatitis

Na Young Yoon, Hyeyoung Lee, Seong Jun Seo, Eunhee Choi, Hye-Young Wang, Minyoung Jung, Eung Ho Choi, Dong Hye Kim

A404 Clinical Utility of Basophil Activation Test (BAT) in the Diagnosis of Drug Induced Anaphylaxis

Joo-Hee KimYoung-Sook Jang, Jeong-Hee Choi, Sunghoon Park, Young Il Hwang, Seung Hun Jang, Ki-Suck Jung

A405 Feeding Shapes the Colonization of Gut Microbiota and Associated with Total IgE in Infant

Mi-Jin KangDongin Suh, Eun Lee, Kil Yong Choi, Young-Ho Jung, Song-I Yang, Bong-Soo Kim, Ha-Jung Kim, Juneyoung Koh, Hyun-Jin Kim, Kangmo Ahn, Youn Ho Shin, Hyun-Ju Cho, Byoung-Ju Kim, Young-Ho Kim, Yean Jung

A406 CD8^+^T Cell-Intrinsic Smad4 Suppresses Th2 Responses in the Pathogenesis of Contact Hypersensitivity

Mizuko Mamura, Jeong-Hwan Yoon, Susumu Nakae, Inkyu Lee, Isao Matsumoto, Takayuki Sumida, Jin Soo Han, Katsuko Sudo, Ji Hyeon Ju

A407 Immune-Modulatory Genomic Properties Differentiate Gut Microbiotas of Infants with and without Eczema

Gaik Chin Yap, Wen Tso Liu, Seungdae Oh, Pei Ying Hong, Chiung Hui Huang, Marion Aw, Lynette Shek, Bee Wah Lee

A408 The Effect of Medication in OSA Patients with Allergic Rhinitis

Young Seok Byun, Sung Wan Kim, Tae Kyung Koh, Joong-Saeng Jo, Kun Hee Lee, Chul Kwon, Sung-Hwa Dong

A409 A Case of Generalized Pustular Psoriasis Mimicking Acute Generalized Exanthematous Pustulosis

Myung Shin Kim, Chansun Park

A410 Anaphylaxis Caused By Gummy Jelly Ingestion: A Case Report

Han Seok Cho, Min-Ju Kim, Min Ji Kim, Young Ok Park, Hye Yeong Lee, Hee Seong Kim, Eun Lee, Hyun-Ju Cho, Jinho Yu, Soo-Jong Hong, Keum Hee Hwang

A411 Serum Folliculin As a Novel Biomarker for Asthma

Jung-Hyun Kim, You Sook Cho, Sae-Hoon Kim, Hyouk-Soo Kwon, Mira Yoo, Hyo-Jung Kim, So-Young Park, Bomi Shin, So Young Park, Bomi Seo, Min-Gu Kim, Hee-Bom Moon, Jin-Ah Park, Tae-Bum Kim, Jaemoon Lee

A412 Corticosteroid Nasal Irrigations after Endoscopic Sinus Surgery in the Management of Chronic Rhinosinusitis with Asthma

Jin Hyeok Jeong, Tae Wook Kang, Han Seok Yoo, Yong Hee Cho, Seok Hyun Cho, Kyung Rae Kim

A413 Capsaicin Injection in Neonatal Period Potentiates Intensity and Duration of Atopic Dermatitis of Rats.

Jue Seong Lee, Sun-Ho Kee, Sewon Kim, Young Yoo, Heung Sik Na, Seung Keun Back

A414 Comparison Between the Impulse Oscillometry System, Spirometry, Feno, Lung Clearance Index and Asthma Control and Exacerbation Status.

Seung Jin Lee, Bo Seon Seo, Ji Hyeon Baek, Kyung Suk Lee, Young-Ho Jung, Hye Mi Jee, Youn Ho Shin, Man Yong Han, Mi-Ae Kim

A415 The Association of Exhaled Nitric Oxide and Airway Hyperresponsiveness in Patients with and without Asthma

Young-Hee Nam, Dong Sub Jeon, Soo-Keol Lee

A416 Effects of Air Pollution on Allergic Rhinitis in Korea

Jisun Park

A417 Exhaled Nitric Oxide in Korean Children with Allergic Rhinitis

Seung Hyun Moon

A418 A Questionnaire of Children with Asthma or Asthma and Allergic Rhinitis

Rong Jun Lin, Ren Zheng Guan

A419 A Case of Trimebutine-Induced Morbilliform Skin Eruption

Gyeong Yul Park, Hyun-Sun Yoon

A420 Comparison of Methacholine and Mannitol to Predict Exercise-Induced Bronchoconstriction in Children with Asthma

Woo-Hyeok Choi, Heysung Baek

A421 Different Inflammatory Mechanisms of Human Metapneumovirus and Respiratory Syncytial Virus

Jin-Sung Park, Eunmi Kwon, Zac Callaway, Chang-Keun Kim, Takao Fujisawa

A422 Sputum Microbiota in Chinese Adults with Eosinophilic Versus Non-Eosinophilic Asthma

Qingling Zhang, Rihuang Qiu, Naijian Li, Zhaowei Yang, Jing Li, Kian Fan Chung, Nanshan Zhong

A423 Which Clinical Features Are Useful in Predicting Presence of *Staphylococcus Aureus* colonization/Infection in Childhood Atopic Dermatitis?

Kam Lun E. Hon, Yin Ching K. Tsang, Ting Fan Leung

A424 Clinical Significance of Increased VEGF, TGF-Î^2^_1,_ and YKL-40, a Chitinase like Protein, in Serum of the Children with Asthma

Yoon Young Jang, Hai Lee Chung, Seung Gook Lee, Ji Hyun Na, Jong Hoon Lee

A425 Analysis of Follow-up Results of Mannitol Challenge Test in Asthma Patients

Young-Hee Nam, Dong Sub Jeon, Soo-Keol Lee

A426 Analysis of 68 Oral Walnut Challenge Tests

Mikita Yamamoto, Sakura Sato, Noriyuki Yanagida, Ayako Ogawa, Kanako Ogura, Kyohei Takahashi, Kenichi Nagakura, Shigehito Emura, Tomoyuki Asaumi, Katsuhito Iikura, Motohiro Ebisawa, Yu Okada

A427 Effectiveness of Air Filters Intervention in Allergic Rhinitis

Jiaying Luo, Xiao Lan, Baoqing Sun, Zhao Chen, Guiyuan Sun, Shimin Li, Jiaqing Hu

A428 The Relationship Between Airway Hyperresponsiveness to Mannitol and Atopy in Asthmatic Children

Woo-Hyeok Choi, Heysung Baek

A429 Anaphylactoid Reactions to N-Acetylcysteine in the Treatment of Aacetaminophen Overdose

Young-Hee Nam, Dong Sub Jeon, Hee-Joo Nam, Yeo Myeong Noh, Sang Hee Kim Kim, Ye Suel Park, Soo-Keol Lee

A430 Effect of Prenatal Maternal Distress and GSDMB Polymorphism on the Development of Recurrent Wheezing in Early Childhood: COCOA Study

Yean Jung Choi, Si Hyeon Lee, Young-Ho Kim, Mi-Jin Kang, Hyun-Ju Cho, Eun Lee, Song-I Yang, Youn Ho Shin, Kangmo Ahn, Kyung Won Kim, Yoon Hee Kim, So-Yeon Lee, Hyoung Yoon Chang, In Ae Choi, Kyung-Sook Lee, Yee-Jin Shin

A431 Vitamin D Level in Allergic Rhinitis: A Systemic Review and Meta-Analysis

Yoon Hee Kim, Min Jung Kim, In Suk Sol, Seo Hee Yoon, Young a Park, Kyung Won Kim, Myung Hyun Sohn, Kyu-Earn Kim, Yong Ju Lee

A432 Implication of Inspiratory and Expiratory Resistance and Reactance in Children with Asthma

In Suk Sol, Kyu-Earn Kim, Yoon Hee Kim, Min Jung Kim, Seo Hee Yoon, Yong Ju Lee, Kyung Won Kim, Young a Park, Myung Hyun Sohn

A433 The Association of Asthma Predictive Index with Asthma in Preschool Children with Recurrent Wheeze

Sung Joo Park, Ji-Won Kwon, Woo Kyung Kim, Hyung Young Kim, Hyo-Bin Kim, Ju-Hee Seo, So-Yeon Lee, Gwang-Cheon Jang, Young-Ho Jung, Soo-Jong Hong, Byoung-Ju Kim, Dae-Jin Song, Yun Seok Yang, Jung Yeon Shim

A434 Clinical Significance of Serum Total IgE Levels in Children with RSV-Associated Lower Respiratory Illness

Yoon Young Jang, Hai Lee Chung, Ji Hye Kim, Hyun Seok Lee, Chang Ho Lee

A435 Development of a Oak Pollen Emission and Transport Modeling Framework in South Korea

Changbum Cho, Yun-Kyu Lim, Kyu Rang Kim, Mijin Kim, Baek-Jo Kim

A436 Temperature, Humidity, and Air Pollution Affect Atopic Dermatitis Symptoms in Infants and Young Children

Young-Min Kim, Youngshin Han, Jihyun Kim, Hae-Kwan Cheong, Byoung-Hak Jeon, Kangmo Ahn

A437 Effects of Compound V on Pulmonary Fibrosis Model

Chuang/Yao Ming, Jiu-Yao Wang, Ye/Yi Ling

A438 Vitamin D Level and the Correlation with IgE in Children with Allergic Respiratory Diseases in Guangzhou China

Huimin Huang, Baoqing Sun, Yun Chen, Peiyan Zheng, Nili Wei, Wenting Luo

A439 Two Case Reports of Eosinophilic Gastroenteritis Associated with Allergic Disease

Do Hyeong Lee, Gil-Soon Choi, Hee-Kyoo Kim, Han Su Park

A440 Genome-Wide Association Study (GWAS) May Identify Common Genetic Variations Both in Immediate and Delayed Drug

So-Young Park, Hyo-Jung Kim, Bomi Seo, Jung-Hyun Kim, Min-Gu Kim, Hyouk-Soo Kwon, You Sook Cho, Hee-Bom Moon, Tae-Bum Kim, Yoon Su Lee

A441 Development of a Questionnaire for Secular Change of Atopic Dermatitis from Birth to 19-Year-Old.

Akio Tanaka, Satoshi Morioke, Yukihiro Ohya, Naoki Shimojo, Akira Akasawa, Michihiro Hide, Hiroko Shizukawa

A442 Evaluation of the Adherence Starts with Knowledge-20 (ASK-20) to Inhaled Drug in Patients with Bronchial Asthma

Naoto Watanabe

A443 The Epidemiology and Clinical Manifestation of Hmpv Infection in Children during Recent 4 Years: 2011-2014

Meeyong Shin, Myeong Sun Jang

A444 Neutropenia Induced By Intravenous Immunoglobulin

Young-Hee Nam, Yeo Myeong Noh, Dong Sub Jeon, Hee-Joo Nam, Sang Hee Kim Kim, Ye Suel Park, Soo-Keol Lee

A445 A First Case of Lymphocytic Interstitial Pneumonitis in Healthy Child

Ji-in Jung, Ha-Su Kim, Hyun-a Kim, Jin-a Jung

A446 Cytokine Production upon House Dust Mite Stimulation of Cord Blood Mononuclear Cells from Caesarean Section-Delivered Singaporean Infants

Anne Goh, Rajeshwar Rao, Bindu Nandanan, Ruurd Van Elburg, Chua Mei Chien, Juandy Jo, Johan Garssen, Johan Garssen, Leon Knippels, Elena Sandalova, Wen Chin Chiang

A447 Dress Syndrome with Acute Interstitial Nephritis Caused By Quinolone and Nonsteroidal Anti-Inflammatory Drugs

Young-Hee Nam, Ji Young Juong, Soo Jin Kim, Eun Young Kim, Su Mi Lee, Young Ki Son, Hee-Joo Nam, Ki-Ho Kim, Soo-Keol Lee

A448 IL-23 Roles in the Development of House Dust Mite Allergic Sensitization and Asthma

Da-Eun Park, Hye-Ryun Kang, Heung Woo Park, Hyun Seung Lee, Yoon-Seok Chang, Jung-Won Park, Sang-Heon Cho, Kyung-up Min, Woo-Jung Song

A449 Exposure Profile of Indoor Risk Factors in Dwellings of Children with Atopic Dermatitis

Hyunwook Lim, Sungchul Seo, Ji Tae Choung, Young Yoo, Jun-Sik Park, Byung Kwan Kim

A450 Epidemiological Characterization of Blood Eosinophils in the Elderly Population

Ha Kyeong Won, Hye-Ryun Kang, Byung-Keun Kim, Sung Do Moon, Ju-Young Kim, So-Hee Lee, Woo-Jung Song, Heung Woo Park, Min-Koo Kang, Sun-Sin Kim, Sang-Heon Cho, Kyung-up Min, Yoon-Seok Chang, Kyoung Hee Sohn, Kyung-Mook Kim, Ki-Woong Kim, Hak Chul Jang

A451 Stevens-Johnson Syndrome Caused By Methotrexate in the Treatment of Psoriasis

Young-Hee Nam, Dong Sub Jeon, Hee-Joo Nam, Yeo Myeong Noh, Sang Hee Kim Kim, Ye Suel Park, Soo-Keol Lee

A452 Genetic Determinants for Lung Function Growth in Asthmatic Children

Ting Fan Leung, Man Fung Tang, Hing Yee Sy, Wa Cheong Chan, Wilson Wai San Tam

A453 The Power of Allergen Specific Ig E in the Classification of Rhinitis, Korean National Hanes 2010

Seung Kyu Chung, Sujin Kim, Sang Duk Hong, Hyo Yeol Kim Hyo Yeol Kim, Hun-Jong Dhong, Jong in Jeong

A454 Analysis of Allergen Immunotherapy Practice and PatientsÃ¢â‚¬â„¢ Knowledge and Attitude about Allergen Immunotherapy in a Single Tertiary Hospital in Korea

Young-Hee Nam, Dong Sub Jeon, Soo-Keol Lee

A455 Roles of Staphylococcal Enterotoxin B in House Dust Mite-Induced Acute Asthma Models

Ji Won Lee, Mingyu Kang, Soon-Hee Kim

A456 A Clinical Comparison of Drug Reaction with Eosinophilia and Systemic Symptoms Syndrome in a Single Tertiary Hospital in Korea

Young-Hee Nam, Dong Sub Jeon, Hee-Joo Nam, Yeo Myeong Noh, Sang Hee Kim Kim, Ye Suel Park, Soo-Keol Lee

A457 The Beneficial Effect of Lactobacillus Gasseri PM-A0005 and Its Immunoregulatory Protein PMA5P40 on Milk-Induced Allergic Enteritis

Yung-I Hou, Jiu-Yao Wang

A458 Relationship Between Serum Folate Levels and Risks of Allergic and Respiratory Diseases in Early Childhood: The Mothers and Children’s Environmental Health Study

Ja Hyeong Kim, Seol Jae Hee, Eun-Hee Ha, Hyesook Park, Mina Ha, Yun-Chul Hong, Yangho Kim, Namsoo Chang

A459 Effects of Vitamin D in Patients with Chronic Rhinosinusitis

Yuta Soma, So Watanabe, Ruby Pawankar, Ruby Pawankar, Harumi Suzaki, Harumi Suzaki, Hitome Kobayashi

A460 Clinical Features of Systemic Contact Dermatitis from Ingestion of Rhus

Young-Hee Nam, Chansun Park, Soo-Keol Lee

A461 Sublingual Immunotherapy Efficacy in Patients with Atopic Dermatitis in Korea

Jongrok Lee, Jooyoung Roh, Haryeong Ryu

A462 Characteristics of Serious Adverse Drug Reactions in a Tertiary University Hospital

Cheol-Woo Kim, Jae Hwa Cho, Mi Ra Eom, Ji Young Kang, Hye Gyeung Lee

A463 Eyelid Dermatitis: Patch Test Results during a 15-Year Period in Korea and Evaluation of Metal Contents in Eye Shadows

Hae Young Choi, Hye Jin Lee, Ju Yun Woo, Ji Yeon Byun, You Won Choi

A464 Relationship Between Lipid Levels and Risks of Allergic and Respiratory Diseases in Early Childhood: The Mothers and Children’s Environmental Health Study

Ja Hyeong Kim, Eun-Hee Ha, Hyesook Park, Mina Ha, Yun-Chul Hong, Yangho Kim, Namsoo Chang

A465 IL-32 in the Induced Sputum of Patients with Asthma

Jae-Woo Kwon, Hun Soo Chang, Jeong-Seok Heo, Jong-Uk Lee, Jong-Sook Park, Eusom Kim, Soo Hyun Kim, Choon-Sik Park

A466 Clinical Characteristics of Patients with Chromium Allergy in a Single University Hospital in Korea

Hae Young Choi, Ji Yeon Byun, Ju Yun Woo, You Won Choi

A467 Efficacy and Safety of Oral Acitretin in Chronic Hand Eczema

Hyun-Ju Jin, Jin-Hwa Son, Jeong-Min Kim, Gun-Wook Kim, Je-Ho Mun, Margaret Song, Hyun-Chang Ko, Moon-Bum Kim, Hoon-Soo Kim, Byung Soo Kim

A468 Atypical Antipsychotics and Anticholinergic Agents Mimicking Anaphylaxis

Sheryl Van Nunen, Dinh Van Nguyen, Anthony Elias, Susannah Olivia Lauer

A469 Introducing Reach (Reliable Estimation of Atopic dermatitis in ChildHood): Novel, Questionnaire-Based Diagnostic Criteria for Childhood Atopic Dermatitis

Seung-Chul Lee, Ho-June Lee, Jung Min Bae

A470 Comprehensive Assessment to Identify the Causative Factors in Oral Allergy Syndrome

Emi Ono

A471 Comparison of Interpretation Methods in Allergic Skin Test

Sung-Hwa Dong, Tae Kyung Koh, Young Seok Byun, Sung Wan Kim, Joong-Saeng Jo, Chul Kwon, Kun Hee Lee

A472 Refraining Aminophylline Use Increases Hospitalization Among Children with Acute Asthma: A 10-Years Retrospective Cohort Study

Li-Fan Liu

A473 The Prevalence and Risk Factors of Atopic Dermatitis from Nationwide Study for Korean School Students

Sunghee Lee

A474 Probiotic Recombination Protein Effect on Atopic Dermatitis

Wei-Leng Chen, Jiu-Yao Wang

A475 Allergic Sensitization Status in Various Inflammatory Skin Diseases

Youin Bae, Gyeong-Hun Park

A476 Two Cases of Good’s Syndrome: A Rare Acquired Immunodeficiency Associated with Thymoma

Suk Yeon Kim

A477 IL-23 Has a Role to Play in the Development of Asthma in Short-Term Cigarette Smoke Exposure-Induced House-Dust Mite Allergic Model

Hyun Seung Lee, Woo-Jung Song, Mingyu Kang, Han-Ki Park, Da-Eun Park, Hye-Ryun Kang, Heung Woo Park, Yoon-Seok Chang, Hye-Young Kim, Kyung-up Min, Sang-Heon Cho, Ji-Won Lee, Boram Bae, Jung-Won Park

A478 The Relationship Between the Relevance of Allergic Disease and the Value of Non-Specific IgE

Yasuhiro Suzuki

A479 Two Caces of Prawn Allergy in Adult Patients

Ismet Bulut, Zeynep Ferhan Ozseker

A480 Early Gut *Bifidobacterium Breve* and *B. Catenulatum* Colonisation Differentially Modulate Eczema Risk in Children at High-Risk of Developing Allergic Disease

Intan Hakimah Ismail, Robert Boyle, Paul Licciardi, Frances Oppedisano, Roy Robins-Browne, Mimi Tang

A481 Effects of Chronic Repeated Exposure of Staphylococcal Enterotoxin B on Allergic Asthma Model in Mice

Ji Won Lee, Hyun Seung Lee, Mingyu Kang, Da-Eun Park, Han-Ki Park, Soon-Hee Kim, Woo-Jung Song, Hye-Ryun Kang, Heung Woo Park, Yoon-Seok Chang, Chang-Han Park, Suk-Il Chang, Sook-Hee Song, Kyung-up Min, Sang-Heon Cho, Boram Bae

A482 Skin Prick Test Result and Allergen Immunotherapy in Children with Allergic Rhinitis

Grace Shieh

A483 Role of Brp-39 in RSV-Induced Airway Inflammation in Mice

Min Jung Kim, Jung Yeon Hong, Seo Hee Yoon, Doo Hee Shim, In Suk Sol, Yoon Hee Kim, Mi Na Kim, Kyung Eun Lee, Kyung Won Kim, Myung Hyun Sohn, Kyu-Earn Kim, Jae Myun Lee

A484 Long-Term Outcomes of Twenty-Four Adults with Primary Immunodeficiency from a Single Centre in Singapore

Hiok Hee Chng

A485 Breast Feeding Increases the Risk of Food Sensitization but Does Not Affect Food Allergy in Young Children with Atopic Dermatitis

Dong Chan Kim, Song-I Yang, Hae Ran Lee, An Deok Seo, So Yeon Lee

A486 IgE Immunoadsorption Knocks Down Anaphylaxis.

Alessandro Fiocchi, Maria Cristina Artesani, Paola Francalanci, Lamia Dahdah, Thomas Schreiner

A487 Burden and Correlates of Cigarette Smoking and Respiratory Airway Obstruction: An Observation in Urban Adult Population of West Bengal (India)

Kaushik Chakraborty

A488 Blood Eosinophils Could Predict Sputum Eosinophilia? : A Comparison Between Asthma and Non-Asthmatic Chronic Cough in the Elderly

Ha Kyeong Won, Ju-Young Kim, Eun-Jung Jo, Kyoung Hee Sohn, Kyung-Mook Kim, Heung Woo Park, Yoon-Seok Chang, Sang-Heon Cho, Woo-Jung Song, Byung-Keun Kim

A489 Complementary and Alternative Medicine for Allergic Rhinitis in Japan

Syuji Yonekura, Yoshitaka Okamoto

A490 Impact of Cognitive Impairment on Asthma Control Status in Elderly Asthmatics

Gyu Young Hur, Young Min Ye, Joo-Hee Kim, Ki-Suck Jung, Junga Kim, Jae Jeong Shim, Hae-Sim Park

A491 The Association Between Respiratory Tract Infection and Reactive Oxygen Stress

Kazuhiro Sekimoto, Kazuko Sugai, Keiji Tsuchimoto, Hiromi Uehara, Masanori Ikeda

A492 The Risk Factors and Lung Function of Current Allergic Rhinitis Due to Dust Mite Sensitization

Euncho Chung, Kang Seo Park, Yean Jung Choi, Jeewon Park, Soo-Jong Hong, So Yeon Lee

A493 Cloning and Expression of Recombinant *Blomia Tropicalis* Dust Mite Allergen Blo t 7

Alain Jacquet, Arun Buaklin, Nat Malainual

A494 Seasonal Variations of Airborne Pollen in Bangalore, India

Roopashree S

A495 Pollen Observation and Use of Data

Kyu Rang Kim, Mijin Kim, Changbum Cho, Baek-Jo Kim, Jae-Won Oh, Mae Ja Han

A496 The Effect of Cord Serum 25-Hydroxyvitamin D (25(OH)D) on the Development of Atopic Dermatitis in First 3 Years of Life : Cocoa Study

Hyun-Ju Cho, Youn Ho Shin, Eun Lee, Young-Ho Kim, Darae Lee, Mi-Jin Kang, Song-I Yang, Kangmo Ahn, Kyung Won Kim, Yoon Hee Kim, Hye-Sung Won, Soo Hyun Kim, Suk-Joo Choi, Young Han Kim, Jong Kwan Jun, Eun-Jin Kim, Jeom Gyu Lee, So-Yeon Lee, Soo-Jong Hong, Dongin Suh

A497 Contribution of Stem Cell Factor Autocrine/Paracrine Mechanism to Aberrant Proliferation of Mast Cells

Yosuke Amagai, Akane Tanaka, Hiroshi Matsuda

A498 A Randomized Dbpc Dose-Finding Multicenter Trial of Sublingual Immunotherapy (SLIT) Allergoid Tablets in House Dust Mites (HDM) Allergic Patients

Ralph Mösges, Pauline Dieterich, Anatoli Astvatsatourov, Christoph Hüser, Jaswinder Singh, Kija Shah-Hosseini, Silke Allekotte, Enrico Compalati

A499 Depression and Allergy in the Elderly: A Community Population Analysis

Kyoung Hee Sohn, Woo-Jung Song, Byung-Keun Kim, Ju-Young Kim, Min Suk Yang, So-Hee Lee, Sae-Hoon Kim, Hye-Ryun Kang, Heung Woo Park, Sun-Sin Kim, Kyung-up Min, Sang-Heon Cho, Yoon-Seok Chang

A500 The Integrated Analysis of Correlation Between Total IgE and Other Immunological Factors in Allergic Diseases

Woo-Sung Chang, Ji-Hye Do, Yeon-Seop Kim, Dankyu Yoon, Hye-Sun Lim, Jeom-Kyu Lee, Eun-Jin Kim

A501 Pattern of Allergic Diseases Among Military Servicemen Referred to a Clinical Immunology/Allergy Service in Singapore

Bernard Thong, Yew Kuang Cheng, Jinfeng Hou, Khai Pang Leong, Justina Tan, Faith Chia, Grace Chan, Sze-Chin Tan, Teck Choon Tan, Chwee Ying Tang, Hiok Hee Chng

A502 A Case of Rifampicin-Induced Hypersensitivity Diagnosed By the Lymphocyte Activation Test with Successful Desensitization

Chan-Sun Park, Mi Yeoung Kim, Eun-Young Kim, Jae-Gook Shin, Jae-Hyeog Choi, Saegwang Park, Yeonye Kim

A503 Analysis of Individual Case Safety Reports of Drug-Induced Anaphylaxis Based on Korea Adverse Event Reporting System Database

Kyung-Hwan Lim, Jae Woo Jung, Mingyu Kang, Ju-Young Kim, Ju-Young Kim, Hyun Jeong Kim, Yeon-Ju Woo, Soo-Youn Jung, Hye-Ryun Kang, Hye-Ryun Kang

A504 Impact of Processes Certification on the Liability of Anti-Dust Mites Bed Covers

Thierry Porée, Nabile Boukhettala, Emeline Furon

A505 Localisation Kinetics of Aluminium after Subcutaneous Injection in a Rat Model

Alan David Bullimore, Matthew Heath, Simon Hewings, Murray Skinner

A506 Periostin Levels in Exhaled Breath Condensate of Competitive Athletes, Asthmatics and Healthy Subjects - Associations with Outdoor Ambient Conditions

Marcin Kurowski, Hubert Krysztofiak, Aleksandra Wardzynska, Marzanna Jarzebska, Janusz Jurczyk, Marek L. Kowalski

A507 The Role of PKR Pathway in Acute Exacerbation of Severe Bronchial Asthma

So Ri Kim, Yong Chul Lee, Dong Im Kim, Yang Keun Rhee, Heung Bum Lee, Seoung Ju Park, Yeong Hun Choe Choe, Seung Yong Park

A508 Diversity of Clinical Manifestations and Treatment Responses for Idiopathic Hypereosinophilic Syndrome

Joo-Hee Kim, Sunghoon Park, Young Il Hwang, Seung Hun Jang, Ki-Suck Jung

A509 Effect of Dexamethasone in Th17 Cell Mediating Neutrophilic Asthma

Nong Guang-Min, Jiang Min

A510 Allergen Profile for Asthma/Rhinitis and Eczema Among Patients in North India: An Immunocap Allergen Specific IgE Antibodies Assay Based Study

Nalin Nag

A511 Clinical Profile of Allergic Rhinitis in Children in Jakarta

Wahyuni Indawati

A512 Preclinical Study on the Use of Micro Crystalline Tyrosine (MCT) Adjuvants in Allergy Immunotherapy

Alan David Bullimore, Matthew Heath, Murray Skinner

A513 Genetic Diversity of Filaggrin Mutation and Its Clinical Implication in East Asian Atopic Dermatitis Patients

Seong Jun Seo, Won Jong Oh

A514 Protein and MPL Adsorption Capacities for MCT in Candidate Therapeutic Formulations for Use in Immunotherapy, Compared Against Existing Adjuvants

Alan David Bullimore, Murray Skinner, Matthew Heath, Andrew Bell

A515 Interleukin-22 Gene Variation in Inflammatory Bowel Disease

Alireza Zarebidoki, Hournaz Hasanzadeh, Salman Sadeghzade, Nima Rezaei

A516 Immunomodulatory Effects of Adipose-Derived Stem Cell Secretome in a Mouse Model of Asthma

Kyu-Sup Cho

A517 Diffuse Alveolar Hemorrhage with Positive Anti-Neutrophil Cytoplasmic Antibody in a Child : A Case Report

Sung-Woo Kim, Moo-Young Oh

A518 Clinical Analys the Serum TARC Levels As the Condition Index of Atopic Dermatitis in the Early Infancy

Munemitsu Koizumi, Kazuyo Kuzume

A519 An Analysis of the Filaggrin Gene Polymorphism in Korean Atopic Dermatitis Patients

Kui Young Park, Won Jong Oh

A520 A New Protocol for Wheat Oral Immunotherapy in Patients with Anaphylaxis

Delara Babaie, Mohammad Nabavi, Fariborz Zandieh, Mehrdad Amir Moini, Zahra Chavoshzadeh, Hamideh Seifi, Mitra Sahragard, Mehrnaz Mesdaghi, Mohammad Hassan Bemanian

A521 Clinical Characteristics of Filaggrin-Related Atopic Dermatitis Patients in Korea

Sun Young Choi, Yeon a No

A522 Early Allergy Diagnosis in Children - Self- Administered Questionnaire Vs Medical Verification

Andrzej M. Fal, Dorota Kiedik, Agnieszka Muszynska, Iwona Pirogowicz

A523 Asthma Impact on Children with Food Induced Anaphylaxis

Chikako Motomura, Masatoshi Wakatsuki, Yuko Akamine, Mihoko Iwata, Hiroshi Matsuzaki, Naohiko Taba, Yoko Murakami, Hiroshi Odajima

A524 Case Reports of Stevens-Johnson Syndrome and Stevens-Johnson Syndrome-Toxic Epidermal Necrolysis in Systemic Lupus Erythematosus

Reni Ghrahani

A525 The Effectiveness of Oral Tolerance Induction for Wheat Allergy Using Two Different Intake Levels

Yuri Takaoka

A526 Association of Plasma Interleukin-25 Levels with Development of Aspirin Induced Airway Spasm in Asthma

Jong-Uk Lee, Jeong-Seok Heo, Da-Jeong Bae, Hyun Ji Song, Choon-Sik Park, Jong-Sook Park

A527 Transition of Allergic and Nonallergic Rhinitis after 2 Years in Korean Children: Preliminary Study

Jae Hoon Cho, Ji Ho Choi

A528 Early Onset of Psoriasis Juvenile Idiopathic Arthritis

Budi Setiabudiawan, Fiska Febriana, Reni Ghrahani, Gartika Sapartini

A529 Clinical Features of Immediate Hypersensitivity to Histamine H2 Antagonists and Their Cross Reactivity

Chan-Sun Park, Young-Hee Nam, Mi Yeoung Kim, Gil-Soon Choi

A530 Detection of Galacto-Oligosaccharide Specific IgE *in Vitro*

Chiung-Hui Huang, Chiung-Hui Huang, Jian Yi Soh, Lynette Shek, Lynette Shek, Dianne J. Delsing, Bee Wah Lee, Bee Wah Lee, Si Hui Goh, Wen Chin Chiang, Wenyin Loh

A531 The Association Between Serum Vitamin D Levels and Allergic Diseases in Elementary Schoolchildren

Hea-Kyoung Yang, Ji Young Lee, Minji Kim, Kangmo Ahn, Jihyun Kim, Young-Min Kim, Hye-Young Kim, Yong Mean Park, Woo Kyung KIM, So-Yeon Lee

A532 Serum Levels Specific IgE to Toxic Shock Syndrome Toxin Type 1 in Eosinophilic Chronic Rhinosinusitis with Nasal Polyp in Korean

Jongin Jeong, Sang Duk Hong, Seung Kyu Chung, Hun-Jong Dhong, Hyo Yeol Kim Hyo Yeol Kim, Sujin KIM

A533 Impacts of Rhizosphere Cleaning Effects of Potted Indoor Plants on the Symptoms and Stress of Students with Allergic Rhinitis in Newly Built Schools

Yong-Won Lee, Hana Bak, Hye-Rim Son, Si-Eun Lee, Kwang-Jin Kim, Young-Wook Lim, Ho-Hyun Kim

A534 A Case of Multiple Food Allergies with Recurrent Anaphylaxis Successively Controlled By Omalizumab

Mi-Ae Kim, Man Yong Han, Young-Ho Jung, Hye Mi Jee, Seung Jin Lee, Kyung Suk Lee

A535 Bepotastine-Induced Urticaria, Cross-Reactive with Other Antihistamines

Jasmina Golez, Jaechun Lee, Eunkyoung Lee

A536 Role of SLC26a4 in Ozone - Induced Airway Reactivity and Inflammation

Da-Jeong Bae, Chang-Gi Min, Jong-Uk Lee, Jong-Sook Park, Hun Soo Chang, Choon-Sik Park, An-Soo Jang

A537 PAR2-Antagonist Suppresses Protease-Induced Allergic Inflammation Mediated By Degradation of Lung Epithelial Tight Junction and Generation of ROS

Young-Joon Kim, Bok Kyoung Jung, Seung-Hwa Lee, Mi-Jin Kang, Sekyoo Jeong, Eun Lee, Hyun-Ju Cho, Young-Ho Kim, Song-I Yang, Seo Hee Kim, Soo-Jong Hong

A538 Novel Anti-IL-4Ra Nanocarrier Approach for the Efficient Control of Lung Tissue Inflammation during Asthma

Rabih Halwani, Saleh Al Muhsen, Asma Sultana, Achraf Al-Faraj, Rosan Kanana, Sibtain Afzal, Roaa Al Kufaidi

A539 Clinical Factors for Improved Allergen Reactivities Induced By Subcutaneous Allergen Specific Immunotherapy with House Dust Mites during 1 Year Period

Hee-Kyoo Kim, Chul-Ho Oak, Gil-Soon Choi, Ye-Jin Moon, Eun-Kee Park

A540 Cytokine Gene Polymorphisms in Iranian Patients with Kidney Acute Rejection

Alireza Zarebidoki, Mina Abrari, Ali Akbar Amirzargar

A541 Phthalate Exposure and Obesity in Atopic Dermatitis of Korean Children and Adolescents

Ju-Hee Seo, Mina Ha, Soo-Jong Hong

A542 Which Drives Chronicity of Cough in Adults: Based on the Knhanes 2010-2012

Mingyu Kang, Byung-Ha Cho, Han-Ki Park, Han-Ki Park, Kyung-Mook Kim, Chang-Han Park, Heung Woo Park, Heung Woo Park, Yoon-Seok Chang, Yoon-Seok Chang, Yoon-Seok Chang, Sook-Hee Song, Mi-Kyeong Kim, Mi-Kyeong Kim, Sang-Heon Cho, Suk-Il Chang, Kyung-up Min, Kyung-up Min, Alyn Morice

A543 Elevated Airway CD45RO Memory Cells in Wheezing Children with Lower Respiratory Infection

Jungi Choi, Yusok Han, Jin-Sung Park, Eunmi Kwon, Chang-Keun Kim

A544 Quality of Life in Obese Children with or without Atopic Disease

Gartika Sapartini

A545 Relation of Human microRNA in Sputum of Asthma with Influenza A Virus Infection-Induced Exacerbation

Ji-Na Kim, Seungwoo Shin, Hun Soo Chang, Eun-Young Shim, Ji Ah Jun, Hyeonju Lee, Jong-Sook Park, Choon-Sik Park

A546 Aeropolinologic Monitoring and Distribution of Allergoallergens in Western Georgia

Revaz Sepiashvili, Darejan Khachapuridze, Sofio Gamkrelidze, Manana Chikhladze

A547 Extracorporeal Membrane Oxygenation As Emergency Treatment for Patients with Near-Fatal Status Asthmaticus

Seung-Eun Lee, Yun-Seong Kim, Doo-Soo Jeon, Woo-Hyun Cho, Hye-Ju Yeo, Seong-Hoon Yoon, Seung-Hyun Kim

A548 Relationship of S100calcium Binding Protein A9 with Neutophilic Inflammation in Murine Asthma Model

Taehyeong Lee, Hyun Ji Song, Choon-Sik Park, Ji Ah Jun, Jong-Sook Park

A549 Whole-Exome Sequencing of Aatopic Dermatitis in Korean Childhood

Dankyu Yoon, Yeon-Seop Kim, Woo-Sung Chang, Mi-Jin Kang, Soo-Jong Hong, Jeom-Kyu Lee, Eun-Jin Kim

A550 A Case of Generalized Molluscum Contagiosum in an Adult Patient with Severe Atopic Dermatitis

Minkee Park

A551 Discovery of Putative Macadamia Nut Allergens By Patient IgE Binding and a Label-Free Shotgun Proteomics Approach

Nanju Alice Lee, Johanna Rost, Sridevi Muralidharan, Dianne Campbell, Sam Mehr

A552 Anti-FcÎμri Antibody Inhibits Allergic March in Mice By Suppressing Th17 Pathway Via Suppression of FcÎμri-Mediated Mast Cells Activation

Seung-Hwa Lee, Seon-Joo Yoon, Ha-Jung Kim, Eun Lee, Song-I Yang, Young-Ho Jung, Ho-Sung Yu, Hee-Suk Kim, Yeon Hee Park, So-Yeon Lee, Jun-Sung Park

A553 Clinical Characteristics and the Associated Factors of ATG Hypersensitivity Reaction

Ha Kyeong Won, Min-Koo Kang, Sung Do Moon, Byung-Keun Kim, Ju-Young Kim, Sang-Heon Cho, Hye-Ryun Kang, Ji-Su Shim, Soo Jie Chung

A554 Reference Value and Utility of Total Serum Immunoglobulin E in Korean Schoolchildren

Jaehee Choi, Kangmo Ahn, Kwanghoon Kim, Jihyun Kim, Jiyoung Lee

A555 Two-Step Prescreening Skin Testing May be Useful for Reducing Immediate Hypersensitivity Reaction to Nonionic Contrast Media: Results of 7-Year Period in a Secondary Hospital

Bo Bae Park, In Young Nho, Chang-Han Park, Jang Min Kim, Suk-Il Chang

A556 Prevalence of Allergic Sensitization in Patients with Allergy Rhinitis; Gwangju, Jeonnam State Study

Sun Kyung Kim, Hyung Chae Yang, Kwang Il Nam

A557 Analysis of IgE Binding Components of Walnut in Korean Children Effect of Cooking Methods on the Allergenicity of Walnut Proteins

Jeongmin Lee, Jeongmin Lee, Sooyoung Lee, Kyunguk Jeong, Se-Ah Jeon

A558 Assessment of Autonomic Nervous Function in Subjects with Cholinergic Urticaria Associated with Acquired Idiopathic Generalized Anhidrosis

Midori Fujiwara, Shoko Shindo, Hiroyuki Murota, Mayuko Tahara, Aya Takahashi, Ichiro Katayama

A559 Interleukin 1 Beta in Sputum of Patients with Asthma: Relation with Airway Obstruction and Neutrophilc Inflammation

Jae Woo Jung, Hyun Ji Song, Taehyeong Lee, An-Soo Jang, Jong-Sook Park, Hun Soo Chang, Choon-Sik Park, Byoung Whui Choi

A560 Interleukin 8 in Sputum of Patients with Asthma: Relation with Neutrophilc Inflammation and Exacerbation

Min-Hye Kim, Da-Jeong Bae, Hyun Ji Song, Taehyeong Lee, Ji Ah Jun, Jong-Sook Park, An-Soo Jang, Hun Soo Chang, Young Joo Cho, Choon-Sik Park

A561 Prostaglandin E2 and Transforming Growth Factor-Î^2^ Play a Critical Role in Suppression of Allergic Airway Inflammation By Adipose-Derived Stem Cells

Sue Jean Mun

A562 Inhalation of Fine Particles Kill Alveolar Macrophages to Release IL-1alpha That Promote Inducible Bronchus-Associated Lymphoid Tissue (iBALT) Formation

Etsushi Kuroda, Koji Ozasa, Ken Ishii

A563 Association Between Smoking and Allergic Diseases in the Korean Adult General Population

Sunmi Kim, Gyeong-Hun Park

A564 Relationship of S100 Calcium Binding Protein A9 with Inflammasome Activation in Murine Asthma Model

Hyun Ji Song, Taehyeong Lee, Ji Ah Jun, Hun Soo Chang, Jong-Sook Park, Choon-Sik Park

A565 Cluster Analysis of Asthma Phenotypes to Predict Exacerbation in Korean Population

Mi-Ae Kim, Seungwoo Shin, Jong-Sook Park, Hun Soo Chang, You Sook Cho, Hae-Sim Park, Choon-Sik Park

A566 Effect of AG490 on the Expression of TH17 CELLS and Tregs in the MOUSE MODEL of Neutrophilic Asthma

Zhang Min

A567 Association Between the Clinical Characteristics and Disease Severity in Hospitalized Bronchiolitis Patients Younger Than Two Years Old

Seo Hee Yoon, In Suk Sol, Young a Park, Yoon Hee Kim, Min Jung Kim, Kyung Won Kim, Myung Hyun Sohn, Kyu-Earn Kim

A568 Comparison Between House Dust Mite and Aspergillosis Sensitization in Patients with High Level of Tige

Wu Shiquan

A569 The Prevalence of Metal Allergy in the Patients with Orthodontic Appliance

Yongwon Lee, Hana Bak

A570 Component Resolved Diagnosis and Single Nucleotide Polymorphism Analyses: Towards the Development of Specific Immunotherapy for Allergy

Maricar Wisco Ching, John Donnie Ramos

A571 Clinical Features of Anaphylaxis Caused By Peanut, Tree Nuts and Seeds in Children and Adolescents: Multi-Center Study with 126 Patients

Kyunguk Jeong, Sooyoung Lee, Kangmo Ahn, Myung Hyun Sohn, Kyung Won Kim, So-Yeon Lee, Tae Won Song, Youhoon Jeon, Jihyun Kim, Taek Ki Min, Kyu-Earn Kim, Bok-Yang Pyun, Hyeon-Jong Yang, Hae Ran Lee, Youngmin Ahn, Ji-Won Kwon, Dae Hyun Lim, Jeong Hee Kim, Dongin Suh, Hyung Young Ki

A572 A Report of Two Cases of Anaphylaxis Caused By Perilla Seed in Children

Kyunguk Jeong, Byeong Sub Park, Sooyoung Lee, Se-Ah Jeon, Kyu Jung Park

A573 Prenatal Fine Particulate Matter Affects Wheezing in Children with TLR4 Polymorphism: Cocoa Study

Song-I Yang, Eun Lee, Hyun-Ju Cho, Young-Ho Kim, Mi-Jin Kang, Yean Jung Choi, Kil Yong Choi, Youn Ho Shin, Kangmo Ahn, Kyung Won Kim, Byoung-Ju Kim, So-Yeon Lee, Eun-Jin K

A574 Intensified B Lymphocyte Depletion (IBLD) without Immunosuppressive Maintenance Treatment As a Rescue Therapy in Refractory Lupus Nephritis (LN): a 4-Year Observation.

Roccatello Dario

A575 Relationship Between Th17 Cells and Neutrophilic Airway Inflammation in Childhood Neutrophilic Asthma

Jing Liao

A576 Clinical Applications of Impulse Oscillometry in Asthma Management after Exacerbation in Preschool Children

Yong Feng, Yunxiao Shang

A577 Contact Allergy to Sodium Sulfite and Its Relationship to Facial Cosmetic Contact Dermatitis

Yongwon Lee, Hana Bak

A578 Effect of Exposure to Air Pollution on Asthma and Lung Function Development

Hyung Young Kim, Byoung-Ju Kim, Ji-Won Kwon, Ju-Hee Seo, Eun Lee, So-Yeon Lee, Song-I Yang, Young-Ho Jung, Hyo-Bin Kim, Ho-Jang Kwon, Hee Ju Park

A579 Role and Relational Mechanism of AG490 in Airwayinflammation in the Mouse Model of Neutrophilic Asthma

Zhang Min, Nong Guang-Min, Jiang Min

A580 Incidence of Adverse Reaction to Radioconstrast Media in a Single Tertiary Hospital

Gyu Young Hur, Eun Jung Sim, Sora Yoon, Juwhan Choi, Junga Kim, Jae Keom Sim, Jee Youn Oh

A581 Cow’s Milk Oral Food Challenge: Clinical and Laboratory Features in Korean Children

Kyunguk Jeong, Byeong Sub Park, Jeong-Min Lee, Sooyoung Lee, Eunjae Cheon, Youngjoo Na, Kyu Jung Park, Eunjoo Lee

A582 Validation of the Red Maple Trials Allergen Challenge Theatre for Ragweed Pollen Challenge

William Yang, Suzanne Kelly, Rob Perrins, Jimmy Yang

A583 Preliminary Evaluation of the Red Maple Trials Allergen Challenge Theatre for Grass Pollen

William Yang, Suzanne Kelly, Rob Perrins, Jacob Karsh, Jimmy Yang

A584 The Association Between Tobacco and the Risk of Asthma in Urban and Rural Children in San Francisco, Argentina

Hector Badellino, Alvaro Teijeiro, Mabel Cuello, Marilyn Urrutia Pereira, Gustavo Egues

A585 The Prevalence of Allergic Rhinitis in University Students in Manisa

Ayse Aktas

A586 Allergen Sensitization in Zimbabwean Children with Atopic Dermatitis

Jin-Kyong Chun, Hilda Angela Mujuru, Elopy N Sibanda

A587 Vitamin D Insufficiency in Asthmatic Patients

Andreea Ioana Popescu, Raluca Greblescu

A588 The Prevalence of Hypersensitivity Reactions Against Drugs Among University Students.

Suheyla Rahman, Ayse Aktas

A589 Sublingual Immunotherapy Among Problematic Patients, Suffering from Allergic Rhinitis.

Nataly Tataurshchikova

A590 A Novel Biomarker for Wheezing and Atopy in Early Infancy

Eishika Dissanayake, Yuzaburo Inoue, Naoki Shimojo, Taiji Nakano

A591 Prognostic Factors for Atopic Dermatitis in Spontaneously Born Babies from Low Socioeconomic Background

Conny Tanjung

A592 Higher IgE Antibody Levels Mediate Anti-Cancer Immunity in Transgenic KN1 Hyper-IgE Mice

Erika Jensen-Jarolim, Judit Fazekas, Josef Singer, Anna Lukschal, Reinhard Horvat, Gertrude Achatz-Straussberger, Gernot Achatz

A593 Fructooligosaccharides Intake during Pregnancy and Lactation Increases Gut *Bifidobacterium* and IL-27 in Breast Milk

Yuji Fujita, Shuji Ikegami, Yoshitaka Nakamura, Yuzaburo Inoue, Naoki Shimojo, Yoichi Kohno, Shuichi Suzuki, Naoko Ozawa, Takayuki Kubota, Ken Nonaka, Osamu Ohara, Kentaro Masuda

A594 Effect of Nintedanib on Asthma in Mouse Model

Chin Kook Rhee, Sook Young Lee, Hwa Young Lee, Hea Yon Lee, Ji Young Kang, Sei Won Kim, Soon Seog Kwon, Young Kyoon Kim

A595 Delayed Contrast Media Hypersensitivity after Coronary Angiography

Gun-Woo Kim, Ju-Young Kim, Sang-Heon Cho, Hye-Ryun Kang, Hyo-Soo Kim, Jung Gyu Han, Jin Lee, Ji Young Lee, Ji Young Go, So Jung Park

A596 Gene Expression Profiling in Patients with Chronic Idiopathic Urticaria Reveals Unique Gene Signature Distinct from Healthy Controls

Julie Kim-Chang, Cassandra Love, Patricia Lugar

A597 Failure to Recognize Lymphopenia in Newborn Leads to Undetectable Primary Immunodeficiency

Endah Citraresmi

A598 The Concordance Between Lung Function Test and Indonesian Version of Childhood Asthma Control Test (CACT)

Nastiti Kaswandani, Cynthia Utami, Mardjanis Said

A599 Synergistic Interaction Between Bronchiolitis and PM_10_ Is Modified By *IL-13* Polymorphism on Asthma Development: Replication from Cheer Study

Young-Ho Jung, Song-I Yang, Byoung-Ju Kim, Ji-Won Kwon, Hwan-Cheol Kim, Jong-Han Leem, Ju-Hee Seo, Hyung Young Kim, So-Yeon Lee, Ho-Jang Kwon, Hyo-Bin Kim, Hyun-Ju Cho

A600 The Transcription Factor Ehf Is Involved in TGF-b-Induced Suppression of Fceri and c-Kit Expression and Fceri-Mediated Activation in Mast Cells

Susumu Yamazaki, Nobuhiro Nakano, Asuka Honjoh, Eisuke Inage, Yosuke Baba, Yoshikazu Ohtsuka, Toshiaki Shimizu

A601 The Follow up of the Potential Immunosuppressant Effects of Marijuana (MJA)

Ishaq M, Sameera MI Khan, Imran Khan, Sabeen Khan

A602 Risk Factors of Allergen Sensitization at 3 Years: Results from the Gusto Study

Evelyn Xiu Ling Loo, Anne Goh, Oon Hoe Teoh, Yiong Huak Chan, Seang Mei Saw, Kenneth Kwek, Peter D Gluckman, Keith M Godfrey, Hugo Van Bever, Yap Seng Chong, Bee Wah Lee, Lynette Shek, Alison Joanne Lee

A603 IL-6 Blockade As a Steroid-Sparing Treatment for Rhupus Patients

Daniela Rossi

A604 Examination of Late Pulmonary Toxicity in Children Treated for Malignancies

Agnes Nemeth

A605 Technical Validation of the Repurposing of a Personal Particle Sampler to Determine House Dust Mite Exposure in the Ambient Air

Torsten Sehlinger, Karl-Christian Bergmann, Frank Goergen

A606 Zinc Deficiency in Children with Severe Atopic Dermatitis: More Common Than Generally Thought

Mohammad S. Ehlayel, Abdul Bari Bener

A607 Strong Association Between HLA-B*5801 Allele and Allopurinol – Induced Severe Cutaneous Adverse Reactions in Vietnamese

Hieu Chi Chu, Nga Thi Quynh Do, Dinh Van Nguyen, Ha Thi Thu Nguyen, Huong Thi Minh Le, Sheryl Van Nunen, Christopher Vidal, Suran Fernando

A608 Successful Rapid Desensitization to Glatiramer Acetate: Report of 2 Cases

Fotis Psarros, Ekaterini Syrigou, Ekaterini Politi, Spyridon Chrysoulakis

A609 Asthma Exacerbations Seasonal Variation in Two Perennial Phenotypes during Twenty Years (1995-2014): House Dust Mite Monosensitized and Non Atopic Patients

Dimitrios Vourdas, Konstantinos Petalas

A610 Occupational Allergy to Fungal Spores Among the Farmers of Paddy Fields in West Bengal, India: An Aeromycological and Immunological Approach

Mouli SAHA, Kashinath Bhattacharya

A611 Study of Efficacy of Sublingual Immunotherapy (SLIT) in Cases of Severe Persistent Allergic Rhinitis

Subir Jain

A612 Mesenchymal Stem Cells Suppress Lung Inflammation and Airway Remodeling in Chronic Asthma Rat Model Via PI3K/Akt Signaling Pathway Mesenchymal Stem Cells Suppress Lung Inflammation and Airway Remodeling in Chronic Asthma Rat Model Via PI3K/Akt Signaling

Xiaolian Song, Haiyan Lin

A613 Development of Allergen ELISA Kits for Dust Mites, Pollen, and Pet Dander

Kyohei Nishikawa, Takashi Shimada, Hiroshi Yasueda, Tadao Enomoto, Daisuke Aizawa, Takayoshi Kobayashi

A614 Yoga As a Lifestyle Modification to Improve the Quality of Life in Smokers with Allergic Rhinitis

Chellaa R

A615 Study of Incidence of Severe Persistent Allergic Rhinitis in Different Age Groups,Sex Prevalance and Type of Allergen” Aeroallergen or Food Allergen” Responsible for Severe Persistent Allergic Rhinitis in Central India

Subir Jain

A616 Causative Allergens in Cases of Severe Persistent Allergic Rhinitis in Central India

Subir Jain

A617 Atopic Dermatitis: A New Data on the Mechanisms of Chronic Pruritus

Marina Yudina

A618 The Efficacy and Safety of Peanut Oral Immunotherapy in High-Dose with Predicting Factors

Ishaq M, Sameera MI Khan, Imran Khan, Sabeen Khan

A619 Evaluation of Long-Term Prognosis and Topical Corticosteroid Usage after One Year of Proactive Treatment for Children with Moderate-to-Severe Atopic Dermatitis

Mayako Saito

A620 Allergy Symptoms in the First Two Months of Life

Nurul Iman Nilam Sari

A621 Factors Related to the Seasonal Variation of Allergic Rhinitis

Jae Young Kim, Jaechul Song, Inah Kim, Kyeong Joon Lee, Soo Jin Park, Soo Yong Roh

A622 Allergic Risk Survey in Lao Children at out-Patient Department, Children’s Hospital, Vientiane Capital, Lao PDR

Somxay Billamay

A623 Correlation Between Food Allergy, Aeroinhalant Allergy, Allergic Rhinitis, Atopic Dermatitis, and Acute Upper Respiratory Tract Infections and Levels of Severity of Asthma in Pediatric Medicine Department Saiful Anwar Hospital Indonesia

Muchammad Fahrul Udin

A624 Comparative Study of Pine, Oak, and Ginkgo Pollen Counts in Korea during Last Four Years

Mae Ja Han, Jae-Won Oh, Kyu Rang Kim, Baek-Jo Kim

A625 Effectiveness of Allergy-Test Directed Elimination Diets in Eosinophilic Esophagitis

Jorge A Mazza, Jason Kangeun Ko, David JT Huang

A626 Comparison of Cut-Off Values and Probability Curves for Egg Specific IgE in Diagnosis of Egg Allergy in Young Children

Kanae Furuya, Keigo Kainuma, Takahiro Ito, Mizuho Nagao, Takao Fujisawa, Junya Hirayama, Yu Kuwahara

A627 A Case of Persistent Atopic Dermatitis Associated with Parasitic Infection

Rosanna Qualizza, Cristoforo Incorvaia, Anna Maraschini

A628 Aeroallergenic Profile of Indoor Allergens and Their Clinical Relevance in Allergy and Asthma Patients in Saudi Arabia

Syed Mohammed Hasnain, Abdulrahman Al-Frayh

A629 Oral Exposure to the Amino Acid Glycine Inhibits the Onset of Allergic Disorders

Anita Hartog, Jacqueline Bastiaans, Reinilde Loonstra, Lieke Rutten, Lucien Harthoorn, Jeroen Van Bergenhenegouwen, Johan Garssen, Johan Garssen

A630 Cough As a Key Symptom in Asthma, Allergic Rhinitis, COPD and Rhinosinusitis and Its Impact in Korea

Kwang-Ha Yoo, Sang-Heon Cho, AG Ghoshal, Abdul Razak Bin Abdul Muttalif, Horng- Chyuan Lin, Sanguansak Thanaviratananich, Shalini Bagga, Rab Faruqi, Santwona Baidya, Colman Taylor, De Yun Wang, Hae-Ryun Ahn, Soon-Kwan Hong, Jong-Woong Kim, Gui-Hyun Nam, Mee-Ja Kim, Jae-Kyoung Park

A631 Cysteine Protease Allergen Def f 1 Induces Th2 Cytokines in Mouse Bone Marrow Derived Basophils Via ERK and JNK Dependent Pathways

Myung-Hee Yi, Kyoung Yong Jeong, Ju-Yeong Kim, Tai-Soon Yong

A632 Novel Multiple Allergy Testing Kit Using Parallel Lines Array (PLA) Technology

Bum Joon Kim, Hs Joo, Kj Lim, Jae-Hyun Lee, Jung-Won Park, Kh Yoon, DS Choi

A633 Quantitative Rapid Kit for Human Immunoglobulin

Hanseung Joo, Bum Joon Kim, Kj Lim, MJ KIM, DS Choi, Kh Yoon

A634 Total IgE Measurement By Protia Allergy-Q: Comparison Study with Immunocap

Bum Joon Kim, Hanseung Joo, Woo Sang Jung, Kj Lim, DS Choi

A635 Prevalence and Risk Factors of Asthma Among Korean Farmers

Ji-Hoon Lee, Soon-Chan Kwon, Soo-Jin Lee, Soo Yong Roh, Hogil Kim, Kyeong Joon Lee

A636 Dietary Intake and Perceived Immune Status in Young Dutch Women

Aurora Van De Loo, Amanda Fernstrand, Johan Garssen, Joris Verster

A637 The Effects of Antihistamine Drugs on on-Road Driving Performance

Aurora Van De Loo, Johan Garssen, Joris Verster

A638 Grass Is Guilty: A Case of Anaphylactic Shock and Asthmatic Status in the Same Time in an Individual

Jasmina Golez

A639 Cyclic Gamp-AMP(cGAMP) Induces Allergic Inflammation

Koji Ozasa, Etsushi Kuroda, Ken Ishii, Ken Ishii

A640 Role of Omalizumab in the Setting of Recalcitrate Dermatitis with Extremely Elevated IgE Levels

Muhammad Imran, Selina Gierer, John Martinez

A641 Follow-up Study on the Natural History of Prawn Allergy

Lydia Wong, Bee Wah Lee, Gaik Chin Yap, Genevieve Llanora, Bernard Thong, Lynette Shek

A642 Protein-Losing Dermopathy Impairing Growth in Children with Severe Atopic Dermatitis

Mohammad S. Ehlayel, Ashraf Soliman

A643 Airways Assessment of Aged Nursing Homes Residents

Pedro Martins, João Marques, Joana Gomes-Belo, Teresa Palmeiro, Iolanda Caires, Joana Belo, Maria Amália Botelho, Paula Leiria-Pinto, Nuno Neuparth

A644 Use of Skin Prick Test, Specific IgE to Shrimp and Rpen a1 to Determine Clinical Reaction to Shrimp in Area with High Prevalence of House Dust Mite Sensitization

Narissara Suratannon, Jaichat Mekaroonkamol, Jarungchit Ngamphaiboon, Piyawadee Lertchanaruengrith, Pantipa Chatchatee

A645 Identification of Specific IgE-Binding Proteins in Tree of Heaven (Ailanthus altissima) Pollen

Gholamali Kardar, Ahmad Majd, Youcef Shahali, Farrokh Ghahremaninejad, Zahra Pourpak, Fateme Mousavi

A646 Allergy Immunotherapy Well Tolerated in Children

Mahnaz Sadeghi-Shabestari

A647 Steinert (DM1) Patients Have IgG1 Deficiency and Should be Screened for Immune Deficiency

K. Van Bilsen, O. Manusama, W.a. Dik, M. Van Der Burg, V. H. J. Van Der Velden, V.a.S. H. Dalm, P. M. Van Hagen

A648 The Change of Serous Sige and Three Evaluation before and after Sublingual Immunotherapy with Dermatophagoides Farinae for Persistent Allergic Rhinitis

Yongping Liu

A649 Garlic Extracts Reduce Histamine-Induced Proliferation and Migration of Human Asthmatic Bronchial Smooth Muscle Cells

Yi Yeong Jeong

A650 A Case of Occupational Contact Dermatitis Caused By N-Acetylcysteine

Ji Hye Kim, Moon Gyeong Yoon, Young Min Ye, Yoo Seob Shin, Ga Young Ban, Hae-Sim Park, Hye Min Jung

A651 The Association Between Pollen Change and Asthma Attacks

Soo Yong Roh, Jaechul Song, Ji-Hoon Lee, Hogil Kim, Jae Young Kim, Kyeong Joon Lee

A652 Incidence of Emergency Department Visits and Hospitalizations for Asthma Exacerbations during the Lunar Month in Singapore

Lydia Wong, Mohana Rajakulendran, Haripriya Santhanam, Lynette Shek, Tow Keang Lim

A653 The Prevalence of Positive Reaction for Skin Prick Test in Korean Farmers and Its Occupational Risk Factors

Hogil Kim, Soo-Jin Lee, Ji-Hoon Lee, Soo Yong Roh, Soon-Chan Kwon

A654 Drug Allergy in Children: A Three-Years Experience at Dr. Kariadi Hospital Semarang Indonesia

Wistiani, Galuh H, Ani Wistiani

A655 The Identification of Morphology, Structure and Study of Seasonal Variation of Airborne Fraxinus Excelsior Pollen Grains in the Tehran

Gholam Ali Kardar, Maryam Sharifshoushtari, Ahmad Majd, Taher Nejadsattari, Zahra Pourpak, Mostafa Moin

A656 Sublingual Immunotherapy in Elderly Rhinitis Patients Sensitized to House Dust Mites

Ji Hye Kim, Daehong Seo, Young Min Ye, Hae-Sim Park, Jung-Won Park, Jae-Hyun Lee, Yoo Seob Shin

A657 Healthy Ageing Research Center (HARC) As a Platform for Multidisciplinary Approaches to Respiratory Research in the Elderly

Marek L. Kowalski, Aleksandra Wardzynska, Marcin Kurowski, Malgorzata / Ewa Pawelczyk, Adam Wysokinski, Iwona Kloszewska, Janina Grzegorczyk, Wojciech Piotrowski, Joanna Makowska

A658 Estimation of Cases of Work-Related Asthma Using Capture-Recapture Methods

Soon-Chan Kwon, Jaechul Song, Yong-Kyu Kim

A659 Primary School Students’ Parents Reported ISAAC Questionnaire in a Low Income Area of Ankara

Ilknur Bostanci, Zeynep Sengul Emeksiz, Aysegul Ertugrul, Serap Ozmen, Soner Sahin

A660 Usefulness of PC20 Adenosine Monophosphate in Diagnosis and Treatment in Bronchial Asthma

Sang-Ha Kim, Myoung Kyu Lee, Won Yeon Lee, Suk Joong Yong, Seok Jeong Lee, Ye-Ryung Jung

A661 S100 Calcium Binding Protein A9 in Sputum of Patients with Steroid Naive Asthma: Relation with Airway Obstruction and Nneutrophilc Inflammation

Myung Shin Kim, Jong-Sook Park, An-Soo Jang, Choon-Sik Park

A662 Risk Factor Asthma in Pediatric Pneumonia Patients

Diah Asri Wulandari, Cissy Kartasasmita

A663 Prevalence and Risk Factors of Childhood Asthma and Allergic Disease at Exposed Area By Emission of Cement Padang Factory

Finny Fitry Yani, Rizanda Machmud, Dhina Lydia Lestari

A664 Association Between Serum Level of 25-Hydroxyvitamin D with Atopic Dermatitis Occurrence and Severity in Children

Rusdi Rusdi, Yurmalina Yurmalina, Eryati Darwin

A665 Thiol-Disulfide Balance in Children with Atopic Dermatitis

Ilknur Bostanci, Gulin Karacan, Nazli Ercan, Asuman Colak, Murat Alisik, Gulay Basarir, Ozcan Erel

A666 Recurrent Mouth Ulsers Caused By Braces after Developing a Nickel Allergy in Children

Ilknur Bostanci, Yasemin Keskin

A667 Anaphylactic Reaction to Famotidine with Pheniramine Hypersensitivity

Ilkay Koca Kalkan

A668 Novel Transcriptomic and Immunoproteotomic Approaches in Identifying Cross-Reactive Allergens Between Crustacean and Molluscs

Andreas/Ludwig Lopata, Kyall Zenger, Roni Nugraha, Sandip Kamath

A669 Pollen Season and Climate Change in the Continental United States (CONUS)

Leonard Bielory, Panos Georgopoulos, Yong Zhang, Wheat Mi, Ting Cai

A670 Differences of Change in Der p IgG4 and CD4+CD25+FoxP3+ Treg Cells Between Sublingual and Subcutaneous Immunotherapy with House Dust Mite in Chinese Patients with Allergic Rhinitis

MO Xian, Jing Li, Mulin Feng

## A1 Pirfenidone inhibits TGF-b1-induced extracellular matrix production in nasal polyp-derived fibroblasts

### Heung-Man Lee^1^, Il-Ho Park^2^, Jae-Min Shin^3^

#### ^1^Korea University College of Medicine, Guro Hospital; ^2^College of Medicine, Korea University; ^3^Korea University Guro Hospital, South Korea

##### **Correspondence:** Jae-Min Shin – Korea University Guro Hospital, South Korea

**Purpose:** Pirfenidone has been shown to have anti-fibrotic and anti-inflammatory effects in the lungs. The purpose of this study was to evaluate the inhibitory effects of pirfenidone on transforming growth factor (TGF)-*β*1-induced myofibroblast differentiation and extracellular matrix accumulation. We also determined the molecular mechanisms of pirfenidone in nasal polyp-derived fibroblasts (NPDFs).

**Methods:** NPDFs were isolated from nasal polyps from eight patients who had chronic rhinosinusitis with nasal polyp. Pirfenidone was used to treat TGF-*β*1-induced NPDFs. Cytotoxicity was evaluated using a 3-(4,5- dimethylthiazol-2yl)-2,5-diphenyl-tetrazolium bromide assay. Fibroblast migration was evaluated with scratch assays. Expression levels of *α*-smooth muscle actin (SMA), fibronectin, and phosphorylated Smad2/3 were determined by western blot and/or reverse transcription-polymerase chain reaction and immunofluorescent staining. Total collagen production was analyzed with the Sircol collagen assay and contractile activity was measured by a collagen gel contraction assay.

**Results:** Pirfenidone (0 – 2 mg/ml) has no significant cytotoxic effects in TGF-*β*1-induced NPDFs. Migration of NPDFs was significantly inhibited by pirfenidone treatment. The expression levels of *α–*SMA and fibronectin were significantly reduced in pirfenidone-treated NPDFs. Collagen contraction and production were also significantly decreased by pirfenidone treatment. Finally, pirfenidone significantly inhibited phosphorylation of Smad2/3 pathway in TGF-*β*1-induced NPDFs.

**Conclusions:** Pirfenidone has an inhibitory effect on TGF-*β*1-induced migration, myofibroblast differentiation (*α-*SMA), extracellular matrix accumulation, and collagen contraction by blocking the phosphorylation of Smad2/3 pathways in NPDFs. Thus, pirfenidone may inhibit TGF-*β*1-induced extracellular matrix by regulating Smad2/3.

## A2 The efficacy of a 2-week course of oral steroid in the treatment of chronic spontaneous urticaria refractory to antihistamines

### Hyun-Sun Yoon, Gyeong Yul Park

#### Seoul National University Boramae Hospital, Seoul, South Korea

##### **Correspondence:** Hyun-Sun Yoon – Seoul National University Boramae Hospital, Seoul, South Korea

Even though antihistamines are the mainstay in the treatment of chronic spontaneous urticaria (CSU), some CSU patients have not responded to antihistamines. Many clinicians have accepted the efficacy of steroids in the treatment of CSU. There is, however, little evidence supporting steroid use in antihistamine-resistant CSU. The purpose of this study was to demonstrate the efficacy of, and suggest a regimen for, oral steroid in the treatment of CSU patients who were refractory to a high dosage of antihistamines. We conducted a retrospective chart review of all patients diagnosed with urticaria between Feburary 1, 2012, and December 31, 2014. A total of 98 patients with CSU were included. Of these, 16 patients (16.3%) were antihistamine-resistant and prescribed a 2-week course of steroid. Thirteen patients (81.2%) were successfully controlled with antihistamines only after stopping the first course. Second course of steroid induced remission additionally in two patients (12.5%). No adverse events and complications associated with oral steroid were observed over the study period. This study demonstrated the excellence of a 2-week course of oral corticosteroid in antihistamine-resistant CSU and propose standardized corticosteroid treatment regimen.

## A3 The altered distribution of follicular t helper cells may predict a more pronounced clinical course of primary sjögren’s syndrome

### Margit Zeher

#### Division of Clinical Immunology, Faculty of Medicine, University of Debrecen, Hungary, Debrecen, Hungary

Recent studies emphasized the important role of follicular T helper (T_FH_) cells, which contribute to B-cell differentiation, as well as antibody production. The aim of our study was to investigate the possible role of T_FH_ cells in the pathogenesis of primary Sjögren’s syndrome (pSS). In the first part of the study, we focused on the periphery by analyzing immune-competent cells and serological markers. We enrolled 50 pSS patients and 16 healthy controls in the study. Patients had elevated ratio of peripheral T_FH_ cells, however, when dividing patients into two groups defined by the presence of extraglandular manifestations (EGMs), only patients with EGMs differed from controls significantly. Moreover, T_FH_ cell percentages correlated positively with both activated T cell and Tr1 cell values, but T_FH_ cell percentages showed negative correlation with both IgM and IgG memory B cell proportions. Elevated T_FH_ percentages were observed in the anti-SSA/SSB positive patients. In the second part, we concentrated on the site of the inflammation, and determined the composition of lymphocyte infiltration in labial salivary gland (LSG) biopsies with special emphasis on T_FH_ cells. We selected tissue blocks obtained from 10 patients at the time of disease onset. LSGs were graded based on the organizational level of periductal lymphocytic infiltrates. T_FH_ cell markers occurred predominantly in more organized structures with higher focus scores. The co-expression of CD3 and Bcl-6 markers identified T_FH_ cells close to Bcl-6^+^B cells with the typical formation of germinal centers. Systemic features were developed later in the disease course only in patients with more structured infiltrates.

Our results indicate that the presence of T_FH_ cells in LSGs at the disease onset may predict a more pronounced clinical course of pSS. We expect that the further understanding of the regulation of T_FH_ cells will provide new potential therapeutic targets in the treatment of pSS patients with EGMs.

## A4 Betamethasone suppresses Th2 cell development induced by langerhans cell like dendritic cells

### Katsuhiko Matsui, Saki Tamai, Reiko Ikeda

#### Meiji Pharmaceutical University, Kiyose, Tokyo, Japan

##### **Correspondence:** Katsuhiko Matsui – Meiji Pharmaceutical University, Kiyose, Tokyo, Japan

***Objective*:** It is well known that Langerhans cells (LCs) work as the primary orchestrators in the polarization of immune responses towards T helper type 1 (Th1) or T helper type 2 (Th2) immune responses. In this study, we investigated the effects of tacrolimus and betamethasone on Th2 cell development by LCs.

***Methods:*** LC-like dendritic cells (LDCs) were generated from mouse bone marrow cells. Mice were primed with ovalbumin (OVA) peptide-pulsed LDCs, which had been treated with tacrolimus or betamethasone, via the hind footpad. After 5 days, the cytokine response in the draining popliteal lymph nodes was investigated by enzyme-linked immunosorbent assay. The expression of LDC surface molecules was investigated using reverse transcriptase polymerase chain reaction.

***Results:*** Administration of OVA peptide-pulsed LDCs, which had been treated with betamethasone, into the hind footpads of mice inhibited Th2 development as represented by down-regulation of interleukin (IL)-4 production as well as Th1 development as represented by down-regulation of interferon (IFN)-γ production. However, unlike the LDCs treated with betamethasone, those treated with tacrolimus did not inhibit Th1/Th2 cell development. The inhibition of Th1 cell and Th2 cell development by betamethasone was associated with suppression of CD40 and TIM-4 expression, respectively, in LDCs.

***Conclusion:*** These results suggest that topical application of betamethasone to lesional skin would be beneficial for control of atopic dermatitis by acting on epidermal LCs and inhibiting the development of Th2 cells.

## A5 An evaluation of variousallergens in cases of allergic bronchial asthma at lucknow and neighbouring districts by intradermal skintest

### Drsushil Suri, Dranu Suri

#### Suri Medical Foundation Hospital, Lucknow district, Uttar Pradesh State, India

##### **Correspondence:** Drsushil Suri – Suri Medical Foundation Hospital, Lucknow district, Uttar Pradesh State, India

The work of Kasliwas et al. (1959) and Shivpuri et al. (1971) have proved beyond doubt that there is a definite correlation between pollen peaks and symptomatology of the patients of respiratoty allergy. With these factors in mind the present study was conducted with the aims - to clinically evaluate the cases of bronchial asthma, to know the spectrum of allergens by skin testing in these cases(intradermal skin tests) and to find out seasonal and climatic variations in these cases.

The patients were prepared for intradermal skin test as per Shivpuri et al (1964). The intradermal skin tests were performed by means of a tuberculin syringe and 26 gauge (1”length) short bevelled needle. A negative control test was done in the similar manner with 0.01ml of buffer saline (the diluent of the antigen) and marked as “C’. A positive control test was done by Histamine phosphate solution(100 microgram/ml) and marked as “H”.Interpretation was done as per Shivpuri’s criteria (Shivpuri1962,1969). The 2 to 4 reactions were taken as “markedly positive”. This study showed that the prevalance of allergic manifestations were higher in first degree relatives (50%) than the second degree relatives (8%) of the patients. In this study the pollen antigens which gave “significant positive reactions” (and there clinical correlation with the patient’s history) in the descending order of significance were as Cassiasiamea, Adhantoda, Morus, Parthenium, Artemesia, Argemonemaxicana, Cannabisoccidentalis, Rumex, Asphodelous, Gynandropsis, Amaranthus, Cenchrusalbum, Ricinuscommunis, Crataeva nurvuala, Melia, Kigelia, Prosopsis, Pennisetum, Dodonaea, Azadirechta, Putranjiva rox-burghii. The extensive botanical and aero-biological surveys with clinical evaluation of a very largre number of cases of alleric bronchial asthma should be carried out in future in order to draw the exact polen calender of this region.

## A6 Evaluation ferqency of ADHD in childhood asthma

### Marzieh Heidarzadeh Arani

#### Shahid Beheshti University, Tehran, Iran

**Introduction**

Attention Deficit-Hyperactivity Disorder is one of the most common problems of children and adolescents and is a very common reason for visiting a pediatric psychologist or consultant. This disorder which has a profound effect on the lives of thousands of children and their families manifests in early childhood with symptoms of hyper activity, inattention and impulsivity. In studies conducted in western countries it has been pointed out that there is an association between ADHD symptoms and asthma in asthmatic children, and also the response to asthma treatment in children with ADHD is poor. This study is performed with the aim to evaluate the prevalence of ADHD in children referring to the clinic of Kashan Beheshti hospital.

**Material and Method**

In this case- control study 206 children referred to the pediatric clinic of Kashan Beheshti hospital in the second half of 1388 were evaluated. Spirometery or peak flow metery was used for the diagnosis of ADHD. Using the SPSS software version 19, chi- square test and Fisher’s exact test.

**Result**

It was found that the prevalence of ADHD in children with asthma is significant higher than control group and increases the risk about five fold more. It was also found that having ADHD does not have a significant correlation with the severity of asthma.

**Conclusion**

It was found that asthma is one of the risk factors for developing ADHD, but ADHD is not related to the severity of asthma morbidity.

**Keywords:** Attention Deficit-Hyperactivity Disorder, Asthma

## A7 Steven johnson syndrome caused by typhoid fever in a child

### Azwin Lubis, Anang Endaryanto

#### ^1^Airlangga University Teaching Hospital, Surabaya, Indonesia

##### **Correspondence:** Azwin Lubis – Airlangga University Teaching Hospital, Surabaya, Indonesia

**Background:** Steven-Johnson Syndrome (SJS) in children can caused by infection. Thypoid is one of bacterial etiologies.

**Case report:** A 7 years-old boy with main complaint of blistering all over his body, fever and weak condition. His lips were ulcerated, erythematous, swollen covered with pseudomembran and hemorrhagic crust. There were pruritic hyperpigmented plaques with papilovesicular eruptions at their center (bullous target lesions) covered almost of his whole body. There were also macula erythematous with vesicle and ulceration in his external genital and scrotum. He suffered from blistering since 3 days before admission. The lesion appeared first in the face and mouth, then spreads to the whole body. After that his mucosa of mouth and lips were ulcerated and he had difficulty in swallowing. He had been on medical treatment to a doctor before, but on the 4^th^ day of illness there were red blotches and vesicles appeared on his body, sore throat and oral thrush. So he admitted in hospital for 3 days, but his condition was getting worse and came to our hospital. The O widal test result was 1/320. The assessment were SJS and suspected Typhoid fever. Treatment were supportive, ceftriaxone and corticosteroid. This patient discharged in a good condition.

**Summary:** Thypoid is one of SJS bacterial etiologies in children. Diagnosis established after clinical examination, serological, epidemiologically-prevalent and probable infections in country area. Antibiotic is required for SJS caused by infections.

## A8 Chronic Bronchitis with Radio Contrast Media Hypersensitivity: A Case with Hypothesized GINA Step 1 Asthma

### Shinichiro Koga

#### Tokyo Metropolitan Police Hospital, Tokyo, Japan

**Background:** Nonimmediate reactions induced by radio contrast media (RCM) are an important health problem because nearly 50 % of patients with a suspected nonimmediate reactions to RCM are confirmed as allergic (Gomez E, et al. Curr Opin Clin Immunol 2013;**13**:345-53).

**Case Record:** A 60-year-old female was brought to emergency department with chief complaint of hemoptysis one day after transient dry cough. Past medical history included current smoking in 30 pack-per-years, acute hypersensitivity reaction with nicotinic α4β2 receptor partial agonist valenicline, epilepsy on phenytoin and phenobarbiturate, and pollinosis. Body temperature 98.5 F, blood oxygen saturation 95 % in room air; No crackle, stridor, nor wheeze was audible. Contrast chest computed tomography show atelectasis in left lower lobe, but not about carvenous lesion, pulmonary arteriovenous fistula, nor racemose hemangioma. Bronchoscopy detected thrombus in left lower lobe; *Mycobacterium avium*, *intracellulare*, and *tuberculosis* were negative in bronchoalveolar lavage fluid. Specific immunofluorescence in *M. tuberculosis* was also in negative. Serum test was thoroughly negative in SCC, CYFRA, ProGRP, NSE, IgG4, PR3- and MPO-ANCA, *Aspergillus*, (1,3→) βD-glucan, ANA, ds-DNA-IgG, anti-RNP, anti-sm, and anti-GBM antibodies. Clinical diagnosis of chronic bronchitis was made with findings described above. Pruritus exanthema in trunk one-and-a-half day after contrast media iomeprol infusion (Dona I, et al. J Investig Allergy Clin Imunol 2014;**24**:143-53) was disappeared several days after bepotastine besilate administration.

**Discussions:** Global Initiative of Asthma (GINA) step 1 was hypothesized because patients who were treated with GINA step 1 have reported to have over 3-fold-increased risk to develop acute hypersensitivity reaction as compared with patients who did not have asthma (Kobayashi D, et al. Chest 2012;**141**:1367-8).

**Conclusions:** Pharmacogenetics and/or transcriptomics should be helpful to clarify T-cell mediated mechanisms in development of RCM hypersensitivity reaction (Fernandez TD, et al. Allergy 2014;**69**:150-8).

## A9 The association between asthma and depression in Korean adult : An analysis of the fifth Korea national health and nutrition examination survey (2010-2012)

### Lee Ju Suk

#### Shinichiro Koga – Samsung Changwon Hospital, Seoul, South Korea

**Purpose:** Asthma is a one of the most common allergic disease and depression is an important comorbidity in asthma. However, it is little known about the different prevalence of depression in Korean adult asthmatics than normal adults. This study was performed to find association between asthma and depression and investigate the characteristics in Korean adult asthmatics with depression.

**Methods:** Data were acquired from 18,066 men and women, aged older than 19 years who participated in the Fifth Korea National Health and Nutrition Examination Surveys (KNHANES), which was conducted from 2010 to 2012. The presence of asthma was based on self-reported physician diagnosis of asthma in the Health Interview Surveys.

**Results:** The prevalence of asthma was 3.2 % and depression was 4.2 %. In univariated analysis, adults with asthma were older, single, unemployed, low educated, lower monthly family income, lower household members, obese (*p*<0.05) but sex, residence area and smoking status were not associated with the presence of asthma. The prevalence of hypertension and depression were higher in asthmatics (*p*<0.05), while diabetes mellitus was not associated with asthma. After adjustment for age, marital status, number of household members, monthly family income, body mass index, hypertension, we found unemployment, lower educated status, depression were associated with higher prevalence of asthma (*p*<0.01). Depression was associated with female, unemployment but not associated with lung function in asthmatics.

**Conclusion:** The present study showed that depression was an important risk factor of the prevalence of asthma in Korean adult. These results warrant future studies to explore the mechanisms responsible for the association between depression and asthma.

## A10 Management of allergic disease exacerbations in pregnancy

### Yasunobu Tsuzuki

#### Sapporo Tokushukai Hospital, Japan

**Introduction:** Allergic disease exacerbations can affect maternal and fetal conditions in pregnancy. Some of allergic disease exacerbations may trigger pregnancy complications, life-threatening events of the mother and fetus. Eventually, it predisposes both them to the risk of severe damages. We report 4 cases of maternal allergic disease exacerbations (food allergy(FA) and anaphylaxis, bronchial asthma(BA), atopic dermatitis(AD), allergic rhinitis(AR), atopic eruption of pregnancy(AEP)), their management is discussed.

**Case report:** Case1: a 31-year-old female in 32 weeks of gestation, with a medical history of BA in childhood, AD, AR, FA, was referred by an otolaryngologist because of persistent moderate-to-severe asthma symptoms, severe AR. We introduced long-acting β2-agonist plus inhaled corticosteroids, short term oral methylprednisolone and nasal corticosteroid. Case2: a 26-year-old female in 25 weeks of gestation, with a medical history of AD, but without a history of any FA or anaphylaxis, was brought to the emergency department because of dyspnea, generalized itchy rash, abdominal pain, and severe uterine contractions after having eaten dinner. She was diagnosed as anaphylaxis and threated abortion. She was immediately treated by epinephrine i.m injection 2 times and obsterician. We disclosed buckwheat allergy as the cause of anaphylaxis. Case3: a 30-year-old female in 27 weeks of gestation, with a medical history of AD, was referred by an obsterician because of her severe AD and AR. She had stopped the medication after her pregnancy, which developed severe AD and AR. We introduced local corticosteroids and nasal corticosteroid. Case4: a 34-year-old female in 31 weeks of gestation, with a medical history of AR and AD during her childhood, came to our hospital because of her severe itchy hives in her both arms and body. She was diagnosed as AEP and was introduced to use local corticosteroids. All mothers and their conditions improved immediately and the baibies of 4 cases were normally delivered without neurological deficiency or congenital malformations.

**Discussions:** Allergic disease exacerbations may be common events during pregnancy, that reduce the maternal QOL and can even develop the potential risks of pregnancy complications, life-threatening events of mother and fetus, severe fetal brain damages. To prevent these situations, we should promote the appropriate therapies and educations to allergic diseases, and encourage the mothers to keep allergic medications, not only during pregnancy but prepregnant period. More investigations toward the pharmacologic management in allergic diseases are necessary to keep pregnant women and newborns healthy.

## A11 Subcutaneous immunotherapy mouse model for atopic dermatitis

### Seo Hyeong Kim^1,2^, Jung U. Shin^1^, Ji Yeon Noh^1^, Shan Jin^1^, Shan Jin^2^, Hemin Lee^1^, Jungsoo Lee^1^, Chang Ook Park^1^, Kwang Hoon Lee^2^, Kwang Hoon Lee^1^

#### ^1^Department of Dermatology, Yonsei University College of Medicine; ^2^Brain Korea 21 PLUS Project for Medical Science, Yonsei University College of Medicine, South Korea

##### **Correspondence:** Seo Hyeong Kim – Department of Dermatology, Yonsei University College of Medicine

Subcutaneous immunotherapy (SCIT) is a clinically effective treatment in atopic dermatitis. However, there was no mouse model to understand the mechanism of house dust mite immunotherapy in atopic dermatitis. The aim of this study was to establish a mouse model of SCIT mouse model of house dust mite induced atopic dermatitis. Female NC/Nga mice were treated with *Dermatophagoides farinae (D.farinae)* body extract ointment for 4 weeks to induce atopic dermatitis like skin lesion. Then we separated the mice into 2 groups, control group and immunotherapy (IT) group. Both groups were continuously treated with *D.farinae* body extract ointment for 4 weeks, however, only immunotherapy (IT) group was treated with 8 injections of *D.farinae* (100 ug subcutaneouse) twice a week along with *D.farinae* body extract ointment treatment. Subsequently, clinical severity of skin lesion, histological features, total IgE, IL-4 and IL-10 were measured. We observed that AD-like skin lesions of IT group were milder than control group. In histological examination, epidermis was thinner in IT group than control group. The level of total IgE was decreased in IT group than control group at 3 weeks from the beginning of immunotherapy, while the level of IgG4 was remarkably increased in IT group than control group from the beginning of immunotherapy. Increased level of IL-10 in IT group was observed at 2 weeks from the beginning of immunotherapy than control group. However, there was no significant difference of IL-4 between IT group and control group. In the present study, we have established a house dust mite SCIT mouse model and demonstrated that house dust mite SCIT might improve atopic dermatitis-like skin lesion by decreasing total IgE and increasing the IgG4 and Il-10. Using this model, it will be possible to find novel way to potentiate the effects of allergen immunotherapy.

## A12 Atopic disease and/or atopy are risk factors for local anesthetic allergy in patients with history of hypersensitivity reactions to drugs?

### Fatma Merve Tepetam

#### Sureyyapasa Chest Diseases and Chest Surgery Training Hospital, Turkey

**Background:** There is some concern among physicians that a positive history of adverse reactions to drugs and/or atopic disease remains a risk factor for developing a hypersensitivity reaction to local anesthetics (LA).

**Objective:** In the present study we aimed allergy testing to LA is justified in patients with a positive history of hypersensitivity reactions to drugs additionally by atopy and atopic diseases.

**Methods:** The study was conducted among patients admitted to our allergy clinic who gave a reliable history of urticaria/angioedema, nasoocular symptoms and bronchospasm after ingesting drugs. A standard questionnaire regarding demographic data, history of atopic disease (asthma, allergic rhinitis, atopic dermatitis, urticaria) was filled out, total IgE and eosinophil count were assesed for each patient. Skin prick tests (SPT), intradermal test (IDT) and subcutaneus incremental challenge test (ICT) were perfomed step by step with adrenaline free lidokain and prilokain.

**Results:** 239 patients (63men,176 women) with history of drug allergy were admitted. Asthma prevalence, atopy rate and mean total IgE levels are found higher in the study. When we look at the results of skin tests and provocations with LA; while positive results of SPT werent found, 3 of IDT and 4 of ICT were positive. Of the all 4 patients that reacted to LA, had rhinosinusitis and a positive SPT reaction with aeroallergens, 1 patient had also food allergy, 2 patient had asthma. Although eosinofil counts were normal, 2 patient had high level of total Ig E.

**Conclusion:** There is no increased risk requiring performing skin and provocation test for LA in the patients with a history of hypersensitivity reaction to drugs alone but additionally by presence of atopy, atopic disease and multidrug allergy history can increase the risk of LA allergy, the amount of evidence is scarce.

## A13 Food hypersensitivity in patients with atopic dermatitis in Korea

### Chun Wook Park, Jee Hee Son, Soo Ick Cho, Yong Se Cho, Yun Sun Byun, Yoon Seok Yang, Bo Young Chung, Hye One Kim, Hee Jin Cho

#### Kangnam Sacred Heart Hospital, South Korea

##### **Correspondence:** Chun Wook Park – Kangnam Sacred Heart Hospital, South Korea

**Background:** It is well known that atopic dermatitis (AD) is related to food hypersensitivity, although its prevalence varies among several studies.

**Objective:** To examine the prevalence and status of food hypersensitivity among AD patients in Korea.

**Methods:** The history of food hypersensitivity was collected by interviews. We took blood samples to examine food-specific immunoglobulin E (IgE) levels. Based on patients’ histories and serum IgE levels, open oral food challenge (OFC) testing were performed.

**Results:** 49 (37.7%) of the 130 childhood AD patients and 33 (25.4%) of 130 adult AD patients had histories of food hypersensitivity. The most common suspicious foods in childhood AD were egg, pork and cow milk. In adult AD patients, the most common suspicious foods were instant food, wheat, ramen, beef, pork, chocolate. According to open OFC testing, nine childhood patients showed positive responses. The most common offending food were egg and milk (n=3 each), followed by pork, beef, peanut (n=1 each). In adult AD patients, only one patient showed positive response to pork.

**Conclusion:** The overall prevalence of food hypersensitivity in childhood AD patients was 6.9%, and 0.8% in adult AD patients as assessed by open OFC testing. The number of patients with actual food hypersensitivity was lower than the number of patients reporting a history of food hypersensitivity. Therefore, dietary restriction must be conducted after confirming food hypersensitivity by medical professionals.

## A14 Anaphylaxis caused by an ant (Brachyponera chinensis) in Japan

### Yoshinori Katada^1^, Toshio Tanaka^2^, Akihiko Nakabayashi^1^, Koji Nishida^1^, Kenichi Aoyagi^1^, Yuki Tsukamoto^1^, Kazushi Konma^1^, Motoo Matsuura^1^, Jung-Won Park^3^, Yoshinori Harada^4^, Kyoung Yong Jeong^3^, Akiko Yura^4^, Maiko Yoshimura^4^

#### ^1^Sakai City Medical Center; ^2^Osaka University; ^3^Yonsei University College of Medicine; ^4^Osaka-Minami Medical Center

##### **Correspondence:** Yoshinori Katada – Sakai City Medical Center, Japan

**A) Background: Index patient**

Ant allergy is rare comparing bee allergy. Anaphylaxis caused by Brachyponera chinensis is sporadically reported in Far East nations. We experienced such a case.

An otherwise healthy 63-year-old woman who developed an anaphylactic reaction in a public open-air bath, some five minutes after an ant stung her hip. She fell down there and experiencing diarrhea, dyspnea and urticaria, and transferred to a hospital, where she recovered with catecholamines and antihistamines. Later, an ant named Brachyponera chinensis (Emery, 1895), which is often referred as Pachycondyla chinensis, was caught at the same place. Specific IgE against its venom protein was detected in her serum. This is the first case with specific IgE against ant venom proved in vitro in Japan.

**B) Methods**

We explored medline and a Japanese database seeking literature written English or Japanese for anaphylactic cases by ants in Japan and investigated its nature.

**C) Results**

Including present case, 9 cases (age 20~63, male 5, female 4) with anaphylaxis by ant siting have been reported in Japan since 1987. The numbers of ant stings just before the onset of anaphylaxis were 1 to 3. The intervals between ant stings and onset of anaphylaxis in most cases were less than 15 minutes, except 2 cases without description and 1 case, in which the onset was two hours after the ant sting. Symptoms include wheal and urticaria, diarrhea, dyspnea, and hypotension. As for diagnosis, the causal relationship between ant stings and anaphylaxis was established mainly based on their temporal order. The patients themselves or doctors in charge caught the ants on the same day or later, enabling determination of the ant species. In most cases, the ants were identified as B. chinensis. The proof of IgE antibody against ant protein was done by skin prick test only in 2 cases. Our present case is the first Japanese case in which IgE antibody against B. chinensis protein was directly proved. Epinephrine was used in 5 cases, while only anti-histamine and glucocorticoid were used in other cases with good prognosis. All patients had prior history of ant stings. Most of them had more than one ant sting before. The longest interval between the last prior sting and the trigger stings were 17 months. It is notable that 2 patients including our case had prior stings about a year before and a month before, suggesting that the second stings might have booster effects. Cross-reaction with bee venom was seen in 2 cases out of 5 cases mentioned. In one case, IgE antibody against paper wasp was positive without an obvious former sting by it. The other case had a history of anaphylaxis by a wasp sting.

**D) Conclusions**

Ant allergy is rare. Its nature is similar to bee allergy.

Lee EK, Jeong KY, Lyu DP, Lee YW, Sohn JH, Lim KJ, Hong CS, Park JW.

Characterization of the major allergens of Pachycondyla chinensis in ant sting anaphylaxis patients. Clin Exp Allergy. 2009;39:602-7.

## A15 Anti-allergic effect of anti-IL-33 by suppression of immunoglobulin light chain and inducible nitric oxide synthase

### Tae-Suk Kyung^1^, Young Hyo Kim^1^, Chang-Shin Park^2^, Tae Young Jang^1^, Min-Jeong Heo^1^, Ah-Yeoun Jung^1^, Seung-Chan Yang^1^

#### ^1^Inha University, College of Medicine, South Korea; ^2^Inha University Hospital

##### **Correspondence:** Tae-Suk Kyung – Inha University, College of Medicine, South Korea

**Objective:** IL-33 is involved in several steps in allergic cascade. We aimed to find novel genes, which are significantly induced in allergic individuals and significantly down-regulated by anti-IL-33 treatment.

**Methods:** Thirty-six mice were allocated into Group A (intraperitoneally sensitized and intranasally challenged to saline), Group B (sensitized and challenged to ovalbumin), Group C (null treatment with intraperitoneal saline) and Group D (anti-IL-33 intraperitoneal injection). We checked the number of nose-scratching behaviors in 10 minutes, serum ovalbumin-specific IgE, titers of cytokines (IL-1, IL-4, IL-5, IL-10, IL-13) in bronchoalveolar lavage fluid. Using one whole lung tissue from each mouse, we performed microarray analysis and real-time PCR.

**Results:** Group D showed significantly reduced number of nose-scratching and lower serum ovalbumin-specific IgE. All cytokines in bronchoalveolar lavage fluid were significantly decreased after anti-IL-33 treatment. In microarray analysis, Group B (Immunoglobulin free light chain (*IgFLC*): 89.1 times, Nitric Oxide Synthase 2 (*NOS2*): 11.5 times) and Group C (*IgFLC*: 141.6 times, *NOS2*: 11.7 times) had significantly increased expression of *IgFLC* and *NOS2* gene compared to Group A. After anti-IL-33 treatment, Group D showed significantly decreased expression (*IgFLC*: 49.3 times, *NOS2*: 6.5 times). In real-time PCR, Group B (*IgFLC*: 10.4 times, *NOS2*: 3.8 times) and Group C (*IgFLC*: 29 times, *NOS2*: 4.5 times) had significantly increased expression of these genes. After treatment, Group D showed significantly decreased expression (*IgFLC*: 5.0 times, *NOS2*: 2.5 times).

**Conclusion:** Anti-allergic effect of anti-IL-33 could be explained by suppression of *IgFLC* and *NOS2* in a murine model of allergic rhinitis and asthma.

**Keywords:** Asthma: Animal Models; Immunoglobulins

## A16 Food hypersensitivity in patients with chronic urticaria in Korea

### Hye One Kim^1^, Yong Se Cho^1^, Yun Sun Byun^1^, Yoon Seok Yang^1^, Bo Young Chung^1^, Jee Hee Son^1^, Chun Wook Park^1^, Hee Jin Cho^2^

#### ^1^Kangnam Sacred Heart Hospital; ^2^Chuncheon Sacred Heart Hospital

##### **Correspondence:** Hye One Kim – Kangnam Sacred Heart Hospital, South Korea

**Background:** The etiology of chronic urticaria (CU) remains unknown in most patients. Possible causes in some cases include food, but the role of hypersensitivity to food antigens in patients with CU remains controversial.

**Objectives:** The aim of this study was to evaluate the association between food hypersensitivity and CU in 350 Korean participants.

**Methods:** Patients with CU were assessed for a previous history of food hypersensitivity that caused symptoms of CU. Blood samples were taken from the patients to measure food allergen-specific IgE. Based on history and laboratory results, open oral food challenge (OFC) tests were performed.

**Results:** Of 350 participants, 46 (13.1%) claimed to have experienced previous food hypersensitivity. Pork (n=16) were the main foods mentioned, followed by beef (n=7), shrimp (n=6) and mackerel (n=6). We found that 73 participants (20.8%) had elevated levels of food-specific IgE, with pork (n=30), wheat (n=25), and beef (n=23) being the most common. However, when the open OFC tests were conducted in 102 participants with self-reported food hypersensitivity or raised levels of food-specific IgE, only four participants showed a positive reaction to pork.

**Conclusions:** Although some participants claimed to have a history of CU related to food intake, when an open OFC test was conducted, few of them had positive results. We therefore conclude that food allergy is an uncommon cause of chronic CU.

## A17 Dose optimizing study of a depigmented polymerized allergen extract of phleum pollen by means of conjunctival provocation test (CPT)

### Oliver Pfaar^1^, Angelika Sager^2^

#### ^1^Center for Rhinology and Allergology Wiesbaden; ^2^Leti Pharma Gmbh, Germany

##### **Correspondence:** Angelika Sager – Leti Pharma Gmbh, Germany

**Background**: Clinical efficacy of a depigmented polymerized Phleum pollen extract has been shown in one large phase III studies with 1000 DPP/ml. To date dose-response studies are required to show optimal efficacy of an allergen dose. The conjunctival provocation test (CPT) is an outcome parameter accepted from the regulatory authorities (EMA).

**Method**: 308 (ITT) patients with confirmed rhinitis and/or rhinoconjunctivitis were treated in a double-blind study with 4 doses of 100 DPP/ml, 1000 DPP/ml, 5000 DPP/ml and 10.000 DPP/ml allergen extract over 22 weeks in Germany, Poland, Spain and Czech Republic. A 1-day build-up phase applying 0.1 ml and 2 times 0.2 ml extract was followed by a maintenance period applying 0.5 ml in 3-4 weeks intervals. Before treatment a CPT was performed with increasing doses up to 3 HEP/ml of native Phleum extract, after treatment the CPT was repeated with doses up to 30 HEP/ml. The primary endpoint parameter was the percentage of patients with an increase of allergen extract to provoke a positive CPT after Allergen Immunotherapy (AIT). Secondary parameters were specific IgE, IgG1 and IgG4, Vitamin D baseline level as well as safety.

The primary endpoint parameter was investigated using a hierarchic test procedure comparing the highest dose against the lowest, if statistically significant testing the next lower dose against the lowest until the difference was no longer significant.

**Results**: The responder rates in the 100 DPP/mL and the 1,000 DPP/mL groups were 72.9% and 72.3%, respectively (ITT), in the 5,000 DPP/mL (75.3%) and the 10,000 DPP/mL groups (77.4%). The respective differences to the 100 DPP/mL group were -4.5% for the 10,000 DPP/mL group, -2.5% for the 5,000 DPP/mL group, and 0.6% for the 1,000 DPP/mL group (ITT). No statistically significant difference was found. For the PP set, similar results were observed. Vitamin D baseline results were low in all groups, but had no influence on the results. Specific IgEs remained stable in all groups whereas specific IgG1 and IgG4 showed dose-dependent increases. Systemic reactions occurred in 12.9% (100 DPP/ml), 10.8% (1000 DPP/ml), 26.4% (5000 DPP/ml) and 33.3% (10.000 DPP/ml) of patients. 79.4% of SR were Grade 1, 8.7% grade 2 and 1 grade 3 reaction occurred in the 100 DPP/ml group.

**Conclusion:** We determined increased allergen amounts to obtain a positive CPT after AIT from 1000 -10.000 DPP/ml depigmented polymerized Phleum pollen extract. Since the CPT results did not discriminate the different doses the dose-finding study will be repeated with a different assessment method.

## A18 Correlation of cutaneous sensitivity and cytokine response in children with asthma

### Amit Agarwal^1^, Meenu Singh^1^, Bishnupda Chatterjee^2^, Anil Chauhan^1^

#### ^1^Postgraduate Institute of Medical Education and Research, India; ^2^West Bengal Technical University

##### **Correspondence:** Amit Agarwal – Postgraduate Institute of Medical Education and Research, India

**Background**: Food allergens play a pivotal role in the manifestation of atopic disorders like asthma, rhinitis, and eczema in children. Th17 cells play a key role in the regulation of inflammatory diseases, and there are few studies based on the role of Th17 in food allergic children.

**Methods**: Fifty children with respiratory allergy and history of worsening symptoms by food allergy (Patient group) and 20 non-atopic healthy children (Control group) were enrolled in the study. Food allergy was assessed through Skin prick test (SPT) with various food allergens and total IgE levels was determined by sandwich ELISA. Th1, Th2, and Th17 dependent cytokines were determined by flow cytometric bead array.

**Results**: In the study group, all the 50 children were found to be positive for cutaneous hypersensitivity against various food allergens (Milk and Orange=7, Egg=6, Banana, Fish and Groundnut=10, Mango=4, Wheat=9 and Rice=17). The average level of total IgE in the study group was 316.8±189.8 IU/mL. There was a significant positive correlation of total IgE with IL-17(r=0.796; p<0.0001), IL-13 (r= 0.383; p=0.01) and IL-4 (r=0.263; p=0.043) level, and negative correlation with IFN-γ (r= -0.5823; p<0.0001) and IL-10 (r= -0.4474; p<0.001) level. A negative correlation was noted between the duration of breast feeding and total IgE level (r=-0.31, p=0.03).

**Conclusions**: The study demonstrates positive correlation between total IgE, Th2 cytokines and Th17 cytokines. It also demonstrated that duration of breast feeding is significantly correlated with Total IgE.

## A19 Colabomycin E, a Streptomycete-Derived Secondary Metabolite, Inhibits Proinflammatory Cytokines in Human Monocytes/Macrophages

### Ilja Striz^1^, Eva Cecrdlova^1^, Katerina Petrickova^2^, Libor Kolesar^1^, Alena Sekerkova^1^, Veronika Svachova^1^, Miroslav Petricek^2^

#### ^1^Institute for Clinical and Experimental Medicine, Czech Republic; ^2^Institute of Microbiology, Academy of Sciences of the Czech Republic

##### **Correspondence:** Ilja Striz – Institute for Clinical and Experimental Medicine, Czech Republic

Polyketide antibiotics including macrolides are known to affect signalling pathways of transcription factors and regulate a number of genes involved in their potential anti-inflammatory effects but these properties cannot be used in clinical settings due to a risk of bacterial resistance. The aim of our study was to assess the effect of colabomycin E, a new streptomycete-derived secondary metabolite *, on the mRNA expression and release of proinflammatory cytokines and chemokines from THP-1 monocyte/macrophage cell line and peripheral blood monocytes.

Colabomycin E was isolated and purified from the natural producer Streptomyces aureus SOK1/5-04 by extraction of post-fermentation medias and mycelia with organic solvents and multiple subsequent liquid chromatography purification steps and crystallization. Monocytes/macrophages were cultured in RPMI1640 with 10% fetal calf serum and then stimulated with TNF-α (20ng/ml) under serum free conditions in the presence or absence of colabomycin E. The total mRNA was extracted from cultured monocytes/macrophages with RNAeasy Plus Mini Kit (Qiagen) and quantitative RT-PCR (SABiosciences) was used for the evaluation of 84 different gene expressions in TNF alpha and colabomycin E stimulated cultures and compared to unstimulated cells. The concentrations of cytokines and chemokines in culture supernatants of monocytes and THP-1 cells were measured by ELISA or Luminex.

In THP-1 cells, Colabomycin E inhibited TNF alpha induced mRNA expression of several proinflammatory genes including IL-1β, IL-6, TLR8, and MyD88. The effect was evident after 4h and 8h cultures and in some of the genes persisted for 24h. Furthermore, colabomycin E downregulated TNF alpha induced IL-1β, IL-6 and chemokine CXCL8/IL-8 release in a dose dependent manner from 0.25μM concentration. The secretion of IL-18 from THP-1 cells was only slightly upregulated by TNF alpha and not affected by colabomycin E. Our data suggest that colabomycin E is a potent transcription inhibitor of proinflammatory cytokines in human mononuclear phagocytes.

Supported by IGA MZCR grant NT/13012-4 and by MH CZ - DRO („Institute for Clinical and Experimental Medicine – IKEM, IN 00023001“).

**Reference**

*Petrickova K et al. ChemBioChem 15:1334-45, 2014

## A20 Intravenous immunoglobluin treatment in a child with resistant atopic dermatitis: A brief review on this therapeutic regimen

### Hyuck Hoon Kwon, Kyu Han Kim

#### Seoul National University Hospital, South Korea

##### **Correspondence:** Hyuck Hoon Kwon – Seoul National University Hospital, South Korea

Atopic dermatitis (AD) is one of the most common dermatologic diseases, affecting approximately 20% of children living in industrialized countries. Moreover, approximately 70% of cases of AD affect children less than 5 years of age. Most cases of AD are effectively treated with topical steroids, topical calcineurin inhibitors and intermittent use of immunosuppressive agents, including oral corticosteroids and cyclosporine. However, for recalcitrant AD, continuous use of systemic immunosuppressive agents is limited by severe adverse effects, especially for children. For this subgroup of patients, a few studies testing systemic immunomodulatory drugs have been reported, and high-dose intravenous immunoglobulin (IVIG) therapy has been also intermittently reported to be effective without strong evidences. Herein we report a case of intractable atopic dermatitis in a 5-year-old girl who had a significant clinical improvement after receiving 3 cycles of IVIG treatment (2 g/kg) without notable side effects. Since the first infusion of IVIG, the patient’s skin lesions improved steadily and the improvement persisted until the 8-month follow-up. The EASI score decreased remarkably, while the immunologic parameters did not correlate with clinical improvement. IVIG monotherapy is considered especially useful for children with severe AD since its immunoregulatory function is more profound in the relatively immature immune system of children. The mechanisms of action of immunoglobulin may include cytolysis of target cells through complement or antibody-dependent cell-mediated cytotoxicity, induction of apoptosis of target cells, blockade of co-stimulatory molecules, and neutralization of pathogenic antibodies and soluble factors such as cytokines and their receptors, which ultimately lead to amelioration of the inflammatory process. This case suggests that IVIG therapy can be quite effective and safe for children with resistant atopic dermatitis.

## A21 Wheat allergy is difficult to diagnose then other food allergens

### Suman Kumar

#### Ent and Allergy Centre(INDIA), India

**Background**

Wheat allergy is common in children and adults.Skin prick tests and wheat specific IgE tests has low specific value for wheat then other allergens.

The challenge tests are to be done to confirm Wheat allergy.It has been noticed that in patients of Wheat allergies the reactions may occur late .In normal food allergen challenges maximum number of reactions are found within one hour of food challenge and others in second hour. In wheat allergen challenge less number of reactions occur in first hour followed by some in second hours and rest occur after 2 hours of test.

**Objective**

To see wheather wheat challenge tests have delayed responses in patients of wheat allergy.

**Methods**

Database of patients of Wheat allergy attending ENT AND ALLERGY CENTRE(INDIA)PANCHKULA. Retrospective study of wheat allergy patients for last 3 years.

**Observations**

68 patients of suspected wheat allergy were put on Wheat challenge tesrts.

28 were positive, 2 were still not conclusive and 38 were negative.

In wheat allergy challenge tests 25% patients showed reactions in 1^st^ hour.

55% patients reacted in 2^nd^ hour and 20% reactions occurred after 2 hours.

**Conclusion**

In wheat allergy patients we need to know that reactions can occur even after 2 hours of challenge in comparison with other reallergens in which most reactions occur in 1 st hour of challenge.

## A22 The effects of spirulina (Arthrospira platensis) dietary supplement as an adjunct therapy for children aged 7 to 14 years old with asthma: A randomized - double blind placebo controlled clinical trial

### Lou Ver Leigh Arciaga Manzon^1^, Pilar Agnes Gonzalez Andaya^2^

#### ^1^University of Santo Tomas Hospital, Philippines; ^2^University of Santo Tomas Faculty of Medicine, Philippines

##### **Correspondence:** Lou Ver Leigh Arciaga Manzon – University of Santo Tomas Hospital, Philippines

**A) Background:** The anti-inflammatory effect of Spirulina has been demonstrated to inhibit histamine release from mast cell-mediated allergic reactions. Studies have documented the anti-inflammatory and immunomodulatory properties of supplementation as an adjunct therapy for asthma.

**B) Methods**: This is a randomized, double-blind, placebo-controlled study wherein children 7 to 14 years old diagnosed with mild to moderate persistent asthma were randomly assigned to receive either Spirulina (1,000 mg to 2,000 mg daily) or placebo for three months. Asthma Control Test (ACT) and Composite Asthma Severity Index (CASI) were used for patient report-based measures. Forced expiratory volume at 1 second (FEV1), forced vital capacity (FVC), FEV1/FVC and peak expiratory flow rate (PEFR) were determined through spirometry. Post-supplementation assessment for three months was done.

**C) Results:** A total of 39 patients (Spirulina = 20, placebo = 19) were enrolled in this trial. During the supplementation phase, both the Spirulina and placebo groups showed significant improvement in ACT scores (Spirulina, *P* < 0.0001; placebo, *P* = 0.19) compared to baseline. There was no significant change in CASI scores in both groups. However, during post-supplementation phase, the Spirulina group showed significantly sustained improvement on both the ACT (*P* < 0.0001) and CASI scores (*P* < 0.0001) compared to placebo. The FEV1 (*P* = 0.014), FVC (*P* = 0.008), and PEFR (*P* = 0.0001) of the Spirulina group significantly improved by the end of supplementation. Overall, significant intergroup differences revealed only in FEV1(*P* = 0.0002) and PEFR (*P*< 0.0001).

**D) Conclusion:** Daily supplementation with Spirulina significantly improved asthma control, FEV1 and PEFR compared to placebo.

## A23 The study about cause and clinicopathological findings of injection induced dermatitis

### Bark-Lynn Lew, Youngjun Oh, Dongwoo Suh, Woo-Young Sim

#### Kyung Hee University Hospital at Gang-Dong, South Korea

##### **Correspondence:** Bark-Lynn Lew – Kyung Hee University Hospital at Gang-Dong, South Korea

**Background**: Cases of dermatitis by injection of certain drugs have been reported. This adverse reaction of drugs presents various features like eczematoid patch, and urticarial lesion, and so on.

**Objective:** The aim of this study was to assess the cause and clinicopathologic findings of injection-induced dermatitis, and to reveal whether the reaction have any relations in the age, site, concentration of the drugs, and time interval from injection of drug to occurrence of skin lesion.

**Methods:** This study enrolled 10 patients who had erythematous skin lesion after injection of causative drugs. The lesions are compared to each other based on the site, time interval from injection of drug to occurrence of skin lesion, and clinical characteristics. We perforemed intradermal test and patch test to each patient with different concentration of causative drugs.

**Results:** The most common causative drugs were diclofenac and vitamin K1. Eczematous type was the most frequent clinical feature. Intradermal test showed more positive results than the patch test. Patch test with diclofenac (as is, 2.5%, 5%, and 10%) and vitamin K1 10% were all negative in 10 patients. And intradermal tests with diclofenac as is and vitamin K1 (0.1%, 1%, and 10%) were performed in 8 patients. Six patients had a positive reaction, consisting of erythema, induration and vesiculation after 1 and 2 days.

**Conclusions:** In these results, most common causative agents were found as diclofenac and vitamin K1. And, it seems that intradermal test is more useful in the diagnosis of injection-induced dermatitis.

## A24 IgE reactivity of recombinant allergen pac c 3 of the Asian needle ant pachycondyla chinensis

### Kyoung Yong Jeong^1^, Myung-Hee Yi^1^, Mina Son^1^, Dongpyo Lyu^2^, Jae-Hyun Lee^1^, Tai-Soon Yong^1^, Chein-Soo Hong^3^, Jung-Won Park^1^

#### ^1^Yonsei University College of Medicine; ^2^Sangji University; ^3^Division of Allergy and Immunology, Department of Internal Medicine, Yonsei University College of Medicine

##### **Correspondence:** Kyoung Yong Jeong – Yonsei University College of Medicine, South Korea

**Background:** Stings from the Asian needle ant are an important cause of anaphylaxis in East Asia. A 23-kDa protein homologous to antigen 5 is the major allergen produced by these ants. In this study, we aimed to produce a recombinant 23-kDa allergen.

**Methods:** Recombinant 23-kDa allergen from the Asian needle ant was expressed in *Pichia pastoris* and purified by ammonium sulfate precipitation and Ni-affinity chromatography. IgE reactivity was demonstrated by ELISA and immunoblotting.

**Results:** The recombinant protein was recognized in 5 of 6 serum samples (83.3%) from patients with demonstrated anaphylaxis to ants. IgE reactivity to a 23-kDa allergen from venom sac extract was specifically inhibited by the recombinant protein.

**Conclusion:** A recombinant 23-kDa allergen from the Asian needle ant was successfully produced in the methylotrophic yeast *P. pastoris*. This protein could be useful for the development of component-resolved diagnostics.

## A25 Characterization of specific IgE antibody related to antigen 5 of echinococcus granulosus

### Mohammadreza Siavashi

#### Pasteur Institute of Iran, Iran

**Background:** Anaphylactic reactions, such as urticaria,edema, respiratory symptoms and anaphylactic shock often complicate the course of cystic echinococcosis (CE).

**Methods**: To investigate the role of the IgE immunoreactive antigen 5(Ag 5) in the sero-positive patients with CE, we determined N-terminal of 57 kDa subunit of Ag5 responsible for IgE and C-terminal of this active antigen related to induction of IgG specifically.

**Results**: Immunoblotting analysis showed that specific IgE to 57 kDa subunit related to inter-chain disulphide band of two 22 kDa and 38 kDa components of Ag5 and conformational epitope on these subunits. In addition, since the 57kDa component arise from the removal of the C-termainal portion of 22kDa subunit of Ag 5, thus IgE specifically recognized N-terminal of 22 kDa subunit which remain bounded to the other component, whereas IgG reacted with C-terminal of 38 kDa component of Ag5.

**Conclusion**: Recognition of the specific binding site on the 57kDa subunit of Ag5 can lead to understanding the regulating mechanism of IgE/IgG production in some immune circumstances that IgE tends to be dominated, whereas in other IgG is predominated.

## A26 Development of binary forecast model of asthma exacerbation: asthma index

### Hey Suk Yun^1^, Ha-Na Kang^2^, Jae-Won Oh^3^, Young Jin Choi^1^

#### ^1^Hanyang University Medical Center; ^2^Hanyang University Seoul Hospital; ^3^Hanyang University Kuri Hospital

##### **Correspondence:** Hey Suk Yun – Hanyang University Medical Center, South Korea

**Background:** Asthma is one of the most common chronic diseases worldwide, and a growing public health concern. It is characterized by chronic bronchial inflammation and a multi-factorial etiology that includes genetic, immunological and environmental factors. Our goal was to develop a patient-customizable asthma forecasting system that takes into account meteorological factors, air pollution, and pollen and viral respiratory infections, which are the most common environmental factors known to exacerbate asthma.

**Methods:** We analyzed the health insurance records of patients who visited the emergency departments in Seoul due to an asthma attack and who were treated with salbutamol. Our potential predictors of asthma symptoms included meteorological factors (temperature, humidity, air pressure, and amount of sunshine), air pollution factors (ozone and Asian yellow dust), environmental factors (pollen) and health-related factors (seasonal influenza viral infection). Patients were assigned to five age groups and separated according to gender and season (5 × 2 × 4 groups), which resulted in a total of 40 groups. Three models (a multiple regression model, a logistic regression model, and a decision tree) were tested for their ability to predict exacerbated asthma symptoms (referred to as “attention” symptoms). The suitability of the final model was assessed using predictability skill scores.

**Results:** Logistic regression and the decision tree provided the best forecasts. Using the estimated binary values for “continuous management” versus “attention” symptoms, the optimal threshold was selected.

**Conclusions:** Our binary forecasting model may improve the prediction of asthma exacerbation in a clinical setting.

## A27 Different levels in rantes, IL-5 and TNF-á between the nasal polyps of adolescents with allergic, local allergic and non-allergic rhinitis

### Jae-Won Oh^1^, Young Jin Choi^1^, Ha-Na Kang^2^

#### ^1^Hanyang University Kuri Hospital; ^2^Hanyang University Seoul Hospital

##### **Correspondence:** Ha-Na Kang – Hanyang University Seoul Hospital, South Korea

**Background:** Nasal polyps are the result of chronic inflammation of the upper airways that develops in the ethmoidal and middle turbinate area. They are characterized by the presence of a number of inflammatory cells resulting from infiltration of eosinophils, Th2 cells, mast cells, and macrophages. Nasal mucosal immune-reactivity may occur in varying degrees accompanying polyps in allergic and non-allergic rhinitis. RANTES is locally produced within the nasal microenvironment of polyps and may be responsible for the eosinophil recruitment. Tumor necrosis factor–alpha (TNF-α) along with Th2 cytokines such as interleukin-5 (IL-5) may stimulate nasal polyp fibroblasts to produce inflammatory mediators. We evaluated the differences in levels of RANTES, IL-5, and TNF-α in nasal polyps from adolescents with allergic and non-allergic rhinitis. Our goal was to figure out the pathogenesis of allergic, local allergic and non-allergic rhinitis by evaluate different levels in RANTES, IL-5, and TNF-α between the nasal polyps and serum from them.

**Methods:** We recruited total 38 adolescents with allergic rhinitis (AR) (n=15, mean age: 17.4±4.2 yrs old), local allergic rhinitis (LAR) (n=9, mean age: 15.9±5.5 yrs old), and non-allergic rhinitis (NAR) (n=14, mean age: 15.6±2.9 yrs old) undergoing polypectomy. Atopic status was defined as presenting a sufficiently high total IgE serum concentration (IgE > 200 IU/mL) and a positive skin prick test or serum allergen test such as MAST (Green cross MS, Seoul, Korea) or ImmunoCAP system (Pharmacia, Uppsala, Sweden). Immunoassays were performed using polyp tissue homogenates and sera to quantify the levels of RANTES, TNF-α, and IL-5, and with sera to assess total IgE, eosinophil cationic protein (ECP) produced by them.

**Results:** RANTES levels were higher in LAR than in NAR, but there was no significant difference between AR and NAR. IL-5 and TNF-α levels were higher in AR and LAR than in NAR but IFN-γ levels did not differ. There was significantly correlated between concentration of RANTES between polyp tissue homogenates and serum (R2 =0.51, *P*<0.05, n=38). IL-5, TNF-α, and IFN-γ also demonstrated positive correlation between concentration of that between polyp tissue homogenates and serum, however, there were not significant.

**Conclusions:** RANTES levels were higher in polyp tissue homogenates from LAR than in those from NAR. Therefore, RANTES probably involves in the pathogenesis of LAR. IL-5 and TNF-α levels were higher in polyp tissue homogenates and sera from AR and LAR than in those from NAR. So IL-5 and TNF-α probably play important role in the pathogenesis of AR and LAR.

## A28 Tgfβ1 level is associated with VDR gene polymorphism in children with allergy diseases

### Tatiana Sentsova^1^, Ilya Vorozhko^1^, Olga Chernyak^1^, Vera Revyakina^1^, Anna Timopheeva^1^, Andrey Donnikov^2^

#### ^1^Institute of Nutrition; ^2^DNA-Technology, JSC

##### **Correspondence:** Tatiana Sentsova – Institute of Nutrition, Russia

**Background:** vitamin D receptor (VDR) is intracellulary located on the cells of the immune system and is involved in the immune response. The development of allergic inflammation is controlled by different genes, including the gene VDR.

**Aim**: investigate TGFβ1 levels in children with food allergy and different variants of VDR gene polymorphism.

**Materials and methods**: We examined 130 infants with allergic diseases aged from 1 to 12 months, who were on the artificial feeding. Measurement of TGFβ1 levels was performed by immunoenzyme method. RT-PCR with melting curve analysis was used for TaqI, BsmI and FokI VDR single nucleotide polymorphisms (SNP) detection. For statistical analysis Kruskal-Wallis and post-hoc tests were used.

**Results**: The frequency of allele A, homozygous A/A and heterozygous G/A genotypes in VDR gene site BsmI were significantly increased in children with allergic diseases compare population (OR = 1,81, p = 0.04; OR = 2,03, p = 0,05 and OR = 1,8, P = 0,05 respectively). Significantly decreased serum TGFβ1 levels in carriers of variants A/G and G/G comparably variant A/A carriers in FokI site (Me(q1;q3): A/A - 1134(897;1705) vs A/G – 610(511;919) vs G/G – 838(734;901) pg/mL, p=0.0216 ) were revealed. Statistically significant differences in serum TGFβ1 levels for TaqI and BsmI sites in VDR gene were not detected.

**Conclusion:** FokI site VDR gene polymorphism may indirectly influence on TGFβ1 synthesis in allergy diseases development.

## A29 Dynamics of immunological biomarkers in children with food allergy fed goat milk formula

### Tatiana Sentsova, Ilya Vorozhko, Olga Chernyak, Vera Revyakina, Anna Timopheeva

#### Institute of Nutrition

##### **Correspondence:** Tatiana Sentsova – Institute of Nutrition, Russia

**Background:** Immunology evaluation of different diet therapy types in children with food allergy currently required. Goat milk formula are often used in diet therapy of children with food allergy.

**Aim**: Investigate dynamics of immunological biomarkers in children with food allergy wich used goat milk formula supplementation.

**Materials and methods**: We examined 66 infants with food allergy aged from 1 to 12 months, who were on the artificial feeding using goat milk formula. The dynamics of immunoregulatory substances IL-2,-4,-5,-10,-13, TGF-β1 and allergen-specific IgE and IgG antibodies to cow milk protein, α-lactalbumin, β-lactglobulin, casein, soy and goat milk were measured by immunoenzime method. Observation duration was 28-30 days.

**Results**: Dynamics of immunological biomarkers characterized decreased levels of allergen-specific IgG antibodies to the cow milk protein and its fractions, reduced levels of allergen-specific IgE antibodies to the cow milk protein and α-lactalbumin, and reduction of IL-4, -5, levels. These changes were correlated with clinical food allergy symptoms reduction.

**Conclusion:** Assessment of of allergen-specific IgG antibodies to the cow milk protein and its fractions, allergen-specific IgE antibodies to the cow milk protein and α-lactalbumin, IL-4 and IL-5 can be used for immunology evaluation of the effectiveness goat milk formula supplementation.

## A30 Association between obesity, abdominal obesity and adiposity and the prevalence of atopic dermatitis in young Korean adults: The korea national health and nutrition examination survey, 2008–2010

### Ji Hyun Lee^1^, Young Min Park^1^, Sang Soo Choi^1^, Kyung Do Han^2^, Han Mi Jung^1^, Young Hoon Youn^1^, Jun Young Lee^1^, Yong Gyu Park^2^, Seung-Hwan Lee^1^

#### ^1^Seoul St. Mary’s Hospital, South Korea; ^2^College of Medicine, the Catholic University of Korea

##### **Correspondence:** Ji Hyun Lee – Seoul St. Mary’s Hospital, South Korea

**Background**: Whether obesity is a risk factor for atopic dermatitis (AD) remains unclear. The aim of this study was to investigate the association between obesity and AD in Korean young adults.

**Methods:** We included nationally representative data of 5,202 Korean adults aged 19–40 years, obtained from the cross-sectional Korea National Health and Nutrition Examination Survey 2008–2010.

**Results:** AD prevalence exhibited a U-shape trend in relation to body mass index (BMI), waist circumference (WC), and total body fat (BF) percentage, especially in young adult women. Women with BMI ≥ 25 kg/m^2^, WC ≥ 80 cm, and highest quartile (Q4) of total BF percentage had the highest prevalence of AD. The odds ratio (OR) for participants with both BMI ≥ 25 kg/m^2^ and WC ≥ 80 cm was 3.29 (95% confidence interval [CI] 1.71–3.55); therefore, having both general and abdominal obesity was considered prominent risk factors for AD in young women. After adjustment for confounding factors, including age, smoking, alcohol drinking, exercise, and vitamin D levels, high BMI (≥30 kg/m^2^) (OR = 4.241, 95% CI: 1.60–11.24), high WC (≥80 cm) (OR = 2.062, 95% CI: 1.08–3.94), and high BF percentage (Q4) (OR = 2.073, 95% CI: 1.22–3.52) were shown to be significantly associated with AD in young adult women.

**Conclusions:** In this large-scale nation-wide study in Korean adults, obesity was positively related with the presence of AD in women. Our findings suggest that weight management might help to prevent AD.

## A31 Associations of natural history and environmental factors with asthma among children in rural and urban areas of Guangdong, China

### Jing Li^1^, Mulin Feng^2^, Marjut Roponen^3^, Bianca Schaub^4^, Gary WK Wong^5^, Zhaowei Yang^6^

#### ^1^State Key Laboratory of Respiratory Disease, the First Affiliated Hospital of Guangzhou Medical College; ^2^Guangzhou Institute of Respiratory Disease, the First Affiliated Hospital of Guangzhou Medical College; ^3^University of Eastern Finland; ^4^University Children’s Hospital Munich; ^5^Prince of Wales Hospital; ^6^The First Affiliated Hospital, Guangzhou Medical University, China

##### **Correspondence:** Zhaowei Yang – The First Affiliated Hospital, Guangzhou Medical University, China

**Background:** Asthma is influenced by natural and environmental factors, but associations were still inconsistent.

**Objective:** We aimed to explore associations between natural history and environmental factors and asthma in urban and rural children in China.

**Methods:** A questionnaire survey was performed in 7164 children from urban Guangzhou and 6087 from rural Conghua. Subsamples of 854 children (419 from Guangzhou, 435 from Conghua) were recruited for case-control study including detail questionnaire for natural and family history, environmental exposure and eating behaviors, histamine airway provocation, allergen skin prick test, and serum antibody analysis. House dust samples from 76 Guangzhou and 80 Conghua families were obtained to analyze levels of endotoxin, house dust mite and cockroach allergens.

**Results:** The prevalence of doctor-diagnosed-asthma was lower in children from Conghua (3.4%) than Guangzhou (6.9%, p<0.001) in the screening survey. A lower percentage for confirmed asthma (3.0% vs 28.9%) and sensitization (13.3% vs 50.6%) was found in rural than urban subjects (p<0.001) in the nested case-control study. Parental allergy (3.30 [2.08-5.24]), antibiotic usage (3.29 [1.93-5.60]), high frequency of milk consumption (2.31 [1.45-3.68]) and increased level of house dust *Der f* 1 (1.71 [1.34-2.19]) were positively associated with confirmed asthma (*p*<0.001). Breastfeeding (0.62 [0.39-0.97]), raising dogs (0.50 [0.26-0.94]) and level of house dust endotoxin (0.69 [0.50-0.95]) were negatively associated with confirmed asthma (*p*<0.05).

**Conclusions:** Parental allergy, early antibiotic administration, high frequency of milk intake and level of *Der f* 1 exposure might be risk factors for asthma, while breastfeeding, dog ownership and endotoxin exposure were protective.

## A32 The effect of CO2-enriched atmospheres to producing of allergenic pollen by ragweed

### Young Jin Choi^1^, Ha-Na Kang^2^, Jae-Won Oh^3^

#### ^1^Hanyang University Medical Center, South Korea; ^2^Hanyang University Seoul Hospital; ^3^Hanyang University Kuri Hospital

##### **Correspondence:** Young Jin Choi – Hanyang University Medical Center, South Korea

**Background:** The prevalence of allergic diseases has increased result from exposure to environmental pollutants. Aeroallergen exposure is associated with allergic rhinitis and asthma. Pollens have long been known to be a significant cause of allergic disease. Pollen in heavily polluted zones can express a larger amount of proteins described as being allergenic. In the presence of high CO2 concentrations and temperatures, plants increase their pollen output.

**Method:** Ragweed establishment: 1) Chamber study: Ten plants of ragweed were established in open-top chamber at different concentration of CO2 (380-400, 500-520, 600-620, 1000-1100ppm). 2) Field study: Beginning in March 2012 and 2014, a rural (Pocheon, Kyunggi-do, annual mean CO2: 230ppm) and urban (Kangnam, Seoul, annual mean CO2: 440ppm) locations were established. Seeds of common ragweed (*Ambrosia artemisiifolia*) and giant ragweed (*A. trifida*) were obtained from Daejin University from a common seed lot of ragweed. At final harvest, entire plants were collected. To determine qualitative changes in pollen, harvested pollen grains were suspended in 95% ethanol. The crude soluble pollen protein preparations were stored at -20°. Protein content of the extracts was quantified. Concentration of Allergens (common ragweed (*Amb a* 1) and giant ragweed(*Amb t* 5) was quantified through use of double sandwich ELISA.

**Results:** 1) chamber study: 1) chamber study: Concentration of *Amb a* 1 was increased with increased CO2 Conc. (380-400, 500-520, 600-620, 1000-1100ppm: 18.4±5.0, 30.8±13.1, 42.5±11.2, 50.1±21.2 ng/mL), Concentration of *Amb t* 5 was increased with increased CO2 Conc. (380-400, 500-520, 600-620, 1000-1100ppm: 22.1±6.8, 36.3±11.6, 48.3±19.5, 64.6±21.3 ng/mL), 2) Field study: There were not significantly different between Pocheon (CO2 230ppm: 16.0±2.0ng/mL) and Seoul (CO2 440ppm: 20.3±8.6ng/mL) in Conc. of *Amb a* 1, also Pocheon (CO2 230ppm: 24.5±6.9ng/mL) and Seoul (CO2 440ppm: 28.3±6.2ng/mL) in Conc. of Amb t 5, though Conc. of *Amb a* 1 and *Amb t* 5 were increased at Seoul than those at Pocheon.

**Conclusion:** Increased CO2 significantly influence allergenicity and pollen concentration of common ragweed through the chamber and field study. The elementary example given here demonstrates strong probable links between rising CO2 levels and increased allergic diseases. We suggest that urbanization might provide a alternative to current experimental methods evaluating plant responses to climate change.

**Keywords:** Allergens: Plant; Aeroallergens; Allergens: Environmental Control

## A33 Application evaluation of house dust mite and components specific-IgE and IgG4 in specific immunotherapy with allergic diseases

### Baoqing Sun, Peiyan Zheng

#### First Affiliated Hospital Guangzhou Medical University

##### **Correspondence:** Baoqing Sun – First Affiliated Hospital Guangzhou Medical University, China

**Objective:** To observe the dynamic change of specific IgE (sIgE) and specific IgG4 (sIgG4) to house dust mite including Dermatophagoides pteronyssinus (Der p) and its main components Der p1 and Der p2 after specific immunotherapy (SIT), and to evaluate the effect of its application in clinical monitoring of desensitization.

**Methods:** This study observed the immune indexes of 51 children patients including the serum sIgE and sIgG4 to five periods of pre SIT(Pre)and after SIT (half of year (0.5Y),1 year,2 year(2Y) and 3 year(3Y)). 20 patients by conventional drug treatment were collected as control group.

**Results:** After half of year of SIT, the levels of serum sIgE to Der p, Der p1 and Der p2 increased continuously, however, by then began to decline, after three years treatment, the levels of sIgE to Der p1 and Der p2 were reduced step by step, particularly, sIgE to Der p1 were significantly lower than Pre treatment. The levels of sIgG4 to Der p, Der p1 and Der p2 increased significantly along with the process of SIT. The increasing range of Der p sIgG4 reached to the maximum, followed by Der p1 and then Der p2. The sIgE/sIgG4 ratio of three allergens were decreased after SIT, and the biggest dropped degree was the sIgE/sIgG4 ratio to Der p1. The low age group of children (5 to 8 y) response to immune higher and faster than the high age group of children (9-16 y) after SIT, and the levels of components sIgG4 to Der p1 and Der p2 elevated faster than sIgG4 to Der p.

**Conclusions:** In the early stage of treatment, the levels of Der p, Der p1 and Der p2 to sIgE and sIgG4 in serum by the body in a state of immune stimulating were significantly increased. As the SIT, the levels of sIgE gradually decreased, and the levels to sIgG4 increased, namely the sIgE/sIgG4 ratio reduced gradually, and the biggest declined of sIgE/ sIgG4 ratio was Der p1. the fastest degree to SIT reaction was the low age children with allergies.

## A34 Effect of Asian dust events on asthma according to the socioeconomic status using claim data in KOREA

### Yoon-Sung Park

#### Inha University Hospital, South Korea

**Objective:** The purpose of this study is to evaluate the effects of Asian dust events on asthma by socioeconomic status using claim data.

**Methods:** Case crossover design was used. This study is based on the national health insurance claim (ICD : J45, J46), air pollutants [PM_10_(μg/m^3^), CO(ppb), SO_2_(ppb), NO_2_(ppb), O_3_(ppb)] and climate [temperature(°C), humidity(%), visibility(km), wind speed(m/s), air pressure(hPa)] data from 2007 to 2013 in Seoul and Incheon, Korea. The socioeconomic status was classified into health insurance group and medical aid group. The daily maximum value of air pollutants and daily average climate were calculated. The daily numbers of asthma cases on the ‘Event’ days were compared with ‘Control’ days. To select event days, 2 criteria were applied: 1) exclude weekends 2) excluded reoccurred Asian dust events on the 14 days before and after the Asian dust event. It was observed for 7 days after the events days. Control days is defined as the 7 days before and after the event days. Log poisson regression was used to estimate the ratio of averaged asthma cases using age, gender, region and climate between event and control days.

**Results:** 7 event days were selected. On the event days, the average numbers of asthma cases were much more than the control days on the gender, age, region and socioeconomic status. However, there was no significant difference statistically. Humidity of the event days was lower (*p*=0.0950) but wind speed (*p*=0.0203) and PM_10_ (*p*=0.0376) were higher than control days. The estimated ratio of averaged asthma cases on event days was 0.96 (95% C.I.: 0.95-0.98). According to the socioeconomic status, the ratio of asthma patients during 7 days from ‘day +0’ to ‘day +6’ were as follows: 0.96 (0.95 - 0.97) on the day +0, 1.27 (1.26 - 1.29) on the day +1, 1.11 (1.09 - 1.13) on the day +2, 1.25 (1.23 - 1.26) on the day +3, 1.13 (1.12 - 1.15) on the day +4, 1.06 (1.04 - 1.07) on the day +5 and 0.83 (0.81 - 0.84) on the day +6 in the health insurance group. Also, in medical aids group, the estimated ratio of the each day were 1.00 (0.94 - 1.07), 1.14 (1.06 - 1.22), 1.15 (1.06 - 1.25), 1.18 (1.11 - 1.25), 1.08 (1.01 - 1.15), 1.02 (0.94 - 1.10) and 0.78 (0.74 - 0.83).

**Conclusion:** Asian dust events can worsen asthma and its effects on asthma appear differently by the socioeconomic status. Time lag analysis is needed in studying asthma effect on Asian dust event using claim data.

## A35 TSLP downregulates human â-defensin 2 through STAT3-dependent pathway in keratinocytes

### Sang Wook Son

#### Korea University, South Korea

TSLP downregulates human β-defensin 2 through STAT3-dependent pathway in keratinocytes.

Department of Dermatology, Korea University College of Medicine.

Atopic dermatitis (AD) is a chronic and pruritic inflammatory skin disease associated with a defective skin barrier and a dysregulation of Th2 immune response. Most patients with AD show reduced expression of antimicrobial peptides (AMPs), which may plays an important role in host defense against bacteria, fungi, and viruses infection. Thymic stromal lymphopoietin (TSLP) has a pathogenetic role in the initiation and maintenance of allergic inflammatory diseases including AD. The function of TSLP was revealed in previous studies. However, it remains unknown that the regulatory mechanism of HBD-2 expression by TSLP in primary human keratinocytes.

The present study demonstrates dose-dependent decreases of human β-defensin-2 (HBD-2) mRNA and protein levels in keratinocytes following stimulation to TSLP. In addition, this study demonstrated the effect of TSLP on HBD-2 expression in human skin equivalent models. We further investigated the regulatory mechanisms of TSLP-reduced HBD-2 expression in primary human keratinocytes. TSLP induced STAT-3 protein expression in primary human keratinocytes. Data from immunohistochemical stain for skin lesions of atopic patients demonstrated the increased expression of STAT-3 than normal skin. HBD-2 was regulated through STAT-3-dependent pathway in keratinocytes. Our results from chromatin immunoprecipitation (ChIP) assay and electrophoretic mobility shift assay (EMSA) showed the direct regulation of TSLP on HBD-2 promoter.

Taken together, this study reveals that TSLP stimulation may reduce HBD-2 expression through a STAT–3 dependent mechanism in human keratinocytes. The present study suggests a novel and key role of STAT-3 in TSLP-mediated immune response in keratinocytes. Moreover, it would be helpful for understanding the pathologic signal transduction in AD.

## A36 Effects of anti-IgE on IL-4, IL-5, IL-17, and CD19,20,200 in a case of netherton syndrome (SPINK5 mutation)

### Sukran Kose^1^, Kemal Kiraz^2^, Arzu Didem Yalcin^3^

#### ^1^Tepecik Training and Research Hospital; ^2^Antalya Education and Research Hospital; ^3^Antalya Training and Research Hospital, Turkey

##### **Correspondence:** Arzu Didem Yalcin – Antalya Training and Research Hospital, Turkey

**Aim:** Netherton syndrome (NS) is associated with the mutation in the SPINK5 gene, which codes LEKTI (lymphoepithelial Kazaltype related inhibitor), a serine protease inhibitor. LEKTI is expressed in epithelium, mucosa, and thymus. It is localized in the stratum granulosum of normal skin. NS is a rare genodermatosis characterized by autosomal recessive inheritance pattern, unknown etiology, ichthyosiform cutaneous changes, atopic diathesis, and alterations in the hair shaft. As a result of aging coupled with immune deficiency, clinical symptoms may vary.

**Materials and methods:** A 20-year-old white Caucasian male presented to our Allergy-Immunology Unit with pruritus of the face and feet, skin desquamation, and sparse and thin hair. Dermatological examination demonstrated brittleness and scaling of the hairs, eyebrows, and eyelashes; erythema and desquamation of the cheeks; pinkish-red macules with scales; hypopigmented macular lesions on the ichthyosiform skin involving the area beginning from the patella and extending to the distal part of the leg; and lichenified, erythematous plaques with patchy fissures in both antecubital and popliteal regions. Microscopic examination of the material collected from the nails showed no fungal components. Subungual hyperkeratosis, discoloration, and destruction were noted in the toe nails. Patient presented to our clinic with sparse and brittle hair along with pruritic, erythematous, and scaling cutaneous lesions. Patient underwent a clinical examination and laboratory analyses. Based on the clinical and laboratory findings, patient diagnosed with Netherton syndrome.

**Results:** Laboratory analyses yielded normal results except for a leukocyte count of 7,300/μL, CRP of 25.6 mg/mL (normal range:0-5 mg/mL) and a total IgE of 31700 IU/mL (normal range:0-100 IU/mL) and IgG of 20g/L(normal range:7-16), IgA of 4g/L(normal range:0.7-4), IgM of 0.9g/L(normal range:0.4-2.3) and cow’s milk,mite,grass, egg, hazelnut, orange, wheat, strawberry allergy were detected in the specific IgE studies. Anti nuclear antibody, hepatitis markers, HIV and rheumatoid factor were negative in patient. Renal function tests, complement-4 (C4), C3 levels were within normal ranges. During omalizumab treatment and after a short-term (4 months) treatment with omalizumab, he had a decreased CRP, IL-4, IL-5, IL-17, IL-1β and CD19-20, CD200 and had an increased CXCL8 levels.

**Conclusion:** NS, peeling skin syndrome type B, and skin dermatitis--multiple severe allergies--metabolic wasting syndrome are 3 autosomal recessive disorder resulting from aberrant regulation of epidermal desquamation. To our knowledge, this is the first time an association between omalizumab use and NS has been documented. As a conclusion allergic skin symptoms (pruritus, erythema, desquamation) and mucosal symptoms were decreased in patient.

## A37 Augmentation of arginase 1 expression exacerbates airway inflammation in murine asthma models

### Sehyo Yune^1^, Jae-Won Paeng^2^, Mi-Jung Oh^3^, Byung-Jae Lee^1^, Dong-Chull Choi^4^, Young Hee Lim^5^, Kyoung Won Ha^6^, Jin-Young Lee^7^

#### ^1^Samsung Medical Center; ^2^Samsung Biomedical Research Institute; ^3^Bundang Jaesang Hospital; ^4^Samsung Medical Center, Sungkyunkwan University School of Medicine; ^5^Ansan Sarang General Hospital; ^6^Seoul Samsung Medical Clinic; ^7^Health Promotion Center, Samsung Medical Center, South Korea

##### **Correspondence:** Jin-Young Lee – Health Promotion Center, Samsung Medical Center, South Korea

**Purpose**: The expression of nitric oxide synthase-2 (NOS-2) and levels of exhaled nitric oxide (NO) are increased in allergic airway diseases. However, whether the upregulation of NOS has a deleterious effect on airway inflammation and hyperresponsiveness remains controversial. We aimed to clarify the pathophysiological role of NOS-2 in competition with arginase, using NOS-2 knockout mice in models of allergic asthma.

**Methods:** We compared airway inflammation and hyperresponsiveness, as well as arginase expression, using NOS or arginase inhibitors in 6-week-old female C57BL/6, and NOS-2 knockout mice.

**Results:** Airway hyperresponsiveness was unaffected by NOS-2 depeletion or NOS inhibition. However, airway inflammation was aggravated in NOS-2 knockout mice than in wild-type mice with augmentation of arginase I expression. Inhibition of arginase attenuated airway inflammation in wild-type and NOS-2 knockout mice.

**Conclusions:** NOS-2 knockout mice showed increased ovalbumin-induced airway inflammation with augmentation of arginase I expression. Our results suggested that imbalance between NOS-2 and arginase with augmentation of arginase I expression resulted in aggravation of airway inflammation, and inhibitors of arginase I may be effective in suppressing allergic inflammation.

## A38 Caregivers of children with no food allergy – their experiences and perception of the condition

### Kiwako Yamamoto-Hanada, Masami Narita, Masaki Futamura, Yukihiro Ohya

#### National Center for Child Health and Development, Japan

##### **Correspondence:** Kiwako Yamamoto-Hanada – National Center for Child Health and Development, Japan

**Background:** Food allergy (FA) is one of the most important health issues in school children. Although one of the commonest places where pediatric anaphylaxis occurs is the home of a peer, the perception and experiences of caregivers who do not have a child with FA are unclear.

**Methods:** Anonymous paper-based questionnaire were distributed to caregivers of FA children (FA caregivers) in National Center for Child Health and Development and caregivers of non-FA children (non-FA caregivers) in public school in Tokyo. We examined the perception and experiences of FA among three groups: Group1, non-FA caregiver who had not witnessed adverse reactions and near-miss events related to FA; Group 2, non-FA caregivers who had witnessed adverse reactions and near-miss events related to FA; and Group3, caregivers who had a child with FA.

**Results:** Epinephrine auto-injector was recognized by 43.6% non-FA caregivers. Only 2.8% of non-FA caregivers had experienced a child being bullied, harassed or refused by a school because of FA. There were more caregivers in Group 2 who thought they could take the appropriate action if they witnessed a child with adverse response to causal food compared to Group 1 (p<0.05). All groups felt anxious over adverse events related to FA.

**Conclusion:** The experiences and perception of FA was lacking low among non-FA caregivers. Therefore, it is also important to provide FA education for non-FA caregivers in order to improve the quality of life of FA children.

## A39 Evaluation of Drug Provocation Tests in Korean Children: A Single Center Experience

### Jihyun Kim^1^, Jinwha Choi^1^, Kwanghoon Kim^1^, Jaehee Choi^2^, Kangmo Ahn^1^

#### ^1^Samsung Medical Center, South Korea; ^2^Sahmyook Medical Center

##### **Correspondence:** Jihyun Kim – Samsung Medical Center, South Korea

**Background:** The aims of this study were to evaluate the common causative drugs of type B adverse drug reactions (ADRs) and to analyze the relationships between host factors and the results of drug provocation tests (DPTs) in Korean children.

**Methods:** We retrospectively reviewed the medical records of all children younger than 19 years of age who underwent a DPT between November 1994 and November 2014. Open provocation tests were performed with non-steroidal anti-inflammatory drugs (NSAIDs), acetaminophen, aminopenicillins, cephalosporin, non-β-lactam antibiotics, antiepileptic drugs, or other drugs.

**Results:** Overall, 84 DPTs were performed in 56 patients whose median age was 7.5 years (range, 6 months to 18 years). DPTs were positive in 25 (29.8%) of 84 cases, which translated to 18 (32.1%) positive findings in 56 patients. Drugs that provided positive results included NSAIDs (7 cases, 28.0 %), aminopenicillins (5 cases, 20.0%), acetaminophen (4 cases, 16.0%), cephalosporins (3 cases, 12.0%), and non-β-lactams (2 cases, 8.0%). Anaphylaxis was noted in 5 (20.0%) of 25 cases. There were no serious complications of DPTs in any of the subjects. The median age was 10.5 years for children who had a positive result following the DPT and 5.0 years for those with negative results (*P*value = 0.019).

**Conclusion:** The overall positive DPT rate was 29.8%, with a lower rate noted in younger children. DPTs can be performed safely in children with suspected ADRs in order to achieve a correct diagnosis.

## A40 Danyoung classification 2015 update by digital HD endoscopic evaluation

### Sun-Ho/Brian Chang

#### King Sejong ORL-Hns Clinic, South Korea

**Purpose**

After I first joined at 1996 AAO-HNSF Annual Meeting in Washington, DC as residency, I became interested in high resolution nasal endoscopy and I planned study design for allergic rhinitis patients. Research diagram of DANYOUNG classification consist of the study design, with the three periods at 20 years strategic plan. Using digital HD 3chip endoscopy, digital HD Flexible videoendoscopy and 3chip endoscopy, DANYOUNG classification 2015 update is achieved by better objective visual data files.

**Method**

From Jun 2001 to Apr 2015, nasoendoscopic video data files were collected from allergic rhinitis patients. Nasoendoscopic video system is consist of stryker digital HD 3chip video camera system 1488 model, kay pentax digital HD Flexible videoendoscopy VNL-1190STK model and stryker 3chip video camera system 888, 988, 1088 model. Video data storage system is consist of stryker SDC-pro, SDC-HD, SDC3 recording system and NAS-LG system.

**Result**

DANYOUNG classification hypothesis based on surface change of allergic rhinitis patient’s inferior turbinate mucosa.This classification consist of 3 stages. Stage 1 is hypertrophy state. Stage 2 is dimple state. Stage 3 is wrinkle state. A clear definition of stage is more characterized under digital HD endoscopic evaluation. Especially, irreversible change of nasal mucosa such as dimple and wrinkle shape is confirmed again.

**Conclusion**

DANYOUNG classification has very simple, objective advantage and useful on early diagnosis of allergic rhinitis. When OMU-CT is using together for allergic rhinitis patient, this staging is clear more than. DANYOUNG classification 2015 update can related on ARIA 2010 update, can be one in the future.

**Keywords**: allergic rhinitis, stryker digital HD 3chip endoscopy, kay pentax digital HD Flexible videoendoscopy, Danyoung classification 2015 update

## A41 Effect on quality of life of the mixed house dust mite/weed pollen extract immunotherapy in polysensitized patients

### Lisha Li

#### Peking Union Medical College Hospital, China

**Background:** Although many patients with allergic rhinitis have symptoms due to sensitization with more than one kind of allergens, and mixed allergen extracts are widely used for immunotherapy, there are few published trials.

**Methods:** We performed a 1-year single-centre cohort study of subcutaneous immunotherapy using house dust mite extract, weed pollen extract, or mixed house dust mite/weed pollen extract in 44 allergic rhinitis patients. All the allergens responsible for the symptom of each patient were included in his immunotherapy. Quality of life was evaluated with the Rhinoconjunctivitis Quality of Life Questionnaire (RQLQ) before and after 1-year immunotherapy.

**Results:** After 1-year subcutaneous immunotherapy, RQLQ score of the house dust mite group 1.02±0.82(n=12)was significantly better than baseline RQLQ score 2.25±1.29 (p=0.024) ; RQLQ score for the weed pollen season of the weed pollen group 1.53±1.13 (n=21)was significantly better than baseline level 3.08±1.22 (p=0.000); RQLQ score for the weed pollen season of the mixed house dust mite/weed pollen group 1.78±1.02 (n=11) was significantly better than baseline level 2.92±1.25 (p=0.004), RQLQ for ordinary times of the mixed house dust mite/weed pollen group 0.62±0.62 (n=11) was significantly better than baseline score 1.23±0.84 (p=0.002). The reduction of RQLQ score in the house dust mite group was 1.23±1.63, and 1.55±1.24 in the weed pollen group after 1-year treatment. In the mixed house dust mite/weed pollen group, RQLQ for ordinary times deceased by 0.60±0.47, with no difference compared to the house dust mite group (p=0.224); RQLQ for the weed pollen season decreased by 1.14±1.01, with no difference compared to the weed pollen group (p=0.358).

**Conclusions:** There was no significant difference between the effect on quality of life of the mixed house dust mite/weed pollen extract immunotherapy and the effect of the house dust mite extract or the weed pollen extract immunotherapy. The efficacy of multi-allergen immunotherapy was not weaker than that of single-allergen immunotherapy.

## A42 Ambient desert dust and allergic symptoms: A time series analysis from a national birth cohort (JECS)

### Yu-Ichi Adachi^1^, Kumiko Tsuji Kanatani^2^

#### ^1^Toyama University School of Med; ^2^Kyoto University, Japan

##### **Correspondence:** Kumiko Tsuji Kanatani – Kyoto University, Japan

**Background:** Accumulating evidence suggests that desertification and climatic variability can contribute to increased desert dust formation in the air. Desert dust has been shown to exert adjuvant effects in animals.

**Objective:** To examine if desert dust enhances allergic symptoms in real-life settings and to investigate its effect modifiers.

**Methods:** We conducted a cohort study for 3,327 pregnant women during spring and fall in the period spanning October 2011 to May 2013 in three regions in Japan as an adjunct study of the Japan Environment & Children’s Study. We timely acquired participants’ daily symptom scores by sending a web-based questionnaire on high desert-dust days and on some randomly selected other days (control days) for each participant.

**Results:** Pregnant women showed an increased risk of allergic symptom on protocol-defined high desert-dust days (OR 1.24, 95%CI 1.16 – 1.32). The risk elevation was observed from a low level of desert dust in a dose-dependent manner even on control days. The risk-increase according to desert dust levels was observed when the air simultaneously contained pollen from Japanese cedar and cypress among subjects with positive serum IgE antibodies to Japanese cedar pollen, while no clear risk-increase was observed in the absence of pollen in the air.

**Conclusions:** Ambient desert dust level was associated with increased risk of allergic symptom exacerbation in pollen-sensitized pregnant women when pollen was present in the air. The increase was dose-dependent, and was observed from a very low level. These results support a hypothesis that desert dust exerts adjuvant effects in human.

## A43 Individuals Allergic to Cow’s Milk Should be Vigilant When Consuming Beef Because It May be Injected Beef

### Shigeyuki Narabayashi^1,2^, Ikuo Okafuji^2^, Yuya Tanaka^2^, Satoru Tsuruta^2^, Nobue Takamatsu^3^

#### ^1^Kakogawa City West Hospital, Japan; ^2^Kobe City Medical Center General Hospital, Japan; ^3^Beppu University

##### **Correspondence:** Shigeyuki Narabayashi – Kakogawa City West Hospital, Japan

**Background:** Natural “marbled” beef is popular in Japan, but is expensive. “An injected beef”, which is processed to improve texture of dairy cow’s meat to give palate feeling of marbled beef by injection of fat, is sometimes served at relatively low price in restaurants. Such meat might contain milk protein (casein) as one of food additives.

**Methods:** A 2-year-old boy with a history of adverse reactions to hens’ eggs, cow’s milk, and wheat from the age of 6 months had an anaphylactic reaction after eating beef steak at a hotel restaurant. This reaction occurred even though his parents asked the restaurant to ensure that the dish was free of hen’s eggs, cow’s milk, and wheat. We tried to discover the cause of this anaphylactic reaction. We asked the hotel restaurant by telephone about the ingredients of the dish they had served and to send some pieces of beef. We undertook a skin prick test with the meat that they sent. We determined the quantity of cow’s milk protein in a piece of injected beef using an enzyme-linked immunosorbent assay (FASTKIT™; Nipponham, Tokyo, Japan).

**Results**: The hotel restaurant did not use hens’ eggs, cow’s milk, or wheat, but they used injected beef that was labeled as containing casein, and they were not aware of this fact. The skin prick test was strongly positive for raw injected beef and heated beef. A piece of injected beef contained 1.193 mg/mL of cow’s milk protein, which was sufficient to induce an allergic reaction.

**Conclusions:** Even if injected beef is labeled appropriately, an allergic reaction can occur if chefs do not know that casein is a milk protein. Chefs need to be informed (in detail) about food allergies.

## A44 Quality of life of chronic rhinosinusitis patients with or without nasal polyps in Korea

### Soo Whan Kim^1^, Do Hyun Kim^2^

#### ^1^College of Medicine, the Catholic University of Korea, South Korea; ^2^The Catholic University of Korea

##### **Correspondence:** Soo Whan Kim – College of Medicine, the Catholic University of Korea, South Korea

**Background:** This is the first study of its kind to investigate the relationship between chronic rhinosinusitis (CRS) with or without nasal polyps (NP) and health-related quality of life (HRQoL) within the Korean population.

**Objective:** We sought to evaluate the association between CRS and HRQoL after adjustment for confounding factors in the general adult Korean population. We also evaluated HRQoL according to presence of NP in CRS patients.

**Methods:** In this cross-sectional study we used nationally representative samples from the 5th Korea National Health and Nutrition Examination Survey (2010–2012). A total of 17,490 participants were included in the study, of which 613 were diagnosed with CRS. Univariate analysis was conducted on healthy versus CRS groups, segregated by gender with weighted prevalence of demographic characteristics, socioeconomic status, and comorbid diseases. Subanalysis was carried out to evaluate the relationship between CRS with or without NP and HRQoL using EuroQol 5-Dimension (EQ-5D). The odds ratios for EQ-5D were estimated by multiple logistic regression analyses with confounder adjustment.

**Results:** Weighted prevalence of CRS of adult male participants was found to be 3.7% and CRS with nasal polyps (CRSwNP) 0.5%, while female CRS was 3.3% and CRSwNP 0.3%. There was no significant difference between the groups (P =0.3321). The scores for female, EQ-5D index (P for trend <0.0001) and EQ-VAS (P for trend =0.0024) showed decreasing trend from healthy participants to CRS without nasal polyps (CRSsNP) and from CRSsNp to CRSwNP. After adjusting for demographic characteristics, socioeconomic status, and comorbid diseases, EQ-5D scores; EQ-5D index (P <0.0001) and EQ-VAS (P <0.0001) exhibited poorer HRQoL compared to healthy participants, exclusively within the female group.

**Conclusion:** These data suggest that female patients with CRS are at higher risk of poor HRQoL. In addition, HRQoL of female CRSwNP was lower compared to those of CRSsNP and healthy participants.

## A45 House dust mite sensitization and exacerbation of asthma in the fall in children

### Jong-Seo Yoon, Jin Tack Kim, Hwan Soo Kim, Yoon Hong Chun, Hyun Hee Kim, Sul Mui Won

#### The Catholic University of Korea, South Korea

##### **Correspondence:** Jong-Seo Yoon – The Catholic University of Korea, South Korea

**Background:** Exposure and sensitization to house dust mite (HDM) allergens can trigger asthma exacerbation. The indoor concentration of HDM allergens and the prevalence of asthma exacerbation are higher in the fall than during the other seasons in South Korea.

**Methods**: The study participants were children who visited the emergency room for acute asthma exacerbation. We measured their serum levels of total immunoglobulin E (IgE), HDM-specific IgE (sIgE), cytokines, and chemokines.

**Results**: Total IgE and HDM-sIgE levels were higher in the sera from children presenting with asthma exacerbation in the fall. We found no difference in the levels of cytokines and chemokines between the fall and the other seasons.

**Conclusion**: Based on the fact that acute asthma exacerbation is most prevalent in the fall and that the indoor concentration of HDM allergens is the highest in fall, the higher levels of serum HDM-sIgE in children with asthma exacerbation presenting in the fall may suggest that HDM sensitization could be a risk factor for asthma exacerbation in the fall.

## A46 Evidence-based health advice for childhood eczema and household pets

### Kam Lun E. Hon, Chung Mo Chow, Ting Fan Leung

#### Prince of Wales Hospital, Hong Kong

##### **Correspondence:** Kam Lun E. Hon – Prince of Wales Hospital, Hong Kong

**Background:** Many parents seek healthcare advice if household pet keeping may be detrimental in atopic eczema (AE).

**Aim:** We investigated if skin sensitization by cat/dog dander was associated with disease severity and quality of life in children with eczema.

**Methods:** Demographics, skin prick test (SPT) results, disease severity (Nottingham eczema severity score NESS), Children Dermatology Life Quality Index (CDLQI), skin hydration (SH), transepidermal water loss (TEWL) of a cohort of AE patients were reviewed.

**Results:** 325 AE patients followed at a pediatric dermatology clinic were evaluated. Personal history of asthma was lowest (20%) in the dog-dander-positive-group but highest (61%) in both-cat-and-dog-dander-positive group (p=0.007). Binomial logistic regression ascertained that cat-dander sensitization was associated with increasing age (adjusted odds ratio [aOR], 1.056; 95% Confidence Interval [CI], 1.006 to 1.109; p=0.029), dust-mite sensitization (aOR, 4.625; 95% CI, 1.444 to 14.815; p=0.010), food-allergen sensitization (aOR, 2.330; 95% CI, 1.259 to 4.310; p=0.007) and keeping-cat-ever (aOR, 7.325; 95% CI, 1.193 to 44.971; p=0.032); whereas dog-dander sensitization was associated with dust-mite sensitization (aOR, 9.091; 95% CI, 1.148 to 71.980; p=0.037), food-allergen sensitization (aOR, 3.568; 95% CI, 1.341 to 9.492; p=0.011) and keeping-dog-ever (aOR, 6.809; 95% CI, 2.179 to 21.281; p=0.001). However, neither cat nor dog sensitization were associated with asthma, allergic rhinitis, parental or sibling atopic status, disease severity or quality of life.

**Conclusion:** This study summarizes evidence for parental/patient guidance. There is no direct correlation between AE severity or quality of life with skin sensitization to cats or dogs. However, furry pets are a good source of house-dust mite and it had been shown in the study that AE patients are likely to be sensitive to them. Sensitized patients especially those with concomitant asthma and severe symptoms may consider non-furry alternatives if they plan to have a pet. Highly sensitized individuals, especially those with asthma co-morbidity, may have to remove their pet for a trial period to determine if symptoms improve.

## A47 Relationship between allergic rhinitis and mental health in Korea

### Do Hyun Kim^1^, Soo Whan Kim^2^

#### ^1^The Catholic University of Korea, South Korea; ^2^College of Medicine, the Catholic University of Korea

##### **Correspondence:** Do Hyun Kim – The Catholic University of Korea, South Korea

**Background:** There has been no nationwide population-based investigation of relationship between allergic rhinitis (AR) and mental health in Korea.

**Objective:** To evaluate the association between AR and mental health status in the general adult Korean population. Also, to investigate relative burden of AR on mental health using Allergic Rhinitis and Its Impact on Asthma (ARIA) classification.

**Methods:** A cross-sectional study was performed by using data of 11,154 individuals 19 years old or older, collected through the Korean National Health and Nutrition Examination Survey from 2011 to 2012. Univariate analysis was conducted on Healthy, AR groups with weighted prevalence of demographic characteristics, socioeconomic status and comobid diseases. Subanalysis classifying AR severity according to the ARIA classification was carried out to evaluate the relationship of AR severity with mental health. The odds ratios (ORs) for each components representing mental health status were estimated by multiple logistic regression analyses with confounder adjustment.

**Results:** Univariate analysis with Chi-square test after adjusting age, sex, BMI, smoking status, alcohol use status and exercise status, components representing mental health status showed linear trend with the severity of AR according to ARIA classification. Stress, depressive mood, suicidal thought, psychological consultation factors were correlated with AR after adjusting demographic characteristics, socioeconomic status. Even after adjusting comorbid allergic diseases the correlation remained significant with stress, depressive mood, psychological consultation factors (OR [95% CI]; 1.227 [1.042, 1.445], 1.368 [1.095, 1.71], 1.804 [1.096, 2.969]).

**Conclusion:** Patients with AR appear to be at a higher risk for mental health disorders in the general adult Korean population. Moreover, persistent or severe AR was correlated with poor mental health. Therefore, better control of AR may be conducive to better mental health and more attention should be paid to the psychological status of AR patients.

## A48 Oscillometric bronchodilator response in 3 to 5 years old healthy and asthmatic Filipino children

### Gemmalyn Esguerra, Emily Resurreccion, Kristine Elisa Kionisala, Jenni Rose Dela Cruz

#### Philippine Children’s Medical Center, Philippines

##### **Correspondence:** Gemmalyn Esguerra – Philippine Children’s Medical Center, Philippines

**Background:** Assessment of respiratory function is vital in the diagnosis and monitoring of children with asthma. Measurement of response to bronchodilator (salbutamol) is ideal for children 3-5 years old because it is not effort dependent, less invasive and requires less cooperation from the patient.

**Objectives:** To compare the change in oscillometric parameters after inhalation of a beta 2- agonist among healthy and asthmatic children aged 3 to 5 years old using impulseoscillometry.

**Methods:** The respiratory impedance at baseline and after 15 minutes of one dose of Salbutamol nebulization was measured with the impulse oscillometry(IOS) using the VIASYS (Healthcare ,Leibnizstr. Hoechberg Germany)at resistance at 5Hz and 20 HZ and reactance at 5HZ in 310 children aged 3-5years old. For the calculation of threshold or cutoff values, receiver operating characteristic (ROC) curves were drawn and was determined by the Youden index (J = max{sensitivity + specificity – 1}). Partial correlation study was done among multiple parameters to determine best positive correlation for diagnosis of asthma.

**Results:**

Fifty-six (18.1%) asthmatic subjects and 254 (81.9%) healthy subjects were able to complete the study. Mean percent (standard error of the mean) baseline pre bronchodilator indices for asthma were 1.21 ± 0.02 kPa/L/s for Z5Hz; 1.15 ± 0.02 kPa/L/s for R5Hz; 0.83 ± 0.01 kPa/L/s for R20Hz and -0.37 ± 0.01 kPa/L/s for X5Hz. In normal healthy subjects, the baseline mean values were 1.09 ± 0.01 kPa/L/s for Z5Hz; 1.04 ± 0.01 kPa/L/s for R5Hz; 0.79 ± 0.01 kPa/L/s for R20 Hz and -0.31 ± 0.01 kPa/L/s for X5Hz. In mean percent change initial values of asthmatics were -29.03% ± 0.73 for Z5Hz; -28.77% ± 0.81 for R5Hz; -22.96 % ± 0.97 for R20 Hz and 36.91 % ± 1.62 for X5Hz. Cut off values for bronchodilator response in diagnosing asthma using the percent change initial were as follows: -19.98% for Z5Hz with sensitivity of 100% and specificity of 96%; -21.25% for R5Hz with sensitivity of 95% and specificity of 98%; -13.96 % for R20 Hz with sensitivity of 93% and specificity of 78% and -24.25 % for X5Hz with sensitivity of 88% and specificity of 88%. Percent initial change of Z5Hz and R5Hz (r = 0.938, p<0.001) are significantly correlated.

**Conclusion:**

Values at baseline and postbronchodilator on the different IOS parameters were significantly higher in the asthmatic group. A post-bronchodilator change of -20% in Z5Hz and -21% in R5Hz from the initial has strong positive correlation for the diagnosis of asthma. The computed percent change post-bronchodilator for Z5Hz and R5Hz are the best parameters that showed better accuracy in terms of specificity and sensitivity in the diagnosis of asthma using impulse oscillometry in our population.

## A49 The use of aeroallergen immunotherapy to treat eosinophilic esophagitis

### Muhammad Imran

#### Kansas University Medical Center, USA

**Introduction:** Eosinophilic esophagitis (EoE) is characterized by symptoms of esophageal dysfunction, eosinophilia of the esophagus, and may be associated with other atopic disorders. The pathogenesis of EoE is likely a mixed IgE and non-IgE food-mediated reaction with Th2 cytokines driving esophageal eosinophilia. Hence, therapy is aimed at inflammation control, with corticosteroids (oral or topical) and/or food antigen avoidance. However, these treatments are not specific, impair quality of life, and have significant side effects. Therefore, there is an ongoing effort to design alternative therapies.

**Background:** To determine if EoE patients with concomitant seasonal or perennial allergic rhinitis improve with aeroallergen immunotherapy (IT).

**Methods:** We present a case series of 3 Caucasian patients with a history of atopy and EoE based on characteristic clinical symptoms, EGD findings, and esophageal biopsy. One had oral allergy syndrome. None of the patients had peripheral eosinophilia. Two had seasonal exacerbations of their EoE symptoms. These patients were all started on aeroallergen immunotherapy for atopic disease other than EoE.

**Results:** In this small group of aeroallergen and food sensitized adult patients, we demonstrated a lack of improvement in clinical symptoms of EoE with aeroallergen immunotherapy while making no modifications in diet or oral medications. It is difficult to ascertain improvement due to the lack of a validated adult symptom score and inherent restraints of repeat esophageal endoscopy due to cost and procedural risks. It is also unclear if the biopsy always accurately reflects disease activity. There are several limitation of this small sample which includes absence of a control group, no statistical validity, and possible selection bias.

**Conclusions:** We would like to continue further evaluation of the prevalence of aeroallergen sensitized adults with EoE and will consider a similar evaluation of pediatric patients. We will review further data at an attempt to isolate a population in which improvement with aeroallergen immunotherapy may be successful in improving control of EoE without aggressive dietary modifications or medication management.

## A50 A study of the eczema herpeticum in Korean

### Yun Seon Choe, Kyu Han Kim, Mira Choi

#### Seoul National University Hospital, South Korea

##### **Correspondence:** Yun Seon Choe – Seoul National University Hospital, Seoul, South Korea

**Background**

Eczema herpeticum (EH) is a widespread herpes simplex virus infection, most often presenting as complication of atopic dermatitis (AD). Along with increased incidence of AD, the number of patients of EH seems to be increased over the years. However, population-based epidemiologic data on EH are insufficient because of its rarity. We aimed to evaluate the epidemiologic trends of EH in Korean during last 10 years, comparing those with prevalence of AD.

**Methods**

A retrospective analysis was carried out for patients diagnosed with EH between January 2005 and December 2014 at a single tertiary hospital in Korea. Variables of interest included age, sex, season of onset, treatment, outcomes, recurrence, and whether they had underlying AD or not. The prevalence of AD at the same period was investigated by the years.

**Results**

Total 1,043 episodes of EH in 621 patients were diagnosed during ten years. The number of EH episodes per patient was 1.68 (range 1 - 6), with more than one episode in 288 patients. Mean age at onset was 23.4 years, and sex ratio was M:F = 1:1.4. The patients who had recurrent episodes tend to be older (25.9 years) than those with EH episode only once (21.3 years), and there was no significant difference of sex ratio between two groups. Seasonal variation of onset was not found. Duration of treatment was 7.8 days, and most of treatment (93.7%) included systemic antiviral agent. Eleven patients were hospitalized and improved by intravenous antiviral agent, but six of them showed recurrence after discharge. Majority of patients with EH had underlying AD except only four patients. At the same period, total 33,692 patients were diagnosed as AD in our clinic, with continuous increase of prevalence annually. The ratio of EH/AD was 1.83% in average, and it was steadily decreased by years: 2.52% in 2005, 1.36% in 2014. EH was more commonly complicated in old age, showing significantly increased EH/AD ratio above 30 years old.

**Conclusions**

EH is not a rare complication of AD, affecting about 2% of AD patients. While the prevalence of AD is increased over the years, total number of EH patients was little changed, leading to slight but continuous decline of EH/AD ratio. As patients getting older, EH could be more commonly complicated in AD, and show more recurrent course than in younger patients.

## A51 Specific sublingual immunotherapy in Korean patients with atopic dermatitis

### Byung Soo Kim^1^, Hyun-Joo Lee^1^, Jeong-Min Kim^1^, Jeong-Min Kim^2^, Gun-Wook Kim^1^, Je-Ho Mun^1^, Je-Ho Mun^2^, Hoon-Soo Kim^1^, Margaret Song^1^, Hyun-Chang Ko^1^, Hyun-Chang Ko^2^, Moon-Bum Kim^1^

#### ^1^Pusan National University, South Korea; ^2^Pusan National University Yangsan Hospital

##### **Correspondence:** Byung Soo Kim – Pusan National University, South Korea

**Background:** Sublingual immunotherapy (SLIT) with house dust mites (HDM) preparation has recently been proven to be beneficial for treating allergic rhinitis and asthma. However, there has been no report regarding the efficacy and safety of SLIT in Korean patients with atopic dermatitis (AD).

**Objective:** To investigate the efficacy and safety of SLIT in Korean patients with AD.

**Methods:** A total of 34 patients with AD and IgE-proven HDM sensitization (Class ≥ 3) were recruited from Pusan National University Hospital between July 2011 and September 2014. Patients were treated with SLIT for at least 12 months. Eczema area and severity index (EASI) score, total serum IgE level, results of specific IgE assays to *Dermatophagoides pteronyssinus* and *D. farinae*, and adverse effects were recorded at each scheduled visit. “Responder” was defined as a patient with ≥ 30% improvement in EASI score after SLIT.

**Results:** Twenty-three patients continued SLIT for 12 months or more, and 11 patients (32.4%) dropped out because of exacerbation of dermatitis or were lost to follow-up. The average duration of SLIT treatment was 22.4 months (range, 12–32 months). EASI scores reduced significantly after 12 months of treatment (*p* < 0.001) compared with those at baseline. A total of 19 patients (19/23; 82.6%) were determined to be responders to SLIT after 12 months. Total and specific IgE serum levels did not significantly reduce after SLIT. No patients experienced serious adverse events, with the exception of two patients who developed transient lip and tongue swelling.

**Conclusion:** Our study demonstrated that SLIT with HDM extracts is effective and tolerable in Korean patients with AD. Further controlled long-term trials are required to reinforce the current results.

## A52 Association between polymorphisms in bitter taste receptors genes and clinical features in Korean asthmatics

### Sun-Young Yoon

#### Asan Medical Center, University of Ulsan College of Medicine, South Korea

**Background:** Bitter taste receptors (TAS2R) in human airway smooth muscle have been recently shown to have an important role in bronchodilation, together with β2-adrenergic receptors.

**Object:** To evaluate the association between genetic variations in TAS2R and clinical features, including bronchodilator response and asthma control.

**Method:** We analysed the association between single nucleotide polymorphisms (SNPs) of TAS2R10 and TAS2R14 and variables including demographic data, atopy, duration of disease, asthma control status, including variables such as asthma control test (ACT) score, percent predicted value of FEV_1_, forced vital capacity (FVC), and FEV_1_/FVC ratio, and bronchodilator response (BDR), in 721 asthma patients in Korea.

**Result:** Three novel SNPs of M207I, H203Q, and -79G/A in TAS2R10 and three known SNPs of -815C/T, -1267A/G, and -1897C/T in TAS2R14 were analysed. Increased BDR was significantly associated with SNP of -815T>C [OR (95% confidence interval (CI)) = 1.88 (1.01 – 3.49), p=0.04], -1267 A>G [OR (95% CI) = 2.07 (1.03 – 4.15), p=0.04] and -1897C>T [OR (95% CI) = 3.05 (1.01 – 9.23), p=0.04, and OR=1.91 (1.08−3.36), p=0.02] of the TAS2R14 gene. There was a significant association between -815 T>C and low mean ACT score [OR (95% CI) = 5.84 (1.94 – 17.61), p=0.001]. In haplotype analysis, TGT, CAT, and TGT, or TG and CA haplotypes on TAS2R14 were significantly associated with increased BDR; CAT and CA haplotypes were significantly associated with low ACT score.

**Conclusion:** Genetic variations in TAS2R may be valuable genetic markers to predict therapeutic response and outcomes in asthma, although we had not sufficiently clarified the relationship between genetic polymorphism in TAS2R genes and the response to ß2-adrenoreceptor agonists. Further research in an independent cohort is needed.

## A53 Effect of glycosides based standardized fenugreek seed extract in bleomycin-induced pulmonary fibrosis in rats

### Amit Kandhare

#### Bharati Vidyapeeth Deemed University, India

**Abstract**

***Background*:** Idiopathic pulmonary fibrosis (IPF) is a chronic progressive multifactorial disease with limited treatment options.

***Aim*:** To evaluate the efficacy of glycosides based standardized fenugreek seed extract (SFSE-G) on behavioral, biochemical, molecular and ultrastructural changes in bleomycin (BLM) induced pulmonary fibrosis in the laboratory rats.

***Materials and Method*:** IPF was induced in male Sprague-Dawley rats by single intratracheal BLM (6 IU/kg) injection followed by SFSE-G (5, 10, 20 and 40 mg/kg, p.o.) or Methylprednisolone (10 mg/kg, p.o.) treatment for 28 day. Sham control rats received saline instead of BLM. The lung function test, biochemical, molecular, histopathological and ultrastructural changes were analyzed in lung and bronchoalveolar lavage fluid (BALF) after 14 and 28 days after the drug treatment.

***Results*:** Treatment with SFSE-G significantly restored the BLM induced alteration in body weight, lung index, lung function test and hematology. SFSE-G treatment significantly restored the altered total and differential cell count in BALF and blood. The BLM induced peripheral blood oxygen content reduction was significantly reversed by SFSE-G treatment. SFSE-G significantly enhanced the BALF and lung antioxidant status, through modulating the SOD, GSH, T-AOC, MDA, NO level and Nrf2, HO-1 mRNA expression. There was a significant reduction in lung 5-HT level by SFSE-G treatment. The altered mRNA expression of lung inflammatory markers (TNF-α, IL-1β, IL-6 and IL-8), fibrotic markers (TGF-β, collagen-1, ET-1, Muc5ac, NF-kB, VEGF, Smad-3) and apoptotic markers (Bax, Bcl-2 and Caspase-3) were significantly restored by SFSE-G treatment. BLM induced histological inflammatory, and fibrotic and ultrastructural changes of lungs were reversed by SFSE-G treatment.

***Conclusion*:** SFSE-G has potential efficacy against the fibrotic pathogenesis of BLM induced pulmonary fibrosis probably through anti-inflammatory and anti-apoptotic pathways.

**References**

1. Bei Y, Hua-Huy T, Duong-Quy S, Nguyen VH, Chen W, Nicco C, *et al*. Long-term treatment with fasudil improves bleomycin-induced pulmonary fibrosis and pulmonary hypertension via inhibition of Smad2/3phosphorylation. *Pulmonary Pharmacol Therap* 2013; 26: 635-643.

2. Chitra P, Saiprasad G, Manikandan R, Sudhandiran G. Berberine attenuates bleomycin-induced pulmonary toxicity and fibrosis via suppressing NF-κB dependant TGF-β activation: A biphasic experimental study. *Toxicol Lett* 2013; 219: 178-193.

## A54 A kampo formula, ogi-kenchu-to, decreases side-effects of steroid ointment for infantile atopic dermatitis: Three cases report

### Noriko Yahiro

#### Yashiro Dermatology Clinic, Japan

Ogi-kenchu-to is one of the traditional Japanese medical system called *“Kampo”* medicine formulae. It has been reported that Ogi-kenchu-to shows dominant effects of parasympathetic nerve systems, and clinically used as anti-fatigue effect, immune-activation, and relaxation of peripheral capillaries. Here, I report three infant cases of severe atopic dermatitis with impoverish skin which shows a therapeutically promising efficacy by the treatment of Ogi-kenchu-to.

Three infants visited my clinic for the treatment of atopic dermatitis. All three infants showed full body atopic skin with high level of IgE and TARC in serum. Two of three cases had erythroderma, and the other one had persistent airway inflammation. Sufficient volume of steroid ointment treatment was necessary for all three patients to prevent atopic march. However, impoverish skin was found in all cases at initial administration because of long-term insufficient management. Since the impoverish skin is caused by chronic inflammation under the continuous activation of eosinophil which controlled by dominant effect of sympathetic nerve systems, Ogi-kenchu-to was an administrated for tree patients in combination with sufficient steroid ointment treatment. After 3-6 months later, both impoverish skin and atopic dermatitis were improved, and the serum level of IgE and TARC was decreased, which resulted in the decrease of the steroid ointment dose.

It has been difficult to continue steroid ointment treatment only for atopic infants with the impoverish skin. My cases indicated that Ogi-Kenchu-to might play an important role for the treatment of impoverish skin in patients with intractable atopic dermatitis. Although the efficacy of kampo medicine has not been clarified enough, several immune-pharmacological studies reported that components related Kampo formulae including Ogi-Kenchu-to might effect on the anti-allergic actions. In conclusion, the therapy in combination with Kampo formulae might achieve a better response for atopic dermatitis with impoverish skin, thereby should be extended to common use as complementary medicine.

## A55 To test use of jet nebulizers NE-C802 as a drug delivery system in the children with asthma

### Amit Agarwal^1^, Meenu Singh^2^, Jasleen Kaur^2^, Ruby Pawankar^3^, Pankaj Pant^2^, Sukhmanjeet Singh^2^

#### ^1^Postgraduate Institute of Medical Education and Research, Japan; ^2^Postgraduate Institute of Medical Education and Research; ^3^Nippon Medical School

##### **Correspondence:** Amit Agarwal – Postgraduate Institute of Medical Education and Research, India

**Background:** Asthma is a chronic inflammatory disorder of the airway characterized by recurrent episodes of wheezing, breathlessness, chest tightness and coughing, particularly at night or early in the morning. These episodes are usually associated with variable airflow obstruction within the lungs that is often reversible either spontaneously or with treatment.

**Methods:** Two hundred patients were initially screened in the outpatient clinic, Department of the Pediatrics, Post Graduate Institute of Medical Education and Research, Chandigarh. Out of the 200 patients, 30 were selected. Informed consent of one of their parents was obtained prior to enrolment into the study. Included patients were also be on daily Budesonide therapy for asthma control and Living in and around Chandigarh. The patients were then assessed every two weeks for symptomatic control of asthma and the participants and their parents were explained and demonstrated about the functioning of the nebulizers**.** PEFR, Height and Weight measurements were taken at every visit when nebulizer was used. Patients were assessed for improvement or deterioration of symptoms. The study was approved by Institute Ethics Commitee (PGI/IEC/2014/2337).

**Results:** Seventy percent patients/parents/guardians preferred using nebulizer for one of the following reasons: Better symptom control, Decreased frequency of exacerbations or Management of exacerbations can be done at home by increasing the duration of drug delivery through the nebulizer.

Thirty percent patients/parents/guardians preferred using spacer because they felt that their children could use a spacer easily as compared to nebulizer.

**Conclusion:** Thirty patients completed eight weeks of inhalation therapy with NE-C802 nebulizers. There were no significant deteriorations in symptoms during the therapy. NE-C802 nebulizer preferred over the use of spacer as a drug delivery system in control the symptoms of Asthma.

## A56 Immunoglobulin e to allergen components of house dust mite in Korean children with allergic disease

### Hwan Soo Kim, Jong-Seo Yoon, Sul Mui Won, Yoon Hong Chun, Jin Tack Kim, Hyun Hee Kim

#### The Catholic University of Korea, South Korea

##### **Correspondence:** Hwan Soo Kim – The Catholic University of Korea, South Korea

**Background**

House-dust mites (HDMs) are important sources of indoor allergens. Our aim was to define the sensitization rate of specific immunoglobulin E (IgE) in Der p and its components and investigate their clinical features in children with allergic disease.

**Methods**

We performed a prospective evaluation of 80 HDM sensitized patients with histories of allergic rhinitis (AR), atopic dermatitis (AD), asthma and urticaria (UC). The patients underwent an allergen identification test using ImmunoCAP for total IgE, Der p, Der f, Der p 1, Der p 2, and Der p 10.

**Results**

Seventy-nine patients had detectable serum levels of Der p specific IgE, and 80, 66, 63, and 7 patients were sensitized to Der f, Der p 1, Der p 2, and Der p 10, respectively. The level of Der p 1-specific IgE was significantly lower in the UC group than in the AD and AR groups. Total IgE level was significantly higher in the Der p 10-sensitized group. Der p 10 serum IgE level was highly correlated with crab and shrimp specific IgE. A significant positive correlation was observed between total IgE and specific IgE to Der p and its components and Der f.

**Conclusions**

Sensitization to HDM and its components in our study is similar to that reported in previous studies from temperate climate. There was a significant difference in Der p 1 in patients with UC, AD and AR.

## A57 Effectiveness of premedication and rapid desensitization in hypersensitivity to l-asparaginase

### Hwan Soo Kim, Sul Mui Won, Yoon Hong Chun, Jong-Seo Yoon, Hyun Hee Kim, Jin Tack Kim

#### The Catholic University of Korea, South Korea

##### **Correspondence:** Hwan Soo Kim – The Catholic University of Korea, South Korea

**Background**

L-asparaginase is a crucial chemotherapeutic agent for the treatment of acute lymphoblastic leukemia. However, hypersensitivity to L-asparaginase is common which limits its usage. We aimed to evaluate the usefulness of premedication and desensitization in L-asparaginase hypersensitivity.

**Methods**

We performed 44 cases premedication and 3 cases of desensitization in 16 patients with hypersensitivity to L-asparaginase.

**Results**

With premedication, 33 cases completed L-asparaginase injection with no hypersensitivity reaction. 11 cases showed mild hypersensitivity reaction, such as urticaria. Desensitization was performed in 3 cases: 2 cases were successful and in 1 case, medication was switched to *Erwinia* asparaginase.

**Conclusion**

Premedication and desensitization appears to be useful in helping patients receive desired dose of L-asparaginase in pediatric patients with acute lymphoblastic leukemia.

## A58 Angioedema with Eosinophilia: The First Report from Thailand

### Thatchai Kampitak

#### Samitivej Sukhumvit Hospital, Thailand

**Background:** Angioedema with eosinophilia (AE) is an uncommon form of angioedema. Although its pathogenesis remains unclear, it can be classified into episodic angioedema with eosinophilia (EAE) and nonepisodic angioedema with eosinophilia (NEAE). While EAE has been generally observed in the Western populations, almost all patients with NEAE were Asian, exclusively Japanese and Korean. Previously, AE has never been reported in Thai patients.

**Methods:** A case series of 3 patients with AE from Thailand is described.

**Results:** Three Thai patients with AE were identified during April 2014-March 2015. All but 1 patient were female. The mean age at the onset of symptoms was 32 years (27-39). All patients presented with symmetrical swelling of distal extremities without systemic symptoms. No fever or weight gain was observed. Arthralgia and urticaria were present in 1 patient, respectively. No potential triggers were identified except 1 patient had preceding upper respiratory tract infection 1 week prior to the swelling. Previous history of significant medical or allergic diseases was unremarkable in all except 1 patient was hepatitis B carrier and had recurrent eczema. All patients had eosinophilia with the mean eosinophil count of 4,083/mm^3^ (1,006-9,420). Blood chemistries, liver and kidney function tests, inflammatory markers (ESR and CRP) and immunoglobulin levels were within the normal ranges in all patients. Secondary disorders that might be responsible for eosinophilia were excluded. Recurrent episodes of angioedema were observed in 1 patient. Antihistamine was briefly prescribed in 1 patient with coexisting urticaria. All patients had complete spontaneous resolution of symptoms in parallel with the normalization of eosinophil count within few months after the presentation.

**Conclusions:** AE should be considered in young Asian women whose presenting features include peripheral swelling and eosinophilia without constitutional symptoms, internal organ involvement and elevated immunoglobulin levels. Similar to previous reports from Japan and Korea, NEAE is more prevalent in Thai patients than EAE. No corticosteroid therapy is generally required since most patients have a self-limiting disease.

**Keywords:** Hypereosinophilic Syndromes; Angioedema

## A59 Evaluation of anti-pruritic and anti-inflammatory effects of Korean red ginseng extract on atopic dermatitis murine model

### So Min Kim, Hyun Joo Lee, Hei Sung Kim, Jeong Deuk Lee, Sang Hyun Cho

#### Incheon St. Mary’s Hospital, South Korea

##### **Correspondence:** So Min Kim – Incheon St. Mary’s Hospital, South Korea

**Background:** Korean red ginseng (KRG) and ginsenoides have showed several biologic effects in various field including anti-inflammatory and anti-allergic effects. We aimed to investigate the anti-inflammatory and anti-pruritic effects of KRG on atopic dermatitis murine model.

**Material and methods:** The atopic dermatitis-like skin lesions were induced by percutaneous challenge of 2,4,6-trinitro-1-chrolobenzene (TNCB) on the ear and back of NC/Nga mice. The KRG extract, evening primrose oil, cyclosporine and phosphate-buffed saline were administered orally by gastric tube. The effects of KRG and other drugs were assessed by measuring the clinical severity score, ear thickness, transepidermal water loss (TEWL), the number of scratching counts, total systemic IgE and IL-31 by ELISA, histologic changes of skin, and mRNA expression of TNF-α, IFN-γ, TSLP and IL-31. Each study group was divided into scratch-able and non-scratch-able subgroups to evaluate the impact of scratching behavior in atopic dermatitis.

**Results:** Oral administration of KRG significantly reduced clinical severity, ear thickness, and TEWL elevation. The number of scratching counts was also effectively lowered in KRG administered group compared to other treatment groups. The clinical results were also revealed in serologic and histologic changes. In the KRG group, the systemic IgE and IL-31 level was significantly lowered on the last day of the experiment. Histologically, epidermal hyperkeratosis, parakeratosis, and hyperplasia and dermal leukocytes and mast cell infiltration were suppressed by KRG. Immunochemistry of TNF-α, TSLP and IL-31 expression and quantitative RT-PCR showed that KRG effectively suppressed proinflammatory cytokines and Th2 response. Additionally, proactive restriction of scratching behavior by physical barrier reduced scratching counts and this improved clinical symptoms. Also, in non-scratch-able group there was lower inflammatory cell infiltration and lower TNF- α, TSLP, Il-31, and IFN-γ expression in the back tissue as well as lower systemic IL-31 level.

**Conclusion:** Therefore, we expect that the oral administration of KRG may control pruritus and skin inflammation by inhibiting the Th2 response. In addition, restriction of scratching behavior in early stage could be helpful in suppressing the itch-scratch vicious cycle and improving the clinical and systemic inflammations.

## A60 Subcutaneous autologous serum therapy in chronic urticaria

### Kiran Godse

#### D.Y.Patil Medical College and Hospital, India

Chronic Spontaneous urticaria (CSU) is a vexing problem are also subjected to a huge antihistamine pill burden.

The symptoms are more in autoreactive urticaria (AU) where auto-antibodies in blood flares-up the condition. Search for newer effective modalities which can reduce pill burden is a felt need.

URTICRIA is one of the most challenging therapeutic problems faced by a dermatologists.

Auto serum therapy is a Therapy in which repeated injections of autologous serum are administered subcutaneously.

Complete absorption is possible in subcutaneous autoseru, therapy.This study evaluates the effectiveness of subcutaneous autologous serum therapy (AST) in CSU and also determines its usefulness in Autoreactive Urticaria.

• Single blind, Placebo controlled

•parallel group, randomized, controlled study. 24 patients (11M: 13 F) were given subcutaneous AST and

• 17 (7 M:10F) patients were given subcutaneous injection normal saline (placebo), along with levocetirizine in an on-demand basis in both groups.

• Age group 19-54 years Mean age 29.7yrs

• Duration of urticaria 6 months to 80 months

• Associated conditions:

–Eosinophilia - 5 patients

–Hypothyroidism - 4 patients

–Microcyctic Hypochromic anemia - 2 patients

–Autologous serum skin test +ve

–11/24 serum group

–7/17 saline group

Urticaria activity score (UAS) came down form average 35.74 to 7 at the end of 9 weeks.Saline group did not show reduction in UAS. Daily requirement of antihistamines also came down in serum group.

AST is a useful adjunct to antihistamines in CU. The effect persisted even four months after cessation of therapy and thus improves the quality of life.

This therapy could be useful in India as cost effective & beneficial (a poor man’s biologic!) for chronic urticaria patients.

## A61 Allergic bronchopulmonary aspergillosis in asthma and lung tuberculosis

### Juwita Soekarno^1^, Sarie Ratnasari^2^, E. Alwi Datau^2^, Eko Surachmanto^2^, JC Matheos^2^

#### ^1^Medical Faculty of Univesity of Sam Ratulangi, Indonesia; ^2^Samratulangi University Manado, Indonesia

##### **Correspondence:** Juwita Soekarno – Medical Faculty of Univesity of Sam Ratulangi, Indonesia

**Background:** Allergic bronchopulmonary aspergillosis (ABPA) is a lung disease that is caused by hypersensitivity reaction to the fungus Aspergillus fumigatus, where its colonization often found in chronic respiratory disease, either in bronchial asthma and lung tuberculosis. History of asthma in ABPA is often obtained from 5-10 years earlier. Lung tuberculosis is often difficult to distinguish with ABPA due to similar radiological features, often found as misdiagnosis in a previous study.

**Methods:** Observational descriptive analytic with cross sectional approach, conducted in Prof. Dr.R.D. Kandou Hospital in Manado, from July-December 2013. Total 75 samples from 3 groups, each 25 samples: moderate-severe persistent asthma, Acid-Fast Bacilli (AFB) positive lung TB and AFB negative lung TB. Chest x-ray examination, skin prick test (SPT) using A. fumigatus antigen (rapid reaction/type I early and late/8 hours type III), total IgE, and IgG anti A. fumigatus are obtained. Greenberger assessment criterias are used for ABPA.

**Results:** ABPA was found 1.55% (4/258) in moderate-persistent asthma, 0.64% (3/469) in FAB positive lung TB and 0.57% (6/1054) in AFB negative lung TB. Correlation between type I SPT and total IgE showed significant results (p<0.05) in moderate-severe persistent asthma and AFB negative lung TB, but is not significant in AFB positive lung TB, while the correlation between IgG A. fumigatus and type III SPT has very significant (p <0.001) results in all groups; 

**Conclusion:** ABPA is found either in asthma or lung tuberculosis patients. Significant correlation between IgG A.fumigatus and type III SPT may indicate SPT to replace IgG A. fumigatus examination which is currently a research kit. SPT examination may be considered as screening of suspected ABPA.

## A62 Infantile eczema is associated with campylobacter and roseburia subpopulations but not microbial diversity in stool samples of Chinese newborns

### Ting Fan Leung^1^, Jamie Sui-Lam Kwok^2^, Christine Kit-Ching Tung^2^, Man Fung Tang^1^, Stephen Kwok-Wing Tsui^2^, Gary WK Wong^1^, Kam Lun Ellis Hon^1^, Wing Hung Tam^1^, Hing Yee Sy^1^

#### ^1^Prince of Wales Hospital, Hong Kong; ^2^The Chinese University of Hong Kong

##### **Correspondence:** Ting Fan Leung – Prince of Wales Hospital, Hong Kong

**Background:** Gut microbiota is increasingly recognised to play crucial roles in the pathogenesis of asthma, obesity and autoimmune diseases. Faecal microbiome is likely ethnic and diet-specific, but such data is lacking in Asians. This study characterised faecal microbial compositions of Hong Kong Chinese infants.

**Methods:** Random stool samples were obtained from 4-week-old infants with eczema (n=15) and without any allergy (n=15) at 9 months. Genomic DNA extracted by PowerSoil DNA Isolation Kit (MO BIO Laboratories) was sequenced using Ion PGM Seqeuncing 200 Kit v2, Ion 318 TM Chip v2 on Ion PGM System (Ion Torrent). Reads from each patient were filtered for low quality (Phred <20). Microbial diversity was evaluated using Shannon-Weaver diversity index in Swedish (J Allergy Clin Immunol 2012;129:434-40). The taxonomic classification of the reads was assigned by BLASTn.

**Results:** 5 controls had insufficient DNA for sequencing. No significant association was detected between eczema and any bacteria with ≥1% relative abundance, including *Bacteroides*, *Escherichia*, *Klebsiella, Bifidobacterium*, *Streptococcus* and *Lactobacillus*. Among the less abundant genera (relative abundance <1%), *Campylobacter* was more abundant in cases (median 0.008%, IQR 0.003-0.022%) than controls (median 0.001%, IQR 0.001-0.004%) while *Roseburia* was less abundant in eczema (median 0%, IQR 0-0.063%) than controls (median 0.055%, IQR 0.002-0.270%). Nonetheless, Shannon-Weaver diversity index of stool microbiota at 4 weeks was similar between infants with eczema and non-allergic controls at 9 months (median [IQR]: 1.28 [0.94-1.93] versus 1.47 [1.31-1.80]; *P* = 0.698). Comparing microbial compositions in our newborns and Swedish, *Escherichia coli* was found among top 5 genera only in both our cases and controls whereas enterobacter only in Swedish newborns. Clostridium, parabacteroides and lactobacillus were found only in Chinese eczema and healthy Swedish newborns.

**Conclusions:***Campylobacter* and *Roseburia* appear to be less frequently detected in stool of 4-week-old Chinese infants who subsequently develop eczema. Microbial diversity is not associated with eczema susceptibility. This study confirms ethnic-specific early-life faecal microbial compositions.

**Funding:** Research Committee Group Research Scheme (3110087) and Direct Grant for Research (2013.2.033), CUHK

## A63 Association between serum chitinase level and toll-like receptor polymorphisms in bakery workers

### Sohee Lee

#### Ajou University School of Medicine, South Korea

**Background and objectives:** Bakery workers are exposed to wheat allergens, bacterial endotoxins and fungus, which interact to induce allergic responses and work-related respiratory symptoms (WRSs). Our previous studies demonstrated that the WRSs of bakery workers were associated with *TLR4* polymorphisms as well as Th2 immune responses, indicating a possible involvement of innate immune responses in the pathogenic mechanisms of baker’s asthma. We hypothesized that Chitinase in wheat flour may involve in the development of WRS in bakery workers. We measured serum Chitinase level in bakery workers and analyzed associations with TLR4 polymorphisms in a single cohort of bakery workers.

**Methods:** Three hundred eighty three bakery workers and as controls, 106 unexposed healthy subjects were enrolled. WRSs were evaluated using a questionnaire survey. Serum levels of Chitinase, IL-18, MPO and specific IgE/IgG antibodies to wheat flour extracts were measured by ELISA. The promoter polymorphisms of *TLR4* at -2027AG and -1608TC were genotyped.

**Results:** Serum Chitinase levels were significantly higher in bakery workers than in unexposed controls (*P=0.026*), however, no significant differences were noted according to the presence of WRSs and the prevalence of serum specific IgE or IgG antibodies to wheat flour (*P>0.05,* respectively). The workers carrying *TLR4* -2027GG had significantly higher Chitinase levels than those with *TLR4* -2027 AA/GG (*P=0.021*). Haplotype analysis indicated that the workers with ht1 [AT] had significantly higher Chitinase level that those without it (*P=0.22*). A significant correlation was found between serum Chitinase and IL-18 level (*P=0.021*), while no significant correlation was found with serum MPO level.

**Conclusions:** These findings suggest that Chitinase may contribute to develop WRSs in bakery workers through the modulation of *TLR4* function.

## A64 IFN-gamma contributes to nasal polypogenesis by inducing epithelial-to-mesenchymal transition via non-smad pathway

### Hyun-Woo Shin^1,2^, Mingyu Lee^1^, Dae Woo Kim^3^, Roza Khalmuratova^2^

#### ^1^Seoul National University College of Medicine; ^2^Seoul National University Hospital, South Korea; ^3^Boramae Medical Center

##### **Correspondence:** Hyun-Woo Shin – Seoul National University College of Medicine

**Purpose**: Nasal polyps (NP) imply a refractory clinical course in a case of chronic rhinosinusitis (CRS). Although, numerous etiologic factors including allergy, infection and hypoxia associate with nasal polyps (NP), the mechanism underlying NP is not fully understood. Previously, we reported that hypoxia-induced epithelial-to-mesenchymal transition (EMT) is frequently observed in Asian NPs, and HIF-1α could be a therapeutic target for nasal polyposis (Ref 1). However, the nasal epithelial cells in NP patients are not only exposed to hypoxia but also to diverse types or combinations of inflammatory milieu. So we hypothesized that specific immunologic endotypes could induce or accelerate EMT in nasal epithelial cells, and lead to nasal polyp formation.

**Methods**: Human nasal epithelial cells (hNEC), RPMI2650 and A549 cell lines were used. Immunoblotting, immunofluorescence and immunohistochemistry were done to evaluate EMT markers and signaling molecules in vitro and in sinonasal tissues from CRS patients with or without NP. Boyden transwell system was utilized to measure the capacity of migration.

**Results**: Four different cytokines, IL-5, IL-17, TNF-α and IFN-γ, were treated to both hNEC and RPMI2650 cells respectively, and EMT markers were traced. Among them, IFN-γ could most induce EMT, which was confirmed by the spindle-shape of cell morphology, modest cytoskeleton rearrangement, increased migration potential and EMT marker changes. Mechanistically, IFN-γ-induced EMT via ERK and p38 pathway, which were known as non-smad pathway of EMT. Next, we investigated whether p38 and ERK inhibitors could prevent EMT phenomenon. Actually, both p38 inhibitor (SB203580) and ERK inhibitor (PD98059) suppressed IFN-γ-driven EMT. Finally, we checked IFN-γ and EMT marker levels in human nasal mucosa tissues. IFN-γ expression was upregulated in NP mucosa compared with tissues of control and CRS only patients. In addition, this IFN-γ expression was found to correlate with E-cadherin (an epithelial marker) loss and α-smooth muscle actin (a mesenchymal marker) expression.

**Conclusion**: IFN-γ induce EMT in hNEC and this process is critically mediated by ERK and p38 pathway. This study shows that IFN-γ-induced EMT is likely to contribute to nasal polyposis in CRS, and suggests that p38 and ERK inhibitors be viewed as a therapeutic target for nasal polyposis.

**Reference**

Ref. 1. Shin HW et al., Hypoxia-inducible Factor 1 Mediates Nasal Polypogenesis by Inducing epithelial-to-Mesenchymal Transition. Amer J Resp Crit Care Med, 2012, p944-954.

## A65 Management and education status of anaphylaxis patients who visit our emergency room (ER)

### Mi Yeoung Kim^1^, Jaewon Jeong^2^, Chansun Park^3^

#### ^1^Busan Paik Hospital, Inje University College of Medicine, South Korea; ^2^Ilsan Paik Hospital, Inje University College of Medicine; ^3^Haeundae Paik Hospital, Inje University College of Medicine

##### **Correspondence:** Mi Yeoung Kim – Busan Paik Hospital, Inje University College of Medicine, South Korea

**Background**

We evaluate the relevance of management and education to anaphylaxis patients and emphasize the importance of education and understanding the disease.

**Method**

One hundred and ninety five patients who visited ER were enrolled from three hospitals. We analyzed clinical features, prior history of anaphylaxis, management and education. For analyzing associated factors with injection of epinephrine, Pearson chi-square test was used by SPSS version 21.

**Results**

Ninety nine (51%) patient visited ER by oneself. Time latency from symptom onset to ER visit was 82±161.5 min. Drug (56.2%) was the most frequency suspicious cause of anaphylaxis. Cutaneous (88.7%) and respiratory (72.7%) symptoms were frequent. Hypotension was presented in 114 (58.8%) patients. 47 (24.2%) patients had history of anaphylaxis and 33 patients had same suspicious cause with current anaphylaxis. Mean observation time in ER was 12±25.7 hrs. Epinephrine was injected in 114 (62%) patients. In 68 patients, epinephrine were injected via muscle with mean dose of 0.3±0.10mg. Associated factor with injection of epinephrine in patients of anaphylaxis was hypotension (p value=0.000). Twenty four patients needed to hospitalize to ICU or ward. Auto-injective epinephrine were prescribed only in 5 patents and just 34 (19%) patients were consulted to allergist in ER and 72 (40%) patients were consulted to outpatient department of allergy.

**Conclusions**

We suggested that management and education of anaphylaxis were not fully carried out in ER. For avoidence of re-experience of anaphylaxis and the education of action plan in emergency state, it is necessary to consult to allergists.

## A66 Hypoallergen-Encoding DNA Plasmids As Immunoprophylactic Vaccines of Shrimp Tropomyosin Hypersensitivity

### Christine Yee Yan Wai^2^, Patrick S.C. Leung^3^, Nicki Y.H. Leung^1^, Ka Hou Chu^1^

#### ^1^The Chinese University of Hong Kong, Hong Kong; ^2^The Chinese University of Hong Kong; ^3^University of California, Davis

##### **Correspondence:** Christine Yee Yan Wai – The Chinese University of Hong Kong, Hong Kong

**Background:** Shellfish is the second most common food allergen that causes sensitization in children under 6 years old. Despite its prevalence, clinical management of shellfish allergy is limited to conventional approaches and avoidance. Although tropomyosin has been identified as the major shellfish allergen, no allergen specific immunotherapy (SIT) currently exists to treat or prevent shellfish allergies. To investigate the possibility of DNA vaccines, we constructed two hypoallergens of the shrimp tropomyosin Met e 1: MEM49 and MED171 (Wai et al. 2014 *PLoS One* 9: e111649) and expressed them in plasmid pCI-Neo. Here we used an established mouse model of shrimp hypersensitivity to examine the immunoprophylactic potential of these two hypoallergen-based DNA vaccines.

**Methods:** 3-4 week old Balb/c mice were randomly divided into five groups (n = 8 per group). Two groups were intradermally injected with 100 μg pCI-Neo clones of MEM49 or MED171 thrice at weekly interval. The remaining groups were injected with naked pCI-Neo or PBS and served as vector, PBS or negative controls. One week after the last injection, all groups (except the negative control mice which were injected with PBS throughout the experiment) were sensitized subcutaneously with Met e 1 adsorbed to Freund’s complete and incomplete adjuvant and then orally challenged using a high dose of Met e 1. Blood, spleen and small intestine were collected for antibody, cytokine, gene expression and histological analysis.

**Results:** The PBS and vector control groups displayed typical Th2 responses upon Met e 1 challenge. These mice exhibited high levels of specific IgE and Th2-linked cytokines (IL-4, IL-5 and IL-13), as well as inflammatory responses (mast cell and eosinophil infiltration, goblet cell hyperplasia) in the gut. In contrast, vaccinated groups did not show any systemic allergic symptoms or inflammatory responses in the gut upon challenge. Met e 1-specific IgE and Th2-linked cytokines in these mice remained at basal levels, with the MEM49-encoding DNA vaccine providing more robust protection against Th2 responses. The protection offered by both vaccines included higher levels of specific IgG2a antibodies that possess both *in vitro* and *in vivo* blocking abilities, as well as higher splenic levels of Th1-linked cytokines (IL-12 and IFN-γ). MEM49 vaccination increased expression of TGF-β in the small intestine, while MED171 vaccination increased Foxp3 expression.

**Conclusions:** Hypoallergen-based DNA vaccines could effectively protect against tropomyosin sensitization in mice via the establishment of Th1-oriented responses, recruitment of regulatory T cells, and induction of blocking IgG antibodies.

[This work was fully supported by the Health and Medical Research Fund (02130206), HKSAR Government].

## A67 The relationship between sputum pentraxin 3 levels and childhood asthma

### Hee Seon Lee, Kyung Eun Lee, Jung Yeon Hong, Mi Na Kim, Min Jung Kim, Yoon Hee Kim, In Suk Sol, Seo Hee Yoon, Kyung Won Kim, Myung Hyun Sohn, Kyu-Earn Kim

#### Yonsei University College of Medicine, South Korea

##### **Correspondence:** Hee Seon Lee – Yonsei University College of Medicine, South Korea

**Background:** Pentraxin 3 (PTX3) is soluble pattern recognition receptor, and acute phase protein that has emerged as a new serological marker reflecting tissue inflammation and damage under diverse diseases. We determined whether sputum PTX3 levels are elevated in patients with childhood asthma. We also investigated the relationship between sputum PTX3 levels and airway inflammation, pulmonary function, and bronchial hyperresponsiveness in children.

**Methods:** A total of 260 children (140 patients with asthma and 120 control subjects) were enrolled in this study. PTX3 levels were measured in sputum supernatants with ELISA. We performed spirometrys and methacholine challenge tests while measuring total eosinophil count, and serum levels of total IgE and ECP in all children.

**Results:** Sputum PTX3 concentration was significantly higher in children with asthma (mean ± SE, 1094.55 ± 224.65 pg/mL) than control subjects (mean ± SE, 177.36 ± 30.00 pg/mL, p < 0.001). Positive significant correlations were found between sputum PTX3 and bronchodilator response (r = 0.25, p = 0.013). Sputum PTX3 levels negatively correlated with FEV1 (r = -0.30, p = 0.001), FEV1/FVC (r = -0.27, p = 0.002), FEF25-75 (r = -0.392, p < 0.001). Sputum PTX3 levels also showed significant negative correlation with post- bronchodilator (BD) FEV1 (r = -0.25, p < 0.001) and post-BD FEV1/FVC (r = -0.25, p < 0.001).

**Conclusions:** Our results could support that PTX3 is involved in the pathogenesis of asthmatic airway. Sputum PTX3 would be a supportive biomarker reflecting asthmatic airway inflammation and remodeling in childhood asthma.

## A68 The role of local antibody responses in the nasal inflammation of allergic rhinitis (AR) patients

### Ji Hye Kim^1^, Hae-Sim Park^1^, Yoo Seob Shin^1^, Young Min Ye^1^, Daehong Seo^2^, Moon Gyeong Yoon^1^, Young Mok Lee^3^

#### ^1^Ajou University School of Medicine, South Korea; ^2^Choongmoo Hospital; ^3^GF Clinic

##### **Correspondence:** Ji Hye Kim – Ajou University School of Medicine, South Korea

**Background and purpose**: AR is a common and increasing allergic disease, in which D.farinae is the most common causative allergen. The aim of this study is to compare locally produced antibodies to D.farinae in nasal mucosa between positive and negative responders to nasal provocation test(NPT) to D.farinae and evaluate relationships with the levels of inflammatory mediators.

**Subjects and methods**: Sixty AR patients with went through NPT to D.farinae. The sinus packs were placed into patients’ both nasal cavities for 5 minutes to earn nasal secretion after NPT. Total IgE, specific IgE to D. farinae, ECP, IL-8, VEGF and tryptase levels were measured by using ImmunoCAP (ThermoFisher, Uppsala, Sweden). D.farinae-specific IgE, IgA, IgG and secretory IgA antibodies were measured by ELISA. IL-8 and VEGF levels were measured by ELISA kit(Endogen, Woburn, MA and R&D Systems, Inc, Minneapolis, MN, respectively).

**Results:** High levels of total IgE, specific IgE, specific IgG, specific IgA, secretory IgA as well as ECP, IL-8, VEGF and tryptase were detected in nasal secretion, but showed no significant differences between positive and negative responders. Inflammatory mediators including ECP, IL-8 and VEGF were not only detected but well correlated with specific antibodies to D.farinae (*P*<0.05, respectively). Compared to ELISA method, ImmunoCAP system is more sensitive in detection of specific IgE to D.farinae. Difference between right and left nasal secretion had no statistical significance.

**Conclusion**: These findings confirmed the presence of specific antibodies to D.farinae-sensitive AR patients. Localized antibodies abundant in nasal mucosa may have a role in the nasal inflammation of AR patients sensitized to D.farinae.

## A69 A case of ofloxacin-induced anaphylaxis by non-IgE, but specific IgG4-mediated responses

### Daehong Seo^1^, Ji Hye Kim^2^, Young-Mok Lee^3^, Young Min Ye^2^, Hae-Sim Park^2^

#### ^1^Cheonan Chungmu Hospital, South Korea; ^2^Ajou University School of Medicine; ^3^GF Allergy Clinic

##### **Correspondence:** Daehong Seo – Cheonan Chungmu Hospital, South Korea

Anaphylaxis induced by ofloxacin has been rarely reported, and its pathogenetic mechanism has not yet been fully understood. This is a report of non-IgE mediated ofloxacin-induced anaphylaxis in a 20-year-old female patient who suffered from allergic rhinitis and had a previous history of acute urticaria induced by nonsteroidal anti-inflammatory drugs. She developed generalized urticaria and anaphylaxis following oral ingestion of ofloxacin. When we measured specific serum antibodies to ofloxacin-human serum albumin (HSA) conjugate using ELISA, a high level of specific serum IgG4 was detected, but serum specific IgE was not detectable. Moreover, a basophil activation test showed a significant up-regulation of CD203c with addition of ofloxacin and anti-IgG4 antibody in the patient with no significant changes in 3 non atopic healthy controls. These findings suggest that ofloxacin can induce anaphylaxis via pathogenetic mechanisms involving non IgE-mediated, but specific IgG4-mediated responses.

## A70 Serum LTE4 metabolite as a biomarker for aspirin exacerbated respiratory disease

### Ga Young Ban^1^, Kumsun Cho^2^, Seung-Hyun Kim^1^, Yong Eun Kwon^3^, Moon Gyeong Yoon^1^, Ji Hye Kim^1^, Yoo Seob Shin^1^, Young Min Ye^1^, Dong-Ho Nahm^1^, Hae-Sim Park^1^

#### ^1^Ajou University School of Medicine, South Korea; ^2^Seoul National University College of Medicine; ^3^Chosun University College of Medicine

##### **Correspondence:** Ga Young Ban – Ajou University School of Medicine, South Korea

**Background**: Provocation tests with aspirin are the most reliable method to confirm the diagnosis of aspirin exacerbated respiratory disease (AERD). To date, there is not an appropriate in vitro test to diagnose AERD.

**Objectives:** To further understand the pathogenic mechanisms of AERD and investigate the potential biomarkers for AERD using metabolomics.

**Subjects and Method:** Forty five AERD, 44 aspirin tolerant asthma (ATA) and 28 normal controls were enrolled. Urine and serum samples were collected from all asthma subjects before and after lysine-aspirin bronchoprovocation test (Lys-ASA BPT). The metabolites of LTE_4_ and PGF_2_α were analyzed using liquid chromatography with mass spectrometry.

**Results**: Baseline serum and urine LTE_4_ levels were significantly higher in AERD than ATA (18.14 ± 7.19 pg/ml vs. 14.65 ± 4.39 pg/ml, *P* = 0.007; 7.36 ±13.19 pmol/mg creatinine vs. 2.69 ±3.62 pmol/mg creatinine, *P*=0.047, respectively). The ROC curve analysis that discriminates AERD from ATA indicated the cutoff value of the serum LTE_4_ was 14.5 pg/ml, with 71.1% sensitivity and 52.3% specificity (AUC = 0.651, *P* = 0.014) which was improved further if combined with serum periostin level (81.4% sensitivity, 63.4% specificity, AUC = 0.761, *P* < 0.001). However, the ROC curves were not formed using urine LTE_4_ level. There were no significant correlations between serum and urine levels of two metabolites. Urine LTE_4_ levels were significantly correlated with those of PGF_2_α before and after Lys-ASA BPT (*r* = 0.562, *P* < 0.001; *r* = 0.627, *P* < 0.001). Baseline urine LTE_4_ and PGF_2_α were significantly correlated with fall of FEV_1_ % after Lys-ASA BPT (*r* = 0.463, *P* = 0.008, and *r* = 0.610, *P* <0.001, respectively).

**Conclusion**: Serum LTE_4_ level can be a useful serum biomarker for representing the phenotype of AERD considering serum collection is simpler than any other sample collections. Overproduction of LTE4 and mast cell activation can contribute to induce bronchoconstriction to inhaled aspirin in the pathogenic mechanisms of AERD.

## A71 Local and systemic reactions of dust mite subcutaneous immunotherapy (SCIT) among children in a tertiary care hospital

### Pilar Agnes Gonzalez Andaya

#### University of Santo Tomas Faculty of Medicine, Philippines

**Background:** Allergen-specific immunotherapy is currently the only curative modality in treating allergy. Previous studies have demonstrated that local cutaneous reactions after injection are common. Some reports on life-threatening anaphylactic reactions, further limit its clinical use.

**Methods**: To determine the prevalence of dust mite (*Dermatophagoides farinae* and *Dermatophagoides pteronyssinus*) subcutaneous immunotherapy reactions among children, 104 pediatric patients with allergic rhinitis and/or bronchial asthma undergoing subcutaneous immunotherapy to dust mites were included. Local (small, large, consecutive, and recurrent) and systemic reactions were recorded.

**Results**: Between January 2008 to August 2013, 104 pediatric patients received a total of 6277 injections. The local reaction rate was 9.38% (CI 8.70–10.12%), small local reaction rate was 2.01% (CI 1.68-2.39%), and large local reaction rate was 7.54% (6.89-8.22%). The systemic reaction rate was 0.14% (0.06 – 0.27%). Consecutive local reactions increase the probability of developing either a small or large local reaction (p<0.001). No significant correlation (p=0.367) between consecutive local reactions and systemic reactions was found. Local reaction rates of patients with a diagnosis of concomitant asthma and allergic rhinitis were significantly higher (p=0.018) than patients with allergic rhinitis alone. Potential risk factors for systemic reactions were bronchial asthma, build-up phase of immunotherapy, and high allergen extract concentration.

**Conclusion**: Majority of the reactions after dust mite (*D. farinae* and *D. pteronyssinus*) subcutaneous immunotherapy among children with respiratory allergy were local, with a relatively low local reaction rate at 9.38%. Mild systemic reactions occurred in 0.14% of injections. Consecutive and recurrent local reactions are not presumptive of a systemic reaction.

## A72 Effects of carboxymethyl glucan (CM-glucan) in children with allergic rhinitis and asthma: A randomized controlled trial

### Pilar Agnes Gonzalez Andaya

#### University of Santo Tomas Faculty of Medicine, Philippines

**Background:** Beta-glucans are known immunomodulators with anti-carcinogenic properties. They may cause skewing of the T helper (Th) 2-mediated immune response to a Th1-mediated response, thus having a potential role in decreasing allergic symptoms among patients with rhinitis and asthma.

**Methods**: To evaluate the effect of CM-glucan on allergic rhinitis symptoms and asthma control among children, 50 poly-sensitized children aged 7 to 18 years with allergic rhinitis and asthma were enrolled. Patients were randomized to receive CM-glucan (10 mg per orem bid) or placebo over a period of 90 days. The Total Nasal Symptoms Score (TNSS) and Asthma Control Test (ACT) questionnaires were used to assess symptom improvement at baseline, 2 weeks, and post-treatment. Lung function parameters and nasal eosinophil counts (%) were measured.

**Results**: Out of 50 patients (CM-glucan, n=26; placebo, n=24) included in the study, 40 patients (CM=glucan, n=20; placebo, n=20) completed the study. After 90-days treatment, CM-glucan significantly improved runny nose (rhinorrhea) (p=0.002). Nasal congestion, itchy nose, post-nasal drip, and sneezing improved in both treatment groups but did not differ significantly (p> 0.05). The mean nasal eosinophil counts (p=0.025) significantly decreased from baseline to post-treatment; however, no significant difference (p=0.486) between the 2 groups was seen. Similarly, CM-glucan significantly improved nocturnal asthma symptoms (wheezing, coughing, and shortness of breath)(p=0.020). The mean FVC (p<0.001), FEV1 (p<0.001), FEF_25-75_ (p<0.001), and PEFR (p<0.001) significantly increased in both groups, although no difference (p>0.05) was found when comparing post-treatment improvements between the 2 groups.

**Conclusion**: CM-glucan significantly reduces rhinorrhea and nocturnal asthma symptoms among children with allergic rhinitis and asthma.

## A73 Autophagy mechanisms in patients with severe asthma: A new therapeutic target

### Ga Young Ban^1^, Chang Gyu Jung^2^, Seung-Ihm Lee^1^, Duy Le Pham^1^, Dong-Hyeon Suh^1^, Eun-Mi Yang^1^, Young Min Ye^1^, Yoo Seob Shin^1^, Hae-Sim Park^1^

#### ^1^Ajou University School of Medicine, South Korea; ^2^Uiseong Public Health Center

##### **Correspondence:** Ga Young Ban – Ajou University School of Medicine, South Korea

**Background**: Recent study implied a possible involvement of autophagy in severe asthma (SA).

**Objective**: To investigate the role of autophagy in SA pathogenesis.

**Method**: We enrolled 33 SA patients, 14 non-severe asthma (NSA) patients and 33 normal healthy controls (NC). Autophagy was evaluated in sputum granulocytes and peripheral blood cells (PBCs) using Western blot, confocal microscopy, transmission electron microscopy, and flow cytometry. To induce autophagy in vitro, HL-60 cells and primary eosinophil cells were treated with interleukin (IL)-5; A549 cells and primary airway epithelial cells were treated with IL-1β.

**Results**: Increased expression of microtubule-associated protein light chain (LC3)-II was noted in the sputum granulocytes and PBCs from SA group compared to NSA and NC groups. In confocal microscopy, autophagosomes were observed in the eosinophils from sputum granulocytes and PBCs of SA patients. IL-5 induced autophagy in HL-60 cells with increased eosinophil cationic protein expression. When IL-5 was treated on primary eosinophils of SA patients, autophagy expression increased which were not affected by dexamethasone. After treatment of IL-1β on A549 cells and primary airway epithelial cells, autophagy expression and IL-8 production increased which were attenuated by autophagy inhibitors including E64D+Pepstatin A, 3-MA and LY294002, but not by dexamethasone. Atg5-knockdown or Beclin1-knockdown of A549 cells significantly decreased the IL-8 production as well as the LC3-II. Autophagy expression in peripheral eosinophils from SA patients were significantly higher than those from NC (*P*=0.013).

**Conclusion**: We suggest that autophagy play a pivotal role in SA pathogenesis. The autophagy modulation may be a novel therapeutic target for conventional therapy-resistant SA patients.

## A74 Aggravation of airway inflammation and hypperresponsiveness following nasal challenge with dermatophagoides pteronyssinus in perennial allergic rhinitis patients without symptoms of asthma

### Wan Jun Wang, MO Xian, Yan Qing Xie, Jing Ping Zheng, Jing Li

#### State Key Laboratory of Respiratory Disease, the First Affiliated Hospital of Guangzhou Medical University, China

##### **Correspondence:** Wan Jun Wang – State Key Laboratory of Respiratory Disease, the First Affiliated Hospital of Guangzhou Medical University, China

**Background**: To investigate the changes in upper and lower airway inflammation and responsiveness following *Dermatophagoides pteronyssinus* (Der-p) nasal provocation test (NPT) in Der-p sensitized rhinitis patients without asthmatic symptoms.

**Methods**: Study subjects included 15 non-asthmatic Der-p sensitized rhinitis (AR) patients with airway hyperresponsiveness (AHR) (AR+AHR+), 15 AR patients without AHR (AR+AHR-), 15 healthy control (HC) with Der-p sensitization (HC+DP+) and 15 HC without Der-p sensitization (HC+DP-). They underwent Der-p NPT. Nasal lavage and resistance, sputum induction, forced expiratory volume in 1 second (FEV_1_) and airway responsiveness to histamine bronchoprovocation (PD_20_-FEV_1_) and exhaled nitric oxide (FeNO) were performed before and 6 hours after NPT. Number of eosinophils in nasal lavage fluid and induced sputum eosinophils were determined.

**Results**: The nasal airway resistance increased after NPT in subjects of all four groups (P<0.05). FEV1 % predicted decreased in AR+AHR+ patients after NPT (P<0.05). Eosinophils in nasal lavage fluid and sputum increased significantly after NPT in AR+AHR+ and AR+AHR- patients (P<0.001). PD_20_-FEV_1_ was decreased and FeNO was increased significantly after NPT only in AR+AHR+ patients (P<0.05), wherease, no significant changes were observed in HC+DP+ and HC+DP- subjects after NPT. The number of eosinophils from nasal lavage was strongly correlated with the sputum eosinophils, the level of FE_NO_ (r=0.737, p=0.000 and r=0.736, p=0.000), and was negatively correlated with FEV_1_, PD_20_ (r=-0.287, p=0.026 and r=-0.436, p=0.000).

**Conclusions**: House dust mite nasal provocation test may induce upper and lower airway inflammation and hyperresponsiveness in patients with persistent allergic rhinitis without asthmatic symptoms.

## A75 Serum 25-hydroxyvitamin d in early childhood is non-linearly associated with allergy

### Emma Merike Savilahti^1^, Outi Mäkitie^1^, Anna Kaarina Kukkonen^2^, Sture Andersson^1^, Heli Viljakainen^1^, Erkki Savilahti^1^, Mikael Kuitunen^1^

#### ^1^Children’s Hospital, Finland; ^2^Hospital for Allergic and Skin Diseases

##### **Correspondence:** Emma Merike Savilahti – Children’s Hospital Finland

**Background:** Vitamin D has several immunological functions. Data on the relation of vitamin D status and allergy are controversial.

**Methods:** The study investigated the association between serum concentrations of 25-hydroxyvitamin D (25-OHD) and allergy in childhood. The study population (n=819) was part of a randomized, double-blind placebo-controlled trial where mothers of offspring with high risk for allergy received for the last 4 weeks of pregnancy a mixture of probiotics, or placebo, and after birth, the child received the same for 6 months. Study subjects were followed for the emergence of sensitization and allergic symptoms for 5 years, with medical examinations at the ages of 3 and 6 months, 2 and 5 years, and in the case of allergic symptoms. Levels of 25-OHD were measured in umbilical cord blood samples (n=724) and serum samples drawn at the age of 2 years (n=369); the data were categorized in tertiles and quartiles. The relation between 25-OHD levels and sensitization and allergy were analyzed with multivariable logistic regression analysis.

**Results:** 25-OHD levels in the second tertile in umbilical cord blood were associated with higher risk for sensitization by the age of 2 years and allergic disorder by the age of 5 years. In serum samples at the age of 2 years, the third quartile of 25-OHD levels was associated with higher risk of sensitization and IgE-mediated allergies by the age of 5 years.

**Conclusions:** The 25-OHD levels in early childhood associate with the emergence of allergy but the association appears to be non-linear.

## A76 Fric test in dermographism

### Kiran Godse

#### D.Y.Patil Medical College and Hospital, India

This instrument is known as the Fric Test or friction test dermatographometer (BiomedizinischeWekstatte der Charite, Berlin, Germany). It is a flat, rectangular plastic template with four smooth plastic tips of varying lengths (2.5 mm, 3.0 mm, 3.5 mm, and 4.0 mm), set 2 cm apart. Each tip is about 3 mm in diameter with a slightly rounded end.

Thirty diagnosed adult cases (18 Females and 12 Males) of symptomatic dermatographism (on the basis of history and clinical findings) were enrolled in the study after obtaining a written informed constent.Likewise, thirty patients with no active pruritic skin disease were allocated to the control group.Patients on antihistamines, steroids, and otherimmunosuppresants were excluded from the study as were children, pregnant females, and those unwilling to be enrolled.

The results of the utility of the Fric test over the traditional ball point pen test were quite remarkable. out of the 30 subjects who had voluntarily enrolled in our study, an astounding 28 (93%) of them displayed a positive Fric test, which is defined as one which produces a wheal of more than 1 centimeter diameter with at least one tip. Unsurprisingly, only 3 (10%) of the 30 individuals generated a positive Fric test (all positive with the 4 mm tip length) in our control group. The comparative results with different tip lengths in patients with a positive Fric test were also recorded and tabulated . As expected, the length of the tip of the instrument positively correlated with the probability of a positive test. All of the subjects were positive with 4 mm length but only 14% showed positivity for the 2.5 mm length.

When the same procedure was carried out using a ball point pen on the left forearm, 18 (60%) out of the 30 subjects developed a positive ball point pen test (development of a wheal of more than 1 centimeter diameter within 10 minutes after provocation), whilst a mere 4 (13.3%) out of the 30 individuals developed a wheal in our control group.

With the advent of the Fric test, many of drawbacks of ball point pen can possibly be eliminated. Thus the practicing clinician could assess the severity of the disease and the response to treatment without taking recourse to more expensive investigations.

Fric test is useful instrument for assessing severity of symptomatic dermographism.

## A77 Neutrophil autophagy and extracellular trap could contribute to asthma severity

### Duy Le Pham^1^, Ga Young Ban^1^, Seung-Hyun Kim^1^, Eun-Mi Yang^1^, Hae-Sim Park^1^, Ji-Ho Lee^2^, Yong-Joon Chwae^1^

#### ^1^Ajou University School of Medicine, South Korea; ^2^The Armed Forces Chun Cheon Hospital

##### **Correspondence:** Duy Le Pham – Ajou University School of Medicine South Korea

**Background:** Previous studies suggested the involvement of activated neutrophils in the pathogenesis of severe asthma; however the mechanisms of neutrophil activation are not fully understood. We hypothesized that neutrophil autophagy and extracellular trap (NET) production may contribute to activate neutrophils in severe asthma.

**Methods:** Neutrophils isolated from peripheral blood of patients with severe asthma (SA, n=34) and non severe asthma (NSA, n=38) were left untreated or treated with IL-8 (100 ng/ml) for 16 hr. Autophagy level was evaluated by microtubule-associated light chain 3 (LC3) expression using Western blot and immunoflruorescent microscopy. NET production was measured by picogreen assay and immunoflruorescent microscopy. The effect of autophagy inhibitors (LY294002 and hydrochloroquine-HCQ) on neutrophil migration was assessed by neutrophil chemotaxis assay using ChemoTX® system.

**Results:** Both naïve and IL-8 treated neutrophils of the SA group produced significantly higher LC3 expressions than those of the NSA group (*P*<0.05 and *P*<0.01, respectively). IL-8 treated neutrophils generated significantly higher autophagy levels compared to naïve neutrophils in the SA group (*P*<0.001), which was not observed in the NSA group. NET production levels from naïve neutrophils were not different between the SA and NSA groups; however, NET levels produced by IL-8 treated neutrophils of the SA group were significantly higher than to those of the NSA group (P<0.01). A significantly positive correlation between autophagy and NET production was observed in both naïve and IL-8 treated neutrophils (*P*<0.001 for both). LY294002 (20 nM) inhibited LC3 expression while HCQ (10 μM) induced LC3 accumulation by blocking autophagosome degradation; both of which could significantly inhibit IL-8-induced migration of neutrophils (*P*<0.01 and *P*<0.05, respectively).

**Conclusions:** Autophagy and NET could contribute to the severity of asthma. Modulation of autophagy may help to improve asthma severity by reducing neutrophil migration into the asthmatic airways.

## A78 Redox Modulation for the Treatment of Toluene Diisocyanates-Induced Lung Inflammation

### Li-Ming Chin, Chi-Chang Shieh

#### National Cheng Kung University, Taiwan

##### **Correspondence:** Li-Ming Chin – National Cheng Kung University, Taiwan

Toluene diisocyanates (TDI) is a low-molecular-weight, high reactive chemical that widely used in industry for the production of polyurethane foams, vanish, paint, and isolation material. Workers expose to TDI may cause the occupational asthma and show the mimic symptom as asthma. Several mechanism of TDI-induced occupational asthma has been suggested, indicating that the pathogenesis may be more complex than other types of asthma. One such candidate mechanism is oxidant stress. In our previous investigation, we found that mice lacking leukocyte NADPH oxidase, which produce ROS after activation, may have less lung inflammation and airway hyperresponsiveness than wild-type (WT) mice after TDI exposure. These results implicated that TDI exposure may induced oxidant stress in mice lung and cause lung inflammation **(Liu et al., 2011)**. Therefore, in this study we are testing the use of clinical antioxidant drug, *N*-acetylcysteine (NAC) to treat the mice which suffered from TDI. In our experimental design, we treated the mice with NAC in three different methods: (1) Pre-treatment: The mice were pre-treated with NAC before the sensitization of TDI. (2) Continuously treatment:The mice were treated with NAC continuously during the sensitization of TDI. (3) Post-treatment:The mice were treated with NAC after the sensitization of TDI. Then, we used MDA detection assay kit to determine the oxidant stress. The histological analysis and cytokine profiles screening were performed to determine the inflammation level. Last, we measured mice lung resistance and compliance with FinePointe Resistance and Compliance system. The lung MDA production, which represents the oxidation level, were successfully reduced in each methods of NAC treatment, indicating that NAC treatment successfully reduced the oxidant stress which produced from TDI exposure. Histopathological analysis showed, reduced level of inflammation, lung fibrosis and mucus production after NAC treatment in TDI-induced mice. Moreover, TDI-treated mice with NAC treatment had better lung function parameters than the group without NAC treatment. The treatment of NAC in three different methods could successfully cure the mice suffered from TDI exposure. The mechanism of an antioxidant drug reveals a therapy for TDI- induced occupational asthma.

Reference

Liu, S.Y., Wang, W.Z., Yen, C.L., Tsai, M.Y., Yang, P.W., Wang, J.Y., Ho, C.Y., and Shieh, C.C. (2011). Leukocyte nicotinamide adenine dinucleotide phosphate-reduced oxidase is required for isocyanate-induced lung inflammation. The Journal of Allergy and Clinical Immunology *127*, 1014-1023.

## A79 A case of occupational asthma and rhinitis with anaphylaxis to Korean ginseng and sanyak

### Ji Hye Kim^1^, Hye-Soo Yoo^2^, Moon Gyeong Yoon^1^, Ga Young Ban^1^, Ga Young Ban^1^, Yoo Seob Shin^1^, Young Min Ye^1^, Hae-Sim Park^1^

#### ^1^Ajou University School of Medicine, South Korea; ^2^Suwon Center for Environmental Disease and Atopy

##### **Correspondence:** Ji Hye Kim – Ajou University School of Medicine, South Korea

Korean ginseng (*Panax ginseng*) and Sanyak (*Dioscorea batatas*) are widely used as a food or herbal medicine. There have been a few cases with ginseng-induced occupational asthma (OA) and rhinitis to inhaled powder of herbal materials, such as ginseng. Here we report a case of anaphylaxis due to ginseng and Sanyak which were sensitized via inhalation.

A 55-year-old woman presented with symptoms of indigestion, abdominal pain, dyspnea and chest discomfort after ingestion of fresh ginseng and hemp juices. She had been diagnosed as having non-allergic asthma and rhinitis. However she had an OA due to various herbal materials such as ginseng and Sanyak powders which were sensitized working as a pharmacist. Serum total IgE level was increased (247KU/L). Serum specific IgE to ginseng was undetectable, but positive to Sanyak extract by enzyme-linked immunosorbent assay (ELISA). High serum specific IgG4 to ginseng extract was noted however, serum specific IgG4 to Sanyak was not detected IgE ELISA inhibition test showed significant inhibitions by hemp, not by ginseng, while IgG4 ELISA inhibition test showed significant inhibitions by ginseng, not by Sanyak, indicating that both extracts did not have a cross-reactivity which is comparable with taxonomical classification 4-20% sodium dodecyl sulfate-polyacrylamide gel electrophoresis (SDS-PAGE) and IgG4-immunoblot analysis revealed two IgG4 binding components (17kDa, 24kDa). These findings suggest that although IgE or IgG4 sensitization occurs via inhalation routes, repeated exposure via oral route can induce severe food allergy and generalized symptoms such as anaphylaxis. Further additional studies including basophil activation tests with these two extracts will be needed.

## A80 Factors of influencing epidermal permeability barrier defects in atopic dermatitis children

### Myong Soon Sung^1^, Jin Uck Choi^2^, Sung Won Kim^2^, Yong Jin Hwang^2^

#### ^1^Gumi CHA University Hospital, South Korea; ^2^Busan St. Mary’s Hospital

##### **Correspondence:** Myong Soon Sung – Gumi CHA University Hospital, South Korea

**Background**: Even though eczema herpeticum and hypoalbuminemia are serious and increasingly prevalent complication of AD, just little studies have investigated in AD children. So we investigated the relationship between the clinical factors and the allergic laboratory features in a group of the AD children depending on the presence of eczema herpeticum and hypoalbuminemia.

**Method**: This cross-sectional study was carried out on 105 patients aged 3 months to 18 years between June 2007 and April 2015. A retrospective data collection through individual medical record, depending on the presence of EH and hypoalbuminemia, AD children were divided into three groups- EH^+^ (group 1), hypoalbuminemia (group 2) and severe AD (group 3).

**Results**: The male gender was related to the presence of EH (OR, 2.56; 95% CI, 1.19-5.53, *P*=0.01), but age were not related. The age was related to the presence of hypoalbuminemia (OR, 3.12; 95% CI, 2.21-6.33, *P*=0.01), but gender were not related. Serum total IgE and ECP levels were higher in the EH group, but serum total eosinophil count levels were higher in the severe AD. There was a statistical difference between three groups in the skin culture (*P*<0.05). Even after adjusting for age and gender the correlation between the positive result of skin cultures and the presensce of EH was significant (*P*<0.01), and MRSA was related to only the EH^+^group (OR, 0.19; 95% CI, 0.04-0.92, *P*=0.03).

**Conclusion**: We have identified the male gender, the positive result of skin cultures, and MRSA, as factors influencing factors of EH and age is as influencing factors of hypoalbuminemia in AD children.

## A81 Innate type 2 response to aspergillusfumigatus in a murine model of atopic dermatitis-like skin inflammation

### Arum Park^1^, Eun Lee^1^, Song-I Yang^2^, Hyun-Ju Cho^1^, Jinho Yu^1^

#### ^1^Asan Medical Center Seoul Korea, South Korea; ^2^Hallym University Sacred Heart Hospital

##### **Correspondence:** Arum Park – Asan Medical Center Seoul Korea, South Korea

Innate type 2 response to *Aspergillusfumigatus* in a murine model of atopic dermatitis-like skin inflammation.

**Purpose**: Atopic dermatitis (AD) is a chronic and relapsing inflammatory skin disease mediated by Th2 cells in acute phase. Type 2 innate lymphoid cells (ILC2s) have a role in initiating Th2 response. We investigated whether ILC2s are involved in the skin inflammation in a murine model of AD-like skin induced by *Aspergillusfumigatus* (*Af*).

**Method**: We applied *Af*crude extract (40μg) to the dorsal skin of BALB/c mice 5 times a week repeatedly with an interval of 2weeks. Clinical score and transepidermal water loss (TEWL) were assessed and histology was examined. The levels of interferon (IFN)-γ, IL-13, IL-17 in skin draining lymph node (LN) and immunoglobulin E (IgE) in serum were measured using ELISA. The mRNA expressions of IL-25, IL-33, thymic stromal lymphopoietin (TSLP) in the skin were measured using Real-Time PCR. The ILC2s of Lin^-^ CD25^+^ IL-33R^+^cells in the skin and LN were analyzed using flow cytometry.

**Result**: The clinical score and TEWL increased in mice applied with *Af*(*Af*group), compared with control group. Histologic findings showed that epidermal and dermal thickness, and eosinophilic and mast cell infiltration in the skin of *Af*group. The levels of total IgE were increased in the serum of *Af* group. Moreover, *Af*group showed increased levels of IL-13 in the supernatant from culture of skin draining LN stimulated with *Af*. The populations of Lin- CD25+ IL-33+cells were increased in the skin of *Af* group. The mRNA expression of IL-33 was increased in the skin of *Af*group.

**Conclusion**: This study suggests that innate type 2 response to *Af* may have a role in Th2-mediated skin inflammation in a murine model of AD-like skin lesions induced by *Af*.

## A82 Activin a receptor 1C may implicate in the development of sensitive skin

### Dong Hun Lee, Eun Ju Kim, Yeon Kyung Kim, Eun Jin Doh, Hee Chul Eun, Jin Ho Chung, Young Mee Lee, Seon Pil Jin

#### Seoul National University Hospital, South Korea

##### **Correspondence:** Dong Hun Lee – Seoul National University Hospital, South Korea

**Background:** Sensitive skin is a hyperactive skin condition characterized by sensory symptoms showing exaggerated reactions in response to internal stimulants and external irritants. Recently, using microarray-driven approach, we found that several signaling pathway regulated genes including activin A receptor 1C (ACVR1C) were down-regulated in sensitive skin. ACVR1C, a type I serine/threonine kinase receptor for the transforming growth factor (TGF)-β family, is activated by various ligands such as nodal, activin B.

**Methods:** In this study, we investigate the roles of ACVR1C in the pathogenesis of skin hypersensitivity using an *in vitro* model as well as human skin.

**Results:** ACVR1C mRNA and protein expressions were significantly decreased in sensitive skin compared with non-sensitive skin. A decreased level of pH in sensitive skin can induce an increase of calcium influx through transient receptor potential cation channel subfamily V member 1 (TRPV1). Indeed, sensitive skin showed excessive calcium accumulation as compared with non-sensitive skin. RD cells transfected with ACVR1C siRNA exhibited markedly enhanced responses in intracellular Ca^2+^ concentration following calcium ionophore treatment. Furthermore, knockdown of ACVR1C induced the expression of TRPV1 and calcitonin gene-related peptide (CGRP) indicating the causative role of ACVR1C in the pathogenesis of pain in sensitive skin. Finally, we investigated whether stimulation of ACVR1C pathway has a therapeutic potential to alleviate sensitive skin. Intriguingly, treatment of RD cells with Nodal induced a substantial reduction in the Ca^2+^ influx and the expression of TRPV1 and CGRP which was increased by knockdown of ACVR1C, supporting the possible therapeutic role of ACVR1C stimulation in sensitive skin.

**Conclusions:** Taken together, our results demonstrate that ACVR1C play crucial roles in the pathogenesis of sensitive skin and the activation of these signaling pathways *in vitro* successfully reversed pain sensation in RD cells, thereby raising the possibility of a novel therapeutic approach for skin hypersensitivity.

## A83 Genetic association and eQTL analyses of genes associated with allergy in atopic/non-atopic asthma

### Xingnan Li^1^, Naftali Kaminski^2^, Sally Wenzel^3^, Eugene Bleecker^1^, Deborah Meyers^1^

#### ^1^Wake Forest School of Medicine, USA; ^2^Yale School of Medicine; ^3^Division of Pulmonary, Allergy, and Critical Care Medicine

##### **Correspondence:** Xingnan Li – Wake Forest School of Medicine, USA

**Background**: Genome-wide association studies (GWASs) of allergic sensitization or self-reported allergic rhinitis (Ramasamy, *JACI*, 2011; Hinds, *Nat Genet*, 2013; Bonnelykke, *Nat Genet*, 2013) consistently identified nine genomic regions: *IL1RL1-IL18R1*, *LPP*, *IL2-IL21*, *TLR1-TLR6*, *SLC25A46-TSLP*, *TSLP-WDR36*, *HLA-DQB1*, *C11orf30-LRRC32*, and *CLEC16A*. Atopic and non-atopic asthma represent two distinctive subphenotypes, however, the genes involved in atopic/non-atopic asthma have not been studied.

**Methods**: Genetic association analysis of one single nucleotide polymorphism (SNP) from each candidate region was performed in non-Hispanic white asthmatic subjects from SARP, CSGA, ACRN, and TENOR cohorts (n = 1,209 and 154 for atopic and non-atopic asthma, respectively) using logistic regression model. Expression quantitative trait loci (eQTL) analysis, using linear regression model, of the candidate SNPs was performed in cells from human bronchial epithelial biopsy (BEC, n = 107) and bronchial alveolar lavage (BAL, n = 94) from the SARP cohort (GEO series accession number GSE67940).

**Results**: SNPs in seven genes (*IL18R1*, *LPP*, *SLC25A46*, *WDR36*, *HLA-DQB1*, *C11orf30*, and *CLEC16A*) were associated with general physician-diagnosed asthma in the GABRIEL study (*P* = 3.5x10^-12^ - 4.4x10^-3^) (Moffatt, *NEJM*, 2010). In our study, SNPs in five genes (*IL18R1*, *LPP*, *IL21*, *TLR6*, and *C11orf30*) were associated with atopic status in asthma subjects (*P* = 4.6x10^-3^ - 0.05). SNPs in *LPP* and *IL21* showed opposite risk alleles between asthma and autoimmune diseases. The gene expression pattern between BEC and BAL were distinct. In BAL, rs1464510, rs7696175, rs1043828, rs6906021, and rs7936562 were cis-correlated with mRNA expression levels of *LPP*, *TLR6*, *TSLP*, *HLA-DQB1*, and *C11orf30*, respectively (*P* = 1.1x10^-10^ - 0.04).

**Conclusions**: Most of the genes associated with allergic sensitization or self-reported allergic rhinitis are also associated with general asthma, indicating shared genetic factors among allergic diseases. *IL18R1*, *LPP*, and *C11orf30-LRRC32* are associated with atopic asthma and general asthma, however, the association effects (odds ratio) are stronger in atopic asthma. *IL21* and *TLR6* are associated with atopic status in asthma, but not associated with general asthma, indicating the importance to perform genetic analysis in more homogeneous asthma subphenotypes. Asthma and autoimmune diseases have shared immunopathogenesis pathways but in opposite directions. There is a tissue-specific gene expression regulation. SNPs in *LPP*, *TLR6*, and *C11orf30* are associated with atopic asthma and cis-correlated with their gene expression in BAL, indicating they are likely to be functional SNPs.

(This abstract is funded by NIH HL87665 and Go Grant RC2HL101487)

## A84 Gastroscope feature and clinical characteristics in 172 cases of children with henoch-schonlein purpura

### Zeng Huasong

#### Guangzhou Women and Children’s Medical Center, China

**Objective:** To investigate gastroscopic features and explore the relationship between clinical characteristics and gastroscopic features of Henoch-Schonlein puepura (HSP) in children.

**Methods:** To take gastroscope in 172 cases of children with HSP in our hospital and summarize the gastroscopic performance. All the case were divided into two groups by gastroduodenal mucosal bleeding or not. It was compared among the total time of abdominal pain, pain relief, hospitalization, fasting and kidney injury case in the groups.

**Results:** Gastroscope with varying degrees of injury of 172 cases has accounted for 169 cases (98.3%). Gastroscopic mainly revealed gastroduodenal mucosal congestion, edema, rough, erosion, bleeding and ulcer which involved 148 cases of gastric (86.0%), 158 cases of duodenal involvement (91.9%). Mucosal erosion and bleeding occurs mainly in duodenum, mostly in the descending duodenum. Duodenal bleeding accounted for 36 cases (20.9%) in the bulb and 92 cases (53.5%) in the descendant. Only five cases (2.9%) of ulcer occurred in the duodenum, where four cases of bulbar ulcer, one case of descending ulcer. Esophageal and gastric cardia mucosal just occurred in one case. There were not significant difference (P> 0.05) among the time of abdominal pain, pain relief, hospitalization and fasting in the group. There was no significant difference (P> 0.05) in the incidence of kidney injury between two groups of children during hospitalization. **Conclusions:** Gastroscopic features of HSP in children is characterized by bleeding, erosion of duodenal mucosa and occasional duodenal ulcer formation, which mostly involve the antral mucosa, rarely involving the esophagus, cardia. There was no significant difference (P> 0.05) among the severity of gastroscopic performance and the time of abdominal pain, fasting, hospitalization and kidney injury of the cases during hospitalization.

## A85 The role of TRPV1 in CD4+ t cell mediated inflammatory response of allergic rhinitis

### Ji-Hun Mo^1^, Ramachandran Samivel^1^, Eun-Hee Kim^1^, Ji-Hye Kim^1^, Jun-Sang Bae^1^, Young-Jun Chung^1^, Dae Woo Kim^2^

#### ^1^Dankook University, South Korea; ^2^Seoul National University

##### **Correspondence:** Ji-Hun Mo – Dankook University, South Korea

**Background:** The transient receptor potential vanilloid 1 (TRPV1), identified as a molecular target for the activation of sensory neurons by various painful stimuli, was reported to play a role in signaling and activation of CD4^+^ T cells. However, the role of TRPV1 remains poorly understood in allergic rhinitis.

**Objective:** To exploit the role of TRPV1 using TRPV1 antagonist such as *N*-(4-Tertiarybutylphenyl)-4-(3-cholorphyridin-2-yl)tetrahydropyrazine -1(2*H*)-carboxamide (BCTC) and TRPV1 knockout mice in allergic rhinitis mice models and using samples of patients with allergic rhinitis and to evaluate the molecular mechanism of TRPV1 in CD4+ T cell mediated signaling pathway in allergic rhinitis.

**Methods**: TRPV1 expression was measured in CD4^+^ T cell and cytokine analysis and T cell receptor signaling pathways were evaluated in BCTC pretreated T cell lines and TRPV1 ^(-/-)^ T cells. Allergic parameters were evaluated using TRPV1 antagonists and TRPV1 knockout mice in OVA-challenged mice model. Additionally, TRPV1 expressions were assessed in patients with allergic rhinitis.

**Results:** TRPV1 expression was localized in CD4^+^ T cell. BCTC pretreatment and TRPV1 knockout suppressed T cell cytokine production and suppressed T cell receptor signaling pathways, including NF-kB, MAP kinase and NFAT signaling in both Jurkat cell line and CD4 + T cells *in vitro*. TRPV1 antagonists (BCTC and theobromine) significantly reduced allergic parameters such as symptoms, total- and ova-specific IgE levels in the mice model of allergic rhinitis. In TRPV1 knockout and BCTC treated mice, nasal eosinophil infiltration and nasal mucosal cytokines transcriptional activities were decreased, when compared OVA-challenged wild-type mice. In human nasal mucosa, TRPV1+ inflammatory cells was frequently observed. TRPV1/CD4 double positive inflammaotry cells were increased in nasal mucosa in patients with allergic rhinitis as compared with non-allergic rhinitis and normal controls.

**Conclusion:** TRPV1 activation on CD4^+^ T cells is involved in TCR signaling and could be a novel therapeutic strategy in allergic rhinitis.

## A86 A Phenotype of Rhinitis from School Children Is Associated with the Development of Bronchial Hyperresponsiveness

### Eun Lee^1^, Si Hyeon Lee^2^, Young-Ho Kim^3^, Hyun-Ju Cho^3^, Ho-Sung Yu^4^, Mi-Jin Kang^4^, Song-I Yang^5^, Young-Ho Jung^6^, Hyung Young Kim^7^, Ju-Hee Seo^8^, Byoung-Ju Kim^9^, Hyo-Bin Kim^10^, So-Yeon Lee^11^, Ho-Jang Kwon^12^, Soo-Jong Hong^13^

#### ^1^Asan Medical Center, USA; ^2^Asan Institute for Life Sciences, University of Ulsan College of Medicine; ^3^Asan Medical Center; ^4^Institute for Life Science, Asan Medical Center; ^5^Hallym University Sacred Heart Hospital; ^6^Bundang CHA Medical Center, CHA University School of Medicine; ^7^Pusan National University Yangsan Hospital; ^8^Korea Cancer Center Hospital; ^9^University of Cincinnati College of Medicine; ^10^Inje University Sanggye Paik Hospital; ^11^Hallym University Sacred Heart Hospital; ^12^Dankook University College of Medicine; ^13^Childhood Asthma Atopy Center, Environmental Health Center, Asan Medical Center, University of Ulsan College of Medicine

##### **Correspondence:** Eun Lee – Asan Medical Center, South Korea

**Background:** Allergic rhinitis (AR) has a wide range of clinical aspects, and comorbid allergic diseases may accompany. We aimed to identify rhinitis phenotypes in school children and to predict the prognosis of developing bronchial hyperresponsiveness (BHR).

**Methods:** As a part of Children’s HEalth and Environmental Research (CHEER) study, a prospective follow-up study for 4 years with every 2 year interval, 2,491 children aged 6 to 14 years-old were enrolled in the first survey. Among them, 512 children had current rhinitis, defined as parental-reported doctor-diagnosed rhinitis and having rhinitis symptoms in the last 12 months. Variables including age, sex, body mass index, parental allergic history, income, maternal education level, AR treatment during the last 12 months, environmental tobacco smoking exposure, total serum IgE levels, eosinophil percentage, diagnosis of atopic dermatitis and asthma, lung function tests, BHR to methacholine and skin prick tests were used in the latent class analysis.

**Results:** We identified 4 phenotypes of rhinitis as the best fit in this study. Cluster types were characterized as "non-atopy with low socioeconomic status" (33% of sample, Cluster 1), "atopy with normal lung function" (39%, Cluster 2), "atopy with impaired lung function" (14%, Cluster 3), and “non-atopy and high socioeconomic status" (15%, Cluster 4). Total serum IgE levels and serum eosinophil percentages were highest in cluster 3. Children in cluster 3 showed highest prevalence of new development of BHR during 4-year follow-up (P=0.039).

**Conclusions:** From rhinitis phenotypes, rhinitis cluster with high atopy and impaired lung function in children is associated with new development of BHR. This finding suggests that identification of distinctive rhinitis phenotype will help to prevent the progression of new development of BHR in the aspect of allergic march.

## A87 Increased basal activation status was noted in adult anaphylaxis patients

### Sailesh Palikhe, Hae-Sim Park, Seung-Hyun Kim, Ji Hye Kim, Eun-Mi Yang

#### Ajou University School of Medicine, South Korea

##### **Correspondence:** Sailesh Palikhe – Ajou University School of Medicine, South Korea

**Background:** Anaphylaxis to food, drugs and diagnostic reagents have been increasingly reported in adult patients; however, its pathogenic mechanisms or an efficient diagnostic test have not been settled. Basophil activation test (BAT) using two activation markers, CD203c and CD63, has been readily used to assess basophil activation status. Considering that mast cell/basophil play an important role in anaphylaxis, we evaluated the basophil activation status using CD203c and CD63 expressions in anaphylaxis patients compared to allergic patients without anaphylaxis.

**Method:** 41 patients with various allergic diseases including asthma/rhinitis, chronic urticaria as well as anaphylaxis and 23 normal healthy controls (NC) were enrolled in the study. Allergic patients were divided into two groups: anaphylaxis (n=13) and non-anaphylaxis groups (n=28), based on the presence of anaphylaxis to different drugs, foods and diagnostic reagents. BAT using CD203c and CD63 expression was performed in baseline and after stimulation with anti-IgE or calcium (Ca^2+^) ionophore. The BAT is based on double staining with anti-CD123 and anti-HLA-DR and subsequent determination of the percentage of activated basophils by flow cytometry.

**Results:** Baseline % expressions of both CD203c (30.28±23.10 vs. 13.76±14.60, *P*=0.036) and CD63 (17.67±20.48 vs. 3.31±3.73, *P*=0.028) on basophils were significantly higher in anaphylaxis group compared to NCs, while no differences were noted in non-anaphylaxis group. When the positive cutoff value for a positive BAT result was defined as mean+2SD of CD203c expression, positive BAT rate tended to be higher in anaphylaxis group (38.46%) than in non-anaphylaxis group (17.85%, *P*=0.20), while no differences were noted in CD63 expression levels. There were no significant differences in baseline % expressions of CD203c and CD63 or those induced by anti-IgE/Ca^2+^ ionophore according to age, sex, atopic status, total IgE and ECP levels.

**Conclusion**: These findings suggest that increased basal activation status of basophils may contribute to develop anaphylaxis in adult patients regardless of atopy status, serum total IgE and ECP levels. BATs with applying each allergen will be useful to confirm the causative agent.

## A88 Clinical values of interferon-gamma enzyme-linked immunospot assays for management of antibiotic hypersensitivity in hospitalized patients

### Suda Sibunruang^1^, Jettanong Klaewsongkram^2^

#### ^1^Chulalongkorn University, Thailand; ^2^Allergy and Clinical Immunology Research Group, Chulalongkorn University

##### **Correspondence:** Suda Sibunruang – Chulalongkorn University, Thailand

**Background**

Antibiotic hypersensitivity in hospitalized patients is a challenging dilemma, as sometimes a thorough history might not be sufficient to identify the culprit agents. Vulnerable conditions often hamper investigational in vivo tests. Meanwhile, the decision of which drugs to be further continued or substituted is urgently needed. Enzyme-linked Immunospot (ELISPOT) assay has previously shown effectiveness in detecting drug-specific T cell response. Thus, we conduct this study to assess the role of ELISPOT for management of antibiotic hypersensitivity in clinical setting.

**Methods**

The medical records of inpatients who were diagnosed or developed non-immediate allergic reactions to antibiotics and underwent Interferon-gamma ELISPOT assay at King Chulalongkorn Memorial Hospital, Bangkok between 2012 and 2015 were retrospectively determined.

**Results**

Total 60 patients were evaluated (mean age 55.7 years, range 7-96 years), 33 (55 %) were female. Twenty-eight patients (46.7%) had underlying diseases including hematologic malignancy, solid tumor cancer, HIV infection, or autoimmune diseases. Fifteen (25%) individuals were concurrently on systemic corticosteroids. The majority of subjects (49.1%) experienced maculopapular exanthems (MPE), while 36.7 % had severe cutaneous adverse reactions. The mean duration from drug intake to the onset of symptoms was 8 days and the mean interval from symptoms onset to the collection of peripheral blood mononuclear cells (PBMCs) was 9.5 days. In most cases, the number of drug-specific IFN-gamma secreting cells was later analyzed with ELISPOT by incubating PBMCs with the culprit drugs or potential alternative drugs. Beta - lactams occupied 70.8% of the analysis. Penicillins, cephalosporins and carbapenems were the most frequently suspected compounds. The number of drug-specific IFN-gamma secreting cells more than 20 spot-forming cells/10^6^ (PBMCs) was considered a positive test. Among those examined for responsible drugs, 22 of 48 tests (45.8%) yielded positivity. The proportion of positive outcomes was 66.7% in acute generalized exanthematous pustulosis, 46.2% in MPE, 40.0% in drug rash with eosinophilia and systemic symptoms and lowest in Stevens Johnson syndrome (16.6%). Subsequently, twenty-four persons underwent drug challenges test. All were able to tolerate their alternative medications of which ELISPOT displayed negative results.

**Conclusions**

Providing excellent negative predictive value and favorable sensitivity in particular T cell-mediated reactions, IFN-gamma ELISPOT might be applicable to confirm antibiotic hypersensitivity in patients with a history of non-immediate reactions and reduce further allergic risk prior to receiving potential allergenic drug.

## A89 VDR gene polymorphism and 25-hydroxy vitamin d levels in children with food allergy

### Tatiana Sentsova, Ilya Vorozhko, Anna Timopheeva, Olga Chernyak, Vera Revyakina, Andrey Sokolnikov

#### Institute of Nutrition, Russia

##### **Correspondence:** Tatiana Sentsova – Institute of Nutrition, Russia

**Background:** Recent studies have shown that serum 25[OH]D levels were inversely associated with food allergy development. At the same time studies on the relationship between VDR polymorphisms and serum 25[OH]D levels in allergy are very limited.

**The aim** of the current study was to investigate 25-hydroxy vitamin D levels in children with food allergy and different variants of TaqI, BsmI and FokI VDR polymorphisms. Understanding the functional mechanism of VDR gene polymorphism may provide a strategy for prevention and treatment of food allergy.

**Materials and methods**: We examined 86 infants with food allergy aged from 1 to 12 months, who were on the artificial feeding. Measurement of 25[OH]D levels was performed by immunoenzyme method. RT-PCR with melting curve analysis was used for TaqI, BsmI and FokI VDR single nucleotide polymorphisms (SNP) detection. For statistical analysis Kruskal-Wallis, post-hoc and unpaired Aspin-Welch tests were used.

**Results:** Frequencies of allelic variants AA:AG:GG in FokI site were 0.20:0.64:0.16 respectively, as AA:AG:GG 0.23:0.48:0.29 in BsmI site and CC:TC:TT 0.23:0.42:0.35 in TakI site. Significantly decreased serum 25[OH]D levels in carriers of variants AG and GG comparably variant AA carriers in FokI site (32.95±2.118 vs 40.25±1.898 ng/mL, p=0.0188) were revealed. Statistically significant differences in serum 25[OH]D levels for TaqI and BsmI sites were not detected.

**Concluson:** FokI VDR polymorphism determine vitamin D status in children with food allergy.

## A90 An analysis of 145 oral almond challenge tests

### Makoto Nisihino, Yu Okada, Noriyuki Yanagida, Motohiro Ebisawa, Sakura Sato, Kiyotake Ogura, Tomoyuki Asaumi, Kenichi Nagakura, Tetsuharu Manabe, Hirotoshi Unno

#### Sagamihara National Hospital, Japan

##### **Correspondence:** Makoto Nisihino – Sagamihara National Hospital, Japan

**A) Background:** Tree nuts are known to be capable of causing severe allergic reactions. However, little has been reported about the details of oral challenge tests of individual tree nuts. The purpose of the study was to analyze the risk factors of a positive almond challenge test performed at Sagamihara National Hospital.

**B) Method:** Subjects were 145 patients who had received an open almond challenge test of greater than 3g from March, 2006 to April, 2014 at Sagamihara National Hospital. The oral food challenge (OFC) was administered in either 2 divided doses in a 1 hour interval or 3 divided doses in 30 minute intervals. After each challenge, the patient was observed for at least 3 hours. At any sign of subjective or objective symptoms deemed clinically significant, the challenge was terminated and necessary treatment was provided. We had measured patients’ almond specific IgE within one year of the challenge. Patient characteristics, positive rate of OFCs, and symptoms induced by OFCs were retrospectively analyzed.

**C) Results:** The age range of the 145 subjects was from 1.0 to 16.0 years (median, 7.0 years). There were 103 male (71%) and 42 female (29%) patients. Median almond specific IgE ranged from less than 0.35 to 68.1 kU_A_/L (median, 2.84 kU_A_/L). Almond had been eliminated from the children’s diet due to an immediate reaction to almond in 10 (6.9%) patients, due to a positive almond specific IgE in 117 (80.7%) patients, and for other reasons such as anxiety of parents in 18 (12.4%) patients. None of the 10 patients with a history of an immediate reaction to almond had experienced a case of anaphylaxis. Associated atopic disorders were atopic dermatitis in 72 (50%) patients, asthma in 35 (24%) patients, allergic rhinitis in 52 (36%) patients, and allergic conjunctivitis in 35 (24%) patients. OFC was positive in 7 (4.7%) of 145 patients. Symptoms in positive OFCs were oral mucosal symptoms in 5 patients, cutaneous symptoms in 3 patients, and gastrointestinal symptoms in 2 patients. There was no case of anaphylaxis. In the 4 patients who required treatment, only a dose of oral antihistamine was needed. In the comparison of OFC positive and negative patients, a significant difference was seen in history of an immediate reaction to almond (43% vs 5%, p=0.007). No significant difference was seen in other factors including almond specific IgE (1.38 kU_A_/L vs 2.84 kU_A_/L, p=0.33).

**D) Conclusions:** The only risk factor of a positive almond challenge was a history of an immediate reaction to almond. OFCs should be performed in patients sensitized to almond to confirm the diagnosis, especially in those patients without a history of an immediate reaction to almond.

## A91 Effect of creatine supplementation in fish allergenic potential; A proteomics study

### Pedro M Rodrigues^1^, Denise Schrama^1^, Gadija Mohamed^2^, Lizex Hüsselmann^2^, Lizex Hüsselmann^3^, Bongani Ndimba^3^

#### ^1^Ccmar, Universidade Do Algarve, Portugal; ^2^University of the Western Cape, Naprsu, National Agricultural Proteomics Research and Services Unit; ^3^Agricultural Research Council, Infruitec, Proteomics Unit

##### **Correspondence:** Pedro M Rodrigues – Ccmar, Universidade Do Algarve, Portugal

**Objectives**: In this study we tested a specific formulated diet supplemented with creatine to decrease the expression of **β**-parvalbumin (main fish allergen) in the muscle of *Sparus aurata (S. aurata).* The effects of creatine in the muscle proteome were also analyzed.

**Methods:** Aquaculture allows fish to be farmed under strict controlled conditions. However, knowledge on how farming practices can be used to modulate fish allergenicity is inexistent. Creatine is a nitrogenous organic acid that occurs naturally in vertebrates and helps to supply energy to cells, primarily muscle. Creatine was verified to reduce in 75% the expression of parvalbumin in rat skeletal muscle [1]. A trial was performed with *S. aurata* using 3 different concentrations of creatine (2%, 5% and 8%) that were added to a control diet (no creatine supplementation) and tested in tanks of 25 individuals in triplicate. At the end of the trial, plasma and muscle were individually collected for further analysis. Creatine levels in muscle samples were analysed. Cortisol was measured in plasma to address fish welfare/stress levels. Parvalbumin detection in muscle samples was studied using both proteomics and Western Blot techniques and an ELISA commercial kit (Bio-Check, UK). Comparative proteomics was performed on muscle samples to study differences in protein expression between the various treatments and further understand the effect of creatine in fish metabolism. To address this we used 2D Difference Gel Electrophoresis (DIGE). Differences in expression were analysed using the Samespots software (Totallab). Proteins with significant differences (P<0.05) were excised manually and identified by MALDI-TOF/TOF.

**Results:** Cortisol levels are similar to basal levels reported for *S. aurata*, although the treatment with 8% creatine shows a significant reduction compared with the control diet (p=0.03). Parvalbumin concentrations show no significant differences between the treatments. Creatine concentrations in muscle show a slight decrease in fish fed with supplemented diets. Comparative proteomics in muscle show some differences in protein expression submitted to diets supplemented with creatine. The four protein spots identified as parvalbumins show no significant differences in expression. Proteins differentially expressed are currently being identified by MALDI-TOF/TOF.

**Conclusion and current work:** No accumulation of creatine was found in muscle of fish fed with diets supplemented with creatine. The tested creatine percentages in fish diets did not significantly affect *S. aurata* allergenic potential. Current work involving new formulated diets and/or different fish species is underway. We expect in a near future to develop a specific fish diet that will target the expression of fish parvalbumins in order to reduced its allergenic potential.

Reference

[1] Gallo, M., et al. 2008. *Am J physiol,* 294, R1319–R1328.

## A92 Flagellin modulates the function of invariant NKT cells via dendritic cells in asthma patients

### Jae-Uoong Shim^1^, Young Il Koh^1^, Joon Haeng Rhee^2^, Ji-Ung Jeong^1^

#### ^1^Chonnam National University, South Korea; ^2^Chonnam National University Hospital

##### **Correspondence:** Jae-Uoong Shim – Chonnam National University, South Korea

**Backgrounds**: Invariant natural killer T (iNKT) cells play a critical role in the pathogenesis of asthma. We previously reported the association between blood Th2-like iNKT cells and lung function in asthma patients and the suppressive effect of Toll-like receptor 5 ligand flagellin B (FlaB) on asthma in a mouse model.

**Objective**: We investigated whether FlaB modulates the function of blood iNKT cells in asthma patients.

**Methods**: Peripheral blood mononuclear cells (PBMCs) were treated with FlaB and then iNKT cells-derived and intracellular cytokines were determined by ELISA and flow cytometry, respectively, following the stimulation with a-galactosylceramide (a-GalCer). Foxp3^+^ iNKT cells were measured. To determine the effect of FlaB-treated dendritic cells (DCs) on iNKT cells, CD14^+^monocyte-derived DCs and T cells from patients with house dust mite-sensitive asthma were co-cultured, in which intracellular cytokines of iNKT cells were determined. In some experiments, IL-10R mAb was used.

**Results**: FlaB treatment reduced the productions of IL-4 and IL-17 from iNKT cells in PBMCs cultures, which effects were ameliorated following the addition of IL-10R mAb. FlaB-treated DCs decreased the frequencies of IL4^+^ and IL-17^+^ iNKT cells, which effects were eliminated after the addition of IL-10R mAb. In contrast, Foxp3^+^iNKT cells were induced by FlaB treatment, which effect disappeared after the addition of IL-10R mAb.

**Conclusion**: FlaB may inhibit Th2- and Th17-like iNKT cells and enhance Foxp3^+^ iNKT cells via DCs in an IL-10-dependent fashion in asthma patients. In patients with asthma phenotype in association with iNKT cells, FlaB will be the effective immunomodulator for iNKT cell-based immunotherapy.

## A93 Clinical and subclinical manifestations of allopurinol – induced severe cutaneous adverse reactions in Vietnam

### Dinh Van Nguyen^1,2^, Hieu Chi Chu^3^, Mui Thi Tran^4^, Christopher Vidal^5^, Suran Fernando^1^, Sheryl Van Nunen^6^, Sy Van Than^4^

#### ^1^Sydney Medical School - Northern, University of Sydney, Australia; ^2^Hanoi Medical University, Vietnam; ^3^Bach Mai Hospital; ^4^Hanoi Medical University; ^5^Royal North Shore Hospital; ^6^Sydney Medical School

##### **Correspondence:** Dinh Van Nguyen – Sydney Medical School - Northern, University of Sydney, Australia

**Background:** Allopurinol, a xanthine oxidase inhibitor, has been used since the 1960s for gout, hyperuricaemia associated with treatment for malignancy and renal calculi due to hyperuricosuria. It is known as the leading cause of severe cutaneous adverse drug reactions (SCARs) comprising Stevens-Johnson Syndrome (SJS)/Toxic Epidermal Necrolysis (TEN) and HyperSensitivity Syndrome (HSS)/Drug-Rash with Eosinophilia and Systemic Symptoms (DRESS) (Halevy et al. 2008). In Vietnam, we observed a high prevalence of allopurinol- induced SCARs, likely due to both its common use and the high prevalence of HLA-B*5801 (6.5% - Hoa et. al. 2008). The role of viral activation in SCARs is well established. We reviewed patients with allopurinol- induced SCARs to reveal possible non-genetic risk factors.

**Methodology:** The clinical history, examination findings and results of laboratory investigations in eighty- eight confirmed cases of SCARs caused by allopurinol seen between 2011 and 2014 were surveyed.

**Results:** A total of 88 patients comprised 33 SJS (37.5%), 3 TEN (3.4%) and 52 HSS/DRESS (59.1%). The mean age was 59.9±14.4 years (median 57.5) (SJS/TEN: 57.92±15.02 vs HSS/DRESS: 61.33±13.887, p =0.284) and a male preponderance was noted (male: female =62:26). Indications for allopurinol were chronic gout (22.7%), acute gout (12.5%), asymptomatic hyperuricaemia (45.5%) and unknown (19.3%). 100% of patients commenced allopurinol at a dose of 300mg or above. Co-medications were diuretics (3.4%), anti- hypertensive agents (18.2%), colchicine (12.5%), digoxin and probenecid (1.1%). Co-morbidities consisted of hypertension (29.5%), renal insufficiency (13.6%), diabetes (9.1%), dyslipidemia (5.7%), cardiac diseases (4.5%) and liver diseases (4.5%). The index-day was 17.5 ± 10.8 days (median 17, range: 1 - 66 days, SJS/TEN: 19.58±13.03 vs HSS/DRESS: 15.98±8.68, p <0.001). Virus serology: Human simplex virus (HSV) was positive in 34/40 (85%) with IgG and 3/40 (7.5%) with IgM; Epstein Barr Virus (EBV) was positive in 34/41 (85%) with IgG, 4/41 (9.8%) with IgM; Cytomegalovirus (CMV) was positive in 35/41 (83.3%) with IgG and 2/41 (4.9%) with IgM. An abnormal creatinine was seen in 51.9%, and an elevated AST and ALT in 44.7% and 62.7% patients respectively. Mortality rate was 2.3% (observed only in TEN). In those with SJS/TEN, the average number of natural cavities affected was 2.28 ± 0.45. Ocular lesions occurred in 25 patients (69.4%). Mean of SCORTEN was 1.69 ± 0.95. In HSS/DRESS, 38 (73.1%) had fever over 38.5 °C, 17 (32.7%) had eosinophilia with a median 1.71 G/L. Only 3 (5.8%) patients had facial oedema observed.

**Conclusions:** The presence of virus infection is very common in allopurinol- induced SCARs patients in Vietnam. A significant number of these SCARs could be prevented by avoiding treatment of asymptomatic hyperuricaemia and reducing the starting dose of allopurinol.

## A94 Time course of serum inhibitory activity for facilitated allergen-IgE binding during house dust mite immunotherapy

### Mulin Feng, Jing Li

#### Guangzhou Medical University, China

##### **Correspondence:** Mulin Feng – Guangzhou Medical University, China

**Background:** Specific allergen immunotherapy (SIT) is an effective treatment for IgE-mediated allergic disease and involve with specific IgG4 (sIgG4) level increase. Elevation of sIgG4 is accompanied by increase in IgG-dependent serum inhibitory activity for IgE-facilitated allergen binding (IgE-FAB) assay.

**Objectives:** As this ‘functional’ assay of inhibitory antibodies may be correlate more closely with clinical outcome, we investigated the time course of serum inhibitory activity for IgE-FAB during different period of *Dermatophagoides pteronyssinus* subcutaneous immunotherapy (Der p-SIT) in rhinitis and/or asthma patient.

**Methods:** This study involved 20 adult patients with allergic rhinitis and/or asthma receiving a 156-week course of Der p-SIT, and 20 adult patients with allergic rhinitis and/or asthma receiving drug therapy only as control. Symptom and medication scores, forced expiratory volume in one second (FEV1), Der p-sIgG4 levels and the serum inhibitory activity at weeks 0, 4, 12, 16, 52, 104 and 156 were analyzed.

**Results:** Rhinitis and/or asthma symptom and medication scores, as well as FEV1% predicted showed improvement at week 52, 104 and 156 than 0 week with significant difference in SIT patients (p<0.05), and the symptom scores is not significant difference between 104 week and 156 week (p>0.05). Levels of Der p-sIgG4 showed a significant increase after 16 weeks of Der p-SIT (p < 0.01) and continued to increase during the 156-week SIT period. Serum obtained during Der p-SIT significantly inhibited Der p-IgE binding to B-cells (IgE-FAB) after 16 weeks of SIT (% relative Der p-IgE complex binding to B cells: 0 w = 104±24%; 4 w =104±20%, 12 w =105±17%, 16 weeks = 53±22%; 52 w = 35±22%; 104 w = 29±18%; 156 w = 27±15%; P<0.001). The IgE-FAB is not significant difference between 104 week and 156 week (p>0.05).

**Conclusion:** Serum sIgG4 levels and inhibitory activity for IgE-FAB increased significantly during SIT. Inhibitory activity for IgE-FAB may be more relevant for clinical efficacy of Der p-SIT.

## A95 Periostin is a novel biomarker in eosinophilic nasal polyps of chronic rhinosinusitis

### Dong-Kyu Kim^1^, Seung-No Hong^2^, Kyoung Mi Eun^3^, Hong Ryul Jin^3^, Dae Woo Kim^2^

#### ^1^Chuncheon Sacred Heart Hospital, South Korea; ^2^Seoul National University Hospital; ^3^Boramae Medical Center

##### **Correspondence:** Dong-Kyu Kim – Chuncheon Sacred Heart Hospital, South Korea

**Background:** Periostin, an extracellular matrix protein, has been known to play an important role in the process of tissue remodeling. Recently periostin has been discovered as a novel mediator in allergic diseases such as bronchial asthma and atopic dermatitis; however, the role of periostin in patients with chronic rhinosinusitis (CRS) remains unclear. Therefore, the objective of this study was to investigte the role of periostin in the pathophysiology of CRS patients.

**Methods:** We investigated periostin expression and its cellular origins in uncinate process mucosa (UP) and nasal polyp (NP) tissues by immunohistochemistry (IHC), quantitative reverse transcription PCR (qRT-PCR), and enzyme-linked immunosorbent assay (ELISA). Correlations between periostin expression and other inflammatory markers, including of interleukin (IL)-5, IL-13, IL-17A, interferon (IFN)-γ, were also explored.

**Results:** Periostin expression was upregulated in NP mucosa from patients with CRSwNP compared with the uncinate process (UP) tissue of control, CRSsNP and CRSwNP patients. Overexpression of periostin in eosinophilic NP compared to non-eosinophilic NP was confirmed by qRT-PCR, and ELISA. There was a positive correlation between periostin protein concentration and Lund-Mackay CT scores in eosinophilic NP.

Double IHC staining showed that tryptase^+^ cells were one of the main sources of periostin among immune cells. In addition, periostin mRNA expression was positively correlated with the expression of tryptase^+^ cells and total IgE homogenate in eosinophilic NP. Overexpression of epithelial integrin αV was detected in NP mucosa from CRSwNP patients compared with UP from control and CRSsNP. Moreover, in eosinophilic NP, the expression of epithelial integrin αV was higher than non-eosinophilic NP and positively correlated with the concentration of periostin. Furthermore, periostin mRNA expression in eosinophilic NP patients was positively correlated with IL-5, IL-13 and negatively related with IL-17A; however, there was no association between those and IFN-γ.

**Conclusions:** Our data suggest a role for periostin in the pathogenesis of nasal polypogenesis, especially in eosinophilic NP. Therefore, periostin protein might be a new treatment target for patients with CRSwNP.

## A96 Dominance of Th1-response in children with refractory mycoplasma pneumoniae pneumonia

### Jun Bao, Yi-Xiao Bao

#### Xinhua Hospital, China

##### **Correspondence:** Jun Bao – Xinhua Hospital, China

**Background:** To investigate DNA copy numbers of Mycoplasma pneumoniae and expressions of helper T (Th) cell producing cytokines in bronchoalveolar lavage fluid (BALF) from children with refractory MP pneumonia (MPP).

**Methods:** Of the 90 enrolled children with MPP, 30 cases were assigned as refractory MPP who failed to respond to at least 1-week treatment with macrolide antibiotics, while the other 60 cases were common MPP. A total of 30 children with congenital bronchial atresia or stenosis were included as controls. ELISA was used to assess BALF levels of IFN-γ, IL-4, IL-8 and TNF-α. RT-PCR was used to determine MP-DNA copy numbers in BALF.

**Results:** Compared with the common MPP group, the refractory MPP group had a significantly higher MP-DNA copy number (3715352 ± 3162 vs. 2570 ± 5495; /ml; P<0.01). Children with refractory MPP achieved significantly increased BALF levels of IFN-γ, IL-8 and TNF-α than those with common MPP and the controls (40.55 ± 22.03 vs. 29.71 ± 11.18 vs. 27.54 ± 9.80; 213.58 ± 80.05 vs. 169.83 ± 83.56 vs. 79.50 ± 55.47; 240.90 ± 68.33 vs. 121.85 ± 63.15 vs. 101.33 ± 42.56; pg/ml; P<0.01). No significant difference in BALF levels of IL-4 was found among the three groups (23.00 ± 11.24 vs. 21.96 ± 11.94 vs. 17.61 ± 10.55; pg/ml; P>0.05).

**Conclusion:** Refractory MPP may be associated with high MP load. Th1 mediated response may be dominant in the development of refractory MPP.

## A97 Studies on the role of CD14 polymorphism among pollen and mold induced asthmatics of kolkata, India

### Sanjoy Podder^1^, Goutam Kumar^2^, Shampa Dutta^1^, Amlan Ghosh^3^

#### ^1^Barasat Government College, India; ^2^Calcutta University; ^3^Presidency University

##### **Correspondence:** Sanjoy Podder – Barasat Government College, India

**Background**: There has been a surge in the incidence of bronchial asthma around the world especially in developing countries like India, because of many factors such as change in ambient air quality, increased air pollution, metamorphic change in living habits and lifestyle and climate. The allergens like pollens, fungi, etc. present in the air plays a pivotal role in pathogenesis of several allergic complaints such as allergic rhinitis, bronchial asthma etc. Studies revealed the complex interactions of genetic and environmental factors are involved in asthma. India is the home to around 15–20 million asthmatics and asthma prevalence is increasing in India especially in Kolkata. To provide the patients with best possible diagnosis and treatment, the identification of offending allergens are of major importance. However early detection of individuals who are genetically at risk of developing pollen and mold allergy is also an essential element to adopt effective avoidance strategies and to design appropriate therapies. As accustomed, the information in this respect is mostly available from developed and western countries while preliminary information from developing countries like India, particularly Kolkata Metropolitan areas is still fragmentary and insufficient. Therefore, present study involved identification of offending outdoor aeroallergens and also associated genetic pathway in nasobronchial asthma among Kolkata population.

**Methods**: Skin-prick test was done among 950 asthmatic patients against 17 common aeroallergens and total serum IgE concentration was measured. PCR-RFLP was done in patients and non-asthmatic control (n=220 in each) to characterize a functional polymorphism, C(-159)T, of CD14, a positional candidate gene for allergy. Association of genetic polymorphisms was made with Clinicopathological conditions.

**Result**: Present study identified *Cocos* as the most predominant outdoor aeroallergen in Kolkata followed by *Caesalpinia* and *Peltophorium*, all of which belong to pollen category. Patients with childhood-onset of asthma were significantly more sensitive towards aeroallergens and had significantly higher serum IgE level than that of adult-onset. No significant difference was found in distribution of SNP genotypes of CD14 among case and control. However among patients, frequency of C allele is significantly higher in childhood-onset group than that of adult-onset and concordantly in former, CC genotype was associated with significant higher level of total serum IgE than CT and TT.

**Conclusion**: In Kolkata, pollen is common outdoor aeroallergen and *Cocos* is predominant among pollens. Childhood-onset and adult-onset of asthma showed significant difference in allergen sensitivity and differential association of CD14 polymorphism might be involved in this process.

## A98 House dust mite allergy - Indian perspective

### Goutam Kumar Saha^1^, Sanjoy Podder^2^, Salil Kumar Gupta^2^

#### ^1^Calcutta University, India; ^2^Barasat Government College

##### **Correspondence:** Goutam Kumar Saha – Calcutta University, India

**Background:** Although the prevalence of allergic diseases are more common in westernized and developed countries, the incidence is increasing in a rapid pace in developing countries like India too, becoming doubled, tripled and even quadrupled in the last few decades. However, data in this regard is still fragmentary except few scattered information in Indian context. In a rough estimate, In India, 250 million people are suffering from one or more allergic manifestations.

**Methods:** During last more than 20 years, studies on different aspects of house dust mite allergy have been carried out on Kolkata population including entomological, clinical and immunological parameter through identification of allergenic mites, allergy skin tests and estimation of total IgE level, and identification of allergen specific IgE antibodies.

**Results:** An inventory of house dust mite fauna of Kolkata, India has been prepared. A total of 53 species belonging to 34 genera, 12 families and 3 orders have been identified, of which 18 species have been recorded for the first time from India and 7 species were identified as new to science. The genus *Dermatophagoides* alone constituted 60% of the total acarine fauna, predominated by *D.pteronyssinus* (47%) followed by *Blomia tropicalis*, *D. farinae* and *Austroglyciphagous geniculatus.* Both *D. pterinyssinus* (DP) and *D. farina* (DF) co-existed in the same habitat and maximum number of mites isolated from an individual dust sample was 13750/gm of dust. Seasonal trend indicates mite count were higher in pre-monsoon and minimum during winter. Preliminary screening through Skin Prick Test showed 83% patients reacted positively towards allergens of mites (either DP or DF), 92% patients had elevated levels of serum IgE and the mean value higher than control sera (p<<0.001). 85% patients of the study group showed allergen specific IgE antibodies against house dust and *Dermatophagoides* mites.

**Conclusions:** The study confirms that DF is the main source of allergen in house dust of Kolkata Metropolis followed by DP. Increased use of heavy curtains, blankets, padded furnitures, sofa sets, soft toys and use of foam mattress instead of conventional cotton mattress favours the growth and multiplication of house dust mites, which ultimately increases the chances and duration of exposure to those indoor allergen. Metamorphic changes in life style including diet and dietary habits, acquisition of Western lifestyle, low standard of indoor environment, increasing air pollution and over all intolerable psychological stress are blamed for such an increased occurrence and frequent recurrences in India.

## A99 Increased expression of purinergic (P2Y12) receptor and cysteinyl leukotriene receptors in the lung tissue of a mouse model of allergic asthma

### Tu/Hoang Kim Trinh, Yoo Seob Shin, Hae-Sim Park, Jing-Nan Liu, Duy Le Pham

#### Ajou University School of Medicine, South Korea

##### **Correspondence:** Tu/Hoang Kim Trinh – Ajou University School of Medicine, South Korea

**Background and objectives:** Cysteinyl leukotrienes C4, D4 and E4 mediate allergic inflammation by interacting with type 1 and 2 cysteinyl leukotriene receptors (CysLT1R, CysLT2R), G protein-coupled receptor (GPR) 99 and indirectly with purinergic receptor P2Y12 (P2Y12R). P2Y12R expressed on platelets and eosinophil granules are required for platelet activation, thus resulting in the recruitment of eosinophils in the lungs ^(1), (2)^. To understand the role of these leukotriene related receptors in allergic asthma, we compared the expressions of CysLT1R, CysLT2R, GPR99 and P2Y12R in an Ovalbumin-induced allergic asthma mouse model.

**Methods:** BALB/c mice were injected intraperitoneally with ovalbumin (OVA) followed by nebulized OVA challenges, from which bronchoalveolar lavage fluid (BALF) cells and lung tissues were collected. Vehicle was treated as a control group. For platelet removal, mice were injected with anti-CD42b antibody. Western blot, immunocytochemistry and immunohistochemistry were applied to evaluate the expressions of these receptors.

**Results:** P2Y12R, CysLT1R and CysLT2R signals were distributed in epithelial lining and lung parenchyma of platelet-depleted and non-platelet-depleted mice, respectively. The expressions of P2Y12R, CysLT1R and CysLT2R were significantly higher in the lung tissue of OVA-sensitized mice than in vehicle-treated mice (*P* = 0.047; *P*= 0.04; *P* =0.045, respectively). The expression ratios of CysLT1R and CysLT2R to P2Y12R were 1.4 and 1.1 to 1 in vehicle-treated mice; 1.3 and 1.1 to 1, in OVA-sensitized mice. P2Y12R, CysLT1R and CysLT2R were localized to eosinophils from BALF and were elevated markedly after OVA challenges in OVA-sensitized mice, while GPR99 was found with the lowest level in BALF cells and lung tissue.

**Conclusions:** Increased expressions of P2Y12R, CysLT1R and CysLTR2 were noted in the lung tissue of a mouse model of allergic asthma. Additional effects of P2Y12R antagonists on CysLTR1 antagonists should be investigated as a future therapeutic target.

References

(1) S.C. Pitchford, Blood, 105 (2005), pp. 2074–2081

(2) Cummings H.E, *J Immunol*, 2013; 191(12), 10.4049/jimmunol.1302187

## A100 Autologous serum skin test in chronic idiopathic urticaria - relationship with autoimmune markers and disease severity

### Hyun-Chang Ko^1^, Byung Soo Kim^2^, Moon-Bum Kim^2^

#### ^1^Pusan National University Yangsan Hospital South Korea; ^2^Pusan National University

##### **Correspondence:** Hyun-Chang Ko – Pusan National University Yangsan Hospital, South Korea

**Background**: The aims of this study were to verify the association between autologous serum skin test (ASST) and laboratory markers for autoimmunity in the patient with chronic idiopathic urticaria (CIU) and to evaluate whether parameters like ASST, thyroid autoantibodies (TA), anti-nuclear antibody (ANA), and serum total immunoglobulin E (IgE) could predict CIU severity including urticarial activity and refractoriness.

**Methods:** Total 878 CIU patients were performed ASST to identify autoreactivity. Further, serum antibodies to thyroglobulin (ATG) and thyroid peroxidase (ATPO), ANA, and total IgE were measured. Clinical severity of CIU was estimated by urticaria severity score (UAS) and maximum level of medication (1∼4) to abrogate wheal and pruritus. We analyzed association among ASST, laboratory markers and clinical severity in different subgroups based on ASST and laboratory markers.

**Results:** A total of 255 (29.0%) patients were tested positive in the ASST and 28/192 (14.6%) patients were tested positive for ATPO or ATG. Positive percent of ANA was 18.6% (106/571) with female preponderance. Serum total IgE level was measured in 521 of the 878 patients and was found to be elevated in 258 (49.5%). When ASST was analyzed in relation to laboratory markers, inverse correlation between ASST and serum IgE level was observed but significantly higher percentage of positive ANA was found in patients with positive ASST (31.5%) compared to patients with negative ASST (13.4%). Correlations of ATPO and ATG with ASST had no significant statistical difference. The patients with positive ASST had significantly higher scores of UAS compared with the patients with negative ASST. However, there was no significant statistical difference between maximum level of medication and ASST. Maximum levels of medication in the patients with elevated IgE were significantly higher than those with normal IgE.

**Conclusion:** Significant association between ASST and thyroid autoimmunity was not identified in this study. Patients with positive ASST had more disease activity but patients with elevated total IgE were found more refractory to lower level of medication like antihistamine.

## A101 Anxiety and depression levels in severe asthma patients treated with omalizumab

### Ömer Özbudak, Fatih Üzer

#### Akdeniz University Faculty of Medicine, Turkey

##### **Corresponcence:** Ömer Özbudak – Akdeniz University Faculty of Medicine, Turkey

**Introduction**

Asthma, because of its chronic nature, may adversely affect the life quality of patients leading to mental disturbances. Anxiety and depression can be accure more than on these patients compared with the healthy population. Life quality survey can be used to evaluate effect of asthma in the terms of physical, psychological and social function on the patients life. In particular, the importance of emotional factors come to the forefront more in the case which symptoms can not be controlled. Anxiety is the most common psychological disorders among patients who have respiratory system disease.

**Materials and methods**

We aimed to compare anxiety and depression levels of the patients with severe asthma before and after the omalizumab treatment. Patients’ anxiety levels were measured with state trait anxiety inventory (STAI) and depression levels were measured with beck depression scale.

**Findings**

Five male (%25), fifteen female (%25) patients enrolled in study. The average age of the patients were 50,25. The average score of beck depression scale of patients was 25,35 and 8,55 before and after treatment, relatively. The score was higher before treatment, it was statically meaningful when it compared with after treatment scores (p<0.001). The STAI average score was 52,05 before treatment and 42.95 after it. There was a statistically meaningful difference between the anxiety score average before and after the treatment (p<0.0001). The anxiety score was higher before the treatment.

Failure in symptom controlling in asthma can lead to depression. We think that these patients should be examined for psychological disorders. It may improve the quality of life.

**Keywords:** Anti-IgE; Asthma In Adults; Quality Of Life

## A102 Economic burden of refractory chronic spontaneous urticaria on Kuwait health system

### Mona Al-Ahmad^1^, Maryam Alowayesh^2^, Norman Carroll^3^

#### ^1^Al-Rashed Allergy Center, USA; ^2^Faculty of Pharmacy; ^3^School of Pharmacy

##### **Correspondence:** Mona Al-Ahmad – Al-Rashed Allergy Center, Kuwait

**Introduction & Objectives:** Chronic spontaneous urticaria (CSU) is a common debilitating problem worldwide. Despite its prevalence in the Middle East, very little data is available on the economic impact of CSU on the public health system. This study evaluates the direct medical costs of treating refractory CSU patients in Kuwait. It also evaluates the budget impact of omalizumab (monoclonal anti-IgE antibody) use in these patients.

**Methods:** Prevalence of CSU was estimated through the Delphi method. Data regarding drug utilization and health care system utilization was collected retrospectively from charts of refractory CSU patients who were followed at the Al-Rashed Allergy center in Kuwait. Costs were calculated from a health system perspective. One-way sensitivity analyses were conducted on the price and utilization of each cost component.

**Results:** Before omalizumab use, the total direct costs of treating 1,293 refractory CSU patients was estimated to be 1,072,837 KD (US$ 3,570,185) per year, corresponding to around 829.7 KD per patient per annum (US$ 2,761). The total cost was principally generated by outpatient visits 1,072,837 KD; which corresponds to 82.45% of the total cost). After omalizumab use, the cost was estimated to be 4,654,800 KD (US$ 15,490,234) per year, corresponding to around 3,600 KD per patient per annum (US$ 11,980). The total cost was principally generated by omalizumab costs 4,762,895 KD; which corresponds to 97% of the total cost. All other direct costs of treating CSU patients were decreased after the use of omalizumab. Conventional medication costs and hospitalization costs were reduced by 90% and ER costs were reduced by 97%. Cost estimates were most sensitive to variations in the price and utilization of outpatient visits and the price of omalizumab.

**Conclusion:** The economic burden of refractory CSU in Kuwait is high. The introduction of omalizumab on the health care system is costly because of its high price; however, omalizumab has proven to be effective and is driving all other direct costs down.

## A103 IgE-mediated maize allergy in India: A 28 kd protein responsible for food-induced allergic reaction

### Anand Bahadur Singh

#### Institute of Genomics and Integrative Biology, India

**Background**

Maize is a major crop grown in India and is consumed in various forms. Although many patients report allergic symptoms to maize but a systematic study on its allergenic roperties has been lacking. The investigation was aimed at to find out maize sensitization in Indian patients of respiratory allergy and Characterize allergens of clinical significance by clinico immunologic evaluation.

**Methods**

Patients attending allergy clinic for respiratory allergy were screened for Maize sensitization by SPT and by allergen specific IgE by ELISA. The IgE binding epitopes were identified by Immunoblot after transferring SDS- PAGE separated proteins on to NC membrane. The 28kd major allergen was identified in Indian patients and was further subjected to *in situ* trypsin digestion and MALDI-TOF to determine the molecular masses of peptides and sequence similarity. Cross reactivity of maize seed extract was evaluated with extracts of pea nut, rice, and unrelated *Putranjiva roxburghii*pollen extract by Immunoblot inhibition studies.

**Results**

Antigen extracted from maize seeds contained 4.5±0.7 mg/ml protein. The extract was subjected to SDS protein profile with 12 protein bands in molecular weight range of 152 - 10 kD. Five protein fractions of 68, 38, 25, 14 & 10 kD are recorded as stable under 60 min of simulated gastric fluid (SGF) digestion assay. Using clinically maize positive patient sera and significantly raised maize specific IgE titer and IgE binding activity of 28 kD protein smear is recorded. Cross Inhibition studies suggested cross reactivity reactivity of maize with rice, on account of the complete disappearance of observed 28 kD immunoreactive protein smear. However biochemical characterization of the smear corresponds to two protein bands of 25kD and 19 kD on 1D gel which on MS spectra are observed with hypothetical molecular weight of 14.3 kD and 11.6 kD, sharing sequence homology greater than 41% with un annotated rice seed proteins in database.

**Conclusion**

Two important immuno reactive protein fractions of maize have been identified which are speculated to be belonging to major family of endosperm proteins “zeins.

## A104 Liposomal encapsulation of house dust mite allergens and dexamethasone modulates allergic response in a murine model of asthma

### Yordanis Pérez-Llano^1^, María Del Carmen Luzardo Lorenzo^1^, Wendy Ramírez González^2^, Carlos Calcines Cruz^1^, Rady Laborde Quintana^1^, Alain Morejón^3^, Virgilio Bourg^2^, Marilé Hechavarría Stoker^1^

#### ^1^University of Havana, Havana, Cuba; ^2^National Center of Bioproducts, Mayabeque, Cuba; ^3^National Center of Bioproducts, Bejucal, Mayabeque, Cuba

##### **Correspondence:** Yordanis Pérez-Llano – University of Havana, Havana, Cuba

**Background:** House dust mite (HDM) allergens are a major cause of asthma worldwide. HDM extracts or purified allergens are the current treatment of choice known as specific immunotherapy. The aim in such strategy is to drive immune reaction away from the allergic Th2 response by inducing Th1 and/or Treg cellular response. In that regard, Toll Like Receptor-activating adjuvants have been used for inducing Th1 response, but adjuvants capable of inducing the somehow safer Treg response are poorly investigated.

**Methods:** We describe the anti-allergic properties, in a therapeutic murine model of asthma, of HDM allergens Der s1 and Der s2 co-encapsulated with dexamethasone into dehydration-rehydration vesicles (DRV). Dexamethasone is a cortisol analogue with immune-suppressor activity known to induce specific Treg response, still with some adverse effects associated to systemic or prolonged use. Optimal lipid composition in terms of physical-chemical and anti-allergic properties was assessed using DRV liposomes of cholesterol and different phosphatidyl-cholines (PC) encapsulating Der s1+Der s2 allergens. Dipalmitoil-PC:cholesterol liposomes encapsulating different doses of dexamethasone were used to assess anti-allergic effects.

**Results:** All liposomal compositions produced similar vesicle size, protein encapsulation and overall safe profile in treated mice. Preliminary results indicate that encapsulation of HDM allergens Der s1+Der s2 and dexamethasone into liposomes diminishes allergic response traits such as IL-5 interleukin and IgG1 and IgE antibody levels.

**Conclusion:** These results highlight the possibility of using delivery systems such as liposomes to modulate immune response and to prevent adverse reactions to free or soluble pharmacological compounds like dexamethasone.

## A105 Immune Suppressive Effects of Tonsil-Derived Mesenchymal Stem Cells for Eosinophilic Rhinosinusitis with Nasal Polyps in a Mouse Model

### Jun-Sang Bae, Ramachandran Samivel, Eun-Hee Kim, Ji-Hye Kim, Ji-Hun Mo

#### Dankook University, South Korea

##### **Correspondence:** Jun-Sang Bae – Dankook University, South Korea

**Background:** Chronic rhinosinusitis with nasal polyps (CRSwNP) is one of the more prevalent chronic inflammatory diseases with significant impact on morbidity and quality of life, yet little is known about its pathogenesis.

**Objective:** We sought to evaluate the immunomodulatory effects of tonsil derived mesenchymal stem cells (T-MSC) in a mouse model of eosinophilic rhinosinusitis with nasal polyp(ERSwNP).

**Methods:** The effect of T-MSCs was evaluated in 32 BALB/c mice that were randomly divided into 4 groups (negative control group; nasal polyp group; T-MSC group and T-MSC(AD) group(T-MSC incubated with adipogenic differentiated medium)). After induction of OVA- induced ERSwNP model, T-MSCs were administered intravenously (T-MSC and T-MSC(AD) groups) on weeks5 to 12 (one time per week)and subsequent OVA+SEB (three times per week in OVA and one time per week in SEB) challenge was conducted until 12 weeks. We studied mRNA and protein expression profiles of cytokine, chemokine and adhesion molecules in nasal mucosa, spleen and lymphnode using molecular, biochemical, histopathologicaland immunohistological methods.

**Results:** Intravenous injection of T-MSCs significantly reduced allergic symptoms, eosinophil, neutrophil, nasal polyp count and serum OVA specific-IgG1 levels. Moreover, the nasal, lymphnode and systemic Th2 cytokine profile and nasal innate cytokines such as IL-25 and IL-33, and chemokines (CCL11, CCL24, Cxcl1, CxCl2, ICAM1 and VCAM1) expression were reduced in T-MSCs injected groups, as compared to the nasal polyp group. Usually T-MSC(AD) group showed better inhibitory effects of inflammation than T-MSC group. In addition, our results showed that the T-MSCs injected groupssignificantly increased IL-10 and Treg positive cells (CD4^+^CD25^+^FoxP3^+^ cells) in cervical lymphnode, as compared to the nasal polyp group.

**Conclusion:** We demonstrate the administrationof T-MSCs effectively reduced polyp formation, inflammatory cell influx, cytokine profile, chemokine molecule expression, and T-cell subset distribution, suggestive of the mechanism of reduced CRS inflammation and less polyp formation in mouse model of ERSwNP. Therefore, T-MSC treatment is potentially an alternative therapeutic modality in CRSwNP.

## A106 Second line treatments of dermographic urticaria refractory to antihistamines

### Keiko Hanaoka, Michihiro Hide, Akio Tanaka, Makiko Hiragun, Mikio Kawai

#### Hiroshima University, Japan

##### **Correspondence:** Keiko Hanaoka – Hiroshima University, Japan

**A) Background**

Dermographic urticaria(DU)is a subtype of urticaria characterized by strong itch and wheals induced by mechanical scratching. Oral histamine H1-receptor antagonist (antihistamine) is recommended as the first line treatment and may reduce symptoms of a certain population of the patients. However, DU of many patients are refractory to antihistamines and seriously impair their QOL.

Several medications, such as high dose antihistamines, montelukast and cyclosporine, are suggested as the second line therapies in all Japanese, European and American guidelines. However, clinical evidences of these treatments were mostly obtained for chronic spontaneous urticaria, and the effectiveness of them has not been specifically studied on DU. We here report 27 cases of antihistamine-resistant DU treated with the second line medications suggested in the guidelines.

**B) Methods**

Thirty-five patients who visited Hiroshima University Dermatology clinic from January 2009 to April 2015 for the treatment of DU were studied retrospectively.

**C) Results**

DU in 27 patients were refractory to standard doses of antihistamines. However, all or most symptoms disappeared in 7 out of 27 patients who increased the dose of an antihistamine up to twice. Ten out of 21 patients who were treated with both an antihistamine and montelukast cleared the symptoms. Other 10 patients whose symptoms had not subsided by high doses of antihistamines and montelukast additionally took cyclosporine. In six of them, most symptoms diminished, but two of them did not show any change. The other two patients dropped out because of side effects.

**D) Conclusions**

Approximately 77% (27 out of 35) of the patients with DU was refractory to a standard dose of antihistamine. However, 85 % (23 out of 27) were satisfactorily treated by a higher dose of antihistamine and/or a combination with other medications. The second line treatments suggested in the guidelines for urticaria are worth trying for DU refractory to antihistamines.

## A107 Diagnostic Value of Specific IgE to Peanut and Ara h 2 in Korean Children with Peanut Allergy

### Kwanghoon Kim^1^, Hye-Young Kim^1^, Jihyun Kim^2^, Kangmo Ahn^2^, Youngshin Han^2^

#### ^1^Medical Research Institute of Pusan National University Hospital, South Korea; ^2^Samsung Medical Center

##### **Correspondence:** Kwanghoon Kim – Medical Research Institute of Pusan National University Hospital, South Korea

**Background:** There have been only few reports of peanut allergy and peanut component-resolved diagnostics (CRD) in Korean children. In the present study we tried to establish the diagnostic decision point (DDP) of peanut- sIgE antibodies for predicting the outcome of oral food challenge (OFC). In addition, we evaluated the usefulness of IgE antibodies to recombinant peanut components (Ara h 1, 2, 3, 8, and 9) in diagnosing peanut allergy.

**Methods:** Korean children aged over 12 months with suspected IgE-mediated peanut allergy were enrolled. The diagnosis for peanut allergy was confirmed by an open OFC or through the presence of anaphylaxis history. The cutoff levels of sIgE for peanut and peanut components were determined by analyzing the receiver operating characteristic curves.

**Results:** Forty-eight children (22 boys and 26 girls) with suspected peanut allergy were analyzed. The previously established DDP for peanut-sIgE antibodies (>14 kU/L) showed the sensitivity of 22.7%, specificity of 100%, PPV of 100%, and NPV of 60.4% in our study population. The median levels of peanut-sIgE (5.44 kU/L vs. 1.14 kU/L, *P*<0.0001) and Ara h 2–sIgE (0.8 kU/L vs. 0.0 kU/L, *P*<0.0001) were statistically significant higher in the peanut-allergic group than in the peanut-tolerant group. The peanut-sIgE concentration indicating a positive predictive value (PPV) of 100% was 10.3 kU/L. The Ara h 2-sIgE level of 4.03 kU/L showed PPV of 100%.

**Conclusion:** Our result showed that the previously established DDP of peanut-sIgE level may be useful predictor for the outcomes of OFC in Korean children suspected of peanut allergy and Ara h 2 is the most important allergen in Korean children with peanut allergy. The cutoff levels for peanut (10.3 kU/L) and Ara h 2 (4.03 kU/L) might be helpful for the diagnosis of peanut allergy in Korean children.

## A108 Inappropriate amounts of topical tacrolimus applied on Korean patients with eczema

### Gun-Wook Kim^1^, Hyun-Chang Ko^2^, Byung Soo Kim^1^, Moon-Bum Kim^1^, Margaret Song^1^

#### ^1^Pusan National University, South Korea; ^2^Pusan National University Yangsan Hospital

##### **Correspondence:** Gun-Wook Kim – Pusan National University, South Korea

**Background:** The limited efficacy of topical tacrolimus may result from insufficient frequency of application or amount applied in eczema patients.

**Objective:** To investigate the frequency of application and amount of use of topical tacrolimus in patients with various types of eczema.

**Methods:** The frequency of application and the applied amount of topical tacrolimus were assessed over 2 weeks.

**Results:** A total of 200 eczema patients completed this study. The average number of applications per day was 1.75 ± 0.53, despite instructions to apply the topical tacrolimus twice daily. With respect to the frequency of application, 147 (73.5%) and 122 (61.0%) of patients followed the prescription in the first and second weeks, respectively. The average amount applied per 2% of total body surface area was 0.54 ± 0.52 g. Only 53 (26.5%) patients applied between 80% and 120% of expected amount of topical tacrolimus.

**Limitations:** The frequency of application was self-reported, possibly resulting in limited accuracy.

**Conclusion:** Korean patients with eczema tend to apply topical tacrolimus less frequently and in inappropriate amounts. Clear instructions regarding both the frequency and amount of application are needed to improve the therapeutic outcome with treatment with topical tacrolimus.

## A109 Identification of an IgG1-mediated anaphylaxis marker and its application in evaluating the antigenicity of infant formulas

### Takeshi Matsubara, Hiroshi Iwamoto, Yuki Nakazato, Kazuyoshi Namba, Yasuhiro Takeda

#### Morinaga Milk Industry Co., Ltd. Japan

##### **Correspondence:** Takeshi Matsubara – Morinaga Milk Industry Co., Ltd. Japan

**Background:** In clinical practice, the basophil activation test (BAT) is thought to be a safe and sensitive test for diagnosing IgE-mediated allergic disease. We tested whether the BAT can be used in mice. In the course of developing a murine BAT using CD200R1 as an IgE-mediated activation marker, we found that the expression of a murine basophil identification marker, CD200R3, on antigen-sensitized basophils decreased following specific antigen challenge. Interestingly, this decrease did not always harmonize with increased CD200R1 expression. Since IgG, as well as IgE, contributes to mouse anaphylaxis, we hypothesized that the decrease in CD200R3 on basophils was induced by IgG-mediated cell activation.

**Objective:** We aimed to establish whether CD200R3 is a marker of IgG-mediated basophil activation and whether its expression is correlated with IgG-mediated anaphylaxis in a mouse model. Furthermore, we attempted to evaluate whether the BAT, using CD200R1 and CD200R3 as basophil activation markers, indicates the antigenicity of cow’s milk-based infant formulas, compared with a systemic anaphylaxis test in mice.

**Methods:** Mouse basophils were stimulated via FcεRI and FcγRs, and levels of CD200R1 and CD200R3 were analyzed by flow cytometry. Basophils derived from naïve mice were passively sensitized with antiserum for β-lactoglobulin (β-LG) or an IgG/IgG subclass-depleted antiserum, and challenged with β-LG. Systemic anaphylaxis was induced by i.v. injection of anti-FcγRIII/II monoclonal antibody, and CD200R3 expression on peripheral basophils was analyzed. To evaluate the antigenicity of infant formulas, basophils from mice actively sensitized to β-LG or casein were stimulated with serially diluted conventional, partially hydrolyzed, or extensively hydrolyzed infant formulas. In addition, systemic anaphylaxis was assessed following i.v. injection of formula into the immunized mice.

**Results:** Stimulation via FcεRI induced a significant increase in CD200R1 expression but had only a small effect on that of CD200R3. However, anti-FcγRIII/II stimulation strongly reduced CD200R3 expression. In passive sensitization experiments, the decrease in CD200R3 expression induced by antigen challenge was canceled by the depletion of IgG or IgG1. Intravenous injection of anti-FcγRIII/II led to CD200R3 down-regulation on basophils, accompanied by a drop in rectal temperature. Basophil activation was induced by the conventional but not the extensively-hydrolyzed formula. The partially hydrolyzed formula induced basophil activation somewhat weakly. Induction of systemic anaphylaxis by formula was correlated with the BAT results.

**Conclusions:** Use of CD200R1 and CD200R3 as activation markers enables the evaluation of IgE- and IgG-mediated murine basophil activation, respectively, and the BAT system would be useful for evaluating food antigenicity.

## A110 Nitric oxide as a screening tool for evaluation of postoperative state of chronic rhinosinusitis

### Jae Hoon Lee, Woo Yong Bae

#### Department of Otorhinolaryngology-Head and Neck Surgery, Dong-a University College of Medicine, South Korea

##### **Correspondence:** Jae Hoon Lee – Department of Otorhinolaryngology-Head and Neck Surgery, Dong-a University College of Medicine, South Korea

**Background:** Nitric oxide might have a various roles in development or defense of the upper airway inflammation. Fractional exhaled nitric oxide (FeNO) and nasal nitric oxide (nNO) are used as a screening tool to evaluate the airway inflammation such as chronic rhinosinusitis (CRS), allergic rhinitis and so on. But whether the level of FeNO or nNO is increased or decreased at each disease is not evident. This study was performed to assess whether FeNO can be used the screening tool for evaluation of CRS patients and whether it can be used to evaluate postoperative state of CRS.

**Methods:** Thirty patients with CRS and twenty normal control group were recruited. The FeNO measurement was performed using a handheld electrochemical analyzer (NObreath®) through the nasal and oral route and the mean value of the 3 consecutive measurements was recorded. Nasal FeNO and oral FeNO were checked preoperatively and postop-1 month and 3 months.

**Results:** Preoperative nasal and oral FeNO (nasal: 62.02 ± 45.03 ppb, oral: 30.63 ± 17.71 ppb) in patients with CRS were significantly higher than normal control group (nasal: 25.53 ± 10.52 ppb, oral: 14.45 ± 7.69 ppb, p=0.0001). Nasal and oral FeNO were decreased after operation in time manner. Three month after operation, nasal FeNO and oral FeNO were significantly decreased compared to preoperative level (nasal: 62.02 ± 45.03 ppb vs. 37.68 ± 26.26 ppb, oral: 30.63 ± 17.71 ppb vs. 24.79 ± 19.24 ppb, p<0.05).

**Conclusions:** The FeNO could be used for the screening tool to assess the evaluation of CRS patients. Also it can be used to evaluate postoperative state of CRS through the change of FeNO level.

## A111 Comparison of different medical treatment options for crswnp: Doxycycline, methylprednisolone, mepolizumab, omalizumab

### Els De Schryver, Lien Calus, Philippe Gevaert, Thibaut Van Zele, Claus Bachert

#### Ghent University, Belgium

##### **Correspondence:** Els De Schryver – University Hospital Ghent, Belgium

**Background**: Chronic rhinosinusitis with nasal polyps is hard to treat. The therapeutic effect of doxycycline, oral glucocorticoids, mepolizumab and omalizumab with significant reduction of nasal polyp score was previously investigated by 3 randomized controlled trials. The aim of this study is to compare the effect of these treatments on polyp score, symptom scores and inflammatory parameters.

**Methods**: In total, 100 patients were randomly assigned to receive doxycycline for 20 days (n=14), methylprednisolone in decreasing doses for 20 days (n=14), mepolizumab 2 single intravenous injections (n=20), omalizumab 2 to 4 subcutaneous doses (n=15) or placebo (n=37) in three separate clinical double-blind, placebo-controlled trials. Participants were followed for 8 weeks. Endoscopic evaluation of nasal polyp score, assessment of symptom score and measurement of markers of inflammation in nasal secretions and serum occurred at baseline, week 4 and week 8. All treatments were completed at week 4, except for 2 patients who received a fourth subcutaneous dose of omalizumab at week 6.

**Results**: All treatment options significantly reduced nasal polyp score as compared to baseline, but not placebo. Methylprednisolone has initially the most dramatic effect on symptom scores, but after cessation of treatment symptom scores worsen progressively and return to baseline after 4 weeks. Mepolizumab, doxycycline and methylprednisolone each had a specific effect on local and systemic inflammatory markers. Omalizumab did not alter eosinophilia or markers of inflammation.

**Conclusions**: Omalizumab, mepolizumab and oral doxycycline cause a long-term reduction in nasal polyp size, whereas methylprednisolone initially causes the strongest reduction in polyp size but recurrence occurs earlier. Total symptom scores parallel this trend. Doxycyline works on eosinophilic and neutrophilic inflammation in nasal polyps, whereas the effect of mepolizumab and methylprednisolone is most apparent on eosinophilic markers. Omalizumab did not reduce markers of eosinophilic inflammation, nor neutrophilic inflammation in nasal secretions. The beneficial effect of omalizumab seems to be mediated through a direct effect on IgE or their receptor, rather than through an effect on markers of the eosinophilic cascade.

## A112 Successful treatment of steroid resistant asthma model by blocking CD28 signal

### Akio Mori, Satoshi Kouyama, Miyako Yamaguchi, Yo Iijima, Akemi Abe-Ohtomo, Hiroaki Hayashi, Kentaroh Watai, Chihiro Mitsui, Chiyako Oshikata, Kiyoshi Sekiya, Takahiro Tsuburai, Mamoru Ohtomo, Yuma Fukutomi, Masami Taniguchi

#### National Hospital Organization, Sagamihara National Hospital, Japan

##### **Correspondence:** Akio Mori – National Hospital Organization, Sagamihara National Hospital, Japan

**Rationale:** To investigate the role of helper T (Th) cells in steroid resistant (SR) asthma, steroid sensitive (SS) and resistant (SR) Th clones were selected *in vitro*, and then adoptively transferred into unprimed mice. Effect of CTLA4-Ig was analyzed both *in vitro* and *in vivo*.

**Methods:** For *in vitro* evaluation, ovalbumin (OVA) reactive Th clones were cultured with antigen presenting cells and OVA in the presence of various concentrations of dexamethasone (DEX). Proliferative responses of Th clones were measured by ^3^H-thymidine incorporation. For *in vivo* assessments, unprimed BALB/c mice were transferred with Th clones, challenged with OVA, and administered with DEX subcutaneously. Bronchoalveolar lavage fluid (BALF) was obtained 48 hr after challenge, and the number of infiltrating cells was differentially counted. CTLA4-Ig was administered through nasal inhalation or venous injection.

**Results:** SS and SR clones were selected based on the effect of DEX on the proliferative responses of antigen-stimulated Th clones. Airway infiltration of eosinophils and lymphocytes of mice transferred with SS clones were effectively inhibited by the administration of DEX. In contrast, those of mice transferred with SR clones were not significantly inhibited by DEX. Administration of CTLA4-Ig significantly suppressed the proliferation of DEX-treated SR clones *in vitro*, and the eosinophil infiltration of SR asthma model transferred with SR clones *in vivo*.

**Conclusions:** Steroid sensitivity of Th clones assessed *in vitro* was consistent with that of adoptively transferred asthma model assessed *in vivo*. Costimulatory signal mediated through CD28 is crucial for the induction of steroid resistance both *in vitro* and *in vivo*.

## A113 Serum periostin levels was not associated with allergic rhinitis and allergic sensitization in Korean children

### Ju Wan Kang^2^, Jeong Hong Kim^1^, Jeong Hong Kim^2^, Keun-Hwa Lee^2^, Hye-Sook Lee^2^, Seong-Chul Hong^2^, Jaechun Lee^3^

#### ^1^Jeju National University Hospital, South Korea; ^2^Jeju National University, South Korea; ^3^Jeju National University School of Medicine

##### **Correspondence:** Ju Wan Kang – Jeju National University Hospital, South Korea

**Background**

Periostin is a matricelluar protein, which was synthesized in airway epithelial cells induced by interleukin 4 and 13. Recently, serum periostin level was suggested as a biomarker of TH 2 airway inflammation and a prognostic indicator of asthma treatment. However, serum periostin level in allergic rhinitis was not elucidated well, we aim to investigate the relationship between serum periostin and allergic rhinitis in Korea children.

**Methods**

A total of 561 children (273 boys, 288 girls) aged 11-12 years were enrolled in this study. Serum periostin level was measured and the skin prick test was performed with 26 aeroallergens commonly found in Korea. Other information was collected, including sex, age, BMI, parental allergy history, and parental smoking status. Multivariate analysis was used to confirm the association between serum periostin level and allergic rhinitis.

**Results**

Of the 561 children, 217 children showed allergic rhinitis symptoms during past 12 months and 283 children showed positive skin prick test to at least one allergen. Serum periostin level was not differ between allergic sensitization group and control group (49.98 ± 12.98 vs 49.47 ± 13.58). When we divided all subject into 4 groups; control (A; n=124), sensitization without allergic rhinitis symptoms (B; n=52), allergic symptoms without sensitization (C; n=36), and allergic rhinitis symptoms with sensitization (D; n=72). There were no statistical difference of mean value between 4 groups (A-D; 47.81 ± 13.66, 44.56 ± 11.68, 49.38 ± 15.51, and 47.26 ± 11.4). Also, we could not find the significant association between serum periostin and allergic rhinitis in multivariate analysis.

**Conclusions**

Allergic rhinitis and allergic sensitization in Korean children did not influence the serum periostin level. Further studies were needed to elucidate the meaning of serum periostin level in allergic rhinitis.

## A114 Roles of ADAM10 and ADAM17 in allergic rhinitis

### Ji Won Seo, Jae Hoon Lee, Woo Yong Bae

#### Department of Otorhinolaryngology-Head and Neck Surgery, Dong-a University College of Medicine, South Korea

##### **Correspondence:** Ji Won Seo – Department of Otorhinolaryngology-Head and Neck Surgery, Dong-a University College of Medicine, South Korea

**Background:** A disintegrin and metalloproteases (ADAMs) are a multi-functional gene family, they contribute to the homeostasis of the extracellular matrix, transduction of specific intracellular signals, organogenesis, inflammation, tissue remodeling, adhesion, and cell migration. ADAM17 is the best characterized of the sheddases, and its putative substrates are widespread, including various inflammatory modulators. ADAM10 is the most similar to ADAM17 in terms of protein sequence and the structural properties of their catalytic domains.

**Objective:** To assess the roles of ADAM 10 and ADAM 17 in allergic rhinitis by assaying for expression of these materials.

**Methods:** The expression of ADAM 10 and 17 was investigated in the allergic nasal mucosa compared with non-allergic nasal mucosa. Tissue samples were analyzed by real time polymerase chain reaction (PCR) and Western blotting.

**Results:** The ADAM17 mRNA and protein levels were significantly lower in allergic nasal mucosa than in the non-allergic nasal mucosa (*P* < 0.05). The ADAM10 mRNA did not differ significantly between allergic nasal mucosa and non-allergic nasal mucosa. ADAM10 protein levels were significantly higher in the non-allergic nasal mucosa than in the allergic nasal mucosa (P < 0.05).

## A115 Mechanism of oral and topical polyprenol action in atopic dermatitis

### Ivans Sergejs Kuznecovs, Galina Kuznecova

#### Preventive Medicine Institute, Latvia

##### **Correspondence:** Ivans Sergejs Kuznecovs – Preventive Medicine Institute, Latvia

**Background:** Atopic dermatitis (AD) presence and activity correlates with loss of filaggrin. Dysregulation of DPAGT1 (Dolichyl-phosphate (UDP-N-acetylglucosamine) N-acetylglucosaminephosphotransferase 1 (GlcNAc-1-P transferase) causes disturbances in filaggrin expression. The findings indicate that DPAGT1 overexpression in keratinocytes treated with IL-4 and IL-13 can be overcomed by PP, which provides a dolichyl phosphate substitute for DPAGT1 normal expression, N-glycosylation and filaggrin loss prevention. The aim of the present study is to investigate the clinical efficacy of Polyprenol (PP) in children with AD.

**Methods:** SCORAD index was used to measure the severity of the disease and to evaluate the effect of treatment in 100 children (6-24 months old). Leukotriene E4 and dolichol (Dol) were assayed in urinal excretion, immunoCAP total IgE levels were measured in serum, dolichol phosphate N-acetyl-glucosamine-1 phosphate transferase (GPT) activity was defined in dermal fibroblasts by metaboling labeling (ML) method with [2-(3)H]-mannose. PP (1 mg/kg/day, per os) and topical ointment with PP 5% were given in a randomized, double-blind, placebo-controlled study. The effect of the treatment was evaluated weekly up to six months.

**Results:** Initially patients with AD were found to have a statistically significant increase in leukotriene E4 (4-fold) and Dol (6,2-fold) excretion, total Ig E level and GPT activity in fibroblasts in comparison to controls. Overexpression of DPAGT1 was 5-fold higher in AD skin biopsies than in normal skin biopsies. AD cells differ from normal one in filaggrin content lost by 3-4 times. IL-4 and IL-13 cause overexpression and abberant N-glycosylation of filaggrin in DPC. The study showed overexpression of DPAGT1 and 6-fold DPC intermediates decrease in keratinocytes in presence of IL-13 and 2-fold in presence of IL-4 cells. The normalization up to 90% of Dol excretion was achieved after 2 weeks of treatment, IgE and E4 in 3 weeks, GPT after one month in 70% of patients with remission for more than 6 month. Significant difference in AD scores between PP and placebo (P <0.01) was recognized.

**Conclusion:** The present study demonstrates alleviation of AD in children with the use of oral and topical Polyprenol. The mechanism of activity involves the main links of AD pathogenesis.

## A116 Technical and clinical validation of a mobile chamber for allergen exposure tests

### Karl-Christian Bergmann^1^, Torsten Zuberbier^2^, Joseph Salame^1^, Torsten Sehlinger^3^, Georg Bölke^2^

#### ^1^Allergy-Centre-Charité, Germany; ^2^Charité-Universitaetsmedizin Berlin; ^3^Bluestone Technology Gmbh, Woerrstadt

##### **Correspondence:** Karl-Christian Bergmann – Allergy-Centre-Charité, Germany

**Rationale:** The performance of a newly developed mobile allergen exposure chamber, allowing multicenter trials with exactly the same standard of procedure, had to be validated from the technical and clinical point of view. Technical parameters included particle (e.g. pollen) dispersion stability and spatial particle distribution at each subject position during a test period, temperature, O_2_ and CO_2_ and relative humidity stability. Since the chamber uses individual pollen exposure, each seating position has its own particle source, and all particle related parameters had to be measured at each seating position. To be accepted as an appropriate alternative to natural allergen exposure for clinical trials the clinical validation must document a high reliability of provoked symptoms in repeated provocations.

**Methods:** Temperature, relative humidity, O2 and CO2 levels within the chamber have been monitored, with and without subjects being present in the chamber. These tests have been executed during several days, at outside temperatures ranging from -2°C to 26°C. The pollen distribution was validated using sedimentation measurements at 22 positions and at various particle concentrations. For all particle tests, grass pollen have been used**.** The chamber was used for exposure with grass and birch pollen in adult subjects with or without allergic symptoms due to birch and/or grass pollen during the last two seasons. Exposure for 90 - 240 min has been done outside the seasons. Nasal, conjunctival and bronchial symptoms were recorded as subjective parameters every 10 min. As objective parameters spirometry, peak-flow, exhaled nitrogen oxide (FeNO), peak nasal inspiratory flow and nasal secretion were obtained at baseline and after exposure.

**Results:** Measurements showed a temperature variance of +/- 0.5K and humidity variance of +/- 3% with or without subjects being present. In a circular area with a diameter of 50 cm, the particle concentration is +/- 16% of the defined concentration and the accuracy remains over the course of 4 hours. No particles were found in the breathable air of non-active seating positions. Refloating measurements showed no particle concentration increase. The repeated exposures (up to four times) with birch and grass pollen in different concentrations elicited reproducible clinical symptoms on all the three organs and significant differences between placebo and verum pollen exposure. Generally, the symptoms started to occur after 10-30 min. and reached a plateau following 70 - 90 min of continuous exposure to pollen. Strongest symptoms were of nasal origin.

**Conclusions:** The novel Mobile Allergen Exposure Chamber fulfills the need for a reproducible and very well controlled pollen exposure and is regarded to be appropriate for allergen immunotherapy studies phase one and two, pharmaceutical drugs/anti-allergic drugs.

## A117 The association between serum lead level and total immunoglobulin e according to allergic sensitization

### Yoo Suk Kim^1^, Jung Hyun Chang^2^, Jeong Hong Kim^3^, Ju Wan Kang^3^

#### ^1^Ajou University School of Medicine, South Korea; ^2^National Health Insurance Corporation Ilsan Hospital; ^3^Jeju National University

##### **Correspondence:** Yoo Suk Kim – Ajou University School of Medicine, South Korea

**Background**: Lead exposure could cause various immunologic effects in humans. Several studies have shown that blood lead concentration is positively associated with total immunoglobulin E (IgE). However, no study has investigated whether allergic sensitization could be responsible for the association between lead exposure and total IgE. We investigated whether there was difference in the association between lead exposure and total IgE depending on the presence or absence of *Dermatophagoides farinae* (Df) sensitization, based on data from a large population-based survey.

**Methods:** We used data from the Korea National Health and Nutrition Examination Survey (KNHANES) conducted in 2010. Serum levels of heavy metals such as mercury, cadmium, and lead were measured. Total and Df specific IgE were measured, and urinary cotinine level was investigated. Information about participant sex, age, body mass index (BMI), and household income were also obtained. Data from 2184 participants were analyzed. Multivariate linear regression analyses were used to determine the independent effects of these variables.

**Results:** We found that serum lead concentration was positively correlated with total IgE using Spearman’s correlation test (Spearman’s rho = 0.150, *p* < 0.001). BMI, serum mercury level, and urine cotinine level was also positively correlated with total IgE. In an adjusted linear regression analysis, only serum concentration of lead among the three heavy metals was positively associated with logarithmic transformed total IgE [LogTIgE; coefficient (B) = 0.026, 95% confidence interval (CI) = 0.008-0.044]. When we performed the same analysis on groups divided by allergic Df sensitization status, we found a significant positive association between serum lead and LogTIgE in subjects with Df sensitization (B = 0.076, 95% CI = 0.003-0.150) but not in subjects without Df sensitization (B = 0.015, 95% CI = -0.008-0.039).

**Conclusions:** Serum lead level was positively associated with total IgE level. However, this correlation was statistically significant in subjects with Df sensitization. This result suggests that the immunologic effects of lead exposure may be greater in people with allergic sensitization.

## A118 Clinical and laboratory characteristics of nasal obstruction dominant allergic sensitization

### Seung-No Hong^1^, Doo Hee Han^1^, Chae-Seo Rhee^2^

#### ^1^Seoul National University Hospital, South Korea; ^2^Seoul National University Bundang Hospital

##### **Correspondence:** Seung-No Hong – Seoul National University Hospital, South Korea

**Background:** Allergic rhinitis (AR) is defined clinically by the symptoms including nasal obstruction, rhinorrhea, nasal itching, sneezing with a positive allergen sensitivity test. However a positive skin prick test (SPT) does not always imply the occurrence of clinical symptoms. If an asymptomatic allergen sensitized patient is accompanied with septal deviation (DSN) which could cause nasal obstruction, it can be easily confused with a typical symptomatic allergic rhinitis patient.

**Objective:** The aim of this study was to investigate the clinical and laboratory characteristics of nasal obstruction dominant allergic sensitizers (NODS) based on an allergic rhinitis cohort study.

**Methods:** Patients from a nationwide allergic rhinitis cohort study (ARCO) which was conducted by 8 university hospitals were investigated. Allergic rhinitis was diagnosed when there were at least one rhinitis symptoms with a positive SPT result. The NODS group included patients who had severe nasal obstruction with less other symptoms and positive skin prick test with septal deviation. Clinical and laboratory characteristics were compared between the NODS group and the typical allergic sensitization group.

**Results:** Total 695 patients were included. The average age was 32.6 and 68% of the patients had septal deviation. Laboratory test results reveal that the eosinophil level was lower in NODS group while total IgE level did not show any difference. SPT analysis showed that House dust mite was less sensitized to NODS patients. There was significant sex difference that the male to female ratio was higher in the NODS group. However, no statistically significant difference was found by age, family history and BMI.

**Conclusion:** Asymptomatic allergen sensitized people with septal deviation can mimic allergic rhinitis. When we met the allergen sensitized people with nasal obstruction predominantly, septal deviation should be considered before diagnosis of allergic rhinitis.

## A119 Nasal provocation test is useful for the diagnoses of allergic, non- allergic, and local allergic rhinitis

### Young-Joo Ko, Young Hyo Kim, Dae-Young Kim, Tae Young Jang

#### Inha University, College of Medicine, South Korea

##### **Correspondence:** Young-Joo Ko – Inha University, College of Medicine, South Korea

**Background:** No standard study protocol or diagnostic criteria based on nasal provocation test (NPT) and acoustic rhinometry (AR) results are available for allergic rhinitis.

**Objective:** We aimed to evaluate the usefulness of NPT plus AR for the differential diagnosis of local allergic rhinitis (LAR), allergic, and nonallergic rhinitis.

**Methods:** The medical records and skin prick test (SPT) and NPT results of 262 patients with symptoms of chronic rhinitis were reviewed. Patients were allocated to one of three groups, that is, Group A (n=110, negative SPT result for *Dermatophagoides pteronyssinus* [DP]), Group B (n=53, weakly positive result), or Group C (n=99, strongly positive result).

**Results:** Twelve patients had a negative SPT result and provoked response in NPT (≥29% decrease of minimal cross-sectional area [MCA] after DP challenge) were diagnosed to have LAR. After DP challenge, Group C showed significant aggravation of nasal symptoms and a greater decrease in acoustic parameters than Groups A and B (*P*<0.01). In patients with a ≥ 2 Visual Analogue Scale (VAS) increase in nasal obstruction after DP challenge, the criterion ‘a change of total nasal symptom score [TNSS] of ≥ 6.5’, had 90.6% sensitivity and 77.4% specificity for the diagnosis of allergic rhinitis, whereas the diagnostic criterion ‘a TNV change at 30 minutes after DP challenge of ≥ 27.6%’ had 73.4% sensitivity and 58.1% specificity.

**Conclusion:** NPT with AR could be a useful tool for the differential diagnosis of allergic, non-allergic, and local allergic rhinitis.

## A120 Aspirin facilitates the intestinal absorption and oral sensitization of food allergens in rats

### Tomoharu Yokooji^1^, Taiki Hirano^1^, Hiroaki Matsuo^2^

#### ^1^Hiroshima University, Japan; ^2^Hiroshima University Hospital

##### **Correspondence:** Tomoharu Yokooji – Hiroshima University, Japan

**Background:** Aspirin-facilitated absorption of ingested allergens is considered to be an exacerbating factor in the development of food allergy. In this study, we examined the effect of aspirin on oral sensitization to an egg-white allergen, ovalbumin (OVA) as well as OVA absorption, in rats.

**Methods:** To clarify the intestinal absorption mechanisms of OVA and the effect of aspirin, in situ intestinal re-circulating perfusion study was performed using OVA and fluorescein isothiocyanate-labeled dextran 40 (FD-40), a marker for non-specific-absorption pathways in the presence or absence of various endocytosis inhibitors and aspirin. In addition, plasma concentrations of OVA and FD-40 were measured after oral administration by gavage in rats. To investigate the effect of aspirin on the oral sensitization to OVA, rats were orally administered with OVA by gavage at 3 times a week every other day for 8 weeks. Aspirin or absorption enhancer, spermine was administered 30 min before or simultaneously with OVA, respectively. To evaluate the sensitization to OVA, plasma levels of OVA-specific IgE and IgG_1_were determined by ELISA at 2, 4, 6 and 8 weeks after initiation of OVA sensitization.

**Results:** The absorption rate of OVA in the distal intestine was higher compared with that for a marker of FD-40. Colchicine (general endocytosis inhibitor), bafiromycin A_1_ (inhibitor for receptor-mediated endocytosis) and phenylarsine oxide (inhibitor for clathrin-mediated endocytosis) suppressed the OVA absorption whereas mehyl-β-cyclodextrin (inhibitor for caveolin-mediated endocytosis) exerted no significant effects at distal region, indicating that OVA is preferentially absorbed from the distal intestine via paracellular, and receptor- and clathrin-mediated endocytic pathways. Aspirin increased intestinal absorptions of OVA and FD-40 via the paracellular pathway and exhibited higher plasma levels of OVA-specific IgE and IgG_1_ than those in control at 6 and 8 weeks. Spermine also increased the oral absorptions of OVA almost to the same extent of aspirin whereas the plasma IgE and IgG_1_levels exhibited no significant differences against those in untreated control.

**Conclusions:** Facilitation of OVA absorption via paracellular pathway due to impairment of intestinal barrier function by aspirin is considered to be one of the reasons of enhanced oral sensitization with OVA by aspirin. However, our results have also shown that facilitated oral sensitization to OVA cannot be ascribed to increased absorption of OVA from the intestinal tract only.

## A121 Gestational Secondhand Smoke Exposure Could Affect Maternal n-Glycosylation and Cause Filaggrin Loss in Children with Atopic Dermatitis

### Galina Kuznecova, Ivans Sergejs Kuznecovs

#### Preventive Medicine Institute, Latvia

##### **Correspondence:** Galina Kuznecova – Preventive Medicine Institute, Latvia

**Background:** Atopic dermatitis (AD) affects approximately 10% of children with rising tendency. The exact cause of AD is not known, but more than 50% of pregnant women are still exposed to secondhand smoke in Latvia and smoke exposure during pregnancy might increase the risk of AD in children. The aim of the present study is to investigate the mechanism of AD development in children, born of pregnancies affected with secondhand smoke. The objectives are in favour of the idea that N-glycosylation in keratinocytes cells is limited by Dolichyl Phosphate Cycle (DPC) intermediates, regulated by DPAGT1 (Dolichyl-phosphate (UDP-N-acetylglucosamine) N-acetylglucosaminephosphotransferase 1 (GlcNAc-1-P transferase). Dysregulation of DPAGT1 causes disturbances in filaggrin expression. In epithelial cells loss of filaggrin correlates with atopic dermatitis (AD) presence and activity.

**Methods:** Two groups of mothers and children were compared. The samples were obtained from 154 women with self-reported secondhand smoke during pregnancy and their 156 children (group 1) and 180 women who did not have contact with tobacco smoke during pregnancy (group 2). Cotinine (Cot) and dolichol (Dol) were measured in blood and urine during pregnancy. Filaggrin expression was measured in skin biopsies in newborns with follow up for 2 years. Immunohistochemical and Western blotting methods were used to detect the changes in the expression levels of filaggrin and DPAGT1. Intermediates of DPC fractions were analysed by HPLC method.

**Results:** In group 1 during the period of observation 117 (75%) of children were diagnosed with AD. Mothers of these children (92%) have had Cot in blood and elevated urinal Dol excretion in pregnancy. In group 2 during the period of observation 9 (5%) of children were diagnosed with AD and 16 mothers have had elevated urinal Dol excretion. Overexpression of DPAGT1 was 5-fold higher in AD skin biopsies than in normal skin biopsies and differ from normal one in filaggrin content lost by 3-4 times. Suspected urinary Dol level in pregnancy with positive Cot for AD risk in newborns is calculated as 18.0 mkg/mmol.

**Conclusion:** Secondhand smoke during pregnancy could cause DPAGT overexpression and dysregulation of N-glycosylation in keratinocytes which leads to AD fenotype affecting the stability of tight assembly and adherence junctions in skin of newborns. There is a reason to suggest that elevated Dol correlated with detected Cot in urine in pregnancy may evidence of secondhand smoke related disorder of N-glycosylation. Dol appeared in urinary excretion in pregnancy affected by secondhand smoke is one of the first manifestation of AD risk for newborns. Dol detection in urine during gestational secondhand smoke exposure opens up possibilities for environmental control and for additional motivation to prevent AD in children.

## A122 Allergen specific immunotherapy in the treatment of allergic rhinitis and asthma--a randomized prospective study from kashmir valley-north of India

### Roohi Rasool Wani^1^, Shafia Alam Syed^1^, Ghulam Hassan^1^, Ayaz Gul^1^, Saniya Nissar^2^, Zaffar Amin Shah^2^

#### ^1^Sheri-Kashmir Institute of Medical Sciences(SKIMS), India; ^2^Skims

##### **Correspondence:** Roohi Rasool Wani – Sheri-Kashmir Institute of Medical Sciences (SKIMS), India

**Background:** Kashmir valley has been witnessing an increase in allergy related disorders with aeroallergens being the most prevalent causative agent. Allergen-specific immunotherapy (allergen-SIT) has been a potentially curative treatment modality in allergic diseases that acts by inducing the peripheral T cell tolerance and promoting the formation of regulatory T-cells. Our study was designed to analyse the treatment response of patients with allergic rhinitis and allergic asthma in Kashmir Valley by using allergen-SIT approach.

**Method:** A total of 754 patients suffering from Allergic Rhinitis and Allergic Asthma were recruited in this study. Skin Prick test (SPT) was performed with panel of aeroallergens. Allergen-SIT was given as a therapeutic modality to 218 SPT positive patients. The symptom score and medication requirement was analysed during the time course of allergen-SIT.

**Results:** Allergen-SIT was effective in reducing severe symptoms in 87% patients of SPT-positive allergic rhinitis and 76% SPT-positive allergic asthma patients. Moreover, allergen-SIT showed reduction in medication of 73% SPT-positive allergic rhinitis and 69% SPT-positive allergic asthma patients during their maintenance therapy, usually after period of one year.

**Conclusion:** Our study proves the efficacy and beneficial effects of allergen-SIT in patients with allergic rhinitis and allergic asthma in Kashmir Valley. Our study shows that allergen-SIT is not only an effective therapy for reducing the allergic symptoms but also acts specifically to restore normal immunity against allergens in the long-term course of the disease.

## A123 Sleep disorders in latin-American children with asthma and/or allergic rhinitis and normal controls

### Marilyn Urrutia Pereira^20^, Carmen Fernandez^1^, Dirceu Sole^2^, Herberto Jose Chong Neto^3^, Veronica Acosta^4^, Alfonso Mario Cepeda^5^, Mirta Alvarez Castello^6^, Claudia Almendarez^7^, Jose Santos Lozano Saenz^8^, Juan C. Sisul^9,10,11^, Nelson Rosario Filho^12^, Antonio Castillo^13^, Marylin Valentin Rostan^14^, Jennifer Avila^15^, Hector Badellino^16^, Maria Carolina Manotas^17^, Raúl Lázaro Castro Almarales^18^, Mayda González León^19^

#### ^1^Centro Pediátrico Paidos – Universidad Nacional Del Este, Asunción, Paraguay; ^2^ Brazilian Society, Sao Paulo, Brazil; ^3^Universidade Federal Do Paraná, Curitiba, Brazil; ^4^Hospital Dr Avelino L Castelán, Resistencia-Chaco, Argentina; ^5^Hospital Universitario Metropolitano, Barranquilla, Colombia; ^6^Hospital Universitário General Calixto Garcia, Havana, Cuba; ^7^Centro De Asma y Alergia, Tagucigalpa, Honduras; ^8^Centro medica San Angel, Xalapa, Mexico; ^9^Juan De Salazar; ^10^Fellow of the American College of Allergy, Asthma & Immunology; ^11^State Director Gard/ARIA; ^12^University of Parana, Curitiba, Brazil; ^13^Centro De Medicina Avanzada , Santo Domingo, Dominican Republic; ^14^Rafael Barradas 1671, Montevideo, Uruguay; ^15^Pediatric Program of Asthma Prevention (PIPA), Uruguaiana, Brazil; ^16^Clínica Regional Del Este, San Francisco-Cordoba, Argentina; ^17^Hospital Universitario Metropolitano, Barranquilla, Colombia; ^18^National Center of Bioproducts, Mayabeque, Cuba; ^19^Cuban Society of Integr, Havana, Cuba; ^20^Brazilian Sociaty, Brazil

##### **Correspondence:** Marilyn Urrutia Pereira – Brazilian Sociaty, Brazil

**Background:** Asthma and/or allergic rhinitis have been associated with sleep disorders. The aim of this study was to evaluate sleep disorders in Latin-American children (4 to 10 years) from 10 countries, with persistent asthma (A) and/or allergic rhinitis (AR) and in normal controls (C).

**Methods:** Parents from 454 C and 700 A and/or AR children followed in reference clinics answered the Children Sleep Habits Questionnaire (CSHQ) that is an one-week retrospective questionnaire composed by 33 questions and divided in 7 subscales (bedtime resistance, sleep duration, sleep anxiety, night awaking, parasomnias, sleep-disordered breathing and daytime sleepiness). Total CSHQ scale and subscales were compared between C and A+AR, A (n=285) *vs* AR (n=390), and controlled A (CA, n=103) *vs* partially controlled/uncontrolled A (UA, n=182).

**Results:** comparison between C and A+AR showed no significant differences in age (6.7 vs 7.0 years, respectively) and total CSHQ (53.3 vs 63.2, respectively) and subscales were significantly higher among A+AR group. Comparison between A and AR groups, except for sleep anxiety, show significantly higher values for total CSHQ (66.9 vs 61.0, respectively) and the other subscales. UA showed significantly higher values for total CSHQ and subscales in comparison to CA (71.1 vs 59.4, respectively).

**Conclusions:** Latin-American asthmatic and/or allergic rhinitis children showed to have sleep disorders defined by the CSHQ when compared to normal controls. Despite being treated, asthma causes sleep compromise, mainly if uncontrolled.

## A124 Association between respiratory symptoms and exhaled nitric oxide in Afghanistan

### Woo Kyung Kim^1^, Hae-Sun Yoon^2^

#### ^1^Seoul Paik Hospital/Inje University College of Medicine, South Korea; ^2^Seoul Paik Hospital/Inje University

##### **Correspondence:** Woo Kyung Kim – Seoul Paik Hospital/Inje University College of Medicine, South Korea

**Background**: Epidemiological studies mainly from USA, the Europe, and Asia indicate a high prevalence of asthma. However, little is known about asthma among the population of the Afghanistan. In the patients with asthma have high levels of exhaled nitric oxide fraction (FeNO). We aimed comparing with respiratory symptoms and FeNO and lung function test in Afghanistan.

**Methods**: Respiratory symptoms were assessed by a pulmonology physician (n=117). The FeNo was measured by NIOX MINO device (Aerocrine AB, Solna, Sweden). Lung function was examined by a portable spirometer and airway hyper-resposiveness. Asthma Control Questionnaire (ACQ), blood eosinophils were assessed at the same time.

**Results**: Respiratory symptoms were cough (100%), dyspnea (88%), wheezing at the physical exam (56%). Lower FEV1 (p<0.0001) and higher FeNO values (P=0.001) were observed in Afghanistan. Between blood eosinophil counts and FeNO did not show association.

**Conclusions**: This is the first study was performed in Afghanistan people. Respiratory symptoms and FeNo had association in Afghanistan. In future more subjects enroll and environmental factor are needed.

## A125 ATP, a danger signal, activates human eosinophils via P2 purinergic receptors

### Takehito Kobayashi^1^, Tooru Noguchi^1^, Tomoyuki Soma^1^, Kazuyuki Nakagome^1^, Hidetomo Nakamoto^1^, Hirohito Kita^2^, Makoto Nagata^1^

#### ^1^Saitama Medical University, Japan; ^2^Mayo Clinic College of Medicine, USA

##### **Correspondence:** Takehito Kobayashi – Saitama Medical University, Japan

**Background:** Eosinophils recognize various stimuli resulting in their accumulation in mucosal tissues and the progression of inflammation. Uric acid (UA) is an important endogenous danger signal released from injured cells by inflammation and infection. Eosinophils are also involved in innate Th2-type immune responses mediated through endogenous danger signals, including IL-33, uric acid (UA), or ATP, in non-sensitized mice exposed to environmental allergens. However, the mechanism involved in eosinophil responses to these danger signals remains insufficiently understood.

**Methods:** Eosinophils isolated from peripheral blood of normal individuals were incubated in the presence or absence of monosodium urate (MSU) crystals and ATPγS, a non-hydrolysable ATP analogue. To determine the involvement of P2 or P2Y_2_ receptors in eosinophil responses to UA and ATP, eosinophils were preincubated with a pan-P2 receptor inhibitor, oxidized ATP (oATP), or anti-P2Y_2_antibody before incubation with MSU crystals or ATPγS.

**Results:** MSU crystals induced adhesion of eosinophils to recombinant human (rh)-ICAM-1 and induced production of superoxide anion. oATP abolished eosinophil responses to MSU crystals, suggesting involvement of endogenous ATP and its receptors. Furthermore, exogenous ATP, as ATPγS, activated eosinophils and induced migration across a model of basement membrane, adhesion to rh-ICAM-1, generation of superoxide anion, and degranulation of eosinophil-derived neurotoxin (EDN). oATP and anti-P2Y_2_significantly reduced these eosinophil responses.

**Conclusions:** UA stimulated eosinophils to release ATP. ATP serves as an essential mediator of functional responses in human eosinophils. Thus, human eosinophils may respond to particulate damage-associated endogenous danger signals. These responses by eosinophils to tissue damage may explain the self-perpetuating nature of airway inflammation in patients with asthma.

## A126 Atopic dermatitis and sleep disorders in latin American children

### Marilyn Urrutia Pereira^1^, Dirceu Sole^1^, Herberto Jose Chong Neto^2^, Alfonso Mario Cepeda^3^, Raúl Lázaro Castro Almarales^4^, Juan C. Sisul^5,6,7^, Marylin Valentin Rostan^8^, Hector Badellino^9^, Miguel Alejandro Medina Avalos^10^, Antonio Castillo^11^, Claudia Almendarez^12^, Nelson Rosario Filho^13^, Caridad Sanchez Silot^14^, Jennifer Avila^15^, Felicia Berroa Rodriguez^11^, Jose Santos Lozano Saenz^16^, Mirta Alvarez Castello^17^, Carmen Fernandez^18^

#### ^1^Brazilian Society, Brazil; ^2^Universidade Federal Do Paraná, Brazil; ^3^Hospital Universitario Metropolitano, Colombia; ^4^National Center of Bioproducts, Cuba; ^5^Juan De Salazar; ^6^Fellow of the American College of Allergy, Asthma & Immunology; ^7^State Director Gard/ARIA, Paraguay; ^8^Rafael Barradas 1671, Uruguay; ^9^Clínica Regional Del Este, Argentina; ^10^Servicio De Alergologia Del Hospital Issste, Mexico; ^11^Centro De Medicina Avanzada Dr. Abel González, Dominican Republic; ^12^Centro De Asma y Alergia, Honduras; ^13^University of Parana, Brazil; ^14^University Hospital Infantil Sur, Cuba; ^15^Pediatric Program of Asthma Prevention (PIPA), Brazil; ^16^Centro Medica San Angel, Mexico; ^17^University Hospital Calixto García, Cuba; ^18^Centro Pediátr, Paraguay

##### **Correspondence:** Marilyn Urrutia Pereira – Brazilian Society, Brazil

**Background:** Atopic dermatitis (AD) has been associated with impairment of sleep. The aim of this study was to evaluate sleep disorders in AD Latin-American children (4 to 10 years) from 10 countries, and in normal controls (C).

**Methods:** Parents from 454 C and 340 AD children followed in reference clinics answered the Children Sleep Habits Questionnaire (CSHQ) that is an one-week retrospective questionnaire composed by 33 questions and divided in 7 subscales (bedtime resistance, sleep duration, sleep anxiety, night awaking, parasomnias, sleep-disordered breathing and daytime sleepness). Total CSHQ scale and subscales were compared between C and DA groups. Spearman’s correlation coefficient between SCORAD (Scoring atopic dermatitis) with all subscales and total CSHQ were also obtained.

**Results:** C and DA groups were similar regarding age, however, significantly higher values for total CSHQ (62.2±16.1 *vs*53.3±12.7, respectively) and subscales were observed among DA children in comparison to C, and they were higher among those with moderate (54.8%) or severe (4.3%) AD. Except for sleep duration (r=-0.02, p=0.698), there were a significant Spearman’s correlation index for bedtime resistance (0.24, p<0.0001), sleep anxiety (0.29, p<0.0001), night awaking (0.36, p<0.0001), parasomnias (0.54, p<0.0001), sleep-disordered breathing (0.42, p<0.0001), daytime sleepiness (0.26, p<0.0001) and total CSHQ (0.46, p<0.0001).

**Conclusions:** Although properly treated, Latin-American children with AD showed to have sleep disorders evaluated by the CSHQ. Children with moderate to severe forms of AD were those who had the biggest changes in CSHQ.

## A127 Der p 23: A Major House Dust Mite Allergen in Spite of Limited Release from Fecal Pellets and Prominent Protease Sensitivity

### Wai Tuck Soh^1^, Alain Jacquet^1^, Kiat Ruxrungtham^1^, Emmanuel Nony^2^, Maxime Le Mignon^2^

#### ^1^Chulalongkorn University, Thailand; ^2^Stallergenes, France

##### **Correspondence:** Wai Tuck Soh – Chulalongkorn University, Thailand

**Background:** The recently identified house dust mite (HDM) allergen Der p 23 has been considered as a major allergen, according to its high IgE reactivity which is quite similar to that measured for the major HDM allergens Der p 1 and Der p 2. Whereas the high IgE binding frequencies to Der p 1 and Der p 2 could be partially explained by the relative abundance of these proteins in the mite fecal pellets, there is a discrepancy between the IgE reactivity to Der p 23 and the very poor amount of intact Der p 23 in mite feces aqueous extracts. The goal of the study was to evaluate the release of Der p 23 from fecal pellets as well as the allergen sensitivity to proteases.

**Method:** HDM enriched fecal pellets were extracted with PBS or 6 M Urea with 2% SDS and 10 mM DTT. The release of nDer p 23 in the extracts as well as in a commercial mite bodies extract (Greer) was measured by western blot. Recombinant Der p 23 (rDer p 23) and GST-Der p 23 were produced in *P. pastoris* and *E. coli* respectively. These two recombinant forms were incubated with activated natural Der p 1, trypsin or HDM extracts. The Der p 23 proteolysis was monitored by SDS-PAGE and western blot.

**Results:** Very tiny amounts of nDer p 23 is released from fecal pellets upon incubation with PBS. Unexpected high molecular weight immunoreactive bands present also in very low amount were detected when extraction was performed under denaturing and reducing conditions. Intact nDer p 23 was not present in mite bodies extract. Recombinant Der p 23 can be degraded by Der p 1 but not by Trypsin. The Der p 23 O-glycosylation partially reduced the proteolysis. The Der p 23 sensitivity to cysteine proteases from HDM fecal pellets extracts was also demonstrated.

**Conclusions:** Our results suggest that natural Der p 23, through probably its tight interactions with the peritrophic matrix, is very poorly released from the fecal pellets. According to nDer p 23 very low abundance in mite extracts and high sensitivity to proteolytic degradation, recombinant Der p 23 may be a valuable material for the diagnosis as well as the treatment of Der p 23 sensitivities.

## A128 Anaphylactic Reaction After Inhalation of Budesonide

### Mary Lee-Wong^1^, Suzanne McClelland^2^, Suzanne McClelland^3^, Nanette B. Silverberg^1,4,5^, Christian E. Song^6^

#### ^1^Mount Sinai Beth Israel, USA; ^2^Comprehensive Pharmacy Services; ^3^Skagit Valley Hospital; ^4^Mount Sinai St. Luke’s; ^5^Mount Sinai Roosevelt; ^6^Massachusetts Eye and Ear

##### **Correspondence:** Mary Lee-Wong – Mount Sinai Beth Israel, USA

**Background:** Hypersensitivity reactions to corticosteroids are known to occur, but are an unexpected phenomenon. However, immediate hypersensitivity with severe anaphylactic reactions is scarce in literature.

**Methods:** Diagnosis is confirmed using a patch test for suspected delayed type hypersensitivity. Skin prick and/or intradermal tests are for immediate type hypersensitivity to identify the responsible agent and potential cross-reactivity patterns[i].

**Results:** A patient presented with seasonal allergies and asthma not adequately controlled with inhaled albuterol. Inhaled budesonide/formoterol daily was prescribed for treatment. The first dose was well tolerated, but 15 minutes after the second dose the next day, the patient developed shortness of breath, a feeling of throat tightness, swelling of the lips and tongue and blisters along the oral mucosa. The patient was treated with an oral antihistamine and symptoms abated within one hour. The patient was unaware of any previous allergies to corticosteroids and reported using various topical preparations to treat dermatitis for more that one year without resolution.

An open test with an application of budesonide/formoterol was sprayed onto the patient’s arm resulting in an erythematous plaque at 72 hours. Patch testing revealed delayed reactions at 48 hours to tixocortol-21-pivalate 1%, budesonide 0.01% and hydrocortisone 1%. Skin tests[ii]were performed to further evaluate and document corticosteroid hypersensitivity using ciclosenide, methylprednisolone, mometasone, budesonide, budesonide/formoterol, formoterol, fluticasone/salmeterol along with normal saline and histamine as controls. Within 24 hours positive results for two different inhaled budesonide formulations and one for budesonide/formoterol were observed.

**Conclusions:** Inhaled corticosteroids are first line agents in the treatment of persistent asthma. In patients with sensitivity to this drug class, clinicians should be aware of cross-reactivity patterns to identify an appropriate corticosteroid for therapy and test to identify the class of products which would be deemed safe. Furthermore, the practitioner should be aware that prior atopy is a risk factor for sensitization to topically applied therapeutics. Lastly, the anti-inflammatory effects of a corticosteroid may mask the allergy. Although patch and skin tests supported delayed hypersensitivity reactions, this patient presented with an immediate hypersensitivity reaction that is suspected to have occurred from previous sensitization of topical corticosteroid use.

References

[i] Coopman S, Degreef H, Dooms-Goossens A. *Identification of cross-reaction patterns in allergic contact dermatitis from topical corticosteroids*. Br J Dermatol. 1989; 121:27-34.

[ii] Asakawa H, Araki T, Imai I, Tsutsumi Y, Kawakami F. *Skin tests of Steroid Allergy*. Allergy. 1999; 54:645-6.

## A129 Lipidomic analysis of mattress dust from urban and rural schoolchildren in China

### Zhaowei Yang^5^, Jiukai Zhang^1^, Wentao Zheng^2^, Nanshan Zhong^3^, Jing Li^4^

#### ^1^Agro-Product Safety Research Center; ^2^Department of Allergy and Clinical Immunology; ^3^State Key Laboratory of Respiratory Disease; ^4^Guangzhou Medical University; ^5^The First Affiliated Hospital, Guangzhou Medical University, China

##### **Correspondence:** Zhaowei Yang – The First Affiliated Hospital, Guangzhou Medical University, China

**Background**: House dust harbors ambient immunomodulatory particulates and reflects the living environments. Lipid compounds may have manifold impact on host immunity but have not yet been extensively described.

**Objective**: To investigate and compare the lipids of mattress dust from urban and rural school children in China.

**Methods**: Dusts from beddings of twenty schoolchildren in urban Guangzhou and rural Conghua were collected and extracted following the Bligh and Dyer method. Lipidomic profiling was carried out by ultra-performance liquid chromatography/quadrupole-time-of-flight (Q-TOF)-MS-based approach using a Waters Xevo G2 Q-TOF mass spectrometer with an electrospray ion source (ESI). Mass spectra were acquired in the range of *m/z* 50~1200 in both positive and negative ionization mode. Raw data were then processed using XCMS, SIMCA-P and multivariate statistical analysis, then compared with database to identify lipid components.

**Results**: The established models of lipid profiling were statistically valid and well fit. Urban and rural dusts showed differential composition of lipid molecules. A total of 8986 and 4742 metabolites were detected in positive and negative ionization mode, respectively. Fourteen lipid molecules were finally identified. Oleamide, Cer (d18:0/14:0), MG (0:0/18:3/0:0), two LysoPAs, 16-hydroxy hexadecanoic acid and phytomonic acid were abundant in rural dust, whereas Cer (t18:0/16:0), DG (36:7), two LysoPCs, PA (16:0e/18:0), PC (32:1) and PG (P-16:0/14:1) were abundant in urban dust.

**Conclusions**: This is the first report suggesting that children from urban and rural areas are exposed to discrepant environmental lipids. Potential responses to the identified lipids should be further investigated.

## A130 Improvements in quality of life in children with allergic rhinitis after adenotonsillectomy

### Jung Ho Bae, Young Joo Cho, Joo Yeon Kim

#### Ewha Womans University, South Korea

##### **Correspondence:** Jung Ho Bae – Ewha Womans University, South Korea

**Background**

Chronic nasal obstruction in children is a very common symptom. Allergic rhinitis, a chronic inflammatory disease of the nasal mucosa, is one of the most common cause of nasal obstruction. Besides, adenotonsillar hypertrophy is also the first to blame. Because two diseases are quite common in children, allergic rhinitis and adenotonsillar hypertrophy often coexist. We evaluated the surgical outcome of adenotonsillectomy in children with allergic rhinitis. The goal of this study is to better understand the role of adenotonsillar hypertrophy and its impact on quality of life in allergic rhinitis children.

**Methods**

We conducted this study on 27 pediatric patients with allergic rhinitis and adenotonsiller hypertrophy and 33 patients with adenotonsillar hypertrophy only. Demographic data, clinical examination including oral and nasal endoscopy, and skull lateral view were obtained. The mean age, Brodsky tonsil size, adenoid size, and BMI were similar between two groups. A follow-up evaluation, which included questionnaires of clinical scores, physical examinations and skull lateral view were performed 3, 6 months after adenotonsillectomy.

**Results**

When the preoperative and postoperative results were compared, postoperative scores for sleep related symptoms were significantly improved from preoperative scores in both groups. The history of frequent URI and antibiotic therapy after surgery was no significant differences between two groups. The improvements of nasal symptoms including nasal obstruction, were significantly higher in non-allergic group (*p<0.01*). The adenoid regrowth rate was slightly higher in non-allergic group (*p<0.05*).

**Conclusions**

Our findings confirm that adenotonsillectomy is a satisfactory treatment for sleep related in adenotonsillar hypertrophic children with or without allergic rhinitis. Though some nasal symptoms depends on post operative allergic treatments, there is a dramatic improvement in the quality of life after adenotonsillectomy in allergic rhinitis children.

## A131 The seasonal variation of asthma exacerbations in patients allergic to pollens in Greece

### Dimitrios Vourdas^1^, Christos Grigoreas^1^, Konstantinos Petalas^2^

#### ^1^251 General Air Force Hospital; ^2^Hellenic Air Force, Greece

##### **Correspondence:** Konstantinos Petalas – Hellenic Air Force, Greece

**Background:** The recognition of clear seasonal (monthly) patterns of Asthma Exacerbations (AE), in a particular area, may allow preventive strategies to be developed. Athens is the capital city of Greece, a Mediterranean country where the prevailing pollens are different comparing to Central or Northern Europe countries. The aim of this study was to determine the seasonal (monthly) variation in AE with allergy to pollens, in Greece.

**Methods:** Data were obtained from our department in Athens, between October 1994 and September 2014 (240 months). We evaluated all AE occurring this period for patients allergic only to pollens, according to the results of skin prick testing. A positive SPT was defined as a wheal size at least 3 mm greater than the negative control. AE were defined as a deterioration in asthma resulting in an unscheduled visit (i.e patient-initiated) leading to change in asthma treatment or the need for oral steroids for>3 days and/or emergency room visit/hospitalization. Data are presented as monthly averages of these years of combined data, as a percent above (+) or below (-) the average monthly value (%) for the 240 months under study.

**Results:** There were 1.723 AE (1.099 in males, 624 in females) in 1.219 asthmatic patients (798 males, 421 females). From 1.723 AE, 218 were identified in patients 0-14 years, 615 in patients 15-29 years, 600 in patients 30-44 years and 290 in patients >45 years. The results by month, AE number and percent above or below an average monthly value (%) were respectively the following:January-54-(-62.3%), February-45-(-68.6%), March-93-(-35.1%), April-230-(+60.4%), May-826-(+476%), June-192-(+33.9%), July-60-(-58.1%), August-37-(-74.2%), September-43-(-70%), October-53-(-63%), November-51-(-64.4%), December-39-(-72.8%)

**Conclusion:** These findings suggest that AE in the Athens region have a clear cut seasonal (monthly) variation in patients allergic to pollens. An increase in AE occurred in three months (April, May, June) and a decrease in the rest of the months. It is of great interest to note that especially in May occurred a huge increase (peak) in AE (+476%) in patients allergic to pollens (May epidemic). This study suggests that aeroallergens (pollens) can exacerbate asthma, especially in May, in Greece and the results may offer significant opportunities for improved disease management.

## A132 Whole-genome sequencing study in allergic rhinitis nuclear families

### Yuan Zhang

#### Beijing Institute of Otolaryngology, China

4 nuclear families which included 9 patients suffering from house dust mites allergic rhinitis (AR) were enrolled in present study. Whole -genome sequencing were employed to screen the possibly candidate variations which were susceptible to AR. 85 common variations shared in the study subjects and were reported to be related to allergic diseases previously were detected. Moreover, 108 low-frequency and rare variations were identified and some of them involved in antigen processing and presentation, endocytosis and phagosome pathways. Replications of the variations found through whole-genome sequencing were performed in 402 independent AR patients and healthy controls. We demonstrated 4 gene variations or region which have been proved to be associated with allergy, including rs204993 in PBX2, rs2228145 in IL6R, rs1128670 in MRPL18 and rs34874585 in IGHV1-3. In addition, we still discovered 5 novel AR gene variations and regions that involved rs2259816 in HNF1A, rs2298428 in YDJC, rs1128670 in MRPL18, rs2227983 in EGFR as well as rs1059091 in IFITM2.

## A133 Effect of the production of extracellular matrix from nasal fibroblasts by eosinophils activated with airborne fungi

### Seung-Heon Shin, Mi-Kyung Ye, Jeong-Kyu Kim

#### Daegu Catholic University Hospital, South Korea

##### **Correspondence:** Seung-Heon Shin – Daegu Catholic University Hospital, South Korea

**Backgrounds**

The pathogenesis of nasal polyps is associated with the interaction between mucosal epithelial cells, extracellular matrix and inflammatory cell, and their chemical mediators. Fibroblasts are major structural components of nasal mucosa and play an important role as a source of chemical mediators and tissue remodeling. The aim of this study was to evaluate the effect of airborne fungi in the production of extracellular matrix from nasal fibroblasts with the interaction of activated eosinophils.

**Methods**

Nasal fibroblasts were isolated from nasal polyp and normal inferior turbinates. Nasal fibroblasts were stimulated with *Alternaria alternate* at 100 ug/ml and *Aspergillus fumigatus* at 50 ug/ml. Eosinophils from peripheral blood were extracted then stimulated with both fungi. Nasal fibroblasts were co-cultured with activated eosinophils with or without physical contact for 24 hours. Then α-smooth muscle actin (α-SMA), collagen type I, TIMP-1, MMP-9, fibronectin mRNA expression were determined with real time RT-PCR and protein production was determined with western blot method.

**Results**

*Alternaria* and *Aspergillus* enhanced extracellular matrix mRNA expression from nasal polyp fibroblasts about 2.4 to 7.1 times than normal control. However, only Alternaria induced the production of eosinophil peroxide and superoxide from eosinophils. Co-culture of activated eosinophils with fungi and nasal fibroblasts enhanced extracellular matrix mRNA expression 1.8 to 2.6 times than co-cultured with non-activated eosinophil group.

**Conclusions**

Airborne fungi enhance the extracellular matrix mRNA expression and protein production not only by direct stimulation of fibroblasts, but also by the interaction of activated eosinophils with fibroblasts. In the pathogenesis of nasal polyps, fungi play an important role through the production of extracellular matrix from fibroblasts and inducing tissue remodeling.

## A134 The study of clinical characteristics, lung function and bronchodilator responsiveness in infants with RSV bronchiolitis

### Yong Feng, Yunxiao Shang

#### Shengjing Hospital of China Medical University, China

##### **Correspondence:** Yong Feng – Shengjing Hospital of China Medical University, China

**Background**

Respiratory syncytial virus (RSV) is the main etiologic agent of bronchiolitis worldwide and RSV bronchiolitis seems to be a more severe disease than that caused by other viruses. The aim of our study is to observe the clinical, pulmonary function and bronchodilator responsiveness differences between respiratory syncytial virus (RSV) and non-RSV bronchiolitis.

**Methods**

96 bronchiolitis inpatients of Shengjing Hospital from November 2012 to March 2014 were enrolled. RSV detection was performed at enrollment and the patients were divided into RSV group and non-RSV group. Patient’s information of demography, allergy and duration of onset were collected, infant pulmonary function, bronchial dilation test and serum total IgE were tested, and modified-Tal score and length of hospitalization were also recorded at admission.

**Results**

RSV bronchiolitis (n = 40 [46.17%]) were younger at hospitalization (5±3.97 vs 7.38±4.42 months, p=0.008) and disease severity defined by length of hospitalization and modified-Tal score was significantly worse in children with RSV bronchiolitis. There is no difference in sensitization between the two groups. The respiratory rate (RR) and respiratory system resistance (Rrs) were significantly increased, while the tidal volume per kilogram (VT/kg), ratio of time to peak tidal expiratory flow and total expiratory time (tPTEF/TE), ratio of volume to peak expiratory flow and total expiratory volume (VPTEF/VE), and respiratory system compliance per kilogram (Crs/kg) were significantly decreased as compared with those in non-RSV bronchiolitis. The plethysmographic functional residual capacity (FRCp) showed no statistically significant differences. With bronchodilators, VT/kg, tPTEF/TE and VPTEF/VE were significantly improved in RSV bronchiolitis. There were good correlations between disease severity and lung function.

**Conclusions**

In conclusion, this study supports that younger age is a risk factor for RSV bronchiolitis. The airway obstruction of RSV bronchiolitis seems to be more severe and have higher bronchodilator responsiveness. Infant pulmonary function may reflect disease severity, while we need to consider the affected factors when assessing the disease severity based on infant pulmonary function.

## A135 GIS-based association between PM10 and allergic diseases in seoul: Implication for health and environmental policy

### Sungchul Seo^1^, Ji Tae Choung^1^, Dohyeong Kim^2^, Young Yoo^1^, Hyunwook Lim^3^

#### ^1^Korea University, South Korea; ^2^The University of Texas at Dallas, USA; ^3^Korea University Medical Center, South Korea

##### **Correspondence:** Sungchul Seo – Korea University, South Korea

**Purpose:** The role of PM_10_ in development of allergic diseases remains controversial among epidemiological studies, partly due to the inability to control for spatial variations of large-scale risk factors. This study aims to investigate spatial correspondence between the level of PM_10_ and allergic diseases at the sub-district level in Seoul, Korea, in order to evaluate whether the impact of PM_10_ is observable and spatially varies across the sub-districts.

**Methods:** PM_10_ measurements at 25 monitoring stations in the city were interpolated to 424 sub-districts where annual inpatient and outpatient count data for three types of allergic diseases (atopic dermatitis, asthma and allergic rhinitis) were collected. We estimated multiple ordinary least square regression models to examine the association of the PM_10_ level with each of the allergic diseases, controlling for various sub-district level covariates. Geographically weighted regression models were conducted to evaluate how the impact of PM_10_ varies across the sub-districts.

**Results:** PM_10_ was found to be a significant predictor of atopic dermatitis patient count (p<0.01), with greater association when spatially interpolated at the sub-district level. No significant effect of PM_10_ was observed on allergic rhinitis and asthma when socioeconomic factors were controlled for. Geographically weighted regression models revealed spatial variation of PM_10_ effects on atopic dermatitis across the sub-districts in Seoul. The relationship of PM_10_ levels to atopic dermatitis patient counts is found to be significant only in Gangbuk region (p<0.01), along with other covariates including average land value, poverty rate, level of education and apartment rate (p<0.01).

**Conclusions:** Our findings imply that PM_10_ effects on allergic diseases might not be consistent throughout Seoul. GIS-based spatial modeling techniques could play a role in evaluating spatial variation of air pollution impacts on allergic diseases at the sub-district levels, which could provide valuable guidelines for environmental and public health policymakers.

## A136 The relationship between rhinovirus and recurrent wheezing

### Wenjing Zhu^1^, Chuanhe Liu^1^, Li Sha^1^, Li Chang^1^, Min Zhao^1^, Linqing Zhao^1^, Yuan Qian^1^, Yuzhi Chen^2^

#### ^1^Capital Institute of Pediatrics, China; ^2^Capital of Pediatric Research for Asthma Center

##### **Correspondence:** Wenjing Zhu – Capital Institute of Pediatrics, China

**Background**

Early studies showed that Respiratory Syncytial Virus (RSV) was the main trigger of wheezing in infants. New evidences indicated that rhinovirus (RV) may play a significant role in the development of asthma.

**Objectives**

To investigate the role of RV infection in the episode of recurrent wheezing in young children (5 years and younger) by case-control study. The clinical features and duration of symptoms were analyzed according to the result of the follow-up in 4 weeks.

**Methods**

Children with recurrent wheezing during attack and children without wheezing were included in this study. Specimen of nasopharyngeal aspirates were obtained for detection of virus. RV, Human metapneumovirus (hMP), Bocavirus (hBoV) were tested by reverse transcription-polymerase chain reaction. RSV, parainfluenza viruses type I, II, III, influenza virus A, B and adenoviruses (ADV) was confirmed by detection of viral antigens via fluoroimmunoassay. Counting of white blood cells, severity of disease was accessed in the first visit. Duration of symptoms and interval between episodes were recorded in the 4 weeks follow-up.

**Results**

Nasopharyngeal aspirates were collected from 109 recurrent wheezing children and 70 non-wheezing children. Compared with control group, the wheezing children were higher positive rate for total detective virus (53.2% vs15.7%, *χ*^*2*^=25.3, P<0.01), RV (31.2% vs 12.9%, *χ*^*2*^=7.9, P=0.005) and RSV(19.3% vs 4.3%, *χ*^*2*^=8.2, P=0.004). The positive rates of RV was higher in toddler wheezing than infant wheezing group (44.8% vs 20.0%, *χ*^*2*^=6.0, P=0.015). The EOS count was 0.38 (0.12-0.57) *10^^9^/ L in RV-positive wheezing children, which was much higher than that in other virus-positive cases 0.21 (0.07-0.38) *10^^9^/ L (U=252, P=0.04). Compared with other virus-positive cases, RV-positive wheezing children have higher LYMP and WBC counts (P<0.05). There was no different between virus-negative and virus-positive cases in the symptoms of wheezing, coughing and nasal in from of 4 weeks follow-up (P>0.05).

**Conclusions**

High in of respiratory virus is found in children with wheezing exacerbation. RV is the most important pathogen in recurrent wheezing patients. The RV induced wheezing is associated with high level of EOS, LYMP and WBC counts. It implies RV may associated with atopy and immunoreaction. The clinical symptom is not different between RV and other virus infection in short time.

## A137 Dominancy of Staphylcoccus Aureus in the Skin of Atopic Dermatitis Patients Compared to Healthy Subjects through Metagenomic Analysis

### Min-Hye Kim^1^, Young Joo Cho^1^, Mina Rho^2^, Jung-Won Kim^1^, Yeon-Mi Kang^1^, Kyung-Eun Yum^3^, Hyeon-Il Choi^3^, Jun-Pyo Choi^3^, Han-Ki Park^3^, Taek-Ki Min^4^, Bok-Yang Pyun^5^, Yoon-Keun Kim^3^

#### ^1^Ewha Womans University, School of Medicine, South Korea; ^2^Department of Computer Engineering, Hanyang University; ^3^Ewha Institute of Convergence Medicine, Ewha Womans University Medical Center; ^4^Soonchunhyang University Hospital; ^5^Soonchunhyang University

##### **Correspondence:** Min-Hye Kim – Ewha Womans University, School of Medicine, South Korea

**Background:***Staphylococcus aureus* is known to be the most frequent cause of skin infection or aggravating factor in atopic dermatitis patients. The colonization rate of *S. aureus* reaches to 90% of atopic dermatitis patients, which is much higher than in the healthy subjects. Until now, bacteria could be identified only through the bacterial culture technique, by which only 1% of total bacteria can be identified. Here, we performed metagenomic analysis to determine major colonizing bacteria in the skin of atopic dermatitis patients vs. healthy control subjects.

**Methods:** Seventeen patients with atopic dermatitis and 6 healthy control subjects were enrolled. Skin washing fluids were obtained from the moistened gauze which loaded on the skin lesion in the cubital fossa of atopic dermatitis patients and on the same part of normal controls. After genomic DNA was extracted from the skin washing fluids, 16s ribosomal DNA was amplified using the universal primer, sequenced through the next generation sequencer, and then the sequenced data was analyzed using bioinformatics.

**Results:***Staphylococcus spp.* was dominant in the skin from atopic dermatitis patients, while it was undetectable in the control subject skins. The mean proportion of Staphylococcus in the total bacterial DNA of atopic dermatitis patients was 68%, however that was 0% in normal controls. Additional analysis for the species of *Staphylococcus spp.* revealed that most of *Staphylococcus spp.* was *Staphylcoccus aureus*. The following frequent bacteria were *Pseudomonas* and *Streptococcus* spp., and their proportions were 11% and 10% in the patients vs. 2% and 1% in the controls, respectively. On the other hand, Alcaligenaceae family and *Sediminibacterium spp.* were the most frequent bacteria in the skin of the controls, and their proportions were 39% and 13% in the controls vs. 0% and 0% in patients, respectively.

**Conclusions:***Staphylcoccus aureus* is the predominant colonizer in the atopic dermatitis skin through the metagenomic analysis of bacterial DNA.

## A138 Micronized Cellulose Powder Reduces the Dose of Locally Applied Glucocorticoids in Patients with Allergic Rhinitis

### Xueyan Wang

#### Beijing Shijitan Hospital, China

**Background:** The aim of this study was to estimate the efficacy and the safety of the combination of micronized cellulose powder and locally applied glucocorticoids on the symptoms of allergic rhinitis.

**Methods:** This single blind controlled study was conducted in 120 subjects (2-60 years of age, mean age 38.5) with allergic rhinitis, 66 men and 54 women. All participants had a positive medical history for allergic rhinitis. They were randomized to 1 puff mometasone furoate followed by 1 puff of commercially available micronized cellulose powder in the morning, and only 1 puff micronized cellulose powder in the evening (test treatment, TT) or 1 puff mometasone furoate, Bid (reference treatment, RT). Participants were allowed to take oral antihistamine or eye drops they wished. The symptom scores were recorded before and after the treatment. Mean values for the sum of all scores were calculated. The pre- vs post-treatment differences were compared using t-test. Also the decrease of the symptom scores between groups were compared using t-test. The efficacies were analyzed using Chi-2 tests.

**Results:** Both treatments could reduce the symptom scores significantly (P<0.001), and the improvement rates were similar (P=0.052). Total efficacies of RT and TT were 95% and 100% respectively without significant differences (P=0.244). There were no adverse reaction found in both groups.

**Conclusions:** The combination of micronized cellulose powder and locally applied glucocorticoids could reduce the dose of glucocorticoids, and the treatment is safe.

## A139 New strategy for atopic dermatitis therapy with modulation of calcium ion channels

### Woo Kyung Kim^1^, Yu Ran Nam^1^, Joo Hyun Nam^2^

#### ^1^Dongguk University Graduate School of Medicine; ^2^Dongguk University College of Medicine; ^3^Dongguk University Ilsan Hospital, Graduated School Dongguk University, Seoul, South Korea

##### **Correspondence:** Woo Kyung Kim – Dongguk University Ilsan Hospital, Graduated School Dongguk University, Seoul, South Korea

**Background:** Intracellular Ca^2+^ signaling *via* various calcium channels, such as Orai1, Transient receptor potential (TRP)A1, TRPV1, and TRPV3, has been shown to directly modulate not only inflammation but also barrier homeostasis, and inflammation. Ca^2+^ influx through these channels eventually generates intracellular Ca^2+^ signaling that results in different outcomes dependent on the individual Ca^2+^channel type, for example, keratinocyte proliferation and migration through Orai1, epidermal barrier formation and keratinocyte differentiation through TRPA1, and keratinocyte cornification through TRPV3. Therefore, a specific agonist/antagonist for each calcium channel is required for maintaining skin barrier homeostasis and for the treatment of atopic dermatitis.

**Objectives:** To identify applicable topical botanically derived chemicals for the treatment of atopic dermatitis.

**Method:** Novel modulators of Orai1, TRPA1, TRPV1, and TRPV3 were identified were identified by screening the 70% methanol (MeOH) extracts (plus their fractions) of 30 medicinal herbs and their constituents. The potencies of the activating and inhibitory compounds of each channel were determined by an automated patch clamp system. The biophysical properties of channel modulation by hit products were re-analyzed using conventional whole-cell patch clamp and fluorometric calcium imaging.

**Results:** We prepared 70% MeOH extracts of 30 medicinal herbs, performed bioassay-guided fractionation of the active extracts, and then isolated and identified the bioactive constituents. By performing the combination of automated and conventional whole-cell patch clamp studies, we found eight medicinal herb fractions for Orai1, four for TRPV1, two for TRPA1, and one for TRPV3 that showed >50% inhibition rates at 30 μg/mL. We also found three fractions with TRPA1 agonist activity. Further, we also identified chemical constituents that inhibit Orai1 (compound V: 95 ± 5% inhibition at 90 μM) and TRPV1 (compound M: 93.9 ± 2.45% inhibition at 90 μM; compound CYP: 61 ± 5% inhibition at 90 μM). Chemical constituents that showed agonist/antagonistic effects on TRPA1 and TRPV3 also will be discussed.

**Conclusions:** Considering that most regional plants have not been investigated chemically or pharmaceutically, they remain as untapped potential sources of topical agents for drugs and other application. We found major active components and chemical constituents of plant extracts for the modulation of various calcium ion channels, which may have potential clinical applications for atopic dermatitis.

**Limitations:** Extensive clinical studies of the lead compounds are needed to develop topical agents for atopic dermatitis that relate to skin barrier functions.

## A140 Difference in the Systemic Bacterial Composition of Atopic Dermatitis Patients Compared to Healthy Subjects through Metagenomic Analysis of Urine

### Jung-Won Kim^1^, Min-Hye Kim^1^, Mina Rho^2^, Yeon-Mi Kang^1^, Kyung-Eun Yum^3^, Hyeon-Il Choi^3^, Jun-Pyo Choi^3^, Han-Ki Park^3^, Taek-Ki Min^4^, Young Joo Cho^1^, Bok-Yang Pyun^5^, Yoon-Keun Kim^3^

#### ^1^Ewha Womans University, School of Medicine, South Korea; ^2^Department of Computer Engineering, Hanyang University; ^3^Ewha Institute of Convergence Medicine, Ewha Womans University Medical Center; ^4^Soonchunhyang University Hospital; ^5^Soonchunhyang University

##### **Correspondence:** Jung-Won Kim – Ewha Womans University, School of Medicine, South Korea

**Background:** Microbial infection is one of the local factors that contribute to the pathogenesis of atopic dermatitis. However, many studies had been reported the systemic effect of the microorganism, especially of the lactic acid bacteria. Until now, they only have been studying the systemic effects of local microorganisms, not the systemic microbial distribution. No study has revealed that relationship between the systemic bacteria and atopic dermatitis. Here, we performed metagenomic analysis to determine systemic bacteria distribution of atopic dermatitis patients vs. healthy control subjects.

**Methods:** Twenty-eight patients with atopic dermatitis and 8 healthy control subjects were enrolled. Urine was obtained in all subjects and serum was obtained in eighteen atopic dermatitis patients. After genomic DNA was extracted from the urine and serum, 16s ribosomal DNA was amplified using the universal primer, sequenced through the next generation sequencer, and then the sequenced data was analyzed using bioinformatics.

**Results:** The bacterial composition was nearly identical between serum and urine. However, there was notable difference of bacterial composition in the urine of the normal control and the atopic dermatitis patients. In the control group, proportion of *Lactococcus, Leuconostoc, Lactobacillus, Lactobacillales(o)* were significantly higher than in the patients group, and that of *Alicyclobacillus, Propionibacterium, Streptophyta(o)* were increased in the patients group than in the control group. Pseudomonas was commonly found in the both groups. Before treatment, *Alicyclobacillus* and *Comamonadaceae* were frequently found, however their proportion were decreased and *Acinetobacter* and *Oxalobacteraceae(f)* were increased after treatment in the urine of atopic dermatitis patients.

**Conclusions:** We confirmed the systemic bacterial composition in the atopic dermatitis and normal controls through the metagenomic analysis of bacterial DNA in the urine and serum.

## A141 Occurrence and physiological function of immune complexes of food proteins and IgA in human saliva

### Hiroshi Narita^1^, Junko Hirose^2^, Kumiko Kizu^3^, Ayu Matsunaga^1^

#### ^1^Department of Food and Nutrition, Japan; ^2^Department of Food Science and Nutrition; ^3^Department of Life and Living

##### **Correspondence:** Hiroshi Narita – Department of Food and Nutrition, Japan

**A) Background**: We have revealed the occurrence and physiological function of immune complexes (ICs) of food proteins and IgA in human breast milk (Ref.1,2). Universality of these findings was examined this time in another exocrine fluid, human saliva.

**B) Methods**: ICs of several food proteins in human exocrine fluids were determined with sandwich ELISAs constructed with anti-individual protein and anti-human IgA antibodies. The IC fraction obtained from human pooled saliva by ammonium sulfate precipitation and gel filtration was administered orally for successive 6 days to 8 weeks old BALB/c mice fed casein-based commercially available mouse diet. At day 8, they were immunized with egg white proteins in Freund’s complete adjuvant and boosted at day 22 with egg white proteins in Freund’s incomplete adjuvant. Blood was drawn from orbital vein and serum ovalbumin and ovomucoid specific IgG1s were measured at day 36. As the control, free ovalbumin corresponding to its amount in the IC fraction was administered.

**C) Results**: Food proteins were detected as respective ICs in healthy human saliva. IgG1 production to ovalbumin and ovomucoid was significantly suppressed (p<0.05) in the IC administered mice in comparison with the control mice.

**D) Conclusions**: ICs of food proteins and IgA could be determined in human saliva as well as in breast milk. Evidence indicating their physiological function as the inducers of oral tolerance was proved in mice. ICs of food proteins and IgA in human exocrine fluids are thought to be “Natural Drinkable Vaccine”, functioning in the prevention of food allergy through induction of oral immune tolerance.

**E) References**

1) Biosci. Biotech. Biochem., 65, 1438-1440, 2001

2) Food and Nutr. Sci., 6, 221-233, 2015

## A142 Association between DNA hypomethylation at IL13 gene and allergic rhinitis in house dust mite-sensitized subjects

### Jingyun Li, Yuan Zhang, Luo Zhang

#### Beijing Institute of Otolaryngology

##### **Correspondence:** Jingyun Li – Beijing Tongren Hospital, China

**Background:** Allergic rhinitis (AR) is a complex disease, in which gene-environment interactions contribute to its pathogenesis. Epigenetic modifications such as DNA methylation play an important role in the regulation of gene function. As *IL13*, a pleiotropic cytokine, may be important in conferring susceptibility to AR, the aim of the present work was to assess the relationship between a CpG island methylation status at the upstream of *IL13*gene and house dust mite (HDM)-sensitized AR in Han Chinese subjects.

**Methods:** A total of 60 HDM-sensitized AR patients and 65 control subjects were enrolled as two independent cohorts from Beijing and Liaoning. MassARRAY EpiTYPER was used to systematically screen the status of DNA methylation in peripheral blood leukocytes. *IL13*mRNA expression was measured by real-time quantitative PCR.

**Results:** The mean level of methylation was decreased in the AR patient-group compared with the control-group (*P* = 0.01). Two of a total of 33 *IL13*CpG units analyzed (CpG units 24:25:26 and 38:39) showed significant differences in methylation status between the AR patient-group and the control-group; with DNA hypomethylation at CpG38:39 significantly associated with higher risk of HDM-sensitized AR in both independent cohorts and a combined cohort (Beijing: OR=1.22, 95%CI=1.03–1.43, *P*=0.018; Liaoning: OR=1.53, 95%CI=1.06–2.38, *P*=0.022; combined OR=1.23, 95%CI=1.06–1.43, *P*=0.006). Methylation level of CpG38:39 correlated negatively with both *IL13*mRNA expression and serum total IgE level.

**Conclusions:** DNA hypomethylation of *IL13* gene may be associated with increased risk of AR from HDM-sensitization.

## A143 Effect of dietary methyl donors on asthma and atopy is modified by MTHFR polymorphism

### Yean Jung Choi^1^, Hye Lim Shin^1^, Song-I Yang^2^, So-Yeon Lee^2^, Sung-Ok Kwon^3^, Young-Ho Jung^4^, Ji-Won Kwon^5^, Hyung Young Kim^6^, Ju-Hee Seo^7^, Byoung-Ju Kim^8^, Hyo-Bin Kim^9^, Se-Young Oh^3^, Ho-Jang Kwon^10^, Eun Lee^11^, Mi-Jin Kang^12^, Soo-Jong Hong^13^, Yun-Jeong Lee^14^, Joonil Kim^15^

#### ^1^Asan Institute for Life Sciences, South Korea; ^2^Hallym University Sacred Heart Hospital; ^3^Kyung Hee University; ^4^Bundang CHA Medical Center, CHA University School of Medicine; ^5^Seoul National University Bundang Hospital; ^6^Pusan National University Yangsan Hospital; ^7^Korea Cancer Center Hospital; ^8^University of Cincinnati College of Medicine, USA; ^9^Inje University Sanggye Paik Hospital; ^10^Dankook University College of Medicine; ^11^Asan Medical Center, University of Ulsan College of Medicine; ^12^Asan Institute for Life Science, Asan Medical Center; ^13^Childhood Asthma Atopy Center, Environmental Health Center, Asan Medical Center, University of Ulsan College of Medicine; ^14^Asan Medical Center, University of Ulsan College of Medicine; ^15^Asan Medical Center, Univers

##### **Correspondence:** Yean Jung Choi – Asan Institute for Life Sciences, South Korea

**Background and objective**

Recent data have shown that the prevalence of asthma and allergic disease continuously increase. Some diet can prevent asthma or allergic disease by epigenetic change, including DNA methylation. The aim of this study was to investigate the association between dietary methyl donors (folate, vitamin B2, vitamin B6) and the development of asthma and allergic sensitization in children.

**Methods**

Children aged 7-13 years in a Korean elementary school were surveyed in 2006 as part of the first Children’s Health and Environmental Research (CHEER) survey and 2,333 children were included in this study. Korean version of the International Study of Asthma and Allergies in Childhood (ISAAC) questionnaire and food frequency questionnaire (FFQ) were done by their parents. The skin prick test was performed using 18 common allergens in Korea. Genotyping for *MTHFR* (rs1801133) polymorphism was performed by TaqMan assay.

**Results**

Dietary folate intake was a protective factor for wheezing symptoms in the past 12 months (aOR, 0.69; 95% CI, 0.53-0.91) and asthma diagnosis (aOR, 0.73; 95% CI, 0.53-0.99) and vitamin B6 was also related to reduced risk of allergen sensitization (aOR, 0.74; 95% CI, 0.56-0.99). High intake of folate, vitamin B2 and vitamin B6 was a protective factor for allergen sensitization [(aOR, 0.48; 95% CI, 0.25-0.93), (aOR, 0.50; 95% CI, 0.26-0.95) and (aOR, 0.40; 95% CI, 0.21-0.76), respectively] and atopic wheezing symptoms in the past 12 months [(aOR, 0.17; 95% CI, 0,04-0.80), (aOR, 0.26; 95% CI, 0,07-1.01) and (aOR, 0.26; 95% CI, 0,07-0.99), respectively], especially in CT or TT genotype at nucleotide 677T *MTHFR* compared to those with low intake and CC at this position.

**Conclusions**

These results indicate that dietary methyl donors decrease the risk of asthma and atopy, which may be modified by *MTHFR* polymorphism.

**Funding**

This study was supported by a grant of the Environmental Health Center and Children’s Health and Environmental Research funded by the Ministry of Environment, Republic of Korea.

## A144 The effect of TSLP in a murine model of allergic asthma

### Joon Young Choi, Ji Young Kang, Seok Chan Kim, Sei Won Kim, Seung Joon Kim, Young Kyoon Kim, Chin Kook Rhee, Hea Yon Lee, Hwa Young Lee, Sook Young Lee

#### St. Mary’s Hospital, Seoul, South Korea

##### **Correspondence:** Joon Young Choi – St. Mary’s Hospital, Seoul, South Korea

**Objective:** Thymic stromal lymphopoietin (TSLP) is an epithelial-cell derived cytokine that may be important in initiating allergic inflammation. This study aimed to investigate whether TSLP reduced airway inflammation in a murine model of asthma. **Methods:** BALB/c mice were sensitized and challenged with ovalbumin (OVA), and the effect of TSLP on airway inflammation and airway hyperresponsiveness (AHR) was evaluated. Furthermore, we measured changes in various cytokines in the bronchoalveolar lavage (BAL) fluid when treated with TSLP. **Results:** We observed that TSLP exert a negative regulation on OVA mediated allergic airway inflammation. TSLP treatment reduced total cell counts and eosinophil counts in BAL fluid and AHR to methacholine. The Th2 cytokines, such as IL-4, IL-5 and IL-13 in BAL fluid also decreased after TSLP treatments. **Conclusions:** These results suggest that TSLP has a therapeutic potential for allergic asthma through inhibition of Th2 cytokine production.

## A145 Evaluation of Aspirin Hypersensitivity in Chronic Rhinosinusitis Patients

### Tae Kyung Koh^1^, Sung Wan Kim^1^, Kun Hee Lee^2^, Chul Kwon^1^, Joong-Saeng Jo^1^, Sung-Hwa Dong^1^, Young Seok Byun^1^

#### ^1^Kyung Hee Medical Center, South Korea; ^2^Kyung Hee University Hospital at Gangdong

##### **Correspondence:** Tae Kyung Koh – Kyung Hee Medical Center, South Korea

**Background and Objectives**: FAspirin-exacerbated respiratory disease (AERD) is a clinical tetrad of nasal polyps, chronic hypertrophic eosinophilic sinusitis, asthma, and sensitivity to aspirin. Aspirin provocation test is the gold standard for diagnosing AERD. The aim of this study was to evaluate the clinical features and prognosis after surgical treatment in chronic rhinosinusitis (CRS) patients who have aspirin hypersensitivity.

**Subjects and Method**: FWe conducted an analysis of 100 CRS patients who underwent endoscopic sinus surgery at the Department of Otorhinolaryngology of our hospital from October 2012 to March 2013. We measured nasal volume change and symptom change before and after the aspirin nasal provocation test (ANPT), and examined patient’s asthma history, allergy, Lund-Mackay score (LMS), total immunoglobulin E, peripheral eosinophil percentage, and objective measurement relapse.

**Results**: FChronic rhinosinusitis patients with nasal polyps (CRSwNP) were more likely to have a positive ANPT test results compared to patients without nasal polyps (CRSsNP) (21.4% vs. 5.5%). ANPT (+) group had higher LMS and required more revision endoscopic sinus surgery than ANPT (-) group. These results were similar to those of CRSwNP compared with CRSsNP.

**Conclusion**: F LMS and recurrence rates were higher in ANPT (+) compared to ANPT (-). Especially, recurrence rates were higher in ANPT (+) regardless of nasal polyp. Thus, careful endoscopic examination is required at follow up if CRS patients showed positive ANPT results.

## A146 Chronic cough without wheezing in young children as a manifestation of chronic sinusitis

### Charles Song

#### Harbor-UCLA, USA

**Background**: Chronic cough without wheezing in young children often presents diagnostic challenge.

**Objective:** To investigate the usefulness of nasal endoscopy in differentiating bacterial sinusitis from viral upper respiratory infection, cough variant asthma, allergic rhinitis, and GERD.

**Method:** We have retrospectively analyzed data from 14 young children under the age of 5 who presented in our clinic with chronic cough without wheezing. Nine children were evaluated with nasal endoscopy.

**Results:** All of 9 (100%) children evaluated with nasal endoscopy had thick purulent discharge in the nasopharynx and five (56%) of them had adenoid enlargement for the age. Ten of 13 (77%) children given antibiotic treatment reported symptom resolution in their two week follow-up appointment.

**Conclusion:** Using nasal endoscopy, we found that the majority of children with chronic cough without wheezing had chronic bacterial sinusitis and had a striking response to an appropriate antibiotic therapy. Adenoid hypertrophy may be the cause or, more likely, the result of chronic upper airway infection.

## A147 Expression of muscarinic receptors and effect of tiotropium bromide on chronic asthma according to age in a murine model

### Ji Young Kang^1^, Hwa Young Lee^1^, In Kyoung Kim^1^, Sei Won Kim^1^, Chin Kook Rhee^1^, Seung Joon Kim^1^, Seok Chan Kim^1^, Sook Young Lee^1^, Young Kyoon Kim^1^, Soon Seog Kwon^2^, Joon Young Choi^1^

#### ^1^Seoul St. Mary’s Hospital, South Korea; ^2^Pucheon St.Mary’s Hospital

##### **Correspondence:** Ji Young Kang – Seoul St. Mary’s Hospital, South Korea

**Background:** The aim of this study was to investigate the expression of muscarinic receptors and resultantly the effect of muscarinic antagonist, tiotropium bromide depending on aging process in a murine model of chronic asthma.

**Methods:** Different aged female BALB/c (6 weeks old, 9 and 15 months old) female BALB/c mice were sensitized and challenged with ovalbumin (OVA) about for three months. Tiotroipum bromide of 0.1 mM was administered via intranasal route before OVA challenge. We measured cell counts and Th2 cytokines in bronchoalveolar lavage fluid and airway hyperresponsiveness. Parameters of airway remodeling and the expression of muscarinic receptor subtypes, M2/M3, were assessed.

**Results:** Airway resistance decreased in aged (9 and 15 months old) OVA group than young (6 weeks old) OVA group. Inflammatory cells including eosinophils had a decreased tendency according to age among the OVA groups. Administration of tiotropium showed the improvement of the infiltration of inflammatory cells and Th2 cytokines such as IL-4 and IL-13. Goblet cell hyperplasia and smooth muscle hypertrophy, pivotal markers of airway remodeling, ameliorated in the tiotropium treated group than OVA group in the three aged group. The OVA group had an increased expression of M3 subtype and a decreased expression of M2 subtype according to age.

**Conclusion:** This study shows that tiotropium bromide could have the effect on not only airway inflammation but also remodeling regardless of age and the effect might be related with the pattern of expression of muscarinic receptors subtype.

## A148 Discrimination between non-eosinophilic and eosinophilic chronic rhinosinusitis with nasal polyps

### Pona Park^1^, Hong Ryul Jin^1^, Dong-Kyu Kim^2^, Dae Woo Kim^1^

#### ^1^Boramae Medical Center, South Korea; ^2^Chuncheon Sacred Heart Hospital

##### **Correspondence:** Pona Park – Boramae Medical Center, South Korea

**Background:** Chronic rhinosinusitis with nasal polyps (CRSwNP) is classified as eosinophilic or non-eosinophilic CRSwNP depending on histopathological features. However, there are few useful markers for the differential diagnosis of CRSwNP. Therefore, we sought to investigate useful surrogate markers for CRSwNP endotyping in Asian patients.

**Methods:** A total of 81 patients (45 with non-eosinophilic nasal polyps and 36 with eosinophilic nasal polyps) were enrolled. Clinical information and computed tomography (CT), endoscopic, and histological findings were investigated. Tissue samples were analyzed for total IgE protein concentration levels and for mRNA expression levels of interleukin (IL)-4, IL-5, IL-13, interferon (IFN)-γ, tumor necrosis factor (TNF)-α, IL-17A, IL-22, IL-23p19, transforming growth factor (TGF)-β1, TGF-β2, TGF-β3, and periostin. Immunostaining assessment of Ki-67 as a proliferation marker was performed.

**Results:** Non-eosinophilic CRSwNP showed greater localized and maxillary involvement but lesser olfactory involvement on CT in early stage disease compared with eosinophilic CRSwNP. In addition, ethmoidal/maxillary CT scores, indicating ethmoidal dominant involvement, were positively correlated with levels of T_H_2 inflammatory markers, such as IL-5, periostin mRNA expression and total IgE levels in nasal polyp (NP) tissues, whereas scores were inversely correlated with levels of the T_H_1 cytokine, IFN-γ. In non-eosinophilic NPs, Ki-67 expression was upregulated, especially in epithelia. Additionally, epithelial ingrowing patterns such as pseudocysts were more frequently observed in histologic and endoscopic evaluations of non-eosinophilic NPs compared with eosinophilic NPs. A criterion combining the cutoff level (2,167) of ethmoidal/maxillary CT scores and the presence of pseudocysts on endoscopic examination yielded a sensitivity of 100.0% and a specificity of 71.0% for the diagnosis of non-eosinophilic CRSwNP.

**Conclusion:** We demonstrate that the combination of ethmoidal/maxillary CT scores and the presence of pseudocysts by endoscopy may be used as a surrogate marker to distinguish non-eosinophilic CRSwNP from eosinophilic CRSwNP in Asian patients.

## A149 Significant reduction in allergic features in the offspring of mice supplemented with specific non-digestible oligosaccharides during lactation

### Astrid Hogenkamp

#### Utrecht University, Netherlands

**Background:** Earlier it was shown that maternal supplementation with non-digestible carbohydrates during pregnancy led to a significant reduction in the development of several allergic asthma features in adult offspring. In the current study, it was investigated whether maternal supplementation during lactation only would have similar effects.

**Method:** Mice were mated 2 weeks after arrival at 10 weeks of age. Directly after birth of the offspring, mice in the lactation group were transferred to the AIN93 control diet supplemented with short-chain galacto- and long-chain fructo-oligosaccharides (scGOS/lcFOS; ratio 9:1). Mice in the sham and control groups were kept on control AIN93. The male offspring were sensitized to OVA at the age of 6 weeks, with the exception of those in the sham group, and the acute allergic skin response was measured at the age of 8 weeks. Airway hyperreactivity to metacholine was measured after 3 consecutive airway challenges with OVA aerosol.

**Results:** Although the acute allergic skin response and the airway hyperreactivity did not differ between the control group and the lactation group allergic inflammation was significantly down-regulated by the dietary intervention during lactation. Total cells numbers, and percentages of eosinophils and lymphocytes in the bronchoalveolar lavage fluid as markers for allergic inflammation were significantly decreased in the offspring of dams fed scGOS/lcFOS during lactation. Analysis of total and OVA specific immunoglobulin levels showed that the specific diet did lead to lower levels of OVA-specific and total IgG1 levels. OVA-specific IgE levels did not differ between the lactation and the control group, although levels of total IgE were significantly lower in the lactation group.

**Conclusion:** Maternal supplementation with scGOS/lcFOS during lactation did down-regulate allergic inflammation in the lungs. In addition immunoglobulin levels, relevant for allergic disease, were down-regulated as well. In contrast, allergic skin reactions and lung functions were not affected. These data are comparable to studies performed earlier in which dietary intervention with scGOS/lcFOS was performed during pregnancy only although in these animals skin reactions and lung function were affected as well. Altogether, our data suggest that early life dietary intervention with non-digestible carbohydrates may be beneficial for the allergic outcome later in life, which may also be highly relevant for the development of atopic disease in humans.

## A150 Allergenicity assessment of hydrolysed infant formula; A multicenter comparison of a mouse model and a Guinea pig model for cow’s milk allergy

### Leon Knippels^1,2^, Betty C.a.m. Van Esch^1,3^, Jolanda Van Bilsen^4^, Prescilla V. Jeurink^3^, Marjan Gros^5^, Johan Garssen^1,3^, Joost J Smit^6^, Raymond H. H. Pieters^6^

#### ^1^Utrecht University; ^2^Senior Scientist; ^3^Nutricia Research; ^4^Tno; ^5^Friesland Campina; ^6^Institute for Risk Assessment Sciences

##### **Correspondence:** Leon Knippels – Senior Scientist

This study is part of a multi-phase project aiming to validate a mouse model to assess the potential allergenicity of hydrolysed infant formulas. The sensitizing properties of 3 partially hydrolysed whey proteins (pWH-A, -B and -C) were investigated in the mouse model as well as the classically used guinea pig model. Mice and guinea pigs were orally sensitized with whey by gavage or *ad libitum* via the drinking water respectively. In mice, whey-IgE, acute allergic skin responses, mMCP-1 release, body temperature and anaphylactic shock symptoms were determined upon oral challenge in 4 research centers. In the guinea pigs anaphylactic shock symptoms upon intravenous challenge were measured as a single parameter at 1 center. Elevated levels of whey-specific IgE/IgG1 were detected in whey-sensitized mice in all centers, although the group-average did not reach significance for IgE in center 2. In contrast to whey-sensitized mice, no acute skin response or mMCP-1 release upon whey challenge was observed in pWH-A-treated mice which corresponded with the absence of anaphylactic shock symptoms in both the mouse and guinea pig model. For pWH-B and pWH-C, that showed positive in the guinea-pig ASA-test, results in mice were inconclusive. None of the centers was able to differentiate between the residual sensitizing capacities of the pWH-B and -C based on a single elicitation parameter and results per center differed. To determine the potential influence of an altered microbiota at the different locations on the sensitizing capacity, an analysis of the microbial composition at the start and end of the study was conducted. Results show that for a well-balanced prediction on the potential allergenicity of hydrolysed infant formulas a multiple parameter model is needed. Therefore, it is concluded that the murine model is suitable to give a safe and nuanced prediction on the allergenicity of pWH’s. A future challenge is to develop an overall scoring system for proper risk assessment, taking all assessed parameters into account.

## A151 Clinical significance between the allergic test and serum eosinophil cationic protein

### Boo-Young Kim, Soo Whan Kim

#### College of Medicine, the Catholic University of Korea, South Korea

##### **Correspondence:** Boo-Young Kim – College of Medicine, the Catholic University of Korea, South Korea

**Introduction:** the eosinophil cationic protein (ECP) is a small polypeptide that originates from activated eosinophil granulocytes. In other studies, the human neutrophils from allergic pateitns were able to produce ECP by IgE-dependent mechanism. Eosinophils have been shown to contribute to T-lymphocyte activation and an increased inflammatory responses during allergic inflammation.

**Objectives:** the purpose of this study was to evaluate the relationship between allergic test; skin test and multiple allergosorbent test system (MAST); and ECP count.

**Methods:** A total of 975 patients who underwent skin test or MAST were included in this studies. We tested the serum ECP, total Ig E, eosinophil counts, datas of skin test, datas of MAST in all patients. Various parameters were compared to ECP using the Mann-Whitney U test. Pearson’s correlation coefficient was used to analyze the relationship between variables and ECP in each datas.

**Results:** In total, 975 patients (578 men and 367 women) were included in our study. Regression analysis showed that ECP was significantly correlated with the asthma (r = 0.76, p =0.023), the score of MAST for Dermatophagoides farinae (r = 0.144, p = 0.000), the score of MAST for mite, Dermatophagoides pteronyssinus (r = 0.123, p = 0.000), and the score of skin test for Der.f (r = 0.171, p =0.032). There were no difference between the ECP and other datas significantly.

**Conclusions:** There might be positive relationship between the ECP and MAST for Dermatophagoides.

## A152 Hydroclorothiazide-induced acute non-cardiogenic pulmonary edema

### Ramon Lleonart

#### Hospital Universitari De Bellvitge, Spain

Hydroclorothiazide (HCTZ) is widely used in the treatment of essential hypertension. It may be given alone or together with other antihypertensive agents in fixed combination preparations. The most frequent adverse reactions are hypotension and disturbances in the level of serum electrolytes. Nevertheless severe adverse reactions such as shock have also been described.

We describe 5 patients that have suffered episodes of sudden-onset pulmonary edema immediately after the ingestion of HCTZ.

Five female patients aged between 54 and 76 years-old presented with episodes of dyspnea, malaise, chills, dizziness, fever and, in two of the patients, loss of consciousness. Chest X-rays showed bilateral interstitial infiltrates suggestive of pulmonary edema. All the patients were admitted in hospital, three had a severe respiratory failure and two of them were intubated and mechanically ventilated. Common causes of shock and pulmonary edema were ruled out in all cases. All the patients had received a dose of HCTZ (12.5-50mg), 10-60 minutes before the onset of symptoms but all them were discharged without being diagnosed.

Cutaneous (prick and intradermal) tests and patch tests to HCTZ were negative. The 5 patients were diagnosed in the Allergy Unit as having HCTZ-induced non-cardiogenic pulmonary edema. The close temporal relationship between the ingestion of HCTZ and the onset of symptoms, the clinical pattern, the fast recovery after-withdrawing the drug and the relapse with re-exposure, supported this diagnosis. According to Naranjo adverse drug reaction probability scale, in 4 of the patients the diagnosis was considered definite and in the fifth patient, the score indicated a probable relationship between the hypersensitivity reactions and HCTZ.

We report a potentially fatal adverse drug reaction due to HCTZ that was not recognized during the patients’ hospital stay.

All the cases reported affected women according to female predominance in previously published literarure

Doctors should consider this potentially life-threatening adverse drug reaction early and withdraw the drug in such an event.

## A153 A Synbiotic Mixture of Scgos/Lcfos and *Bifidobacterium Breve* M-16V Is Able to Restore the Delayed Colonization of *Bifidobacterium* Observed in C-Section Delivered Infants

### Christophe Lay^1^, Kaouther Benamor^1^, Chua Mei Chen^2^, Jan Knol^1^, Charmaine Chew^1^, Voranush Chongsrisawat^3^, Anne Goh^4^, Wen Chin Chiang^4^, Rajeshwar Rao^4^, Surasith Chaithongwongwatthana^3^, Nipon Khemapech^3^

#### ^1^Danone Nutricia Research, Netherlands; ^2^Kk Hospital, Singapore; ^3^King Chulalongkorn Memorial Hospital, Thailand; ^4^Kk Women’s and Children’s Hospital, Singapore

##### **Correspondence:** Christophe Lay – Danone Nutricia Research, Netherlands

**Introduction**

Infants born by C-section miss the exposure to the maternal vaginal microbiota and this absence of microbial inoculation has been associated with a delayed colonization of commensal bacterial members such as *Bifidobacterium*. This compromised microbial inoculation may impact the health of the newborn. Epidemiological data from cohort studies indicate associations between C-section and, immune and metabolic disorders such as asthma, and obesity.

The objective of this study was to determine the effect of a specific mixture of short-chain galactooligosaccharides and long-chain fructooligosaccharides (scGOS/lcFOS, ratio 9:1) and *Bifidobacterium breve* M-16V in restoring the delayed colonization of *Bifidobacterium* observed in term C-section delivered infants.

**Methods**

In a multi-country, double-blind, randomised controlled study, 153 infants born by elective C-section were randomised to receive (1) an infant formula supplemented with scGOS/lcFOS (0.8g/100ml) and *B. breve* M-16V (7.5×10^8^CFU/ 100ml), or (2) a formula supplemented with scGOS/lcFOS (0.8g/100ml), or (3) a control formula from birth until age 4 months. As a reference group, 30 vaginally born, breast fed infants were studied in parallel. Stool samples were collected at day 3, day 5, week 4, week 8, week 12, week 16, and week 22 (6 weeks post-intervention). The proportion of bifidobacteria, different *Bifidobacterium* species such as *B. longum* and the probiotic strain *B. breve* M-16V were determined with molecular tools, pH and SCFA were also measured in the stool samples.

**Results**

The data confirm the delayed colonization of *Bifidobacterium* in C-section delivered infants. The synbiotic supplementation resulted in a significant higher proportion of bifidobacteria from the first days of life (p=0.006) and this bifidogenic effect remained significant until 1 month of age (p=0.029) compared to the control group. The prebiotic group showed a significant bifidogenic effect at age 1 month (p=0.048) compared to the control group. In the synbiotic arm, *B. breve* M-16V was still detected in 37 % of the infants at week 22 indicating a persistence of the probiotic strain. A significant lower faecal pH and a higher acetate level were observed in the synbiotic group from the first days of life and this remained significant until 1 month of age compared to the control group. A lower number of subjects with adverse events of eczema/atopic dermatitis was reported in the synbiotic group (n=3) compared to the control (n=10), and prebiotic group (n=9), after correction for family allergy history.

**Conclusion**

An infant formula supplemented with scGOS/lcFOS and *B. breve* M-16V is able to restore the delayed colonization of *Bifidobacterium* in C-section delivered infants from the first days of life. This bifidogenic effect is associated with a positive modulation of the gut ecosystem milieu. These effects may have potential long term health benefits.

## A154 Atopic characteristics of patients with asthma-COPD overlap syndrome

### Ji Young Yhi, Sang-Heon Kim, Dong Won Park, Ji-Yong Moon, Tae Hyung Kim, Jang Won Sohn, Dong Ho Shin, Ho Joo Yoon, Seok Hyun Cho

#### Hanyang University College of Medicine, South Korea

##### **Correspondence:** Ji Young Yhi – Hanyang University College of Medicine, South Korea

**Background:** While the diagnostic criteria for asthma-COPD overlap syndrome (ACOS) is lacking, it is often perceived to share both characteristics of asthma and COPD. However, the atopic characteristics of ACOS has not been well described. We aimed to assess the atopy-related characteristics of ACOS in comparison with COPD.

**Methods:** In this retrospective study, ACOS was defined as physician-diagnosed cases and both ACOS and COPD met the spirometric standards (FEV_1_/FVC <0.7). We compared the clinical characteristics of atopy between ACOS and COPD.

**Results:** We enrolled the patients with physician-diagnosed ACOS (n = 207) and COPD (n = 258) from the medical records in a university hospital. The patients with ACOS had younger age and lower FEV_1_ than COPD patients. With regard to skin prick test (SPT) responses, the overall positive rate was higher in ACOS than in COPD (22.3% vs. 14.3%, *P* = 0.027). However, the positive SPT responses to each allergen were not different between ACOS and COPD. In addition, there was no significant difference in levels of peripheral blood eosinophil percentage and total IgE.

**Conclusions:** In conclusion, SPT positivity was higher in ACOS, but the other features of atopy were not different between ACOS and COPD. In the diagnosis of ACOS from COPD, SPT positivity needs to be considered as an important clinical feature.

## A155 Perceptions and practices of severe asthma and asthma-COPD overlap syndrome among specialists: A questionnaire survey

### Sang-Heon Kim^1^, Ji-Yong Moon^1^, Jae-Hyun Lee^2^, Ga Young Ban^3^, Sujeong Kim^4^, Mi-Ae Kim^5^, Joo-Hee Kim^6^, Min-Hye Kim^7^, Chan-Sun Park^8^, Hyouk-Soo Kwon^4^, Jae-Woo Kwon^9^, Jae Woo Jung^10^, Hye-Ryun Kang^11^, Jong-Sook Park^12^, Tae-Bum Kim^13^, Heung Woo Park^11^, You Sook Cho^4^, Kwang-Ha Yoo^14^, Yeon-Mok Oh^13^

#### ^1^Hanyang University College of Medicine, South Korea; ^2^Yonsei University College of Medicine; ^3^Ajou University School of Medicine; ^4^Asan Medical Center, University of Ulsan College of Medicine; ^5^Bundang Medical Center, CHA University; ^6^Hallym University College of Medicine; ^7^Ewha Womans University, School of Medicine; ^8^Inje University College of Medicine; ^9^Kangwon National University College of Medicine; ^10^Chung-Ang University Hospital; ^11^Seoul National University Hospital; ^12^Soonchunhyang University Bucheon Hospital; ^13^University of Ulsan College of Medicine; ^14^Konkuk University College of Medicine

##### **Correspondence:** Sang-Heon Kim – Hanyang University College of Medicine, South Korea

**Background:** Severe asthma and asthma-COPD overlap syndrome (ACOS) are gaining more and more attention since these diseases are hard to control and often associated with poor clinical outcomes. However, the diagnosis and managements varies depending on clinicians because there is no agreement on the definition and therapeutic approaches in these diseases. To evaluate the current understandings and clinical practices on severe asthma and COPD among asthma and COPD specialists in Korea, We designed a questionnaire survey using e-mail and web-based platform.

**Methods:** Subjects were selected based on their clinical specialty from the members of the Korean Academy of Asthma, Allergy and Clinical Immunology and the some study groups of the Korean Academy of Tuberculosis and Respiratory Diseases. Of 432 subjects who received e-mail, 103 subjects (58 allergists and 37 pulmonologists) responded and submitted their answers by online administration.

**Results:** Regarding severe asthma features, the most common type was asthma aggravation by stepping down treatment (21.8%) followed by frequent exacerbation (20.6%), uncontrolled asthma despite higher treatment step (13.4%) and severe exacerbation (12.6%). The subjects responded that the proportion of severe asthma was 13.8% of the asthma patients in their clinic and there was no difference between allergists and pulmonologists. ACOS was estimated to be 20.7% of asthma, 37.9% of severe asthma and 29.5% of COPD, while allergists gave more proportions of ACOS among COPD than pulmonologists (35.4% vs. 22.6%). Regarding the diagnostic criteria for ACOS among asthma patients, smoking history (84.5%), persistently low FEV_1_ (80.6%) and low FEV_1_ variation (71.8%) were most frequently chosen for major criteria. Contrarily, the highly selected major criteria for ACOS among COPD patients were high FEV_1_ variation (85.4%), positive bronchodilator response (77.7%) and personal history of allergy (75.7%).

**Conclusions:** The asthma and COPD specialists had diverse view on the perceptions and clinical practices on severe asthma and ACOS. These heterogeneity needs to be considered in developing guidelines and health policies of severe asthma and ACOS.

## A156 A case of surgical diagnosed eosinophilic enteritis with intussusception in adult patient

### Sang-Rok Lee

#### Cheongju St Mary’s Hospital, South Korea

**Introduction**

Eosinophilic enteritis is rare diseases, which is eosinophilic infiltration of colon mucosa without definite cause of eosinophilia. Patient who has eosinophilic enteritis with intussusceptions is extremely rare in adult, and this case is second report in Korea.

**Case**

Twenty-two year-old man visited office due to two months lasting abdominal discomfort and diarrhea. He had no underlying medical diseases, and any other allergic history and family history except being allergic to pupa. On physical examinations, RUQ tenderness had shown.

Laboratory findings showed Hemoglobin level 16.6 mg/dL, platelet count 265,000/mm3, WBC counts 6300/mm3 with differential 16.7% of eosinophils on corrected blood chemistry. Blood chemistry excluded the presence of other organ involvement, such as kidney and liver. Serologic marker of parasite and stool study which might cause gastrointestinal symptoms and peripheral eosinophilia such as Ancylostoma, Anisakis, Ascaris, Strongyloides, Toxocara, and Trichinella were done, but any of them had not shown positive results, only positive response to mite and cockroach on MAST. There is no evidence of eosinophilic infiltration on lung and heart in chest x-ray, pulmonary function test and echocardiogram. Performed computed tomography showed proximal small bowel intussusception in ascending colon. Pathologic findings represented eosinophilic infiltration of entire bowel layers. Patient’s symptoms were relieved after surgery and followed blood chemistry showed improvement of peripheral eosinophilia 16.7% to 3.5%.

**Conclusion**

Eosinophilic gastroenteritis is diagnosed by eosinophilic infiltration of gastrointestinal tract without other causes of eosinophilia. The most common cause of disease is exposure to sensitive food allergen, but the pathogenesis is not well-known. Treatment is based on limited evidence and varies based upon symptoms. In this case, uncertain cause of symptom derive to surgery for diagnostic and therapeutic method. We suggest further clinical experiences and studies would be necessary to make out disease.

## A157 Reference values of total IgE in estonian children

### Kaja Julge^1,2^, Maire Vasar^1^, Maire Vasar^2^, Tiia Voor^2^, Tiina Rebane^1^

#### ^1^Children’s Clinic of Tartu University Hospital; ^2^Department of Pediatrics of University of Tartu, Estonia

##### **Correspondence:** Kaja Julge – Children’s Clinic of Tartu University Hospital

**Background:** The value of total IgE varies in wide limits (0 – 5000 kU/l) and depends on a lot of factors: genetics, allergy, parasitic infection, age, gender, immune status and geographic region. The aim of this study was to find out the factors influencing the values of total IgE in Estonian children and to establish own reference values.

**Methods:** The study group comprised 385 children from two prospective allergy studies followed from birth up to the age 16 years. Data about allergy symptoms were collected from the interview-questionnaires, clinical examinations were carried out and skin prick tests were made with the most common food and inhalant allergens during the follow-up investigations. The measurements of total IgE level in sera were made at birth, 3, 6, 12, months and 2, 5, 10/12, 16/18 years of life using UniCAP method.

**Results:** The cord blood IgE level did not have any predictive value for allergy development during the first 12 years of life. The value of total IgE increased with the age from 0.4 (95%CI 14.4) kU/l at 3 months of age up to 37.0 (95%CI 45.5) kU/l at the age of five years. The teenage reference value at 10/12 years was lower (34.2 (95%CI 41.2) kU/l) as compared to the preschool children and at the age of 16/18 years (30.0 (95%CI 38.7). Males had higher IgE values in comparison with females at every age, but statistically significant was the difference only at 6 months (3.28 vs. 1.76 kU/L; p=0.01) and at two years (21.8 vs. 11.9 kU/l; p=0.01) of age. The total IgE was higher in children with allergic diseases as compared to non-allergic only at the age of 10/12 years (75.8 vs. 44.3 kU/l; p=0.008), which might indicate quite high prevalence of parasites in non-allergic children. We tested IgE antibodies against *Ascaris* in children with total IgE level over 20 kU/ at 6 and 12 months and over 200 kU/l at 2, 5, 10/12 and 16/18 years of life. Antibodies against *Ascaris* were found in 29 of 50 preschool children and in 31 of 56 teenagers.

However, there was positive correlation between total IgE and atopy considering positive skin prick test results and presence of allergen specific IgE antibodies in sera in all age groups.

**Conclusion**: The peak value of total IgE was at the age of five and not in teenage as usually referred to in the laboratory manuals. There was positive correlation between total IgE and skin prick test results, allergen-specific IgE antibodies and IgE antibodies against *Ascaris* in blood but not with allergic diseases except at the age of 10/12 years.

## A158 A case of eosinophilic granulomatosis with polyangiitis accompanied by rapidly progressive glomerulonephritis

### Yu Jin Kim^1^, Sang Min Lee^1^, Shin Myung Kang^1^, Sojeong Kim^1^, Sun Young Kyung^1^, Sung Hwan Jeong^1^, Jeong-Woong Park^1^, Hyunjung Hwang^1^, Yong Han Seon^1^, Sanghui Park^2^, Sang Pyo Lee^1^

#### ^1^Gachon University Gil Medical Center, Incheon, South Korea; ^2^Ewha Womans University School of Medicine, Seoul, South Korea

##### **Correspondence:** Yu Jin Kim – Gachon University Gil Medical Center, Incheon, South Korea

A 58-year-old man visited local clinic complaining of malaise, weight loss, fever, and dyspnea. Complete blood count was performed, which revealed eosinophilia in peripheral blood. He received antibiotics for 3 weeks, however his symptoms were not alleviated, and multiple purpuric papules on both ankles, left forearm, and buttock with acute renal dysfunction developed. Then, he was referred to university hospital. Pulmonary function test was carried out, which yielded decreased lung function with positive bronchodilator response. Kidney biopsy and skin biopsy were performed, and histological examination showed acute necrotizing crescentic glomerulonephritis and leukoclastic vasculitis in skin, which led to the diagnosis of Churg-Strauss syndrome (eosinophilic granulomatosis with polyangiitis, EGPA) combined with rapidly progressive glomerulonephritis (RPGN). The patient received pulse steroid therapy with parenteral methylprednisolone followed by oral prednisolone. Clinical and laboratory findings improved dramatically and remission was attained rapidly. The patient continued to be in remission for 5 months. EGPA should be considered in asthma patients who present with severe systemic symptoms and eosinophilia. Progressive renal insufficiency can occur during the acute phase of EGPA accompanied by renovascular involvement. Prompt and aggressive treatment with systemic corticosteroid is mandatory to control disease activity and to achieve remission.

## A159 Associations Between Infectious Diseases and Urticaria

### Marius Iordache

#### St. Spiridon Emergency Hospital, Romania

**Background:** Urticaria is a frequent skin disease but the majority of cases have an unknown cause (idiopathic urticaria). There is a strong association between infectious triggering factors in the pathogenesis of Acute Urticaria (AU) and infectious triggering factors may also be involved in Chronic Urticaria (CU).

**Methods:** We determined the prevalence of bacterial, viral and parasitic infectious diseases in 217 patients (169 females and 48 males) from North-Eastern Romania with age from 16 to 86 years, admitted in our department in the last 6 months with acute episodes of urticaria (107 females and 27 males) and chronic idiopathic urticaria (62 females and 21 males). In order to identify the infectious triggers, blood samples were used to evaluate the level of antibodies for *Toxocara cannis*, *Toxoplasma gondii*, Herpes simplex virus, Epstein–Barr virus, cytomegalovirus and measles virus. Additionaly, microscopic stool examination and fecal antigens essays for *Helicobacter pylori* and *Giardia lamblia*were performed. Serum anti-HCV antibodies and HBs antigen were assessed.

**Results:** The most frequent infectious agent detected in cases with AU and CU was *Giardia lamblia* (17.97% - 21 cases with AU and 18 cases with CU), followed by *Toxocara cannis* (8.29% - 11 cases with AU and 7 cases with CU), hepatitis C virus (5.99% - 8 cases with AU and 5 cases with CU), *Helicobacter pylori* (5.99% - 8 cases with AU and 5 cases with CU) and hepatitis B virus (1.38% - 2 cases with AU and 1 case with CU). Acute infections with Epstein–Barr virus (2 patients) and measles virus (1 patient) were diagnosed only in AU and no cases were found among patients with CU. Only 3 patients (2 with AU and 1 with CU) were diagnosed with acute infection with *Herpes simplex* virus. All patients had negative results for cytomegalovirus IgM antibodies. Parasitic infections with *Blastocystis hominis* were found in 6 patients (5 cases with AU and 1 case with CU) and one patient with CU presented *Ascaris lumbricoides*.

**Conclusions:** Infectious triggering factors, especially parasitic infections are prelevant in developing countries of the world and may play an important role not only in acute episodes of urticaria but also in chronic urticaria. Although we found that parasitic infections are present in both types of urticaria, further studies are needed to elucidate the association between infectious triggering factors and urticaria, and to identify new infectious triggers.

## A160 Sleep in Infants in Korea – Finding of BISQ Survey

### Yeongsang Jeong, Sohee Eun, Byung Min Choi, Ji Tae Choung, Wonhee Seo

#### Korea University, South Korea

##### **Correspondence:** Yeongsang Jeong – Korea University, South Korea

**Background**

Many social and cultural factors have influences on sleep patterns, and sleep condition of each child may be different from that of each other depending on the approaches and concerns of their parents. The objective of this study is to assess sleep condition of Korean infants.

**Method**

In 2014, a total of 627 Korean parents/caregivers of infants(48.6% boys) aged 0 till 18 months completed the internet-based expanded version of the Brief Infant Sleep Questionnaire(BISQ), which included specific questions of infants’ daytime and nighttime sleep patterns as well as their sleep related behaviors.

**Results**

Among 627 subjects, the number of boys are 305(48.6%). Most of the infants went to bed at about 21.80±1.72 hours at night and woke up at around 7.41±1.55 hours in the morning. The night sleep time duration was 9.43±1.75 hours, and the total sleep time for a day was 12.62±2.62 hours. 0-6 months old children group had less nighttime sleep duration (8.90±2.13 vs 9.78±1.23 vs 10.07±1.01, hours, p< 0.05) and more daytime sleep duration (4.30±2.49 vs 2.51±1.05 vs 2.36±1.23, hours, p< 0.05) than older children. There were no significant differences in number of night-waking between 0-6 months old children group and 7-12 months old children group, but 13-18 months old children group had significant less frequent night-waking (2.45±1.58 vs 2.38±1.59 vs 1.70±1.41). More than half of children in this survey sleep in their parents’ bed (59.8%), and most common bedtime routine was milk feeding until sleep (37.1%), and the second one was cradling (29.0%).

**Conclusion**

Korean infants have a short sleep time and bedtime routine and bed-share of Korean infants showed difference patterns compared with western countries. These results suggest that aggressive education for sleep condition is needed for Korean infants and their parents.

## A161 Increased Expression of Filaggrin, TSLP, Periostin, IL13 and IL-33 in Nasal Polyps

### Liang Zhang^1,2^, Ruby Pawankar^1^, Manabu Nonaka^3^, Miyuki Hayashi^1^, Shingo Yamanishi^1^, Harumi Suzaki^4^, Yasuhiko Itoh^2^, So Watanabe^5^, Hitome Kobayashi^5^

#### ^1^Nippon Medical School, Tokyo, Japan; ^2^The First Hospital Affiliated to China Medical University, Shenyang, China; ^3^Tokyo Women’s Medical University; Tokyo, Japan; ^4^Tokyo General Hospital, Tokyo, Japan; ^5^Showa University School of Medicine, Tokyo, Japan

##### **Correspondence:** Liang Zhang – Nippon Medical School, Tokyo, Japan

**Purpose**

Chronic rhinosinusitis (CRS), with nasal polyposis is a chronic inflammatory disease of the upper airways often associated with asthma and characterized by markedly increased numbers of eosinophils, Th2 type lymphocytes, fibroblasts, goblet cells and mast cells. The inflammation leads to a proliferative response in the extracellular matrix (ECM). Periostin is an ECM protein known to play a role in tissue remodeling in inflammatory diseases of the upper and lower airways. Furthermore epithelial-derived genes such as filaggrin have been highlighted in asthma or atopic dermatitis (AD) or both via its role in barrier function. Here we investigated the expression of periostin and filaggrin in nasal polyps (NP) from atopics and non-atopics in comparison with the nasal mucosa from patients with allergic rhinitis (AR) and its potential role in nasal polyposis.

**Methods**

Nasal polyp specimens and biopsies of nasal mucosa were obtained at surgery as part of the treatment for removal of NP or for hypertrophied turbinates. Immunoreactivity for periostin, TSLP, filaggrin and IL-13 in NP from atopic and non-atopic patients and in the nasal mucosa of patients with AR was analyzed by immunohistochemistry using the peroxidase-based Avidin-Biotin Complex (ABC) method. Cell counts were analyzed using an objective micrometer and the density of immunoreactivity was quantified by Image J analysis system. Real Time PCR was done for analyzing the mRNA expression of IL-33.

**Results**

Filaggrin immunoreactivity was detected in epithelial cells and inflammatory cells. The number of Filaggrin+ cells in the epithelium of patients with NP was significantly higher than that in the nasal mucosa of the AR patients. The number of filaggrin+ cells in the lamina propria of patients with NP was significantly higher than that in the nasal mucosa of the AR patients. There was no difference in the number of filaggrin+ cells between nasal polyps from atopic and non-atopic patients. Periostin immunoreactivity was mainly detected in the basement membrane and the density of immunoreactivity of periostin in NP was significantly higher in NP than in the nasal mucosa of the AR patients. TSLP was detected in epithelial cells and in immune cells and greater in NP than AR mucosa. There was no difference in the density of immunoreactivity of periostin between NP from atopic and non-atopic patients. IL-13+ cells and IL-33 expression were also higher in NPs and there was a good correlation between the IL-13+ cells and periostin.

**Conclusions**

Based on our previous findings of the high levels of IL-13 and TGF-beta in NP and the present findings of the increased expression of filaggrin and periostin in NP irrespective of the atopic status, filaggrin may potentially play a role in the barrier function and periostin may play a role.

## A162 Asymptomatic bacteruria increases the risk of edematous attacks in patients with hereditary angioedema due to C1 inhibitor deficency (C1-INH-HAE)

### Zsuzsanna Zotter^1,2^, Henriette Farkas^1^, Lilian Varga^1^, Nora Veszeli^1^, Eva Imreh^1^, Gabor Kovacs^2^, Marsel Nallbani^2^

#### ^1^Semmelweis University; ^2^Hungarian Defence Forces, Medical Center, Hungary

##### **Correspondence:** Zsuzsanna Zotter- Semmelweis University

**Introduction:** Although urinary tract infections (UTIs) are considered among the most common infectious disorders in humans, these usually follow an uncomplicated course. Various infections may have a role in inducing HAE attacks. Further, danazol treatment has been associated with hematuria. Our study intended to evaluate the abnormalities of the urinalysis of C1-INH-HAE patients.

**Methods:** Urine specimens contributed by 139 C1-INH-HAE patients at the annual control visits were studied retrospectively (RBC and WBC counts, microorganisms). We analyzed these laboratory parameters in relation of the clinical symptoms and in view of the long-term danazol therapy.

**Results:** Taking into account 3 randomly selected urine specimens, we found that the cumulative number of edematous attacks was higher in patients with than in those without bacteriuria (*p*=0.019, *p*=0.022, *p*=0.014). Considering the same patients (n=76), attack number was significantly higher (14.51 *vs.* 8.63) in patients with than in those without bacteriuria (*p*<0.0001). The cumulative incidence of microhematuria found upon a single or repeated examination was 74,8% after the annual check-up per patient. Taking into account an observation period of 3 years, the alterations detected in the urinary sediment were unrelated to treatment with or the dose of danazol.

**Conclusion:** The cumulative incidence of microhematuria was substantially higher compared with the historical data of healthy individuals. As regards the background of this phenomenon, we did not found any relationship with danazol therapy. The main finding of our study was that the increase incidence of edema was associated with bacteriuria. This finding emphasizes the triggering role of bacteriuria in the occurrence of edematous episodes.

Supported by OTKA grant 100886 and 112110.

## A163 Gastric Erosions Cause Spontaneous Urticaria Independent of Helicobacter Pylori

### Semen Zheleznov^1^, Galina Urzhumtseva^2^, Natalia Petrova^2^, Zhanna Sarsaniia^1^, Nikolai Didkovskii^1^, Torsten Zuberbier^3^

#### ^1^Scientific Research Institute of Physical-Chemical Medicine, Moscow, Russia; ^2^City Clinical Hospital N7, Moscow, Russia; ^3^Charité-Universitaetsmedizin Berlin, Berlin, Germany

##### **Correspondence:** Semen Zheleznov – Scientific Research Institute of Physical-Chemical Medicine, Moscow, Russia

**Background:** Investigation of the role of Helicobacter Pylori (HP) and erosions or ulcers (EU) of upper gastrointestinal tract (esophagus, stomach or duodenum) independently from each other in the development of spontaneous urticaria.

**Methods:** 36 adult patients, 5 with acute and 29 with chronic spontaneous urticaria were examined with upper gastrointestinal endoscopy (UGIE) and HP-testing in gastric biopsies, before and after treatment of the observed abnormalities.

**Results:** HP was found in 26 patients, or 72,2% (54,8; 85,8), what was less than across able-bodied population of Moscow - 87,9% (85,6; 90,0)^1^, difference insignificant. Wherein EU were found in 50,0% (32,9; 67,1) of patients, and gastric erosions in 41,7% (25,5; 59,2), what was 6,1 times more than in 1311 asymptomatic volunteers - 6,8% (5,5; 8,3)^2^. Two patients with most severe urticaria had duodenal ulcer. Despite these facts, only 23 patients (15,3%) reported mild gastrointestinal complaints after thorough questioning.

All 26 HP-positive patients received eradication therapy, and 4 HP-negative patients with EU received only antacid and antisecretory therapy. Second HP-determination by PCR after the therapy was carried out only in 13 patients with chronic spontaneous urticaria (11 HP-positive and 2 HP-negative at first UGIE, 10 patients with EU), as only 13 agreed for a new examination. Where in 7 from 11 HP-positive patients eradication was successful, and in 4 failed. From 7 patients with successful eradication remission was achieved in 2 cases, and in 5 cases there was no remission. In 4 patients with failed eradication remission was achieved also in 2 cases, and in 2 cases there was no remission. Fisher exact test with 1-tailed p=0,47, 2-tailed p=0,58 showed highly insignificant result of eradication therapy in the treatment of urticaria in followed patients.

At second UGIE, 3 patients from 10 with EU appeared with healed erosions. All of them showed complete remission of symptoms. Whereas 7 patients with unhealed EU, had no improvement at all. Fisher exact test showed significant difference with 1-tailed and 2-tailed p=0,008. Patients with acute spontaneous urticaria were not included in the statistical tests due to high possibility of spontaneous remission.

**Conclusions:** Erosions of upper gastrointestinal mucosa seem to have a very important role in the development of spontaneous urticaria, independently from HP.

**References**

1. German SV, Zykova IE, Modestova AV, Ermakov NV. Epidemiological characteristics of Helicobacter pylori infection in Moscow. Gig Sanit2011;(1):44-8

2. Toljamo K. Gastric Erosions - Clinical Significance and Pathology. A long-term Follow-up Study. Acta Universitatis Ouluensis. D, Medica (1152). University of Oulu, 2012. p. 27.

## A164 The Effect of G2 Vaccine on the Gene Expression NKG2D and Receptor Presenting on the Surface of NK Cells in Peripheral Blood

### Nader Dashti Gerdabi^1,2^, Ali Khodadadi^1^, Zahra Abdoli^1^, Mehri Ghafourian^1^, Mohammad Ali Assarehzadegan^1^, Khodayar Ghorban^2^

#### ^1^School of Medicine, Ahvaz Jundishapur University of Medical Sciences, Ahvaz, Iran; ^2^School of Medicine, Aja University of Medical Sciences, Tehran, Iran

##### **Correspondence:** Nader Dashti Gerdabi – School of Medicine, Ahvaz Jundishapur University of Medical Sciences, Ahvaz, Iran

**Introduction:** Natural killer (NK) cells are a subset of lymphocytes and that have an important role in innate immunity. NK cells release cytokines such as TNF-α IFN-ɣ during infection. These cytokines stimulate and increased activity of the innate and *adaptive* immune responses. NKG2D is one of the most stimulating NK receptors that bind to the MIC-A, MIC-B and ULBPs. These ligands are on the tumor and virus-infected cells,which leads to increasing NK secretion lytic proteins such as perforin, granzyme against target cells, and play an important role in the activation of cellular immune function, as well as destruction and elimination of cancer cells. Previous studies have confirmed G2 Vaccine has an important role in the control of asthma via affecting TH2 cells and controls allergic response through preventing increasing eosinophil, basophil, and inhibits TH2-related responses. Thus, in this study, we evaluated the effect of G2 Vaccine on the gene expression and NKG2D receptor presenting on NK cells in peripheral blood.

**Materials and Methods:** To the obtain Nk cell, Blood mononuclear cells after isolation, *The 1×10*^*6*^*of viable cells were cultured in medium RPMI 1640* and affects by G2 Vaccine in the times of 12, 24 and 48 hours at of 37°. G2 Vaccine is the buffalo spleen extracts that act as TH cells stimulants and first time has been registered by Saleh Mohaghegh Hazrati in IRAN. Then extracting RNA of the cells, cDNA synthesis was performed and gene expression was evaluated by Real-Time PCR. Also receptors presenting on the cell surface was evaluated via monoclonal antibodies by flow cytometry.

**Results:** The results of our study have shown that G2 Vaccine leads to up-regulating gene *expression* and NKG2D receptor presenting on NK cells. This can lead to increasing NK cells cytotoxicity through this receptor.

**Conclusion:** Due to the increasing NKG2D receptor on NK cells,that can increase NK cell cytotoxicity activity against viral infections and cancers Also, this Vaccine regulate the balance betweenTH1and TH2 and can induce the stimulation of Th1cells. Therefore, In the future this Vaccine can be used in the cancer immunotherapy and treatment of allergic diseases.

**Keywords:** natural killer, NKG2D, flow cytometry, qRT- PCR, Immunotheraphy.

References

1. Boskabady MH, Neamati A, Hazrati SM, Khakzad MR, Moosavi SH, Gholamnezhad Z. The preventive effects of natural adjuvants, G2 and G2F on tracheal responsiveness and serum IL-4 and IFN-?(th1/th2 balance) in sensitized guinea pigs. Clinics. 2014;69(7):491-6.

## A165 Ethnic differences in lifetime prevalence and indoor environmental factors for childhood eczema

### Hyo-Bin Kim^1^, Hui Zhou^2^, Jeong Hee Kim^3^, Rima Habre^2^, Theresa Bastain^2^, Frank Gilliland^2^

#### ^1^Sanggye Paik Hospital, Seoul, South Korea; ^2^Keck School of Medicine, Los Angeles, CA, USA; ^3^Inha University Hospital, Incheon, Seoul, South Korea

##### **Correspondence:** Hyo-Bin Kim – Sanggye Paik Hospital, Seoul, South Korea

**Background:** The prevalence of eczema varies markedly across the globe. It is unclear whether the geographic variation is due to race/ethnic differences, environmental exposures or genetic factors. We investigated the effects of ethnicity and environmental exposures on eczema in Hispanic white (HW) and non-Hispanic white (NHW) children participating in the Southern California Children’s Health Study (CHS).

**Methods:** We examined socio-demographic predictors and environmental exposures among HW and NHW children aged 4-8 years enrolled in the CHS, 2002-2003.

**Results:** Eczema prevalence differed by ethnicity: HWs showed lower prevalence (13.8%) compared to NHWs (20.2%) and adjustment for socio-demographic factors did not account for the ethnic difference (odds ratio [OR]=0.79, 95% confidence intervals [CIs]=0.65-0.95). Parental history of allergic disease had a larger effect in HWs than NHWs (*P* for interaction=0.005). High maternal education level (OR=1.46, 95% CI=1.14-1.87), parental history of allergic disease (OR=2.21, 95% CI=1.78-2.76) and maternal smoking during pregnancy (OR=1.44, 95% CI=1.06-1.95) increased the risk of eczema. Indoor environmental factors (e.g., mold, water damage and humidifier use) increased the risk of eczema in NHWs independent to parental history of allergic disease, but in HWs, increased risks were observed primarily in children without parental history of allergic disease.

**Conclusions:** HW children in southern California have a lower prevalence of eczema than NHWs and this ethnic difference is not accounted for by socio-demographic differences. The effects of parental history of allergic disease and indoor environmental exposures on eczema varied by ethnicity suggesting that the etiology of eczema may differ in HWs and NHWs.

## A166 A case of methazolamide-induced toxic epidermal necrolysis

### Jong-Wook Bae^1^, Kyu-Hyung Han^1^, Young-Koo Jee^1^, Misoo Choi^1^, Seung-Phil Hong^1^, Seung-Hyun Kim^2^

#### ^1^Dankook University College of Medicine, Cheonan, South Korea; ^2^Ajou University School of Medicine, Suwon, South Korea

##### **Correspondence:** Jong-Wook Bae – Dankook University College of Medicine, Cheonan, South Korea

Among the various dermatological entities, toxic epidermal necrolysis (TEN) is a rare but potentially fatal delayed hypersensitivity reaction to numerous medications. Methazolamide is one of the rare culprit drug. We here report the patient who presented TEN after taking methazolamide.

A 38 year-old male visited clinic because of systemic hypersensitivity reaction such as high fever, pain in the eyes and diffuse pruritic erythematous maculopapular eruption with multiple targetoid plaques which became vesicular and bullous and involved oral mucosa and conjunctivae. The first sign appeared about 1 week after taking methazolamide (50mg BID) due to glaucomatous eyes. Blood tests on admission showed hemoglobin 16.1 g/dL, total leukocyte count 5265 /uL (neutrophils 78.7%, lymphocytes 11.2%, eosinophils 4.9%), AST 47 IU/L, ALT 49 IU/L, serum bilirubin 0.55 mg/dL, serum creatinine 0.91 mg/dL and blood glucose 95 mg/dL. Urinalysis showed no red blood cells or white blood cells. Although methazolamide was discontinued blistering and skin denuation progressed to affecting up to 80% of the body surface area and a positive Nikolsky sign was noted. High fever was also persisted.

He was managed with fluid supplements, total parenteral nutrition, daily dressing of the involved body surface. IVIG was administered as 0.6g/kg/day for 3 days in addition to methylprednisolone, acetylcysteine and moxifloxacin. Skin lesions started to improve after 2 weeks of management and fever was subsided. Cutaneous lesions were improved with minimal permanent sequele in 2 months later. HLA-B*5901 was found by high-resolution genotyping.

Strong genetic association between HLA B*5901 and methazolamide-induced SJS/TEN has been suggested in Koreans. Screening for HLA-B*5901 may be useful for avoiding the methazolamide-induced SJS/TEN. Therefore, methazolamide should not be prescribed for HLA-B*5901 positive patients.

## A167 Inflammatory responses of human adipose-tissue derived stem cells to LPS and nanoparticles

### Hee-Kyoo Kim^1^, Gil-Soon Choi^2^, Jeonghoon Heo^3^, Young-Ho Kim^3^, Eun-Kee Park^2^

#### ^1^Kosin University Gospel Hospital, Busan, South Korea; ^2^College of Medicine, Kosin University, Busan, South Korea; ^3^College of Medicine, Kosin Univ., Busan, South Korea

##### **Correspondence:** Hee-Kyoo Kim – Kosin University Gospel Hospital, Busan, South Korea

**Background:** Human adipose tissue-derived stem cells (hADSCs) have various influences on many types of cells through production and secretion of several cytokines. Some studies presents they have some abilities to suppress or modulate inflammatory responses. By the way, they can show various inflammatory behaviors when meet certain materials or environment. Thus, we investigated the effects of LPS and titanium dioxide (TiO2) nanoparticle (P25) on production of the inflammatory cytokines from hADSCs and A549.

**Methods:** Human adipose tissue-derived stem cells (hADSCs) were cultured in DMEM/F12 medium containing 10% FBS, 10 ng/ml EGF and 2 ng/ml bFGF. A549 cells were cultured in RPMI1640 containing 10% FBS, Cells were treated for 4 hr with LPS (100 ng/ml) and P25 TiO2 nanoparticles (10 ug/ml, 50 ug/ml, 250 ug/ml) and medium as control. Cell viability was determined by MTT assay. The expression levels of IL-1a, IL-8, TNFa, and IL-10 mRNAs were determined by real-time PCR.

**Results:** The viability of hADSCs and A549 cells were not affected by the treatment of LPS and P25 TiO2. LPS increased the expression of IL-8 and TNFa mRNAs, but decreased the expression of IL-10 in hADSCs. P25 TiO2 increased the expression of IL-1a and IL-8 mRNAs, but decreased the expression of IL-10 in hADSCs. In A549 cells, LPS and P25 TiO2 decreased the expression of IL-1a and IL-10 mRNAs, but did not affect the expression of IL-8 and TNFa mRNAs.

**Conclusion:** This study shows the different inflammatory responses of hADSCs and A549 cells to different stimulating materials. The response of hADSCs to inflammatory agents are more dynamics than A549 cells, which suggests that hADSC may facilitate some manipulation in certain inflammatory situation, such as chronic airway disease.

## A168 Analysis of 71 Cashew Nut Oral Challenge Tests

### Takashi Inoue, Kiyotake Ogura, Noriyuki Yanagida, Hirotoshi Unno, Kenichi Nagakura, Tetsuharu Manabe, Tomoyuki Asaumi, Sakura Sato, Yu Okada, Motohiro Ebisawa

#### Sagamihara National Hospital, Japan

##### **Correspondence:** Takashi Inoue – Sagamihara National Hospital, Japan

**A) Objective**

The purpose of the study is to analyze the risk factors of a positive oral food challenge (OFC) to cashew nut (CN) performed at Sagamihara National Hospital.

**B) Methods**

Subjects were 71 patients who had received an OFC of more than 1g of CN for the purpose of diagnosis or confirmation of tolerance acquisition from June, 2006 to August, 2014. We had measured those patients’ CN specific IgE within 1 year of OFC. We retrospectively analyzed OFCs and the patients’ background. When clear objective symptoms were seen, OFC was judged as positive. When symptoms were unclear at OFC, we concluded the final diagnosis by confirming the reproducibility of the symptoms at home until their next visit at outpatient.

**C) Results**

Of the 71 patients 50 were male, 21 were female. Median age was 7.5 years with a range from 3.3 to 22.5 years. The reason for elimination of CN in 8 patients (including 2 with anaphylaxis) was a history of immediate reaction to CN. Fifty seven patients had eliminated CN due to a positive CN specific IgE. Atopic dermatitis was seen in 76% (54/71), asthma 45% (32/71), allergic rhinitis 36% (27/71), allergic conjunctivitis 17% (13/71). History of immediate reaction to nuts other than CN was seen in 23% (16/71) of the patients and history of immediate reaction to peanut in 31% (22/71). Eighteen % (13/71) of the patients were positive in OFC and 82% (58/71) negative. Of the 13 patients with a positive OFC, anaphylaxis was seen in 5 patients. Oral mucosal symptoms were seen in 9 cases, gastrointestinal symptoms in 9 cases, cutaneous symptoms in 7 cases, respiratory symptoms in 6 cases, neurologic symptoms in 3 cases and cardiovascular symptoms in 1 case. Seven patients were treated with antihistamine, 5 patients with steroids, 4 patients with inhaled β2 stimulant and 1 patient with adrenaline. In the comparison of OFC between positive and negative patients, a significant difference (p<0.01) was seen in a history of immediate reaction to CN and CN specific IgE. There was no significant difference in other factors including sex, age, history of immediate reaction to nuts other than CN, immediate reaction to peanut and anaphylaxis due to CN.

**D) Conclusions**

A history of immediate reaction to CN and high CN specific IgE were risk factors for a positive OFC. CN OFCs in patients with these risk factors should be performed with caution, considering the possibility of anaphylaxis.

## A169 Fungal sensitization is associated with asthma exacerbation

### Min-Gu Kim^1^, You Sook Cho^1^, Tae-Bum Kim^1^, Hee-Bom Moon^1^, Jung-Hyun Kim^1^, Hyo-Jung Kim^1^, So-Young Park^1^, Bomi Seo^1^, Hyouk-Soo Kwon^1^, Jaemoon Lee^2^, Taehoon Lee^3^

#### ^1^Asan Medical Center, University of Ulsan College of Medicine, South Korea; ^2^Asan Medical Center; ^3^Ulsan University Hospital

##### **Correspondence:** Min-Gu Kim – Asan Medical Center, University of Ulsan College of Medicine, South Korea

Fungal sensitization is not associated with asthma severity: data from a single tertiary hospital in Korea

**Background:** Despite the implementation of guideline-based asthma treatment, 5-10% of asthma population still suffer from severe asthma. In a recent study, fungal allergy was recognized as an important risk factor for severe asthma.We aimed to analyze the prevalence of fungal sensitization in a retrospective cohort of asthma patients, and evaluate differences in clinical characteristics by the presence of fungal sensitization.

**Materials and Methods:** We reviewed medical records of 689 asthma patients who visited a tertiary referral hospital from May 2005 to Jul 2012 and performed skin prick test and pulmonary function test. Cross-sectional data (serum total IgE, blood eosinophil, FEV1, bronchodilator response, PC20, asthma severity), and longitudinal data (number of exacerbations, mean ICS dose, FEV1 variability) were compared by the presence of fungal sensitization, defined as positive (A/H > 1 or mean wheal diameter greater than 3mm) skin prick test to fungal allergen (*Aspergillus, Alternaria, Cladosporium, Epicoccum, Fusarium, Penicilliium, Trichopyton*). Furthermore, we compared these variables according to the degree of sensitization (negative, A/H ratio > 0.5, A/H ratio > 1). FEV1 and ACT variability was calculated as the average of absolute value of subtraction (FEV1, ACT values which were measured every 3 months), and severe asthma was defined as pre-bronchodilator FEV1 lower than 60%.

**Results:** The proportion of severe asthma among asthma patients with fungal sensization was approximately 7% (11/74) and 11% (133/634) among those without fungal sensization and the difference was not statistically significant. Serum total IgE was increased with higher degree of fungal sensitization (p=0.001), however, FEV1, PC20, bronchodilator response, blood eosinophil, number of exacerbation, FEV1 variability, mean ICS dose were similar across the three groups.

**Conclusions:** Fungal sensitization did not correlate with asthma severity and the degree of fungal sensitization was not associated with any other clinical variable than total IgE. Additional studies with larger number of patients are required for better understanding of the role of fungal sensitization in asthma.

## A170 Individual therapeutic patient education and consultation in children with atopic dermatitis

### Hye-Soo Yoo^1,2^, Jieun Kim^1^, Inok Kim^1^, Haejin Kim^1^, Younhee Chang^1^, Hae-Sim Park^2^, Sooyoung Lee^2^, Sooyoung Lee^1^

#### ^1^Suwon Center for Environmental Disease and Atopy; ^2^Ajou University School of Medicine, South Korea

##### **Correspondence:** Hye-Soo Yoo – Suwon Center for Environmental Disease and Atopy

**Introduction**: Atopic dermatitis (AD) is the most common chronic inflammatory skin disease with a prevalence of up to 20% in children. Children with AD and their parents face difficulties related to daily care and management during a relapsing course that may be aggravated by multiple triggers. Recently therapeutic patient education (TPE) and individual consultation have been in use in the treatment of many chronic diseases for optimal management. Therefore, we preliminarily evaluated the necessities of TPE and individual consultation program of therapeutic management in AD. **Subjects and Methods**: We organized an individually tailored TPE and consultation program provided by multispecialty of allergist, nurse, nutritionist and environmental coordinator. The program is a patient-centered process consisting of informative consultation which addresses the patient’s specific problems including psychosocial support. Enough time was allowed for parents to ask questions during the program. We conducted a questionnaire survey on the disease knowledge before and after the program at the center initially visited. After 3.5 months (min-max: 1-6), participants were contacted by nurses for a telephone interview (TI) with a structured questionnaire (efficiency of the TPE program, symptoms, and quality of life (QOL)). The severity of AD was assessed by allergist using SCORing Atopic Dermatitis (SCORAD). **Results**: Parents of 135 children with AD took a TPE program and completed the questionnaire survey between May and November 2014. Children with AD were most commonly in the age group of 1~3 years (48, 36%), followed by < 1 age group (36, 27%). The mean SCORAD index of patients was 30.8 (0.0-78.0). The percentages of children with mild, moderate and severe AD were 24, 46, and 30 %, respectively. There was significant improvement in disease awareness by 17.2 from 78.9 to 96.1. Among 135 parents, 100 responded to the questionnaire on the TI. At follow-up TI, 92% answered the TPE program was useful in managing problems related with AD and 86% experienced improvement of the AD symptoms. Furthermore, there was positive change of the QOL in 85% parents. **Conclusion**: The results of this study demonstrate the usefulness and need of TPE and individual consultation to enable children and their families to care themselves in daily conditions and prevent avoidable complications while improving QOL, ultimately for optimal therapy of AD.

## A171 Utility of Alpha-Lactalbumin Specific IgE Levels Using Immulite 2000 3gAllergy in Predicting Clinical Severity of Milk Allergy

### Kazuyo Kuzume, Munemitsu Koizumi, Koji Nishimura, Michiko Okamoto

#### Ehime Graduated School of Medicine, Japan

##### **Correspondence:** Kazuyo Kuzume – Ehime Graduated School of Medicine, Japan

**Background:** IMMULITE 2000 3gAllergy (3gAllergy) is a new method to measure serum antigen-specific IgE levels. Alpha-lactalbumin is one of milk components but its utility in milk allergy has been rarely reported.

**Objectives:** The purpose of this study was to evaluate the utility of alpha-lactalbumin (ALA) specific IgE levels using 3gallergy in predicting clinical tolerance in patients with milk allergy.

**Method:** Forty-three (43) patients with milk allergy (30 boys and 13 girls, age 12 months to 13 years, median 4 years old) were examined for ALA specific IgE levels using 3gAllergy and ImmunoCAPspecific IgE assay (ImmunoCAP). In addition, specific IgE for cow’s milk, casein, and beta-lactoglobulin (BLG) were measured by 3gAllergy and ImmunoCAP. Subjects were categorized into 3 groups according to how much heated milk the patient could tolerate, group A, less than 10ml (n=14), group B, 10ml to 100ml (n=15), and group C, 100ml or more (n=14). Two patients had avoided cow’s milk product strictly because of severe anaphylactic history with milk intake and others had been undertaking oral immunotherapy with heated milk. Since all data were not assumed Gaussian distributions, they were shown as (median, range(minimum - maximum), and were analyzed by Spearman’s method or one-way ANOVA with Dunn’s multiple comparisons.

**Results:** The levels of cow’s milk specific IgE using 3gAllergy were significantly correlated to the levels using ImmunoCAP (r=0.958, p< 0.0001). Likewise, the casein, BLG, and ALA specific IgE levels showed similar correlations: (r=0.9766, p< 0.0001, r=0.9793, p< 0.0001, and r=0.821, p<0.0001, respectively). Month of age and serum total IgE levels were not significantly different among the groups.

The ALA specific IgE levels with 3gAllergy in group A (44.6 UA/ml, (2.92-478)) and in group B (8.43 UA/ml, (1.09-152) were significantly higher than those in group C (0.994 UA/ml, (<0.1-53.1), p< 0.0001 and p<0.05). but there was no significant difference between levels in group A and in group B. Levels of milk, casein, and BLG showed similar results. However, the ALA specific IgE - total IgE ratios in group A (0.05, (0.0132-0.1146) were significantly higher than those in group B (0.0155 (0.0024-0.0691)) and those in group C (0.0034 (0.0007-0.0234), p< 0.0001 and p<0.05), as well as the ratios in group B were significantly higher than those in group C, (p< 0.05). On the other hand, there were no significant differences of ALA data using ImmunoCAP among the groups.

**Conclusions:** The ALA specific IgE levels using 3gAllergy is useful to evaluate the clinical tolerance of milk allergy, especially when considered influence of total IgE levels.

## A172 Isoniazid/rifampicin-specific t-cell responses in patients with anti-tuberculosis –induced dress syndrome

### Seung-Hyun Kim^1^, Young Min Ye^1^, Gyu Young Hur^2^, Hae-Sim Park^1^, Sang-Heon Kim^3^, Young-Koo Jee^4^

#### ^1^Ajou University School of Medicine, South Korea; ^2^Korea University College of Medicine; ^3^Hanyang University College of Medicine; ^4^Dankook University College of Medicine

##### **Correspondence:** Seung-Hyun Kim – Ajou University School of Medicine, South Korea

**Background:** Anti-tuberculosis drugs (ATDs) which are a combination of isoniazid, rifampicin, pyrazinamide and ethambutol are commonly used for the treatment of tuberculosis, but occasionally associated with drug- hypersensitive immune reactions such as drug rash with eosinophilia and systemic symptoms (DRESS) syndrome and hepatitis. The culprit drug and mechanistic basis of the hypersensitive reaction has not been defined. Objectives: The aim of this study was to find whether drug-responsive T cell response was detectable in patients with ATD-related DRESS and characterize the mechanistic features of the T-cell response. **Methods:** A lymphocyte transformation test (LTT) and IFNγ-ELISpot assay using ATDs were performed using peripheral blood mononuclear cells from the patient. Subsequently, drug-specific T-cell clones were generated by serial dilutions. **Results:** High proliferative responses to isoniazid or rifampicin were detectable in the patient with DRESS by LTT. Isoniazid/rifampicin- specific T-cell clones were generated from blood of the patients, but not pyrazinamide or ethambutol. The T cell clones proliferated and secreted IFNγ when stimulated with isoniazid or rifampicin. They did not cross-react with each other. **Conclusion:** These studies identify isoniazid/rifampicin- specific T-cells in peripheral blood of certain patients with ATD-induced DRESS. Further studies are needed to define the mechanisms of drug-responsive T cell activation.

## A173 Genetic biomarkers associated with aspirin-exacerbated respiratory disease (AERD) phenotype based on genome-wide association study

### Seung-Hyun Kim^1^, Hyunna Choi^2^, Young Min Ye^3^, Hae-Sim Park^3^

#### ^1^Ajou University School of Medicine, South Korea; ^2^Ajou University Hospital; ^3^Ajou University School of Medicine

##### **Correspondence:** Seung-Hyun Kim – Ajou University School of Medicine, South Korea

***Background***: Common clinical syndromes of aspirin hypersensitivity, aspirin-exacerbated respiratory disease (AERD) and aspirin-exacerbated cutaneous disease (AECD), was subjected to a genome-wide association study to identify differential genetic biomarkers for aspirin hypersensitivity in a Korean population. ***Methods***: A comparison of SNP genotype frequencies on an Affymetrix Genome-Wide Human SNP array of 179 AERD patients, 211 AECD patients and 1989 healthy normal control subjects (NC) revealed SNPs on chromosome 2 and 6 that were associated with AERD, not AECD. To validate the association, we enrolled a second cohort comprising AERD, normal healthy control and disease-control (aspirin tolerant asthma; ATA) groups. A diagnostic value was evaluated by the area under the curve (AUC) value of receiver operating characteristic (ROC) curves for each combination. ***Results***: Three SNPs of DPP10 gene on chromosome 2 and two SNPs of HLA-DPB1 gene on chromosome 6 showed a significant association with the AERD phenotype. The minor genotype frequency (AG or AA) of a particular SNP, rs3128965, in the HLA-DPB1 region was higher in the AERD group compared to the ATA or NC group (*P*=0.001, *P*=0.002, in a co-dominant analysis model, respectively). Comparison of rs3128965 alleles with the clinical features of asthmatics revealed that the patients harboring the A allele of a particular SNP, rs3128965, in the HLA-DPB1 region showed increased bronchial hyperresponsiveness to inhaled aspirin, methacholine, and higher 15-HETE levels, than those without the A allele. Three intronic SNPs of DPP10 gene are significantly associated with the AERD phenotype. Serum DPP10 level was significantly higher in AERD compared to control groups, ATA and NC. A combination of SNP of DPP10 gene and serum DPP10 level showed a good diagnostic value in a close correlation with c-Kit and YKL. ***Conclusions***: This implies the potential of two SNPs, rs3128965 of HLA-DPB1, rs17048175 of DPP10, as genetic biomarkers for the AERD phenotype.

## A174 Assessment of ORAL drug provocation test in the diagnosis of NON-steroidal ANTI-inflammatory drugs hypersensitivity

### Bui VAN Khanh^1^, Hieu Chi Chu^1^, Nguyen Nhu Nguyet^2^, Nguyen Hoang Phuong^1^

#### ^1^Bach Mai Hospital, Vietnam; ^2^Hanoi Medical University, Vietnam

##### **Correspondence:** Bui VAN Khanh – Bach Mai Hospital, Vietnam

***Background*:** Hypersensitivity to aspirin and other Non-steroidal anti-inflammatory drugs (NSAIDs) is the second most frequent allergic reaction to drug, which include immunological and nonimmunological with airway and/or skin manifestations. Provocation test is the “gold standard” to diagnose NSAIDs hypersensitivity. Although some of the NSAIDs are reported to be safe for NSAID-intolerant patients, they should not be suggested before confirming the safety by single-blind oral drug provocation tests.

***Objective*:** To assess the role of oral drug provocation test in the diagnosis of NSAIDs hypersensitivity and find out the safe alternative drugs.

***Method*:** Provocation tests with four types of NSAIDs (aspirine - A, ibuprofene - I, meloxicam - M, etoricoxib - E) were administered in patients suspected to be intolerant to NSAIDs by specialists in the Centre of Allergy and Clinical Immunology in Bach Mai hospital.

***Results***: A total of 156 patients having history suspected of being allergic to NSAIDs (F/M: 81/75: 51,9%/48,1%, mean age: 34,9 ± 14,2 years) were enrolled between May 2012 and April 2015. 624 oral provocation tests with NSAIDs were performed and 299 of them were positive. 142/156 patients (91%) had positive tests, 24 (16,9%) of them were positive for one drug (A: 11 patients, I: 7 patients, M: 5 patients, E: 1 patients) and 83,1% with two or more drugs. Prevalence of A, I, M and E hypersensitivity were 90,8%, 88%, 9,2% and 0,7%; respectively. The average dose (mg) for positive result of A, I, M, E was 60,9±37,6, 118,4±43,4, 6,9±1,1, 30; respectively. The average response time (minutes) from the last dose of A, I, M, E was 63,4±24,1, 69,3±23,4, 78,1±18,7, 120; respectively. Prevalence of cross-reactivity with A of I, M, E was 91,5%, 6,2%, 0%. The main clinical manifestation as positive test was combined allergic rhinitis and urticaria angioedema (42,5%), only one patient had bronchospasm and no patient had anaphylaxis.

***Conclusion***: This research showed oral provocation test is a safe test and also a “gold standard” for NSAIDs hypersensitivoty diagnosis. Choosing altenative NSAIDs should base on single-blind provocation tests.

## A175 Korean treatment guideline of atopic dermatitis

### Joo Young Roh^1^, Hyun Jeong Kim^2^, Jung Eun Kim^3^, Bark-Lynn Lew^4^, Kyung Ho Lee^5^, Seung-Phil Hong^6^, Yong Hyun Jang^7^, Kui Young Park^8^, Seong Jun Seo^8^, Jung Min Bae^9^, Eung Ho Choi^10^, Ki Beom Suhr^11^, Seung Chul Lee^12^, Hyun-Chang Ko^13^, Young Lip Park^14^, Sang Wook Son^15^, Young Jun Seo^16^, Yang Won Lee^17^, Sang Hyun Cho^18^, Chun Wook Park^19^

#### ^1^Gil Medical Center, South Korea; ^2^Seoul Medical Center; ^3^St. Paul’s Hospital; ^4^Kyung Hee University Hospital at Gang-Dong; ^5^Bucheon St. Mary’s Hospital; ^6^Dankook University College of Medicine; ^7^Kyungpook National University; ^8^Chung-Ang University Hospital; ^9^St. Vincent’s Hospital; ^10^Yonsei University, Wonju College of Medicine; ^11^SA Dermatology Clinic; ^12^Chonnam National University Hospital; ^13^Pusan National University Yangsan Hospital; ^14^Soonchunhyang University Hospital; ^15^Korea University; ^16^Chungnam National University Hospital; ^17^Konkuk University Hospital; ^18^Incheon St.Mary’s Hospital; ^19^Kangnam Sacred Heart Hospital

##### **Correspondence:** Joo Young Roh – Gil Medical Center, South Korea

**A) Background:** Treatment guideline of Korean atopic dermatitis was released by Korean Atopic Dermatitis Association(KADA) work group in 2006. Due to the recent advances in atopic dermatitis (AD) research and controlled clinical studies, establishment of updated evidence-based treatment guideline for Korean atopic dermatitis is demanding.

**B) Methods:** Task force team convened by KADA collected database of references from relevant systematic reviews and guidelines of atopic dermatitis. They raised all the relevant key statements on the management of AD. Evidence of each statement was graded and classified the strength of recommendation for each statement. The evidence of each statement was graded using the classification of the Oxford centre for evidence-based medicine (level 1-level 5). The strength of recommendation was classified as follows;

A, level 1; B, level 2 and 3; C, level 4; D, level 5. Fifty four council members of KADA were asked to vote on the statements of the draft guidelines. Participants indicated their level of agreement with each draft statement using a voting scale of 1–9 (where 1=strong disagreement and 9=strong agreement). Consensus was defined as ≥75% of participants scoring within the 7–9 range (agreement). After three rounds of votes, expert consensus of recommendations were established.

**C) Results:** Updated guideline provides up-to-date evidence based systematic combined treatment algorithm including basic, proactive, induction and maintenance treatment. In addition, average agreement scores of experts panel were presented considering Korean healthcare system and patients’ adherence. The guideline comprises of the topical management of AD via bathing and skin care, avoidance of exacerbating factors, education and psychosocial support, moisturizer and topical anti-inflammatory drugs, antibiotics, and anti-pruritic drugs and the systemic management of AD including antihistamines, antimicrobials, systemic immunomodulators, allergen-specific immunotherapy (ASIT), phototherapy, and complementary and alternative medicines. Clinical questions were focused on the therapeutic effect, action plans in detail, side effects, cost effectiveness, and measures to enhance patients’ compliance of each treatment.

**D) Conclusions:** To achieve high treatment efficacy and patient satisfaction, treatment decisions should be made jointly by the physician and patient. We expect this evidence- and experience-based treatment recommendations of AD experts will be a reference guide for physicians and AD patients choosing the appropriate treatment to improve quality of life and decrease unnecessary social medical costs.

## A176 Systemic side reaction of subcutaneous immunotherapy(SCIT) for perennial allergic rhinitis

### Kun Hee Lee^1^, Sung Wan Kim^2^

#### ^1^Kyung Hee University Hospital at Gangdong, South Korea; ^2^Kyung Hee Medical Center

##### **Correspondence:** Kun Hee Lee – Kyung Hee University Hospital at Gangdong, South Korea

**Background:** Subcutaneous immunotherapy(SCIT) is one of the evidence-based treatment for allergic rhinitis. However, with the advent of sublingual immunotherapy(SLIT) SLIT is more preferred over SCIT because of the proven safety. Although it is reported SCIT showed some systemic reactions which can be fatal in some patients the fatality of SCIT still needs to be elucidated.

**Objectives:** The objective of this study is to categorize the side reaction of SCIT into local or systemic one and identify correlations between antecedent local reaction and systemic reaction and between the dose, concentration of allergen and systemic reaction.

**Methods:** Allergic rhinitis patients under SCIT over age of 4 were included. Retrospectively with the medical chart review we investigated the incidence of side reaction of SCIT, relationship between the doses, concentration of allergen.

**Results:** 130 patients met criteria for inclusion, 80 patients (61.5%) had one or more side reactions. 59 patients (73.8%) had only local reaction, 9 patients (11.3%) had only systemic reaction. 12 patients (15%) had both local and systemic reaction. Among 21 patients who had systemic reaction 12 patients showed no antecedent local reaction. Among systemic reaction urticaria was the most common (57.1%) followed by aggravation of rhinitis symptoms (38.1%) and dyspnea (4.8%). 23.8% of patients showed systemic reaction over 30 minutes after the shot. The more concentration and dose were injected the more systemic reaction occurred.

**Conclusions:** Although systemic side reaction was common severe symptom like dyspnea was very rare. Local side reaction cannot forewarn the systemic reaction. It is advised the caution is needed when administering high dose and concentration.

## A177 Clinical baseline characteristics of Asian patients suffering from refractory chronic spontaneous urticaria (CSU) in three phase 3 omalizumab clinical trials

### Chia-Yu Chu^1^, Derrick Aw^2^, Young-Min Ye^3^, Giovanni Bader^4^, Fabrizio Dolfi^4^, Nathalie Oliveira^4^

#### ^1^National Taiwan University Hospital, Taiwan; ^2^National University Hospital, Singapore; ^3^Ajou University School of Medicine, South Korea; ^4^Novartis Pharma AG, Switzerland

##### **Correspondence:** Chia-Yu Chu – National Taiwan University Hospital, Taiwan

**Rationale:** The clinical profile of Asian patients suffering from Chronic Spontaneous Urticaria (CSU) is relatively unknown. The demographic and baseline characteristics of Asian versus non-Asian patients of three randomized, placebo-controlled omalizumab trials (ASTERIA I, ASTERIA II and GLACIAL) are described and compared.

**Method:** The pooled baseline data of Asian and non-Asian patients suffering from CSU that remained symptomatic despite anti H1-antihistamines (ASTERIA 1 and ASTERIA 2) and anti H2-antihistamines and/or leukotriene receptor antagonists (GLACIAL) treatments were assessed in this post-hoc descriptive analysis. Demographic and baseline disease characteristics are described and compared.

**Results:** Among 975 (mITT population) patients enrolled 32 (3.3%) were Asians. Most of the patients were enrolled in the United States and Europe. There were 22 (68.7%) females in the Asian group and 694 (73.6%) in the non-Asian. Mean age was 41.2 years; mean body weight was 70.3 kg and the mean BMI 26.9 kg/m2 in the Asian subgroup and 42.4 years, 83.2 kg and 29.7 kg/m2, respectively, in the non-Asian subpopulation. Mean duration of the disease was 6.7 years in Asians and 7.0 years in non-Asians. Baseline IgE levels were 163.2 and 169.8 IU/mL in Asians and non-Asians, respectively. Baseline mean urticaria activity score over 7 days (UAS7) was 30.7, weekly itch severity score 13.9, weekly number of hives score 16.8 in Asians and 30.9, 14.1, 16.8, respectively in non-Asians. Angioedema was present in 11 Asian (34.4%) patients and 449 (47.6%) non-Asian. Dermatology Life Quality Index (DLQI) was 11.12 and 13.29 in Asian and non-Asians, respectively. At baseline the Sleep Interference Score was 12.1, and the Daily Activity Interference score was 12.1 in Asians, while in non-Asian, they were and 12.1 and 12.8, respectively. Previous number of CSU medications, such as anti-histamines, LTRA and corticosteroids was 3.8 in Asian and 5.1 in non-Asians.

**Conclusions:** With the exception of body weight and previous history of CSU treatment, the clinical profile of patients with refractory CSU is similar between Asians and non-Asians.

## A178 A metagenomic approach through t-RFLP to the microbiome of asthma

### Jae Chol Choi^1^, Jae Woo Jung^1^, Hye-Ryun Kang^2^, Kijeong Kim^3^, Byoung Whui Choi^4^

#### ^1^Chung-Ang University Hospital, South Korea; ^2^Seoul National University Hospital; ^3^Department of Mircobiology; ^4^Chung Ang University

##### **Correspondence:** Jae Chol Choi – Chung-Ang University Hospital, South Korea

**Background:** Recent metagenomic approaches succeeded in characterizing the microbial communities of airway diseases present in bronchoalveolar lavage and supernatants of induced sputum but those results were limited by small sample size and lack of microbial information from inflammatory cells and epithelial cells of airways. We investigated the use of terminal restriction fragment length polymorphism (T-RFLP) analysis, in conjunction with multivariate statistical methods, to examine the difference of micro biota in whole induced sputum (inflammatory cells and epithelial cells in addition to supernatant) between normal control and asthma.

**Methods:** Induced sputum samples were obtained from 36 normal nonsmoker subjects and 89 patients with steroid naïve asthma. Through T-RFLP analysis, operational taxonomic units (OTUs), representing bacterial species or taxonomically related groups, and relative amounts of each OTUs were evaluated.

**Results:** The global *R* value for differences between normal control and asthma was 0.148 (*P*=0.002). Dissimilarities in bacterial communities of induced sputum between normal control and asthma was 28.74%. OTUs contributing to normal control-asthma differences were identified. OTU 789 (Lachnospiraceae, 3.1 fold), 517 (Comamonadaceae, Acetobacteraceae, Chloroplast, 1.9 fold), 633 (Prevotella, 1.9 fold), 645 (Actinobacteria, Propionibacterium acnes, 1.8 fold), 607 (Lactobacillus buchneri, Lactobacillus otakiensis, Lactobacillus sunkii, Rhodobacteraceae, 1.8 fold), 661 (Acinetobacter, Pseudomonas, Leptotrichiaceae, 1.8 fold), 650 (Brevibacteriaceae, Lachnospiraceae, Fusobacterium, 1.5 fold), 821 (Lachnospiraceae, Acinetobacter, Actinomyces, 1.5 fold), 415 (Comamonadaceae, Streptomycetaceae, 1.5 fold) were significantly more prevalent in the sputum of asthma patients than normal control.

**Conclusion:** Patients with asthma have an altered microbial composition in the respiratory tract.

## A179 Clinical characteristics and ten-year trend of peripheral blood eosinophilia among health screening program recipients at a tertiary hospital of South Korea

### Jong Wook Shin^1^, Jae Woo Jung^1^, Jae Chol Choi^1^, In Won Park^1^, Byoung Whui Choi^2^, Jae Yeol Kim^1^

#### ^1^Chung-Ang University Hospital, South Korea; ^2^Chung Ang University

##### **Correspondence:** Jong Wook Shin – Chung-Ang University Hospital, South Korea

**Background:** Eosinophilia in specific conditions such as allergic diseases, helminthic infection, and drug-induced reaction is well recognized. In the present study, we evaluated the clinical characteristics of eosinophilia and change in prevalence over 10 years in recipients of health screening program at a tertiary hospital of South Korea.

**Methods:** We collected the data of health screening program recipients at the health promotion center of Chung-Ang University Hospital from 2004 to 2013. Eosinophilia was defined when the absolute eosinophil count exceeds 500/μL in the peripheral blood. We reviewed health-related questionnaires and laboratory findings of health screening program which might be related to eosinophilia.

**Results:** The cumulative prevalence of eosinophilia was 4.0% (1,963 out of 48,858). Most of eosinophilia cases (96.6%) were in mild degree (500 to 1500/μL). Eosinophilic subjects were older and more male-predominant (*P*<0.001, respectively). Subjects with eosinophilia showed lower level of FEV1%, FVC% and FEV1/FVC than those without eosinophilia (*P*<0.001, respectively). Of note, annual prevalence was in decreasing trend from 2004 to 2013 (OR=0.942, 95% CI: 0.926-0.958, *P*<0.001). Eosinophilic subjects showed higher positive rate for common parasite ELISA (*P*<0.001). The positive rate for ELISA to parasites increased with advancing age in subjects with eosinophilia (*P*=0.002).

**Conclusions:** Eosinophilia in general, healthy population was not uncommon and usually in mild degree. The prevalence of eosinophilia decreased from 2004 to 2013, which might be related to decrease in parasitic infection in young Korean population.

## A180 The prevalence of toxocariasis and diagnostic value of serologic tests in asymptomatic Korean adults

### Jin-Young Lee^1^, Kyoung Won Ha^2^, Yun-Jin Jeung^3^, Sehyo Yune^4^, Byung-Jae Lee^4^, Dong-Chull Choi^5^, Mi-Jung Oh^6^, Young Hee Lim^7^

#### ^1^Health Promotion Center, Samsung Medical Center, South Korea; ^2^Seoul Samsung Medical Clinic; ^3^Clean Medicine Allergy Clinic; ^4^Samsung Medical Center; ^5^Samsung Medical Center, Sungkyunkwan University School of Medicine; ^6^Bundang Jesaeng Hospital; ^7^Ansan Sarang General Hospital

##### **Correspondence:** Jin-Young Lee – Health Promotion Center, Samsung Medical Center, South Korea

**Purpose:** Toxocariasis is the most common cause of peripheral blood eosinophilia in Korea and produces eosinophilic infiltration into various organs, including the lung. However, the prevalence of toxocariasis in the general population is rarely reported. **Methods**: We investigated the seroprevalence of *Toxocara* larval antibody among asymptomatic people who attended Samsung Medical Center for a health checkup, including low-dose chest computed tomography (CT) between March 2012 and December 2013. A total of 633 people (400 men and 233 women) were recruited. **Results:** The *Toxocara*-seropositive rate was 51.2% using the current cutoff value based on *Toxocara* enzyme-linked immunosorbent assay (ELISA) (67.0% for men and 24.0% for women). In the multivariate-adjusted model, age (odds ratio [OR], 1.08; 95% confidence intervals [CI], 1.05-1.11), male sex (OR, 3.47; 95% CI, 2.26-5.33), rural residence (OR, 1.55; 95% CI, 1.05-2.30), and history of raw liver intake (OR, 8.52; 95% CI, 3.62-20.11) were significantly associated with *Toxocara* seropositivity. When subjects were divided into 3 groups using cutoff values base on weak positive and strong positive control optical densities (ODs), the ORs for peripheral blood eosinophilia and serum hyperIgEaemia were 0.31 (95% CI, 0.02-2.89) in the weakpositive group and 36.64 (95% CI, 11.73-111.42) in the strong positive group compared to the seronegative group. Similarly, ORs for the solid nodule with surrounding halo were 2.54 (95% CI, 0.60-10.84) in the weak positive group and 15.08 (95 CI 4.09-55.56) in the strong positive group compared to the seronegative group. **Conclusions:** The study indicated that the *Toxocara*-seropositive rate obtained by using the current cutoff value based on ELISA was high in the asymptomatic population in Korea. The results of this study suggest that active toxocariasis may be more frequently seen in the *Toxocara*-strong positive group than in the *Toxocara-*weak positive group.

## A181 Cutaneous Drug Hypersensitivity Reaction in Korean Children: An Analysis of KAERS Database on 2012-2013

### Eui Jun Lee^1^, Dongin Suh^1^, Dongin Suh^2^, Sung-Il Woo^3^, Sung-Il Woo^2^, Hwa Jin Cho^4^, Hwa Jin Cho^2^, Eun Hee Chung^5^, Eun Hee Chung^2^, Soo Youn Chung^6^

#### ^1^Seoul National University Hospital; ^2^Pediatic SIG, Korea Institute of Drug Safety and Risk Management; ^3^Chungbuk National University Hispital; ^4^Chonnam National University Hospital; ^5^National Medical Center; ^6^Korea Institute of Drug Safety and Risk Management

##### **Correspondence:** Eui Jun Lee – Seoul National University Hospital, South Korea

**Background**

Since a large proportion of adverse drug reaction (ADR) affects the skin, investigations of cutaneous drug hypersensitivity reaction (DHR) are important to evaluate their impact in dermatology and health care in generals as well as their burden for affected patients. However, little is known about the characteristics of drug-induced cutaneous reactions in Korean children.

**Methods**

We analyzed Individual Case Safety Reports (ICSRs) of cutaneous adverse reactions kept in the Korea Adverse Event Reporting System (KARES). From January 2012 to December 2013, cases of cutaneous DHR were selected and analyzed regarding the age, gender, causative agents, and fatal cases. Based on the WHO-UMC causality assessment system, cases that assessed as ‘unlikely’, ‘unclassified’ or ‘unassessable’ were excluded.

**Results**

A total of 2,577 cases were identified. 1406 cases were male (54.6%), and mean age was 5.97 ± 6.48 years. The most common agent was vancomycin (6.1%), followed by amoxicillin (4.0%), ampicillin (4.0%). The most common adverse reaction was rash (29.2%), followed by urticaria (20.8%), itching (14.0%). Among the ‘certain’ cases by WHO-UMC causality assessment system, the most common agent was acetylsalicylic acid (13.5%), followed by paracetamol (6.7%), iopromide (5.6%).

There were 55 cases of serious ADR. Among the serious ADRs, Acute generalized exanthematous pustulosis (AGEP) was most common (37 cases), followed by Steven-Johnson Syndrome (SJS, 14 cases), Toxic Epidermal Necrolysis (TEN, 2 cases), and Drug reaction with Eosinophilia and Systemic Symptoms (DRESS, 2 cases). Paracetamol was most common causes of serious ADRs (5 cases).

**Conclusions**

The brief analysis of ICSRs-KARES ADR report reveals that the antibiotics and commonly used antipyretics were most common causative agent of cutaneous reaction. More active reporting about cutaneous DHRs should be encouraged to indicate unlabeled ADRs.

## A182 Comparison of clinical characteristics, quality of life and sleep in patients with allergic rhinitis when categorised as "sneezers and runners" and "blockers"

### Ashok Shah^1^, Kamal Gera^2^

#### ^1^Respiratory Medicine; ^2^Vallabhbhai Patel Chest Institute, University of Delhi, India

##### **Correspondence:** Kamal Gera – Vallabhbhai Patel Chest Institute, University of Delhi, India

**Background:** Patients with allergic rhinitis (AR), as per their predominant symptoms, can be classified into “sneezers and runners (SR)” and “blockers”. [*Ann Allergy Asthma Immunol.* 2005;94:60-4.] Since these two groups have distinct profiles, they were assessed and compared in terms of quality of life (QoL) and sleep disturbances.

**Methods:** The study comprising 106 consecutive patients (males:60/females:46), 18 to 60 years with AR, diagnosed as per ARIA guidelines, were enrolled from outpatients department of VP Chest Institute, University of Delhi. Patients were categorised into “SR” (group1) and “blockers” (group2) with the help of a visual analog scale (VAS) of 10 centimetres with 0 being “no symptoms” and 10 being “symptoms extremely bothersome”. Scores for global VAS, sneezing, runny nose, nasal congestion, post nasal drip and loss of smell were recorded and patients classified as “SR” and “blockers”. Impact on QoL was assessed with Sinonasal Outcome Test 22 (SNOT-22) and sleep was assessed by Nocturnal Rhinoconjunctivitis Quality of Life Questionnaire (NRQLQ), the Epworth Sleepiness Scale (ESS) and the Pittsburgh Sleep Quality Index (PSQI) instruments.

**Results:** Just over two thirds (n=73:68.9%) of the patients, were categorised as “SR”(group1) while remaining third (n=33:31.1%) were categorised as “blockers”(group2). The significant features in group1 were: age at onset lower than 20 years (n=60:82.2% P=0.002); birth dates were between June and September (n=42:57.5% P=0.003); family history of atopy (n=59:80.8% P=0.001); itching of skin (n=25:34.2% P=0.002), eye (n=30:41.1% P=0.001), ears (n=28:38.4% P=0.002), and throat and palate (n=42:57.5% P=0.001); and aggravation with dust (n=65:89.1% P=0.001). History of breathlessness (n=28:84.8% P=0.002), mouth breathing (n=28:84.8% P=0.003), loss of smell (n=13:39.4% P=0.004), and prior nasal surgery (n=8:24.2% P=0.001) were significantly higher in group2. Patients in group1 were significantly more sensitised to seasonal allergens like pollens [Kigelia (P=0.045); Salvador (P=0.005)] while patients in group2 had more sensitisation to perennial allergens like house dust (P=0.001), house dust mite (P=0.044) and fungus including Aspergillus species (P=0.001). Mean SNOT-22 scores (group1:60.89;group2:61.66 P=0.763) and mean NRQLQ scores (group1:47.66;group2:50.91 P=0.238) were not significantly different between the groups, while mean ESS (group1:10.52;group2:12.30 P<0.001) and mean global PSQI scores (group1:9.71;group2:11.27 P=0.009) were significantly higher in group2.

**Conclusions:** The two groups differ significantly in terms of their respective profiles, whether be demographic or clinical. Further, the sensitivity patterns to allergens differ significantly between the groups. “Blockers” experienced significantly more sleep disturbances as compared to “sneezers and runners”.

## A183 Role of s-nitrosoglutathione reductase (GSNOR) in the murine strain differences of airway hyperresponsiveness

### Kyoung Won Ha^1^, Mi-Jung Oh^2^, Young Hee Lim^3^, Sehyo Yune^4^, Jae-Won Paeng^5^, Mi-Jin Jang^5^, Byung-Jae Lee^4^, Dong-Chull Choi^6^, Jin-Young Lee^7^

#### ^1^Seoul Samsung Medical Clinic; ^2^Bundang Jesaeng Hospital; ^3^Ansan Sarang General Hospital; ^4^Samsung Medical Center; ^5^Samsung Biomedical Research Institute; ^6^Samsung Medical Center, Sungkyunkwan University School of Medicine; ^7^Health Promotion Center, Samsung Medical Center, South Korea

##### **Correspondence:** Jin-Young Lee – Health Promotion Center, Samsung Medical Center, South Korea

**Background**: S-nitrosothiols (SNOs) are potent endogenous bronchodilators. Genetic ablation of S-nitrosoglutathione reductase (GSNOR), which catalyzes the metabolism of SNOs, results in protection from airway hyperresponsiveness (AHR) in mouse. This study was performed to know whether activity of GSNOR of mice may explain the strain differences in AHR.

**Methods**: Three different strains of mice - A/J, BALB/c, and C57BL/6 - were untreated (control, each n=5) or were sensitized and challenged with ovalbumin (asthma model, each n=5). AHR was measured using methacholine and activity of GSNOR was measured in whole lung lysate.

**Results:** Persistent increase of AHR was observed in A/J compared to BALB/c or C57BL/6 control mice. GSNOR activity was significantly higher in A/J than BALB/c or C57BL/6 control mice (596.8±217.5, 400.0±184.8, 263.1±121.7; au, nmol/min/mg, p <0.05). In asthma model, AHR were also increased in AJ mice compared to other strains, and GSNOR activity was higher than BALB/c or C57BL/6 asthma model mice (749.7±192.5, 466.3±218.1, 474.4±24.0; au, nmol/min/mg).

**Conclusion**: GSNOR activity may contribute to the difference of AHR between mouse strains.

## A184 Protection from airway bronchoconstriction by gsno

### Mi-Jin Jang^1^, Jae-Won Paeng^1^, Yun-Jin Jeung^2^, Young Hee Lim^3^, Mi-Jung Oh^4^, Kyoung Won Ha^5^, Byung-Jae Lee^6^, Dong-Chull Choi^7^, Sehyo Yune^6^, Jin-Young Lee^8^

#### ^1^Samsung Biomedical Research Institute; ^2^Clean Medicine Allergy Clinic; ^3^Sarang General Hospital; ^4^Bundang Jesaeng Hospital; ^5^Seoul Samsung Medical Clinic; ^6^Samsung Medical Center; ^7^Samsung Medical Center, Sungkyunkwan University School of Medicine; ^8^Health Promotion Center, Samsung Medical Center, South Korea

##### **Correspondence:** Jin-Young Lee – Health Promotion Center, Samsung Medical Center, South Korea

**Background:** S-nitrosoglutathione reductase (GSNOR) is an important regulator for S-nitrosoglutathione (GSNO), the main source of bioavailable NO, and protects cells from nitrosative stress. We hypothesized that aerosol delivery of GSNO could reduce bronchoconstriction and pulmonary inflammation in a mouse model of asthma.

**Methods:** To evaluate whether GSNO could ameliorate AHR and inflammation, we compared AHR and inflammation in the 6-week-old female BALB/c mice treated with 0.3 cc aerosolized 0-10 mM GSNO.

**Results:** GSNO inhalation significantly decreased airway hyperresponsiveness with the increasing GSNO dose up to 0.03 ng/ml. But increasing the dose of GSNO more over than 0.03 ng/ml, there was absence of bronchoprotection or even aggravation of bronchocontriction.

**Conclusions:** These results suggested that GSNO may be an important factor to control airway hyperresponiveness, and there might be a concentration threshold for an effective action of GSNO.

## A185 Does EIA-targeted asthma treatment improve daily physical activity of children?

### Takahiro Ito

#### Allergy Center and Department of Clinical Research, Mie National Hospital, Japan

**Objectives**: Exercise-induced asthma (EIA) is a significant burden for children with bronchial asthma because EIA often interfere with exercise activities of children if it is not properly treated and children with uncontrolled EIA may fall into inactive, sedentary life. We hypothesized that daily activity of children with EIA can be improved by controlling EIA. To test the hypothesis, we evaluated physical activity in children with EIA before and after EIA-targeted therapy. “Achieve Active life by Controlling EIA for asthmatic Kids (ACE kids) study”.

**Methods**: We enrolled 7-10 years old boys with untreated EIA. The patients were eligible if they had current history of exercise-induced symptoms, >12%reduction in FEV1 by treadmill exercise challenge at entry and had no anti-inflammatory treatment. Physical activity of the subjects were monitored using an accelerometer, Lifecoder EX®(Suzuken), during 2 weeks of run-in period and 8 weeks of treatment with salmeterol/fluticasone combination (SFC). Primary outcome was the length of strenuous activity in a day and secondary outcomes were %fall in FEV1 after exercise challenge, peak flow and exhaled NO.

**Results**: Eight patients (mean 9.5 ± 0.6 years) were enrolled and 6 gave full dataset. The length of strenuous activity a day increased in 5 patients out of 6. Fall in FEV1 after exercise challenge was significantly smaller after treatment. Morning and evening peak flow significantly increased and exhaled NO significantly decreased after SFC.

**Conclusions**: EIA-targeted treatment for boys with EIA improved their physical activity and measurement of physical activity with an accelerometer may be a surrogate marker for asthma control in children.

## A186 Wheezing as a clue to the diagnosis of cough variant asthma and nonasthmatic eosinophilic bronchitis

### Jin-Young Lee^1^, Sehyo Yune^2^, Byung-Jae Lee^3^, Dong-Chull Choi^3^, Mi-Jin Jang^4^, Jae-Won Paeng^4^, Young Eun Kim^2^, Young Nam Kim^2^, Yongseok Lee^2^, Jihye Kim^2^

#### ^1^Health Promotion Center, Samsung Medical Center; ^2^Samsung Medical Center, South Korea; ^3^Samsung Medical Center, Sungkyunkwan University School of Medicine; ^4^Samsung Biomedical Research Institute

##### **Correspondence:** Jihye Kim – Samsung Medical Center, South Korea

**Background:** Clinical reports on cough variant asthma (CVA) or nonasthmatic eosinophilic bronchitis (NAEB) rarely suggest any symptom as an important diagnostic clue. The aim of this study was to investigate whether history of wheezing is useful in detection of CVA or NAEB among patients with chronic cough.

**Methods:** Patients with cough persisting for more than 8 weeks were prospectively enrolled. They were divided into two groups according to the presence of wheezing in the past. Results of methacholine bronchial challenge and induced sputum examination were compared between two groups.

**Results:** Four hundred sixty patients (165 men and 295 women) were enrolled and analyzed. Their mean age was 47.4 years and the median duration of cough was 13.5 months. Patients who had experienced wheezing were 164 (35.6%). The prevalence of CVA (36.4% vs. 5.8%, p<0.01) and NAEB (15.4% vs. 4.4%, p<0.01) were significantly higher in wheezing group compared to non-wheezing group.

**Conclusion:** History of wheezing in the past significantly increases the chance of CVA or NAEB in patients with chronic cough. Careful history taking is important in diagnosing the cause of chronic cough.

## A187 Antagonism of microRNA-21 suppressed the airway inflammation in a mouse model of bronchial asthma

### Hwa Young Lee^1^, Sook Young Lee^1^, Soon Seog Kwon^2^, Young Kyoon Kim^1^, Chin Kook Rhee^1^, Sei Won Kim^1^, Hea Yon Lee^3^, Joon Young Choi^1^, In Kyoung Kim^1^

#### ^1^Seoul St. Mary’s Hospital, South Korea; ^2^Pucheon St.Mary’s Hospital; ^3^St. Mary’s Hospital

##### **Correspondence:** Hwa Young Lee – Seoul St. Mary’s Hospital, South Korea

**Background:** In previous reports, microRNA-21 (miR-21) was upregulated in allergic airway inflammation mediating Th2 immune response [1,2]. This study was designed to investigate the effect of miR-21 antagonism on mouse model of acute bronchial asthma.

**Methods:** BALB/c mice were sensitized and challenged with ovalbumin (OVA). Anti-miR-21 antagomir and control scrambled RNA was daily injected by intranasal inhalation from the day of sensitization 5 times a week. Changes of cell counts, Th2 cytokines in bronchoalveolar (BAL) fluid and airway hyperresponsiveness (AHR) were evaluated. Histopathologic changes and expression of miR-21 were compared in lung tissues between antagomir treatment group and control groups.

**Results:** Treatment of anti-miR-21 antagomir suppressed AHR compared with OVA challenged group and scrambled RNA treatment group. Antagomir treatment reduced total cell counts and eosinophil counts in BAL fluid. Th2 cytokines such as IL-4, IL-5 and IL-13 were significantly decreased in BAL fluid of anti-miR-21 antagomir treatment group.

**Conclusions:** Antagonism of miR-21 by antagomir inhalation had suppressive effects on development of allergic airway inflammation in acute bronchial asthma model. These results suggest that miR-21 antagomir could be a potential target agent for the treatment of bronchial asthma.

**References**

1) Lu TX, Munitz A, Rothenberg ME. MicroRNA-21 is up-regulated in allergic airway inflammation and regulates IL-12p35 expression. Journal of immunology 2009;182:4994-5002.

2) Lu TX, Hartner J, Lim EJ, Fabry V, Mingler MK, Cole ET, et al. MicroRNA-21 limits in vivo immune response-mediated activation of the IL-12/IFN-gamma pathway, Th1 polarization, and the severity of delayed-type hypersensitivity. Journal of immunology 2011;187:3362-73.

## A188 Chlorhexidine anaphylaxis: A report of two cases

### Maria Ascension Aranzabal^1^, Alejandro Joral^2^, Susana Lizarza^2^, Miguel Echenagusia^3^, EVA Maria Lasa^2^, Jose Antonio Navarro^2^

#### ^1^Hospital De Zumarraga; ^2^Hospital Universitario Donostia, Spain; ^3^Hospital De Mendaro

##### **Correspondence:** Jose Antonio Navarro – Hospital Universitario Donostia, Spain

**Background**

Chlorhexidine is a widely used antiseptic and disinfectant. An increasing number of immediate, IgE-mediated, reactions to this drug have been reported. We present two cases of very severe reactions after its topical use.

**Methods**

Two patients, aged 3 and 77, who suffered anaphylactic shock after contact with topical chlorhexidine were studied. The first case was a 3-year-old boy who underwent hypospadias surgery; among other drugs, chlorhexidine was used. He developed sudden hypotension, desaturation and facial swelling. The boy did not respond to adrenalin and efedrine; inotropic support at the Paediatric Intensive Care Unit was required. The second case was a 77-year-old woman who had a wound that was sutured at the Emergency Room (ER). She presented cough, dysphonia, palmar itching, universal erythema and hypotension that was refractary to intramuscular adrenalin. Mepivacaine was used as local anaesthetic and clorhexidine as disinfectant. In both cases, tryptase and specific IgE to clorhexidine and latex were measured (ImmunoCAP FEIA, Thermo Fisher Scientific), skin tests to clorhexidine (0.5% chlorhexidine digluconate in 0.9% saline) and the other implicated drugs were performed, and also challenge tests were done when needed.

**Results**

*Case 1*. Tryptase during reaction: 13 μg/L; basal tryptase < 1 μg/L. Prick test to clorhexidine: 8x12mm. Negative prick test to latex, negative prick and intradermal tests to rocuronium, fentanyl and bupivacaine were obtained. IgE to clorhexidine: 2.31 kU/L. Subcutaneous challenge test to bupivacaine: negative.

*Case 2*. Tryptase during reaction: 19 μg/L; basal tryptase 6.8 μg/L. Prick test to clorhexidine:11x12 mm. Negative prick test to latex, negative prick and intradermal tests to mepivacaine were obtained. IgE to clorhexidine:3.64 kU/L. Subcutaneous challenge test to mepivacaine: negative.

**Conclusion**

Allergic reactions to chlorhexidine are not common if we consider its widespread use in healthcare settings. However, these reactions can be life-threatening even after topical use so awareness is needed, especially at the ER and at perioperative environments. IgE determination and skin tests are useful tools to get an accurate diagnosis of these patients. Chlorhexidine can be used as an excipient of several medications and cosmetics, therefore a thorough information must be given to chlorhexidine allergic patients in order to avoid further contact with this agent.

## A189 Effects of Particulate Matter on Respiratory Allergic Diseases Considering Meteorological Factors in Busan, Korea

### Eun-Jung Jo^1^, Sun-Mi Jang^1^, Seung-Eon Song^1^, Hae-Jung Na^1^, Chang-Hoon Kim^1^, Woo-Seop Lee^2^, Hye-Kyung Park^1^

#### ^1^Pusan National University Hospital, South Korea; ^2^APEC Climate Center

##### **Correspondence:** Eun-Jung Jo – Pusan National University Hospital, South Korea

**Background:** Ambient pollution has been associated with adverse respiratory health effects. Although meteorological variables have been known to impact on respiratory hospitalization, few studies have investigated the effects of ambient pollution considering temperature or humidity. Here, we identified the effects of ambient particulate matter for daily respiratory admissions considering meteorological factors.

**Methods:** We used daily hospital admissions for respiratory diseases in Busan from hospital records using the International Classication of Diseases (ICD-10) for the period 2007-2010. The respiratory allergic diseases used in our study are allergic rhinitis (J30) and asthma (J45-46). Age was categorized as group I (0-15), group II (16-64) and group III (≥65 yr). Hourly particulate matter < 10μm (PM_10_) levels and particulate matter < 2.5μm (PM_2.5_) levels were obtained from 19 monitoring stations in Busan, and collected by and made available from the Korean Ministry of Environment. The meteorological observation data in Busan provided by the Korean Meteorological Administration included temperature and relative humidity.

**Results:** The mean value of hospital admission for allergy rhinitis and asthma was 4.4±6.1 people and 3.3±3.3 people respectively. A daily-average value of PM_10_ and PM_2.5_ concentrations level was 49.6±20.5 μg/m^3^ and 24.2±10.9 μg/m^3^ respectively. The mean temperature was 15.1±7.9°C, relative humidity was 62.0±18.0%. Admission for allergic rhinitis was associated with increasing temperature anomaly (*P*=0.003) and decreasing relative humidity (*P*<0.001). When the relative humidity was constant, the increase in PM_10_ levels was significantly associated with higher admission for allergic rhinitis. In asthma, hospitalization was correlated with decreasing relative humidity (*P*<0.001) and increasing PM_10_ (*P*=0.008). Even after considering relative humidity, higher PM_10_ levels still were associated with higher hospitalization rate. In subgroup analysis according to age, admission rate in group I and group III was more strongly affected by PM_10_ levels than group II (IRR 7.4 and 6.4, respectively). Furthermore, it showed the association between higher PM_2.5_ levels and admission for asthma, regardless of the effect of PM_10_levels.

**Conclusions:** Particulate matter levels and meteorological factors have an effect on hospitalization for allergic rhinitis or asthma. After adjusting meteorological factors, PM_10_ or PM_2.5_ levels increase significantly admission rate for respiratory allergic diseases.

## A190 Clinical characteristics of neutrophilic asthma

### Sachiko Miyauchi, Yoshitaka Uchida, Tomoyuki Soma, Susumu Yamazaki, Toru Noguchi, Takehito Kobayashi, Kazuyuki Nakagome, Makoto Nagata

#### Saitama Medical University, Japan

##### **Correspondence:** Sachiko Miyauchi – Saitama Medical University, Japan

**Background**: There has been increasing evidences that some asthmatic patients demonstrate neutrophilic airway inflammation. However, clinical features of neutrophilic asthma remain to be elucidated.

**Objective**: To determine clinical features of neutropilic asthma, we investigated clinical variables in patients with asthma attending to our hospital regularly.

**Methods**: Stable patients with bronchial asthma with maintenance treatment underwent sputum induction with administration of the hypertonic saline by a nebulizer. Patients were classified into neutrophilic (NA) or non-neutrophilic asthma (NNA) according to = 40 % or > 40 %, or <40% of neutrophils ratio in sputum. Characteristics of asthma sub-phenotypes were analyzed by disease severity, pulmonary function, blood eosinophil counts, total IgE levels in serum, and specific IgE antibody to environmental allergens.

**Results**: One hundred nineteen patients were divided into 54 NA or 65 NNA. There were no significant differences in the distribution of severity, FEV1 percent predicted, blood eosinophil counts and total IgE levels. In NA, 37 patients (68.5%) had specific IgE antibody to environmental allergens whereas 42 patients (64.6%) in NNA.

**Conclusions**: Proportion of patients with NA sensitized to environmental allergen was equal to NNA. Th2 immune response to aero-allergens might be contributed to development of neutrophilic inflammation in asthma.

## A191 Current Practice of Infants and Children with Acute Urticaria at a Single Wide Regional Emergency Medical Center

### Hea Lin Oh^1^, Do Kyun Kim^1^, Dongin Suh^2^, Young Yull Koh^1^

#### ^1^Seoul National University College of Medicine, South Korea; ^2^Seoul National University Hospital

##### **Correspondence:** Hea Lin Oh – Seoul National University College of Medicine, South Korea

**Background**

Urticaria is a common disease and is one of the most common cause of emergency unit visit in children. However, there is no standardized management about acute urticaria and few articles report about urticaria in infants and children. The aim of this study was to define the clinical features, causes, and current status of treatment in infants and children in emergency department.

**Methods**

We retrospectively reviewed all children aged less than 18years who visited emergency department for acute urticaria or angioedema at Seoul national university hospital, Seoul, Korea, from July 1, 2014 through December 31, 2014. The diagnosis of urticaria and angioedema was made on clinical grounds. The review included a history based on a standardized questionnaires such as precipitating events (eg, food intake, drugs, insect bites or other factors), complete physical examination, further evaluations trying to find the etiology (skin-prick test, serum-specific IgE or multiple allergen stimulation tests) and treatment performed in emergency unit.

**Results**

212 consecutive infants, aged less than 18years, visited emergency department with a final diagnosis of acute urticaria (195 patients, 91.9%) and angioedema (17 patients, 8.01%). The causative event was identified in 112 patients (52.8%), the rest of patients are unidentified. Associated with foods were identified in 84 patients (39.6%), drug intake in 17 patients (8.0%) and others causes in 11 patients (5.1%). The other causes were vaccination, contact with a dog, insect bite or grass exposure. All patients were treated with oral anti-histamine. Anti-histamine injection was given in 134 patients (63.2%), systemic steroid in 25 patients (11.8%) and bronchodilator in 5 patients (2.3%). All the patients but one has discharged with oral anti-histamine, one children was hospitalized suspected of Stevens-Johnson Syndrome. The allergy test was performed in 19 patients (8.9%) at outpatient clinic and 16 patients (7.5%) showed positive results.

**Conclusions**

Causative factors in urticaria are identified in 52.8% and the allergy test are preformed in 8.9% of the patients who visited emergency department with urticaria or angioedema. The causes are dominated by foods, drugs and other causes, in order. Routine treatment included oral anti-histamine therapy and anti-histamine injection, systemic steroid or bronchodilator were added according to the clinical characteristics. The outcome was quiet good.

## A192 Discordance between sputum eosinophilia and exhaled nitric oxide

### Jin-Young Lee^1^, Byung-Jae Lee^2^, Dong-Chull Choi^2^, Jae-Won Paeng^3^, Mi-Jin Jang^3^, Jihye Kim^4^, Young Nam Kim^4^, Sehyo Yune^4^

#### ^1^Health Promotion Center, Samsung Medical Center; ^2^Samsung Medical Center, Sungkyunkwan University School of Medicine; ^3^Samsung Biomedical Research Institute; ^4^Samsung Medical Center, South Korea

##### **Correspondence:** Sehyo Yune – Samsung Medical Center, South Korea

**Background:** Fractional exhaled nitric oxide (FeNO) and induced sputum eosinophil count (ISE) are noninvasive methods to assess airway inflammation in patients with asthma. Despite the significant correlation between FeNO and ISE, they frequently disagree with each other. This study was performed to investigate the characteristics of patients showing discordance of the two parameters.

**Method:** Data from non-smoking patients with asthmatic symptoms from 2011 to 2014 at a single tertiary center was analyzed. FeNO levels were classified into 3 groups (high; FeNO≥50ppb, intermediate; 25ppb≤FeNO<50ppb, and low; FeNO<25ppb). The cutoff value differentiating high and low ISE was 3% of eosinophils in induced sputum. Combining the two criteria, patients were classified into 3 groups; concordant (high FeNO and high ISE, or low FeNO and low ISE), intermediate (intermediate FeNO and either level of ISE), and discordant (high FeNO and low ISE, or low FeNO and high ISE).

**Result:** Among the total of 591 patients, 76 (12.8%) patients were classified into discordant group. Among them, 67 (88.2%) showed high FeNO and low ISE, while only 9 (11.8%) showed low FeNO and high ISE. Comparison between concordant and discordant groups showed that male sex, absence of airway hyperresponsiveness, increased age, and increased neutrophil count in sputum were more frequently seen in discordant group. Current or previous use of corticosteroid, presence of atopy, and salivary contamination of induced sputum were not significantly different between the two groups.

**Conclusion:** Discordance between FeNO and ISE was observed in quite a few patients. Most of the discordance results from high FeNO and low ISE. Further study is necessary to find a proper method to assess airway inflammation in this subset of patients.

## A193 Association between genetic polymorphisms of costimulatory molecules and antituberculosis drugs induced hepatitis

### Sang-Hoon Kim^1^, Jang Won Sohn^2^, Ho Joo Yoon^2^, Dong Ho Shin^2^, Jae Hyung Lee^1^, Byoung Hoon Lee^1^, Youn-Seup Kim^3^, Jae-Seuk Park^3^, Young-Koo Jee^3^, Sang-Heon Kim^2^

#### ^1^Eulji University School of Medicine; ^2^Hanyang University College of Medicine, South Korea; ^3^Dankook University College of Medicine

##### **Correspondence:** Sang-Heon Kim – Hanyang University College of Medicine, South Korea

**Backgrounds:** While the pathomechanisms of antituberculosis drugs (ATD)-induced hepatitis is poorly understood yet, a growing body of evidence supports roles of adaptive immune response in ATD-induced hepatitis. Costimulatory molecules have modulatory effects on activation of T cell, B cell and antigen presenting cell. We examined if the polymorphisms in costimulatory molecules (CD28, CTLA-4, CD40 and CD40L) are associated with ATD-induced hepatitis.

**Methods:** We enrolled 80 patients with ATD-induced hepatitis and 238 ATD-tolerant subjects. DNA was isolated from whole blood and genotyped for the single nucleotide polymorphisms (SNPs) in *CD28, CTLA4, CD40 and CD40LG*. Genotype frequencies of SNPs and haploptyes were compared between patients with ATD-induced hepatitis and ATD-tolerant patients.

**Results:** In selected SNPs of *CD28* (rs3116496), *CTLA4* (rs5742909, rs231775, rs3087243, rs17268364), *CD40* (rs1800686, rs1883832) and *CD40LG* (rs3092952), genotype frequencies were not different between case and control subjects. In the analysis of haplotypes of *CTLA4* and *CD40LG* genes, there was no significant relationship with ATD-induced hepatitis.

**Conclusions:** These findings indicate that genetic polymorphisms of costimulatory molecules (CD28, CTLA-4, CD40 and CD40L) are not associated with the development of ATD-induced hepatitis in Korean population, and suggest that co-stimulatory molecules do not play important roles in the pathogenesis of ATD-induced hepatitis.

## A194 The prevalence of gastroesophageal reflux disease in chronic unexplained cough

### Jin-Young Lee^1^, Jae-Won Paeng^2^, Mi-Jin Jang^2^, Dong-Chull Choi^3^, Byung-Jae Lee^3^, Yongseok Lee^4^, Young Eun Kim^4^, Sehyo Yune^4^

#### ^1^Health Promotion Center, Samsung Medical Center; ^2^Samsung Biomedical Research Institute; ^3^Samsung Medical Center, Sungkyunkwan University School of Medicine; ^4^Samsung Medical Center, South Korea

##### **Correspondence:** Sehyo Yune – Samsung Medical Center, South Korea

**Background:** The common co-existence of cough and gastroesophageal reflux disease (GERD) is well established. Several respiratory guidelines for the management of chronic unexplained cough in adults advocate empirical treatment of GERD. In contrast, guidelines from some gastroenterological societies conclude that cough is unlikely to be related to GERD in the absence of heartburn or acid regurgitation. This study was performed to assess the prevalence of GERD in adults with chronic unknown cough.

**Methods:** Adult patients with cough that persists more than 8 weeks were prospectively enrolled from January 2007 to December 2011. Patients having upper airway cough syndrome, cough variant asthma and nonasthmatic eosinophilic bronchitis were excluded. The enrolled patients underwent 24-hour impedance-pH monitoring of esophagus.

**Results:** The prevalence of GERD in chronic unexplained cough, as evidenced by an abnormal impedance or pH profile, was 46.3% (68 of 147 patients). Among 49 patients who were given anti-reflux medication for at least 3 months, 39 patients (79.6%) achieved total or near-total elimination of cough.

**Conclusion:** GERD, which is readily detected by 24-hour impedance-pH monitoring, is a common cause of chronic unexplained cough and can be successfully managed with anti-reflux therapy.

## A195 Risk Factors of Allergic Rhinitis in Preschool Children and Clinical Utility of Feno

### Jisun Yoon^1,2^

#### ^1^Childhood Asthma Atopy Center, Environmental Health Center, Asan Medical Center, University of Ulsan College of Medicine; ^2^Research Center for Standardization of Allergic Diseases, University of Ulsan College of Medicine, South Korea

**Background**

The nature of allergic rhinitis (AR) in preschool children has yet to be clearly characterized. The aim of this study was to investigate the prevalence and its risk factors of AR and its relationship with FeNO in preschool children.

**Method**

This is a population-based, cross-sectional survey of 1757 preschool children, aged 3-7 years in Korea in 2010. A modified International Study of Asthma and Allergies in Childhood (ISAAC) questionnaire was used. In addition, skin prick tests (SPT), serum total IgE and specific IgE were assessed. Current AR was defined as having nasal symptoms within the last 12 months and diagnosed AR by clinicians. Atopy was defined who had one or more positive reactions on SPT.

**Results**

The prevalence of current AR in preschool age was 23.0% (392 out of 1702) and atopic current AR was 9.65% (93 out of 964). Parental AR (aOR, 3.60; 95% CI, 1.74-7.47), past history of asthma diagnosis (aOR, 2.09; 95% CI, 1.39-3.16) and past history of atopic dermatitis diagnosis (aOR, 1.46; 95% CI, 1.13-1.89) were the independent risk factors for current AR. Specifically, mold exposure during infancy (aOR 1.46; 95% CI, 1.13-1.88) and use of antibiotics in infancy (aOR, 1.89; 95% CI, 1.46-2.45) were also associated with an increased risk of current AR, whereas having older sibling (aOR, 0.53; 95% CI, 0.41-0.69) reduced the risk. Furthermore, atopic current AR without asthma diagnosis had significantly higher geometric mean FeNO levels compared with non-atopic current AR (12.47; 95% CI, 9.85-15.78 vs. 8.72; 95% CI, 8.31-9.15, P=0.0007) as well as higher compared with non-atopic healthy children (8.55; 95% CI, 7.97-9.17, P=0.0004). IgE level (232.9; 95% CI, 179.4-302.4 vs. 68.43; 95% CI, 62.51-74.91, P=<.0001) and eosinophil fraction (4.65; 95% CI, 3.96-5.46 vs. 2.86; 95% CI, 2.72-3.01, P=<.0001) of atopic current AR were higher than non- atopic current AR group.

**Conclusion**

AR is a common disease in Korean preschool children. Mold exposure and antibiotics use during infancy increased the risk whereas older sibling decreased the risk of AR. Furthermore, FeNO levels, IgE, and eosinophil were higher in atopic current AR without asthma group, which suggests FeNO can be a useful diagnostic tool of AR in preschool children.

**Funding**

This study was supported by grant of the Korean Health Technology R&D Project, Ministry of Health & Welfare, Republic of Korea (A092076 and HI13C1674).

## A196 Relationship between serum 25-hydroxyvitamin d and asthma exacerbation severity in children

### Li Zhang, Xuxu Cai, Yong Feng

#### Shengjing Hospital of China Medical University, China

##### **Correspondence:** Yong Feng – Shengjing Hospital of China Medical University, China

**Background**

Vitamin D deficiency has been declared a public health problem for both adults and children worldwide. The growing data suggests that vitamin D deficiency plays an important role in the development of childhood asthma. The objective of this study was to investigate the potential relationship between asthma exacerbation severity and serum 25-hydroxyvitamin D in children.

**Methods**

This study was conducted in Shengjing Hospital of China Medical University, from September 2013 to March 2014. A total of 49 children with asthma exacerbation, aged between 3 and 14 years, were enrolled. The children should not have underling diseases such as chronic lung disease, congenital heart disease, chronic renal disease, etc. and vitamin D supplement in recent 3 months. According to GINA assessment of asthma exacerbation, they were divided into mild, moderate and severe exacerbation group. Serum 25-hydroxyvitamin D were assessed in all 49 children.

**Results**

The 49 patients were 28 boys and 21 girls, mean age 5.28±2.28 years old. According to GINA assessment of asthma exacerbation, 20 (40.82%) children were enrolled in mild exacerbation group, 15 (30.61%) children were enrolled in moderate exacerbation group and 14 (28.57%) children were enrolled in severe exacerbation group. There was no significant differences in age, height and weight among the three groups (p>0.05). The concentration of serum 25-hydroxy vitamin D in mild, moderate and severe exacerbation groups was 35.77±13.64 nmol/L, 15.30±4.97 nmol/L and 13.87±3.33 nmol/L separately. The serum 25-hydroxyvitamin D of mild exacerbation group is significantly higher than moderate and severe exacerbation groups. But no significance was found between moderate and severe exacerbation groups.

**Conclusions**

Children with moderate and severe asthma exacerbations had lower serum 25-hydroxy vitamin D than mild cases, which suggests that 25-hydroxyvitamin D is associated with asthma exacerbation severity in children. Further research should be focused on vitamin D supplementation for the prevention of childhood asthma.

## A197 Usefulness of Specific IgE Antibody Levels to Wheat, Gluten and Ï‰-5 Gliadin for Wheat Allergy in Korean Children

### Jong-Seo Yoon^1^, Kyunguk Jeong^1^, Hye-Soo Yoo^2^, Sooyoung Lee^2^, Sooyoung Lee^1^

#### ^1^Ajou University School of Medicine, South Korea; ^2^Suwon Center for Environmental Disease and Atopy

##### **Correspondence:** Jong-Seo Yoon – Ajou University School of Medicine, South Korea

**A) Backgroud**: The aim of this study was to assess the clinical usefulness and added diagnostic value of specific IgE antibodies to wheat, gluten and ω-5 gliadin in diagnosing wheat allergy and distinguishing wheat anaphylaxis.

**B) Methods**: This study included 196 children who visited Ajou University Hospital for suspicious food allergy. The subjects were divided into two groups; wheat allergy (WA) group and non-wheat allergy (Non-WA) group. The subjects with wheat allergy were further divided into two groups according to their symptoms: wheat anaphylaxis (WA^Ana^) group and non-wheat anaphylaxis (WA^Non-Ana^) group. The serum concentrations of total IgE and specific IgE antibodies to wheat, gluten and ω-5 gliadin were measured. The diagnostic values of each specific antibody were analyzed and compared using the Mann-Whitney test and receiver operating characteristic curves.

**C) Results**: The median values of specific IgE antibodies to wheat, gluten and ω-5 gliadin were significantly higher in WA compared to those in Non-WA, and the optimal cutoff points were 0.90 kU_A_/L, 0.43 kU_A_/L and 0.19 kU_A_/L respectively. The positive decision points (specificity of 95%) of specific IgE antibodies to wheat, gluten and ω-5 gliadin were 3.12 kU_A_/L, 2.61 kU_A_/L and 0.21 kU_A_/L respectively, and the accuracies at these levels were 91.8%, 92.9% and 93.4% correspondingly. The combination of specific IgE antibodies to wheat and ω-5 gliadin resulted in the highest accuracy of 93.9% in diagnosing wheat allergy. In differentiating WA^Ana^ from WA^Non-Ana,^, only the specific IgE antibodies to ω-5 gliadin showed significant difference (p=0.013) at the optimal cutoff point of 1.56 kU_A_/L.

**D) Conclusion**: Our results show that the individual levels of specific IgE antibodies to wheat, gluten or ω-5 gliadin had considerably high accuracies in diagnosing wheat allergy, and specific IgE antibody to ω-5 gliadin was particularly useful in predicting wheat anaphylaxis.

## A198 Neutralization of stratum corneum accelerates the progress from atopic dermatitis to asthma-like lesion in flaky tail mice treated by house dust mite allergen

### Hae-Jin Lee^1^, Noo Ri Lee^1^, Bo-Kyung Kim^1^, Minyoung Jung^1^, Dong Hye Kim^1^, Catharina S. Moniaga^2^, Kenji Kabashima^2^, Eung Ho Choi^1^

#### ^1^Yonsei University, Wonju College of Medicine, South Korea; ^2^Kyoto University Graduate School of Medicine, Japan

##### **Correspondence:** Hae-Jin Lee – Yonsei University, Wonju College of Medicine, South Korea

**Background:** A disrupted skin barrier of atopic dermatitis (AD) permits easier penetration and sensitization of external allergens and then induces the development of asthma and allergic rhinitis, so called the ‘atopic march’. Maintenance of normal acidity in the stratum corneum (SC) is an important factor for normal skin barrier function. Elevation of SC pH accompanied by the increase of serine protease activity and TSLP expression provokes skin inflammation.

**Objective:** We determined whether the neutralized SC environment can accelerate airway inflammation more easily in flaky tail mice, a representative AD murine model with congenital skin barrier abnormality, after the exposure of house dust mite (HDM).

**Methods:***Dermatofagoides pteronyssinus* (Dp), a HDM was applied on the dorsal skin of flaky tail mice twice a week, which accompanied by the application of neutral cream (pH 7.4) and acidic cream (pH 2.8) twice a day for 6 weeks. Intranasal inhalation of Dp was done daily during last 3 days. Gross findings, functional study for skin barrier, blood sampling, bronchoalveolar lavage and biopsies of skin and lung were done at 24 h after last treatment.

**Results:** Repeated topical applications followed by intranasal inhalations of Dp to flaky tail mice made the respiratory allergic inflammation as well as the AD-like skin lesion. Accompanying neutral cream treatment accelerated or aggravated the allergic inflammation in the respiratory system as well as the skin.

**Conclusion**: Atopic march-like progress from AD to asthma-like lesion can be observed in flaky tail mice after topical and intranasal application of HDM, and the neutralization of SC can accelerate or aggravate it.

## A199 Trends in oral food challenges in Japan: A six-year prospective study

### Noriyuki Yanagida, Sakura Sato, Chizuko Sugizaki, Motohiro Ebisawa

#### Sagamihara National Hospital, Japan

##### **Correspondence:** Noriyuki Yanagida – Sagamihara National Hospital, Japan

The number of patients with food allergies is increasing on a global level. The estimated prevalence in Japan is 5–10% among infants and 1–2% among schoolchildren. The use of oral food challenges (OFCs) is the gold standard for the diagnosis of food allergies. As of 2015, Japan is currently the only country in the world in which OFC testing is covered by health insurance. We performed a questionnaire survey to all specialist training facilities belonging to the Japan Pediatric Society one time per year, excluding the 2013 fiscal year prospectively. Of the total 2,579 facilities that received the questionnaire during the six-year period, 1,993 responses (77.2%) were returned. The total OFC testing rates, including outpatient and inpatient OFC, in the 2008-2011 and 2013 fiscal years were 71.4%, 71.9%, 73.7%, 74.6%, and 85.7%, respectively. The percentage of nonspecialists performing inpatient OFC testing in the 2013 fiscal year (66.7%) was significantly higher in comparison with all other years (fiscal year 2008 [51.5%, p=0.014], fiscal year 2009 [47.8%, p<0.001)], fiscal year 2010 [53.0%, p=0.032], fiscal year 2011 [49.8%, p=0.004]). The total estimated OFC testing fill-rate in Japan for each year was 6.4%, 8.8%, 9.3%, 10.0%, and 17.1%, respectively. These results indicate that the increased percentage of non-allergists performing inpatient OFC testing lead to increase of total estimated OFC testing fill-rate in Japan.

## A200 The Gut Microbiome in the Food Allergic Host

### Jamie Kiehm, Punita Ponda, Sherry Farzan, Jared Weiss, Claudia Elera, Catherine Destio, Cristina Sison, Annette Lee

#### Shore Long Island Jewish Health System, USA

##### **Correspondence:** Jamie Kiehm – North Shore Long Island Jewish Health System, USA

**A) Background:** Alterations in the diversity and composition of the human gut microbiota have been associated with a myriad of diseases including inflammatory bowel disease and metabolic syndrome. In our study, we explore the association of the host gut microbiome with food allergy.

**B) Methods:** 12.5 ng of isolated DNA from fecal samples from 8 children with peanut allergy (peanut specific IgE >15 kUA/L) and 10 healthy, non-atopic controls were amplified using primers specifically for the V3-V4 region of the 16S rRNA gene which is unique to bacteria. Sequence differences within the V1-V9 variable region were used for bacterial classification. Once amplified, the amplicons were dual-indexed for multiplex sequencing. Pooled libraries were sequenced on the Illumina MiSeq using 300 cycle paired end reads. Sequence data was processed through the MiSeq Reporter Metagenomics 16S application and the Greengenes 16S ribosomal RNA gene database. Data regarding family history of atopic disease, history of breastfeeding, antibiotic treatment per year, medications, and history of other atopic disease was collected via questionnaires. The Mann-Whitney *U-*test was used for comparison of continous variables between the two groups. The Fisher’s exact test was used to compare proportions between groups. All analyses were carried out using SAS V9.3.

**C) Results:** The median age of subjects with peanut allergy was 6 years (average peanut specific IgE=75.1 kUA/L), and median age of healthy controls was 4 years. All of the patients with peanut allergy had a history of other atopic diseases. The phylogentic differences showed that peanut allergic subjects had an increased proportion of *Firmicutes* (median*=*71% vs 59%; p<0.03*)* and a decreased proportion of *Proteobacteria* (median*=*1% vs 3%; p<0.009) when compared to healthy controls. At the class level, *Clostridia* was more abundant in the peanut allergic subjects (median*=*69% vs. 57%; p<0.03). *Clostridiales* order was significantly more abundant in the peanut allergic subjects (median*=*69% vs. 56%; p<0.03). *Alcaligenaceae* family was found in 6/10 healthy controls and in none of the 8 peanut allergic subjects (60% vs 0%; p<0.013). 16 genera were identified in healthy controls while 12 genera were identified in peanut allergic subjects. The 12 genera identified in the peanut allergic subjects accounted for 82.76% of the total sequences and were all found in the healthy controls. The 16 genera found in healthy controls accounted for 79.97% of the total sequences.

**D) Conclusions:** Analysis of the human gut microbiome in children with peanut allergy and healthy, non-atopic controls revealed differences in the specific composition and diversity of the microbiota that may contribute to the pathogenesis of food allergy. More studies with a larger sample size are needed to further investigate these associations.

## A201 Cord blood cytokines and maternal environmental exposure during pregnancy

### Soo Hyun Ri, Chang Hoon Lim

#### Korea University Medical Center, South Korea

##### **Correspondence:** Soo Hyun Ri – Korea University Medical Center, South Korea

**A) Background:** It has been suggested that initial priming of the T-cell system to environmental allergens may occur before birth. Cytokine expression in cord blood has been increasingly used as useful markers to assess the neonatal immunological development and future lifetime risk for various allergic diseases among offspring. The aim of this study is to evaluate the relationship between cytokine expression in neonatal cord blood and exposure to house dust mite (HDM) and food intakes during pregnancy.

**B) Methods:** 405 pregnant women were recruited between April 2010 and November 2010. 111 women completed the study. The pregnant women with the genetic diseases, autoimmune diseases, and endocrinologic diseases were excluded. Parental allergic diseases, allergen sensitization, exposure to HDM, maternal diet during pregnancy, and cytokine expression in neonatal cord blood of all subjects were investigated. Information on physician–diagnosed allergic diseases of the subjects and/or their spouses had been gathered from the questionnaires and allergy specialist confirmed diagnosis after examining the subjects who declared to have allergic diseases on the questionnaires. Allergen sensitization was performed by using the skin prick test including Der p 1, Der p 2, tree mixture, grass mixture, weed, dog and cat. Dietary intakes and nutrition during the last 3 months of pregnancy period were assessed using the semi-quantitative food questionnaire and indoor environments of each household including exposure to dust mites were investigated by semi-quantitative rapid test. Expressions of IL-4, IL-13, and INF-γ of neonatal cord blood were examined by using quantitative real-time PCR.

**C) Results:** Among subjects, the pregnant women with diagnosed allergic diseases were 54 and most common allergic disease was allergic rhinitis. (61.1%) 54 women were exposed to tobacco smoking and 5 women were living with pets.

All subjects showed significant differences in expression of IL-4 and IFN-γ. (n=111, p<0.05) IL-4 /IFN- γ ratio was 4.63±8.72. (mean±SD) No significant relationship is observed between IL-4 and IFN-γ of neonatal cord blood and subject’s environments including exposures to tobacco smoking, pet ownership, carpet use, detectable HDM and bedding for protecting HDM use.

HDM was detected in 29 pregnant women’s household, but significant difference between HDM detected and HDM non-detected was not showed. IL-4 and IFN-γ in cord blood of neonate with allergic mother had higher expression than those of non-allergic mother. (p<0.05) The amounts of nutrition weren’t related to neonatal cord blood IL-4 and IFN- γ.

**D) Conclusions:** This is the study about the relationship between maternal food intakes and exposure to HDM during pregnancy and fetal immunity. The study found that 1) infants had Th1/Th2 polarization at birth and that 2) Expression of IL-4 and INF-γ was related to maternal allergic disease.

## A202 Rupatadine pharmacokinetics in Japanese healthy volunteers after single and repeated oral doses of 10, 20 and 40 mg

### Iñaki Izquierdo Pulido^3^, Jorg Taubel^1^, Georg Ferber^2^, Eva Santamaria Masdeu^3^

#### ^1^Richmond Pharmacology, England; ^2^Statistik Georg Ferber Gmbh, Switzerland; ^3^J. Uriach y Compañía, S.a, Spain

##### **Correspondence:** Iñaki Izquierdo Pulido – J. Uriach y Compañía, S.a., Spain

**Background**

Rupatadine is a second generation anti-H1 antihistamine and PAF antagonist authorised for the treatment of allergic rhinitis and urticaria at the dose of 10 mg. The aim of this study is to assess the pharmacokinetics and safety of rupatadine following single and multiple oral administrations to healthy Japanese subjects.

**Methods**

This was a phase I, randomised, double-blind, placebo controlled, parallel group study with oral single and multiple doses that were administered to male and female healthy Japanese subjects from 20 to 45 years of age. Twenty seven subjects (nine per each cohort of dose) were randomised in a 3.5:1 ratio to rupatadine (10, 20 and 40 mg) or matching placebo. On Day -1, all subjects received a single dose of placebo, followed by one single daily oral dose on Day 1 and once single daily doses on Days 2 to 5. Plasma samples collected at different time-points throughout the study were analysed by means of a validated LC-MS/MS analytical method to determine rupatadine and UR-12790 and UR-12788 metabolites concentration. Pharmacokinetic parameters were evaluated by using a noncompartmental analysis, and regression models were used to assess dose linearity.

**Results**

PK exposure after rupatadine administration of single and multiple doses in Japanese subjects was found to increase in a dose dependent manner. The dose proportionality analysis revealed that the 90% CI of slope for Cmax (rupatadine: 0.65-1.23; UR-12790: 0.87-1.38) and AUC0-∞ (rupatadine: 0.75-1.25; UR-12790: 0.80-1.18) on Day 1 and Cmax (rupatadine: 0.55-1.19; UR-12790: 0.89-1.24) and AUC0-tau (rupatadine: 0.75-1.22; UR-12790: 0.83-1.20) on Day 5 for rupatadine and UR-12790 were very close to the range 0.8 to 1.25 and did clearly include 1. Single and multiple oral doses of rupatadine were well tolerated. There were no serious adverse events in this study and no subject withdrew due to safety reasons or any other reasons.

**Conclusions**

The PK data are not incompatible with dose proportionality and appear to be even more conclusive after repeated doses instead of single doses. Additionally, the PK values in Japanese show similarity to the historic values obtained in Caucasian studies. Rupatadine was safe and well tolerated by Japanese subjects. There were no SAEs in this study and no subject withdrawals due to AEs or any other reason.

## A203 A safe and effective method to desensitize patients with wheat allergy

### Alireza Khayatzadeh^1^, Masoud Movahedi^1^, Motohiro Ebisawa^2^, Mohammad Gharagozlou^1^

#### ^1^Children’s Medical Center, Iran; ^2^Sagamihara National Hospital, Japan

##### **Correspondence:** Alireza Khayatzadeh – Children’s Medical Center, Iran

**Background**

At present the only available management for food allergy is avoidance but wheat avoidance results in dietary limitations and can affect the quality of life. Oral Immunotherapy (OIT) is a method that has been shown to increase tolerance to wheat in allergic patients.

**Objective**

The aim of this study was desensitizing patients above five years with wheat allergy and evaluating the safety and efficacy of Rush Oral Immunotherapy (ROIT) for children with IgE mediated wheat allergy.

**Methods**

According to patient’s history and symptoms that occurred during Double-Blind Placebo-Controlled Food Challenge test (DBPCFC), eleven children above 5 years old with IgE-mediated wheat allergy and anaphylaxis underwent ROIT. Skin prick test, measurement of serum specific IgE against wheat flour and oral food challenge (OFC) performed before and after OIT. Our protocol was consisting of initial build up phase following maintenance phase. Maximum dose during build up phase was 5.2 g wheat protein. We used bread containing 10% wheat protein for ROIT. In this method the patients hospitalized and build up phase performed during several days. After completion the buildup phase, the patients were asked to ingest the maintenance dose (52 g of bread) "daily" for 3 months at home. Then they received confirmed OFC. If this challenge was negative, we regarded them as desensitized state and they had to eat wheat products daily without interruption.

**Results**

Among 11 patients, 9 (81.8%) were male and the average age of patients at beginning of the study was 6.2 years old (range, 5-11 years). All patients completed buildup phase successfully. One patient discontinued the maintenance phase for personal reason. Ten patients completed maintenance phase successfully and became desensitized. They are now consuming wheat products freely without any complications. Threshold doses in the wheat DBPCFC was from 0.08 g to 1.3 g of wheat protein. Build up phase continued in 3 to 7 days. Eight of 11 patients showed symptoms (72.7%) during this period, the total number of doses were 101 in which 25 of applied doses (24.8%) showed symptoms which mostly were mild reactions. Number of epinephrine that we took advantage was 9 in total 101 applied doses (8.9%). During maintenance phase seven patients showed mild reaction in first month of maintenance phase. The total number of doses in maintenance phase was 900 in which 52 of applied doses (5.8%) showed mild symptoms.

**Conclusions**

Wheat OIT through this method could be simple, safe and effective in selected patients with wheat allergy, although it needs further investigations with more patients.

## A204 RNA Binding Protein Hur Regulates CD4+ T Cell Differentiation and Is Required for Allergic Airway Inflammation and Normal IL-2 Homeostasis

### Ulus Atasoy^1^, Patsharaporn Techasintana^2^, Matt Gubin^3^, Jacqueline Glascock^2^, Suzanne Ridenhour^2^, Joseph Magee^2^

#### ^1^University of Missouri-Columbia, USA; ^2^University of Missouri; ^3^Washington University

##### **Correspondence:** Ulus Atasoy – University of Missouri-Columbia, USA

Posttranscriptional control by RNA binding proteins (RBPs) of CD4^+^ T cell development is poorly understood. We previously demonstrated that the RBP HuR (*elavl1*) controls Th2 differentiation via increased stabilization of Gata-3, IL-4 and IL-13. HuR CD4^+^ T from transgenic mice which over-expressed HuR had significant increases in Th2 but not Th1 cytokines. IL-2 is the primary growth factor for activated T cells. When IL-2 engages its’ low affinity receptor, IL-2Rα (CD25) this results in phosphorylation of stat5 which turns off IL-2 via prdm1, which encodes the IL-2 transcriptional repressor, blimp-1. p-stat5 also opens up accessibility of the IL-4 gene to transcription, leading to Gata-3 activation, which is essential for Th2 differentiation. We hypothesized HuR regulates CD4^+^ T cell differentiation and is required for normal IL-2 homeostasis. We generated a HuR conditional KO model to study effects of HuR ablation in CD4^+^ T cell activation (distal *lck-cre*-ROSA HuR^fl/fl^). The ROSA gene expresses YFP which is used to track HuR KO cells. We sorted CD4^+^ T cells into YFP^+^ and YFP- groups and activated them with anti-CD3/CD28. Activated YFP^+^ CD4^+^ T HuR KO cells had profound defects in Gata-3, IL-4, IL-5 and IL-13 expression and could not shut off IL-2. Up to 97% of activated T cells were unable to turn off IL-2 expression. HuR KO T cells had defects in proliferation and JAK-STAT signaling with reduced p-stat5 and were unable to up-regulate CD25. We found significantly increased IL-2 but decreased Gata-3, prdm-1, IL-4 and CD25 transcription. We investigated whether HuR directly controlled CD25 expression. We verified HuR interacts with defined AU-rich elements (AREs) in CD25 3’UTR. CD25 mRNA stability was unchanged in HuR KO T cells but there were translational defects in recruitment of CD25 mRNA transcripts to heavy polysomes. To determine *in vivo* relevance, we used the ovalbumin challenge model of allergic airway inflammation. HuR KO mice had significant decreases in lung neutrophils, lymphocytes, eosinophils and IL-13. Remarkably, immunized HuR KO mice had comparable levels of total lung inflammation as un-immunized controls. We confirmed increased IL-2 and reduced CD25 phenotype in CD4^+^ T cells from immunized HuR KO mice. In summary**,** these data suggest that HuR plays a major role in CD4^+^ Th2 differentiation and normal IL-2 homeostasis by controlling CD25 mRNA translation. Furthermore, HuR KO in T cells completely abrogates allergen induced lung inflammation. Therefore, HuR-CD25 interactions are required for normal IL-2 homeostasis and Th2-mediated allergic airway inflammation.

## A205 Time Trends in the Epidemiology of Recurrent Wheezing in Infants from South America

### Nelson Rosario Filho^9^, Herberto Jose Chong Neto^1^, Gustavo Falbo Wandalsen^2^, Ana Caroline Dela Bianca^3^, Carolina Aranda^4^, Dirceu Sole^5^, Javier Mallol^6^, Luis Garcia-Marcos^7^, Luis Garcia-Marcos^8^

#### ^1^Federal University of Parana, Brazil; ^2^Federal University of Sao Paulo, Brazil; ^3^Universidade Faderal De Pernambuco, Brazil; ^4^WAO Junior Member; ^5^Brazilian Society; ^6^University of Santiago De Chile (USACH), Chile; ^7^Imib-Arrixaca Research Institute; ^8^Arrixaca University Children’s Hospital. University of Murcia, Spain; ^9^University of Parana, Brazil

##### **Correspondence:** Nelson Rosario Filho – University of Parana, Brazil

**Background:** The changes over time in the prevalence and severity of recurrent wheezing (≥3 wheezing episodes) in infants, are virtually unknown. To determine the trend of RW prevalence and severity of RW in infants during the first year of life between two surveys separated by seven years.

**Method:** This is a part of International Study of Wheezing in Infants (EISL), a population-based study, in infants aged 12-15 months, compares data from two surveys (S1 and S2) performed using the same methodology, in three large cities that participated in both surveys: Curitiba and São Paulo (Brazil) and Santiago (Chile).

**Results:** The average prevalence of wheezing once or more during the first year of life decreased from 52.9% (95%CI 51.8-54) in S1 to 46.9% (95%CI 45.2-48.07) in S2, p = 0.0001. There was a significant decrease in the mean prevalence of RW between S1 (23.3%, 95%CI 22.3-24.3) and S2 (20.4%, 95%CI 19.0-21.8), p=0.004. In infants with RW the mean prevalence of severity markers between S1 and S2 remained high (severe episode: 56.9% to 54.2%, p = 0.32); ED visits (68.1% to 70.9 %, p = 0.21), with a significant increase in admissions for wheezing (21.1% to 26.7%, p = 0.004).

**Conclusion:** The prevalence of RW and severity markers during the first year of life remained high between both surveys. The infants who suffer from RW have markedly high prevalence of severity markers as visits to ED and admissions for wheezing, indicating that an important group of these infants has a troublesome progression that certainly affect quality of life and put infants at risk of more severe complications.

## A206 Successful Cyclophosphamide Desensitization in a Pediatric Patient with Systemic Lupus Erythematosus

### Jennifer Toh^1^, Yoomie Lee^1^, Joyce Huang^1^, Elina Jerschow^2^, Jenny Shliozberg^1^

#### ^1^Children’s Hospital at Montefiore, USA; ^2^Montefiore Medical Center

##### **Correspondence:** Jennifer Toh – Children’s Hospital at Montefiore, USA

**Background:** Cyclophosphamide (CYC) is an alkylating agent used to treat malignancies and autoimmune diseases. It is often given with mercaptoethane sulfonate (mesna) to prevent bladder toxicity. Hypersensitivity to CYC has been well described in adult patients with malignancy. These reactions are typically IgE-mediated to CYC or its metabolites after a previous exposure. Most of these patients have tolerated subsequent treatments by desensitization. We report the first described case of anaphylaxis after initial exposure of CYC in a pediatric patient with systemic lupus erythematosus (SLE) who successfully tolerated subsequent CYC by desensitization.

**Method:** Case description.

**Results:** An 11-year-old girl with SLE and Class III/V nephritis was admitted for hematuria, anasarca, and uncontrolled hypertension consistent with SLE nephritis flair. Her treatment was switched from mycophenolate mofetil to monthly CYC (945mg, 0.75 gm/m2) intravenously (IV) with mesna infusions given 30 minutes prior and 3, 6 and 9 hours after CYC infusion. Immediately after completing her first CYC infusion, she developed increased erythema at her IV site, angioedema of the lips and tongue, facial flushing, shortness of breath, chest tightness, and wheezing. She was treated for anaphylaxis with intramuscular epinephrine, IV diphenhydramine, methylprednisolone, and famotidine with An hour later, she became hypotensive (BP 91/31 from BP 147/69). Antihypertensives were held and she continued receiving IV diphenhydramine and methylprednisone. She received her post-treatment mesna infusions without any adverse event. She had no history of allergies or exposure to alkylating agents in the past. CYC was the preferred therapy as her renal function improved after her first treatment and Allergy was consulted to evaluate whether her anaphylaxis was secondary to mesna or CYC and for possible desensitization. Allergy evaluation revealed negative percutaneous testing to mesna (10mg/ml skin prick; 0.01mg/ml and 0.1mg/ml intradermal). CYC skin testing was deferred by the patient’s guardian. She was admitted to the intensive care unit to receive CYC via a 12-step desensitization protocol. Her daily prednisone 40 mg dose was increased to methylprednisone 1 gm at 45 minutes prior to desensitization. She was also premedicated with diphenhydramine 25 mg and famotidine 20 mg at 20 minutes prior to desensitization. She tolerated desensitization without any adverse reactions. Since then, she has received CYC by desensitization every month at an outpatient infusion setting without any adverse event.

**Conclusions:** Hypersensitivity to CYC can be seen in pediatric patients with autoimmune disease. It can occur after initial exposure and can be as severe as anaphylaxis. Desensitization is one approach for continued treatment with CYC in pediatric patients with SLE which has been successful.

## A207 The Fatty Acid Binding Protein Der p 13 Is a Minor House Dust Mite Allergen Able to Activate Innate Immunity

### Pattraporn Satitsuksanoa^1^, Narissara Suratannon^2^, Jongkonnee Wongpiyabovorn^2^, Pantipa Chatchatee^2^, Kiat Ruxrungtham^2^, Alain Jacquet^2^

#### ^1^Faculty of Medicine, Thailand; ^2^Chulalongkorn University

##### **Correspondence:** Pattraporn Satitsuksanoa – Faculty of Medicine, Thailand

**Background:** Compared with other group 13 house dust mite (HDM*)* allergens, Der p 13 remains very poorly characterized. We recently produced a recombinant form of Der p 13 in *P.pastoris* and demonstrated that this allergen binds lipids/fatty acids. This study investigated IgE sensitization to rDer p 13 in a large thai HDM allergic cohort as well as the allergen-induced airway epithelial cell activation.

**Methods:** The IgE reactivity to rDer p 13 was analysed by ELISA using 644 sera with a positive skin prick test (SPT) to *D.pteronyssinus* and collected from four different hospitals in Bangkok. The allergenic activity of rDer p 13 was tested using IgE-loaded rat basophil leukaemia cells (RBL) expressing human FcεRI. The direct airway epithelial cell activation by rDer p 13 was also evaluated.

**Results:** Only 6% of 644 HDM-allergic patients showed IgE-reactivity to rDer p 13 whereas the IgE binding frequency to rDer p 2 reached more than 60%. In RBL assays, rDer p 13 triggered basophil degranulation but the effector activity was lower than that measured for rDer p 2. Treatment of BEAS-2B respiratory epithelial cells with rDer p 13 triggered the production of IL-8 in a *concentration-dependent* manner*.*

**Conclusions:** Although rDer p 13 displays allergenic activity, its weak IgE reactivity clearly confirmed that Der p 13 is a minor allergen. We hypothesized that the poor IgE binding frequency of Der p 13 is in line with the apparent very limited amount of this allergen in mite fecal pellets aqueous extracts. Nevertheless, Der p 13, through its fatty acid binding capacity, could play a role in the activation of innate signaling pathways to initiate the allergic response.

## A208 Epidemiology of Stevens-Johnson Syndrome and Toxic Epidemal Necrolysis: An Administrative Database Study

### Min Suk Yang^1^, Jin Yong Lee^1^, Ja Yeun Kim^2^, Han-Ki Park^3^, Ju-Young Kim^4^, Woo-Jung Song^4^, Hye-Ryun Kang^4^, Heung Woo Park^4^, Yoon-Seok Chang^5^, Sang-Heon Cho^4^, Kyung-up Min^4^, Chang-Han Park^6^, Suk-Il Chang^6^, Sook-Hee Song^7^

#### ^1^Smg-Snu Boramae Medical Center, South Korea; ^2^Seoul National University; ^3^Ewha Institute of Convergence Medicine, Ewha Womans University Medical Center; ^4^Seoul National University Hospital; ^5^Seoul National University Bundang Hospital; ^6^Sung-Ae General Hospital; ^7^Seoul Medical Center

##### **Correspondence:** Min Suk Yang – Smg-Snu Boramae Medical Center, South Korea

**Background**: Nationwide incidence of Stevens-Johnson syndrome (SJS) and toxic epidermal necrolysis (TEN) is hard to estimate. We report nationwide incidence of SJS and TEN using an administrative database.

**Method**: We used the database of the Health Insurance Review and Assessment Service (HIRA) in Korea. We employed the HIRA database from 2009 to 2013 to estimate the annual incidence, in-hospital mortality, related complications due to SJS and TEN. In this study, using the International Classification of Diseases-10th Revision (ICD-10), target study population was defined as patients with SJS or TEN, who had the primary diagnostic codes of L511 (SJS) or L512 (TEN), respectively.

**Result**: During four-years study period, estimates of annual incidence of SJS and TEN were 4.9-5.5 and 0.9-1.4 per million people. Mortality rate were 5.7% for SJS and 15.1% for TEN. Mean age was about 50 years old and female predominance was not so apparent in our data. Ocular and urethral sequelae were the most significant sequelae clinically that more than 40% of patients with both diseases suffered from ocular sequelae and about 6% of SJS and 9% of TEN patients were affected by the urethral sequelae. Mortality rate increased as the patients’ age got older.

**Conclusion**: The incidence of SJS and TEN was not so much changed since 1990’s. However, the mortality rate was decreased and this would be due to the evolution of supportive management.

## A209 Regional Differences of Vitamin D and Food-Induced Anaphylaxis in Korea

### Si-Heon Kim^1^, Gil-Soon Choi^2^, Su-Chin Kim^1^, Ji Hye Kim^1^, Ga Young Ban^1^, Yoo Seob Shin^1^, Hae-Sim Park^1^, Young Min Ye^1^

#### ^1^Ajou University School of Medicine South Korea; ^2^Kosin University Gospel Hospital

##### **Correspondence:** Si-Heon Kim – Ajou University School of Medicine, South Korea

**Background:** It has been suggested that vitamin D deficiency has an influence on increase in the prevalence of allergic disease. A few studies have been reported that epinephrine prescriptions varied from latitude of the regions. The present study aimed to examine the regional differences in incidence of food-induced anaphylaxis (FIA) and serum vitamin D level in Korea.

**Method:** Nationwide data on FIA from2011 to 2013 were obtained from the Health Insurance Review and Assessment Service. We used serum vitamin D levels from the Korea National Health and Nutrition Examination Surveys during the period corresponding to FIA. Regions were divided into 2 categories based on the latitude N36.2° (Region 1 for high latitude and Region 2 for low latitude). We examined the differences in incidence of FIA and serum vitamin D levels between 2 regions after adjusting for age.

**Results:** The number of cases of FIA was 2,851, and serum vitamin D levels were obtained in 15,368 persons from 2011 to 2013. FIA incidence was significantly higher (2.00 vs 1.73 per 100,000 person-years, p=0.03), and serum vitamin D level was lower (16.5 vs 18.1 ng/ml, p<0.001) in Region I as compared with Region 2. For all age groups of <20, 20-59, and ≥60 years, serum vitamin D levels were significantly higher in Region 2 than in Region 1. The regional difference of FIA incidence was significant in persons aged <20 years (1.90 in Region1 vs 1.15 in Region 2, p<0.001), but insignificant in other age groups. Serum vitamin D level in Region 1 was higher than that in Region 2 for both males and females. However, there was no difference in FIA incidence between 2 regions according to gender.

**Conclusions:** Overall, higher FIA incidence and lower vitamin D levels were found in relatively higher latitude region compared with lower region. After stratification by age groups and gender, FIA incidences and serum vitamin D levels were inconsistent in aspect of the regional differences. Further investigations are necessary to identify the relationships between FIA and vitamin D.

## A210 Triggering Factors of Atopic Dermatitis By Severity

### Yoon Ha Hwang

#### Busan St.Mary’s Hospital, South Korea

**Background**: Atopic dermatitis is exacerbated by several triggering factors, which are allergens, stress, infections climates and so on. We investigated triggering factors for better control of atopic dermatitis in children.

**Methods**: Subjects were diagnosed as atopic dermatitis by pediatricians of allergy clinic of Busan St.Mary’s Hospital from April, 2014 to March, 2015. We got history taking, allergy test (SPT, ImmunoCap), Skin culture, and physical examination for checking triggering factors. We also classified by severity (SCORAD score), and analyzed each group’s triggering factors.

**Results**: Subjects number was 301. (male 185, 3month to 18 years old, average 4.6). Triggering factors were skin infections, foods (immediate 29, delayed 10), itching (21), traditional medicine (18), and systemic infections (16). Skin infections were by bacteria (S.aureus(SA) 178, methicillin-resistant SA(MRSA) 77), Fungi (Malassezia 78, average IgE 7.1kUA/L), and virus(Kaposi varicelliform eruption 58). Mild groups were 157, (male 89, average age 4.1) and triggered by SA/MRSA (77/31), malassezia (29, IgE 4.3), KVE (12), foods (immediate/delayed 14/6), itching (8), sweat(7), irritation(6), systemic infection(5), and environment(4) in order of frequency. Moderate group were SA/MRSA (87/39), malassezia (37, IgE 7.2), KVE (34), foods (immediate/delayed 12/4), itching(10), traditional medicine(10), systemic infection(9), topical ointment tolerance(7), and sweat(5). Severe group were 22, (male 15, average 8.0) triggered by SA/MRSA (14/7), malassezia (12, IgE 13.4), KVE (12), traditional medicine(7), foods (immediate/delayed 3/0). itching(3), systemic infection(2), and irritation(1, dyeing).

**Conclusion**: Skin infections, traditional medicine are high proportions of triggering factors of atopic dermatitis in severe group. Mild-moderated group were mainly triggered by skin infection, foods, and itching.

## A211 Clinical Features of Adverse Drug Reactions of Monoclonal Antibodies in Korea

### Da Woon Sim^1,2^, Kyung Hee Park^1^, Kyung Hee Park^2^, Hye Jung Park^1^, Hye Jung Park^2^, Jung-Won Park^1^, Jung-Won Park^2^, Jae-Hyun Lee^1^, Jae-Hyun Lee^2^

#### ^1^Yonsei University College of Medicine; ^2^Severance Hospital Regional Pharmacovigilance Centre, South Korea

##### **Correspondence:** Da Woon Sim – Yonsei University College of Medicine

**Background:** Recently monoclonal antibodies (mAbs) are widely used in various clinical fields. Although their safety was demonstrated prior to approval, targeted pharmacovigilance is essential for the recognition and assessment of adverse drug reactions (ADRs). The purpose of this study is to identify the frequency and major clinical features of ADRs referred to mAbs in Koreaa.

**Methods:** ADRs attributed to eighteen mAbs submitted spontaneously to the Korea Adverse Event Reporting System (KAERS) were extracted from the database for January 2000 to June 2014. We analyzed these reports for information related to patient’s characteristics and types or ADRs.

**Results:** A total of 17,346 ADRs were obtained from 8,591 patients. The most frequent symptoms of ADRs were abnormalities of leukocytes (9.0%), followed by infections (6.3%), drug eruptions (6.2%) and drug fevers (3.4%). Hypersensitivity reactions were reported almost ten percent. Furthermore mAbs induced total 5,545 serious ADRs from 2,862 patients, including severe infections (9.6%), neutropenia (9.3%), drug fever (4.1%) and visual dysfunctions (2.8%). 279 patients died due to ADRs of mAbs. The mAbs with the highest number of ADR reports were rituximab (20.3%), followed by adalimumab (16.7%), cetuximab (15.0%) and bevacizumab (13.9%).

**Conclusions:** Near ten percent of the ADRs were allergic-like, and no previously unrecognized ADRs were observed. Increased awareness among healthcare professionals is required to signal and prevent the consequences of adverse reactions caused by monoclonal antibodies.

## A212 Food Allergy with Eczema Is Associated with Reduced Growth in the First Four Years of Life

### Katrina Allen^1,2,3^, Cara Beck^1,3^, Jennifer Koplin^1,3^, Melanie Matheson^4^, Mimi Tang^1,3^, Anne-Louise Ponsonby^1,3^, Lyle Gurrin^3,4^, Shyamali Dharmage^3,4^, Melissa Wake^1,3^, Vicki Mcwilliam^1,3^

#### ^1^University of Melbourne, Royal Children’s Hospital, Australia; ^2^University of Manchester, UK; ^3^Murdoch Childrens Research Institute, Australia; ^4^University of Melbourne, Australia

##### **Correspondence:** Katrina Allen – University of Melbourne, Royal Children’s Hospital, Australia

**Background:** Food allergy has previously been associated with impaired growth in children, however this has not been investigated in a longitudinal study to investigate growth over time and previous studies have not accounted for the impact of co-existent eczema. We aimed to examine the association between IgE-mediated food allergy at 12 months of age and anthropometric measures at 1 and 4 years of age, and whether this association differed by eczema status.

**Methods:** The HealthNuts population-based cohort consists of 5300 children recruited at age 1 year. All infants underwent skin prick test to egg, peanut and sesame, and those sensitized had food challenges. At age 4 years, food challenges were repeated in those children previously identified as food allergic to determine persistence or resolution. Weight and height at 1 and 4 years were reported by parents from the child health record. Weight and height z-scores were determined from the World Health Organisation growth charts, standardised for age and sex. Multivariate linear regression models were fitted to examine the effect of food allergy and eczema at age 1 on weight and height z-scores at ages 1 and 4, adjusted for birthweight, prematurity, socioeconomic index, ethnicity and duration of breast feeding.

**Results:** Compared to children with no food allergy or eczema, children with both eczema and food allergy at age 1 had lower weight (β=-0.217, p<0.001) and height (β=-0.206, p=0.008) at age 1 and lower weight at age 4 (β=-0.159, p=0.026) after controlling for potential confounders. There was no difference in children with only food allergy or only eczema. At age 1 the height differences were greater in those with egg allergy and eczema (weight β=-0.240, p<0.001; height β=-0.215, p=0.009) than with peanut allergy and eczema (weight β=-0.292, p=0.011; height β=-0.145, p=0.281) compared to those with no eczema or food allergy. The differences continued at age 4 for children with egg allergy and eczema at age 1 (weight: β=-0.162, p=0.032 height: β=-0.120, p=0.234), particularly those who had persistent egg allergy and eczema at age 4 (weight β=-0.277, p=0.061; height β=-0.490, p=0.013). Peanut allergy, with or without eczema, at age 1 was not associated with differences in weight or height at age 4.

**Conclusions:** Children with IgE-mediated food allergy with eczema at age 1 have reduced growth parameters at age 1 and age 4, while eczema or food allergy alone was not associated with reduced growth. These results emphasise the need for adequate nutritional follow up in food allergic children in infancy, particularly those with eczema.

## A213 The Preliminary Study on Clinical Efficacy and Impact Factors of One Year’s Dust Mite Specific Immunotherapy in Allergic Asthma and Rhinitis Children Sensitized to Dust Mite

### Xiaoying Liu, Jing Wang, Li Xiang, Qun Wang

#### Beijing Children’s Hospital, China

##### **Correspondence:** Xiaoying Liu – Beijing Children’s Hospital, China

**Background:** To preliminary determine the impact factors on the clinical efficacy by periodically follow-up visiting dust mite allergic asthma and rhinitis children with one year treatment of dust mite specific immunotherapy.

**Methods:** All of 70 dust mite allergic asthma and rhinitis children with mild to moderate severity were enrolled (male 52, female 18, 4 to 14 years old) February to November in 2011. 57 patients took add-on therapy of sublingual dust mite specific immunotherapy (SLIT) while the other 13 patients with subcutaneous immunotherapy (SCIT). The patients were visited at the baseline period and followed up every three months to assess asthma control test (ACT), visual analog scale (VAS) of asthma and rhinitis symptom and spirometry for pulmonary function test. All the subjects were required to continuously record daily symptom and medication score (SMS) by diary card and to monitor morning and night peak expiratory flow value (PEFR) each day. The controller medication were adjusted to step up or down according to the control level. At the baseline period and after treatment for 6 months and 12 months, serum sIgE and sIgG4 to dermatophagoides pteronyssinus and dermatophagoides farinae, total IgE were determinated by enzyme-linked immunoassay with immunCAP system.

**Results:** (1)The analysis of clinical efficacy of dust mite specific immunotherapy in allergic asthma and rhinitis children. 54 patients completed treatment for 12 months. The average daily SMS was used as the evaluation index, the clinical response to SIT (effective cases) at 3, 6, 9 and 12 months after SIT were 72.2%, 75.9%, 81.5% and 87.0%, respectively. The ACT/C-ACT assessment, average daily SMS, VAS score, FEV1%pred, MMEF%pred, FEV1%pred, MMEF%pred were all improved than before treatment. (2)The analysis of the impact factors in the clinical efficacy of dust mite specific immunotherapy. The SMS at the baseline period, the asthma history and PEF%pred at the baseline period could affect the clinical efficacy of dust mite specific immunotherapy.

**Conclusions:** 87.0% of the patients showed effective response to SIT for one year. The patients with higher baseline SMS, shorter asthma history and lower PEF%pred responsed more effectively to SIT.

## A214 Lipopolysaccharide Signaling through Toll- like Receptor 4 Could be Augmented By Dermatophagoides Farinae in the Human Middle Ear Epithelial Cell

### Ji-Eun Lee^3^, Dong-Young Kim^1^, Chae-Seo Rhee^1^, Chae-Seo Rhee^2^

#### ^1^Seoul National University; ^2^Seoul National University Bundang Hospital; ^3^Chosun University, South Korea

##### **Correspondence:** Ji-Eun Lee – Chosun University, South Korea

**Background:** While otitis media with effusion (OME) is a well-known disease entity of a chronic inflammatory disease of the middle ear space characterized by the accumulation of fluid, but allergic otitis media is still not well- recognized. Previous investigations have suggested that the composition of the inflammatory substrate in the effusions of allergic otitis media is similar to the late-phase allergic response seen elsewhere in the respiratory tract, such as in asthma and in allergic rhinitis. In this study, we aimed to determine whether the prior treatment of Der f can effect on the inflammatory response induced by the subsequent LPS infection and which signaling pathway is involved.

**Method:** Primary human middle ear epithelial cells (HMEEC) were exposed to Der f crude body extract, LPS or both in different sequences, and the magnitude of each immunologic response produced by the HMEEC was compared. The mRNA expression level of mucin gene (MUC) 8, GM-CSF, TNF-a, TLR 4 and MD 2 were evaluated by using real-time polymerase chain reaction (qRT-PCR). The MUC proteins level before and after knocking out the TLR 4 and MD2 via siRNA transfection were assessed by Western blot analysis. Accordingly, the involved cell signaling pathway was evaluated by Western blot analysis and confocal microscopic image.

**Results:** The inflammatory response of cytokines (GM-CSF, TNF-a) and the expression of MUC 8 were augmented by the pretreatment of Der f followed by LPS, however, sequential treatment of HMEEC with LPS and Der f or adding together at the same time did not induce the same amount of response. The MUC expression was decreased by prior knockdown of TLR4 with siRNA but not by the MD2-siRNA. The MUC proteins level were increased by the pretreatment of Der f followed by LPS and decreased by the treatment of SB203580 (p38 inhibitor) and Bay (NF-kB inhibitor). The nuclear factor kB (NF-kB) translocation was demonstrated in the pretreatment of Der f followed by LPS condition.

**Conclusion:** Theses results suggest that Der f may act as a substitute for MD2 and make a strong augmentative response to the subsequent LPS infection. There was an increase in p38 and NF-kB activation within human middle epithelial cells, suggesting an important role for the development of OME in patients with concealed allergy airway sensitization.

## A215 Drug Allergy in Pregnant Adolescents: Relation with Familial and Personal Atopy, and Substances Use

### Francisco Vazquez-Nava

#### Department of Research, Faculty of Medicine, Tampico Mexico

**Abstract**

**Background**. Drug allergy and its negative effects, such as anaphylaxis, constitute an important health problem worldwide.

**Objective**. To determine the relationship between familial and personal antecedent of allergy, active smoking, and alcohol consumption with drug allergy in pregnant adolescents.

**Methods**. We conducted research on 785 pregnant adolescents by means of a cross-sectional study. Data collection was performed by using a self-administered questionnaire. We evaluated the difference of drug-allergy risk in 785 pregnant adolescents through familial and personal antecedent of allergy, active smoking, and alcohol consumption, calculating Odds ratios (OR) and 95% Confidence intervals (95% CI) by both uni- and multivariate regression analyses.

**Results**. Prevalence of drug allergy was 9.2% and of familial and personal antecedent of allergy, 8.7 and 21.0%, respectively. Percentage of active smoking was 17.6% and of alcohol consumption, 38.1%. Results of multivariate logistic regression analysis show that the familial history of atopic (Adjusted OR = 3.51; 95% CI = 1.85–6.29; p = 0.000), personal antecedent of allergy (Adjusted OR = 4.11; 95% CI = 2.48–6.81; p = 0.000), active smoking (Adjusted OR = 1.92; 95% CI = 1.10–3.35; p = 0.021), and alcohol consumption (Adjusted OR = 2.44; 95% CI = 1.48-4.06; p = 0.000), are significantly associated with drug allergy.

**Conclusion**. Familial and personal antecedent of allergy, active smoking, and alcohol consumption appear to be important factors for development of drug allergy in pregnant adolescents aged 13-19 years.

## A216 Patients and Physicians Concept of Well-Controlled Asthma: Findings from Realise Asia

### Sang-Heon Cho^6^, Jaewon Jeong^1^, Diahn-Warng Perng^2^, David Price^3^, Glenn Neira^4^, Jiangtao Lin^5^

#### ^1^Ilsan Paik Hospital, Inje University College of Medicine, South Korea; ^2^Department of Chest Medicine, Taipei Veterans General Hospital, Taiwan; ^3^Research in Real Life; ^4^Mundipharma Pte Ltd, Singapore; ^5^China-Japan Friendship Hospital, China; ^6^Seoul National University Hospital, South Korea

##### **Correspondence:** Sang-Heon Cho – Seoul National University Hospital, South Korea

**Background:** REALISE Asia is a two-part study (Part 1 - patient survey and Part 2 - physician survey) conducted to understand patients’ and physicians’ perceptions and perspectives towards asthma and its management. We report here the extent of discrepancy in the understanding of well-controlled asthma between patients and physicians in Asia.

**Methods:** REALISE Asia was conducted in two parts across the following 8 countries in Asia: China, Hong Kong SAR, Indonesia, Malaysia, Philippines, Singapore, South Korea, and Taiwan. Part 1 was an online, questionnaire based survey involving 2,467 patients with asthma aged 18-50 years. Part 2 was carried out through face-to-face and online interviews amongst 375 physicians managing asthma patients, 54% of which are specialists (i.e. respiratory medicine, allergy, and clinical immunology) and the rest in primary care practice.

**Results:** A significantly higher proportion of specialists (95%) compared to primary care physicians or PCPs (65%) reported that they use Global Initiative for Asthma (GINA) in assessing asthma control. Only 2% of specialists stated that they do not use any guidelines compared to 22% of PCPs. While 89% of patients considered their asthma to be controlled, physicians perceived 53% of their own patients as well-controlled. Both are overestimation of the actual proportion of patients achieving control (18%) based on GINA-defined criteria. Seven out of 10 physicians mentioned that their patients’ definition of well-controlled asthma is aligned with their definition. Physicians perceived that patients relate well-controlled asthma to minimal impact on daily life (50%), absence of symptoms (48%), and no/reduced attacks (21%). This is in stark contrast with patients understanding of the concept as they related control more to having medications to cope with their symptoms (26%) or quickly control asthma attacks (15%) reflective of the crisis-oriented mind-set they have towards their disease.

**Conclusions:** Physicians and patients overestimate their level of asthma control. These highlight the importance of accurate assessment in clinical practice, and the role of physicians in improving the understanding of concept of well-controlled asthma amongst their patients. Standardized tools may aid in better assessment of control while using shared language in discussing treatment goals may help ensure physicians’ and patients’ concept of well-controlled asthma are more aligned.

## A217 The Role of Vasoactive Intestinal Peptide in the Pathophysiology of Acute Asthma

### Olga Semernik

#### Rostov State Medical University Russia

**Background:** Previous research has shown neurohumoral factors to play an important role in the pathogenesis of asthma in children. The relationship between plasma levels of norepinephrine, vasoactive intestinal peptide and cortisol and clinical status and pulmonary function in patients with asthma during the period of exacerbation have been investigated.

**Method:** 30 children (aged 6 to 18 years) with asthma were examined in accordance with the purpose of the study. The control group consisted of 30 healthy volunteers of the appropriate age and gender. We compared clinical severity, spirometry, and neuroendocrine factors at asthmatic patients and healthy volunteers. We used multiple analyses of variance (repeated measures) to interpret the data. In addition, we used the Pearson Product Moment Test to investigate correlations among the different variables.

**Results:** The levels of vasoactive intestinal peptide, norepinephrine and cortisol were significantly higher in patients with asthma than in healthy volunteers (P < 0.001). It was found the concentration of VIP in serum at patients with asthma during the period of exacerbation to be 110.60 ± 11.89 nmol/l, that was significantly higher than the control group values - 53.26 ± 16.08 nmol/l (p = 0.016). In patients with asthma during the period of exacerbation, the level of vasoactive intestinal peptide correlated positively with the clinical severity rating (r = 0.621, P < 0.01) and negatively with FEV1 (r = -0.768, P < 0.001). In patients with mild attack of asthma concentration VIP in blood was 88.81 ± 31.14 nmol/l, with moderate - 100.27 ± 16.27 nmol/l, severe - 107.34 ± 22.70 nmol/l. In addition, the clinical severity rating showed a negative correlation with FEV1 (r = -0.359, P < 0.01). It can be assumed that the mobilization of the body defense at a child in the acute period of the disease in the form of additional ejection neurotransmitter and it can lead to a more rapid relief of obstruction airway.

**Conclusion:** It was found the vasoactive intestinal peptide to be the only neuroendocrine factor closely associated with clinical severity and pulmonary function suggesting this factor play an important role in the pathophysiology of acute asthma.

## A218 Comparison of Serum Cytokine Levels According to the Severity in Atopic Dermatitis

### Ha-Su Kim, Jin-a Jung, Ji-in Jung

#### Dong-a University Hospital, South Korea

##### **Correspondence:** Ha-Su Kim – Dong-a University Hospital, South Korea

**Purpose:** Atopic dermatitis(AD) is one of the most common allergic disease in Korean children. The aim of this study was to find the association between serum IL-10, Il-17, IL-4 levels according to the severity of atopic dermatitis in Korean children.

**Methods:** One hundred twenty-five AD children were assessed according to the SCORAD index. (mild< 15, moderate 15-40, severe > 40). Serum specific IgE to egg white, cow’s milk, soy bean, wheat, peanut and house dust mite(*Df*) was measured by ImmunoCAP system or skin prick test. Serum IL-4, IL-10, IL-17, INF-γ were measured by means of flow cytometry(Luminex-200®).

**Results:** Bronchial asthma and allergic rhinitis were combined more frequently in mild atopic dermatitis. However, there were no differences in allergic diseases of family. There was no difference in total IgE among 3 groups. However, serum total eosinophil count was highest in severe atopic dermatitis (*P*<0.05). Sensitizatioin rates to egg white, cow’s milk, soy bean, peanut and wheat were increasing significantly according to the SCORAD index (*P*<0.05). Sensitization rate to house dust mite was no significant difference according to the SCORAD index (*P*=0.55). Although there were no differences in serum IL-4, IL-17 and INF-γ levels among 3 groups, IL-10 was highest in severe atopic dermatitis (*P*<0.05).

**Conclusions:** We suggest that sensitization rates to common food allergens are higher in severe AD and serum IL-10 level is higher in severe AD.

## A219 The Different Influence on the Regulatory T Cell Response Between Subcutanous Immnuotherapy(SCIT) and Sublingual Immunotherapy(SLIT) in Children with Asthma

### Qing Miao, Li Xiang

#### Beijing Children’s Hospital, China

##### **Correspondence:** Qing Miao – Beijing Children’s Hospital, China

**Objective:** To compare the influence on the regulatory T cell (Treg) by different house dust mite specific immunotherpy route- subcutaneous immunotherapy(SCIT) versus sublingual immunotherapy(SLIT).

**Methods:** All of 86 dust mite allergic asthma children with mild to moderate severity were enrolled (male63,female23) in asthma center of Beijing Children’s Hospital from February in2012 to October in 2013. Among of them, 29 patients (male22, female 7) took sublingual dust mite specific immunotherapy (SLIT group),13 patients (male8,female 5) took subcutaneous immunotherapy (SCIT group), 14 subjects (male14, female 0) had completed the3-years treatment process (after SCIT treatment group), and the other 30 asthmatic children (male19, female11)without immunotherapy was enrolled as control group. Peripheral blood mononuclear cells (PBMC) were isolated from all the subjects and in vitro stimulated with HDM extracted (0μg/mL, 2.5 μg/mL, 5 μg/mL) for 48 hours, and then the relative percentage of Treg were measured by flow cytometry.

**Results:** In the unstimulation condition(Der p1=0μg/mL), the the baseline relative percentage of Treg in each group was significantly higher than that under stimulated condition. Besides, the baseline Treg% in SCIT group was significantly higher than that in the control group and in the SLIT group,however,there is no significant difference in the Treg% between SCIT group and those had accomplished the 3-years SCIT treatment.

**Conclusion:** It seemed that it is more likely and effectively to induce regulatory T cells in asthmatic children by SCIT when compared with SLIT, and this effect could remain for a period of time even after the whole specific treatment procedure.

## A220 Asthma State of Affairs in Asia: Seeing through Physicians’ and Patients’ Lenses

### Sang-Heon Cho^6^, Jaewon Jeong^1^, Diahn-Warng Perng^2^, Jiangtao Lin^3^, David Price^4^, Glenn Neira^5^

#### ^1^Ilsan Paik Hospital, Inje University College of Medicine, South Korea; ^2^Department of Chest Medicine, Taipei Veterans General Hospital, Taiwan; ^3^China-Japan Friendship Hospital, China; ^4^Research in Real Life; ^5^Mundipharma Pte Ltd, Singapore; ^6^Seoul National University Hospital, South Korea

##### **Correspondence:** Sang-Heon Cho – Seoul National University Hospital, South Korea

**Background:** Asthma remains a serious healthcare burden in Asia. Despite the availability of efficacious treatments, asthma control remains suboptimal. REALISE Asia aimed to identify treatment gaps by exploring patients’ perceptions and attitudes on their disease and its treatment, as well as insights from physicians managing this disease. While the main objective is to provide a broad view of asthma in the region, country-specific variations are reported here.

**Methods:** REALISE Asia was conducted in two-parts in the following countries: China, Hong Kong SAR, Indonesia, Malaysia, Philippines, Singapore, South Korea, and Taiwan. Part 1 was an online survey which involved 2,467 patients with asthma (aged 15-50 years, with access to social media). Part 2 was done through face-to-face and online interviews among 375 physicians involved in asthma patient-care.

**Results:** The disparity in the proportion of well-controlled asthma between patient-reported and physician-perceived ranged from 18% (Malaysia) to 44% (Hong Kong). A significantly higher proportion of physicians in Hong Kong (40%) reported that they do not use guidelines to assess asthma control (versus < 17% in other countries). Length of consultations ranged from 11.5 and 4.9 minutes for initial and follow-up in Korea, and 24.3 and 14.9 minutes in the Philippines, respectively. The percentage of patients who agreed they had full discussion with their physician about the best medication to treat their asthma ranged from ~60% (Korea) to over 80% (China, Indonesia, and Philippines). Almost 90% of physicians indicated they explain the purposes of inhalers on initial consultation, though this was significantly lower in China (62%). On average, 80% of physicians believed a combination (corticosteroid + long acting bronchodilator) inhaler can improve patient adherence, but this was significantly lower in Indonesia (53%). The proportion of patients who reported that they use controller inhaler daily ranged from 9% in Korea to 26% in Singapore. Only half of patients on inhalers said that their inhaler techniques were checked by a healthcare professional in the past 12 months, and this was lowest in Korea (42%) and Hong Kong (39%).

**Conclusions:** While variations among the countries in perceptions and management of asthma exist, there are common aspects of care that need to be optimised (e.g. adherence to controllers, inhaler techniques, patient education). Understanding these differences in the context of healthcare systems in these countries can provide additional insights which will be useful in the development of relevant interventions to improve asthma control.

## A221 Identification of Aspirin Exacerbated Respiratory Disease (AERD) Phenotypes Using Two Step Cluster Analysis

### Hyun Young Lee^1^, Hae-Sim Park^1,2^, Young Min Ye^2^, Su Chin Kim^1^

#### ^1^Ajou University Medical Center, South Korea; ^2^Ajou University School of Medicine

##### **Correspondence:** Hyun Young Lee – Ajou University Medical Center, South Korea

**Background**

Asthma is known a disease with many variations, and one of the subtypes is Aspirin Exacerbated Respiratory Disease (AERD). AERD patients experience severe bronchoconstriction to aspirin or other NSAIDs. AERD patients in general have relatively severe asthma with a high proportion requiring long-term oral corticosteroid (OCS). We aimed to classify AERD phenotype and its clusters.

**Method**

302 AERD patients who followed up at least 1 year in Ajou university hospital from Jan 1996 to Jul 2013 were enrolled. To fracture AERD phenotype using atopy, chronic rhinosinusitis (CRS) and urticaria, we performed two step cluster analysis. Severe exacerbation was defined as patients having taken intravenous steroid treatment or at least 2 burst of OCS in a year. The mean follow up duration was 7.2±5.2. Clinical characteristics among clusters were examined by Fisher’s exact test and ANOVA. The generalized estimating equation was applied for the possession rate of anti-asthmatics and severe exacerbation in a year.

**Results**

In the AERD, 4 clusters having good quality (0.7 silhouette) were obtained according to the urticarial, CRS and atopy: cluster 1 (AERD with CRS and atopy, and without urticaria), cluster 2 (AERD with only CRS), cluster 3 (AERD with only atopy) and cluster 4 (AERD with urticaria). There were significantly difference in severe asthma (27.3%, 14.8%, 19.7% and 7.7% in cluster 1, 2, 3 and 4, respectively, *p*=0.042), total IgE (564.3±732.5, 216.5±266.4, 352.0±398.7 and 345.5±440.7, *p*<0.001), sputum eosinophil (%)(31.5±37.8, 29.4±39.7, 13.4±27.3 and 18.7±30.9, *p*=0.024), and peripheral eosinophil count (485.0±374.8, 523.0±561.4, 349.3±379.3 and 245.1±242.5, *p*<0.001).

The occurrence of severe exacerbation was significant higher in cluster 1 than cluster 3 (OR: 2.121 (1.281-3.510), *p*=0.004). The patients of cluster 1 showed significantly higher possession rate of ICS+LABA than cluster 2, 3 and 4 (*p*<0.05), and of OCS than cluster 3 (*p*=0.010). The possession rate of LTRA was significantly higher in cluster 1 and 2 (vs. cluster 3 and 4, *p*<0.05). The possession rate of anti-histamines was significantly lower in cluster 3 (vs. 1,2 and 3, *p*<0.05).

**Conclusion**

Severe exacerbation rate and the possession rate of major anti-asthmatics were quite different among 4 clusters. Even AERD is recognized as a subtype of asthma, our results suggested that sophisticated therapeutic approach is needed for patients with AERD.

## A222 Dusty Air Pollution Are Associated with an Increase Risk of Allergic Diseases in General Population

### Shokrollah Farrokhi^1^, Mohammadkazem Gheiby^2^

#### ^1^Bushehr University of Medical Sciences, Iran; ^2^Department of Immunology, Asthma and Allergy

##### **Correspondence:** Shokrollah Farrokhi – Bushehr University of Medical Sciences, Iran

**Background.** Concerns have been raised about the adverse impact of dusty air pollution (DAP) in Iran on human health; but there is no study showing the effect of DAP on immune system toward allergic diseases.

**Methods.** The effects of ambient DAP exposures (based on PM_10_) on cytokine profiles and lymphocyte immunophenotypes in blood among 148 individuals of general population in hazardous (AQI >300) and good condition weather (AQI <50) was examined. We measured cytokine production (IL-4, IL-10, IL-13, IFN-γ) using ELISA as well as blood samples using a FACSort flow cytometer to determine phenotypes of T-lymphocytes (CD4+ and CD8+), CD19+ B-lymphocytes, CD25+ and CD4+ CD25+ cells.

**Results.** The mean serum level of IL-4 (33.4± 2.9 *vs* 0.85± 0.65 pg/dl) and IL-13 (15.1± 4.4 *vs* 0.12± 0.7 pg/dl) in subjects who exposure to ambient DAP were increased significantly than individuals in good condition weather (P= 0.001 for both). In addition, CD19+ B-lymphocytes (12.6± 4.9 *vs* 8.9± 3.2%) and CD4+ CD25+ cells (13.6± 4.6 *vs* 7.7± 3.8%) counts in peripheral blood were increased parallel with increased DAP exposure levels (P= 0.035 and P= 0.004, respectively).

**Conclusions.** The study may suggest ambient DAP may affect immune system shifting allergic inflammation in general population.

**Key words.** Dusty air pollution, immune system, allergic diseases, cytokine, lymphocyte

## A223 A Genome-Wide Association Study of Antituberculosis Drugs-Induced Hepatitis

### Sang-Heon Kim^4^, Heung Woo Park^1^, Sang-Hoon Kim^2^, Young-Koo Jee^3^

#### ^1^Seoul National University Hospital; ^2^Eulji University School of Medicine; ^3^Dankook University College of Medicine; ^4^Hanyang University College of Medicine, South Korea

##### **Correspondence:** Sang-Heon Kim – Hanyang University College of Medicine, South Korea

**Backgrounds:** Antituberculosis drugs (ATD) are the major causes of drug-induced liver injury (DILI). Although genetic factors are known to play a role in the development of ATD-induced hepatitis, the genetic susceptibility to this condition is poorly understood yet.

**Methods:** We performed a genome-wide association study (GWAS) to identify the genetic variants associated with the risk of ATD-induced hepatitis in a Korean population. DNA obtained from 40 patients with ATD-induced hepatitis and 119 ATD-tolerant subjects were genotyped on Affymetrix Genome-Wide Human SNP Array 6.0. After identification of genetic variants with significant associations, gene set enrichment analysis was done to find the relevant pathways associated with ATD-induced hepatitis.

**Results:** In the case-control analysis, we found significant genetic variants at *ID2* (rs345904, *P*=4.06 x 10^-7^, OR 8.03, 95% CI 3.34-19.35), *PLXNA4* (rs156978, *P*=8.48 x 10^-6^, OR 12.78, 95% CI 3.31-49.36) and *ZNF804B* (rs2718306, *P*=8.26 x 10^-6^, OR 10.72, 95% CI 3.18-36.11). Pathway analysis identified several pertinent pathways including axon guidance, cGMP effects, developmental biology, signaling by Rho GTPases and interaction between L1 and ankyris.

**Conclusions:** This GWAS identified genetic variants and pathways underlying the development of ATD-induced hepatitis. These results provides insights into the genetic susceptibility to ATD-induce hepatitis.

## A224 The Role of peroxiredoxin6 of Bronchial Epithelial Cells in Regulating Mitochondrial Function Under Oxidative Stress By Translocation to Outside Mitochondrial Membrane

### Sunjoo Park^1^, Keun Ai Moon^1^, Hyouk-Soo Kwon^2^, Tae-Bum Kim^2^, You Sook Cho^2^, Hee-Bom Moon^2^, Kyoung Young Lee^1^, Gyong Hwa Hong^1^, Eun Hee Ha^1^

#### ^1^Asan Institute for Life Sciences, South Korea; ^2^Asan Medical Center, University of Ulsan College of Medicine

##### **Correspondence:** Sunjoo Park – Asan Institute for Life Sciences, South Korea

Oxidative stress plays a role in the pathogenesis of chronic obstructive airway diseases such as asthma and COPD. Thus, the regulation of both reactive oxygen species (ROS) and anti-oxidant defense is also critical. In general, major source of ROS is mitochondrial respiration of cells and some of the antioxidants exist as a mitochondria-specific form which is important for regulating the significant quantities of ROS. The peroxiredoxin6 (PRDX6), known as a dual function protein with GSH peroxidase and phospholipase A2 activities, is reported to be predominantly expressed in airway epithelium. Recently, PRDX6 knockdown was reported to induce mitochondrial dysfunction although it is well known that PRDX6 is mainly located in cytoplasm, secretory organelles, and lysosomes. The aim of this study was to investigate if PRDX6 have an influence on mitochondrial function through its cellular trans-localization to mitochondria under oxidative stress. Mitochondrial translocation from subcellular fractionation of human bronchial epithelial cell line and mouse lung tissue under oxidative stress were analyzed by immunoblotting. We evaluated changed mitochondrial function by measuring O_2_ consumption, ATP synthesis, and ROS generation. PRDX6 expression was increased in oxidative stress inducer (paraquat and H_2_O_2_). The lipid peroxidation and protein carbonylation were significantly higher in PRDX6 knock-down cells compared to PRDX6 overexpressed cells. The intracellular trans-localization of PRDX6 under enhanced oxidative stress was confirmed by immunoblotting of subcellular fractionation and confocal microscopic analysis of immunofluorescence probes. The results of this study demonstrated that PRDX6 plays an important role in regulating mitochondria under oxidative stress in the airway. Dysregulation of PRDX6 function might be critical in the development and perpetuation of chronic obstructive airway disease. Clarification of the precise functioning mechanism of PRDX6 may be valuable to understand pathogenesis of the disease.

## A225 Toxic and Adjuvant Effects of 3 Types of Silica Nanoparticles on Airway System

### Heejae Han^1^, Hye Jung Park^1^, Yoon Hee Park^1^, Yoon-Jo Kim^1^, Kangtaek Lee^2^, Jung-Won Park^1^, Jae-Hyun Lee^1^

#### ^1^Yonsei University College of Medicine, South Korea; ^2^Yonsei University

##### **Correspondence:** Heejae Han – Yonsei University College of Medicine, South Korea

**Background:** Silica nanoparticles (SNPs) can be easily exposed via inhalation owing to their low particle weight and ease of dispersion. However, toxic and adjuvant effects of SNPs on the airway system according to their surface pattern are not well established. We evaluated these effects on the airway system in a murine model using 3 types of SNPs.

**Methods:** Female, 6-week-old, BALB/c mice were intranasally administered 3 types of SNPs (spherical-type [S-SNPs], mesoporous-type [M-SNPs], and polyethylene glycol conjugated type [P-SNPs]) with or without ovalbumin (OVA), thrice weekly for 2 weeks. All mice were sacrificed 48 h after the last dose. We evaluated airway hyper-responsiveness (AHR), bronchoalveolar lavage fluid (BALF), cytokine levels, and histology of the lungs.

**Results:** In the model administered SNPs alone, only M-SNPs induced significant AHR as compared to sham group, whereas all SNP-treated groups showed a significant increase in total cell, macrophage, and neutrophil counts in BALF. S-SNPs and M-SNPs induced an increase in cytokine (interleukin [IL]-5, IL-1β, and interferon-γ) levels. In the model administered SNPs with OVA, S-SNPs and M-SNPs induced significant AHR as compared to sham group and those administered OVA alone. Overall, greater inflammatory cell infiltration in BALF, extensive pathological changes, and higher cytokine levels (IL-5, IL-13, IL-1β, and IFN-γ) were observed in the group administered SNPs with OVA than those administered SNPs or OVA alone. However, P-SNPs induced lesser inflammation than the other types of SNPs in both models.

**Conclusion:** SNPs alone has significant toxic effectc on the airway system. Moreover, SNPs when administered with OVA cause adjuvant effects. We recommend the use of P-SNP because of its relative safety compared with S-SNP and M-SNP.

## A226 Procedure for Diagnostic and Selection of Immunotherapy Method for Children with Different Immunopathogenetic Phenotypes of Atopic Dermatitis

### Tatiana Slavyanskaya^1,2,3^, Vladislava Derkach^1,2^

#### ^1^People’s Friendship University of Russia; ^2^Institute of Immunophysiology, Russia; ^3^Pacific State Medical University

##### **Correspondence:** Tatiana Slavyanskaya – People’s Friendship University of Russia

**Objective of study:** To develop the procedure for assessment, diagnostic and treatment of children (Ch) with different immunopathogenetic phenotypes (IPP) of atopic dermatitis (AD).

**Materials and methods:** The participants of study were 94 Ch aged 5 to 17 with moderate course of AD in the exacerbation phase. The control group was comprised of 30 healthy Ch of the same age. The patients underwent general physical examination, clinical assessment and immunoallergological assessment (IAA) aimed at detection of clinical and laboratory signs of allergen-induced inflammation. General physical examination included clinical blood and urine analyses and screening for parasitic and viral infections. IAA consisted of: immunogram determination of levels of pro- and antiinflammatory cytokines (by fluorescence immunoassay); skin testing, challenge or elimination testing (if indicated), total and specific IgE blood testing. House dust mite allergens (HDMA) were used as a cause-significant allergen. Clinical assessment was aimed at collection of allergic anamnesis and evaluation of severity of clinical symptoms according to SCORAD scale.

**Results:** The integrated study conducted enabled us to divide Ch into two groups based on their principal IPGP of AD: IgE-mediated and non-IgE-mediated form of AD. Ch with non-IgE-mediated type of AD showed no IgE-sensitization to HDMA and lower parameters of macrophage-phagocytic component of immune system (MPCIS). IgE-mediated AD phenotype included 3 forms of AD – allergic form, mixed form (in combination with allergic rhinitis, bronchial asthma) and combined form. In studying allergic and mixed form of AD we proved the existence of sensitization to non-eliminated HDMA, which promote allergization and provoke development of symptoms in the course of AD, and in Ch with combined form of AD a decrease in parameters of MPCIS (such as phagocytic number, phagocytic index, NBT Reduction Test) was also found. On the basis of the revealed disorders, several multimodality immunotherapy (MIT) programs were developed. For IgE-mediated forms of AD we proposed a MIT including basic therapy (BT) + parenteral antigen-specific immunotherapy (ASIT) as an accelerated treatment regimen and immunomodulator (IM) selected on the basis of its capacity to affect the cells of monocytic-macrophagal nature, increase of macrophages’ cytotoxicity toward bacterial antigens and viruliferous cells, as well as correction of imbalance in cytokine profile Th1/Th2 and intensifying the production of IFN-y, which would eventually contribute to lower rate of infectious complications of AD. For non-allergic form of AD, the BT + IM were used.

**Conclusion.** The procedure of assessment and diagnostic of Ch with AD developed by us provides an opportunity to correctly select an adequate MIT in accordance with IPP of the disease taking into account the revealed immune disorders.

## A227 Prediction of the Success of Our Desensitization Protocol with Symptoms and Results of a Skin Prick Test in Patients with Hypersensitivity to Platinum-Based Chemotherapy

### Hye Jung Park^1,2^, Chein-Soo Hong^2^, Jae-Hyun Lee^1,2^, Sungryeol Kim^1,2^, Kyung Hee Park^1,2^, Choong-Kun Lee^2^, Beodeul Kang^2^, Seung-Hoon Beom^2^, Sang Joon Shin^2^, Minku Jung^2^, Jung-Won Park^1,2^

#### ^1^Severance Hospital Regional Pharmacovigilance Centre; ^2^Yonsei University College of Medicine, South Korea

##### **Correspondence:** Hye Jung Park – Severance Hospital Regional Pharmacovigilance Centre

**Background:** The prevalence of immediate hypersensitivity reaction (IHSR) to platinum-based chemotherapy has been rising, with the increase of chemotherapy usage. Although there is a proven ultimate solution, desensitization protocol, it has not yet applied in many institutes because of impracticalities such as cost, procedure duration and lack of trained manpower. Prediction of the success of a desensitization protocol will help reduce unnecessary workload.

**Objective:** We aimed to determine the clinical characteristics and currently adopted measures against oxaliplatin IHSR and to predict the success of our newly developed practical desensitization protocol.

**Methods:** We retrospectively reviewed 2,640 cases of oxaliplatin IHSR in 271 oxaliplatin users who admitted to Severance hospital. We prospectively used our new desensitization protocol 31 times in 12 patients with hypersensitivity to platinum-based chemotherapy. The new desensitization protocol was conducted with escalating of infusion rate regularly regardless of the concentration of bags, in order from 60 mL/h to 120 mL/h and 240 mL/h about every 15 minutes.

**Results:** In 271 patients who administered oxaliplatin, 45 patients (16.6%) experienced oxaliplatin IHSR. In 39 patients, who experienced IHSR but need to keep oxaliplatin, 6 patients (15.4%) stopped due to the IHSR and 33 patients (84.6%) kept the regimen regardless of the anticipated risk. Any significant risk factor for the IHSR was not found. The new desensitization protocol was successfully completed in 12 patients (100%). However, among these 12 patients, the protocol was ineffective in 3 patients having fever without urticaria and a negative response in a skin prick test.

**Conclusions:** Many patients who experience oxaliplatin IHSR are required to stop the effective regimen or maintain the regimen without desensitization with the anticipated risk of IHSR. Our new practical desensitization protocol might be applied easily and conveniently in real clinical practice. Fever without urticaria and a negative response in a skin prick test indicate that this desensitization protocol might be ineffective.

## A228 Anti-Allergic Effect of Intralymphatic Injection of OVA-Flagelin Mixture in Mouse Model of Allergic Rhinitis

### Eun-Hee Kim, Ji-Hye Kim, Ji-Hun Mo, Young-Jun Chung

#### Dankook University, South Korea

##### **Correspondence:** Eun-Hee Kim – Dankook University, South Korea

**Background:** Bacterial flagellin, which is a Toll-like receptor5 agonist, is used as an adjuvant for immunomodulation. Recently, intranasal administration of OVA flagellin mixture was reported to be effective in allergic inflammation. In this study, we aimed to evaluate the effect and its mechanism of intralymphatic administration of OVA-flagellin mixture in the treatment of allergic rhinitis.

**Materials and Methods:** BALB/c mice was sensitized with OVA and treated with OVA-flagellin (FlaB) mixture via intranasal, sublingual and intralymphatic routre to evaluate the effect of intralymphatic administration. Then several parameters of allergic inflammation was assessed including symptom score, eosinophil and neutrophil infiltration in the nasal mucosa, systemic cytokine levels, and Total and OVA-specific imuunoglobulin E. To evaluate the mechanism of intralymphatic injection, local cytokine, chemokine, and innate cytokine analysis was undertaken using real time PCR, western blot and immunohistochemistry.

**Results:** Intralymphatic injection by OVA-FlaB mixture reduced symptom score, eosinophil infiltration in the nasal mucosa and total and OVA-specific IgE levels more significantly than intranasal and sublingual administration. The systemic cytokines(IL-4, IL-5, IL-6, IL-17 and IFN-r) and local cytokines(IL-4, IL-5) production were also decreased significantly in intralymphatic injection by OVA-FlaB. Double administration of mixture was more effective than single administration. Moerover, the expression of innate cytokine such as IL-25, IL-33 in nasal epithelial cells were decreased and the chemokine expression such as CCL24(eotaxin-2) and CXCL1,2 were decreased in the nasal mucosa, suggesting the underlying mechanism of intralymphitc administration of OVA+FlaB mixture.

**Conclusion:** Intralymphatic administration of OVA+FlaB mixture was more effective in alleviating allergic inflammation than intranasal and sublingual administration in mouse model of allergic rhinitis and this effect could be attributed to reduced expression of innate cytokine and chemokines. This modality could be considered as a new therapeutic method and agent.

## A229 Serum Periostin Level Is Higher in Respiratory Type of NSAID Hypersensitivity Than Cutaneous Type

### Mi-Ae Kim^3^, Hae-Sim Park^1^, Moon Gyeong Yoon^1^, Young-Soo Lee^2^, Ji Hye Kim^1^, Ga Young Ban^1^, Hye-Soo Yoo^1^, Yoo Seob Shin^1^, Young Min Ye^1^, Dong-Ho Nahm^1^

#### ^1^Ajou University School of Medicine; ^2^Department of Allergy and Clinical Immunology, Ajou University School of Medicine; ^3^Bundang Medical Center, CHA University, South Korea

##### **Correspondence:** Mi-Ae Kim – Bundang Medical Center, CHA University, South Korea

**Background**

Nonsteroidal antiinflammatory drugs (NSAIDs) hypersensitivity is a commonly found drug allergy, in which two major phenotypes, respiratory (aspirin-exacerbated respiratory disease <AERD>) and cutaneous (aspirin-exacerbated cutaneous disease <AECD> or aspirin-intolerant acute urticaria <AIAU>) types were noted in this country. Periostin is an extracellular matrix protein and structurally homologous with fasciclin I, an insect adhesion molecule. Previous study demonstrated that serum periostin level was significantly higher in AERD than in aspirin tolerant asthma. To evaluate serum periostin level as a biomarker for differentiating the phenotypes of NSAIDs hypersensitivity, we compared serum periostin levels between respiratory and cutaneous types of NSAID hypersensitivity.

**Methods**

Serum periostin levels were measured by human periostin ELISA in sera from 326 adult patients with NSAID hypersensitivity and 87 healthy normal controls (NC). The phenotype of NSAID hypersensitivity was defined according to previous histories of adverse drug reaction and/or aspirin provocation test.

**Results**

There were 45.7% of respiratory type of NSAID hypersensitivity (n=149) and 54.3% of cutaneous type (n=177). Mean serum periostin level was significantly higher in respiratory type (82.6 ± 38.8 ng/mL) than in cutaneous type (39.7 ± 31.1 ng/ML) and NC group (46.2 ± 29.0 ng/mL). However, there were no significant differences of serum periostin levels between AECD and AIAU groups (*P* = 0.708), between AECD and NC groups (*P* = 0.195), and between AIAU and NC groups (*P* = 0.110). The ROC analysis revealed that serum periostin level could differentiate AERD from cutaneous type of NSAIDs hypersensitivity (AUC = 0.826, *P* < 0.001) and cut-off level was 42.5 ng/mL with 93.3% of sensitivity and 61.0% of specificity.

**Conclusion**

These findings suggest that serum periostin level can be a useful biomarker for predicting the phenotype of AERD among NSAID hypersensitivity patients.

## A230 A Retrospective Analysis of Allergy Blood Testing in Beijing Children’s Hospital in the Year of 2013: A Single-Center Research

### Qing Miao, Li Xiang

#### Beijing Children’s Hospital, China

##### **Correspondence:** Qing Miao – Beijing Children’s Hospital, China

**Background:** An early identification of sensitization pattern in suspected allergic children may supply evidence–based clues for prevention and management of allergic disease.

**Objective:** To analyze the sensitization status to common inhalant allergens for children with suspected allergies in Beijing Children’s Hospital in year of 2013.

**Methods:** The enrolled children were screened for allergies through a semi-quantitative testing method (Mediswiss Allergy Screen allergen testing system, Germany), and a further comparison for sensitization patterns among different subgroups was performed.

**Results and conclusion:** A total of 21,134 children had screened for allergies. In out-patient subjects, the positive rate of sIgE was 45.99% (8266/17974 cases), the top three positive allergens were mold, dog hair and dust mites. For the inpatient group, the positive rate of sIgE was 29.34% (927/3160 cases) and the top three allergens were dust mites, dog hair, and mold. It was also found that the highest positive rate was from children suffering from the nose or eye disease (out-patient:2092/3126,66.92%; in-patient:14/26,53.85%). Especially for the inpatients HSP children, when co-infected with lower respiratory tract infections(LRI), the positive rate was increased (HSP: 370/1024, 36.1%; HSP+LRI: 370/1024, 42.7%, *P*<0.05), and it was more likely to lead to the appearance the multiple sensitization. Therefore, it is necessary to keep a watch on the variation in the sensitization state, and to pay greater attention on interpreting testing results, which enables to supply the pediatrician a rational treatment and prophylactic strategy on the allergen avoidance.

## A231 Role of Nrf2 in the Allergic Airway Inflammation Differ Between BALB/c and C57BL/6 Mice

### Ying-Ji Li^1^, Takako Shimizu^1^, Hirofumi Inagaki^1^, Yukiyo Hirata^1^, Hajime Takizawa^2^, Arata Azuma^1^, Masayuki Yamamoto^3^, Tomoyuki Kawada^1^

#### ^1^Nippon Medical School, Japan; ^2^Kyorin University Hospital; ^3^Tohoku University Graduate School of Medicine

##### **Correspondence:** Ying-Ji Li – Nippon Medical School, Japan

Oxidative stress has been postulated to play an important role in the pathogenesis of allergic airway inflammation. Nrf2 is involved in the transcriptional regulation of many antioxidant genes. In the present study, we investigated the role of Nrf2 on an experimental model of Ovalbumin (OVA) - sensitized and challenged allergic airway inflammation in both of BALB/c and C57BL/6 mice.

*Nrf2*−/− and *Nrf2*+/+ mice were used in both of BALB/c and C57BL/6 mice. Allergic airway inflammation was generated in the mice by intraperitoneal sensitization with OVA/alum on days 0, 6 and 7. The mice were challenged with OVA intranasally on day 21. After OVA challenge twenty-four hours, we examined the cell populations in bronchoalveolar lavage (BAL) fluid, and the IgE levels in the serum. Airway hyperresponsiveness (AHR) was assessed by whole-body plethysmography with a free-moving application.

The number of total cells, macrophages and eosinophils in BAL fluid was decreased in *Nrf2*-/- compared with *Nrf2*+/+ mice, this was also the case for the AHR changes in both of BALB/c and C57BL/6 mice. The number of neutrophils in BAL fluid and the IgE levels in the serum were significantly increased in *Nrf2*-/- compared with *Nrf2*+/+ in BALB/c mice; in contrast, there was no significantly different between *Nrf2-*/- and *Nrf2*+/+ in C57BL/6 mice.

In conclusion, the role of Nrf2 in the generation of allergic airway inflammation differs markedly between mouse strains. Our results suggest that Nrf2 may play a key role in the development of allergic airway inflammation related to neutrophils in BALB/c mice.

## A232 Effect of Human Mesenchymal Stem Cell on Neutrophilic Asthma Model

### Min-Gu Kim^2^, Gyong Hwa Hong^1^, Kyoung Young Lee^1^, Eun Hee Ha^1^, Keun Ai Moon^1^, Sunjoo Park^1^, Hyouk-Soo Kwon^2^, Tae-Bum Kim^2^, Hee-Bom Moon^2^, You Sook Cho^2^, Jung-Hyun Kim^2^, Hyo-Jung Kim^2^, So-Young Park^2^, Bomi Seo^2^

#### ^1^Asan Institute for Life Sciences; ^2^Asan Medical Center, University of Ulsan College of Medicine, South Korea

##### **Correspondence:** Min-Gu Kim – Asan Medical Center, University of Ulsan College of Medicine, South Korea

**Background:** Neutrophils play an important role in the development of persistent airflow limitation in asthma, particularly in patients who show poor response to corticosteroids. Human mesenchymal stem cells (hMSCs) has emerged as a new treatment option due to its immunomodulatory effect, however, the role of hMSCs in neutrophilic asthma is not yet understood.

**Method:** BALB/c mice were exposed to ovalbumin and poly IC to induce neutrophilic airway inflammation. hMSC was administered intravenously, and then, bronchoalveolarlavage (BAL) was done to evaluate differential cell count and measure inflammatory cytokine including IL-8, IL-5, IL-10, IFN-gamma. We also measure cytokines include IL-17, IL-4, IL-10, IFN-gamma released from lymphocyte in pulmonary lymph node. In addition, lung histopathology was done to show peribronchial and perivascular inflammation.

**Result:** hMSC treatment decreased BAL total cell count (P < 0.001), neutrophils (P < 0.001) and lymphocyte (P < 0.01) IL-5 in BAL fluid was decreased in hMSC treatment group (P < 0.01), however, IL-8, IL-10 and IFN-gamma showed no differences compared with wild type. IL-17 released from lymphocyte in pulmonary lymph node was significantly decreased (P < 0.05). Histopathologic examination revealed that peribronchial inflammation was dramatically decreased in hMSC treatment group.

**Conclusion:** hMSCs inhibited airway inflammation in poly IC induced neutrophilic asthma model. The mechanism underlying its immunomodulatory effect might be associated with down regulation of IL-17 which has key role in Th17 mediated inflammation.

## A233 Immunomodulatory Effect of Tonsil Derived Mesenchymal Stem Cells in a Mouse Model of Allergic Rhinitis

### Ji-Hye Kim, Ramachandran Samivel, Eun-Hee Kim, Young-Jun Chung, Ji-Hun Mo

#### Dankook University, South Korea

##### **Correspondence:** Ji-Hye Kim – Dankook University, South Korea

**Background:** Although several studies have claimed that mesenchymal stem cells (MSCs) derived from human tissues can ameliorate allergic airway inflammation, the immunomodulatory mechanism of MSCs remains unclear.

**Objective:** We aimed to determine the effects and underlying mechanism of tonsil derived MSCs (T-MSC) on allergic inflammation as compared to adipose tissue derived stem cells (ASCs) in mouse model of allergic rhinitis (AR).

**Methods:** MSCs were isolated from human palatine tonsil (T-MSC) and adipose tissues (ASC), and the surface markers were analyzed. The effect of T-MSCs was evaluated in 24 BALB/c mice that were randomly divided into 4 groups (negative control group; positive control group; T-MSC group and ASC group). MSCs were administered intravenously to OVA-sensitized mice (T-MSC and ASC groups) on days 18 to 23 and subsequent OVA challenge was conducted daily from days 24 to 28. Several parameters of allergic inflammation were assessed.

**Results:** T-MSC and ASC had similar characteristics in surface markers. Intravenous injection of T-MSC significantly reduced allergic symptoms, eosinophil infiltration, serum total and OVA specific-IgE and the nasal and systemic Th2 cytokine profile. Further analysis revealed that nasal innate cytokines such as IL-25 and IL-33, and chemokines such as CCL11, CCL24 induction were suppressed in T-MSCs injected groups, explaining their underlying mechanism. Additionally, the T-MSC group had more inhibition of allergic inflammation than the ASC group, which might be attributed to the more proliferative activity of T-MSC.

**Conclusion:** Administration of T-MSC effectively reduced allergic symptoms and inflammatory parameters in the mouse model of AR. T-MSC treatment reduced Th2 cytokines and OVA specific IgE secretion from B cells. In addition, innate cytokine (interleukin-25 and inteleukin-33) expression and eotaxin mRNA expression was inhibited in the nasal mucosa, suggestive of the mechanism of reduced allergic inflammation. Therefore, T-MSC treatment is potentially an alternative therapeutic modality in AR.

## A234 Alternative Therapy Such As Yoga May be a Low Cost Tool for Improving the Quality of Life of Patient’s with Allergic Rhinitis and Asthma

### Soumya M.S.^1^, G Inbaraj^1^, R Chellaa^1^, Ruby Pawankar^2^

#### ^1^Department of ENT, St Johns Medical College Hospital, Bangalore, India; ^2^Department of Pediatrics, Nippon Medical School, Tokyo, Japan

##### **Correspondence:** Soumya M.S. – Department of ENT, St Johns Medical College Hospital, Bangalore, India

**Objective:** Allergic rhinitis (AR) and asthma are a major global health problem in developed and developing countries like India. These diseases have an adverse impact on the patient’s quality of life (QOL) and also pose a big socio-economic burden. Yoga has shown to have some beneficial effect on improving the sleep quality, lung function and quality of life in patients with asthma. But there is no scientific study on the effect of Yoga on airway resistance in patients with AR. The aim of this study was to assess the subjective and objective effects of yoga and also to see if Yoga can be adapted as a cost cutting intervention for better control of asthma and AR.

**Methods:** The present study was conducted on 31 adult subjects with mild-moderate persistent AR with or without asthma. These patients were trained in specific Hatha yogasanas which are known to improve respiratory functions. The participants practised these asanas for 12 weeks. The subjective and objective outcome measures were assessed at baseline (Day 0) and at 12 weeks post yoga, using Rhinomanometry, Spirometry, Sino Nasal Outcome Test questionnaire and QOL Short Form 12.

**Results:** There was a significant reduction in the Total Nasal Resistance (TNR) at 150 Pa/ml/s (p<0.001) and a significant increase in Total Nasal Airflow at 150 Pa/ml/s (p<0.01) after yoga as compared to the corresponding baseline values at day 0. Indices of pulmonary function such as FVC (p<0.001), FEV1 (p<0.05), Forced mid-expiratory flow at 75% of FVC (FEF 75%) (p<0.05) and PEFR (p<0.01) showed significant improvement. QOL questionnaire, Short Form 12 showed a highly significant improvement in both physical (p<0.001) and mental (p<0.01) composite score along with significant reduction in the Sino Nasal Outcome Test score (p<0.001) post yoga as compared to the corresponding baseline values.

**Conclusions:** The direct cost of treatment such as medications and hospital visits etc., as well as the indirect cost due to loss of productivity is significantly high in patients with AR and asthma. The results of this present study conclude that the practice of yoga offers a significant advantage in patients with AR by reducing their nasal resistance, increasing nasal airflow, improving lung functions and their quality of life. Further studies are needed to analyze the immunologcal mechanisms involved in this form of therapy, the impact on acute exacerbations, the need for rescue medication and the long term effects of yoga. A true patient-physician partnership further empowers the patients compliance and adherence, thereby their function and health.

## A235 Substantial Impairment of the Quality of Life in Adult Patients with Chronic Urticaria

### Wonsun Choi, Hae-Sim Park, Young Min Ye, Ji Hye Kim, Ga Young Ban, Yoo-Seob Shin

#### Ajou University School of Medicine

##### **Correspondence:** Wonsun Choi – Ajou University School of Medicine, South Korea

**Background and Objectives:** Chronic urticaria (CU) is a common skin disorder characterized by hives and itching for at least 6 weeks. The QOL in CU can be substantially impaired due to its unpredictable symptoms and long-term nature. This study was aimed to evaluate the impact of CU on QOL by using the CU-specific QOL measurements, previously validated in Korea, and to identify the predictors of QOL in CU patients.

**Methods:** We enrolled 390 adult patients with chronic spontaneous urticaria who were followed in the Allergy and Clinical Immunology Clinic in Ajou university hospital from March 2009 to December 2012. The CU-QOL questionnaires, urticaria activity score (UAS), the presence of angioedema, and serum total IgE levels were investigated.

**Results:** The average CU-QOL scores was 70.6 of 100 points. The CU-QOL scores significantly correlated with the UAS, particularly with our 15-point UAS (UAS15, coefficient -0.532, *P* <0.01) than the 6-point UAS (-0.502, *P*<0.01). Total CU-QOL scores significantly decreased in patient with severe CU (UAS15 score≥13) than non-severe CU (52.3 vs 72.1, P <0.001). In cases having angioedema, the urticaria symptom domain scores significantly decreased (37.4 vs 46.9, P = 0.004) than those with urticaria only. Multivariate analysis showed that severe CU, high log[total IgE], and the presence of angioedema were significant predictors of CU-QOL impairment (<85 points).

**Conclusions:** It is important to consider QOL impairment and severe CU, log[total IgE], and the presence of angioedema are significant CU-QOL predictors in Korean patient with CU.

## A236 Dietary Galacto-Oligosaccharides Reduce Airway Eosinophilia and Enhance the Th2 Suppressive Effect of Budesonide in House Dust Mite-Induced Asthma in Mice

### Saskia Braber, Kim Verheijden, Aletta Kraneveld, Johan Garssen, Linette Willemsen, Gert Folkerts

#### Utrecht University, Netherlands

##### **Correspondence:** Saskia Braber – Utrecht University, Netherlands

**Background:** In house dust mite (HDM) allergic asthma, symptoms occur due to airway eosinophilia and Th2 cell activation. Budesonide is used to treat airway inflammation and hyper-responsiveness. We showed that dietary non-digestible galacto-oligosaccharides (GOS) suppress symptoms in a murine model for HDM-induced asthma. The aim is to study combined dietary GOS and budesonide treatment on allergic asthma in mice.

**Methods:** BALB/c mice were intranasally (i.n.) sensitized with PBS in presence or absence of 1μg HDM and challenged i.n. with PBS or 10μg HDM on days 7 till 11 while being fed a diet containing 0, 1 or 2.5 w/w% GOS. On day 7, 9, 11, and 13 budesonide was either or not instilled *oropharyngeally*. On day 14, airway resistance to metacholine and inflammation were determined. Leukocyte subtypes were analyzed in the broncho-alveolar lavage (BAL) and in lung cell suspensions. Mucosal mast cell protease-1 (mmcp-1) was measured in serum and cytokines in lung homogenates.

**Results:** HDM-allergy significantly increased airway responsiveness and BAL leukocyte numbers. Budesonide treatment suppressed this, which reached significance in mice fed GOS. Budesonide reduced the number of lymphocytes and eosinophils in the BAL. Feeding GOS in absence of budesonide treatment reduced the number of eosinophils as well. In addition, both GOS as well as budesonide reduced mmcp-1 serum concentrations. Interestingly, only in the GOS fed mice, budesonide treatment reduced IL-33 and IL-13 concentrations and the frequency of Th2 cells in the lung.

**Conclusions:** Dietary intervention using GOS may be a novel way to improve effectiveness of anti-inflammatory drug therapy in asthma.

This study was performed within the framework of Carbohydrate Competence Center (WP25).

## A237 Production and Characterization of Recombinant Periplaneta Americana Allergens for Component Resolved Diagnosis

### Stephanie Eichhorn^1^, Fatima Ferreira^1^, Isabel Pablos^1^, Bianca Kastner^1^, Bettina Schweidler^1^, Sabrina Wildner^2^, Peter Briza^1^, Jung-Won Park^3^, Naveen Arora^4^, Stefan Vieths^5^, Gabriele Gadermaier^1^

#### ^1^University of Salzburg, Austria; ^2^Christian Doppler Laboratory for Innovative Tools for the Characterization of Biosimilars, Austria; ^3^Division of Allergy and Immunology, Department of Internal Medicine, Yonsei University College of Medicine, South Korea; ^4^Csir-Institute of Genomic and Integrative Biology, India; ^5^Paul-Ehrlich-Institut, Germany

##### **Correspondence:** Stephanie Eichhorn – University of Salzburg, Austria

**Background**

American cockroach (*Periplaneta americana*) is a major source of indoor allergens in tropic and sub-tropical regions, frequently causes allergic reactions and asthma. Since no dominant major allergen exists in this source, component resolved diagnosis (CRD) is required to determine patients’ sensitization and pointing out treatment options. Therefore, the ERA-Net New Indigo project GENALL (Genetically engineered allergens for component-resolved diagnosis and immunotherapy of airway allergies) aimed to produce a set of well-characterized recombinant *Periplaneta americana* allergens to establish CRD.

**Methods**

Per a 1.0103 (residues 197 – 378) a microvillar membrane associated protein homologue, Per a 2.0101 an unusual aspartic protease, and the C-terminal domain of Per a 3.0101 (residues 426 - 675), a hemocyanin, were produced recombinantly in *E.coli*. The primary sequence of purified proteins was confirmed by amino acid analysis (AAA) and mass spectrometry (MS). Secondary structure elements were determined by circular dichroism spectroscopy (CD) and proteins were analyzed for aggregation behavior. Models of the proteins were generated with Swiss-Model using homologues *Blattella germanica* allergens as templates. Sera from cockroach allergic patients from India and South Korea were used to test IgE sensitization to these proteins in ELISA.

**Results**

The identity of the three purified proteins was confirmed by MS and concentrations ranging from 0.3 - 1 mg/ml were determined by AAA. CD revealed partially intact secondary structural elements of the proteins, resembling the modeled structures. While for Per a 2 and Per a 3 a sensitization frequency of 50 – 92% was observed in ELISA experiments, reactivity to Per a 1 was limited in the tested patients’ cohort.

**Conclusion**

Purified, recombinant Per a 1, Per a 2 and Per a 3 were physico-chemically characterized and their ability to bind IgE was shown. By including all currently described *Periplaneta americana* allergens and testing diverse patients’ cohorts from Europe, Asia and America, the sensitization pattern of these allergens can be elucidated.

## A238 Assessment of Characteristics of Itch in Patients with Hand Eczema

### Sung-Min Park^2^, Won-Ku Lee^1^, Jeong-Min Kim^2^, Gun-Wook Kim^1^, Je-Ho Mun^2^, Hoon-Soo Kim^1^, Margaret Song^1^, Hyun-Chang Ko^2^, Moon-Bum Kim^1^, Byung Soo Kim^1^

#### ^1^Pusan National University; ^2^Pusan National University Yangsan Hospital, South Korea

##### **Correspondence:** Sung-Min Park – Pusan National University Yangsan Hospital, South Korea

**A) Background:** Hand eczema is a common inflammatory dermatosis which influences the quality of life especially when it comes to accompanying itching. However, there is no research regarding the characteristics of itch and neurologic association in hand eczema so far. We performed this study to objectify the influence on quality of life in patients with hand eczema and to investigate the association with cutaneous nerve.

**B) Methods:** we conduct a hand eczema severity scoring using HECSI score, a questionnaire contains Leuvin itch scale and dermatology life quality index(DLQI), and a neurologic exam using von-Frey filament and neurometer.

**C) Results:** Forty-four patients were enrolled the study and 36 patients were also done neurologic examination. Itch occurred at least once daily in all study patients, and finger and palm were the most commonly affected itch areas (25.0%). The Leuvin itch scale was directly proportional to HECSI score (p=0.272), and DLQI was inversely proportional to it (p=0.019). The sensory threshold force measured by von-Frey filament was significantly higher in lesion than normal skin (p<0.05) and the pain threshold using neurometer was also significantly decreased in lesion (p<0.05).

**D) Conclusion:** This study is a unique trial which describes the characteristics of itch experienced in hand eczema and investigates the relationship between hand eczema and cutaneous nerve. We thought it could be a good basis in development of future therapeutic modalities such as neurotransmitters in hand eczema.

## A239 The Hidden Culprit: A Case of Repeated Anaphylaxis from Cremophor Hypersensitivity

### Young Nam Kim^1^, Sehyo Yune^1^, Jin-Young Lee^2^, Jihye Kim^1^, Young Eun Kim^1^, Jae-Won Paeng^3^, Mi-Jin Jang^3^, Dong-Chull Choi^4^, Byung-Jae Lee^4^, Yongseok Lee^1^

#### ^1^Samsung Medical Center, South Korea; ^2^Health Promotion Center, Samsung Medical Center; ^3^Samsung Biomedical Research Institute; ^4^Samsung Medical Center, Sungkyunkwan University School of Medicine

##### **Correspondence:** Young Nam Kim – Samsung Medical Center, South Korea

Drug-induced anaphylaxis is a big pitfall in patients receiving antineoplastic chemotherapy. We report a case of lung cancer patient who experienced two near-fatal anaphylactic reactions that resulted from paclitaxel and multivitamin, separately. Recurrent severe reactions to different agents led to further investigation to which material the patient was hypersensitive. The skin prick test revealed sensitization to cremophor, which is a commonly used emulsifying agent. This case emphasizes the importance of correctly identifying the culprit drug of anaphylaxis to avoid potentially fatal reaction.

## A240 Spectrum of Anaphylaxis in Children and Adults at Emergency Departments in Singapore

### Si Hui Goh^3^, Bee Wah Lee^1^, Jian Yi Soh^2^

#### ^1^National University of Singapore; ^2^National University Hospital; ^3^Kandang Kerbau Women’s and Children’s Hospital Singapore

##### **Correspondence:** Si Hui Goh – Kandang Kerbau Women’s and Children’s Hospital, Singapore

**Background**

We aim to describe the epidemiology and triggers of anaphylaxis related Emergency Department visits in children and adults in Singapore.

**Method**

We performed a prospective study of patients visiting the Emergency Department(ED) for anaphylaxis. We collected data from the emergency departments of three major tertiary hospitals from April 2014 to March 2015. The electronic records of patients with the diagnoses of allergy, angioedema, urticaria and anaphylaxis (ICD codes 9953, 9951, 7080, 9950, 7089) were obtained.

**Results**

There were 271 cases of anaphylaxis identified, making up 7% of all ED visits for allergy-related symptoms. The median age was 22 years (range 5 months-89 years). Children (below 16 years) made up 35% (94/271) of the cohort, with 139 males (51%). Patient ethnicity included Chinese (180 cases, 66%), Malay (44, 16%), Indian (18, 7%) and others (29, 11%).

The most common trigger was food (n=126, 46%), followed by drugs (n=50, 18%) and stinging insects (n=14, 5%). The trigger could not be identified at initial presentation in 62 cases (23%). The most common food allergen was shellfish (n=36, 29%), followed by peanuts and other nuts (n=16, 13%). There were 2 new cases of galacto-oligosaccharide allergy that were confirmed on follow-up.

Age distribution varied with the triggering factor. Those with drug-induced anaphylaxis were older than those with food allergy (mean age 32.5 vs 24.5 years, p = 0.015). All cases of insect-related anaphylaxis occurred in adults (age range 22-68 years). The cases of shellfish anaphylaxis were older than those with peanut/tree-nut anaphylaxis [mean 29.7 years (range 6-75, SD 19.7 years) vs mean 14.2 years (range 2-51, SD 13.9 years); p = 0.006].

The hospitalization rate was 52%. On both univariate and multiple regression analysis, the only significant factor associated with hospitalization was the age of the patient (Spearman’s correlation coefficient 0.169, p=0.006).

**Conclusion**

Food and drug allergies are common causes of anaphylaxis. Shellfish is the most common food allergen. Anaphylaxis caused by drugs, stinging insects and shellfish was more common in adults, whilst anaphylaxis caused by peanuts and tree nuts is more common with children.

## A241 Improved Quality of Life through an Integrated Health Care Service for Children with Atopic Dermatitis

### Hyungoo Kang, Hyunhee Kim, Hye-Yung Yum

#### Seoul Medical Center, South Korea

##### **Correspondence:** Hyungoo Kang – Seoul Medical Center, South Korea

**Background:** Atopic dermatitis (AD) is a chronic, relapsing inflammatory skin disease characterized by dry skin with severe itching. Children with AD and their caregivers report disease associated sleep disruption, irritability, anxiety, behavior problems. Moreover, AD places a significant economic burden on the patient, family and society. So, an integrated health care service can be useful to comprehensively evaluate triggers and response to treatment, address confounding factors including psychological problems, and educate patients and family.

**Method:** This study was designed to evaluate the effectiveness of integrated health care service in children with AD according to quality of life and clinical symptom scores. 134 children were referred from local health care office to Seoul Medical Center for management of AD from July to December, 2011. The questionnaire developed by the ‘Atopy Free Seoul’ research project in 2008 was used for quality of life (QOL) survey, and SCORing of Atopic Dermatitis (SCORAD) was done at each visit.

**Results:** The study targets were 134 children with the average age of 6.11 years with AD and visited the hospital 2.01 times on average. It was found that the QOL scores of patients participated in our integrated health care service was reduced by 10.43 after treatment compared before intervention (p<0.0001). In 46 children among them, SCORAD also averagely decreased by 5.78 after treatment (p<0.0001). Moreover there is positive correlation between changes in scores of QOL and SCORAD of 46 patients (r=0.46, P<0.001). 74.6% was satisfied with improvement degree of symptoms after integrated health care service and 70.1% was satisfied with improvement degree of daily life.

**Conclusion:** Integrated health care service for children with AD improved disease severity and quality of life. The results from our multidisciplinary approach supported the need for and feasibility of integrated care for children with AD and their families.

## A242 Criteria Combining Autologous Serum Skin Test and Clusterin for Predicting Antihistamine-Refractoriness in Patients with Chronic Spontaneous Urticaria

### Young Min Ye, Hae-Sim Park, Ga-Young Ban, Ji Hye Kim, Yoo Seob Shin

#### Ajou University School of Medicine, South Korea

##### **Correspondence:** Young Min Ye – Ajou University School of Medicine, South Korea

**Backgrounds:** A substantial proportion of patients with chronic urticaria (CU) is refractory to antihistamines. It remains unknown how we identify a subpopulation whose urticaria is not completely controlled by antihistamines. Autologous serum skin test (ASST) response has been suggested as a potential predictor of urticaria control. We sought to identify proteins that were differentially expressed in the sera between patients with positive and negative ASST.

**Method:** The proteomic analysis was performed using sera from 3 patients with positive results on ASST compared with those with negative ASST. Seven upregulated and 5 down-regulated proteins were identified by matrix-assisted laser desorption/ionization time-of-flight mass spectrometry in the positive ASST group compared with the negative ASST group.

**Results:** Proteins differentially expressed according to the ASST results in CU patients were classified into 5 groups: apolipoproteins, glycoproteins, modified albumin, haptoglobulin, and plectin. Among them, apolipoprotein J or clusterin was validated by using ELISA. Clusterin levels in 69 ASST positive patients were significantly higher than those in 69 ASST negative patients and in 86 healthy controls (median 227.6, min-max 108.5-359.9 vs 209.4, 117.3-288.0 vs 133.2, 0.06-277.6 μg/ml, *P* <0.001). Furthermore, it differs significantly between patients with well responsive and refractory to antihistamine treatment within 3 months (227.6, 117.3-359.9 vs 197.5, 108.5-309.8 μg/ml, *P* = 0.002). Receiver-operating characteristic (ROC) curve analysis yielded 202 ug/ml of serum clusterin as the optimal cut-off for discriminating the responsiveness to antihistamines in CSU patients (AUC 0.759, 95% CI 0.679-0.839, *P* < 0.001). Criteria combining ASST results and serum clusterin levels can predict 92.7% of CU patients whose urticaria would be refractory to antihistamines.

**Conclusion:** Considering on that clusterin is able to modulate complement function, we suggest that serum clusterin can be a prognostic marker to determine the responsiveness to antihistamine treatment in patients with CU.

This work was supported by grants from National Research Foundation of Korea funded by the Korean Government (MSIP: NRF-2012R1A5A2048183).

## A243 Urinary Leukotriene E4 Levels in Wheezing Infants

### Takumi Takizawa^1^, Masahiko Tabata^2^, Akira Aizawa^1^, Hisako Yagi^1^, Yutaka Nishida^1^, Hirokazu Arakawa^1^, Akihiro Morikawa^3^, Solongo Orosoo^1^

#### ^1^Gunma University Graduate School of Medicine, Japan; ^2^Donguri-Kodomo Clinic; ^3^Kitakanto Allergy Institute

##### **Correspondence:** Takumi Takizawa – Gunma University Graduate School of Medicine, Japan

Several biomarkers have been developed to address airway inflammation in bronchial asthma (BA), including exhaled nitric oxide, sputum eosinophils. However it is challenging and sometimes considered to be rather invasive to appropriately obtain those biomarkers in young children. Since urine is a non-invasive and easy-to-obtain samples in children, urinary leukotriene E4 (uLTE4) is one of the most potent biomarkers to address airway inflammation in infants. To ask whether uLTE4 can be used for the diagnosis of BA in young children, we determined concentrations of uLTE4 of wheezing children. A total of 184 patients at an outpatient clinic in Saitama prefecture in Japan for wheezing from February 2012 to August 2013 participated in the study. Urine samples were collected and immediately frozen until use. LTE4 was collected with an anti-LTE4 antibody and the concentrations were determined by ELISA. Urine creatinine (uCr) levels were also determined. Since uCr levels differ among different ages, we first corrected all the uCr values with those at corresponding ages. uLTE4 was then normalized to age-corrected uCr levels. Patients consisted of the first wheezers with or without a family history of BA, the first wheezers with RSV infection, intermittent BA, mild persistent BA and controls with fever but not wheezing. uLTE4 levels of the first wheezers with a family history who responded to inhalation of bronchodilator (n=33) and intermittent BA (n=25) were higher (p<0.01) than those of controls. There were no significant differences between uLTE4 in other groups and controls. These results suggest that uLTE4 can be a marker of allergic airway inflammation in young children. We are currently following clinical courses of those patients to ask whether uLTE4 can be a predictive marker for the development of BA.

## A244 Allergic Sensitization to Whey in Mice Is Facilitated By the Mycotoxin Deoxynivalenol (DON)

### Saskia Braber^1^, Marianne Bol-Schoenmakers^1^, Peyman Akbari^1^, Prescilla V. Jeurink^1,^^2^, Priscilla De Graaff^1^, Joost J. Smit^1^, Betty C. A. M. Van Esch^1,2^, Johan Garssen^1,2^, Johanna Fink-Gremmels^1^, Raymond H. H. Pieters^1^

#### ^1^Utrecht University, Netherlands; ^2^Nutricia Research

##### **Correspondence:** Saskia Braber – Utrecht University, Netherlands

**Background:** Orally ingested food proteins normally result in the induction of oral tolerance, whereas allergic sensitization to food proteins in mice is induced in the presence of a mucosal adjuvant like cholera toxin (CT). CT is therefore often used as a tool to unravel the mechanisms behind allergic sensitization, although CT is not involved in the onset of allergic sensitization in humans. The mycotoxin deoxynivalenol (DON) is among the most frequently detected contaminants of wheat and wheat-based products, and is able to impair intestinal barrier function. As such, we hypothesize that DON may represent a more human-relevant mucosal adjuvant and therefore the present study investigated the capacity of DON to act as a mucosal adjuvant in a mouse model of whey-induced food allergy.

**Methods:** Female C3H/HeOuJ mice (n=8 per group) were orally exposed to DON plus whey once a week for 5 weeks, while control mice received DON in PBS. Acute allergic skin responses, change in body temperature and other anaphylactic shock reactions were measured upon whey-challenge. Allergen-specific antibodies and ST2 were measured in serum. mRNA expression of claudin-2 and -3, E-cadherin and IL-33 were determined in intestinal tissue and ST2 was measured in serum 6h after a single oral DON-exposure.

**Results:** Mice exposed to DON plus whey showed whey-specific IgG1, IgG2a and IgE antibodies in serum and an acute allergic skin response upon intradermal whey challenge compared to control mice. Furthermore, a significant time-dependent increase in soluble ST2 in serum was observed in DON plus whey sensitized mice compared to control mice. In addition, analysis of intestinal tissues, isolated 6h after a single oral exposure to DON, revealed increased mRNA expression of the tight junction proteins claudin-2 and -3 and the adherens junction E-cadherin, as well as an increase in IL-33 mRNA accompanied by an increase in the soluble IL-33 receptor ST2 in serum.

**Conclusions:** Together, these results demonstrate that DON facilitates allergic sensitization and may thus serve as a model adjuvant. Our data therefore illustrate the possible contribution of food contaminants, like DON, in allergic sensitization in humans.

## A245 How to Define Chronic Cough: Based on a Systematic Review of the Epidemiological Literature

### Gun-Woo Kim^1^, Eun-Jung Jo^2^, Sujeong Kim^3^, Woo-Jung Song^1^, Yoon-Seok Chang^4^, Shoaib Faruqi^5^, Ju-Young Kim^1^, Mingyu Kang^6^, Min-Hye Kim^7^, Jana Plevkova^8^, Heung Woo Park^1^, Sang-Heon Cho^1^, Alyn Morice, So-Hee Lee^1^, Sun-Sin Kim^1^, Seoung-Eun Lee^2^

#### ^1^Seoul National University Hospital, South Korea; ^2^Pusan National University Hospital, South Korea; ^3^Asan Medical Center, University of Ulsan College of Medicine, South Korea; ^4^Seoul National University Bundang Hospital, South Korea; ^5^Castle Hill Hospital, UK; ^6^Chungbuk National University Hospital, South Korea; ^7^Ewha Womans University, School of Medicine, South Korea; ^8^Comenius University, Slovakia

##### **Correspondence:** Gun-Woo Kim – Seoul National University Hospital, South Korea

**Background:** Lately, the evidence shows a huge epidemiological burden of chronic cough in general populations. However, the definitions of chronic cough varied, and no definitions were validated for clinical relevance. We examined existing epidemiological definitions in detail and investigated the operational characteristics.

**Methods:** A systematic literature review was conducted for the epidemiological studies that reported the prevalence of chronic cough in general adult populations, which were published in the peer-reviewed journals during the years 1980 to 2013. The operational characteristics of the most common definition were examined by meta-analyses of the male-to-female ratio in chronic cough prevalence.

**Results:** The systematic review included 70 studies. The most common definition was cough ≥ 3 months (12-month prevalence) without specification of phlegm (n=50), which conflicts with the criteria in clinical guidelines of cough ≥ 8 weeks.

Meta-analyses were conducted for the male-to-female ratio of chronic cough among 28 studies that reported sex-specific prevalence using the most common definition; however, the pooled male-to-female odds ratio was 1.26 (95% confidence interval 0.92–1.73) with significant heterogeneity (*I*^*2*^=96%, *P*<0.001), which was in contrast to previous clinical observations of female predominance in cough clinics.

**Conclusions:** This study indicates two important issues in defining chronic cough in further epidemiological studies. A conflict between epidemiological and clinical definitions in duration criteria needs to be resolved. Another unexpected difference in the gender preponderance between the community and clinics warrants clinical validation of the existing definition.

## A246 Asko Study: Comparison of Behavior and Habits in Diagnosis and Treatment of Adult Asthma and COPD Patients

### Bilun Gemicioglu^11^, Zeynep Misirligil^1^, Arif Hikmet Cimrin^2^, Hakan Gunen^3^, Tevfik Ozlu^4^, Aykut Cilli^5^, Levent Akyildiz^6^, Hasan Bayram^7^, Esra Uzaslan^8^, Oznur Abadoglu^9^, Mecit Suerdem^10^

#### ^1^Ankara University Faculty of Medicine; ^2^Dokuz Eylul University Faculty of Medicine; ^3^Sureyyapasa Pulmonary Diseases Hospital and Research Center; ^4^Karadeniz Technical University Faculty of Medicine; ^5^Akdeniz University Hospital; ^6^Mardin Medical Park Hospital; ^7^Gaziantep University Faculty of Medicine; ^8^Uludag University Faculty of Medicine; ^9^Cumhuriyet University Faculty of Medicine; ^10^Selcuk University Faculty of Medicine; ^11^Istanbul University Cerrahpasa Faculty of Medicine, Turkey

##### **Correspondence:** Bilun Gemicioglu – Istanbul University Cerrahpasa Faculty of Medicine, Turkey

**Background:** The objective of this study is to investigate the physicians’ approach to asthmatic or COPD patients, living in different areas of Turkey. In this report baseline demographics, risk factors, and adherence to therapy of asthmatic and COPD patients are compared.

**Methods:** A total of 1892 newly diagnosed adult patients (1116 asthmatic, 776 COPD) from 136 secondary or tertiary centers of different geographic locations took part in this study, and a standard web-based questionnaire including items related with demographic, clinical, laboratory and pharmacological parameters was applied from July 2012 to May 2014.

**Results:** Asthmatic patients were mostly female (64.4%), while the male patients were higher (88.1%) in COPD. The percentage of patients whose age is ≥65 years was significantly higher in COPD patients compared to asthmatic patients (30.4% and 7.1%, respectively) (p<0.001). Evaluation of the disease severity showed that nearly half of the asthmatic patients were in “moderate persistent” category (45.0%) and more than half of the COPD patients (54.4%) were in “GOLD B”. More than half of both asthmatic and COPD patients had at least one accompanying disease (53.9% and 52.8%, respectively) and hypertension was the most seen disease in both asthmatic and COPD patients (13.9% and 21.1%, respectively) (p<0.0001). Nearly half of the asthmatic patients (45.5%) stated asthma and 13.7% stated COPD in their family history, while nearly ¼ of COPD patients stated COPD (22.2%) and 8.2% stated asthma. Evaluation of smoking anamnesis showed that there was a significant difference between asthmatic and COPD patients by means of “currently smoking" status; percentages were 27.9% and 56.3%, respectively (p<0.001). It is found that the percentage of COPD patients (37.0%) who have been exposed to dust, gas and/or vapor in work place was significantly higher than asthmatic patients (25.2%) (p<0.001). Evaluation of trigger factors showed that air pollution was the most common trigger in both asthma (54.1%) and COPD (54.6%) groups. Evaluation of adherence to the study visits showed that percentage of the patients coming to one control visit was higher in asthmatics compared to COPD patients (61.6% and 55.9%, respectively) (p=0.014). However, when groups were compared in terms of 3rd follow-up visit, adherence was low in both groups (20.3% and 18.7% for asthma and COPD). Compliance to the treatment in percentages of regular medication use was significantly better in COPD patients compared to asthmatic patients (p=0.021).

**Conclusion:** COPD and asthma are associated with significant economic burden. Identification and reduction of exposure to risk factors are important in the treatment and prevention. Patient compliance may be the key to better disease management. We need new strategies to improve adherence in patients.

## A247 Changes in Pulmonary Function in the Treatment of Obesity in Children

### Keigo Kainuma

#### Mie National Hospital, Japan

**Background:** Associations between obesity and asthma in adults and children have been implicated but causal mechanisms, especially those related to respiratory physiology, are not well understood. We reported previously that obesity caused in abnormal reactance values in lung physiology. To further dissect the link, we analyzed changes in pulmonary function during the treatment of obesity in children.

**Methods:** Eleven obese children (9 boys and 2 girls) from 8 to 15 years of age were enrolled in this study. 3 of them were hospitalized for more over 3months and the others were outpatients for 6 months. Eight had no asthma and 2 of them had asthma in mild intermittent severity without need of controller medications. Spirometry and two forced oscillation technique (FOT) tests (MostGraph® and Master Screen IOS®) were performed to assess lung function before and after treatment of obesity (diet and exercise). Patients were divided into two groups, the success group (n=5) and the failure group (n=6), based on outcome in weight loss. Success was defined as more than 10% improvement in percentage of overweight (POW) in the observation period.

**Results:** In FOT, R5-R20, resonant frequency (Fres), reactance area (AX) (IOS®) and R5, Fres (MostGraph®) were significantly improved in the success group, not in the failure group. As previously reported, FEV1.0% was improved significantly in the success group, not in the counterpart.

**Conclusions:** Respiratory function, not only FEV1 but reactance and resistance parameters in FOT, was improved in parallel with weight loss in obese children, which may implicate functional relationship between obesity and asthma.

## A248 Changes of Feno and Nasal Feno Levels after Treatment in Pediatric Allergic Rhinitis

### Hyun-a Kim^1^, Ha-Su Kim^1^, Woo Yong Bae^2^, Jin-a Jung^1^

#### ^1^Dong-a University Hospital, South Korea; ^2^Department of Otorhinolaryngology-Head and Neck Surgery, Dong-a University College of Medicine

##### **Correspondence:** Hyun-a Kim – Dong-a University Hospital, South Korea

**Purpose:** The fractional concentration of exhaled NO (FeNO) has been shown to be increased in inflammatory airway diseases, including bronchial asthma (BA), allergic rhinitis(AR), and chronic rhinosinusitis. Recently, there are several reports about increasing of nasal FeNO in patients with AR. In the present study, we examined the differences of oral FeNO and nasal FeNO levels in AR patients with or without BA.

**Methods:** In the cross-sectional study, 31 patients with mild-persistent or severe-intermittent AR patients, 23 AR patients with BA, and 19 normal subjects were enrolled. The FeNO levels were measured by using a handheld electrochemical analyzer (NObreath®; Bedfont Scientific LTd., Rochester, Kent, UK) before treatment and at one month after treatment. The nasal FeNO levels were measured by using a nose adaptor. All patients were measured serum total eosinophil count, total IgE, allergen-specific IgE against house dust mite(Dp, Df), Alternaria, cat dander, cockroach, tree mixes by the ImmunoCAP or skin prick test.

**Results:** There were no significant differences in the serum total eosinophil count, total IgE levels between AR patients and AR patients with BA. The oral FeNO levels before and after treatment showed no significant differences between AR patients and AR patients with BA. The nasal FeNO levels before treatment were significantly higher in the AR patients with BA than the AR patients.(P=0.005) After treatment, the nasal FeNO levels were no significant differences between diseases groups. The nasal FeNO levels after treatment were significantly lower compared to the nasal FeNO levels before treatment in both AR and AR patients with BA. (AR patients; P=0.044, AR patients with BA; P=0.004).

**Conclusion:** After treatment, the nasal FeNO levels were significantly decreased in the AR patients with or without BA. The nasal FeNO measurement in AR patients is suitable method for monitoring the effect of treatment modalities.

## A249 Prevalence of Vitamin D Deficiency in Exclusively Breastfed Infants in Kenya

### Rose Kamenwa, William Macharia, Nusrat Said

#### Aga Khan University Hospital, Kenya

##### **Correspondence:** Rose Kamenwa – Aga Khan University Hospital, Kenya

**Introduction**

Vitamin D deficiency in infants is a recognized cause of rickets. evidence has emerged linking it with lower respiratory tract infections, food allergy, diabetes, schizophrenia and various other extra skeletal health effects. Exclusively breastfed infants are especially vulnerable to vitamin D deficiency due to their dependence on previous trans-placental transfer of vitamin D from the mother, dietary vitamin D from breast milk and cutaneous synthesis of vitamin D on exposure to sunlight. The worldwide epidemic of Vitamin D deficiency in pregnancy and the low content of vitamin D in breast milk underlie the high risk of deficiency in exclusively breastfed infants. Data regarding the magnitude of vitamin D deficiency among exclusively breastfed infants in Kenya is needed to inform supplementation policies.

**Objectives**

This study aimed to determine the prevalence of vitamin D deficiency in exclusively breastfed infants and to evaluate its relationship between PTH in this population.

**Study Design**

This was a cross sectional study that included three to six month old exclusively breastfed infants at the Aga Khan University Hospital, Nairobi.

**Methods**

Ninety-eight infants were enrolled in the study and their serum 25(OH) D, calcium, phosphate and PTH measured. Data on sunlight exposure and maternal vitamin D supplementation was also collected**.**

**Results**

Prevalence of vitamin D deficiency when using 20 ng/ml cut-off, was 24 % (95% CI 14.9%-32.0%). A further 32% of the infants had insufficient levels of vitamin D and 45% of the infants were replete. There were no cases of secondary hyperparathyroidism with vitamin D level above 40ng/ml and one case had an elevated PTH with vitamin D level above 30ng/ml. Using 30ng/ml as the “presumed normal” cut-off for this population, the prevalence of vitamin D deficiency doubled to 55%. Only four mothers received vitamin D supplementation during breastfeeding and all their infants were vitamin D replete. Less than 5% of the study population had skeletal signs of rickets.

**Conclusion**

Prevalence of Vitamin D deficiency is high in this very young population despite the abundnce of sunshine in this geographical region. A policy on vitamin D supplementation is recommended in this population to mitigate the impact of vitamin D deficiency especially on chronic non-communicable diseases.

## A250 In-Vitro Screening of Atopy in the Indian Population: Are Current Methods Adequate, Keeping Local IgE Seroprevalence for Common Food & Inhalant Allergens in Mind?

### Vidya Nerurkar, Meenal Patel, Simi Bhatia

#### Srl Ltd, India

##### **Correspondence:** Vidya Nerurkar – Srl Ltd, India

**Background**

Total serum IgE and Phadiatop^TM^ are 2 key tests employed for in-vitro screening of atopy. Acknowledging the possibility of unique allergen prevalences in the Indian subcontinent, aim of the study was to understand the IgE seroprevalence of common food & inhalant allergens, in Indian adult population with suspected atopic disease & compare performance of Phadiatop^TM^ (US version) & Total IgE as screening tests.

**Methods**

The study was carried out in an accredited private medical laboratory in Mumbai, India. It involved retrospective data analysis on 545 sera, collected between 2013 & 2014, from patients with suspected allergic disease. Tests included Allergen specific IgE testing for 29 common food and inhalant allergens (ImmunoCAP, Ms. Thermofischer Phadia, Sweden) .335 sera had also been simultaneously screened using Adult Phadiatop^TM^ (ImmunoCAP, Ms. Thermofischer Phadia, Sweden), while 210 were screened for Total IgE (Immulite, Siemens healthcare, USA). These were designated as Group I & Group II respectively. Both groups had similar age and gender distribution.

**Results**

446/545 patients (81.8%) showed sensitization to at least 1 or more allergens. Multisensitization was common and seen in 73.2% sera. Most prevalent allergens were Housedust mite-D pternyssinus (58.5%), Cockroach (57.4%), Housedust mite-D farinae (56.8%), Shrimp (47.7%), House dust (45.13%), Weed Chenopodium album pollen (44.2%), Candida albicans (41.8%), Weed Artemisia vulgaris-Mugwort pollen and Wheat (32%).

Using Allergen specific IgE as standard (Cut off of 0.1 kua/l), the sensitivity & specificity of Phadiatop in Group I was 62.1% and 98.4% respectively. Phadiatop sensitivity increased if only Allergen specific IgE values >0.5 kua/l were considered, i.e those more likely to be involved in symptomatic allergic disease. Phadiatop missed cockroach and shrimp positive sera. Though Phadiatop is not intended to screen for food allergies, cross reactivity between certain pollens and foods yielded few positive screening results. The sensitivity & specificity of Total IgE in Group II was 40.8 % and 100% respectively. Total IgE not only missed low level sensitization, but also missed some sera with high positive allergen specific IgE results.

**Conclusion**

Phadiatop^TM^ was found to be a satisfactory screening test for atopy and outperformed Serum Total IgE. Looking at the trends in inhalant and food allergen seroprevalence, current study was able to establish a preliminary list of allergens relevant in the Indian context. These could be tested in addition to a Phadiatop or as a follow-on to a negative Phadiatop^TM^ in the event of strong clinical history of allergic disease.

## A251 Usefulness of House Dust Mites Nasal Provocation Test in Asthma

### Inseon S Choi, Soo-Jeong Kim, Joo-Min Won, Myeong-Soo Park

#### Chonnam National University Medical School, South Korea

##### **Correspondence:** Inseon S Choi – Chonnam National University Medical School, South Korea

**Background:** We previously reported that skin prick test was sensitive and serum specific IgE test was specific for predicting positive airway responses to house dust mites (HDM) in asthma. Because nose and bronchus are one airway, nasal provocation test would be more specific than skin test for predicting bronchial responses to HDM.

**Methods:** Allergy skin prick test, and nasal and bronchial provocation tests using HDM *Dermatophagoides farinae* were done in 35 young men (19~28 years-old) who wanted a military certification for asthma. The nasal responses to HDM were scored according to the severity of 3 nose symptoms (rhinorrhea, sneezing, and nose itching).

**Results:** The prevalence of positive skin test (≥3+) to HDM was not significantly different between the patients with (n=23) and without (n=12) a positive response (early or late) to bronchial challenge (87.0% vs. 66.7%, *P*=0.200). However, the nasal response score was significantly higher in the responders than the others (1.00±0.24 vs. 0.25±0.13, *P*=0.011). The concordance of positive response not to the skin test (*k*=0.225, *P*=0.154) but to the nasal test (cutoff score: ≥2) (*k*=0.306, *P*=0.012) was significant with the positive bronchial response. The diagnostic sensitivity of the nasal test (47.8% at cutoff≥1, 39.1% at cutoff≥2) was lower, but the specificity (75.0% and 100%, respectively) was higher than that of the skin test (sensitivity: 87.0%, specificity: 33.3%).

**Conclusion:** The skin test is more sensitive, whereas the nasal test is more specific for predicting a positive bronchial response to HDM in asthma.

## A252 Biomarker-Based Treatment Option for Preschool Children with Recurrent Wheeze

### Mizuho Nagao

#### Allergy Center and Department of Clinical Research, Mie National Hospital, Japan

**Background:** Recurrent wheeze in preschool age consists of several phenotypes, each of which may represent different underlying pathology and differential response to treatment. However, phenotype-based recommendations for treatment have not been established since most of clinical trials have targeted to broad ranges of phenotypes, not to a specific phenotype. In order to identify a novel clue to the drug choice for preschool wheeze, we investigated possible utility of biomarkers by comparing efficacy of 2 treatment options in a specific biomarker-defined subgroup. Serum eosinophil-derived neurotoxin (EDN) and sensitization to house dust mite (HDM) were employed as biomarkers in this study.

**Methods:** Children of 1 to 5 years old who had more than 3 episodes of recurrent wheeze with documented reversibility were enrolled in the study. They were tested for specific IgE to HDM and serum EDN at the entry. Then, they were randomized to receive budesonide or montelukast for 12 weeks if they were HD-sensitized, high serum EDN >53 ng/ml and symptomatic during run-in period. Primary outcome was asthma controlled days (ACDs). Non-eligible patients were also followed up for the same period with administration of montelukast.

**Results:** Ninety-eight subjects were enrolled in the study and 42 were eligible for randomization. Both group responded well to treatment and there was no difference in ACDs between the groups. However, serum EDN levels significantly decreased in montelulast group, not in budesonide group. Serum EDN levels in non-eligible group treated with montelukast also significantly decreased.

**Conclusions:** Budesonide and montelukast had the same efficacy to HDM-sensitized and high EDN subgroup of preschool wheeze and the biomarkers did not represent differential responses to the 2 treatment options. EDN reducing effect of montelukast, however, warrants further investigation.

## A253 Anti-Tuberculosis Drugs-Induced Liver Injury in Patients with Connective Tissue Diseases

### Dong Won Park, Jang Won Sohn, Ji Young Yhi, Ji-Yong Moon, Sang-Heon Kim, Tae Hyung Kim, Dong Ho Shin, Ho Joo Yoon

#### Hanyang University College of Medicine, South Korea

##### **Correspondence:** Dong Won Park – Hanyang University College of Medicine, South Korea

**Background:** Anti-tuberculosis drugs (ATD) are major causes of drug induced liver injury (DILI) around the world. Compared with general population, the patients with connective tissue diseases (CTD) are suspected to be at higher risk of DILI, since they are frequently exposed to various medications including immunosuppressive agents. We aimed to assess the incidence and severity of DILI in CTD patients in comparison with non-CTD patients.

**Methods:** In this retrospective case control studies, we enrolled the patients with newly diagnosed tuberculosis and treated with the first line ATD for two years in a university hospital. DILI was defined as increase of serum aspartate aminotransferase (AST) and/or alanine aminotransferase (ALT) greater than threefold of the upper limit of normal (ULN).

**Results:** Of a total of 279 enrolled patients, 40 patients (14.3%) had CTD, such as rheumatoid arthritis (n = 19), systemic lupus erythematosus (n = 10) and ankylosing spondylitis (n = 6). The frequency of DILI caused by ATD in CTD patients was not significantly different from non-CTD patients (7.5% vs. 11.3%, *P* = 0.346). While severe DILI (AST/ALT > 5 x ULN) was observed more frequently in CTD than controls, there was no statistical significance (7.5% vs. 5.9%, *P* = 0.451).

**Conclusion:** The frequency of DILI in patients with CTD was not significantly different from non-CTD patients. These findings suggest that CTD is not a significant risk factor for DILI induced by ATD.

## A254 Ocular Symptoms of Cedar Pollinosis in Otolaryngology Patients

### Yukiyoshi Hyo

#### Kawasaki Medical School, Japan

**Background:** Japanese cedar pollinosis is a disease with a variety of symptoms; in particular, ocular and nasal symptoms occur frequently. The incidence of these cedar pollinosis symptoms reportedly differs year by year, and due the large amount of Japanese cedar pollen dispersed in Japan, they are often more severe than the symptoms of seasonal allergic rhinitis in Europe and the United States. Although pollen allergy prevalence symptoms in Europe (Canonica et al. 2007) and the United States (Schatz 2007) has been reported, the prevalence of ocular symptoms due to cedar pollinosis in Japan has yet to be determined.

**Methods:** We used the Japanese Rhinoconjunctivitis Quality of Life Questionnaire to examine symptoms and quality of life in 633 patients who consulted our hospital or ear, nose, and throat clinic between 2009 and 2014 during the peak cedar pollen season and who had not received any previous treatment.

**Results:** Ocular symptoms were seen in 87% of patients. Itchy eyes were more prevalent than watery eyes, with 84%of patients experiencing itchy eyes and 63% watery eyes, even in a year with low pollen dispersal. Responses for the occurrence of nasal and ocular symptoms indicated that a more severe score for nasal symptoms was correlated with better eye symptoms. Comparison of annual pollen counts revealed a correlation between worsening of itchy eyes and increased pollen counts. However, the severity of watery eye symptoms did not differ significantly between years with small and moderate pollen levels, indicating that watery eyes develop when the amount of pollens is high.

**Conclusions:** This study revealed that ocular symptoms of Japanese cedar pollinosis are prevalent even in years with low cedar pollen dispersal, and that pollinosis patients with ocular symptoms were likely to have more severe nasal symptoms.

**References**

Canonica GW, Bousquet J, Mullol J, Scadding GK, Virchow JC. Survey of the burden of allergic rhinitis in Europe. Allergy 2007;62 Suppl 85:17-25.

Schatz MA. Survey of the burden of allergic rhinitis in the USA. Allergy. 2007;62 Suppl 85:9-16.

## A255 The Clinical Characteristics of Adverse Drug Reactions Reported in a Regional University Hospital for 6 Years and the Suggestions for the Reporting System

### Jaechun Lee^1,2^, Su Hee Kim^1^, Eunkyoung Lee^1^

#### ^1^Jeju National University Hospital; ^2^Jeju National University School of Medicine, South Korea

##### **Correspondence:** Jaechun Lee – Jeju National University Hospital

**Background:** Adverse drug reaction (ADR) increases in-hospital stay, cost of care, and even mortality. Prevention of ADRs leads to marked socioeconomic benefits. We performed this study to investigate the incidence, actual reporting status and clinical features of ADRs, for improving ADR reporting system and preventing recurrent ADRs.

**Methods:** A retrospective study was performed in a regional university hospital located in Jeju, Korea. ADR cases were recruited by review of medical records from 2009 to 2013. An ADR event was defined as either of ADR-related diagnosis in a patient or ADRs reported through in-hospital ADR reporting system. The incidence, culprit drug, clinical manifestation, source of reporting, severity, treatment, and recurrence rate were assessed.

**Results:** In 1112 patients, 1375 ADR events were enrolled, estimated as 0.06% of total patient-visit during the study period. Diagnostic contrast agent (46.4%) was most common as culprit drugs, followed by antibiotics (22%), non-steroidal anti-inflammatory drugs (9.9%), and opioids (4.5%). Cutaneous involvement (67.5%) such as rash and hives was the most frequently observed manifestation. In two thirds of ADR cases, additional medical attentions were noted. In severity, 180 (13.1%) were categorized in severe ADRs. Nineteen (1.4%) experienced re-exposure to the culprit drugs, resulting recurrent ADR and 4 (0.3%) died of ADRs. Physicians were the most frequent ADR reporter using in-hospital ADR reporting system.

**Conclusions:** Large proportions of ADR events might be omitted in medical records or in reporting system. ADRs due to re-exposure to the culprit drugs were not rare. To prevent avoidable ADRs, an effective reporting and alerting system is necessary.

## A256 Changes in Skin Prick Test Results over 3 Years in School-Aged Children

### Hahn Jin Jung^1^, Jaehyun Lim^1^, Seung-No Hong^1^, Doo Hee Han^1^, Chae-Seo Rhee^2^

#### ^1^Seoul National University Hospital, South Korea; ^2^Seoul National University Bundang Hospital

##### **Correspondence:** Hahn Jin Jung – Seoul National University Hospital, South Korea

**Objectives:** Determining the allergen is important for the diagnosis and management of allergic diseases. The skin prick test (SPT) has been widely used to identify allergens. Skin sensitivity to allergens can change due to changes in lifestyle and outdoor environments. Little is known about the changes of allergen sensitivity in young children.

**Methods:** In this Allergic Rhinitis Cohort Study for Kids (ARCO-kids), consecutive pediatric patients with rhinitis symptoms underwent skin prick tests (SPTs). 1689 children were assessed for allergen sensitivity from 2009 to 2011. SPTs were performed with inhalant allergens at initial survey and re-performed at 3-year follow-up survey.

**Results:** 280 children fulfilled the follow-up SPT. Initially 72 children were negative for SPT and 25 (34.7%) developed skin sensitivity during the 3-year study. Among 25 children, 20 (80%) were newly sensitized to house dust mites (HDM). 194 (69.3%) were sensitized to HDM at initial survey and 213 (76.1%) were sensitized to HDM at follow-up survey. Skin sensitivity to cat, dog, tree, grass, fungus also increased during the study. 102 children were sensitized only to HDM. After 3-year follow up, 41 (40.2%) were newly sensitized to other antigens such as cat, dog, trees.

**Conclusion:** Our study showed the changes in skin sensitivity to inhalant allergens over 3 years in school-aged children. Skin sensitivity was increased in both non-sensitizer and mono-sensitizer to HDM.

## A257 The Analysis of Risk Factors and Features of Food Allergy in Korean Children: Nationwide Cross-Sectional Survey

### Kun Song Lee

#### Dankook University Hospital, South Korea

**Background:** To analyze related risk factors and features of food allergy in Korea children through a cross sectional survey.

**Methods:** We used a stratified cluster sampling design and chosen from a random sample of 45 primary and 40 middle schools in South Korea between October and November 2010. Total 7,725 children aged 6-7 years and 13-14 years were involved. We acquired that the information of food allergy from the questionnaires and analyzed the mean value of continuous variable (Total Ig E and Eosinophil count) and the risk factors which were associated with food allergy using multivariate logistic regression.

**Results:** The prevalence of food allergy in this study was 13.4% and the mean age at the beginning of the first allergic symptoms was 58.4 ± 41.9 months (1-168). The most common food, cause of allergic symptoms, was egg (21%) and following order common cause for food allergies symptoms were fruits, shellfish, and milk in the sequence. The mean total Ig E level and eosinophil count was higher in food allergy group (362.1 ± 555.0 IU/mL, 4.1 ± 3.2%) than no food allergy group (241.6 ± 414.4, 3.5 ± 2.9; *P* < 0.001) The risk factors of food allergy in multivariate logistic regression were asthma history (OR, 1.465; 95% CI, 1.078-1.991), atopic dermatitis history (2.841; 2.334-3.458), father and mother food allergy history (1.764; 1.189-2.617 and 2.009;1.342-3.009), and breast milk feeding for more than 7 months (1.359; 1.110-1.663).

**Conclusion:** The prevalence of food allergy in Korean children was 13.4% and the risk factors of food allergy were the present of asthma history, atopic dermatitis, and parent food allergy and breast milk feeding for more than 7 months.

## A258 A Sequential Indirect-Direct Bronchial Provocation Test for Diagnosis of Asthma: A Pilot Study

### Jaechun Lee^3^, Sun Young Yang^1^, Mi Young Ahn^1^, Jong Hoo Lee^1^, Jasmina Golez^2^

#### ^1^Jeju National University Hospital, South Korea; ^2^Ambulanta Meznar, Slovenia; ^3^Jeju National University School of Medicine, South Korea

##### **Correspondence:** Jaechun Lee – Jeju National University School of Medicine, South Korea

**Backgrounds:** Asthma is a chronic inflammatory disorder in the airways, which subsequently leads characteristic airway hyperresponsivenss (AHR). Measuring or proving the existence of AHR is the mainstay in its diagnosis. In Korea, airway provocation test with methacholine (direct test) had been used in clinical practices until the recent official objection of KFDA. Instead, airway provocation test with mannitol is used (indirect test), which shows lower sensitivity and consumes longer test-time compared with those in methacholine, despite higher specificity in the diagnosis of asthma. To overcome its demerits, a sequential indirect-direct provocation test was designed.

**Methods:** A prospective observational study was conducted in asthma-suspicious subjects. Indirect test was performed with skipped doses but up to 640 mg in accumulation. Methacholine inhalation (8mg/mL) by 2-min tidal breathing method was applied thereafter in the subjects who showed negative (PD15 > 640 mg of mannitol). Regardless of the results, at the end of the tests, bronchodilator was applied. AHR positive were defined as either PD15 < 640 mg of mannitol, PC20 < 8mg/mL of methacholine, or 20% increment of FEV1 in bronchodilator.

**Results:** Twenty four asthma-suspicious subjects (median age of 54, 11 males, 45.8%) were enrolled. The test-time test was theoretically shortened at least 10 min compared with that of suggested indirect test. In AHR, two subjects (8.3%) showed positive in mannitol provocation. Five (20.8%) showed positive in methacholine provocation compared with baseline FEV1 and 2 (8.3%) compared with FEV1 after 640mg of mannitol. Four (16.7%) showed positive in bronchodilator. Overall, 5 subjects (22.7%) showed AHR positive among 22 negatives in indirect test. No adverse reaction except for coughing during inhalation of mannitol was identified.

**Conclusions:** The sequential indirect-direct provocation facilitates to detect AHR in higher sensitivity and shorter test-time. As a confirmative test of asthma in clinical practice, it can be applied effectively after modification and verification.

## A259 Association of *VDR* and *CYP2R1* Polymorphisms with Persistent Allergic Rhinitis in a Han Chinese Population

### Hui-Qin Tian, Lei Cheng, Xin-Yuan Chen

#### The First Affiliated Hospital of Nanjing Medical University, China

##### **Correspondence:** Hui-Qin Tian – The First Affiliated Hospital of Nanjing Medical University China

**A) Background:** As recent studies have described an association between vitamin D and allergic rhinitis, we hypothesized that genes encoding components of the vitamin D pathway may be candidate genes for susceptibility to allergic rhinitis. This study sought to evaluate whether genetic variants in the vitamin D pathway-related genes are associated with allergic rhinitis in a Han Chinese population.

**B) Methods:** A hospital-based case-control study consisting of 519 patients with mite-sensitized persistent allergic rhinitis (PER) and 447 healthy controls was conducted. Five single nucleotide polymorphisms (SNPs) in *VDR* and *CYP2R1* were selected for genotyping.

**C) Results:** The *CT* genotype of rs2228570 in *VDR* exhibited a significantly decreased risk (adjusted OR = 0.63, 95% CI = 0.41-0.96), while the *AA* genotype of rs2060793 in *CYP2R1* exhibited a significantly increased risk (adjusted OR = 1.80, 95% CI = 1.02-3.16) of PER in the juvenile subgroup (<18 years old). Both the *AG* and *AG*/*GG* genotypes of rs731236 in *VDR* exhibited a significantly decreased risk (*AG*: adjusted OR = 0.43, 95% CI = 0.21-0.89; *AG*/*GG*: adjusted OR = 0.46, 95% CI = 0.23-0.94) of PER in the female subgroup. Analysis of the locus-locus interactions of *VDR* and *CYP2R1* revealed two models were statistically significant (*P* < 0.05).

**D) Conclusions:** Age and gender may have an impact on the association of three SNPs (rs2228570, rs731236, and rs2060793) in genes of the vitamin D pathway with the risk of mite-sensitized PER in this Han Chinese population. The *VDR* and *CYP2R1* polymorphisms may be involved in genetic interactions in the pathogenesis of allergic rhinitis.

## A260 Associations of Metabolic Syndrome with Asthma and Atopy in Korean Adults

### Ji-Yong Moon, Sang-Heon Kim, Tae Hyung Kim, Ji Young Yhi, Ho Joo Yoon, Jang Won Sohn, Dong Ho Shin, Dong Won Park

#### Hanyang University College of Medicine, South Korea

##### **Correspondence:** Ji-Yong Moon – Hanyang University College of Medicine, South Korea

**Background:** Whereas obesity is suggested to have a link to asthma, the epidemiological association of metabolic syndrome with asthma have been poorly demonstrated.

**Objective:** We aimed to explore the relationship of metabolic syndrome with asthma and atopy in Korean adult population.

**Methods:** Using the data from Korean National Health and Nutrition Examination Survey (KNHANES) in 2010, the study population aged 19 or older were divided into subjects without metabolic syndrome and those with metabolic syndrome that was defined by criteria of the International Diabetic Federation.

**Results:** The prevalence rates of asthma, determined by self-reported current wheezing, in the subjects without metabolic syndrome and in those with metabolic syndrome were 6.3% and 9.2%, respectively. Multivariate logistic regression analysis adjusting for age, gender and smoking status found that metabolic syndrome was significantly associated with asthma (adjusted OR 1.425, 95% CI 1.040 – 1.952, P = 0.028). There was no significant relationship in metabolic syndrome with atopy, determined by serum levels of the specific IgE to *Dermatophagoides farinae*, cockroach or dog.

**Conclusion:** In general population of Korea, subjects with metabolic syndrome have increased risk of asthma.

## A261 Clinical Manifestation and Treatment Outcome of Eosinophilic Gastroenteritis in Korean Children

### Won Im Cho, Jong Sub Choi, Dongin Suh, Gyeong Hoon Kang, Jin Soo Moon, Jae Sung Ko, Kyung Jae Lee, Shin Jie Choi

#### Seoul National University Hospital, South Korea

##### **Correspondence:** Won Im Cho – Seoul National University Hospital, South Korea

There are few studies for EGE (Eosinophilic gastroenteritis) in pediatrics patients due to its low prevalence. So we reviewed medical records of suspected patients retrospectively and 24 patients were confirmed as EGE.

The mean age at diagnosis was 5.4 years. Most patients had leukocytosis (79.2%) and peripheral eosinophilia (91.7%) and had gastrointestinal symptoms such as diarrhea (54.2%) and abdominal pain (45.8%). Eighteen patients (79.2%) were classified as mucosal layer disease. Five patients (20.8%) showed subserosal eosinophilic infiltration or eosinophilia in ascites or surgical biopsy, and 1 patient with muscular eosinophilic infiltration was revealed by surgical biopsy. Totally three patients had surgery to diagnose or treat EGE. Three patients showed gastroduodenal ulcers in endoscopic findings. Some patients had protein-losing enteropathy (25%). Anemia was in 13 patients (54.2%). Five patients (20.8%) had improved with only conservative managements such as hypoallergenic diet and food restriction. Other 19 patients (79.2%) needed steroids and 15 patients showed improvement finally. EGE relapsed in 8 patients (33.3%) and 4 patients had symptoms steadily despite steroid treatment. Association of steroid dependency or resistance with gastroduodenal ulcer is statistically significant.

To diagnose EGE, suspicious pediatric patients with peripheral eosinophilia and gastrointestinal symptoms should have endoscopic or surgical biopsy. Most patients have improved with conservative managements or steroids. However, some patients have steroid dependency or resistance, which are associated with gastroduodenal ulcer.

## A262 The Sensitization Model and Correlation of Bermuda and Timothy Grass Pollen Allergen in Allergic Patients in Southern China

### Wenting Luo, Baoqing Sun

#### Guangzhou Institute of Respiratory Diseases, First Affiliated Hospital of Guangzhou Medical University, China

##### **Correspondence:** Wenting Luo – Guangzhou Institute of Respiratory Diseases, First Affiliated Hospital of Guangzhou Medical University, China

**Background**

Using the allergen components detection technology, to study on the sensitization model and correlation of bermuda and timothy grass pollen allergen in respiratory allergic patients in Southern China.

**Methods**

258 cases of patients with allergic diseases, including 92 asthma patients, 78 allergic rhinitis patients, 88 rhinitis with asthma, 43 children, 215 adults. Serum sIgE of bermuda and timothy were detected by ImmunoCAP100. If the bermuda-sIgE were positive, sIgE of Cyn d 1, Phl p 1, Phl p 4, peanut, Ara h 1, Ara h 8, birch, Bet v 1 and CCD were tested.

**Results**

1, 22.5% (58/258) patients were sIgE-positive to bermuda, 13.6% (35/258) sIgE-positive to timothy. The positive rate of bermuda and timothy had no significant differences between children and adult, nor between male and female groups (*P*>0.05).

2, 53.5% (31/58) bermuda sIgE-positive patients were sensitized to Cyn d 1. The concentration of sIgE of bermuda in Cyn d 1 positive patients were significantly higher than Cyn d 1 negative levels (Z=3.103, *P*=3.103).

3, In the 58 bermuda sIgE-positive patients, 35 patients were timothy sIgE-positive. The concentration of sIgE of bermuda in timothy positive patients were significantly higher than timothy negative (Z=4.735, *P*=4.735).

4, 100% (35/35) timothy-positive patients were sensitized to Phl p 4 , but only 17.1% (6/35) were positive to Phl p 1.

5, 56.90% (33/58) bermuda-positive patients with were sensitized to peanut. Ara h 1 were positive in 6 patients with peanut-sIgE higher than 17.5kU/L. 3 of them were sensitized to Ara h 8. 32.76% (19/18) were sensitized to birch. 6 cases of birch-positive were sensitized to Bet v 4, which sensitized to Ara h8 simultaneously.

6, 39.66% (23/58) bermuda-positive patients with CCD sensitization, and CCD sIgE levels had good correlation with bermuda, Cyn d 1, peanut, birch, timothy and Phl p 4 (*P*<0.001).

**Conclusion**

22.5% patients were sIgE-positive to bermuda grass and 13.6% sIgE-positive to timothy grass in Southern China. Bermuda sIgE-positive patients were sensitized to timothy grass, peanuts, CCD and birch and related components in different degree. 53.45% bermuda-positive patients were sensitized to Cyn d 1, and the higher of bermuda sIgE level, the more likely to sensitize to Cyn d 1. 100% timothy-positive patients were sensitized to Phl p 4 , but only 17.1% were sensitized to Phl p 1. 18.18% peanut sensitization patients were sensitized to Ara h 1, 9.09% were reacted to Ara h 8. 31.57% birch-positive patients were sensitized to Bet v 4. CCD had good correlations with grass pollen, peanut and birch allergens, indicated wide cross-reaction between them were caused by carbohydrate cross-react determinants.

## A263 A Pilot Study on the Outcomes of Respiratory Allergic Diseases at Pre-School Age in Chinese Infants with Atopic Dermatitis

### Qi Gao, Li Xiang, Kunling Shen

#### Beijing Children’s Hospital, China

##### **Correspondence:** Qi Gao – Beijing Children’s Hospital, China

**Background:** “Allergy march” is a postulated progression of allergic disease in infants with food allergy associated atopic dermatitis (AD) to subsequently develop asthma and allergic rhinitis (AR), evidence of which were mostly from the European countries. It was unclear how it presents in Chinese children. In this pilot study, we aim to investigate the prevalence of allergic diseases in previous AD infants, to assess the children’s sensitization profiles of inhalant and food allergens in pre-school ages.

**Methods:** During the year of 2008 to 2010, 69 infants were diagnosed with food protein allergy (FPA) associated AD in Beijing Children’s Hospital through clinic. Follow-up of these subjects was performed in their pre-school ages. Medical histories related to allergies during toddler’s and pre-school ages were acquired through questionnaires. Serum specific IgE levels were tested for both food and aeroallergens. Skin prick test of aeroallergens was also performed.

**Results:** The overall follow-up rate was 79.71% (55/69). By pre-school years, these children’s prevalence of physician diagnosed AD after infancy was 41.82%, who were also identified as persistent AD. Prevalence of physician diagnosed AR and asthma were 32.73% and 10.91% respectively. Thirty two children accepted serum IgE level tests. Although all the subjects showed positive test results for food allergens in infancy, only 40.63% remained sensitized to food by pre-school years. Twenty six of the subjects had received screening test for aeroallergens in infancy and all proved negative results, but in pre-school follow-up, 71.88% of the followed subjects were sensitized to aeroallergens. Twenty nine children accepted skin prick test of inhalant allergens, of whom the positive rate was 82.76% (24/29). Respiratory allergic diseases were significantly more prevalent in children with intermediate to severe aeroallergen sensitization than others (P=0.027). Multivariable logistic regression analysis showed that persistent AD was an independent risk factor for airway allergies(OR=10.500, 95%CI: 2.670-41.292, P=0.001).

**Conclusion:** A considerable proportion (34.55%) of FPA associated AD infants develop AR or asthma by pre-school ages. Most of the food sensitized infants will outgrow it 4 or 5 years later, while tend to evolve to aeroallergens sensitization, usually from a negative result in infancy. By pre-school years, children with moderate to severe sensitization to aeroallergens are more vulnerable to respiratory allergic diseases than others. Persistent AD might be an independent risk factor for developing AR or asthma.

## A264 Activation of Toll like Receptor 1 and 6 By House Dust Mite Enhances the Expression of Tight Junction Protein in Epidermal Keratinocytes

### Yong Hyun Jang

#### Kyungpook National University School of Medicine, South Korea

**Background:** The function of innate receptors including toll like receptor (TLR), conventionally recognized for their antimicrobial effects, has recently been extended to include epithelial barrier regulation. TLR 2 activation was shown to aid tight junction (TJ) repair in atopic dermatitis (AD). However, the role of other TLRs in modulating epidermal barrier *in vitro* and *in vivo* remains poorly understood. We investigated the changes of TJ proteins following the activation of TLR 1 and 6 by house dust mite (HDM).

**Methods:** Firstly, the mRNA levels of TLRs and TJ elements were assayed using real-time RCR in epidermal keratinocytes and a murine model with AD-like lesions activated by HDM allergens. The association between specific TLR activation and the changes of certain TJ proteins was studied in TLR deficient mice.

**Results:** Both *in vitro* and *in vivo*, HDM activated TLR 1 and TLR 6. In addition, HDM induced increased gene expression of TJ proteins including claudin-1, claudin-23 and occludin. These results provide the evidence that HDM triggers the activation of pattern recognition receptors such as TLR 1 and TLR 6 and thus stimulates the modulation of TJ proteins. Moreover, gene expression of TJ proteins including claudin-1, claudin-23 and occludin was not increased in knockout mice of TLR1 (*tlr1*^-/-^) and TLR6 (*tlr6*^-/-^).

**Conclusions:** Our data demonstrate that the innate immune activation of the keratinocytes by HDM allergens may lead the preservation of TJ integrity.

## A265 Pollen Exposure in a Mobile Exposure Chamber: Comparing Real-Life Symptoms with Exposure Symptoms

### Karl-Christian Bergmann^1^, Torsten Sehlinger^2^, Georg Bölke^3^, Uwe Berger^4^, Torsten Zuberbier^3^

#### ^1^Allergy-Centre-Charité, Germany; ^2^Bluestone Technology Gmbh, Woerrstadt, Germany; ^3^Charité - Universtaetsmedizin Berlin, Germany; ^4^Medical University of Vienna, Austria

##### **Correspondence:** Karl-Christian Bergmann – Allergy-Centre-Charité, Germany

**Rationale:** As required by the EMA and the US FDA for pivotal trials involving allergen immunotherapy (AIT) products, clinical efficacy assessment is currently based on DBPC field studies with natural allergen exposure. Problems with the field studies include the variability of allergen exposure in different trial sites, the uncertainty of time exposure and confounding environmental factors. A novel mobile Allergen exposure chamber was designed to operate with stable and reproducible allergen exposure under standardized environmental conditions. To be accepted as an appropriate alternative to natural allergen exposure for clinical trials the clinical validation of the Exposure Chamber must document that the symptoms provoked in the chamber reflect the kind and severity of symptoms in real-life.

**Methods:** To determine the pollen-induced allergic nasal, eye and bronchial symptoms and their severity in real life we used data from patients suffering on rhinitis reported by using an electronic “patient hay-fever diary” (PHD) since 2009-2011 in a public app (Pollen App 3.0). For each organ and their symptoms a severity score of from 0 – 3 is possible, resulting in a total symptom score (TSS) of 12. Also, comparisons of symptoms with pollen concentration in their surrounding leading to a “symptom load index” (SLI) were used to define the severity of “real-life symptomatology” in pollen allergic patients. In the chamber birch and grass pollen allergic adults were exposed to different birch and grass pollen loads (4.000 to 8.000) for 90 – 240 min outside the pollen seasons. Spirometry, FeNO and nasal secretion was measured before and after exposure. Nasal, conjunctival and bronchial symptoms were recorded each 10 min, peak-flow and peak nasal inspiratory flow every 30 min.

**Results:** Using > 60.500 data sets from >1.600 PHD users the mean TSS for 2009 to 2011 in Germany was calculated with 3.0 to 5.4 for birch seasons and 3.9 to 4.4 for grass seasons. The SLI (TSS) was calculated on a total of 293,098 data entries ranging for birch season from 4.5 to 5.6 and for grass season from 3.4 to 4.7 points. The repeated exposures with birch and grass pollen with 4000 – 8000 grains/m3 elicited reproducible symptoms on all 3 organs and significant differences between placebo and verum pollen exposure. Generally, the symptoms started to occur after 10 min and reached a plateau following 70 - 90 min of continuous exposure. The TSS for birch pollen reached an average of 5.8 and for grass 6.5 points.

**Conclusions:** Exposures with birch and grass pollen in the mobile exposure chamber allow to induce clinical symptoms in the same kind and severity in patients with allergic rhinitis due to pollen experience in real-life. Therefore, they are an alternative to natural allergen exposure.

## A266 Retrospective Analysis of the Incidence of Allergy in Patients with Contact Eczema

### Joanna Kolodziejczyk, Milena Wojciechowska, Anna Hnatyszyn-Dzikowska, Micha Chojnacki, Zbigniew Bartuzi

#### Nicolaus Copernicus University in Toruñ, Ludwik Rydygier Medical College in Bydgoszcz, Poland

##### **Correspondence:** Joanna Kolodziejczyk – Nicolaus Copernicus University in Toruñ, Ludwik Rydygier Medical College in Bydgoszcz, Poland

**Background**

Contact eczema is a complex polymorphic skin eruptions disease formed on inflammatory, which arises from skin contact with irritants or allergens. In a substantial proportion of patients exist also a contact allergy, however no less is sensitization to inhalant and food allergens. The aim of this study was epidemiological analysis to determine the frequency of contact, inhaled and food allergy in patients with contact eczema.

**Methods**

The main research method in the study was a retrospective analysis of medical records of patients who from January 2012 to December 2013 were registered to allergy clinic to diagnosed skin changes characterized of contact eczema. The analysis included only patients who underwent patch testing.

**Results**

The study analyzed the histories of 79 patients with contact eczema: 61 women in age 18-83 years (mean age 46.5 years) and 18 men in age 22-80 years (mean age 54.3 years). The most common locations of contact eczema were hands (n = 28; 35.4%) and faces (n = 22; 27.8%). Positive patch tests results were obtained in 29 (36.7%) patients, the majority in patients with hands (n = 12; 42.8%) and faces (n = 6; 27.2%) contact eczema. The most common sensitizing haptens were nickel sulfate (n = 16, 55.1%), para-phenylenediamine (n = 13; 44.8%) and potassium dichromate (n = 12; 41.3%). Prick tests with inhalant allergens were performed in 65 patients and with food allergens were performed in 29 patients. In 27 (41.5%) patients were registered positive skin prick tests results to inhaled allergens, in the majority of patients with hands (n = 13; 20%), faces (n = 7; 10.7%) and the upper limb (n = 4; 6.1%) contact eczema. Food allergy was diagnosed in 6 (20.6%) patients, 2 (33%) of patients were with hands, faces and feet contact eczema. Among the inhaled allergens, the most sensitizing were D. Farinae, D. Pteronyssimus and grass, while the most sensitizing food allergens were celery, pepper and flour.

**Conclusions**

The hands and faces are the most common location of contact eczema. In a large proportion of patients with contact eczema occurs contact allergy, especially to metals and para-phenylenediamine. A significant problem for patients with contact eczema are coexist inhalable and food allergies. Because of a high incidence allergies in patients with contact eczema, diagnosis of disease is complicated. Fact of that and various clinical and chronic profiles of disease may decreased quality of life of patients.

## A267 Effect of Fungal Sensitization in Patients with Severe Asthma

### Katsunori Masaki^1^, Koichi Fukunaga^1^, Takashi Kamatani^1^, Kengo Ohtsuka^1^, Takae Tanosaki^1^, Masako Matsusaka^1^, Takao Mochimaru^1^, Hiroki Kabata^1^, Soichiro Ueda^1^, Yusuke Suzuki^1^, Katsuhiko Kamei^2^, Koichiro Asano^3^, Tomoko Betsuyaku^1^

#### ^1^Keio University Hospital, Japan; ^2^Chiba University; ^3^Tokai University School of Medicine

##### **Correspondence:** Katsunori Masaki – Keio University Hospital, Japan

**Rationale:** Fungal exposure triggers asthma exacerbation, and some reports suggest that fungal sensitization and asthma severity are associated.

**Objective:** To investigate the effect of fungal sensitization in severe asthmatic patients.

**Methods:** We collected data from 146 patients with severe asthma for analysis of the following variables: asthma control test (ACT) score, pulmonary function, fractional exhaled nitric oxide (FeNO), and sensitization to fungi (*Aspergillus, Alternaria, Cladosporium, Penicillium, Trichophyton, and Schizophyllum commune*) and non-fungal antigen sources (house dust mites, dogs, cats, cockroaches, moths, and chironomids). Fungal sensitization was diagnosed when an increase in the serum IgE levels specific to these allergens was detected.

**Results:** Eighty-seven patients (60%) were sensitized to one or more antigens; 37 (25%) were sensitized to fungal allergens, the most common of which were *Aspergillus* (22 patients, 15%) and *Trichophyton*(19 patients, 13%). Of the 87 patients, six were only sensitized to fungus (Group F), 50 only to non-fungal allergens (Group N), and 31 to both fungal and non-fungal antigens (Group F + N). The sex distribution, pulmonary function, and dose of inhaled corticosteroid were not significantly different between the three groups; however, the ACT score was significantly lower for Group F + N than for the other groups. The FeNO and the ratio of subjects dependent on oral steroid therapy were higher in Group F + N than in Group N.

**Conclusion:** Fungal sensitization, in addition to non-fungal sensitization, is associated with poor control of asthma in patients with severe asthma.

## A268 SCIg Patient Preference Pump Versus Push

### Karlee Trafford

#### Toronto Allerrgy Immunology Clinic, Canada

**Background**

The administration of subcutaneous Immunoglobulin (SCIg) is a safe and convenient alternative to intravenous immunoglobulin (IVIg) therapy. Administration of SCIg can occur via an infusion pump or rapid push technique. Based on patients at a Canadian site where patients are presented with both decisions and the direct cost differences to patients explained. Ourexperience has shown that the pump has an overwhelming higher preference amongst patients.

**Methods**

This is a chart review of immune deficient patients at a private clinic who initiated treatment with SCIg therapy between January 2014 and April 2015. Patients were given the option to choose which method they desired after being shown both techniques and determining the difference in cost for each method. Their dosage amounts were confirmed, and they were each shown the amount of time it would take for them to infuse via the pump versus rapid push.

**Results**

Charts for the first 25 patients (12 male, 13 female, ranging in age from 40 to 80) were reviewed. Fifteen patients chose the pump and 5 patients chose the push method. Out of the 15 patients, only 2 patients were not covered by insurance. Out of the 5 patients who chose the push method, 3 had to pay out of pocket. The 5 remaining patients were either lost to follow up or switched back to IVIg. Dosage amounts varied in both methods of delivery.

**Conclusion**

The results clearly show that immune deficient patients did have a higher preference for administering SCIg through the pump rather than pushing by hand, and it wasn’t in relation to the time spent, but the overall cost and freedom it granted the patient. This may be a result of more immune deficient patients being diagnosed within the aging population particularly for secondary immune deficiencies.

## A269 Fixed Drug Eruption Induced By Ornidazole and Diclofenac

### Ismet Bulut, Zeynep Ferhan Ozseker

#### Süreyyapasa Chest Disease and Chest Surgery Training and Research Hospital, Turkey

##### **Correspondence:** Ismet Bulut – Süreyyapasa Chest Disease and Chest Surgery Training and Research Hospital, Turkey

**Introduction**

Fixed drug eruption (FDE) is a drug reaction characterized by erythema, edema and sometimes bullae appearing in the same skin or mucosal region due to repeated intake of a drug. Most commonly causative drugs are sulfonamides, barbiturates and nonsteroid antiinflamatory drugs.

Hereby a FDE developing due to intake of ornidazole for the treatment of vaginitis case is presented.

**Case**

27 years old woman applied to our clinic with complaint of purple-violet coloured symmetrical, regular bordered, itchy macular erythematous lesions of 5x3 cm in diameter on dorsum of both hands; and oral lesions. Medical history revealed intake of mixovul (ornidazole) one day prior to onset of complaints. He patient had also experienced similar lezions on skin and oral mucosa 6 months ago, 24 hours after taking ornidazole. The patient had similar symptoms on skin and oral mucosa also after intake of diclofenac.

**Method**

Ornidazole skin prick test was found to be negative. Ornidazole patch test was similarly negative. Prick test and patch test for diclofenac were found to be negative as well.

Amoxicillin- Claculonic acid prick test was done in order to determine an alternative safe antibiotic and it was found as negative too. Oral provocation test did not reveal any early or late onset allergic reaction either. Prick test and oral provocation test with meloxicam was done in order to find an alternative analgesic and these were negative too.

**Result**

With this case, we wanted to point out that ornidazole, a commonly used anti-infection agent, can be the cause of fixed drug eruption in some cases. Also, it should be noted that fixed drug eruptions can occur for multiple drugs in the same patient.

## A270 Transepidermal Water Loss Measurement during Infancy Can Predict the Subsequent Development of Atopic Dermatitis

### Kenta Horimukai^1^, Hideaki Morita^2^, Masami Narita^2^, Hironori Niizeki^2^, Kenji Matsumoto^3^, Yukihiro Ohya^2^, Hirohisa Saito^3^, Shigenori Kabashima^2^, Mai Kondo^2^, Eisuke Inoue^2^

#### ^1^Jikei University Katsushika Medical Center, Japan; ^2^National Center for Child Health and Development; ^3^National Research Institute for Child Health & Development

##### **Correspondence:** Kenta Horimukai – Jikei University Katsushika Medical Center, Japan

**Background:** Atopic dermatitis (AD) is characterized by skin barrier dysfunction. Few studies have used non-invasive skin measurement techniques to measure epidermis function in asymptomatic neonates. We therefore conducted a post-hoc analysis to determine whether skin barrier function in the first week of life predicted AD development and allergen sensitizationby age 32 weeks.

**Methods:** Data of 116 infants collected during our previous randomized controlled study were analyzed. Skin barrier function was measured through transepidermal water loss (TEWL), stratum corneum hydration (SCH), and pH. Study participants were divided into 2 groups based on the results of Cox regression analyses of skin measurement values and cumulative AD incidence by 32 weeks of age. A Kaplan–Meier analysis and log–rank test were used to analyze skin barrier function and cumulative AD incidence. Allergic sensitization based on IgE levels to egg white and ovomucoid at 32 weeks of age was assessed by a Chi–squared test.

**Results:** TEWL (with an optimal cut-off level of 6.5 g/m^2^/h) measured on the forehead within the first week of life showed a lower p-value than TEWL measured on the lower leg, SCH, and pH measurements. We found a significant difference in cumulative AD incidence between the high and low TEWL groups (p <0.05). Moreover, the high TEWL group exhibited a higher (but not statistically significant) rate of sensitization to ovomucoid (p = 0.07).

**Conclusions:** TEWL measured on the forehead during the first week of life is associated with AD development.

## A271 Inhalant Allergens on Soft Toys: A Literature Review

### Robert Siebers^1^, Francis F. S. Wu^2^

#### ^1^University of Otago, New Zealand; ^2^Chung Chou University of Science and Technology, Taiwan

##### **Correspondence:** Robert Siebers – University of Otago, New Zealand

**Background:** Inhalant allergens in the indoor environment are associated with sensitization and asthma symptoms exacerbations. Children’s soft toys can harbor allergens. The purpose of the study was to review the literature regarding inhalant allergens from soft toys.

**Methods:** Major data bases were searched for the terms allergens and toys. Excluded were articles dealing with fragrance or contact allergens.

**Results:** From 1982 to present there have been six published studies presenting allergen levels on soft toys. In all of these allergens from house dust mites (Der p 1 and Der f 1) were measured with widely different levels. Lower levels were found in soft toys from child centres. Two of the studies presented dog (Can f 1) and cat (Fel d 1) allergen levels with one study finding that if there was a dog or cat in the home this resulted in higher levels of Can f 1 or Fel d 1. One presented cockroach allergen (Bla g 1 and Bla g 2) levels, albeit at very low levels. Factors associated with allergen levels on soft toys included age of the toys, location, cleaning activities and presence of pets.

**Conclusions:** Given their wide spread use by infants, soft toys are an important source of inhalant allergen exposure that needs to be taken into account in epidemiological studies and more research is required to find exposure reduction techniques.

## A272 Fractional Exhaled Nitric Oxide in Elderly Asthmatics

### Robert Siebers^1^, Francis F. S. Wu^2^, Ming-Hui Ting^2^, Hung-En Laio^3^, Tsung-Huai Kuo^4^, Pei-Yuan Lee^4^

#### ^1^University of Otago, New Zealand; ^2^Chung Chou University of Science and Technology, Taiwan; ^3^Asia University, Taiwan; ^4^Show Chwan Memorial Hospital, Taiwan

##### **Correspondence:** Robert Siebers – University of Otago, New Zealand

**Background:** Fractional exhaled nitric oxide (FeNO) is increasingly used to non-invasively assess airway inflammation. Little is known regarding eNO and its determinants in the elderly. This study assessed eNO and atopy in elderly asthmatics.

**Methods:** Fifty elderly asthmatics (24 female; 26 male) aged 55 to 83 years (mean: 63.8) were consecutively recruited from a respiratory outpatient clinic. 39/50 were on inhaled steroids. eNO was measured with a NIOX MINO analyzer and specific IgE (sIgE) to inhalant and food allergens measured in blood by fluorescence enzyme immunoassay (Phadia 100). Atopy was defined as any sIgE ≥ 0.35 KU/L. Results are presented as geometric mean levels with 95% confidence intervals (95% CI). Comparisons were by Mann-Whitney U-test with significance set at the p 0.05 level.

**Results:** Twenty subjects were atopic with 14/20 atopic to house dust mite (HDM). Geometric mean FeNO level (95% CI) for all 50 subjects was 23.1 ppb (95%CI: 18.1-29.3) with no significant differences between females and males. FeNO levels were significantly higher in atopic vs non-atopic subjects (33.4 ppb, 95% CI: 21.2-52.6 vs 18.3, 95% CI: 14.4-23.4; p=0.003). FeNO geometric mean level in HDM-sensitized subjects (n=14) was 38.8 ppb (95% CI: 22.9-65.8) while in non-HDM sensitized atopics (n=6) this was 23.5 ppb (95% CI: 7.4-74.1).

**Conclusions:** Atopy in elderly asthmatics in Taiwan is associated with significantly higher FeNO levels predominantly driven by sensitization to HDM.

## A273 Dye and Preservative Challenge in Meal-Associated Urticaria and Angioedema: A Low-Yield Diagnostic Maneuver

### Daniel Eugene Maddox

#### Mayo Clinic, USA

**Background:** Juhlin has attempted to estimate the incidence of urticaria in the Swedish population, coming up with an estimated 0.1% incidence, based on an examination of 36,000 persons. Goodman et. al. reported urticaria triggered by the food preservatives butylated hydroxyanisole [BHA] and butylated hydroxytoluene [BHT]. Many reports have been made regarding dye reactions. Therefore, some subset of the urticaria sufferers are thought to be triggered by exposure to dyes or preservatives commonly used in foods and beverages.

**Method:** Since the Allergic Diseases Clinical Laboratory was computerized in 1991, creating a searchable database for all laboratory patient investigations, we have performed 210 placebo-controlled dye and preservative challenges in 202 individual patients [193 adults, 9 children] presenting primarily with urticaria, angioedema, and meal-related gastrointestinal symptoms. The challenges were carried out by first presenting a series of blinded liquid doses, some of which contained a mixture of FD&C Blue #1 and Blue #2, Red #3 and Red #40, and Yellow #5 & #6. The initial dye dosing was with 0.2% solutions, and if these were negative, 2% solutions were then presented. If there was no reaction to the dye solutions and placebos, we then administered a mixture of preservative compounds [sodium benzoate, p-hydroxybenzoic acid, butylated hydroxyanisole, and butylated hydroxytoluene] or placebos in capsule form, with early dosing at 4 capsules of 50 mg of the preservative mix, and later dosing with 4 capsules of 250 mg of the preservative mix.

**Results:** We documented only 4 positive challenges in this series of 210, and two of those were not reproducible in repeat challenges conducted on subsequent days. There were 40 placebo responders. Three of the four positives appeared to be related to the dyes. In the one dye-reactor that was reproducible, the causative dye appeared to be one of the two blue dyes.

**Conclusions:** We conclude that taken together, dyes and preservatives are frequently suspected but rarely actually participate as triggers of urticaria and angioedema.

## A274 The Changes of Allergic Sensitization with Age in Children with Allergic Rhinitis

### Gwanghui Ryu, Hyo Yeol Kim, Hun-Jong Dhong, Sang Duk Hong, Seung-Kyu Chung

#### Samsung Medical Center, South Korea

##### **Correspondence:** Gwanghui Ryu – Samsung Medical Center, South Korea

**Background and Objectives:** Allergic disease in children has diverse characteristics & sensitization with age because of allergen, pathophysiologic and immunologic differences. There have been few reports regarding the changes of allergic sensitization with age in children in South Korea, so we aimed to find out these changes by CAP test. **Materials and Methods:** A total of 8994 children (6238 boys, 2756 girls, mean 10.5 years, range from 5 months to 18 years) with allergic rhinitis were subjected to this study. They were classified by age groups into ≤3, 4~6, 7~9, 10~12, 13~15 and 16~18 year-old. All children underwent the CAP test (Fluoroenzyme immunoassay) about two subtypes of house dust mites (*D. pteronyssinus, D. farinae*), cat, dog, tree mixture and weed mixture allergens. In addition, allergens were classified into indoor, outdoor, or both. We analyzed age-based differences according to the types of allergens. Also we analyzed the total IgE. **Results:** CAP test analysis of allergic rhinitis patients in children showed that the age group 10-12 had higher titer to house dust mite allergens (DP 18.40±27.64 U/ml, DF 30.56±27.62U/ml) than other groups and the highest degree of positive reaction to multiple allergens (p<0.001). Age under 10 groups (≤3, 4~6, 7~9) were revealed significant difference of *D. pteronyssinus* IgE titer among other groups (p<0.001). Age over 10 groups (10~12, 13~15, 16~18) showed difference with age under 10 year-old groups (p<0.001) but no difference in each other (p=1.0, 0.984, 0.967) with Kruskal-Wallis test and post-hoc Tukey test. Same results were obtained in the *D. farinae* test. Sensitization ratio to weed and tree allergens tended to increase with age, while sensitization ratio of cat and dog did not present significant differences according to age. Certain tendencies depending on types of allergens were found - sensitization by indoor allergens were high but did not indicate age-based differences, while sensitization by outdoor allergens tended to grow with age. In addition, total lgE presented difference between age under 7 groups (≤3, 4~6) and age over 7 groups (7~9, 10~12, 13~15, 16~18) with statistical significance (p<0.001) and increased with age in general, with boys showing significantly higher lgE titer than girls (p<0.001). **Conclusion:** The sensitization in children with allergic rhinitis was changed with age in some allergens by CAP test.

## A275 Component-Specific IgE and IgG4 Levels in Milk Allergy Children Tolerated Baked Milk Products

### Osamu Higuchi^2^, Yu-Ichi Adachi^3^, Toshiko Itazawa^4^, Yoko Adachi^4^, Miki Hamamichi^4^, Motokazu Nakabayashi^4^, Yasunori Ito^4^, Takuya Wada^4^, Gyoukei Murakami^5^, Miki Takao^6^, Junko Yamamoto^7^

#### ^1^Koseiren Takaoka Hospital; ^2^University of Toyama, Japan; ^3^Toyama University School of Medicine; ^4^University of Toyama; ^5^Murakami Allergy and Pediatric^6^; Takashige Memorial Hospital; ^7^Saiseikai Takaoka Hospital

##### **Correspondence:** Osamu Higuchi – Koseiren Takaoka Hospital

**Background:** It was recently reported that a substantial percentage of milk allergy children tolerate baked milk products. However, little has been known the roles of milk component-specific IgE and InG4 in the tolerance to baked milk.

**Methods:** Milk, casein, alpha-lactoalbumin and beta-lactoglobulin-specific IgE and IgG4 antibodies were measured by ALLASTAT 3G system in milk allergy children who performed oral baked milk challenge.

**Results:** Out of 19 children aged 2-20 years old, 9 (47%) tolerated baked milk product (50mL containing muffin) and 10 reacted. There were no significant differences in mean age, gender and history of milk-induced anaphylaxis between two groups. In children tolerated baked milk, specific IgE titers to milk, casein and alpha-lactoalbumin were significantly lower, but not to beta-lactoglobulin. Furthermore, each component-specific IgE/IgG4 ratio was significantly lower compared to children who reactive to baked milk.

**Conclusions:** Approximately a half of milk allergy children can tolerate baked milk product. Component-specific IgE and IgG4 titers could be useful as a predictor for tolerability of baked milk.

## A276 Serum Surfactant Protein(SP)-D Level: A Potential Biomarker for Aspirin-Exacerbated Respiratory Disease

### Hyun Jung Jin, Moon Gyeong Yoon, Young Min Ye, Yoo-Seob Shin, Seung-Hyun Kim, Hae-Sim Park

#### University School of Medicine

##### **Correspondence:** Hyun Jung Jin – College of Medicine, Yeungnam University, South Korea

**Background:** SP-D is a hydrophilic lectin and involves in a host defense mechanism against respiratory pathogens and modulates immune responses against bacteria or allergens. 

**Purpose:** The aim of this study was to evaluate serum SP-D level as a potential biomarker for aspirin-exacerbated respiratory disease (AERD) compared to aspirin tolerant asthma (ATA) patients. 

**Subjects and methods:** Ninety-three subjects with AERD, 172 ATA and 75 healthy controls were enrolled at Ajou University Hospital (Suwon, Korea). Serum SP-D levels were measured using commercially available ELISA kit. 

**Results:** The SP-D levels in sera of AERD patients were significantly lower than those of ATA and control groups. The ROC curve indicated that SP-D at 1755.82pg/ml can be an optimal cutoff value for predicting the phenotype of AERD with 64.3% sensitivity and 62.4% specificity. No associations were found with clinical parameters such as atopy, chronic rhino-sinusitis, eosinophilic/neutrophilic asthma or severe/non-severe asthma. A significant negative correlation was found between serum SP-D level and the fall of FEV1 (%) after inhaled lysine-ASA in asthmatic patients. 

**Conclusion:** These findings suggest that SP-D may involve in the pro-inflammatory response of airway mucosa which contribute to bronchoconstrictive response to inhaled ASA in AERD patients.

## A277 Clinical Characteristics of Anaphylaxis in Korean Childrens

### Taek-Ki Min^1^, Bok-Yang Pyun^1^, So-Yeon Lee^2^, Hyun Hee Kim^3^, Gwang-Cheon Jang^4^, Jinho Yu^5^, Dongin Suh^6^, Sooyoung Lee^7^, Yong Mean Park^8^, Jeong Hee Kim^9^, Hye-Yung Yum^10^, Kyung Won Kim^11^, Hyeon-Jong Yang^1^, Kangmo Ahn^12^, Ji-Won Kwon^13^, Myung Hyun Sohn^11^, Hae Ran Lee^14^, Jung Hyun Kwon^15^, Kyu-Earn Kim^11^, Soo-Jong Hong^16^

#### ^1^Soonchunhyang University College of Medicine, South Korea; ^2^Hallym University Sacred Heart Hospital; ^3^The Catholic University of Korea; ^4^NHIS Ilsan Hospital; ^5^Asan Medical Center Seoul Korea; ^6^Seoul National University Hospital; ^7^Ajou University School of Medicine; ^8^Konkuk University School of Medicine; ^9^Inha University Hospital, Incheon; ^10^Seoul Medical Center; ^11^Yonsei University College of Medicine; ^12^Samsung Medical Center; ^13^Seoul National University Bundang Hospital; ^14^Hallym University Medical Center; ^15^Department of Pediatrics, Korea University College of Medicine, Seoul, Korea; ^16^Research Center for Standardization of Allergic Diseases, University

##### **Correspondence:** Taek-Ki Min – Soonchunhyang University College of Medicine, South Korea

**Background:** The incidence of anaphylaxis has been increasing in western countries. Although anaphylaxis is recognized as an important life-threatening condition, no nationwide report has yet investigated the clinical features of anaphylaxis in Korean children.

**Objective:** We sought to investigate the clinical features of anaphylaxis in Korean children.

**Methods:** A retrospective medical record review was performed on children patients diagnosed with anaphylaxis between 2009 and 2013 in 23 tertiary hospitals of South Korea.

**Results:** A total 991 cases (66% male, mean age 5.89±5,24) were reported, with 36.6% below 2 years of age. The most common past medical history was food allergy. A previous reaction to the same allergen was reported by 23.1%. Cutaneous symptoms (96.1%) were the most common symptoms followed by respiratory (85.0%), gastrointestinal (26.6%), neurologic (15.5%), and cardiovascular (14.3%). Although, cardiovascular symptoms of anaphylaxis were rare in young children (7.7%), cardiovascular symptoms were increased with age. Cardiovascular symptoms were common in reactions caused by insect sting(50.0%). The mainstay of first-line treatment in hospital included antihistamine (61.2%), corticosteroids (42.2%) and epinephrine(34.0%). Epinephrine auto-injectors were prescribed for 26.6%.

**Conclusion:** We identified differences in the symptoms of anaphylaxis according to age and triggers. Rate of use of epinephrine as the first-aid medication is too low, and prescription rate of adrenalin auto-injector is too low regardless of age of patients.

## A278 Immunological Changes Induced By Intramuscular Injections of Autolologous Immunoglobulin in Patients with Severe Atopic Dermatitis

### Su-Mi Cho

#### Ajou University Hospital, South Korea

**Background:** The management of patients with severe atopic dermatitis (AD) is frequently difficult for both patients and physicians. We previously reported that repeated intramuscular injections of autologous immunoglobulin (autologous immunoglobulin therapy) induced significant clinical improvements in patients with severe AD. To evaluate a therapeutic mechanism of autologous immunoglobulin therapy, we analyzed the changes of serum concentrations of total IgE and allergen-specific IgE in patients with severe AD before and after treatment.

**Methods:** Sixteen adult patients with severe AD sensitized to house dust (HDM) mite were treated by intramuscular injections of 50 mg autologous immunoglobulin (mainly IgG with purity ≥ 97%) twice a week for 4 weeks. Autologous immunoglobulin was purified from autologous plasma by affinity chromatography using Protein A. The serum concentrations of total IgE, specific IgE to *Dermatophagoides farinae* (*D. farina*e), and specific IgE to recombinant Der f 2 (rDer f 2) at baseline, 4, 8, and 12 weeks were measured using enzyme-linked immunosorbent assay.

**Results:** The serum concentrations of total IgE significantly decreased from 25678.9 ± 22826.4 kU/L (mean ± SD) at baseline to 22361.4 ± 23020.4 kU/L at 4 weeks, 21306.1 ± 20585.7 kU/L at 8 weeks, and 18143.8 ± 16852.4 kU/L at 12 weeks (Wilcoxon signed-rank test, P < 0.05). The serum concentrations of specific IgE to *D. farinae* significantly decreased from 503.3 ± 885.5 kU/L at baseline to 282.1 ± 415.7 kU/L at 4 weeks, 227.8 ± 378.7 kU/L at 8 weeks, and 191.7 ± 294.2 kU/L at 12 weeks (P < 0.05). The serum concentrations of specific IgE to rDer f 2 significantly decreased from 47.9 ± 47.6 kU/L at baseline to 34.4 ± 35.5 kU/L at 8 weeks, 30.2 ± 28.0 kU/L at 12 weeks (P < 0.05).

**Conclusions:** Repeated intramuscular injections of autologous immunoglobulin significantly decreased serum concentrations of total IgE and allergen-specific IgE antibodies in patients with severe AD.

**Reference**

Nahm DH, Cho SM, Kim ME, Kim YJ, Jeon SY. Autologous immunoglobulin therapy in patients with severe recalcitrant atopic dermatitis: a preliminary report. Allergy Asthma Immunol Res 2014;6:89-94

## A279 Identification of Subtypes in Subjects with Mild to Moderate Airflow Limitation and Their Clinical and Socioeconomic Implications

### Jin Hwa Lee^1^, Chin Kook Rhee^2^, Hye Yun Park^3^, Woo Jin Kim^4^, Yong Bum Park^5^, Kwang-Ha Yoo^6^

#### ^1^Ewha Womans University School of Medicine, South Korea; ^2^Seoul St. Mary’s Hospital; ^3^Sungkyunkwan University School of Medicine; ^4^Environmental Health Center, Kangwon National University Hospital; ^5^Hallym University Kangdong Sacred Heart Hospital; ^6^Konkuk University College of Medicine

##### **Correspondence:** Jin Hwa Lee – Ewha Womans University School of Medicine, South Korea

**Background:** Asthma and chronic obstructive pulmonary disease (COPD) are the most common airway diseases. Both diseases share clinical features, and each disease shows phenotypic heterogeneity. Even patients with relatively preserved lung function can experience frequent exacerbation linked to poor quality of life. The aim of this study was to identify subtypes in patients with mild to moderate airflow limitation and to evaluate their clinical and socioeconomic implications.

**Methods:** We analyzed data from the fourth Korean National Health and Nutrition Examination Survey and National Health Insurance claims in 2007-2012. Subjects who were 19 years old and more and had forced expiratory volume in 1 second (FEV_1_) ≥ 60% predicted and a ratio of FEV_1_ to forced vital capacity (FVC) < 0.7 were included. K-means clustering was performed to explore subtypes. For clustering analysis, six key input variables, age, body mass index (BMI), FEV_1_% predicted, the presence or absence of self-reported wheezing, smoking status, and pack-years of smoking were selected.

**Results:** Among a total of 2,140 subjects, five subgroups identified through k-means clustering include putative “near-normal (n=232)”, “asthmatic (n=392)”, “COPD (n=37)”, “asthma-overlap (n=893)” and “COPD-overlap (n=586)” subtypes. Among five subgropus, near-normal subgroup showed the oldest mean age (72±7 years) and the highest FEV_1_ (102±8% predicted), and asthmatic subgroup was the youngest (46±9 years). Asthma-overlap subgroup had the lowest FEV_1_ (77±9% predicted). COPD and COPD-overlap subgroups were male-predominant (100% and 98%, respectively) and all current or ex-smokers. When applying the lower limit of normal FEV_1_/FVC as a criterion for airway obstruction, asthma group showed the highest prevalence of airway obstruction. While COPD, asthma-overlap and COPD-overlap subgroups showed high prescription rate of respiratory medicine, asthmatic subgroup had the lowest prescription rate despite the highest proportion of self-reported wheezing. Except asthmatic subgroup, comorbidities such as hypertension, diabetes mellitus, hyperlipidemia and coronary artery disease were frequently observed. Although COPD subgroup represents only 2% of total subjects, they showed the highest mean medical cost and health utilization, comprising 5% of the total cost. When calculating a ratio of total medical expense to household income, mean ratio was the highest in COPD subgroup.

**Conclusion:** Subjects with mild to moderate airflow limitation exhibited clinical and epidemiological heterogeneity. Each subgroup may have a different level of demand for health resources.

## A280 Cephalosporin-Induced Dress (Drug Rash with Eosinophilia and Systemic Symptoms) Syndrome in a 7-Year-Old Boy

### Heejeong Kang, Hyeon-Jong Yang, Taek-Ki Min, Bok-Yang Pyun

#### Soonchunhyang University College of Medicine, South Korea

##### **Correspondence:** Heejeong Kang – Soonchunhyang University College of Medicine, South Korea

A 7-year-old boy with acute osteomyelitis in right distal tibia, was treated by the combination of intravenous (IV) vancomycin and cefotaxime. He was tolerable for the given treatment and rapidly improved. Persistent fever had occurred from the day 17th with pruritic, maculopapular rash on day 19^th^. Laboratory test performed on day 20^th^ revealed marked eosinophilia. Under suspicion of DRESS syndrome, both IV antibiotics were stopped and oral prednisolone was given. Three days after the cessation of the antibiotics, fever and skin manifestations had rapidly improved. However, as soon as we retried IV cefotaxime, his skin manifestation had immediately flared up. Intradermal test performed at 6 weeks after complete-recovery, was positive for cefotaxime.

## A281 Maternal Depression Is Associated with Children’s Asthma: An Analysis of the Fifth Korea National Health and Nutrition Examination Survey (2010-2012)

### Lee Ju Suk^1^, Cheol Hong Kim^2^

#### ^1^Samsung Changwon Hospital, South Korea; ^2^International St. Marry’s Hospital

##### **Correspondence:** Lee Ju Suk – Samsung Changwon Hospital, South Korea

**Purpose:** Asthma and atopic dermatitis are common chronic diseases and depression is an important comorbidity in allergic diseases. However, it is little known about the association between maternal depression and child allergic diseases. This study was performed to find the association between maternal depression and child allergic diseases.

**Methods:** Data were acquired from 4695 family who participated in the Fifth Korea National Health and Nutrition Examination Surveys (KNHANES), which was conducted from 2010 to 2012.

**Results:** The prevalence of childhood asthma was 5.3 % and childhood atopic dermatitis was 14.1%. the prevalence of maternal depression was 3.6 %. In univariated analysis, maternal depressions was associated with single mother, low economic state, maternal asthma, atopic dermatitis, children’s asthma. (*p*<0.05) but sex, age, education status and smoking were not associated with the presence of maternal depression. After adjustment, maternal depression were associated with lower house income, maternal asthma, maternal atopic dermatitis, children’s asthma.

**Conclusion:** The present study showed that maternal depression is associated with children’s asthma and not children’s atopic dermatitis. These results warrant future studies to explore the mechanisms responsible for the association between maternal depression and children’s asthma.

## A282 Increased Length of Hospitalization Associated with Infiltration on Chest Radiography in Pediatric Asthma Patients

### Jung Hyun Kwon, Sang Hyun Lee, Wonhee Seo

#### Department of Pediatrics, Korea University College of Medicine, Seoul, Korea

##### **Correspondence:** Jung Hyun Kwon – Department of Pediatrics, Korea University College of Medicine, Seoul, Korea

**Purpose:** Childhood asthma is common reason for the hospitalization and the deterioration of asthma accompanied with respiratory infection. The burden of treatment asthma is high around the globe also in Asia and the assessment of asthma exacerbation in children is difficult and limited. We aimed to investigate the factors associated to hospitalization period in those were inpatients of pediatric asthma.

**Methods:** We conducted the chart review of subjects admitted with asthma exacerbation from 2009 to 2014, retrospectively. The subjects consisted of the patients at the age under 18 years visit a single tertiary Hospital. We investigated the characteristics of those patients including clinical symptom and laboratory tests (serum total immunoglobulin (Ig) E, eosinophil counts). We assess the severity of asthma by Pulmonary Score (PS). We assessed the duration of hospitalization to related factors by using bivariate correlation analysis and multiple linear regression analysis using SPSS 20.

**Results:** A total of 357 subjects with 232 (65.3%) boys and the median age was 5.77 ± 6.1 years old. The mean of hospitalization period were 4.0 ± 1.9 days. The rate of total IgE over than 45 IU/mL was 66.9% and eosinophil count over than 470 /μL was 26.6%. The mean PS was 3.5 ± 1.1 days. The infiltration of chest radiography were 74 cases (20.7%) and body temperature over 38.3 °C were 91(25.5%). The duration of hospitalization was correlated with age (p=0.036, r=0.11). In simple linear regression, PS score positively related with the duration of hospitalization but was not significant (β=0.127, R^2^=0.037, p=0.16). In multiple linear regression model, the duration of hospitalization was positively related the infiltration finding of chest radiography (β=0.962, p=0.008) and age (β=0.082, p=0.03) with R^2^=0.052, independent of fever, c-reactive protein, PS, past history of allergy, family history of allergy, elevated IgE and eosinophil count, multiple allergen sensitization and past history of visiting or admitt from asthma exacerbation.

**Conclusions:** Hospitalization period in pediatric asthma patients has positively correlated with the infiltration finding of chest radiography and no association with PS, total IgE and eosinophil counts. It could be implied that the prediction of morbidity of asthma exacerbation in children has to include the findings on chest radiography that presents respiratory infection. Also, new asthma severity score system for hospitalization of pediatric asthma patients would be needed.

## A283 A Case of 16-Year-Old Boy with Smoking-Induced Acute Eosinophilic Pneumonia

### Kang-in Kim, Young Cheon Park, Hyeon-Jong Yang, Taek-Ki Min, Bok-Yang Pyun

#### Soonchunhyang University College of Medicine, South Korea

##### **Correspondence:** Kang-in Kim – Soonchunhyang University College of Medicine, South Korea

Acute eosinophilic pneumonia (AEP) is a rare disease characterized by acute febrile respiratory insufficiency, marked eosinophilia in bronchoalveolar lavage fluid (BALF), diffuse bilateral and thickening of interlobular septa on chest radiographic findings. Pathogenesis is not well understood, however direct or indirect exposure to smoke has been repeatedly reported as a cause of AEP. We present a case of 16-year-old boy, who developed an idiopathic AEP with no peripheral eosinophilia, marked eosinophilia in BALF (36.6%), decreased DLco (75%), typical radiologic findings, and dramatically improved after corticosteroid treatment without use of antibiotics. Despite best efforts, no evidence of infection was found. Although he initially denied a current smoking, we repeatedly asked the previous history of smoking cigarettes. He finally told the truth that he had started to smoke 19 days before hospitalization in an enclosed area with friends, and gradually increased smoking-dose. Conclusively, we suggest that no peripheral eosinophilia does not exclude an AEP, and clinical suspicion with exact history taking and radiologic findings, and early BALF exam will be the best approach to avoid use of unnecessary antibiotics and for the proper management.

## A284 A Case of Pranlukast Induced Anaphylactic Shock

### Sujeong Kim, Sun Jin, Jong-Myung Lee, Hye-Jin Jung, Jung-Wha Park

#### Kyungpook National University School of Medicine, South Korea

##### **Correspondence:** Sujeong Kim – Kyungpook National University School of Medicine, South Korea

Leukotriene receptor antagonists have been increasingly used in the treatment of a variety of allergic diseases such as asthma, allergic rhinitis, chronic urticaria, and others and they are generally considered safe drugs with few adverse drug reactions. Type I hypersensitivity to montelukast has been rarely reported, with only a handful of anaphylaxis cases being reported worldwide. There is a lack of information about the severe adverse drug reaction associated with pranlukast. We experienced an extremely rare case of severe hypersensitivity reaction associated with pranlukast. A 65-year-old woman developed anaphylactic shock presented with generalized urticaria, angioedema, collapse and loss of consciousness after taking pranlukast, and which was confirmed by oral challenge test and skin prick test. The present case reminds that pranlukast can induce anaphylaxis, which is mediated by IgE-dependent pathway.

## A285 Comparison of Asthma-Related Outcomes Between Metabolically Healthy Obese and Metabolically Unhealthy Obese Asthma Patients

### Hyo-Jung Kim^1^, Tae-Bum Kim^1^, You Sook Cho^1^, Hee-Bom Moon^1^, Hyouk-Soo Kwon^1^, So Young Park^1^, So-Young Park^1^, Jung-Hyun Kim^1^, Bomi Seo^1^, Min-Gu Kim^1^, Youn Yee Kim^2^

#### ^1^Asan Medical Center, University of Ulsan College of Medicine, South Korea; ^2^Seoul Metropolitan Dong-Bu Hospital

##### **Correspondence:** Hyo-Jung Kim – Asan Medical Center, University of Ulsan College of Medicine, South Korea

**Background**

Obesity is a known risk factor for the development of asthma and obese asthmatics are prone to be severe and poorly controlled. Obesity is also associated with increased risk of various metabolic diseases but recent studies suggested that not all obese subjects are at increased risk of such diseases and the ‘metabolically healthy obese’ (MHO) phenotype may exist in the absence of metabolic abnormalities. Due to the paucity of data regarding asthma-related outcomes of obese asthmatics according to their metabolic status, we aimed to investigate the differences between MHO and metabolically unhealthy obese (MUO) asthmatics in terms of asthma-related clinical outcomes.

**Materials and Methods**

A total of 206 patients who were diagnosed with asthma in Asan Medical Center asthma cohort from June 2005 to Mar 2013 were subject to analysis, after sorting out patients whose BMI were ≥25. We divided these patients into two groups, MHO asthmatics (n=106) and MUO asthmatics (n=100), referring to the NCEP ATP III identification of the metabolic syndrome. Then we compared asthma-related clinical characteristics between these two groups.

**Results**

The patients in MUO asthma group had older age (60.9 ±10.3 *vs* 54.7 ±13.6, *p-value* <0.001) and lower FEV1 % predicted (79.2 ±20.9 *vs* 73.0 ±19.6, *p-value*= 0.03) compared with MHO asthmatics. Also, MUO asthmatics had higher frequency to develop severe persistent asthma (20.7 % *vs* 36.0 %, *p-value*= 0.02). Moreover, MUO asthma group showed lower mean FEV1 % predicted value for 1year and 3years follow-up after enrollment and used higher mean ICS dosage compared at 3 years after enrollment.

**Conclusions**

Obese asthmatics demonstrated different clinical characteristics according to their metabolic status, as MUO asthmatics were older with lower pulmonary function and higher tendency to develop severe asthma at enrollment and longitudinal follow-up. This study suggests that different therapeutic approach should be implemented for obese asthma patients according to subtype of obesity.

## A286 Rick Factors Associated with Longer Length of Stay in Infants Hospitalized with Respiratory Syncytial Virus Bronchiolitis

### Yena Lee^1^, Taek-Ki Min^1^, Hyeon-Jong Yang^1^, Bok-Yang Pyun^1^, Suk Hee Han^1^, Suyeon Park^2^, Jeongho Lee^1^, Won-Ho Hahn^1^

#### ^1^Soonchunhyang University College of Medicine, South Korea; ^2^Soonchunhyang Medical Center

##### **Correspondence:** Yena Lee – Soonchunhyang University College of Medicine, South Korea

**Background**

The aim is to identify risk factors associated with longer length of hospital stay (LOS) in infants with respiratory syncytial virus (RSV) bronchiolitis.

**Methods**

We conducted an observational study in infants, under 24-month of age, hospitalized with first episode of RSV bronchiolitis between November 2014 and February 2015, retrospectively. Demographic and clinical characteristics were collected including doctor diagnosed atopic dermatitis, days of illness before hospitalization, 25-hydroxy Vitamin D (25(OH)D), modified-Tal score, RSV genomic load at presentation, and LOS by comprehensive review of medical record. Kaplan-Meier estimator, survival analysis, and Cox proportional hazard analysis were used to identify risk factors for the longer LOS.

**Results**

Total 112 infants were identified, and 6 of extreme premature birth and 3 of congenital heart disease were excluded. The age of months distributed from 0 to 23 month of age (median, 10.5). The median LOS was 94 hours (range 20-224 hours), and those of modified-Tal score was 2 point (range 1-9). Higher RSV genomic load (threshold cycle < 13.68) was not associated with longer LOS (*P*=.1). Lower 25(OH)D (<10 mg/dL) had a higher cumulative incidence of longer LOS (≥96 hour; *P*=.05, ≥72 hour; *P*=.04): the adjusted hazard ratio for the lower 25(OH)D was 0.07 (95% CI, 0.91-15.40, *P*=.07). Mode of delivery, exclusive breast-milk feeding, sex, co-infection, doctor diagnosed atopic dermatitis, and modified Tal-score were not associated with longer LOS.

**Conclusion**

The concurrent hypovitaminosis D in infants with first episode of RSV bronchiolitis, rather than RSV viral load had a higher risk for longer LOS.

## A287 Urinary Excretion of 9Î±, 11Î^2^-Prostaglandin F_2_ and Leukotriene E_4_ in Patients with Exercise-Induced Bronchoconstriction

### Youhoon Jeon, Joo-Hee Kim, Tae-Rim Shin, Cheol-Hong Kim, In-Gyu Hyun, Jeong-Hee Choi

#### Hallym University, College of Medicine

##### **Correspondence:** Youhoon Jeon – Dongtan Sacred Heart Hospital, College of Medicine, Hallym University, South Korea

**Background:** Increased levels of mast-cell-derived eicosanoids, such as prostaglandin (PG)D_2_ and cysteinyl leukotrienes (CysLTs), have been reported in patients with exercise-induced bronchoconstriction (EIB), suggesting that mast cell activation is involved in the mechanism of EIB. However, it is still controversial since these results have not been reproduced in other studies. The aim of this study was to evaluate the role of PGD_2_ and LTE_4_ in adult asthma with EIB, as measuring urinary levels of their metabolites - 9α,11β-PGF_2_ and LTE_4_ before and after an exercise challenge test.

**Methods:** Eight patients with asthma and EIB and five normal controls without EIB were enrolled. Exercise challenge tests comprised of 6 min of treadmill exercise or free running were performed in all study subjects, and urine samples before and 1 h after the challenge were collected. Urinary levels of 9α,11β-PGF_2_ and LTE_4_ were measured by enzyme immunoassay.

**Results:** No significant differences were observed in 9α,11β-PGF_2_ and LTE_4_ levels before/after the exercise challenge between patients with EIB and normal controls. No significant increases in urinary levels of 9α,11β-PGF_2_ or LTE_4_ were detected during the exercise challenge in patients with EIB and normal controls. No significant correlations were observed between the percent decrease in forced expiratory volume in 1 second or percent changes in 9α,11β-PGF_2_ and LTE_4_ levels after the exercise challenge.

**Conclusions:** Urinary 9α,11β-PGF_2_ and LTE_4_ levels did not increase after an exercise challenge in patients with EIB, suggesting that urinary excretion of 9α,11β-PGF_2_ and LTE_4_ may not be a good marker of mast cell activation in patients with EIB.

*** This study was supported by a grant from the Basic Science Research Program through the National Research Foundation of Korea (NRF), as funded by the Ministry of Education, Science, and Technology (2012R1A1A1012349).

## A288 The Aeroallergen Sensitization Pattern and Effect on Airway Hyperresponsiveness in Busan, Korea

### Sun-Mi Jang, Hae-Jung Na, Seung-Eon Song, Hye-Kyung Park, Eun-Jung Jo

#### Pusan National University Hospital, South Korea

##### **Correspondence:** Sun-Mi Jang – Pusan National University Hospital, South Korea

**Background:** Ambient components such as aeroallergen and particulate matter affect respiratory diseases, their compositions are different depending on the region. We investigated aeroallergen sensitization pattern and the effect on airway hyperresponsiveness (AHR) and airway inflammation considering particulate matter and meteorological factors in Busan facing the sea unlike other metropolitan cities in Korea.

**Methods:** We reviewed retrospectively the clinical data of subjects who underwent skin prick tests to aeroallergens, induced sputum analysis, and methacholine bronchial provocation test between February 2011 and August 2014 to evaluate their respiratory symptoms. Age was categorized as group I (15-64 yr) and group II (≥65 yr)

**Results:** A total of 863 subjects were analyzed (mean age: 48.4±15.1, female 63.5%). AHR was demonstrated in 10.5%; sputum eosinophilia, in 12.4%; and sensitization to at least one aeroallergen in skin tests, in 30.6%. Their diagnoses were asthma (31.1%), allergic rhinitis (37.1%), non-allergic rhinitis (7.0%), chronic rhinosinusitis (18.9%), eosinophilic bronchitis (7.5%), and chronic obstructive pulmonary disease (1.2%). The most commonly sensitized allergen was house dust mite (*Dermatophagoides pteronyssinus* 16.5%, *Dermatophagoides farina* 15.5%), followed by early blossoming tree pollen mix (8.9%) and late blossoming tree pollen mix (7.9%). AHR was associated with sensitization to *Dermatophagoides pteronyssinus* (*P*=0.013), *Dermatophagoides farina* (*P*=0.036), early blossoming tree pollen mix (*P*=0.003), and late blossoming tree pollen mix (*P*=0.002), after adjusting age, gender, daily particulate matter levels and meteorological factors. However, in subgroup analysis according to age, group II didn’t show any association between AHR and aeroallergen. There was no association between airway eosinophilic inflammation and sensitization to any aeroallergen.

**Conclusion:** In Busan, sensitization to house dust mite and tree pollens is common, it significantly affects airway hyperreponsiveness.

## A289 Multicenter Questionnaires on Current Management of Atopic Dermatitis Among Korean Patients and Caregivers

### Dong Hun Lee^2^, Jin-Young Lee^3^, Yang Park^4^, Jae-Won Oh^5^, Mi Hee Lee^6^, Soo-Jong Hong^7,8^, So-Yeon Lee^9^, Joon Soo Park^10^, Dong-Ho Nahm^11^, Hye-Yung Yum^12,13^

#### ^1^Seoul National University College of Medicine, South Korea; ^2^Seoul National University, South Korea, South Korea; ^3^Health Promotion Center, Samsung Medical Center, Sungkyunkwan University School of Medicine; ^4^Wonkwang University Sanbon Medical Center; ^5^Hanyang University Kuri Hospital; ^6^Seoul Women’s Hospital; ^7^Research Center for Standardization of Allergic Diseases, University of Ulsan College of Medicine; ^8^Childhood Asthma Atopy Center, Environmental Health Center, Asan Medical Center, University of Ulsan College of Medicine; ^9^Hallym University Sacred Heart Hospital; ^10^Soonchunhyang University College of Medicine; ^11^Ajou University School of Medicine; ^12^Seoul Medical Center; ^13^The Korean Academy of Asthma, Allergy and Clinical Immunology Work Group on Severe/Recalcitrant Atopic Dermatitis

##### **Correspondence:** Dong Hun Lee – Seoul National University College of Medicine, South Korea

**Background:** The effective management of atopic dermatitis (AD) adjusted to individual clinical courses and demands can be challenging for both patients and physicians. Understanding of actual situations, experienced and perceived by patients with AD and their caregivers, is essential to improve clinical outcomes and satisfaction in real practice.

**Methods:** This multi-center survey was conducted in patients with AD or their caregivers from 9 centers with questionnaires on diagnosis and management of AD.

**Results:** A total of 324 patients and caregivers participated in the study. Initial diagnosis of AD was mostly made by physicians (80.6 %), followed by self-diagnosis. Patients and caregivers believe that allergic substances such as house dust mite, food, pollution are responsible for AD development. Allergy tests were performed for 194 patients (59.9%), but allergen avoidance strategy was instructed in only 81 subjects (25.0%). Regarding the measures of AD management, they thought that moisturization, environmental control, improvement of body constitution are important. Major topical medications are steroid (81.8%) and topical immunomodulators (34.3%), while systemic medications include systemic steroid (42.6 %), anti-histamines (36.4 %), and cyclosporins (2.8 %). 181(55.9%) subjects have tried complementary alternative medicine including Oriental medicine. Many subjects wished for personalized management, use of specialized institutions for AD, and evidence-based, effective, and sustainable treatment to be incorporated in their sessions.

**Conclusion:** Our findings suggest there is still an unmet healthcare need for patients with AD in real practice. Customized, evidence-based, and multi-disciplinary approach including therapeutic patient education should be implemented to achieve the best possible outcomes.

## A290 Der p 1, Der p 2 and Der p 10 IgE Reactivities in Allergic Rhinitis Patients in Korea

### Kyu Young Choi^1^, Dong-Young Kim^2^

#### ^1^Hallym University College of Medicine, South Korea; ^2^Seoul National University

##### **Correspondence:** Kyu Young Choi – Hallym University College of Medicine, South Korea

**A) Background:** To evaluate the IgE reactivity profiles to purified house dust mite (HDM) allergen molecules (i.e. nDer p 1, rDer p 2 and rDer p 10) in Korean allergic rhinitis (AR) patients. Also, symptomatic and serologic changes after sublingual immunotherapy (SLIT) were analyzed according to positive IgE profiles.

**B) Methods:** Sixty AR patients already diagnosed to have HDM allergy were analyzed for the presence IgE antibodies against nDer p 1, rDer p 2, rDer p 10 and native Dermatophagoides pteronyssinus (Dp). Symptom scores and laboratory tests were followed-up after SLIT.

**C) Results:** Prevalence rates of IgE for Dp, Der p 1, Der p 2 and Der p 10 were 100%, 98.3%, 93.3% and 8.3%, respectively. After one year of SLIT, symptom scores and laboratory findings improved but did not show significant difference between Der p 10-positive and -negative patients.

**D) Conclusions:** In Korean AR patients, specific IgE to Der p 1 or Der p 2 presents in most of Dp-allergic patients, while reactivity to Der p 10 is very low. Changes in symptom and serology after SLIT (not containing the allergen Der p 10) did not differ much between Der p 10-positive and -negative patients.

## A291 De-Labeling Beta-Lactam Hypersensitivity: An Experience from a Tertiary Care Hospital in Thailand

### Sirinoot Palapinyo^1^, Jettanong Klaewsongkram^2^

#### ^1^Chulalongkorn University, Thailand; ^2^Allergy and Clinical Immunology Research Group, Chulalongkorn University

##### **Correspondence:** Sirinoot Palapinyo – Chulalongkorn University, Thailand

**Background**

A history of beta-lactam (BL) hypersensitivity is one of the most encountered problems in clinical practice and limits its further use. Determining whether patients with BL allergy could safely receive beta-lactams can be a difficult task. We summarize data from patients with a presumed allergic history to BL who underwent drug allergy verification and report the actual allergic rate.

**Methods**

Patients with a history of an immediate reaction or undetermined allergic onset to beta-lactams, who underwent BL hypersensitivity evaluation at King Chulalongkorn Memorial Hospital, Bangkok between 2012 and 2015, were retrospectively reviewed. The verification process was conducted according to the updated parameter on drug allergy included skin testing with both major and minor determinants of penicillin, amoxicillin, clavulanate, and/or the culprit BL. Intradermal test (IDT) was performed after negative skin prick test (SPT), and oral challenge test (OCT) was then carried out in patients who gave informed consent. Serum penicilloyl- and amoxycilloyl- specific IgE levels have also been evaluated.

**Results**

A total of 86 patients (mean age 40.7 years) were included. Forty-eight patients (56%) were female and 42 patients (48.8%) had underlying medical illnesses. Most patients have allergic history to penicillin (45.4%) or amoxicillin (29.1%). Sixty percent of patients developed symptoms within 2 hours. The majority experienced skin symptoms; urticarial rash (24.3%), angioedema (21.4%), and maculopapular rash (20%), while 3 patients reported anaphylaxis. BL allergy was confirmed in 7 patients (8.1%); identified by positive skin tests in 2 patients, positive specific Ig-E levels in 3 patients, and positive OCT in 4 patients. For those with positive skin tests; one had positive SPT but negative Ig-E levels and another one had positive results for both IDT and specific Ig-E levels. Sixty patients (71.4%) were underwent OCT and 54 patients (90%) demonstrated negative challenge. Serum specific IgE levels were determined in 46 patients and 44 of them (95.7%) showed negative results. Mild non-immediate reactions developed in 4 patients upon OCT after negative skin tests.

**Conclusions**

The reliability of self-reported BL allergy is low and questionable. Skin tests and specific IgE determination are safe to diagnose patients with an immediate reaction to beta-lactams. Drug allergy de-labeling is needed to distinguish true allergic patients from non-allergic cases, particularly in certain circumstances when alternative antibiotics are not available nor appropriate.

## A292 Sonic Hedgehog Signaling: Evidence for Its Protective Role in Endotoxin Induced Acute Lung Injury Mouse Model

### Xing Chen, Yuting Jin, Xiaoming Hou, Fengqin Liu, Chunyan Guo, Yulin Wang

#### Shandong Provincial Hospital Affiliated to Shandong University, China

##### **Correspondence:** Xing Chen – Shandong Provincial Hospital Affiliated to Shandong University, China

**Background:** To investigate the protective role of the sonic hedgehog (SHH) signaling associated with a lipopolysaccharide (LPS)-induced acute lung injury (ALI) in a mouse model.

**Methods:** Male BALB/c mice were randomly divided into three groups, control, LPS, and LPS-C group. ALI was induced by LPS ip injection (5 mg/kg). The sonic hedgehog inhibitor cyclopamine was given to the LPS-C group (50 mg/kg) at 30 min after LPS injection. Lung injury was observed histologically in hematoxylin and eosin (HE) stained tissue sections, semi-quantified by lung tissue injury score, and the lung tissue mass alteration was measured by wet to dry weight ratio (W/D). mRNA levels of TNF-α, SHH, Patched (PTC) and GLI1 in lung tissue were studied with real time quantitative PCR (RT-PCR), while the protein expression of SHH and GLI1 was determined by western blot analysis.

**Results:** Lung tissue injury score, W/D, and mRNA expression levels of TNF-α were significantly higher in the ALI mice than the normal mice (P<0.05). The mRNA levels of SHH, PTC, and GLI1 in the ALI mice were significantly higher at 12h and 24h after LPS injection, but not at the 6h time point. Protein production of SHH and GLI1 at 6h, 12h, and 24h in the lungs of ALI mice significantly increased, in a time-dependent manner, compared with that in normal mice. Cyclopamine intervention in ALI mice led to a reduction in mRNA levels of SHH, PTC, and GLI1 as well as SHH and GLI1 protein levels; meanwhile, the pathological injury scores of lung tissues, W/D, and mRNA expression levels of TNF-α increased compared to mice receiving LPS only.

**Conclusions:** The SHH signaling pathway was activated in response to LPS-induced ALI, and up-regulation of SHH expression could alleviate lung injury and be involved in the repair of injured lung tissue.

## A293 Analyses of the Factors behind the Negative Attitudes Toward the Administration of Adrenaline Auto-Injectors in School Settings

### Ikuo Okafuji^1^, Yuya Tanaka^1^, Shegeyuki Narabayashi^2^, Satoru Tsuruta^1^

#### ^1^Kobe City Medical Center General Hospital, Japan; ^2^Kakogawa City West Hospital

##### **Correspondence:** Ikuo Okafuji – Kobe City Medical Center General Hospital, Japan

**Background**

Knowledge and skills required for the use of adrenaline auto-injectors (AAI) are lacking in school settings, causing considerable uneasiness among teaching staff about administering AAI to students.

**Objective**

This study aimed to elucidate the factors behind the negative attitudes toward the administration of AAI in school settings.

**Methods**

After the lecture presentations on emergency treatment for food allergy organized by regional school boards in Hyogo Prefecture, we conducted a questionnaire survey among its participants. We then analyzed the answers received from 1,433 participants using a logistic regression model to investigate independent factors associated with the negative attitudes toward the administration of AAI.

**Results**

The respondents were comprised of nurse teachers (44%), general education teachers (32%), nutrition teachers (5%), assistant principals (10%) and principals (10%). Those who were negative about administering AAI to students accounted for 10.8% of total. The percentage differed significantly between different specialties/positions; nutrition teachers had the highest percentage (16.7%), followed by general education teachers (15.9%), nurse teachers (10.6%), assistant principals (8.1%) and principals (2.7%). The most frequent factor behind the uneasiness about the use of AAI was “the timing of AAI administration” (69.4%). By teaching specialty/position, a greater number of nurse teachers were concerned about “collaborating within the school” and “collaborating with a hospital” than teachers in other specialties/positions. Comparing between the group with negative attitudes and the group with positive attitudes toward AAI administration, the former was significantly more worried about “how to use AAI” (odds ratio [OR]: 2.1), “the timing of AAI administration” (OR: 02.9), “collaborating with the parents” (OR: 01.6), “the act of injection itself” (OR: 3.1) and “violating the laws” (OR: 3.5).

**Conclusion**

Most of the factors behind the negative attitudes toward the administration of AAI in school settings were associated with technical concerns.

## A294 Low Vitamin D Levels Are Related to High House Dust Mite Sensitization in Patients with Severe Atopic Dermatitis

### Yong Hyun Jang

#### Kyungpook National University School of Medicine, South Korea

**Background:** The relationship between atopic dermatitis (AD) and low vitamin D levels has been studied. Emerging evidence has implicated vitamin D as a critical regulator of immunity, playing a role in both the innate and cell-mediated immune systems. However, the effect of vitamin D on house dust mite (HDM) sensitization in patients with AD has not been established. We investigated the association between vitamin D levels and HDM sensitization according to AD severity.

**Methods:** In total, 80 patients (43 men and 37 women) with AD were included. We classified AD severity using Rajka and Langeland scores. Laboratory tests included serum 25-hydroxyvitamin D3, total immunoglobulin E (IgE), and specific IgE antibody titer against Dermatophagoides (D.) farinae and D. pteronyssinus.

**Results:** There were no differences in vitamin D levels between the mild or moderate AD and severe AD groups. In the severe AD group, high HDM sensitization group had lower serum vitamin D levels compared to low HDM sensitization group with statistical significance. In addition, a significant negative correlation was found between vitamin D levels and HDM sensitization in the severe AD group. These results did not depend on the type of HDM, D. farinae or D. pteronyssinus.

**Conclusions:** Our results demonstrate that low vitamin D levels may link to high HDM sensitization in patients with the severe AD. Further elucidation of the role of vitamin D in HDM sensitization may hold profound implications for the prevention and treatment of AD.

## A295 Appendicular Skeletal Muscle Mass Index: A Potential Predictor of Skeletal Muscle Abnormality According to the Severity Airflow Limitation of COPD

### Jun-Hong Ahn^1^, Dong-Won Lee^2^, Jin Hong Chung^1^, Hyun Jung Jin^3^, Min-Su Sohn^1^

#### ^1^Yeungnam University College of Medicine, South Korea; ^2^Andong Sungso Hospital; ^3^College of Medicine, Yeungnam University

##### **Correspondence:** Jun-Hong Ahn – Yeungnam University College of Medicine, South Korea

**Objective:** To investigate the effect of COPD on the skeletal muscles, particularly in patients with limb muscle dysfunction. The appendicular skeletal muscle mass index (ASMI), an index of sarcopenia, which was recently in the limelight, is an index of the limb muscle mass and is considered useful for predicting skeletal muscle abnormality in COPD patients. Thus, their relationships were examined.

**Method:** This research was conducted using the data from the 4th and 5th Korea National Health and Nutrition Examination Surveys (KNHANES), which were conducted from 2008 to 2011. Of the subjects on whom dual-energy x-ray absorptiometry (DXA) was performed to confirm their body composition, 1,219 COPD patients aged over 40 years showed an FEV1/FVC < 70%. COPD is classified into three groups--mild, moderate, and severe--according to the airflow limitation. In this study, 534 subjects had mild COPD; 613, moderate; and 72, severe. For the criteria for sarcopenia, the recommended criteria of the European Working Group on Sarcopenia in Older People (EWGSOP) and of the Asia Working Group for Sarcopenia (AWGS) were used.

**Results:** The ASMI of each group was categorized according to the COPD severity into 7.04±1.034, 6.83±1.030, and 6.45±1.071, respectively. Thus, there were differences between all the groups, and the higher the severity, the lower the results were(p < 0.001).When the sarcopenia classification of EWGSOP according to the ASMI was applied, each group’s FEV1(L) was 2.26±0.673, 2.20±0.633, and 1.94±0.730, respectively. In the case of Class II sarcopenia, it was lower than that in the normal case and in the case of the Class I sarcopenia.(P=0.003)When the sarcopenia criteria of AWGS was applied, the FEV1(L) was 2.30±0.667 and 2.09±0.651, and the pulmonary function of the sarcopenia group was low (p < 0.001). The correlation of the FEV1(L) with the ASMI was analyzed as 0.521, higher than with the BMI or the FFMI (p < 0.001); and the regression analysis also confirmed that the ASMI had a higher R2 and standardized regression coefficient than the BMI, FFMI, and skeletal muscle mass index (SMI) (p < 0.001).It was found that when the ASMI was used, the sarcopenia risk increased in all the cases in which the criteria recommended in EWGSOP and AWGS were used; and when the criteria of the AWGS were used, the moderate and severe stages showed the odds ratios of 1.587 (95% Cl, 1.109-2.271, p = 0.012) and 3.127 (95% Cl, 1.438-6.802, p = 0.004), respectively, compared with those of the mild stage.

**Conclusion:** ASMI is a fast and accurate predictor of skeletal muscle abnormality caused by an increase in the severity of the airflow limitation of COPD patients.

## A296 Etiology and Clinical Feature of Oral Allergy Syndrome in Children

### Young a Park^1^, Kyunguk Jeong^2^, Yoon Hee Kim^1^, In Suk Sol^1^, Seo Hee Yoon^1^, Kyung Won Kim^1^, Myung Hyun Sohn^1^, Kyu-Earn Kim^1^, Sooyoung Lee^2^

#### ^1^Yonsei University College of Medicine, South Korea; ^2^Ajou University School of Medicine

##### **Correspondence:** Young a Park – Yonsei University College of Medicine, South Korea

**Background:** Oral allergy syndrome (OAS) is defined as the symptoms of IgE-mediated immediate allergy localized in the oral mucosa, and the characteristics depend on the lability of the antigen. OAS is regarded as uncommon manifestations of pediatric population. This study focused on the allergenic relationship between pollen and food allergen of oral allergy syndrome in Korean children.

**Methods:** The study was based on a data analysis of patients, who were diagnosed with oral allergy syndrome at Ajou University hospital, Severance children’s hospital, Kangnam severance hospital from January 2008 to December 2014. Clinical details were collected by medical history and telephone survey.

**Results:** The subjects were 59 male and 38 female with median aged 9 years. In 97 children with oral allergy syndrome, most common causative food of allergy syndrome was apple. Pollen with the highest rate of positive responses is birch. In children with oral allergy syndrome, children sensitized birch have high risk of reaction to apple. The youngest patient with oral allergy syndrome is 3 years old girl. 65 children had reaction to more than 2 foods.

**Conclusion:** oral allergy syndrome may commonly affect children who are allergic to pollen. For children with oral allergy syndrome, knowledge of specific sensitization patterns has consequences for dietary management.

## A297 Traffic-Related Pollution Levels and Poorly Controlled Asthma in Adults

### Ho Kim, Ja Yeun Kim

#### Seoul National University, South Korea

##### **Correspondence:** Ho Kim – Seoul National University, South Korea

**Background:** Numerous epidemiological studies have shown adverse associations between increases in outdoor air pollution and health outcome. The majority of studies focused on daily concentrations of air pollutions and small-scale variation in daily averages and peak concentrations has not been able to characterize. We investigated the seasonal association between diurnal variation of traffic-related air pollution and the exacerbations of asthma symptoms among the middle-aged and the elderly in urban settings.

**Methods:** To address the health effect of diurnal variation of traffic-related air pollutants on asthma-related emergency department (ED) visits, we applied generalized linear model with over dispersed poison distribution to daily asthma-related ED visits between 2008 and 2011 in Seoul, Korea. The indicator variable of diurnal variation of traffic-related air pollutant, the diurnal range of NO_2_ (drNO_2_) was adopted and defined as the difference level of NO_2_ between 10:00 and 05:00 in the morning. The statistical analysis was conducted to estimate the effect of drNO_2_ adjusted for temperature, relative humidity, air pressure, PM_10_, O_3_, influenza epidemic indicator, day of week, and time trend. Age-specific effects and seasonality were tested and the age-specific groups were defined as the middle-aged aged between 50 and 74 and the elderly aged above 75.

**Results:** Among the total 19,702 asthma-related ED visits during study period, 6,933 were recruited with the middle-aged 4,503, and the elderly 2,430 and the increased overall risk were suggested with relative risk percent change with 95% confidence interval (95% CI); middle-aged [2.1 (95% CI; -11.3, 17.6)], and the elderly [23.6 (95% CI; 3.1, 48.3)] at lag0-8 by 1 interquartile range (IQR) increase of drNO_2_. Season specific effect for the middle-aged were [-12.4, (95% CI; -31.3, 11.6)], [8.3, (95% CI; -16.8, 40.8)], [-9.3, (95% CI; -34.2, 25.1)], and [28.4, (95% CI; -3.9, 71.6)] and the elderly were [23.0, (95% CI; -9.1, 66.5)], [20.2, (95% CI; -15.0, 70.0)], [-1.9, (95% CI; -36.0, 50.3)], and [52.5.0, (95% CI; 3.8.1, 123.9)] at lag0-8 for spring (March- May), summer (June- August), fall (September- November), and winter (December- February) respectively. Among the middle-aged group, the estimated effect during winter were detected at lag0-4 [24.6, (95% CI; 2.3, 51.6)], lag0-5 [32.1, (95% CI; 5.7, 65.2)], lag0-6 [31.2, (95% CI; 2.2, 68.4)], and lag0-7 [32.8, (95% CI; 1.4, 73.9)] during winter. This study suggests an adverse relationship between ambient drNO_2_ with the risk of asthma-related ED visits and the level of drNO_2_ was related to asthma exacerbations especially during spring and winter period and the delayed effect were varied by age-groups.

**Conclusion:** This provides evidence that heavily increased levels of traffic-related pollutant are associated with poorly controlled asthma among the adults during cold period.

## A298 Anaphylaxis in Korean Children, 2009-2013 : Triggers of Anaphylaxis By Age Groups

### So-Yeon Lee^1^, Taek-Ki Min^2^, Tae-Won Song^3^, Kangmo Ahn^4^, Jihyun Kim^4^, Gwang-Cheon Jang^5^, Hyeon-Jong Yang^6^, Bok-Yang Pyun^7^, Ji-Won Kwon^8^, Myung Hyun Sohn^9^, Kyu-Earn Kim^9^, Jinho Yu^10^, Soo-Jong Hong^11^, Jung-Hyun Kwon^12^, Sung-Won Kim^13^, Sooyoung Lee^14^, Woo Kyung Kim^15^, Hyung Young Kim^16^, Hye-Young Kim^17^, Youhoon Jeon^18^

#### ^1^Hallym University Sacred Heart Hospital, South Korea; ^2^Soonchunhyang University Hospital; ^3^Ilsan Paik Hospital; ^4^Samsung Medical Center; ^5^NHIS Ilsan Hospital; ^6^Soonchunhyang University College of Medicine; ^7^Soonchunhyang University; ^8^Seoul National University Bundang Hospital; ^9^Yonsei University College of Medicine; ^10^Asan Medical Center Seoul Korea; ^11^Research Center for Standardization of Allergic Diseases, University of Ulsan College of Medicine; ^12^Ewha Womans University Hospital; ^13^Pusan Saint Maria Hospital; ^14^Ajou University School of Medicine; ^15^Seoul Paik Hospital; ^16^Pusan National University Yangsan Hospital; ^17^Medical Research Institute of Pusan National University Hospital; ^18^Dongtan Sacred Heart Hospital, College of Medicine, Hallym University

##### **Correspondence:** So-Yeon Lee – Hallym University Sacred Heart Hospital, South Korea

**Background:** Although anaphylaxis is recognized as an important life-threatening condition, data are limited regarding its characteristics in the Korean children.

**Objective:** We sought to estimate the triggers of anaphylaxis by age groups in Korean children.

**Methods:** A retrospective medical record review was performed on children patients diagnosed with anaphylaxis between 2009 and 2013 in 23 tertiary hospitals of South Korea.

**Results:** A total 991 cases (66% male, mean age 5.89±5,24) were reported, with 36.6% below 2 years of age. Food was the most common cause (74.7%), followed by drugs and radiocontrast media (10.7%), idiopathic (9.2%), and exercise (3.6%). The most common offending food allergen was milk, followed by egg white, walnut, wheat, buckwheat, and peanut. Milk was the most common trigger of anaphylaxis in young children. In older children, seafood was the most common trigger of anaphylaxis. The rate of drugs in triggers of anaphylaxis was increased by age.

**Conclusion:** Food was the commonest trigger of anaphylaxis in Korean children. The common triggers of food induced anaphylaxis have changed over time in Korean children.

## A299 Maternal Allergy Is Associated with Acute Bronchiolitis Severity in Infant

### Chang Hoon Lim^1^, Yeongsang Jeong^2^, Su Jung Kim^3^

#### ^1^Korea University Medical Center, South Korea; ^2^Department of Pediatrics, Korea University College of Medicine, Seoul, Korea; ^3^Kepco Medical Center, Seoul, Korea

##### **Correspondence:** Chang Hoon Lim – Korea University Medical Center, South Korea

**Background**

Acute bronchiolitis is a common cause of hospitalization in children and has been identified as a risk factor of respiratory failure in young infants. There are few studies for determines the severity of acute bronchiolitis that may be helpful in the initial assessment of these infants.

**Methods**

Retrospective chart review of infants who hospitalized at single tertiary hospital between December 2014 and April 2015 was done. The exclusion criteria were atopic dermatitis, preterm, congenital heart disease, other congenital anomaly and metabolic disease. Bronchiolitis severity score (BSS, general appearance, lung sound, dyspnea, respiration rate, oxygen saturation by pulse oxymetry) was evaluated at admission and within 72 hrs after admission. Outcome measures included feeding pattern, allergy history of parents, virus, initial body weight, current body weight and, height.

**Results**

We enrolled 51 hospitalized infants, all under 12 months old (3.79±2.64 months of age) and 66% were male (n=34). Mean body weight at admission was 7.11±1.88 kg. The mean (±SD) duration of hospitalization was 5.67 ±2.2 days and it had positive relation with BSS (P<0.05). There were significant association between BSS and maternal allergy, height and age (p<0.05). However, no significant association was observed between BSS and body weight, amount of increased weight from birth and infected virus.

**Conclusions**

Maternal allergy, age and height of infant were found to be significant factors in the severity of acute bronchiolitis in infant. Further study is needed to determine if maternal allergy allow for prediction of long term respiratory outcomes, such as asthma, following bronchiolitis because infants with severe bronchiolitis were more likely to have a familial atopic predisposition, which may partly explain subsequent increased asthma risk.

**Keywords:** Allergy: Pediatric

## A300 Evaluation of Inflammatory Mediator Profiles in Sputum of Asthmatics As an Endotype for Refractory Asthma

### Hun Soo Chang^1^, Jeong-Seok Heo^1^, Da-Jeong Bae^2^, Jong-Uk Lee^1^, Ji-Na Kim^1^, Chang-Gi Min^1^, Hyun Ji Song^2^, Jong-Sook Park^2^, Soo Hyun Kim^3^, Choon-Sik Park^2^

#### ^1^Soonchunhyang University, South Korea; ^2^Soonchunhyang University Bucheon Hospital; ^3^Konkuk University

##### **Correspondence:** Hun Soo Chang – Soonchunhyang University, South Korea

**Background:** Refractory asthma is characterized by poor response to corticosteroid treatment followed by increased burden of the disease. Therefore, development of endotypes related with refractory asthma is important for diagnosis and treatment. The aim of the study was to determine the level of inflammatory mediators in sputum and to evaluate usefulness of the protein profiles as an endotype of refractory asthma.

**Methods:** Total 238 asthmatics (216 never smokers and 22 exsmoker less than 10 pack years) were enrolled and sputum was induced using isotonic saline containing a short-acting bronchodilator. Differential cell count was performed. The concentration of S100A9, IL-1b, -5, -8, -13, -17A, -32 and -33 was determined using ELISA, then normalized with total protein in supernatant of sputum. Statistical analyses, clustering and decision tree analysis were performed using SPSS 12.0.

**Results:** There were positive correlations between levels of S100A9 with those of IL-1b and IL-13, between IL-8 with IL13 and IL17A, between IL-17A with IL13, IL32 and IL33 and between IL-32 and IL-33 (p<0.05). The level of IL-13 was inversely correlated with IL-33. The subjects were clustered into four major groups according to the sputum level of cytokines; subjects with high level of IL-17A and IL-1b (group 1), with relatively low levels of cytokines with modest increase of IL-33 (group 2), with high IL-8 and IL-13 (group 3) and with highest S100A9 with moderate increase of IL-8 (group 4). The clinical features of the group 1 were younger, high airway hyperreactivity and increased numbers of WBC in blood. While group 2 showed highest % of eosinophils and low neutrophils in sputum, Group 3 showed highest % of neutrophils. Group 4 showed 2ndly highest % of neutrophils in sputum, old age, and relatively normal airway hyperreactivity. However, lung functions and exacerbation status were comparable between groups. In decision tree analysis for exacerbation, the patient were classified into three groups according to the level of cytokines; group 1 with lower IL-17A level (<=1.093 pg/ug), group 2 with higher IL-17A (>1.093 pg/ug) and low IL-32 (<=1.336 pg/ug) and IL-33 levels, and group 3 with higher IL-17A, IL-32 and IL-33 levels. The patient in group 1 was old-age and greatest pack years of smoking. They showed significantly higher serum level of total IgE, and neutrophil% in sputum than those of other groups. The lung function of the group was lowest in spite of treatment with highest dose of inhaled corticosteroid. Patients in group 3 showed significantly frequent exacerbation per year (P = 0.0001).

**Conclusion:** The level of IL-17A, IL-32 and IL-33 in sputum may be a surrogate set of markers for predicting treatment outcomes in asthmatics and an endotype for refractory asthma.

## A301 Autophagy Is Associated with the Severity of Asthma in an Ovalbumin-Specific Mouse Model of Allergic Asthma

### Jing-Nan Liu^1^, Youngwoo Choi^2^, Yoo Seob Shin^1^, Hae-Sim Park^1^

#### ^1^Ajou University School of Medicine, South Korea; ^2^Pohang University of Science & Technology (POSTECH)

##### **Correspondence:** Jing-Nan Liu – Ajou University School of Medicine, South Korea

**Background:** Autophagy has been investigated to be involved in many inflammatory diseases, but its expression and clinical significance in asthma has rarely been studied. The aim of this study was to evaluate the role of autophagy and the therapeutic potentials in an OVA-specific mouse model of severe allergic asthma. **Method:** BALB/c mice were sensitized by intraperitoneal injection of ovalbumin (OVA) on days 0 and 14, followed by aerosolized primary OVA challenges on day 28 - 30. Two weeks after the primary allergen challenges, mice received a secondary allergen challenges with two different concentrations (1% or 2%) of OVA solution on days 44 – 46. Forty eight hours after the last OVA challenge, mice were assessed for airway responsiveness (AHR) to inhaled methacholine, cell composition and cytokine levels in bronchoalveolar lavage (BAL) fluid. LC3 expression in lung homogenate was measured by Western blot to evaluate the development of autophagy. Finally, 3-methyladenine (3-MA), and Atg5 knockdown were applied to investigate the potential role of autophagy in severe allergic asthma of mice. **Results:** The AHR, total airway inflammatory cell number and eosinophilia in BAL fluid were significantly higher in 2% OVA challenged mice compared to those of 1% OVA challenged mice (P < 0.01 and P < 0.05, respectively) indicating that 2% OVA challenges could induce a mouse model presenting more severe eosinophilic asthma. The severe allergic asthma mice showed significant higher LC3 expression compared to that of mild asthma mice. Additionally, more prominent autophagosomes were noted in the cytoplasm of eosinophilis. Inhibition of autophagy by 3-MA treatment and Atg5 knockdown could induce significant improvement of AHR, eosinophilia and IL-5 levels in BAL fluid and airway inflammation in histology. Anti-IL-5 antibody treatment significantly reduced eosinophil counts and IL-5 levels in BAL fluid, as well as LC3 expression in the lung tissue homogenate. **Conclusions:** Our findings suggest that the expression of autophagy is correlated with the severity of asthma through eosinophilic inflammation, and autophagy inhibitions may provide novel therapeutic targets for the treatment of severe allergic asthma.

## A302 Interleukin-9 and Interleukin-33 Levels in Children with Asthma

### Nima Rezaei^1^, Nima Rezaei^2^, Sedigheh Bahrami Mahneh^1^, Arezou Rezaei^1^, Maryam Sadr^1^, Masoud Movahedi^1^

#### ^1^Tehran University of Medical Sciences; ^2^Usern, Iran

##### **Correspondence:** Nima Rezaei – Tehran University of Medical Sciences, Iran

**Background:** As of possible role of interleukin-9 (IL-9), a pro-inflammatory and Th2 cytokine, and interleukin-33 (IL-33), a member of IL-1 cytokine family, in pediatric asthma, this study was performed to measure IL-9 and IL-33 serum levels in pediatric patients with asthma.

**Methods:** In this study, 61 patients with asthma and 63 healthy age- and sex- matched subjects were enrolled. Serum levels of IL-9 and IL-33 were measured in asthmatic patients and healthy subjects by sandwich ELISA.

**Results:** Serum IL-9 level was significantly higher in patients group, compared to the controls (p=0.03). In addition, patients with severe asthma had significantly higher serum IL-9 levels, compared to the controls (p<0.001). Mean serum level of IL-33 asthmatic children was also significantly higher than the controls (p=0.02), which also showed statistically significant associated with the severity disease (p=0.02).

**Conclusions:** Elevated serum levels of IL-9 and IL-33 were found to be associated with pediatric asthma and the disease severity.

## A303 Pediatric Anaphylaxis at a University Hospital in Cheonan, Korea, 2013~2014

### Jun Seak Gang^1^, Joon Soo Park^1^, Seung Soo Kim^1^, Hyun Ho Bang^1^, Kyeong Bae Park^1^, Hye Sun Kim^1^, Tae Ho Kim^1^, Young Hwangbo^2^, Hyun Jung Lee^1^, Gyeong Hee Yoo^1^, Young Chang Kim^1^

#### ^1^Soonchunhyang University Hospital, South Korea; ^2^Soonchunhyang University

##### **Correspondence:** Jun Seak Gang – Soonchunhyang University Hospital, South Korea

**Background**

Epinephrine is the first treatment for anaphylaxis. To investigate the clinical features of pediatric anaphylaxis, including the rate of using epinephrine and prescribing epinephrine auto-injector.

**Methods**

We performed a retrospective study in in-patients, out-patients, and emergency department visitors, who were under 15 years old, at Soonchunhyang University Hospital, Cheonan, Korea, from Jan. 2013 through Dec. 2014. A total of 68 patients were diagnosed with anaphylaxis by criteria at the time.

**Results**

The causes of anaphylaxis were food (77.9%), drug (10.3%) and idiopathic (11.8%). The involved organs were cutaneous (88.2%), respiratory (80.9%), cardiovascular (20.6%), and gastrointestinal tract (14.7%). Patients were treated with systemic steroid (92.6%), anti-histamine (88.2%) and epinephrine (76.5%). 53 (77.9%) patients re-visited our pediatric allergy clinic and epinephrine auto-injectors were prescribed for 25 (36.8%) patients.

**Conclusion**

Epinephrine was not used in more than 20% and epinephrine auto-injectors were not prescribed for more than 60% of pediatric anaphylaxis patents. Physicians should make an effort to use epinephrine as the initial treatment of anaphylaxis and to prescribe epinephrine auto-injector.

## A304 Initial Antigen-Specific IgE Levels Predict Clinical Outcome of Rush Oral Immunotherapy for Food Anaphylaxis

### Sakura Sato, Noriyuki Yanagida, Motohiro Ebisawa

#### Sagamihara National Hospital, Japan

##### **Correspondence:** Sakura Sato – Sagamihara National Hospital, Japan

**Background**

Oral immunotherapy (OIT), a novel therapeutic approach to food allergy, appears to be effective in increasing the threshold for clinical reactivity to food. The aim of the study is to assess the risk factors associated with rush OIT (ROIT) for treating anaphylaxis induced by allergy to hen’s egg (HE), cow’s milk (CM), wheat (W), or peanut (P).

**Methods**

HE, CM, and W anaphylaxis in our department confirmed by the positive double-blind, placebo-controlled food challenge test received following treatment with ROIT. Patients were treated with a combination of rush phase followed by a slow build-up of maintenance doses. patients who had ingested trial foods (HE, heated-whole egg [60 g]; CM, 200 ml; W, Udon noodle [200 g]; P, peanut powder [3 g]) without any symptoms for several months, then they underwent the final oral food challenge (OFC) after allergen avoidance for 2 weeks to confirm the development of clinical tolerance. Any changes in antigen specific IgE levels during OIT treatment were documented.

**Results**

A total of 224 subjects (HE, n = 70; CM, n = 87; W, n = 38; P, n = 29) with an average age of 9.1 years, who had been receiving ROIT for 1 & 1/2 year or more, were enrolled in the study. One and half year later, 75 subjects (33%) passed the final OFC (tentative tolerance group, HE, 33; CM, 17; W, 11; P, 14), whereas 149 subjects (67%) had reacted to the trial foods (Allergic group). After 1 & 1/2 year of ROIT, the median antigen-specific IgE levels (kU/L) were significantly decreased compared to the levels prior to OIT (egg white: 23.4 vs. 7.0; ovomucoid: 20.3 vs. 5.3; milk: 56.4 vs. 14.0; casein: 54.5 vs. 14.8; wheat: 213.0 vs. 43.8; omega-5 gliadin: 6.4 vs. 1.3; peanut: 33.2 vs. 12.5; Ara h 2: 27.4 vs. 10.8, *p* < 0.05), whereas antigen specific IgG4 increased significantly (0.9 vs. 43.7 for egg white; and 2.2 vs. 9.1 for casein; p < 0.05). In the tentative tolerant group of HE and CM-ROIT, the initial antigen-specific IgE levels were significantly lower than that of the allergic group (tentative tolerant group vs. allergic group, egg white: 18.3 vs. 32.6; ovomucoid: 14.3 vs. 31.8; milk: 26.1 vs. 57.3; casein: 24.2 vs. 62.5; *p* < 0.05). No significant differences were found in patients allergic to W and P.

**Conclusions**

In this study, ROIT induced a significant decrease in antigen specific IgE levels. For HE and CM anaphylaxis, the patients who had relatively low antigen-specific IgE at the beginning of ROIT appeared to respond better to ROIT within 1 & 1/2 year.

## A305 *ABCC4* gene Polymorphism Is Associated with High Periostin Levels in Asthmatic Patients

### Sailesh Palikhe, Hae-Sim Park, Seung-Hyun Kim, Ri-Yeon Kim, Eun-Mi Yang

#### Ajou University School of Medicine, South Korea

##### **Correspondence:** Sailesh Palikhe – Ajou University School of Medicine, South Korea

**Background:***ABCC4* (ATP-binding cassette, sub-family C, member 4) is a protein-coding gene which codes for transmembrane protein. ABCC4 protein transports various molecules across extra- and intra-cellullar membranes, including PGE_2_ and cAMP. Studies have reported that PGE_2_ and cAMP play an important role in the regulation of immune responses and airway inflammation. In this study, we investigated the associations of *ABCC4* gene polymorphisms and clinical characteristics of asthmatic patients to understand the role of *ABCC4*gene in asthma pathogenesis.

**Methods:** 269 asthma patients and 118 normal healthy controls were enrolled in the study. Two single nucleotide polymorphisms (SNP) (-642G>C and -1508A>G) in the *ABCC4*promoter region were genotyped by TaqMan allelic discrimination assay. Functional variabilities in the promoter polymorphisms were analyzed by gene reporter assay. Inflammatory cytokine levels in serum were measured by ELISA.

**Results:** There were no significant differences in genotype frequencies between two study groups. Asthmatic patients carrying G allele at -1508A>G had significantly higher periostin levels in sera (*P*=0.018) and lower PC_20_ methacholine levels (*P*=0.008) compared to non-carriers. No significant difference was noted in sputum eosinophil count according to each genotype. Gene reporter assay showed that the promoter SNP tagged by G-1508 allele conferred significantly higher promoter activity (*P*<0.01) compared with A-1508 allele in A549 cell line.

**Conclusion:** These findings suggest that *ABCC4* gene polymorphism at -1508A>G may enhance periostin production and could involve in eosinophilic asthma.

## A306 The Role of Clinical Phenotype and Allergen Sensitization at 2 Years As Predictors of Atopic Disorders at 5 Years

### Li Yuan Gabriella Nadine Lee^2^, Marion Aw^1,2^, Bee Wah Lee^1,2^, Evelyn Xiu Ling Loo^3^, Yiong Huak Chan^1^, Lynette Shek^2,1^, I-Chun Kuo^1,2^, Phaik Ling Quah^1,2^, Genevieve Llanora^2^, Gerez Irvin^2^

#### ^1^National University of Singapore, Singapore; ^2^National University Health System, Singapore; ^3^Agency of Science, Technology and Research

##### **Correspondence:** Li Yuan Gabriella Nadine Lee – National University of Singapore, Singapore

**Introduction:** From a birth cohort of at risk Asian infants, we prospectively investigated the role of early onset allergen sensitization and clinical phenotypes as risk factors for atopic disorders at the age of 5 years.

**Methods:** The study recruited 253 families with a history of allergic disease in a first degree relative from an antenatal clinic in Singapore. The children were followed prospectively to assess clinical outcomes and skin prick test was performed at 2 and 5 years of age.

**Results:** Clinical phenotype alone at 2 years of age was associated with increased risk of atopic disorders at 5 years. This risk was further increased by the presence of concomitant allergen sensitization (food and/or house dust mites). For eczema, eczema alone at 2 years old increased the risk of eczema at 5 years (adjOR 7.1 95%CI: 1.8-27.8). This risk was further increased by the presence of allergen sensitization (adjOR 25.4, 95%CI: 4.7-138.5). For wheeze, wheeze alone at year 2 increased the risk of wheeze at year 5 (adjOR 4.5 95%CI: 1.4-14.8) and this risk tripled with the concomitant presence of allergen sensitization and eczema (adjOR 13.9, 95%CI: 1.2-168.5). As rhinitis symptoms were not apparent at the age of 2 years, allergen sensitization alone at 2 years without the rhinitis phenotype increased the risk of rhinitis at 5 years (adjOR 5.6 95%CI: 1.1-29.2). However, the eczema phenotype at 2 years alone also increased the risk of rhinitis at 5 years (adjOR 6.8 95%CI: 2.0-23.1).

**Conclusion:** In this Asian birth cohort, the clinical phenotypes alone and with concomitant allergen sensitization were predictors of eczema and wheeze at 5 years. Except for the rhinitis outcome, allergen sensitization alone at 2 years could not predict atopic clinical outcomes at 5 years.

## A307 The Effect of Korean Red Ginseng (KRG) on Rhinovirus Infection in Human Nasal Epithelial Cells

### Joo Hyun Jung^1^, Il Gyu Kang^2^, Seon Tae Kim^3^, Hyoungmin Park^2^

#### ^1^Gachon University Gil Medical Center, South Korea; ^2^Gil Hospital; ^3^Gil Medical Center, Gachon Medical School, South Korea

##### **Correspondence:** Joo Hyun Jung – Gachon University Gil Medical Center, South Korea

**Background:** Korean red ginseng (KRG) is reported to have anti-allergic properties, including beneficial effects on asthma and atopic dermatitis. However, its effect on allergic rhinitis and rhinovirus infection has not been studied extensively. This study examined whether KRG affected rhinovirus infection in human nasal epithelial cells.

**Methods:** Primary human nasal epithelial cells were obtained by digesting inferior turbinate mucosa removed from patients during turbinoplasty. After nasal epithelials cell were cultured, cells were infected with rhinovirus serotype 16 (RV-16). To confirm preventive and therapeutic effect of KRG on rhinovirus infection, 50, 100mg/ml of KRG were administered to cultured nasal epithelial cells before and after rhinovirus infection provoked. The morphology of nasal epitherlial cells was checked.

**Results:** The morphology of nasal epithelial cells were changed after rhinovirus infection. The degree of morphologic change were decreased by KRG treatment in both group (treatment for before and after rhinovirus infection), however, the nasal epithelial cells were less changed by KRG administration after rhinovirus infection.

**Conclusions:** KRG have preventive and therapeutic effect in human nasal epithelial cells

## A308 The Effect of Korean Red Ginseng on the Symptoms and Allergic Inflammation in Patients with Allergic Rhinitis

### Seon Tae Kim^1^, Joo Hyun Jung^2^, Il Gyu Kang^3^, Hyoungmin Park^3^, Kwang-Pil Ko^3^

#### ^1^Gil Medical Center, Gachon Medical School, South Korea; ^2^Gachon University Gil Medical Center; ^3^Gil Hospital

##### **Correspondence:** Seon Tae Kim – Gil Medical Center, Gachon Medical School, South Korea

**Introduction:** Korean Red Ginseng has been traditionally used in Korea for health improvement. However, the clinical effect of KRG intake on the symptoms in allergic rhinitis patients remains unknown. Our study was performed to identify the clinical effects of KRG on allergic rhinitis patients and to examine the effect of KRG on allergic inflammatory reaction.

**Patients and Methods:** We evaluated 50 allergic rhinitis. All of the patients were treated for 4 weeks. The patients were divided into three groups according to the medication. Twenty patients were treated with KRG, 20 patients with the placebo and 10 patients with the antihistamine. The patients recorded their symptoms in a daily symptom diary card. The patients checked the peak inspiratory flow (PNIF) rate two times a day, in the morning and evening, during the medication period. Total serum IgE and serum specific IgE for D.P. and D.F. were measured by ImmunoCap method before and after 4 week medication. The Th2 cytokines IL-4, IL-5 and IL-10 were checked in the serum before and after the 4-week treatment using ELISA methods. The eosinophil counts in the nasal smears were checked before and after the 4-week treatment.

**Results:** The KRG group demonstrated a significant improvement of allergic symptoms, except in the smelling difficulty category. PNIF demonstrated no differences between the periods before and after treatment in either the KRG group or the antihistamine treatment group. Both the antihistamine and KRG groups showed a significant decrease in total IgE level at the end of treatment. The serum IL-4 level and eosinophil counts in the nasal smears were significantly decreased both in the antihistamine and the KRG groups.

**Conclusion:** KRG might be a useful treatment modality in allergic rhinitis patients. However, we need to better understand the long-term effects of KRG with further studies.

## A309 Validation of the Newly Developed Multiple Allergen Simultaneous Test in Korea

### Jungsoo Lee^1^, Howard Chu^1^, Hemin Lee^1^, Jung U Shin^1^, Chang Ook Park^1^, Kwang Hoon Lee^1,2^, Hong Kyu Kang^1^

#### ^1^Yonsei University College of Medicine, Department of Dermatology, South Korea; ^2^Brain Korea 21 PLUS Project for Medical Science, Yonsei University College of Medicine

##### **Correspondence:** Jungsoo Lee – Yonsei University College of Medicine, Department of Dermatology, South Korea

**Background**

Screening system ‘A’ is the most commonly used allergen-specific IgE detecting system in Korea. With high sensitivity and low specificity, it has been used as a screening system. However, the correlation rates with ImmunoCAP® System or skin prick test are relatively lower in food allergens than inhalant allergens.

**Purpose**

To validate the new allergen-specific IgE detecting system, ‘B’, in comparison with ‘A’ and ImmunoCAP® System.

**Methods**

We evaluated 101 Korean atopic dermatitis (AD) patients’ results from ‘A’ and newly developed ‘B’. We also compared the correlation coefficient with ImmunoCAP-test, the gold-standard diagnostic tool for extrinsic AD.

**Results**

When we compared the correlation coefficient between ‘A’ and ImmunoCAP-test of 5 antigens including D*.farinae,* D*.pteronyssinus,* soyben, cow milk and white egg, results of cow milk, D*.farinae,* D*.pteronyssinus* antigens were significantly matched. Moderate strength of agreement was found in egg white and fair agreement was observed in wheat. However, only D.*farinae* and D.*pteronyssinus*results from ‘B’ showed significant agreement strength with CAP-test and other 4 antigens only had slight agreements.

**Conclusion**

Overall, more accurate results were observed with a new screening system ‘B’ when compared with ‘A’. Further study will be performed to analyze the results from other antigens.

## A310 Assessment of Symptoms Severities of Allergic Rhinitis Patients Sensitive to Multiple Allergens in Skin Prick Test

### Dong Chang Lee, Geun Jeon Kim, Jae Hyung Hwang, Jin Bu Ha, Su Hee Jeong

#### Dae-Jeon St. Mary’s Hospital, South Korea

##### **Correspondence:** Dong Chang Lee – Dae-Jeon St. Mary’s Hospital, South Korea

**Background and Objectives:** To assess the symptoms severities of allergic rhinitis patients sensitive to multiple allergens compared to single house dust mites allergen.

**Subjects and Method:** A total of 104 allergic rhinitis patients were classified into 2 groups. The multiple allergens group was defined as patients sensitive to multiple allergens including house dust mite. The single allergen group was defined as sensitive to only house dust mite in skin prick test. Severity of allergic rhinitis was evaluated according to the visual analogue scale (VAS) for following nasal symptoms. In vitro test, total IgE and blood eosinophil count was analysed.

**Results:** Total 104 allergic rhinitis patients were classified into 67 multiple allergens and 37 single house dust mite sensitive groups. The visual analogue scale of nasal symptoms were no difference between two groups as in vitro test, total IgE and blood eosinophil count.

**Conclusion:** Most subjective symptoms severities of allergic rhinitis patients sensitive to multiple allergens showed no difference rather than single house dust mite.

## A311 Diurnal Temperature Range and Emergency Department Visits for Asthma in Korea 6 Cities

### Ho Kim, Shinha Hwang, Whahee Lee

#### Seoul National University, South Korea

##### **Correspondence:** Ho Kim – Seoul National University, South Korea

**Purpose:** Asthma is a major public health problem, and many studies have shown that diurnal temperature change (DTR) is associated with asthma mortality and emergency department(ED) visits as asthma morbidity. These studies represented that DTR and asthma ED visit showed non-linear relationship and had lagged effect up to 7-14 days, however only few study considered these two characteristics simultaneously. This study aim to find an association between DTR and asthma ED visit including non-linear and lagged effects.

**Method:** ED data of 6 Korean cities (Seoul, Busan, Daejeon, Daegu, Gwangju, and Incheon) were collected from January 1, 2008 to December 31, 2010, through the National Emergency Department Information System (NEDIS) for the National Emergency Medical Center. We applied 2 stage distributed lag non-linear model (DLNM) with 10 lag days and an allowance of over-dispersion adjusting for meteorological conditions and time trend.

**Result:** From 2 stage analysis, we found DTR was adversely associated with asthma-related ED visits in Korea and as DTR increased, so did the risk of ED visit. The relative risks of asthma-related ED visit were estimated with Mid-high (80^th^ percentile versus minimum mortality DTR), High (90^th^ percentile versus minimum mortality DTR) and Extreme high (99^th^ percentile versus minimum mortality DTR). Relative risks were 1.032[95% CI, 0.82, 1.29] at Mid-high condition, 1.132[0.88, 1.46], at High and 1.543[0.69, 3.46] at Extreme high.

**Conclusion:** We found that DTR had adverse effect to ED visits in Korea. And effect of DTR showed non-linear relationship and was significant up to 10 days. Therefore as DTR increases, the more vulnerable people and public health authorities should pay special attention to an acute exacerbation of asthma.

## A312 Mannan-Binding Lectin Serum Levels in Atopic Mongolian Adults

### Enkhbayar Bazarsad^1^, Logii Narantsetseg^2^, Munkhbayarlakh Sonomjamts^3^

#### ^1^Ach Medical University, Mongolia; ^2^Department of Cellular Biology and Biochemistry, School of Pharmacy and Bio-Medicine, Mongolian National University of Medical Sciences, Ulaanbaatar, Mongolia; ^3^School of Medicine, Mongolian National University of Medical Sciences, Ulaanbaatar, Mongolia

##### **Correspondence:** Enkhbayar Bazarsad – Ach Medical University, Mongolia

**Backround.** Mannan-binding lectin (MBL) is a serum protein involved in opsonization and complement activation, which contains lectin and collagen domains, synthesized in the liver. MBL binds to N-acetylglucosamineand mannan structures on thesurface of bacteria, fungi, viruses and protozoa, leading to opsonization, phagocytosis and activation ofcomplement system through lectin pathway, independent of an antibody. Most of the allergic diseases are dominated by the preferential development of specific Th2 type of adaptive immune responses against innocuous antigens in atopic individuals. Low level of serum MBL is associated with increased susceptibility to infectionsand high risk of some allergic and autoimmune diseases. The recently assigned roles of MBL in inflammatory diseases due to increased complement activation have stimulated research into the contribution of MBL and its associated complement activity in asthma and respiratory allergy.

**Purpose.** Main aim of our study was to determine theprofile of MBL serum level in Mongolian atopic subjects.

**Method.** Blood samples were collected from Mongolian individuals (n=216) with allergic rhinitis: 86 males and 130 females in 4 different age groups (20-30, 31-40, 41-50 and 51-60). Their mean age was 32.5 years. All subjects had a positive reaction on skin prick tests to one or more inhalant allergens. We were analyzed for MBL level in serum by using double-antibody sandwich ELISA method.

**Results.** The mean and median serum levels of MBL were 3088.29 ± 671.4 ng/ml and 3147.09 ng/ml.

MBL level in male were 3012.8± 787.8 ng/ml, female 3138.2± 583.1 ng/ml. No association was observed between sex and MBL concentrations in serum.

3192,11 ± 536,64ng/ml at 20-30 years of age, 2994,28 ± 695,41ng/ml at 31-40 years, 3279,24 ±537,36ng/ml at 41-50 years and 2608,08 ± 1008,15ng/ml in 51-60 years. There were no significant differences in the mean concentration of MBL among different age groups.

**Conclusions.** MBL serum levelof Mongolian allergic patients is comparatively higher than some other reported study.

## A313 Prevalence of Doctor Diagnosed Atopic Eczema, during 2003-2014 in KOREA ; Using Big Data of 48.1 Million South Korean Health-Care Records

### Gwang-Cheon Jang^1^, Hyun-Hee Lee^2^, Chang-Jong Lee^3^, Huynsun Lim^1^

#### ^1^NHIS Ilsan Hospital, South Korea; ^2^Catholic Kwandong University College of Medicine; ^3^National Health Insurance Service

##### **Correspondence:** Gwang-Cheon Jang – NHIS Ilsan Hospital, South Korea

**Background:** The prevalence of atopic eczema has increased worldwide for past few decades. However, in some countries, it remains stable or even decreased. The aim of this study was to estimate the prevalence of atopic eczema in Korea.

**Methods:** To investigate the prevalence of atopic eczema (L20), we did analyze the nationwide database (National Health Insurance Corporation) which included the health-care records of 48.1 million individuals between January 1, 2003, and December 31, 2014.

**Results:** Prevalence of atopic eczema in Korea showing a decreased tendency; 2.42% (2003), 2.44% (2004), 2.40% (2005), 2.26% (2006), 2.27% (2007), 2.26% (2008), 2.15% (2009), 2.14% (2010), 2.05% (2011), 1.97% (2012), 1.96% (2013), and 1.89% (2014). Also, child groups decreased between 2003 and 2014 ; under 6 years old age group had decreased 14.20% to 10.74%, 7~12 years group 5.24% to 4.73%. However, adolescent and adult age groups were increased between 2003 and 2014: 13~19 years group 1.82% to 2.79%. 20~29 years group 1.25% to 1.58%, 30~39 years group 0.68% to 0.89%, 41~49 years group 0.48% to 0.63%, 50~59 years group 0.53% to 0.62%, 60~69 years group 0.60% to 0.76%, and over 70 years group 0.66% to 0.96%.

**Conclusion:** Prevalence of atopic eczema in Korea showing decreased tendency, however increased in adult age groups. Interestingly, under 6 years old age group had been showing abruptly decreased pattern after 2009 endemic influenza infecion. So, further study was needed.

## A314 Association of Recurrent Wheeze with Lung Function and Airway Inflammation in Preschool Children

### Ji-Eun Soh^1^, Dae-Jin Song^2^, Ji-Won Kwon^3^, Hyung Young Kim^4^, Ju-Hee Seo^5^, Byoung-Ju Kim^6^, Hyo-Bin Kim^7^, So-Yeon Lee^8^, Gwang-Cheon Jang^9^, Woo Kyung Kim^10^, Young-Ho Jung^11^, Soo-Jong Hong^12^, Jung Yeon Shim^13^

#### ^1^Kangbuk Samsung Hospital, South Korea; ^2^Guro Hospital; ^3^Seoul National University Bundang Hospital; ^4^Pusan National University Yangsan Hospital; ^5^Korea Cancer Center Hospital; ^6^University of Cincinnati College of Medicine; ^7^Inje University Sanggye Paik Hospital; ^8^Hallym University Sacred Heart Hospital; ^9^National Health Corporation Ilsan Hospital; ^10^Seoul Paik Hospital; ^11^Bundang CHA Medical Center, CHA University School of Medicine; ^12^Research Center for Standardization of Allergic Diseases, University of Ulsan College of Medicine; ^13^Kangbuk Samsung Hospital/Sungkyunkwan University School of Medicine

##### **Correspondence:** Ji-Eun Soh – Kangbuk Samsung Hospital, South Korea

**Objective:** Recurrent wheeze is one of the predictive markers of asthma in preschool children. The aims of this study were to investigate airway inflammation, lung function, airway hyperresponsiveness (AHR), and the prevalence of allergic rhinitis (AR) and atopic dermatitis (AD) in preschool children according to recurrent wheeze. **Methods:** We performed a population-based, cross-sectional study with 933 children aged 4-6 years. A total of 900 children completed a modified International Study of Asthma and Allergies in Childhood questionnaire and eligible for the study. We measured exhaled nitric oxide (eNO), spirometry, methacholine bronchial provocation, and impulse oscillometry. Recurrent wheeze was defined as having a lifetime wheeze more than 3 times. **Results:** The prevalence of recurrent wheeze was 13.4%. Children with recurrent wheeze showed higher prevalence of lifetime and current AR and lifetime AD, not current AD. Recurrent wheeze was associated with lifetime emergency room visit more than 1 time and history of more than one admission within 12 months due to wheezing episode. High eNO, post-bronchodilator change of R_5_Hz, and blood eosinophils as well as low FEF_25-75%_ were associated with recurrent wheeze. However, dose response slope by methacholine test, prevalence of atopy or AHR, and serum IgE levels showed no significant differences between two groups. **Conclusions:** Recurrent wheeze in preschool children may be associated with lower lung function and airway inflammation, not with AHR or atopy.

## A315 Mannan-Binding Lectin Serum Levels in Healthy Mongolian Adults

### Enkhbayar Bazarsad^1^, Logii Narantsetseg^2^, Munkhbayarlakh Sonomjamts^3^

#### ^1^Ach Medical University, Mongolia; ^2^Department of Cellular Biology and Biochemistry, School of Pharmacy and Bio-Medicine, Mongolian National University of Medical Sciences, Ulaanbaatar, Mongolia; ^3^School of Medicine, Mongolian National University of Medical Sciences, Ulaanbaatar, Mongolia

##### **Correspondence:** Enkhbayar Bazarsad – Ach Medical University, Mongolia

**Backround.** Mannose-binding lectin (MBL) is a protein, which contains lectin and collagen domains, synthesized in the liver. MBL binds to N acetylglucosamine and mannan structures on the surface of bacteria, fungi, viruses and protozoa, leading to opsonization, phagocytosis and activation of complement system through lectin pathway, independent of an antibody. MBL is a vital protein of innate immune system and has two critical functions: complement activation through the lectin pathway and opsonization. Low level of serum MBL is associated with increased susceptibility to infections and high risk of some allergic and autoimmune diseases.

**Purpose.** The purpose of our study was to determine the profile of MBL serum level in Mongolian healthy population the first time.

**Method.** We were collected 112 serum of Mongolian healthy adult blood donors: 58 males and 54 females in 4 different age groups (20-30, 31-40, 41-50 and 51-60). Their mean age was 37.5 years. We were analyzed for MBL level in serum by using double-antibody sandwich ELISA method.

**Results.** The mean and median serum levels of MBL were 2165.07 ± 711.7 ng/ml and 2172.9 ng/ml.

MBL level in male were 2073.33 ± 672.4 ng/ml, female 2250.63 ± 726.4 ng/ml. No association was observed between sex and MBL concentrations.

2483,44 ± 697.58 ng/ml at 20-30 years of age, 2171,73 ± 743.46 ng/ml at 31-40 years, 1806,66 ± 601.26 ng/ml at 41-50 years and 2144,02 ± 493.4 ng/ml in 51-60 years. There were no significant differences in the mean concentration of MBL among different age groups.

**Conclusions.** MBL serum level of Mongolian healthy population is comparatively higher than some other nations’ population. The MBL values of this study can be used as a normal reference range for future studies in Mongolian population.

## A316 Rotanebuliser

### Prabhakarrao Pv^1^, Ranjitha Nadendla^2^

#### ^1^Mnr Medical College, India; ^2^Sai Hospital

##### **Correspondence:** Prabhakarrao Pv – Mnr Medical College, India

**Background:** Many sufferers of Bronchial Asthma and COPD need nebuliser therapy in acute conditions.The rotahaler wih rotacaps delivers only 15 to 20% of the drug. Quite a few subjects do not use rotahalers properly inspite of adequate training. In acute exacerbations, their plight is worse. Hence for proper drug delivery in acute conditions, rotacap with rotahaler may not be adequate. Hence an attempt is made for effective delivery of the entire 200mcg salbutamol through a mechanical nebuliser. In the market only electronically operated nebulisers are available.The cost is still prohibitive .Most of the urban and rural areas do not have regular power supply. Even in the hospitals, the nebulisers are far from few. Conamination is a distinct possibility in the wards and ICU settings. There is also lot of wastage in the side stream of electronic nebuliser. It takes atleast 15 to 20 mins for the entire medicine to be nebulised. Hence an Air Pump driven mechanical nebuliser is developed.

**Material and Methods:** An Air Pump made up of PVC weighing 600gms is fabricated for this device.This has a cylindrical chamber sliding on a steel rod. This has inbuilt posterior and anterior apertures to draw and propel the air out under pressure. There is a pedal mounted on the chamber.The whole unit rests on a metal frame. There is nylon lining for sliding of the cylinder on the inner axial rod. There is no grease applied for the cylindrical chamber to slide over the axial rod. The anterior end is attached to the nebulising unit, which is availabe in the market. The rotacap containing 200mcg of salbutamol is opened .The nebulising chamber is charged with the salbutamol rotacap powder, dissolving in one to two ml of normal saline. The user connects the mask to face and the other end to the the anterior end of the air pump, which has a nozzle. On pedalling the pump, the mist is released. There is no side stream, avoiding wastage.Thus its a self operating one. If required a helper may pedal the pump.

**Results:** One hundred adult patients suffering from bronchial asthma were treated with this device, using salbutamol rotacap powder with normal saline as solvent. Apart from subjective assesment , the improvement was measured with Mini Wright Peak Flow Meter(PEFR). Averge PEFR was 200 lits per min before and 350 lits per min after rota nebulisation. Twenty five subjects with similar complaints were taken as control.They were assesed with salbutamol inhaler attached to spacer. The revesibility of the airways obstruction is better with this device as the entire 200mcg salbutamol is delivered properly. In contrast the drug delivery with the inhaler attached to the spacer is supposed to be only 40%. Over and above the users preferred this device as there is no inspiratory effort.

**Advantages:** 1.Portability 2.Independent of Electricity 3.Ubiquitous Use.4.Long lasting 5.use of rotacap powder instead of costly inhaler.

## A317 The Level of Serum Interleukin 13 and Interleukin 17A and Its Effect Factors in Children with Asthma

### Juan Fang^1^, Jing Zhao^2^

#### ^1^Capital Institute of Pediatrics, China; ^2^Affiliated Children Hospital of Capital Institute of Pediatrics

##### **Correspondence:** Juan Fang – Capital Institute of Pediatrics, China

**Background**

To explore the level of serum Interleukin13(IL-13) and Interleukin 17A (IL-17A) in children with asthma.

**Methods**

A case-control study was performed.88 asthmatic children in asthma clinic and 28 children hospitalized in surgical for noninfectious elective surgery who came to Affiliated Children’s Hospital of Capital Institute of Pediatrics in March to December 2014 were enrolled in the study. Boys 78 and girls 38, aged 5 to 14 years old, the mean age is 7 years 5 month. The level of serum IL-13 and IL-17A were measured by the enzyme-linked immunosorbent assay (ELISA) method. The material of history, blood test, lung function test, the concentrations of fraction of exhaled nitric oxide (FeNO), vitro allergern test of asthmatic children were reserved.

**Results**

The level of serum IL-13 and IL-17A in asthmatics is significantly higher than control (P<0.001). There was a positive relationship between the level of serum IL-17A and the severity of asthma (r_s_=0.369, P<0.001).The level of serum IL-17A between control and chronic persistent, control and exacerbation, chronic persistent and exacerbation are significantly different (P<0.001). The level of serum IL-13 in allergic asthmatic and non-allergic asthmatic are significantly different (Z=-2.655, P<0.01), but there is no difference in the level of serum IL-17A(P>0.05). There is a positive relationship between the level of serum IL-17A and neutrophils in asthmatic children (r_s_=0.216, P=0.047). There is a positive relationship between the level of serum IL-13 and sIgE of inhaled allergens (r_s_=0.343, P<0.01). There is no relationship between the level of serum IL-13 or IL-17A and lung function or FeNO. In severe asthmatic children, there is a positive relationship between the level of serum IL-13 and the level of serum IL-17A (r_s_=0.395, P<0.05).

**Conclusions**

The level of serum IL-13 and IL-17A of asthmatic children is higher than control. There is a positive relationship between serum IL-17A and the severity of asthmatic children. There is a positive relationship between the level of serum IL-17A and neutrophils in asthmatic children. There is a positive relationship between serum IL-13 and sIgE. In severe asthmatic children, there is a positive relationship between the level of serum IL-13 and the level of serum IL-17A.

## A318 Is Vitamin D Insufficiency Also Involved in Childhood Asthma in South Korea?

### Dae-Jin Song^1^, Sungchul Seo^2^, Young Yoo^2^, Yu-Ri Kim^3^, Ji Tae Choung^2^, Jee Hoo Lee^4^

#### ^1^Guro Hospital, South Korea; ^2^Korea University; ^3^Korea University Medical Center; ^4^Korea University Guro Hospital

##### **Correspondence:** Dae-Jin Song – Guro Hospital, South Korea

**Purpose:** Vitamin D is one of essential nutrients for immune modulator effector. Recently, epidemiologic studies have shown that the lack of vitamin D levels may be associated with high asthma prevalence. The purpose of this study was to examine the association of serum vitamin D levels with the prevalence of pediatric asthma in Korea.

**Methods:** This study was conducted on 34 asthmatic children and 30 healthy controls aged 6-14 years. Serum 25-hydroxy vitamin D3 (25-OH vitamin D) levels were measured and compared between the two groups. Moreover, the relationship between serum 25-OH vitamin D levels and pulmonary function test and environmental factors for sunshine were examined in asthmatic patients.

**Results:** Serum 25-OH vitamin D levels in asthmatic patients (16.63 ± 4.20 ng/ml) were significantly (p<0.05) lower than that in healthy controls (24.24 ± 6.76 ng/ml). Also, we found that when serum vitamin D level is 1 ng/ml decreases, the prevalence of asthma increase to 0.788-fold (OR, 0.788; 95% CI, 0.707-0.879; p<0.001). However, there were no associations with vitamin D level and pulmonary function and sunshine related factors, such as housing type, living floor, and indoor/outdoor activity time.

**Conclusion:** These results suggest that serum vitamin D levels were associated with pediatric asthma in Korea. For prevention and treatment of asthma, an intervention study in asthmatic children with low serum vitamin D level, and a vitamin D study assessed the optimal delivery and safety of its supplementation is needed.

## A319 Collection of Nasal Secretions for Measurement of Local IgE: A Quest for the Best Method

### Margot Berings, Natalie De Ruyck, Claus Bachert, Philippe Gevaert, Gabriële Holtappels

#### Ghent University, Belgium

##### **Correspondence:** Margot Berings – Ghent University, Belgium

**Background:** It is of great interest to develop validated, non-invasive methods for local measurement of IgE. This is however challenging, as IgE is the least abundant immunoglobulin isotype and present at low concentrations. We aimed to compare three methods for collection of NS for local measurement of IgE: Filter Disks (FD), Sinus Packs (SP) and Ear Packs (EP). We furthermore evaluated the suitability of using a fixed dilution instead of a fixed volume when processing.

**Methods:** NS were collected with FD and SP in 15 house dust mite (HDM) allergic rhinitis (AR) patients and 15 controls. During processing, saline solution was added in order to mobilize the NS. For each sample, the volume to be added was calculated, based on the weight of collected NS, in order to obtain a *fixed dilution*. In a second experiment, NS were collected in two HDM AR patients (re-recruited from first experiment) with FD and SP on 4 consecutive days. This experiment was performed once with *fixed volume* and once with *fixed dilution*. In a third experiment, NS were collected with FD, EP and SP in 13 HDM AR patients (re-recruited from first experiment). During processing, a *fixed volume* of saline solution was added: 1 ml for FD, 2 ml for EP and 3 ml for SP. Total IgE and hx2-IgE were measured with the UniCap system.

**Results:** In the experiment with fixed dilution, levels of total IgE and HDM IgE were significantly higher in HDM AR patients compared to controls. With the FD, total IgE and HDM IgE were below the detection limit (BDL) in 2 and resp. 6 out of 15 AR patients. With the SP, total IgE and HDM IgE were BDL in 3 and resp. 4 patients. When comparing FD and SP on 4 consecutive days, the reproducibility to measure IgE levels was better with SP. In the third experiment with a fixed volume, total IgE and HDM IgE were BDL in 3 and resp. 6 out of 13 AR patients when using FD and EP. With the SP, total IgE and HDM IgE levels were BDL in 5 and resp. 7 patients.

**Discussion:** Each method has some advantages and disadvantages. The SP method seems to be more reproducible than the FD method, which makes it more suitable for repeated measurements and monitoring. However, SP are unsuitable for serial measurements on the same day, as they cause stimulation of the nasal mucosa. In this setting, FD are preferable. EP are intermediate in size to FD and SP and could be of interest in a pediatric setting. When comparing fixed volume to fixed dilution processing, the latter is more time consuming and prone to error. However, fixed dilution has the advantage of a fixed detection limit for all samples. Therefore, this method is recommended when measuring IgE or other mediators that are present at low concentrations and thus often BDL.

## A320 The Role of Claudin 5 in a Murine Model of Asthma

### Pureun-Haneul Lee^1^, Byeong-Gon Kim^1^, Choon-Sik Park^1^, George D Leikauf^2^, An-Soo Jang^1^

#### ^1^Soonchunhyang University Bucheon Hospital, South Korea; ^2^University of Pittsburgh, USA

##### **Correspondence:** Pureun-Haneul Lee – Soonchunhyang University Bucheon Hospital, South Korea

**Abstract**

**Background:** The tight junction (TJ) protein, claudin 5 (CLDN5) is critical to the control of endothelial cellular polarity and pericellular permeability.In the past, the role of CLDN5 in asthma has had limited attention.

**Objective:** The aim of study was to identify the expression of CLDN 5 and response to steroid treatment in a mouse model of asthma (i.e. ovalbumin (OVA)-induced allergic lung inflammation) and in human lung microvascular endothelial cells (HLMVEC) and normal human bronchial epithelial (NHBE) cells.

**Methods:** Mice were treated with saline (sham), OVA sensitized and challenged (OVA), or OVA with dexamethasone. Lung CLDN5 levels were assessed with qRT-PCR, ELISA, immunoblotting, immunohistochemical stain, and confocal imaging. HLMVEC were treated with host dust mite peptidase (Der p1) or interleukin 4 (IL-4) and CLDN5 mRNA and transmembrane endothelial electrical resistance (TEER) measured.

**Results:** Airway inflammation, hyperresponsiveness, cytokines, and CLDN5 transcript and protein increased in OVA sensitized/challenged mice and these responses were reduced by dexamethasone treatment. The AKT1/FOXO1/CTNNB1 pathway was involved in CLDN5 protein expression. Der p1 increased CLDN5 protein expression in HLMVEC (but not NHBE) cells and decreased trans-endothelial electrical resistance. IL-4 also increased CLDN5 in HLMVEC, decreased TEER, and these effect were inhibited by dexamethasone.

**Conclusion:** CLDN5 is implicated in the pathogenesis of bronchial asthma and represent a potential target for therapeutic intervention.

**Keywords:** Tight junction, claudin 5, bronchial asthma

## A321 Claudin-4 in a Murine Model of Asthma: Modulation By Acrolein, a Highly Reactive Unsaturated Aldehyde

### Byeong-Gon Kimc, Pureun-Haneul Lee, Choon-Sik Park, An-Soo Jang

#### Soonchunhyang University Bucheon Hospital, South Korea

##### **Correspondence:** Byeong-Gon Kimc – Soonchunhyang University Bucheon Hospital, South Korea

**Abstract**

**Background:** The dysfunction of airway barriers contributes to the development and/or exacerbations of allergic airway inflammation. A major irritant in smoke, acrolein can affect bronchial asthma by altering tight junction protein. But the impact of acrolein on asthma remains poorly understood.

**Objective:** The aim of this study was to identify the expression of claudin 4 (Cldn4) and the impact of acrolein on Cldn4 in a mouse model of allergic asthma.

**Methods:** Using mice sensitized with ovalbumin (OVA) and OVA challenged (OVA sensitized/challenged mice) as well as mice treated with saline and challenged with air, and mice exposed to acrolein 5ppm on days 21-23, The effect of acrolein on Cldn4 was estimated using qRT-PCR, ELISA, immunoblotting, immunohistochemical stain, and confocal imaging.

**Results:** Lung Cldn4 transcript and protein were significantly increased in OVA mice than in sham mice. Acrolein exposure reduced the increase in inflammatory cytokine levels, airway inflammation, and bronchial hyperresponsiveness in OVA mice. Increased lung Cldn4 transcript and protein in OVA mice were decreased in acrolein exposed mice.

**Conclusion:** These results indicate that Cldn4 might be involved in protective role in the pathogenesis of bronchial asthma, and acrolein can dysregulate Cldn4.

This research was supported by Basic Science Research Program through the National Research Foundation of Korea (NRF) funded by the Ministry of Education (2013R1A1A2005465).

**Keywords**: Tight junction, claudin-4, acrolein, bronchial asthma

## A322 Efficacy and Safety of Sublingual Immunotherapy in House Dust Mite Sensitized Children with Allergic Rhinitis

### Yang Park

#### Wonkwang University Sanbon Medical Center, South Korea

**Background:** This study investigated the efficacy and safety of sublingual immunotherapy (SLIT) with house dust mite in house dust mite sensitized children with allergic rhinitis.

**Method:** 14 children who were sensitized to house dust mites treated with SLIT enrolled between August 2013 and July 2014. Nasal symptoms (rhinorrhea, sneezing, nasal obstruction, nasal itching, sleep disturbance), antiallergic medications use and presence of adverse events were assessed at 1 month, 2 month visit and thereafter every 3 months visits.

**Results:** The symtoms of allergic rhinitis started to improve after 1 month of SLIT and significantly improved after 12 months of SLIT (P<0.05). The antiallergic medications use decreased significantly with time (P<0.05). The incidence of adverse events was 21.4 % and most occured within the first month of SLIT and disappeared with time. There were no severe adverse events.

**Conclusion:** SLIT for house dust mite is effective and safe in house dust mite sensitized children with allergic rhinitis.

## A323 The Association of Vitamine D Deficiency and Skeletal Muscle Dysfunction in Chronic Airway Disease

### Min-Su Sohn^1^, Hyun Jung Jin^2^, Dong-Won Lee^3^, Jun-Hong Ahn^1^, Jin Hong Chung^1^

#### ^1^Yeungnam University College of Medicine, South Korea; ^2^College of Medicine, Yeungnam University; ^3^Andong Sungso Hospital

##### **Correspondence:** Min-Su Sohn – Yeungnam University College of Medicine, South Korea

**Background**

The aim of this study was to evaluate whether low vitamine D levels were associated with skeletal muscle dysfunction in COPD.

**Methods**

This cross sectional analysis included 1092 COPD participants with FEV1/FVC < 70% (aged >40 years) in Korea National Health and Nutrition Examination Surveys, which were conducted from 2008 to 2011. Body composition were measured by dual-energy x-ray absorptiometry (DXA). Serum 25-hydroxyvitamine D (25(OH)D), parathyroid hormone (PTH) levels were measured. For the criteria for sarcopenia, the recommended criteria of the European Working Group on Sarcopenia in Older People (EWGSOP) and of the Asia Working Group for Sarcopenia (AWGS) were used. We divided subjects to thee group according to 25 (OH)D level. (Deficiency ≤20 ng/ml, insufficiency 21-29 ng/mL, Sufficiency ≥ 30 ng/ml).

**Results**

Vitamine D levels were positive correlated with fat free mass index (FFMI), skeletal muscle mass index (SMI), appendicular skeletal muscle mass index (ASMI) in COPD. (P= 0.010, 0.008, 0.016) However, PTH showed correlation with ASMI only. (P= 0.012) In logistic regression, vitamine D level revealed association with FFMI, SMI, ASMI, which showed lowest P value in ASMI. (P= 0.002, 0.001, <0.001) When classified according to vitamine D level, in deficiency group, prevalence of sarcopenia was higher with trend according to the vitamine D levels. (P <0.001) Using multivariant logistic regression analyses, in deficiency group, the sarcopenia risk increased in which the criteria recommended in EWGSOP and AWGS with the ASMI. (OR 2.519 , P= 0.003; OR 2.066 P= 0.014).

**Conclusion**

Our finding suggest that low vitamine D level is associated to decrease of skeletal muscle mass, particularly in limb muscle dysfunction, which may be a risk factor of sarcopenia in COPD.

## A324 Bacteria Derived Extracellular Vesicles in Indoor Dust Is Closely Associated with Airway Disease and Lung Cancer: Analysis of Indoor Dustâ€™s Microbiome and IgG Sensitization of Indoor Bacteria Derived Extracellular Vesicles

### Sae-in Kim^1^, Han-Ki Park^2^, Do-Yeon Kim^1^, Mina Rho^3^, Jun-Pyo Choi^2^, Yoon-Keun Kim^2^

#### ^1^Ewha Womans University, School of Medicine, South Korea; ^2^Ewha Institute of Convergence Medicine, Ewha Womans University Medical Center; ^3^Department of Computer Engineering, Hanyang University

##### **Correspondence:** Sae-in Kim – Ewha Womans University, School of Medicine, South Korea

**Purpose:** Recent experimental evidence shows that extracellular vesicles (EVs) in indoor dust induce neurtrophilic pulmonary inflammation. In addition, IgG sensitization to indoor dust EVs appears to be a correlation for the development of asthma, COPD, and lung cancer, irrespective of cigarette smoking. In this study, we analyzed indoor dust and dust extracellular vesicles (EVs) microbiome in the apartment and hospitals. Also, we evaluated whether IgG sensitization to bacteria devrived EVs is a risk for the development of asthma, COPD, or lung cancer.

**Methods:** In the apartment and hospital, we collected summer and winter dust. After genomic DNA was extracted from the dust and dust EVs, 16s ribosomal DNA was amplified using the universal primer, sequenced through the next generation sequencer, and then the sequenced data was analyzed using bioinformatics. Then, Serum IgG antibody against major bacteria derived EVs in dust were measured in 90 healthy control subjects, and 294 asthma, 242 COPD, and 325 lung cancer patients.

**Result:** Bacteria and bacteria derived EVs did not differ in diversity and community composition. Our data suggests the composition of a major dust microbiome that includes *Pseudomonas, Acinetobacter, Enterococcus, and Staphylococcus*. As a result of comparing the bacterial composition, *Pseudomonas* was dominant from apartment and summer, while *Acinetobacter* was dominant from hospital and winter. Especially in the winter of hospital, A*cinetobacter* was increased remarlably and diversity was reduced. As a result of Serum IgG antibody against major bacteria derived EVs in dust, adjusted multiple logistic regression revealed that sensitization to each bacteria derived EVs in dust were an independent risk factor for asthma, COPD and lung cancer.

**Conclusion:** Dust microbiome from bacteria and bacteria derived EVs were mostly composed of *Pseudomonas, Acinetobacter, Enterococcus, and Staphylococcus*. IgG sensitization to bacteria derived EVs of indoor dust appears to be a major risk for the development of asthma, COPD, and lung cancer.

## A325 Clinical Care Program for Childhood Asthma (CCP-Childhood Asthma); A Multidisciplinary Team Care at Samitivej International Children’s Hospital

### Wasu Kamchaisatian, Thitikul Hiranras, Surinda Wongpun, Phornthip Chiraphorn, Anupan Tantachun, Wannipa Wongrassamee, Planee Vatanasurkitt, Naratip Somboonkul, Nattipat Juthacharoenwong, Surangkana Techapaitoon, Montri Tuchinda

#### Samitivej International Children’s Hospital, Bangkok Hospital Group, Thailand

##### **Correspondence:** Wasu Kamchaisatian – Samitivej International Children’s Hospital, Bangkok Hospital Group, Thailand

**Background:** Asthma is the most common chronic disease in childhood and is a common cause of hospitalization for children. In 2011, at Samitivej International Children’s hospital (SMICH), there were 210 asthmatic children aged less than 15 years old. 29 of them were hospitalized due to acute asthma exacerbation. One of them was needed to be in PICU and none was dead. A clinical care program to deliver integrated multidisciplinary and organized care plan with continuous quality improvement of hospital system is considered to be the standard care for asthmatic children.

**Methods:** Core team and childhood asthma framework were set up including Pediatric Allergists, Pediatric Pulmonologists, General Pediatricians, Pediatric Nurses at OPD, ER & ward including PICU, Pharmacists at SMICH, which aim to provide comprehensive clinical care program for childhood asthma (CCP-Childhood Asthma) in 2012. We enrolled children with diagnosis of asthma and acute wheezing at OPD/ER, evaluated and considered diagnosis of asthma, then started treatment and re-evaluated for clinical asthma controlled every 1-3 months as to GINA Guideline for Children. All general pediatricians and Pediatric nurses at OPD, ER and ward were trained and implemented about clinical pathway. We initiated Childhood Asthma Camp to provide education about disease to parents and caregivers, together with workshop for inhaler medicines used, self assessment with asthma action plan, and environmental allergen avoidance. Performance measurements included: 1. Administration of systemic corticosteroids during hospitalization within 12 hours, 2. Evaluation of inhaler medicines technique used correctly in each visit, 3. Pulmonary function testing in children older than 7 years old, and 4. Influenza vaccination annually, data were collected and analyzed yearly.

**Results:** Patients in CCP-Childhood Asthma at SMICH were enrolled from 81 children in year 2012 to 119 children in year 2014, the number of hospitalization from asthma exacerbation was decreased from 24 patients in year 2012 to 20 and 13 patients in year 2013 and 2014, respectively. All patients in CCP-Childhood Asthma were received systemic corticosteroids within 12 hours of hospitalization. No any patient was admitted in PICU. More than 80% of patients could demonstrate inhaler drugs used correctly and >60% of them received pulmonary function testing yearly. Influenza vaccination rate in asthmatic children increased from 30.8% in year 2012 to 57.6% and 63% in year 2013 and 2014, respectively. Our CCP-Childhood Asthma was accredited by Joint Commission International (JCI) from USA. in August 2012 which is the first clinical care program outside USA. certified by JCI.

**Conclusion:** Care of children with asthma, which is a chronic disease burdens to their families and needs a comprehensive multidisciplinary team care. This will help improving quality of care for childhood asthma.

## A326 Continuous B Cell Stimulation with CD40 Ligand Induce IgE Isotype Switching

### Sejin An^1^, Jae Ho Lee^2^

#### ^1^Chungnam National University Hospital; ^2^Chungnam National University School of Medicine, South Korea

##### **Correspondence:** Jae Ho Lee – Chungnam National University School of Medicine, South Korea

**Background:** In the T and B cells interaction, activated helper T(Th) cell can stimulate small B cells by the manner of direct physiologic contact each other. The CD40 ligand(CD40L) molecules expressed on the surface membrane of activated Th cells stimulate B cells after binding with bind with B cells. The activated B cells proliferate and differentiate to secrete immunoglobulin IgM, IgG1 and IgE in the presence of lymphokines.

**Methods:** Female BALB/C and CBA/J mice were used between 8 to 16 weeks of age, Plasma membranes were prepared from CD40 mRNA transfected to Baculovirus Sf-9 cells. B cells were incubated with plasma membranes and various cytokines in the round bottomed 96 well plate for various periods of time and B cells were pulsed and harvested to check the DNA synthesis of B cells. The B cell culture supernatants were assayed for IgM, IgG1, and IgE by using isotype specific sandwich ELISA.

**Results:** In the kinetics of B cell proliferation, B cell proliferation was peak at day 4 in the presence of interleukin(IL)-4. In the immunoglobuline synthesis, plasma membrane activated B cells secreted IgM, IgG1, and IgE serially in the presence of Th2 type lymphokines. IgM was produced from second day, IgG1 was third day, and IgE was fifth day of culture.

**Conclusions:** The molecule of CD40L play a major role to activate B cells in the T and B cell interaction. The IL-4 and IL-5 are required to proliferate B cells and induce IgM, and isotype switching to secrete IgG1 and IgE. The dose and duration of reactions with antigen and cytokines play an important role in the B cell proliferation and differentiation.

## A327 Effects of Interleukin-9 on Allergen-Specific Immunotherapy in a Mouse Model of Allergic Rhinitis

### Ji-Hyeon Shin, Soo Whan Kim, Si Won Kim, Jun Myung Kang, Boo-Young Kim, Byung-Guk Kim

#### College of Medicine, the Catholic University of Korea, South Korea

##### **Correspondence:** Ji-Hyeon Shin – College of Medicine, the Catholic University of Korea, South Korea

**Background:** IL-9 is known to participate in induction of allergic responses. The purpose of this study is to investigate the effects of IL-9 on allergen specific immunotherapy in a mouse model of allergic rhinitis.

**Methods:** Six-week-old female BALB/c mice divided into 4 groups: control group, allergic rhinitis (AR) group, immunotherapy (IT) group, and IT with anti-IL-9 antibody (anti-IL-9 Ab) group. All mice except control group were sensitized with ovalbumin (OVA) and aluminum hydroxide 3times for two weeks consecutively. After two weeks, mice except control group and AR group underwent immunotherapy by feeding of OVA. During the immunotherapy, mice in anti-IL-9 Ab group were injected with purified anti-mouse IL-9 Antibody. All sensitized mice were challenged intranasally with OVA. Allergic symptoms and eosinophils in nasal mucosa, interferone-γ, interleukin (IL)-4, IL-9, IL-17, TGF-ß, IL-10, T-bet, GATA-3, ROR- γ t and Foxp3 mRNA expression in nasal mucosa and serum OVA-specific IgE were measured.

**Results:** Serum OVA-specific IgE and Eosinophil counts were significantly decreased in anti-IL-9 Ab group compared with IT group (p<0.05). The levels of mRNA expression of IL-4 were significantly decreased in anti-IL-9 Ab group compared with IT group (p<0.05). The levels of mRNA expression of IL-10 and Foxp3 were significantly increased in in anti-IL-9 Ab group compared with IT group (p<0.05). The levels of mRNA expression of IL-10 and Foxp3 were significantly increased in in anti-IL-9 Ab group compared with IT group (p<0.05).

**Conclusion:** Administration of anti-IL-9 antibody increased the induction of tolerance in a mouse model of allergic rhinitis. These results suggest that anti-IL-9 antibody have immunomodulatory effect on immune tolerance. We claim that the application of this property can enhance the efficiency of allergen-specific immunotherapy.

## A328 Usefulness of Exhaled Nitric Oxide for Evaluating Wheeze and Airway Hyperresponsiveness in Preschool Children

### Ji-Won Kwon^1^, Woo Kyung Kim^2^, Hyung Young Kim^3^, Hyo-Bin Kim^4^, Ju-Hee Seo^5^, So-Yeon Lee^6^, Gwang-Cheon Jang^7^, Young-Ho Jung^8^, Soo-Jong Hong^9^, Byoung-Ju Kim^10^, Dae-Jin Song^11^, Jung Yeon Shim^12^, Jung-Won Lee^13^

#### ^1^Seoul National University Bundang Hospital; ^2^Seoul Paik Hospital; ^3^Pusan National University Yangsan Hospital; ^4^Inje University Sanggye Paik Hospital; ^5^Korea Cancer Center Hospital; ^6^Hallym University Sacred Heart Hospital; ^7^National Health Corporation Ilsan Hospital; ^8^Bundang CHA Medical Center, CHA University School of Medicine; ^9^Research Center for Standardization of Allergic Diseases, University of Ulsan College of Medicine; ^10^University of Cincinnati College of Medicine; ^11^Guro Hospital; ^12^Kangbuk Samsung Hospital/Sungkyunkwan University School of Medicine; ^13^Kangbuk Samsung Hospital, South Korea

##### **Correspondence:** Jung-Won Lee – Kangbuk Samsung Hospital, South Korea

**Objective:** Fractional concentration of exhaled nitric oxide (FeNO) is a known marker of airway inflammation. The aims of this study were to evaluate FeNO, impulse oscillometry (IOS), and spirometry in preschool children and to investigate their relationship with wheeze and airway hyperresponsiveness (AHR).

**Methods:** We performed a population-based, cross-sectional study with 561 children aged 5-6 years. A total of 544 children completed a modified International Study of Asthma and Allergies in Childhood (ISAAC) questionnaire and eligible for the study. We measured FeNO, spirometry, methacholine bronchial provocation, and IOS. AHR was defined as the induction of a 20% decrease in FEV_1_ (PC_20_) by a methacholine concentration ≤ 8.0 mg/dL.

**Results:** Children who had wheeze or AHR had higher FeNO levels than children without these symptoms. However, neither IOS nor spirometry parameters showed significant differences between children with wheeze or AHR and those without. FeNO was associated with AHR, whereas IOS or spirometry parameters showed no association. Mean FeNO levels were positively correlated with a dose-response slope for methacholine, but neither IOS nor spirometry parameters showed significant correlations.

**Conclusions:** FeNO is a more sensitive measurement of AHR and wheeze than spirometry or IOS in preschool children.

## A329 Systemic Cyclosporine Treatment in Hand Eczema Patients

### Kyung Ho Kim

#### Derpartment of Dermatology, National Medical Center, South Korea

**Back ground:** Hand eczema is a commonest disorder afflicting the hands with various morphological forms and with variable severities. Emollients, barrier creams and topical steroid are known to be effective in the majority and form the mainstay of treatment. But in some severe cases or in acute phases of hand eczema, systemic treatment can be very helpful. Among systemic therapy cyclosporine is known to be effect but response rate, remission period and recurrence rate is not well known.

**Objective:** Evaluate the efficacy of systemic cyclosporine in hand eczema patients who are refractory to conventional therapy.

**Methods:** 17 patients with hand eczema were chosen among the patients who had negative patch test results and the patients who had never diagnosed with psoriasis through biopsy. Patients with contraindications of using cyclosporine were excluded. Response rate were evaluated through ‡@Patient’s satisfaction (DLQI), ‡AClinical examination by a physician (PGA) and ‡BPhotographical observation by scoring using total hand eczema severity index (HECSI).

**Result:** Total 17 patients were enrolled and among them 10 were male and 7 were female. Average age was 49 and average disease duration was 2.4 years. 13 patients had hyperkeratotic subtype, 2 with fissured subtype and 2 with pompholyx subtype. 1 patient couldn’t finish the study because of the medication side effect (dizziness). Average initial treatment period was 6.7 weeks 16 patients and all had clinical and subjective improvement after 2-4 weeks of initial treatment. (53% improvement in DLQI, 34.4% improvement in PGA, 63.3% improvement in HECSI) But recur occurred in 4 patients within 4 months after discontinuing the medication (average 2.3 months, recur rate 25%).

**Conclusion:** Systemic cyclosporine can be an effective and relatively safe treatment option in hand eczema patients who are refractory to other treatments, although recur is quite common.

## A330 Lipid Profiles and Adipokines in Korean Children with Atopic Dermatitis

### Young Yoo, Won Suck Yoon, Sungchul Seo, In Soon Kang, Jae Won Choi, Hye-Young Lim, Ji Tae Choung

#### University, South Korea

##### **Correspondence:** Young Yoo – Korea University, South Korea

**Backgrounds:** Atopic dermatitis (AD) is a chronic pruritic recurrent inflammatory skin disorder, which can significant cause of morbidity. Obesity has been shown to have pro-inflammatory immune response. Leptin are adipokine are the obese gene product and secreted by adiposites. The prevalence of both childhood obesity and AD has increased in past few decades. The association between obesity and AD has not been well established.

**Methods:** A total of 227 subjects out of 2207 were defined as having AD based on questionnaire survey. Ninety AD children, aged between 6 and 12 years, completed scoring of severity of AD (SCORAD), blood tests for serum total IgE, blood eosinophil counts, serum eosinophil cationic protein (ECP) and lipid profiles. Serum levels of adipokines such as adiponectin and leptin were measured.

**Results:** There were no significant differences in terms of age, BMI, percentage of breast milk feeding, mode of delivery and prevalence of atopy between boys and girls, and between atopic subjects and non-atopic subjects. Lipid profiles were not different between boys and girls, and between atopic subjects and non-atopic subjects. Regarding to the adipokines, serum leptin levels were significantly higher in girls (2.44 ng/mL [1.40 – 4.22]) compared to boys and, atopic subjects (2.25 ng/mL [1.27 – 3.97]) compared to non-atopic subjects. There were no significant correlations between SCORAD index and serum adiponectin or leptin concentrations.

**Conclusions:** Although serum leptin levels were significantly higher in girls or non-atopic subjects, the SCORAD index was not correlated with those serum lipid profiles or adipokine levels. The lipid profiles and serum adipokine level are not influenced by the severity of AD in these pre-adolescent elementary school children in South Korea.

## A331 Validation of Montelukast and Levocetirizine Combination Tablet Versus Individual Tablets in the Treatment of Moderate to Severe Persistent Allergic Rhinitis Among Adult Filipinos Seen at the Philippine General Hospital-Outpatient Department

### Michelle Buela

#### Philippine General Hospital, Philippines

**Background and Objectives:** Antihistamines and Leukotriene receptor antagonists (LTRA) are both approved treatments of Allergic rhinitis based on the Allergic Rhinitis and its Impact on Asthma (ARIA) guidelines. Recent studies have shown that the combination of second generation antihistamines and montelukast had significant improvement in the nasal symptoms and quality of life of patients with persistent allergic rhinitis. Recently, montelukast and levocetirine combination has been made available in a single tablet form but it has not been evaluated and compared with individual preparations as to its efficacy and control of symptoms in patients with Allergic rhinitis. This study aims to evaluate the efficacy of levocetirizine and montelukast combination tablet versus individual tablets in alleviating symptoms of patients with Moderate to Severe Persistent Allergic Rhinitis.

**Methods:** An 8-week randomized, double-blind, parallel study. Patients were assigned to 2 arms: 14 received levocetirizine 5 mg and montelukast 10 mg individual tablets; 14 received levocetirizine 5 mg plus montelukast 10 mg combination tablet. Symptom scoring using the Specific Immunotherapy Questionnaire (SITQ) were obtained at baseline, then at 2 weeks, 4 weeks and 8 weeks thereafter of treatment.

**Results:** Patients on levocetirizine and montelukast combination tablet had significant improvement on nasal symptoms scores, eye symptom scores, total symptom scores after 2 weeks and 4 weeks of treatment (P<0.05). In patients given levocetirizine and montelukast individual tablets, only the nasal symptoms (diff=2.5, p=0.0123) were significantly improved after 2 weeks of treatment. However, the eye symptom score and total symptom score were significantly improved (p<0.05) after 4 weeks and 8 weeks of treatment**.** The Quality of Life (QOL) scores between the two groups were only significantly different at 8 weeks of treatment with those given the combination tablet having a significant improvement of QOL from baseline (p value=0.03), compared to those given the individual tablets. The mean difference of the symptom scores between the individual tablets and combination tablet of montelukast and levocetirizine showed no significant difference (P>0.05).

**Conclusions:** Treatment with the combination tablet of levocetirizine and montelukast is equally efficacious with the individual preparation in controlling nasal symptoms, eye symptoms, and total symptoms in patients with Moderate to Severe Persistent Allergic Rhinitis. The quality of life scores however, did not show significant improvement after 8 weeks of treatment with the individual tablets. Despite this, results showed no significant mean difference when compared to the change in QOL score of the individual preparation of levocetirizine and montelukast.

## A332 Efficacy of Makyokansekito on Treatment of Wheezing Lower Respiratory Tract Infection in Children: A Retrospective Study of 68 Patients

### Koji Nishimura

#### Ehime Prefectural Niihama Hospital, Japan

**Background:** Lower respiratory tract infection of children is often accompanied by wheezing, and be treated in accordance with bronchial asthma. Makyokansekito is an herbal cough medicine especially for children with asthma. It is not common among pediatricians in Japan. The aim of this study is to evaluate the efficacy of Makyokansekito in lower respiratory tract infection of children.

**Methods:** Subjects were sixty-eight patients (40 boys and 28 girls) hospitalized for wheezing lower respiratory tract infection (bronchitis, bronchiolitis, pneumonia) from April 2012 through March 2014. The median age of subjects was 14 months. The patients treated with Tsumura Makyokansekito (TJ-55) 0.1-0.2g/kg/day were compared with the patients treated without it retrospectively. Comparisons were made with the X^2^-test when appropriate. Values of p<0.05 were considered significant.

**Results:** Makyokansekito significantly decreased the number of days with persistent wheezing (p=0.032, median 2.9 vs 4.2 days) and with feeding humidified oxygen (p=0.035, median 4.7 vs 6.3 days) as compared with control. There were no differences in length of hospital stay. Side effects were not admitted.

**Conclusions:** Makyokansekito is useful in the treatment of wheezing lower respiratory tract infections of children.

## A333 Serum Eosinophilia and Total IgE Are Associated with the Risk of Allergic Sensitization and Allergic Symptoms in Two Years Follow-up, Respectively

### Sang Chul Park^1^, Hyo Jin Chung^1^, Chang-Hoon Kim^1^, Ju Wan Kang^2^, Seong-Chul Hong^2^, Keun-Hwa Lee^2^, Jaechun Lee^3^, Hye-Sook Lee^2^, Jeong Hong Kim^2^

#### ^1^Yonsei University College of Medicine, South Korea; ^2^Jeju National University; ^3^Jeju National University School of Medicine

##### **Correspondence:** Sang Chul Park – Yonsei University College of Medicine, South Korea

**Background**

Allergic rhinitis is increasing steadily in recent days, but there are no specific markers which can predict the risk of allergic sensitization individually. Serum eosinophil, eosinophil cationic protein (ECP), and total IgE (TIgE) are known to increase in patients with allergic disease. However, the clinical significance of these serologic results are not well evaluated. We aimed to investigate whether serum eosinophil, ECP, and TIgE are associated with increased risk of allergic sensitization and allergic symptoms on the basis of 2 years follow-up study.

**Methods**

In 2012, serum eosinophil, ECP, and TIgE were measured in 3rd and 4th grade students of 5 elementary schools. Skin prick test was performed with 26 aeroallergens commonly found in Korea. The presence of allergic symptoms during past 12 months was checked using questionnaire. In 2014, same study was performed in 5th and 6th grade same students attending same schools. The cut off value and usefulness of serologic markers (serum eosinophil, ECP, and TIgE) were calculated using the receiver operating characteristic curve.

**Results**

Serum eosinophil fraction (Cut off value 3.8%) was associated with the newly developed allergic symptoms (sensitivity 41.8, specificity 77.9; Odd ratio 2.424, p = 0.006). Higher serum total IgE (cut off value 17.7 IU/ml) was also associated with the risk of allergic sensitization (sensitivity 85.3, specificity 46.0; Odd Ratio 4.848, p<0.001).

**Conclusions**

Serum eosinophilia and total IgE were associated with the future risk of allergic symptoms and allergic sensitization, respectively. Further study is needed to elucidate the predictability of serum eosinophil and TIgE to consider the future risk of allergy.

## A334 The Sensitization to *Russian Thistle* on Mongolian Patients

### Narantsetseg Logii^1^, N.Nyamdavaa^2^, B.Enkhbayar^3^, B.Oyuntsatsral^2^, S.Munkhbayarlakh^4^

#### ^1^Mongolian National University of Medical Sciences, Mongolia; ^2^Department of Cellular Biology and Biochemistry, School of Pharmacy and Bio-Medicine, Mongolian National University of Medical Sciences, Ulaanbaatar, Mongolia; ^3^Department of Physiology and Molecular Biology, Ach Medical University, Ulaanbaatar, Mongolia; ^4^School of Medicine, Mongolian National University of Medical Sciences, Ulaanbaatar, Mongolia

##### **Correspondence:** Narantsetseg Logii – Mongolian National University of Medical Sciences, Mongolia

**Background:** In Mongolia, out of 203 species of plants which cause allergies, 43 species of plants identified as allergens. In the family *Amaranthaceae (Chenopodiaceae),* several annual species of the genus *Salsola* are the most notorious tumbleweeds.*Salsola tragus* is the so-called "Russian thistle". Over the last few years in Mongolia, spread of allergic diseases among adults and children has been increasing noticeably. A plant, Russian thistle pollen allergy is increasing. Sensitization to Russian thistle is not found that many in the country. So this led to carry out this study.

**Мethods:** The study was made by the Department of Cell biology and biochemistry, School of Pharmacy and Bio-Medicine, MNUMS and Effect allergy and asthma clinic. We were used the method of skin prick test with allergen of Russian thistle (Allergy Laboratories Inc, USA). Wheal sizes were measured as the mean of the 3 extreme diameters 15 to 20 minutes after the prick. Reactions were considered positive if the wheal was larger than the negative control by 3 mm or more. On the basis of this analysis, the sensitization rates of the Russian thistle.

**Result:** We were tested specific activity of Russian thistle with over 40 kinds of allergens to 1031 cases with respiratory allergic anamnesis in allergy. 104 cases (10.08%) out of all cases with age range 3-74 years old and 42.3% were women and 57.7% were men.Severity of allergen sensitization is done by comparing average diameters of wheals from positive results. The result is divided into 4 groups as follows: 3 mm weak, 3.5 - 4 mm^2^ middle, 4.5 - 5.5 mm^2^ sever, 6mm^2^ and above very sever. The mean diameter of wheals that had sensitized to *Russian thistle* was 5.31 ± 0.48 mm^2^ and 0.1% histamine hydrochloride positive control was 4.89 ± 1.32 mm^2^. After the skin prick tests, when calculating people sex along with average diameters of wheals from allergen sensitization to Russian thistle’s pollen, people between age of 3-18 had sever sensitization, 19-39 sensitization was very sever.

**Conclusions:** The sensitization to *Russian thistle* was mild (10.08%) among adult subjects with seasonal allergic rhinitis in Mongolia.

## A335 The Association Between Air Pollution, Allergic Sensitization to Inhalant Allergens and Airway Hyperresponsiveness in Ulaanbaatar, Mongolia

### Enkhbayar Bazarsad^1^, Munkhbayarlakh Sonomjamts^2^

#### ^1^Ach Medical University, Ulaanbaatar, Mongolia; ^2^Mnums, Ulaanbaatar, Mongolia

##### **Correspondence:** Munkhbayarlakh Sonomjamts – Mnums, Ulaanbaatar, Mongolia

**Background:** The prevalence of asthma among population of different countries of the world are 2-18%. In Mongolia, the prevalence of asthma was 2.1% in Ulaanbaatar and 1.1% in countryside villagesin 2000. Also, we were defined prevalence of asthma was 5% among adult population of UlaanbaatarIn 2010. The levels of air pollutants NO_2_, CO, SO_2_, PM_20_ and PM_10_ are significantly increasing in air of Ulaanbaatar city during the last decade and it is dependent from cold season.

**Purpose:** Aim of this study was to define association between the levels of air pollutants, sensitization to inhalant allergens and airway hyperresponsiveness.

**Methods:** We were interviewed by questionnaire from 283 subjects. We used skin prick tests 37 different inhalant allergens (AllergyLabs USA) and detected allergen specific-IgE by immunoblotting (RIDA screen, r-biopharma, Germany). The lung ventilation function were defined FEV_1_, FVC, PEF, FVC/FEV_1_ parameters of spirometer test (Spirostar USB, Medikro OY, Finland). Metacholine challenge test (MCT) were done by 5 steps nebulization (Spira electro 2 dosimeter, Finland) with 2.5 and 25 mg/ml methacholine.

**Results:** We were interviewed 16-60 (average age 35±12) aged 283 subjects with asthma symptoms and normal lung function by spirometer. 89.6% (95% CI: 92.4-86.9) out of subjects had positive results on MCT in winter season with air polluted by CO, SO_2_ and PM_20_.18%, 36% and 46% out of patients with positive results on MCT were severe, moderate and mild changes respectively. 68.9% out of subjects with airway hyperresponsiveness were sensitized to aeroallergens. 73.4% out of them strongly sensitized to mugwort (mean size of wheals 17±3.1 мм) and 17.7% weakly sensitized to dust mites and animal dander (mean size of wheals 9±3.6мм). 79.6% out of subjects were high level of allergen-specific IgE (mean level 4.6±1.1ml/IU) in sera of peripheral blood by the immunoblotting (RIDA screen, r-biopharma, Germany).

**Conclusion:** The winter season is a coldest and highest air polluted period of time by CO, SO_2_ and PM_20_ in Ulaanbaatar and it is become strongly risk factors of airway hyperresponsiveness. The sensitization to mugwort and grasses aeroallergens and the levels of NO_2_ pollutants were related to impact of lung function disorder and airway hyperresponsiveness.

## A336 Pre-Coseasonal Treatment with a 5-Grass Pollen Sublingual Tablet in Adults Demonstrated a Reduction on Asthma Symptoms in Réunion Island

### Bashir Omarjee

#### D’allergologie Et Exploration Du Sommeil, Reunion

**Background:** Asthma is a heterogeneous disease, not only in its clinical expression and course but also in its response to treatment. Most patients are clinically stable with current therapies, while a substantial part of the asthma population develops exacerbation during grass pollen season.

The purpose of this study is to document the impact of a grass allergy immunotherapy tablet (AIT) on the symptom severity in patients with severe allergic rhinitis (SAR) and persistent allergic asthma (PAA).

**Method:** This study included 22 adults, aged 20-45 yrs, with PAA and SAR who met the following inclusion: ≥ two severe asthma exacerbations requiring oral corticosteroids while receiving high dose ICS (≥1600 *ug* BECLOMETHAZONE daily or equivalent), additional controller medications (long acting *B2* agonist) during one year prior to screening. Patients were examined at baseline and received a tablet 300IR (GRAZAX**®** ALK or ORALAIR**®** Stallergènes SA) approximately 2 months before the expected start of the grass pollen season in Réunion Island and then throughout the season between November 2013/April 2014 and November 2014/April 2015.

**Results:** AIT significantly reduced scores from baseline in ocular and nasal symptoms. Reductions were also seen in the asthmatic scores: improvement of FEV_1_ (*p<0.05* of predicted values) and breathlessness scores (*p=0.0002*). The asthma quality-of-life (AQLQ) questionnaire scores improved from baseline after AIT: *p=0.003*. At the final visit only 24% had daily activity impairment and 34% had some sleep impairment.

**Conclusion:** The Pre-Coseasonal treatment with 5-grass AIT showed effective symptom control in severe persistent allergic asthma. Symptoms of comorbidities such as rhinitis and conjunctivitis were decreased.

## A337 Peak Expiratory Flow Rate Reference Values for Children Aged 5–14 Years Old in Beijing Urban Area

### Shuo Li

#### Capital Institute of Pediatrics, China

**Object:** To obtain the peak expiratory flow meter rate (PEFR) normal value in healthy children 5 to 14 years old from Beijing urban area and to establish the predicted equations of PEFR in children. We also compare the values of PEFR measured by the Mini Wright peak flow meter with peak expiratory flow (PEF) measured by the spirometry.

**Methods:** F425 healthy school children (213 boys and 212 girls) aged 5 to 14 years old were chosen from kindergarten, primary and middle schools in Beijing urban area. We used peak flow meter (Mini - Wright, AFS) from PARI company of German to measure peak expiratory flow rate and recorded gender, age, height, weight and other physical parameters. Flow-volume curve was carried out using Jaeger spirometry instrument and peak expiratory flow (PEF) values were adopted. The difference peak expiratory flow values between two measures were compared using SPSS13.0 statistical software. Stepwise multiple linear regression was used to derive the regression equations.

**Results:** The values of PEFR increased along with age among children. There were statistically significant difference between different age groups (*P* <0.05). The PEFR values of boys were higher than those of girls and the difference between males and females reached significant level at the age of 11, 13 and 14 (*P* <0.05). Either in boys or girls, PEFR significantly correlated with age, height, weight, the high degree of correlation existed with height and then age and weight. The predicted equations of PEFR reference values was established in children 5–14 years old living in Beijing urban area as follows: PEFR(L/min)=5.29×H |427.1(boys)and PEFR(L/min)=4.94×H |399.8(girls) respectively. The PEFR value measured by peak flow meter (309.1 ± 74.1 L/min) was higher than those measured by spirometry (298.9±91.3 L/min), and the difference between was statistically significant (*P* < 0.001).

**Conclusions:** New reference values of the peak expiratory flow rate were determined, and the predicted equations were built for children 5 to 14 years old in Beijing urban area, and providing evidence for the clinical management of respiratory diseases.

## A338 Soybean Storage Proteins As the Main Allergen in a Patient with Food-Dependent Exercise-Induced Anaphylaxis Due to Tofu

### Miyuki Hayashi, Ruby Pawankar, Shingo Yamanishi, Toru Igarashi, Yasuhiko Itoh

#### Nippon Medical School Japan

##### **Correspondence:** Miyuki Hayashi – Nippon Medical School, Japan

**Background:** Food-dependent exercise-induced anaphylaxis (FDEIA) is a disorder where exercise following allergen ingestion triggers anaphylaxis although exercise and allergen exposure are independently tolerated. There are an increasing number of reported cases of anaphylaxis due to soybean, but FDEIA due to soybean is a rare disorder.

**Methods:** We characterized the clinical features of a 10 year old boy with a history of walnut allergy who developed FDEIA due to tofu (a soybean product). The patient developed anaphylaxis while running during his physical exercise class after eating tofu. He presented with symptoms of cough, nasal obstruction, generalized urticaria, loss of activity and cyanosis. His symptoms improved an hour after treatment with loratadine but he was not administered epinephrine. In order to detect the causative allergenic food and other cofactors that induced the symptoms of FDEIA, we performed specific IgE test, skin prick test and ISAC. Immunoblot analysis for soybeans and soybean products using the patient’s serum was also performed. Provocation tests with ingestion of tofu followed by exercise is also scheduled to be done to further confirm the diagnosis.

**Results:** Skin prick test with raw soybean product (tofu, fried tofu and soy milk) was strongly positive. The level of serum specific IgE to soybean was 11.90 UA/ml. The ISAC(Phadia, Uppsala, Sweden) results revealed Gly m 4, Gly m 5, and Gly m 6 as 3.3, 0.3, and 7.1 ISU, respectively. About a year and a half later, the specific IgE to soybean, Gly m 4, Gly m 5, Gly m 6 were 40.2, 3.61, 21.4, 51.7 UA/ml, respectively. The patient is well tolerant to soybean products in the absence of any exercise following the intake of the soybean products. Immunoblot analysis of soy powder with patient’s serum showed positive band between 50 and 70 kilo daltons, indicating the presence of specific IgE against storage proteins Gly m 5 and Gly m 6.

**Conclusions:** These results suggest the strong possibility of storage proteins such as Gly m 5 and Gly m 6 as the causative allergen of FDEIA induced by soybean (tofu).

## A339 A Study of Allergy Skin Prick Test with Weed Pollen

### B. Gantulga^1^, B. Enkhbayar^2^, S. Munkhbayarlakh^1^, L. Narantsetseg^3^, Oyuntsatsral Batsaikhan^4^

#### ^1^School of Medicine, Mongolian National University of Medical Sciences, Ulaanbaatar, Mongolia; ^2^Department of Physiology and Molecular Biology, Ach Medical University, Ulaanbaatar, Mongolia; ^3^Department of Cellular Biology and Biochemistry, School of Pharmacy and Bio-Medicine, Mongolian National University of Medical Sciences, Ulaanbaatar, Mongolia; ^4^Mnums Mongolia

##### **Correspondence:** Oyuntsatsral Batsaikhan – Mnums, Mongolia

**Background**

The prevalence of allergic disease has risen in the last few years in Mongolia. Artemisia species is an anemophilous genus included in the *Compositae* family and is widely spread the Mongolian temperate climate zone. Pollen from the various *Artemisia vulgaris (mugwort*) is one of the main causes of allergic rhinitis in late summer and autumn in Mongolia, where the frequency of sensitization approximately 67% of 256 adult patients with sensitized plant pollen in 2010. We aim to determine the sensitivity for pollen allergy of *mugwort* and *lamb’s quarters*.

**Method:** The Research is been done under the department of Cellular biology Biochemistry of Pharmacy –Bio Medicine School, MNUMS with the help of “ Effect” Allergy – Asthma Hospital. During the study of research, one period descriptive research is done by studying the selected 191 patients who are diagnosed positive for the pollen allergens by skin pricking test and these group is chosen from the airborne allergic patients “Effect” Allergy – Asthma Hospital in 2010–2012 census.

**Results:** We were chosen 191 subjects who are sensitized to pollen allergens. All of cases with age range between 3–74 years old and sex ratio is women and men are 42.3% and 57.7%. 169 (88,48%, 95% CI:) out of total subjects were sensitized to allergens of different plants pollen. the average diameters of wheals that had sensitized to mugwort (Artemisia vulgaris) was 9.29±5.01 mm^2^, to lamb’s quarter was 4.85±2.15 mm^2^, to positive control histamine hydrochloride 0.1% was 4.89±1.33 mm^2^.

**Conclusion:** The sensitization of levels to *Mugwort* increased in last few years and the wheals size is increasing year by year.

## A340 The Role of Neurotrophin in a Murine Model of House Dust Mite Induced Allergic Rhinitis

### Pei-Chi Chen^1^, Jiu-Yao Wang^2^

#### ^1^National Cheng Kung University Taiwan; ^2^National Cheng Kung University Hospital

##### **Correspondence:** Pei-Chi Chen - National Cheng Kung University, Taiwan

Allergic rhinitis (AR) is an airway hyper-responsiveness (AHR) and mucosal inflammation disease mediated by IgE-associated processes that is characterised by sneezing, nasal congestion, and rhinorrhea. The imbalance of Th1/Th2 immune response is considered to contribute to allergic diseases, however the interval between inflammation and AHR remains unclear. Growing information illustrated that nerve growth factor (NGF), a neurotrophin, plays an important role in neuroimmune interactions by augmenting an existing Th2 immune response. Since probiotics and biocompatible water-soluble chitosan (WSC) have been demonstrated to have anti-inflammatory properties that could inhibit the development of allergic Th2 response, we aim to assess the effect of WSC and probiotic extracts on NGF in Dermatophagoides pteronyssinus (Der p)-induced AR murine model. Intranasal administration of both WSC and probiotic extracts attenuated AHR in Der p-challenged mice due to a lower respiratory resistance and improved the nasal congestion by manifestation higher respirator rate than non-treated mice. Under management of both WSC and probiotic extracts, the thickness of nasal respiratory epithelium was reduced in microscopy. Both of WSC and probiotic extracts treatment moderated allergic inflammation including a decreased level of total and Der p-specific IgE in the serum, lowered expressions of IL-4, IL-5, and IL-13 in nasal lavage fluid, as well as less eosinphil infiltration in the nasal cavity. In particular, therapeutics with both treatments reduced NGF performance in nasal lavage fluid along with its receptors, p75NTR and TrkA, in the respiratory epithelium of nasal mucosa in Der p-stimulated mice. We suggested that the reduced NGF and its receptor levels may correspond to a decrease in AHR and mucosa inflammation by both WSC and probiotic extracts treatment.

## A341 Mimotopes of the Major Shellfish Allergen Tropomyosin Suppress Splenocyte Proliferation and Local Cytokine Expression in a Mouse Model of Shellfish Allergy

### Nicki Y.H. Leung^1^, Christine Yee Yan Wai^1^, Patrick S.C. Leung^2^, Ka Hou Chu^1^

#### ^1^The Chinese University of Hong Kong Hong Kong; ^2^University of California, Davis, USA

##### **Correspondence:** Nicki Y.H. Leung - The Chinese University of Hong Kong, Hong Kong

**Background:** Mimotopes are short peptides mimicking epitopes. The potential of mimotopes as treatments for allergy diseases were investigated.

**Methods:** Mimotopes specific to the epitopes of the major shellfish allergen tropomyosin were identified by screening the one-bead-one-compound (OBOC) peptide library. The OBOC library is a chemical synthetic library allowing high throughput screening of mimotopes with quantitative estimation on binding affinity. This method is advantageous over the conventional phage-displayed libraries by allowing the use of polyclonal antibodies or even untreated serum samples. The mimicry potential of the mimotopes was validated by both *in silico* and *in vivo* analysis. To investigate the therapeutic potential of mimotopes for allergy diseases, we used a mouse model of shrimp allergy through intragastric gavage of tropomyosin with cholera toxin as adjuvant followed by oral challenge. Splenocyte proliferation and local cytokine expression in the jejunum were analyzed to elucidate possible mechanisms of therapeutic effects of the mimotopes.

**Results:** Twenty-five mimotopes specific to shrimp tropomyosin were identified by screening OBOC peptide library. *In silico* analysis revealed six clusters of mimotopes, with the mimotopes in each cluster sharing at least three or more identical amino acid residues at the same position. With the automated epitope mapping tool EpiSearch, the six clusters of mimotopes could be mapped to six epitope regions of shrimp tropomyosin, of which five were identical to the previous reported epitopes. One mimotope from each cluster were synthesized and conjugated to the carrier protein keyhole limpet hemocyanin (KLH) for *in vivo* analysis. BALB/c mice immunized with mimotope-KLH conjugate were found to have an elevated level of tropomyosin-specific IgG but not in mice immunized with KLH alone or an irrelevant mimotope. The therapeutic potential of these mimotopes were further investigated with the use of the BALB/c mouse model of shrimp allergy. Sensitized mice were injected with a mixture of six mimotope-KLH conjugates, one from each cluster, before receiving a subsequent oral challenge. Compared to the control mice receiving KLH alone, the mimotopes-treated mice demonstrated a suppressed splenocyte proliferation response to tropomyosin and a reduced expression of cytokines in the jejunum.

**Conclusion:** The OBOC peptide library is a useful tool in identifying mimotopes for allergens with multiple epitopes. Mimotopes specific to the tropomyosin were identified by screening OBOC library and validated by *in silico* and *in vivo* experiments. The mimotopes could be potential therapeutic candidates for allergy diseases.

[The present work was supported by grants from the Research Grants Council (CUHK 463911) and the Health and Medical Research Fund (02130206), HKSAR Government and from the Food Allergy and Anaphylaxis Network.]

## A342 A Questionnaire Survey on Understanding of Atopic Dermatitis Among Korean Patients and Caregivers

### Eun Jin Doh^1^, Dong Hun Lee^1,2^, Mira Choi^1,2^, Hyun-Sun Yoon^2,3^, Kyu Han Kim^1,2^, Ji Soo Lim^1^

#### ^1^Seoul National University Hospital South Korea; ^2^Seoul National University; ^3^Seoul National University Boramae Hospital

##### **Correspondence:** Eun Jin Doh - Seoul National University Hospital, South Korea

**Background:** Therapeutic education is important for successful management of Atopic dermatitis (AD). To provide effective therapeutic education, common misunderstandings and demands about AD among patients and caregivers need to be reviewed.

**Methods:** A questionnaire survey about the course, etiology and management of AD was conducted for patients and caregivers who visited Department of Dermatology at Seoul National University Hospital, Seoul, Korea.

**Results:** A total of 177 subjects participated in the study. A few subjects understood natural course of AD. Only 34.5% of subjects was aware of natural course of AD that usually improves with age. Many subjects (52.6%) misunderstood relapse of AD symptoms for development of tolerance to topical steroids. 158 (89.3%) subjects believed that enhancement of patients’ immune system can improve the symptoms of AD. Dietary restriction is considered as an essential management strategy (72.9%), and many of them (55.4%) agreed to postpone the beginning of weaning food in patients with AD. Food, thought to be associated with an aggravation of AD were as follows in the order of; instant food, snack, egg and wheat (38 (25.6%), 32 (21.5%), 19 (12.8%) and 18 (12.1%) of 149, respectively). Most subjects did not have accurate information about cleansing. In particular, 34.3% of subjects reported that they used only water without any cleanser, and 27.3% agreed that soap made of natural ingredients should be used to avoid harmful effects of chemical substances. Most subjects (57 of 115, 49.6%) obtained information about AD from medical doctors, and consider them as the most reliable sources (137 of 164, 83.5%). Subjects prefer printed materials (69 of 162, 42.6%) to seminars or video-clips for obtaining educational contents.

**Conclusion:** In this study, we found that patients and caregivers have lots of misunderstandings about AD. Therapeutic education about the course, etiology and management of AD with printed materials made by physicians will be valuable for the effective management of AD.

## A343 Comparison of the Dosage of Bronchodilators in the Bronchodilator Response Test in Children

### Ji Hyeon Baek^1^, Man Yong Han^2^, Seung Jin Lee^2^, Youhoon Jeon^1^, Kyung Suk Lee^3^, Young-Ho Jung^2^, Hye Mi Jee^3^, Youn Ho Shin^2^

#### ^1^Dongtan Sacred Heart Hospital, College of Medicine, Hallym University South Korea; ^2^CHA University School of Medicine; ^3^Bundang CHA Medical Center, CHA University School of Medicine

##### **Correspondence:** Ji Hyeon Baek - Dongtan Sacred Heart Hospital, College of Medicine, Hallym University, South Korea

**Background:** It is recommended to use 200 (2 puffs) or 400 (4 puffs) ug of salbutamol in the bronchodilator response (BDR) test. We aimed to compare the difference between these two dosages with regard to the small airway dysfunction.

**Methods:** Subjects, who had never been diagnosed as asthma, were consecutively enrolled from June 1st to November 31st, 2013. Based on the subject’s past and family history, we evaluated the possibility of asthma by scoring each subject on a scale of 0 to 10 (pre asthma score). The subjects were randomly assigned the bronchodilator tests of the two dosages without physician’s knowledge and performed the BDR tests using the spirometric and impulse oscillometric lung function. Asthma diagnosis (post asthma score) was later re-evaluated after BDR test.

**Results:** A total of 119 subjects participated in this study, and the mean age was 7.8 (±3.6) years. The number of participants who were assigned 2 puffs and 4 puffs were 59 and 57, respectively. The mean age of 4 puffs group was older than the 2 puffs group (*p*=0.012). Before the BDR test, there was no statistical difference in pre asthma score between the two groups (2 puffs = 5.46 vs. 4 puffs = 4.9) (*p*=0.428). After the BDR test, the post asthma scores of the two groups were 5.8 (±3.4) and 4.7 (±3.4), respectively, which also showed no statistically significant difference between the two groups (*p*=0.098). The pre asthma score was significantly correlated with forced expiratory volume in 1 sec/forced vital capacity (FEV1/FVC) (r=−0.212, *p*=0.021), forced expiratory flow at 25% to 75% (FEF25-75) of FVC (r=−0.184, *p*=0.046) and reactance at 5 Hz (Xrs5) (r=0.201, *p*=0.029) Z score. However, there was no significant difference in FEV1 and FEV1/FVC of spirometric parameters, and resistance at 5 Hz (Rrs5) and Xrs5 of impulse oscillometry system (IOS) value between the 2 puffs group and 4 puffs group.

**Conclusion:** There was no significant relationship between the amount of bronchodilators administered and the small airway dysfunction in children. However, Xrs5 showed a significant correlation with the physician’s asthma predictive score.

## A344 The Expression and Effect of Natural Killer T Lymphocytes in Chidren with Asthma

### Yi Jiang, Miao Liu

#### Renmin Hospital, Wuhan University China

##### **Correspondence:** Yi Jiang - Renmin Hospital, Wuhan University, China

**Objective:** To study the effect of natural killer-T (NKT) lymphocytes and CD4+ NKT lymphocytes levels in peripheral blood onset of children with asthma.

**Methods:** 85 asthmatic children who were diagnosed and treated by pediatric department of Renmin Hospital Affiliated to Wuhan University from Jan. 2012 to Dec. 2014 were selected as asthmatic group. 76 healthy children were selected as control group. The peripheral blood mononuclear cells were collected by using the density gradient centrifugation method. The ratio of peripheral blood NKT cells and CD4+ NKT cells were measured by immunofluorescence and flow cytometry assays. The relationships between the NKT cells number, CD4+ NKT cells and the total IgE level were observed. The levels of IL-4,IL-13, IFN-γ in peripheral blood were detected by enzyme-linked immunosorbent assay after proliferate in response to α-Galcer.

**Results:** Compared with the control group, the ratio of NKT cells and CD4+ NKT cells in peripheral blood in asthmatic group were significantly decreased (t=3.795, P<0.05; t=4.106, P<0.05). There was no significant correlation between the NKT cells, CD4+ NKT cells and the total IgE (t=1.032, P>0.05; t=0.856, P>0.05). The levels of IL-4 and IL-13 in asthmatic group were higher than that of the control group (t=4.683, P<0.05; t=3.992, P<0.05). There was no significant difference in the level of IFN-γbetween the two groups (t=0.877, P>0.05).

**Conclusion:** The dysfunction of NKT cells and CD4+ NKT cells and the functional change of cytokines may play an important role in the pathogenesis of asthma.

## A345 Oral Provocation Test in Non-Steroidal Anti-Inflammatory Drug Hypersensitive Patients Referred to Singapore General Hospital

### Chaw Su Naing, Tze Chin Tan, Yong Yeow Chong

#### Singapore General Hospital Singapore

##### **Correspondence:** Chaw Su Naing - Singapore General Hospital, Singapore

**Background**

Non-steroidal anti-inflammatory drugs (NSAIDs) are frequently prescribed classes of drugs and are easily accessible as over-the-counter anti-inflammatory drugs in Singapore. NSAIDs hypersensitivity is the second most common referral to allergy clinic in a tertiary referral centre.

**Methods**

Referred patients with history of NSAID-induced urticarial, angioedema or anaphylaxis underwent open challenge with (1) putative NSAID to confirm the diagnosis; (2) Aspirin to determine the cross-reactivity or (3) selective cyclooxygenase-2 (COX-2) inhibitor to identify the suitable alternative. Data were analysed retrospectively.

**Results**

Over a 4-year period (2010–2014), 127 patients with mean +/− SD age, 40.7 +/− 15.2 year, underwent a total of 155 open-labelled labelled NSAIDs oral provocation tests (OPT). Patients demographics consisted of female (63.8%, 81) with majority Chinese ethnic group (80.3%, 102). Diclofenac (20.2%) and Naproxen (19.7%) were the two commonest reported culprits NSAIDs. In 32 (25.2%) patients, more than 2 eliciting NSAIDs were recorded to cause hypersensitivity reactions. However, 27 (21.3%) patients reported to tolerate different groups of NSAIDs and 40 (31.5%) had concomitant intolerance to Acetaminophen. Urticaria and/or angioedema were the most frequently reported symptom (87.3%), among which 60.6% were isolated periorbital angioedema. Reaction involving the airways i.e., asthma with or without a naso-occular symptoms were rare (7%). Anaphylaxis was reported by 4(3.1%) patients together with other concomitant drugs.

Breakdown for 155 challenges was as follows: 68 (43.9%) Putative NSAIDS challenge, 29 (18.7%) Aspirin challenge and 58 (37.4%) selective COX-2 inhibitor challenge. Overall positive challenge rate was 24.5% (38 out of 155). Despite having a clinical relevant history of causative, only 29.4% (20 out of 68) had positive OPT to putative NSAIDs. Aspirin challenge resulted in 48.3% (14 out of 29) positive challenge, hence confirming the diagnosis of NSAIDS intolerance. Using selective COX-2 inhibitor challenge, we found only 6.9% (4 out of 54) positive challenge. For the anaphylaxis cases, cautious OPT with putative NSAIDS were done in 75% (3 out of 4) patients. No reaction was found.

**Conclusions**

Without validated skin test, OPT helps evaluate NSAIDS hypersensitivity patients. Our 4-year patients cohort with a positive OPT rate of 24.5% confirmed diagnosis of NSAIDs hypersensitivity. With low positive OPT rate of 6.9%, selective COX-2 inhibitor can be used as alternative in these patients.

## A346 Different Phenotypes of Bhr (bronchial hyperresponsiveness) By Natural Course in Children and It’s Characteristics

### Young-Ho Kim^1^, Eun Lee^1^, Song-I Yang^2^, Hyun-Ju Cho^1^, Hyung Young Kim^3^, Ji-Won Kwon^4^, Young-Ho Jung^5^, Byoung-Ju Kim^6^, Ju-Hee Seo^7^, Ho-Jang Kwon^8^, Hyo-Bin Kim^9^, So-Yeon Lee^2^, Soo-Jong Hong^10^, Soo Hyun Kim^1^

#### ^1^Asan Medical Center South Korea; ^2^Hallym University Sacred Heart Hospital; ^3^Pusan National University Yangsan Hospital; ^4^Seoul National University Bundang Hospital; ^5^Bundang CHA Medical Center, CHA University School of Medicine; ^6^University of Cincinnati College of Medicine USA; ^7^Korea Cancer Center Hospital; ^8^Dankook University College of Medicine; ^9^Sanggye Paik Hospital; ^10^Research Center for Standardization of Allergic Diseases, University of Ulsan College of Medicine

##### **Correspondence:** Young-Ho Kim - Asan Medical Center, South Korea

**Purpose:** Bronchial hyperresponsiveness (BHR) is a key feature of asthma, but the natural course of BHR is heterogenous. We divided the BHR changing pattern into 4 different phenotypes and investigated the characteristics and the risk factors from the longitudinal study.

**Methods:** Total 658 (male 342, female 316) elementary school children were included from the CHEER (children’s health and environment research)study. The ISSAC questionnaire, serum total IgE level, blood eosinophil percentage, skin prick test, pulmonary function test and methacholine challenge test were done at age 7 for baseline and age 11 years after follow up. BHR was defined as provocative concentration of 20% decrease of FEV1 (PC_20_) below 16 mg/m. We divided 4 different phenotypes of BHR change pattern (BHR never, BHR remission, BHR new, BHR persistent). Multinomial logistic regression analysis was done to evaluate the risk factors for each type.

**Results:** Four phenotypes of BHR were composed of 376 (BHR never), 152 (BHR remission), 48 (BHR new), 82 (BHR persistent) respectively. Parental allergic disease (aOR=3.334, 95% CI 1.188-9.358) and asthma (aOR=4.623, 95% CI 1.790-11.944) history at age 7 years were risk factors for BHR remission group. Atopic sensitization (aOR=3.233, 95% CI 1.436-7.279) at age 7 years was a risk factor for BHR new group. Eosinophil percent (aOR=1.280, 95% CI 1.123-1.458), logIgE (aOR=2.757, 95% CI 1.443-5.269), and atopic sensitization (aOR=2.461, 95% CI 1.163-5.208) at age 7 years were risk factors for BHR persistent group. *Dp* sensitization was a risk factor for for BHR new group at age 7 years (aOR=3.036, 95% CI 1.295-7.118). *Dp*, *Df* sensitization were risk factors for BHR persistent group at age 7 years (aOR=2.383, 95% CI 1.120-5.071; aOR=3.084, 95% CI 1.524-6.239). *Df*was a additionally sensitized allergen as a risk factor for BHR new group at 11 years (aOR=3.267, 95% CI 1.388-7.692) and grass pollen for BHR persistent group at 11 years (aOR=6.441, 95% CI 1.239-33.472).

**Conclusion:** Children with BHR remission were associated with family history of asthma and low sensitization at age 7 years. Children with BHR new showed high sensitization and normal lung function, but low eosinophil at age 7 yrs. Children with BHR persistent were associated with high atopic condition including high eosinophil, high IgE, high sensitization, and low lung function at age 7 yrs. These findings suggest that the natural course of childhood BHR has different phenotypes and we can predict the future prognosis of BHR by these phenotypes.

## A347 Spectrum of Allergens Causing Allergic Rhinitis and Asthma in Urban Bangalore, India − a Study of 120 Patients

### Jacqueline Elizabeth Joseph^1^, M. S. Soumya^2^, Ruby Pawankar^3^, Harshitha Kumar^1^

#### ^1^St. John’s Research Institute India; ^2^St. John’s Medical College Hospital; ^3^Nippon Medical School Japan

##### **Correspondence:** Jacqueline Elizabeth Joseph - St. John’s Research Institute, India

**Background:** Allergic rhinitis is usually thought to be a minor irritating disease, but it can cause significant morbidity. Rhinitis is characterized by chronic or recurrent sneezing or by runny or blocked nose. Pollen, fungi, animal dander, house dust mites, domestic pets, and insects are of particular importance as triggering factors.

**Objectives:** This study was designed to determine whether the sensitivity of a spectrum of allergens using skin-prick test correlates with symptom severity in allergic rhinitis and asthma patients.

**Material and method:** A detailed history taking and clinical examination was carried out for each patient (i.e., for those satisfying the inclusion criteria), which includes a diagnostic nasal smear and skin prick testing (SPT). The allergens selected for SPT was based on the reference pollen calendar of Bangalore city created by us and clues from the patient’s exposure to the probable allergens in his surroundings.

**Result:** We studied a total of 120 patients. The overall rate of sensitisation to any allergen was 96 %. The most common allergen was House Dust Mite (HDM 35%), while the most prevalent House Dust Mite was found to be Mite D-Pteronyssinus. Sensitivity towards pollens and fungal spores was 16% respectively. 57.5% of the Allergic Rhinitis patients had Persistent Allergic Rhinitis, out of which 78% were Moderate-Severe grade and 38.3% had Intermittent Allergic Rhinitis, of which 73% were Moderate-Severe.

**Conclusion:** In light of the findings of the present study, it can be concluded that appropriate preventive strategies can decrease the cost and morbidity of therapeutic measures. The representation of the SPT reactivity to the House Dust Mite allergen may be a useful reference to counsel patients with allergic rhinitis.

## A348 High Prevalence of Wheezing Illness and Risk Factor of Atopic Asthma Progression in Korean Preschool Children

### Sohyoung Yang^1^, Sung-Il Woo^2^

#### ^1^Chungbuk National University Hospital South Korea; ^2^Pediatic SIG, Korea Institute of Drug Safety and Risk Management

##### **Correspondence:** Sohyoung Yang - Chungbuk National University Hospital, South Korea

**Backgroud and objective:** Asthma is most common chronic disease in childhood and is well known association between risk factors and asthma progression in children with wheeze. The aim of the present study was to investigate the prevalence of wheeze and risk factors predicting asthma in young children.

**Method:** The Green Breath for Children (GBC), a Korean children in Chungbuk province, have recruited 3194 preschool children ≥ 2 yrs of age, annually since 2011. Physical examinations, questionnaire for allergic and respiratory disease and skin prick test were performed.

**Results:** Among these children, 2745 (85.9%) has completed response to questionnaire. Complete data were available for 2453 (76.7%) children about wheeze, medical history and skin prick test. The prevalence of wheeze was 22.7%. It was found that incidence of current wheezing illness within one year was declined and incidence of aeroallergen sensitization was increased with age (from 21.5% in 2 year old to 39.0% in 6 years old). Two hundred and sixty nine children had wheeze episode ≥ 3 among children > 3 year old (n=2253). Among these children, 175 (65.1%) has current wheeze illness within 1 year. Sensitization with A/H ratio grade ≥6+, allergic rhinitis, parental asthma was significantly higher in children with current wheeze illness. Diagnosis of allergic rhinitis and parent with asthma were also significantly associated in logistic regression.

**Conclusion:** There were high prevalence of wheeze and presence of risk factor of asthma progression, especially higher sensitization rate and atopic dermatitis in preschool children. So it is needed that quantitative measurement of atopic status or biologic marker monitoring for discriminating children who will have asthma progression among preschool children with wheeze.

## A349 Clinical and Laboratory Screening of Primary Immunodeficiency Diseases: International Effects

### Nima Rezaei

#### Tehran University of Medical Sciences, Iran

Primary Immunodeficiency Diseases (PIDs) are a heterogeneous group of inherited disorders, characterized by defects in one or more components of the immune system, leading to a variety of clinical manifestations, particularly recurrent severe infections, autoimmunity, lymphoproliferation, and malignancies.

PIDs usually present with one of the following eight characteristic clinical presentations: Recurrent upper or lower respiratory tract infections, Failure to thrive (FTT) from early infancy, Recurrent pyogenic infections, Unusual severe infections, Recurrent infections with the same type of pathogen, Autoimmune or chronic inflammatory disease and/or lymphoproliferation, Characteristic combinations of clinical features in eponymous syndromes, and a number of characteristic presentation such as angioedema.

The first step in the diagnostic process starts from clinical screening, while suspicious to a number of certain PIDs could be made, according to their clinical phenotypes. Using a limited set of tests which is available in most hospital, including complete blood count (and differential), a first screen for PIDs can be reliably performed; meanwhile screening laboratory tests for each category of defects in the immune system is needed, considering the characteristic clinical presentations; e.g., immunoglobulin assays for antibody deficiency or CH50 and AP(AH)50 assays for complement deficiency in those with recurrent sinopulmonary infections with encapsulated organisms; or B- and T- lymphocyte subsets enumeration for combined immunodeficiency in those with FTT or early onset severe infections; or chemotaxis, nitroblue tetrazolium (NBT) dye reduction test, dihydrorhodamine (DHR) oxidation test for phagocyte defects in those with recurrent pyogenic infections.

It should be noted that more elaborate tests, including specific antibody responses to protein or polysaccharide antigens, lymphocyte proliferation tests, advanced immunophenotyping, random migration, phagocytosis, and intracellular microbial killing by phagocytes, and a chemiluminescence assay can be performed in immunological laboratories. Meanwhile the definite diagnosis of PIDs relies on genetic tests.

## A350 The Effect of *Helicobacter Pylori* Infection in Atopic Individuals

### Sukran Kose^1^, Basak Gol Serin^2^, Arzu Didem Yalcin^3^, Süheyla Serin Senger^1^, Mehmet Erden^1^, Ertan Serin^1^

#### ^1^Tepecik Training and Research Hospital, Izmir, Turkey; ^2^Tepecik Training and Research Hospital, Allergy and Clinical Immunology, Izmir, Turkey; ^3^Near East University, Nicosia, Cyprus

##### **Correspondence:** Sukran Kose – Tepecik Training and Research Hospital, Izmir, Turkey

The Effect of *Helicobacter Pylori* Infection in Atopic Individuals

**Background:** The role *Helicobacter pylori (H. pylori)* infection in the aetiology of atopy remains unclear, although a possible protective role has been hypothesized.

**Objective:** The aim of this study was to evaluate the prevalance of *H. pylori* in the atopic individuals.

**Methods:** We conducted a retrospective, observational and cross-sectional study which included 104 patients, aged between 18 and 70 years. Total serum IgE (Immage 800, Beckman Coulter, Ireland) and *H.pylori IgG* (DIA.PRO, Italy) were measured in all participants by using nephelometric method and ELISA respectively.

**Results:** One hundred four patients included in the study. The avarage age was 38, and 74 of the patients were female (71.2%). The average IgE was measured to be 165 IU/mL. Fifty-two (50%) patients were diagnosed with an allergic disease according to anamnesis, laboratory results, and skin prick test. *H. pylori* infection was found in 61.5% of patients with allergic diseases. *H.pylori* was more frequent in the patients with allergy, unless that difference is not statistically significant (p:0.685, chi –square:0.165)

**Conclusion:** The prevalence of allergic disorders, including asthma, atopic dermatitis,urticare, and allergic rhinitis has been increasing, and the prevalence of *H. pylori* infection has been decreasing. In the previous studies, an inverse association has been observed between H. pylori infection and many allergic diseases such as recent wheezing, allergic rhinitis, dermatitis, eczema or rash. In our study, *H.pylori* was found more frequently unless that difference is not statistically significant (p:0.685, chi –square:0.165).

## A351 Clinical Spectrum and Natural History of Chronic Urticaria in Hong Kong Children

### Ting Fan Leung^1^, Agnes Sze-Yin Leung^2^

#### ^1^Prince of Wales Hospital, Hong Kong, Hong Kong; ^2^The Chinese University of Hong Kong, Hong Kong, Hong Kong

##### **Correspondence:** Agnes Sze-Yin Leung – The Chinese University of Hong Kong, Hong Kong, Hong Kong

**Background:** Chronic urticaria (CU) lasting for 6 weeks or longer can be classified into chronic spontaneous urticaria (CSU), urticarial vasculitis, and inducible (physical) urticaria. A cause for CSU is not identified in approximately 60% of patients. Thirty to 50 percent of adults with CU achieved remission 1-3 years after onset. This study investigated the clinical spectrum and natural course of CU in Chinese children and identified possible predictors of disease remission.

**Methods:** This single-centre retrospective study identified 96 patients with CSU below 18 years of age who were followed in our allergy clinic for ≥ 6 months. Disease-related factors such as occurrence of urticaria, angioedema and anaphylaxis as well as familial history, environmental exposures, co-morbid allergies, immunological investigations and drug treatments were retrieved from medical records. Patients were considered to be in remission when they were symptom-free for ≥ 3 months. Natural history of CU was delineated by Kaplan-Meier analysis, and factors associated with disease remission were analysed by log-rank statistics.

**Results:** The mean (SD) age of patients at baseline was 9.0 (5.2) years, and 53 (55%) of them were male. They were followed for a median of 4.0 years. Coexisting asthma, rhinitis and eczema affected 47%, 51% and 24% of these patients. Sixty-seven percent (53/79) of patients were atopic. Forty-seven (49%) patients had urticarial episodes at least once weekly, and 33 patients had both urticaria and angioedema. Both patients who developed anaphylaxis (one respiratory and one cardiorespiratory) had persistent disease. Seventy-nine patients had concomitant inducible urticaria. Laboratory investigations revealed positive anti-nuclear antibody in 26% (12/47; none with anti-thyroid antibodies), circulating eosinophilia in 24% (14/59), increased serum total IgE in 68% (40/59) and low plasma C3 and/or C4 levels in 30% (16/53). Fifty-six patients were treated with non-sedating antihistamines alone and 15 had combined non-sedating antihistamines and H2 antagonists. Sixty (63%) patients were in remission at a median of 2.4 years from disease onset. None of the clinical and laboratory parameters was associated with disease remission.

**Conclusions:** Childhood CU has in general favourable prognosis, and two-thirds of them achieve disease remission. This study cannot identify any clinical or laboratory factor for the resolution of CU.

**Funding:** Direct Grant for Research (2013.2.033), CUHK

## A352 Skin Prick Test Reactivity to Common Pollen Aeroallergens in Patients with Allergic Rhinitis − in Urban Bangalore, India

### Harshitha Kumar^1^, Soumya M.S.^2^, Jacqueline Elizabeth Joseph^1^, Ruby Pawankar^3^

#### ^1^St. John’s Research Institute, Bangalore, India; ^2^St. John’s Medical College Hospital, Bangalore, India; ^3^Nippon Medical School, Tokyo, Japan

##### **Correspondence:** Harshitha Kumar – St. John’s Research Institute, Bangalore, India

**Background:** Allergic rhinitis (AR) is the most prevalent of all allergic diseases. Aeroallergens play a major role in the pathogenesis of respiratory allergic diseases, like AR and asthma. Pollen, fungi, animal dander, house dust mites, domestic pets, and insects are of particular importance as common triggers.

**Objectives:** The main objective of this study was to assess the sensitivity to common pollen allergens in patients with Allergic Rhinitis (AR) patients visiting the ENT Allergy clinic in a tertiary care hospital in urban Bangalore.

**Material and method:** A detailed history of the symptoms of AR and clinical examination was carried out for each patient. The ARIA classification was used for elucidating the severity of AR. We also performed a nasal smear test for eosinophilia and skin prick testing.

**Result:** Out of the 100 patients with AR, 59% of the patients had persistent AR, out of which 46% were of moderate-severe persistent AR. The overall rate of sensitisation to any allergen was 95.2 %. The most prevalent aeroallergen sensitization was found to be *Parthenium hysterophorus* (33%), *Amaranthus spinosus* (23%), Eucalyptus (21%), *Cynodan dactylon* (20%), followed by *Casuarina equisetifolia* (19%).

**Conclusion:** Bangalore has a high prevalence of AR. The successful treatment of this condition needs appropriate diagnosis and therefore a better understanding of the aeroallergen spectrum and sensitivity patterns.

## A353 Seasonal Patterns of Asthma-Related ED Visits and Admissions in Children and Adolescents Who Visited Emergency Rooms of Korea in 2007-2012

### Eun Hee Chung

#### National Medical Center, South Korea

**Background:** Seasonal variation of asthma-related hospitalizations has long been recognized, however, little is known about asthma-related ICU admissions. To identify seasonal trend of asthma-related hospitalizations and ICU admissions may allow preventive strategies to be developed.

**Methods:** We analyzed the National Emergency Department Information System (NEDIS) records of 117 emergency room in Korea of all patients aged between 3 and 18 years with asthma during six years (from 2007 to 2012). Data was tabulated and graphed to show seasonal trends in the monthly number of ED visits, general ward(GW), and ICU admissions for asthma.

**Results:** A total of 41,128 subject were found and the male to female ratio was 1:0.5. GW admissions as a percent of ED visits were 42.6% (n=17,524), and ICU admissions, 0.8% (n=335). Monthly number of ED visits and GW admissions for asthma showed the seasonal variability with high peaks in fall (September to November) and low rates in summer (June to August). ICU admissions, however, showed different peaks at each year. Despite this finding, ICU admissions were at a minimum in fall as a percent of general ward admissions.

**Conclusions:** There were important differences in the seasonal pattern of ED visits, general ward(GW), and ICU admissions for asthma. The combined analysis of these three data sets provides a new perspective on the epidemiology of asthma.

## A354 Prevalence of Atopic Dermatitis and Its Associated Risk Factors in Elementary School Children: A Cross-Sectional Study in Gyeonggi-Do, South Korea

### Eunji Kim^1^, Young Yoo^1^, Young Yoo^2^, Ji Tae Choung^1^, Ji Tae Choung^2^, Sungchul Seo^2^, Sungchul Seo^1^, In Soon Kang^2^, Jue Seong Lee^1^, Ji Hyen Hwang^1^

#### ^1^Korea University South Korea; ^2^Korea University Anam Hospital

##### **Correspondence:** Eunji Kim – Korea University, South Korea

**Background:** Aims of this study were to investigate prevalence and severity of atopic dermatitis (AD) and to analyze its associated risk factors in a total of 2,109 children (1,040 boys, 1,069 girls) from 5 elementary schools within Gyeonggi-do, South Korea.

**Methods:** We conducted questionnaire survey using a Korean version of International Study of Asthma and Allergies in Childhood (ISAAC) questionnaire and anthropometric evaluation from October to November in 2012. AD was defined by existence of chronic eczema over 6 months based on the ISAAC questionnaire. SCORAD (SCORing Atopic Dermatitis) index were evaluated for 227 children with AD. Children were divided into 2 groups according to the SCORAD index : 1) mild-to-moderate AD (SCORAD index < 40); 2) severe AD (SCORAD index ≥ 40). Skin prick test to 18 allergens and blood test were completed for 188 children with AD.

**Results:** Among 2,109 children, 543 children (25.0%) were designated as having AD. One hundred and ninety children had mild-to-moderate AD (83.7%) and 37 children had severe AD (16.3%). Prevalence of obesity (BMI ≥ 95p) in AD group (n=38, 8.0%) was significantly higher than that of non-AD group (n=62, 4.5%). Proportion of children experienced breast milk feeding over 6 months in AD group (n=250, 46.7%) was significantly higher than that of non-AD group (n=635, 41.3%). There were no significant differences between AD group and non-AD group in terms of sex, age, BMI, history of breast milk feeding ever and mode of delivery. Geometric means (range of 1 SD) of blood eosinophil percentage (5.78% [3.35-10.00]) and serum eosinophil cationic protein concentration (48.98 μg/L [43.84-2,189.52]) in severe AD group were significantly higher than those of mild-to-moderate AD group (3.64% [1.67-7.96], 33.28 μg/L [12.02-92.20]). There were no significant differences between mild-to-moderate AD group and severe AD group in terms of age, BMI, birth weight, obesity, mode of delivery, history of breast milk feeding ever, serum hemoglobin and total IgE concentration. Risk factors for having AD were male sex (aOR 1.25 [1.01-1.55]), obesity (aOR 1.08 [1.17-2.76]) and history of breast milk feeding over 6 months (aOR 1.25 [1.01-1.55]). Atopy in skin prick test to 18 allergens was observed in 70% of AD group children. Majority (57.8%) of children were reactive to house dust mites, followed by pollen (29.9%), animal dander (26.2%), mold (18.7%) and food allergen (9.1%).

**Conclusions:** Prevalence of AD is 25.9% in the Gyeonggi-do, South Korea and it is similar to the prevalence of recent Korean nation-wide study in 2010 (27.0%). Male sex, obesity and history of breast milk feeding over 6 months independently increase risk of having AD.

## A355 Intralymphatic Immunotherapy for *Dermatophagoides Farinae*, *Dermatophagoides Pteronyssinus*, Cat, and/or Dog Allergy in Patients with Allergic Rhinitis: 1 Year Follow-up

### Sang Min Lee, Joo Hyun Jung, Seung Joon Choi, Eugene Joe, Hyunjung Hwang, Shin Myung Kang, Yu Jin Kim, Sun Young Kyung, Jeong-Woong Park, Sung Hwan Jeong, Sang Pyo Lee

#### Gachon University Gil Medical Center South Korea

##### **Correspondence:** Sang Min Lee – Gachon University Gil Medical Center South Korea

**Background:** Recently, several clinical trials reported that intralymphatic immunotherapy (ILIT) for some allergens including cat dander and birch or grass pollen induces tolerance faster than conventional subcutaneous immunotherapy with comparable duration of effect after only 3 injections, but without serious local or systemic reaction. However, the efficacy and safety of ILIT for various allergens in allergic rhinitis still remains to be investigated. We evaluated the efficacy and adverse effect of ILIT for house dust mite, cat, and dog allergy in patients with allergic rhinitis.

**Methods:** A total of 9 subjects with allergic rhinitis sensitized to *Dermatophagoides farinae*, *Dermatophagoides pteronyssinus*, cat, and/or dog allergen were treated with 3 intralymphatic inguinal injections of causal allergen extract (HollisterStier, New Orleans, USA). Rhinoconjunctivitis quality of life questionnaire (RQLQ), sino-nasal outcome test 20 (SNOT-20), and rhinitis symptoms during exposure to causal allergen were evaluated before, 4 and 12 months after 1^st^ injection of ILIT.

**Results:** RQLQ and SNOT-20 were significantly improved 4 months after ILIT from 71.2 (range 50-105) and 34.3 (range 3-70) to 52.3 (range 37-89) and 22.7 (range 8-52), respectively (*P* < 0.05). Allergy symptom during exposure to causal allergen including rhinorrhea, sneezing, nasal obstruction, and itching sensation on eye, nose, and contacted skin were also significantly reduced (*P* < 0.05, respectively). In five subjects who visited hospital 12 months after 1^st^ injection of ILIT, rhinorrhea, sneezing, and nasal obstruction during exposure to causal allergen remained to be alleviated also (*P* < 0.05). We observed two cases of anaphylaxis and one case of severe cutaneous erythema and edema at injection site after ILIT.

**Conclusions:** ILIT can rapidly improve allergy symptoms in daily life, especially those provoked by allergen exposure, and this effect lasts for a year. However ILIT can also cause severe systemic or local hypersensitivity reaction.

**Acknowledgement:** This work was supported by the Gachon University Gil Medical Center (Grant number: 2013-11). We thank to ThermoFisher Scientific Korea for support in measuring serum total and allergen-specific IgE/IgG_4_ (ImmunoCAP®). For providing allergen extracts which were used for NPT, we also appreciate Research Center for Standardization of Allergic Diseases (RCSAD) of Yonsei University supported by a grant from the Korea Healthcare Technology R&D Project, Ministry of Health, Welfare & Family Affairs, Republic of Korea (A092076).

## A356 Respiratory Syncytial Virus Regulates IL-33 Expression in Bronchoalveolar Cells and Lung Tissue in Vivo

### Alina Gaisina^1^, Igor Shilovskiy^1^, Aleksandra Nikonova^2^, Oleg Kamyshnikov^1^, Musa Khaitov^1^, Alexander Mitin^1^, Komogorova Viktoriya^1^, Marina Litvina^1^, Nina Sharova^1^

#### ^1^National Research Center - Institute of Immunology Russia; ^2^Metchnikov’s Research Institute for Vaccines and Sera

##### **Correspondence:** Alina Gaisina – National Research Center - Institute of Immunology, Russia

**Background:** Recent studies in humans have shown that IL-33 production is induced in lungs by rhinovirus infection [Jackson, 2014]. However, there are no available data characterizing IL-33 expression after infection with other respiratory viruses and on cell types producing this cytokine. The aim of this study was to evaluate the effect of respiratory syncytial virus (RSV) infection on the IL-33 expression *in vivo*.

**Methods:** Female BALB/c mice, aged 8 weeks, were divided into 3 groups. The first group was intranasally (i.n.) infected with 50 μl/mouse RSV strain A2 (5×10^5^ TCID_50_/mouse). The second group received UV-inactivated RSV. The third group was treated with PBS only. On day 6 after infection, airway hyperresponsiveness (AHR) to methacholine was measured by whole-body plethysmography. The left lung was removed for histological analysis. One lobe of the right lung was taken for viral RNA (vRNA), mRNA-IL-33 evaluation by qPCR and the other lobes was used for preparing of cell suspension by collagenase digestion. Cell suspension was stained with fluorophore labeled antibodies to determine IL-33 intracellular expression in CD45^+^3^+^ T cells, CD45^+^19^+^ B cells, CD45^+^CD3^-^CD19^-^Ly-6G^-^ cells, CD45^-^324^+^ epithelial cells by flow cytometry.

**Results:** vRNA copy number in lung tissue of RSV-infected animals was 4.3-fold higher than in mice treated with inactivated virus. AHR to inhaled methacholine in RSV-infected animals was increased compared to mice treated with inactivated virus or PBS. Histological analysis revealed the presence of inflammation characterized by infiltration of lymphocytes into the lung tissue of RSV-infected animals. These data indicate RSV infection in the mouse lungs. mRNA-IL-33 expression in lungs was 2.5-fold up-regulated upon RSV infection. Flow cytometry analysis revealed 1.7- and 1.5-fold increase in the percent of IL-33^+^ T-cells and CD45^+^CD3^-^CD19^-^Ly-6G^-^IL-33^+^ cells, respectively. We found that RSV increased (1.5 fold) the mean fluorescence intensity of IL-33 on B-cell population. In addition, IL-33 intracellular protein expression was slightly increased in epithelial cells (1.2-fold).

**Conclusion:** Our results provide evidence for up-regulation of IL-33 in T-, B- and CD45^+^CD3^-^CD19^-^Ly-6G^-^cells in RSV-infected murine lungs that may indicate an important role of IL-33 in virus-induced lung infections. The study was supported by RSF Grant 14-15-00894.

## A357 The Prevalence of Parent-Perceived Food Hypersensitivity in Pre-School Children Attending a Tertiary Care Hospital in Malaysia

### Faiah MJ^1^, Intan H Ismail^2^, E A Miles^3^, Faizah Mohamed Jamli^4^

#### ^1^Department of Paediatrics, Hospital Serdang, Jalan Puchong, Kajang 43000 Malaysia; ^2^Department of Paediatrics, Faculty of Medicine and Health Sciences, Universiti Putra Malaysia, 43400 Serdang, Malaysia; ^3^Human Development and Health Unit, Faculty of Medicine, University of Southampton, SO166YD United Kingdom; ^4^Hospital Serdang Malaysia

##### **Correspondence:** Faizah Mohamed Jamli – Hospital Serdang, Malaysia

**Background:** The prevalence of food allergy has increased world-wide. In Malaysia, food allergy prevalence has never been studied. Prevalence data is important to assess the burden of food allergy in order to establish the requirement of allergy services.

**Objectives:** We aimed to determine the prevalence of parent-perceived food hypersensitivities in Malaysian paediatric population and evaluate the spectrum of clinical manifestations and allergens involved.

**Methods:** We conducted a cross-sectional parent-questionnaire survey among preschool children attended the outpatient general paediatric clinic. We evaluated the associated factors for hypersensitivity reactions to food.

**Results:** A total of 333 children were included in the study. Eighty (24.0%) parents reported that their children have ever had hypersensitivity reactions to food. The major food allergens were shellfish (45%), egg (36.2%), cow’s milk and dairy products (28.8%) and peanut (27.5%). Reactions to multiple foods were reported in 57.7% children. The most commonly reported symptoms were hives and itchiness (46.3%), eczematous skin rash (45%) and chest tightness and wheeze (31.3%). Significant factors associated with parent-reported food hypersensitivity were history of eczema (OR, 7.3: 95% CI 3.15-17.08), and allergic conjunctivitis (OR, 6.2: 95% CI 1.82- 20.57) in the children and siblings with food allergy (OR, 3.72: 95% CI 1.22-11.37).

**Conclusion:** Parent perception of food hypersensitivity reactions is common in pre-school children. Further studies with a larger sample size and longer duration are required to determine the prevalence of food allergy in Malaysia.

## A358 Th2 Dominant Airway Inflammation Induced By House Dust Mite Chitin Is Dependent on TNF-a and NKT Cell

### Jun-Pyo Choi^1^, Han-Byul Choi^2^, Yoon-Keun Kim^1^, Hyeon-Il Choi^1^, Da-Il Yoon^3^

#### ^1^Ewha Institute of Convergence Medicine, Ewha Womans University Medical Center; ^2^Ewha Womans University Mokdong Hospital; ^3^Ewha Womans University Mokdong Hospital South Korea

##### **Correspondence:** Da-Il Yoon – Ewha Womans University Mokdong Hospital, South Korea

**Background:** Chitin is polymer of N-acetyl-b-D-glucosamin and founded in various organism such as house dust mite. Generally, chitin is applied in medical material, because it is known to not induce the immune response. However, according to recent reports, chitin induced innate & adaptive immune response. In this point, we can postulate that HDM chitin induce the immune response, but exact effect & mechanism is unknown.

**Objective:** To evaluate the immunological side in the development of airway inflammation induced by sensitization with allergens plus house dust mite derived chitin.

**Method:** To induce the airway inflammation by HDM derived chitin, 6 weeks-old mice were administrated intranasally four times with 75 μg of ovalbomin (OVA) and 100 μg of HDM chitin, and then challenged intranasally 4 times with 50 μg of OVA on days 14, 15, 21, and 22. Lung inflammation and immunologic parameters were evaluated 48 h after the final allergen challenge and 6 h after allergen challenge on day 21, respectively.

**Result:** Intranasal administration of HDM (house dust mite) induced Th2 dominant, but mixed, airway inflammation and chitinase treatment induced down-regulation of Th2 immune response. Refined HDM chitin with allergen sensitization up-regulated the production of Th2 cytokine, dominantly, and chitinase treatment showed similar manner of HDM treatment results. This immune responses are mediated through TLR2, which is known to receptor of chitin recognition, and macrophage derived TNF-a and NKT cell.

**Conclusion:** These findings indicate that airway inflammation sensitized by HDM derived chitin induces Th2 dominant immune response, which is mainly dependent on TNF-a produced by macrophage cell and NKT cell.

## A359 Geographic Variations in the Patterns of Sensitization to Aeroallergens in Korean Adults: A Multi-Center Study

### Mingyu Kang^1,6^, Mi Yeoung Kim^2^, Sujeong Kim^3^, Eun-Jung Jo^4^, Seoung-Eun Lee^5^, Woo-Jung Song^6^, Sang Min Lee^7^, Chansun Park^8^, Yoon-Seok Chang^9^, Jaechun Lee^10^, Young-Koo Jee^11^, Inseon S Choi^12^, Kyung-up Min^6^, Sang-Heon Cho^6^, Sang-Heon Cho^13^

#### ^1^Chungbuk National University Hospital South Korea; ^2^Hospital; ^3^Kyungpook National University School of Medicine; ^4^Pusan National University Hospital; ^5^Yangsan Pusan National University Hospital; ^6^Seoul National University Hospital; ^7^Gachon University Gil Medical Center; ^8^Haeundae Paik Hospital, Inje University College of Medicine; ^9^Seoul National University Bundang Hospital; ^10^Jeju National University School of Medicine; ^11^Dankook University College of Medicine; ^12^Chonnam National University Medical School; ^13^Seoul National University College of Medicine

##### **Correspondence:** Mingyu Kang – Seoul National University Hospital, Chungbuk National University Hospital South Korea

**Background:** Inhalant allergen sensitization is a major risk factor for allergic disease, which is largely influenced by living environments. Despite substantial geographic variations in allergen sensitization in the literature, comprehensive studies are still lacking in Korean adults.

**Objective:** We aimed to investigate recent patterns of inhalant allergen sensitization among Korean adult patients with suspected history of respiratory allergy, and also examine the geographic variations of the sensitization profiles in Korea.

**Methods:** From 2009 to 2014, a total of 34,289 patient records were retrieved for a retrospective analysis, from 12 referral allergist clinics in 9 different regions. Inclusion criteria were Korean adults (≥18 years old) who underwent inhalant allergen skin prick test for suspected history of respiratory allergy. Primary outcome was the detailed profile of inhalant allergen sensitization. Sensitization to allergens was defined by allergen-to-histamine wheal ratio ≥ 1. Demographic and clinical information, and residential area of participants were also collected. Regional sensitization profiles of individual allergens were calculated after adjusting age and sex. We meta-analyzed the regional sensitization profiles, and then estimated both overall atopy and individual allergen sensitization profiles in general. Geographic variations of sensitization between allergen groups were statistically compared by using Cochrane Q and I^2^-statistics.

**Results:** Overall prevalence of atopy was 44.8% (95% CI [38.5-47.8]). In overall, Der F and Der P were the most commonly sensitized allergens (29.5% and 28.7%, respectively), and followed by cat (8.0) and birch (8.0%), hazel (7.4%), alder (7.2%), mugwort (7.0%), beech (6.7%), oak (6.6%) and Tyrophagus putrescentiae (5.8%). The ten common inhalant allergens were similar between regions. However, in Jeju, 6 among 10 common allergen were different from other regions. Sensitization to Japanese cedar (12.4%), rye (8.7%), velvet (8.3%), Kentucky (8.1%), timothy (7.5%) and vernal grass (7.4%) were more prevalent in Jeju. According to allergen groups, geographic heterogeneity were highest in outdoor molds and cockroaches. Sensitization to animals, weeds and mites showed less dependent to locations. Sensitization to pollen from early- and mid-blooming trees were significantly high in Gangwon, Gyeongbuk and Busan.

**Conclusion:** As overall, common inhalant allergens were Der P, Der F, cat, birch, hazel, alder, mugwort, beech, oak and Tyrophagus putrescentiae. Sensitization to inhalant allergens showed geographic variations, particularly in Jeju. This study was the largest scale conducted, so far, on the aeroallergen sensitizations in Korean adults. We hope our findings could contribute to the establishment of skin prick test panels for use in clinical practice and epidemiological surveys.

## A360 Experimental Mouse Model of Asthma Induced By Dust Mite *Dermatophagoides Pteronyssinus a*llergenic Extract

### Anton Laskin^1^, Oleg Kamyshnikov^1^, Alexander Babakhin^1^, Valentina Berzhets^2^, Musa Khaitov^1^

#### ^1^National Research Center - Institute of Immunology of Federal Medico-Biology Agency of Russia Russia; ^2^Metchnikov’s Research Institute for Vaccines and Sera, Russian Academy of Medical Scienses

##### **Correspondence:** Anton Laskin – National Research Center - Institute of Immunology of Federal Medico-Biology Agency of Russia, Russia

**Background:** The purpose of this study was to develop a mouse model of asthma (MMA) using house dust mite *Dermatophagoides pteronyssinus* (*Der* p) extract.

**Methods:** BALB/c mice were i.p. immunized with different doses of *Der p* lyophilized extract three times in three week interval in the mixture with Al(OH)_3_. 8 weeks after the final immunization mice were challenged with *Der p* during five consecutive days by intranasal applications (INA) or aerosol administration (AA). All mice were divided into 5 experimental groups: group 1 was immunized with 50 μg/mouse of *Der p* (in protein equivalent) in the mixture with 2 mg/mouse Al(OH)_3_ and challenged by INA; group 2 was immunized in the same way and challenged by AA; group 3 was immunized with 100 μg/mouse *Der p* in the mixture with 2 mg/mouse of Al(OH)_3_ and challenged by INA; group 4 was immunized in the same way and challenged by AA; group 5 (negative control) was immunized and challenged with saline. 24 hours after the last challenge airway hyperresponsiveness (AHR) to different concentrations of methacholine was evaluated in all groups by whole-body plethysmography. 48 hours after the last challenge in all groups blood was collected for differential cell count, brochoalveolar lavage fluid (BALF) was sampled for the determination of inflammatory cells and lungs were removed for histological analysis. Histopathological changes were graded according to semi-quantitative scoring system. Serum anti-*Der p* IgE, IgG1 and IgG2a antibodies were detected by ELISA seven days after the last immunization and 48 hours after the challenge.

**Results:** The highest level of serum *Der p*-specific IgE antibodies was observed in group 2. The levels of *Der p*-specific serum IgG1 and IgG2a antibodies in group 2 were significantly higher than that of other experimental groups. The maximum of AHR was observed in groups 1 and 3 challenged by INA. Analysis of cell composition in BALF demonstrated elevated number of basophils in group 3 in comparison with other experimental groups. No significant differences in peripheral blood cell counts were observed among experimental groups. Histological picture of allergic inflammation in lungs (peribronchial and perivascular infiltration with inflammatory cells) was the most expressive (according to score system) in group 3 in comparison with other experimental groups.

**Conclusion:** Data obtained indicate that sensitization with *Der p* in a dose 100 μg/mouse together with Al(OH)_3_ and challenge with *Der p* by INA is a suitable approach for modeling of mouse allergic asthma.

## A361 Severe Refractory Pulmonary Complications in Children with *Mycoplasma Pneumoniae* Pneumonia

### Jae Ho Lee^1^, Sejin An^2^

#### ^1^Chungnam National University School of Medicine; ^2^Chungnam National University Hospital South Korea

##### **Correspondence:** Sejin An – Chungnam National University Hospital, South Korea

**Background:***M. pneumoniae* is one of most common causes of community-acquired pneumonia in children. In the clinical courses, small portions are serious. The mechanisms of severe pulmonary complication of *M. pneumonia* pneumonia are not clear, but exaggerated immune reactions may play a major role in the destruction of lung tissues.

**Methods:** Two patients had severe respiratory symptoms and signs with high fever. The blood test, inflammatory reactant test and serologic test were performed. The mycoplasma pneumonia was confirmed by positive IgM and also rising titers of IgG antibody of *M. pneumoniae* or cold agglutinin more than four times later. The chest radiograph and computed tomography (CT) scan were checked serially.

**Results:** In one case of necrotizing pneumonia, 3-year-boy had protracted clinical course of high fever and moderate respiratory distress despite of the appropriate antibiotic therapy. A chest CT scan revealed profound lung tissue destruction in the right middle lobe. After 2 month of intensive antibiotic therapy, he was finally recovered completely without sequela. In case of bronchiolitis obliterance, 2-year-boy showed a patch infiltration in the right middle lobe. The mycoplasma pneumonia was effectively treated with appropriate antibiotics. After 3 month of discharge, the chest CT scan showed the segmental consolidation of the right middle lobe with bronchial wall thickening and hyper-lucency. He had recurrent pneumonia clinically and the right lung lesions were progress to be collapsed totally, called as destroyed lung till 3 years of infection. After 10 year of follow up, the chest CT scan was stationary without clinical problems.

**Conclusions:** We reported two cases of necrotizing pneumonia and bronchiolitis obliterance followed by destroyed lung after m*ycoplasma pneumoniae* pneumonia.

## A362 Usefulness of Interactive e-Learning Education Program for Asthma Guideline

### Yoon-Seok Chang^1^, Kyung-up Min^2^, Sang-Heon Cho^2^, Sae-Hoon Kim^1^, Yong Eun Kwon^3^, Young-Koo Jee^4^, Tae-Bum Kim^5^, Hee-Bom Moon^5^, Hye-Kyung Park^6^, Sung-Yoon Kang^2^

#### ^1^Seoul National University Bundang Hospital; ^2^Seoul National University Hospital South Korea; ^3^Chosun University College of Medicine; ^4^Dankook University College of Medicine; ^5^Asan Medical Center, University of Ulsan College of Medicine; ^6^Pusan National University Hospital

##### **Correspondence:** Sung-Yoon Kang – Seoul National University Hospital, South Korea

**Background:** Effective educational tools and implementation strategies are important for the dissemination of guideline. We developed the computer-based interactive education program of asthma guideline named as Virtual Learning Center for Asthma Management and evaluated its usefulness in terms of the improvement of awareness and user satisfaction.

**Methods:** 170 physicians were enrolled from six tertiary hospitals. They utilized the interactive education program for 2weeks for the learning of asthma management guideline. We compared awareness of asthma guideline before and after utilization of the program and investigated the user satisfaction with questionnaire survey.

**Results:** Mean age of study subjects was 28.2±3.1 and 78(49.4%) of subjects were male. The total score of awareness of asthma guideline was significantly improved from 80.3±6.3 to 85.1±6.9 (p<0.001). All categories of awareness including knowledge, attitude and practice were improved significantly after learning with the program (p<0.001). Satisfactions of users were high in the aspects of usefulness, convenience, interest, improvement of understanding of guideline and elevation of confidence. The most useful section of the program was virtual cyber management of asthma patient.

**Conclusion:** Interactive education program is a useful and effective tool for dissemination of asthma guideline.

## A363 Airway Inflammation Induced By House Dust Mite Derived Vesicles Is Mainly Induced By LPS Derived from Gram Negative Bacteria in Dust Mite

### Jun-Pyo Choi^1^, Han-Ki Park^1^, Ji-Hyun Lee^2^, Yoon-Keun Kim^1^, Sang-Yoon Kim^2^

#### ^1^Ewha Institute of Convergence Medicine, Ewha Womans University Medical Center; ^2^Ewha Womans University Mokdong Hospital South Korea

##### **Correspondence:** Sang-Yoon Kim – Ewha Womans University Mokdong Hospital, South Korea

**Background:** House dust mite (HDM) is well known organism as source of major allergen. Besides, HDM also possesses bacteria in their digestive system, so it is also founded feces of HDM. According to recent study, bacteria is able to secrete small circular ‘vesicles’, which is contain diverse molecules in its original bacteria. In addition, administration of these vesicles from specific bacteria induced airway inflammation and tissue destruction. In this point, we hypothesized that HDM derived vesicles play an important role on the development of airway inflammation.

**Objective:** To evaluate the role of HDM derived vesicles on the development of airway inflammation, and define the origin of them.

**Method:** To induce the airway inflammation by HDM, 6 weeks-old mice were administrated intranasally four times with 100 μg of HDM, and then challenged intranasally 4 times on days 14, 15, 21, and 22. Lung inflammation and immunologic parameters were evaluated 48 h after the final allergen challenge and 6 h after allergen challenge on day 21, respectively. In the case of HDM derived EV administration, 6 weeks-old mice were administrated intranasally four times with 75 μg of ovalbomin (OVA) and 10 μg of HDM EV, and then challenged intranasally 4 times with 50 μg of OVA on days 14, 15, 21, and 22. Especially, polymyxyn B treatment was done simultaneously during intranasal sensitization.

**Result:** HDM induced Th2 dominant mixed inflammation in the airway, and it contains bacteria. In addition, vesicles was also identified from HDM, and it is possible to induce immune responses in macrophage and epithelial cell. In the side of ability of inducing inflammation, HDM derived vesicles induced airway inflammation potentially compared than free LPS and soluble portion. This immune responses are mediated LPS recognition, and the source of it is gram negative bacteria in HDM.

**Conclusion:** These findings show that HDM derived vesicles induced airway inflammation potentially via recognition of LPS derived from gram negative bacteria in dust mite.

## A364 Changes in the Recognition of Causal Allergen, Its Avoidance, and Allergen Specific Immunotherapy after Skin Prick Test/Intradermal Test, Nasal Provocation Test, and Intralymphatic Immunotherapy in Patients with Allergic Rhinitis: 1 Year Follow-up

### Hyunjung Hwang, Eugene Joe, Sang Min Lee, Seung Joon Choi, Joo Hyun Jung, Yong Han Seon, Shin Myung Kang, Yu Jin Kim, Sun Young Kyung, Jeong-Woong Park, Sung Hwan Jeong, Sang Pyo Lee

#### Gachon University Gil Medical Center South Korea

##### **Correspondence:** Hyunjung Hwang – Gachon University Gil Medical Center South Korea

**Background:** Studies evaluating whether allergy patients change their recognition of causal allergen, its avoidance, and allergen specific immunotherapy (SIT) during diagnosis and treatment of their diseases are relatively rare. The objective of this study is to evaluate those changes after skin prick test / intradermal test (SPT/IDT), nasal provocation test (NPT), and allergen-specific intralymphatic immunotherapy (ILIT) for causal allergen among patients with allergic rhinitis.

**Method:** After informed consent, nine subjects with allergic rhinitis in whom allergens including *D. farinae*, *D. pteronyssinus*, cat hair, and dog hair/dander were proven to provoke their rhinitis symptoms by history taking, skin prick test, and measurement of serum specific IgE were asked to respond to the following questions: “Do you agree that allergen provokes allergic symptoms in daily life?”, “Do you agree that allergen avoidance can reduce allergic symptoms?”, and “Do you agree that allergen specific immunotherapy can reduce allergic symptoms?” Thereafter, they underwent SPT/IDT, NPT, and ILIT for their causal allergens. They were repeatedly asked to respond to those questions immediately after SPT/IDT, NPT, as well as 4 and 12 months after ILIT.

**Results:** The agreement (%) to “Allergen provokes allergic symptoms in daily life” changed from 67.7 ± 34.2 to 79.3 ± 25.7 (after SPT/IDT), 85.7 ± 13.4 (after NPT), 93.1 ± 11.7 (4 months after ILIT), and 90.6 ± 12.9 (12 months after ILIT). The agreement (%) to “Allergen avoidance can reduce allergic symptoms” changed from 67.7 ± 34.2 to 82.2 ± 20.6 (after SPT/IDT), 82.1 ± 27.8 (after NPT), 90.9 ± 12.6 (4 months ILIT), and 90.6 ± 12.9 (12 months after ILIT). The agreement (%) to “Allergen specific immunotherapy can reduce allergic symptoms” changed from 65.6 ± 29.3 to 81.5 ± 15.5 (after SPT/IDT), 85.7 ± 19.7 (after NPT), 86.3 ± 23.3 (4 months after ILIT), and 81.3 ± 11.6 12 months after ILIT).

**Conclusion:** Allergy skin test, nasal provocation, and SIT themselves can intensify patients’ recognition of causal allergen, its avoidance, and allergen-specific immunotherapy.

**Acknowledgement:** This work was supported by the Gachon University Gil Medical Center (Grant number: 2013-11). We thank to ThermoFisher Scientific Korea for support in measuring serum total and allergen-specific IgE/IgG_4_ (ImmunoCAP®). For providing allergen extracts which were used for NPT, we also appreciate Research Center for Standardization of Allergic Diseases (RCSAD) of Yonsei University supported by a grant from the Korea Healthcare Technology R&D Project, Ministry of Health, Welfare & Family Affairs, Republic of Korea (A092076).

## A365 Laboratory Diagnostic of Staphylococcal Sensitization

### Natalya Khramykhoverchenko

#### Semey State Medical University Kazakhstan

**Introduction:** For the diagnosis of staphylococcal sensitization widely used immunological reactions (reaction of leucocytolis – RLL, the reaction of the inhibition of leucocytes migration – RIML, the reaction of blast transformation of lymphocytes – RBTL etc.). However, these methods are time-consuming (RIML, RBTL) or insufficiently informative (RLL).

**Objective:** To develop a simple and reliable method for the diagnosis of staphylococcal sensitization in vitro.

**Material and меthods:** Total surveyed 37 patients with odontogenic and urological staphylococcal infection confirmed by bacteriological analysis. The control group consisted of 10 healthy persons who have not been identified on staphylococcal infection for 6 months. Diagnostics of staphylococcus sensitization was used by authorial methodology, getting the name M-ESR. The test of blood of inspected was mixed up with a staphylococcus antigen in the standard capillary of Panchenkov and incubation 18-24 hours at a room temperature. The increase of results of reaction on 12% and higher by comparison to control of reaction of M-ESR without an antigen, diagnosed bacterial sensitization.

**Results and discussion:** The results of M-ESR compared with the results RIML. Positive results of M-ESR and RIML detected in 81.1% and 78.4%, respectively. In the control group, positive results were observed in 20%.

**Conclusion:** Proposed a simple and reliable method of laboratory diagnostics can be used for the detection of sensitization to staphylococcal infection in oral and maxillofacial surgery, otolaryngology, urology and others.

## A366 Th-17 Regulatory Cytokines Enhance Neutrophil Production of IL-17 during Asthma

### Asma Sultana^1^, Pnhrc^2^, Rabih Halwani^3^, Ahmed Bahammam^4^, Saleh Al Muhsen^3^

#### ^1^Prince Naif Center for Immunology Research, College of Medicine, King Saud University; ^2^King Saud University; ^3^Prince Naif Center for Immunology Research and Asthma Research Chair, Department of Pediatrics, College of Medicine, King Saud University Saudi Arabia; ^4^University Sleep Disorders Center, College of Medicine, King Saud University

##### **Correspondence:** Saleh Al Muhsen – Prince Naif Center for Immunology Research and Asthma Research Chair, Department of Pediatrics, College of Medicine, King Saud University, Saudi Arabia

**Background:** Although IL-17-producing peripheral blood CD177+ neutrophils have recently been shown to increase in allergic asthmatic subjects, the mediators and mechanism regulating this increase in neutrophil derived IL-17 during asthma has not been properly investigated. IL-21, along with IL-6 and IL-23 cytokines, is important for the promotion of naïve CD4+ cells to differentiate toward the Th-17 cell lineage and the release of IL-17 cytokines. In this study, we explored the possibility that IL-21, IL-22 and IL-23 cytokines may activate peripheral blood neutrophils of asthmatic patients to release IL-17 cytokines and postulate that the response to stimulation could be different to that of neutrophils from non-asthmatic subjects.

**Methods:** Peripheral blood neutrophils isolated from asthmatic as well as healthy controls were stimulated, or not, with IL-21, IL-23, and IL-6 cytokines and levels of gene as well as protein expression of IL-17 cytokines were determined using RT-PCR and flow cytometry, respectively. In addition, to investigate the mechanism of IL-21, IL-23, and IL-6 induced IL-17 release, level of Stat3 phosphorylation in neutrophils was determined following stimulation with these cytokines.

**Results:** IL-21, IL-23, and IL-6 induced the production of IL-17 cytokines within peripheral blood neutrophils. Interestingly, the level of induced IL-17 cytokine were significantly higher in asthmatic compared to healthy control neutrophils. Stat3 phosphorylation was required for induction of IL-17 within neutrophils suggesting that a Stat3-RORgt pathway is involved and critical for regulating IL-21, 23, and 6 induced IL-17 production from neutrophils.

**Conclusion:** Th-17 regulatory cytokines, IL-21, IL-23, and IL-6 induce the production of pro-inflammatory cytokine IL-17 from neutrophils in a much higher levels during asthma. This environment of high IL-17 levels could stimulate neutrophils to produce highly reactive oxygen radicals that would exacerbate the airway inflammatory response during asthma via their cytotoxic and tissue-destructive activity.

## A367 Diagnostic Value of Serum Total IgE and Prediction of Cut-Off Value to Recommend Mast in Allergic Rhinitis

### Sun Kyung Kim^1^, Kwang Il Nam^2^, Hyung Chae Yang^2,3^

#### ^1^Gwangju Veterans Hospital; ^2^Chonnam National University Medical School, South Korea; ^3^Chonnam National University Hospital, South Korea

##### **Correspondence:** Hyung Chae Yang – Chonnam National University Medical School

**Objective:** As the avoidance of trigger allergen is a major treatment in allergic rhinitis, evaluation of trigger allergen is important for the treatment and prevention of allergy. However, the correlations between clinical symptom, MAST and total IgE are not clearly identified. In this study, we compared serum total IgE, MAST and allergic symptoms in allergic patients to analyze the diagnostic value of serum total IgE. Also, we analyzed the cut off value of serum total IgE to predict positivity of allergen specific IgE by using the sum of square estimator recently proposed by Froud et al.

**Methods:** A total of 1945 patients with allergic symptoms underwent MAST and serum total IgE tests. 39 panels were evaluated in MAST and allergens with results greater than class 2(≥0.7 IU/mL) in considered as positive. To analyze the results of serum total IgE with clinical symptoms, Total nasal score(TNS) was evaluated as sum of 4 nasal symptoms(rhinorrhea, nasal obstruction, sneezing and itching sense).

The patients were divided into high(≥100 IU/mL) and low(<100 IU/mL) groups of total serum IgE level and the positive rates and number of positive allergen specific IgEs were evaluated in each group. Furthermore, we calculated cut off value of serum total IgE to predict positive allergen specific IgE.

**Results:** Nasal obstruction turned out to be the most common symptom (65.6%). Total score of TNS showed significant correlation with serum total IgE quantity. High total serum IgE group showed significantly higher positive rates and number of positive allergen specific IgEs on MAST. (p<0.05). Number of allergen specific IgEs showed good correlation with serum total IgE(r=0.521, p<0.05).

With use of ROC curve, cut off value of serum total IgE was computed as 108 IU/mL(sensitivity 72.42%, specificity 72.87%). Due to low sensitivity, we analyzed positive predictive value of serum total IgE divided into each group. We suggested 50 IU/mL is more predictable.

**Conclusions:** Serum total IgE appears to be useful in predicting positive results of allergen specific IgEs in MAST. Also, serum total IgE with level of 50 IU/mL turned out to be most reliable to recommend MAST.

## A368 Diagnostic Value of an Increase in FEV1 and/or FVC >12% and >200 mL from Baseline after Bronchodilators for Diagnosis of Asthma

### Jeong-Eun Kim, Ju Suk Lee, Ji Hyun Lee, Kyung Woo Kang

#### Samsung Changwon Hospital, Sungkyunkwan University School of Medicine South Korea

##### **Correspondence:** Jeong-Eun Kim – Samsung Changwon Hospital, Sungkyunkwan University School of Medicine, South Korea

**Background:** The response to bronchodilators for asthma diagnosis is generally defined as an increase in FEV1 >12% and >200 mL from baseline after bronchodilators. However, an increase in FVC >12% and >200 mL from baseline not due to increased expiratory time after bronchodilators could also mean bronchodilation. So we evaluated diagnostic values of the FEV1 and/or FVC bronchodilator response.

**Methods:** The patients who were performed both methacholine challenge tests and pulmonary function tests with bronchodilator for suspected asthma from 2002 to 2013 were selected and the results of the tests from order communication system were reviewed. Diagnostic criteria of asthma were defined by one or more of following: the provocative concentration of methacholine causing a 20% fall in FEV1 from baseline (PC_20_) or extrapolated PC_20_***less than or*** equal to 25 mg/mL (1st criterion), an increase in FEV1 of >12% and >200 mL from baseline after 200 μg of salbutamol (2nd criterion), an increase in FEV1 of >12% and >200 mL from baseline after anti-inflammatory treatment or a variation in FEV1 of >12% and >200 mL between visits within 1 year and FEV1/FVC ≤0.75 at least once (3rd criterion). FEV1 and/or FVC bronchodilator response was defined as increases in FEV1 and/or FVC >12% and >200 mL from baseline after 200 μg of salbutamol. The sensitivity and the specificity of the FEV1 and/or FVC bronchodilator response for asthma diagnosis were calculated.

**Results:** A total 2616 pulmonary function tests with salbutamol and 1496 methacholine challenge tests in 1434 patients from 12 to 89 years old were analyzed. The diagnostic criteria of asthma were satisfied in 874 (60.9%) patients. Among them, numbers of patients who met each criterion were 831 (95.1%) for 1st criterion, 120 (13.7%) for 2nd criterion, 181 (20.7%) for 3rd criterion. Among 1834 pulmonary function tests in the asthma patients, 191 (sensitivity 10.4%) tests showed positive FEV1 and/or FVC bronchodilator response, while only 152 (sensitivity 8.3%) tests showed positive FEV1 bronchodilator response. False positive results in FEV1 and/or FVC bronchodilator response were shown in only 3 of 782 pulmonary function tests of patients without asthma (specificity 99.6%). The false positive results were shown in 3 different patients. Among them 2 patients were real asthma patients according to the results of other pulmonary function tests which were not evaluated in this study and the other patient had history of asthma.

**Conclusions:** An increases in FEV1 and/or FVC >12% and >200 mL from baseline after bronchodilators could have also diagnostic value for asthma.

## A369 Combined Use of Fractional Exhaled Nitric Oxide and Bronchodilator Response in Predicting Future Loss of Asthma Control Among Children with Atopic Asthma

### Je-Kyung Kim, Youn-Soo Hahn, Jae-Yub Jung

#### Chungbuk National University Hospital South Korea

##### **Correspondence:** Je-Kyung Kim – Chungbuk National University Hospital South Korea

**Background:** The aim of the present study was to see whether measurements of bronchodilator response (BDR) and fractional exhaled nitric oxide (FeNO) in combination are informative for upcoming loss of asthma control among children with atopic asthma.

**Methods:** Two hundred one patients aged 8 to 16 years with atopic asthma were recruited. Pulmonary function tests including BDR and FeNO were serially measured 10 times or more over 2 years when subjects were not receiving controller medications. After completion of monitoring, 1-year observation for loss of asthma control was performed.

**Results:** At least 1 positive BDR (≥12% improvement in FEV_1_ in response to inhaled short-acting b_2_-agonist) and high maximum FeNO (mFeNO) (≥35 parts per billion (ppb)) were confirmed over the 2-year observation period in 59% and 77% of study participants. There was no difference in FeNO levels between individuals with positive and negative BDRs. Risk of asthma control loss increased by 40% for patients with mFeNO ≥ 35 ppb [Hazard ratio (HR) = 1.94; *P* < 0.01], and by 26% for those with positive BDRs (HR = 1.40; *P* < 0.01). Risk of asthma control loss was greatest for patients with either (HR = 5.31; *P* < 0.01) or both positive BDRs and mFeNO ≥ 35 ppb (HR = 5.65; *P*< 0.01).

**Conclusions:** High FeNO was better able to predict upcoming loss of asthma control than BDRs, but use of both markers together provided a better indicator of asthma control loss.

## A370 Antigen-Specific IgA Plays an Important Role in Mucosal Immune Response in Allergic Children: Measurement of Secretory IgA and Antigen-Specific IgA

### Yosuke Baba^1,2^, Sususmu Yamazaki^1^, Eisuke Inage^1^, Mari Mori^1^, Yoshikazu Ohtsuka^1^, Masato Kantake^2^, Toshiaki Shimizu^1^, Asuka Honjoh^1^, Tomoaki Yokokura^1^

#### ^1^Juntendo University Faculty of Medicine; ^2^Juntendo University Shizuoka Hospital, Japan

##### **Correspondence:** Yosuke Baba – Juntendo University Faculty of Medicine

**Purpose:** IgA antibody is massively produced in the intestinal Peyer’s patches, and the secretory IgA (sIgA) plays an important role on immune responses. It is considered that sIgA regulates the cause of allergic reactions, but the relationship between IgA levels and allergic reactions is not fully understood. We studied sIgA levels and antigen-specific IgA levels in allergic children and studied their relationship with allergy symptoms.

**Methods:** It is a retrospective study using medical records of infants (from 6 months to 6 years old) who presented to our hospital for evaluation of allergy. We classified the groups according to the results of physical examinations with or without allergic symptom (eczema, wheezing, food allergy). In addition, we investigated the white blood cell counts (eosinophils and basophils) and the serum levels of total IgE, antigen-specific IgE, total IgA, sIgA, antigen-specific IgA.

**Results:** Children who were low levels in sIgA have past histories of atopic dermatitis, and their serum levels of antigen-specific IgE was significantly higher (p=0.008) but their serum IgA level was significantly lower (p=0.021) compared with children who does not have allergic symptoms. And serum levels of antigen-specific IgA were significantly lower (p=0.038) in allergic children. Especially, ovomucoid specific IgA levels were low in children who has ovalbumin allergy.

**Conclusions:** Secretory IgA levels are important to the onset of allergic reactions, and antigen-specific IgA might be played an important role in allergic reactions. IgE antibody contributes to immediate type allergic reactions, and the presence of the specific antibody can be an evidence of the diagnosis of allergic reactions. Moreover, we suggested that measurement of sIgA and antigen-specific IgA can be also useful in prediction of allergic reactions.

## A371 Why Teaching Pediatrics Trainees about Anaphylaxis and Its Acute Management Is Essential: Cross Sectional Survey

### Shaza Ali Mohammed Elhassan^1^, Caroline Beck^2^, Mehdi Adeli^2^

#### ^1^Hamad General Hospital; ^2^Hamad Medical Corporation Qatar

##### **Correspondence:** Mehdi Adeli – Hamad Medical Corporation, Qatar

**Background**

Anaphylactic shock is a life threatening circumstance which requires urgent and proper medical management. Epinephrine is the first-line and life-saving medication in the acute management. The delay in making an accurate diagnosis, initiating appropriate treatment and inappropriate use of epinephrine can lead to death.

**Objectives**

This study is designed to evaluate and emphasize the paramount importance of the trainee knowledge about anaphylaxis, the treatment methods, life-saving medications, the route of administration and the dosage. Our aim is to bridge the gap between knowledge and real life practice and enable the trainee to act undoubtfully when facing a patient with anaphylaxis.

**Method**

This is a cross-sectional two phase questionnaire based survey at Hamad General Hospital’s Pediatrics department, the only tertiary hospital in Qatar.

**Results**

In phase 1, the questionnaires were distributed to 96 trainees. The response was 98% (94 responses), 55 females and 39 males. 84 trainees (89% ) reported knowing how to treat and a total of 44 (50%) claimed not being trained at all. Epinephrine was selected as a life saving drug by 89 (94%). IM as a route of administration was selected by 76 (80%). Correct Epinephrine concentration was known by 77 (83%).

For phase 2, questionnaires were distributed to 94 trainees who responded to the stage 1 and the response rate was 89% (84). 84% claimed they heard about Epinephrine Autoinjector, 5 fellows claimed they never heard about it. 72% claimed knowing when to use it .23 (27%) did not know, 9 of them were Fellows. Anaphylaxis was the case of using it in 71%. Only 43 (51%) know the right location and the method of injection. Sub Cutaneous injection was selected in 20 (23.8%).

**Conclusion**

Although prompt treatment with epinephrine is deemed to be critically important for survival in anaphylaxis, we have huge gap between theoretical knowledge about epinephrine concentration and site of administration of epinephrine and fundamental practice among pediatrics trainees. Analysis of these data necessitates the urgent need of a concrete program for teaching the trainees, especially the pediatrics fellows to solidify their knowledge about anaphylaxis. More important practical guidelines about the site of administration, what concentration and how to act fast when faced with anaphylaxis is needed to be taught to current and future trainees.

## A372 Prevalence and Clinical Characteristics of Local Allergic Rhinitis in Children

### Heysung Baek^1^, Seung Jin Lee^2^, Ji Hyeon Baek^3^, Jungwon Yoon^4^, Sun Hee Choi^5^, Young-Ho Jung^2^, Youn Ho Shin^2^, Man Yong Han^2^, Min Sun Na^2^

#### ^1^Kangdong Sacred Heart Hospital; ^2^CHA University School of Medicine, South Korea; ^3^Dongtan Sacred Heart Hospital, College of Medicine, Hallym University; ^4^Myongji General Hospital; ^5^Kyunghee University Hospital at Gangdong

##### **Correspondence:** Min Sun Na – CHA University School of Medicine, South Korea

**Background**

Evidence demonstrates the existence of local allergic rhinitis (LAR) in nonatopic patients, although its prevalence in the rhinitis population remains unknown in children. The aim of this study was to evaluate the prevalence, clinical characteristics, and severity of LAR in children compared with patients having classical allergic rhinitis (AR) with systemic atopy or nonallergic rhinitis (NAR) with no sensitization to any of the inhaled allergens tested.

**Methods**

Two hundred-eighteen children between 9 months and 19 years old were enrolled and divided into 3 groups: 132 AR children, 68 NAR and 18 healthy controls. A clinical questionnaire and skin prick test (SPT) were evaluated. A nasal allergen provocation test (NPT) with Dermatophagoides pteronyssinus was performed in all subjects. The severity of ocular and nasal symptoms was recorded by visual analogue scale (VAS) of 10 cm. Each symptom was categorized as ‘mild’ (VAS: 0–3 cm), ‘moderate’ (VAS: >3–7 cm), or ‘severe’ (VAS > 7 cm).

**Results**

In the AR group, 43/132 (32.6%) patients presented a positive response to NPT. In the NAR group, 11/69 (15.9%) patients had a positive response to NPT. NPT was negative in 17/18 healthy controls (94.4%). The majority of rhinitis patients had moderate–severe nasal symptoms. LAR and AR subjects had a similar pattern of nasal symptoms in frequency and severity. However, significant differences were detected between LAR and NAR.

**Conclusion**

Local allergic rhinitis is a prevalent entity in patients evaluated with rhinitis. LAR and AR subjects had a similar pattern of nasal symptoms in frequency and severity. Conventional skin tests were significantly well correlated with nasal provocation tests.

## A373 House Dust Mites Sublingual Immunotherapy Can Influence the Long-Term Evolution of Severe Atopic Dermatitis and the Progression to Respiratory Allergy

### Enrico Compalati^1,3^, Maurizio Marogna^2^

#### ^1^Lofarma S.p.a. Italy; ^2^Ospedale Di Cuasso Al Monte; ^3^Allergy and Respiratory Diseases Clinic

##### **Correspondence:** Enrico Compalati – Allergy and Respiratory Diseases Clinic

**Background.** The role of allergen immunotherapy (AIT) in the treatment of patients with atopic dermatitis (AD) is quite controversial, since its efficacy has been poorly investigated in the past years and eczema exacerbations have been reported following AIT. We investigated the tolerability of hypoallergenic AIT, its benefit on eczema and its influence on the progression of the allergic disease.

**Methods.** This study included 12-55-year-old patients with severe AD (SCORAD >36) monosensitized to house dust mites (HDM) but without diagnostic criteria for rhinitis, asthma and food allergy at baseline. 22 subjects were treated in a continuous way for 5 years with sublingual tablets of carbamylated monomeric allergoid extract of HDM (2000 AU/week) and compared with 21 subjects treated with cetirizine 10mg daily. Topical and short course of oral steroids on demand were allowed and counted. The main endpoint was the change in SCORAD score at different time-points up to 10 years after AIT discontinuation. In addition, to assess the disease progression, data on respiratory symptoms and related medications were collected during mite exposition period, with lung function, bronchial hypereactivity, nasal eosinophils, onset of on new sensitizations. The occurrence of local or systemic adverse reactions was registered.

**Results**. No systemic serious reactions occurred during the 5 years. Adherence to the AIT regimen was good till the end of the treatment period and 20 subjects in AIT group and 18 in the control group reached the final observation at 15 years from baseline: the mean SCORAD index in AIT group decreased from 59.72 to 44.55, in control group from 57,04 to 55,55; the use of topical corticosteroids was halved in AIT group vs. control group; the use of oral corticosteroids was decreased only in AIT group; 2/20 patients in AIT group developed rhinitis and asthma, in control group 14/18 developed rhinitis and 9/18 asthma, requiring regular pharmacotherapy; only 2 patients in AIT group developed new sensitizations, whereas all controls resulted polysensitized.

**Conclusion.** AIT tablets of HDM carbamylated monomeric allergoid were well tolerated and provided sustained and long lasting partial improvement of the severity of atopic eczema, with remarkable reduction in the use of corticosteroids and preventive effect on the progression to respiratory allergy and onset of new allergic sensitizations.

## A374 The Positive Distribution Characteristics of 90 Food Specific IgG in Patients with Allergic Diseases

### Huimin Huang^1^, Baoqing Sun^1^, Mingyu Bai^1^, Yiting Huo^1^, Peiyan Zheng^1^, Nili Wei^1^, Wenting Luo^2^

#### ^1^The First Affiliated Hospital of Guangzhou Medical University, China; ^2^First Affiliated Hospital of Guangzhou Medical University, Guangzhou Institute of Respiratory Diseases

##### **Correspondence:** Huimin Huang – The First Affiliated Hospital of Guangzhou Medical University, China

**Objective**

To explore the levels and correlation of 90 food sIgG antibodies in serum in patients with allergic diseases.

**Methods**

Using ELISA to detect 90 kinds of sIgG antibody of 291 patients with allergic diseases in the first affiliated hospital of Guangzhou Medical University for diagnosis and differential diagnosis, Data were analyzed by SPSS19.0 statistical software.

**Results**

The positive rates of ten kinds of food sIgG antibody from high to low are milks and dairy products (33.03%), crustaceans (27.89%), meats and egg (19.31%), legumes (16.3%), fishes (15.56%), others (8.18%), vegetables (7.63%), nuts and oilseeds (6.70%), cereals (5.31%), the lowest is fruits (4.34%); the highest positive rate of food is egg (64.60%), followed by the white soft cheese (56.70%), cow’s milk (54.30%). In 0~3 years old group, the leading kind of positive rate of food is milks and dairy products 71.02%, decreasing with the age, in ≥60 years old group, the positive rate is 6.25%, which is the biggest change in ten categories of food according to age. Except ≥60 years old group, the positive rate of cow’s milk sIgG is higher than the goat’s milk sIgG, *p*<0.05. The positive tate of crustaceans is increasing with the age groups, scallop is the leading food in crustaceans in 7~16,17~44,45~59 and ≥60years old groups, the positive rates are 38.30%, 78.72%, 75.41% and 84.62%, respectively. The leading food of cereals is sesame in each group. In others food, the leaing food is sugar, the positive rate is 30.30% in 0~3years old and 34.88% in 4~6years old, in 7~16years old group, the leading food is coffee 29.79%, in ≥60 years old group, the leading food is tobacco 38.46%.

In 0~3 and 45~59 years old group, the positive rate of sIgG antibody of different categories of food were not statistically different in gender, in 4~6 years old group, the positive rate of cereals, fruits, vegetables of male are higher than female (x^2^=7.068, *p=.*003; x^2^=4.850, *p=.*031;x^2^=4.135, *p=.*042), in 7~16 years old group, the positive rates of vegetables and crustaceans of male are higher than female (x^2^=5.011, *p=.*026; x^2^=5.491, *p=.*019), in 17~44 years old group, the positive rates of vegetables of female are higher than male (x^2^=8.445, *p=.*004), in ≥60years old group, the positive rates of cereals, nuts and oilseeds, vegetables, milks and dairy products, fishes of male are higher than female (x^2^=3.902, *p=.*048; x^2^=6.836, *p=.*006; x^2^=16.228, *p=.*000;x^2^=6.163, *p=.*011;x^2^=8.123, *p=.*003).

A lot of food sIgG antibody levels are associated, there are 51 couples of food highly correlated (r_s_=0.8), including 34 couples of vegetables, 2 couples of fruit and vegetable.

**Conclusion**

There are certain distribution characteristics of 90 kinds of food sIgG antibody in different age and gender, clinical diagnosis should be combined with sex,dietary habits and other factors to make guidance more reasonable for the patient’s diet adjustment.

## A375 Evaluation of Serum Levels of Osteopontin As a Potential Biomarker of Immune Activation in Patients with Allergic Diseases

### Anand Andiappan^1^, Rosalba Minisini^2^, Olaf Rötzschke^1^, Elena Boggio^3^, Luca Gigliotti^3^, Nausicaa Clemente^3^, Annalisa Chiocchetti^3^, Umberto Dianzani^3^, Mario Pirisi^3^, Elisa Villa^2^

#### ^1^Agency for Science, Technology and Research (A*STAR), Singapore; ^2^University of Eastern Piedmont, Italy; ^3^University of Eastern Piedmont “Amedeo Avogadro”

##### **Correspondence:** Elisa Villa – University of Eastern Piedmont, Italy

**A) Background:** Osteopontin (OPN) is a pleomorphic cytokine known to influence a range of immune cells, including macrophages, neutrophils, dendritic cells, T and B cells. High OPN levels are associated with a significantly increased risk of autoimmune lymphoproliferative syndrome, multiple sclerosis and systemic lupus erythematosus, suggesting that OPN is a candidate biomarker of these conditions. In the present cross-sectional study, we aimed to verify if serum levels of OPN may qualify as a biomarker of an activated immune response in allergic patients.

**B) Method:** Serum OPN levels were measured by an enzyme-linked immunosorbent assay (ELISA) (Human Osteopontin Duoset, R&D Systems). A series of 77 adult patients (median age females: 49 years; males: 47 years) with different allergic diseases, was studied: 34 patients (44%) had allergic rhinoconjunctivitis, 15 (19%) asthma, 17 (22%) hymenoptera venom allergy, 5 (6%) allergic contact dermatitis, 3 (4%) food allergy and 3 (4%) IgE-mediated hypersensitivity to beta-lactams. 116 healthy subjects with similar demographic characteristics served as controls. Data were analyzed comparing cases to controls, as well as looking for subgroup differences within the group of allergic patients.

**C) Results:** OPN serum levels were significantly higher in cases in comparison to controls (median 12181 pg/ml, interquartile range 6953 – 19359 pg/ml vs 6099 pg/ml, interquartile range 3122 – 14520 pg/ml; p = 0.0010 by the Mann-Whitney test). The highest serum OPN levels were observed among patients with asthma (median: 15668 pg/ml; p = 0.0156) followed by those observed in the hymenoptera venom allergy group (median: 14239 pg/ml; p = 0.0080). Lower values of OPN were detected in the group of patients with rhinoconjunctivitis (median: 10291 pg/ml; p = 0.0436), allergic contact dermatitis (median: 9088 pg/ml) and food allergy (median: 4386 pg/ml). Patients with IgE-mediated sensitization to beta-lactams had heterogeneous values, not statistically different in comparison to controls.

**D) Conclusions:** Serum OPN levels may represent a novel, potentially useful biomarker of allergic respiratory diseases and hymenoptera venom allergy. Consideration should be given to explore clinical correlates of high OPN levels in these conditions.

## A376 Prevalence of Allergic Rhinitis in 3-6-Year-Old (preschool) Children in Chiba City (urban area), Japan

### Fumiya Yamaide^1^, Syuji Yonekura^1^, Naoki Shimojo^2^, Yuzaburo Inoue^2^, Yoshitaka Okamoto^3^

#### ^1^Chiba University Japan; ^2^Graduate School of Medicine, Chiba University; ^3^Unknown

##### **Correspondence:** Fumiya Yamaide – Chiba University, Japan

**Background**

The sequential development of allergic diseases (beginning with food allergy and atopic dermatitis followed by asthma and allergic rhinitis (AR)) during early childhood is often referred to as the allergy march. Recently, the number of school-age children with AR has shown to increase in Japan. But early onset of AR is poorly described, and it remains unknown about the prevalence of allergic rhinitis in young children.

**Objective**

We aim to evaluate the prevalence, clinical characteristics, and treatment of AR in a population of 3-6-year-old (preschool) children in Chiba city (urban area), Japan.

**Method**

A total of 13,963 children aged 3-6 years in all 84 kindergartens of Chiba city, Japan were surveyed. Prevalence of symptoms of allergic rhinitis was assessed using a modified version of the International Study of Asthma and Allergies in Childhood (ISAAC) questionnaire.

**Results**

A total of 9,822 (70.3%) questionnaires were returned for evaluation (sex: Male 50.5%, Female 49.5%; age 3y = 2.3%, 4y = 31.7%, 5y = 35.1%, 6y = 30.9%). The prevalence of lifetime, current and physician-diagnosed allergic rhinitis were 54.1%, 50.7% and 37.3%, respectively. The prevalence of AR was higher in males than that in females (cf. physician-diagnosed AR; 40.6% vs. 33.6%, P < 0.05) and increased with age (cf. physician-diagnosed AR; 3y = 18.5%, 4y = 28.3%, 5y = 37.3%, 6y = 46.7%). Many children showed AR symptoms during September and April, especially in February and March (cedar pollen allergy season). About 70 % of children with AR visited clinic or hospital, but more than half of them were dissatisfied with their treatment.

**Conclusion**

The prevalence of AR symptoms was high and starting early in life.

## A377 Comparative Efficacy of Combination Nebulized Salbutamol and Fluticasone Propionate and Nebulized Salbutamol in Children with Mild Moderate Asthma Attack

### Retno Asih Setyoningrum, Landia Setiawati, Sri Sumei, Deddy Iskandar

#### Airlangga University/Dr Soetomo Hospital, Indonesia

##### **Correspondence:** Retno Asih Setyoningrum – Airlangga University/Dr Soetomo Hospital, Indonesia

**Background:** Short acting beta-2 agonist (SABA), systemic corticosteroids and oxygen are the primary therapy in asthma attack. Repeated use of systemic corticosteroid is at risk of systemic side effects. Inhaled corticosteroid offer potential benefit because of direct effect on the airways and lower systemic side effects.

**Objective:** To compare the efficacy between combination of nebulized salbutamol and fluticasone propionate and nebulized salbutamol in children with mild and moderate asthma attack.

**Methods:** Thirty children (age 5-14 years) with mild and moderate asthma attacks in outpatient clinic and emergency department of Soetomo hospital were investigated in an open label randomized controlled trial study. Subjects are divided into treatment group (combination of salbutamol and fluticasone propionate) and control group (salbutamol), each of the 15 children. Pulmonary score and side effects (tachycardia and tremor) was evaluated at before and after nebulized at 20, 50 and 60 minutes. The statistical analysis used were t test, Mann Whitney test and the Wilcoxon signed rank test according to the type of data.

**Results:** The decreased pulmonary score at time evaluation was significant than before nebulized in both groups (p<0.05). The decreased pulmonary score in treatment group was greater than control group (p <0.05). Side effects such as tachycardia in both groups were not significantly different (p> 0.05) and tremor was not obtained in both groups.

**Conclusion**: Combination of nebulized salbutamol and fluticasone propionate had better efficacy than nebulized salbutamol in children with mild and moderate asthma attacks

## A378 Characteristics of Children Hospitalized with Asthma in West Nusa Tenggara General Hospital Mataram Indonesia

### Indriyani Sang Ayu Kompiyang

#### West Nusa Tenggara General Hospital Indonesia Indonesia

**Background**

Asthma is a chronic airway disease that largely controllable with proper primary care, and the need for hospitalization can usually be prevented.

**Methods**

A retrospective study to explore the clinical characteristics of children hospitalized with asthma was conducted at Department of Child Health, West Nusa Tenggara General Hospital, Mataram, Indonesia. All data about medical history and physical examination we got from pediatric asthma registry. Pediatric asthma registry in pediatric respirology division was started in October 2014, so that we presented the characteristics of children hospitalized with asthma from October 2014 to April 2015. We were also taken the number of children admitted with asthma in our emergency department and outpatient clinic at the same period for additional information. We presented the characteristics of our study subjects by table and chart.

**Results**

Fourteen children hospitalized with asthma were enrolled in this study from October 2014 to April 2015. Ten (71.4%) admitted from emergency department and 4 (28.6%) from outpatient clinic. The numbers of children with asthma who visited at that period were 368 visits at emergency department and 92 visits at outpatient clinic. We found 12 (85.7%) with moderate exacerbation, and 2 (14.3%) with mild exacerbation. Seven of 14 (50%) were referred as severe exacerbation to our emergency department, but after we do reassessment they were moderate exacerbation. Moderate exacerbation with frequent asthma was 10 (71,4%) of all the cases. Most of our subjects were male 9 (64.3%), aged between 2 and under 5 years old about 7 (50%), normal nutrition status 9 (64.3%), first asthma diagnosis at 2 years old in 6 (42.9%) cases, history of hospitalization before 8 (57.1%), used controller 1 (7.1%), history of atopic 9 (64.3%) and history of asthma in family 5 (35.7%), other allergic disease 3 (21.4%), feathered pet 4 (28.6%), cigarette smoking 8 (57.1%). The triggers for the asthma exacerbation were exercise 5 (35.7%), respiratory infection 4 (28.6%), cold weather 2 (14.3%), house dust 2 (14.3%), and food 1 (7.1%).

**Conclusions**

This study showed that most of children hospitalized with asthma in our setting were moderate exacerbation with frequent asthma, male, aged under 5 years old and first diagnosed at 2 years old, had history of hospitalization before, normal nutrition status, history of atopic in family, and cigarette smoking exposed.

**Keywords:** asthma in children, hospitalized, characteristic

## A379 Identification of Phenotypes in Allergic Bronchopulmonary Aspergillosis Using Cluster Analysis

### Tsuyoshi Oguma, Jun Tanaka, Katsuyoshi Tomomatsu, Koichiro Asano

#### Tokai University School of Medicine Japan

##### **Correspondence:** Tsuyoshi Oguma – Tokai University School of Medicine, Japan

**Background:** Allergic bronchopulmonary aspergillosis (ABPA) is known as type I and III allergic disease for Aspergillus. Clinical presentation of ABPA ranges from asymptomatic mucus plugs to severe asthmatic symptoms or destructive and fibrotic lung disease. The heterogeneity may reflect not only the different clinical stages of the disease, but also different phenotypes. We herein attempt to identify phenotypes in ABPA using cluster analysis.

**Methods:** We analyzed the data of 332 patients with possible ABPA from national-wide survey in Japan executed between 2013 and 2014. Definition of possible ABPA were, (1) positive skin test or specific IgE for Aspergillus, and (2) either a) positive precipitation or IgG antibody for Aspergillus or b) mucoid impaction or bronchiectasis in chest computed tomography. Non-hierarchical cluster analysis using k-means method was performed.

**Results:** Three clusters were identified. Cluster 1 (n=141) included the patients with later age at onset (mean ages, 68 years), female-dominance, and less frequent prevalence of asthma (76%). The patients in cluster 2 (n=95) were middle age at onset (55 years), female-dominant, and showed lower values of total serum IgE. Cluster 3 (n=96) was characterized with early-onset (37 years), male-dominance, and frequent recurrences (59%).

**Conclusions:** Three distinct clinical phenotypes were identified characterized by ages of onset, gender, asthma prevalence, total serum IgE levels, and the frequency of recurrences.

## A380 The Roles of Type 2 Innate Lymphoid Cells (ILC2) in Chronic Rhinosinusitis (CRS)

### Keisuke Uno^1^, Yoshinori Matsuwaki^1^, Kazuhiro Omura^1^, Eika Hayashi^1^, Norifumi Tatsumi^1^, Hirohito Kita^2^, Nobuyoshi Otori^1^, Hiromi Kojima^1^

#### ^1^Jikei University, Japan; ^2^Mayo Clinic, USA

##### **Correspondence:** Keisuke Uno – Jikei University, Japan

**Background:** Chronic rhinosinusitis (CRS) is one of the most frequent chronic diseases, and little is understood about its pathogenesis. Eosinophils are considered to play a major role in its pathology, but we still know little which is causing chronic immune activation and persistent eosinophilic inflammation in CRS. Recently, type 2 innate lymphoid cells (ILC2s, lineage (-), CD45 (+), CD127 (+), CD294 (+)) were identified as a candidate, which produce highly levels of Th2 cytokines such as IL-5 and IL-13, which activates eosinophils. We hypothesized that ILC2s are enriched in blood and nasal polyps in patients with eosinophilic CRS (ECRS) and are associated with its pathology.

**Methods:** FThe patients with CRS or pituitary adenoma (normal sinus) who underwent Endoscopic sinus surgery (ESS) in Jikei University Hospital were enrolled. We used PBMC and nasal polyps (NPs) from patients with CRS or normal subjects, and analyzed the amount of ILC2 by flow cytometry. We also investigated the distribution of ILC2s in NPs by immunohistochemistry. EDN and cytokines in NPs were measured by ELISA to investigate correlation with ILC2s. Lineage negative cells from nasal polyps were cultured in vitro with IL-33 or/and IL-2 to investigate the amount of cytokine produced by ILC2s.

**Results:** EDN and Th2 cytokines are significantly higher in ECRS than non-eosinophilic CRS (NECRS). EDN had strongly correlated with the numbers of ILC2s in NPs. The counts of ILC2s in NPs were significantly higher in ECRS than NECRS. Immunostained ILC2 were showed accumulated in nasal polyps of ECRS, but not in NECRS or normal subjects. ILC2’s CD25 surface expression in PBMC was significantly higher in ECRS than NECRS. ILC2’s IL-17RB surface expression in NPs was significantly higher in NECRS than ECRS. Lineage negative cells from ECRS’ NP, but not from NECRS’, produced IL-5 and IL-13 in both IL-2 and IL-33 stimulation.

**Conclusions:** ILC2 are considered as candidate of the commander in ECRS, which strongly induce Th2 inflammation. There are possibility that ILC2s have several subtypes and the characteristic of ILC2s are differ from their environment.

## A381 Respiratory Symptoms, Signs and Spirometry Indexes Comparision in 7-12 Years Old Girls in Esfahan Metropolis and Its Far Suburb

### Mohammadreza Fatemi Khorasgani

#### Islamic Azad University- Najafabad Branch, Iran

**Introduction**- Esfahan, the famous historical city, now has more than 2.5 million citizens with about one million vehicles and, because of its unique geographic position, takes place as the main industrial center in Iran. Air pollution in Esfahan is a complex problem and its effect in children respiratory quality is the main goal of this research.

**Material and Methods**- In this cross sectional study with convenient sampling during 2013 winter, we have a total 40 girls, 7- 12, from Esfahan CBD and 40 from the city of Tiran, a small and calm city 45 Km. west of Esfahan with nearly same climate. For every participant we complete the questionnaire for anthropomorphic values and past year respiratory symptoms. For spirometry evaluation we used a Spirolab 3.Datae analyzed by SPSS version 20.

**Results**- For Esfahan and Tiran girls age was 9.4+/-1.6 against 9.9+/-1.3, for height 135.5+/-10.6 against 140.5+/-11, for weight 31.8+/-11.3 against 34.8+/-13.8 and for BMI 16.4+/-3.1 against 17.2+/-4.7 which are near to each other. For main respiratory symptoms, cough 18.2% against 6% (P-value=0.53), dyspnea 14% against 5(P-value=0.08) and wheezing 3 person against zero. For spirometry indexes, for vital capacity (VC) percent 114.1+/-34.9 against 87.2+/-16.7(P-value=0.000), FVC 2.0+/- 0.6 against 2.1+/-0.7(P-value=0.56), FEV_1_ 1.59+/- 0.39 against 1.83+/-049(P-value=0.15), for FEV_1_/VC 76.48+/-25.2 against 94.52+/-13.3, for FEF 25 75 1.81+/- 0.62 against 2.61+/- 2.20 (P- value= 0.000).

**Conclusion**- Quality of respiratory air differ normal physiological indexes even in childhood. Some of them are probably compensatory like VC, but others are capable to limit normal activities step by step.

## A382 Induction of Kruppel-like Transcription Factor (KLF4&5) By Baker’s Yeast Mannan in Human Bronchial Epithelial and Smooth Muscle Cells

### Dukhee/Betty Lew, Kim/S. Lemessurier, Joseph/a Moore, Jeoung-Eun Park, Ae-Kyung Yi, Chi/Young Song, Kafait/U Malik

#### University of Tennessee/Le Bonheur Children’s Hospital’s, USA

##### **Correspondence:** Dukhee/Betty Lew – University of Tennessee/Le Bonheur Children’s Hospital’s, USA

**Rationale:** Mannan derived from *Saccharomyces cerevisiae*(SC-MN) modulates allergic asthma pathogenesis in a mouse model. The purpose of this study is to explore downstream transcription factor(s) involved in SC-MN’s beneficial effects in asthma. The KLFs are important transcription factors in epithelial survival, and modulate epithelial mesenchymal transition and smooth muscle proliferation. As KLF4&5 are highly expressed in lung, we hypothesize that SC-MN can induce KLFs in lung cells.

**Methods:** Primary normal human epithelial cells (NHBEC) and bronchial smooth muscle cells were incubated with SC-MN and examined for KLF4, KLF5, p38MAPK levels and phosphorylation over a time course by Western Blot (WB). Normal human bronchial smooth muscle cells were analyzed for SM22alpha levels by WB and alpha-isoactin by indirect immunofluorescent staining.

**Results:** Following exposure to SC-MN, protein levels of KLF4 and KLF5 increased in both NHBEC and NHBSM cells over the subsequent 2-18 h. SC-MN–treated bronchial smooth muscle cells, but not in NHBEC, showed biphasic activation of p38MAPK (5-120 min and 8 h) that is known to lead to KLF phosphorylation in vascular smooth muscle cells. In addition, SC-MN increased smoooth muscle specific alpha-isoactin and SM22alpha at 24 hrs, consistent with a phenotype change.

**Conclusions:** SC-MN can induce KLF4 or KLF5, transcription factors that are important in epithelial survival and regulation of smooth muscle proliferation.

## A383 Korean Profile in Childhood Asthma Severity Classification

### Ja Kyoung Kim^1^, Hyeon-Jong Yang^2^, Bong-Seong Kim^3^, Youn Ho Shin^4^, So-Yeon Lee^5^, Geunhwa Park^6^, Woo Kyung Kim^7^, Hyo-Bin Kim^8^, Heysung Baek^9^, Dae Hyun Lim^10^, Dae Hyun Lim^11^, Jin Tack Kim^12^, Dongin Suh^13^

#### ^1^Department of Pediatrics, Kangwon National University; ^2^Soonchunhyang University College of Medicine; ^3^Gangneung Asan Hospital; ^4^Gangnam CHA Hospitatl; ^5^Hallym University Sacred Heart Hospital; ^6^Busan St. Mary’s Medical Center; ^7^Seoul Paik Hospital; ^8^Sanggye Paik Hospital; ^9^Kangdong Sacred Heart Hospital; ^10^Inha University Hospital, Incheon; ^11^Environmental Health Center for Allergic Rhinitis; ^12^The Catholic University of Korea; ^13^Seoul National University Hospital South Korea

##### **Correspondence:** Dongin Suh – Seoul National University Hospital, South Korea

**Background**

For the proper asthma management, information on the exact distribution of asthma severity is fundamental. In developing Korean pediatric asthma guideline, baseline data such as the severity distribution of or the prescription pattern to Korean asthmatic children are not available.

**Methods**

We have requested 12 pediatric allergists to review the medical records on asthmatic children who visited their own clinic during the most recent 3 months. Based on the subjects’ symptoms, signs and their medications to maintain control, their asthma severities were assessed according to both the Global Initiative for Asthma (GINA) criteria and the Japanese Pediatric Guidelines for the Treatment and Management of Bronchial Asthma (JPGL) criteria.

**Results**

A total of 840 cases (<3 years, 22.2%; 3-6 years, 26.4%; >6 years, 53.3%) were reviewed. Both criteria revealed that about 1/3 of asthmatic children had intermittent asthma whereas only <3% of subjects had severe persistent asthma. The pattern of prescribe controllers varied widely and did not match with asthma severity classification by either GINA or JPGL criteria, which was not confined to those in whom the severity assessed by each criterion was different from each other.

**Conclusions**

In Korea, there were distinctive patterns of distribution in the asthma severity, especially the prevalence of severe asthma was low. In the practice, patterns of prescription did not match with recommended modalities according to the severity assessed by either GINA or JPGL. The results will provide an evidence on the current state of Korean asthma practice in developing new practice guidelines suitable for Korean asthmatic children.

## A384 Prevalence of Food Sensitization, IgE-Mediated and Non-IgE-Mediated Food Allergy Among Pediatric Patients Diagnosed with Autism Spectrum Disorders

### Aimee Lou Manalo Nano

#### University of the Philippines Philippine General Hospital, Philippines

**A) Background:** Autism spectrum disorders and food allergies are conditions with increasing prevalences. Studies have investigated the link between intake of certain food among ASD patients and onset of adverse reactions. Results of these studies are varied and conflicting. This is the first local study on the prevalence of food allergies among these patients.

This study aims to determine the prevalence of food sensitization, IgE-mediated and non-IgE-mediated food allergies among pediatric ASD patients. It also aims to determine the prevalence of perceived food allergies, and its triggers and the types of reactions of perceived food allergies and on open food challenge.

**B) Methods:** This is a cross-sectional, prospective study. Pediatric patients diagnosed with Autism Spectrum Disorders were enrolled in the study. Excluded were: children with uncontrolled asthma, with a recent anaphylactic reaction, and those on chronic high dose steroid therapy. Required sample size is 92. Complete history and PE were obtained and previous reactions to food and suspected allergens were duly noted. All patients underwent skin prick testing (SPT) to cow milk, wheat, soy and other perceived food allergens. Those with perceived food allergies underwent open food challenge to specific food allergens. Frequencies and proportions were determined to analyze the different variables.

**C) Results:** Data were gathered from 84 patients diagnosed with ASD. 32/84 (38%) have perceived food allergies, mostly to milk (31.3%), chocolate (25%), and egg (18.8%). Most commonly perceived allergic reactions to food allergens were: hyperactivity (53.1%), loose stools (25%), pruritus (15.6%), and wheals (12.5%). A total of 17 (20.2%) patients had (+) skin prick test result, hence food sensitization, to at least 1 food allergen, mostly to soy (41.2%) and milk (35.3%). Of these patients, 8 had perceived food allergies. 6 of these patients underwent open food challenge and all of them had (-) results.

**D) Conclusion:** Prevalences of perceived food allergies and food sensitization are higher among ASD patients in this study compared to the general population. The most common perceived food allergens are similar with that of other children. Notably, the common perceived allergic reactions to food were behavioral or gastrointestinal symptoms, which may be non-IgE mediated or not food allergies at all. Hence, ASD patients with adverse food reactions are recommended to undergo complete, systematic evaluation and possible restrictive diets should be based on well-documented food allergies

## A385 Component-Resolved Diagnostic Study of *Dermatophagoides Pteronyssinus* Major Allergen Molecules in a Southern China

### Baoqing Sun^1^, Wenting Luo^2^

#### ^1^First Affiliated Hospital Guangzhou Medical University; ^2^First Affiliated Hospital of Guangzhou Medical University, Guangzhou Institute of Respiratory Diseases China

##### **Correspondence:** Wenting Luo – First Affiliated Hospital of Guangzhou Medical University, Guangzhou Institute of Respiratory Diseases, China

**Background:** Little is known about the data on component-resolved diagnosis (CRD) for allergy to *Dermatophagoides pteronyssinus* (Der p) in the Chinese population. We aimed to measure the prevalence of sensitization to Der p allergen components among patients in southern China.

**Methods:** 200 Der p-positive and 20 Der p-negative subjects were tested for serum immunoglobulin E (sIgE) against Der p 1, Der p 2, and Der p 10 using ImmunoCAP 100. 75 poly-sensitized patients were further examined with ImmunoCAP Immuno Solid-Phase Allergen Chip (ISAC). Der p 10-positive subjects were tested further for sIgE to crude extracts of cockroach, moth, and shrimp.

**Results:** 91.5% (183/200) patients were sensitized to Der p 1 and/or Der p 2. 6% (12/200) Der p-positive patients were sensitized to Der p 10. The positive proportion and median level of sIgE against Der p 1 were higher in children than in adults. Der p 1 and Der 2 correlated with Der p in sIgE levels (r=0.862, 0.799, *P*<0.001). ImmunoCAP ISAC demonstrated 100% specificity and 84% sensitivity in detecting Der p 1, p 2, and p 10 compared to ImmunoCAP 100. According to ImmunoCAP ISAC, 8 of these 12 Der p 10-positive patients were triple positive and 3 patients were triple negative to Pen m 1, Bla g 7, and Ani s 3; one was solely positive to Pen m1. Sensitization to Der p 10 correlated well with sIgE to shrimp, moths, cockroaches, Pen m 1, Bla g 7, and Ani s 3.

**Conclusions:** The detection of Der p 1 and Der p 2 well reflected atopy to Der p in a Chinese cohort. Sensitization to Der p 10 may result from cross-reactivity to seafood and cockroaches in coastal southern China. ImmunoCAP ISAC may offer a useful tool for CRD with performance comparable to ImmunoCAP 100.

## A386 Risk Factors for Systemic and Local Reactions to Subcutaneous Allergen Immunotherapy

### Hikmet Tekin Nacaroglu^1^, Semiha Bahceci Erdem^1^, Ozlem Sumer^1^, Sait Karaman^1^, Canan Sule Unsal Karkiner^1^, Suna Asilsoy^2^, Ilker Gunay^1^, Demet Can^1^

#### ^1^Dr Behcet Uz Children’s Hospital Turkey; ^2^Dokuz Eylul University Hospital

##### **Correspondence:** Hikmet Tekin Nacaroglu – Behcet Uz Children Hospital, Turkey

**Background:** Local, and especially systemic, reactions are important problems in subcutaneous immunotherapy (SCIT). Local reactions develop in 0.7-4% of all injections and systemic reaction develops in 0.2%. The current study aimed to evaluate the frequency and risk factors of reactions developing in patients undergoing SCIT.

**Methods:** Local and systemic reactions developing after 14,308 injections between 2003 and 2013 were retrospectively evaluated in the current study. The grading system for systemic reactions that was recommended by the World Allergy Organization (WAO) was used. The type of allergic disease, the allergens producing sensitivity, the vaccine content, the adjuvant content, and the effects of treatment phase on frequency of adverse effects were investigated.

**Results:** Out of 329 patients included, there was local reaction in 11.9%, large local reaction in 6% and systemic reaction in 4.7%; local reactions were observed in 0.38% of all injections, whereas a systemic reaction was observed in 0.1% of all injections. Local reactions were frequent at the initial phase and systemic reactions were frequent at the maintenance phase (p=0.01). Adverse reactions were more common in patients vaccinated (SCIT) with multiple allergens and house-dust-mites (p=0.002) (p=0.001). No statistically significant difference was found between the content of the adjuvant and the frequency of adverse effects (p=0.319).

**Conclusion:** The frequency of local and wide local reactions during subcutaneous immunotherapy were lower than expected. Although systemic reactions are frequently seen, no fatal reaction was observed in the current study. Mite immunotherapy and multiple allergen use increase the risk of reaction.

## A387 Literature Review and Current Treatment Options for Cyclical Anaphylaxis

### Danielle Kiers

#### Toronto Allergists, Canada

**Background:** Cyclical anaphylaxis is a rare reaction occurring in the luteal phase of menstruation. Presentation may include dyspnea, respiratory distress, cutaneous and gastrointestinal symptoms. Previous successful treatment options of this condition have been limited to medical or surgical ovulatory suppression. At present, the use of IgE-inhibition with the drug omalizumab has shown success in multiple individuals, making it the favorable treatment over oophorectomy for women looking to conceive. We report the first successful case series of four patients treated with omalizumab for cyclical anaphylaxis.

**Methods:** A literature review was conducted of published data pertaining to cyclical anaphylaxis in the luteal phase of menstruation. This information was summarized and the four patients from our center were included in this summary.

**Results:** Omalizumab has resulted in symptom resolution or significant reduction in symptoms in all four patients. There is no consistent literature definition of cyclical anaphylaxis and the nomenclature of similar and/or overlapping conditions makes any uniform treatment decisions challenging to make consensus recommendations for. For cyclical urticaria and anaphylaxis, omalizumab and represents a safer and preferable treatment option for this rare condition(s).

**Conclusion:** Through evaluating data regarding the use of omalizumab it can be noted that this is the preferred treatment of cyclical anaphylaxis for women who would like to maintain the option of conception later in life. Continued research is necessary in order to fully understand the generalizability and reliability of study data to the population. We also propose a more systematic classification of this and related conditions to better direct future research.

## A388 The Effect of Surfactant Protein D in Acute Lung Injury and Pulmonary Fibrosis Induced By Bleomycin

### Jiu-Yao Wang^1^, Hsu Han Yin^2^

#### ^1^National Cheng Kung University Hospital; ^2^National Cheng Kung University Taiwan

##### **Correspondence:** Hsu Han Yin – National Cheng Kung University, Taiwan

Acute lung injury (ALT), which is associated with a high mortality and morbidity in both infants and adults, is caused by severe lung inflammation resulted from a variety of local and systemic infection such as sepsis and pneumonia. According to the disease procession, there are three stages: exudate formation, proliferation, and fibrosis. Idiopathic pulmonary fibrosis (IPF) is another chronic, progressive and lethal fibrotic lung disease. The etiology of IPF is still unknown. However, both diseases have some similar hallmarks such as hypoxemia and respiratory failure. Surfactant protein D (SP-D), a C-type lectin, which is produced by alveolar type II cells, is important on respiratory innate immunity and anti-inflammation. The action of SP-D in these diseases is still unrevealed. In this study, we used bleomycin to induce the animal model of ALI and IPF. Bleomycin is a chemotherapeutic antibiotic drug clinically used in lymphoma and squamous cell carcinomas, but the following overproduction of reactive oxygen can lead to irreversible lung injury. In this animal model, we have found that 14-day-course was the group presenting the most severe resistance and the poorest elastance of lung tissue. In addition, the pro-inflammatory cytokines (interleukin-6, interleukin-17, tumor necrosis factor-α, interferon-γ and nitric oxide) were followed by increased expression of pro-fibrotic cytokines (transforming growth factor-β1). The histological alterations caused by bleomycin such as mural incorporation of collagen and obliteration of the alveolar space are similar to human IPF. In *ex vivo* study, pretreatment with recombinant human SP-D or native SP-D can significantly decrease the production of pro-inflammatory cytokines. In *in vivo* study, treatment with native SP-D in bleomycin-induced lung fibrosis in mice also showed significant body weight increase, recovery of lung function, decline of the production of transforming growth factor-β1 (TGF-β1) and nitric oxide. Therefore, we conclude that SP-D may have prominent anti-inflammatory and anti-fibrotic effects on ALI and IPF, and also have the potential to become a novel treatment of ALI and IPF in the near future.

## A389 Activation of Endothelial Cells to Release Hsp90, an Activator of the Prekallikrein-High Molecular Weight Kininogen (HK) Complex

### Allen Kaplan, Kusumam Joseph, Baby G. Tholanikunnel

#### Medical University of South Carolina USA

##### **Correspondence:** Allen Kaplan – Medical University of South Carolina, USA

We have previously demonstrated that Hsp90 release by activated endothelial cells leads to conversion of prekallikrein to kallikrein if prekallikrein is bound to HK. Kallikrein formation is therefore stoichiometric and occurs in the absence of factor XII. Since kallikrein activates factor XII, we theorized that endothelial cell activation might first generate kallikrein which then recruits factor XII. We now demonstrate that estrogen, interleukin 1, and to a lesser degree TNFa can activate endothelial cells to release Hsp90. The dose range tested for each in ng/ml was 0, 0.5, 1.0, 5.0, and 10.0. A dose-response for estrogen or IL-1 was maximal at the 10 ng/ml dose while TNFa was best between 0.1 and 5 ng/ml. A time course for each up to 4 hrs incubation revealed maximal Hsp90 secretion between 30 and 60 min with a significant increase noted by 15 min for each. Our observations are particularly relevant for types I and II HAE (C1 inhibitor deficiency) where estrogen and inflammation are known triggers of angioedema events and for HAE with normal C1 inhibitor (HAE-N) where estrogen exposure is a key precipitant. For the latter, studies of endothelial cell release of urokinase and tissue plasminogen activator are in progress given recent observations regarding inhibitors of fibrinolysis.

## A390 The Effect of Climatic Treatment in 51 Asthmatic Children from Areas Severely Polluted Environment of Northern Moravia, Czech Republic

### Radim Dudek

#### Medical Professional Institute, Czech Republic

**A) Background**

In the period of September 2014 - March 2015 in the homes of children from northern Moravia, Czech Republic it was only 12 sunny days and in 56 days limits for dust and polyaromatic hydrocarbons were overthrown. In the medical professional institute Metylovice located 410 meters above sea level found in Štramberk highlands during a 4-week stay for treatment with vitamin D, we investigated the effect of climate on the parameters of bronchial asthma and changes of physical parameters by modified Cooperœ test in asthmatic children. The main goal of the study is to assess the effectiveness and benefits of the stay with described treatment.

**B) Methods**

Over a 28-days period 51 children patient underwent the long term stay with the medical treatment in medical institute in Metylovice. In total 31 girls and 20 boys in the same age with bronchial asthma fulfilled the inclusion criterion (GINA 2014) and were included in the study. In all patients, atopic status was evaluated by skin prick testing using common allergen extracts (grass and tree pollen, house dust mite, moulds, cat/dog extracts). Patients blood eosinophils leukocyte count, levels of total sesrum imunoglobulin E, serum eosinophil cationic protein and exhaled nitric oxide levels were determined.Daily the patients received 1500 IU of Vitamin D3 orally.

**C) Results**

The object was to evaluate the effectiveness and benefits of long term stay for asthmatic children after the continuous treatment with vitamin D and physical exercises.The evaluation was done by the comparison of laboratory examination of blood and physical parameters in the beginning of the stay and in the end of the stay. It was determined the hypothesis that the long term stay has significant positive effect on level of vitamin D3 31.80 mmol/l in the beginning of the stay vs 45.58 mmol/l(p<0.001) and some parameters of physical fitness of children in the study done by modified Cooper’stest such as the test of abdominal muscles 18.45 pull s/min vs 27.15 pulls/min (p<0.001).

**Conclusions**

The climate stay with the exclusion of the negative influence of the environment with the activity of vitamin D3 led to a stabilisation of asthma without the need of treatment by salbutamol spray. Subsequently, the higher the intensity of rehabilitation exercises increased muscle strength and improved the quality of breathing.

## A391 Comparison of Some Vitamin Groups in Asthmatic Patients

### Gulden Bilgin, Hatice Surer, Aytun Sadan Kilinc, Dogan Yucel

#### Ankara Training and Research Hospital, Turkey

##### **Correspondence:** Gulden Bilgin – Ankara Training and Research Hospital, Turkey

**Background:** Being a chronic inflammatory disease, asthma accompanies many diseases. For astmatic patients, comparison of some vitamin groups due to severity of asthma and replacement treatment in case of lack can be extremely important.

**Method:** Respiratory fuction tests were conducted on 302 patients (193 female-109 male, totally with age group of 18-79) who applied to Chest Diseases Polyclinics between January-May 2015. Patients were classified as mild, moderate and severe due to GINA 2014 criteria. Vitamin A, vitamin D, vitamin E, folic acid and vitamin B12 levels were investigated. Statistically, Mann Whitney U, Kruskall Wallis-H and Post-Hoc tests were applied.

**Results:** In all age groups, vitamin A and vitamin D values of females were significantly lower than those of males (p<0.05). In patients of age 29 and lower, folic acid values were significantly low. Due to vitamin A, vitamin D, vitamin E, vitamin B12 and folic acid, no statistically significant difference was detected among astmatic levels of patients enrolled in the study (p>0.05).

**Conclusions:** Beside physiopathologic changes during astma, evidence of free oxygen radical release out of inflammatory cells and decrease in antioxidant levels give an impression of oxidant-antioxidant disequilibrium role in astmatic pathogenesis. As a defence mechanism of body against oxydative stress, outsourcing of vitamins is therefore essential. Low values of vitamin A and vitamin D values in astmatic patients encourage astmatic symptom severity, in addition to exhaustion, fatigue, bone ache and eye diseases. Reinforcement of vitamins through nutrition and medication is believed to be useful in treatment of asthma.

## A392 Sensitization in Children with Atopic Dermatitis: A Single Center Study

### Ji Young Lee, Jihyun Kim, Hea-Kyoung Yang, Minji Kim, Sang-Il Lee, Kangmo Ahn

#### Samsung Medical Center, South Korea

##### **Correspondence:** Ji Young Lee – Samsung Medical Center, South Korea

**Purpose:** There are few recent epidemiologic data regarding allergic sensitization of atopic dermatitis (AD) in Korea. The aim of this study was to investigate patterns of sensitization in children with AD.

**Methods:** This retrospective study included pediatric patients (0-18 years old) with AD who visited Samsung Medical Center from 1998 to 2014. The serum specific IgE (sIgE) levels of egg white (EW), cow’s milk (CM), peanut, wheat, soy, buckwheat, tree nuts, crustaceans, meats and house dust mites (HDMs) were reviewed. The sIgE level ≥ 0.35 kU/L was regarded as positive. AD was categorized into the extrinsic type (ADe) and the intrinsic type (ADi) according to the presence or absence of positive sIgE. We compared the proportion of sensitized children according to their ages using Chi-Square Test. The prevalence of immediate-type egg and CM allergies was also evaluated based on the previously reported diagnostic decision point (DDP).^1)^

**Results:** Data were collected from total of 4775 children (2928 boys and 1847 girls). We identified 3321 (69.5%) children with ADe type, and 1455 (30.5%) with ADi type. There was no difference in the proportion of sensitized patients according to their age (*P* value = 0.538). Ratio of positive sIgE among the individual food item was the highest in EW (2348/3994, 58.8%), followed by CM (1776/3836, 46.3%), peanut (1244/3848, 32.3%), wheat (1119/3546, 31.6%), soy (984/3503, 28.1%), and buckwheat (267/1118, 23.9%). Among the food groups, tree nuts (405/715, 56.6%) were the most common allergens. Sensitization to *Dermatophagoides farinae* and *D. pteronyssinus* was found in 43.5% (799/1837) and 39.3% (722/1837), respectively. In addition, 10.9% (435/3994) and 7.4% (284/3836) showed the higher levels of sIgE to EW and CM than previously reported DDP.

**Conclusions:** The frequency of ADe among all the children with AD was 69.5%. The most frequently sensitized food allergen was EW, followed by CM and peanut.

**Reference**

1. Allergy Asthma Immunol Res. 2015;7(4):332–8

## A393 Staphylococcal Enterotoxin IgE Sensitization: A Risk Factor for COPD Overlap in the Elderly Asthma?

### Sung Do Moon^1^, Byung-Keun Kim^1^, Sang-Heon Cho^1^, Kyung-up Min^1^, Yoon-Seok Chang^2^, Heung Woo Park^1^, Hye-Ryun Kang^1^, Woo-Jung Song^1^, Min-Koo Kang^1^, Ju-Young Kim^1^, Kyonghee Sohn^2^, Ha Kyung Won^1^, Seoung-Eun Lee^3^, Kyung-Mook Kim^4^, Claus Bachert^5^

#### ^1^Seoul National University Hospital, South Korea; ^2^Seoul National University Bundang Hospital, South Korea; ^3^Yangsan Pusan National University Hospital, South Korea; ^4^Pogunhan Mom Hospital, South Korea; ^5^University of Ghent, Belgium

##### **Correspondence:** Sung Do Moon – Seoul National University Hospital, South Korea

**Background:** The pathogenesis of asthma-chronic obstructive pulmonary disease overlap syndrome (ACOS) is supposed to be multifactorial but unclear. Recent evidence suggests staphylococcal enterotoxin IgE sensitization (SE-IgE) to be a risk factor for asthma and its severity in adults, including the elderly. We hypothesized SE sensitization contributes to the development of fixed airway obstruction in asthma, leading to an ACOS phenotype. The present study aimed to examine the associations of SE-IgE sensitization with fixed airway obstruction in elderly asthma patient.

**Methods:** We compared baseline characteristics between elderly controls, asthma and ACOS patients (≥65 years). Baseline assessment included demographics, lung functions, comorbidities, and serum total IgE and SE-IgE levels. SE-IgE sensitization was classified as negative (<0.10 kU/L), moderate (0.10-0.35 kU/L), and high (≥0.35 kU/L). SE-IgE sensitization was defined positive as ≥0.35 kU/L. Lung functions were serially checked every 3 month for two years in elderly asthma patients, and their best FEV1/FVC ratio was used to define fixed airway obstruction (persistently FEV1/FVC<0.7 during 2-year of asthma management). Exacerbation frequency was assessed during 1-year prospective follow-up.

**Results:** A total of 338 elderly subjects were analyzed (89 controls, 172 asthma, and 87 ACOS). Serum SE-IgE levels were higher among elderly ACOS and asthma patients, compared to controls (mean±SD; 0.74±1.34, 0.56±2.32, and 0.20±0.36, respectively). SE-IgE sensitization rates significantly differed between groups (high SE-IgE sensitization; 44.2% in ACOS, 27.9% in asthma, and 14.6% in control; P<0.001). The presence of fixed airway obstruction (FEV1/FVC<0.70) showed relationships with male sex, smoking history, chronic rhinosinusitis (CRS), and SE-IgE sensitization. In multivariate analyses, smoking history and high SE-IgE sensitization had independent relationships with fixed airway obstruction. In both of asthma and ACOS groups, SE-IgE levels showed significant correlations with exacerbation frequency.

**Conclusions:** Staphyloccocal enterotoxin could have a pathogenic role in the development of COPD overlap in late-onset elderly asthma. These findings warrant further investigation for mechanisms of SE in mediating airway remodeling.

## A394 The Effects of Probiotics and PparÎ^3^ on the Murine Model of Allergic Asthma

### Miao-Hsi Hsieh^1^, Jiu-Yao Wang^2^

#### ^1^The Institute of Basic Medical Sciences of National Cheng Kung University Taiwan; ^2^National Cheng Kung University Hospital

##### **Correspondence:** Miao-Hsi Hsieh – The Institute of Basic Medical Sciences of National Cheng Kung University, Taiwan

Probiotics are normal inhabitants in the gastrointestinal tracts of man and are widely considered to exert a number of beneficial roles including immunomodulation, interference with enteric pathogens, and maintenance of a healthy intestinal microflora. In recent years, studies of probiotics have also confirmed their extra-intestinal effects, particularly for the prevention of allergic diseases. However, the anti-allergy mechanism of probiotics is still unclear. In the first part of this study, we found that continuous feeding of *Lactobacillus gasseri* (*L. gasseri*) 10^7^ CFU/200 ml or 10^9^ CFU/200 ml for 4 weeks in Der p-sensitized and challenged mice could prevent allergen-induced airway inflammation. There were also significant changes of airway hypersensitivity, T_H_1 and T_H_2 cytokine patterns, lymphocyte proliferations and immunoglobulin production between *L. gasseri* -treated and non-treated mice. In the second part of study, we applied microarray analysis of the lung draining lymph nodes and mesenteric lymph node of mice to detect genes expression signal pathways and genetic profiling of immunological tolerance induced by *L. gasseri* that plays an essential role of in the prevention and therapeutic on allergic asthma. We found that there was significantly decrease of inflammatory and chemokines genes expression and increased of carbohydrate and lipid metabolism genes expression in the *L. gasseri*-treated mice as compared to non-treated sensitized and challenged mice. Thirdly, we have picked up one candidate targeted gene, PPARγ (peroxisome proliferator-activated receptor γ), to study the beneficial effect of probiotics on the allergic induced airway inflammation. Previously, it has been reported that PPARγ is a member of the nuclear hormone receptor family that not only is prominently involved in adipogenesis and metabolic regulation but also exerts pleiotropic anti-inflammatory effects in the lung, we hypothesized that PPARγ may play an important role in allergen-induced airway inflammation. The allergen-sensitized effect on murine model of asthma was applied in PPARγ P456L mutant mice by evaluating AHR, total numbers of inflammatory cells and cytokines secretion in bronchoalveolar fluid (BALF), and lung inflammation after mite allergen sensitization and challenge. Moreover, probiotics treatments PPARγ P456L mutant mice and wide type mice were administrated in allergen-sensitized mice. In summary, our results showed that PPARγ play important role in the inhibitory effect of allergen-induced airway inflammation in mice. And the anti-allergic effect on *L. gasseri* may through activation of PPARγ to alleviate airway inflammation in allergen-sensitized murine model of asthma.

## A395 Adult Patients’ Views on the Design of Adrenaline Autoinjectors

### Helen Smith, Clare Brown, Christina Jones, Mark Davies

#### Brighton and Sussex Medical School, UK

##### **Correspondence:** Helen Smith – Brighton and Sussex Medical School, UK

**Background:** Adrenaline autoinjectors (AAI) are key in the immediate or out of hospital management of anaphylaxis. Many studies have focused on poor utilization by patients. However papers addressing the ideal features of AAIs are neglectful of patients’ views and preferences.

**Objective:** To explore the views of adult patients on AAI design.

**Methods:** Thematic analysis of semi-structured interviews with 30 adult patients prescribed AAIs. Discussion was facilitated by a variety of AAI models (both commercially available and prototypes).

**Results:** All participants spoke spontaneously about the size of the device, they wanted it to be portable without needing a bag. Many favoured a smaller device, but others were tolerant of bulky designs and the inconvenience associated, because of the *‘lifesaving*’ characteristics of their AAI. The increased size of some recent products on the market were not welcomed. However significantly smaller designs were not necessarly perceived as preferable because of the potential difficulties of locating them in an emergency. Some argued for greater visibility achieved by bright colouration, others found bright colours ‘*frightening*’ or attracting unwanted attention to the fact that they carried and AAI. Grey was considered an undesirable colour (*‘Grey is quite depressing’*). Patients wanted instructions that were brief, simple and made full use of illustrations. Middle aged respondents commented on the need for adequate font size, while younger people recognised that if without their reading glasses bystanders assistance could be impaired through existing presentation styles. Integrated instructions on devices, that were intuitive to use, and ‘*not fiddly*’ needed to be prioritised. Consistency was requested; it was recognised that the different ways of highlighting the safety cap on some devices and the needle end on others was potentially confusing. A strong and resilient protective casing was thought to be mandatory, several respondents described casings deteriorating before the AAI expiry date. Several novel design features were suggested by the patients based on many years of experience.

**Conclusions:** Size and aesthetics of were two of the most important AAI design issues for patients. Greater involvement of patients in the development of new AAIs (patient centered design) could potentially increase the carriage of devices. This is important because however good the ballistic characteristics are of any device, the patient cannot benefit from this technology unless the AAI is carried, and utilised when needed.

## A396 CCL22 miRNA modulated Th1 responses and induced therapeutic effects on OVA-induced mouse model of asthma

### Won Suck Yoon

#### Korea University, South Korea

**Background:** Allergic asthma is a chronic pulmonary disease characterized by a Th2 inflammatory response. Th-2-biased immune responses are known to play a key role in the pathogenesis of allergic asthma. In particular, the macrophage derived chemokine CCL22 is directly implicated in Th-2-associated inflammatory reactions. In this study, we investigated the immune modulation using CCL22 miRNA would be induced therapeutic effects on ovalbumin-sensitized and -challenged asthmatic mice.

**Methods:** The recombinant strain of Salmonella typhimurium expressing CCL22 miRNA (ST-miRCCL22) was prepared for in vivo knockdown of CCL22. Using an ovalbumin-induced asthma model, mice were sensitized and challenged, and then treated with ST-miRCCL22.

**Results:** We constructed a recombinant strain of Salmonella typhimurium expressing CCL22 miRNA (ST-miRCCL22) for the in vivo knockdown of CCL22. The treatment of mice with established allergy led to attenuation of eosinophilia, Th2 cytokines and airway hyperresponsiveness. ST-miRCCL22 treatment also induced an increase in OVA-specific IFN-γ levels and in the frequency of lung inflammatory monocytes.

**Conclusions:** In our research, the CCL22 microRNA was treated to modulate imbalances in the disease. The CCL22 miRNA reduced Th1 immune responses and induced therapeutic effects on OVA-Induced mouse model of asthma. These results suggest that CCL22 miRNA could potentially be used as an effective therapeutic agent for treating asthma.

## A397 Clinical, Histological, and Skin Microbiome Characteristics of Head and Neck Dermatitis in Atopic Dermatitis

### Hemin Lee^1^, Howard Chu^1^, Jungsoo Lee^1^, Jung U Shin^1^, Chang Ook Park^1^, Kwang Hoon Lee^2^, Kwang Hoon Lee^1^, Seo Hyeong Kim^1^, Seo Hyeong Kim^2^, Ji Yeon Noh^1^, Ji Hye Kim^2^, Ji Hye Kim^1^

#### ^1^Yonsei University College of Medicine, Department of Dermatology; ^2^Brain Korea 21 PLUS Project for Medical Science, Yonsei University College of Medicine, South Korea

##### **Correspondence:** Hemin Lee – Yonsei University College of Medicine, Department of Dermatology, South Korea

**Background**

Head and neck dermatitis (HND) is a unique subtype of atopic dermatitis (AD) which commonly manifests in late adolescence or adulthood. HND is often refractory to therapy and affects patient’s quality of life greatly. However, presence of HND is often underappreciated in clinics and detailed studies on its characteristics are limited.

**Methods**

This study sought to analyze clinical, histological characteristics of HND patients. Further, as there is a growing number of AD studies focusing on the role of skin microbiome in the disease pathogenesis, microbiome analysis of HND patients in comparison to non-HND (N-HND) AD and normal (NL) subjects was performed.

**Results**

The results showed HND patients comprising approximately 2.4% of AD patients in an outpatient clinic setting. Compared to N-HND patients, HND initially presented with more severe disease status and elevated serum total IgE and serum specific IgE eactivity to *dermatophagoides farinae*. Histologically, HND specimen showed thickened epidermis with increased vascular components and dermal inflammatory infiltrates staining positive to pro-inflammatory cytokines and chemokines. Lastly, the microbiome analysis of HND in comparison to N-HND AD and NL subjects revealed presence of diverse community present in the skin.

**Conclusions**

These findings reaffirm that HND is a clinical subtype of AD that needs distinction from classical AD. In the future, further investigation of skin microbiome and inflammatory factors involved in vasculatures of facial lesions in AD will lead to development of potential therapeutic targets.

## A398 MicroRNA-432 modulates Th1 responses and induced therapeutic effects in atopic like murine model

### Won Suck Yoon

#### Korea University South Korea

**Background:** MicroRNAs (miRNAs) modulate gene transcription in response to environmental stressors and other stimuli. A role for miRNAs in inflammation and immunity has been demonstrated and further evidence suggests that miRNAs also play a role in atopic dermatitis. In this study, we hypothesized the immune suppression using miR-432 would be induced therapeutic effects on atopic diseases.

**Methods:** The Ig-E, Interleukin-4 (IL-4), CCL22 and interferon-g (IFNg) were examined after treatments with miRNAs in murine atopic model. In addition, atopic patient’s blood samples were collected and examined for miRNA expression.

**Results:** miR-432 reduced CCL22 chemokine gene in activated lymphocytes. In mice with a cutaneous disease similar to atopic dermatitis, Interleukin-4 was inhibited and interferon-γ was induced after treatments with miR-432. Furthermore, miR-432 levels were suppressed in the atopic patients.

**Conclusions:** miR-432 suppressed Th1 immune responses and induced therapeutic effects in atopic mouse model.

## A399 Case Report of Near-Fatal Asthma Due to Snail Allergy in a House Dust Mite-Allergic Adult

### Jean-Pierre L’huillier^4^, Jean-Eric Autegarden^1^, Catherine Bertrand^2^, Dominique Tardy^3^

#### ^1^Hôpital Tenon; ^2^Hôpital Henri Mondor; ^3^Hôpital Saint-Camille; ^4^Cabinet De Pneumologie France

##### **Correspondence:** Jean-Pierre L’huillier – Cabinet De Pneumologie, France

**A) Background:** Cross-reaction between food and inhalant allergens usually show mild symptoms. However, severe asthma symptoms, exercise-induced anaphylaxis, urticaria and "recall urticaria" at the site of immunotherapy injection may occur after ingestion of snails by patients allergic to house dust mites (1).

**B) Methods:** We report the case of a 23-years old woman who suffered from an acute severe asthma attack complicated by asphyxic evolution leading to cardiopulmonary arrest. Cardiopulmonary resuscitation was successful, subcutaneous emphysema occurred, left pneumothorax was treated by chest tube. The clinical course was benign, and the patient returned from hospital to home after a few days with no complication. Medical history revealed that she had had asthma for 10 years, with allergy to inhaled allergens (grass pollen, house dust mites, pets dander). She had been treated 9 years earlier by subcutaneous specific immunotherapy against house dust mites for 2 years. Medications taken included albuterol, beclomethasone, salmeterol and montelukast. She ate Norway lobsters and snails 5 hours before the beginning of acute severe asthma attack and had sexual intercourse with no condom 3 hours before.

**C) Results:** Skin prick tests (SPT, diameters of the wheals and flares in mm) and serum specific Ig E (SSIg E, Pharmacia Unicap 1000) confirmed sensitization against snails (SPT = 7/30; SSIg E 7,37 kUI/l) but not against Norway lobsters (SPT = 0/0; SSIg E < 0,35 kUI/l) nor human seminal fluid (SPT = 0/0; SSIg E < 0,35 kUI/l). No Ig E sensitization was later found against prawn nor house dust mites tropomyosin (rPen a 1 < 0,35 kUI/l, Pharmacia Unicap 1000 ; rDer p 10 < 0,10 kUI/l, Phadia Immunocap 1000). No severe asthma attack did occur thirteen years after beginning of eviction diet and continuation of asthma treatment. More than 100 cases of allergic symptoms after consumption of snails among patients allergic to house dust mites were published. These allergic symptoms may occur after the first ever ingestion of snails and may be life-threatening. They begin usually 5 to 60 minutes and rarely 1 to 5 hours after the ingestion of snails (1, 2). Cross-sensitivity between snails and house dust mites is probably due to common epitopes as haemocyanin.

**D) Conclusions:** Allergic patients to house dust mites should be informed about the possibility of severe cross-allergy symptoms after eating snails.

References

1 - van Ree R *et al.* Asthma after consumption of snails in house-dust-mite-allergic patients: a case of IgE cross-reactivity. Allergy. 1996 Jun; 51(6): 387–93.

2 - De Maat-Bleeker F *et al.* Vineyard snail allergy possibly induced by sensitization to house-dust mite (Dermatophagoides pteronyssinus). Allergy. 1995 May;50(5):438–40

## A400 Relationship Between Gut Microbiota in the First 3 Months of Life and Infant Immune Function at Age 12 Months

### Intan Hakimah Ismail^3^, Mimi Tang^1^, Paul Licciardi^1^, Frances Oppedisano^1^, Robert Boyle^2^, Roy Robins-Browne^1^

#### ^1^Murdoch Childrens Research Institute, Australia; ^2^Imperial College London, UK; ^3^Universiti Putra Malaysia (UPM), Malaysia

##### **Correspondence:** Intan Hakimah Ismail – Universiti Putra Malaysia (UPM), Malaysia

**Background:** Distinctive pattern and diversity in early intestinal colonisation are shown to influence immune maturation and potentially development of allergic disease. We recently demonstrated that prenatal supplementation with *Lactobacillus rhamnosus* GG (LGG) during late pregnancy can influence infant gut colonisation by particular *Bifidobacterium* species directing towards healthy infant-type microbiota. We investigated whether this early-life gut colonisation with *Bifidobacterium* species is associates with systemic immune responses at 12 months of age.

**Methods:** Faecal samples were collected from infants during the first 3 months of life. Bacterial DNA was extracted from the faecal samples and *Bifidobacterium longum, B. lactis, B. breve, B. angulatum, B adolescentis, and B. catenulatum* were detected by real time PCR. Infants’ peripheral blood mononuclear cells at 12 months were stimulated with ovalbumin (OVA), heat-killed LGG (HKL) (the probiotic used in the original study), tetanus toxoid (TT), anti-CD3 or without stimulus. Cells were analysed by flow cytometry for markers of dendritic cells phenotype and regulatory T cell (Treg) numbers. Culture supernatants were analysed for IL-4, IL-6, IL-10, IL-13, IFN-γ and TNF-α by multiplex ELISA, while TGF-β1 and IL-12p40 were measured using ELISA.

**Results:** Colonisation with *B. longum* at day 7 of life was associated with significantly higher (p < 0.01) levels of Th1 cytokine (IFN-γ) and pro-inflammatory cytokines (IL-6 and TNF-α) and increased (p < 0.05) secretions of Th2 (IL-13) and regulatory cytokine (IL-10) in infants at 12 months. However, colonisation with *B. adolescentis* at day 3 was associated with higher secretion of IL-4 cytokine. A significantly increased numbers of Treg were observed in infants colonised with *B. adolescentis* at 7 days of age.

**Conclusions:** Colonisation with specific *Bifidobacterium* species in early life can influence cellular immune function, namely cytokine profiles and Treg later at 12 months. This suggests that probiotic treatment during pregnancy may modulate infant immune function as late as 12 months of age, feasibly mediated by modulation of infant microbiota. However, the immune mechanism that might protect against allergic disease is still unclear.

## A401 A Pediatric Case of Food-Dependent Exercise-Induced Anaphylaxis Due to Spice Allergy

### Hisako Yagi, Harumi Koyama, Yutaka Nishida, Takumi Takizawa, Hirokazu Arakawa

#### Gunma University Graduate School of Medicine, Japan

##### **Correspondence:** Hisako Yagi – Gunma University Graduate School of Medicine, Japan

**Background:** Curry spice allergy in children is extremely rare in Japan. In addition, food-dependent exercise-induced anaphylaxis (FEIAn) as a manifestation of spice allergy against curry powder is quite uncommon.

**Methods:** We report a case of FEIAn due to curry spice allergy in a 14-year-old boy. The boy had a history of several episodes of exercise-induced anaphylaxis since the age of 12 years, which were suspected to be FEIAn. He developed pollinosis in spring and autumn, and had increased levels of specific IgE antibodies against many different kinds of allergens such as food and pollens, including, celery, white birch, and mugwort. Among several different foods consumed before the last three episodes of anaphylaxis, we found that curry powder was the common ingredient in all of them. Since the curry powder did not induce symptoms without exercise, we suspected FEIAn caused by curry spices.

**Results:** The results of the exercise challenge test conducted after ingestion of curry were positive and accompanied by skin flare, itching, urticaria, and bulbar conjunctival hyperemia. We recorded a 10.0% decrease in forced vital capacity, and a 13.5% decrease in forced expiratory volume in 1 s on respiratory function testing. The patient was diagnosed as having FEIAn in response to curry powder. The patient was found to be sensitized to coriander, a curry powder ingredient, on ImmnoCAP. Sensitization to several other spices was also detected with skin-prick testing. The patient was instructed to refrain from exercise for 2 h after ingestion of curry powder and has not shown any symptoms since then.

**Conclusion:** Based on history and investigation results, we suspected that the celery-birch-mugwort-spice syndrome was caused by IgE cross-reactivity between pollens and spices.

## A402 Correlations Between Objective Severity Score and Each of the Subjective Severity Intensity in Atopic Dermatitis

### Hong Kyu Kang^1^, Hemin Lee^1^, Jungsoo Lee^1^, Jung U Shin^1^, Kwang Hoon Lee^2^, Kwang Hoon Lee^1^, Howard Chu^1^, Chang Ook Park^1^

#### ^1^Yonsei University College of Medicine, Department of Dermatology, South Korea; ^2^Brain Korea 21 PLUS Project for Medical Science, Yonsei University College of Medicine

##### **Correspondence:** Hong Kyu Kang – Yonsei University College of Medicine, Department of Dermatology, South Korea

**Background:** Atopic dermatitis (AD) is a chronic, relapsing, inflammatory skin disorder characterized by multifactorial pathophysiologic aspects. Pruritus and sleep disturbance are cardinal symptom of atopic dermatitis. Several scoring systems are available, Eczema Area and Severity Index (EASI) is a tool commonly used to measure the severity of atopic dermatitis. Correlation between Visual analogue scale (VAS) of pruritus and loss of sleep (LOS) and the EASI score is not fully investigated yet.

**Objective:** This study was designed to evaluate the association of pruritus and sleep disturbance in atopic dermatitis with objective disease severity and laboratory parameters.

**Methods:** We assessed EASI score as an objective disease severity, VAS of pruritus and LOS (0-10 point) as a severity of pruritus and sleep disturbance in 1877 AD patients at our dermatology clinic from 2007 to 2013. We also measured Eosinophilic count, total IgE and specific IgE in peripheral blood. Correlation of pruriruts and sleep disturbance with disease severity and laboratory parameters were determined by *Pearson’s* correleation analysis using SPSS version 21.0.

**Results:** The mean VAS of pruritus and LOS were 5.93±2.53 (Mean ± SD) and 4.67±3.00. The mean EASI score was 14.0±12.1. Our study revealed that the EASI score showed weak statistical significance with VAS of pruritus and LOS. (EASI vs VAS of pruritus; r = 0.31, EASI vs VAS of LOS; r = 0.36) The eosinophil count revealed weak statistical significance with VAS of pruritus and LOS. (VAS of pruritus; r = 0.10, VAS of LOS; r = 0.23) The total IgE level showed no statistical significance with VAS of pruritus and LOS. Specific IgE titer to *Dermatophagoides farinae* (D2) showed weak statistical significance with VAS of pruritus and LOS. (VAS of pruritus; r = 0.10, VAS of LOS; r = 0.14).

**Conclusions:** Our results demonstrated that EASI score weakly reflect the subjective severity (pruritus and LOS). Since severe pruritus and LOS can affect the quality of life of atopic dermtaittis patients and they are not strongly correlated with disease severity measured by dermatologists, dermatologists should aware the subjective symptoms from patients and consider to manage the subjective symptoms together with objective symptoms.

**References**

Chrostowska-Plak D, Salomon J, Reich A, Szepietowski JC. Clinical aspects of itch in adult atopic dermatitis patients. Acta Derm Venereol. 2009;89:379–383.

Hanifin JM. Diagnostic criteria for atopic dermatitis: consider the context. Arch Dermatol. 1999;135:1551.

## A403 Barrier Related Gene Mutations in Atopic Dermatitis

### Na Young Yoon^5^, Hyeyoung Lee^1^, Seong Jun Seo^2^, Eunhee Choi^3^, Hye-Young Wang^4^, Minyoung Jung^5^, Eung Ho Choi^5^, Dong Hye Kim^5^

#### ^1^College of Health Sciences; ^2^Chung-Ang University Hospital; ^3^Institute of Lifestyle Medicine; ^4^M&d, Inc; ^5^Yonsei University Wonju College of Medicine, South Korea

##### **Correspondence:** Na Young Yoon – Yonsei University Wonju College of Medicine, South Korea

**Background:** Hereditary factors of atopic dermatitis(AD) have been emphasized recently. AD-related gene mutations vary significantly across ethnicities. We tried to find mutations in *FLG, SPINK5* and *KLK7* genes from Korean AD patients and we aimed to develop a reverse blot hybridization assay(REBA) to apply to AD-related genes for the first time.

**Methods:** We divided the AD subjects into moderate to severe AD and mild AD groups and also divided them into extrinsic and intrinsic AD groups. We checked on gene mutations using the REBA in AD and non-atopy control subjects.

**Results:** The mutant type(MT) of *KLK7* was significantly more frequent in AD subjects than in control and higher in the moderate to severe group compared to the mild group. The MT frequency was not different between the intrinsic and extrinsic AD groups. In the *SPINK5*mutation, AD subjects more frequently had the mixed type of 603-49A>T(Glu335Val), the MT of 1188T>C(His396His) and 2475G>T(Glu825Asp) compared to control, but there was no difference between intrinsic and extrinsic AD. Among AD subjects, the moderate to severe AD group had more gene mutations compared to the mild group.

**Conclusion:** We found a correlation between the *KLK7* mutation, the mutations in 1188T>C and 2475G>T, and 603-49A>T of *SPINK5*and AD which have not been reported in Asians including Koreans. Above all things, we verified that the REBA can be applied to detect multiple barrier-related gene mutations in AD easily, simply, and accurately.

## A404 Clinical Utility of Basophil Activation Test (BAT) in the Diagnosis of Drug Induced Anaphylaxis

### Joo-Hee Kim^1^, Young-Sook Jang^1^, Jeong-Hee Choi^2^, Sunghoon Park^1^, Young Il Hwang^1^, Seung Hun Jang^1^, Ki-Suck Jung^1^

#### ^1^Hallym University Sacred Heart Hospital, South Korea; ^2^Hallym University Dongtan Sacred Heart Hospital

##### **Correspondence:** Joo-Hee Kim – Hallym University Sacred Heart Hospital, South Korea

**Background:** Diagnostic work-up in patients suffering life-threatening drug anaphylaxis is difficult in clinical practice owing to the low sensitivity of the laboratory tests and the risk of anaphylaxis using in vivo tests. Flow cytometry-assisted basophil activation test (BAT) is suggested a safe diagnostic method, although it is more expensive and technically challenging compared to conventional *in vitro* or *in vivo* tests. We sought to evaluate the diagnostic utility of this testing in clinical practice.

**Method:** Nineteen patients with a drug-induced anaphylaxis were recruited. Basophil activation test, skin tests, and measurement of commercially available specific IgE to drugs were performed for diagnostic evaluation. A stimulation index≥2 and an absolute activated basophil percentage≥5 were considered positive response to BAT.

**Results:** All patients met the FAAN/NIAID criteria for anaphylaxis. Causality assessment using the WHO-UMC classified them into the categories ‘certain’ or ‘probable’. Male to female ratio was 1:1.1 and the mean age was 46.0 ± 12.0 yrs. Five patients presented severe anaphyalxis such as hypotension, hypoxia or loss of consciousness, and the others were moderate severity. The involved drugs were cephalosporin antibiotics in 9 patients, eperisone 2, ranitidine 3, and aminoglycoside, glimepiride, humalog insulin, paclitaxel, tradamol, propofol in one patient each. BAT using CD 63 marker was positive in 12 (63.2%), and negative in 7 patients, whereas BAT using CD203c was positive in 9 (47.4%) and negative in 10 patients. When both markers applied, 14 patients (70.0%) showed positive to BAT. Skin test was positive in 8 (47.0%), negative in 9, and nonapplicable in two patients.

**Conclusion:** The BAT proves to be a useful diagnostic tool for drug-induced anaphyalxis. In addition, this test can identify the causative drug in patients with negative skin test or unavailable to sIgE measurement.

## A405 Feeding Shapes the Colonization of Gut Microbiota and Associated with Total IgE in Infant

### Mi-Jin Kang^7^, Dongin Suh^1^, Eun Lee^2^, Kil Yong Choi^3^, Young-Ho Jung^4^, Song-I Yang^5^, Bong-Soo Kim^6^, Ha-Jung Kim^7^, Juneyoung Koh^8^, Hyun-Jin Kim^8^, Kangmo Ahn^9^, Youn Ho Shin^10^, Hyun-Ju Cho^5^, Byoung-Ju Kim^11^, Young-Ho Kim^2^, Yean Jung

#### ^1^Seoul National University Hospital, South Korea; ^2^Department of Pediatrics, Childhood Asthma Atopy Center, Environmental Health Center, Asan Medical Center, University of Ulsan College of Medicine, South Korea; ^3^Asan Institute for Life Science, University of Ulsan College of Medicine, South Korea; ^4^Bundang CHA Medical Center, CHA University School of Medicine, South Korea; ^5^Asan Medical Center, South Korea; ^6^Department of Life Sciences, Hallym University, South Korea; ^7^Asan Institute for Life Science, Asan Medical Center, South Korea; ^8^Asan Medical Center, University of Ulsan College of Medicine, South Korea; ^9^Samsung, South Korea; ^10^CHA University School of Medicine, South Korea; ^11^University of Cincinnati College of Medicine, USA

##### **Correspondence:** Mi-Jin Kang – Asan Institute for Life Science, Asan Medical Center, South Korea

**Background:** Bacterial colonization of the infant gut begins at birth and the gut microbiota in infant was unstable. The initial gut microbiota plays an important role in the development of immnue system. Disruption of the gut microbiota has been linked to the development of allergic diseases. Especially, feeding method has a significant impact on allergic diseases.

**Objective:** We investigated the composition of gut microbiota according to feeding method and the association with serum IgE in infants.

**Materials and methods:** Fecal samples were collected at 6 month from 47 infants in the COCOA birth cohort. Microbiota characterization was performed by using 16S rRNA shotgun sequencing.

**Results:** The species richness (alpha-diversity) was not different according to feeding method. The significant higher level of *Firmicutes* and lower level of *Actinobacteria* detected in mixed milk (breast and formula milk) feeding infants than breast milk feeding infants at phylum level. The proportion of *Bifidobacterium* was significantly reduced, while *Clostridium_g4*, *Clostridium*, and *Clostridium_g6* were highly enriched in mixed milk feeding infant than breast milk feeding infant at genus level. And the level of *Escherichia*positively correlated with the total serum IgE at age 1 year in mixed milk feeding infant, but not in breast milk feeding infant.

**Conclusion:** Feeding method is not affect to the diversity of gut microbiota. However it shapes the composition of specific bacteria and *Escherichia*affect to the development of atopy.

This research was supported by Basic Science Research Program through the National Research Foundation of Korea(NRF) funded by the Ministry of Science, ICT and future Planning(NRF-2014R1A2A1A10050687).

## A406 CD8^+^ T Cell-Intrinsic Smad4 Suppresses Th2 Responses in the Pathogenesis of Contact Hypersensitivity

### Mizuko Mamura^1,2^, Jeong-Hwan Yoon^1,2,3^, Susumu Nakae^4^, Inkyu Lee^2^, Isao Matsumoto^5^, Takayuki Sumida^5^, Jin Soo Han^6^, Katsuko Sudo^7^, Ji Hyeon Ju^8^

#### ^1^Department of Molecular Pathology, Tokyo Medical University, Japan; ^2^Department of Internal Medicine, Kyungpook National University School of Medicine, South Korea; ^3^Department of Experimental Pathology, Graduate School of Comprehensive Human Science, University of Tsukuba, Japan; ^4^The Institute of Medical Science, Japan; ^5^Division of Clinical Immunology, Major of Advanced Biomedical Applications, Graduate School of Comprehensive Human Science, University of Tsukuba, Japan; ^6^Institute for the 3Rs, Department of Laboratory Animal Medicine, College of Veterinary Medicine, Konkuk University; ^7^Animal Research Center, Tokyo Medical University, Japan; ^8^Department of Internal Medicine, Catholic University, South Korea

##### **Correspondence:** Mizuko Mamura – Department of Molecular Pathology, Tokyo Medical University, Japan

**Background:** Transforming growth factor-β (TGF-β) plays crucial regulatory roles in T cell-mediated contact hypersensitivity (CHS). Canonical TGF-β signaling pathway is mediated through TGF-β-specific receptor-regulated Smads (R-Smads) and the common Smad, Smad4. However, precise signaling mechanisms whereby TGF-β regulates T cells in CHS are not fully understood.

**Objectives:** We sought to determine the mechanisms how TGF-β signaling through Smad4 regulates the pathogenic effector T cell subsets in CHS.

**Methods:** We used Cd4Cre-loxp system to delete Smad4 in T cell-specific manner (*Cd4Cre*;*Smad4*^*fl/fl,+/+*^). *Cd4Cre*;*Smad4*^*fl/fl,+/+*^ mice were immunized and sensitized by 1-fluoro-2,4-dinitrobenzene (DNFB).

**Results:** We found that T cell-specific deletion of Smad4 exacerbated DNFB-induced CHS with significant expansion and infiltration of CD8^+^ T cells in the draining lymph nodes and the skin lesions. Smad4 deficient CD8^+^ T cells upregulated Th2 differentiation, regardless of Smad4 genotypes of CD4^+^ T cells. Smad4 in combination with Smad3 suppressed the expression of a T-box transcription factor, Eomesodermin (Eomes) in CD8^+^ T cells. Expression of Eomes and the cytotoxic molecules in CD8^+^ T cells at the early phase of sensitization was significantly upregulated in *Cd4Cre*;*Smad4*^*fl/fl*^ mice compared with control *Cd4Cre*;*Smad4*^*+/+*^ mice. Cytolytic molecules in CD8^+^ T cells upregulated by Smad4 deletion induced Th1 cell apoptosis, which resulted in increased Th2.

**Conclusions:** These data highlight CD8^+^ T cell-intrinsic Smad4 as the crucial regulator of effector CD4^+^ T cell subsets in CHS.

## A407 Immune-Modulatory Genomic Properties Differentiate Gut Microbiotas of Infants with and without Eczema

### Gaik Chin Yap^4^, Wen Tso Liu^1^, Seungdae Oh^1^, Pei Ying Hong^2^, Chiung Hui Huang^3,4^, Marion Aw^3,4^, Lynette Shek^3,4^, Bee Wah Lee^3,4^

#### ^1^University of Illinois at Urbana-Champaign, USA; ^2^King Abdullah University of Science and Technology, Saudi Arabia; ^3^National University Health System, Singapore; ^4^National University of Singapore, Singapore

##### **Correspondence:** Gaik Chin Yap – National University Health System, Singapore

**Background:** The gastrointestinal tract is the primary site of interaction between the host immune system and microorganisms. Studies have suggested that selective microbial targets may influence the development of the allergic diseases. But the difference in functional gene composition remains unknown. We aim to assess the structural and functional gene composition of stool microbiota of infants with eczema and their matched (for age, gender, mode of delivery, feeding) controls at the age of 1 month.

**Methods:** Twelve children with eczema and their controls were selected from the placebo arm of a birth cohort of at-risk infants participating in a randomized double-blind trial on the protective effects of supplemental probiotics in early life on allergic outcomes. The four were caesarean delivery followed by formula feeding (eczema = 2 and healthy control = 2) and the eight were vaginal delivery followed by partial breast feeding mixed with formula feeding (eczema = 4 and healthy control = 4). Bacterial genomic DNA were extracted from fecal samples and prepared for Illumina Miseq and Hiseq sequencing. Data analysis such as sequence quality check, contigs assembly and gene annotation were carried out for the DNA sequences obtained from Miseq and Hiseq sequencing.

**Results:** Phylogenetic analysis of metagenomic sequences revealed that four phyla dominated both microbial communities: *Proteobacteria* (54% and 63% for healthy and eczema communities, respectively), *Firmicutes* (26% and 18%), *Actinobacteria* (13% and 8%), *Bacteroidetes* (7% and 8%). Comparative metagenomic analysis showed that immune-regulatory TCAAGCTTGA motifs were significantly enriched in healthy communities, many of which were encoded by *Bifidobacterium* (38% of the total motifs in the healthy communities). Draft genomes of five *Bifidobacterium* species (*B. longum*, *B. bifidum*, *B. breve*, *B. dentium,* and *B. pseudocatenulatum* ) were recovered from metagenomic datasets. The *B. longum* BFN-121-2 genome encoded more TCAAGCTTGA motifs (4.2 copies per 1 million genome sequence) than other *Bifidobacterium* genomes and was significantly overrepresented (*P* < 0.05) in the healthy communities.

**Conclusions:** Our results report distinct immune-modulatory genomic properties of gut microbiotas in healthy infants as compared to children with eczema and provide new insights into potential roles of gut microbiotas in affecting human immune homeostasis.

## A408 The Effect of Medication in OSA Patients with Allergic Rhinitis

### Young Seok Byun^1^, Sung Wan Kim^1^, Tae Kyung Koh^1^, Joong-Saeng Jo^1^, Kun Hee Lee^2^, Chul Kwon^1^, Sung-Hwa Dong^1^

#### ^1^Kyung Hee Medical Center, South Korea; ^2^Kyung Hee University Hospital at Gangdong

##### **Correspondence:** Young Seok Byun – Kyung Hee Medical Center, South Korea

**Objective**

Allergic rhinitis occurs at 10-40% in the world’s population and it cause runny nose, sneezing, itching and qualitative degraded sleep disorder which are causing the failure of social life. Allergic rhinitis can be associated with patient of obstructive sleep apnea. The aim of this study was to investigate the impact of allergic rhinitis on obstructive sleep apnea and the change of the symptoms of obstructive sleep apnea after allergic rhinitis treatment.

**Method**

We examined patients who diagnosed with obstructive sleep apnea using polysomnography in our sleep clinic. All the patient was examined by allergy skin testing and diagnosed with allergic rhinitis. Patients were group of OSA with AR. We did Allergic rhinitis medical treatment for 2 weeks before OSA treatment.

We did the survey of Visual analogue scale, Epworth Sleepiness Scale, Chalder Fatigue Scale, Daily Hassles Scale, Conner-Davidson Resilience Scale, Rhinoconjunctivitis Quality of Life Questionnaire before and after Allergic rhinitis treatment.

**Results**

OSA-AR group was 14 pateints. Age ranged from 17 to 66 years old and mean age 27. prior treatment, OSA-AR group score was less than average in VAS(sleep time, wake time), ESS, CFS, DHS, CD-RISC, RQLQ examination and TNSS, which associated with rhinitis in RQLQ, showed less than average except nasal obstruction in OSA-AR. VAS (sleep time, wake time) and RQLQ in patients who underwent 2weeks medical treatment showed statistically significant difference after treatment. (sleep time P = 0.001, wake time P = 0.000, RQLQ P=.011) The average scores of ESS, CFS, DHS, CD-RISC, RQLQ, TNSS was lower but no significant difference after treatment than before treatment.

**Conclusion**

We could find out Allergic rhinitis treatment contributes to improve subjective symptoms and QOL of patients in OSA-AR treatment patients. But considering lower number of subject, further investigation should be performed.

## A409 A Case of Generalized Pustular Psoriasis Mimicking Acute Generalized Exanthematous Pustulosis

### Myung Shin Kim^1^, Chansun Park^2^

#### ^1^Soonchunhyang University Gumi Hospital South Korea; ^2^Haeundae Paik Hospital, Inje University College of Medicine

##### **Correspondence:** Myung Shin Kim – Soonchunhyang University Gumi Hospital, South Korea

Differentiating between acute generalized exanthematous pustulosis (AGEP) and generalized pustular psoriasis (GPP) can be extremely difficult, as both present as erythematous pustular eruptions. A 60-year-old woman presented at the emergency department with generalized erythematous pustular eruption, generalized edema and fever. She had treated with drug eruption by herbal remedy in dermatologic department for 3 weeks. One week before visiting ED, she received dapsone and zaltoprofen from another hospital. Body temperature measured as 38.7 °C. Blood neutrophil was 85.3 % of total leukocytes. Based on the clinical and laboratory findings, she was diagnosed as AGEP by dapsone or NSAIDs. All the medications were discontinued. After treatment with systemic steroid for 3 days, fever and skin lesions were improving. However, new pustular lesions were developing immediately after the dose reduction of steroid and rapidly aggravated. The results of skin biopsy were compatible with GPP rather than AGEP. Acitretin was started and steroid was slowly tapered. The pustular lesion and scaling had improved. We report a patient with a pustular eruption initially diagnosed as AGEP, with the diagnosis changed to GPP. This case highlights the importance of differential diagnoses in order to treat the condition appropriately.

## A410 Anaphylaxis Caused By Gummy Jelly Ingestion: A Case Report

### Han Seok Cho, Min-Ju Kim, Min Ji Kim, Young Ok Park, Hye Yeong Lee, Hee Seong Kim, Eun Lee, Hyun-Ju Cho, Jinho Yu, Soo-Jong Hong, Keum Hee Hwang

#### Asan Medical Center, South Korea

##### **Correspondence:** Han Seok Cho – Asan Medical Center, South Korea

Gelatin allergy has been well described in type 1 hypersensitivity reactions to vaccines, but only a few case reports have been published about anaphylaxis associated with food.

We report a 5 years old boy that developed anaphylaxis after eating gummy bears. He was referred for evaluation after 2 episodes of anaphylaxis after ingestion of gummy products. The first episode occurred within 10 minutes of consuming more than 1 package of Haribo Gold Bear Candy with development of diffuse urticaria and generalized rash, angioedema, and pruritus with wheezing. Approximately 2 months later, a second episode occurred after ingesting Mygummy fruit snack 1 package. Ten minutes after ingestion, he had generalized urticaria and erythema, which resolved after receiving antihistamine.

Prick to prick skin testing of gelatin-containing gums (Haribo and Mygummy) soaked in water and porcine gelatin were positive reactions. And additional intradermal testing with a 1:100 gelatin was strongly reactive. ImmunoCAP testing showed an increasd IgE level to bovine gelatin, at 4.50 kUA/L(Porcine gelatin in not available in Korea. Mammalian gelatins are well known that there is cross-reactivity).

Double-blind, placebo-controlled food challenge is the gold standard for the diagnosis of food allergy. However, this was not conducted in this patient, because he had already had several reactions to foods that contained gelatin, including anaphylactic reactions, and he had evidence of specific IgE to gelatin, skin testing and intradermal test.

The patent was advised to avoid all gelatin-containing food, medications, and vaccines.

## A411 Serum Folliculin As a Novel Biomarker for Asthma

### Jung-Hyun Kim^1^, You Sook Cho^1^, Sae-Hoon Kim^2^, Hyouk-Soo Kwon^1^, Mira Yoo^3^, Hyo-Jung Kim^1^, So-Young Park^1^, Bomi Shin^4^, So Young Park^1^, Bomi Seo^1^, Min-Gu Kim^1^, Hee-Bom Moon^1^, Jin-Ah Park^2^, Tae-Bum Kim^1^, Jaemoon Lee^1^

#### ^1^Asan Medical Center, University of Ulsan College of Medicine, South Korea; ^2^Harvard T.H. Chan School of Public Health, USA; ^3^Asan Institute for Life Sciences, South Korea; ^4^Asan Medical Center, South Korea

##### **Correspondence:** Jung-Hyun Kim – Asan Medical Center, University of Ulsan College of Medicine, South Korea

**Background**

Asthma is a chronic inflammatory disease, in which the airway progressively undergoes structural changes collectively termed as airway remodeling. Severe refractory bronchospasm elicits mechanical stress of bronchial wall and it is responsible for airway inflammation and remodeling. In this study, we planned to examine the relationship between asthmatic lung functions and the serum folliculin level. Folliculin is released from bronchial epithelial cells in response to compressive stress, mimicking bronchospasm.

**Materials and Methods**

Folliculin levels in serum were measured by ELISA in asthma patients (n=405) who visited the asthma center in a tertiary referral hospital from May 2005 to December 2014, and healthy controls (n=94) who visited the Health Examination Center in the same hospital. Folliculin levels were compared in two groups and we investigated if the level of folliculin is correlated with lung function and other clinical variables within the group of asthma. Patients whose folliculin level over 148pg/ml were defined as “high follicullin group” and we sought to find out clinical characteristics of the ‘high folliculin’ phenotype.

**Results**

The serum concentrations of folliculin were significantly higher in asthmatics (148.92± 240.71 vs 73.57± 78.34 pg/ml, p < 0.001) than in healthy controls. We found negative correlation between serum folliculin level and post bronchodilator FEV1 (R=-0.228, p = 0.00) and post bronchodilator FEV1/FVC (R=-0.155, p = 0.00). The high-folliculin group was older at onset of asthma (65.01±88.08 vs 97.12±121.88 months, P= 0.007), had heavier history of smoking (7.90 vs 13.08 pack-years, p= 0.011), presented more frequent exacerbations (0.36/3months vs 0.57/3months, p=0.041), and showed lower FEV1 % predicted (73.63 vs 66.65, p=0.00).

**Conclusions**

The results demonstrate that serum folliculin concentration is higher in asthmatics and is associated with lower lung function. Thus, follucilin can be a novel biomarker for asthma.

## A412 Corticosteroid Nasal Irrigations after Endoscopic Sinus Surgery in the Management of Chronic Rhinosinusitis with Asthma

### Jin Hyeok Jeong, Tae Wook Kang, Han Seok Yoo, Yong Hee Cho, Seok Hyun Cho, Kyung Rae Kim

#### Hanyang University College of Medicine, South Korea

##### **Correspondence:** Jin Hyeok Jeong – Hanyang University College of Medicine, South Korea

**Background:** In asthma patients, chronic rhinosinusitis with nasal polyp tend to have high recurrence rate after surgery, and gets better and worse along the clinical course of asthma. The oral steroid treatment is often used in these cases because of poor effect of local nasal steroid treatment. Side effect of long-term oral steroid is considered to be a serious concern for the physicians. The off-label use of budesonide nasal irrigation was introduced recently for post operative management of patients with chronic rhinosinusitis. The safety and effect of budesonide nasal irrigation is being accepted to many physicians. The objective of this study is to evaluate the efficacy of postoperative steroid irrigation in asthma patients.

**Methods:** Prospective study was done on 12 chronic rhinosinusitis patients with nasal polyp and asthma who used oral steroid treatment due to recurred or worsen disease. The 22-item Sinonasal Outcomes Test (SNOT-22) and Lund-Kennedy endoscopy scores were performed before nasal budesonide irrigation, 1, 2, 4, and 6 months after the irrigation.

**Results:** The subjects were of 3 male and 9 female patients and the mean age of the patients were 49.9±7.3. SNOT-22 score was 30.8±14.4 before irrigation followed by 17.8±16.6 after 1 month, 14.8±11.1 after 2 months, 14.9±10.9 after 4 months and 14.17±8.7 after 6 months of irrigation. SONT-22 scores were significantly improved with irrigation (p=0.03) Endoscopy score was 7.4±4.7 before the irrigation followed by 3.5±3.3 after 1 month, 1.5±1.8 after 2 months, 1.7±1.5 after 4 months and 2.2±2.7 after 6 months. The scores were significantly improved with irrigation (p<0.001).

**Conclusions:** Nasal irrigation with budesonide is considered to be an effective post operative treatment for chronic rhinosinusitis patients with asthma which recur frequently, reducing the use of oral steroid intake.

## A413 Capsaicin Injection in Neonatal Period Potentiates Intensity and Duration of Atopic Dermatitis of Rats

### Jue Seong Lee, Sun-Ho Kee, Sewon Kim, Young Yoo, Heung Sik Na, Seung Keun Back

#### Korea University, South Korea

##### **Correspondence:** Jue Seong Lee – Korea University, South Korea

**Background:** Atopic dermatitis (AD) is a chronically relapsing inflammatory skin disease, characterized by severe itching and dysfunction of skin homeostasis. Previously, new AD model was established through capsaicin injection to neonatal rats, which displayed itching behavior and skin inflammation from 3 weeks after injection.

**Methods:** Rats were injected with capsaicin and AD skins were analyzed using immunohistochemistry, RT-PCR, and immunoblot analysis.

**Results:** We showed that alteration of filaggrin and corneodesmosin (CDSN) proteolytic processing was co-related with AD development. New-borne rat showed well-developed epidermis, which became thinner till 2 week-age when hair began to grow. After that, epidermal thickness gradually increased, which was co-related with expression of epidermal differentiation markers, suggesting of U-shape epidermal development. To investigate relationship between AD and epidermal development, neonate, 2 and 4 week-age rats were injected with capsaicin and AD symptoms were monitored. A more late injection produced earlier development of AD but AD symptoms were less severe and shorter duration, suggesting of stimulation in neonatal period potentiated AD symptoms. Subsequent immunohistochemical staining showed increase of Lgr6 expression, which is known as epidermal stem cell marker.

**Conclusions:** These results suggested that postnatal epidermal development may influence on AD development.

## A414 Comparison Between the Impulse Oscillometry System, Spirometry, Feno, Lung Clearance Index and Asthma Control and Exacerbation Status

### Seung Jin Lee^4^, Bo Seon Seo^1^, Ji Hyeon Baek^2^, Kyung Suk Lee^3^, Young-Ho Jung^4^, Hye Mi Jee^3^, Youn Ho Shin^4^, Man Yong Han^4^, Mi-Ae Kim^5^

#### ^1^CHA Bundang Medical Center, CHA University; ^2^Dongtan Sacred Heart Hospital, College of Medicine, Hallym University; ^3^Bundang CHA Medical Center, CHA University School of Medicine; ^4^CHA University School of Medicine, South Korea; ^5^Bundang Medical Center, CHA University

##### **Correspondence:** Seung Jin Lee – CHA University School of Medicine, South Korea

**Abstracts**

**Background:** Little is known regarding possible association between lung clearance index (LCI) monitoring the lung heterogeneity and asthma control and exacerbation. The aim of the study was to determine the relationship between LCI, level of asthma control, and the C-ACT (Childhood Asthma Control Test) score in asthma patients.

**Method:** This study included 97 patients who visited the outpatient department and admitted to the Department of Pediatrics, the CHA Bundang Medical Center, CHA University from October 2013 to December 2014 due to asthma control and exacerbation. The level of asthma control was classified according to the GINA guideline and asthma exacerbation was applied to asthma patients who were hospitalized. We measured the baseline IOS (Impulse oscillometry system), FeNO (Fractional exhaled nitric oxide), spirometry, and LCI, and evaluated the C-ACT score for each subject.

**Results:** Of the 97 subjects, the numbers of patients in the asthma control group, the partly controlled group, the uncontrolled group, and the asthma exacerbation group were 33, 23, 18 and 23, respectively. The mean age of each group was 7.64 ± 2.66 years; there was no statistical difference across the groups (*P*=0.733). The Spearman correlation coefficients revealed a significant correlation between C-ACT and LCI 2.5% (*P*=0.001) and Scond VT (*P*=0.003), but no significant correlation between C-ACT and FEV1 (r=0.136, *P*=0.08), FEV1/FVC (r=0.086, *P*=0.47), Rrs5 (r=0.000, *P*=0.998), Xrs5 (r=0.017, p=0.872) Z score, and FeNO (r=0.015, *P*=1.00). There were significant differences in LCI 2.5% (*P*<0.001), ScondVT (*P*<0.001), FEV1 (*P*<0.001), and FEV1/FVC (*P*=0.010), but no difference in Rrs5 (*P*=0.949) and Xrs5 (*P*=0.077) between the controlled group and the uncontrolled group.

**Conclusion:** LCI and Scond VT were closely correlated with the C-ACT score and were good parameters in differentiating the level of asthma control.

## A415 The Association of Exhaled Nitric Oxide and Airway Hyperresponsiveness in Patients with and without Asthma

### Young-Hee Nam, Dong Sub Jeon, Soo-Keol Lee

#### Dong-a University School of Medicine, South Korea

##### **Correspondence:** Young-Hee Nam – Dong-a University School of Medicine, South Korea

**Background:** Chronic inflammation of the airways and airway hyperresponsiveness (AHR) are key pathological features of asthma. The fraction of exhaled nitric oxide (FeNO) is closely related to eosinophilic airway inflammation and corticosteroid responsiveness. AHR is being used as an indirect marker of the degree of airway inflammation, and AHR to mannitol is more closely related to airway inflammation compared with AHR to methacholine. We sought to evaluate the association between FeNO and AHR in adults with and without asthma.

**Methods:** In 304 patients with symptoms suggestive of asthma, FeNO and AHR to mannitol or methacholine were measured. A total of 180 who underwent both tests were analyzed and were divided into four groups : low (<25ppb)/high FeNO and with/ without AHR.

**Results:** FeNO and response to mannitol was measured in 90 patients (group±), and FeNO and response to methacholine in 90 (groupII). Current asthma was diagnosed in 31 (group±, 34.4%) and 37 (groupII, 41.1%). In non-asthmatics, those with low FeNO/-AHR, low FeNO/+AHR, high FeNO/-AHR, high FeNO/+AHR was 78%, 1.7%, 18.6%, 1.7% in group ±; 66%, 0%, 34%, 0% in groupII. Of the asthmatics, 45.2%, 3.2%, 12.9%, 38.7% in group±; 27%, 24.3%, 8.15, 40.6% in groupII, and neither showed significant difference in atopy, duration of asthma, use of inhaled corticosteroid, blood and sputum eosinohils with regard to distribution of FeNO or AHR, except only in FEV1. A significant correlation was observed between log FeNO and log response-dose rate (RDR) mannitol (r=0.411, P=0.024) and also between log FeNO and log RDR methacholine (r=0.336, P=0.042) in only asthma patients.

**Conclusions:** In asthma patients, the association between FeNO and AHR was stronger in mannitol than in methacholine. However, a significant proportion of patients had high FeNO and no AHR to mannitol and low FeNO and AHR to methacholine.

## A416 Effects of Air Pollution on Allergic Rhinitis in Korea

### Jisun Park

#### Inha University Hospital South Korea

**Background:** It is well known that the prevalence of allergic diseases has increased in recent decades in industrialized countries. Considering the relatively short period of time, environmental factors should be involved in this phenomenon. This study was aimed to evaluate the correlation between allergic rhinitis and air pollutants at the metropolitan city of Incheon and Seoul, Korea.

**Methods:** The number of patients who had visited physicians for allergic rhinitis at Incheon and Seoul was obtained from the medical data of the National Health Insurance Corporation. Data of air pollution were obtained from the Air Pollution Measurement System from 2005-2008, which had been measured in 1 hour intervals. The air pollutants measured were CO(ppm), NO_2_(ppm), O_3_(ppm), PM_10_(mg/m^3^), and SO_2_(ppm). The level of air pollutants in each district was interpolated by kriging estimates of a Geographic Information System from measured data of air pollutants. After adjustment of age and annual number of automobile registration, a ridge regression model was used to determine the correlation of the prevalence of allergic rhinitis and the annual average concentration of air pollutants.

**Results:** The prevalence of allergic rhinitis in children and adolescent was 30.47% and 37.69 % in 2005 and 2008, respectively. The air pollutants that showed significant correlation with the prevalence of allergic rhinitis after adjustment were PM_10_ and ozone for 0-9 yrs of age, ozone for 10-14 yrs, and PM_10_ for 15-19 yrs.

**Conclusion:** In conclusion, PM_10_ and ozone may contribute in increasing the risk of allergic rhinitis. Therefore, it is considered that monitoring of both air pollutants and the prevalence of allergic rhinitis should be continued to clarify the effects of air pollutants on allergic rhinitis in Korea.

## A417 Exhaled Nitric Oxide in Korean Children with Allergic Rhinitis

### Seung Hyun Moon

#### Inha University Hospital South Korea

**A) Background:** Fractional concentration of NO in exhaled breath (FeNO) is mainly used to evaluate the eosinophilic inflammation in asthma. It has also been proposed as an index for evaluating the atopic disease such as allergic rhinitis. Many studies suggest the usefulness of FeNO in allergic rhinitis and level of FeNO may be different depending on the race, age, and other environmental factor. But there have been few studies about the FeNO in Korean children with allergic rhinitis. The aim of this study was to investigate the usefulness of FeNO in Korean children with allergic rhinitis.

**B) Methods:** 647 children aged 5 to 17 were enrolled. All subjects underwent skin prick test, pulmonary function test, and methacholine challenge test. Subjects were classified into 5 groups: 139 children with allergic rhinitis(AR), 83 children with non-allergic rhinitis(NAR), 18 children with asthma, 62 children with AR and asthma, and 345 control subjects. FeNO was then measured in all subjects.

**C) Results:** Mean level of FeNO in AR (32.3 ± 25.0 ppb), asthma (31.1 ± 20.5 ppb), and AR-asthma group (34.5 ± 30.4 ppb) was significantly higher as compared to that of NAR (16.8 ± 13.5 ppb) and control group (15.9 ± 12.5 ppb).(*P*<0.05) There was no significant difference in FeNO level among AR, asthma, and AR-asthma group. There was no significant difference in FeNO level between AR group with BHR and AR group without BHR. In skin prick test, mean level of FeNO in greater than or equal to 4+ group (36.6 ± 29.2 ppb) was significantly higher than that of 3+ group (27.6 ± 20.7 ppb). (*P*<0.01) In the ROC curve analysis for prediction of AR, when it showed 90% of positive predictive value, the cutoff level of FeNO was 50 ppb.

**D) Conclusions:** Level of FeNO has a significant correlation with allergic rhinitis. This result suggests that measurement of FeNO may be useful in the diagnosis of allergic rhinitis. And also, it suggests that there is relationship between allergic rhinitis and eosinophilic inflammation like asthma, although there is no expression of systemic eosinophilic reaction or bronchial hyper reactivity.

## A418 A Questionnaire of Children with Asthma or Asthma and Allergic Rhinitis

### Rong Jun Lin, Ren Zheng Guan

#### The Affiliated Hospital of Qingdao University, China

##### **Correspondence:** Rong Jun Lin – The Affiliated Hospital of Qingdao University, China

**Objective:** To explore the relationship between childhood bronchial asthma and allergic rhinitis,calculate the incidence of allergic rhinitis in patients with bronchial asthma,and analyse the correlationship between allergic rhinitis and bronchial asthma in clinical performance.

**Methods:** To investigate 385 cases of children with bronchial asthma from 0-14 year old in Shinan District of Qingdao by a questionnaire survey, including 242 children with simple asthma (group I),146 children with asthma complicated with allergic rhinitis (Group II), and the control group of 385 selected healthy children (Group III).Through the answers of the parents, we comprehended the relevent circumstances of the concomitant disease, allergy history, family history and the onset schedules. Then, we established a corresponding individual database file and analyzed the data.by statistical methods.

**Results:** The children having personal drug allergy history and family allergy history in group I and group II was significantly higher than that in group III (χ^2^=45.73-147.58, P < 0.05), the chilaren having digestion or skin allergy history was significantly higher than that of group I (χ^2^=7.90, P<0.05).

**Conclusion:** Asthma and allergic rhinitis are both allergic airway diseases, there is a close correlationship between them.

## A419 A Case of Trimebutine-Induced Morbilliform Skin Eruption

### Gyeong Yul Park, Hyun-Sun Yoon

#### Seoul National University Boramae Hospital, South Korea

##### **Correspondence:** Gyeong Yul Park – Seoul National University Boramae Hospital, South Korea

Morbilliform drug eruption is the most common form of cutaneous adverse drug reactions, accounting for up to 95% of entire skin side effects. Every drug theoretically has the potential to induce drug eruption despite of frequency differences. Trimebutine has been widely used in the treatment of gastroenteritis, gastroesophageal reflux disease and irritable bowel syndrome. For its common use, however, reports about dermatologic side effects are surprisingly scarce. We report a case of a 61-year-old woman with pruritic morbilliform skin eruption after taking trimebutine. She had visited our clinic 2 years ago with similar skin lesions after taking medication containing trimebutine. The patient underwent oral provocation test and developed similar generalized morbilliform skin eruption a few hours later. To our knowledge this is the first case report of trimebutine-induced morbilliform skin eruption.

## A420 Comparison of Methacholine and Mannitol to Predict Exercise-Induced Bronchoconstriction in Children with Asthma

### Woo-Hyeok Choi, Heysung Baek

#### Kangdong Sacred Heart Hospital, South Korea

##### **Correspondence:** Woo-Hyeok Choi – Kangdong Sacred Heart Hospital, South Korea

**Background:** Bronchial hyper-responsiveness (BHR) can be assessed by performing bronchial provocation tests (BPTs) with direct stimuli such as methacholine, or indirect stimuli such as mannitol. The aim of this study was to examine the diagnostic properties of methacholine and mannitol challenge to predict exercise-induced bronchoconstriction (EIB) in athmatic children.

**Methods:** Eighty-nine asthmatic children between 6 and 15 years old were enrolled. Exercise challenges were conducted in all subjects. 72 subjects underwent methacholine BPTs and 36 subjects underwent mannitol BPTs. 18 subjects underwent both mannitol and methacholine BPTs. BHR to exercise was defined as a ≥ 15% fall in FEV1 after exercise, to methacholine a PC20 ≤ 25 mg/ml and to mannitol a 15% fall in FEV1 at ≤ 635 mg.

**Results:** Thirty-seven (41.6%, 37/89) subjects with asthma had a positive exercise challange test. The maximum decreases in %FEV1 after exercise were positively correlated with mannitol PD15 (r=-0.540, p=0.038) but not correlated with methacholine PC20 in asthmatics with EIB. The sensitivity of methacholine to identify EIB was 93.3% (28/30) and the specificity was 52.4% (22/42). The sensitivity of mannitol was 66.7% (14/21) and the specificity was 40.0% (6/15).

**Conclusion:** The maximum decreases in %FEV1 after exercise was significantly correlated with mannito PD15 but not with methacholine PC20. However, methacholine is more sensitive than mannitol to identify EIB in asthmatic children.

## A421 Different Inflammatory Mechanisms of Human Metapneumovirus and Respiratory Syncytial Virus

### Jin-Sung Park^1^, Eunmi Kwon^1^, Zac Callaway^2^, Chang-Keun Kim^3^, Takao Fujisawa^4^

#### ^1^Sanggye Paik Hospital, South Korea; ^2^Ulsan University, South Korea; ^3^Inje University Sanggye Paik Hospital, Korea South; ^4^Mie National Hospital, Japan

##### **Correspondence:** Jin-Sung Park – Sanggye Paik Hospital, South Korea

**Background:** Human metapneumovirus (HMPV) and respiratory syncytial virus (RSV) share some epidemiological and clinical characteristics; however, few studies have examined whether these viruses induce similar cytokine responses. This study compared cytokine profiles in HMPV and RSV patients to investigate their inflammatory pathways.

**Methods:** 128 nasopharyngeal aspirate specimens were collected from 128 pediatric patients hospitalized with acute respiratory infection including wheezing and tested for 7 common respiratory viruses. They were divided into HMPV (n=27) and RSV groups (n=101). Th1(IFN-γ), Th2(IL-4, IL-13) and Th17(IL-1β) cytokine profiles were analyzed.

**Results:** IFN-γ levels in the 2 groups were statistically similar (P=0.08). IL-4 levels were significantly higher in the RSV compared to HMPV group (P<0.0001). IL-13 levels in both groups were under detection level. IL-1β levels were significantly higher in the HMPV compared to the RSV group (P<0.0001).

**Conclusion:** Our results suggest that HMPV and RSV have different inflammatory mechanisms. HMPV induces airway inflammation by the Th17 pathway through release of IL-1β, whereas RSV acts through the Th2 pathway.

## A422 Sputum Microbiota in Chinese Adults with Eosinophilic Versus Non-Eosinophilic Asthma

### Qingling Zhang^1^, Rihuang Qiu^1^, Naijian Li^1^, Zhaowei Yang^2^, Jing Li^3^, Kian Fan Chung^4^, Nanshan Zhong^5^

#### ^1^Guangzhou Institute of Respiratory Disease, China; ^2^Department of Allergy and Clinical Immunology, State Key Laboratory of Respiratory Disease, China; ^3^State Key Laboratory of Respiratory Disease, the First Affiliated Hospital, Guangzhou Medical University, China; ^4^Imperial College of Science, Tech. & Medicine, UK; ^5^State Key Laboratory of Respiratory Disease, China

##### **Correspondence:** Qingling Zhang – Guangzhou Institute of Respiratory Disease, China

**Background:** Asthma is a chronic inflammatory disease of the airways, the potential link between microbial infections and asthma is now thought to be environment, immunity and genetic factors. The potential role of bacterial colonization or infection of the bronchial mucosa in the pathogenesis of asthma has been raised by several recent reports. The aim of this study was to examine alteration of airway microbiota among asthma phenotypes in Chinese adult patients.

**Methods:** Induced sputum samples were obtained from 49 non-smoking patients with stable asthma and 15 healthy subjects. Total DNA was amplified by using primers specific for the V3-V5 hypervariable region of bacterial 16s rRNA. Samples were barcoded, and sequenced with the 454 GS FLX sequencer. Sequences were assigned to bacterial taxa by comparing them with 16s rRNA sequences in the Ribosomal Database Project.

**Results:** Asthmatics had lower FEV1% predicted (72.2% vs. 98.6%, p<0.001) and higher sputum eosinophil (13.0% vs. 0.5%, p<0.001) compared to healthy controls. There was no statistically difference in OTU numbers and diversity score between asthmatic and non-asthmatic subjects. At phylum level, the difference of OTU relative abundance remained non-significant. Subjects with eosinopihilic asthma (EA) are older and have shorter duration of asthma (9.6 vs. 19.2 years, p=0.041) as well as lower FEV1% (69.3% vs. 79.2%, p<0.001) compared to subjects with non-eosinopihilic asthma (NEA). The highest OTU numbers (183.9 vs. 142.7 vs. 127.2) and diversity scores, including chao index (318.6 vs. 203.1 vs. 190.8) and ace index (419.8 vs. 251.0 vs. 218.5), were found in NEA group, followed by healthy and EA group. At phylum level, EA subjects had higher abundance of Firmicutes (33.7% vs. 27.5%, p=0.099) but lower Proteobacteria (27.5% vs. 35.2%, p=0.090) compared to NEA subjects, although the differences were not significant.

**Conclusions:** Patients with eosinophilic asthma have an altered microbial composition in the respiratory tract compared with subjects with non-eosinophilic asthma. The corresponding biological effect on airway inflammation remains to be determined.

## A423 Which Clinical Features Are Useful in Predicting Presence of *Staphylococcus Aureus* colonization/Infection in Childhood Atopic Dermatitis?

### Kam Lun E. Hon^2^, Yin Ching K. Tsang^1^, Ting Fan Leung^2^

#### ^1^The Chinese Unbiversity of Hong Kong; ^2^Prince of Wales Hospital, Hong Kong

##### **Correspondence:** Kam Lun E. Hon – Prince of Wales Hospital, Hong Kong

**Introduction:***Staphylococcus aureus (SA)* colonization/infection is important in the pathophysiology of childhood atopic dermatitis (AD). This study evaluated which clinical features may predict presence of SA colonization/infection, and reviewed antimicrobial sensitivity of SA in patients with AD.

**Methods:** The associations between bacteriologic culture results of skin swabs (taken at the most severely affected area and at the antecubital fossa) and SCORing-Atopic-Dermatitis (SCORAD), skin hydration, transepidermal water loss (TEWL) and quality of life were evaluated.

**Results:** Moderate-to-heavy growth of *SA* was present in 31% of the swabs of the most severe area and in 16% of the flexural (antecubital fossae) areas of 95 AD patients (12.5±4.8 years). Binomial logistic regression showed moderate-to-heavy growth of *SA* were associated with objective SCORAD (p=0.004) and lesion intensity [erythema (*p*=0.022) and lichenification (*p*=0.035)] in the severe area; and excoriation (*p*=0.024) and TEWL (*p*=0.009) in the antecubital fossa. The relative risk of isolating moderate-to-heavy growth of SA in the most affected area in patients with severe disease (Objective SCORAD >40) is 2.73 (1.43 – 5.21, *p* =0.001). Any growth of *SA* in either swab sites was associated with objective SCORAD and lesion intensity (*p*=0.001-0.019). *SA* had no association with quality of life and other clinical parameters. All specimens of methicillin-sensitive *SA* were sensitive to cloxacillin. All methicillin-resistant *SA* (5.7%) was sensitive to cotrimoxazole and fusidic acid.

**Conclusions:** Clinical features, especially severity and lesion intensity, are useful in “predicting” presence of *SA* colonization/infection in AD patients. Cloxacillin has a favourable sensitivity profile for methicillin-sensitive-SA, and cotrimoxazole and fusidic acid for methicillin-resistant-SA. These findings will facilitate management of patients before bacteriology results become available.

## A424 Clinical Significance of Increased VEGF, TGF-Î^2^_1,_ and YKL-40, a Chitinase like Protein, in Serum of the Children with Asthma

### Yoon Young Jang, Hai Lee Chung, Seung Gook Lee, Ji Hyun Na, Jong Hoon Lee

#### Catholic University Hospital of Daegu, South Korea

##### **Correspondence:** Yoon Young Jang – Catholic University Hospital of Daegu, South Korea

**Background:** Vascular endothelial growth factor (VEGF), transforming growth factor (TGF)-β_1_, and platelet derived growth factor (PDGF) are known to be involved in the pathogenesis of inflammation and remodeling in asthmatic airways. YKL-40, a chitinase-like protein, and clusterin have been reported to be biomarkers for severe asthma. We examined serum levels of growth factors, YKL-40 and clusterin in 223children with acute asthma or stable asthma and investigated their correlation with clinical findings and lung function parameters.

**Methods:** Forty-one children (≥6yrs of age) with asthma were enrolled and 2 groups were defined: 23 patients admitted with asthma exacerbation and 18 patients with stable asthma. Serum levels of VEGF, TGF-β_1,_ PDGF-BB, YKL-40, and clusterin were measured using ELISA, and assessed in relation to the clinical and spirometric parameters. Fifteen age-matched controls were also studied.

**Results:** VEGF, TGF-β_1,_ and YKL-40 levels in children with acute asthma were significantly elevated compared to controls. VEGF and YKL-40 levels in stable asthma group were higher than in controls and not different from those in acute asthma group. VEGF levels in acute asthma group correlated significantly with asthma severity. TGF-β_1_ levels in stable asthma group showed a significant inverse correlation with FEV_1_% and FEF_25-75_%. YKL-40 had no relationship with clinical and spirometric parameters.

**Conclusions:** Our study suggests that increased VEGF and YKL-40 might affect asthmatic airways not only during acute exacerbation but also in stable state. It also suggests that serum TGF-β_1_ might be a biomarker for airway obstruction in children with asthma.

## A425 Analysis of Follow-up Results of Mannitol Challenge Test in Asthma Patients

### Young-Hee Nam, Dong Sub Jeon, Soo-Keol Lee

#### Dong-a University School of Medicine, South Korea

##### **Correspondence:** Young-Hee Nam – Dong-a University School of Medicine, South Korea

**Background:** Asthma is characterized by chronic inflammation associated with airway hyperresponsiveness (AHR) which is measured using bronchial challenge testing. Mannitol as an indirect challenge has showed better reflect the complex effects of inflammation compared with direct challenges, and which is simple, safe, and more practical to use. Following airway hyperreactivity may be a useful strategy in asthma management because hyperreactivity may be abnormal even in patients with mild asthma and normal lung function. Airway hyperreactivity may better reflect airway inflammation and risk for deterioration than dose lung function or asthma symptoms. We investigated to analyze the follow-up results of mannitol challenge tests in asthma patients.

**Methods:** A total of 59 asthmatics repeated mannitol tests at Dong-A university hospital from May 2010 to February 2015. We compared the clinical characteristics between negative conversion and persistent AHR group.

**Results:** Fifty-three (89.9%) showed AHR to mannitol at initial test, 28 (47.5%) of whom had no AHR (negative conversion, groupI) and 25 (42.4%) had persistent AHR (group II) at follow-up test. Six had no AHR at initial test, 2 had response to mannitol at follow-up test. There were no significant differences in sex, age, smoking habits, levels of eosinophil of serum and sputum, and baseline lung function. A longer duration of asthma and high frequency of asthma exacerbation was observed in group IIthan in group I, while the proportion of atopy and steroid-naïve patients were higher in group I. Total IgE and sputum eosinophil was much more decreased in group I compared with group II.

**Conclusions:** In this study, 42.4% of asthma patients showed persistent AHR to mannitol. The negative conversion group of AHR may be associated with atopy, history of no ICS use, and improving of eosinophilic inflammation.

## A426 Analysis of 68 Oral Walnut Challenge Tests

### Mikita Yamamoto, Sakura Sato, Noriyuki Yanagida, Ayako Ogawa, Kanako Ogura, Kyohei Takahashi, Kenichi Nagakura, Shigehito Emura, Tomoyuki Asaumi, Katsuhito Iikura, Motohiro Ebisawa, Yu Okada

#### Sagamihara National Hospital, Japan

##### **Correspondence:** Mikita Yamamoto – Sagamihara National Hospital, Japan

**Background:** There are few reports on oral food challenge (OFC) tests of walnut.

**Objective:** The purpose of this study is to clarify the risk factors predicting positive result in walnut OFC.

**Subjects:** We retrospectively analyzed 68 patients who had undergone walnut OFC from May 2006 to August 2014 at Sagamihara National hospital.

**Methods:** Open OFCs were performed with greater than 3g of walnut. We measured all patients’ walnut specific IgE within 1 year of their OFCs. Furthermore, we also randomly measured 33 subjects’ allergen components (eg. Jug r 1, Jug r 3) of total subjects. We judged as positive when patients had obvious objective symptoms. When their symptoms at walnut OFC were uncertain, we encouraged them to take same quantity of walnut at home. We made the final conclusion by checking reproducibility at next visit at outpatients.

**Results:** Among 68 patients**,** 49 patients were males. Median of the patient’s age was 7.1(range; 3.1-22.4) years old. Reasons for elimination of walnut were as follows; 1) positive for specific IgE (n=37, 54%), 2) history of immediate reactions to walnut (n=23, 34%), 3) anxiety etc. (n=8, 12%). Forty eight patients (71%) had atopic dermatitis, 30 patients (44%) asthma, 31 patients (46%) allergic rhinitis, 13 patients (19%) allergic conjunctivitis. Twenty four patients (35%) had history of immediate reaction to peanut, and 8 patients (12%) had history of immediate reaction to other nuts. OFC was positive in 34 patients (50%). Five patients (7%) experienced anaphylaxis. Twenty four cases (71%) had cutaneous symptoms, 16 cases (47%) respiratory symptoms, 10 cases (29%) gastrointestinal symptoms, 6 cases (18%) mucosal symptoms, 4 cases (12%) neurological symptoms. Twenty five patients (74%) were treated with antihistamine, 11 patients (32%) with β2 stimulant inhalation, 4 patients (12%) with steroid drug, 3 patients (9%) with adrenaline. Comparing positive patients and negative patients in OFC, positive patients had more frequent history of immediate reaction to walnut (50% vs 18%, *p*=0.0096). The walnut specific IgE titers in positive were higher than those in negative (median of specific IgE 3.36 UA/ml vs 0.47 UA/ml, *p*=0.0013). Twelve of 14 patients (67%) positive to Jug r 1 were positive in OFC.

**Conclusions:** Healthcare providers should be prepared for high positive reaction in walnut OFC, possibly anaphylaxis. Risk factors predicting positive reactions in walnut OFC are the past history of immediate reactions to walnut and high titer of walnut specific IgE, positive to Jug r 1.

## A427 Effectiveness of Air Filters Intervention in Allergic Rhinitis

### Jiaying Luo^1^, Xiao Lan^2^, Baoqing Sun^3^, Zhao Chen^2^, Guiyuan Sun^1^, Shimin Li^2^, Jiaqing Hu^2^

#### ^1^Guangzhou Institute of Respiratory Disease, China; ^2^Guangzhou Medical University; ^3^First Affiliated Hospital Guangzhou Medical University

##### **Correspondence:** Jiaying Luo – Guangzhou Institute of Respiratory Disease, China

**Backgrounds:** Allergic disease constitutes a great threat to human health. Studies have shown that the seizure and progress of allergic disease are related to indoor environment. In recent years, environmental control has begun to be adopted in the world.

**Objectives:** In order to probe into the curative effect of allergic rhinitis thought air filters, we test PM (including PM0.3, PM2.5 and PM10) before and after use it.

**Methods:** A total of 13 (33 ± 11 years old) clinically confirmed allergic rhinitis patients were selected to use the air filter for three months. Before and after the use of air filter (every month), indoor and outdoor PM contents of the subjects were tested. The change in PM values was shown in PM indoor/PM outdoor. In addition, based on International Study of Asthma and Allergies in Childhood, Swedish DBH Household Questionnaire and Rhinoconjunctivitis Quality of Life Questionnaire (RQLQ), and in combination with epidemiological characteristics of Guangzhou allergic diseases, observations were made of the symptoms and seizure frequency of anaphylactic disease patients. Also the curative effect of air filters based environmental control means on allergic diseases was evaluated. The questionnaire covered family allergic histories, allergic histories, way of life, living environment, symptoms of AR seizure, seizure frequency, etc. SPSS21.0 statistical software was used for information input and analysis. Descriptive statistics for measurement data. Non-parametric test and repeated measurement ANOVA were used for comparison among groups. Where P<0.05, the difference was of statistical significance.

**Results:** 1 The ratio of PM0.3, 2.5 and 10 declined (P = 0.001) after the application of air filter. There was statistically significant ratio difference (P = 0.012) between the second month following the application of air filter and the time prior to the use of air filter. 2 According to the RQLQ results, the subjects saw significant drop (P=0.023) in nose, eye and emotional scores one month after the application of air filter.

**Conclusions:** The application of air filter will comprehensively enhance the indoor air quality and improve the allergic rhinitis patients’ quality of life.

## A428 The Relationship Between Airway Hyperresponsiveness to Mannitol and Atopy in Asthmatic Children

### Woo-Hyeok Choi, Heysung Baek

#### Kangdong Sacred Heart Hospital, South Korea

##### **Correspondence:** Woo-Hyeok Choi – Kangdong Sacred Heart Hospital, South Korea

**Aim:** The relationship between airway hyperresponsiveness (AHR) and atopy has been previously investigated, but there are still some issues to be clarified. The aim of this study was to assess the link between AHR to mannitol and atopy in asthmatic children.

**Methods:** We evaluated 70 children with asthma, aged six to 16-years-of-age, using skin prick tests (SPTs), serum total and specific immunoglobulin E (IgE) levels. Pulmonary function tets were performed: baseline, postbronchodilator inhalation and mannitol inhalation. The response to mannitol was expressed as the dose causing a 15% decrease in forced expiratory volume in one second (FEV1) (PD15). Atopy as the presence of at least one positive allergen-specific IgE test result (IgE ≥0.35 kU/l) or a finding on SPT.

**Results:** 49 subjects (70%) with asthma showed a positive result in mannitol bronchial provocation test (BPT). In the mannitol BPT-positive group, 43 (43/49, 87.8%) subjects were diagnosed to atopy, In the mannitol BPT-negative group, 20 (20/21, 95.2%) subjects were diagnosed to atopy. There was no significant difference in atopy prevalence between mannitol BPT-positive and BPT-negative group. We found a correlation between mannitol PD15 and serum total IgE (r= -0.326; p=0.031).

**Conclusion:** In children with pediatric asthma, we could not find a significant correlation between AHR to mannitol and atopy prevalence.

## A429 Anaphylactoid Reactions to N-Acetylcysteine in the Treatment of Aacetaminophen Overdose

### Young-Hee Nam^1^, Dong Sub Jeon^1^, Hee-Joo Nam^2^, Yeo Myeong Noh^2^, Sang Hee Kim Kim^2^, Ye Suel Park^2^, Soo-Keol Lee^1^

#### ^1^Dong-a University School of Medicine, South Korea; ^2^Dong-a University Hospital Regional Pharmacovigilance Center

##### **Correspondence:** Young-Hee Nam – Dong-a University School of Medicine, South Korea

Acetaminopen is one of the drugs most commonly used in intentional self-poisoning. N-acetylcysteins (NAC) is an effective antidote for acetaminophen overdose, which is usually given intravenously for 20-36 hour. When used intravenously, NAC can cause anaphylactoid reactions. Most of the adverse reactions are cutaneous manifestation involving flushing, pruritus, rash and urticaria. However, a few are systemic reactions, such as bronchospasm and hypotension. The etiology of the anaphylactoid reaction is not entirely understood, and the data are conflicting. No study has been conducted to evaluated the IgE response to rule out a true anaphylactic reaction to intravenous NAC. They typically occur within 15-60 minute after NAC infusion and appear to be dose related rather than true anaphylaxis. We report a case of anaphylactoid reactions to NAC has not yet been reported in Korea.

A 17-year-old female was admitted via emergency department. She had 2-year history of depression. She ingested 20 tablets of Geworin®(isoporylantipyrine 150mg, acetaminophen 300mg) in a suicide attempt precipitated by a family quarrel. She suffered from dizziness and vomiting. Gastric lavage was done and then she was immediately treated with NAC. The standard regimen consists of intravenous infusion of NAC 150 mg/kg as a bolus, 50 mg/kg over 4h and repeated infusions of 100 mg/kg over 16h until three consecutive recovering values of the INR have been demonstrated. Immediately after infusion of NAC, she developed generalized urticaria, nausea, vomiting, chest tightness, dyspnea, and hypotension. Administration of NAC was stopped, epinephrine and antihistamine was administered. Additionally activated charcoal was used to prevent drug absorption. Laboratory abnormalities was not seen including serum tryptase. She recovered completely without any sequelae within 24hours.

**Acknowledgement**

This research was supported by a grant from Ministry of Food and Drug Safety to operation of the regional pharmacovigilance center in 2015.

## A430 Effect of Prenatal Maternal Distress and GSDMB Polymorphism on the Development of Recurrent Wheezing in Early Childhood: COCOA Study

### Yean Jung Choi^12^, Si Hyeon Lee^1^, Young-Ho Kim^2^, Mi-Jin Kang^1^, Hyun-Ju Cho^3^, Eun Lee^3^, Song-I Yang^4^, Youn Ho Shin^5^, Kangmo Ahn^6^, Kyung Won Kim^7^, Yoon Hee Kim^8^, So-Yeon Lee^8^, Hyoung Yoon Chang^9^, In Ae Choi^10^, Kyung-Sook Lee^11^, Yee-Jin Shin^7^

#### ^1^Asan Institute for Life Sciences, University of Ulsan College of Medicine; ^2^Asan Medical Center; ^3^Department of Pediatrics, Childhood Asthma Atopy Center, Environmental Health Center, Asan Medical Center, University of Ulsan College of Medicine; ^4^Hallym University Sacred Heart Hospital; ^5^CHA University School of Medicine; ^6^Samsung Medical Center; ^7^Yonsei University College of Medicine; ^8^Hallym University College of Medicine; ^9^Ajou University College of Medicine; ^10^Sewon Infant Child Development Center; ^11^Hanshin University; ^12^University of Ulsan College of Medicine, South Korea

##### **Correspondence:** Yean Jung Choi – University of Ulsan College of Medicine, South Korea

**Background:** Recently, we found prenatal maternal depression and anxiety were related to their offspring’s respiratory infection and atopic dermatitis in infancy. Psychological stress should be considered an important programming factor for wheezing and lung development in early life. Moreover, genetic susceptibility influences the effects between offspring’s health and maternal prenatal distress.

**Objective:** To investigate whether prenatal maternal distress is associated with offspring’s recurrent wheezing (RW) and *GSDMB* polymorphism influence this relationship in early childhood.

**Methods:** The study population consisted of 1074 mother baby dyads recruited from COCOA birth cohort study. Prenatal maternal distress was evaluated by self-reported questionnaires at 36^th^ weeks of pregnancy. Center for Epidemiological Studies-Depression-10 (CESD-10) and State-Trait Anxiety Inventory-Trait subscale (STAI-T) were used to measure maternal depression and anxiety, respectively. Genotyping for *GSDMB* (rs4794820) performed by TaqMan assay. Diagnosis of RW was assessed by parental report of a physician’s diagnosis at 6 months, 1, 2, and 3 year of age and RW was defined as ≥3 reports of wheezing in the first 2 years of life. We also used Cox regression to estimate the association between prenatal maternal distress and offspring’s RW.

**Results:** The cumulative incidence (CI) of RW was 18.9% by age 2. Prenatal maternal depression (aOR 2.75, 95% CI 1.28-5.93) and anxiety (aOR 2.83, 95% CI 1.09-7.35) increased their offspring’s RW by age 2. The hazard ratio (HR) of having a RW up to 3 years of follow-up was 1.43 (95% CI 1.08-1.90) for depression and 1.63 (95% CI 1.21-2.20) for anxiety. Furthermore, the GA and AA genotypes of *GSDMB* was associated with a higher risk of RW. With *GSDMB* GA and AA genotypes, prenatal maternal depression (aOR 8.21, 95% CI 2.51-26.86, p for interaction 0.75) and anxiety (aOR 20.94, 95% CI 2.36-186.40, p for interaction 0.34) increased the risk of offspring’s RW.

**Conclusion:** Prenatal maternal depression and anxiety increased the risk of RW in early childhood. In addition, prenatal maternal depression and comorbid anxiety was associated with a higher risk of offspring’s RW. The effect of maternal prenatal distress on the development of RW may be modified by *GSDMB* polymorphism although no significant interaction was found between prenatal distress and *GSDMB*. Our findings suggest that preventive strategies for reduction of prenatal distress may improve the risk of RW in the offspring.

**Funding source:** This research was supported by funds (2008-E33030-00, 2009-E33033-00, 2011-E33021-00, 2012-E33012-00, 2013-E51003-00, and 2014-E51004-00) from the Research of Korea Centers for Disease Control and Prevention.

## A431 Vitamin D Level in Allergic Rhinitis: A Systemic Review and Meta-Analysis

### Yoon Hee Kim^1^, Min Jung Kim^1^, In Suk Sol^1^, Seo Hee Yoon^1^, Young a Park^1^, Kyung Won Kim^1^, Myung Hyun Sohn^1^, Kyu-Earn Kim^1^, Yong Ju Lee^2^

#### ^1^Yonsei University College of Medicine, South Korea; ^2^Hallym Sacred Heart Kangnam Hospital

##### **Correspondence:** Yoon Hee Kim – Yonsei University College of Medicine, South Korea

**Introduction:** Vitamin D has emerged to play a key role in the allergic disease by influencing to the immune system. Some studies had suggested a relationship between vitamin D status and allergic rhinitis, and others did not. We aimed to systematically review observational studies investigating the level of vitamin D on the prevalence of the current allergic rhinitis (AR) and the development of AR.

**Methods:** We used standard Cochrane systematic review methodology. We searched MEDLINE, EMBASE, the Cochrane Library and KoreaMed to February 28, 2015. We put no restrictions on language or year of publication in our search. Two reviewers completed in duplicate and independently study selection, data abstraction, and assessment of risk of bias. We selected the studies about the current 25-hydrohyvitamin D (25OHD) levels and the prevalence of the current AR, and the other was about the 25OHD levels of cold blood or previously sampled serum and the development of AR.

**Results:** We selected 10 cross-sectional studies about the current 25OHD levels and the prevalence of the current AR and 6 prospective studies about the development of AR relating with the previous 25OHD levels. Meta-analysis was performed to pool odd ratios from 10 cross-sectional studies (n=42,925) (odd ratio [OR] = 0.95 [0.76-1.20] for top vs. bottom category of 25OHD) and 6 prospective studies (n=22,184) (OR = 0.89 [0.70-1.15] for top vs. bottom category of 25OHD). The prospective studies analyzed additionally with adjusting of the general epidemiologic characteristics. Meta-analysis was performed to pool adjusted odd ratios (AOR) from 6 prospective studies (n=22,184) (AOR = 0.92 [0.64-1.33] for top vs. bottom category of 25OHD).

**Discussion:** Available evidence from this meta-analysis suggests that the 25OHD level may not relate with neither the prevalence of the current AR nor the development of AR. Since these studies were very heterogeneous and the retrospective or the observational cohort studies, large randomized controlled trials are needed to determine whether vitamin D supplementation may be beneficial in the prevention of AR.

## A432 Implication of Inspiratory and Expiratory Resistance and Reactance in Children with Asthma

### In Suk Sol^1^, Kyu-Earn Kim^1^, Yoon Hee Kim^1^, Min Jung Kim^1^, Seo Hee Yoon^1^, Yong Ju Lee^2^, Kyung Won Kim^1^, Young a Park^1^, Myung Hyun Sohn^1^

#### ^1^Yonsei University College of Medicine, South Korea; ^2^Hallym Sacred Heart Kangnam Hospital

##### **Correspondence:** In Suk Sol – Yonsei University College of Medicine, South Korea

**Background:** Impulse Oscillometry (IOS) was developed as a non-invasive method to evaluate lung function by measuring respiratory resistance and reactance. Respiratory resistance and reactance were measured over tidal breaths (whole-breath analysis) and measured separately during inspiration and expiration (inspiratory-expiratory analysis). It was known that reactance from inspiratory-expiratory analysis can detect expiratory flow limitation. We investigated characteristics of inspiratory-expiratory measurement obtained by IOS in children with asthma.

**Methods:** We enrolled 96 children with asthma (66 male) and 30 healthy controls (16 male) aged 4 to 18 yrs. All children with asthma were diagnosed in accordance with ATS/ERS guideline. Spirometry and whole-breath and inspiratory-expiratory impulse oscillometry were performed in all enrolled children. The measurements were assessed in asthmatic children compared to control subjects.

**Results:** In whole-breath IOS analyses, asthmatic children had increased resistance at 5Hz (0.82 ± 0.3 vs. 0.69 ± 0.2 kPa/L/s, *P* = 0.009), increased R5-R20 (0.64 ± 0.17 vs. 0.54 ± 0.15 kPa/L/s), decreased reactance at 5 Hz (-0.42 ± 0.2 vs.-0.3 ± 0.14 kPa/L/s, *P* = 0.001), and increased reactance area (AX) (3.3 ± 1.8 vs. 2.3 ± 1.2 kPa/L, *P* = 0.001) than control subjects. In inspiratory-expiratory IOS analysis, expiratory AX was higher than inspiratory AX in both asthmatic children (3.5 [2.4 – 4.8] vs. 2.8 [1.8 – 3.7] kPa/L, *P* < 0.001) and control subjects (2.2 [1.5 – 3.4] vs. 2.0 [1.2 – 3.0] kPa/L, P = 0.02). The change in AX between expiration and inspiration (ΔAX) was larger in asthmatic children compared to control subjects (-0.56 [-1.5 - -0.1] kPa/L vs. -0.27 [-0.71 - -0.18] kPa/L, *P* = 0.023). Whereas the change in X5 between expiration and inspiration (ΔX5) was not significant between asthmatic children and control subjects (0.1 [0.03 – 0.28] vs.0.09 [0.01 – 0.17] kPa/L/s, *P* = 0.231).

**Conclusions:** Children with asthma significantly differed from healthy controls in whole-breath impulse oscillometry. Larger inspiratory-expiratory variation in AX analysis asthmatic children than control subjects could reflect airway narrowing on expiration in childhood asthma.

**Keywords:** Impulse oscillation system, Asthma, whole-breath analysis, inspiratory-expiratory analysis

## A433 The Association of Asthma Predictive Index with Asthma in Preschool Children with Recurrent Wheeze

### Sung Joo Park^1^, Ji-Won Kwon^2^, Woo Kyung Kim^3^, Hyung Young Kim^4^, Hyo-Bin Kim^5^, Ju-Hee Seo^6^, So-Yeon Lee^7^, Gwang-Cheon Jang^8^, Young-Ho Jung^9^, Soo-Jong Hong^10^, Byoung-Ju Kim^11^, Dae-Jin Song^12^, Yun Seok Yang^13^, Jung Yeon Shim^1^

#### ^1^Kangbuk Samsung Hospital/Sungkyunkwan University School of Medicine, South Korea; ^2^Seoul National University Bundang Hospital, South Korea; ^3^Seoul Paik Hospital, South Korea; ^4^Pusan National University Yangsan Hospital, South Korea; ^5^Inje University Sanggye Paik Hospital, South Korea; ^6^Korea Cancer Center Hospital, South Korea; ^7^Hallym University Sacred Heart Hospital, South Korea; ^8^National Health Corporation Ilsan Hospital, South Korea; ^9^Bundang CHA Medical Center, CHA University School of Medicine, South Korea; ^10^Asan Medical Center, South Korea; ^11^University of Cincinnati College of Medicine, USA; ^12^Guro Hospital, South Korea; ^13^Kangbuk Samsung Hospital, South Korea

##### **Correspondence:** Sung Joo Park – Kangbuk Samsung Hospital/Sungkyunkwan University School of Medicine, South Korea

**Objective:** Diagnosis of asthma is challenging in preschool children who wheeze. The Asthma Predictive Index (API) is used as a tool to predict asthma and decide whether to initiate controller therapy in preschool children. The aims of this study were to investigate whether the API was associated with doctor’s diagnosis of asthma in preschool children with recurrent wheeze and find the most relevant criteria to asthma.

**Methods:** We performed a population-based, cross-sectional study with 933 children aged 4-6 years. A total of 900 children completed a modified International Study of Asthma and Allergies in Childhood questionnaire and 121 children with recurrent wheeze were enrolled. Recurrent wheeze was defined as having a lifetime wheeze more than 3 times.

**Results:** The prevalence of doctor’s diagnosis of asthma was 39%. The percentage of children who met the API was 79.5% (major; 64.4%, minor; 57.5%). Positive API showed tendency of association with doctor’s diagnosis of asthma in preschool children with recurrent wheeze (OR; 4.69, 95%CI; 0.97-22.61). Among the API criteria, only doctor’s diagnosis of allergic rhinitis (AR) was significantly associated with asthma (OR; 4.16, 95%CI; 1.86-9.30).

**Conclusions:** Doctor’s diagnosis of AR is likely to have the highest association with asthma among the criteria of API in preschool children with recurrent wheeze.

## A434 Clinical Significance of Serum Total IgE Levels in Children with RSV-Associated Lower Respiratory Illness

### Yoon Young Jang, Hai Lee Chung, Ji Hye Kim, Hyun Seok Lee, Chang Ho Lee

#### Catholic University Hospital of Daegu, South Korea

##### **Correspondence:** Yoon Young Jang – Catholic University Hospital of Daegu, South Korea

**Background:** Respiratory syncytial virus (RSV) is the most common cause of lower respiratory illness (LRI) during infancy and early childhood. RSV has been reported to induce Th2 immune response with increased IgE production during acute infection. We aimed to investigate the relationship between serum total IgE levels and clinical characteristics in the children with RSV-associated LRI (RSV-LRI).

**Methods:** One hundred and seven children under 3 years of age who were admitted with RSV-LRI (bronchiolitis and/or pneumonia) were enrolled. The patients were divided into 2 groups according to their total serum IgE levels on admission: High IgE group (N=39) and normal IgE group (N=68). High IgE levels were defined as values higher than 2 standard deviations (SDs) from the age-matched mean value. The medical records of the patients were investigated to determine if there was any difference in demographic characteristics, clinical and laboratory findings during admission, and recurrence of wheezing within 1 year after discharge between these 2 groups. Among 107 patients, 76 had LRIs for the first time in their lives, from whom we re-analyzed the data in relation to IgE levels. Additionally, difference between children with isolated RSV infection (N=107) and mixed infection with other viruses (N=88) was examined.

**Results:** Median age was 15 months in high IgE group and 5.6 months in normal IgE group (P<0.001). Male preponderance was observed only in the high IgE group (*P*<0.01). The frequency and duration of fever, severity of symptoms, and concurrence of respiratory difficulty were significantly higher in high than normal IgE group (*P*<0.05). There was no difference in admission days and parental allergic diseases. Nearly same findings were observed in re-analysis of data from the patients with the 1st RSV-LRIs, but recurrence of wheezing after discharge was significantly higher in high IgE group (*P*<0.05). The children with isolated RSV infection showed more frequent and prolonged wheezing than those with mixed infection.

**Conclusions:** In our study, the children who presented with high serum IgE levels during RSV-LRI had more severe symptoms comparing with those with normal IgE levels. Our results suggest that increased Th2 immune response induced by acute RSV infection might be associated with severe clinical presentation of LRI.

## A435 Development of a Oak Pollen Emission and Transport Modeling Framework in South Korea

### Changbum Cho, Yun-Kyu Lim, Kyu Rang Kim, Mijin Kim, Baek-Jo Kim

#### Korea Meteorological Agency, South Korea

##### **Correspondence:** Changbum Cho – Korea Meteorological Agency, South Korea

Pollen is closely related to health issues such as allergenic rhinitis and asthma as well as intensifying atopic syndrome. Information on current and future spatio-temporal distribution of allergenic pollen is needed to address such issues. In this study, the Asian Dust Aerosol Model 2 (ADAM2) was utilized as a base modeling system to forecast pollen dispersal from oak trees. Pollen emission is one of the most important parts in the dispersal modeling system. Areal emission factor was determined from gridded areal fraction of oak trees, which was produced by the analysis of the tree type maps (1:5000) obtained from the Korea Forest Service. Daily total pollen production was estimated by a robust multiple regression model of weather conditions and pollen concentration. Hourly emission factor was determined from wind speed and friction velocity. Hourly pollen emission was then calculated by multiplying areal emission factor, daily total pollen production, and hourly emission factor. Forecast data from the UM LDAPS (Unified Model Local Data Assimilation and Prediction System) was utilized as input. For the verification of the model, daily observed pollen concentration from 12 sites in Korea during the pollen season of 2014. Although the model showed a tendency of over-estimation in terms of the seasonal and daily mean concentrations, overall concentration was similar to the observation. Comparison at the hourly output showed distinctive delay of the peak hours by the model at the ‘Pocheon’ site. It was speculated that the constant release of hourly number of pollen in the modeling framework caused the delay.

## A436 Temperature, Humidity, and Air Pollution Affect Atopic Dermatitis Symptoms in Infants and Young Children

### Young-Min Kim^1^, Youngshin Han^1^, Jihyun Kim^1^, Hae-Kwan Cheong^2^, Byoung-Hak Jeon^2^, Kangmo Ahn^1^

#### ^1^Samsung Medical Center, South Korea; ^2^Sungkyunkwan University School of Medicine

##### **Correspondence:** Young-Min Kim – Samsung Medical Center, South Korea

**Background:** The objective of this study is to investigate the short-term effects of meteorological factors and air pollutants on the severity and persistence of atopic dermatitis (AD) symptoms in infants and young children.

**Methods:** In the present study, 176 infants and young children with AD aged under 6 years living in Seoul Metropolitan Area in Korea were enrolled and were followed for 17 months between August 2013 and December 2014. AD symptoms describing the degree (a scale of 0 to 4) of itching, sleep disturbance, erythema, dryness, oozing, and edema were recorded on a daily basis. Generalized linear mixed models (GLMM) with binomially distributed errors were used to estimate of the effects of meteorological factors (daily mean temperature, relative humidity, and diurnal temperature range (DTR)) and air pollutants (PM_10_, nitrogen dioxide (NO_2_), and ozone) on the AD symptoms. Potential confounding factors including age, sex, symptom severity (SCORAD) at initial visit, the presence of fever, and day of week were controlled. Moving averages up to previous 5 consecutive days were used to represent the lag effect of meteorological variables and air quality on AD symptoms.

**Results:** Of the 34,978 person-days, the rate of positive AD symptoms was 44.12% during the study period. Increase in daily mean temperature by 5 °C and relative humidity by 5% were significantly associated with 13.36% (95% CI: 11.03 to 15.63) and 2.70% (95% CI: 1.25 to 4.14) of decrease in AD symptoms, respectively. Particularly in spring, an increase in 5 °C of DTR was related with 53.26% (95% CI: 12.39 to 108.99) of increase in AD symptoms when the effect of moving average from the same day through previous three consecutive days was estimated. An increase in 10 μg/m^3^ of PM_10_ concentration increased 2.95% of AD symptoms (95% CI: 1.35 to 4.5). An increased concentrations in 10 ppb of NO_2_ and ozone were associated with increase in AD symptoms by 4.31% (95% CI: 0.75 to 7.99) and by 5.55% (95% CI: 2.78 to 8.39), respectively. The hazardous effect of PM_10_ on AD symptoms was noted significantly in spring and those of NO_2_ and ozone were found in winter.

**Conclusions:** Short-term exposure to temperature, humidity, and air pollutants are strongly associated with the AD symptoms. Clean air quality is essential for the appropriate management of AD symptoms in children.

## A437 Effects of Compound V on Pulmonary Fibrosis Model

### Chuang/Yao Ming^1^, Jiu-Yao Wang^2^, Ye/Yi Ling^1^

#### ^1^National Formosa University, Taiwan; ^2^National Cheng Kung University Hospital

##### **Correspondence:** Chuang/Yao Ming – National Formosa University, Taiwan

**Background:** Pulmonary fibrosis is a lung disease which is hardly to cure and has a high mortality rate. Recently, the studies suggested that TGF-β plays a central role in the pathogenesis of pulmonary fibrosis. Compound V, isolated from natural source, is used as the antioxidant, however, we find this compound can suppress the expression of collagen on TGF-β treated lung fibroblast cell line. Therefore, we would like to realize the influence of this compound on lung fibrosis animal model.

**Methods:** To establish pulmonary fibrosis of animal model, we treat mice with bleomycin on day 0 by intratracheal injection, so as to treat compound V by oral daily from day -7 to day 7.Afterwards, we collected mice bronchoalveolar lavage fluids, spleen and lung section and observed the effect of compound V on pulmonary fibrosis of animal model through ELISA, histology and SCIREQ(the machine which detect mice lung function).

**Results:** Compound V can improve lung fibrosis in physiology significantly by SCIREQ. The collagen expression in lung sections also decreased apparently.

**Conclusions:** These findings indicate that compound V can reduce collagen expression on bleomycin-induced pulmonary fibrosis animal model. We will further figure out the mechanism of compound V on pulmonary fibrosis.

## A438 Vitamin D Level and the Correlation with IgE in Children with Allergic Respiratory Diseases in Guangzhou China

### Huimin Huang^1^, Baoqing Sun^2^, Yun Chen^3^, Peiyan Zheng^2^, Nili Wei^1^, Wenting Luo^4^

#### ^1^The First Affiliated Hospital of Guangzhou Medical University, China; ^2^First Affiliated Hospital Guangzhou Medical University; ^3^Guangzhou Yuexiu District Maternal and Child Health Care Hospital; ^4^First Affiliated Hospital of Guangzhou Medical University, Guangzhou Institute of Respiratory Diseases

##### **Correspondence:** Huimin Huang – The First Affiliated Hospital of Guangzhou Medical University, China

**Objective:** To explore the vitamin D(Vit D) level and analyse the correlation with immunoglobulin E (IgE) in children with allergic respiratory diseases in Guangzhou China.

**Methods:** 40 children(4.6±2.6yr) from 209 patients with allergic respiratory diseases(Jun to August) in 2013 and 2014 as the experimental group, detecting 11 kinds of serum specific IgE and total IgE antibody. 40 health children as control group,both using Elecsys to detect the Vit D concentration.

**Results:** The Vit D average concentration of the experimental group is 35.4±9.7ng/ml(Male 35.4 ±10.3ng/ml,Female35.3±9.1 ng/ml), the control group is 33.3±9.8 ng/ml(Male 30.4±8.4 ng/ml, Female36.3±10.4 ng/ml), there were no significant difference between two groups and sex. Age and Vit D concentration were negatively correlated(experimental group:r_s_=-0.605, *p*=0.000; control group: r_s_=-0.328, *p*=0.039). In experimental group, levels of total and Dp specific IgE were negatively correlated with serum concentration of vitamin D (r_s_=-0.579, *p*=0.001; r_s_ =-0.334, *p*=0.035, respectively), level of cow’s milk specific IgE was positively correlated with serum concentration of vitamin D (r_s_=0.544, *p*=0.000). Using multivariate linear regression analysis after adjusted with sex and age,there is significant association between the total IgE and serum vitamin D (B -4.386, 95%CI -8.488- -0.285, *p*=0.037) in 40 children with allergic respiratory diseases, and also significant association between both total and Dp specific IgE and serum vitamin D (B -9.56, 95%CI -15.405- -3.715, *p*=0.003; B -0.857, 95%CI -1.561- -0.153, *p*=0.02, respectively) in 21 children with Dp sensitization.

**Conclusions:** Serum Vit D level could be associated with an increased risk of allergic disease development.

## A439 Two Case Reports of Eosinophilic Gastroenteritis Associated with Allergic Disease

### Do Hyeong Lee, Gil-Soon Choi, Hee-Kyoo Kim, Han Su Park

#### Kosin University Gospel Hospital, South Korea

##### **Correspondence:** Do Hyeong Lee – Kosin University Gospel Hospital, South Korea

**Introduction:** Eosinophilic gastroenteritis(EG) is a rare disease with various gastrointestinal symptoms, and characterized by prominent eosinophilic infiltration. EG has heterogeneous clinical manifestations and its etiology remains unknown. We here describe two cases of EG associated with allergic disease.

**Cases:** First case is a 35-year-old man who visited our emergency room complaining abdominal pain, diarrhea, and nausea for the last 5 days after upper respiratory infection. He had an experience of being treated for EG and pancreatitis with ascitis 10 years ago. In his laboratory finding, peripheral WBC count was 21,600 mm^3^with 60% eosinophilia. After endoscopic study and abdominal computed tomography, he was diagnosed with EG and referred to allergic clinic. He didn’t complain any food related allergy symptoms except intermittent mild rhinitis symptoms and cough. He exhibited elevated serum total IgE level and positive responses to house dust mite and Japanese hop pollen on skin prick test. Lung function test showed obstructive pattern with positive response to bronchodilator. Finally, he was diagnosed as EG with allergic rhinitis and bronchial asthma. He was treated with prednisolone therapy for EG, recommended to take anti-allergic medication as well.

The second case is a 42-year-old female with nausea and indigestion of several months. She has been treated for eosinophilic gastritis on the endoscope for one month at another hospital. But, the symptoms were persisted and more aggravated after milk ingestion. Moreover, urticarial was newly developed form 3 weeks ago. WBC count was 6750 mm^3^ with 5.5 % eosinophilia. Skin prick test showed positive response to house dust mite and grass pollen. High serum specific IgE levels to milk were noted. She was diagnosed as EG with food allergy and urticaria. She was recommended avoiding milk with oral antihistamine therapy. Three months after food restriction and medication, her symptom was improved and endoscopic study was normal.

**Conclusion:** We report two cases of EG associated with respiratory and food allergy, respectively. Although EG has various clinical manifestation, our report suggests that the possibility of co-existence of EG and allergic disease should be considered.

## A440 Genome-Wide Association Study (GWAS) May Identify Common Genetic Variations Both in Immediate and Delayed Drug Hypersensitivity

### So-Young Park^1^, Hyo-Jung Kim^1^, Bomi Seo^1^, Jung-Hyun Kim^1^, Min-Gu Kim^1^, Hyouk-Soo Kwon^1^, You Sook Cho^1^, Hee-Bom Moon^1^, Tae-Bum Kim^1^, Yoon Su Lee^2^, Eun-Soon Shin^3^

#### ^1^Asan Medical Center, University of Ulsan College of Medicine, South Korea; ^2^G-SAM Medical Center; ^3^DNA Link

##### **Correspondence:** So-Young Park – Asan Medical Center, University of Ulsan College of Medicine, South Korea

**Background**

There are many studies that investigate genetic factors that cause predisposition to drug hypersensitivity. However, most studies have focused on specific phenotypes or drugs. Thus, we planned to perform a genome-wide association (GWAS) study to discover the common genetic markers associated with both in immediate and delayed drug hypersensitivity reactions.

**Methods**

We studied 190 patients who were diagnosed with drug hypersensitivity at a tertiary referral hospital and 133 control subjects. We divided the patients into 2 groups; immediate-type hypersensitivity reaction (n=109) and delayed-type hypersensitivity reaction (n=81), according to the type of drug hypersensitivity. Genome-wide SNP genotyping of the patients was performed using genomic DNA from peripheral blood lymphocytes.

**Results**

In the immediate-type hypersensitivity reaction group, we found signification associations with a total of 11 genes, whereas five genes showed signification association in the delayed-type hypersensitivity reaction group. The top 5 SNPs were rs17732181, rs12674658, rs10463158, rs10028040, and rs1330221 in the immediate-type hypersensitivity groups. As well, rs10028040, rs17732181, rs10463158, rs28786672, and rs12674658 have also been identified in the delayed-type hypersensitivity groups. Interestingly, there were two genes that overlapped in the two groups, namely, the Lipopolysaccharide-Responsive Beige-like Anchor (LRBA) gene and the Methionine adenosyltransferase 2 beta (MAT2B) gene. Associations with the SNPs of these genes were commonly found in both immediate and delayed type hypersensitivity reactions (rs10028040 of LRBA, P = 5.63 x 10^-14^, P = 7.19 x 10^-12^ respectively, and rs10463158 of MAT2B, P = 6.40 x 10^-12^, P = 5.25 x 10^-12^ respectively).

**Conclusions**

Genetic variants of the LRBA and the MAT2B genes might be significant genetic markers of drug hypersensitivity reactions. Replication studies are also needed to confirm our findings.

## A441 Development of a Questionnaire for Secular Change of Atopic Dermatitis from Birth to 19-Year-Old

### Akio Tanaka^1^, Satoshi Morioke^1^, Yukihiro Ohya^2^, Naoki Shimojo^3^, Akira Akasawa^4^, Michihiro Hide^1^, Hiroko Shizukawa^1^

#### ^1^Hiroshima University, Japan; ^2^National Center for Child Health and Development; ^3^Graduate School of Medicine, Chiba University; ^4^Tokyo Metropolitan Children’s Hospital

##### **Correspondence:** Akio Tanaka – Hiroshima University, Japan

**A) Background:** The clinical course of atopic dermatitis (AD) is highly diverse. Most of previous epidemiologic studies of the prevalence and disease severities of AD show conditions at a certain time point of the patients. We here developed a questionnaire about the secular change of AD severities and performed a web-based study of patients with AD in Japan.

**B) Methods:** We first created a pilot questionnaire that consists of nine basic patterns and a space for free drawing of secular change of AD from birth to 19-year-old. The analysis of the answers of 76 patients with AD, who visited Department of Dermatology, Hiroshima University, revealed that more than 90% of subjective clinical courses of the patients fell into one of the 9 basic patterns. Next, we completed the questionnaire for patients to choose one of the nine basic patterns or not applicable (N/A), and used it for web-based epidemiological studies using the Macromill online research system (Macromill Inc, Tokyo, Japan). The first study was conducted with 10,347 participants over 20 years of age in March 2014. In total, 1,496 individuals (14.5%) declared that they had ever been diagnosed as AD. Among the subjects with AD or a history of AD, 73.9% individuals chose one of the presented nine patterns and 26.1% individuals chose N/A. We then added an option for choice “(their) AD was developed after 20-years-old” and a few words to prompt the subjects to avoid N/A, and performed another survey using the revised questionnaire for 3,090 subjects with AD in February 2015.

**C) Results:** The latest study showed that 5.9% individuals chose N/A, 17.3% individuals chose “(their) AD was developed after 20-years-old”, and the other 76.8% chose that their secular changes of AD were classified as one of the nine representative patterns. Interestingly, 32.3% chose improving curves, 12.0% chose a nearly flat curve, 19.7% chose exacerbating curves, 8.3% chose a curve representing improvement after exacerbation curve, and 4.5% chose a curve representing exacerbation after improvement.

**D) Conclusion:** The epidemiological survey using this new questionnaire gives us a new clinical aspect of AD. The secular change of disease severity may be influenced by acquired conditions including treatments or any environmental factors. The epidemiological studies about the correlation between secular change and treatment or patient characteristics including medication phobia may give us a new insight into the management of AD.

## A442 Evaluation of the Adherence Starts with Knowledge-20 (ASK-20) to Inhaled Drug in Patients with Bronchial Asthma

### Naoto Watanabe

#### Tokyo Allergy and Respiratory Disease Research Institute, Japan

**Background**

Adherence Starts with Knowledge-20 (ASK-20) is a questionnaire to investigate the cause decreasing medication adherence.

**Purpose**

In this study, we investigated inhaled drug adherence status of asthma patients by ASK-20.

**Subjects**

Seventy-four patients with bronchial asthma whose symptoms are relatively stable (mean age 48 year-old, 36 males, 38 females) used inhaled drug.

**Method**

We analyzed and evaluated for each items from 5 points to 1 point in the order of the heavy degree among five choice answers in based on describe about inhaled drug of the ASK-20 by subjects.

**Results**

Lifestyle: Ratio of over 4 points was 48.6% in Q1 and 12.2 %, 2.7%, 10.8%, 9.5%, 18.9% in Q2, Q3, Q4, Q5 and Q6.

Attitudes and Beliefs: Ratio of below 2 points was 83.8% in Q7 and 63.5% in Q8.

Help from Others: Ratio of below 2 points was 55.4% in Q9.

Talking with Healthcare Team: Ratio of below 2 points was 87.8%, 89.2%, 87.8% in Q10, Q11 and Q12.

Talking Medicines: Ratio of over 4 points in Q13, Q14 and Q15 was each 36.5%, 21.6% and 5.4%.

Have You…: Ratio of over 4 points in Q16, Q17, Q18, Q19 and Q20 was each 31.1%, 5.4%, 4.1%, 1.4% and 27.5%.

**Conclusion**

In results of Q1 and Q2, patients who sometimes forget medicine were about half ratio, but about 90% of patients had been consulted before drugs are eliminated.

Noteworthy, 36.5% of patients didn’t like inhaling more than twice a day in results of Q13 and Q14. And about 30% of patients was in the wrong number and amount of taking medicine by result of Q16. Or about 20% of patients did not have the medicine to the specified time in result of Q20.

**Consideration**

It is considered that their adherence increases if we could switch to inhaled drug for once a day because approximately 40% patients have thought troublesome about twice inhalation a day.

## A443 The Epidemiology and Clinical Manifestation of Hmpv Infection in Children during Recent 4 Years: 2011-2014

### Meeyong Shin, Myeong Sun Jang

#### Soonchunhyang University, South Korea

##### **Correspondence:** Meeyong Shin – Soonchunhyang University, South Korea

**Background:** This study was performed to investigate the epidemiologic and clinical features of human metapneumovirus (hMPV).

**Methods:** We performed realtime RT-PCR with nasopharyngeal samples of 2,403 children who were hospitalized with acute respiratory infection. Then medical records of 120 children, who were diagnosed with hMPV respiratory infection between 2011 and 2014, were analyzed retrospectively and compared to the epidemiologic data on the respiratory virus infection reported by ‘Korea Centers for Disease Control and Prevention’.

**Results:** We detected 120(6.9%) hMPV-positive cases out of 1723 virus-positive specimens, which prevailed mostly in Spring between March and May. The respiratory infection with hMPV was more common in female population(55%). The age distribution of hPMV infection was 7.5% in <12 month, 23.3% in 1-2 years, 26.7% in 2-3 years, 20% in 3-4 years, and 11.7% ≥5 years of age. 11.7%, showing that hPMV was prevalent among the children aged 1-4 years.

Coinfections with other respiratory viruses were observed in 34 patients (28.3%) of 120 hMPV-positive cases; rhinovirus (52.9%), adenovirus (17.6%), bocavirus (17.6%), RSV (5.9%), parainfluenza (3%) and coronavirus (3%). The outbreak of hMPV infection in 2014 was found. Among 120 hMPV-positive patients during recent 4 years, most patients (68.3%) were diagnosed in 2014, and in 2014 hMPV detection rate was 8.99% among virus-positive cases. During 2011-2014 influenza virus infection was prevalent mainly from January to March, and hMPV infection started to appear just after the end of influenza virus outbreak. In 2014, hPMV infection was mostly presented with pneumonia (81.0%), and it was more common in younger children, compared to hMPV infection in 2011-2013 (mean age; 2.04±0.84 vs 3.78±2.57, p=0.001).

**Conclusions:** Infection with hPMV was most prevalent among children 1-4 years of age, and hMPV was the most common causing organism of viral pneumonia during Spring season in Korean children. The hMPV infection pandemic was observed in 2014 and the clinical importance of hMPV has been increasing recently. Therefore additional studies are required to define the epidemiology, disease characteristics caused by hMPV and the cause of recent outbreak.

## A444 Neutropenia Induced By Intravenous Immunoglobulin

### Young-Hee Nam^1^, Yeo Myeong Noh^2^, Dong Sub Jeon^1^, Hee-Joo Nam^2^, Sang Hee Kim Kim^2^, Ye Suel Park^2^, Soo-Keol Lee^1^

#### ^1^Dong-a University School of Medicine, South Korea; ^2^Dong-a University Hospital Regional Pharmacovigilance Center

##### **Correspondence:** Young-Hee Nam – Dong-a University School of Medicine, South Korea

Intravenous immunoglobulin (IVIg) is used as an immunomodulatory agent in various autoimmune disease and generally considered a safe therapy. Most of the adverse effects (AEs) associated with IVIg administration are mild and transient. The immediate (AEs) include headache, flushing, fever, and anaphylactic reactions, especially in IgA-deficient patients. Late AEs are rare and include acute renal failure, thromboembolic events, aseptic meningitis, and neutropenia. Patients with antibody deficiencies are more prone to develop acute netropenic episodes even during IVIg replacement. IVIG-induced neutropenia is transient and mild or moderate, and no severe infectious complications have been reported. We report a case of neutropenia in a patient with Guiilian-Barre syndrome (GBS) after infusion of IVIg.

A 57-year-old female presented with a 4-day history of quadriparesis. She had no previous medical history. She developed myalgia and progressive descending weakness involving both upper and lower extremities. Electrophysiologic study showed motor dominant neuropathy. There were no abnormal findings on brain magnetic resonance imaging. A raised protein concentration but a normal cell count was observed in cerebrospinal fluid (CSF). No virus-specific antibodies, bacteria, and fungi were detected in CSF, blood, and urine. She was administrated IVIg (0.4g/kg daily) for GBS. She gradually recovered strength in her extremities after 2 days. On day 3 administration of IVIg, severe neutropenia (absolute neutrophil count < 1500 cells/mm^3^) occurred. Tue infusion of IVIg was stopped, however, neutropenia lasted for 5 days. She developed a fever and was given granulocyte colony-stimulating factor. She improved and was discharged with no infectious and neurologic complications on the 15^th^ hospital day.

**Acknowledgement**

This research was supported by a grant from Ministry of Food and Drug Safety to operation of the regional pharmacovigilance center in 2015.

## A445 A First Case of Lymphocytic Interstitial Pneumonitis in Healthy Child

### Ji-in Jung, Ha-Su Kim, Hyun-a Kim, Jin-a Jung

#### Dong-a University Hospital, South Korea

##### **Correspondence:** Ji-in Jung – Dong-a University Hospital, South Korea

**Introduction:** Lymphocytic interstitial pneumonitis(LIP) is an uncommon histopathologic entity characterized by infiltration of the interstitium and alveolar spaces of the lung by lymphocytes, plasma cells and other lymphoreticular elements. LIP in children has been most commonly associated with human immunodeficiency virus(HIV) infection. We report a first case of LIP in a HIV-negative healthy adolescent.

**Case Report:** An 13-year-old boy represented with pneumonia on chest X-ray in routine health examination. He had no respiratory symptoms and there was no abnormal findings on physical examination, A chest CT showed multiple ill defined centrilobular nodules and peribronchial air space nodules with mild volume loss in RLL. There was no increase of IgG Mycoplasma pneumoniae antibody titer in 3 serial tests between 7 days interval. Tuberculin skin test and Tbc specific antigen induced interferon-gamma(IGRA) test were negative. Chest x-ray in regular intervals during 6 months showed improvement of pneumonic consolidation, but new pneumonic consolidation on other site. Therefore, open lung biopsy was performed, and it showed diffuse interstitial lymphocyte infiltrate. The lung tissue was negative for EBV by DNAPCR and EBV serology showed all-negative. Serum immunoglobulins were normal and autoimmune studies were negative. The total lymphocyte count and lymphocyte subset were normal. He was started on oral prednisone (1 mg/kg/day) for 2 months and the chest x-ray returned to normal. Then he was tapered oral prednisone for 2 months and follow-up chest CT showed no abnormal finding. He has been followed for 8 months after diagnosis without complications.

**Conclusion:** We report a first case of HIV-negative, EBV-negative LIP in a healthy child.

## A446 Cytokine Production upon House Dust Mite Stimulation of Cord Blood Mononuclear Cells from Caesarean Section-Delivered Singaporean Infants

### Anne Goh^1^, Rajeshwar Rao^1^, Bindu Nandanan^2^, Ruurd Van Elburg^3^, Chua Mei Chien^1^, Juandy Jo^2^, Johan Garssen^4^, Johan Garssen^3^, Leon Knippels^4^, Elena Sandalova^2^, Wen Chin Chiang^1^

#### ^1^Kk Women’s and Children’s Hospital, Singapore; ^2^Danone Nutricia, Singapore; ^3^Nutricia Research, Netherlands; ^4^Utrecht University, Netherlands

##### **Correspondence:** Anne Goh – Kk Women’s and Children’s Hospital, Singapore

**Background:** Epidemiological data from cohort studies indicate associations between Caesarean section and development of allergy in infants. Besides the delayed colonization of commensal bacteria (which in turn affect the immune system development), the cytokine profile in C-section delivered infants could be different, which may be implicated in infant’s immunity.

**Methods:** A clinical study was conducted at KK Women’s and Children’s Hospital, where 55 cord blood samples from Caesarean section-delivered infants were collected to evaluate the response to allergens in the cord blood cells. Freshly isolated cord-blood mononuclear cells (CBMCs) were cultured in a 96-well plate at 100,000 cells per well (in triplicate per condition) and stimulated with the extracts from either one of major house dust mite species: *Blomia tropicalis* (BloT) and *Dermatophagoides pteronyssinus* (DerP). *Phytohemagglutinin* (PHA) and *Lipopolysaccharide* (LPS) were used as positive controls. The supernatants of these cutures were harvested after 5-day stimulation and were analyzed using the Bio-Plex Pro^TM^ Human Cytokine assay. The production of IL-5, IL-13, IFN-gamma and TNF-alpha by the allergen-stimulated CBMCs were analyzed.

**Results:** DerP stimulation induced a higher response as compared to BloT stimulation. Following DerP stimulation, the median value of IL-5 was 2.5 pg/mL (0-172 pg/mL), IL-13 – 9.1 pg/mL (0-158 pg/mL), IFN-gamma - 321 pg/mL (69-1978 pg/mL) and TNF-alpha - 1393 pg/mL (74-5658 pg/mL). Among the 55 analysed subjects, while 3 subjects (5%) showed higher expression of IFN-gamma (above 1000 pg/mL) and lower expressions of IL-5 and IL-13 (both below 20 pg/mL), 6 subjects (11%) displayed higher expressions of IL-5 and IL-13 (both above 20 pg/mL). TNF-alpha levels were detected high in most subjects. Of note, no correlation between strong production of T_H_2-like cytokines (IL-5 and IL-13) and parental allergy history was observed.

**Conclusion:** We observed a strong production of T_H_2-like cytokines in 11% of C-section-delivered infants upon DerP stimulation. Interestingly, these infants did not have any history of parental allergy. This finding might indicate in utero sensitization of these infants. The TH2-like cytokine profile in cord blood might be useful as an early sign of atopic manifestations in future. We plan to follow-up these subjects in order to monitor the development of allergic response later in life.

## A447 Dress Syndrome with Acute Interstitial Nephritis Caused By Quinolone and Nonsteroidal Anti-Inflammatory Drugs

### Young-Hee Nam^1^, Ji Young Juong^1^, Soo Jin Kim^1^, Eun Young Kim^1^, Su Mi Lee^1^, Young Ki Son^1^, Hee-Joo Nam^2^, Ki-Ho Kim^1^, Soo-Keol Lee^1^

#### ^1^Dong-a University School of Medicine, South Korea; ^2^Dong-a University Hospital Regional Pharmacovigilance Center

##### **Correspondence:** Young-Hee Nam – Dong-a University School of Medicine, South Korea

Drug reaction with eosinophilia and systemic symptoms (DRESS) syndrome is a rare and severe drug-induced hypersensitivity syndrome characterized by hematological abnormalities and multiorgan involvement. Liver involvement is the most common visceral manifestation. However, renal failure has been rarely described. The common culprit drugs are anticonvulsants and allopurinol. We experienced a patient with DRESS syndrome with acute interstitial nephritis caused by concomitant administration of quinolone and non-steroidal anti-inflammatory drugs (NSAIDs).

A 41-year-old man presented with a diffuse erythematous rash and fever which developed after administration of quinolone and NSAIDs for a month due to prostatitis. He was diagnosed with DRESS syndrome. Skin rash, fever, eosinophilia, and elevations of liver enzymes improved with conservative treatment and discontinuation of the causative drugs. However, deterioration of his renal function occurred on day 8 of admission. The levels of blood urea nitrogen and serum creatinine increased and oliguria, proteinuria and urinary eosinophils were observed. Ultrasonography showed diffuse renal enlargement. The clinical features were compatible with acute interstitial nephritis. Despite intravenous rehydration and diuretics, renal function did not improve. After hemodialysis, his renal function recovered completely within 2 weeks without administration of systemic corticosteroid.

**Acknowledgement**

This research was supported by a grant from Ministry of Food and Drug Safety to operation of the regional pharmacovigilance center in 2015.

## A448 IL-23 Roles in the Development of House Dust Mite Allergic Sensitization and Asthma

### Da-Eun Park^1^, Hye-Ryun Kang^2^, Heung Woo Park^3^, Hyun Seung Lee^1^, Yoon-Seok Chang^3^, Jung-Won Park^4^, Sang-Heon Cho^1^, Kyung-up Min^1^, Woo-Jung Song^3^

#### ^1^Institute of Allergy and Clinical Immunology, Seoul National University Medical Research Center, South Korea; ^2^Seoul National University Hospital; ^3^Seoul National University College of Medicine; ^4^Division of Allergy and Immunology, Department of Internal Medicine, Yonsei University College of Medicine

##### **Correspondence:** Da-Eun Park **–** Institute of Allergy and Clinical Immunology, Seoul National University Medical Research Center, South Korea

**Background:** IL-23 has been postulated to be a critical mediator contributing to airway inflammation. House dust mite (HDM) is one of the most common allergens. However, the role of IL-23 in HDM-induced mouse model is not well understood.

**Objective:** To evaluate roles of IL-23 in HDM-induced allergic asthma model, particularly during the allergen sensitization period.

**Methods:** BALB/c mice were repeatedly administered with HDM intra-nasally, to develop acute allergic asthma model. Anti-IL-23p19 antibody was given during HDM sensitization period. And we analyzed the activation of DC in LDLN after last sensitization. In addition, to evaluate the roles of IL-23 at bronchial epithelium contacted with HDM, in vitro BEAS-2B cell experiments were done with HDM stimulation and/or anti-IL23 antibody treatment.

**Results:** Anti-IL-23 antibody treatment during allergen sensitization significantly diminished several phenotypes of allergic asthma; particularly, eosinophilic inflammation and airway hyperresponsiveness were markedly reduced. In bronchoalveolar lavage fluid, IL-4, IL-5, and IL-17A cytokine levels were significantly reduced in anti-IL-23 antibody treated mice. And the activation of DC in LDLN was significantly reduced by anti-IL-23 after last sensitization. In in vitro study, anti-IL-23 treatment prevented pro-inflammatory and pro-allergic cytokine responses in HDM-stimulated BEAS-2B cells.

**Conclusion:** IL-23 may play a significant role in allergic asthma during allergen sensitization period. Particularly, it may be significantly involved in allergen-contacted epithelial cell responses finally leading to allergic sensitization.

## A449 Exposure Profile of Indoor Risk Factors in Dwellings of Children with Atopic Dermatitis

### Hyunwook Lim^1^, Sungchul Seo^2^, Ji Tae Choung^3^, Young Yoo^2^, Jun-Sik Park^1^, Byung Kwan Kim^4^

#### ^1^Korea University Medical Center, South Korea; ^2^Korea University; ^3^Korea University Anam Hospital; ^4^Korea University Guro Hospital

##### **Correspondence:** Hyunwook Lim – Korea University Medical Center, South Korea

**Background:** High prevalence rates of asthma and allergic rhinitis have recently been shown in South Korea. For better prevention of development or exacerbation of both allergic diseases, the secondary prevention from symptoms of atopic dermatitis (AD) could be effective, in the aspect of “allergic march”. For this reason, we aimed to explore how the variation of indoor risk factors may be linked with the change of symptoms of AD or development of asthma and/or allergic rhinitis.

**Methods:** We recruited homes of AD patients aged with around 24 months old, along with SCORing Atopic Dermatitis (SCORAD) Index of greater than 25 through the both Environmental Health Centers for Asthma and Atopic allergy. Total volatile organic compounds (TVOC), formaldehyde (HCHO), airborne mold and bacteria, and particulate matter with diameter less than 10 mm (PM_10_) were measured in their dwellings in both indoor and outdoor. Particularly, the levels of TVOC and PM_10_ were monitored for 24 hrs using direct reading instruments as well.

**Results:** Both environmental surveys were performed in a total of 51 houses. The mean age (± standard deviation, SD) and SCORAD Index of subjects was 2.2 ± 0.2 years old and 39.5±18.3, respectively. The geometric mean (GM) concentrations of airborne mold were 526.6–565.5 CFU/m^3^, which refer to above the recommended level of WHO. However, the corresponding value of airborne bacteria were below the Korean standard guideline (800 CFU/m^3^). For exposure level of gaseous pollutants in indoor, TVOC and HCHO ranged from 566.6–748.3 μg/m^3^ and 31.0–35.9 μg/m^3^, respectively, but the outdoor levels were 382.4–453.1 μg/m^3^; for TVOC and 4.0–4.6 μg/m^3^ for HCHO. The highest levels of TVOC and PM_10_monitored using each direct reading instrument were observed after midnight and in the evening, respectively.

**Conclusions:** Our findings indicate that a comparable amount of bioaerosols and gaseous pollutants in indoor were observed in patient’s dwellings. Particularly, different exposure patterns for particulate matter and TOVC were shown time –dependently, and so a proper prevention strategy should be prepared.

## A450 Epidemiological Characterization of Blood Eosinophils in the Elderly Population

### Ha Kyeong Won^1^, Hye-Ryun Kang^1^, Byung-Keun Kim^2^, Sung Do Moon^1^, Ju-Young Kim^1^, So-Hee Lee^1^, Woo-Jung Song^3^, Heung Woo Park^3^, Min-Koo Kang^1^, Sun-Sin Kim^1^, Sang-Heon Cho^4^, Kyung-up Min^4^, Yoon-Seok Chang^3^, Kyoung Hee Sohn^3^, Kyung-Mook Kim^5^, Ki-Woong Kim^2^, Hak Chul Jang^2^

#### ^1^Seoul National University Hospital, South Korea; ^2^Seoul National University Bundang Hospital; ^3^Seoul National University College of Medicine; ^4^Seoul National University Medical Research Center; ^5^Pogunhan Mom Hospital

##### **Correspondence:** Ha Kyeong Won – Seoul National University Hospital, South Korea

**Background:** Peripheral blood eosinophilia is a common clinical manifestation which draws medical attention. However there are little reports on blood eosinophilia in the elderly. We aimed to investigate the normal ranges of blood eosinophils and associated factors including allergic parameters, using the dataset of a community-based random elderly population in Korea.

**Methods:** A cross-sectional analysis was performed using the database of Korean Longitudinal Study on Health and Aging, a community-based elderly cohort. The primary outcome was peripheral blood eosinophil counts and the upper limit of the 95% confidence interval for absolute count of blood eosinophil counts was calculated for normal ranges. Demographic, socioeconomic, metabolic, comorbidities, allergic parameters, and lung function were analyzed. Atopy was defined with skin test by positive to any of 12 common inhalant allergens. Allergic history and symptoms were assessed by questionnaires.

**Results:** Cutoff value for blood eosinophilia was less than 406.6x10^3^/mL (n=1,000). The prevalence of eosinophilia (>500x10^3^/mL) and hypereosinophilia (>1,500x10^3^/mL) were 4.12% and 0.20%, respectively. In univariate analyses, blood eosinophil counts was significantly related to male sex, and smoking status. However, in multivariate analyses, the association of blood eosinophil count with male sex appeared to be at least partly dependent on smoking status. Other potential determinants (*P*<0.10 in univariate analyses) were body mass index, atopy, current wheeze, and physician-diagnosed asthma. In multivariate linear regression analyses, the relationships between blood eosinophil counts and potential determinant factors were examined.

**Conclusion:** The normal range of peripheral blood eosinophils in the Korean elderly was ≤ 406.6 x10^3^/mL (upper 95th percentile), which was almost identical to the reference range in the literature. Smoking was a major determinant factor for eosinophilia, and explained the association of eosinophilia with male sex. Other determinant factors were body mass index, atopy, and asthma. These findings could suggest a potential role of blood eosinophils as an epidemiologic marker for atopy and asthma in the elderly population.

## A451 Stevens-Johnson Syndrome Caused By Methotrexate in the Treatment of Psoriasis

### Young-Hee Nam^1^, Dong Sub Jeon^1^, Hee-Joo Nam^2^, Yeo Myeong Noh^2^, Sang Hee Kim Kim^2^, Ye Suel Park^2^, Soo-Keol Lee^1^

#### ^1^Dong-a University School of Medicine, South Korea; ^2^Dong-a University Hospital Regional Pharmacovigilance Center

##### **Correspondence:** Young-Hee Nam – Dong-a University School of Medicine, South Korea

Stevens-Johnson syndrome (SJS) is severe acute mucocutanous bullous disorders that are most commonly drug-induced. Antibiotics are the most common cause of SJS, followed by analgesics, nonsteroidal anti-inflammatory drugs, anticonvulsants, and antigout drugs. Methotrexate, a folic acid antagonist, has been used in the treatment of psoriasis, rheumatoid arthritis and neoplastic disease including leukemia and lymphoma. Its principal toxic effects are bone marrow suppression, gastrointestinal mucositis, hepatitis, renal impairment, and erythematous rashes. SJS has been reported in a few patients receiving intermediate or high dose methotrexate. Whether the epidermal necrolysis is an allergic or dose-related toxicity reaction is still controversial. We report a case of SJS in a patient receiving low dose methotrexate for psoriasis.

A 67-year-old male presented with a generalized erythematous rash and erosion, and severe oral ulcers. He had started allopurinol and well tolerated for 1 year. The patient had a history of low-dose methotrexate treatment (5 mg/day) 6 days before the development of his complaints. On the second day after methotrexate treatment, erythematous itchy and edematous rash developed on his legs, which spread widely on his trunk and extremities with subsequent bullous formation. He suffered from aggravation of oropharyngeal ulcer. He was admitted to a local hospital and treated with systemic corticosteroids for 5 days. Skin lesions improved, but he was transferred to our hospital for persistent painful ulcer and odynophagia.

He was treated with topical steroid for skin lesion and total parenteral nutrition had been started because he had difficulty in eating. He recovered gradually and was discharged with supportive therapy but without additional systemic corticosteroid.

**Acknowledgement**

This research was supported by a grant from Ministry of Food and Drug Safety to operation of the regional pharmacovigilance center in 2015.

## A452 Genetic Determinants for Lung Function Growth in Asthmatic Children

### Ting Fan Leung^1^, Man Fung Tang^1^, Hing Yee Sy^1^, Wa Cheong Chan^1^, Wilson Wai San Tam^2^

#### ^1^Prince of Wales Hospital, Hong Kong; ^2^Yong Loo Lin School of Medicine, Singapore

##### **Correspondence:** Ting Fan Leung – Prince of Wales Hospital, Hong Kong

**Background:** Asthma traits are determined by complex interactions between predisposition genes and environmental influences. The GABRIEL consortium identified through meta-analysis of genome-wide association study ten asthma loci. Nonetheless, the relevance of these loci on longitudinal changes in patients’ lung function remains unknown. This prospective cohort study investigated the effects of these asthma loci on changes in spirometric indices among Chinese asthmatic children.

**Methods:** 158 Chinese asthmatic children aged 6-12 years were recruited from our paediatric allergy clinic. These patients were prospectively followed for five years. Pre-bronchodilator spirometry was recorded at baseline and then monitored at least annually. Spirometric indices were compared with local references. Genomic DNA from these patients was genotyped for single-nucleotide polymorphisms (SNPs) on the 10 asthma loci by TaqMan genotyping assays. Generalised estimating equation was used to analyse effects of these SNPs on longitudinal changes in lung function parameters.

**Results:** The mean (SD) age of patients at baseline was 10.0 (1.8) years, and 104 (66%) of them were male. Twenty-eight percent had passive smoking and 58% ever received inhaled corticosteroid (ICS) treatment during follow-up. About three quarters of these patients had family history of allergies. Rs3894194 and rs9273349 were not genotyped due to unavailable TaqMan assays. Adjusting for age, sex, passive smoking exposure, ICS treatment and presence of upper respiratory infection within two weeks, rs1342326 of *IL33* was significantly associated with FEV_1_ (B=1.952, *P*<0.001), FVC (B=1.215, *P*<0.001), FEV_1_/FVC (B=0.752, *P*<0.001) and FEF_25-75_ (B=1.219, *P*=0.005). Rs2305480 of *GSDMB* was also associated with FEV_1_/FVC (B=0.940, *P*<0.001). The other six SNPs from *SLC22A5*, *IL13*, *IL2RB*, *RORA*, *SMAD3* and *IL18R1* did not show significant association with longitudinal change in any spirometric index.

**Conclusions:** This is the first Asian study of genetic determinants for lung function growth. *IL33* appears to be a candidate gene for longitudinal changes in several spirometric indices among Chinese children with asthma. Larger cohorts are needed to replicate our findings due to low frequency of risk allele in *IL33*_rs1342326 among our patients.

**Funding:** Research Grants Council General Research Funds (469908 and 470909); Research Committee Group Research Scheme (3110060) and Direct Grant for Research (2013.2.033) of CUHK

## A453 The Power of Allergen Specific Ig E in the Classification of Rhinitis, Korean National Hanes 2010

### Seung Kyu Chung^1^, Sujin Kim^1^, Sang Duk Hong^1^, Hyo Yeol Kim Hyo Yeol Kim^1^, Hun-Jong Dhong^2^, Jong in Jeong^1^

#### ^1^Samsung Medical Center, South Korea; ^2^Samsung Seoul Hospital

##### **Correspondence:** Seung Kyu Chung – Samsung Medical Center, South Korea

**A) Background:** Usually rhinitis is classified simply allergic and non-allergic rhinitis according to the allergic reaction to airborne allergens. Ig E–mediated inflammation is confirmed with positive skin test or presence of specific IgE and the positive result from the one of the two tests is usually enough for the diagnosis do allergic rhinitis. We are interested in the diagnostic power of the allergen specific Ig E. Recently, during Korean National Health and Nutrition Examination Survey 2010, presence of rhinitis and the specific Ig E for major airborne allergens were checked in the population. Purpose of this study is to evaluate the significance of allergen specific Ig E in the diagnosis of allergic rhinitis.

**B) Method:** The data were obtained from the 2010 Korean National HANES, which was a cross-sectional survey of non-institutionalized population all around the country. Presence of rhinitis was defined as “Have you experience the rhinitis symptoms” or “Have you diagnosed as have allergic rhinitis from doctor”. Serum specific IgE was checked for Dermatophagoides farinae, cockroach and dog. Data was obtained from 1,922 adult (older than 18). The positive predictive values (PPV) of allergen specific IgE on the diagnosis of allergic rhinitis were calculated according to the level of the specific Ig E.

**C) Results:** The specific Ig E higher than 0.34 kU/L(+) was found in 63.6 % in population with rhinitis and 39.7 %in the population without rhinitis. The PPVs were 61% with level of 0.35 kU/L and 66% with the level of 3.5 kU/L(+++). The prevalence of the allergic rhinitis increased with the increasing levels of the specific IgE, from 37.3 % to 77.4 % and the PPV also increased. However the positive predictive rate was 72.4 % with the level of 3.5 kU/L and 82.6 % with the level of 17.5 kU/L(++++).

**D) Conclusion:** Diagnostic power of specific IgE on the classification of rhinitis is not high and the level of specific Ig E higher than 3.50 kU/L may be more clinically significant.

## A454 Analysis of Allergen Immunotherapy Practice and PatientsÃ¢â‚¬â„¢ Knowledge and Attitude about Allergen Immunotherapy in a Single Tertiary Hospital in Korea

### Young-Hee Nam, Dong Sub Jeon, Soo-Keol Lee

#### Dong-a University School of Medicine, South Korea

##### **Correspondence:** Young-Hee Nam – Dong-a University School of Medicine, South Korea

**Background:** Allergen immunotherapy (AIT) is currently the only immune-modifying treatment for allergic disease. The clinical efficacy of AIT for the treatment of allergic rhinitis and bronchial asthma is well documented. However, many factors including inconvenience, cost, side effects, and adherence influence the initiation and persistence with AIT. We sought to evaluate the AIT practice pattern and patients’ attitude and behavior about AIT.

**Methods:** We conducted a retrospective analysis of medical records of 157 patients received AIT, and compared the clinical characteristics between conventional (CIT) and rush immunotherapy (RIT). A total of 56 were performed a questionnaire survey.

**Results:** Of 157 patients, 108 (68.8%) were treated with CIT, and 49 (31.2%) with RIT. There were no significant differences in allergic diseases, allergens in immunotherapy, and the frequency of systemic adverse reactions during build-up phase. The rate of missing patients was higher in CIT than RIT (18.5% vs 10.2%). Patients initiation with AIT was mainly according to physician recommendation (76.3% for CIT vs 55.6% for RIT). Patients with RIT had personal insurance more and showed better treatment satisfaction than those with CIT. Concern about adverse events was the main reason for start CIT, while frequent hospital visits for start RIT.

**Conclusions:** A majority patients initiated AIT according to physician recommendation and showed good treatment satisfaction. RIT may give better clinical outcomes than CIT.

## A455 Roles of Staphylococcal Enterotoxin B in House Dust Mite-Induced Acute Asthma Models

### Ji Won Lee^1^, Mingyu Kang^2^, Soon-Hee Kim^3^, Boram Bae^4^, Sujeong Kim^4^, Hye-Ryun Kang^5^, Yoon-Seok Chang^6^, Woo-Jung Song^6^, Da-Eun Park^4^, Hyun Seung Lee^4^, Heung Woo Park^6^, Han-Ki Park^4^, Jung-Won Park^7^

#### ^1^Seoul National University Medical Research Center, South Korea; ^2^Chungbuk National University Hospital, South Korea; ^3^Seoul National University Bundang Hospital, South Korea; ^4^Institute of Allergy and Clinical Immunology, Seoul National University Medical Research Center, South Korea; ^5^Seoul National University Hospital, South Korea; ^6^Seoul National University College of Medicine, South Korea; ^7^Division of Allergy and Immunology, Department of Internal Medicine, Yonsei University College of Medicine, South Korea

##### **Correspondence:** Ji Won Lee – Seoul National University Medical Research Center, South Korea

**Background:** Immune responses to staphylococcal enterotoxins may contribute to the pathogenesis of asthma.

**Objective:** To investigate roles of staphylococcal enterotoxin B (SEB) in mouse asthma models

**Methods:** BALB/c mice were intranasally sensitized with *Dermatophagoides pteronyssinus* (Der p) and/or SEB to develop acute asthma models. Mice were grouped to see effects of SEB and Der p interactions during the sensitization and challenge periods. Outcomes were evaluated for methacholine airway hyperresponsiveness (AHR), lung inflammatory cells, lung cytokines, serum IgE, and *in vitro* Der p-stimulated splenocyte responses.

**Results:** Intranasal SEB exposure increased lung macrophage, lymphocyte, and neutrophilic infiltration; but SEB alone did not develop any typical asthmatic phenotypes. Effects of SEB on asthma phenotypes were the most evident when co-administered with Der p during the sensitization period, developing more AHR and lung eosinophilic inflammation on Der p challenge. Serum Der p specific IgE levels were also amplified by SEB co-sensitization. In *in vitro* Der p re-stimulation experiments, IL-5 and IL-13 responses were also increased in splenocytes from the mice co-sensitized by SEB and Der p.

**Conclusion:** The present findings indicate the roles of SEB in Der p-induced allergic asthma, particularly during the sensitization period.

## A456 A Clinical Comparison of Drug Reaction with Eosinophilia and Systemic Symptoms Syndrome in a Single Tertiary Hospital in Korea

### Young-Hee Nam^1^, Dong Sub Jeon^1^, Hee-Joo Nam^2^, Yeo Myeong Noh^2^, Sang Hee Kim Kim^2^, Ye Suel Park^2^, Soo-Keol Lee^1^

#### ^1^Dong-a University School of Medicine, South Korea; ^2^Dong-a University Hospital Regional Pharmacovigilance Center

##### **Correspondence:** Young-Hee Nam – Dong-a University School of Medicine, South Korea

**Background:** Drug reaction with eosinophilia and systemic symptoms (DRESS) syndrome is a rare disease which can cause severe morbidity and mortality. We have reported DRESS syndrome was not more uncommon than generally recognized in the previous study.

**Objective:** We investigated the clinical manifestation of DRESS syndrome and compared with previous data.

**Methods:** A total of 100 patients were prospectively collected from October 2011 to March 2015 (period I, 43 months). We compared these data to those of 42 DRESS patients from September 2009 to August 2011 (period II, 24 months).

**Results:** The most common causative drugs were antibiotics, followed by anticonvulsants and antituberculosis drugs in both periods (37%, 17%, 17% in period I vs 31%, 24%, 13% in period II, respectively). Eosinophilia in peripheral blood and hepatic involvement was more frequently noted in period II, while enlarged lymph nodes in period I significantly. The mean latency period was 32.6±107.5 days (range, 1-1095) in period I, and 20.2±24.3days (range, 2-120) in period II (P=0.255). The longest latency period was noted for the antituberculosis drugs, followed by anticonvulsants and antibiotics in both periods. Systemic corticosteroids were administered to 22 patients (20%) and 10 (22%) in each period. All the patients showed complete recovery in period ±, while 2 patients (4.4%) had poor outcomes.

**Conclusions:** The clinical manifestation of DRESS syndrome was variable. Antibiotics were the most frequently implicated drugs and antituberculosis drugs had a long latency period. A majority of patients showed good clinical outcomes without administration of systemic corticosteroid.

**Acknowledgement**

This research was supported by a grant from Ministry of Food and Drug Safety to operation of the regional pharmacovigilance center in 2015.

## A457 The Beneficial Effect of Lactobacillus Gasseri PM-A0005 and Its Immunoregulatory Protein PMA5P40 on Milk-Induced Allergic Enteritis

### Yung-I Hou^1^, Jiu-Yao Wang^2^

#### ^1^National Cheng Kung University, Taiwan; ^2^National Cheng Kung University Hospital

##### **Correspondence:** Yung-I Hou – National Cheng Kung University, Taiwan

Food allergy is one of the common allergic diseases in the world. Probiotics are shown to promote the endogenous host defense and modulate the host’s immune responses to potentially harmful antigens. In this study, we first established a mouse model of food allergey by i.p. sensitization with OVA or β-lactoglobulin, and were orally challenged with protein allergen, OVA, or β-lactoglobulin in BALB/c mice. We found that the body weights and body temperatures immediately after food allergen challenged were significantly decreased as compared with the control mice. And the symptoms of food allergy, the levels of total IgE, allergen-specific IgE, IgG1, IgG2a, IgA of sera and the levels of IgE, IgA, IL-4 and IL-17 of intestine lavage fluids (ILF) of food allergen mice were significantly increased as compared with the control mice. After oral administration with 10^7^ CFU *Lactobacillus gasseri* PM-A0005 (*L. gasseri* ) at the same time during food allergen sensitization and challenge, the body temperature, body weight, the levels of antigen-specific IgA of sera, and IL-10 concentration of the ILF, and the levels of IL-10, IL12, INF-γ, TGF-β of the culture medium and the cellular numbers of CD11c^+^CD103^+^, CD11b^-^CD8a^+^ dendritic cells (DCs), CD4^+^FoxP3^+^ (Treg cells), CD4^+^T bet^+^ (TH1 cells) in the Peyer’s patches and draining lymph nodes of *L. gasseri*-treated mice were significantly increased as compared with the non-treated food allergy mice. In contrast, the food allergy symptoms, the levels of antigen-specific IgE, IgG1 of sera and the levels of total IgE, antigen-specific IgE, IgG1, α1-antitrypsin, IL-17, IL-6 of the ILF and the concentrations of TNF-α, IL-23, IL-6 of the culture medium and the cellular numbers of CD4^+^RORγt^+^ (TH17 cells), CD4^+^GATA3^+^ (TH2 cells), lymphocytes proliferation and the intestinal inflammation of *L.gasseri-*treated mice with food hypersensitivity were significantly decreased as compared with the non-treated control mice. To further explore the anti-allergy effect of *L. gasseri* of dendritic cells (DCs) on food allergy development, bone marrow-derived dendritic cells (BMDCs) isolated from naïve mice were stimulated for 24 hrs with recombinant of *L. gasseri.* The PMA5P40-primed BMDCs were collected and adoptive transfer to mice sensitized and challenged with food allergens. These finding suggested that *L. gasseri* and recombinant of protein PMA5P40 have anti-allergy effect on the mice sensitized and challenged with food allergens, such as OVA and cow’s milk. And this anti-allergic effect may be mediated through the tolergenic effect of dendritic cells that enhance immune-regulatory effect on T and B lymphocytes, which may have clinical application in patients suffered with food allergy.

## A458 Relationship Between Serum Folate Levels and Risks of Allergic and Respiratory Diseases in Early Childhood: The Mothers and Children’s Environmental Health Study

### Ja Hyeong Kim^1^, Seol Jae Hee^1^, Eun-Hee Ha^2^, Hyesook Park^2^, Mina Ha^3^, Yun-Chul Hong^4^, Yangho Kim^1^, Namsoo Chang^2^

#### ^1^Ulsan University Hospital, South Korea; ^2^Ewha Womans University; ^3^Dankook University College of Medicine; ^4^Seoul National University College of Medicine

##### **Correspondence:** Ja Hyeong Kim – Ulsan University Hospital, South Korea

**Background:** Folate, a dietary methyl donor, is known to alter gene expression influencing immune system through epigenetic modification. However, the relationships between folate level and the risk of allergic and respiratory diseases in children are still unknown.

**Objectives:** To investigate whether or not serum folate levels are associated with atopic biomarkers and also with the risk of allergic and respiratory diseases in children.

**Methods:** Data of 462 children with complete information from a birth cohort in South Korea were available. Serum folate levels were analyzed at 24 months of age in children. Atopic biomarkers such as total Ig E, IL-10 and eosinophil counts were also measured at 24 months. Information on maternal demographic and obstetrical characteristics and that on children’s allergic and respiratory outcomes was obtained from questionnaire.

**Results:** Serum folate levels were inversely associated with eosinophil counts (*r* = - 0.192, *P <* 0.001). Total IgE levels and eosinophil counts decreased significantly in the group whose serum folate was above the median value (17.6 ng/mL) compared to the other counterpart group (*P* = 0.013, *P <* 0.001 respectively). A multivariate logistic regression analysis revealed that folate level above the median value (17.6 ng/mL) was associated with a decreased risk for atopic dermatitis (AD) (adjusted odds ratio [aOR], 0.57; 95% confidence interval [CI], 0.34-0.95) at 24 months of age. However, no significant association was observed between serum folate levels and respiratory outcomes in children.

**Conclusions:** Serum folate level is associated with a lower risk of developing AD in early childhood.

## A459 Effects of Vitamin D in Patients with Chronic Rhinosinusitis

### Yuta Soma^1^, So Watanabe^1^, Ruby Pawankar^2^, Ruby Pawankar^1^, Harumi Suzaki^1^, Harumi Suzaki^3^, Hitome Kobayashi^1^

#### ^1^Showa University School of Medicine, Japan; ^2^Nippon Medical School; ^3^Tokyo General Hospital

##### **Correspondence:** Yuta Soma – Showa University School of Medicine, Japan

**Background:** The dysregulated mucosal inflammation in chronic rhinosinusitis (CRS) is often difficult to control with pharmacotherapy alone. We sought to determine whether the immunomodulatory properties of vitamin D might have beneficial effects on CRS.

**Methods:** We performed a randomized, placebo controlled trial in adult subjects meeting the research criteria for CRS, with low serum vitamin D levels (<40 ng/mL), and having no contraindications to vitamin D therapy. Subjects were randomized to receive vitamin D (n=12) or placebo (n=12) for 12 weeks in addition to their standard regimen. Peripheral blood mononuclear cells (PBMCs) and nasal epithelial cells (NECs) were collected at the beginning and end of the trial and we analyzed the upregulation of molecules involved in mucosal immunity by RT-PCR. Clinical response was analyzed using SNOT-22 and the SF-36.

**Results:** The Levels of vitamin D in the serum were elevated in the vitamin D group and unchanged in the placebo group. Cathelicidin, human b-defensin and autophagy related protein light chain 3 alpha (LC3A, a molecule involved in autophagy) were upregulated after vitamin D administration in PBMCs with similar results observed in NECs. There was no significant change in the upregulation of these mediators in the placebo group. No symptomatic changes were observed in SNOT-22 or SF-36 scores in either group. There were no side effects or adverse events during the study.

**Conclusions:** These results suggest that vitamin D might modulate mediators involved in immune responses in CRS. Further studies are needed to clarify the clinical efficacy of vitamin D in this disorder.

## A460 Clinical Features of Systemic Contact Dermatitis from Ingestion of Rhus

### Young-Hee Nam^1^, Chansun Park^2^, Soo-Keol Lee^1^

#### ^1^Dong-a University School of Medicine, South Korea; ^2^Haeundae Paik Hospital, Inje University College of Medicine

##### **Correspondence:** Young-Hee Nam – Dong-a University School of Medicine, South Korea

**Background:** Systemic contact dermatitis (SCD) is an inflammatory skin disease that may occur in persons with contact allergy when they are exposed to the allergens systematically including orally, transcutaneously, per rectum, intravesically, intravenously, or by inhalation. The most common causes of SCD are drugs used both topically and systemically. Other causes are ubiquitously occuring haptens, such as the metals nickel, cobalt, gold, and chromate, and aromatic substances such as spices. SCD from plants has been seen following ingestion of various rhus preparations. In Korea, Rhus has been used as a folk medicine to cure gastrointestinal diseases and as a health food, it is common to observe patients with accidental or occupational Rhus dermatitis, and also SCD caused by ingestion of Rhus.

**Objective:** We investigated the clinical features of SCD caused by Rhus.

**Methods:** We conducted a retrospective analysis of 11 patients with SCD caused Rhus in Dong-A University hospital and Haeundae Paik Hospital.

**Results:** Nine (81.8%) were women, and average age was 58.91±10.29 years (range 45-75). The way of Rhus ingestion was boiled chicken with Rhus (n=9, 81.8%), Rhus vegetables (n=1), and Rhus sap (n=1). All patients experienced generalized pruritus and erythematous maculopapular rash. The patients developed erythema multiforme (n=2), vesiculobullous lesion (n=3), urticaria (n=3), and angioedema (n=1). Extracutaneous symptoms were duodenal ischemia (n=1), hypotension (n=2), fever (n=4), and oral mucositis (n=1). The mean latency period was 1.45±0.69 days (range 1-3), and mean recovery times were 12.27±3.77 days (range 5-18). Systemic corticosteroid was administered in 10 (90.9%) for 18.09±15.44 days (range 4-47).

**Conclusions:** SCD from ingestion of Rhus showed various cutaneous manifestation mainly erythematous maculopapuar eruption, and also extracutaneous symptoms. Most of the patients recovered well with the treatment of systemic corticosteroids.

## A461 Sublingual Immunotherapy Efficacy in Patients with Atopic Dermatitis in Korea

### Jongrok Lee, Jooyoung Roh, Haryeong Ryu

#### Gilhospital, South Korea

##### **Correspondence:** Jongrok Lee – Gilhospital, South Korea

**Background:** Atopic dermatitis (AD) is currently regarded as an allergic inflammatory disease. Many studies have shown AD to have multiple causes that activate complex immunological and inflammatory pathways. However, aeroallergens, and especially the house dust mite (HDM), play a relevant role in the elicitation or exacerbation of eczematous lesions in many AD patients. Accordingly, allergen-specific immunotherapy has been used in AD patients with the aim of redirecting inappropriate immune responses. Sublingual immunotherapy (SLIT) is especially preferred because of its easy application and safety. The aim of this study was to describe the effect of SLIT in patient with AD.

**Methods:** Patients with AD who allergic to HDM were evaluated. Patients who allergic to HDM have been treated with SLIT for over 1yr. The effect of patients on clinical course of SLIT with *Dermatophagoides pteronyssinus* and *Dermatophagoides farinae* in a standardized extract was assessed and the side effect of SLIT were recorded.

**Results:** The records of 20 patients (13 males and 7 females) were studied. The mean age for starting SLIT was 16.3 years (aged 6-36 years, median age 13years). Treatment was deemed effective or very effective in 70% of the patients. The majority of the patients were satisfied with their treatment, which was well tolerated. There were no serious side effects except transient nausea, abdominal pain and oral itching sensation in the first few days. SLIT had to be discontinued in 2 patients (10%) because of exacerbation of asthma and dermatitis. Especially, the compliance of patients with AD and allergic rhinitis were better than patients with AD only.

**Conclusions:** SLIT reduces symptoms of AD, the amount of drug consumption and the progression of the disease. Especially, SLIT is recommendable to patients with AD and allergic rhinitis. We believe SLIT can be an alternative, safe treatment option for allergic AD refractory to long-term conventional treatment.

## A462 Characteristics of Serious Adverse Drug Reactions in a Tertiary University Hospital

### Cheol-Woo Kim, Jae Hwa Cho, Mi Ra Eom, Ji Young Kang, Hye Gyeung Lee

#### Inha University Hospital, South Korea

##### **Correspondence:** Cheol-Woo Kim – Inha University Hospital, South Korea

**Background:** Adverse drug reactions (ADRs) frequently occur in hospital setting, and serious ADRs (SAEs) may threaten the patient’s life and lead poor clinical outcome. Early detection and urgent medical intervention is required to prevent development of SAEs. This study was conducted to investigate the clinical characteristics of SAEs in a single university hospital.

**Methods:** ADRs reported to hospital Pharmacovigilance Center were collected from Jan 2012 to Dec 2014, and cases of SAEs were selected. Clinical information was collected from electronic medical records.

**Results:** A total of 283 (3.7%) SAEs among 7,629 ADRs were identified through spontaneous reporting system. A gradually decreased tendency in the frequency and incidence of SAE was noted from 2012 to 2014. SAEs were reported by doctors (40.6%), nurses (11.0%), and pharmacists (48.4%). ADR related-hospitalization or prolongation of existing hospitalization (53.2%) was the most common cause of SAEs, and other medically important event was the second cause. SAEs were developed from the injections (54.4%), PO medications (44.9%), and the patch type agents (0.7%). Antineoplastic agents (40.9%), anti-infectives (16.8%), and anti-tuberculosis drugs (6.9%) were the drug class commonly involved. White cell and RES disorders (21.6%) were frequently involved system organ classes, and skin and appendages disorders (16.4%) were the next. Leukopenia and neutropenia were the most frequently noted SAEs.

**Conclusion:** Antineoplastic agents, anti-infectives, and anti-tuberculosis drugs can elicit SAEs most frequently. A decreased tendency for the development of SAEs was noted, and this might be result at least partly from the pharmacovigilance activity and ADR monitoring. Comprehensive prophylactic approaches will be required to prevent development of predicted SAEs, and to reduce the chance of unpredicted SAEs.

## A463 Eyelid Dermatitis: Patch Test Results during a 15-Year Period in Korea and Evaluation of Metal Contents in Eye Shadows

### Hae Young Choi^1^, Hye Jin Lee^1^, Ju Yun Woo^1^, Ji Yeon Byun^2^, You Won Choi^2^

#### ^1^Ewha Womans Mokdong Hospital, South Korea; ^2^Ewha Womans University Mokdong Hospital

##### **Correspondence:** Hae Young Choi – Ewha Womans Mokdong Hospital, South Korea

**Background.** Allergic contact dermatitis is a common diagnosis in eyelid dermatitis. Sensitization to metals are prevalent in eyelid dermatitis and color cosmetic products are frequently suspected as the source of metal exposure. This study was performed to investigate the recent contact allergens for eyelid dermatitis and to assess metal contents in eye shadow products.

**Methods.** Data were collected in the department of dermatology of Ewha Womans University hospital from December 1998 to February 2014. A total of 983 patients were patch tested during the period and 67 patients had eyelid dermatitis among them. To examine metal elements in color cosmetic products for eyes, randomly selected 10 eye shadows were analyzed.

**Results.** Frequent allergens were metals, thimerosal, and phenylenediamine in patients with eyelid dermatitis. The sensitization rates of individual allergens were not significantly different between patients with eyelid dermatitis and without eyelid dermatitis. All 10 eye shadow products contained more than 5 ppm of at least one element, nickel, cobalt or chromium.

**Conclusion.** Metals were top-rank allergens in eyelid dermatitis and eye shadow products contained significant amount of nickel, cobalt or chromium to elicit allergic reactions. Patients with eyelid dermatitis and metal allergy should be informed that color eye make-up products can elicit or aggravate their symptoms.

## A464 Relationship Between Lipid Levels and Risks of Allergic and Respiratory Diseases in Early Childhood: The Mothers and Children’s Environmental Health Study

### Ja Hyeong Kim^1^, Eun-Hee Ha^2^, Hyesook Park^2^, Mina Ha^3^, Yun-Chul Hong^4^, Yangho Kim^1^, Namsoo Chang^2^

#### ^1^Ulsan University Hospital, South Korea; ^2^Ewha Womans University; ^3^Dankook University College of Medicine; ^4^Seoul National University College of Medicine

##### **Correspondence:** Ja Hyeong Kim – Ulsan University Hospital, South Korea

**Background:** Recent studies suggest that dietary change may influence programming of the immune system in favor of development of allergic disease. However, the results for the effect of fat status on development of allergic or respiratory disease in children are still conflicting.

**Methods:** A total 426 children were included in this study from a birth cohort in South Korea. Data regarding the children’s allergic and respiratory outcomes were obtained from standardized questionnaires completed by the mothers. Serum total cholesterol, triglyceride (TG) and high-density cholesterol (HDL) levels were measured in children at 24 months of age. Atopic biomarkers including total Ig E, IL-10 and eosinophil counts were also measured at 24 months.

**Results:** Serum HDL cholesterol levels were inversely associated with the eosinophil counts counts (*r* = - 0.221, *P <* 0.001). Total IgE, eosinophil counts, and IL-10 levels increased significantly in the group whose serum HDL cholesterol levels was above the median value (51mg/dL) compared to the other counterpart group (*P* = 0.014, *P* = 0.003, *P* = 0.040 respectively). However, there was no association between lipid levels and risks of respiratory outcomes and AD at 24 months in logistic regression analysis.

**Conclusions:** We found that the HDL cholesterol levels were associated with lower atopic biomarkers in children at 24 months, but not with risk of allergic or respiratory diseases.

## A465 IL-32 in the Induced Sputum of Patients with Asthma

### Jae-Woo Kwon^4^, Hun Soo Chang^1^, Jeong-Seok Heo^1^, Jong-Uk Lee^2^, Jong-Sook Park^1^, Eusom Kim^3^, Soo Hyun Kim^3^, Choon-Sik Park^1^

#### ^1^Soonchunhyang University Bucheon Hospital; ^2^Soonchunhyang University; ^3^Konkuk University; ^4^Kangwon National University College of Medicine, South Korea

##### **Correspondence:** Jae-Woo Kwon – Kangwon National University College of Medicine, South Korea

**Background**

Interleukin-32 (IL-32), first reported as an inducer of tumor necrosis factor-α, is a pro-inflammatory cytokine involved in various chronic inflammatory diseases. This study evaluated the relationship between sputum IL-32 expression and severity of asthma.

**Methods**

IL-32 was determined in induced sputum samples of patients with asthma (n = 137) using sandwich ELISA. Relationships among sputum IL-32, airway obstruction (FEV_1_), inflammation (neutrophil and eosinophil % of the airway) and exacerbation frequency were evaluated.

**Results**

Sputum IL-32 levels was well correlated with FEV_1_(%) in the total study subjects (r=-0.232, P = 0.006) and in the stable asthmatics (r=-0.312, P = 0.003). The levels was not different by use of inhaled steroid (P=0.068). When the all subjects were grouped into two inflammatory patterns by eosinophil% (over 3%, under 3%) or Neutrophils% (over 70%, under 70%), IL-32 levels was not different by the inflammatory patterns. IL-32 levels was significantly increased in the subject experiencing exacerbation (n=69) compared to those not experiencing exacerbation (n=53) (p<0.05). The sputum levels of IL-32 were well correlated with the annual rate of exacerbation in the total subjects.(r=0.231, P < 0.022).

**Conclusion**

Sputum IL-32 may be associated with severity of the asthma regardless of inhaled steroid treatment, and may be a surrogate marker for exacerbation risk of asthma.

## A466 Clinical Characteristics of Patients with Chromium Allergy in a Single University Hospital in Korea

### Hae Young Choi^1^, Ji Yeon Byun^2^, Ju Yun Woo^1^, You Won Choi^2^

#### ^1^Ewha Womans Mokdong Hospital, South Korea, ^2^Ewha Womans University Mokdong Hospital

##### **Correspondence:** Hae Young Choi – Ewha Womans Mokdong Hospital, South Korea

**Background:** Chromium allergy has been traditionally caused by occupational skin contact with cement and related construction materials, which involves hands most commonly.

**Objective:** To investigate the clinical characteristics of patients with chromium allergy in Korea.

**Methods:** Patch test data from June 1998 to January 2014 were retrospectively analyzed (n=975; male=289, female=686). Patients who showed positive reactions to potassium dichromate (chromium (+) group; n=58) and who showed positive reactions to other allergens except potassium dichromate (others (+) group; n=497) were identified and the characteristics of both groups were compared. The prevalence of chromium allergy was additionally analyzed in each 4-year period to study the change over time.

**Results:** The sensitization rate of potassium dichromate was 5.9% (n=58) and women comprised 72.4% (n=42) of chromium (+) group. Hands and feet were more frequently affected in chromium (+) group (*P*=0.002, 0.019). Occupational dermatitis was not significantly more common in chromium (+) group. Chromium allergy was most prevalent in patients in their 50s (8.4%). The prevalence of chromium allergy was 13.5% during 1998-2002 but decreased to 5.4% during 2010-2014.

**Conclusion:** The characteristics of patients with chromium allergy suggested that chromium exposure in daily activities, including leather exopsure, is more relevant than occupational exposure in most patients. The prevalence of chromium allergy has been decreased in Korea, which may be an effect of voluntary regulation of chromium extent in cement by manufacturers

## A467 Efficacy and Safety of Oral Acitretin in Chronic Hand Eczema

### Hyun-Ju Jin^1^, Jin-Hwa Son^1^, Jeong-Min Kim^2^, Gun-Wook Kim^1^, Je-Ho Mun^2^, Margaret Song^1^, Hyun-Chang Ko^2^, Moon-Bum Kim^1^, Hoon-Soo Kim^1^, Byung Soo Kim^1^

#### ^1^Pusan National University, South Korea; ^2^Pusan National University Yangsan Hospital

##### **Correspondence:** Hyun-Ju Jin – Pusan National University, South Korea

**A) Background:** Chronic hand eczema (CHE) may severely reduce patient’s quality of life. CHE refractory to topical corticosteroids currently have limited treatment options suited for chronic use. In demand of safe new therapy, oral alitretinoin, 9-cisretinoic acid, was proposed, but the economic burden limits its broad use. Acitretin is a well-known second generation vitamin A derivative, widely used in palmoplantar psoriasis with less cost. The investigation was performed to assess the efficacy and safety of acitretin in chronic hand eczema patients.

**B) Methods:** Total of 25 patients diagnosed with CHE were enrolled in the prospective open-label study. All patients were administered with oral acitretin, start dose of 10mg twice daily and tapered to 10mg once daily, during 8 week period. CHE severity was evaluated using a hand eczema severity index and 5-grade physicial global assessment (PGA).

**C) Results:** Severity of hand eczema decreased by 58% in average. The improvement was more remarkable in hyperkeratotic type than other types of hand eczema (p<0.004). Clinical responses, defined as clear or almost clear, were achieved in up to 36% of patients. No serious side effects were reported except of mild cheilitis in 2 patients and abdominal discomfort in 1 patients.

**D) Conclusion:** Oral acitretin is well-tolerated with effectively improving chronic hand eczema, especially in hyperkeratotic type.

## A468 **Atypical Antipsychotics and Anticholinergic Agents Mimicking Anaphylaxis**

### Sheryl Van Nunen^1^, Dinh Van Nguyen^2^, Anthony Elias^3^, Susannah Olivia Lauer^4^

#### ^1^Sydney Medical School - Northern, University of Sydney, Australia; ^2^The University of Sydney; ^3^Royal North Shore Hospital; ^4^Griffith University

##### **Correspondence:** Sheryl Van Nunen – Sydney Medical School - Northern, University of Sydney, Australia

**Introduction**

Laryngeal dystonia is a rare, potentially fatal complication of therapy with atypical antipsychotics or anticholinergic agents. We report a case of laryngeal dystonia due to each of these agents, both of which were ascribed initially to anaphylaxis.

**Case 1**

A 28-year old man presented to an Emergency Department after progressively developing a sensation of severe throat swelling and slurred speech following ingestion of his regular ziprasidone. Intramuscular adrenaline was given without benefit for a putative diagnosis of anaphylaxis. His symptoms gradually resolved over hours. Mast cell tryptase levels were normal and allergen-specific IgE to temporally relevant allergens was undetectable. He reported a similar episode a decade prior after taking risperidone. Allergist review noted diagnostic criteria for anaphylaxis were lacking and ziprasidone had been taken after a night of excessive alcohol intake. His recurrent, atypical antipsychotic- induced laryngeal and pharyngeal dystonia was managed successfully by recommencing ziprasidone on condition that he avoided excessive alcohol and took the drug at a regular time.

**Case 2**

A 26 year-old man presented to an Emergency Department with a sensation of throat and tongue swelling and voice hoarseness. He had earlier self-medicated with intra-muscular metoclopramide, intra-muscular prochlorperazine and oral ondansetron for intractable vomiting due to cyclical vomiting syndrome. No evidence of haemodynamic compromise, urticaria or airway oedema was found. Promethazine & intravenous hydrocortisone were given for suspected anaphylaxis, followed by low dose midazolam aimed at laryngeal relaxation. His symptoms improved rapidly thereafter. Mast cell tryptase level was normal during the event and allergen-specific IgE to temporally relevant allergens was undetectable. A similar episode a few years previously followed self-administered large doses of metoclopramide. This case represents recurrent laryngeal dystonia in response to large doses of metoclopramide, possibly aggravated by co-administration of prochlorperazine. Fortuitously, the anticholinergic effect of the promethazine would have helped relieve the dystonia.

**Discussion**

Both previous reports of ziprasidone- induced laryngeal dystonia followed intramuscular use (Mellecheruvu et al. 2007). Our first case is the first report of orally administered ziprasidone causing laryngeal and pharyngeal dystonia. Our second case is the fourth report of metoclopramide-induced laryngeal dystonia. Given that the rightful emphasis is on first line treatment with adrenaline for anaphylaxis, awareness of the propensity for these drugs to provoke such reactions is crucial to timely specific treatment with anticholinergics, along with increased suspicion when predisposing factors (youth, male gender, alcohol ingestion, previous reactions) are present.

## A469 Introducing Reach (Reliable Estimation of Atopic dermatitis in ChildHood): Novel, Questionnaire-Based Diagnostic Criteria for Childhood Atopic Dermatitis

### Seung-Chul Lee^1^, Ho-June Lee^1^, Jung Min Bae^2^

#### ^1^Chonnam National University Medical School, South Korea; ^2^St. Vincent’s Hospital

##### **Correspondence:** Seung-Chul Lee – Chonnam National University Medical School, South Korea

**Background:** Questionnaire-based diagnostic criteria for atopic dermatitis (AD) are limited by their low diagnostic accuracy at the individual level.

**Objective:** To develop novel, questionnaire-based diagnostic criteria to improve the accuracy of estimations of AD prevalence and individual diagnoses of childhood AD in epidemiologic surveys.

**Methods:** Candidate questions for questionnaire were selected by preliminary surveys. The Reliable Estimation of Atopic dermatitis in ChildHood (REACH) diagnostic criteria were derived from a population-based survey.

**Results:** Thirteen questions (2 major and 11 minor criteria) were included in the final version of the questionnaire. The REACH diagnostic criteria were defined as fulfillment of the two major criteria (2M) for ‘typical AD’, or one major plus four or more minor criteria (2M + 4m) for ‘atypical AD’, from the derivation set (*n*=1,129). The REACH criteria demonstrated superior performance compared to the ISAAC both in terms of estimated AD prevalence and diagnostic accuracy in a validation set (*n*=1,191).

**Limitations:** Further validation is required using a more-diverse range of populations.

**Conclusions:** We propose that the comprehensive, questionnaire-based REACH diagnostic criteria can be used for childhood AD, not only in epidemiological surveys but also in the context of personalized medical advice.

## A470 Comprehensive Assessment to Identify the Causative Factors in Oral Allergy Syndrome

### Emi Ono

#### Osaka University, Japan

**Background**

Oral allergy syndrome (OAS) may be the most common food-related allergy in adults. Even of OAS, pollen-food allergy is complicated because of cross-reactivity to proteins.

The ImmunoCAP ISAC(Immuno Solidphase Allergen Chip) allows detection of specific IgE to 112 molecular components from 51 allergenic sources. This new approach helps to clarify the molecular bases of primary sensitization and cross-reactivity phenomena. We used this approach to analyze allergen component comprehensively.

**Methods**

We retrospectively analyzed sera from 11 patients who had been examined based on suspicion of allergy at the Department of Dermatology of the Osaka University.

Of the 11 patients, 10 patients have symptom such as pruritus or tingling of the lips, oral mucosa, and throat. One patient did not have symptom.

ImmunoCAP ISAC was used for screening allergen component and compared with other tests results such as skin prick test. To specigy the antigen, ELISA and western blot and blood testing were also performed.

**Results**

This test is useful for the cross reaction of an allergy patient sensitized to a large number of allergens, the grasp of the sensitization pattern with the common antigen.

Younger patients, who are under 14y.o, had the lower number of positive components than that of over 14y.o.

Tick, cat and dog related components were characteristic patients with dermatitis. Result of exclude the tick, cat and dog related components, number of positive components of patients with AD or eczema (contact dermatitis and PPP) were higher than with no dermatitis.

**Conclusion**

We found that the both age and dermatitis were the factors influencing to the causative factors in OAS.

Our finding suggest that the number of the positive components was higher in the subject accompanied with AD compared to the subjects without AD. Thus, it assumed that the subjects with AD might be highly susceptible to OAS.

## A471 Comparison of Interpretation Methods in Allergic Skin Test

### Sung-Hwa Dong^1^, Tae Kyung Koh^1^, Young Seok Byun^1^, Sung Wan Kim^1^, Joong-Saeng Jo^1^, Chul Kwon^1^, Kun Hee Lee^2^

#### ^1^Kyung Hee Medical Center, South Korea; ^2^Kyung Hee University Hospital at Gangdong

##### **Correspondence:** Sung-Hwa Dong – Kyung Hee Medical Center, South Korea

**Objectives**

Allergic skin test is a commonly used test to evaluate an immediate immune response either by skin prick test (SPT) or intradermal test. SPT is widely used Several methods have been proposed to interpret SPT. However, there has been no comparison study about inter-test relationship. The aim of this study compare three different interpreting methods and define the relationship among them.

**Methods**

We examined SPT to 147 patients who visited allergy clinic complaining of nasal symptoms from January to December 2014. Three different interpreting methods were used for defining positivity in SPT such as interpretation by comparing wheal sizes of allergen and histamine, the biggest diameter of the wheal size more than 3 mm, and the biggest diameter of the erythema greater than 15 mm. And we compared the positivity among 3 different interpretation methods. The validity of each interpreting method was evaluated by comparing positivity to TNSS.

**Result**

Positivity in SPT were reported as 48.2% in A/H ratio method, 64.0% in Wheal size method and in Erythema size method, respectively. Subjects who showed negative result in A/H ratio method have positive results in Wheal size method and Erythema size method were 15.8%, respectively. Subjects showing negative result in A/H ratio but positive result in Erythema size and Wheal size method had significantly higher rhinorrhea, nasal obstruction and TNSS than subjects showing both negative results.

**Conclusion**

Comparing TNSS in each method, TNSS of SPT positive group is higher than those of negative group, except TNSS of Nasal obstruction of A/H method. Thus diagnosing allergic rhinitis patients, we may consider Wheal size method and Erythema size method than A/H ratio method.

## A472 Refraining Aminophylline Use Increases Hospitalization Among Children with Acute Asthma: A 10-Years Retrospective Cohort Study

### Li-Fan Liu

#### College of Medicine, National Cheng Kung University, Taiwan

Asthma is a leading cause of emergency department (ED) visits and hospitalizations for children in developed countries. The study was to determine which variables and treatment protocols were associated with hospitalization among children with asthma-related ED visits. We retrospectively collected data from the clinical records of children aged 2 to 18 years who were diagnosed with asthma and treated in the ED of a referral tertiary center in southern Taiwan from June 2004 to May 2014. Regression-based analyses were performed to determine factors associated with hospital admissions, and to investigate whether administering intravenous aminophylline was associated with hospital admissions from asthma-related ED visits. A total of 2,375 patients were included in the analysis. Body weight (adjusted odds ratio [AOR]=0.97; 95% confidence interval [CI]=0.95-0.99), fever episode (AOR=1.96; 95% CI=1.47-2.60), pulse rate (AOR=1.02; 95% CI=1.02-1.03) and previous hospitalization due to asthma episode (AOR=1.39; 95% CI=1.03-1.88) were significantly associated with hospital admission among children with asthma-related ED visits during the whole study period. Hospital admission rates sharply increased from 9.9% + 3.2% in 2004-2008 to 24.3% + 2.8 % in 2009-2013. Children with a hospital admission during the second 5-year time period (2009-2013) received significantly less aminophylline treatment than those without a hospital admission in the first 5-year time period (2004-2008) (AOR=0.30; 95% CI=0.17-0.51). In conclusion, we found body weight, fever episode, pulse rate and previous hospitalization were predictive factors of hospitalization in children with asthma exacerbation at ED visits. Refrain intravenous aminophylline use may increase hospital admission among children with acute asthma.

## A473 The Prevalence and Risk Factors of Atopic Dermatitis from Nationwide Study for Korean School Students

### Sunghee Lee

#### Department of Pediatrics, Childhood Asthma Atopy Center, Environmental Health Center, Asan Medical Center, University of Ulsan College of Medicine, South Korea

**Purpose:** Atopic dermatitis (AD) has increased in worldwide and nationwide. However, we do not have well designed randomized national epidemiologic studies, but only some selected data about prevalence of AD. We investigated the prevalence of AD and risk factors in nationwide random children and adolescents of Korea.

**Methods:** We conducted a questionnaires survey with 1,820 children, aged 6 to 19 years old, nationwide in Korea from 2012 to 2013. The subjects were selected by a stratifying sampling method by school grade and five regions. The population was composed of 914 elementary school, 445 middle school, and 461 high school students. Current AD was defined as having recent AD symptoms within 12 months and diagnosed as AD from physicians ever. Atopic sensitization was described by skin prick test for 18 common allergens.

**Results:** The prevalence of current AD and recent AD treatment within 12 months in elementary school, middle school, and high school was 18.8%, 14.6%, and 13.4%, and 12.3%, 8.6%, and 9.5%, respectively. Risk factors, based on multivariate analysis, for current AD from merged data included household pet keeping (aOR, 1.35; 95% CI, 0.95-1.89) as well as parental allergic disease (aOR, 1.87; 95% CI, 1.44-2.44), mold exposure during infancy (aOR, 1.42; 95% CI, 1.06-1.89), recent mold exposure within 12 months (aOR, 1.54; 95% CI, 1.16-2.03), and atopic sensitization (aOR, 1.47; 95% CI, 1.12-1.93).

**Conclusion:** The nationwide prevalence of current AD in elementary school, middle school, and high school students were 18.8%, 14.6%, and 13.4%, respectively. The risk factors for current AD included parental allergic disease, mold exposure during infancy, recent mold exposure within 12 months, atopic sensitization, and household pet keeping.

**Funding sources:** This study was supported by the National Institute of Environmental Research(NIER)

## A474 Probiotic Recombination Protein Effect on Atopic Dermatitis

### Wei-Leng Chen^1^, Jiu-Yao Wang^2^

#### ^1^National Cheng Kung University, Taiwan; ^2^National Cheng Kung University Hospital

##### **Correspondence:** Wei-Leng Chen – National Cheng Kung University, Taiwan

Recently, probiotic treatment becomes a new therapy for allergic diseases. Based on the previous research from our lab, we discovered the enzyme dehydrogenase is the major material of probiotic extract. We want to investigate what the recombinant enzyme effect on allergic disease. Then we used *Dermatophagoides pteronyssinus* (Der p)-induced atopic dermatitis (AD) mouse model to demonstrate the effect of probiotic recombinant protein. We found probiotic recombinant protein could decrease mouse serum total IgE and Der p-specific IgE. In the on other hands, probiotic protein also could recover the physiological function of skin, decrease Langerhans cell infiltration into epidermal and attenuate cytokine thymic stromal lymphopoietin (TSLP) production in skin tissue. Next, we postulate the recombinant protein could enhance physiological function of the anatomical barrier, the skin. We used the wound healing model of human keratinocyte, HaCaT cell and pre-treated cell with the enzyme before wound formation. The experiment determined the enzyme promote wound healed faster than untreated group. Through these results, we may explore the effect and mechanisms of recombination dehydrogenase in Der P.-induced AD model.

## A475 Allergic Sensitization Status in Various Inflammatory Skin Diseases

### Youin Bae, Gyeong-Hun Park

#### Hallym University Dongtan Sacred Heart Hospital, South Korea

##### **Correspondence:** Youin Bae – Hallym University Dongtan Sacred Heart Hospital, South Korea

**Background:** A variety of skin diseases are known to be associated with allergic inflammation, but there has not been sufficient data on the status of allergic sensitization in adult patients with various inflammatory skin diseases.

**Objective:** We sought to compare the status of immunoglobulin E sensitization in various inflammatory skin diseases.

**Methods:** Serum specific immunoglobulin E (IgE) levels for 41 common food and aeroallergens were measured in a total of 380 adult patients who were diagnosed as allergic contact dermatitis (n = 31), atopic dermatitis (n = 44), acute urticaria (n = 103), chronic urticaria (n = 109), irritant contact dermatitis (n = 19), other eczema (n = 47), and pruritus (n = 27). Sensitization to allergens was defined as specific IgE levels equal to or greater than 0.35 kU/L.

**Results:** There was no statistically significant difference in the numbers of the sensitized allergens between the skin conditions (*p* = 0.176). Sensitizations to milk and beef antigens were higher in atopic dermatitis, allergic contact dermatitis, irritant contact dermatitis, and pruritus compared to the others (*p* = 0.041 and 0.006, respectively). In addition, sensitizations to yeast, cladosporium, and candida were significantly higher in atopic dermatitis compared to the others (*p* = 0.001, 0.027, and <0.001, respectively). However, there was no statistically significant difference in the rate of sensitization to the other allergens.

**Conclusions:** The overall status of allergic sensitization was similar in various inflammatory skin diseases. However, several allergens were more closely associated with specific skin conditions.

## A476 Two Cases of Good’s Syndrome: A Rare Acquired Immunodeficiency Associated with Thymoma

### Suk Yeon Kim

#### Hallym University Sacred Heart Hospital, South Korea

Good’s syndrome is an acquired immunodeficiency state associated with thymoma. It is characterized by recurrent infection, autoimmune disease, and immunologic abnormality. The insufficient immunity can be supported by intravenous immunoglobulin(IVIG) replacement therapy. We describe 2 patients who presented with cough and dyspnea caused by Pneumocystis jiroveci pneumonia and Cytomegalovirus pneumonia respectively after thymectomy for a thymoma. Immunologic study revealed hypogammaglobulinemia with very low B-cell count, consistent with Good’s syndrome. They were successfully treated with trimethoprim/sulfamethoxazole and gancyclovir respectively, and now they are doing well without additional infections, having regular IVIG replacement.

## A477 IL-23 Has a Role to Play in the Development of Asthma in Short-Term Cigarette Smoke Exposure-Induced House-Dust Mite Allergic Model

### Hyun Seung Lee^1^, Woo-Jung Song^2^, Mingyu Kang^3^, Han-Ki Park^1^, Da-Eun Park^1^, Hye-Ryun Kang^4^, Heung Woo Park^2^, Yoon-Seok Chang^2^, Hye-Young Kim^5^, Kyung-up Min^6^, Sang-Heon Cho^6^, Ji-Won Lee^1^, Boram Bae^1^, Jung-Won Park^7^

#### ^1^Institute of Allergy and Clinical Immunology, Seoul National University Medical Research Center, South Korea; ^2^Seoul National University College of Medicine; ^3^Chungbuk National University Hospital; ^4^Seoul National University Hospital; ^5^Medical Research Institute of Pusan National University Hospital; ^6^Seoul National University Medical Research Center; ^7^Division of Allergy and Immunology, Department of Internal Medicine

##### **Correspondence:** Hyun Seung Lee – Institute of Allergy and Clinical Immunology, Seoul National University Medical Research Center, South Korea

**Background:** Recently, it has been reported that cigarette smoke exposure during allergen sensitization period facilitates the development of house dust mite (HDM) sensitization and subsequent allergic asthma. However, the mechanisms remain elusive. Several researchers have recently shown that IL-23 is involved in antigen-induced airway inflammation. Therefore, we hypothesized that IL-23 is involved in this pathway.

**Objective:** To evaluate roles of IL-23 in short cigarette smoke exposure-induced HDM-allergic asthma mouse model

**Methods:** BALB/c mice were exposed to HDM and/or cigarette smoke extracts (CSE) during HDM sensitization period (day 1, 2, 3, and 14). Anti-IL-23p19 antibody was given during the sensitization period. And we analyzed several asthmatic phenotypes after last allergen challenge. In addition, we also analyzed the change of DC activation in LDLN and cytokines profile after last sensitization.

**Results:** CSE exposure during sensitization period promoted the development of HDM-allergic sensitization and asthma phenotypes. The proportion of innate lymphoid type 2 cells also increased by CSE and HDM co-exposure, compared to a single exposure. Anti-IL-23 antibody treatment during allergen sensitization period significantly diminished several phenotypes of allergic asthma. Anti-IL-23 treatment also reduced the recruitment of innate lymphoid type 2 cells. Along with, the activation of DC in LDLN was significantly reduced by anti-IL-23 after last sensitization.

**Conclusion:** IL-23 may play a significant role in the development of short cigarette smoke-induced allergic sensitization and asthma.

## A478 The Relationship Between the Relevance of Allergic Disease and the Value of Non-Specific IgE

### Yasuhiro Suzuki

#### Tokyo Medical and Dental Unibersity, Japan

**Background**

In outpatient clinic, we meet many patients who are suffering from allergic disease. In our department, we usually ask the patient, who is suffering from allergic diseases such as allergic rhinitis, to take blood test after giving informed consent. We calculate the ratio of eosinophils, the level of non-specific and specific IgE, and so on. However, the ratio of eosinophils is fluctuated, and there seems to be no connection between the level of non-specific IgE and the severity of allergic disease. In this study, we retrospectively studied whether the results of blood test are associated with the relevance of allergic disease or not.

**Method**

We sampled 64 patients (32 men and 32 women) who visited our department and took blood test from January 2014 to December 2014. We measured the ratio of eosinophils, the level of non-specific and specific IgE, and so on. In this study, we divided these patients into 4 groups according to the level of non-specific IgE (less than 100 <Group 1>, 100 or more and less than 500 <Group 2>, 500 or more and less than 1000 <Group 3>, more than 1000 <Group 4>). Then we investigated them from several aspects.

**Results**

The most common disease in Group 1 is seasonal allergic rhinitis (44.7%), followed by vasomotor rhinitis (28.9%) and perennial allergic rhinitis (21.1%). Group 2 has the same tendency as Group 1 (seasonal allergic rhinitis (47.1%), vasomotor rhinitis (29.4%), perennial allergic rhinitis (23.5%)). Group 3 and Group4 have small number of patients. It is difficult to calculate the percentage of diseases in both groups, but seasonal allergic rhinitis and chronic sinusitis were tending to have high relevance.

Next, we evaluated the relationship between the level of non-specific IgE and the relevance of diseases. In Group 4, the relevance of chronic sinusitis and perennial allergic rhinitis was significantly higher than Group 1. Eosinophilic sinusitis, seasonal allergic rhinitis, and vasomotor rhinitis had no significant differences.

**Conclusions**

Non-specific IgE level may be related to the severity of chronic sinusitis and perennial allergic rhinitis. Non-specific IgE level may also be useful in order to estimate the future relevance and severity of nasal diseases.

## A479 Two Caces of Prawn Allergy in Adult Patients

### Ismet Bulut^2^, Zeynep Ferhan Ozseker^1^

#### ^1^Sureyyapasa Chest Diseases and Chest Surgery Training Hospital; ^2^Süreyyapa°a Chest Disease and Chest Surgery Training and Research Hospital, Turkey

##### **Correspondence:** Ismet Bulut – Süreyyapa°a Chest Disease and Chest Surgery Training and Research Hospital, Turkey

**M,S, 30 years old, Works at a restaurant.**

**Complaint:** Sneezing, clogged nose, impaired sensitivity of smelling, post nasal drip, sneezing in the mornings.

Sneezing provocated by exposition to prawn smell, itching sensation in the nose, nasal drip, Itching and wetness in the eyes, shortness of breath, pressure sensation in the chest.

**History of Complaints:** Fort he last 5 years, he has been working at the kitchen of a restaurant that serves sea products including prawn (Large Shrimp). For 1 year, he has been experiencing sneezing, post nasal drip and itching sensation in the eyes, all of which are provocated by exposure to smell of prawn while it is being cleaned and prepared. Exposure to prawn smell caused edema and swelling in the lips and eyelids, suggestive of angioedema. Prawn smell inhalation also caused shortness of breath, pressure sensation in the chest, stridor. Medical history also revealed angioedema triggered by intake of appranax (naproxen).

**Laboratory**

Eosinophils %13.83 (0.5-11.0),Total Ig E: 138 IU/ML (1.31-165.3) Specific Ig E ( mixed 5 ) (food panel 2): (morino fish, Prawn, shell, tuna fish, salmon fish): 26.5 KU/L (0-0.35 KU/L), wchich indicates strong positive result.Inasmuch as our patient had allergic rhinitis, a skin-prick test with respiratory allergen panel was conducted in order to determine the causative inhalatory allergen. The results were as follows: Der P: 13x11 mm, Der F: 17x11 mm, B.Germenica: 7x7 mm, Negative Control: (-), Histamin.7x7 mm.

We subjected our patient to prick-to-prick test: Prawn: 37x18 mm, strong positive, Negative control: (-), Histamin : 7x7 mm.

**A.O , 27 years old male patient**

**Complaint:** The patient has been experiencing sensation of itching in the mouth, swelling in tongue and throat, difficulty to swallow; all of which occured following consumption of prawn.

**History of complaints**

None of these complaints occured before the age of 27. The patient has been experiencing those symptoms fort he last 1 year, and upon consumption of prawn.

While our patient experienced only swelling in the tongue and throat, along with an itching sensation in these localisations when he consumed prawn in the restaurant A; he experienced swelling in tongue and throat, laryngeal edema accompanied by dizziness and sensation of fainting and hypotension, suggesting anaphylactic reaction, when he consumed prawn in the restaurant B.The patient favoured prawn a lot and did not quit consuming prawn in spite of all these complaints. He had about 3 or 4 such allergic episodes when he had applied to our clinic.

**Result**

The patient was issued an adrenalin auto-injection certificate. He was instructed about auto injection technique.

He was adviced not to ever consume prawn from now on. He was also adviced to be cautious about restaurants serving prawn since certain kitchen equipments might be contaminated with prawn particles.

## A480 Early Gut *Bifidobacterium Breve* and *B. Catenulatum* Colonisation Differentially Modulate Eczema Risk in Children at High-Risk of Developing Allergic Disease

### Intan Hakimah Ismail^3^, Robert Boyle^1^, Paul Licciardi^2^, Frances Oppedisano^2^, Roy Robins-Browne^2^, Mimi Tang^2^

#### ^1^Imperial College London, UK; ^2^Murdoch Childrens Research Institute, Australia; ^3^Universiti Putra Malaysia (UPM), Malaysia

##### **Correspondence:** Intan Hakimah Ismail – Universiti Putra Malaysia (UPM), Malaysia

**Background:** The increasing prevalence of allergic disease has been attributed in part to reduced microbial exposures associated with a modern lifestyle. An altered compositional signature and reduced diversity of the intestinal microbiota are linked to development of allergic disease. We investigated the relationship between dominant *Bifidobacterium*species in stool during the early postnatal period and subsequent development of eczema, IgE-associated eczema and sensitisation in the first year of life.

**Methods:** Faecal samples were collected at age 1 week, 1 month and 3 months from infants at high risk of allergic disease, who were followed prospectively to age 12 months. *Bifidobacterium*species were analysed by quantitative PCR and terminal restriction fragment length polymorphism. Infants were examined at 3, 6 and 12 months, and skin prick test performed at 12 months. Eczema was diagnosed according to the UK-Working Party criteria.

**Results:** The presence of *B. catenulatum* at 3 months was associated with a higher risk of developing eczema (OR_adj_ = 4.5; 95% CI 1.56 to 13.05, P_adj_ = 0.005). Infants colonised with *B. breve* at 1 week (OR_adj_ = 0.29; 95% CI 0.09 to 0.95, P_adj_ = 0.041) and 3 months (OR_adj_ = 0.15; 95% CI 0.05 to 0.44, P_adj_ = 0.00001) had a reduced risk of developing eczema. Furthermore, the presence of *B. breve* at 3 months was associated with a lower risk of atopic sensitisation at 12 months (OR_adj_ = 0.38; 95% CI 0.15 to 0.98, P_adj_ = 0.046). *B. breve*colonisation patterns were influenced by maternal allergic status, household pets and number of siblings.

**Conclusions:** There are temporal variations of *Bifidobacterium* colonisation patterns early in life and these variations differentially modulate later development of eczema and/or atopic sensitisation in infants at high risk of allergic disease. Modulation of the early microbiota may provide a means for prevention of eczema in high risk infants.

## A481 Effects of Chronic Repeated Exposure of Staphylococcal Enterotoxin B on Allergic Asthma Model in Mice

### Ji Won Lee^1^, Hyun Seung Lee^2^, Mingyu Kang^3^, Da-Eun Park^2^, Han-Ki Park^2^, Soon-Hee Kim^4^, Woo-Jung Song^5^, Hye-Ryun Kang^6^, Heung Woo Park^5^, Yoon-Seok Chang^5^, Chang-Han Park^7^, Suk-Il Chang^7^, Sook-Hee Song^8^, Kyung-up Min^1^, Sang-Heon Cho^1^, Boram Bae^2^

#### ^1^Seoul National University Medical Research Center, South Korea; ^2^Institute of Allergy and Clinical Immunology, Seoul National University Medical Research Center; ^3^Chungbuk National University Hospital; ^4^Seoul National University Bundang Hospital; ^5^Seoul National University College of Medicine; ^6^Seoul National University Hospital; ^7^Sung-Ae General Hospital; ^8^Seoul Medical Center

##### **Correspondence:** Ji Won Lee – Seoul National University Medical Research Center, South Korea

**Background:** Recent epidemiological studies demonstrate significant associations of Staphylococcal enterotoxins (SE) with asthma. In addition, clinical studies suggest that the enterotoxin exposure could contribute to the development of fixed airway obstruction in asthma, leading to a COPD-overlap phenotype. In previous experiments of mice, a short-term exposure to staphylococcal enterotoxin B (SEB) induced extensive inflammatory responses in lungs, including lymphocytes, neutrophils and eosinophils. Therefore, we hypothesized chronic repeated exposure to SEB could induce tissue remodeling in lower airways.

**Objective:** To investigate effects of repeated SEB exposure on airway remodeling in OVA-induced chronic allergic asthma model.

**Methods:** BALB/c mice were intra-peritoneally sensitized with ovalbumin (OVA)-aluminum hydroxide at days 0 and 14, and were intra-nasally challenged with OVA and/or SEB for the next 6 weeks(50 ug OVA, 10 or 100 ng of SEB, 3 times per week over a period, total 18 exposure). At 8 weeks, mice were sacrificed and assessed for histopathology, bronchoalveolar lavage fluid (BALF) cell counts, lung cytokines, methacholine airway hyper-responsiveness (AHR), and serum immunoglobulins.

**Results:** OVA sensitization followed by repeated OVA exposure resulted in the development of Th2 asthma and inflammation, including BALF cell counts, lung cytokines, histopathology, AHR and serum immunoglobulins. However, repeated SEB exposure did not aggravate OVA-induced pathologic changes, or induce any remarkable pathologic changes when administered alone. Serum total IgE levels were also not influenced by repeated SEB exposure. Rather, chronic repeated SEB-exposed mice showed lower levels of lung inflammation and remodeling, but higher levels of IL-10 and TGF-β, compared to non-SEB-exposed mice.

**Conclusion:** The present study demonstrates unpredicted suppressive effects of chronic repeated exposure of SEB in allergic asthma models, leading to the speculation that SEB exerts bi-directional effects depending on the timing of exposure. The mechanisms of anti-inflammatory effects warrant further investigation.

## A482 Skin Prick Test Result and Allergen Immunotherapy in Children with Allergic Rhinitis

### Grace Shieh

#### UST Hospital, Philippines

**Background:** Allergic rhinitis represents a global health problem that causes major illness and disability worldwide. Immunotherapy is effective in the management of allergic rhinitis. Skin prick test is a reliable method to diagnose IgE-mediated allergic disease. Up-to-date, no biomarker has yet been validated to be a good predictor of clinical response to allergen immunotherapy.

**Method:** This is a cohort study to determine the association of skin prick test result to allergen immunotherapy efficacy in children with allergic rhinitis. Patients seen at a tertiary hospital diagnosed with allergic rhinitis and on allergen immunotherapy were included in the study. Skin prick test results were recorded and total nasal symptom score (TNSS) were obtained at the start of immunotherapy and monthly for six months. The correlation of the baseline skin prick test to the change in TNSS for each monthly endpoint was done to check for the association between the baseline skin prick test and the efficacy of immunotherapy measured as the improvement in TNSS scores.

**Results:** A total of 18 patients who have completed at least six months of immunotherapy were included in this study. Average age is 13.22 + 3.62 years old, and were predominantly male (11 out of 18, 61.1%). The average skin prick test to *D.farinae* was 7.72 ± 3.80 mm, while that of *D.pteronyssinus* was 8.56 ± 4.34 mm. At baseline, the TNSS of the subjects had a median score of 7.5 which gradually decreased to a median score of 3 by the sixth month of immunotherapy. There was generally low and inverse correlation between *D.pteronyssinus* immunotherapy and skin prick test results for the first four months of immunotherapy, after which, there was a direct and moderate correlation from the fourth to sixth month of immunotherapy (*p=0.3128)*, however it was not statistically significant. There was a low and inverse correlation between *D.farinae* immunotherapy and skin prick test results for the first four months of immunotherapy, after which, there was a direct but low correlation from the fifth to sixth month of immunotherapy (*p=0.2550*).

**Conclusion:** The larger the size of the skin prick test to *D.pteronyssinus*, the greater the improvement of the total nasal symptom score from the baseline at six months of immunotherapy.

## A483 Role of Brp-39 in RSV-Induced Airway Inflammation in Mice

### Min Jung Kim, Jung Yeon Hong, Seo Hee Yoon, Doo Hee Shim, In Suk Sol, Yoon Hee Kim, Mi Na Kim, Kyung Eun Lee, Kyung Won Kim, Myung Hyun Sohn, Kyu-Earn Kim, Jae Myun Lee

#### Yonsei University College of Medicine, South Korea

##### **Correspondence:** Min Jung Kim – Yonsei University College of Medicine, South Korea

**Background:** The chitinase 3-like 1 (CHI3L1), has been demonstrated its requirement for optimal allergen sensitization and Th2 inflammation in various chronic inflammatory disease including asthma. But, the role of Chi3L1 in airway hyperresponsiveness (AHR) induced by respiratory viruses has not been proved yet. The purpose of this study is to figure out the relationship between BRP-39, mouse chitinase 3-like 1 protein (CHI3L1), and Respiratory syncytial virus (RSV)-induced AHR.

**Methods:** We used C57BL/6 mice and BRP-39 null mice. Mice were inoculated with live A2-strain RSV and control PBS. Methacholine challenge test to measure airway resistance worked on day 5 after inoculation. And bronchoalveolar lavage fluid (BALF) samples were obtained and lung specimens were also harvested on days 5 after inoculation to assess lung inflammation, cytokine expression and BRP-39 production. BRP-39 expression was evaluated by ELISA. RSV loads were assessed by culture and real-time polymerase chain reaction (PCR). Histological evaluations of H&E and PAS staining were used to evaluate inflammation and tissue remodeling.

**Results:** Level of BRP-39 in BALF was significantly increased in wild-type (WT) mice after RSV infection, but not observed in BRP-39 null mice. Inflammatory changes induced by RSV infection were less in BRP-39^-/-^ mice rather than WT mice. In WT mice, RSV infection caused loss of body weight and significant increase of total cells, macrophages and neutrophils in BALF. And exaggerated AHR was also noted in WT mice after RSV infection. But, BRP-39^-/-^ mice showed decreased responses in each of these parameters. Between RSV infection groups, histological tissue inflammation was also decreased in BRP-39 ^-/-^mice.

**Conclusions:** The role of early-onset wheezing with respiratory viral infections in childhood asthma inception has received attention. RSV has known as a significant risk factor for asthma that extends into adolescence and adults and its mechanism is still under investigation. In this study, expression of BRP-39 increased by RSV infection in mice. And inflammatory changes and AHR induced by RSV were decreased in BRP-39 null mice. These findings suggest Chi3L1 could contribute to airway inflammation induced by RSV infection in mice. Furthermore, Chi3L1 might be considered as a therapeutic target against viral wheezing or virus-induced asthma.

## A484 Long-Term Outcomes of Twenty-Four Adults with Primary Immunodeficiency from a Single Centre in Singapore

### Hiok Hee Chng

#### Tan Tock Seng Hospital, Singapore

**Background:** Primary immunodeficiency diseases (PID) in adults are often under-recognised resulting in delay in diagnosis and significant morbidity and mortality. We review the long-term outcomes of adults with PID from the Tan Tock Seng Hospital PID Registry.

**Methods:** Chart review of adults with PID onset at age 18 years or above who were longitudinally followed-up from time of diagnosis between 1 Jan 1989 and 1 May 2015.

**Results:** There were 24 patients, 13(54.2%) males and 11(45.8%) females. Eighteen(75%) were ethnic Chinese, 5(20.8%) Malay and 1 Indian. The mean±SD age at diagnosis was 46.3±17.9 years (range 18.4-69.1). By IUIS grouping, antibody deficiencies was most common (14 patients, 58.3%), comprising common variable immunodeficiency [CVID] (8), selective IgA deficiency alone (3), selective IgA with IgG2 and G4 deficiency (1), selective IgG2 and G4 deficiency alone (1) and hypogammaglobulinaemia (1, who had ring chromosome 18). Ten patients (41.7%) had other PIDs, namely immunodeficiency with thymoma (8), chronic mucocutaneous candidiasis [CMC] (1) and hyperIgE syndrome (1).

Half of the patients already had bronchiectasis and 7(29.2%) had chronic sinusitis at diagnosis of PID. Seronegative arthritis developed in 2 females, one was a year following diagnosis of CMC, and the other was coincident with diagnosis of CVID. Systemic lupus erythematosus predated the diagnosis of selective IgA deficiency by 7 years in one patient. There were 7(29.2%) deaths. Four(57.1%) of them had bronchiectasis at diagnosis, and died from chronic respiratory failure with or without pneumonia despite regular adequate IVIG replacement. A 47-year-old female with CVID, who was irregular with IVIG replacement, died from persistent *Elizabethkingia meningoseptica* bacteraemia and septic shock following consumption of frogs. Two other patients who died were males with thymoma and immunodeficiency; one from metastatic thymoma and the other from multiple infections associated with T cell defects (cytomegalovirus colitis and retinitis, disseminated candida esophagitis and hepatitis, ocular toxoplasmosis).

**Conclusions:** The spectrum of adult PID patients managed in our centre is as reported elsewhere. Many of our patients present late with established sinopulmonary complications which contributed to their death.

## A485 Breast Feeding Increases the Risk of Food Sensitization but Does Not Affect Food Allergy in Young Children with Atopic Dermatitis

### Dong Chan Kim, Song-I Yang, Hae Ran Lee, An Deok Seo, So Yeon Lee

#### Hallym University Sacred Heart Hospital, South Korea

##### **Correspondence:** Dong Chan Kim – Hallym University Sacred Heart Hospital, South Korea

**Background**

Breast feeding is recommended for the prevention of allergic diseases, particularly in high risk infants, but the evidence of a protective effect on food sensitization and food allergy(FA) remains elusive. The aim of this study is to investigate the association between breast feeding and food sensitization and FA in children under 2 years with atopic dermatitis

**Methods**

We reviewed the medical records of 384 children with atopic dermatitis under 2 years old who visited our pediatric allergic clinic between March 1, 2009 and December 31, 2014. We assessed symptoms of FA, duration of breast feeding, history of parental allergic diseases, and other concomitant allergic diseases. Laboratory tests including serum total IgE, eosinophil(%), and specific IgE to egg white, milk, soy, wheat, and peanut were measured. On the basis of sensitization (IgE ≥ 0.35 KU/L) and clinical history, children were as having FA and having no FA. Having no FA group was divided with sensitized but tolerant, or not allergic/not sensitized.

**Results**

Two hundred-forty (150 male, 62.5%) were included in the FA group, while one hundred forty-four (92 male, 63.9%) were included in the non-FA group. Children with having FA had a higher blood total IgE levels(238.7±414.6 IU/mL vs. 50.7±96.0 IU/mL, *P*=0.001) and eosinophil(%)(7.0% ± 5.0% vs. 4.7% ±3.4%, *P*=0.001), and more frequent parental allergic history(70% vs. 59%, *P*=0.025). The frequency of breast feeding were relatively higher in FA group than in non-FA group(77.9% vs. 61.8%, *P*= 0.001). Subanalysis between children with FA(n=240) and children with food sensitization but tolerant group(n=53) were not showing significant difference in frequency of breast feeding(77.9% vs. 75.5%, *P*=0.700).

Adjusting for reverse causation by excluding children of age over 6 months or with parental allergic diseases or without parental allergic diseases did not alter these results.

**Conclusions**

Breast feeding increases the risk of food sensitization but does not affect FA in children with atopic dermatitis.

## A486 IgE Immunoadsorption Knocks Down Anaphylaxis

### Maria Cristina Artesani^1^, Paola Francalanci^1^, Lamia Dahdah^1^, Thomas Schreiner^2^, Alessandro Fiocchi^3^

#### ^1^The Bambino Gesù Children’s Research Hospital, Vatican City; ^2^Miltenyi Biotech, Germany; ^3^Ospedale Pediatrico Bambino Gesù, Vatican City

##### **Correspondence:** Alessandro Fiocchi – Ospedale Pediatrico Bambino Gesù, Vatican City

The effects of an immunoadsorption procedure, specifically designed to remove IgE, on food-induced anaphylaxis have never been evaluated. We evaluate the effects of IgE removal on the allergic thresholds to foods.

A six year-old boy with anaphylaxis to multiple foods and steroid-resistant unstable allergic asthma displayed serum IgE levels of 2800 - 3500 kU/L. In order to lower IgE serum concentrations, which could be overridden by a high dose of Omalizumab, 1.5 Vol plasma were exchanged in 6 apheresis sessions. During the procedure, serum IgE levels fell to 309 kU/L. After the procedure:

- the threshold of reactivity to baked milk increased from 0.125 to 5 g of milk protein (full tolerance) after the first session.

- the threshold of reactivity to hazelnut increased from 0.270 to 1.030 g of protein after the first session, to 2.730 g after the sixth session.

Immediately after the sixth IgE immunoadsorption, we started omalizumab therapy. In the following 40 days, the threshold of reactivity to hazelnut increased to 7.730 (full tolerance). Asthma control was obtained, lung function was improved and treatment with Montelukast was stopped. Fluticasone was tapered from 500 to 175 mcg/day. The boy became partially or fully tolerant to all the foods that had caused anaphylaxis and quality of life was improved.

IgE-immunoadsorption, used to establish the starting basis for Omalizumab administration, is able *per se* to increase the tolerance threshold to foods.

## A487 Burden and Correlates of Cigarette Smoking and Respiratory Airway Obstruction: An Observation in Urban Adult Population of West Bengal (India)

### Kaushik Chakraborty

#### Barrackpore Population Health Research Foundation, India

**Background**

Cigarette smoking is the predominate risk factor for developing chronic airway obstruction. Globally COPD is one of the leading causes of mortality and morbidity. The purpose of the study was to assess the burden and correlation of cigarette smoking and respiratory airway obstruction among healthy adults.

**Methods**

The cross-sectional study included 2572 healthy adult without any respiratory illness and non communicable diseases. Smoking and other information were collected using a standardized questionnaire. Lung function test was performed by spirometer and cutoff point derived from GOLD guideline. Multivariate logistic regression analysis was performed.

**Results**

1034 (40.20%) were male and 1538 (59.80%) female. 304 (11.82%) were currently heavy Smokers, among them two were female. Male smokers 421 (40.71%) and female 4 (0.26%). Overall airway obstruction was 646 (25.12%), 297 (28.72%) were male and 349 (22.69%) female.

Compare to never smokers, former smokers [OR=1.75, 95% CI: 1.15, 2.68, p=0.0096] and current heavy smokers [OR=2.01, 95% CI: 1.49, 2.69, p<0.001] were associated with obstruction. Increasing age was associated with increasing obstruction [OR=1.05, 95% CI: 1.04, 1.06, p<.0001]. Obstruction was associated with shortness of breath [OR=1.31, 95% CI: 1.03, 1.65, p=0.0245], cough [OR=1.83, 95% CI: 1.20, 2.80, p=0.0049], phlegm [OR=2.38, 95% CI: 1.53, 3.68, p=.0001], wheezing [OR=3.70, 95% CI: 2.00, 6.85, p<.0001] compare to no obstruction. Smokers with obstruction were associated with phlegm [OR=2.40, 95% CI: 1.09, 5.27, p=0.0294], wheezing [OR=9.61, 95% CI: 2.08, 44.45, p=0.0038] reference to smokers without obstruction. Among no obstruction SOB [OR=1.87, 95% CI: 1.15, 3.08, p=0.0114] and phlegm [OR=2.65, 95% CI: 1.07, 6.57, p=0.0352] were associated with smokers reference to non-smokers.

**Conclusion**

One in four or one quarter of the apparent healthy adults of the cohort suffered from undiagnosed airway obstruction. Association had been documented between cigarette smoking and the development of airway obstruction and symptoms.

## A488 Blood Eosinophils Could Predict Sputum Eosinophilia?: A Comparison Between Asthma and Non-Asthmatic Chronic Cough in the Elderly

### Ha Kyeong Won^1^, Ju-Young Kim^1^, Eun-Jung Jo^2^, Kyoung Hee Sohn^3^, Kyung-Mook Kim^4^, Heung Woo Park^3^, Yoon-Seok Chang^3^, Sang-Heon Cho^5^, Woo-Jung Song^3^, Byung-Keun Kim^6^

#### ^1^Seoul National University Hospital, South Korea; ^2^Pusan National University Hospital; ^3^Seoul National University College of Medicine; ^4^Pogunhan Mom Hospital; ^5^Seoul National University Medical Research Center; ^6^Seoul National University Bundang Hospital

##### **Correspondence:** Ha Kyeong Won – Seoul National University Hospital, South Korea

**Background:** Sputum eosinophilia is a useful biomarker for corticosteroid response in patients with asthma or chronic cough. However, sputum induction requires specialized personnel and facilities and is not always feasible. Recent evidence suggests a moderate degree of diagnostic utility of less invasive biomarkers, such as blood eosinophils, in predicting sputum eosinophilia in asthma. However, none in the literature has examined the diagnostic utility of blood eosinophils for sputum eosinophilia in patients with non-asthmatic chronic cough.

**Methods:** We examined the datasets of elderly asthma and non-asthmatic chronic cough recruited from previously established cohorts. The inclusion criteria were as follows: 1) elderly subjects (≥65 years old), 2) no current oral or inhaled corticosteroids and 3) no significant comorbidities. Sputum eosinophilia was defined as induced sputum eosinophils≥3%. The diagnostic utility was assessed using receiver operating curve (ROC) analyses for blood eosinophils in predicting sputum eosinophilia.

**Results:** A total of 74 elderly asthma and 75 non-asthmatic chronic cough patients were analyzed. There were no significant difference in their demographic profiles (age, gender and smoking) between two groups. Of them, 59 elderly asthma and 45 non-asthmatic chronic cough patients had sputum eosinophilia. In Spearman tests, the correlations between blood eosinophils% and sputum eosinophil% were significant in eosinophilic asthma (r=0.577, p<0.001) but not in non-asthmatic eosinophilic bronchitis (r=0.019, p=0.870). In ROC analyses for sputum eosinophilia, blood eosinophils showed a moderate utility (the area under the ROC curve [AUC] 0.838) in asthma, but no utility (AUC 0.489) in non-asthmatic chronic cough.

**Conclusions:** Unlike asthma, blood eosinophils did not have any diagnostic utility for sputum eosinophilia in non-asthmatic chronic cough. These findings could suggest a different pathophysiology in airway eosinophilic inflammation between two entities.

## A489 Complementary and Alternative Medicine for Allergic Rhinitis in Japan

### Syuji Yonekura, Yoshitaka Okamoto

#### Chiba University, Japan

##### **Correspondence:** Syuji Yonekura – Chiba University, Japan

**Background:** Complementary and alternative medicine (CAM) is extensively used in patients with allergic diseases worldwide. The purpose of this study was to investigate the actual situation of CAM practice in the treatment of allergic rhinitis.

**Methods:** We distributed questionnaires to otolaryngologists at 114 facilities in Japan. The subjects who participated in this study included children < 16 years of age and adults 16 or > 16 years of age diagnosed with allergic rhinitis by otolaryngologists. The survey was performed in the period from September 2007 to August 2009. Furthermore, we performed the same investigation out of the hospital setting, such as during general health examinations. All questionnaires were returned to Chiba University and analyzed.

**Results:** The proportions of patients who had ever experimented with CAM in the hospital survey were 7.1% (225/3,170) and 19.2% (1416/7,363) of children and adults, respectively. Approximately 36.2% of the adult patients thought that the treatments were effective. The main reasons for CAM use were safety, convenience and low price. However, the group who spent more than $1,000 on CAM felt more dissatisfaction and anxiety related to treatment at the hospital. The situation of CAM practice was not consistent and was instead influenced by the backgrounds of the subjects.

**Conclusion:** Many patients who receive CAM report feeling that the effects of treatment provided by hospitals are insufficient and have concerns about the side effects of such treatments. Information regarding standard treatments, as described in the guidelines, should become widely known and diffused, and strong communication with patients should be considered.

## A490 Impact of Cognitive Impairment on Asthma Control Status in Elderly Asthmatics

### Gyu Young Hur^1^, Young Min Ye^2^, Joo-Hee Kim^3^, Ki-Suck Jung^4^, Junga Kim^1^, Jae Jeong Shim^1^, Hae-Sim Park^2^

#### ^1^Korea University College of Medicine, South Korea; ^2^Ajou University School of Medicine; ^3^Hallym University, College of Medicine; ^4^Hallym University Sacred Heart Hospital

##### **Correspondence:** Gyu Young Hur – Korea University College of Medicine, South Korea

**Background:** The assessment of disease severity and courses is important to achieve well-controlled status of asthma. In elderly population, cognitive and physical impairments are noted with aging process, which may impact on asthma control. We aimed to evaluate the impact of cognitive function on the assessment of asthma control in elderly asthmatics.

**Method:** Fifty mild to moderate asthmatics were enrolled over 60 years old. Questionnaires including ACT, asthma specific quality of life (AQOL), and geriatric depression scale (GDS) were performed. Seoul neuropsychological screening battery-dementia version (SNSB-D), Korean version of mini mental status examination (K-MMSE), and Seoul instrumental activities of daily living scale (SI-ADL) were done for neuropsychological assessment.

**Results:** Mean age was 67.0 ± 4.9 years. Thirty patients were female (60.0%). According to GINA, 12(24%) were in well-controlled, and 38(76%) were in not-controlled. However, according to ACT, 37(74%) were in ≥20 group, and only 16(32%) were in <20 group. The sensitivity and specificity of ACT to determine well-controlled asthma were 91.7% and 39.5%, respectively. Overestimation of asthma control status was 56%, using ACT compared to GINA. Regarding neuropsychological assessment, 22(44%) had mild cognitive impairment, 4(8.7%) had dementia, and 17(34%) had depression. Depression was more common in patients with uncontrolled asthma(42.1% vs. 8.3%, *P*=0.039). Total SNSB-D score was significantly higher in patients with ACT≥20 (187.9 vs. 217.3, *P*=0.015). The ACT score was significantly correlated with degree of cognitive function (adjusted using age, sex, education, and GDS; *P*=0.004).

**Conclusion:** There is discrepancy between self-reported ACT and physician’s decision by GINA in the assessment of asthma control in elderly asthma, in which ACT score is affected by cognitive function. Elderly asthmatics with higher cognitive function can achieve better asthma control.

## A491 The Association Between Respiratory Tract Infection and Reactive Oxygen Stress

### Kazuhiro Sekimoto^1^, Kazuko Sugai^1^, Keiji Tsuchimoto^1^, Hiromi Uehara^1^, Masanori Ikeda^2^

#### ^1^National Hospital Organization Fukuyama Medical Center, Japan; ^2^Okayama University Graduate School of Medicine, Dentistry and Pharmaceutical Sciences

##### **Correspondence:** Kazuhiro Sekimoto – National Hospital Organization Fukuyama Medical Center, Japan

**Background:** Many aspects of the relationship between allergic inflammation and reactive oxygen stress are unclear.

**Aims:** To elucidate the associations between respiratory syncytial virus (RSV) infection and reactive oxygen stress and between wheezing illness and reactive oxygen stress.

**Method:** Subjects were 61 children aged ≤4 years who were hospitalized with RSV infection(42 patient; RS group), bronchial asthma without RSV (8 patients; BA group), and acute bronchitis and pharyngitis(11 patient; Br group). Levels of blood nitric oxide (NOx), high mobility group box-1 (HMGB-1), thioredoxin, and eosinophil-derived neurotoxin and urine 8-hydroxydeoxyguanosine (8-OHdG), NOx, biopyrrin, and 8-isoprostanes were measured on admission day(acute phase), on hospital days 3–5(recovery phase), and 1–2 weeks after discharge(late phase). This study was approved by the Ethics Committees of National Hospital Organization Fukuyama Medical Center, and the parents of all subjects provided written informed consentin accordance with the Declaration of Helsinki.

**Results:** HMGB-1 was higher in the RS and BA groups during the recovery and late phases than during the acute phase, and higher during the late phase in the RS and BA groups than in the Br group(RS group: recovery/acute 1.19, late/acute 1.37; BA group: 1.20,1.47; Br group:1.12,1.14,respectively). Urine 8-OHdG was higher in the RS group than in the BA and Br groups during the recovery and late phases (RS group: recovery/acute 4.03, late/acute 1.92; BA group: 1.51,0.61; Br group:1.77,0.93, respectively). No significant differences were found in other biomarkers.

**Conclusion:** In young children with acute respiratory tract illness with wheeze, reactive oxygen stress was high during the recovery and late phases. Lower respiratory tract inflammation might persist after the acute phase leading to bronchial hyperresponsiveness.

## A492 The Risk Factors and Lung Function of Current Allergic Rhinitis Due to Dust Mite Sensitization

### Euncho Chung^1^, Kang Seo Park^2^, Yean Jung Choi^3^, Jeewon Park^1^, Soo-Jong Hong^3^, So Yeon Lee^4^

#### ^1^Presbyterian Medical Center, South Korea; ^2^Pediatrics, Presbyterian Medical Center; ^3^Asan Medical Center; ^4^Hallym University Sacred Heart Hospital

##### **Correspondence:** Euncho Chung – Presbyterian Medical Center, South Korea

**Purpose:** The effect of allergic rhinitis [AR] on pulmonary function and risk factors of AR are controversial. The purpose of this study was to analyse the risk factors and pulmonary function in dust mite sensitized, current AR children who were never diagnosed as having asthma.

**Methods:** A cross-sectional study of 1,792 children aged 9-12 years from Korea was conducted. Demographic and disease related information was obtained via a detailed questionnaire, skin prick test, pulmonary function test, and methacholine challenge test.

**Results:** A total of 672 children were included in the analysis. 583 children who were not sensitized to common 16 allergens and not having any allergic diseases were classified as the control group. 89 children were classified as the current AR with dust mite sensitization group. The binary logistic regression analysis evidenced that non-farming parents(adjusted odds ratio[aOR] 1.95, 95% CI 1.00-3.08 ), no older siblings(aOR 1.99, 95% CI 1.22-3.25), use of antibiotics during infancy(aOR 2.18, 95% CI 1.13-3.70), helminth infection(aOR 2.61, 95% CI 1.13-6.03), low income (aOR 0.33, 95% CI 0.12-0.92), and pet ownership(aOR 0.24, 95% CI 0.11-0.51) were risk or protective factors. There was no difference in spirometry between control and current AR groups. None of children showed bronchodilator response. However, methacholine PC20(provocative concentration of methacholine causing a 20% fall in forced expiratory volume in 1 second[FEV1]) less than 25, less than 16, less than 8mg/mL were 8.5%, 7.1%, 2.1% in control group and 28.7%, 23.0%, 8.0% respectively in allergic rhinitis group (p=0.00).

**Conclusions:** We might reduce the prevalence of dust mite sensitized current AR by controling some environmental factors. Even the spirometry seems to be normal, bronchial hyperresponsiveness occurs more frequently in children with dust mite sensitized AR than normal children.

## A493 Cloning and Expression of Recombinant *Blomia Tropicalis* Dust Mite Allergen Blo t 7

### Alain Jacquet^1^, Arun Buaklin^1^, Nat Malainual^2^

#### ^1^Chulalongkorn University, Thailand; ^2^Mahidol University

##### **Correspondence:** Alain Jacquet – Chulalongkorn University, Thailand

**Background**

*Blomia tropicalis* is a common house dust mite in tropical regions. Although the predominant sensitizations to *Blomia* allergens were clearly demonstrated in some South-East Asian countries as Singapore or Malaysia, the prevalence of *B.tropicalis* hypersensitivities as well as the identification of the major allergens remains to be elucidated in Thailand. The goal of the study is the cloning and expression of recombinant Blo t 7 (rBlo t 7) allergen in *Pichia pastoris*.

**Methods**

The cDNA encoding mature Blo t 7 was amplified from *B. tropicalis* total cDNA template using specific primers derived from the allergen mRNA sequence (GenBank accession number AAQ24545.1). The cDNA was subsequently cloned into the pPicZα A *P.pastoris* expression vector, downstream to the a mating factor leader sequence for protein secretion. *P. pastoris* KM71H cells were transformed with linearized recombinant pPicZα A-Blo t7 plasmid by electroporation. rBlo t 7 expression was subsequently assayed in zeocin-resistance colonies following methanol induction. The recombinant allergen was purified to homogeneity by anion-exchange and gel filtration chromatographies.

**Results**

The amplified cDNA of mature Blo t 7 encoded 195 amino acids. Protein sequence alignment with the mature Blo t 7 reference primary structure (UniprotKB/TrEMBL, accession number A1KXI4) evidenced four point mutations but also a twenty amino acids insertion. Multiple alignments showed that this Blo t 7 sequence segment is a conserved domain shared with other group 7 mite allergens (Tyr p 7, Aca s 7, Lep d 7, Gly d 7, Der p 7 and Der f 7). Mature rBlo t 7 was successfully expressed and secreted from *P.pastoris* following induction with 0.5% methanol at 25°C. The expressed protein migrated onto SDS-PAGE as a 25kD band. The difference with the predicted molecular weight (21.18 kD) could be attributed to glycan structures from the predicted N-glycosylation site (NTT, aa 187-189).

**Conclusions**

The correction of the mature Blo t 7 cDNA sequence together with the successful recombinant allergen production in P. pastoris offers great opportunities to initiate the component-resolved diagnosis of *B. tropicalis* allergy in Thailand.

## A494 Seasonal Variations of Airborne Pollen in Bangalore, India

### Roopashree S

#### University, India

**Abstract:** Using a Burkard 7-day volumetric sampler a survey of airborne pollen grains in Bangalore was carried out from January 2012 – December 2013 to assess the qualitative and quantitative occurrence of pollen grains during different months of the year, and to characterize the pollen seasons of dominant pollen types in the atmosphere of Bangalore City. 38 pollen types were identified out of the total pollen catch of 32144 pollen grains/m^3^. Bulk of the pollen originated from anemophilous trees and grasses. Eight pollen types recorded more than 1% of the annual total pollen catch. *Peltophorum pterocarpum* formed the major component of the pollen spectrum constituting 56 %of the total pollen catch followed by other significant pollen contributors were from the genera such as *Delonix regia, Parthenium hysterophorus, Samanea saman*, Poaceae and *Amaranthus spinosus.* Highest pollen counts were obtained in the month of May and lowest in August. The pollen types recorded marked the seasonal pattern of occurrence in the atmosphere. February-May was the principle pollen season with maximum number of pollen counts and pollen types. September – October was the second pollen season with grasses being the main source of pollen. Airborne pollen spectrum reflected the vegetation of Bangalore city. A significant negative correlation was found of daily pollen counts with minimum temperature, relative humidity and rainfall. The skin prick test was performed on 486 patients with Allergic rhinitis. The test was performed with eleven pollen allergen extracts.

## A495 Pollen Observation and Use of Data

### Kyu Rang Kim^1^, Mijin Kim^1^, Changbum Cho^1^, Baek-Jo Kim^1^, Jae-Won Oh^2^, Mae Ja Han^3^

#### ^1^Korea Meteorological Agency, South Korea; ^2^Hanyang University Kuri Hospital; ^3^Korea Meteorological Administration

##### **Correspondence:** Kyu Rang Kim – Korea Meteorological Agency, South Korea

As there are more and more people who take outdoor activities, pollinosis patients having bronchial asthma, allergic conjunctivitis, and allergic rhinitis are increasing. The pollen is produced by various plants. Especially, anemophilous pollen is mostly related to allergic diseases. In Korea, pollinosis is mainly caused by pollen from both trees in spring and weeds in fall, respectively. Oak, birch, and cedar are the main tree species generating allergenic pollen and ragweed, mugwort, and humulus are the main weed species. National Institute of Meteorological Research (NIMR) and the Korean Academy of Pediatric Allergy and Respiratory Disease have jointly operated the Nation-wide pollen observation network. Currently, fourteen Hirst type traps (Burkard Seven-day recording volumetric spore trap) are operating at twelve cities. Airborne pollen was collected every day from all samplers at all collection sites. Each pollen species was morphologically identified and classified by its size, color, pore shape, and surface pattern. The observed pollen is composed of fifteen tree species including pine, and nine weed species.

Based on the observed data, NIMR has established a system issuing day-to-day warnings of allergenic pollens from trees and weeds and offers the risk index of allergic pollen from a dedicated web page. The pollen risk index has four risk levels as mild, moderate, severe, and dangerous based on the symptom levels of pollen allergy patients. Continuous observation through the pollen monitoring network will support both long-term changes in the allergenic plants and the improvement of the day-to-day pollen forecast.

## A496 The Effect of Cord Serum 25-Hydroxyvitamin D (25(OH)D) on the Development of Atopic Dermatitis in First 3 Years of Life : Cocoa Study

### Hyun-Ju Cho^1^, Youn Ho Shin^2^, Eun Lee^3^, Young-Ho Kim^3^, Darae Lee^3^, Mi-Jin Kang^4^, Song-I Yang^5^, Kangmo Ahn^6^, Kyung Won Kim^7^, Yoon Hee Kim^7^, Hye-Sung Won^3^, Soo Hyun Kim^2^, Suk-Joo Choi^6^, Young Han Kim^7^, Jong Kwan Jun^8^, Eun-Jin Kim^9^, Jeom Gyu Lee^9^, So-Yeon Lee^5^, Soo-Jong Hong^1^, Dongin Suh^10^

#### ^1^Department of Pediatrics, Childhood Asthma Atopy Center, Environmental Health Center, Asan Medical Center, University of Ulsan College of Medicine, South Korea; ^2^CHA Medical Center; ^3^Asan Medical Center; ^4^Asan Institute for Life Science, Asan Medical Center; ^5^Hallym University Sacred Heart Hospital; ^6^Samsung Medical Center; ^7^Yonsei University College of Medicine; ^8^Seoul National University College of Medicine; ^9^Korea National Institute of Health; ^10^Seoul National University Hospital

##### **Correspondence:** Hyun-Ju Cho – Department of Pediatrics, Childhood Asthma Atopy Center, Environmental Health Center, Asan Medical Center, University of Ulsan College of Medicine, South Korea

**Background:** The association between serum vitamin D deficiency at birth and atopic dermatitis (AD) is uncertain. The aim of this study was to investigate the relationship between cord serum 25(OH)D on the development of AD with a prospective birth cohort study.

**Methods:** Children aged 0 through 3 yr from a birth cohort in the Cohort for Childhood Origin of Asthma and allergic diseases (COCOA) study were enrolled. The cord blood obtained from 655 at birth. The 25(OH)D and DNA from their cord blood are measured and specific IgE antibodies against egg and milk were performed at 1, 3yr of age. Also skin prick test were conducted at 3yr of age.

**Results:** The median cord serum 25(OH)D was 17.9 ng/ml. Low cord serum 25(OH)D (<20 ng/mL) is associated with milk sensitization at 1yr of age. Severe cord serum 25(OH)D deficiency (<10 ng/mL) increased the risk of AD at age 2 (aOR, 3.287; 95% CI, 1.587-6.808; p-value, 0.001) and age 3 (aOR, 2.686; 95% CI, 1.167-6.182; p-value, 0.020). We also found that cord serum 25(OH)D may affect outcome of AD. Low cord serum 25(OH)D reduced the remission of AD (aOR, 0.321; 95% CI, 0.96-1.072; p-value, 0.065) and severe deficiency ((<10 ng/mL) is associated with newly development of AD (aOR, 8.446; 95% CI, 0.905-78.792; p-value, 0.061).

**Conclusions:** Cord serum 25(OH)D were associated with milk sensitization and AD. And low cord serum 25(OH)D reduce the remission of AD and severe deficiency is associated with newly development of AD. These data suggest that cord serum 25(OH)D affect the development and prognosis of AD.

## A497 Contribution of Stem Cell Factor Autocrine/Paracrine Mechanism to Aberrant Proliferation of Mast Cells

### Yosuke Amagai, Akane Tanaka, Hiroshi Matsuda

#### Tokyo University of Agriculture and Technology, Japan

##### **Correspondence:** Yosuke Amagai – Tokyo University of Agriculture and Technology, Japan

**A) Background:** Mast cells originated from canine mast cell tumors are useful tools to investigate KIT-dependent or -independent proliferation of mast cells. It is well known that gain of function mutations in the c-*kit* gene are seriously associated with neoplastic proliferation of mast cells in humans, rodents, and dogs. However, KIT-independent malignant proliferation of mast cells has been rarely explored. Since most patients of mast cell leukemia/sarcoma and more than 70% of patients with cutaneous mastocytosis preserves wild type KIT but not mutant KIT, we attempted to find out KIT-independent mechanisms that induce tumorigenic proliferation of mast cells.

**B) Methods:** We investigated a mechanism of tumorigenesis using a wild-type KIT-expressing canine mast cell line. Western blot analysis was conducted to detect KIT phosphorylation. Both RT-PCR and flow cytometry analysis were carried out to examine the stem cell factor (SCF) expression. Inhibitory effects of a SCF neutralizing antibody and RNA interference for SCF on the cell proliferation were also evaluated. SCF production in xenografts consisted of wild-type KIT-expressing tumor was identified.

**C) Results:** High expression of SCF was detected in the canine mast cell line with wild-type KIT used in this study. In the cells, KIT was spontaneously phosphorylated. Neutralization of SCF as well as SCF gene silencing inhibited the growth of the cells, suggesting SCF autocrine/paracrine action. Production of SCF was also observed in several mast cell lines originated from humans and rodents, which was enhanced after PMA/ionomycin stimulation. Ki-67-positive cells in the xenografts were markedly positive for SCF. Moreover, SCF was strongly detected in 3 of 5 samples isolated from canine mast cell tumors that express wild-type KIT.

**D) Conclusion and discussion:** These results indicate the broad contribution of SCF autocrine/paracrine to the neoplastic proliferation of wild-type KIT-expressing mast cells not only in dogs but also both humans and rodents. It suggests that targeting SCF production may become a novel strategy for treatment of mast cell malignancies.

## A498 A Randomized Dbpc Dose-Finding Multicenter Trial of Sublingual Immunotherapy (SLIT) Allergoid Tablets in House Dust Mites (HDM) Allergic Patients

### Ralph Mösges^1^, Pauline Dieterich^1^, Anatoli Astvatsatourov^1^, Christoph Hüser^1^, Jaswinder Singh^1^, Kija Shah-Hosseini^1^, Silke Allekotte^1^, Enrico Compalati^2^

#### ^1^Institute of Medical Statistics, Informatics and Epidemiology, University Hospital of Cologne, Germany; ^2^Lofarma S.p.a., Italy

##### **Correspondence:** Ralph Mösges – Institute of Medical Statistics, Informatics and Epidemiology, University Hospital of Cologne, Germany

**Background:** Mites tablets of monomeric allergoids have been developed for sublingual immunotherapy in patients suffering from allergic rhinoconjunctivitis (ARC). The purpose of this trial was to determine the efficacy and safety of four different doses of mites tablets compared to placebo.

**Method:** Out of 160 patients recruited, 131 adult patients with ARC induced by HDMs were randomized for this dbpc phase II study (EudraCT No 2013-000617-20) conducted in Germany. Treatment consisted of either 300 UA/d; 1,000 UA/d; 2,000 UA/d; 3,000 UA/d or placebo over a course of 12 weeks. Efficacy was assessed by the improvement of reactions to a titrated conjunctival allergen challenge. Safety was assessed by frequency, type and severity of treatment-related adverse events (TRAE).

**Results:** After a 12-week course of immunotherapy, 88.5% and 76.0% of the patients treated with 2,000 UA/d and 1,000 UA/d, respectively, showed a tenfold improvement in the threshold of allergen concentration compared to 64.2% under placebo (p<0,05 and p=0.358). Neither treatment related SAEs nor cases of anaphylaxis were reported, so there was no need for the use of epinephrine. In total, of all patients under active treatment 4.95% experienced local TRAEs while 6.93% had systemic TRAEs.

**Conclusions:** The treatment with mite monomeric allergoids is a well-tolerated and safe treatment for patients suffering from HDM induced ARC. The highest proportion of patients with improvement in the CPT threshold of allergen concentration was found in patients treated with 2.000 UA/d corresponding to approximately 168.000 UA cumulative dose during the course of the trial.

## A499 Depression and Allergy in the Elderly: A Community Population Analysis

### Kyoung Hee Sohn^1^, Woo-Jung Song^2^, Byung-Keun Kim^3^, Ju-Young Kim^2^, Min Suk Yang^4^, So-Hee Lee^2^, Sae-Hoon Kim^5^, Hye-Ryun Kang^2^, Heung Woo Park^2^, Sun-Sin Kim^2^, Kyung-up Min^1^, Sang-Heon Cho^2^, Yoon-Seok Chang^3^

#### ^1^Seoul National University College of Medicine, South Korea; ^2^Seoul National University Hospital; ^3^Seoul National University Bundang Hospital; ^4^Smg-Snu Boramae Medical Center; ^5^Harvard T.H. Chan School of Public Health, USA

##### **Correspondence:** Kyoung Hee Sohn – Seoul National University College of Medicine, South Korea

**Background:** Depression and allergic diseases have been reported to be frequently comorbid. However, their associations have been under evaluated in a comprehensive way, particularly in the elderly.

**Objective:** We aimed to examine the associations between allergic parameters and depression in a community-based elderly population.

**Methods:** The present analyses were performed using the baseline data set of the Korean Longitudinal Study of Health and Aging, consisting of 1,000 elderly subjects (aged over 65) randomly recruited from an urban community. Depression was assessed by geriatric depression scale (GDS), the Center for Epidemiologic Studies Depression Scale (CES-D scale), the Hamilton Rating Scale for Depression (HRSD). Allergic parameters included questionnaires and allergen skin tests.Asthma symptoms and history were defined by structured questionnaires, and atopy was defined by inhaled allergen skin prick test. General quality of life scale (SF-36) and comorbidity were assessed.

**Results:** The prevalence of asthma and major depression disorder were 6.6 % and 5.3%, respectively. The prevalence of depressive symptoms was not statistically significant between non-asthmatic and asthma group (19.0% vs. 10.6%; p = 0.088). In additional analyses, however, individuals reporting symptom such as usual cough, chronic cough and nocturnal cough were at higher risk of major depression and lowered SF-36 (Adjusted using age, gender, education and income, p<0.05). Furthermore, rhinitis, atopy and eosinophilia were not related to depression. Risk factors for geriatric depression were identified as female sex, advanced age, low income (≤1 million won a month), dementia, solitary life and comorbid medical condition.

**Conclusion:** It was concluded that asthma is not associated with depression in the elderly. In comparison, asthma symptoms profoundly affect objective depression scales. The present study indicates that an appropriate treatment of asthma control has the potential decreased depressive disorder.

## A500 The Integrated Analysis of Correlation Between Total IgE and Other Immunological Factors in Allergic Diseases

### Woo-Sung Chang, Ji-Hye Do, Yeon-Seop Kim, Dankyu Yoon, Hye-Sun Lim, Jeom-Kyu Lee, Eun-Jin Kim

#### Korea National Institute of Health, Korea Center for Disease Control and Prevention, South Korea

##### **Correspondence:** Woo-Sung Chang – Korea National Institute of Health, Korea Center for Disease Control and Prevention, South Korea

**Background:** Allergic diseases are mainly mediated by immune responses with IgE antibodies specific for allergens and orchestrated by various immunological factors including immune cells and cytokines. It was known that the level of total IgE, helper T cells and some cytokines were associated with allergic inflammation and disease development. Evaluation of the correlation between them is needed to examine their possibilities as a reference factor for the diagnosis and monitoring of allergic diseases.

**Methods:** We previously investigated the serum level of total IgE, 22 allergy-related cytokines, and the ratio of Th1/Th2 cells respectively in both normal individuals and allergic patients. Each factor was compared in normal and allergic participants to examine their significant difference. The correlation of total IgE with other immunological factors was investigated by various statistical analysis using integrated results of each factors.

**Results:** The serum level of total IgE in allergic patients was significantly higher than normal subjects (265.6 vs. 47.16 kU/L, *p*<0.0001). Th1 cell percentage was also different (6.54 vs. 8.60, *p*=0.001), but Th2 cell percentage and Th1/Th2 ratio showed no significant difference between normal and allergic participants. Nine of 22 cytokines were analyzed in allergic patients and their levels increased in patients compared to normal individuals, particularly Platelet-Derived Growth Factor BB (PDGFBB) was much higher in allergic patients (1491.3 vs. 536.0 pg/ml, *p*<0.0001). By integrated analysis in all participants, total IgE had a significant correlation with Th1/Th2 ratio and Th2 cell percentage (*p*=0.02 and *p*=0.04, respectively).

**Conclusion:** In this study, we found that Th1 cell percentage decreased in allergic patients, supporting Th1 cells might be important roles in allergic responses. Our results also showed that PDGFBB could be responsible for allergic responses, suggesting its possibility as a reference factor for allergic diseases. We demonstrate that the correlation of total IgE with Th1/Th2 ratio and Th2 cell percentage might be relevant to corroborate the immunological function of Th2 cells for IgE-related responses.

## A501 Pattern of Allergic Diseases Among Military Servicemen Referred to a Clinical Immunology/Allergy Service in Singapore

### Bernard Thong, Yew Kuang Cheng, Jinfeng Hou, Khai Pang Leong, Justina Tan, Faith Chia, Grace Chan, Sze-Chin Tan, Teck Choon Tan, Chwee Ying Tang, Hiok Hee Chng

#### Tan Tock Seng Hospital, Singapore

##### **Correspondence:** Bernard Thong – Tan Tock Seng Hospital, Singapore

**Background:** To study the pattern of allergic diseases among military servicemen and women referred from the Singapore Armed Forces (SAF).

**Methods:** Referrals to the Tan Tock Seng Hospital Clinical Immunology/Allergy Clinic from 1 Jan 1998 to 15 May 2015 were retrospectively reviewed. The demographic profile of servicemen, types of allergic/immunologic diseases and definitive therapies prescribed were studied.

**Results:** There were 247 referrals comprising 90.7% males, predominantly active full-time national servicemen (NSF) and regulars. The mean age at diagnosis was 24±6 years. They comprised 88.3% Chinese, 5.3% Malays and 3.3% Indians. The most common referring diagnoses were for insect venom allergy (37.5%), urticarial/angioedema (18.3%), anaphylaxis (17.8%); drug allergy (15.4%), food allergy (9.1%), nonsteroidal anti-inflammatory drug [NSAID] hypersensitivity (6.3%) and allergic rhinitis (5.8%). Following evaluation by the allergist, insect venom allergy (36.5%), anaphylaxis (24.0%), allergic rhinitis (23.8%) and NSAID hypersensitivity (20.7%) were the most common conditions. Of the 32 servicemen diagnosed with insect venom anaphylaxis, 9 (28.1%) underwent allergen immunotherapy (AIT), of whom 6 were regulars and 3 NSF. All received yellow jacket and paper wasp venom AIT, and 1 in addition received honey bee venom AIT. No serviceman developed systemic reactions during AIT. Only 1 serviceman has completed 5 years of AIT, the mean duration of all servicemen on AIT to date being 2.2±1.3 years.

**Conclusions:** Insect venom allergy, anaphylaxis, allergic rhinitis and NSAID hypersensitivity were the most common referrals from the SAF. Medical officers in the military should be trained and equipped to manage military servicemen with these conditions at primary care level: in particular knowledge of the anaphylaxis action plan, and when and how to use epinephrine autoinjectors. Knowledge of NSAID hypersensitivity reactions is also important especially since non-selective NSAIDs are commonly used in the treatment of musculoskeletal injuries during training.

## A502 A Case of Rifampicin-Induced Hypersensitivity Diagnosed By the Lymphocyte Activation Test with Successful Desensitization

### Chan-Sun Park^1^, Mi Yeoung Kim^2^, Eun-Young Kim^1^, Jae-Gook Shin^1^, Jae-Hyeog Choi^1^, Saegwang Park^1^, Yeonye Kim^1^

#### ^1^Inje University College of Medicine, South Korea; ^2^Busan Paik Hospital, Inje University College of Medicine

##### **Correspondence:** Chan-Sun Park – Inje University College of Medicine, South Korea

Anti-tuberculosis (Tb) drugs can cause various adverse drug reactions (ADRs) including hypersensitivity syndrome. Because multiple drugs are concomitantly administered, the detection of culprit drug is essential for successful treatment. Lymphocyte activation test (LAT) is one of the promising methodologies to evaluate delayed drug hypersensitivity, but its role in anti-Tb hypersensitivity remains controversial. A 41-year-old man was referred to allergy clinic with high fever, headache, and skin rash on both arms and legs. He was diagnosed as pulmonary Tb and has started combination anti-Tb drug therapy consisting of isoniazid, rifampicin, ethambutol, and pyrazinamide for 10 days. Body temperature was 38.0 °C. Erythematous maculopapular rash was on both upper and lower extremities and several tender lymph nodes were palpable on cervical area. There were increased levels of aminotransferase (AST) 110 IU/L and alanine transaminase (ALT) 180 IU/L. All anti-Tb drugs were ceased. Patch test showed weak reaction to both rifampin and pyrazinamide. However, only rifampin was strong positive in LAT test. We successfully reintroduced rifampicin by oral desensitization without complication. Our experience suggests that LAT could be helpful to determine culprit drug in poly-pharmacy, especially in anti-Tb drugs.

## A503 Analysis of Individual Case Safety Reports of Drug-Induced Anaphylaxis Based on Korea Adverse Event Reporting System Database

### Kyung-Hwan Lim^1,2^, Jae Woo Jung^3^, Mingyu Kang^4^, Ju-Young Kim^1^, Ju-Young Kim^5^, Hyun Jeong Kim^6^, Yeon-Ju Woo^6^, Soo-Youn Jung^6^, Hye-Ryun Kang^1^, Hye-Ryun Kang^5^

#### ^1^Seoul National University Medical Research Center, South Korea; ^2^The Armed Forces Capital Hospital, South Korea; ^3^Chung-Ang University Hospital; ^4^Chungbuk National University Hospital; ^5^Seoul National University Hospital; ^6^Korea Institute of Drug Safety and Risk Management

##### **Correspondence:** Kyung-Hwan Lim – Seoul National University Medical Research Center, South Korea

**Background:** Anaphylaxis is a catastrophic systemic reaction and drugs are responsible for 20% to 40% of anaphylaxis. However, little is known about the characteristics of drug-induced anaphylaxis in Korea. The aim of this study is to investigate causal drugs and clinical features of the drug-induced anaphylaxis in Korean by using data from the adverse drug reaction (ADR) reporting system to the Korea Institute of Drug Safety & Risk Management (KIDS).

**Methods:** Among Individual Case Safety Reports (ICSRs) to KIDS from January, 1989 to June 2014, cases of drug-induced anaphylaxis were selected and age, gender, causative agents, and fatal cases resulting in death were analyzed.

**Results:** A total of 2,190 cases were identified. Male was 912 (41.6%) and mean age was 49.81 ± 18.40 years. Most common causal drug was antibiotics (576, 26.3%), followed by aspirin/non-steroidal anti-inflammatory drugs (NSAIDs) (390, 17.8%), contrast media (339, 15.5%), and anticancer drug (273, 10.7%). There were 25 fatal cases and antibiotics (8 cases) and contrast media (4 cases) were the two most common causative drug category. Of 186 drugs reported at least two times as suspected causative agents, 19 drugs (10.2%) did not reflect anaphylaxis in their drug labeling information.

**Conclusions:** Antibiotics, aspirin/NSAIDs, contrast media, and anticancer drugs were 71.3% of causative drugs among anaphylaxis ICSRs in Korea. Antibiotics and contrast media were also main causative agents responsible for fatal drug induced anaphylaxis.

## A504 Impact of Processes Certification on the Liability of Anti-Dust Mites Bed Covers

### Thierry Porée, Nabile Boukhettala, Emeline Furon

#### Laboratoire Protec’som, France

##### **Correspondence:** Thierry Porée – Laboratoire Protec’som, France

**Background**

Anti-mite barrier covers are manufactured from textiles with filtration properties usually validated by different certification labels. Although only textile certifications are mandatory, some covers are manufactured using processes ISO 13485 certified, subjecting covers to more stringent quality control measures.

The objective of the study was to evaluate the benefit of ISO 13485 certification on the liability of the final product.

**Methods**

In this study six different batches of micro-woven (MWC) and non-woven polyester polyamide (NWP) textiles went through the quality control processes required by the norm ISO 13485.

Upon reception, textiles pore sizes were measured using an optical microscope (Bresser, USA).

Textile permeability was then tested using a Rotomitest, apparatus composed of two compartments separated by the sample. Der p1 allergens were placed in compartment 1 and the apparatus was set to rotate for 18 hours.

Der p1 allergens that passed through the sample to compartment 2 were then measured with a Der p1 ELISA kit (Citeq Biologics, Netherlands).

Values, expressed as mean +/- SEM, were compared using two-way ANOVA.

**Results**

Pore sizes were found to be smaller for MWC than for NWP textiles (4 vs 11 μm).

NWP fabrics showed irregular pore sizes, with 2 batches having pore size greater than 5 μm.

Permeability results showed that NWP fabrics are significantly more permeable to Der p1 allergen compared to MWC tissue (23,6ng +/- 0.7 vs 3,4ng +/- 0.02).

Thus, ISO 13485 quality controls allowed for dismissal of 2 out of 6 batches of unsatisfactory NWP textiles.

**Conclusions**

In addition to the initial test on textiles, as for medical devices of higher class, monitoring manufacturing processes certification is necessary to ensure the quality of the finished product, especially when using nonwoven fabric. Therefore, ISO 13485 certification is a relevant criteria for anti-mite covers quality and thus effectiveness.

## A505 Localisation Kinetics of Aluminium after Subcutaneous Injection in a Rat Model

### Alan David Bullimore, Matthew Heath, Simon Hewings, Murray Skinner

#### Allergy Therapeutics, UK

##### **Correspondence:** Alan David Bullimore – Allergy Therapeutics, UK

Subcutaneous immunotherapy is an effective treatment for allergy. It works by helping to re-balance an individual’s immune response to allergens and the ability to drive an antibody titre response is greatly improved by the use of adjuvants, the most common being aluminium hydroxide. No data or pre-clinical model on the localisation kinetics of aluminium after subcutaneous injection, based on allergy formulations, currently exists.

**Methods**

Albino rats of the Crl:WI(Han) strain each received a single subcutaneous administration on 4 occasions with a 3 or 4 day intervals of a Birch concentrate formulated with either Alhydrogel or L-Tyrosine as the representative depot adjuvant. Dose sites were extracted and digested up to 6 months after final administration and aluminium (Al^3+^) analysed *via* ICP-MS.

**Results**

A significant proportion of aluminium (~50%) was retained at the injection site 3 months post final injection. The rate of clearance of aluminium from the dose site was calculated over a 6 month time period. As an estimate (from D14 and D180 data), the terminal half-life for clearance from SC dose site would be approx 240 days (i.e. time taken to remove half of the dose). Therefore, estimated time to clear 95% dose from SC site would take approx 1.2 years in the rat model.

**Conclusions**

The localisation kinetics of aluminium after subcutaneous injection, based on allergy formulation, has been investigated with the rate of clearance of aluminium from the injection site calculated from a rat model. Granuloma formations are one of the most common unwanted adverse reactions when a patient receives allergy subcutaneous immunotherapy. The results presented herein support current understanding that aluminium has the propensity to form focal accumulations in the body, beginning at the site of administration.

## A506 Periostin Levels in Exhaled Breath Condensate of Competitive Athletes, Asthmatics and Healthy Subjects - Associations with Outdoor Ambient Conditions

### Marcin Kurowski^1^, Hubert Krysztofiak^2^, Aleksandra Wardzynska^1^, Marzanna Jarzebska^1^, Janusz Jurczyk^2^, Marek L. Kowalski^1^

#### ^1^Medical University of Lodz, Poland; ^2^National Centre for Sports Medicine

##### **Correspondence:** Marcin Kurowski – Medical University of Lodz, Poland

**Background:** Periostin is considered a biomarker of Th2-driven allergic inflammation and predictor of airway eosinophilia. Periostin levels in exhaled breath condensate (EBC) in athletes have not been evaluated. Aims of the present study included: comparison of periostin levels in EBC from athletes, asthmatics and healthy controls (HC); assessment of the effect of exercise and the influence of ambient conditions on EBC periostin.

**Methods:** Study group consisted of 15 competitive athletes (5 speed skaters and 10 swimmers) aged 15-25. Control groups comprised 10 mild-to-moderate asthmatics aged 19-39 (asthma controls, AC) and 7 healthy, non-smoking subjects aged 21-27 (healthy controls, HC). Control subjects were not performing sports on a reglar basis. Athletes were assessed in two time-points: in-training (period 1) and out-of-training (period 2) depending on individual training schedule. Treadmill exercise challenge was conducted according to the ATS guidelines. EBC was collected immediately before and 30 minutes after exercise. Periostin levels in EBC were assessed by ELISA.

**Results:** Periostin EBC levels before and after exercise challenge were significantly decreased in athletes during in-training period as compared with out-of-training period (1.87±0.69 vs 2.36±0.77 ng/ml, mean±SD, p<0.02) and with AC subjects (1.87±0.69 vs 2.72±0.62 ng/ml, p<0.01). Exercise challenge did not induce significant changes in EBC periostin in any of the groups. In athletes during training period, significant positive correlation was observed between baseline EBC periostin levels and mean daily air temperature on the assessment day (day 0) (R=0.57; p=0.02), mean temperature during 7 days preceding the assessment (R=0.56, p=0.02), dew point temperature on day 0 (R=0.60, p<0.02) and mean dew point temperature during 7 days preceding the assessment (R=0.57, p=0.02). In asthmatics a significant negative correlation was observed between baseline EBC periostin and mean daily air temperature on day 0 (R=-0.58; p=0.04). No correlations were observed in athletes during off-training period and in HC subjects.

**Conclusions:** Regular exercise may contribute to decreased expression airway periostin level. Ambient conditions seem to influence periostin release into the airways.

## A507 The Role of PKR Pathway in Acute Exacerbation of Severe Bronchial Asthma

### So Ri Kim, Yong Chul Lee, Dong Im Kim, Yang Keun Rhee, Heung Bum Lee, Seoung Ju Park, Yeong Hun Choe Choe, Seung Yong Park

#### Chonbuk National University Medical School/Hospital, South Korea

##### **Correspondence:** So Ri Kim – Chonbuk National University Medical School/Hospital, South Korea

Asthma exacerbations are an exaggerated lower airway response to an environmental exposure. Triggers of asthma exacerbation include virus infection, allergen, environmental pollutants, occupational sensitisers and irritants, and some medicine such as aspirin. It is well known that respiratory viral infection is the most common cuase for severe asthma exacerbation. The double-stranded RNA (dsRNA)-activated serine/threonine kinase R (PKR) is well characterized as an essential component of the innate antiviral response. Moreover, PKR activation is associated with IgE class switching and subsequent induction of IgE-mediated disorders such as allergy and asthma. Meanwhile, PKR phosphorylates e-IF2α, one of branches for unfoled protein response (UPR). Conversely, endoplasmic reticulum (ER) stress activates PKR which stimulates various inflammatory signaling pathways. However, to date, there is little information on its role the asthma exacerbation, especially acute exacerbation of steroid-resistant severe asthma. In this study, we investigated whether PKR activation is involved in the induction of asthma exacerbation using a mouse model of acute asthma exacerbation induced by the administration of poly (I:C). We found that the administration of poly (I:C) aggravated the all severe asthmatic features compared to those in mice sensitized with ovalbumin (OVA) and lipopolysaccharide (LPS) and challenged with OVA (OVA_LPS_-OVA mice); the number of airway inflammatory cells in bronchioalveolar lavage (BAL) fluids, airway hyperresponsiveness, and the expression of Th2 IL-17 and KC in lung tissues. Interestingly, the PKR expression was also more increased in lung tissues from OVA_LPS_-OVA mice treated with poly (I:C) than OVA_LPS_-OVA mice. Moreover, the phosphorylation of PKR in primary cultured tracheal epithelial cells was further enhanced in OVA_LPS_-OVA mice treated with poly (I:C). This study indicates that PKR activation plays an important role the induction of acute exacerbation of severe neutrophilic asthma, highlighting the therapeutic potential of PKR inhibitor as a potent controller of acute asthma exacerbation in severe asthmatic patients.

## A508 Diversity of Clinical Manifestations and Treatment Responses for Idiopathic Hypereosinophilic Syndrome

### Joo-Hee Kim, Sunghoon Park, Young Il Hwang, Seung Hun Jang, Ki-Suck Jung

#### Hallym University Sacred Heart Hospital, South Korea

##### **Correspondence:** Joo-Hee Kim – Hallym University Sacred Heart Hospital, South Korea

**Background:** Idiopathic hypereosinophilic syndrome (IHES) is a rare disorder defined by persistent blood eosinophilia, absence of secondary causes, and evidence of eosinophil-associated organ dysfunction. In some patients with hypereosinophilia (HE) may cause life-threatening complications, whereas other patients, referred to “hypereosinophila of unknown significance (HE, US)” do not exhibit any measurable organ damage. Although clinical diversities of HES have been recognized, only isolated case reports are available in Asia. This retrospective analysis sought to summarize the baseline demographic, clinical, and laboratory characteristics in a cohort of patients with HES and HE, US and to review responses to treatment.

**Method:** Clinical and laboratory data from 25 patients with IHES or HE, US, seen between January 2004 and December 2014 at Hallym Sacred Heart Hospital, were collected retrospectively after chart review.

**Result:** A total of 25 patients were enrolled; 7 patients (28.0%) were diagnosed with HE, US and 18 patients (72.0%) with IHES. The mean number of white blood cell and peak total eosinophil count (TEC) were significantly increased in patients with IHES, compared to those with HE, US (p=0.008, respectively). Fip1-like1–platelet-derived growth factor receptor a (FIP1L1-PDGFRA) mutation analysis was done in 9 of 25 patients using in situ hybridization, and all showed negative results. In patients with IHES, the most common clinical presenting symptom was gastrointestinal (44.4%), followed by constitutional symptoms (33.3%) such as fever, myalgia, weakness, and weight loss, and pulmonary (11.1%). All patients received corticosteroids (0.25~1.0 mg/kg) as initial therapy. Seven patients (38.8%) showed complete response and 11 patients (61.1%) with partial response within a month. However, 9 patients (50.0%) recurred during tapering or discontinuation of corticosteroid. There was no significant association between treatment response and laboratory parameters including peak TEC, total IgE, eosinophil cationic protein or marrow eosinophilia.

**Conclusion:** The majority of patients with IHES respond to corticosteroid therapy, however, discontinuation of corticosteroid is associated with recurrence. There are no clinical or laboratory markers to predict the prognosis of IHES.

## A509 Effect of Dexamethasone in Th17 Cell Mediating Neutrophilic Asthma

### Jiang Min^1^, Nong Guang-Min^2^

#### ^1^The First Affiliated Hospital of Guangxi Medical University, China; ^2^Department of Pediatrics, the First Affiliated Hospital of Guangxi Medical University, Nanning, China

##### **Correspondence:** Nong Guang-Min – Department of Pediatrics, the First Affiliated Hospital of Guangxi Medical University, Nanning, China

**Objective:** Evidence suggests that dexamethasone involved in the apoptosis of differentiation Th17 cells, and the apoptosis of neutrophil was delayed in Neutrophilic Asthma mice model. Here we investigate the effect of dexamethasone on Th17 cells involved in the mechanisms of neutrophil asthma(NA).

**Methods:** NA and neutrophilic asthma dexamethasone intervention(NAD) mice were sensitized by LPS and OVA airway delivery. Eosinophilic asthma(EA) and eosinophilic asthma dexamethasone intervention(EAD) mice were sensitized through the peritoneum with OVA and aluminum hydroxide. During the challenge stage, the NA and EA mice were exposed to a 1% OVA aerosol. Before the aerosol, NAD and EAD mice were treated with 1mg/kg dexamethasone through the peritoneum. And control mice with no treatment. Data were collected after the last exposure. ELISA, flowcytometry and quantitative PCR were used to measure cytokines, TH17 cell subsets, intracellular cytokines and signaling molecule. Invasive measurements of airway resistance were used to measure airway hyperreactivity(AHR).

**Results:** (1) BALF IL-6 levels in NAD groups were no significant difference compared with the NA and EAD group, but all were higher than in control. (2) BALF TGF-β levels in NAD groups were no significant difference compared in NA and EAD group, but all were markedly higher in control. (3) BALF IL-7 levels in NAD groups were no significant difference with the NA group, but both were markedly higher in control. (4) The RORγt-mRNA expression levels in NAD group were markedly down-regulated compared with NA group, and were no significant difference with the EAD group and control. (5) The SOCS3-mRNA levels in the NAD group were markedly up-regulated compared with the NA and control, but were markedly down-regulated compared with EAD group. (6) The IL-7-mRNA expression levels in NAD group were markedly up-regulated compared with NA group, and was markedly higher than in EAD and control. (7) The SOCS1-mRNA levels in the NAD group were no significant difference with the NA group. And the NAD group were significant down-regulated compared with the EAD group. (8) The Th17^+^STAT5^+^ levels in the NAD group were markedly decrease compared with the NA group, but were markedly higher than in EAD and control. (9)The Th17^+^Bcl-2^+^ levels in NAD group were markedly decrease compared with the NA group, but were markedly higher thanin EAD and control. (10) The Th17^+^Caspase3^+^ levels in the NAD group were markedly decrease compared with the NA group, but were markedly lower than in EAD group and NAD group.

**Conclusions:** Dexamethasone can decrease the differentiation of Th17 cells to dominant down-regulated the RORγt and up-regulated the SOCS3 in NA mice. After treat with dexamethasone, high level of IL-7 can inhibit apoptosis and promote their survival of Th17 cells, connected to dominant of JAK-STAT5 signal pathway and Bcl-2, Caspase-3.

## A510 Allergen Profile for Asthma/Rhinitis and Eczema Among Patients in North India: An Immunocap Allergen Specific IgE Antibodies Assay Based Study

### Nalin Nag

#### Indraprastha Apollo Hospitals, India

**Background:** As the prevalence of allergic diseases like asthma, rhinitis and eczema are gradually increasing in India, knowledge of the prevalence of common allergens causing allergic asthma/rhinitis and eczema will be useful for the optimum use of therapeutic modalities like allergen avoidance and immunotherapy to reduce the burden of these diseases. Most of the studies in India are based on skin prick or intra dermal testing which are conducted by non standardized techniques. There is lack of studies based on the measurement of specific IgE antibodies by ImmunoCAP technique which has been recently introduced in India. Therefore ImmunoCAP specific serum IgE antibody assay was done to determine the prevalence of 94 allergens in patients with eczema and allergic asthma/rhinitis in India.

**Method:** This study was conducted in New Delhi, India for six months. 170 patients with allergic rhinitis/asthma and eczema were recruited in the study to investigate the pattern of 94 allergen prevalence using ImmunoCAP allergen specific IgE antibody assay. Data was analyzed using SPSS 17.

**Results:** All 170 patients enrolled in the study showed a positive response to one or more allergens. In both the genders, Dust mites, Cockroach, Bermuda grass and Ragweed were found to be most prominent allergens. Dust mites were more prominent in the age group below 5 years. Other important allergens were Ragweed, Bermuda grass, Johnson grass and Cockroach. Also, in the age group 5years and above, Dust mites were the most important allergen followed by Cockroach, Bermuda grass, Ragweed and Cocklebur.

In patients with Asthma/rhinitis, the dominant allergens found were Dust mites ( 41.24%), Cockroach (40.20%), Bermuda grass (28.87%) and Ragweed (24.74%) respectively (P<0.05). While in patients with Eczema, Cockroach (37.5%) was more prominent followed by Dust mites, Wheat and Shrimp each having a prevalence of 31.5% (P<0.05).

**Conclusion:** In India, this is one of the first study of its kind which uses FDA approved and WHO standardized gold standard method of ImmunoCAP specific IgE assay to estimate the prevalence of different allergens in patients with asthma/allergic rhinitis and eczema. One or more allergens are responsible for causing Asthma/Rhinitis and Eczema and the prevalence is increasing in India. Avoidance of known allergens and specific immunotherapy can significantly reduce the need for ongoing expensive pharmacological long term treatment and enhance remission.

## A511 Clinical Profile of Allergic Rhinitis in Children in Jakarta

### Wahyuni Indawati

#### Cipto Mangunkusumo Hospital, Indonesia

**Background:** There has been limited publication regarding characteristics of allergic rhinitis in children in Indonesia.

**Objective:** We aim to investigate the clinical characteristics of allergic rhinitis (AR) in children.

**Methods:** A retrospective cross-sectional study was conducted at pediatrics clinic in a tertiary referral hospital, Cipto Mangunkusmo Hospital in Jakarta, Indonesia, during 2010-2011.

**Results:** Out of 112 AR diagnosed patients, 60.7% were boys. Most common age was under 5 years old (49.9%) with median of 4.65 (range 2 months–17 years old). The most common symptoms were rhinorrhea (93.7%) and cough (83.9%). In general, patients had atopy (72,3%) and family history of atopy (78.6%). Duration of illness mostly was more than 2 weeks (75.9%). Based on interview, cold air had been the most common triggering factor (54.5%), followed by food (12.5%) and dust (9.8%). The most common signs were allergic shiner (9.8%). The second generation of antihistamine (58.9%) and intranasal steroid (28.6%) were the most prescribed drug. Asthma (9.8%) as the comorbidity only found in 9.8%, while rhinosinusitis as the complication occurred in 12.5%.

**Conclusion:** Younger age was found in most of the cases. The most frequent type of AR is persistent AR. Despite of high number of atopic history only a small proportion of children have asthma.

## A512 Preclinical Study on the Use of Micro Crystalline Tyrosine (MCT) Adjuvants in Allergy Immunotherapy

### Alan David Bullimore, Matthew Heath, Murray Skinner

#### Allergy Therapeutics, UK

##### **Correspondence:** Alan David Bullimore – Allergy Therapeutics, UK

**Background**

Vaccines and allergen-specific immunotherapy typically contain adjuvants that facilitate immune responses in humans and animals. For almost a century, salts of aluminium (hydroxide and phosphate) were the only approved adjuvants in humans. One major problem of aluminium adjuvants is that they are not biodegradable and that they typically stimulate so-called T-helper type 2 (Th2) as opposed to Th1 immune responses, which again affects the type of antibody responses produced. The goals of new adjuvants are therefore (i) to facilitate recognition of antigen/allergen, (ii) to be biodegradable and biocompatible, (iii) to be without toxic or inflammatory side effects, and (iv) to trigger protective Th1-like immune responses as well as allergen-neutralising antibodies. Co-precipitates of micro crystalline tyrosine (MCT) and proteins have been suggested as candidate adjuvants for allergen-specific immunotherapy.

**Methods**

Immunogenicity testing of MCT-ovalbumin vaccines in naïve BALB/c and C57BL/6 mice was undertaken. Three injections were performed at 2 week intervals, and the mice were tail bled prior to each injection as well as at different time points after the last injection. The obtained sera were analysed for OVA-specific antibodies, while spleen cells were tested for T-cell responses including cytokine secretion after re-stimulation of the cells in vitro with ovalbumin.

**Results**

*MCT* has good adjuvant properties, comprising a high adsorptive power for proteins, and enhancement of Th1-like and associated immune responses, highlighting its potential of action as a biodegradable depot adjuvant in allergen-specific immunotherapy. MCT is naturally metabolised and the pharmacokinetics of MCT present a half-life at the injection site of 48 hours; this is a particular benefit for allergy SCIT, a traditionally long course treatment, minimising the need for accumulation of non-biodegradable adjuvant.

**Conclusions**

Results from pre-clinical immunogenicity and MCT mode-of-action studies in mice have demonstrated the potential of MCT as a non-toxic and biodegradable alternative to conventional adjuvants for use in allergen immunotherapy.

## A513 Genetic Diversity of Filaggrin Mutation and Its Clinical Implication in East Asian Atopic Dermatitis Patients

### Seong Jun Seo, Won Jong Oh

#### Chung-Ang University Hospital, South Korea

##### **Correspondence:** Seong Jun Seo – Chung-Ang University Hospital, South Korea

**Background:** Since the report on the association of Filaggrin(*FLG)* mutations with atopic dermatitis(AD), genetic diversity of *FLG* mutation was intensively investigated for European and other ethnic populations. However, except for the well-established racial difference between European and Asian, detailed analysis on the genetic diversity and its clinical implication of *FLG*mutation for East Asian ancestry was not performed, yet.

**Objectives:** We study geographic distribution of *FLG* mutations in East Asia and evaluate clinical significance of *FLG mutations* in East Asian AD patients.

**Methods:** We summarized data related to *FLG* mutations across East Asian countries, and then analyzed geographic distribution of *FLG* mutations in the light of human migration history in East Asia, and finally, we investigated the clinical significance of *FLG*mutations in East Asian AD patients.

**Results:** Through the genealogical analysis on the genetic diversities found in *FLG* mutations across East Asia, we confirmed that the distribution of *FLG* mutations among East Asian populations was consistent with hypothetical waves of human migrations along the southeastern coast of Asia. Similar to Europeans, *FLG* mutations were significant risk factor of AD for East Asian and palmar hyperlinearity was a useful clinical marker in screening carriers of *FLG* mutation, however, in contrast to European, *FLG* mutations do not increase the risk of concomitant asthma for East Asian.

**Conclusion:** Genetic diversity of *FLG* mutation across East Asia is consistent with hypothetical waves of human migration along the costal border of East Asia. And European and Asian of *FLG* mutation have similarity and differences.

## A514 Protein and MPL Adsorption Capacities for MCT in Candidate Therapeutic Formulations for Use in Immunotherapy, Compared Against Existing Adjuvants

### Alan David Bullimore, Murray Skinner, Matthew Heath, Andrew Bell

#### Allergy Therapeutics, UK

##### **Correspondence:** Alan David Bullimore – Allergy Therapeutics, UK

**Background**

The World Health Organisation recommends adsorption of 80% or more of tetanus and diphtheria toxoid antigens by aluminium containing adjuvants. The protein adsorption capacities from aluminium and calcium adjuvants are well documented. Modified Allergen Tyrosine Adsorbed-Monophosphoryl Lipid A (MATA-MPL) formulations have been shown to be effective therapeutics in allergy immunotherapy. The micro crystalline tyrosine (MCT) in these formulations has been shown to consistently adsorb both allergoid and MPL on manufacture. However MCT adsorption capacity factors for proteins and MPL have not been measured for direct comparison to aluminium and calcium adjuvants.

**Methods**

Adsorption capacities of MCT for MPL and protein were calculated from quantitative determinations of both in the formulations. A gas chromatography method was used to determine MPL contents and a Bradford method was used to determine protein contents. Adsorption capacities of aluminium and calcium adjuvants were calculated in a similar way for direct comparison to MCT.

**Results**

MCT demonstrated greater adsorption capacities for MPL than both aluminium and calcium adjuvants.

**Conclusions**

The ability of MCT to readily adsorb MPL compared to aluminium and calcium adjuvants supports a characteristic association based on both tyrosine’s structure and MCT’s physical properties. MCT is an effective depot candidate for allergy immunotherapy formulations and vaccines.

## A515 Interleukin-22 Gene Variation in Inflammatory Bowel Disease

### Hournaz Hasanzadeh^1^, Salman Sadeghzade^2^, Nima Rezaei^3^, Alireza Zarebidoki^4^

#### ^1^Center for Research and Training in Skin Diseases and Leprosy, Tehran University of Medical Sciences; ^2^Department of Cell and Molecular Biology, Azad University of Ashkezar Branch; ^3^Department of Immunology, Tehran University of Medical Sciences; ^4^Molecular Immunology Research Center (MIRC), Tehran University of Medical Sciences, Iran

##### **Correspondence:** Alireza Zarebidoki – Molecular Immunology Research Center (MIRC), Tehran University of Medical Sciences, Iran

**Background:** Inflammatory bowel disease (IBD) is an inflammatory disorder of unknown pathogenesis. Recent studies showed that interleukine-22 plays an important role in inflammatory processes during the disease. The purpose of this case-control study was to explore the association between IL-22 gene polymorphism (rs2227501) and susceptibility to IBD.

**Methods:** 89 patients and 201 healthy individuals referred to the Namazi Hospital of Shiraz, Iran were entered in this study. Blood samples were collected and genomic DNA was extracted. Restriction fragment length polymorphisms polymerase chain reaction (PCR-RFLP) was performed, and data were analyzed using Chi-square and Bonferroni tests.

**Results:** The frequency of the A allele was significantly greater, and the T allele was significantly lower in patients compared to controls (P value = 0.02). In addition, there was a statistically significant relationship between AA genotype and IBD (P value = 0.01).

**Conclusion:** This is the first study of evaluating rs2227501 in Iranian patients with IBD. More studies are required in order to clarify the exact role of IL-22 polymorphisms in pathogenesis and susceptibility of inflammatory bowel disease.

## A516 Immunomodulatory Effects of Adipose-Derived Stem Cell Secretome in a Mouse Model of Asthma

### Kyu-Sup Cho

#### Pusan National University, South Korea

**Background and Objectives:** Several studies have demonstrated that adipose-derived stem cells (ASCs) can ameliorate allergic airway inflammation by shifting to a Th1 from a Th2-biased immune response. The ASCs secrete a variety of autocrine/paracrine factors, called secretome, that protect cells from apoptotic cell death and modulate immune system. In this study, we evaluated the effects of ASCs-derived secretome on allergic airway inflammation in ovalbumin (OVA) induced asthmatic mouse model.

**Materials and Methods:** C57BL/6 mice were sensitized to OVA by intraperitoneal injection and challenged intranasally with OVA. To evaluate the effect of ASCs-derived secretome on allergic airway disease, 1 μl/ml of ASCs supernatant were administrated intranasally before OVA challenge. We evaluated airway hyperresponsiveness (AHR), the proportion of eosinophils in bronchoalveolar lavage fluid (BALF), lung histology, serum total and OVA-specific antibody, cytokine profile of BALF and lung draining lymph nodes (LLN), and T cell population of LLN.

**Results:** ASCs-derived secretome significantly inhibited eosinophilic inflammation in the lung. AHR, total immune cell and eosinophils in the BALF, and mucus production were significantly reduced after ASCs-derived secretome administration. ASCs-derived secretome significantly decreased the serum total and allergen-specific IgE and IgG1 level. ASCs-derived secretome significantly inhibited Th2 cytokines (IL-4, IL-5, and IL-13) and enhanced Th1 cytokine (IFN-γ) and regulatory cytokines (IL-10 and TGF-β) in the BALF and LLN. In addition, CD25^+^Foxp3^+^ and IL-10^+^ T cells in LLN were significantly increased after ASCs-derived secretome administration.

**Conclusions:** ASCs-derived secretome ameliorated allergic airway inflammation and improved lung function through the induction of Tregs expansion. Secretome may be a promising candidate for a novel cell-free therapy for allergic airway diseases that has many advantages in overcoming the limitations and risks associated with the cell-based therapeutics.

## A517 Diffuse Alveolar Hemorrhage with Positive Anti-Neutrophil Cytoplasmic Antibody in a Child: A Case Report

### Moo-Young Oh^1^, Sung-Woo Kim^2^

#### ^1^Busan Paik Hospital, Inje Universit College of Medicine, Busan, Korea; ^2^Department of Pediatrics, Busan Paik Hospital, Inje Universit College of Medicine, Busan, Korea

##### **Correspondence:** Sung-Woo Kim – Department of Pediatrics, Busan Paik Hospital, Inje Universit College of Medicine, Busan, Korea

Alveolar hemorrhage with anti-neutrophil cytoplasmic antibody (ANCA) is a rare disease in children.

ANCA is associated with certain diseases such as granulomatosis with polyangiitis (GPA), microscopic polyangiitis (MPA) and eosinophilic granulomatosis with polyangiitis (EPGA). This group of disease, characterized by necrotizing small-vessel vasculitis show autoantibodies in patients serum, directed against neutrophil cytoplasmic constituents, especially proteinase 3 (PR3) and myeloperoxidase (MPO). In these diseases, affected vessels have changed with focal necrosis in pathologic findings and patients suffer from pulmonary, cerebral, gastrointestinal, other hemorrhagic complication and also renal dysfunction represented by crescentic glomerulonephritis.

A 8-year-old previous healthy girl presented with acute onset dyspnea, cough, hemoptysis, chest discomfort, fever and hypoxemia. Her chest CT scan showed diffuse consolidation and ground glass opacity in both lungs, hemoglobin was 3.9g/dL, hematocrit 12%, so she was diagnosed as diffuse alveolar hemorrhage.

Bleeding diatheses were excluded by laboratory testing, and Anti nuclear antibody(ANA) and P-ANCA were positive. Myeloperoxidase-ANCA (MPO-ANCA) was positive but proteinase 3-ANCA was negative. Antiglomerular basement membrane antibodies(Anti-GBM) was negative. urinalysis showed RBC 21-30/HPF, creatinine 0.48mg/mL but there were no RBC casts or protein.

This patient had been diagnosed for microscopic hematuria, but her kidney function was normal and other symptoms of ANCA associated vasculitis had never been appeared. The patient’s alveolar hemorrhage recovered on immunosuppresive therapy with cyclophopamide and corticosteroids. We report 8-year old girl case, treated for severe alveolar hemorrhage and microscopic hematuria with positive ANCA.

## A518 Clinical Analys the Serum TARC Levels As the Condition Index of Atopic Dermatitis in the Early Infancy

### Munemitsu Koizumi, Kazuyo Kuzume

#### Graduated School of Medicine, Japan

##### **Correspondence:** Munemitsu Koizumi – Ehime Graduated School of Medicine, Japan

**Background:** The correct severity assessment is important for treatment of Atopic dermatitis (AD) Although the levels of serum Thymus and activation-regulated chemokine (TARC)/CCL17 is well known as a proper means for assessing severity of AD, the levels of TARC are different according to the month of age. We examined a correlation of serum TARC level and Severity Scoring of Atopic Dermatitis (SCORAD) index in patients with AD in early infancy.

**Methods:** Thirty-three (33) patients with atopic dermatitis (19 boys and 14 girls, age 3 to 5 months) were recruited We examined the correlation between SCORAD index and TARC levels as a primary outcome, as well as the correlations between SCORAD index and serum total IgE levels, peripheral eosinophil counts, and serum Lactate dehydrogenase (LDH) levels as secondary outcomes. Spearman’s rank correlation coefficients were used for each statistic evaluation.

**Results:** The median of the SCORAD index in patients was 14 (range, 1-69). Also, the medians of the TARC levels, the peripheral eosinophil counts, and the serum LDH levels were 2213pg/dl (range, 514-21776 pg/dl), 6%(range 0%-18%), and 290IU/ml(range 211-432 IU/ml), respectively. There was significant positive correlation between the SCORAD index and the TARC levels (r=0.623, p < 0.001), as well as between the SCORAD index and the peripheral eosinophil counts, (r=0.638, p < 0.001). On the other hand, there was no significant correlation between the SCORAD index and the serum total IgE levels. Also. the SCORAD index and the serum LDH levels were not correlated significantly.

**Conclusions:** Serum TARC levels and peripheral eosinophil counts may be useful to assess severity of AD in early infancy.

## A519 An Analysis of the Filaggrin Gene Polymorphism in Korean Atopic Dermatitis Patients

### Kui Young Park, Won Jong Oh

#### Chung-Ang University Hospital, South Korea

##### **Correspondence:** Kui Young Park – Chung-Ang University Hospital South Korea

**Background:** Research of the filaggrin(*FLG)* mutation in various ethnic groups revealed non-overlapping mutation patterns. In addition, Japanese and Chinese atopic patients showed somewhat different mutations. These ethnic differences make the research on Korean patients mandatory; however, no systematic research on Korean atopic dermatitis patients has been performed.

**Objective:** This study aims to investigate the genetic polymorphism of *FLG* in Korean atopic dermatitis patients.

**Methods:** The study was made up of three groups including 9 IV patients, 111 AD patients and 55 normal controls: the ichthyosis group was incorporated due to the reported association between the *FLG* mutation and ichthyosis vulgaris. In comparison to other sequencing methods, the overlapping long-range PCR was used.

**Results:** We revealed the genetic polymorphism of *FLG* in Koreans, and at the same time, we discovered new nonsense mutations, p.Y1767X and c.526delA, while confirmed that the previously reported c.3321delA, c.3222del4, p.K4022X in Korean patients.

**Conclusion:** By using *FLG* sequencing techniques confirmed in this study, new mutations or genetic polymorphisms with ethnic characteristics would be detected and further larger studies of repeat number polymorphisms could be performed.

## A520 A New Protocol for Wheat Oral Immunotherapy in Patients with Anaphylaxis

### Delara Babaie^1^, Mohammad Nabavi^2^, Fariborz Zandieh^3^, Mehrdad Amir Moini^1^, Zahra Chavoshzadeh^1^, Hamideh Seifi^1^, Mitra Sahragard^1^, Mehrnaz Mesdaghi^1^, Mohammad Hassan Bemanian^4^

#### ^1^Mofid Children’s Hospital, Shahid Beheshti University of Medical Sciences Iran; ^2^Hazrate Rasool Hospital, Iran University of Medical Scienses; ^3^Bahrami Childrens’ Hospital, Tehran University of Medical Sciences; ^4^Hazrate Rasool Hospital, Iran University of Medical Sciences

##### **Correspondence:** Delara Babaie – Mofid Children’s Hospital, Shahid Beheshti University of Medical Sciences, Iran

**Background:** IgE-mediated wheat allergy affects around 0.5% of the population, the only accepted treatment was complete food avoidance. However, accidental exposure may occur frequently since it is difficult to avoid major foods. Recently, several studies have changed the dilemma of avoidance. We have proposed a new wheat oral immunotherapy (OIT) protocol in anaphylactic patients.

**Method:** Children with history of anaphylaxis, who met the inclusion criteria, were recruited to the study. Patients having uncontrolled asthma, cardiovascular disease, significant systemic disease, and poor compliant patients were excluded. Anaphylaxis was confirmed by DBPCFC in all patients. Then the OFCs (oral food challenges) was performed for all patients with cake to determine the initial dose of OIT. We designed the 3 step build up phase, which contains cake, Bread A and Bread B. According to the OFC results, patients started the protocol. If the reaction threshold was under 1gr of wheat protein, they would start with cake and continue till 25 gr. Beyond the 1 gr, they were asked to take bread A, which was contained 5.28gr protein in 100 gr of flour. The amount of bread was increased twice a week under the medical supervision, when it reached to 30 gr, it was changed to full wheat bread (8.8 gr of proteins in 100 gr flour), and was risen to 60 gr. After finishing build up phase, they were continued to take 60g of bread in a day (maintenance phase) for next 3 months. In addition to clinical measures, some immunological mechanisms of desensitization were measured at the first day, and at the end of buildup and Maintenance phase.

**Results:** 9 patients (2-14years) which were recruited to the study, 8 (88.8%) were finished the protocol. One couldn’t accomplished the protocol because of recurrent sever anaphylactic reaction and remained on 10 gr of bread B. SPT wheal was significantly reduced at the end of maintenance phase.

**Conclusion:** We have demonstrated that wheat tolerance could be achieved in a stepwise OIT regimen in anaphylactic patients. Apparently, further investigation will need to confirm safety and effective wheat OIT protocol.

## A521 Clinical Characteristics of Filaggrin-Related Atopic Dermatitis Patients in Korea

### Sun Young Choi, Yeon a No

#### Chung-Ang University Hospital, South Korea

##### **Correspondence:** Sun Young Choi – Chung-Ang University Hospital, South Korea

**Background:** Filaggrin is a key protein involved in skin barrier function. Mutations in the gene encoding filaggrin (*FLG*) have been identified as the cause of ichthyosis vulgaris and have been shown to be major predisposing factors for atopic dermatitis (AD).

**Objective:** The purpose of this study was to investigative the clinical characteristics of AD patients with *FLG* mutations and determine the differences between AD patients with and without *FLG*mutation.

**Methods:** We identified the *FLG*mutations of AD patients by complete sequencing and snapshot methods and then analyzed the data on clinical characteristics from questionnaire responses.

**Results:** We found that earlier age of AD onset (p=0.047), tendency to respiratory atopy (p=0.029), more severe AD clinical characteristics (higher EASI score, p=0.018), and decrease in skin hydration (p=0.04) were associated with *FLG*-related AD.

**Conclusion:** Our data demonstrate that *FLG* mutations are indicators of a poor prognosis in AD and are predisposing factors that exist in early infancy and persist into adulthood.

## A522 Early Allergy Diagnosis in Children - Self- Administered Questionnaire Vs Medical Verification

### Dorota Kiedik^1^, Agnieszka Muszynska^1^, Iwona Pirogowicz^1^, Andrzej M. Fal^2^

#### ^1^Wroclaw Medical University; ^2^Wroclaw Medical University, National Institute of Public Health, Poland

##### **Correspondence:** Andrzej M. Fal – Wroclaw Medical University, National Institute of Public Health, Poland

**Background**

The aim of the project ‘Early diagnosis of asthma and allergies among children attending primary schools in Wroclaw’ was to preselect the group of high risk of asthma/allergy development using a questionnaire and then to confirm the diagnosis at the physician’s office.

**Methods**

The study was carried out in the years 2011-2013 All children attending 3 grade at grammar schools (8-9 ys of age) (Wrocław, Poland) entered the study. Those with prior diagnosis of asthma ana allergy or being under allergists care were excluded. During the first part all parents were educated on the allergy symptoms and prevention. They were given a specially prepared screening questionnaire. The questionnaire included questions concerning family history, early exposure of their children to different allergens etc., children’s medical history: infections, symptoms (cough, wheezing, shortness of breath, runny nose, rash), hospitalizations etc. During the second part all children who scored above a fixed level in the questionnaire were subjected to allergist’s check-up (including prick-tests and spirometry) to confirm or deny the possible diagnosis of asthma or allergy.

**Results**

The study in its first part involved 2,690 children. After the analysis of the questionnaires 375 children visited the study allergist. In 29% of them the diagnosis of asthma or allergies was confirmed. Especially early symptoms and selected allergen exposures correlated well with the final diagnosis (p < 0.05 was noted between exposure to moisture, mold and diagnosed in a child: of asthma: Phi = 0.093; p = 0,00; of allergic rhinitis: Phi = 0.114 ; p = 0,00; of atopic dermatitis: Phi = 0.052; p = 0.007; food allergy Phi = 0.048 ; p = 0.012).

**Conclusions**

The questionnaire proved to be a fast, cheap and reliable tool in allergy detection. It has been introduced in Wrocław,s schools for standard use.

## A523 Asthma Impact on Children with Food Induced Anaphylaxis

### Chikako Motomura, Masatoshi Wakatsuki, Yuko Akamine, Mihoko Iwata, Hiroshi Matsuzaki, Naohiko Taba, Yoko Murakami, Hiroshi Odajima

#### Fukuoka National Hospital, Japan

##### **Correspondence:** Chikako Motomura – Fukuoka National Hospital, Japan

**Rationale:** Patients who have both food allergies and asthma are at increased risk for anaphylactic reactions and life-threatening asthmatic reaction. The aim of this study is to determine the asthma impact of on characteristics of children with food induced anaphylaxis adrenaline autoinjectors were prescribed.

**Methods:** 90 patients (28female, 62male, range 2-11 year-old) with food allergy adrenaline autoinjector were prescribed between April and October 2013 were recruited. We excluded cases treated by oral immunotherapy. We evaluated causal food, symptom of recent anaphylactic reaction, serum mite-specific IgE, adrenaline treatment for anaphylaxis. Their factors of asthmatic group (n=53, asthma onset 2.7 years, 23 treated with inhaled corticosteroid) were compared with those of Non-asthmatic group (n=37).

**Results:** Number of adrenaline treatment for anaphylactic reaction in asthmatic group was higher than those of Non-asthmatic group significantly (P<0.05). There was no significant difference in mite-specific IgE, symptom of anaphylaxis between asthmatic group and Non-asthmatic group. Forty three (81%) of anaphylactic reactions in asthmatic group induced by milk, wheat and egg were higher than 19(51%) in Non-asthmatic patients (p<0.01). In contrast, peanuts and nuts induced anaphylactic reactions in Non-asthmatic group more frequently than those of asthmatic group (30% vs.9%).

**Conclusion:** In asthmatic children, anaphylactic reactions occurred frequently by accidental ingestion of daily foods, for example, milk, wheat and egg. Bronchial hyperresponsiveness may induce anaphylactic reaction easily by small amount of causal food.

## A524 Case Reports of Stevens-Johnson Syndrome and Stevens-Johnson Syndrome-Toxic Epidermal Necrolysis in Systemic Lupus Erythematosus

### Reni Ghrahani

#### Faculty of Medicine Universitas Padjadjaran/Dr. Hasan Sadikin General Hospital, Indonesia

**Background:** Stevens-Johnson Syndrome (SJS) and Toxic Epidermal Necrolysis (TEN) are both immune-mediated disorders that can be life-threatening and frequently related to prior drugs consumption. Autoimmune disorders have been suggested to have a predisposing effect on SJS, SJS-TEN, or TEN; however, it is difficult to decide whether the eruption is induced by drugs or a manifestation of lupus itself.

**Objective:** This case report presents cases of SJS and SJS-TEN in Systemic Lupus Erythematosus (SLE).

**Case 1:** A 12 years old female came to hospital with a chief complaint of blisters and scalded skin all over her body, hands, feet, and face which had started 2 days prior to admission after consuming herbal medication for 2 days. Additional symptoms were fever, multiple painless oral ulcers, and painful swelling of joints that made patient unable to walk since 3 months prior to admission. On physical examination, general multiple discrete, round, size Æ 0.5 cm–1.2 cm, mostly finely demarcated, some raised, mostly dry, in form of erythema, bullae, erosion, discoid pustules, bullous pustules, and discoid were found. There were ulcerations on oral and genital mucous. Blood examination were performed and hemolytic anemia, positive anti nuclear antibody (ANA) and Anti dsDNA, as well as proteinuria were observed. The patient was diagnosed as suffering from SJS in SLE.

**Case 2:** A 12 years old female came to hospital with a chief complaint of peeling skin on her body, hands, and face for 1 day prior to admission. This patient was referred by another hospital with cefixime and gentamicin treatment history. On physical examination, there were fever, scaly lesions on body, hands, and face with scalded lesion on the back. There was erosion on lips and oral mucous. Laboratory findings were pancytopenia, positive of direct coomb’s test, reactive ANA and high titer Anti dsDNA as well as proteinuria. The patient was then diagnosed as suffering from SJS-TEN in SLE.

**Conclusion:** Patients when presented with a SJS or SJS-TEN-like picture with SLE background, it becomes difficult to decide whether the eruption is drug induced or a manifestation of lupus itself.

## A525 The Effectiveness of Oral Tolerance Induction for Wheat Allergy Using Two Different Intake Levels

### Yuri Takaoka

#### Osaka Prefectural Hospital Organization Osaka Prefectural Medical Center for Respiratory and Allergic Diseases, Japan

**Objective:** Many reports exist on the effectiveness of oral tolerance induction for food allergies. However, few comprehensive reports exist on its effectiveness for wheat allergy.

**Subjects:** Among subjects who had a positive result in an oral food challenge test for *udon* (wheat noodles), informed consent was obtained from 49 subjects who were judged to be capable of starting intake at 0.5–5 g dried noodle weight based on the final dose and induced symptoms.

**Method:** Oral tolerance induction was performed after randomly dividing the subjects, with consideration of age, into the following two groups according to intake frequency: the frequent group, intake at six times/week or more; and the intermittent group, intake at two times/week. After six months of tolerance induction, the ability of these patients to ingest the noodles at the target dose was evaluated.

**Results:** Of the 49 subjects, 32 were finally considered in this study, and each group had 16 subjects in whom intake could be totalled based on intake diaries, and who were able to maintain intake at five times/week or more (frequent group) or two times/week (intermittent group). After six months, the proportion of subjects who had a negative result on testing with the target dose (20 g dried noodle weight for subjects ≤3 years of age, and 50 g dried noodle weight for those ≥4 years of age) or who were capable of the target intake within six months was 73% on the whole, and no differerence in both groups.

**Conclusion:** The findings suggest that even when intake frequency in oral tolerance induction for wheat is reduced to twice/week, no clear difference is seen with the target dose after six months of tolerance induction.

## A526 Association of Plasma Interleukin-25 Levels with Development of Aspirin Induced Airway Spasm in Asthma

### Jong-Uk Lee^1^, Jeong-Seok Heo^1^, Da-Jeong Bae^2^, Hyun Ji Song^2^, Choon-Sik Park^2^, Jong-Sook Park^2^

#### ^1^Soonchunhyang University, South Korea; ^2^Soonchunhyang University Bucheon Hospital

##### **Correspondence:** Jong-Uk Lee – Soonchunhyang University, South Korea

**Background:** Aspirin exacerbated respiratory diseases (AERD) are characterized as an acute bronchospasm by ingestion of aspirin and heavy infiltration of eosinophils, which is contributed by altered synthesis of cycteinyl leukotrienes. Although the acquired Th2 response is apparently revealed in IgE - dependent asthma, but not obvious in AERD. Recently, IL-25, TSLP and IL-33 appears as key molecules of the innate Th2 response in allergic inflammation. Thus, we tried to reveal the relation of the innate Th2 molecules with development of AERD.

**Methods and Materials:** The level of IL-25, IL-33 and TSLP were measured in plasma before and after aspirin challenge to the subjects with AERD (n=18) and ATA (n=16) using an enzyme-linked immunosorbent assay (ELISA), and then normalized with plasma protein.

**Results:** The plasma level of IL-25, IL-33 and TSLP were comparable before aspirin challenge between AERD and ATA. After aspirin challenge, however, concentrations of IL-25 were robustly increased in AERD compared to those before aspirin challenge while did not change in ATA. The post challenge level of IL-25 was significantly higher in AERD more than that of ATA (0.34 pg/mg vs. 2.31 pg/mg; *p=*0.03), while those of IL-33 and TSLP were not changed. There was a significant correlation between concentrations of IL-25 and aspirin induced - fall rate of FEV1 in total subjects (r^2^=0.172, *p=*0.037).

**Conclusions:** Among the innate Th2 cytokines, IL-25, but not IL-33 and TSLP, is related with development of airway spasm after aspirin challenge, which suggest that IL-25 may be play roles in pathogenesis of AERD via modulation of Th2-type immune response.

## A527 Transition of Allergic and Nonallergic Rhinitis after 2 Years in Korean Children: Preliminary Study

### Jae Hoon Cho, Ji Ho Choi

#### Konkuk University Hospital, South Korea

##### **Correspondence:** Jae Hoon Cho – Konkuk University Hospital, South Korea

**Objectives:** Allergic and nonallergic rhinitis are very common disease for children, however, little is known about their natural courses. The purpose is to evaluate the natural course of allergic and nonallergic rhinitis in children.

**Study Design:** Individual cohort study

**Methods:** We analyzed data from Snoring Child Cohort of 208 children. The data encompassed questionnaire about chronic rhinitis, and the result of skin prick test (SPT) for 5 inhalent and specific IgE for 2 allergens. Children were classified into four groups, namely allergic rhinitis (rhinitis plus positive SPT), nonallergic rhinitis (rhinitis plus negative SPT), sensitization only (no rhinitis plus positive SPT), and control (no rhinitis plus negative SPT).

**Results:** Finally, the data of 124 children were analyzed. Among 18 children with allergic rhinitis at 7 year, 13 (72.2%) became sensitization only after 2 years and only 5 (27.8%) were remained as allergic rhinitis. Five (26.3%) out of 19 with children nonallergic rhinitis at 7 year turned into allergic rhinitis at 9 year and 7 (36.8%) into control. Twenty six (86.7%) out of 30 children with sensitization only at 7 year were remained as same at 9 year. Among 59 control children at 7 year, 2 (3.3%) became those with allergic rhinitis, 7 (11.9%) with nonallergic rhinitis, and 16 (27.1%) with sensitization only at 9 year.

**Conclusion:** The status of chronic rhinitis and allergen sensitization is very changeable in children.

## A528 Early Onset of Psoriasis Juvenile Idiopathic Arthritis

### Fiska Febriana^1^, Reni Ghrahani^1^, Gartika Sapartini^2^, Budi Setiabudiawan^3^

#### ^1^Faculty of Medicine Universitas Padjadjaran/Dr. Hasan Sadikin General Hospital, Indonesia; ^2^Dr. Hasan Sadikin General Hospital; ^3^Department of Child Health, Faculty of Medicine, Universitas Padjadjaran, Dr Hasan Sadikin General Hospital, Bandung, West Java, 40162, Indonesia

##### **Correspondence:** Budi Setiabudiawan – Department of Child Health, Faculty of Medicine, Universitas Padjadjaran, Dr Hasan Sadikin General Hospital, Bandung, West Java, 40162, Indonesia

**Background:** Psoriasis juvenile idiopathic arthritis (PsJIA) is an autoimmune disease, which constitutes a small part of Juvenile Idiopathic Arthritis (JIA) and classified within the spectrum of JIA and psoriatic lesion. The onset often occurs between the ages 7-13 years, but some of them occurs under 5 years. Its morbidity, such as: leg discrepancy, irreversible joint damage, contracture, visual loss caused by chronic uveitis, and persistent pain can impact the life quality of the patient. It is important to make an early and precise diagnosis, and give the treatment as soon as possible in order to make the quality of life of the patient optimum.

**Methods:** A six months longitudinal case report about a 3-year-old-boy with early onset psoriasis JIA and the result of the treatment.

**Results:** A 3-year-old-boy presented with a year history of right knee swelling accompanied by inflamation sign, joint stiffness, dactylitis, skin redness, and also white scales that generalized over his body. At first he was diagnosed with Ichtyosis vulgaris, because of the persistent arthritis (>6 weeks), he was referred from Dermatology and Venereology Department to Pediatric Immunology-Allergy Department with the highly suspected of JIA. He was born at term, from P_1_A_0_mother by spontaneous vaginal delivery, with a birth weight 3400 grams. There is no history of JIA or psoriasis in his family. From the laboratory examination, there were anaemia, with a small increased of the erythrocyte sedimentation rate (ESR), but C-reactive protein (CRP), liver and kidney function were within normal limits. Antinuclear antibodies (ANA), rheumatoid factor (RF) and anti-ds-DNA were negative. Before the treatment, the patient looked stiff, could not stand up well and could not move his hands, fingers and feet. The fingers bent like claws. After 5 months therapy of methotrexate, NSAIDs, physiotherapy and also topical momethasone furoate 0,1%, he experienced laboratory and clinically remission of arthritis and psoriasis.

**Conclusions:** The goals of therapy are to control pain and inflammation, prevent joint damage, preserve range of motion and muscle strength, strive for normal function, growth, nutrition, physical and psychosocial development and to control systemic manifestations. A multi-disciplinary team approach is essential in optimizing results. Physical and occupational therapy are important for some patients. Methotrexate (MTX) has been recognized as the most effective DMARDs and applicable in almost every countries. Genetic counseling and education about the disease is needed for every parents with affected child.

## A529 Clinical Features of Immediate Hypersensitivity to Histamine H2 Antagonists and Their Cross Reactivity

### Young-Hee Nam^1^, Mi Yeoung Kim^2^, Gil-Soon Choi^3^, Chan-Sun Park^4^

#### ^1^Dong-a University School of Medicine; ^2^Busan Paik Hospital, Inje University College of Medicine; ^3^Kosin University Gospel Hospital; ^4^Inje University College of Medicine, South Korea

##### **Correspondence:** Chan-Sun Park – Inje University College of Medicine, South Korea

**Background:** Histamine H2 antagonists are generally well-tolerated. Severe hypersensitivity reactions to these are rare. However, an IgE-dependent mechanism has been suggested for hypersensitivity induced by H2-receptor antagonists. In addition, the possible cross-reactivity among H2-receptor antagonists has been existed.

**Objective:** To investigate the clinical features of immediate hypersensitivity induced H2 antagonists and their cross reactivity.

**Methods:** We retrospectively evaluated clinical characteristics of patients diagnosed as immediate hypersensitivity to H2 antagonists at 4 University hospitals in Busan, Korea and analyzed the data from skin test to various H2 antagonists.

**Results:** A total of 12 patients were enrolled in this study. The mean age was 48 ± 4 years (rage 26-68) and 6 patients were female. Most common culprit drug was ranitidine (N=10, 83%) followed by cimetidine (N=1) and nizatidine (N=1). Half of patients had previously allergic reaction to H2 antagonists but were not diagnosed before. The mean latent time was 42.09 + 18.4 minutes (range, immediate~210). Anaphylaxis was the most common symptom (N=7, 58%), followed by local urticaria (N=3, 25%), and systemic urticaria (N=2, 17%). All patients except one had positive reactions in skin test (9 in prick test, 2 in intra-dermal test). All of the cross-reactions were between ranitidine, famotidine, and cimetidine. One patient had positive reaction to all histamine H2 antagonists.

**Conclusion:** Histamine H2 antagonists are widely used and generally tolerated. However, anaphylaxis was the most common symptom in histamine H2 antagonists induced immediate hypersensitivity and ranitidine was the most common causative drug. In addition, our data showed significant cross reactivity between drugs.

## A530 Detection of Galacto-Oligosaccharide Specific IgE *in Vitro*

### Chiung-Hui Huang^1,2^, Jian Yi Soh^3^, Lynette Shek^2^, Lynette Shek^1^, Dianne J. Delsing^4^, Bee Wah Lee^1^, Bee Wah Lee^2^, Si Hui Goh^5^, Wen Chin Chiang^6^, Wenyin Loh^6^

#### ^1^National University of Singapore; ^2^National University Health System, Singapore; ^3^National University Hospital; ^4^Frieslandcampina, Netherlands; ^5^Kkwoman’s and Children’s Hospital; ^6^Kk Women’s and Children’s Hospital

##### **Correspondence:** Chiung-Hui Huang – National University of Singapore

**Background:** Anaphylaxis to galacto-oligosaccharide (GOS), a prebiotic, is an entity unique to the Asian region. Despite being a pure carbohydrate, we have shown via skin prick (SPT) and basophil activation tests (BAT) that this reaction is IgE-mediated. Our preliminary studies using biotinylation of GOS and streptavidin binding to solid phase to develop an *in-vitro*GOS specific IgE assay were unsuccessful.

**Aim:** We sought to develop an *in vitro* IgE assay to GOS for screening populations for GOS sensitization, and compare its accuracy to the BAT.

**Methods:** GOS (degree of polymerization≥4) was conjugated to human serum albumin (HSA) by means of Maillard reaction. Sera from 12 GOS allergic patients and 9 GOS SPT negative controls were studied. In addition, sera from 14 GOS SPT positive volunteers who underwent oral GOS challenge (resulting in 7 positive challenges) were included. Sera were pre-absorbed with HSA prior to the detection of GOS-specific IgE using GOS conjugated HSA (GOS-HSA) by ELISA. The mean plus two standard deviation of the IgE level from 9 GOS SPT negative subjects was set as the cut of value to determine the positive or negative IgE response to GOS among the 28 tested sera.

**Results:** In the GOS challenge cohort, the sensitivity of the GOS-HSA IgE assay was 14.3% (95% CI 0.8 – 58.0%) and specificity was 71.4% (95% CI 30.3 – 94.9%). Among 12 patients who had presented with GOS allergy, 8 had a positive GOS-HSA IgE assay result giving a sensitivity of 66.7% (95% CI 35.4 – 88.7%). In contrast, BAT had a sensitivity of 83.3% (95% CI 50.8 – 97.1%) and specificity of 100% in the GOS challenge cohort. All patients had a strongly positive BAT at GOS concentrations of 10 μg/ml or higher.

**Conclusions:** The GOS-HSA IgE assay is able to detect GOS specific IgE. However, the BAT assay is superior to this assay in terms of sensitivity and specificity.

## A531 The Association Between Serum Vitamin D Levels and Allergic Diseases in Elementary Schoolchildren

### Hea-Kyoung Yang^1^, Ji Young Lee^1^, Minji Kim^1^, Kangmo Ahn^2^, Jihyun Kim^1^, Young-Min Kim^1^, Hye-Young Kim^3^, Yong Mean Park^4^, Woo Kyung KIM^5^, So-Yeon Lee^6^

#### ^1^Samsung Medical Center, South Korea; ^2^Samsung; ^3^Medical Research Institute of Pusan National University Hospital; ^4^Konkuk University School of Medicine; ^5^Seoul Paik Hospital/Inje University College of Medicine; ^6^Department of Pediatrics, Hallym Sacred Heart Hospital, Hallym University College of Medicine

##### **Correspondence:** Hea-Kyoung Yang – Samsung Medical Center, South Korea

**Purpose:** There are controversies about the relationship between vitamin D and allergic diseases, although there has been growing interest in vitamin D insufficiency. The aim of this study was to investigate the association between concentration of serum vitamin D levels and recent symptoms of allergic diseases in elementary schoolchildren.

**Methods:** A nationwide cross-sectional survey was conducted in the first grade students from randomly selected 45 elementary schools. The participants of this survey were selected using a stratified two-stage cluster sampling design. The prevalence of atopic dermatitis (AD), allergic rhinitis (AR), and asthma was obtained through the Korean version of International Study of Asthma and Allergies in Childhood questionnaire. All the children were examined by a pediatrician to determine the presence of eczema in their neck and flexural areas of both arms. Serum 25-hydroxyvitamin D [25(OH)D] was measured by chemiluminescent microparticle immunoassay. For categorical analysis by 25(OH)D concentrations, we used cutoffs of <20 ng/mL (50 nmol/L), 20-29 ng/mL (50-70 nmol/L), and ≥ 30 ng/mL (70 nmol/L). Multivariable logistic regression analysis was applied, adjusting for gender, maternal education levels, allergic diseased of parents, family income and urbanization.

**Result:** The overall prevalence rates of vitamin D insufficiency (20 to 29 ng/mL) and deficiency (<20 ng/mL) were 64.1% and 18.4%. Vitamin D level was not associated with the symptom of AD, AR, or asthma in the last 12 months (*P* value = 0.071, 0.976, and 0.757, respectively). Vitamin D level was not related to the presence of treatment for AD, AR, or asthma in the last 12 months (*P* value = 0.256, 0.794, and 0.703, respectively). In addition, there was no significant association between vitamin D level and the presence of pediatrician-confirmed AD on the day of survey (*P* value = 0.195).

**Conclusion:** A high prevalence of vitamin D deficiency and insufficiency was found in Korean elementary schoolchildren, but vitamin D level was not associated with the recent symptoms of allergic diseases.

## A532 Serum Levels Specific IgE to Toxic Shock Syndrome Toxin Type 1 in Eosinophilic Chronic Rhinosinusitis with Nasal Polyp in Korean

### Jongin Jeong^1^, Sang Duk Hong^1^, Seung Kyu Chung^1^, Hun-Jong Dhong^2^, Hyo Yeol Kim Hyo Yeol Kim^1^, Sujin Kim^1^

#### ^1^Samsung Medical Center, South Korea; ^2^Samsung Seoul Hospital

##### **Correspondence:** Jongin Jeong – Samsung Medical Center, South Korea

**Background:** Staphylococcus aureus enterotoxins (SE-IgE) has been associated with pathophysiology of chronic rhinosinusitis (CRS). The aim of this study was to determine the importance of serum specific IgE to toxic shock syndrome toxin type 1 (TSST-1) that is one of the SE-IgE in eosinophilic CRS with nasal polyp (NP) in Korean population.

**Methods:** Retrospective review with prospectively collected data. 221 CRS patients who underwent endoscopic sinus surgery (ESS) were enrolled. All tissues were microscopically examined for the presence of eosinophilia (10≥high power field). Blood eosinophil count and serum levels of total IgE, specific IgE to TSST-1 and eosinophil cationic protein (ECP) using ImmunoCAP were analyzed.

**Results:** Fifty six CRS without NP (47 non-eosinophilic and 9 eosinophilic) and 165 CRS with NP (114 non-eosinophilic and 51 eosinophilic) patients were included. The level of blood eosinophil count, total IgE and ECP were not significantly different, but serum specific IgE to TSST-1 was different between CRS with NP and without NP (p=0.26). Positive rate of serum specific IgE to TSST-1 was higher in Eosinophilic CRS with NP (54.9%) than CRS without NP (23.2%) and noneosinophilic CRS with NP (25.4%) (p=0.001).

**Conclusions:** This study demonstrates higher positive rate of serum specific IgE to TSST-1 in patients with eosinophilic CRS with NP but not in noneosinophlic CRS with NP. Preoperative examination of serum specific IgE to TSST-1 can help the prediction of eosinophilic NP that has been shown to higher recurrence rate after ESS.

## A533 Impacts of Rhizosphere Cleaning Effects of Potted Indoor Plants on the Symptoms and Stress of Students with Allergic Rhinitis in Newly Built Schools

### Hana Bak^1^, Hye-Rim Son^2^, Si-Eun Lee^2^, Kwang-Jin Kim^3^, Young-Wook Lim^2^, Ho-Hyun Kim^2^, Yong-Won Lee^4^

#### ^1^Cheongdam Hana Dermatology Clinic; ^2^Institute for Environmental Research, College of Medicine, Yonsei University; ^3^National Institute of Horticultural & Herbal Science, Rural Development Administration; ^4^Catholic Kwandong University International St. Mary’s Hospital, College of Medicine, South Korea

##### **Correspondence:** Yong-Won Lee – Catholic Kwandong University International St. Mary’s Hospital, College of Medicine, South Korea

**Background:** Although there have been several studies reporting that rhizosphere cleaning effects of potted indoor plants (RCEIP) could decrease indoor pollutants (VOCs, particulate matter, etc.) and habitants’ stress, impacts of RCEIP on allergic rhinitis (AR) have not been fully evaluated.

**Methods:** Total 115 students (male=54, 11.9±0.3 years) from the two newly built elementary schools were included. Their demographic data and skin prick results (for 30 common inhalant allergens) were collected initially. For RCEIP, potted indoor plants of eight plant-species were introduced to four classrooms (77 students) for three months, and the others were included to the controls. This process was done randomly and the single blinded (investigator) study scheme was kept until the completion. AR symptom-questionnaire (ARIA 2008 based), Korean Daily Hassles Scale for Children, Stress-Arousal Checklist, and Indoor Attractiveness Scale were surveyed before and after introducing indoor plants.

**Results:** Comparisons of the inhalant allergen sensitization rates based on the allergy skin prick test with 30 common inhalant allergens between the two groups (plant introduction vs. control) were all insignificant. In stratified and propensity score matching analyses with AR students (74 suspected, and 45 confirmed), AR symptoms were not changed by RCEIP. Increase in the teachers/school-life related stress was suppressed by RCEIP in subjects, but not in controls (p<0.05). Indoor attractiveness was maintained by RCEIP, but decreased in controls (p<0.05).

**Conclusions:** Three-month-Introduction of indoor plants for RCEIP suppressed school stress and increased indoor attractiveness, but did not aggravate AR symptoms of students in newly built schools.

*This work was carried out with the support (PJ010205042015) of Rural Development Administration, Republic of Korea (South Korea).

## A534 A Case of Multiple Food Allergies with Recurrent Anaphylaxis Successively Controlled By Omalizumab

### Man Yong Han^1^, Young-Ho Jung^1^, Hye Mi Jee^1^, Seung Jin Lee^1^, Kyung Suk Lee^1^, Mi-Ae Kim^2^

#### ^1^Bundang CHA Medical Center, CHA University School of Medicine; ^2^Bundang Medical Center, CHA University, South Korea

##### **Correspondence:** Mi-Ae Kim – Bundang Medical Center, CHA University, South Korea

Food allergy is defined as an adverse health effect that occurs reproducibly on exposure to a given food. Food allergy is classified into Immunoglubulin E (IgE)-mediated and non-IgE-mediated reactions. Clinical manifestations include gastrointestinal, cutaneous, and respiratory symptoms. IgE mediated food allergies usually manifest as acute gastrointestinal hypersensitivity, pollen-food allergy syndrome, acute urticaria, angioedema, allergic rhinoconjunctivitis, and acute bronchospasm. A diagnosis can be made by history, skin prick test, or serum food-specific IgE level although oral food challenges confirm the diagnosis. Managements of food allergy are food avoidance and treatment of acute reactions. We report here a case of multiple food allergies with recurrent anaphylaxis successively controlled by omalizumb.

A 43-year-old male patient visited allergy department for evaluation of chest tightness, throat swelling and dyspnea 20 minutes after an ingestion of two strawberries. The patient had repeatedly suffered from multiple food allergies to peach, plum, apricot, raspberry, and apple since his childhood, which was presented as acute urticaria. Laboratory test results showed an elevated blood eosinophil count (1800 /mm^3^) and an elevated serum specific IgE level to strawberry (measured by ImmunoCAP® [ThermoFisher Scientific, Uppsala, Sweden] system, 3.98 KU/L). Spirometry showed normal range. We assessed him as food allergy to strawberry and eosinophilia was thought to be resulted from the food allergy. After using systemic corticosteroid and antihistamines, blood eosinophil count decreased to normal range and the patient’s symptoms were resolved. Five months later, he complained of acute urticarial, dizziness, dyspnea after ingestions of pineapple, celery, and sesame. Blood eosinophil count was 900 /mm^3^ and serum specific IgE levels to pineapple, celery, and sesame were 0.68 KU/L, 0.93 KU/L, and 0.77 Ku/L, respectively. One month later, the patients visited emergency department for acute urticaria, dyspnea, general weakness, and numbness of legs after an ingestion of almond. Blood eosinophil count was 1,600 /mm^3^ and serum specific IgE level to almond was 4.63 KU/L. Recurrent episode of anaphylaxis due to multiple food was occurred with eosinophilia and food avoidance strategy was failed because of allergic reactions to unknown food. Furthermore, sesame was widely used to various Korean food as a spice. Omalizumab was treated monthly to him to control the IgE-mediated food allergy. After the treatment, there was no episode of anaphylaxis to him and the patient performed his daily tasks without disturbances. When complete avoidance of food allergy is impossible, omalizumab can be an alternative treatment to control symptoms.

## A535 Bepotastine-Induced Urticaria, Cross-Reactive with Other Antihistamines

### Jaechun Lee^1^, Eunkyoung Lee^2^, Jasmina Golez^3^

#### ^1^Jeju National University School of Medicine, South Korea; ^2^Jeju National University Hospital, South Korea; ^3^Ambulanta Meznar, Slovenia

##### **Correspondence:** Jasmina Golez – Ambulanta Meznar, Slovenia

Second-generation antihistamines are widely prescribed for the control of symptoms of allergic inflammation such as itchy hives, coryza, and itchy eyes. In rare circumstances, these drugs can provoke allergic inflammation. Hypersensitivity to bepotastine besilate, a second-generation antihistamine has never been reported.

A 17-year-old schoolgirl, whose paroxysmal itchy hives had been controlled with bepotastine, experienced aggravation of the hives. An oral provocation test confirmed her hypersensitivity to bepotastine and cross-reactivity to levocetirizine. She showed no reaction to chlorpheniramine, ketotifen, or olopatadine among the 13 antihistamines tested.

While searching for an antihistamine to control her itchy hives, we found that she also exhibited cross-reactivity to various antihistamines with different chemical structures from that of bepotastine, which is not predicted according to the chemical classification of antihistamines.

We report on a case of hypersensitivity to bepotastine besilate in a patient with chronic spontaneous urticaria.

## A536 Role of SLC26a4 in Ozone - Induced Airway Reactivity and Inflammation

### Da-Jeong Bae^1^, Chang-Gi Min^2^, Jong-Uk Lee^2^, Jong-Sook Park^1^, Hun Soo Chang^1^, Choon-Sik Park^1^, An-Soo Jang^1^

#### ^1^Soonchunhyang University Bucheon Hospital, South Korea; ^2^Soonchunhyang University

##### **Correspondence:** Da-Jeong Bae – Soonchunhyang University Bucheon Hospital, South Korea

**Background:** Acute or chronic exposure to ozone is characterized by an increase of airway hyperreactivity (AHR) with a neutrophilic inflammation of the airway in humans. Recently, meaningful increase of SLC26a4 was observed in the transcriptom data of ozone exposed mice. Since SLC26a4 gene induces mucin genes and intraluminal acidification in the airways, SLC26a4 is supposed to be a key molecule in the ozone - induced AHR and neutrophilic inflammation. Thus, we evaluated the role of SLC26a4 gene in a chronic ozone exposure murine model.

**Materials and Methods:** 6weeks old BALB/C female mice were exposed to filtered air or O3 (2ppm for daily exposure 3hr time, 3hrs to 21days). Airway resistance was measured using Flexi vent and bronchoalveolar lavage fluid (BALF) cells were differentially counted. Thiocyanate, as a metabolite produced by SLC26a4, was measured in BAL fluids. SLC26a4 and Muc5ac proteins and genes were assessed by western blotting and immune stain, and real-time PCR. Tetra-ammonium (0.01mg/kg) was administered into the ozone-exposed animals via intra-trachea route to block the effect of SLC26a4.

**Result:** Airway resistance and number of neutrophils in BAL fluids were increased significantly in the ozone - exposed mice in a time dependent manner. Thiocyanate amount are gradually decreased in lung lysates and BALF after ozone exposure. There was a correlation between the number of neutrophils and the amount of Thiocyanate. SLC26a4 and Muc5ac RNA and protein levels were increased and picked at the 21day in the ozone exposed mice. Tetra-ammonium significantly suppressed the ozone induced increase of AHR and inflammatory cells, particularly neutrophils.

**Conclusion:** SLC26a4 may exert a key role gene in the ozone exposure - related traits including AHR and neutrophilic inflammation in murine model.

## A537 PAR2-Antagonist Suppresses Protease-Induced Allergic Inflammation Mediated By Degradation of Lung Epithelial Tight Junction and Generation of ROS

### Ha-Jung Kim^1^, Young-Joon Kim^1^, Bok Kyoung Jung^1^, Seung-Hwa Lee^1^, Mi-Jin Kang^1^, Sekyoo Jeong^2^, Eun Lee^3^, Hyun-Ju Cho^3^, Young-Ho Kim^3^, Song-I Yang^4^, Seo Hee Kim^3^, Soo-Jong Hong^3^

#### ^1^Asan Institute for Life Sciences, South Korea; ^2^Neopharm Co., Ltd; ^3^Asan Medical Center; ^4^Hallym University Sacred Heart Hospital

##### **Correspondence:** Ha-Jung Kim – Asan Institute for Life Sciences, South Korea

**Background:** Protease in the common allergens (e.g., cockroach, mold) can induce a pathogenesis on allergic diseases. Protease activated receptor 2 (PAR2) is reported to trigger immune response on allergic asthma.

**Aim:** This study aimed to investigate the effects of a PAR2-antagonist on protease-induced allergic inflammation and identify the mechanism.

**Methods:** PAR2-antagonist was administered intranasally in the mouse model of asthma induced by German cockroach extract (GCE). In addition, human lung epithelial cells (A549 cells) were treated with GCE, PAR2-antagonist, and NAC to confirm the mechanism related in the *vivo* results.

**Results:** Airway hyperresponsiveness, total IgE production, and pulmonary inflammation were significantly suppressed by NAC and the PAR2-antagonist in the mouse model. Th2-cytokines and TSLP in the lung were suppressed by the NAC and PAR-2 antagonist. Tight junction protein, claudin-1 was disturbed by GCE and restored by PAR2-antagonist and NAC in the lung. TSLP and claudin-1 showed the same correlation in the human lung epithelial cells by the two *in vitro*.

**Conclusions:** GCE elicits allergic inflammation mediated by disruption of tight junction triggered by PAR2 and generation of ROS.

## A538 Novel Anti-IL-4Ra Nanocarrier Approach for the Efficient Control of Lung Tissue Inflammation during Asthma

### Rabih Halwani^1^, Saleh Al Muhsen^1^, Asma Sultana^2,3^, Achraf Al-Faraj^4^; Rosan Kanana^2,3^, Sibtain Afzal^2^, Roaa Al Kufaidi^2^

#### ^1^Prince Naif Center for Immunology Research and Asthma Research Chair, Department of Pediatrics, College of Medicine, King Saud University, Saudi Arabia; ^2^Prince Naif Center for Immunology Research, College of Medicine, King Saud University; ^3^Pnhrc, King Saud University; ^4^Department of Radiological Sciences, College of Applied Medical Sciences, King Saud University

##### **Correspondence:** Rabih Halwani – Prince Naif Center for Immunology Research and Asthma Research Chair, Department of Pediatrics, College of Medicine, King Saud University, Saudi Arabia

**Background.** Optimal asthma symptom control cannot always be achieved in severe cases due to the risk of steroid resistance and lack of response to beta-agonists. Therefore, alternative approaches are needed to achieve reversal of pathological airway remodelling in steroid resistant severe asthma. IL-4 and IL-13 cytokines are critical for asthma pathogenesis as they modulate IgE synthesis, chemokine production, airway eosinophilia, smooth muscle hyperplasia, and mucus production during asthma. During the past decade, several nanoparticles approaches have been developed and tested for efficient drug and anti-inflammatory agents delivery at various body sites during cancer other chronic inflammatory diseases. The current study evaluated the anti-inflammatory responses following intratracheal instillation of functionalized and PEGylated nanocarriers containing blocking anti-IL4Rα antibodies to the inflamed lungs of asthmatic mouse model.

**Methods.** Anti-IL-4Rα loaded nanoparticles were administered intrapulmonary to asthmatic mice. Particles distribution within the lungs were then examined and their targeting of specific IL-4R+ inflammatory cells was investigated using MRI and histological analysis (immunohistochemistry, immunofluorescence). Multiple gene expression studies (RT-PCR), flow cytometry, cytokine arrays (Luminex) and histological analyses were performed on treated asthmatic lungs tissue and cells to evaluate the anti-inflammatory responses of these nanocariers.

**Results.** Targeting and localization of nanocarriers with Perl’s (iron), PEG (anti-PEG antibodies), immunofluorescence (SPIO nanocarriers with FITC labelled antibody) confirmed their localization with anti-IL-4R+ lung inflammatory cells. Following treatment of asthmatic mice with anti-IL-4R nanocarriers, a significant decrease in BAL levels of IL-1β, IL-10, IL-6, IL-13, GM-CSF, IL-5, IL-2, IL-4, MCP-1, IP-10/CXCL-10, MIG and IFN-γ was observed compared to Ova-sensitized mice. BAL levels of lymphocytes, neutrophils and eosinophils decreased significantly (p<0.01) in mice receiving anti-IL-4Ra loaded nanocarriers. Lung inflammation was significantly decreased as observed in histological analysis as well as gene expression of inflammatory cytokines (genes). In addition, a significant decrease in activation and functionality of lung inflammatory cells was observed suing FACS analysis following treatment with the IL-4R-nanocarrier.

**Conclusions.** Treatment of inflamed lungs with anti-inflammatory IL-4Rα-nanocarriers was safe and efficient in reducing lung inflammation and controlling asthma pathogenesis. This approach could be effective in attaining asthma control in severe cases where alternative approaches for steroids are needed.

## A539 Clinical Factors for Improved Allergen Reactivities Induced By Subcutaneous Allergen Specific Immunotherapy with House Dust Mites during 1 Year Period

### Hee-Kyoo Kim^1^, Chul-Ho Oak^1^, Gil-Soon Choi^1^, Ye-Jin Moon^2^, Eun-Kee Park^3^

#### ^1^Kosin University Gospel Hospital, South Korea; ^2^Kosin University; ^3^College of Medicine, Kosin University

##### **Correspondence:** Hee-Kyoo Kim – Kosin University Gospel Hospital, South Korea

**Background:** Allergen specific immunotherapy(SIT) is able to significantly improve symptoms as well as reduce the need for symptomatic medication. The SIT acts on basic immunologic mechanisms, and has the potential to change the pathological allergic immune response. The immunologic changes by treatment are achieved for a certain period of time, usually over 3~5 years. By the way, some patients present reduced allergic responses through immunotherapy in early stage. Immunologic responses to allergen may affect favorable clinical outcome during 1 year treatment.

**Methods:** From 2009 to 2014, the patients with allergic airway diseases who received subcutaneous SIT with house dust mites and both allergen skin prick tests before therapy and after it on time about 1 year period were investigated for retrospective analysis. The main parameters of immunologic changes were skin reactivities(wheal ratio of allergen/histamine in skin prick test) for house dust mites ongoing immunotherapy. The numbers of positive allergen skin responses and some blood tests for eosinophil count, total IgE, ESR were collected as well.

**Results:** Total 58 patients were analysed, the mean ages at initial immunotherapy time were 23.3 year old (5~60), males were 34 (58.6%), allergic rhinitis only were 35 (60.3%), bronchial asthma only were 1 (1.7%), both were 22 (37.9%). The median numbers of strongly sensitized allergens among 45 aeroallergens were two items at initial phage. The mean skin reactivities for target allergens before and after 1 year immunotherapy were 2.44 (0.5~7.75) and 1.19 (0~2.92), respectively. The skin reactivity for target allergen of ongoing immunotherapy were significant reduced in younger age group(<40 years old) (p=0.45 ), the groups who had small numbers of total allergen sensitization (p<0.05), house dust mites(HDM) sensitization only rather than those of mixed with HDM and pollens(p<0.05) and high reactivity(≥2 on A/H ratio) of target allergens (p<0.001) at initial time. But, there were not significant different among initial immunotherapy methods(rush or conventional), underline allergic airway disease, eosinophil count, total IgE and ESR level.

**Conclusion:** This study suggests some clinical factors associated favorable allergen reactivity change may affect good clinical outcomes in early phase of immunotherapy.

## A540 Cytokine Gene Polymorphisms in Iranian Patients with Kidney Acute Rejection

### Mina Abrari^1^, Ali Akbar Amirzargar^2^, Alireza Zarebidoki^3^

#### ^1^Department of Cell and Molecular Biology, University of Tehran; ^2^Department of Immunology, Tehran University of Medical Sciences; ^3^Molecular Immunology Research Center (MIRC), Tehran University of Medical Sciences, Iran

##### **Correspondence:** Alireza Zarebidoki – Molecular Immunology Research Center (MIRC), Tehran University of Medical Sciences, Iran

**Background:** Acute rejection (AR) in kidney transplantation is one the most important causes of rejection in Iran and the world. Considering the role of inflammatory cytokines in this process and due to this fact that genetic polymorphisms can alter the function of these cytokines, we aimed to evaluate various single nucleotide polymorphisms (SNPs) related to TNF-α, IL-6, IFN-γ and IL-1β cytokine genes.

**Methods:** Genomic DNA was extracted from whole blood of 56 patients with acute rejection, and 56 patients with a stable graft function (SGF). A Polymerase chain reaction with the sequence specific primers (PCR-SSP) was performed using related kits. The results were analyzed by statistical software SPSS and Epiinfo.

**Results:** The frequency of A and G alleles related to -308 and -138 positions of TNF-α, and alleles C and G of the -174 position related to IL-6 showed a significant association in patients with a transplanted kidney (Both AR and SGF) compared to controls (P value<0.05). Data related to both TNF-α SNPs, and GG, CG genotypes of -174 position (IL-6) revealed a significant relationship between AR and healthy controls. In addition, results from the comparison of SGF and healthy controls in -238(TNF-α) and -174(IL-6) positions showed a significant correlation. Haplotype analysis among study groups also displayed statistically significant associations.

**Conclusion:** This study found an association between TNF-α and IL-6 gene polymorphisms in kidney graft rejection or survival processes. More studies with greater samples of various populations are needed in order to confirm this finding.

## A541 Phthalate Exposure and Obesity in Atopic Dermatitis of Korean Children and Adolescents

### Mina Ha^1^, Soo-Jong Hong^2^, Ju-Hee Seo^3^

#### ^1^Dankook University College of Medicine; ^2^Department of Pediatrics, Childhood Asthma Atopy Center, Environmental Health Center, Asan Medical Center, University of Ulsan College of Medicine; ^3^Department of Pediatrics, Korea Cancer Center Hospital, South Korea

##### **Correspondence:** Ju-Hee Seo – Department of Pediatrics, Korea Cancer Center Hospital, South Korea

**Background**

Phthalates are widely used in our daily lives, including flooring, toys, food wrapping, plastic ware, emulsifying agent, lotion and shampoo. Several studies reported the association between the exposure to phthalates and allergic disorders. Also, some conflicting results reported whether atopic dermatitis is associated with overweight or obesity. We evaluated the association of phthalate exposure and overweight/obesity in atopic dermatitis (AD) of Korean children and adolescents.

**Methods**

The nationwide representative survey was conducted with 1820 children and adolescents aged 6 to 18 years in Korea. The information of atopic dermatitis was collected by the International Study of Asthma and Allergies in Childhood (ISAAC) questionnaire. Urine monobenzyl phthalate (MBzP), mono-n-butyl phthalate (MnBP), mono-2-ethyl-5-carboxypentyl phthalate (MECPP), mono-2-ethyl-5-hydroxyhexyl phthalate (MEHHP) and mono-(2-ethyl-5-oxohexyl) phthalate (MEOHP) were measured. All phthalate metabolites were adjusted with urine creatinine. The subjects, who were overweight or obese, were defined that body mass index was between 85 and 95 percentile or over 95 percentile of age and gender, respectively.

**Results**

The prevalence of AD diagnosis and current AD (diagnosed and presented symptoms during recent 12 months) was 29.4% and 16.4%, respectively. The proportion of subjects who were overweight and obese was 13.3% and 6.7%, respectively. We divided 4 groups according to the urinary phthalate levels (below or over average) and BMI (normal or overweight/obese). The subjects who were overweight or obese and exposured to higher urinary MnBP, MEOHP or MEHHP were the highest odds ratios compared to other groups in diagnosis of atopic dermatitis [OR(95% CI) 1.584(1.043-2.406) in MnBP; 1.632(1.066-2.498) in MEOHP; 1.584 (1.049-2.393) in MEHHP)]. The subjects who were overweight/obese and exposured to higher urinary MnBP or MEHHP were the highest odds ratios compared to other groups in current symptoms of atopic dermatitis [OR(95% CI) 2.091(1.294-3.378) in MnBP; 1.797(1.101-2.932) in MEHHP)].

**Conclusion**

Overweight or obesity and exposure to higher phthalate, especially to MnBP, MEOHP or MEHHP could be associated with an increased prevalence of diagnosis or current symptoms in atopic dermatitis.

This study was supported by the National Institute of Environmental Research (NIER).

## A542 Which Drives Chronicity of Cough in Adults: Based on the Knhanes 2010-2012

### Mingyu Kang^1,2^, Byung-Ha Cho^1^, Han-Ki Park^3^, Han-Ki Park^4^, Kyung-Mook Kim^5^, Chang-Han Park^6^, Heung Woo Park^7^, Heung Woo Park^2^, Yoon-Seok Chang^8^, Yoon-Seok Chang^2^, Yoon-Seok Chang^7^, Sook-Hee Song^9^, Mi-Kyeong Kim^10^, Mi-Kyeong Kim^1^, Sang-Heon Cho^11^, Suk-Il Chang^6^, Kyung-up Min^7^, Kyung-up Min^2^, Alyn Morice

#### ^1^Chungbuk National University Hospital, South Korea; ^2^Seoul National University Medical Research Center, Seoul, South Korea; ^3^Institute of Allergy and Clinical Immunology, Seoul National University Medical Research Center, Seoul, South Korea; ^4^Ewha Institute of Convergence Medicine, Ewha Womans University Medical Center; ^5^Pogunhan Mom Hospital, Seoul, South Korea; ^6^Sung-Ae General Hospital, Seoul, South Korea; ^7^Seoul National University College of Medicine, Seoul, South Korea; ^8^Seoul National University Bundang Hospital, Seoul, South Korea; ^9^Seoul Medical Center, Seoul, South Korea; ^10^Chungbuk National University, Cheongju, South Korea; ^11^Seoul National University Hospital, Seoul, South Korea

##### **Correspondence:** Mingyu Kang – Chungbuk National University Hospital, South Korea

**Background:** Recent meta-analyses demonstrated a high global epidemiological burden of chronic cough in general populations. However, there is still a lack of nationwide studies on the epidemiology of cough in general Korean populations.

**Objective:** We aimed to calculate the nationwide prevalence of current cough, and to explore the factors underlying the epidemiology in Korean general adult populations.

**Methods:** We analyzed cross-sectionally the Korean National Health And Nutritional Examination Survey (KNHANES) database surveyed during 2010-2012. Presence and duration of current cough was defined by structured questionnaires, and classified as acute (<3 weeks), subacute (3-8 weeks), and chronic (>8 weeks). Demographic and clinical parameters were also examined in relation to cough. Weighted prevalence of cough according to duration was calculated, and risk factors with cough were evaluated by using multivariate logistic regression.

**Results:** Among a total of 18,071 adults, 5.9% (n=1,076) had current cough. Among the subjects with current cough, proportions of acute, subacute, and chronic cough were 43.1%, 13.1%, and 43.8%, respectively. Of those with chronic cough, 65.4% had been coughing longer than 1 years. The epidemiology of cough significantly differed between age groups; acute cough was more prevalent in younger adults (18-45 years old), whereas chronic cough was significantly more prevalent in elderly subjects (≥65 years old). Prevalence of chronic cough increased with aging in both gender (in males, 2.4% in subjects aged 18-45 years, 3.4% in subjects aged 45-65 years, and 5.4% in subjects aged ≥65 years; in females, 1.2%, 1.9% and 3.9%, respectively; both p for trends <0.01). After adjusting for demographic and risk factors, several factors were associated with chronic cough independent of age: current smoking, chest X-ray abnormality, HbA1c and rhinitis or rhinosinusitis (all p<0.05). In female, menopause and estrogen replacement therapy were also related with chronic cough (all p<0.05).

**Conclusion:** The present study indicates a considerable epidemiological burden of cough in Korean general adult populations. The epidemiology of cough significantly differed between age groups. These findings warrant further elucidation for various factors underlying a high burden of chronic cough in older adults.

## A543 Elevated Airway CD45RO Memory Cells in Wheezing Children with Lower Respiratory Infection

### Jungi Choi^1^, Yusok Han^1^, Jin-Sung Park^1^, Eunmi Kwon^1^, Chang-Keun Kim^2^

#### ^1^Sanggye Paik Hospital, South Korea; ^2^Inje University Sanggye Paik Hospital

##### **Correspondence:** Jungi Choi – Sanggye Paik Hospital, South Korea

**Introduction:** No data on airway T cell subsets in “wheezing” children (WC) is available. CD25, found on activated T cells and CD45RO “memory”cells, associated with T cell development, T cell activation and IgE synthesis, were analyzed in BAL from WC and normals.

**Methods:** We evaluated 18 WC with a minimum of 2 episodes of wheezing or prolonged wheezing ≥2 months in a 6 month period with BAL. WC were further sub-divided into children with history of RSV (WC+RSV)(n=10, median age 17.0 months) or without RSV (WC-RSV)(n=8, median age 9.3 months). Comparisons were made with normal controls (Control)(n=5, median age 19.5 mos). BAL cell counts and differentials were determined, and 10^6^ cells were fixed in acetone and embedded in glycol methacrylate resin. Cell blocks were cut and immunohistochemically stained for the following T cell surface markers: CD3, CD4, CD8, CD25, and CD45RO. Co-expression of CD25 or CD45RO on CD3, CD4, and CD8 T cells was evaluated by an image analysis program on sequential sections. Comparisons between all the groups were first made using the Kruskal-Wallis test. In case of significant difference, individual groups were compared by using the Mann-Whitney U test.

**Results:** The total number of CD3 cells was significantly elevated in the WC+RSV compared with the Control (p=0.008). WC+RSV had significant (p=0.003) elevations in # of CD45RO cells/1000 cells compared to Control and the total number of CD45RO was significantly elevated in the WC+RSV compared with the WC-RSV (p=0.033) and the Control (p=0.001). Although the % of CD4 T cells expressing CD45RO was greater in WC+RSV than WC-RSV, the elevation was only statistically significant compared to Control (p=0.005).

**Conclusions:** These findings suggest that specific T cell profiles exist in WC+RSV which differ from WC-RSV and Control. Furthermore CD45RO cells may play a role in the immune processes of WC+RSV.

## A544 Quality of Life in Obese Children with or without Atopic Disease

### Gartika Sapartini^1,2^

#### ^1^Faculty of Medicine, Universitas Padjadjaran, Indonesia; ^2^Dr. Hasan Sadikin General Hospital, Indonesia

##### **Correspondence:** Gartika Sapartini – Faculty of Medicine, Universitas Padjadjaran, Indonesia

**Background:** Obesity and atopic disease are two increasingly important population health issues. Obesity could be a risk factor for atopic disease. To have a full vision of the health condition of the patients with obesity and atopic disease, it is important for the pediatricians to make a Quality of Life (QOL) Questionnaire as part of the patient’s follow-up. Few studies suggest that obesity decreases QOL in children with atopic disease such as asthma. The aim of this study was to evaluate the QOL in obese children with or without atopic disease.

**Methods:** A cross-sectional study with an initial sample of 109 children (6-11 years old) recruited from some private elementary schools in Bandung, Indonesia. The subjects were divided into obese children with or without atopic disease and normal weight children with or without atopic disease. The standard definition for obesity and atopic disease in children was based on the World Health Organization. To determine the effect of obesity and atopic disease on QOL, the Pediatrics Quality of Life Inventory™ (PedsQL™) was applied to the subjects and also their parents. Mann-Whitney test was used to test the significance of categorical data and significance was determined by p < 0.05.

**Results:** Out of 109 patients in the study, 54.1% were males and 45.9% females, with a ratio of 1.2:1 and a mean age of 9.4 years ± 1.58 (standard deviation). The results of the four scales evaluated in the questionnaire (physical, emotional, social, school functioning), showed significant differences for the emotional functioning in QOL between obese-atopic disease and obese-non atopic disease groups (p = 0.04), but no significant differences in other scales.

**Conclusions:** This study revealed a significant differences for the emotional functioning in QOL between obese-atopic disease and obese-non atopic disease groups, but no significant differences in other scales. Further studies is needed to understanding the relationship between obesity and atopic disease to the patient’s QOL.

**References**

vant Gent R, van der Ent CK, Rovers MM, et al. Excessive body weight is associated with additional loss of quality of life in children with asthma. J Allergy Clin Immunol 2007;119:591–96.

Vijil VB, Navarro DR, Eslava AB, Monge JJLS. Quality of life in pediatric patients with asthma with or without obesity: a pilot study. Allergol et Immunopathol 2004;32(5):259–64

## A545 Relation of Human microRNA in Sputum of Asthma with Influenza A Virus Infection-Induced Exacerbation

### Ji-Na Kim^1^, Seungwoo Shin^2^, Hun Soo Chang^2^, Eun-Young Shim^2^, Ji Ah Jun^1^, Hyeonju Lee^1^, Jong-Sook Park^2^, Choon-Sik Park^2^

#### ^1^Soonchunhyang University, South Korea; ^2^Soonchunhyang University Bucheon Hospital

##### **Correspondence:** Ji-Na Kim – Soonchunhyang University, South Korea

**Background:** Exacerbations of asthma are most frequently attributed to upper and lower airway infections by respiratory viruses such as rhinovirus and influenza viruses. Host cells protect against these virus via innate and acquired immune responses. MicroRNAs act as key regulatory molecules in complicated interaction networks between viruses and hosts. However, a few studies have been performed on miRNA related with influenza infection. The aim of this study is to search for candidate miRNAs by in silico analysis, and validate the relation of the miRNAs in sputum with exacerbation of asthma.

**Methods and Materials:** Information on sequences of mature human miRNAs was obtained using miRBase (http://www.mirbase.org) and applied to the whole genome sequence of influenza viruses A (http://www.ncbi.nlm.nih.gov/) by searching complementarity. Viral RNA and miRNA were extracted from sputum of exacerbated asthmatics using Viral Gene-spin¢â kit (iNtRON Biotechnology, Seoul, Korea) and miRNeasy kits (Qiagen, CA, USA), respectively. RT-PCR and Real-time PCR were applied to the discovery of 7 respiratory viruses (adenovirus, human metapneumovirus, parainfluenza virus 1/2/3, influenza A/B virus, respiratory syncytial virus A/B and rhinovirus A) and the measurement of miRNAs, respectively.

**Results:** Total 2578 human miRNAs were used for analysis. Among them, miR-23b-3p was predicted to match with 7 nucleotides in one location +1915-+1921 of polymerase basic protein 2 mRNA and to three locations +244-+251, +1446-+1453 and +1470-+1477 of haemagglutinin mRNA of influenza A. Respiratory viruses were identified in 21 patients out of total 37 patients and 5 patients were infected by influenza A virus. The levels of miR-23-3p were much lower in patients infected with influenza A and rhinovirus (n=9) than those of other virus-infected (n=7) or uninfected patients (n=16). The levels of miR-23b-3p in influenza A-infected patients were comparable with those in rhinovirus-infected subjects.

**Conclusions:** Down regulation of miR-23b-3p expression may be associated with infection with influenza A virus and might exert a negative regulatory activity on the influenza A virus replication.

## A546 Aeropolinologic Monitoring and Distribution of Allergoallergens in Western Georgia

### Revaz Sepiashvili^1,2^, Darejan Khachapuridze^1^, Sofio Gamkrelidze^1^, Manana Chikhladze^1^

#### ^1^Institute of Allergy Asthma and Clinical Immunology, Georgia; ^2^Peopels Friendship University of Russia, Russia

##### **Correspondence:** Revaz Sepiashvili – Institute of Allergy Asthma and Clinical Immunology, Georgia

Due to the high frequency of allergic diseases and its special growing rate among the world population, in medicine, the 21st century is called the century of allergy. It is known that allergic diseases (pollinosis, bronchial asthma), the highest percentage comes on the allergens- aeropolutants, that are represented in many plants and herbs in the form of dust (ragweed pollen, alder, birch, maple, walnut, mallow, cotton plant etc).

Since 2006, Institute is actively working in this direction gradually after the World Allergy Organization (WAO) presented the apparatus - Burkhard Pollen Trap (UK) to the Institute.

Research is actively continued in this direction and the results are published periodically in international journals and became public as the reports at scientific congresses.

According to the all above-mentioned, the aim of the study at this stage is as follows: identification of specific aeropollutants and elaboration of annual calendar of plants blossoming for the reality of Imereti region.

In this study have been involved 69 patients of different ages (among them 34 males and 35 females) with allergic rhinitis and asthma, who applying to the S/R Institute of Allergy, Asthma and Clinical Immunology of Academy of Sciences of Georgia (Tskaltubo, Georgia) for allegro-diagnostics, revealed increased levels for Phadiatop in blood, on the existence of atopic allergen only to the inhaled allergen.

The study covered the following allegro-diagnostic stages:

I stage - For precise verification of the allergen, patient’s blood serum was examined on a particular specific-IgE antibodies by modern automated system - "Immuno CAP 100" (PHADIA, Switzerland).

II stage - Monitoring of the concentration of aeropoluments was conducted by using aeropolinometer “Burkard Trap” (Great Britain).

The analysis of the laboratory results, obtained through the automated system "ImmunoCAP 100"* showed that the studied patients had high titers of specific IgE on the weeds (Wx2) - ambrosia, plantain, absinth, atriplex 47 (68%); tree dust (Tx9) - alder, lactarius piperatus, nuts, oak, willow - 21 (30%); cereals (Gx1) - festuca pratensis, lolium temulentum, timoti grass, poa - 19 (28%).

A specific IgE concentration was detected for each positive panel, to reveal the concrete allergen.

The mentioned patients were provided with: the data of aero-polinometer- "Burkard Trap", annual calendar for distribution of aeroallergens reflecting concentrations of blossoming plant-trees and atmospheric aerosols in the air in Imereti region at a given period of time.

## A547 Extracorporeal Membrane Oxygenation As Emergency Treatment for Patients with Near-Fatal Status Asthmaticus

### Seung-Eun Lee, Yun-Seong Kim, Doo-Soo Jeon, Woo-Hyun Cho, Hye-Ju Yeo, Seong-Hoon Yoon, Seung-Hyun Kim

#### Yangsan Pusan National University Hospital, South Korea

##### **Correspondence:** Seung-Eun Lee – Yangsan Pusan National University Hospital, South Korea

Extracorporeal membrane oxygenation (ECMO) has been used primarily to treat respiratory failure due to acute respiratory distress syndrome that failed to respond to maximal medical therapy. The use of ECMO in status asthmaticus is limited to case reports. We present three cases of patients with near-fatal status asthmaticus not relieved by conventional treatment, in whom early administration of ECMO resulted in a good outcome. In case 1 and 2, ECMO was instituted because of sustained hypercapnia and respiratory acidosis within 2 hours after initiation of mechanical ventilation. Patient 3 was supported by ECMO at 10 hours after intubation because of severe hypotension and hypercapnia. The lung status in these patients was rapidly recovered within days, and they were extubated at 31, 67 hours and 4 days after initiation of ECMO, respectively. Successful weaning of ECMO was complete the next day after extubation in all patients. There was no significant complication related with ECMO in these patients. Mechanical ventilation for the patients with refractory status asthmaticus can have deleterious effects due to worsening dynamic hyperinflation and increase intrathoracic pressure. Early ECMO application is a useful treatment options for these patients failed to conventional therapy.

## A548 Relationship of S100calcium Binding Protein A9 with Neutophilic Inflammation in Murine Asthma Model

### Taehyeong Lee^1^, Hyun Ji Song^2^, Choon-Sik Park^2^, Ji Ah Jun^1^, Jong-Sook Park^2^

#### ^1^Soonchunhyang University, South Korea; ^2^Soonchunhyang University Bucheon Hospital

##### **Correspondence:** Taehyeong Lee – Soonchunhyang University, South Korea

**Background:** We previously reported elevation of S100 calcium binding protein A9 (S100A9) protein in sputum of neutrophilic uncontrolled asthmatics compared with stable asthmatics, [Annels of allergy, asthma, immunology on 2013 Oct;111(4):268-275], suggesting the possible role of S100A9 in neutrophilic severe asthma. The aim of this study was to validate the role of S100A9 expression in neutrophilic airway inflammation of OVA/CFA-sensitized/challenged murine asthma model.

**Methods:** Expression of S100A9 mRNA and protein was measured using a RT-PCR and western blot, respectively. Spatial expression of S100A9 protein and neutrophil elastase was visualized using immunofluorescene stain and confocal microscopy. To evaluate the potency of S100A9 on neutrophilic inflammation, temporal changes of neutrophil infiltration was analyzed in lung tissues of the OVA/CFA model and in those of C57BL/6 mice after intra-tracheal S100A9 protein (10ug) administration. To block the S100A9 in the airway, mice were treated with 20ul of anti-S100A9 antibody via intra-nasal route at 3 hour before challenge of S100A9.

**Results:** S100A9 started to increase from day 14 and peaked at day 23 while the number of total cells, macrophage and neutrophils significantly increased with a concomitant escalation of airway hyper-responsiveness (AHR) at day 23. Neutrophil elastase and S100A9 were co-localized in peri-bronchially infiltrating cells and in apical portion of bronchial epithelium. Intra-tracheal instillation of S100A9 induced a rapid increase of neutrophils in BAL fluid from 2 hr, which peaked at 24hr, and thereafter progressively decreased till 80 hr. Intra-nasal administration of anti-S100A9 antibody restored the increase of the numbers of inflammatory cells and AHR in CFA/OVA mice to those of sham mice.

**Conclusion:** S100A9 directly activates neutrophilic inflammation into the airway and neutrophils may be the major cell source of S100A9 protein, which suggests main role of the S100A9 protein in neutrophilic experimental asthma.

## A549 Whole-Exome Sequencing of Aatopic Dermatitis in Korean Childhood

### Dankyu Yoon^1^, Yeon-Seop Kim^1^, Woo-Sung Chang^1^, Mi-Jin Kang^2^, Soo-Jong Hong^3^, Jeom-Kyu Lee^1^, Eun-Jin Kim^1^

#### ^1^Korea National Institute of Health, Korea Center for Disease Control and Prevention, South Korea; ^2^Asan Institute for Life Sciences; ^3^Department of Pediatrics, Childhood Asthma Atopy Center, Environmental Health Center, Asan Medical Center, University of Ulsan College of Medicine

##### **Correspondence:** Dankyu Yoon – Korea National Institute of Health, Korea Center for Disease Control and Prevention, South Korea

**Background:** Atopic dermatitis (AD) is a complex and heterogeneous disease influenced by genetic and environmental factors. In the last decade, previous efforts in finding AD associated loci have identified unprecedented amount of associated loci. However, the previously reported genetic loci explain only a small proportion of the heritability. Thus, further analysis on unrevealed genetic component is required to solve the missing heritability problem. In this context, whole-exome sequencing analysis have gathered much attention to find functional variants with relatively high genetic effects. In the present study, we performed whole-exome sequencing study to identify functional variants responsible for severe AD in Korean childhood.

**Methods:** The case-control samples of 32 severe AD patients and 20 normal individuals were recruited from the Childhood Asthma Atopy Center of Asan Medical Center, diagnosed by physician. The cases and controls were sequenced by Illumina Hi-Seq 2500 with Agilent SureSelectXT Human ALL Exon V4+UTR Kit. Sequence alignment was performed using Burrows-Wheeler Aligner. Variants were called using Genome Analysis Toolkit (GATK). We identified 233,254 single nucleotide variants (SNVs), of which 131,321 SNVs passed quality control criteria (HWE p-value<10-3, Missing rate >10%, Minor allele count <2). Of these, 34,991 variants were non-synonymous SNPs. A Chi-square test was conducted to find disease-associated variants.

**Results:** We identified three missense variants in a 112bp window at ZNF443(p-value<10^-4^). For p-value<10^-2^, 137 missense variants were discovered. Among them, 2 variants at NLRP10 and CYP24A1 were located nearby previously reported AD associated regions. Functional enrichment analysis showed that 47 SNVs were related with immune related genes such as ZBP1, FBXO38, MTR, TTN.

**Conclusion:** In summary, we identified 137 missense variants susceptible to AD and replicated 2 previously reported loci. To validate the genetic effect of discovered variants, the replication analysis in an independent cohort is required.

## A550 A Case of Generalized Molluscum Contagiosum in an Adult Patient with Severe Atopic Dermatitis

### Minkee Park

#### Dankook University College of Medicine, South Korea

Most cutaneous molluscum contagiosum (MC) infection occurs in children, but it may be found in adults, especially who have been infected with human immunodeficiency virus (HIV).

A 37-year-old man presented with multiple pea-sized firm skin-colored round papules, which first appeared on the arms and then quickly spread over whole body for last 2 months. He has been treated for severe atopic dermatitis with systemic immunosuppressive therapies for 2 years. Screening test for HIV was negative. The lesions were diagnosed as MC based on histopathologic findings of typical eosinophilic cytoplasmic molluscum inclusion bodies on the acanthotic epidermis as well as dermal inclusion cyst.

This is a very unusual case because widespread MC infection in adults was reported in some HIV positive patients. We supposed that our patient’s disseminated infection may be related with the combination of perturbed skin barrier and systemic immunosuppressive therapy under severe atopic condition.

## A551 Discovery of Putative Macadamia Nut Allergens By Patient IgE Binding and a Label-Free Shotgun Proteomics Approach

### Nanju Alice Lee^1^, Johanna Rost^1^, Sridevi Muralidharan^1^, Dianne Campbell^2^, Sam Mehr^2^

#### ^1^University of New South Wales, Australia; ^2^Children’s Hospital Westmead

##### **Correspondence:** Nanju Alice Lee – University of New South Wales, Australia

**Backgrounds:** Macadamia nut is a tree nut listed as a major allergen to be labelled on pre-packaged foods globally. At least 19 cases of macadamia nut allergy have been reported to date, however, the eliciting allergenic proteins have not been identified and consequently component resolved diagnosis has not been developed. This study aims to identify putative allergenic proteins in macadamia nut by combining patient IgE recognition with an allergenomics approach. The challenge is that macadamia nut genome sequence is only partially complete.

**Methods:** The proteomic profile was studied using a label-free shotgun proteomics approach. As the genome sequence of macadamia nut allergens is not available, homologies to other known allergens and affiliations to protein families were determined. The results from the allergenomic screening method were used to predict potential allergenic proteins and cross-reactivity with other plants particularly. The molecular weight distribution of proteins was determined by gel electrophoresis. Following in-gel digestion with trypsin, proteins were subjected to liquid chromatography coupled tandem mass spectrometry. Based on the shared peptide evidence, the identified proteins were clustered and the allergenic proteins were identified. Immunoreactive proteins were identified by immunoblotting with three patient sera confirmed to exhibit IgE-mediated reaction.

**Results:** Peptides matched to the sequences of 21 allergenic proteins belonging to different protein families such as seed storage proteins (conglutins and vicilins), rubber elongation factor proteins, phosphate binding proteins and detoxifying methylglyoxalases were identified. This included peptide sequence homologies to 5 conglutins, which are known allergens from *Lupin angustifolius*.

**Conclusion:** Allergenic proteins were confirmed to be seed storage proteins belonging to 11S, 7S and 2S proteins. Significant number of peptide sequence homologies to conglutins from *Lupin angustifolius* was observed*,* suggesting that there may be cross-reaction between macadamia and lupin allergens

## A552 Anti-FcÎμri Antibody Inhibits Allergic March in Mice By Suppressing Th17 Pathway Via Suppression of FcÎμri-Mediated Mast Cells Activation

### Seung-Hwa Lee^1^, Seon-Joo Yoon^2^, Ha-Jung Kim^1^, Eun Lee^3^, Song-I Yang^4^, Young-Ho Jung^5^, Ho-Sung Yu^6^, Hee-Suk Kim^1^, Yeon Hee Park^1^, So-Yeon Lee^7^, Jun-Sung Park^8^, Hyun Ok Jun^8^

#### ^1^Asan Institute for Life Sciences, South Korea; ^2^Neopharm Co., Ltd.; ^3^Department of Pediatrics, Childhood Asthma Atopy Center, Environmental Health Center, Asan Medical Center, University of Ulsan College of Medicine; ^4^Hallym University Sacred Heart Hospital; ^5^Bundang CHA Medical Center, CHA University School of Medicine; ^6^Asan Institute for Life Science, Asan Medical Center; ^7^Department of Pediatrics, Hallym Sacred Heart Hospital, Hallym University College of Medicine; ^8^Asan Medical Center

##### **Correspondence:** Seung-Hwa Lee – Asan Institute for Life Sciences, South Korea

**Background:** Immunoglobulin E (IgE) triggers multiple inflammatory allergic responses and cytokine release when it binds to high-affinity IgE Fc receptor (FcεRI) on mast cells in atopic dermatitis (AD) and asthma. Anti-FcεRI antibody is a new option for treatment that may block IgE-FcεRI binding and therefore may reduce inflammatory cascade in allergic diseases. However, the potential effects and mechanism of anti-FcεRI antibody remain poorly understood.

**Objective:** We investigated the effect and mechanisms of anti-FcεRI antibody by blocking the combination of IgE-FcεRI antibody in allergic march (AM) mice model.

**Methods:** We developed mice model of AM with three 1-week exposures (separated by 2-weeks interval) to an ovalbumin (OVA) or saline followed by OVA inhalation (challenge). In order to develop a mice model of AM, the day before sacrifice, all mice inhaled 1% OVA as the airway challenge. Anti-FcεRI antibody was administered to the mice intraperitoneally for 4 consecutive days before the end of study. Identification of interleukin (IL)-17 expression was performed by immunohistochemistry and real time PCR on skin and lung specimens.

**Results:** Anti-FcεRI antibody treated AM mice had significantly decreased phenotypes (e.g., clinical score, airway hyperresponsiveness, and pathology) of AD and allergic asthma. In addition, the levels of total IgE, OVA-specific IgG1 and IL-13 in serum were significantly lower in AM mice treated with anti-FcεRI antibody. The level of prostaglandin D2 and the number of mast cells in skin were also decreased in the anti-FcεRI antibody treated with AM mice. Furthermore, the skin and lung expressions of IL-17 were reduced after the treatment of anti-FcεRI antibody.

**Conclusion:** IgE-FcεRI blockade by Anti-FcεRI antibody can suppress the IgE-mediated phenotypes and inflammatory responses in AM. And its mechanism may be the decrease in IL-17 via the suppression of FcεRI–mediated mast cell activation.

## A553 Clinical Characteristics and the Associated Factors of ATG Hypersensitivity Reaction

### Ha Kyeong Won^1^, Min-Koo Kang^1^, Sung Do Moon^1^, Byung-Keun Kim^2^, Ju-Young Kim^1^, Sang-Heon Cho^3^, Hye-Ryun Kang^1^, Ji-Su Shim^1^, Soo Jie Chung^1^

#### ^1^Seoul National University Hospital, South Korea; ^2^Seoul National University Bundang Hospital; ^3^Seoul National University Medical Research Center

##### **Correspondence:** Ha Kyeong Won – Seoul National University Hospital, South Korea

**Background:** Anti-thymocyte globulin (ATG) is an immunosuppressant derived from horse or rabbit-immunized with human thymus lymphocytes and commonly used for the prevention and treatment of acute rejection in organ transplantation and aplastic anemia. Hypersensitivity to ATG can be life-threatening but there are not many clinical data from the real practice. Therefore, this study aimed to investigate the clinical characteristics and outcomes of ATG hypersensitivity.

**Methods:** Cases of hypersensitivity reaction to rabbit ATG were retrieved from a database of individual case safety reports in Seoul National University Hospital from 2010 to 2015. Clinical characteristics of hypersensitivity reactions were analyzed according to involved organ system and severity was assessed according to Common Terminology Criteria for Adverse Events (CTCAE) version 4.03.

**Results:** Among 82 patients, male was 36 (44.4%). The average age was 21.5 ± 19.6. High fever (100%) was the most frequent symptom followed by chill (95%) and cutaneous symptoms (65%) such as itching sense, flushing, urticaria and rash. The following majority of symptoms is gastrointestinal symptoms such as nausea, vomiting, abdominal pain and diarrhea. The mean severity was CTCAE grade 2.7 ± 0.9. Although all patients were premedicated with anti-histamine and steroid, 51.2% had grade 3 or 4 reactions including cases presented with profound hypotension (36.0%). After the development of ATG hypersensitivity, most patients were able to continue the following ATG infusion by increasing the doses of anti-histamine and steroid (76.5%) or by slowing infusion rate (13.6%), and desensitization (7.4%).

**Conclusion:** ATG hypersensitivity reactions presented as a severe form in half of cases reported. However, most patients with ATG hypersensitivity were able to continue ATG infusion by increasing premedication or modifying infusion protocol.

## A554 Reference Value and Utility of Total Serum Immunoglobulin E in Korean Schoolchildren

### Jaehee Choi^3^, Kangmo Ahn^1^, Kwanghoon Kim^2^, Jihyun Kim^1^, Jiyoung Lee^1^

#### ^1^Samsung Medical Center; ^2^Medical Research Institute of Pusan National University Hospital; ^3^Sahmyook Medical Center, South Korea

##### **Correspondence:** Jaehee Choi – Sahmyook Medical Center, South Korea

**Background:** Total serum Immunoglobulin (IgE) is known to be an essential diagnostic tool for atopy and allergic diseases. It is difference according to host factors including sex, age, races. We evaluated the distribution of total IgE levels in Korean children and utility in the diagnosis of atopy and allergic diseases.

**Methods:** In this nationwide cross-sectional study, 3,753 elementary schoolchildren (6-7 yr olds) and 3,930 middle schoolchildren (12-13 yr olds) were enrolled. Total IgE levels were measured and skin prick tests were performed for 18 common inhalant allergens. Children and parents answered the International Study of Asthma and Allergies in Childhood (ISAAC) questionnaire. We analyzed the diagnostic value of total IgE by using ROC (Receiver operating characteristic) curve and compared total IgE levels according to atopy and allergic diseases such as atopic dermatitis, bronchial asthma and allergic rhinitis.

**Results:** The total IgE ranged from 1.5 to 4,523.1 kU/L in elementary schoolchildren and 1.5 to 3,000 kU/L in middle schoolchildren. The median total IgE level was 86.7 kU/L (75th percentile, 292.6 kU/L; 90th percentile, 698.5 kU/L; 95th percentile, 1,200.7 kU/L) in elementary schoolchildren and 94.7 kU/L (75th percentile, 284.3 kU/L; 90th percentile, 625.1 kU/L; 95th percentile, 990.7 kU/L) in middle schoolchildren. The median total IgE level was significantly higher in atopic group defined as any positive SPT (elementary schoolchildren, 246.5 kU/L; *P*<0.0001, middle schoolchildren, 206.1 kU/L; *P*<0.0001) and any allergic disease group (elementary schoolchildren, 108.3 kU/L; *P*<0.0001, middle schoolchildren, 141.2 kU/L; *P*<0.0001). In ROC analysis of total IgE for diagnosing atopy, AUC was 0.7835 (95% CI, 0.7688-0.7982) in elementary schoolchildren and 0.8165 (95% CI, 0.8032-0.8297) in middle schoolchildren. At the optimal cut-off value of 127.7 kU/L in elementary schoolchildren and 63.0 kU/L in middle schoolchildren, sensitivity, specificity, and positive and negative predictive values were 67.06%, 75.38%, 65.44%, and 76.70% in elementary schoolchildren and 81.91%, 66.63%, 75.01% and 75.08% in middle schoolchildren respectively.

**Conclusions:** Total serum IgE level was higher in children with atopy or allergic diseases, but the value of total serum IgE as a diagnostic test for atopy is limited due to the low sensitivity and specificity.

## A555 Two-Step Prescreening Skin Testing May be Useful for Reducing Immediate Hypersensitivity Reaction to Nonionic Contrast Media: Results of 7-Year Period in a Secondary Hospital

### Bo Bae Park, In Young Nho, Chang-Han Park, Jang Min Kim, Suk-Il Chang

#### Sung-Ae General Hospital, South Korea

##### **Correspondence:** Bo Bae Park – Sung-Ae General Hospital, South Korea

**Background**

Severe immediate hypersensitivity reaction (IHR) to contrast media (CM) is one of the legal problems in the hospital. The overall prevalence of IHR was 0.16%-7.7% with nonionic CM. In order to reduce immediate drug adverse reaction and legal problem, prescreening skin testing has been before administration of antibiotics such as penicillin and cephalosporin. It may be important to avoid cross-reactive agent and to select the alternative safe drug. Clinical value of CM prescreening skin testing is controversy. CM prescreening skin testing might be useful for reducing the IHR. We aimed to evaluate the incidence of IHR to nonionic CM in two-step prescreening skin testing group.

**Method**

This is a retrospective study. We reviewed CT cases performed between Jan. 2008 and Dec. 2014. All patients were performed skin testing just before their pending nonionic CM-enhanced computed tomogram (CT). Skin testing and CT scan were performed through two-step process. One step; 1^st^ Skin test was performed with a common nonionic CM. If 1^st^ skin test negative, nonionic CM-enhanced computed tomogram (CT) was performed. Second step; If 1^st^ skin test positive, 2^nd^ skin tests were performed with two other nonionic CM and saline (negative control). If 2^nd^ skin test negative, 2^nd^ skin test negative nonionic CM-enhanced CT was performed. If 2^nd^skin test positive, clinician gave patients premedication before enhanced CT or exchanged alternative radiologic test without CM.

**Results**

IHR were noted in 21 of total 26638 patients (0.08%). 5 of 26638 patients (0.01%) had severe IHR. Symptoms of IHR were nause/vomiting, erythema/urticaria/pruritis,dyspnea, chest tightness, angioedema, hypotension and syncope.

**Conclusion**

Nonionic CM prescreening skin testing through two-step process may be useful for reducing IHR and for selecting safe CM.

## A556 Prevalence of Allergic Sensitization in Patients with Allergy Rhinitis; Gwangju, Jeonnam State Study

### Sun Kyung Kim^3^, Hyung Chae Yang^1^, Kwang Il Nam^2^

#### ^1^Chonnam National University Medical School and Chonnam National University Hospital; ^2^Chonnam National University Medical School; ^3^Gwangju Veterans Hospital, South Korea

##### **Correspondence:** Sun Kyung Kim – Gwangju Veterans Hospital, South Korea

**Objective**

The evaluation of trigger allergen is important for the treatment and prevention of allergic rhinitis (AR). Several nationwide studies revealed that there were regional differences in prevalence of allergy sensitization for individual allergens and allergen types. In this study, using MAST CLA, we evaluate the allergen in AR patients in state level; Gwangju, Jeonnam area. We evaluated the differences in allergic sensitization across the levels of urbanization.

**Methods**

A total of 873 patients from Gwangju-Jeonnam region with allergic symptoms underwent MAST. Total nasal score (TNS) and serum total IgE were evaluated. 39 panels were evaluated in MAST and allergens with results greater than class 2(≥0.7 IU/mL) in considered as positive. Prevalence and distributions of allergen-specific IgEs and their correlations to serum total IgE and TNS were analyzed according to levels of urbanization and age.

**Results**

The mean age of study population was 30.3 years. A total of 873 patients enrolled in this study; 513 patients from urban, 327 patients from rural regions. Among study population, 65.8% had positive test results to at least 1 of the 39 allergen and 80.1% of patients had positive test results to multiple allergen. The prevalence of allergic sensitization differed significantly by age. Younger patients tend to had positive test result to perennial allergen. However, older patients tend to had positive test result to seasonal allergen. There was no difference in positive test result between patients from urban and rural areas.

**Conclusions**

A large portion of the study population is sensitized to indoor and outdoor allergens. However, the overall prevalence of sensitization and the prevalence of sensitization to individual allergens and allergen type showed no differenced according to the level of urbanization in a state.

## A557 Analysis of IgE Binding Components of Walnut in Korean Children Effect of Cooking Methods on the Allergenicity of Walnut Proteins

### Jeongmin Lee^1,2^, Sooyoung Lee^1^, Kyunguk Jeong^1^, Se-Ah Jeon^1^

#### ^1^Ajou University School of Medicine; ^2^Maeil Dairies, Co. Ltd, South Korea

##### **Correspondence:** Jeongmin Lee – Ajou University School of Medicine

**Background:** English walnut (Juglansregia) is one of the most common allergenic foods but the influence of processing on the allergenicity has been scarcely known. This study aims to evaluate the relevant walnut allergens in walnut allergic Korean young children and to examine the influence of different cooking methods on walnut proteins.

**Methods:** Protein extracts from boiled, roasted, and pickled walnuts were compared with that of raw walnut using sodium dodecyl sulfate-polyacrylamide gel electrophoresis (SDS-PAGE). Raw walnut protein extracts were immunoblotted with individual sera from 11 children with levels of walnut specific IgE of 0.7kU/L or higher (ImmunoCAP, ThermoFisher Scientific, Waltham, Mass). Additionally, we produced peanut extract and sera were tested with enzyme linked immunosorbentassay inhibition test.

**Results:** At least 8 or more distinct protein bands (9-108kDa) were visible from raw walnut by SDS-PAGE. The 9-kD (Jug r 3) and 16-kDa (Jug r 1) bands were stable in boiled and roasted walnut. The intensity of9-kD band was even enhanced in roasted and boiled walnut, whereas that of 16-kDa band was slightly decreased in roasted walnut. Meanwhile, the 28-kDa and 60-kDa (Jug r 4) bands profoundly disappeared in roasted walnut. No protein bands were shown in the walnut treated by vinegar for one month. By IgE-immunoblotting with individual sera from walnut-sensitive children, at least 8 protein components were identified. The 60-kDa band corresponding to Jug r 4 wasreacted with 72.5% of patients and the 16-kDa band (Jug r 1) and 9-kDa band (Jug r 3) were reacted with 54.5% of patients. Walnut in the IgE-ELISA was not inhibited by peanut extract.

**Conclusion:** This study indicatesthat IgE-binding bands suspected as Jug r 1, Jug r 3, and Jug r 4 are major problematic allergens in walnut allergic children. Also, the amount of components weighted 28kDa, 47kDa, 60kDawere reduced by heating whereas that of 9kDa and 16kDa were relatively stable.

## A558 Assessment of Autonomic Nervous Function in Subjects with Cholinergic Urticaria Associated with Acquired Idiopathic Generalized Anhidrosis

### Midori Fujiwara, Shoko Shindo, Hiroyuki Murota, Mayuko Tahara, Aya Takahashi, Ichiro Katayama

#### Osaka University Graduate School of Medicine, Japan

##### **Correspondence:** Midori Fujiwara – Osaka University Graduate School of Medicine, Japan

**Background:** Cholinergic urticaria associated with acquired idiopathic generalized anhidrosis (CU-AIGA) strongly impairs quality of life by tingling of skin surface, increased body temperature, and general fatigue in a warm circumstance. The pathogenesis of this disease remains obscure.

**Objective:** The aim of this study was to clarify the relationship between cholinergic urticaria and autonomic function.

**Methods:** Clinical and demographic information of subjects with CU-AIGA consulted to our clinic was summarized retrospectively. Digital blood flow (tissue blood flow, blood volume, and flow velocity) was measured with laser tissue blood flow meter before and after the standing position. Quantitative sudomotor axon reflex test (QSART) was used to measure the sweat volume after administration of acethylcholine by iontophoresis.

**Results:** All subjects with CU-AIGA failed to restore the digital blood flow after standing position indicating the impaired autonomic nerve abnormality. Results in QSART showed extremely decreased sweat volume after stimulation with acethylcholine. After therapeutic intervention (e.g. antihistamines, chinese herbal medicine, or steroid pulse therapy), improved these evaluation items was observed in some cases accompanied with urticarial symptom improvement.

**Conclusion:** These results indicated the possible involvement of abnormal autonomic nervous function in cholinergic urticaria. The knowledge of possible causal relationship between CU-AIGA and autonomic nervous function may contribute to formulating the novel therapeutic strategies for this disease.

## A559 Interleukin 1 Beta in Sputum of Patients with Asthma: Relation with Airway Obstruction and Neutrophilc Inflammation

### Jae Woo Jung^4^, Hyun Ji Song^1^, Taehyeong Lee^1^, An-Soo Jang^1^, Jong-Sook Park^1^, Hun Soo Chang^1^, Choon-Sik Park^2^, Byoung Whui Choi^3^

#### ^1^Soonchunhyang University Bucheon Hospital; ^2^Soonchunhyang University Hospital; ^3^Chung Ang University; ^4^Chung-Ang University Hospital, South Korea

##### **Correspondence:** Jae Woo Jung – Chung-Ang University Hospital, South Korea

**Background:** Interleukin 1 beta (IL-1β), which is produced by inflammasome activation, involves to the various processes of chronic inflammatory diseases. Recently, although inflammsome activation is observed in chronic inflammatory airway diseases, its role in asthma has not yet been studied. The aim of the study was to investigate the relation of sputum IL-1β with inflammatory phenotypes and severity of asthma.

**Methods:** Hypertonic saline induced sputum was obtained from asthma in stable state (n=143) and in exacerbated state (n=48). Differential cell count of induced sputum was done. IL-1β was measured using sandwich ELISA in induced sputum. The levels were analyzed in terms of airway obstruction (FEV1%) and inflammation (neutrophil % and eosinophil % of induced sputum) and exacerbation frequency and lung function over 1 year or longer follow up.

**Results:** IL-1β levels were significantly correlated with the neutrophil cell counts of induced sputum (r=0.186, p=0.010), but not with initial FEV1% in total asthmatics (p>0.05). The correlations of IL-1β levels with neutrophil % (r=0.188, p=0.025) and eosinophil % (r=-0.178, p=0.033) were observed in stable asthmatics. In the exacerbation group, IL-1β levels were inversely correlated with FEV1% (r=-0.326, p=0.024). In long term follow up of stable asthmatics (n=71) over more than 1 year, IL-1β levels were correlated with the follow up FEV1/FVC (r=-0.318 p=0.007). Annual average exacerbation rate was also well correlated with the IL-1β levels in the subjects with neutrophilic inflammation (>70%) in sputum.

**Conclusion:** Sputum IL-1β may be related with neutrophilc inflammation. In exacerbated state and long - term follow up of asthma, sputum IL-1β may also be related with the extent of airway obstruction. These data suggest the IL-1β may participate to airway obsturction and exacerbation frequecy, especially in neutrophilic inflammation.

## A560 Interleukin 8 in Sputum of Patients with Asthma: Relation with Neutrophilc Inflammation and Exacerbation

### Min-Hye Kim^1^, Da-Jeong Bae^2^, Hyun Ji Song^2^, Taehyeong Lee^2^, Ji Ah Jun^2^, Jong-Sook Park^2^, An-Soo Jang^2^, Hun Soo Chang^2^, Young Joo Cho^1^, Choon-Sik Park^2^

#### ^1^Ewha Womans University, School of Medicine, South Korea; ^2^Soonchunhyang University Bucheon Hospital

##### **Correspondence:** Min-Hye Kim – Ewha Womans University, School of Medicine, South Korea

**Background:** Neutrophilic airway inflammation is often observed in non-atopic adult asthma. Interleukin 8 (IL8) is a potent pro-inflammatory cytokine recruiting and activating neutrophils. The relation of IL8 has been revealed with exacerbation of asthma, however its role has not been revealed in terms of prognosis in asthma. The aim of the study was to investigate the relation of sputum IL-8with inflammatory phenotypes, severity and long - term prognosis of asthma.

**Materials and Methods:** Hypertonic saline induced sputum was obtained from asthma in stable state (n=88) and in exacerbation (n=55). Differential cell count was done. IL-8 was measured using sandwich ELISA. The levels were analyzed in terms of airway obstruction (FEV1) and inflammation (neutrophil and eosinophil % of the airway) and exacerbation frequency and lung functions over 1 year or longer follow up.

**Results:** IL-8 levels were significantly correlated with the percentages of neutrophils (r=207, p=0.012) and neutrophils count (r=0.277, p=0.001) in sputum, and inversely with the levels of FEV1% (r=-0.277, p=0.028) in total asthmatics. The correlations of IL-8 levels with percentages of neutrophils (r=0.312, p=0.003) and FEV1% (r=-0.252, p=0.018) were also observed in stable asthmatics. In the exacerbation group, IL-8 levels were inversely correlated with FEV1% predicted values (r=-0.272, p=0.045). In long term follow up over more than 1 year, IL-8levels were positively correlated with annual number of exacerbation (n=109, r=0.227, p=0.017).

**Conclusion:** sputum IL-8 is related with neutrophilc inflammation rather than eosinophilc inflammation of asthma. In long - term follow up of asthma, increase of sputum IL-8 may be one of susceptible factors for frequenct exacerbation.

## A561 Prostaglandin E2 and Transforming Growth Factor-Î^2^ Play a Critical Role in Suppression of Allergic Airway Inflammation By Adipose-Derived Stem Cells

### Sue Jean Mun

#### Pusan National University Yangsan Hospital, South Korea

**Background:** To know the major soluble factors responsible for immunomodulatory effects of adipose-derived stem cells (ASCs) in an asthmatic mouse, we evaluated the effects of ASCs on allergic inflammation in the indoleamine 2,3-dioxygenase knockout (IDO-KO) mice and mice treated with prostaglandin E2 (PGE2) inhibitor and transforming growth factor-β (TGF-β) specific neutralizing antibodies.

**Methods:** ASCs were injected intravenously in wild-type (WT) and IDO-KO asthmatic mice. PGE2 inhibitor and TGF-β neutralizing antibodies were injected intraperitoneally on four consecutive days at the approximate time of ASCs injection. We investigated the immunomodulatory effects of ASCs between WT and IDO-KO mice, WT mice treated with and without PGE2 inhibitor, and WT mice treated with and without anti-TGF-β antibodies respectively.

**Results:** In WT and IDO-KO asthmatic mice, ASCs significantly reduced airway hyperresponsiveness, total inflammatory cells and eosinophils in the bronchoalveolar lavage fluid (BALF), eosinophilic inflammation, goblet cell hyperplasia, and serum total and allergen-specific IgE and IgG1. ASCs significantly inhibited Th2 cytokines (IL-4, IL-5, and IL-13) and enhanced Th1 cytokine (IFN-γ) and regulatory cytokines (IL-10 and TGF-β) in the BALF and lung draining lymph nodes (LLNs). Furthermore, ASCs engraftment caused significant increases the regulatory T cells (Tregs) and IL-10^+^ T cells populations in LLNs. However, when treating mice with PGE2 inhibitor and TGF-β neutralizing antibodies, blocking PGE2 and TGF-β, but not IDO-KO mice, eliminated the immunosuppressive effect of ASCs in allergic airway inflammation.

**Conclusion:** ASCs themselves are capable of secreting PGE2 and TGF-β, which may play a role in inducing Tregs expansion. Furthermore, PGE2 inhibitor and TGF-β neutralizing antibodies eliminated the beneficial effect of ASCs treatment in asthmatic mice, suggesting that PGE2 and TGF-β are the major soluble factors in suppressing the allergic airway inflammation.

## A562 Inhalation of Fine Particles Kill Alveolar Macrophages to Release IL-1alpha That Promote Inducible Bronchus-Associated Lymphoid Tissue (iBALT) Formation

### Etsushi Kuroda^3^, Koji Ozasa^1,2^, Ken Ishii^1,3^

#### ^1^National Institute of Biomedical Innovation, Health and Nutrition; ^2^Yokohama City University Graduate School of Medicine; ^3^WPI Immunology Frontier Research Center, Osaka University, Japan

##### **Correspondence:** Etsushi Kuroda – WPI Immunology Frontier Research Center, Osaka University, Japan

Recently, the number of patients suffering from allergic diseases such as asthma or rhinitis has increased especially in developed countries. The reason is unclear, but many study have demonstrated that particle pollutants such as diesel exhaust and sand dust may exacerbate allergic responses. Furthermore, several nanometer- to micrometer-sized tiny particulates, such as particulate matter 2.5 (PM2.5) that is less than 2.5 micrometers in diameter, could enter into the respiratory tract and settle deep in lungs, causing pulmonary chronic inflammation such as asthma. Most particulates including particle pollutants are considered to function as immune adjuvants to enhance allergen-specific type 2 responses. However, the basis for the adjuvanticity of these particulates and the mechanisms by which they elicit type 2 responses remain poorly understood. Here, we show that particulate induce inducible bronchus-associated lymphoid tissue (iBALT) in the lung as a consequence of cell death of alveolar macrophages and IL-1α release.

Particulate, alum or silica, was administered by intratracheal (i.t.) instillation and then we analyzed the particulate-induced lung inflammation. A histological analysis showed that, in addition to the infiltration of inflammatory cells, many lymphoid clusters, with the size of 100-300 mm in diameter, were induced in the lung. These clusters were mainly composed of B cells, and were characterized by area of B220^+^ and CD21^+^ cells, that is inducible bronchus-associated lymphoid tissue (iBALT). These clusters contained germinal center (GC) B cell area and T cell areas and generated CD138^+^ cells (plasmablasts), indicating that alum-induced iBALT structures function as tertiary lymphoid organ in the lung. I.t. alum instillation induced IL-1α released in the lung by alveolar macrophage (AM) cell death, and the number of iBALT formation was clearly reduced in IL-1R-deficient mice. Interestingly, IgE responses were also attenuated in IL-1R-deficient mice, coincident with decreased number of iBALT structure.

Our findings suggest that particulates induce unique immune responses in the lung through AM cell death and tertiary lymphoid organ formation, and that AM-IL-1α-iBALT axis may be a unique therapeutic target of particulate-induced allergic inflammation.

## A563 Association Between Smoking and Allergic Diseases in the Korean Adult General Population

### Sunmi Kim^1^, Gyeong-Hun Park^2^

#### ^1^Kangwon National University Hospital, South Korea; ^2^Dongtan Sacred Heart Hospital, Hallym University College of Medicine

##### **Correspondence:** Sunmi Kim – Kangwon National University Hospital, South Korea

**Background:** The present study sought to investigate the association between smoking and allergic diseases including atopic dermatitis, asthma, and allergic rhinitis in the Korean adult general population.

**Methods:** A cross-sectional study was performed using data from 33,943 subjects aged 19 years or more who participated in the fourth and fifth Korean National Health and Nutrition Examination Survey performed in 2007-2012, which represents the Korean general population. Multiple logistic regression analyses were conducted to estimate the odds ratios of each allergic condition according to the smoking status with adjustment for potential confounding factors including age, sex, region of residence, level of education, income, and alcohol consumption.

**Results:** After adjusting for potential confounders, neither atopic dermatitis nor asthma was associated with the smoking status (*p*=0.385 and 0.340, respectively). In contrast, compared to never-smokers, the odds of allergic rhinitis was significantly lower in current smokers (odds ratio [95% confidence interval] 0.76 [0.66-0.87], *p* < 0.001), and higher in ex-smokers (1.16 [1.02-1.32], *p* = 0.028) after adjusting for confounders.

**Conclusions:** The present results may suggest the complex relationship between smoking and allergic conditions.

## A564 Relationship of S100 Calcium Binding Protein A9 with Inflammasome Activation in Murine Asthma Model

### Hyun Ji Song^1^, Taehyeong Lee^2^, Ji Ah Jun^2^, Hun Soo Chang^2^, Jong-Sook Park^2^, Choon-Sik Park^2^

#### ^1^Soonchunhyang University, South Korea; ^2^Soonchunhyang University Bucheon Hospital

##### **Correspondence:** Hyun Ji Song – Soonchunhyang University, South Korea

**Backgrounds:** We previously reported elevation of S100A9 protein in sputum of neutrophilic severe uncontrolled asthmatics compared with stable asthmatics [Annals of allergy, asthma, immunology on 2013 Oct;111(4):268-275] suggesting possible role of S100A9 in neutrophilic severe asthma. IL-1beta(IL-1β) and IL-18, which are released by activated inflammasome, exert neutrophil - activating activities. The aim of this study was to evaluate the temporal relationship of S100A9 with inflammasome activation in neutrophilicmurine C57BL/6 mice model using CFA/OVA-sensitization/challenge.

**Methods:** Expression of S100A9, S100A8 mRNA and protein levels and activated caspase-1 were measured in the lung tissues of the CFA/OVA modelusing a RT-PCR, real-time PCR and western blot. Spatial expression of S100A9 protein and inflammasome - related proteins were visualized by immune fluorescent stain. To evaluate the relation of S100A9 on activation of inflammsome, temporal changes of neutrophil infiltration and activation of caspase-1 were analyzed after intra-tracheal administration of 10ug S100A9 protein.

**Results:** S100A9 and P20 - activated caspase-1 started to increase from day 14 and peaked at day 23,earlier than the number of total cells, macrophage and neutrophils significantly increased with concomitant increase of airway resistance at day 23. Neutrophil elastase, S100A9 and activated caspase-1 co-localized on peri-bronchially infiltrating cells and apical portion of bronchial epitheliumusing confocal microscopy. Intra-tracheal instillation of S100A9 protein induced rapid increase of activated caspase-1 in the lung tissue from 2 hr, peaked at 8 hr, then progressively decreased till 80 hr with concomitantincrease of neutrophilsin BAL fluids.

**Conclusions:** S100A9 may induces neutrophilic inflammation via direct activation of inflammsome in the airway.

## A565 Cluster Analysis of Asthma Phenotypes to Predict Exacerbation in Korean Population

### Mi-Ae Kim^4^, Seungwoo Shin^1^, Jong-Sook Park^1^, Hun Soo Chang^1^, You Sook Cho^2^, Hae-Sim Park^3^, Choon-Sik Park^1^

#### ^1^Soonchunhyang University Bucheon Hospital; ^2^Asan Medical Center, University of Ulsan College of Medicine; ^3^Ajou University School of Medicine; ^4^Bundang Medical Center, CHA University, South Korea

##### **Correspondence:** Mi-Ae Kim – Bundang Medical Center, CHA University, South Korea

**Background**

Asthma is a heterogeneous disease and has been classified into several phenotypes on the basis of allergic, inflammatory and clinical manifestations, such as atopic or non-atopic, eosinophilic or neutrophilic, and severe or non-severe subtypes. Cluster analysis is the task of grouping a set of objects in such a way that objects in the same group are more similar to each other than to those in other groups. Cluster analysis is applied to development of asthma sub-phenotypes and demonstrated the differences in clinical response to treatment, distinct phenotypes of severe asthma, clustering in extended populations including both asthma and chronic obstructive pulmonary disease, and clusters using additional parameters such as inflammatory biomarkers and obesity. In the present study, we investigated clusters reflecting the prognosis of asthma in terms of exacerbation over one or more years.

**Methods**

Clinical and demographic data on 1843 asthmatics registered in an asthma cohort in Korea were analyzed retrospectively. They were ethnic Koreans. Asthma was diagnosed by physicians on the basis of the Global Initiative for Asthma (GINA) guidelines. Among them, 632 subjects, who were regularly followed up for longer than 1 year, were included for the analysis after excluding current smokers and ex-smokers of 10 pack year or more. At the baseline visit, demographic information such as enrollment age, sex, BMI, asthma onset age, asthma duration, and smoking amount was collected. Uniform cluster analysis method was applied to each population using two-tiered approach, including hierarchical cluster analysis and K-mean cluster analysis.

**Results**

The subjects were classified into 4 major clusters using Ward’s method. Age, age of onset, duration of asthma, BMI, atopic status, FEV1%, and ΔFEV1 were robustly different among 4 clusters. Clinical characteristics of 4 clusters are as below: Cluster 1, early-onset with high frequency of atopy and relatively well preserved FEV1; Cluster 2, early-onset with long duration and moderately impaired FEV1; Cluster 3, middle age onset with short duration and severely impaired FEV1; Cluster 4, old age onset with high BMI, but well preserved FEV1. The annual average frequency of exacerbation was significantly different between the clusters (p=0.019), and markedly higher in the cluster 3 (0.46/year).

**Conclusion**

Exacerbation-prone asthma phenotype is found by using cluster analysis and clinical characteristics of the phenotype are middle-age onset, short duration, and severely impaired FEV1. Noticing this asthma phenotype can be useful in clinical practice to predict asthma exacerbation.

## A566 Effect of AG490 on the Expression of TH17 CELLS and Tregs in the MOUSE MODEL of Neutrophilic Asthma

### Zhang Min

#### The First Affiliated Hosital of Guangxi Medical University Pediatrics, China

**Objective** To investigate the expression of Th17 cells and Tregs in the mouse model of Neutrophilic Asthma effects of STAT3 inhibition AG490

**Methods** 36 female C57BL mice were equally randomly divided into three groups:NA group, NA treated with AG490(NAAG)group, and normal control(NC) group. The above mice were treated as fowllow: Mice in NA group or NAAG group were sensitized by a mixture consists 100ug of ovalbumin(OVA,Grade V) and 0.1ug of lipopolysaccharide(LPS) via airway delivery on the experiment days 0,6,13. Besides, NAAG mice were injected intraperitoneally with AG490 in the dose of 500ug three times a week. In the case of intervention of AG490 encountering sentisization with OVA and LPS, we injected the mice with AG490 30 minutes before they were sensitised through airway dilivery. Once the sensitization stage were finished, mice in NA group and NAAG group were challenged consecutively by exposure to a 1% OVA aerosol produced by an inhalation delivery system for one hour once a day, from day 21 to 22. At the time of 24 hours after the last challenge, all mice were disposed and relative index were measured in time. Bronchoalveolar lavage fluid(BALF) was collected and total white blood cell count was determined on blood cell counting plate, and classification of proportion was determined under the optical microscope with Diff-Quick staining. Lung tissue was separated and HE and PAS stain were done to obseve pathologic changes and goblet cell hyperplasia under the optical microscope. BALF IL-17A cytokine concentration was determined by means of enzyme-linked-immunosorbent serologic assay(ELISA). Percentages of Th17 cells and Treg cells in the lung tissue were determined by means of Flow cytometry(FCM).

**Results** BALF total cell count, neutrophil percentages and eosinophil percentages count in the NAAG group were lower than that of the NA group (P<0.05), but were still higher than that of the NC group(each P<0.05); Lung tissue pathologic changes and goblet cell hyperplasia were markedly improved in NAAG group compared with that of the NA group,but there were still significant change compared with that of the NC group;BALF IL-17 cytokine concentrations in the NAAG group was lower than that of the NA group, but was still higher than that of the NC group(each P<0.05);Th17 cell percentage in the lung of the NAAG group was lower than that of the NA group, but was still higher than that of the NC group(each P<0.05). Treg cell percentage in the lung of the NA group was higher than that of the NAAG group (each P<0.05).

**Conclusions** Airway inflammation in the mouse model of neutrophilic asthma was improved by early treatment of AG490,which probably via down-regulating the espression of Th17 cells and up-regulating the espression of Tregs.

## A567 Association Between the Clinical Characteristics and Disease Severity in Hospitalized Bronchiolitis Patients Younger Than Two Years Old

### Seo Hee Yoon, In Suk Sol, Young a Park, Yoon Hee Kim, Min Jung Kim, Kyung Won Kim, Myung Hyun Sohn, Kyu-Earn Kim

#### Yonsei University College of Medicine, South Korea

##### **Correspondence:** Seo Hee Yoon – Yonsei University College of Medicine, South Korea

**Background:** The purpose of this study was to characterize the clinical presentation of virus-induced wheezing in bronchiolitis patients younger than 2 years of age. We also aimed to verify whether the clinical index have a good association with the disease severity.

**Methods:** We retrospectively reviewed the medical records of hospitalized children younger than 2 years old with acute bronchiolitis, which caused by respiratory virus between April 1, 2012 and April 1, 2015 in Severance children’s hospital. Specific viral etiologies were detected in nasopharyngeal aspirates by multiplex reverse transcription polymerase chain reaction at the time of admission. Clinical severity score, based on the age, respiratory rate, wheezing, chest wall retraction, and percutaneous oxygen saturation at admission; duration of fever, use of oxygen therapy and inhaled corticosteroid within 24 hours after admission were investigated. According to the scores, all patients were divided into a mild to moderate and severe bronchiolitis group. Host factors and type of respiratory viruses, were compared among the severity groups. Multivariate logistic regression analyses were performed to verify the risk factors for severe bronchiolitis.

**Results:** A total of 780 children were studied. A single virus was identified in 530(68%) and multiple viruses in 250(32%). Respiratory syncytial virus (RSV), rhinovirus (RV), RSV+RV, influenza viruses (Flu), human metapneumovirus (hMPV) and parainfluenza virus were detected in 34, 27, 14, 12, 7 and 6 % of samples respectively. RSV and rhinovirus were the viruses most frequently identified in mixed infections. There were no differences in age and severity scores between patients with prevalent viruses (RSV and RV) and those with less common infections. Patients with coinfections were 2.25 times (95% confidence interval, 1.02 to 8.02) more at risk for severe bronchiolitis than those with a single viral infection. Host factors associated with severe bronchiolitis included younger age, prematurity and chronic cardiopulmonary diseases. Type of viruses, personal and family history of allergic diseases was not significantly associated with bronchiolitis severity.

**Conclusions**: We compared the clinical characteristics of respiratory viral infections in wheezy bronchiolitis patients younger than 2 years old. Viral coinfections and host factors, including younger age, prematurity, and chronic cardiopulmonary diseases are risk factors for severe bronchiolitis.

## A568 Comparison Between House Dust Mite and Aspergillosis Sensitization in Patients with High Level of Tige

### Wu Shiquan

#### The First Affiliated Hospital of Guangzhou Medical University, China

**Background:** Among the patients with serum total immunoglobulin E (TIgE) higher than 1000ku/L, who suffered from respiratory diseases, we could found the majority of them were sensitized to house dust mite (HDM) and Aspergillosis. We aimed to find out if there any correlation with TIgE among serum specific immunoglobulin- -E (sIgE) against house dust mite along with Aspergillosis in the patients.

**Methods:** 64 subjects with high level of TIgE were tested for serum sIgE-derp1 and sIgE-Af by using ImmunoCAP 100.

**Result:** 68.8% (44/64) patients were sensitive to HDM while 40.6% (29/64) were sensitive to aspergillosis. What’s more, 37.5% (24/64) patients could be detected sIgE (derp1,Af) while 15/64(23.4%) patients were found sIgE(derp1, Af)-free. 20/64 (31.3%) patients were merely sensitive to HDM while 5/64 (7.8%) were sensitive to Aspergillosis. Among the patients with TIgE higher than 1000ku/L, those patients sensitive to HDM was significantly more than those sensitive to Aspergillosis (ka=9.00, t= P<0.01). sIgE-Af (r=0.410) was correlated more with TIgE than sIgE-derp1 (r=0.228) was (P<0.05).

**Conclusion:** Most of the patients with high level of TIgE, complicated with respiratory diseases, were more likely sensitive to HDM rather than Aspergillosis. Compared to sIgE against HDM, sIgE-Af contributed more to the level of TIgE. The detection of sIgE against HDM and Aspergillosis was important for the patients complicated with respiratory and high level of TIgE.

## A569 The Prevalence of Metal Allergy in the Patients with Orthodontic Appliance

### Yongwon Lee^1^, Hana Bak^2^

#### ^1^Catholic Kwandong University International St. Mary’s Hospital, College of Medicine, South Korea; ^2^Cheongdam-Hana Dermatology

##### **Correspondence:** Yongwon Lee – Catholic Kwandong University International St. Mary’s Hospital, College of Medicine, South Korea

During the last decades, the prevalence of metal contact allergy has been high. Among Danish female patients as well as North American patients, the occurrence of metal sensitization has increased. The importance of metal exposure from fixed orthodontic appliance is under discussion as fixed orthodontic appliance contain metals including nickel, chromium, cobalt and mercury. As our knowledge, it would be the first report about relationship between metal allergy and orthodontics.

We investigated the association with prevalence of metals including nickel, chromium, cobalt, and mercury by patch test and orthodontic appliance. Additionally we investigated the coincidence of resin and colophonium sensitization which is adhesive materials of orthodontic appliance with the patients with orthodontic appliance. In total, 150 patients were included and performed patch test. The patients characteristics including orthodontic appliance history were collected with a questionnaire and clinical investigation.

The incidence of metal allergy was significantly high in the patients with experience of orthodontic appliance. Especially nickel and cobalt allergy were more significantly correlated with orthodontics history. In metal allergy, orthodontics could be an important role.

## A570 Component Resolved Diagnosis and Single Nucleotide Polymorphism Analyses: Towards the Development of Specific Immunotherapy for Allergy

### Maricar Wisco Ching^1^, John Donnie Ramos^2^

#### ^1^Centro Escolar University, Philippines; ^2^University of Santo Tomas

##### **Correspondence:** Maricar Wisco Ching – Centro Escolar University, Philippines

The atopic triad of allergic asthma, allergic rhinitis, and atopic dermatitis are reaching epidemic proportions. Epidemiological studies show that 20-40% of the world population is afflicted with allergies. In local setting, more than 10 million Filipinos are reported to suffer from allergic diseases. Allergies are multifactorial immunological disorders that involve genetic and environmental factors in its development, chronicity and severity.

The pathogenesis of allergic diseases involves complex interactions between well- characterized environmental allergens and poorly-understood genetic factors. In addition, the decline in the number of infectious stimuli during the development of the immune system may contribute to allergy pathogenesis, a paradigm called "Hygiene Hypothesis" that supports the steep rise of allergies in developed populations. In contrast to the effect of environmental allergens, pathogenic and non-pathogenic microoganisms or their structural components can exert pressure on the immune system to modulate the development of allergic responses. Furthermore, the effect of different polymorphisms may play important role in an individual’s predisposition to allergic diseases. In recent years, findings on the combined effect of environmental allergens, genetic polymorphisms, the absence of infections and other environmental factors are starting to converge, producing fascinating results on gene-environment interactions that may explain the development of allergies.

Understanding the biochemical nature of allergens and the identification of genetic polymorphisms implicated in allergies has advanced our knowledge on the disease prevalence, healthcare burden and pathogenesis of allergies. Elucidating the mechanisms of interactions between the genes and the allergens involved in the pathogenesis of allergic diseases will help enhance the present medical armamentarium for the diagnosis and therapy of allergies.

## A571 Clinical Features of Anaphylaxis Caused By Peanut, Tree Nuts and Seeds in Children and Adolescents: Multi-Center Study with 126 Patients

### Kyunguk Jeong^1^, Sooyoung Lee^1^, Kangmo Ahn^2^, Myung Hyun Sohn^3^, Kyung Won Kim^3^, So-Yeon Lee^4^, Tae Won Song^5^, Youhoon Jeon^6^, Jihyun Kim^2^, Taek Ki Min^7^, Kyu-Earn Kim^3^, Bok-Yang Pyun^7^, Hyeon-Jong Yang^7^, Hae Ran Lee^4^, Youngmin Ahn^8^, Ji-Won Kwon^9^, Dae Hyun Lim^10^, Jeong Hee Kim^10^, Dongin Suh^11^, Hyung Young Ki

#### ^1^Ajou University School of Medicine, South Korea; ^2^Samsung Medical Center, Sungkyunkwan University; ^3^Yonsei University College of Medicine; ^4^Hallym University Sacred Heart Hospital; ^5^Ilsan Paik Hospital, Inje University College of Medicine; ^6^Dongtan Sacred Heart Hospital, College of Medicine, Hallym University; ^7^Soonchunhyang University College of Medicine; ^8^Eulji University Hospital; ^9^Seoul National University Bundang Hospital; ^10^Inha University Hospital; ^11^Seoul National University Hospital

##### **Correspondence:** Kyunguk Jeong – Ajou University School of Medicine, South Korea

**Background**

Peanut (PN) and tree nuts (TNs) are well-recognized as major causes of anaphylaxis in Western countries but no information is available in Korea. The purpose of this study was to feature clinical characteristics of anaphylaxis caused by PN, TNs and seeds in Korean children and adolescents.

**Methods**

A retrospective medical record review was performed on patients (0~18 years old) diagnosed with anaphylaxis from PN, TNs (walnut, almond, hazelnut, pine nut, cashew nut, pistachio, pecan and macadamia) and seeds (sunflower, sesame and perilla) between 2009 and 2013 in fourteen university hospitals in Korea.

**Results**

One hundred and twenty-six cases of anaphylaxis from PN, TNs and seeds were identified (64.3% in male patients) and the mean age was 4.9 years (0.8-18.9 years), with 81.7% of subjects under seven years old. The number of patients increased from 9 cases in 2009 to 57 cases in 2013. PN accounted for 32.5%, walnut (WN) accounted for 41.3%, pine nut accounted for 7.1%, and other TNs and seeds accounted for 19.1%. The most common system involved was cutaneous (96.0%), followed by respiratory (87.3%) and gastrointestinal (26.2%). The proportion of patients with cardiovascular symptoms was significantly higher in older patients than in younger children (p = 0.001). The time intervals between ingestion of triggering food and onset of symptoms were immediate in 19.0% and less than two hours in 42.9%. Among 104 cases (82.5%) in which serum levels of specific IgE (sIgE) to corresponding allergens were measured, the median values of sIgE to PN, WN and pine nut were 10.50 (0.39-100.00) kU_A_/L, 8.74 (0.04-100.00) kU_A_/L and 4.61 (0.60-4.61) kU_A_/L, respectively. Among 50 cases managed in the emergency department, patients were treated with intramuscular epinephrine in 52.0%, with systemic steroid in 66.0%, with antihistamine in 94.0%, with oxygen in 36.0%, and with bronchodilator in 48.0%. The overall percentage of patients prescribed an epinephrine auto-injector was 48.4%, with no significant differences between age groups.

**Conclusions**

Among anaphylaxis caused by PN, TNs and seeds in Korea, WN, PN and pine nut were the 3 most common triggers in order, and 9 other kinds of TNs and seeds were also identified as triggers. About 82% of cases were in children under the age of seven. The median levels of sIgE to PN and WN were 10.50 kU_A_/L and 8.74 kU_A_/L, respectively, and some of cases showed remarkably low sIgE levels, and < 0.35 kU_A_/L in three subjects.

* This study was done by Food Allergy and Atopic Dermatitis Study Group in the Korean Academy of Pediatric Allergy and Respiratory Diseases.

## A572 A Report of Two Cases of Anaphylaxis Caused By Perilla Seed in Children

### Kyunguk Jeong, Byeong Sub Park, Sooyoung Lee, Se-Ah Jeon, Kyu Jung Park

#### Ajou University School of Medicine, South Korea

##### **Correspondence:** Kyunguk Jeong – Ajou University School of Medicine South Korea

Perilla (*Perilla frutescens*) seed, also known as wild sesame seed is one of the popular spices in Asia but serious allergic reactions to perilla seed have been rarely reported. Herein we report two cases of anaphylaxis caused by perilla seed in children. A 25-month-old boy presented to the outpatient clinic at Ajou University Hospital with previous experiences of urticaria and facial angioedema immediately after ingestion of soup containing perilla seed powder at ages of 13 months and 21 months. He had past history of asthma and previous experience of lip angioedema after ingesting kiwi for the first time at 9 months old. As the specific IgE (sIgE) test to perilla seed is not commercially available, the sIgE to sesame seed, which belongs to *Lamiales* like perilla, was measured instead. The sIgE level to sesame seed was 2.98 kU_A_/L at 25 months, and was 3.37 kU_A_/L at 4 years of age. The sIgE level to kiwi was 1.42 kU_A_/L and multiple allergosorbent test chemiluminescent assay (MAST CLA) revealed no remarkable sensitization to inhalant allergens. To confirm the causal relationship, an open oral food challenge (OFC) with perilla seed powder was performed when he was 4 years old. Immediately after the contact of extremely small amount of perilla seed powder around his lips, he developed urticaria, facial angioedema, cough and rhinorrhea. He was treated with intramuscular epinephrine, after which his symptoms resolved completely. The second patient, a 5-year-old boy, presented to the outpatient clinic with a previous history of urticaria, lip angioedema, pruritis of tongue and hoarseness immediately after ingestion of seaweed soup containing perilla seed a few months before. He had past history of asthma, allergic rhinitis and atopic dermatitis and was previously diagnosed as allergic to egg white and peanut. He also had oral allergy syndrome to kiwi fruit. Moderate sensitization to alder, birch and white oak was noted in MAST CLA and sIgE levels to sesame seed, egg white, peanut and kiwi were 1.01 kU_A_/L, 1.47 kU_A_/L, 2.82 kU_A_/L and 3.19 kU_A_/L, respectively. The OFC test with perilla seed was not performed in this patient because his initial symptoms were compatible with anaphylaxis. For the etiologic confirmation, Enzyme-Linked ImmunoSorbent Assay (ELISA) and IgE western blot with crude extract of perilla seed produced in our own laboratory are in progress for both patients. To the best of our knowledge, this is the first report of anaphylaxis caused by perilla seed in children.

## A573 Prenatal Fine Particulate Matter Affects Wheezing in Children with TLR4 Polymorphism: Cocoa Study

### Song-I Yang^8^, Eun Lee^1^, Hyun-Ju Cho^1^, Young-Ho Kim, Mi-Jin Kang^2^, Yean Jung Choi^3^, Kil Yong Choi^2^, Youn Ho Shin, Kangmo Ahn^5^, Kyung Won Kim^6^, Byoung-Ju Kim^7^, So-Yeon Lee^8^, Eun-Jin K

#### ^1^Department of Pediatrics, Childhood Asthma Atopy Center, Environmental Health Center, Asan Medical Center, University of Ulsan College of Medicine, South Korea; ^2^Asan Institute for Life Science, University of Ulsan College of Medicine, South Korea; ^3^Asan Medical Center, South Korea; ^4^Gangnam CHA Hospital, South Korea; ^5^Samsung Medical Center, South Korea; ^6^Department of Pediatrics, Yonsei University College of Medicine, Seoul, South Korea; ^7^University of Cincinnati College of Medicine, USA; ^8^Department of Pediatrics, Hallym Sacred Heart Hospital, Hallym University College of Medicine, South Korea

##### **Correspondence:** Song-I Yang – Department of Pediatrics, Hallym Sacred Heart Hospital, Hallym University College of Medicine, South Korea

**Background:** Particulate matter (PM) associated with more wheezing episodes, increased risk of asthma symptoms and respiratory tract infection in children. However, the impact of prenatal exposure to indoor PM on the health of children is poorly understood yet. Toll-like receptor 4 (TLR4) plays a critical role in responses induced by air pollution.

**Objective:** To investigate whether prenatal exposure to indoor fine particulate matter (PM_2.5_) affects susceptibility to wheezing in children, and to determine whether genetic factor modify this environmental effect.

**Methods:** The study population consisted of the 323 children with indoor PM_2.5_ data in a birth cohort. Recurrent wheezing was determined as 2 or more wheezing episodes diagnosed by physicians in the first 2 years of age. Indoor PM_2.5_ was measured during pregnancy. Genotyping for *TLR4* (rs1927911) was performed by TaqMan.

**Results:** Prenatal indoor PM_2.5_ exposure increased the risk of recurrent wheezing in 2 years of age (aOR 3.52; 95% CI 1.50-8.30). *TLR4* CC increased the effect of prenatal indoor PM_2.5_ exposure on recurrent wheezing (aOR 7.00; 95% CI 1.41-34.73; p for interaction 0.153).

**Conclusion:** Indoor PM_2.5_ exposure during the prenatal period increased susceptibility to recurrent wheezing. This effect was modified by polymorphisms in *TLR4*. Reducing PM_2.5_ exposure from the prenatal period may prevent wheezing in susceptible children.

## A574 Intensified B Lymphocyte Depletion (IBLD) without Immunosuppressive Maintenance Treatment As a Rescue Therapy in Refractory Lupus Nephritis (LN): a 4-Year Observation

### Roccatello Dario

#### Cmid, G.Bosco Hospital Italy

**Background**

B-lymphocytes (BL) play a critical role in Systemic Lupus Erythematosus (SLE). BL depletion therapy still remains an attractive option, despite the disappointing results of RCTs.

**Methods**

Twelve SLE patients [2 males, mean age 43.8 yrs (29-54)] with polyarthralgia and multiorgan involvement including class IV or III/V (ISN/RPS) glomerulonephritis (9 cases), skin lesions (9 cases, with necrotizing ulcers in3), polyneuropathy (7cases, with CNS involvement in 2), lymphoadenopathy (6) e polysierositis (5) have been treated withan IBLD protocol for intolerance to conventional immunosuppressive therapy (6 cases) or as a front line therapy (6 cases). Protocol: Rituximab 375 mg/sm on days 1, 8, 15, 22, and 2 more doses after one and two months, associated with 2 IV administrations of 10 mg/kg of cyclofosfamide, and 3 infusions of methylprednisolone (15 mg/kg) followed by oral prednisone (0.8 mg/die, rapidly tapered to 5 mg/day in 10 weeks).

No further immunosuppressive maintenance therapy has been given.

**Results**

IBLD obtained a complete depletion of CD20+ BL for 12-18 months. Patients had been followed-up for 48.9 (25-93) months. A significant decreases (p<0.05) were found in the levels of ESR (baseline mean value: 54.2 mm; 3 months: 33; end of follow-up: 14.9), anti-dsDNA antibodies (baseline: 192 U; 3 months: 112; end of follow-up: 17) and proteinuria (baseline: 4.9 g/24 hours; 3 months: 0.97; end of follow-up: 0.22). Conversely, C4 values (baseline 11 mg/dl) significantly increased (p<0.05) after 3 months (22 mg/dl) and at the end of the follow-up (20 mg/dl). Three patients relapsed after 36, 41 and 72 months, respectively. They showed again a complete remission after retreatment over 13-48 months of observation.

**Conclusions**

These data confirm the opportunity to reconsider the regimens of BL depletion in the treatment of the most severe forms of SLE despite the disappointing results of RCTs. A promising role of Rituximab in protocols of “intensified induction therapy” in selected patients for whom avoiding immunosuppressive maintenance therapy is particularly appealing can be envisaged.

## A575 Relationship Between Th17 Cells and Neutrophilic Airway Inflammation in Childhood Neutrophilic Asthma

### Jing Liao

#### The First Affiliated Hospital of Guangxi Medical University China

**Objective:** To explore the role of Th17 cells in neutrophilic airway inflammation in childhood neutrophilic asthma.

**Methods:** Twenty-eight children with exacerbated asthmatics without using any corticosteroids were divided into three groups: eosinophilic asthma group (EA group) (n = 12), neutrophilic asthma group (NA group) (n = 10) and paucigranulocytic asthma (PGA group) (n = 6) according to induced sputum cytology.Ten healthy children were recruited as healthy control group (HC group) (n =10). Th17, Th2 cells in peripheral blood and Ki-67, STAT5, BCL-2 expressed in Th17 cells were detected by flow cytometry. Expression of RORγt-mRNA in peripheral blood mononuclear cell (PBMC)was detected by real-time quantitative polymerase chain reaction .Concentrations of IL-17, IL-8 and IL-5 in induced sputum supernatant, as well as concentrations of IL-17 in plasma and in the culture supernatant from PMA-stimulated PBMC were measured by enzyme linked immunosorbent assay.

**Results:** The percentage of Th17, Ki-67+Th17, STAT5+Th17, BCL-2+Th17 cells in peripheral blood and expression level of RORγt-mRNA in PBMC were significantly higher in NA group than in EA group, PGA group and HC group (both P<0.01). The level of IL-17 and IL-8 in sputum and level of IL-17 in culture supernatant from PMA-stimulated PBMC were significantly higher in NA group than in EA group, PGA group and HC group (both P<0.01). The percentage of Th2 cells in peripheral blood and level of IL-5 in sputum supernatant were significantly higher in EA group than in NA group, PGA group and HC group (both P<0.01). The percentage of Th17 cells in peripheral blood, the levels of IL-17 in sputum and PBMC culture supernatant, and the level of IL-8 in sputum supernatant were all correlated positively with the percentage of neutrophils in sputum.

**Conclusions** Both Th17 cells and Th2 cells are involved in the pathogenesis of asthma in children. Th17 cells and IL-17 may mediate neutrophilic airway inflammation in asthma, indicating an important role for Th17 cells in childhood neutrophilic asthma. Th17 cells may be maintain their survival through activation of the JAK/STAT5 signaling pathway.

## A576 Clinical Applications of Impulse Oscillometry in Asthma Management after Exacerbation in Preschool Children

### Yong Feng, Yunxiao Shang

#### Shengjing Hospital of China Medical University, China

##### **Correspondence:** Yong Feng – Shengjing Hospital of China Medical University, China

**Background**

Determination of the values of specific physiologic tests has not been well studied in long-term asthma management in preschool children. We sought to determine the utility of impulse oscillometry in a long-term management in preschool children after asthma exacerbation.

**Methods**

40 outpatients, aged 3 to 5 years old, with mild-to-moderate asthma exacerbation from Shengjing Hospital of China Medical University were enrolled. The impulse oscillometry was performed immediately after enrollment (T_0_). And then during 24 weeks of therapy with inhaled corticosteroid, which were adjusted according to GINA report, impulse oscillometry was performed at 4 (T_1_), 12 (T_2_) and 24 (T_3_) weeks separately for every children. The differences of resistance at 5Hz (R5), resistance at 20Hz (R20), R5-R20, resonant frequency (Fres) and low frequency integrated reactance from 5Hz to Fres (AX), among four visits were measured by repeated-measures analysis.

**Results**

For the 40 children, 26 were boys, the average age was 3.68±0.58 years old, the weight was 17.74±3.17 kg and the height was 103.95±6.49 cm. R5 was 1.27±0.33, 1.12±0.26, 1.01±0.26 and 0.89±0.24 kPa/L/s at T_0_, T_1_, T_2_ and T_3_ separately and the differences were significant when compared in pairs. R20 was 0.77±0.19, 0.67±0.16, 0.66±0.16 and 0.59±0.15 kPa/L/s separately, and the differences were significant except between T_1_ and T_2_. R5-R20 was 0.50±0.24, 0.45±0.18, 0.35±0.19 and 0.30±0.20 kPa/L/s separately, and the differences were significant except between T_0_ and T_1_. The Fres was 25.38±6.91, 22.70±3.19, 21.41±2.40 and 20.13±2.69 Hz separately, and the differences were significant. The AX was 4.29±1.91, 3.23±1.33, 2.48±1.28 and 1.81±0.90 kPa/L separately, and the differences were significant.

**Conclusions**

In preschool children, with the management of asthma after exacerbation, lung function assessed by impulse oscillometry improved in different degrees. R5, Fres and AX may reflect the ongoing improvements. Assessment of respiratory mechanics over time with oscillometry might offer useful insights into the response of asthmatic preschool children to therapy. Further studies should focus on longer term of management and the relationship between impulse oscillometry and airway inflammations.

## A577 Contact Allergy to Sodium Sulfite and Its Relationship to Facial Cosmetic Contact Dermatitis

### Yongwon Lee^1^, Hana Bak^2^

#### ^1^Catholic Kwandong University International St. Mary’s Hospital, College of Medicine, South Korea; ^2^Cheongdam-Hana Dermatology

##### **Correspondence:** Yongwon Lee – Catholic Kwandong University International St. Mary’s Hospital, College of Medicine, South Korea

Sulfites are in widespread use as preservatives or antioxidants even in cosmetic products. It was published that sodium metabisulfite could be a marker for sulfite allergy in cometics. However contact allergy to sodium sulfite is less well recognized. The relationship with sodium sulfite and cosmetic contact dermatitis is rarely known.

We sought to establish the prevalence of positive patch test reactions to sodium sulfite in our patient population with facial cosmetic contact dermatitis. 130 patients with facial contact dermatitis were included and performed Korean standard patch tests, some of cosmetic patch tests and sodium sulfite patch test.

In result, positive allergic reactions occurred to sodium sulfite in 3.5% of the tested patients. Clinically the patients with sodium sulfite positive reaction showed aggravation of facial symptoms after using cosmetic products including sulfites. Interestingly these patients experienced aggravation after eating sulfite containing food including dried fruit, fried potato and canned food, etc.

## A578 Effect of Exposure to Air Pollution on Asthma and Lung Function Development

### Hyung Young Kim^10^, Byoung-Ju Kim^1^, Ji-Won Kwon^2^, Ju-Hee Seo^3^, Eun Lee^4^, So-Yeon Lee^5^, Song-I Yang^6^, Young-Ho Jung^7^, Hyo-Bin Kim^8^, Ho-Jang Kwon^9^, Hee Ju Park^10^

#### ^1^University of Cincinnati College of Medicine, USA; ^2^Seoul National University Bundang Hospital, South Korea; ^3^Department of Pediatrics, Korea Cancer Center Hospital, South Korea; ^4^Asan Medical Center, South Korea; ^5^Department of Pediatrics, Hallym Sacred Heart Hospital, Hallym University College of Medicine, South Korea; ^6^Hallym University Sacred Heart Hospital, South Korea; ^7^Bundang CHA Medical Center, CHA University School of Medicine, South Korea; ^8^Inje University Sanggye Paik Hospital, South Korea; ^9^Department of Preventive Medicine, College of Medicine, Dankook University, South Korea; ^10^Pusan National University Children’s Hospital, South Korea

##### **Correspondence:** Hyung Young Kim – Pusan National University Children’s Hospital, South Korea

**Background:** Children are susceptible to air pollution, which is known to be related to reduced lung function and the development of asthma.

**Objective:** This study evaluated long-term effects of air pollution on the lung development and the incidence of asthma in children.

**Methods:** A total of 4791 children in elementary school selected from rural areas, industrial areas, and metropolitan cities were included in a baseline survey when they were first or second year students. Individual exposure to air pollution was estimated by using a geometric information system with the 5-year mean concentration of air pollutants.

**Results:** Higher exposure to carbon monoxide, nitrogen dioxide, and particles measuring 10 μ or less (PM_10_) was associated with newly diagnosed asthma during a 4-year period (odds ratio [OR] = 2.00, 95% CI [confidence interval] = 1.19-3.26; OR = 1.80, 95% CI = 1.10-2.95; OR = 2.04, 95% CI = 1.23-3.38, respectively). In children with higher exposure to ozone and sulfur dioxide, lung function measured as FEV1 and FVC was significantly decreased.

**Conclusion:** Exposure to air pollutants was significantly associated with the development of asthma and negative effects on lung-function growth in children.

## A579 Role and Relational Mechanism of AG490 in Airwayinflammation in the Mouse Model of Neutrophilic Asthma

### Zhang Min^3^, Nong Guang-Min^1^, Jiang Min^2^

#### ^1^Department of Pediatrics, the First Affiliated Hospital of Guangxi Medical University, Nanning, China; ^2^The First Affiliated Hospital of Guangxi Medical University; ^3^The First Affiliated Hosital of Guangxi Medical University Pediatrics, China

##### **Correspondence:** Zhang Min – The First Affiliated Hosital of Guangxi Medical University Pediatrics, China

**Objective:** Tyrphostin AG490 is a Janus kinase (JAK3) inhibitor that is effective in various models of inflammatory and autoimmune diseases. In this study, we examined the effects of AG490 on the treatment of the mouse model of Neutrophilic Asthma(NA).

**Methods:** 32 female C57BL mice were divided into NA group, NA treated with AG490(NAA)group and normal control(NC) group by means of a random number table. NA and NAA mice were sensitized by low-dose lipopolysaccharide(LPS) and ovalbumin(OVA) airway delivery on days 0, 6 and 13. During the challenge stage, the NA and NAA mice were exposed to a 1% OVA aerosol for 60 minutes from day 21 to day 27, which was generated using an inhalation delivery system. Before the aerosol, NAA mice were treated with 500ug AG490 through the peritoneum on days 21, 23, 25 and 27. The data were collected on the day 28. Bronchoalveolar lavage fluid (BALF) was collected and white blood cell counts was operated. Lung tissue stainning with HE, concentration of IL-17 in BALF by ELISA, expression of Bcl-2 and Caspase-3 on Th17 cells by flow cytometry were determined.

**Results:** The total cell, neutrophil percentages, and eosinophil percentages count in BALF of the NAA groups was lower than that of the NA group (*P*<0.05); Lung tissue pathologic changes were improved in NAA group compared with that of the NA group; BALF IL-17 cytokine concentrations in the NAA group was lower than that of the NA group, but was still higher than that of the NC group(*P*<0.05); Th17 cell percentage in the lung of the NAA group was lower than that of the NA group, but was still higher than that of the NC group(each *P*<0.05); The Th17^+^Bcl-2^+^expression levels in the NAA group were decrease compared with the NA group(P<0.05); The Th17+Caspase-3^+^ expression levels in the NAA group were higer compared with the NA group(P<0.05);

**Conclusions:** Airway inflammation in the mouse model of neutrophilic asthma was improved by treatment of AG490, which probably via down-regulating the espression of Th17 cells and BALF IL-17 cytokine concentrations; In the mouse model of neutrophilic asthma, AG490 can promote the apoptosis of Th17 cells, connected to dominant of JAK-STAT5 signal pathway and Bcl-2, Caspase-3.

## A580 Incidence of Adverse Reaction to Radioconstrast Media in a Single Tertiary Hospital

### Gyu Young Hur^1^, Eun Jung Sim^1^, Sora Yoon^1^, Juwhan Choi^2^, Junga Kim^2^, Jae Keom Sim^2^, Jee Youn Oh^2^

#### ^1^Regional Pharmacovigilance Center, Korea University Guro Hospital; ^2^Korea University College of Medicine, South Korea

##### **Correspondence:** Gyu Young Hur – Korea University College of Medicine, South Korea

**Background:** Adverse reactions to radiocontrast media (RCM) are common cause of adverse drug reactions. Spontaneous reporting of adverse reactions is the simplest and useful method for monitoring the safety issues of RCM. In this study, we purposed to investigate the incidence adverse reactions to RCM in a single tertiary hospital.

**Methods:** Individual case safety reports by RCM submitted spontaneously to our regional pharmacovigilance center from March 2014 to February 2015. We analysed all cases of adverse reactions after intravenous injection of iodinated contrast media (ICM; iohexol, iomeprol, ioversol, iopamidol, iopamide, and iobitridol) and gadolinum contrast (ganotenate, gadobutrol, gadobenate, and gadoxetate).

**Results:** Of total 293 adverse reactions of RCM, 278 (94.9%) were developed after ICM injection, and 15 (5.1%) was caused by gadolinum contrast. Overvall incidence of ICM adverse reactions was 1.28%. Mean age of case subjects was 54.15 ± 14.80, and 139 (46.5%) were men. There was no subject who had positive response to pre-test skin test to predict adverse reaction before imaging. Among ICMs, iobitridol showed highest rate of all adverse reactions (2.60%). Most common adverse reactions were cutaneous reactions (236, 84.9%), and gastrointestinal reactions (29, 10.4%). Classified into severity, mild reactions were 202 (72.7%), moderate and severe reactions were 65 (23.4%) and 11 (4.0%), respectively. Incidence of severe reactions was iobitridol (0.14%), iopromide (0.054%), and ioversol (0.027%). Regarding adverse reactions by gadolinium contrast, all reactions were mild (11, 73.3%), and moderate (4, 26.7%). There was no severe reaction caused by gadolinium contrast.

**Conclusions:** RCM skin testing for screening shows no efficacy to predict adverse reactions. The incidence of adverse reactions was relatively higher in ICM than gadolinium contrast. Among ICMs, iobitridol may develop adverse reactions more frequently, and more serious ones.

## A581 Cow’s Milk Oral Food Challenge: Clinical and Laboratory Features in Korean Children

### Kyunguk Jeong, Byeong Sub Park, Jeong-Min Lee, Sooyoung Lee, Eunjae Cheon, Youngjoo Na, Kyu Jung Park, Eunjoo Lee

#### Ajou University School of Medicine, South Korea

##### **Correspondence:** Kyunguk Jeong – Ajou University School of Medicine, South Korea

**Background**

Oral food challenge (OFC) is the gold standard for the diagnosis of food allergy, but in many cases, we would reduce the OFC if we can predict the outcome of OFC. The purpose of this study was to examine the relationship between clinical characteristics and the outcome of cow’s milk (CM) OFC.

**Method**

Medical records of 44 children who underwent CM OFC, from January 2013 to March 2015 at Ajou University Hospital, were reviewed. Data were analyzed to determine the relationship between OFC outcome and clinical parameters, including specific IgE (sIgE) levels to milk and casein measured before OFC.

**Results**

Twenty-nine of 44 patients (65.9%) were male, and the mean age was 3.3 years (6-177.6 months) with 95.5% aged 12 months or over. Thirteen had no history of CM ingestion and 31 had previous history of immediate reactions after CM ingestion (OFC performed for detection of clinical tolerance). Among them, 64.5% reported acute urticaria and 32.3% experienced anaphylaxis at initial CM ingestion. The number of subjects who passed OFC (successfully ingested cumulative dose of 200 mL of CM without allergic reactions, Group P) was 30 out of 44 (68.2%). Among the 14 children who failed OFC (Group F), cutaneous symptoms were reported in 100%, while upper respiratory, lower respiratory and gastrointestinal symptoms were noted in 35.7%, 7.1% and 14.3%, respectively. Seven patients experienced anaphylaxis (three of them with anaphylaxis history on initial CM ingestion) but none of them had cardiovascular or nervous symptoms during OFC. The median cumulative dose of CM at symptom onset during OFC was 22.0 mL. The median level of milk-sIgE in Group F was higher than that in Group P (5.04 kU_A_/L vs 1.42 kU_A_/L, p = 0.003), and the median level of casein-sIgE in Group F was also higher than that in Group P (7.56 kU_A_/L vs 0.53 kU_A_/L, p=0.003). Using receiver operating characteristic curves, the optimal cutoff points for milk-sIgE and casein-sIgE in children aged 12 months or over, were 1.71 kU_A_/L and 7.00 kU_A_/L, respectively. The number of subjects younger than 12 months was only two, so the optimal cutoff points in this group were not obtained. Initial levels of milk-sIgE and casein-sIgE, age at OFC, and anaphylaxis at initial CM ingestion were not significantly related with the outcome of OFC. Past history of asthma was a significant factor associated with increased risk of OFC failure (p=0.045) in crude analysis, but the relation was not significant after adjustment with confounding factors.

**Conclusions**

The levels of sIgE to milk and casein were significantly higher in patients who failed CM OFC compared to those in cases who passed OFC. The cutaneous symptoms occurred in all patients who failed OFC, and 7 patients experienced anaphylaxis during OFC. The comorbidity of asthma was the only significant factor associated with increased risk of OFC failure in crude analysis.

## A582 Validation of the Red Maple Trials Allergen Challenge Theatre for Ragweed Pollen Challenge

### William Yang, Suzanne Kelly, Rob Perrins, Jimmy Yang

#### Red Maple Trials Inc., Canada

##### **Correspondence:** William Yang – Red Maple Trials Inc., Canada

**Background:** Spatial and temporal variability in seasonal pollen concentrations can confound the results of field studies for new allergy medications. Allergen exposure facilities such as the Red Maple Trials Allergen Challenge Theatre™ (ACT) provide stable, controlled pollen concentrations year round and offer an alternative to field studies. A full scale validation of the ACT for ragweed (*Ambrosia artemisiifolia*) pollen challenge was performed.

**Methods:** The ACT is a 4-zone facility holding up to 100 seats in a series of elevated rows. Pollen was injected into the air supply and blown into the facility through ducts located across the top of the front wall. Pollen concentrations in the ACT were measured using laser particle counters (LPC) and impact samplers (IS). For the technical validation, LPC were used to assess the long-term stability of pollen levels; IS were used to determine pollen uniformity at 15 locations in the room. For the clinical validation, thirty ragweed allergic subjects were exposed to ragweed for 4 hours on two separate days to assess the reproducibility of rhinitis symptoms. Pollen was monitored at 30 min intervals by 3 IS located in the patient seating area.

**Results:** Three-hour stability results were comparable for LPC (5107 ± 244 g/m^3^) and IS (4858 ± 414 g/m^3^). Uniformity testing gave an average ragweed concentration of 4,622 g/m^3^; with a front-to-back SD of ± 544 g/m^3^ and a side-to-side SD of ±333 g/m^3^.

For the subject challenges, the mean 4-hour pollen concentration was 3,929 g/m^3^ for Day 1 and 4,099 g/m^3^ for Day 2. Plateau (2-4 hour) nasal symptom scores were 6.28 ± 1.49 and 6.19 ± 2.14, p=0.74, respectively. Ocular symptom scores were 2.82 ± 1.4 and 2.93 ± 1.82, p=0.59, respectively.

**Conclusion:** The validation studies showed that ragweed pollen levels in the ACT could be maintained stable over long periods. Subjects responded to the allergen challenge with reproducible rhinitis symptoms on different days. Facilities such as the ACT can provide a suitable means to test new allergy medications.

## A583 Preliminary Evaluation of the Red Maple Trials Allergen Challenge Theatre for Grass Pollen

### William Yang, Suzanne Kelly, Rob Perrins, Jacob Karsh, Jimmy Yang

#### Red Maple Trials Inc., Canada

##### **Correspondence:** William Yang – Red Maple Trials Inc., Canada

**Rationale:** Allergen challenge chambers expose allergen-sensitive subjects to a predetermined concentration of allergen in a closed, controlled environment and provide a mechanism to induce clinical symptoms and measure the effect of medication.

**Methods:** The Red Maple Trials (RMT) Allergen Challenge Theatre (ACT) is a 4-zone facility holding up to 100 seats in a series of elevated rows. Grass pollen (*Phleum pratense*) was injected into the air supply and blown into the facility through ducts located across the top of the front wall. A technical validation was performed to determine stability and uniformity of pollen concentrations in the room. Pollen counts were measured by a laser particle counter (LPC) positioned 5 ft above floor level and by impact samplers (IS) set at face level. IS were installed in 5 sections of a T-shaped quadrant and measurements taken every 30 minutes for 180 minutes. To evaluate the clinical response to grass pollen exposure, subjects with a history of grass allergy and positive skin prick test underwent two 3-hour challenges to grass pollen. Nasal and ocular symptom scores were recorded at baseline and every 30 min during the challenge.

**Results:** For the technical validation, pollen counts measured by LPC were stable for 3 hours at 4,800 ± 500 g/m^3^. LPC pollen counts correlated well with IS counts over a range from 1,500 to 7,500 g/m^3^ (LPC =0.849IS -269.0, r^2^=0.85). Mean pollen counts in each of the 5 sections of the quadrant ranged from 2,821 ±303 to 5,726 ±249. For the clinical validation, 17/32 patients had at least one Total Nasal Symptom Score (TNSS) ≥5 at challenge 1. In these subjects, mean change from baseline TNSS was 4.35±2.32 for challenge 1 and 4.94±2.41 at challenge 2, p=0.48. Total ocular symptom scores (TOSS) were 1.29±1.31 and 2.29±2.17, p=0.029, respectively.

**Conclusion:** The Red Maple Trials ACT demonstrated the capacity to achieve and maintain a stable grass pollen concentration associated with the ability to induce nasal and ocular symptoms of appropriate intensity upon a three-hour allergen challenge.

## A584 The Association Between Tobacco and the Risk of Asthma in Urban and Rural Children in San Francisco, Argentina

### Hector Badellino^4^, Alvaro Teijeiro^1^, Mabel Cuello^1^, Marilyn Urrutia Pereira^2^, Gustavo Egues^3^

#### ^1^Pediatric Hospital, Argentina; ^2^Brazilian Sociaty, Brazil; ^3^Hospital Santa Caterina, Spain; ^4^Clínica Regional Del Este, Argentina

##### **Correspondence:** Hector Badellino – Clínica Regional Del Este, Argentina

**Background**

Exposure to parental smoking is associated with wheeze in early childhood. There are plenty of papers showing the relationship between parental smoking in urban settings but very few in rural settings.

**Aim**

To examine the association between maternal and paternal smoking and prevalence of asthma.

**Methods**

Following ISAAC Study procedures, parents of children aged 6-7 years, living in San Francisco, Argentina , and surrounding rural areas were asked about symptoms of asthma, maternal smoking in the child’s first year of life and current maternal and paternal smoking

**Results**

A total of 1315 urban and 572 rural children were studied

Maternal and paternal smoking was associated with an increased risk of asthma, although the magnitude of the ORs is higher in the rural setting

There was a strong interaction between maternal and paternal smoking.

**Conclusion**

This study has confirmed the importance of parental smoking, mainly in the rural setting.

## A585 The Prevalence of Allergic Rhinitis in University Students in Manisa

### Ayse Aktas

#### Celal Bayar University School of Medicine, Manisa, Turkey

***BACKGROUND AND OBJECTIVE***

Allergic rhinitis (AR) is the most common chronic and allergic disease in adults. Prevalence ranges from 11.7% to 21.2% in adults in Turkey. AR leads to substantial socioeconomic costs and loss in quality of life. It typically begins in childhood or adolescence. Hereditary factors and gender plays an important role in the development.

The prevalence of self-reported and physician-diagnosed AR exhibits significant variability across the seven geographical regions in Turkey.

The purpose of this study was to assess the prevalence of AR in the Turkish adult population.

***METHODS***

An epidemiological study was planned among the students of Celal Bayar University from various regions of Turkey to find the prevalence of allergic rhinitis and search for geographical differences in Turkey. A questionnaire was designed to collect data on sociodemographic features, symptoms of allergic rhinitis, and any prior diagnosis of allergic rhinitis made by a physician.

***RESULTS***

A questionnaire related with allergic rhinitis and asthma was asked to 1086 students (454 males, 632 females). The current prevalences of the seasonal and perennial rhinoconjunctivitis, urticaria and eczema, drug intolerances, pollen and pet animal hypersensitivities were 20.7%, 22.5%, 4.6%, 16.3% and 0.2% respectively. All of hypersensitivities reaction rates were distinctively more common in the females. Seasonal rhinitis and pollen allergy were more prevalent in west (22.1%) region than northern region (12.0%). No significant geographical difference was observed regarding the drug and pet hypersensitivities.

***CONCLUSIONS***

Our study has shown the importance of asthma and allergic diseases as a public health problem. Explanation of the observed geographical differences needs further studies.

## A586 Allergen Sensitization in Zimbabwean Children with Atopic Dermatitis

### Jin-Kyong Chun^1^, Hilda Angela Mujuru^1^, Elopy N Sibanda^2^

#### ^1^University of Zimbabwe, Zimbabwe; ^2^Asthma Allergy and Immune Dysfunction Clinic

##### **Correspondence:** Jin-Kyong Chun – University of Zimbabwe, Zimbabwe

**Background:** The identification of food allergens implicated in the manifestation of atopic dermatitis is central to the prevention of serious hypersensitivity reactions and avoiding unnecessary and costly elimination diets. We investigated the profile of allergens sensitized in Zimbabwean children presenting with atopic dermatitis.

**Methods:** Total 111 pediatric patients with atopic dermatitis attending an allergy clinic of Zimbabwe were evaluated with Euroimmun®immunoblotting assays from Jan. 2010 to Dec. 2014. The median age of subjects was 5 years of age (range: 0-16 years of age). Total of 14 allergens were tested in each patient.

**Results:** 56.8% (63/111) were sensitized to at least one food allergen. Potato-specific IgE was detected with highest frequency as 32/111 (28%). Two-thirds 64.5% (40/62), of the tested children under 7 years of age were food allergen sensitized. Approximately half, 47% (23/49) of children older than 7 years were sensitized. There was no statistically significant difference between the two groups (relative risk: 1.39, 95%CI: 0.96-1.99). In terms of egg allergy, there was a statistically significant difference between the under and over 7 years of age groups (relative risk: 1.89, 95%CI: 1.47-2.50, *P* < 0.001). Among 63 patients who showed positive result, 40 patients (63%) showed multiple sensitizations to more than 3 allergens. 89% of patients who have wheat allergy also showed sensitization against rice as well. There was no concordance between milk allergy and soy allergy. Peanut sensitization was found in 27% of enrolled patients, 2/3 of patients with peanut allergy showed cross-reactivity with hazelnut. The most serious adverse effect of peanut allergy in this population was the oral allergy syndrome. There was no serious anaphylactic reaction in patients with peanut or potato allergy.

**Conclusions:** Food allergen sensitization is common amongst children with atopic dermatitis. The highest sensitogens, potato and peanut were not associated with severe allergic reactions. Egg allergy is predominantly seen in children under 7 years of age.

## A587 Vitamin D Insufficiency in Asthmatic Patients

### Andreea Ioana Popescu^1^, Raluca Greblescu^2^

#### ^1^Pop De Basesti Medical Centre, Romania; ^2^Medas Clinic

##### **Correspondence:** Andreea Ioana Popescu – Pop De Basesti Medical Centre, Romania

**Background**

Vitamin D has important functions in the immune system. In recent years, several studies have reported an association between vitamin D levels, atopy, asthma and respiratory tract infections.

**Objective**

We aimed to investigate whether vitamin D insufficiency in asthmatic adults associates with atopy, poor asthma control, and more frequent respiratory tract infections, and whether these patients can benefit from vitamin D supplementation.

**Methods**

The study included 62 adult patients diagnosed with asthma. At the first visit, patients underwent pulmonary function testing, skin prick testing with panel of aeroallergens, and 25-Hydroxyvitamin D levels were measured. Asthma Control Test (ACT) questionnaires were administered and records were obtained regarding number of visits to a medical centre for respiratory tract infections for the previous year. Subsequently, patients with vitamin D deficiency received 2000 I.U. vitamin D3 daily for 6 months (from October to March). At the end of that period, all patients were reassessed.

**Results**

Seventeen patients (27%) were found to have vitamin D levels lower than 30 ng/ml, considered to be insufficient. These patients had poorer asthma control as indicated by ACT scores, and had a significantly higher rate of allergic sensitization, compared to the vitamin D sufficient asthmatics. Patients with vitamin D insufficiency had an average number of medical visits for respiratory infections the previous year of 3.94, while after vitamin D supplementation the average was 2.17. ACT scores increased significantly at the end of the 6 month period, while spirometric values did not change significantly.

**Conclusions**

We found that vitamin D insufficiency in patients with asthma correlates with allergic sensitization. Vitamin D supplementation in these patients resulted in better asthma control, possibly due to fewer episodes or respiratory tract infections.

## A588 The Prevalence of Hypersensitivity Reactions Against Drugs Among University Students

### Suheyla Rahman, Ayse Aktas

#### Celal Bayar University School of Medicine, Manisa, Turkey

##### **Correspondence:** Suheyla Rahman – Celal Bayar University School of Medicine, Manisa, Turkey, Turkey

***BACKGROUND AND OBJECTIVE:***

Drug hypersensitivity reactions (DHR) are a common public health problem. Recent studies have confirmed that the frequency of drug allergy is overestimated by both patients and physicians.

The aim of this study is to determine the prevalence and characteristics of DHR in university students. Data about the epidemiology of DHR in outpatient populations is relatively scarce. This study is designed to determine the prevalence of self- reported drug hypersensitivity and related factors among young adults.

**METHODS**

A structured questionnaire was administered to the university students.

**RESULTS**

A total of 1085 students (mean age:21.16+1.90 years, F/M:631/454) from all grades responded to the survey. The mean prevalence of self-reported drug hypersensitivity was 4.6% (49/1085). The most frequently involved drugs were antibiotics 28 (68.3%), followed by analgesisc (26.8%). The most common allergic reactions were rash 53.2%, and cardiovascular reactions 12.8%, respiratory reactions 4.3%, aforementioned two systems involvement were 22.3%, anaphylaxis 7.4%. Personal history of allergic diseases 11%, family history of drug hypersensitivity 22.4%. There was no differences in the prevalence of drug allergy in female patients compared with male patients. The mean age of first drug allergy reaction year was 13±7.2. Five hundred fifty five (57.4%) students had antibiotics and 641(65.7%) students had analgesics in case of need.

***CONCLUSIONS***

The mean prevalence of self-reported drug hypersensitivity was 4.6% in this study. Antibiotics and analgesics are the two drug families most frequently suspected. Among the self-reported drug hypersensitivity reactions, the most common manifestations are cutaneous symptoms. Drug provocation tests need to be included in diagnostic protocols in order to evaluate suspected DHR. This study showed that self-reported hypersensitivity reactions to drugs is highly prevalent and the education of both patients and physicians on the management of drug hypersensitivity seems to be necessary.

## A589 Sublingual Immunotherapy Among Problematic Patients, Suffering from Allergic Rhinitis

### Nataly Tataurshchikova

#### Peoples’ Friendship University of Russia, Russia

**Background**

One of the key strategic ways of allergic diseases treatment is the update of methodology and creation of new immunothropic drugs for sublingual immunotherapy or SLIT, that determine mucosal tolerance development process. Combination of allergic rhinitis and palindromic herpetic infection (HSV and/or ÑMV) is usually characterized by rapid mucosal immunity barrier function violation, mucosal barrier permeability change and inevitable progress of allergic disease. Taking in consideration high prevalence of herpetic infection spread among patients, suffering from allergic diseases, search of optimal SLIT treatment schemes is becoming significantly important.

Purpose of the current study is to evaluate SLIT effectiveness among immunocompromised patients, suffering from allergic rhinitis, using up-to-date tableted allergen extract (produced in Kazakhstan).

**Methods**

Within open prospective research effectiveness and tolerance of SLIT course, maintained using up-to-date tableted allergen extract, produced in Kazakhstan was analyzed among 30 patients aged 18 to 40, suffering from allergic rhinitis (3 to 10 years) and herpetic infection (HSV and/or ÑMV). Among SLIT effectiveness criteria: VAS – visual analog scale, SMS - symptom medication score, patients’ adherence to treatment continuation. Laboratory effectiveness evaluation was conducted using profile cytokine evaluation (immunoenzyme method, proinflammatory - α-TNF – tumor necrosis factor, IL-8, γ – INF, antiphlogistic - IL-4 of serum and local fractions. Material for study – blood serum, taken fasting from ulnar vein and nasal lavage.

**Results**

Assessment result - excellent and good effect was stated among 73% (21) patients. Among them, 20 patients (68,9%) noted the decrease in pharmaco-medicine need when contacting corresponding allergen., overall health status improvement - 21 patients (73%). None of the patients claimed main disease flow worsening.

Cytokine profile research after treatment showed IL-4 and ϒ – INF content level normalization both in local and serum fractions. Among 5 patients (16,6%) rapid increase of IL-8 è TNF – α in local fractions was stated before treatment, and it was the key reason to change SLIT mode and to add immunomodulating therapy. 27 patients (93%) agreed to continue treatment next year.

**Conclusions**

Full SLIT course was successfully implemented to all patients using modern tableted allergen extract (produced in Kazakhstan), despite violations in virus defense system and mucosal immunity system. Stated change in α-TNF and IL-8 content level among a group of patients allowed to change SLIT mode and complete the course successfully.

SLIT using modern tableted medicine among immunocompromised (problematic) patients, suffering from allergic diseases is a successful and high effective method and complies with all criteria of rational pharmacotherapy.

## A590 A Novel Biomarker for Wheezing and Atopy in Early Infancy

### Eishika Dissanayake^1^, Yuzaburo Inoue^1^, Naoki Shimojo^1^, Taiji Nakano^2^

#### ^1^Graduate School of Medicine, Chiba University, Japan; ^2^Chiba University

##### **Correspondence:** Eishika Dissanayake – Graduate School of Medicine, Chiba University, Japan

**Background**

MicroRNAs (miRNAs) have been implicated in the pathogenesis of many diseases including atopy and asthma. We investigated whether we could identify serum microRNAs in early childhood that could be used to predict the development of wheezing in children.

**Methods**

The study protocol was approved by the Committee on Human Research of Chiba University. Preliminary microarray analysis revealed the dysregulation of several miRNAs including hsa-miR-185-5p. The samples used in this study were from the birth cohort samples consisting of cord blood, 1-year and 2-year samples. The samples were categorized according to wheezing status and allergen-specific IgE levels at 2 years. Children with more than 3 episodes of wheezing during the preceding 12 months were considered "wheezers", while those without were "non-wheezers". MiRNAs were extracted from the serum of birth cohort samples using a commercial column-based kit. The hsa-miR-185-5p levels were quantified using real-time PCR. Ce_miR-39_1 was used as the spike-in-control and the difference in expression level of the hsa-miR-185-5p and Ce_miR-39_1 was used as the cycle threshold (ΔCt). The data was analyzed using non-parametric tests on GraphPad Prism version 6.0.

**Results**

The hsa-miR-185-5p levels were significantly elevated between the ages of 1 and 2 years in non-wheezers at the age of 2 years, while the levels were consistent in wheezers. This suggests that increase of hsa-miR-185-5p levels may be a negative indicator of recurrent wheezing and potentially asthma in later childhood. We did not see any correlation between allergen-specific igE levels and hsa-miR-185-5p.

**Conclusions**

Hsa-miR-185-5p may be a useful to identify potential wheezing during the first years of life. The target genes and the regulators of this miRNA are being verified.

## A591 Prognostic Factors for Atopic Dermatitis in Spontaneously Born Babies from Low Socioeconomic Background

### Conny Tanjung

#### Pantai Indah Kapuk Hospital, Indonesia

***Background*.** The incidence of atopic dermatitis has markedly increased in this recents recent years. Atopic dermatitis develops due to interacting genetic and environmental factors. There is a lack of information on predictors of atopic dermatitis in vaginally born babies in low-income populations.

***Objective.*** To describe risk markers of atopic dermatitis for the occurrence of atopic dermatitis in low-income population in Indonesia.

***Methods.*** Prospective survival analysis study was done in babies born vaginally in Kemayoran Community Health Centre in Central Jakarta over a 10 months period. Occurrence of atopic dermatitis until the age of 10 months was diagnosed based on Hanifin-Rajka criteria. Prognostic factors for the development of atopic dermatitis were analyzed by SPSS Statistic 20 using Kaplan-Meier test and Cox regression model with a level of significance < 0.05.

***Results.*** Some 400 healthy term infant were enrolled into this study. Male-to-female ratio was 1.04:1. The mean age at of the onset of atopic dermatitis was 5.4 months (ranging from 1 until 10 months). Family income varied from only 15 to 769 US$/ month with a mean income of 150 US$. Atopic dermatitis occurred in 53 (13.3%). Smoking, number of people in the house and number of siblings in the family were not associated with the occurrence of atopic dermatitis in bivariate analysis. Atopic family history, higher degree of mother’s education and not being exclusively breastfed predicted occurrence of atopic dermatitis. In multivariate analysis only atopic family history (HR 5.0, 95% CI 2.3-10.9) and lower mother’s education (HR 0.5, 95% CI 0.2;0.9) were identified as prognostic factors.

***Conclusion*.** Atopic family history and higher mother’s education are independent prognostic factors for the occurrence of atopic dermatitis in infants from low income population in Indonesia.

## A592 Higher IgE Antibody Levels Mediate Anti-Cancer Immunity in Transgenic KN1 Hyper-IgE Mice

### Erika Jensen-Jarolim^1,2^, Judit Fazekas^1,2^, Josef Singer^1,2^, Anna Lukschal^1,2^, Reinhard Horvat^3^, Gertrude Achatz-Straussberger^4^, Gernot Achatz^4^

#### ^1^Messerli Research Institute of the University of Veterinary Medicine Vienna, Medical University Vienna and University Vienna, Austria; ^2^Institute of Pathophysiology and Allergy Research, Center of Pathophysiology, Infectiology and Immunology, Medical University of Vienna, Austria; ^3^Medical University of Vienna; ^4^University of Salzburg

##### **Correspondence:** Erika Jensen-Jarolim – Messerli Research Institute of the University of Veterinary Medicine Vienna, Medical University Vienna and University Vienna, Austria

**Background**

The introduction of monoclonal antibodies into clinical oncology yielded survival benefits for cancer patients by selectively targeting tumor-associated antigens (TAAs). Current research aims at making these immunotherapies even more effective. One promising approach is to use antibody classes other than IgG such as IgE. This study thus investigates the potential of specific anti-HER2 (human epidermal growth factor receptor-2) IgE antibodies in a vaccination approach comparing wildtype (wt) with transgenic hyper- or hypo-IgE mice.

**Methods**

Three different mice strains were employed: wt BALB/c mice, KN1 mice displaying 4- to 6- fold elevated mean serum IgE levels (1), and DM1M2 transgenic mice that lack the transmembrane and cytoplasmic domains of membrane-bound IgE resulting in reduced serum IgE levels down to 5% of wt mice (2). Each strain was divided into groups 1-4 (n=8): Groups 1. were immunized against HER-2 prior to tumor grafting, 2. against irrelevant antigen BSA, 3. passively received the anti-HER-2 IgG mouse monoclonal antibody 4D5, 4. remained naïve. Then, all mice were grafted with D2F2E2 tumor cells overexpressing HER-2 and monitored for tumor growth and survival.

**Results**

When remaining naïve, all KN1 hyper-IgE mice showed significantly slower tumor growth and longer survival compared to the wt BALB/c controls and DM1M2 hypo-IgE mice. In all strains of mice passive immunotherapy with 4D5 rendered significant survival benefits compared to the untreated groups. Vaccination against HER-2 resulted in the overall longest survivals and lead to significant survival benefits compared to naïve as well as passively 4D5 antibody treated groups. Most importantly, all KN1 hyper-IgE mice showed a significant survival benefit independent of any of the treatments.

**Conclusions**

Active vaccination surmounts the effects of passive antibody therapy in the mouse model, but the presence of IgE tops the antigen-specific experiments. This study therefore suggests that IgE antibodies may play a significant role in innate cancer surveillance as well as during active anti-cancer immune responses.

**References**

*(1) Migration of antibody secreting cells towards CXCL12 depends on the isotype that forms the BCR. Achatz-Straussberger G et al. European Journal of Immunology 2008;38(11):3167-77.*

*(2) Effect of transmembrane and cytoplasmic domains of IgE on the IgE response. Achatz G et al. Science 1997; Apr 18;276(5311):409-11.*

## A593 Fructooligosaccharides Intake during Pregnancy and Lactation Increases Gut *Bifidobacterium* and IL-27 in Breast Milk

### Yuji Fujita^2^, Shuji Ikegami^1^, Yoshitaka Nakamura^1^, Yuzaburo Inoue^2^, Naoki Shimojo^2^, Yoichi Kohno^2^, Shuichi Suzuki^3^, Naoko Ozawa^2^, Takayuki Kubota^2^, Ken Nonaka^4^, Osamu Ohara^4^, Kentaro Masuda^5^

#### ^1^Meiji Co Limited; ^2^Chiba University, Japan; ^3^National Shimoshizu Hospital; ^4^Kazusa DNA Research Institute; ^5^Masuda Maternity Clinic

##### **Correspondence:** Yuji Fujita – Chiba University, Japan

**Background:** We previously reported that the consumption of fructooligosaccharides (FOS) by pregnant and lactating women increases the production of IL-27 in breast milk in a randomized, placebo-controlled, double-blind trial (Kubota, et al. 2013), but the underlying mechanism remains to be clarified.

**Methods:** Healthy pregnant woman were enrolled with written informed consent. Subjects (84 individuals) were randomly assigned to the FOS (Meioligo-P, obtained from Meiji Food Materia Company Limited) (FOS, *n* 41) or sucrose (placebo, *n*43) intake groups. Trial compound (4 g) was taken after breakfast and supper daily from 26 weeks of gestation to 1 month after delivery. Colostrum samples were collected during hospitalization after childbirth and concentration of IL-27 was measured by ELISA. Fecal samples at 26 and 36 weeks of gestation were collected at home or the hospital by the subjects and the gut microbiota were quantified by quantitative real-time PCR.

**Results:** After excluding subjects with insufficient test compound intake, 29 in placebo and 34 in FOS groups were analyzed. There were no differences of the characteristics between 2 groups. The concentrations of IL-27 in colostrum samples were significantly higher in the FOS group (range: <0.156–46.6 ng/ml; median: 2.4 ng/ml) than in the placebo group (range: <0.156–95.8 ng/ml; median: 0.2 ng/ml) (*P*<0.05). Fecal numbers of *Bifidobacterium* were significantly increased in the FOS group but not the in the placebo group and were significantly higher in the FOS group (range: 8.9–11.0 log_10_ cfu/g; median: 10.4 log_10_ cfu/g) than in the placebo group (range: 8.8–10.72 log_10_ cfu/g; median: 10.1 log_10_ cfu/g) after the intervention (*P*<0.05). The concentrations of IL-27 in colostrum showed weak correlation to the *Bifidobacterium*numbers of 36 weeks of gestation.

**Conclusions:** Our data suggest that mother’s intake of prebiotics increases IL-27 expression in breast milk by modulating gut microbiota.

## A594 Effect of Nintedanib on Asthma in Mouse Model

### Chin Kook Rhee^1^, Sook Young Lee^1^, Hwa Young Lee^1^, Hea Yon Lee^2^, Ji Young Kang^1^, Sei Won Kim^1^, Soon Seog Kwon^3^, Young Kyoon Kim^1^

#### ^1^Seoul St. Mary’s Hospital; ^2^St. Mary’s Hospital; ^3^Pucheon St.Mary’s Hospital

##### **Correspondence:** Chin Kook Rhee – Seoul St. Mary’s Hospital, South Korea

**Objectives:** Nintedanib (BIBF1120) is tyrosine kinase inhibitor of VEGFR1/2/3, FGFR1/2/3, PDGFRα/β. Recently, nintedanib showed beneficial effect on patients with IPF. However, little has been known regarding the effect of nintedanib on asthma. Since VEGF and PDGF are well known mediator in the pathogenesis of asthma, we aimed to evaluate effect of nintedanib on asthma in acute and chronic mouse model.

**Methods:** Female BALB/c mice, 8–10 weeks of age, were used. We developed a mouse model of both acute and chronic asthma in which ovalbumin (OVA)-sensitized mice were repeatedly exposed to intranasal OVA administration. Mice were treated with nintedanib during the OVA challenge.

**Results:** Compared with control mice, the mice exposed to OVA developed sustained eosinophilic airway inflammation and airway hyperresponsiveness (AHR). Administration of nintedanib significantly decreased total cell and eosinophilic count in bronchoalveolar lavage (BAL) fluid. Also the level of IL-4, 5, and 13 in BAL fluid was significantly lower in nintedanib group compared with control. In a histologic analysis, there were fewer inflammatory cell infiltration in nintedanib group. Nintedanib treatment significantly attenuated airway remodeling including fibrosis and smooth muscle thickening. The protein and gene expression level of VEGF, FGF, and PDGF were lower in nintedanib group. In vitro experiment showed that nintedanib significantly decreased fibroblast proliferation.

**Conclusion:** These results suggest that nintedanib administration can attenuate airway inflammation, AHR, and remodeling in mouse model of asthma. The beneficial effect of nintedanib was via blocking of VEGF, FGF, and PDGF pathway.

## A595 Delayed Contrast Media Hypersensitivity after Coronary Angiography

### Gun-Woo Kim^1^, Ju-Young Kim^1^, Sang-Heon Cho^1^, Sang-Heon Cho^2^, Hye-Ryun Kang^1^, Hye-Ryun Kang^2^, Hyo-Soo Kim^1^, Jung Gyu Han^1^, Jin Lee^1^, Ji Young Lee^1^, Ji Young Go^1^, So Jung Park^1^

#### ^1^Seoul National University Hospital, South Korea; ^2^Seoul National University Medical Research Center

##### **Correspondence:** Gun-Woo Kim – Seoul National University Hospital, South Korea

**Background:** Coronary angiography (CAG) is a standard method for diagnosing coronary artery disease. Iodinated contrast media (ICM) used in CAG can induce both immediate and delayed hypersensitivity reactions. However, studies on delayed hypersensitivity reactions to ICM are relatively few and the reported incidence varies greatly. Therefore, we aimed to investigate the incidence and clinical features of ICM-induced delayed hypersensitivity reactions following CAG.

**Methods:** We prospectively monitored ICM-induced delayed hypersensitivity in patients who underwent CAG from February 2015 to May 2015 at the Seoul National University Hospital. The ICM agents used were iodixanol and iopamidol. Symptoms were monitored from one hour to three weeks after the completion of CAG.

**Results:** A total of 265 patients received CAG during the study period (mean age 63.6 years, male 70.5%). Delayed hypersensitivity reactions occurred in 35 patients (13.2%). The most common manifestation was skin rash (88.5%), followed by chest discomfort and gastrointestinal symptoms. The majority (87%) of the skin reactions were mild (involving < 25% of the body surface), mostly occurring within 3 days of CAG (within 24 hrs: 40%, between 1-3 days: 42.8%; more than 3 days: 17.1%). Subgroup analysis by ICM agent used, gender, and age did not show any significant differences in the reaction incidence or clinical manifestations.

**Conclusions:** The incidence proportion of delayed hypersensitivity reactions to ICM was 13.2%, which was higher than previously reported. To our knowledge, this is the first study in Korea to investigate the incidence of ICM-induced delayed hypersensitivity following CAG.

## A596 Gene Expression Profiling in Patients with Chronic Idiopathic Urticaria Reveals Unique Gene Signature Distinct from Healthy Controls

### Julie Kim-Chang, Cassandra Love, Patricia Lugar

#### Duke University Medical Center, USA

##### **Correspondence:** Julie Kim-Chang – Duke University Medical Center, USA

**Background:** Chronic Idiopathic Urticaria (CIU) is a relatively common disease which accounts for up to 30% of office visits to allergists. Although designated idiopathic the pathophysiology and mechanism of disease is reported to result from immune activation due to variety of causes. CIU is a manifestation of immune activation with approximately 50% of CIU patients demonstrating autoimmunity as evidenced by presence of IgG antibody to the FceR1 receptor present on mast cells and basophils. Autoantibodies to thyroid tissue and many other cellular and cell surface receptors commonly coexist. Further elucidation is lacking at the genetic and molecular level. Ideally, a better understanding of the underlying biology could improve not only prognostic indicators, but also provide a background for generating predictor models of treatment responders.

**Methods:** We collected peripheral blood mononuclear cells (PBMC’s) from 21 patients with active CIU disease and 10 non-atopic healthy controls. RNA was extracted from PBMC’s and prepared for Affymetrix GeneChip® Human Gene U133 plus 2.0 Array hybridization. The raw data were normalized and the gene expression of CIU patients was compared to that of normal controls.

**Results:** We found 149 genes that predicted CIU from normal controls (FDR<5%). The genes in the predictor are differentially expressed (P<0.001) with a minimum 2-fold difference between CIU patients and controls. The predictor was 100% successful in distinguishing patients with CIU from controls, and tested using leave one out cross-validation. Additionally we found that the upregulated genes seen in CIU most strongly correlated with monocytes, identified as cells expressing CD14 cell surface marker (p<0.001, r >0.8). Gene set enrichment analysis of CIU patients further revealed activation of a specific arm of the MAP-Kinase pathway utilized by monocytes (p<0.001, r >0.8).

**Conclusions:** Gene expression profiling can reliably distinguish patients with CIU from healthy controls. Our study findings suggest potential underlying mechanisms of CIU. These findings may provide guidance in developing biomarkers that would help determine subsets of patients who would be more likely to respond to variety of treatment options available.

## A597 Failure to Recognize Lymphopenia in Newborn Leads to Undetectable Primary Immunodeficiency

### Endah Citraresmi

#### Harapan Kita Women & Children Hospital, Indonesia

**Background**

Primary immunodeficiency (PID) has not been well known in Indonesia. Most physicians considered that PID is difficult to diagnose because the laboratory equipment in Indonesia is not complete. However, using simple laboratory tests such as CBC, a lot of data can be retrieved and be used as a clue in diagnosing PID.

**Case**

A baby girl, 7 days old, was referred to neonatology unit from other hospital due to thrombocytopenia with mark increased in AST and ALT. She received IVIG for 3 days in previous hospital. Her laboratory tests showed the percentage of lymphocytes ranged between 35.6 % - 66 %. Retrospective calculation revealed total lymphocyte count ranged between 2760 – 3830/uL (normal value >3400/uL). Neutrophil count within normal limits. Levels of IgM and IgG antibodies to rubella increased, accompanied by increased levels of IgG antibodies to toxoplasma, CMV and HSV1. She received PRC and TC transfusions. She was diagnosed as having rubella infection, discharged in good condition after 24 days of treatment. She then had Hepatitis B and BCG vaccination in outpatient clinic.

At age 3 months, she experienced dyspnea, admitted to PICU for pneumonia that needed ventilator support. Her laboratory tests again showed lymphopenia. Further exploration found increased levels of IgM, with normal level of IgG, IgA and IgE, also decreased CD4 and C3 levels. HIV PCR was negative. IgM CMV was positive; she was treated with gancyclovir. She was died after 30 days of hospitalization.

**Discussion**

CBC test result does not automatically include the value of total lymphocytes; it included only the percentage of lymphocytes. Because the lymphocytes percentage were always within normal limits, the treating physicians were not concerned with the low value of total lymphocytes, reflecting the low levels of T and/or B lymphocytes. Unfortunately, the patient then underwent severe infection and further tests found low CD4 lymphocyte levels with increased levels of IgM. There should be an opportunity to explore PID, especially in this case the possibility of Severe Combined Immune Deficiency (SCID), in the first hospitalization during newborn period. If we were aware of PID, this patient could be treated and prevented for possible severe infections.

**Conclusions**

Simple tests such as CBC needs to be interpreted in more detailed of the presence of lymphopenia or neutropenia to be indicative of primary immunodeficiency.

## A598 The Concordance Between Lung Function Test and Indonesian Version of Childhood Asthma Control Test (CACT)

### Nastiti Kaswandani, Cynthia Utami, Mardjanis Said

#### Faculty of Medicine University of Indonesia, Indonesia

##### **Correspondence:** Nastiti Kaswandani – Faculty of Medicine University of Indonesia, Indonesia

**Background.** Childhood Asthma Control Test (CACT) is a questionnaire to measure the level of asthma control in children. CACT is commonly known easy and simple to be implemented. Indonesian version of CACT has not been widely used because it is not known its correlation to clinical parameters since physician should consider the linguistic barrier of using it.

**Aim.** To evaluate the concordance of Indonesian version of CACT with spirometry of children with asthma.

**Methods.** A cross-sectional study of children aged 4-11 years-old with asthma. The severe asthma children and those who have mental problems were excluded. The subjects were consecutively recruited to do the lung function test and peak flow meter twice a day for 2 weeks. All children had to fulfill the CACT and data was analyzed by Pearson/Spearman test to see its concordance to spirometry.

**Results.** We included 66 subjects aged 7.89 years-old (5.25-11.83). The majority of subjects are boys 62.1%, most of all have good nutritional status and their parents are highly educated. Majority of subjects 60.4% showed uncontrolled asthma (CACT ≤19). Mean of FEV1 was 80% (SD 16.74) and mean of variability was 10.32% (SD4.93). There was no significant correlation between Indonesian version of CACT with FEV1(r =-0,024; p=0,846) and weekly variability (r=-0,218; p=0,079).

**Conclusion.** Indonesian version of CACT showed no significant correlation with FEV1 and variability PFR so that it should not replace the need of regular spirometry in childhood asthma.

## A599 Synergistic Interaction Between Bronchiolitis and PM_10_ Is Modified By *IL-13* Polymorphism on Asthma Development: Replication from Cheer Study

### Young-Ho Jung^10^, Song-I Yang^1^, Byoung-Ju Kim^2^, Ji-Won Kwon^3^, Hwan-Cheol Kim^4^, Jong-Han Leem^4^, Ju-Hee Seo^5^, Hyung Young Kim^6^, So-Yeon Lee^1^, Ho-Jang Kwon^7^, Hyo-Bin Kim^8^, Hyun-Ju Cho^9^

#### ^1^Hallym University Sacred Heart Hospital, South Korea; ^2^University of Cincinnati College of Medicine, USA; ^3^Seoul National University Bundang Hospital, South Korea; ^4^Inha University Hospital, South Korea; ^5^Department of Pediatrics, Korea Cancer Center Hospital, South Korea; ^6^Pusan National University Yangsan Hospital, South Korea; ^7^Dankook University College of Medicine, South Korea; ^8^Inje University Sanggye Paik Hospital, South Korea; ^9^Department of Pediatrics, Childhood Asthma Atopy Center, Environmental Health Center, Asan Medical Center, University of Ulsan College of Medicine, South Korea; ^10^Bundang CHA Medical Center, CHA University School of Medicine, South Korea

##### **Correspondence:** Young-Ho Jung – Bundang CHA Medical Center, CHA University School of Medicine, South Korea

**Rationale:** Asthma is a disorder that is driven by interactions between genetic and environmental factors. The aim was to investigate the interactions between bronchiolitis, PM_10_ exposure and *IL-13*polymorphism in the development of asthma from a longitudinal study.

**Methods:** The study population was derived from a prospective 2-year follow-up survey of Children’s Health and Environmental Research (CHEER). 5,443 children from 16 elementary schools in seven cities were enrolled in the CHEER. Parents of the participants were asked to respond to an International Study of Asthma and Allergies in Childhood (ISAAC) questionnaire to evaluate the presence of allergic diseases and risk factors. *IL-13* (rs20541) genotypes were determined by TaqMan assay. PM_10_ exposure was estimated by the kriging method.

**Results:** The parental history of asthma (adjusted odds ratio [aOR], 3.73; 95% confidence interval [CI], 2.63-5.31), and bronchiolitis history within the first 2 years of life (aOR, 4.77; 95% CI, 3.66-6.21) were independent risk factors for physician-diagnosed asthma. But high PM_10_ exposure for recent 5 years was not significant. When past episodes of bronchiolitis and high exposure to PM_10_ were combined, the prevalence of asthma was significantly increased (aOR, 5.28; 95% CI, 3.61-7.72; *P* for interaction=0.007), also increased risk of asthma especially in children with *GA+AA* type of *IL-13* polymorphism (aOR, 4.13; 95% CI, 1.89-9.07; *P* for interaction=0.101). New asthma diagnosis during follow-up period increased with higher PM_10_ exposure and bronchiolitis history (aOR, 3.18; 95% CI, 1.49-6.80, *P*for interaction = 0.107).

**Conclusions:** PM_10_ exposure and bronchiolitis history synergistically increased asthma risk and new development of asthma in children, particularly if they had the *IL-13* (rs20541) polymorphism. These findings suggest that PM_10_exposure and bronchiolitis in early life contribute to the development of asthma, especially in susceptible children.

## A600 The Transcription Factor Ehf Is Involved in TGF-b-Induced Suppression of Fceri and c-Kit Expression and Fceri-Mediated Activation in Mast Cells

### Susumu Yamazaki^4^, Nobuhiro Nakano^1^, Asuka Honjoh^2^, Eisuke Inage^2^, Yosuke Baba^3^, Yoshikazu Ohtsuka^2^, Toshiaki Shimizu^2^

#### ^1^Atopy (Allergy) Research Center; ^2^Juntendo University Faculty of Medicine; ^3^Juntendo University Shizuoka Hospital; ^4^Juntendo University, Japan

##### **Correspondence:** Susumu Yamazaki – Juntendo University, Japan

**Background)**

The high-affinity IgE receptor, FcεRI, which is composed of α-, β-, and γ-subunits, plays an important role in IgE-mediated allergic responses. TGF-β1 has been reported to suppress FcεRI and stem cell factor receptor c-Kit expression on mast cell surfaces and to suppress mast cell activation induced by cross-linking of FcεRI. However, the molecular mechanism by which these expressions and activation are suppressed by TGF-β1 remains unclear. In this study, we found that the expression of Ets homologous factor (Ehf) is significantly up-regulated by TGF-β1 in mouse bone marrow-derived mast cells (BMMCs). But the roles of Ehf in mast cells has not been reported. Here, we show that the transcription factor Ehf contributes to TGF-β1 induced suppression of FceRI and c-Kit expression, and FceRI-mediated activation, through down-regulation of GATA-1, GATA-2, and Stat5 expression in mast cells.

**Methods)**

BMMCs were generated by culturing bone marrow cells isolated from BALB/c mice. Two-weeks cultured bone marrow cells were cultured in BMMC medium in the presence of SCF, and recombinant murine IL-9 with or without recombinant human TGF-β1. Two-weeks cultured bone marrow cells were cultured for a further 14 days in media containing IL-3/SCF/IL-9/TGF-β1, or were transfected with a retroviral vector or lentiviral vector encoding the FLAG-tagged mouse Ehf or siEhf gene or mock vector.

**Results)**

TGF-β1 suppresses the expression of the transcription factors GATA-1, GATA-2, and PU.1, which are positive regulators of the transcription of genes encoding FcεRIα, FcεRIβ, and c-Kit, in mouse bone marrow-derived mast cells (BMMCs). In addition, the expression of Ets homologous factor (Ehf), a member of the Ets family of transcriptional factors, is up-regulated by TGF-β/Smad signaling in BMMCs. Forced expression of Ehf repressed the transcription of genes encoding FcεRIα, FcεRIβ, and c-Kit, resulting in a reduction in cell surface expressions of FcεRI and c-Kit. Furthermore, forced expression of Ehf suppressed FcεRI-mediated degranulation and IL-6 and IL-13 production. The mRNA levels of *Gata1*, *Gata2*, and *Stat5b* were lower in BMMCs stably expressing Ehf compared with control cells.

**Conclusions)**

TGF-β1 suppresses FcεRI and c-Kit expression, and suppresses FcεRI-mediated activation, through up-regulation of Ehf in mast cells.

## A601 The Follow up of the Potential Immunosuppressant Effects of Marijuana (MJA)

### Ishaq M^4^, Sameera MI Khan^1^, Imran Khan^2^, Sabeen Khan^3^

#### ^1^Consultant Inova; ^2^Cardiac Care Center; ^3^George Washington Hospital, USA; ^4^Al-Junaid Medical Center Kpk, Pakistan

##### **Correspondence:** Ishaq M – Al-Junaid Medical Center Kpk, Pakistan

The Long standing use of Marijuana may cause acute & chronic bronchitis, premalignant transformation of the bronchial lining mucosa, excessive assembly of macrophages, diminished efficacy of the immune effecter cells in the production of the inflammatory cytokines and defensive mechanism. The principal consequences of the long term use of MJA are protracted pulmonary infection and neoplastic malformation. Pulmonary infection results from the impaired mucociallary clearance reduced immunological function of the macrophages and the contamination of the MJA products by mixed flora of the pathogens (fungal and bacterial microorganisms).Individuals with AIDS may have additional susceptibility to pathogens. The drastic changes as regards to infectious and neoplastic malformation were more amongst MJA along with tobacco use than MJA use alone.

MJA contains an immunosuppressant tetrahydrocannabinolamine (THC) impairing the immunological reactivity of the T-Killer cells, macrophages and T lymphocytes and production of the immunoinhibitory helper T cells, type -2 cytokines, interleukin-10 and interleukin-4. It is presumable that THC works to impair the immunological efficacy thereby promoting host invasion by bacterial pathogens. This may be consequent upon the recurrent pulmonary infection in MJA users.

Results: The short term use of MJA may have different outcomes than long term use.

Conclusions: The active principal as derived from MJA when used in patients with AIDS may have some beneficial role as regards appetite improvement and pain relief.

## A602 Risk Factors of Allergen Sensitization at 3 Years: Results from the Gusto Study

### Evelyn Xiu Ling Loo^9^, Anne Goh^1^, Oon Hoe Teoh^1^, Yiong Huak Chan^2^, Seang Mei Saw^2^, Kenneth Kwek^3^, Peter D Gluckman^4^, Keith M Godfrey^5^, Hugo Van Bever^6^, Yap Seng Chong^7^, Bee Wah Lee^2^, Lynette Shek^8^, Alison Joanne Lee, Yong

#### ^1^Kk Women’s and Children’s Hospital, Singapore; ^2^National University of Singapore, Singapore; ^3^Department of Maternal Fetal Medicine, Kk Women’s and Children’s Hospital, Singapore; ^4^Growth, Development and Metabolism Programme, Singapore Institute for Clinical Sciences (SICS), Agency for Science, Technology and Research (A*STAR), Singapore, Singapore; ^5^Nihr Southampton Biomedical Research Centre, University of Southampton and University Hospital Southampton NHS Foundation Trust, SO16 6YD, Southampton, United Kingdom; ^6^Department of Paediatrics, Yong Loo Lin School of Medicine, National University of Singapore, Singapore; ^7^Department of Obstetrics & Gynaecology, Yong Loo Lin School of Medicine, National University of Singapore, Singapore; ^8^National University Health System; ^9^Agency of Science, Technology and Research, Singapore

##### **Correspondence:** Evelyn Xiu Ling Loo – Agency of Science, Technology and Research, Singapore

**Background**

Immune responses in allergic diseases begin with allergen sensitization which develops in early infancy. Allergen sensitization involves a complex interplay of genetic and environmental factors and sensitization patterns are reported to change with age.

**Objective**

In this follow-up study, we aim to determine the risk factors of allergen sensitization at 3 years in the GUSTO ( Growing Up in Singapore Towards healthy Outcomes) birth cohort.

**Methods**

We analyzed the electronic databases of the GUSTO study cohort which was followed up since birth. Interviews were carried out and information gathered included demographics, family history of allergy, social data and lifestyle. Mothers were administered questionnaires to obtain information about their child’s health. Eight hundred and fifty-eight infants who completed skin prick testing (SPT) to the allergens (the inhalant allergens: the house dust mites, *Dermatophagoides pteronyssinus*, *Dermatophagoides farinae,* and *Blomia tropicalis* as well as to the food allergens, egg, peanut and cow’s milk) at 36 months were analysed.

**Results**

Two hundred children (23.2%) had a positive SPT to dust mites and/or food allergens at 36 months. One hundred and ninety four subjects (22.6%) had a positive SPT to dust mites while 18(2.1%) had a positive SPT to food allergens. Early onset of atopic dermatitis before 6 months.

( Adjusted odds ratio (AdjOR) 5.11, 95% Confidence Interval (CI) :1.39-18.83, p =0.01), onset of eczema between 7 months and 12 months (AdjOR 14.43, 95%CI: 1.50-138.62, p=0.02) and having a positive SPT at 18 months(AdjOR 14.29, 95%CI: 4.36-46.72, p=<0.01) were significantly associated with a positive SPT at 36 months.

**Conclusions**

In conclusion, we found that early onset of atopic dermatitis is a risk factor for allergen sensitization. Sensitization to allergens may occur through the cutaneous route and an impaired skin barrier facilitates this.

## A603 IL-6 Blockade As a Steroid-Sparing Treatment for Rhupus Patients

### Daniela Rossi

#### G. Bosco Hospital, Turin, Italy

Overlap of Rheumatoid Arthritis (RA) and Systemic Lupus Erythematosus (SLE) is a rare clinical condition that was first described in 1974 as Rhupus. Rhupus is a rare clinical entity with a prevalence of about 0.09%. The main differential diagnoses include RA with extra-articular manifestations, SLE with rheumatoid-like articular lesions, and mixed collagenous tissue disease. Some Authors consider Rhupus to be an overlap condition of polyarthritis and SLE, since anti-CCP antibodies (which are highly specific for RA) and anti DNA /Sm (which are highly specific for SLE) coexist in Rhupus.

Therefore, diagnosis of Rhupus bases on the combination of inflammatory symmetrical erosive polyarthritis and clinical features of SLE. Recognition of these patients is important, since therapy and outcome differ from patients having RA or SLE alone.

For patients whose disease is resistant to or dependent on corticosteroids, methotrexate (MTX) and azathioprine (AZA) has been used as steroid-sparing second-line treatment with conflicting results. While MTX seemed to be effective in controlling symptoms, data on AZA remain controversial, while the TNF-blocking agents Infliximab and Etanercept have been shown to be unable to induce and maintain disease remission.

IL-6 could play an important role in the pathogenesis of Rhupus. Indeed, IL-6 levels are elevated in active disease.

We are reporting on three women 59, 51 and 46 year-old with refractory Rhupus (disease duration of 14, 19 years and 13, respectively) who were at high risk for long lasting high dose of CS, and were successfully treated with humanised anti-IL-6 receptor antibody, Tocilizumab, given monthly at the dose of 8 mg/kg.

## A604 Examination of Late Pulmonary Toxicity in Children Treated for Malignancies

### Agnes Nemeth

#### Semmelweis University, Faculty of Medicine, Hungary

The chemotherapeutical drugs and the therapeutic irradiation can demage the lungs during the anticancer therapy of childhood malignancies.

The present investigation was based on a survey in 2005, in which the authors found pulmonary function abnormalities in survivors of childhoood cancer who were treated with anticancer therapy.

The purpose of the present study was to follow- up childhood cancer survivors and detect late pulmonary toxicity.

Lung function test was performed with spirometry in 26 survivors participated in this study (10 felames, 16 males, mean age 19,4 years at the time of second follow-up evaluation). The avarage time periods from treatment until the first and second follow-up evaluation were 4.5 and 10 years, respectively.

The authors found 14 patients with pathological pulmonary function test results at the time of the first follow-up evaluation from which 7 patients had obstructive, 5 patients had mixed and 2 patients had restrictive abnormalities. However there were only 6 patients who had abnormal pulmonary function at the time of the second follow-up evaluation (2 patients with obstructive and 4 patients with restrictive pulmonary function test).

Restrictive pulmonary disorder was detected in only small part of treated patients. The obstructive pulmonary abnormalities caused by the treatment showed an improving tendency over time.

## A605 Technical Validation of the Repurposing of a Personal Particle Sampler to Determine House Dust Mite Exposure in the Ambient Air

### Torsten Sehlinger^2^, Karl-Christian Bergmann^1^, Frank Goergen^2^

#### ^1^Charité-Universitätsmedizin Berlin; ^2^Bluestone Technology Gmbh

##### **Correspondence:** Torsten Sehlinger – Bluestone Technology Gmbh, Woerrstadt Germany

**Background:**

Knowing an individual’s natural exposure to HDM is still a challenging task. The approach to solve this issue was to validate the suitability of the personal particle sampler, originally designed to sample pollen and fungal spores, to collect HDM material in its natural appearance and quantify the aeroallergen material.

**Methods:**

The personal particle sampler is a wearable device sampling ambient air and guiding the air to an adhesive stripe, where particles deposit. The stripe is contained in an exchangeable cartridge. After exposure, the stripe can be removed and analyzed microscopically or biochemically.

Two properties of the personal particle sampler were examined: The correlation of sampled HDM allergen to concentration of allergen in the ambient air, and the maximum particle size of HDM material, which could be sampled. To analyze the correlation, several particle samplers were placed in an exposure chamber, which is able to generate a definable and constant HDM particle concentration over a dedicated period of time using grinded HDM material (less than 5um). The sampled material was then analyzed using a standard antigen test (Der p1 and Der f1).

To analyze the maximum detectable particle size, different grain sizes where used in trickle tests, using full HDM bodies as the reasonable limit. The particles adhering to the stripe were verified microscopically.

**Results:**

In the environmental chamber test, a proportional relation between ambient HDM material concentration and allergen content sampled was proven. The proportional relation was significant considering the variance of the allergen concentration due to the dispersal system as well as the accuracy of the allergen analysis itself.

In the maximum particle size test, even whole HDM bodies could be sampled, thus stuck on the stripe.

**Conclusion:**

The personal particle sampler is suitable for determining an individual’s exposure to HDM material. However, it still has to be proven, that the concentration measured by the personal particle sampler correlates with an allergenic person’s recognized symptoms in real life.

## A606 Zinc Deficiency in Children with Severe Atopic Dermatitis: More Common Than Generally Thought

### Mohammad S. Ehlayel^1^, Abdul Bari Bener^2^

#### ^1^Hamad Med Corp, Qatar; ^2^Istanbul University, Turkey

##### **Correspondence:** Mohammad S. Ehlayel – Hamad Med Corp, Qatar

**Introduction:** Severe atopic dermatitis (AD), seen in 1-15% of cases may present with rare uncommon complications such as poor weight gain, malnutrition, trace elements (including zinc) deficiency. These were published as case reports only, with on large studies, particularly on children.

**Objectives:** to determine frequency and severity of zinc deficiency in AD children, and find out if there is any association between zinc level and AD severity or body mass index.

**Methods:** retrospective, case control study. Review of records of all children (<14 years) seen at Ped Allergy-Immunology clinics of Hamad General Hospital during Jun 2014-2015 with severe AD who had zinc level tested. In addition to demographic data, lab. tests (CBC with differential, IgE, serum zinc, vitamin D, food allergens tests of SPT or specific IgE), SCORAD and weight and height were collected.

**Results:** 23 children (out of 130 children, 17.7%) were found to have serum zinc-low (ZL). We case-controlled them with a group of 23, age-matched, normal-zinc (ZN) AD children. The 2 groups were similar in age (48 vs. 46 months), sex, co-existing allergies (26.1%) vs 26.1%), and no. of positive food allergens per patient (1.9 vs 2) and type of food allergens. ZL had higher positive family history of allergies (91.3% vs. 69.6%) and parental consanguinity (39.1% vs 26.1%) than ZN. Besides ZL had higher WBC (15,554 vs. 11,223 cells/ul), peripheral blood eosinophils (846 vs 685 cells/ul), and IgE (3,132 vs. 2,086 Ku/L), but lower serum zinc (8.3 vs. 12.3 umol/L) levels, lower BMI (15% vs.18%). Both groups had similar SCORAD (62 vs 63). There was no significant association between zinc level with BMI or SCORAD compared to control group.

**Conclusions:** Zinc deficiency is quite common among children with severe AD. It seems positive family history and parental consanguinity are risk factors for it. AD zinc deficient children have elevated allergic inflammatory makers irrespective of AD severity.

## A607 Strong Association Between HLA-B*5801 Allele and Allopurinol – Induced Severe Cutaneous Adverse Reactions in Vietnamese

### Hieu Chi Chu^6^, Nga Thi Quynh Do^1^, Dinh Van Nguyen^2^, Ha Thi Thu Nguyen^2^, Huong Thi Minh Le^3^, Sheryl Van Nunen^4^, Christopher Vidal^5^, Suran Fernando^4^

#### ^1^National Institute of Hygiene and Epidemiology, Vietnam; ^2^Hanoi Medical University, Vietnam; ^3^Vietnam National Hospital of Paediatrics, Vietnam; ^4^Sydney Medical School - Northern, University of Sydney, Australia; ^5^Royal North Shore Hospital, Australia; ^6^Bach Mai Hospital, Vietnam

##### **Correspondence:** Hieu Chi Chu – Bach Mai Hospital, Vietnam

**Background:** The association between HLA-B*5801 and severe cutaneous adverse drug reactions (SCARs) consisting of Stevens - Johnson Syndrome (SJS)/Toxic Epidermal Necrolysis (TEN) and HyperSensitivity Syndrome (HSS)/Drug Rash with Eosinophilia and Systemic Symptoms (DRESS) has been strongly reported in different ethnic populations. The strongest association has been described in Taiwanese study which HLA–B*5801 was found in 51/51 patients with allopurinol – induced SCARs (OR = 580.3 (95%CI: 34.4–9780.9). In Vietnam, however, this allele has not been yet confirmed or revealed as a relevant pharmacogenetic risk factor for the situation of allopurinol – induced SCARs while its prevalence is as high as 6.5%. Therefore, in this study, we sought to determine the association between HLA-B*5081 with allopurinol – induced SCARs in the large teaching center of allergy and clinical immunology at Bach Mai Hospital.

**Methodology:** Twenty - two cases of SCARs caused by allopurinol and confirmed by using established criteria, were recruited. Genomic DNA was extracted from whole peripheral blood with EDTA using Qiagen kits, as instructed by supplier. Polymerase chain reaction using sequence - specific primers was used to detect HLA-B*5801. Genotyping of the amplified products were determined using agarose gel electrophoresis. A random sample of positive samples was validated using direct DNA sequencing. After comparing results from both methodologies 100% agreement was found.

**Results:** A total of 22 patients comprised 13 SJS/TEN (59.1%), Overlap 1 (4.5%) and 8 HSS/DRESS (36.4%) were genotyped. Out of 22 individuals, 21 (95.9%) patients were positive for HLA-B*5801.

**Conclusion:** Our result suggests a strong association between HLA-B*5801 and allopurinol – induced SCARs in Vietnamese. These finding is the first to be described in the Vietnamese population. Considering that HLA-B*5801 is present in 6.5% of the general Vietnamese population, our study shows that there is a significant risk that someone carrying the HLA-B*5801 allele will suffer from a drug induced SCAR if treated with allopurinol. More individuals suffering from SCAR are also of a burden on the health system and thus needs to be addressed. Future studies will be performed on a larger sample of individuals followed by a case-control study.

## A608 Successful Rapid Desensitization to Glatiramer Acetate: Report of 2 Cases

### Fotis Psarros^3^, Ekaterini Syrigou^1^, Ekaterini Politi^2^, Spyridon Chrysoulakis^3^

#### ^1^“Sotiria” General Hospital; ^2^Araiteion Hospital Greece; ^3^Athens Naval Hospital

##### **Correspondence:** Fotis Psarros – Athens Naval Hospital, Greece

Glatiramer acetate (GA) is a generally safe, effective and well tolerated drug, but discontinuation of treatment may be required in up to 10% of cases due to severe systemic postinjection reactions. Notably, there are only sparse data on desensitization protocols for patients with hypersensitivity to GA.

We present 2 cases of successful desensitization to Glatiramer acetate (GA).

A 51-year-old female with MS was treated with GA for the last four years without any adverse reaction. Suddenly, after GA injection she developed generalized urticaria. Skin prick (SPT) and intradermal (ID) testing to GA and mannitol (an inactive ingredient of Copaxone^R^ with allergenic potential) was performed. Histamine and NaCl was used as positive and negative control. SPT showed a borderline reaction to GA (wheal diameter=3mm) at a concentration of 20mg/ml, while a positive reaction was shown on intradermal skin testing at a concentration of 0,002mg/ml. SPT and ID testing to mannitol, were negative. A procedure of desensitization to GA was carried out in an outpatient setting under close medical supervision. Increasing dosages of GA were administered subcutaneously every 15 minutes, at a starting dose of 20ng followed by gradual dose escalation up to 20mg. The entire desensitization procedure lasted 3 hours and 15 minutes. The desensitization procedure was well tolerated with no adverse events. The patient was able to resume GA treatment with no recurrence during a follow up period of 12 months.

A 39 years old female was treated for MS with GA for 3 months without any adverse reaction until her last injection. Immediately after administration of Copaxone^R^ she experienced, life threatening anaphylaxis with urticaria, angioedema, abdominal cramps, dyspnea, drop in blood pressure and loss of consciousness. The patient was referred to our department for further evaluation. Allergy testing, with the already described procedure, revealed positive ID test at 0,002mg/ml. We used exactly the same protocol of desensitization in an inpatient basis. During the process she experienced anaphylactic reactions were successfully treated. After 2 days the patient was able to tolerate 20mg of GA. The patient receives GA daily after 5 months without any adverse event.

These 2 cases illustrates that rapid subcutaneous desensitization may allow continuation of GA treatment in patients with a history of systemic reactions.

## A609 Asthma Exacerbations Seasonal Variation in Two Perennial Phenotypes during Twenty Years (1995-2014): House Dust Mite Monosensitized and Non Atopic Patients

### Dimitrios Vourdas^2^, Konstantinos Petalas^1^

#### ^1^Hellenic Air Force; ^2^251 General Air Force Hospital Greece

##### **Correspondence:** Dimitrios Vourdas – 251 General Air Force Hospital Greece

**Background:** Asthma exacerbations (AE) are caused for a variety of reasons including unrecognized disease, undertreated or unresponsive to conventional therapy asthma, recent exposure to triggers like viruses, usually rhinovirus or allergens. AE is defined as a worsening of asthma sufficient to require medical intervention and usually administration of oral steroids reflected objectively as a fall of PEF, FEV1 ≥20% or 30%. This study was performed to investigate the epidemics of exacerbations in mites monosensitized asthmatics. Seasonal variability of AE in such patients could be used as a tool for a convenient and preventive management. Non atopic asthmatic patients were used as a control group and this way we could understand how important trigger is the allergen comparing to virus.

**Methods:** We have studied, from 1995 to 2014, AE in patients suffering from these two different phenotypes of asthma. Seasonal variation of AE in each phenotype separately and consequently comparison of them were evaluated by applying the x^2^ –test and the null hypothesis. The null hypothesis is that there is not difference in the number of AE occurred in each month of the year.

**Results:**

Mite monosensitized patients (Month/AE number): Jan / 32, Feb / 41, Mar / 58, Apr / 59, May / 74, Jun / 55, Jul / 37, Aug / 10, Sep / 73, Oct / 67, Nov / 54, Dec / 30.

By applying the χ2-test for 11 d.f we obtained χ2=84,319 (P-value<0,0001). Therefore the null hypothesis is not accepted and there is statistical significant difference in the number of AE occurred during the year (higher in spring and autumn months).

Non atopic patients (Month/AE): Jan / 249, Feb / 239, Mar / 245, Apr / 237, May / 194, Jun/ 173, Jul / 124, Aug / 69, Sep / 249, Oct / 309, Nov / 283, Dec /. 

By applying the χ2-test for 11 d.f we obtained χ2=229,940 (P-value<0,0001). Therefore the null hypothesis is not accepted and there is statistical significant difference in the number of AE occurred during the year (higher in spring and winter months).

Comparison of both time distributions revealed significant difference between them, χ2=62.042 and P-value<0.0.

**Conclusions**: Our results showed that in Greece during 20 years (from 1995 to 2014) AE are seasonal variable in non atopic and mites monosensitized asthmatics but different between them. The results indicate that atopics react to viruses in a different way comparing to non atopics.

## A610 Occupational Allergy to Fungal Spores Among the Farmers of Paddy Fields in West Bengal, India: An Aeromycological and Immunological Approach

### Mouli Saha, Kashinath Bhattacharya

#### Visva Bharati, Santiniketan, India

##### **Correspondence:** Mouli SAHA – Visva Bharati, Santiniketan, India

**Introduction:** A large number of farmers work in paddy field around the world, suffering from asthma, allergy and systemic mycosis; however, it appears that adequate information on the fungal aerosols over the paddy field of 24-Parganas (North) and their allerginic effects of farmers are largely lacking.

The aim of the study was to assess the concentration of the major airborne pathogenic fungal spore over paddy fields with an object to identify the fungal allergen which are causing respiratory allergy among farmers, by immune-clinical techniques.

**Method:** Volumetric assessment of airborne culturable and non-culturable fungal spores was performed in the experimental site (North 24 Parganas, West Bengal, India) for 2 consecutive years (November 2011–October 2013) by using an Andersen Two Stage volumetric sampler and a Burkard Personal Slide Sampler for trapping culturable and non-culturable types of fungal spores. Culturable fungal spores were sub-cultured in medium for isolating and identifying individual species. The fungal antigens were prepared from the prevalent culturable types of selected fungal spores in Y-cell lysis reagent (buffer). The allergenic potential of the antigens were evaluated on atopic farmers by SPT (*in vivo*) followed by ELISA & IgE-specific Immunoblotting (*in vitro*). The antigens resolved in 11% SDS PAGE and IgE-reactive allergen were identified by western immunoblotting.

**Result:** A total of 34 types of fungal spore and 24 types of viable colony-forming fungal spores were recorded. The major perennial cultured fungal spore types included Aspergilli group, *Fusarium oxysporum*, *Cladosporium cladosporioides*, *Nigrospora oryzae, Helminthosporium oryzae*, *Alternaria alternata*, *Drechslera* sp. etc. showed higher sensitivity in SPT. Among these fungal spores Aspergilli group (*A.niger, A. fumigatus, A. flavus, A. clavatus, Penicillium claviforme*) showed highest (23.7%) reactivity followed by *Fusarium oxysporum, Helminthosporium oryzae, Alternaria alternate, Cladosporium cladosporioides, Nigrospora sp.* in SPT carried out in 214 adult agricultural field workers with respiratory disorders. Total fungal protein of *A.niger, A. fumigatus, A. flavus, A. clavatus, Penicillium claviforme, Fusarium oxysporum, Helminthosporium oryzae, Alternaria alternate, Cladosporium cladosporioides, Nigrospora sp.* resolved into 11,15,17,13,16,12,10, 18,13 and 12 distinct bands respectively in 11% SDS PAGE and in immunoblotting 5, 6, 4, 6, 7, 3, 4, 4, 5 and 3 IgE reactive allergens were identified respectively.

**Conclusion:**

The airborne fungal spores not only cause the pathogenic infection to rice plants but also cause respiratory allergy among the paddy field farmers. Western immunoblotting revealed the major IgE reactive fungal allergens. The finding indicates that occupational allergy to fungal spores is an important health hazards among farmers of West Bengal.

## A611 Study of Efficacy of Sublingual Immunotherapy (SLIT) in Cases of Severe Persistent Allergic Rhinitis

### Subir Jain

#### Ent Centre, India

Title: Study of efficacy of sublingual immunotherapy (SLIT) in cases severe persistent allergic rhinitis.

**Purpose:** To asses efficacy of sublingual immunotherapy[SLIT] in treatment of severe persistent allergic rhinitis in the age group from 7 years to 63 years of either sex.

**Material & Method:** 214 patient of severe persistent allergic rhinitis were included to asses efficacy of sublingual immunotherapy [SLIT]. These patients were regular in treatment for more than 36 months. Symptom score were recorded on regular intervals 1st at the beigning of SLIT, then at completion of 1st maintenance vial i.e. 3rd vial then at every next maintenance vial. All patients follow up done about 4 to 5 times during Sublingual immunotherapy[SLIT] at regular intervals. Rescue medications for exacerbations of symptoms during pollen seasons were also taken in to account.

Number of Aeroallergens included ranges from average of 2 to 4 allergens.Glycerinated aqueous allergenic extract from various allergens were taken as per their sensitivity pattern.Different Allergen extracts suspended in extracting fluid [coca solution] containing 50% glycerine i.p. according to w/v ratio. Usual concentrations were Initiation 1:500,build up 1:250,maintainance 1:100, 1:50, 1:20,1:10.

**Result:** In above study results were encouraging. In symptom score scale of 0 to 3.[0 no symptoms, 1 mild, 2 moderate, 3 severe]All patients were having severe symptoms before starting of sublingual immunotherapy. At the completion of 36 months of sublingual immunotherapy. Out of 214 patients 144 patients were on score 1, 54 patients on score 2 and 16 patients on score 0. Number of rescue medications along with SLIT was remarkably reduced even during pollen season.

**Conclusion:** Sublingual immunotherapy showing promising results in cases of chronic allergic rhinitis.

## A612 Mesenchymal Stem Cells Suppress Lung Inflammation and Airway Remodeling in Chronic Asthma Rat Model Via PI3K/Akt Signaling Pathway Mesenchymal Stem Cells Suppress Lung Inflammation and Airway Remodeling in Chronic Asthma Rat Model Via PI3K/Akt Signaling

### Xiaolian Song, Haiyan Lin

#### Shanghai Tenth People’s Hospital, Tongji University, China

##### **Correspondence:** Xiaolian Song – Shanghai Tenth People’s Hospital, Tongji University, China

**Background:** Mesenchymal stem cells (MSCs) came out to attract wide attention and had become one of the hotspots of most diseases’ research in decades. But at present, the mechanisms of how MSCs work on chronic asthma remain undefined. Our study aims at verifying whether MSCs play a role in preventing inflammation and airway remodeling via PI3K/AKT signaling pathway in the chronic asthma rats model.

**Methods:** First, an ovalbumin (OVA)-induced asthma model was built. MSCs were administered to ovalbumin-induced asthma rats. The total cells in a bronchial alveolar lavage fluid (BALF) and inflammatory mediators in BALF and serum were measured. Histological examination of lung tissue was performed to estimate the pathological changes. Additionally, the expression of phosphorylated-Akt (p-Akt) in all groups was measured by western blot and immunohistochemistry (IHC).

**Results:** Compared to normal control group, the degree of airway inflammation and airway remodeling was significantly increased in asthma group. On the contrary, they were obviously inhibited in MSCs transplantation group. Moreover, the expression of p-Akt was increased in lung tissues of asthmatic rats, and suppressed by MSCs transplantation.

**Conclusion:** Our results demonstrated that MSCs transplantation could suppress lung inflammation and airway remodeling via PI3K/Akt signaling pathway in rat asthma model.

## A613 Development of Allergen ELISA Kits for Dust Mites, Pollen, and Pet Dander

### Kyohei Nishikawa^1^, Takashi Shimada^1^, Hiroshi Yasueda^2^, Tadao Enomoto^2^, Daisuke Aizawa^3^, Takayoshi Kobayashi^3^

#### ^1^Nichinichi Pharmaceutical Co., Ltd.; ^2^Jpn Health Promotion Supporting Network; ^3^Kanto Chemical Co., Inc.

##### **Correspondence:** Kyohei Nishikawa – Nichinichi Pharmaceutical Co., Ltd., Japan

**<Background>**Allergic diseases such as asthma, atopic dermatitis and allergic rhinitis have remarkably increased, and they are caused mainly by allergens such as dust mites, pollen and pet dander. For the measures to prevent and control allergic diseases, it is important to measure allergens in the environment.

**<Objective>**We established a highly sensitive enzyme-linked immunosorbent assay (ELISA) to quantify specifically Dermatophagoides mite allergens (Der p 1, Der f 1 and Der 2), Japanese cedar pollen allergens (Cry j 1 and Cry j 2) and cat allergen (Fel d 1).

**<Methods>**Allergen samples were incubated in the wells coated with a primary antibody for 2 hours at 37°C. After washing, a biotinylated secondary antibody against the allergen was incubated for 1 hour at 37°C. The allergens were detected using HRP-conjugated streptavidin and its substrate at 450 nm.

**<Result>**The working range was 500 to 32,000 pg/mL for Der p 1 and Der f 1, 100 to 6,400 pg/mL for Der 2, 250 to 16,000 pg/mL for Cry j 1, 2,500 to 160,000 pg/mL for Cry j 2, and 100 to 6,400 pg/mL for Fel d 1. In all systems, the intra- and inter-assay coefficient of variation was less than 5%. Furthermore, each assay showed no significant cross-reactivity with other allergens.

**<Conclusion>**Our established ELISA systems for these allergens are useful for not only the qualification of allergens in the environment but also the quality control of allergenic products for sublingual immunotherapy and the evaluation of the allergen inactivation effect in industrial products.

## A614 Yoga As a Lifestyle Modification to Improve the Quality of Life in Smokers with Allergic Rhinitis

### Chellaa R

#### St.Johns National Academy of Health Sciences, India

**Background & Objective:**

Yoga and meditation have proven to be an alternative drug free treatment for smoking cessation. Allergic Rhinitis is common and has a serious impact on the quality of life of tobacco smokers. There have been no scientific studies done to assess the effect of Yoga on the airway resistances of tobacco smokers. The main objective of the study was to see if yoga can improve airway resistances and hence improve the quality of life in tobacco smokers.

**Methodology:**

15 smokers with allergic rhinitis were chosen for the study and they underwent an ENT examination. The subjects were taught specific yogasanas and were asked to practice the same for a period of 2 months and reduce the number of cigarettes. The objective analysis and subjective analysis were done before the yoga training and after 2 months of yoga practice. The Objective analysis for upper airway resistance was measured using a Rhinomanometer and the lower airway resistance was measured using a Spirometer. The quality of life analysis was done using the Short form -12 health survey (SF-12) questionnaire and Sino Nasal Outcome Test (SNOT) questionnaire.

**Results:**

The data was analyzed by doing a Paired (2-tailed) T- Test, using the SPSS (Software package for the social sciences) version 16.The mean standard deviation of the nasal airway resistance was found to be 0.36 ± 0.10 Pa/cm3/s before doing yoga and it was 0.30 ± 0.10 Pa/cm3/s at 150 Pa pressure after doing yoga (‘p’ value was <0.01). The mean standard deviation of the FEV1/FVC % before doing yoga was 73.6 ± 18.7 and after doing yoga was 81.8 ± 9.9 (‘p’ value was <0.05). Quality of life questionnaire, SF-12 showed highly significant improvement in both Physical (p<0.05) and Mental (p<0.05) composite score and there was significant reduction in SNOT score (p<0.001) after the practice of yoga.

**Conclusion:**

Results indicate there is an improvement in the nasal airway resistance after the practice of yoga. The quality of life improvement was understood with the help of the SF-12 and SNOT questionnaire. A healthy discipline of yoga as a lifestyle modification also helped the smokers to decrease the number of cigarettes and lead a better life.

## A615 Study of Incidence of Severe Persistent Allergic Rhinitis in Different Age Groups,Sex Prevalance and Type of Allergen” Aeroallergen or Food Allergen” Responsible for Severe Persistent Allergic Rhinitis in Central India

### Subir Jain

#### Ent Centre, India

**Title:** Study Of Incidence Of Severe Persistent Allergic Rhinitis In Different Age Groups,Sex Prevalance And Type of Allergen” Aeroallergen Or Food Allergen” Responsible For Severe Persistent Allergic Rhinitis In Central India.

**Purpose:** To Know Which Age Group,Sex, Is Maximum Affected From Severe Persistent Allergic Rhinitis And Which Type Of Allergen Are Responsible For Severe Persistent Allergic Rhinitis.

**Material and Method:** 810 patients of Severe Persistent Allergic Rhinitis of different Age groups of either Sex ranging from 5 years to 63 years were taken.

After detailed history. Clinical Examination of ENT, Routine blood counts, absolute Eosionophil count, Total serum IgE estimation. Patients were off the Antihistaminic for minimum of seven days. Allergy test by Modified Skin Prick Test method was performed on upper limbs on palmer aspect of forearms. Glycerinated Histamine Acid Phosphate as positive control and Glycerinated Buffer Saline as negative control were used. Each patient was tested for same 140 Allergens. Wheal & Flare response recorded.

**Result:** In the above study with 810 patients of either sex, following were the observations.

Incidence Of Severe persistent Allergic Rhinitis In Different Age Groups was as follows.

05 to 10 years – 17 patients – 2.09%, 11 to 20 years – 173 patients – 21.35%, 21 to 30 years – 287 patients – 35.43%, 31 to 40 years – 212 patients – 26.17%, 41 to 50 years – 88 patients – 10.86%, 51 to 60 years – 31 patients – 3.82%, 61 to 70 years – 2 patient – 0.24%.

Previlance Of Severe Persistent Allergic Rhinitis In Different Sex Groups was as follows.

Male patients – 422 – 52%, Female patients – 388 – 48 %.

Type Of Allergen Responsible For Severe persistent Allergic Rhinitis was as follows.

Different Aero allergen – 640 – 79%, Different Food allergens – 408 – 50%.

**Conclusion:** In Central India Incidence Of Severs Persistent AllergicRhinitis Is Maximum In The Age Group Of 21 to 30 Years 35.43%. Males are more affected than Females in the ratio of 52% : 48%. Aeroallergens are mainly responsible as causative factor for Severe Persistent Allergic Rhinitis.

## A616 Causative Allergens in Cases of Severe Persistent Allergic Rhinitis in Central India

### Subir Jain

#### Ent Centre, India

**Title:** Causative Allergens In Cases Of Severe Persistent Allergic Rhinitis In Central India.

**Purpose:** To know the Allergen pattern of Central India in cases of Severe Persistent Allergic Rhinitis. From year 2006 to 2014.

**Material & Method:** 810 patients of Severe Persistent Allergic Rhinitis in the age group of 06 to 63 years of age of either sex were taken for study of Allergen Pattern by Modified Prick Test.

After detailed history. Clinical Examination of ENT, Routine blood counts, absolute Eosionophil count, Serum IgE estimation. Patient were off the antihistaminic for minimum of seven days. Allergy test by modified prick method was performed on upper limbs on palmer aspect of forearms. Glycerinated Histamine acid phosphate as positive control and Glycerinated buffer saline as negative control were used. Each patient was tested for same 140 Allergens. Wheal & flare response recorded.

**Result:** In above study with 810 patients following were the percentage of different Allergens positive in above group.

House dust mite 18% with D. Farinae 18%, Pollens 78.5% with Prosopis Juliflora 15.5%, Fungus 13.5% with Aspergillus Flavus 4.5%, Insects 64.5% with Cockroach female 35.5%, Dusts 38.5% with Grain Dust Rice 22%, Danders 21% with Human Dander 6.5%, Fabrics 5% with Silk 2%, Foods 50% with Milk 5%, Miscellaneous 7.5% with Parthenium Leaves 4%.

**Conclusion:** The above study gave us the common Allergen pattern in Central part of India in cases of Severe Persistent Allergic Rhinitis as Pollens 78.5%, Insects 64.5%, Food 50%, Dusts 38.5% were mostly responsible for Chronic Allergic Rhinitis.

## A617 Atopic Dermatitis: A New Data on the Mechanisms of Chronic Pruritus

### Marina Yudina

#### European Medical Center, Russia

**Background and Objective:** Pruritus or itch is defined as a cutaneous sensation that provokes a desire to scratch. Pruritus is one of the major symptoms in atopic dermatitis (AD), and is of great interest. Mechanisms of itch and its neuronal pathways are being investigated using different approaches; still they are not yet fully understood. The aim of this study was to achieve new data on the mechanisms of chronic pruritus by means of innovative neurophysiological methods of itch research.

**Methods:** Short-latency and long-latency pain-related somatosensory electrically evoked potentials (SEP) as well as long-latency evoked potentials to thermal stimulation with the innovative Contact Heat Evoked Potential Stimulator (CHEPS) were studied in 38 AD-patients and 26 healthy volunteers. **Quantitative Sensory Testing (**QST**)** of thermal perception thresholds was performed in 22 AD-patients and in 15 healthy volunteers.

**Results:** AD patients showed lowered motor and pain thresholds to electrical stimulation while SEP recordings as compared with healthy individuals (g=0.004). Brain hyperactivity to electrical stimuli, delayed thermal evoked potentials and elevated cold, warm and cold-induced pain thresholds were revealed in AD-group as compared with healthy controls (p=0.007).

**Conclusions:** The data indicate small nerve fibers dysfunction in AD-patients that may contribute to the pathogenesis of AD and chronic itch. This dysfunction may determine a predisposition to atopic dermatitis, the severity of pruritus and the tendency to its chronic course, although further evaluation is required. Overall, the neurophysiological data provide the evidence of an alteration in the central responses to afferent inputs in AD-patients suffering from chronic itch. The study demonstrates objective approaches to assess the function of small nerve fibers in patients with chronic pruritus.

## A618 The Efficacy and Safety of Peanut Oral Immunotherapy in High-Dose with Predicting Factors

### Ishaq M^4^, Sameera MI Khan^1^, Imran Khan^2^, Sabeen Khan^3^

#### ^1^Consultant Inova; ^2^Cardiac Care Center; ^3^George Washington Hospital, USA; ^4^Al-Junaid Medical Center Kpk, Pakistan

##### **Correspondence:** Ishaq M – Al-Junaid Medical Center Kpk, Pakistan

**OBJECTIVE**

Is to test the efficacy and safety of a novel oral immunotherapy (OIT) protocol for peanut allergy.

**METHOD**

In the study concerned, Forty peanut-allergic children (mean age 10 years) had oral allergen preparation. This was administered with serial rising titers and dosing at about 2 weekly to 850 mg peanut protein. There was maintenance of 28 weeks.

**RESULTS**

All children had positive challenges. Of the total, 30 had tolerated the rising concentration of the titer. 2 of the total then dropped out, while 4 out of the total had tolerated the rising concentration of the allergenic material containing 250mg -500 mg. Some 30 out of 40 tolerated higher dosing and maintenance at 850 mg protein/day.

Two had some severe degree of allergenic reaction mandating the administration of adrenaline, whereas the rest had mild allergic reaction that subsided without resorting to adrenaline solution. Twelve with pre-immunotherapy peanut IgE over 28.3 kU/L required no dose adjustment compared with 7 with pre-immunotherapy peanut IgE level almost 27. kU/L. Eighteen of the 40 mandated a slight dose reduction as the reactions possibly linked to factors, such as exertion, infection and fatigue ability. However after 7 weeks, 25 out of 40 individuals had no reaction to a 2.7 g protein challenge.

**CONCLUSIONS**

This study with serial dilution and dose increments has been followed with better outcome than the former trials. A higher fold such as 1000 and over in the amount of peanuts is tolerated with excellent safety profiles. Except a few cases there was no serious adverse effects observed in the rest of individuals.

## A619 Evaluation of Long-Term Prognosis and Topical Corticosteroid Usage after One Year of Proactive Treatment for Children with Moderate-to-Severe Atopic Dermatitis

### Mayako Saito

#### National Center for Child Health and Development, Japan

**Background:** Proactive treatment of atopic dermatitis (AD) with intermittent application of anti-inflammatory agents after induction of remission has been recommended as a maintenance therapy for the long-term management of AD. Most of the previous studies examined the effect of twice-weekly application of topical corticosteroid (TCS). In our hospital, once remission can be achieved with this dosage, we will attempt to reduce the frequency with the aim of ceasing the usage of TCS.

**Objective:** To investigate the prognosis and topical corticosteroid usage after one year of proactive treatment for children with moderate-to-severe AD.

**Methods:** A retrospective chart review was conducted for patients under 15 years of age with moderate-to-severe AD (SCORAD >30) hospitalized for the first remission induction at the National **Center** for Child Health and Development in Tokyo, Japan, between January 2009 and April 2014. The frequency of TCS application one year after admission, together with the level of serum thymus and activation-regulated chemokine (TARC), were assessed.

**Results:** Seventy-two patients met the inclusion criteria and were analyzed. The median age was 14.5 months old (range: 2 months to 14years) at admission and baseline median SCORAD was 75.5 (IQR: 64.9 -85.85). All patients went into remission within 1-2 months of induction treatment at our hospital. During proactive treatment after remission, most patients used 0.1% hydrocortisone butyrate for the face and 0.1% betamethasone valerate for the body. At the follow-up visit after 12 ±1 months from admission, 49 patients (69.0%) maintained the remission for their face with less than twice-weekly application of TCS (once or less than once-weekly, n=20; emollient only, n=10; topical tacrolimus, n=19). Twenty-nine patients (40.3%) maintained good condition for their body with less than twice-weekly TCS application (once or less than once weekly, n=28; emollient only, n=1). Eleven patients (15.3%) needed TCS three to four times a week due to minor recurrence during proactive treatment. The median level of serum TARC was 5,928 pg/mL (n=72) at admission, which was decreased to 786.5 pg/mL (n=50) after 12±4 months.

**Conclusions:** Our proactive treatment for moderate-to severe AD resulted in favorable long term-prognosis such as one-year remission with twice-weekly or less application of TCS.

## A620 Allergy Symptoms in the First Two Months of Life

### Nurul Iman Nilam Sari

#### Harapan Kita Mother and Children Hospital, Indonesia

**Allergy Symptoms in Infants: Preliminary Study of The First Two Months of Life**

**Abstract**

**BACKGROUND AND OBJECTIVE**

The Prevalence of atopic disease has been rising since the latter part of the 20^th^century. Genetic and environmental factors determine the dysregulation and the development of an atopic disease. The first atopic disease to manifest is eczema, which usually commences in early infancy. For up to 40% of children with atopic dermatitis, most likely to develop respiratory symptoms before 5 years of age. This progression in atopic disease is termed the “atopic march”. We performed database to show the profile of allergy symptoms in infants during the first two months of life.

**METHODS**

We studied the incidence of allergy symptomps in a prospective birth cohort. A total of 81 infants has been followed up for two months. Atopic disease in parents and siblings were recorded at birth. Allergy symptoms such as atopic dermatitis, wheezing, and gastrointestial symptomps have been recorded.

**RESULTS**

The common initial symptom to occur in early infants was atopic dermatitis (16%) with a family history of atopic disease (22,2%). Atopic dermatitis occurred in infants with history of atopy was 69,2%; of those with single or double parental atopic history, 33,3% and 66,7%, respectively. Infants with atopic dermatitis born by section caesarean 53,8%. Incidence of atopic dermatitis was 15,4% in exclusive breastfeeding infants. Wheezing and Gastrointestinal symptoms were not detected in this study.

**CONCLUSIONS**

Atopic dermatitis is the first clinical manifestation of allergy and the highest incidence during the first two months of life.

**Keywords:** allergy symptoms, atopic dermatitis, infants

## A621 Factors Related to the Seasonal Variation of Allergic Rhinitis

### Jae Young Kim^1^, Jaechul Song^1^, Inah Kim^1^, Kyeong Joon Lee^1^, Soo Jin Park^2^, Soo Yong Roh^1^

#### ^1^Hanyang University College of Medicine, South Korea; ^2^Hanyang University

##### **Correspondence:** Jae Young Kim – Hanyang University College of Medicine, South Korea

**Introduction**

This study is for investigating AR(allergic rhinitis) affecting factors in Korea with a sample region. AR is one of the respiratory diseases(ICD10:J00-J99) which have close connection with the health of Korean people(Among 15 diseases of top rank in 2010 on outpatients’ hospital visiting, 4 diseases were related with respiratory diseases in Korea).

**Method**

Target area was Seongdong which is one of 25 districts in Seoul, Korea. Dependent variable is ARV(AR outpatients’ hospital visiting) with ICD10 codes J301~304. Independent variables are meteorological variables(T_M_, T_avr_, T_m_, Prcp(precipitation), humidity(RH), DTR(Diurnal Temp. Range), WV(Wind velocity), Pollen data(Poll, The count sum of grass, ragweed, wormwood, JapHop, alder, birch, hazelnut and oak). Statistical researches including factor analysis, correlation analysis, time series analysis(autocorrelation), independent t-test were performed.

**Result**

(1) ARV of Seongdong can represent those of Seoul and Korea(R=0.968, p<.000 respectively).

(2) 3 factors(T_M_, T_avr_, RH) were proved not to be the main affecting factors by corr. analysis. Factor analysis with the rest 5 variables can make 3 main groups: 1^st^group (T_m,_ Prcp); 2^nd^group(DTR, Poll); 3^rd^group(WV) (KMO=.484, Bartlett’s χ^2^ =164.846, p<.000). Time series analysis(autocorrelation) shows these 5 variables and ARV have the highest autocorrelation coefficients at every 12^th^month (p<.000 respectively).

(3) there are 2 AR increase and 2 AR decrease in a year(1^st^AR increase(Feb~Apr), 1^st^AR decrease(Apr~Jul), 2^nd^AR increase(Jul~Nov), 2^nd^AR decrease(Nov~Feb of the next year).

(4) By independent t-test with 2 tops and 2 bottoms of ARV, periodical affecting factors were revealed as follows; 1^st^AR increase(p=.308) ,DTR(p=.016), T_m_(p=.000), WV(p=.046); 1^st^AR decrease(p=.043), DTR(p=.001), T_m_(p=.000), Prcp(p=.034), WV(p=.021); 2^nd^AR increase(p=.010), T_m_(p=.000), Prcp(p=.028). 2^nd^AR decrease(p=.070), T_m_(p=.035), WV(p=.046).

(5) The ranges of 5 main affecting variables in 1 year(A) and in AR increase (when AR Z-value>0)(B) are as followings: T_m_((A)-9.9~25.6°C, (B)-9.9~22.1°C). Prcp((A)0.0~498.8mm, (B)0.0~270.5mm). DTR((A)3.5~13.6°C, (B)3.5~13.6°C). Poll((A)0~1870, (B)0~1870). WV((A)1.4~3.8m/sec, (B)1.4~3.8m/sec). This shows ARV didn’t occur when T_m_>22.1°C or Prcp>498.8mm.

**Summary**

Seongdong which can represent Seoul and Korea(R=.968, p<.000) has 2 AR increase and 2 AR decrease in a year. The only affecting factor on ARV all the year round was T_m_. Prcp(Apr~Nov) and WV(Nov~Apr) are significantly noticeable. T_m_and Prcp have the range in which ARV didn’t occur.

**Acknowledgement**

KCDC(2013E2100101), AR DB(NHIC), Meteorological DB(KMA), Pollen DB(Advanced Research on Applied Meteorology of the National institute of Meteorological Research).

## A622 Allergic Risk Survey in Lao Children at out-Patient Department, Children’s Hospital, Vientiane Capital, Lao PDR

### Somxay Billamay

#### Children’s Hospital, Laos

**Rationale and Background:** Allergic diseases are increasing in Lao children in the recent years. In Lao PDR, no studies have been conducted to study the allergic risk to develop allergic diseases in children.

**Methodology:** Using 3519 questionnaires of main symptoms of asthma, cow milk allergy, allergic rhinitis, atopic dermatitis and secondary symptoms of urticaria, drug allergy, food allergy, allergic conjunctivitis, data was collected from parents and family members of patients. A scoring system of main and secondary symptoms was designed, with a score of 2, or greater than 2, placing them in the high risk category to develop allergic diseases. The survey was conducted from January 4 to May 30, 2015.

**Results:** All patients are between 0-15 years of age, 77% are younger than 5, 52% of patients are male. All family members of patients had the main symptoms of allergic rhinitis 7.1%, asthma 1.8%, atopic dermatitis 1.3% and cow milk allergy 0.3%. The highest percentage of secondary symptoms is 3% with urticaria, drug allergy 1%, food allergy 0.8% and allergic conjunctivitis 0.3%. The survey results indicate that 9.5% of patients had a score of 2 or greater, placing them in the high risk category, the remaining 90.5% had a score of less than 2.

**Conclusion:** The highest main symptom of patients’ family members is allergic rhinitis and the highest of secondary symptoms is urticaria. The allergic risk to develop allergic diseases of Lao children is 9.5% in this survey.

## A623 Correlation Between Food Allergy, Aeroinhalant Allergy, Allergic Rhinitis, Atopic Dermatitis, and Acute Upper Respiratory Tract Infections and Levels of Severity of Asthma in Pediatric Medicine Department Saiful Anwar Hospital Indonesia

### Muchammad Fahrul Udin

#### Saiful Anwar Hospital, Indonesia

**Background:** Child who has asthma usually also has another event of food allergy, aeroinhalant allergy, allergic rhinitis, atopic dermatitis or acute upper respiratory tract infections (URTI). Unfortunately, there were lack evidences support correlations between these factors and the severity of asthma in Indonesian children. The aims of this study is to find correlation between food allergy, aeroinhalant allergy, allergic rhinitis, atopic dermatitis or acute upper respiratory tract infections (URTI), and the severity of asthma in Pediatric Medicine Department Saiful Anwar Hospital Indonesia

**Method:** Cross sectional study was done to all children who diagnosed as asthma and came to Pediatric Outpatient Clinic of Saiful Anwar Hospital Malang since 2014 to 2015. The classification of asthma’s severity in these children were based on GINA’s criteria: intermitten, mild persistent, moderate or severe persistent. Food allergy or aeroinhalant allergy was based on history of this allergy and positive result of skin prick test. Diagnosis of allergic rhinitis was based on AIRA criteria. The diagnosis of atopic dermatitis was based on history taking and clinical finding. Mann whitney analysis was used to find out the correlation between these risk factors and the severity of asthma.

**Results:** There were 221 participants, the proportion between boys and girls was 3:1. The risk factors who had the highest percentages were aeroinhalant allergy and food allergy, 66.5% and 57% respectively. No risk factors had significant correlation with the severity of asthma (p<0.05).

**Conclusions:** No risk factors had significant correlation with the severity of asthma in Pediatric Medicine Department Saiful Anwar Hospital Indonesia. However, the events of aeroinhalant allergy and food allergy were high in pediatric asthma, and it needs a great concern. Another research is needed to explore correlation between both risk factors and the other component of asthma,such as the severity of asthma attack.

## A624 Comparative Study of Pine, Oak, and Ginkgo Pollen Counts in Korea during Last Four Years

### Mae Ja Han^3^, Jae-Won Oh^1^, Kyu Rang Kim^2^, Baek-Jo Kim^2^

#### ^1^Hanyang University Kuri Hospital; ^2^Korea Meteorological Agency; ^3^Korea Meteorological Administration, South Korea

##### **Correspondence:** Mae Ja Han – Korea Meteorological Administration, South Korea

**Background**

Airborne pollen is a major cause of allergic disease (pollinosis). Recently sensitization rates for pollens are continuously increased in Korea. Allergenic pollen in Korea has identified 25 species. Oak is known a strong allergen, the cross-reactivity has also reported in birth and alder. Although the pine pollen has been known as “weak allergen”, the skin sensibility of pine pollen is getting increased in South Korea recently. The region and seasonal specific pollen data is essential for allergic patients. To provide fundamental airborne pollen data, we present the annual variation of pollen distribution and concentrations during spring in Korea for last four years.

**Methods**

We collected the airborne pollen from 10 representative sites in Korea using 7 days-Burkard sampler during 2011-2014 from February to May; Seoul, Guri, Daejeon, Daegu, Busan, Gangneung, Jeonju, Gwangju, and Jeju. Airborne pollen grains were counted and identified by using Light Microscope (x 200, x 400) after stained with Calberla’s fuchsin dye.

**Results**

The pollen distribution patterns were similar nationwide, however the calendar and distribution of pollen was different between inland and Jeju Island. Pine, Oak and Ginkgo trees were most frequent airborne pollen in Korea (67%-98%) with highest rate of pine pollen except Jeju Island. Japanese cedar restricted in Jeju and southern area of Korea (Kwangju and Busan) and also was the most common pollen in Jeju Island in spring. Interestingly, the pine pollen was continuously decreased in Jeju. It could be related with outbreak of pine wilt disease from 2010. The other background is the number of oak and ginkgo increased annually. The pine still is the most common pollen in South Korea so far. Otherwise, It is noteworthy that the concentration of ginkgo pollen is continuously increased at 6 sites; western area of Seoul, Jeonju, Busan, Daejeon, Gwangju, and Jeju. The high rate of Ginkgo pollen could be related with urbanization. Actually 41 % of street trees are Ginkgo in Seoul, Ginkgo pollen number at western pollen station of Seoul showed drastically increased from 611 grains/m^3^ (2012 year), to 5,527 grains/m^3^(2014 year). Meanwhile the other areas showed oak pollen increased as second highest rate.

**Conclusion**

Our results showed that the concentration of pollen could be affected by various factors like vegetation structure, a kind of street trees, endemic tree diseases, or artificial interference. This study may suggest 1) trees have physiological circadian change tri-or bi-annually. 2) Some city administration officially decided oak or ginkgo as street trees. 3) leaves of oak and gingko may intercept chasmogamy and breeding. The monitoring of airborne pollen on common and growing the species of trees likes pine, oak, and ginkgo is especially essential to demontrate the relationship between allergic sensitization rate and concentration of these pollen in Korea.

## A625 Effectiveness of Allergy-Test Directed Elimination Diets in Eosinophilic Esophagitis

### Jorge A Mazza^1^, Jason Kangeun Ko^2^, David JT Huang^1^

#### ^1^University of Western Ontario, Canada; ^2^Schulich School of Medicine & Dentistry

##### **Correspondence:** Jorge A Mazza – University of Western Ontario, Canada

**Background**

Currently there is no consensus on the optimal treatment of eosinophilic esophagitis (EoE). One of the options, an elimination diet guided by allergy test results, has been shown to be effective to varying degrees with greater efficacy in pediatric cohorts [1]. However, results in adults have been less promising as higher rates of negative allergy tests hinder efforts to guide the diet [2]. This study explores the results of allergy-test directed elimination diets in a group of patients (n = 16) treated at the Allergy & Immunology Clinic in London, Ontario.

**Methods**

A retrospective chart review was performed using a database of patients diagnosed with EoE (histology >=15 eo/hpf in esophageal biopsy) and treated at the Allergy & Immunology Clinic at St. Joseph’s Hospital, London, ON. Patients were included in the review if they had been prescribed an elimination diet guided by allergy testing (skin-prick test (SPT) and/or atopy patch test (APT)), and had esophageal biopsies taken >=8 weeks after initiation of the diet.

Pre-treatment eosinophil counts were recorded from peak eosinophil counts of the most recent biopsy prior to initiation of the elimination diet.

**Results**

The average age of subjects at diet initiation was 43.75 years. On average, each subject eliminated 1.7 foods from their diet. Post-diet biopsies displayed a significantly lower level of peak eosinophils/hpf compared to pre-diet biopsies on average (37.9 vs. 89.8, p = 0.043). Three patients had post-diet biopsies below the histological threshold of diagnosis for EoE (<15 eo/hpf).

**Conclusions**

This chart review shows that patients undergoing elimination diets directed by allergy tests have significant histological improvement after their diets. It must be noted that this review did not control for the effects of confounding treatments such as PPIs and corticosteroids, with the latter being of greater concern to histological results. Half of the subjects examined (8 out of 16) had been, at some time before the post-diet biopsy, prescribed swallowed corticosteroids for EoE. However, only 1/4 of steroid-prescribed subjects reported using the steroids during the 30-day period immediately prior to their post-diet biopsy.

**References**

1. Spergel JM, Brown-Whitehorn TF, Cianferoni A, Shuker M, Wang ML, Verma R, Liacouras CA: **Identification of causative foods in children with eosinophilic esophagitis treated with an elimination diet.***The Journal of allergy and clinical immunology* 2012, **130:**461–467 e465.

2. Vernon N, Shah S, Lehman E, Ghaffari G: **Comparison of atopic features between children and adults with eosinophilic esophagitis.***Allergy and asthma proceedings : the official journal of regional and state allergy societies* 2014, **35:**409–414.

## A626 Comparison of Cut-Off Values and Probability Curves for Egg Specific IgE in Diagnosis of Egg Allergy in Young Children

### Kanae Furuya, Keigo Kainuma, Takahiro Ito, Mizuho Nagao, Takao Fujisawa, Junya Hirayama, Yu Kuwahara

#### Allergy Center and Department of Clinical Research, Mie National Hospital, Japan

##### **Correspondence:** Kanae Furuya – Allergy Center and Department of Clinical Research, Mie National Hospital, Japan

**Background:** Numerous food specific IgE (sIgE) cut-off values and probability curves for OFC outcomes have been reported using the ImmunoCAP (CAP) assay, whereas, limited studies are available using the IMMULITE® 2000 3gAllergy™ (3g), a sIgE assay with an extended measurement range. In order to establish interpretation criteria for 3g, we prospectively analyzed the relationships between egg OFC outcomes and sIgE values using 3g and CAP for two specific needs: 1) diagnosis of egg allergy in infants who had never eaten eggs because of documented egg sensitization (group A), and 2) diagnosis of tolerance to egg in preschool children who had eliminated eggs due to confirmed egg allergy (group B).

**Methods:** A multicenter prospective observation study was performed and 433 children were enrolled, comprising of groups A (n = 220; 1 year old) and B (n = 213; 2-6 years old). OFC was performed with cooked egg powder for all the participants and with raw egg powder for patients who passed the initial cooked egg challenge. sIgE values to egg white (EW) and ovomucoid (OM) were measured by 3g and CAP. The relationship between the sIgE and OFC outcomes was analyzed using ROC and logistic regression analyses. Fitted predicted probability curves (PCs) were plotted using logistic regression.

**Results:** Out of 433 children, 243 (56%) passed cooked egg challenge. From the subset of 243 who passed the challenge, 113 children were given the raw egg challenge. Out of these 113 children, 55 (49%) passed the challenge. The two sIgE tests in the cooked egg challenge group showed similar performance, with area under the curve (AUC) of 0.791 and 0.778 in group A, 0.848 and 0.828 in group B, for OM sIgE with 3g and CAP, respectively. However, 3g showed higher cut-off values than CAP, at 21.5 IU_A_/L and 3.1 U_A_/L in group A and 45.4 IU_A_/L and 9.0 U_A_/L in group B, respectively. The PCs of EW and OM sIgE by 3g showed a wider distribution of data across the x-axis (sIgE values) and y-axis (probability) in comparison to CAP. In the raw egg challenge group, EW sIgE tests showed similar performance with AUCs at 0.678 and 0.671 in group A and 0.771 and 0.751 in group B with 3g and CAP, respectively. However, PCs for raw egg challenge showed significantly larger 95% confident intervals than those for cooked egg challenge.

**Conclusion:** Overall results from 3g demonstrated similar but slightly better performance than CAP in predicting the OFC outcomes.

## A627 A Case of Persistent Atopic Dermatitis Associated with Parasitic Infection

### Rosanna Qualizza^1^, Cristoforo Incorvaia^1^, Anna Maraschini^2^

#### ^1^Istituti Clinici Di Perfezionamento, Italy; ^2^Irccs Fondazione Ca’ Granda Ospedale Maggiore

##### **Correspondence:** Rosanna Qualizza – Istituti Clinici Di Perfezionamento, Italy

**Background** Infection from *Ascaris lumbricoides* may induce high level of IgE and favour the development of clinical allergy. We report the case of a child with atopic dermatitis and asthma cured by anti-helminthic therapy with mebendazole.

**Methods** The patient was a 12-year old female suffering from the age of two months from atopic dermatitis and from the age of 10 months from asthma. Both skin and respiratory symptoms were perennial, with worsening in spring and autumn. Allergy testing, performed at the age of 18 months, gave positive results to Dermatophagoides pteronyssinus (prick test +++; specific IgE 27 kU/L) and D. Farinae (prick test +++, specific IgE 23 kU/L). Also tomato (++), hen’s egg (++) and cow milk (+) were positive to prick tests, total IgE were 355 kU/L. A mild eosinophilia (7.2%) was detected in peripheral blood. Following environmental measures to control house dust mites and the elimination of tomato, egg and milk from the diet there was an improvement of asthma but not of atopic dermatitis. At 3 years of age the value of total IgE was 3253 kU/L. At 6 years of age there was a worsening of dermatitis with modest response to topical corticosteroids, while asthma was no longer present. Prick tests confirmed the positive results to *Dermatophagoides*, tomato and cow milk. A further worsening of atopic dermatitis occurred at 9 years of age, which was treated with oral bethametasone and topical picrolimus. In September 2014 the patient was referred to our Unit, we found a peripheral eosinophilia of 14.4% and, suspecting parasitic infections, we evaluated specific IgE to Ascaris, which had a value of 32.50 kU/L, while total IgE were 8324 kU/L. Anthelmintic therapy was prescribed using mebendazole (one100 mg 1 tablet b.i.d. for three days), repeated after 20 and 50 days.

**Results** One month after the first two cycles of therapy the patient showed a progressive improvement of symptoms, and eosinophilia was 12%. Six months after the end of therapy the skin was free from dermatitis and a decrease was observed for eosinophilia (10.1%) and Ascaris-specific IgE (27.50 kU/L).

**Conclusion** This case shows that the role of infection from *Ascaris lumbricoides* in patients with long-lasting dermatitis should not be overlooked. In fact, an adequate anti-helminthic treatment may result in a complete recovery from the disease.

## A628 Aeroallergenic Profile of Indoor Allergens and Their Clinical Relevance in Allergy and Asthma Patients in Saudi Arabia

### Syed Mohammed Hasnain^2^, Abdulrahman Al-Frayh^1^

#### ^1^King Khalid University Hospital; ^2^King Faisal Specialist Hospital and Research Centre, Saudi Arabia

##### **Correspondence:** Syed Mohammed Hasnain – King Faisal Specialist Hospital and Research Centre, Saudi Arabia

**Background:** Bronchial asthma and other respiratory allergic diseases are not only prevalent in Saudi Arabia and the Middle-East but the published data indicate that such diseases are on the rise. Investigations of extrinsic and intrinsic factors causing such diseases have been undertaken at different places in the Kingdom of Saudi Arabia. However, studies on the presence of indoor allergens and their clinical relevance still lack a clear understanding in the region.

**Methods:** House dust samples were collected in sterile ALK filters (ALK Laboratory, Denmark) and analyzed for various indoor allergenic factors including Der p 1, Der f 1, Bla g 1, Per a 1 and Fel d 1 using antibodies and ELISA technique. Indoor allergenic fungal flora was also examined by collection of samples and culture using dilution plate technique. The samples were collected through the individual’s own vacuum cleaner after the operation on their respective home carpets and mattresses. These samples were extracted individually and allergens were quantitated using specific antibodies from Indoor Biotechnologies (U.S.A) and ALK Laboratories (Denmark) by ELISA procedure. At some selected locations, Skin Prick Tests were conducted on allergic patients with various indoor allergens using both commercial and indigenous species.

**Results:** The data for the two house dust mites *Dermatophagoides pteronyssinus* (Der p) and *Dermatophagoides farinae* (Der f) showed a higher concentration in the humid areas of the country particularly in Jeddah with concentration exceeding threshold levels for both sensitization and exacerbation of acute attack asthma. The central region with very low humidity showed negligible presence of both mite species. The data from Jeddah and Abha showed variation in qualitative nature, one species being prevalent in Jeddah while the other being prevalent in Abha. The variation was also noted in the Skin Prick Testing in Taif. Data for *Blattella germanica* (Bla g 1 and Bla g 2), *Periplaneta americana* (Per a 1) and *Felis domesticus* (Fel d 1) showed quantitative regional variation as well.

**Conclusion:** The results obtained for both the prevalent indoor aeroallergens and their IgE Mediated Skin Prick Test reactivities indicate: (1). There are regional qualitative and quantitative variations in the appearance of these allergens, and (2). the prevalence of these allergens in the region are significantly relevant in the IgE sensitization of many patients and probably cause of the symptoms. Data from other countries in the Middle-East are either limited or none for any comparison. It is therefore recommended that a regional aeroallergenic profile should be prepared by conducting joint stuides with standardized protocol for better clinical diagnosis and treatment in the region.

## A629 Oral Exposure to the Amino Acid Glycine Inhibits the Onset of Allergic Disorders

### Anita Hartog^1,2^, Jacqueline Bastiaans^1^, Reinilde Loonstra^1^, Lieke Rutten^1^, Lucien Harthoorn^1^, Jeroen Van Bergenhenegouwen^1,2^, Johan Garssen^1,2^

#### ^1^Nutricia Research; ^2^Utrecht University, Netherlands

##### **Correspondence:** Anita Hartog – Nutricia Research

**Background:** The non-essential amino acid glycine (Gly) has been shown to act as an anti-inflammatory trigger in animal models of ischemic perfusion, post-operative inflammation, periodontal disease, arthritis and obesity (inflammation and Th1/Th17 models). Gly exerts its actions by binding to a glycine-gated chloride channel (GlyR) which has been demonstrated on neurons, as well as on immune cells (macrophages, polymorphonuclear neutrophils and lymphocytes). The present study aims to evaluate the effect of Gly on allergy development using an experimental model of cow’s milk allergy (Th2 model).

**Methods:** Female C3H/HeOuJ mice were supplemented with or without Gly by oral gavage (50 or 100 mg/mouse) 4 hours before sensitization with the cow’s milk protein whey, using cholera toxin as adjuvant. Acute allergic skin responses and systemic anaphylaxis were assessed after intradermal allergen challenge in the ear. Mouse mast cell protease-1 (mMCP-1) and whey specific IgE levels were assessed one hour after an oral allergen challenge.

**Results:** Intake of Gly significantly opposed allergy development in a concentration dependant manner as indicated by a reduction in; acute allergic skin response (63:40:30 μm whey-induced ear-swelling), anaphylaxis (127:107:89 AUC), serum mMCP-1 (1244:109:66 μg mMCP1/ml) and, serum levels of whey specific IgE (785:318:155 AU IgE/ml). All results are depicted in the following order; allergic control: 50 mg Gly: 100 mg Gly.

**Conclusion:** The present study indicates, for the first time, that oral intake of the free amino acid glycine protects against whey induced allergy development. Additional studies are warranted to elucidate the underlying mechanisms involved and to demonstrate effectiveness in humans.

## A630 Cough As a Key Symptom in Asthma, Allergic Rhinitis, COPD and Rhinosinusitis and Its Impact in Korea

### Kwang-Ha Yoo^16^, Sang-Heon Cho^1^, AG Ghoshal^2^, Abdul Razak Bin Abdul Muttalif^3^, Horng- Chyuan Lin^4^, Sanguansak Thanaviratananich^5^, Shalini Bagga^6^, Rab Faruqi^7^, Santwona Baidya^8^, Colman Taylor^8^, De Yun Wang^9^, Hae-Ryun Ahn^10^, Soon-Kwan Hong^11^, Jong-Woong Kim^12^, Gui-Hyun Nam^13^, Mee-Ja Kim^14^, Jae-Kyoung Park^15^

#### ^1^Seoul National University Hospital, South Korea; ^2^National Allergy Asthma Bronchitis Institute, India; ^3^Institute of Respiratory Medicine, Malaysia; ^4^Chang Gung Memorial Hospital, Taiwan; ^5^Khon Kaen University, Thailand; ^6^Merck & Co., Inc., USA; ^7^Merck & Co., Inc. (retired), USA; ^8^Optum, Australia; ^9^National University of Singapore, Singapore; ^10^Dami IM Clinic, South Korea; ^11^Coco ENT Clinic, South Korea; ^12^Jong-Woong Kim IM Clinic, South Korea; ^13^Hamchun Medical Clinic, South Korea; ^14^Myung ENT Clinic, South Korea; ^15^Seoulbom IM Clinic, South Korea; ^16^Konkuk University Medical Center, South Korea

##### **Correspondence:** Kwang-Ha Yoo – Konkuk University Medical Center, South Korea

**Background:** The Asia-Pacific Burden of Respiratory Disease (APBORD) study, was a cross-sectional, observational study conducted to examine the burden of disease in adults with allergic rhinitis (AR), asthma, chronic obstructive pulmonary disorder (COPD) and rhinosinusitis in six countries. The aim of this study was to report the results for Korea, including the percentage of patients receiving care for respiratory diseases and the frequency of presenting symptoms.

**Methods:** Patients aged ≥ 18 years, presenting to a physician with primary diagnosis of asthma, AR, COPD or rhinosinusitis were enrolled. Patients completed a survey which contained questions related to demographics, respiratory symptoms, healthcare resource use and quality of life.

**Results:** A total of 4,439 patients were screened, of whom 1,779 (40.1%) were eligible and 999 (56.2%) consented and were enrolled. The highest percentage of patients receiving care for a respiratory disorder had primary diagnosis of AR 12.5%, (95%CI: 11.6%, 13.5%), followed by asthma 7.2% (6.4%, 8.0%), rhinosinusitis 1.6% (1.2%, 2.0%) and COPD 1.3%, (1.0%, 1.6%). Patients were frequently diagnosed with multiple respiratory disorders (42.8%), with asthma/AR (23.2%) and AR/rhinosinusitis (15.4%) the most frequently diagnosed combinations – with or without other conditions. Among all symptoms reported, cough or coughing up phlegm was most frequently reported by participants with a primary diagnosis of asthma (68%), followed by rhinosinusitis (62%), COPD (59%), and AR (49%). In addition, cough or coughing up phlegm was the main reason for the medical visit for patients with a primary diagnosis of asthma and COPD whereas nasal symptoms (watery runny nose, blocked nose and congestion) were the main reasons for patients with a primary diagnosis of AR and rhinosinusitis.

**Conclusions:** Asthma, AR, COPD and rhinosinusitis represent a significant percentage of patients with respiratory disorders presenting to healthcare professionals in the Korea, with many patients presenting with concomitant disease. Timely identification of symptoms is important in implementing effective disease management, especially in patients with multiple respiratory diseases.

## A631 Cysteine Protease Allergen Def f 1 Induces Th2 Cytokines in Mouse Bone Marrow Derived Basophils Via ERK and JNK Dependent Pathways

### Myung-Hee Yi, Kyoung Yong Jeong, Ju-Yeong Kim, Tai-Soon Yong

#### Yonsei University College of Medicine, South Korea

##### **Correspondence:** Myung-Hee Yi – Yonsei University College of Medicine, South Korea

**Background**

Recent studies have shown that basophils contribute to the initiation of Th2 cytokine–mediated inflammation. However, the underlying mechanism by which protease allergens triggering basophils to produce Th2 cytokine remains unclear.

**Objectives**

The objective of this study is to demonstrate the role of the mitogen activated kinase (MAPK) family in mouse bone marrow-derived basophils (BMBs) when treated with cysteine protease allergen Der f 1.

**Methods** BMBs were selected as DX5+FceRI+c-Kit– by flow cytometry of the mouse bone marrow culture. Sorted BMBs were stimulated with proteolytically active recombinant Der f 1 expressed in the yeast *Pichia pastoris*. The expression levels of Th2 cytokines, IL-4 and IL-13, were examined by real time PCR and ELISA. The activities of various intracellular MAPK signaling components were assessed by FACS.

**Results**

Production of both IL-4 and IL-13 in mouse basophils was induced by the treatment of enzymatically active Der f 1, whereas inactive Der f 1 did not. Extracellular signal-regulated kinase (ERK) and c-Jun N-terminal kinases (JNK) were phosphorylated upon treatment of active Der f 1 on BMBs. Additionally, treatment of pharmacological inhibitors PD098059 (ERK inhibitor) and SP600125 (JNK inhibitor) were able to inhibit IL-4 and IL-13 secretion. However, p38 MAPK was not inhibited by its inhibitor (SB203580).

**Conclusions**

These data suggest involvement of ERK, and JNK MAPKs in signal pathway for the production of Th2 cytokines such as IL-4 and IL-13 in BMBs when treated with a cysteine protease allergen rDer f 1.

## A632 Novel Multiple Allergy Testing Kit Using Parallel Lines Array (PLA) Technology

### Bum Joon Kim^1^, Hs Joo^1^, Kj Lim^1^, Jae-Hyun Lee^2^, Jung-Won Park^2^, Kh Yoon^1^, DS Choi^1^

#### ^1^Proteometech Inc., South Korea; ^2^Division of Allergy and Immunology, Department of Internal Medicine, Yonsei University College of Medicine

##### **Correspondence:** Bum Joon Kim – Proteometech Inc., South Korea

**Background**

There are many kinds of *in vitro* allergy testing kits to be developed by various manufacturers and widely utilized for diagnosis of allergy. The *in vitro* testing kit for allergy measures the concentration of IgE in patient’s serum or plasma, which binds to allergens coated at the membrane support. Nitrocellulose membrane is usually used to immobilize many kinds of allergen. Since the line blot assay rather than dot blot assay has good repeatability, reproducibility and robustness, many types of *in vitro* allergy testing kit adopt the technology, so that many kinds of allergens are generally arrayed by 20 ~ 30 lines per test strip. However, the traditional line blot assay is difficult to increase the number of testing items because of its long-narrow structure, which is easy to spill-over of solution during the incubation of sample or reagents.

**Methods**

If the number of tested allergens should be increased, it can be achieved by 3 different options. One approach is to elongate the length of the strip without changing of gap between the lines. In this case, it must devise the solution to overcome the problem of spill-over. Another option is to coat the allergen more compactly in the equal-sized testing strip. But, if the bands in the testing strip go to be thinner, the robustness of assay would be diminished. The other possibility is to arrange the strips of two or more. It is a common way to be adopted by all the allergy testing products, where use two testing strips per single patient. However, we developed a novel technology, Parallel Lines Array (PLA), can test simultaneously up to 64 lines in a single strip.

**Results**

We produced 2 kinds of narrow strips of width of 1.95mm and then assembled the strips side by side in single allergy testing kit. The each strip has 25 or 32 lines to be coated with allergen or our standard, so we can measure 50 or 64 items in a single test. Since the Protia Allergy-Q use only one strip per single patient, rather than two strips per person at traditional allergy testing kits, it can save the testing time and blood volume. Furthermore, its diagnostic performance was compare to ImmunoCAP with 1,799 paired assay, which resulted in good concordance for most allergens (k=0.713-0.898, p<0.001).

**Conclusions**

The Protia Allergy-Q, adopting PLA technology, has comparable diagnostic performance to traditional multiple allergy testing kits.

## A633 Quantitative Rapid Kit for Human Immunoglobulin

### Hanseung Joo, Bum Joon Kim, Kj Lim, MJ Kim, DS Choi, Kh Yoon

#### Proteometech Inc., South Korea

##### **Correspondence:** Hanseung Joo – Proteometech Inc., South Korea

**Background**

The rapid diagnostic kit such as a pregnancy test is widely used. Samples of urine, serum and plasma are usually diluted with diluent buffer and separated by an immunochromatographic method. The analytes in the test can be immobilized to the nitrocellulose membrane which was coated with specific antibody or antigen and visualized by specific Ag or Ab conjugated with gold nanoparticles. However, their application is limited to the quantitative assay due to the Hook effect, which shows the false negative result in case of high concentrations of analyte in the sample.

**Methods**

To overcome the Hook effect, we devised an innovative rapid kit which has a unique competition method as well as a classical sandwich method in a single kit. In this study, test line 1 was prepared with mouse monoclonal anti-human IgG or anti-human IgE like classical rapid kits. Test line 2, a competitive line, which was coated with human IgG or human IgE, was designed to improve the false negative result due to the Hook effect. The control line was prepared with goat anti-mouse IgG. Goat anti-human IgG antibody and mouse IgG conjugated with gold nanoparticles were prepared in our laboratory and placed in the conjugate pad for further test.

**Results**

Immunecheck human IgG (Proteometech Inc, Seoul, Korea) can detect IgG range from 1.2 to 40,000 ug/ml. To know the degree of agreement against other quantitative assay method, we compared our assay results of 30 human sera with an immunoturbidimetric assay using Cobas Integra 800 (Roche, Indianapolis, IN, USA). This comparison showed that Immunecheck human IgG had 74.2 – 132.1% of degree of agreement and 0.9243 of coefficient of determination (R^2^) against an immunoturbidimetric method. The detection limit of Immunecheck human IgE was 80 IU/ml of total IgE and their signals were increased depending on the concentrations of IgE.

**Conclusions**

We developed a novel and innovative rapid immunoassay system which can overcome the hurdle of hook effect and detect the wide range of target molecule. This system will be beneficial to reduce the misleading of test results which cannot be possible with a current conventional immunoassay system.

## A634 Total IgE Measurement By Protia Allergy-Q: Comparison Study with Immunocap

### Bum Joon Kim^1^, Hanseung Joo^1^, Woo Sang Jung^2^, Kj Lim^1^, DS Choi^1^

#### ^1^Proteometech Inc., South Korea; ^2^Kyung Hee University

##### **Correspondence:** Bum Joon Kim – Proteometech Inc., South Korea

**Background**

The elevated total IgE, in spite of some well-known limitations, is frequently included as a diagnostic criterion for allergic diseases. There are many kinds of in vitro allergy diagnostic product to measure the concentration of total IgE. Because the multiple allergy screening kits, such as AlleryScreen® (Mediwiss Analytic GmbH and r-biopharm), Euroline (Euroimmun AG), Optigen® (Hitachi Chemical diagnostics, Inc) and Polycheck® (Biocheck GmbH), are low-cost test, their uses in clinic are rapidly growing nowadays. However, there are few comparison studies for total IgE level between multiple allergy screening kits and ImmunoCAP, a well-known quantitative diagnostic kit for allergy. The aim of this study is to compare the total IgE concentration in serum by Protia Allergy-Q, a multiple allergy screening kit, with ImmunoCAP and to evaluate the clinical usability of Protia Allergy-Q.

**Methods**

392 patients who visited the Hospital of Korean Medicine, Kyung Hee Medical Center were enrolled in this study. Informed consent was obtained from all patients. After five milliliters of the whole blood was collected in a vacuum tube, serum was separated by centrifugation at 3,000 rpm for 5 min, aliquoted into several round-bottom tubes and frozen at -80°C until use. ImmunoCAP was tested by Green Cross Laboratories (Yongin-si, Korea) and Protia Allergy-Q was performed by us. The correlation between two assays was analyzed by Passing-Bablok regression analysis.

**Results**

The average of total IgE from 392 sera measured by ImmunoCAP and Protia Allergy-Q was 167.1 and 149.4 kU/l, respectively. Passing-Bablok regression analysis demonstrated that ImmunoCAP and Protia Allergy-Q exhibited almost perfect correlation (intercept, 5.6676 [95% confidence interval {CI} 4.3282, 6.9388] and slope, 0.8676 [95% CI 0.8211, 0.9107]).

**Conclusions**

Protia Allergy-Q, a multiple allergy screening kit, is a low-cost screening test for patients with suspected allergy. It provides 60 kinds of specific IgE level but also total IgE level. Even though it is a semi-quantitative assay, total IgE concentration tested by Allergy-Q is nearly equal to that by ImmunoCAP. Therefore, it may be very convenient tool to measure not only specific IgE but total IgE concentration.

## A635 Prevalence and Risk Factors of Asthma Among Korean Farmers

### Ji-Hoon Lee, Soon-Chan Kwon, Soo-Jin Lee, Soo Yong Roh, Hogil Kim, Kyeong Joon Lee

#### Hanyang University College of Medicine, South Korea

##### **Correspondence:** Ji-Hoon Lee – Hanyang University College of Medicine, South Korea

**A) Background:** Asthma is considered as a common respiratory disease among farmers in many researches. In 13’ International Journal of Hygiene and Environmental Health, farmers showed higher prevalence (8.0%) than all other population (6.7%). But there is no large-scale research of the prevalence and current state of asthma among Korean farmers.

**B) Methods:** Structured questionnaire was used to evaluate the demographic characteristics such as age, sex, place of residence, smoking, prevalence of asthma based on doctor diagnosis and possible risk factors like crop types, farming period, pesticide usage, indoor cultivation, respiratory protection usage. From 2013 to 2014, total 951 farmers answered the questionnaire.

**C) Results:** Prevalence of asthma among male farmer was 5.2% and the prevalence among female farmer was 6.0%. Based on 2013 Korean national health and nutrition examination survey, prevalence of asthma among Korean male was 2.7% and the prevalence among female was 4.1%. The odds ratio of asthma for total pesticide use year (more than 20 years) was 2.07(1.20-3.58) and the odds ratio for indoor cultivation was 0.97(0.49-1.90).

**D) Conclusions:** The prevalence of asthma among farmers was higher than other population in Korea. Pesticide usage is considered as a risk factor of asthma. In further research, exposure measures and work-relatedness measures should be needed.

## A636 Dietary Intake and Perceived Immune Status in Young Dutch Women

### Aurora Van De Loo, Amanda Fernstrand, Johan Garssen, Joris Verster

#### Utrecht University, Netherlands

##### **Correspondence:** Aurora Van De Loo – Utrecht University, Netherlands

**Background:** Dietary intake can have a positive or negative impact on both actual and perceived general health and immune status. The purpose of this study was to examine the impact of dietary intake of a variety of nutrients on perceived immune status in young adult women.

**Methods:** A survey on dietary intake and perceived immune status was held among young Dutch women, aged between 18-30 years old. Perceived current immune status was scored on a scale ranging from 0 (very poor) to 10 (excellent). Subsequently, participants could indicate whether they perceived their immune status as normal or reduced. A food frequency questionnaire was completed recording past week food and beverage intake. From this data, the amount of various nutrients could be estimated, including fibers, sugar, tryptophan, beta-carotene, calcium, niacin, folate, thiamin, carbohydrates, riboflavin, vitamin A (retinol), vitamin B6, vitamin B12, vitamin C, vitamin D, and cholesterol. Non-parametric correlations (Spearman’s r) were used to examine the association between perceived heath, immune status and nutritients. Dietary intake of those with normal and reduced perceived immune status was compared using the Mann-Whitney U Test.

**Results:** N=329 young women (mean [SD] age: 20.7 [2.7]) completed the survey. Significant correlations were found between immune ratings and consumption of tea (r= 0.124, p=0.023), sugar (r= -0.173, p=0.002), and carbohydrates (r= -0.125, p=0.022). N=109 women (33.1%) reported having a reduced perceived immune status. Women with a perceived reduced immune status reported consuming significantly more alcohol (p=0.025), and had significantly higher dietary levels of cholesterol (p=0.022), sugar (p=0.044), and vitamin B12 (p=0.021) when compared to women reporting a normal immune status.

**Discussion:** The findings show that dietary intake is related to perceived immune status. However, some nutrients have a bigger impact on perceived immune status than others. Future studies, including objective measurement of immune biomarkers and nutrients, should confirm these findings.

## A637 The Effects of Antihistamine Drugs on on-Road Driving Performance

### Aurora Van De Loo, Johan Garssen, Joris Verster

#### Utrecht University, Netherlands

##### **Correspondence:** Aurora Van De Loo – Utrecht University, Netherlands

**Background:** Antihistamines can cross the blood-brain barrier and thus may cause drowsiness. As a result, daily activities such as driving a car may be impaired. The purpose of this review was to compare the effects of different antihistamines on driving performance.

**Methods:** PubMed and cross references were searched to identify double-blind placebo-controlled clinical trials. Studies were selected that used the standardized 100-km on-road highway driving test in normal traffic to examine driving performance. Patient studies were excluded. Subjects are instructed to maintain a steady lateral position and a constant speed (95 km/h). The Standard Deviation of Lateral Position (SDLP, cm), i.e. the weaving of the car, is the primary outcome measure of the test. The magnitude of driving impairment for antihistamine drugs was compared to SDLP increments seen at Blood Alcohol Concentration (BAC) 0.05% (ΔSDLP= +2.4 cm) and BAC 0.08% (ΔSDLP= +4.3 cm), i.e. the most common legal limits for driving.

**Results:** Eighteen studies were included. Regarding acute effects, impairment greater than BAC 0.08% was found after single dosages of diphenhydramine, emedastine, and hydroxyzine. Impairment after clemastine, triprolidine, mizolastine, acrivastine, dexchlorpheniramine, and mequitazine was comparable to BAC 0.05%. Results for cetirizine were mixed. No significant impairment was found for terfenadine, loratadine, levocetirizine, desloratadine, ebastine, bilastine, fexofenadine and rupatadine. Regarding sub-chronic effects (4-8 days of daily drug treatment), significant driving impairment was found for emedastine, diphenhydramine, clemastine, triprolidine, ebastine, and hydroxyzine. Mixed results were found for cetirizine, terfenadine and loratadine. No significant driving impairment was found for levocetirizine, acrivastine, fexofenadine, dexchlorpheniramine, bilastine, and mequitazine.

**Discussion:** Antihistamine drugs may significantly impair driving performance. Impairment is often seen with acute use of first- and second-generation antihistamines, and the magnitude of impairment is comparable to that seen at legal BAC limits for driving. Tolerance to the impairing effect after chronic daily use of antihistamines develops slowly. The newer antihistamines levocetirizine, fexofenadine and desloratadine did not significantly impair driving performance.

## A638 Grass Is Guilty: A Case of Anaphylactic Shock and Asthmatic Status in the Same Time in an Individual

### Jasmina Golez (jasmina.golez@gmail.com)

#### Ambulanta Meznar, Gregorciceva 5, Celje, Slovenia

Abstract

Anahylactic shock and astmatic status are both serious complications of allergic diseases that might have deadly outcome. As known in the literature, it’s very rare to occur together in the same time in one patient.

A 29-year-old-male athlete was introduced to our intensive care unit for experiencing anaphylactic shock and asthmatic status after running through grass fields near home. Upon arrival of emergency unit team, he had low blood preassure of 70/50 mmHg, and low oxygen saturation in 82% with altered mental status. After immediate application of epinephrine, prednisolone and salbutamol, his vital functions turned normal. He had mild asthma in childhood, but for the last 10 years, he had been asymptomatic without medication. For the recent 4 yeas, he had hay fever to grass pollen treated with intranasal glucocorticoid occasionaly and urticaria when exposed to almond and pork. Ten days before the reaction, he had an episode of hives while eating cake decorated with almond and after few minutes he had shortness of breathing, which was resolved on antihistamines. While testing sIgE, we found strong sensitization to grass and wheat, but not to insects or food he claimed to be eating.

He was prescribed a self-injectable epinephrine and asked to avoid running thrue grass fields.

We report a case of a male athlete who suffered from hypotension and asthmatic attack, provoked by grass polen.

**Keywords:** anaphylactic shock, asthmatic status, grass polen

Tel: 0038631315042

## A639 Cyclic Gamp-AMP(cGAMP) Induces Allergic Inflammation

### Koji Ozasa^1,2^, Etsushi Kuroda^3^, Ken Ishii^2,3^

#### ^1^Yokohama City University Graduate School of Medicine; ^2^National Institute of Biomedical Innovation, Health and Nutrition, Japan; ^3^WPI Immunology Frontier Research Center, Osaka University

##### **Correspondence:** Koji Ozasa – Yokohama City University Graduate School of Medicine

**Background**

It is known that respiratory viral infection in infancy cause asthma. But the detailed mechanisms underlying the induction of allergic inflammation by virus infection are is not fully understood. Many papers have shown that, not only pathogen-derived factors such as virus DNA and RNA, factors released from dying or stressed cells, damage-associated molecular patterns (DAMPs), are recognized by immune cells and contribute to inflammation. Interestingly, host DNA as a DAMP is known to induce type 2 immune responses and exacerbates allergic inflammation. Host DNA is recognized by intracellular DNA sensors and activates cyclic GMA-AMP synthase (cGAS). Then activated cGAS generates cyclic GMP-AMP(cGAMP) as a second messenger. We hypothesized that host DNA release from damaged cells by virus participates in allergic inflammation via the action of cGAMP. In this study, we investigated the effect of cGAMP on the onset of asthma.

**Methods**

House dust mite antigen (HDM) was administered intranasally to C57B/6J mice with or without cGAMP as an adjuvant, and then mice were challenged with intranasal administration of house dust mite antigen (HDM) on days 7, 9, 11, and 13. Twenty-four hours after last challenge, we collected blood, BALF, lungs. Serum antibodies were measured by ELISA, and cells in BALF were analyzed by FACS. We performed similar experiment using gene-knockout mice to evaluate the factors involved in this inflammation model.

**Result**

The number of eosinophils in BALF was significantly increased in mice sensitized by HDM with cGAMP. Histological analysis revealed that infiltration of inflammatory cells and mucus-containing goblet cells in the lung were also increased after the sensitization of HDM with cGAMP. Moreover cGAMP-adjuvanted HDM led to airway hyper-responsiveness. These effects were dependent on STING/TBK1, that are downstream factors of cGAMP signaling pathway.

**Conclusion**

cGAMP acts an adjuvant and induces allergic inflammation in the lung. These results suggest that cGAMP may be involved in viral infection-induced asthma through the release of DAMPs.

## A640 Role of Omalizumab in the Setting of Recalcitrate Dermatitis with Extremely Elevated IgE Levels

### Muhammad Imran, Selina Gierer, John Martinez

#### Kansas University Medical Center, USA

##### **Correspondence:** Muhammad Imran – Kansas University Medical Center, USA

**Introduction**

Omalizumab, the humanized recombinant monoclonal antibody against IgE, reduces serum free IgE and down-regulaties IgE receptors on mast cells and basophils. It is approved for the treatment of moderate to severe persistent asthma and chronic idiopathic urticarial. Bard *et al.* reported successful treatment of severe recalcitrant eczematous dermatitis in the setting of HIES with omalizumab. Due to high serum IgE level, recalcitrant dermatitis and prolonged usage of systemic corticosteroids, omalizumab was initiated in our patient with a favorable outcome.

**Methods**

A 54 year old man presented with a diffuse pruritic rash for 2 years. Physical examination revealed multiple discrete and confluent erythematous macules and plaques on the trunk, back, and all extremities. Laboratory findings showed extremely elevated IgE levels, 64285 IU/ML, absolute eosinophils 5,400 cells/μl, and a normal tryptase. Multiple skin biopsies showed non-specific dermatitis. Bone marrow biopsy, cytogenetics and imaging studies were unremarkable. The patient was evaluated by Dermatology and Hematology/Oncology, but no specific diagnosis was given. The patient denied a history of recurrent infections, skin abscesses, hyperextensible joints, bone fracture, or scoliosis. The patient had failed multiple systemic and topical medications, but improved with prednisone.

**Results**

The patient was treated empirically with omalizumab 300 mg every 28 days. One month after initiating omalizumab treatment, his dermatitis and eosinophilia markedly improved.

**Conclusions**

Although omalizumab was effective for our patient’s chronic dermatitis in the setting of severely elevated IgE, prospective studies and long term follow-up are required to confirm the efficacy of omalizumab in recalcitrate dermatitis and elevated IgE.

## A641 Follow-up Study on the Natural History of Prawn Allergy

### Lydia Wong^1^, Bee Wah Lee^2^, Gaik Chin Yap^2^, Genevieve Llanora^1^, Bernard Thong^3^, Lynette Shek^1^

#### ^1^National University Health System, Singapore; ^2^National University of Singapore; ^3^Tan Tock Seng Hospital

##### **Corresponding:** Lydia Wong – National University Health System, Singapore

**Background:** The natural history of prawn allergy is not well described. The aim of this study was to evaluate the natural history of prawn allergy in an Asian cohort.

**Methods:** From a previously described cohort of 56 prawn allergic patients (Thayalasingam, Clin Exp Allergy 2014), 17 patients agreed to be recruited. Six monthly questionnaires were administered and skin prick tests (SPT) to house dust mites (HDM): *Dermatophagoides pteronyssinus* (Dp), *Dermatophagoides farina* (Df), *Blomia tropicalis* (Bt), crude extracts to crab, prawn and custom made extracts of 3 prawn species (*Penaeus mono*don [tiger prawn], *Litopenaeus vannamei* [glass prawn], and *Panadalus borealis*[angka prawn]). Sera for IgE sensitization measured via ImmunoCAP to Dp, Df , Bt and Shrimp were also collected and compared to previous data from the same patients.

**Results:** Of the 17 patients, 2 had a history of anaphylaxis, 8 had a positive oral food challenge (OFC), 7 had a negative OFC at recruitment. Their mean age was 39 years (range:8 - 61 years) at recruitment. The duration of follow-up ranged from 34 to 76 months (mean 48 months). Sixteen (94%) (anaphylactic=2, OFC(+)=8, OFC(-)=6) continued to eat prawn and 14 (87.5%) still had ongoing allergic reactions. Two patients tolerated regular consumption of prawn, 1 of whom had a previous positive OFC.

Comparison of the clinical symptoms showed that the most common symptoms were urticaria (80%), throat itchiness (53.35%), generalized redness of skin (40%), nasal congestion (33.3%) and lip swelling (33.3%). There was a significant decrease in number of hospital visits for allergic reactions (at recruitment=29.4% to follow-up=0%, p<0.05).

The frequency of positive SPT to prawn extracts decreased from 11/17 subjects at recruitment compared to 7/17 subjects on follow up, but this was not statistically significant. There was no change in specific IgE levels to shrimp and dust mites except for a significant decrease in sIgE Df (p<0.05).

**Conclusion:** Prawn allergy is relatively longstanding. OFC negative subjects may continue to have allergic reactions on exposure, suggesting loss of tolerance or intermittent presence of co-factors lowering threshold for allergic reactions

## A642 Protein-Losing Dermopathy Impairing Growth in Children with Severe Atopic Dermatitis

### Mohammad S. Ehlayel^1,2,3^, Ashraf Soliman^2,4^

#### ^1^Hamad Medical Corporation, Qatar; ^2^Weill-Cornell Medical College; ^3^Section of Pediatric Allergy Immunology, Hamad Medical Corporation, Doha, Qatar; ^4^Section of Pediatric Endocrinology, Alexandria University, Alexandria, Egypt

##### **Correspondence:** Mohammad S. Ehlayel – Hamad Medical Corporation, Qatar

**Introduction:** Skin barrier defects play central role in the pathogenesis of atopic dermatitis (AD) affecting local immunity and skin hydration. Severe AD is seen in 1-15% of cases and its effects on growth and nutrition are not known.

**Objectives:** to 1) determine frequency and severity of hypoalbuminemia and hypoproteinemia in severe AD its relationship with AD severity 2) to study effect of hypoalbuminemia and hypoproteinemia on the growth of these children.

**Methods:** A retrospective study of 135 records of all children (<14 years) seen at Ped Allergy-Immunology clinics of Hamad General Hospital during Jun 2014-2015 with severe AD with serum albumin and protein tests. We also reviewed demographic data and lab. tests (CBC with differential, IgE, IgG, IgA, IgM, serum zinc, food allergens tests of SPT or specific IgE). SCORAD and anthropometric data were collected.

**Results:** They were 42 month old with 78 (57.8%) males and 57(42.2%) females. Other allergies were found in 40 patients (29.6%) and positive family history of allergy in 105 patients (77.8%). Majority (77%) tested positive to one food. SCORAD was 61.3 ±22.3, and body mass index (BMI) was 14.6%. WBC were 11,827 ±3,992.3 cells/ul, eosinophils 955±946.1 cells/ul, and total IgE 3,2598,708.6 ku/L. Hypoproteinemia was present in 78 patients (57.8%), and hypoalbuminemia in 56 patients (41.5%). 26 hypoalbuminemic patients had low BMI 11.2±2 % compared to 26 normoalbuminemic patients who had BMI 19.1±38.1%. Hypoproteinemic patients had BMI of 11.2±1.2% compared to normoproteinemic patients who had BMI 22.5±11.8%. SCORAD was higher in hypoalbuminemic-low-BMI patients compared to normoalbuminemic-normal-BMI patients (67.9±22.1 vs 58.3±22.5), and in hypoproteinemic-low-BMI patients compared to normoproteinemic-normal-BMI patients (73±21.1 vs 59.9±20.5)

**Conclusions:** In patients with severe atopic dermatitis, 58% of patients had hypoproteinemia and 42% hypoalbuminemia that were associated with low BMI denoting a significant effect on growth. These were related to AD severity. It is important to closely monitor growth, nutrition and biochemical makers in the management of severe AD.

## A643 Airways Assessment of Aged Nursing Homes Residents

### Pedro Martins^1,2^, João Marques^2^, Joana Gomes-Belo^2^, Teresa Palmeiro^1^, Iolanda Caires^1^, Joana Belo^3^, Maria Amália Botelho^1^, Paula Leiria-Pinto^1,2^, Nuno Neuparth^1,4^

#### ^1^Cedoc, NOVA Medical School / Faculdade De Ciências Médicas, Universidade Nova De Lisboa; ^2^Serviço De Imunoalergologia, Hospital De Dona Estefânia, Centro Hospitalar De Lisboa Central, Epe, Portugal; ^3^Escola Superior De Tecnologia Da Saúde De Lisboa, Instituto Politécnico De Lisbo0a, (ESTeSL-IPL); ^4^Serviço De Imuno

##### **Correspondence:** Pedro Martins – Cedoc, NOVA Medical School/Faculdade De Ciências Médicas, Universidade Nova De Lisboa

**Background:** Asthma is a common chronic respiratory disease that reaches 6.8% of the Portuguese population. It can be associated to serious health problems among persons over 65 years old. Even though, asthma is often undertreated or misdiagnosed in this age group. For this reason, spirometry could be important in the airways assessment of older adults.

**Methods:** Within the scope of the Phase II of the GERIA project 17 nursing homes from Lisbon were randomly selected. Every nursing home resident who met the inclusion criteria (to be at least 65 years old and to reside in the nursing home for more than 6 months) and did not present any contraindication for lung function tests was invited to answer a respiratory health questionnaire administered by an interviewer and to perform a spirometry. A descriptive analysis was carried out in order to assess the frequency of asthma, wheezing in the previous 12 months, chronic bronchitis and spirometric changes. Cohen´s kappa coefficient was used to evaluate the agreement between symptoms and spirometric parameters.

**Results:** The analysed sample included the 242 residents who performed a validated spirometry and a bronchodilator test. The majority (78.5%) were females with a mean age of 83 years (SD 7 years). Wheezing in the previous 12 months and asthma were reported by 38 (15.7%) and 22 (9.2%) residents, respectively. Chronic bronchitis symptoms occurred in 16 (6.6%). In the surveyed sample 71 (30%) residents presented a FEV_1_ < 80% and 90 (37%) a FEV_1_/FVC < 0.70, after bronchodilator. Twenty (8.3%) residents had a significant post-bronchodilator improvement of the FEV_1_(>12% and > 200 ml). No agreement was found between symptoms and spirometric parameters.

**Conclusions:** Spirometry seems to be an important tool to complement the questionnaire assessment in this age group as under-report of respiratory disease exists. Moreover it provides a better characterization of the obstructive lung diseases (PTDC/SAU-SAP/116563/2010).

## A644 Use of Skin Prick Test, Specific IgE to Shrimp and Rpen a1 to Determine Clinical Reaction to Shrimp in Area with High Prevalence of House Dust Mite Sensitization

### Narissara Suratannon^1^, Jaichat Mekaroonkamol^2^, Jarungchit Ngamphaiboon^1^, Piyawadee Lertchanaruengrith^2^, Pantipa Chatchatee^1^

#### ^1^Chulalongkorn University, Thailand; ^2^Chulalongkorn University Hospital

##### **Correspondence:** Narissara Suratannon – Chulalongkorn University, Thailand

**Background:** Shrimp is a widely consumed shellfish and the most common cause of anaphylaxis in Southeast Asia. Diagnosis of shrimp allergy using skin prick test(SPT) or specific IgE(sIgE) may be interfered by sensitization to house dust mite(HDM) which is also prevalent in the area. Sensitization to both allergens can occur due to cross reaction by tropomyosin.

**Objective:** In this study we sought to evaluate the roles of SPT to shrimp, sIgE to shrimp or component resolved diagnostic in the diagnosis of shrimp allergy in area with high prevalence of HDM sensitization.

**Methods**: Subjects with sensitization to shrimp and Dermatophagoides pteronyssinus (Derp) determined by SPT were enrolled from the allergy clinic. Shrimp allergy was diagnosed by oral food challenge test or convincing history of repeated allergic symptoms after shrimp ingestion or history of shrimp induced anaphylaxis. Mean wheal size of SPT to shrimp extract, cooked Pacific white shrimp, sIgE to Der p, shrimp and rPen a1 were determined.

**Results:** Fifty-one subjects were enrolled and classified into 2 groups according to shrimp reactions; shrimp allergy (n=22) and shrimp tolerance (n=29). Ages of shrimp allergic subjects ranged from 4 to 66 years, with 45% were male and 55 % were female. One-third of subjects with shrimp allergy had isolated oral itching symptoms while half of subjects had anaphylaxis. Median SPT wheal size to shrimp extract and fresh shrimp were significantly higher in shrimp allergic subjects compared to shrimp tolerance; 6.25 vs 4.25 mm, p=0.022 and 7 vs 4.5 mm, p=0.003 accordingly. SPT to shrimp extract >3.5 mm or positive shrimp sIgE(>0.35 kUA/) provided 90% sensitivity and negative predictive value (NPV) of 0.86. SPT to fresh shrimp provided better sensitivity and NPV (95% and 0.90) than shrimp extract. Using the cut of value of 0.35, sIgE to rPen a1 was found to have low sensitivity (45%) but high specificity (85%) and positive predictive value of 0.71 in identifying patients with shrimp allergy. Size of SPT, levels of shrimp sIgE and sIgE to rPena1 were not significantly difference between anaphylactic and non-anaphylactic subjects.

**Conclusions:** In subjects who demonstrated sensitization to both shrimp and HDM, sIgE to rPen a1 can be helpful in predicting clinical reaction while SPT and sIgE to shrimp are good screening tests. SPT to fresh shrimp had higher sensitivity and NPV than shrimp extract. SIgE to rPen a1 cannot be used to predict severity in our shrimp allergic population.

## A645 Identification of Specific IgE-Binding Proteins in Tree of Heaven (Ailanthus altissima) Pollen

### Gholamali Kardar^1^, Ahmad Majd^2^, Youcef Shahali^3^, Farrokh Ghahremaninejad^4^, Zahra Pourpak^1^, Fateme Mousavi^4^

#### ^1^Tehran University of Medical Sciences, Iran; ^2^Islamic Azad University, North Tehran Branch; ^3^Armand Trousseau Hospital; ^4^Kharazmi University

##### **Correspondence:** Gholamali Kardar – Tehran University of Medical Sciences, Iran

**Background:** The tree of heaven *has spread* from a prized ornamental plant to a highly invasive species in some regions worldwide. This tree was introduced in arid and semiarid regions of Iran as an ornamental species over the last two decades. Despite the increasing reports of *Ailanthus* spp. pollinosis, there have been very few molecular studies on this topic. The aim of this study was to identify potential allergenic proteins in *A. altissima*pollen using an animal model.

**Methods:***A. altissima* pollens were collected from urban green spaces of Tehran, Iran, during the pollination period from Mid-April to Mid-May 2014.The pollen proteins were extracted in phosphate-buffered saline (PBS). The protein content of *A. altissima* pollen (AP) extract was studied using the Bradford protein assay followed by a 10% SDS-PAGE. Thirty male BALB/c mice were randomly divided into two groups of AP extract sensitized and sham that received PBS. The AP extract was injected intraperitoneally at regular intervals. One week after the last injection, intradermal skin tests (ID) were performed on the abdomen skin of each animal. Specific IgE-binding proteins of AP extract were identified by immunoblotting using pooled sera of sensitized mice.

**Results:** The total protein concentration of AP extract was 4.47 μg per μl. The AP extract SDS-PAGE pattern revealed 17 protein bands ranging from 10 kD to 100 kD. The mean wheal diameter induced by ID was significantly higher in the AP extract sensitized group compared to the sham group (P=0.00). Immunoblotting using pooled sera of AP extract sensitized mice revealed a major IgE-binding component of approximately 42 KD and several minor IgE-binding proteins ranging from 12 to 37 kD.

**Conclusions:** In the present study, several AP extract IgE-binding proteins have been identified as a potential cause of immediate hypersensitivity reactions in sensitized subjects. These findings open up avenues for identification of AP extract allergenic proteins and its allergen-based diagnosis in patients suffering from allergic symptoms during the pollination period of this species.

## A646 Allergy Immunotherapy Well Tolerated in Children

### Mahnaz Sadeghi-Shabestari

#### Children Hospital, Immunology Research Center, Tabriz University of Medical Science, Iran

**Background**

Allergy immunotherapy use to treat allergies that was not effectively relieved by medications and recommended for all age .On the other hands, although allergy shots is usually safe but sometimes has side effects from localized swelling and erythema at the place where the shot given to anaphylaxis shock.

The aim of this study is comparison of reactions of allergy shots in adults and children.

**Methods**

In this study, side effects of allergy shots has compared in patients under and over 19 years. All patients got their shots on standard schedule (every week or every month) and stay at the office for about 20 minutes every time after get shot. In patients with late onset reactions to shots prospectus did by telephone.

**Results**

From all patients (121) who were gotten shot, 32 (26.4%) patients were under 20 years and 18(56%) patients were under 10 years. All patients had allergic rhinitis or allergic asthma isolated or in combination with other types of allergy.

In adult group, anaphylactic shock occurred in one patient. Eight attacks of generalized urticarial with wheezing, five attacks of generalized urticarial with abdominal pain and severe cramps observed.

In children group, we did not have any systemic reactions, only two cases had flushing, and two cases experienced headache after shot.

Sometimes local swelling and erythema occurred in place of shot in two groups of patients.

In addition, improvement in symptom control for disease in children was better than in adults.

**Conclusion**

Although it seems that tendency for allergy shot in children is less than adults, but reactions in children is less than adults and children better tolerate allergy immunotherapy.

## A647 Steinert (DM1) Patients Have IgG1 Deficiency and Should be Screened for Immune Deficiency

### K. Van Bilsen, O. Manusama, W.a. Dik, M. Van Der Burg, V. H. J. Van Der Velden, V.a.S. H. Dalm, P. M. Van Hagen

#### Erasmus MC, Netherlands

##### **Correspondence:** K. Van Bilsen – Erasmus MC, Netherlands

**Introduction**

Myotonic dystrophy type 1 (DM1) or Steinert’s disease, is a genetically determined disorder belonging to the group of muscle dystrophies. DM1 is mainly characterized by myotonia and progressive weakness and atrophy of skeletal muscles. Patients are heterogeneously affected by the disease varying from having only slight clinical signs to being fully handicapped. Pneumonia is an important primary and secondary cause of death in DM1 patients. Due to muscle weakness there is an increased susceptibility to mainly respiratory infections, however another factor may be hypogammaglobulinaemia as reported in 40-50% of DM1 patients. Remarkably to date no data can be found about the nature and impact of hypogammaglobulinemia on infection rate in these patients.

**Methods**

The immune status was evaluated and retrospective data analysis was performed of 17 DM1 patients. Standard screening comprised a questionnaire about (recurrent) infections, frequent use of antibiotics and hospital stays due to infections. Standard laboratory tests comprised serum IgA, IgM, IgG and IgG subclasses, NK, B and T cell counts, granulocyte, leucocyte and monocyte counts by FACS analysis. Also a vaccination response test was performed.

**Results**

The majority of the patients (71%) had hypogammaglobulinemia (IgG<7 g/L) ranging from 3.9 g/l and 6.5 g/l. Strikingly, IgG1 was selectively below normal range in fifteen out of sixteen patients (94%). None of the patients were on immune suppressive medication. The overall rate of pneumonia was 53%. Nine out of seventeen patients suffered from pneumonia at least once between January 2000 and October 2012, four of those patients had recurrent pneumonia or sinopulmonal infections. Other infections also occurred, e.g. recurrent urinary tract infection, recurrent facial herpes simplex and erysipelas. In the patients with hypoglobulinemia the rate of pneumonia was 58% compared to 40% in patients whose total IgG was normal (p = 0,52). Interestingly three patients with low IgG serum levels also showed insufficient vaccination responses for *H. influenzae* type B and/or *S. pneumoniae.* Two of which had recurrent infections, including pneumonia. These patients met the criteria for common variable immune deficiency (CVID).

**Discussion**

Our statistical data are limited due to the small number of patients included in this pilot study. However, the results are astounding with 94% having an IgG1 deficiency and 71% hypogammaglobulinemia. These results imply that screening for immune deficiency should be part of standard care for patients with DM1, however studies are warranted to investigate if IgG substitution therapy may lead to a decrease in infections and improving the quality of life.

## A648 The Change of Serous Sige and Three Evaluation before and after Sublingual Immunotherapy with Dermatophagoides Farinae for Persistent Allergic Rhinitis

### Yongping Liu

#### The Second Affiliated Hospital, Guangzhou Medical University, China

**Abstract Objective:** To investigate the efficacy of sublingual immunotherapy(SLIT) with dermatophagoides farinae for persistent allergic rhinitis coincidence between the symptoms improvement and the change of serous SIgE and SIgG. **Method:** Sixty-four patients of allergic rhinitis Who were divided into children group and adult group were treated with SLIT dermatophagoides farinae for 52 weeks, total nasal symptom score nasal sign score and VAS score were evaluated before and afte SLIT. **Result:** The three scores were descended significantly (P<0.05) after the treatment both two groups, but decline of SIgE and ascent of SIgG were not clear. **Conclusion:** The efficacy of SLIT with dermatophagoides farinae for persistent allergic rhinitis was prominent according to three scores, but it was not cohere between three scores and serous SIgE and SIgG.

## A649 Garlic Extracts Reduce Histamine-Induced Proliferation and Migration of Human Asthmatic Bronchial Smooth Muscle Cells

### Yi Yeong Jeong

#### Gyeongsang National University School of Medicine, South Korea

It is well-known that proliferation and migration of airway smooth muscle cells (ASMCs) play an important role in airway remodeling in asthma. Inflammation affects cell proliferation and migration, and antagonism of histamine receptors has been shown to be anti-inflammatory in asthma. This study was performed to identify whether and how garlic extracts show antagonistic effect on histamine-induced increase in proliferation and migration of human asthmatic bronchial smooth muscle cells (HABSMC). Treatment of HABSMCs with histamine (100 μM) significantly increased cell proliferation and migration compared to control (p < 0.05). Wound healing assays showed that histamine increases cell proliferation and migration in a time-dependent manner. Pretreatment with garlic extracts suppressed histamine-induced proliferation and migration by 50.1±3.7% and 45.8±6.9%, respectively. In addition, histamine-induced increase in proliferation and migration of HABSMCs was inhibited by pretreatment with MAPK inhibitors (PD98059, SB203580, and SP600125). Pretreatment with garlic extracts downregulated histamine-stimulated MAPK activation. These results show that garlic extracts reduce histamine-induced cell migration and proliferation by down-regulation of MAPK pathways and suggest that garlic extracts could be a potential asthma-preventive food.

## A650 A Case of Occupational Contact Dermatitis Caused By N-Acetylcysteine

### Ji Hye Kim, Moon Gyeong Yoon, Young Min Ye, Yoo Seob Shin, Ga Young Ban, Hae-Sim Park, Hye Min Jung

#### Ajou University School of Medicine, South Korea

##### **Correspondence:** Ji Hye Kim – Ajou University School of Medicine, South Korea

N-acetylcysteine(NAC) is widely used drug as a mucolytic in chronic respiratory diseases, an antidote in acetaminophen overdose and a protective agent for renal function in contrast-induced nephropathy. NAC is considered a well-tolerated and safe medication and no serious adverse reactions have been reported. Here we report a case of contact dermatitis to NAC.

A32-year-old woman presented with a symptoms of eczematous skin lesion on exposed area of hands, arms and face for several weeks. She was suffering with symptoms of her rash and itching sensation on the lesion. The patient was diagnosed as allergic rhinitis sensitized to house dust mite, but the symptoms were not bothersome. She had a previous history of drug allergy to non-steroid anti-inflammatory drugs. She was a nurse working in general ward of a tertiary hospital. Her symptoms had developed since she started working in the ward, mixing NAC and various antibiotics with normal saline. Serum total IgE level was not increased (69KU/L) and skin prick test revealed sensitization to various inhalant allergens including house dust mites. Serum specific IgE and skin prick test to antibiotics were negative. Intradermal test with NAC at 1mg/ml and 10mg/ml showed a positive result, both immediate and late reactions (after 48hours). Patch test using NAC also revealed a positive result after 96 hours. Serum specific IgE, IgG1 and IgG4 to NAC-human albumin conjugate were not detected by enzyme-linked immunosorbent assay. We report a case of allergic contact dermatitis due to NAC which was exposed in the workplace.

## A651 The Association Between Pollen Change and Asthma Attacks

### Soo Yong Roh, Jaechul Song, Ji-Hoon Lee, Hogil Kim, Jae Young Kim, Kyeong Joon Lee

#### Hanyang University College of Medicine, South Korea

##### **Correspondence:** Soo Yong Roh – Hanyang University College of Medicine, South Korea

**(1) Introduction**

There are pollen, temperature, air pollutants and other occupational factors as representative risk factors of allergic diseases. Among them, Pollen is an important role in the pathogenesis of asthma. Depending on global warming and climate change, recently changes in pollen is appeared. This study purpose is to survey the change of asthma from 2006 to 2010, to evaluate the relation between pollen and asthma attack and to compare the influence between Seong-Dong gu, Seoul as representative urban area, Guri, Gyungggido as suburban area, and Jeju as rural area.

**(2) Method**

We utilized a national health insurance data, meteorological data, air pollution data, and the pollen data of the National Weather Service from 2006 to 2010. Target area were Seong-dong gu which is one of 25 districts in Seoul, the capital of Korea, Guri which is similar to seong-dong gu in aspect of latitude and longitude, but is suburban area, and Jeju which is different to two areas of the front in aspect of latitude, is island, and is rural area. We considered The number of asthma attacks as the dependent variable. Asthma attack was defined if the case in that the oral steroid was used to treat asthma attacks was confirmed. In order to distinguish pediatric asthma, we restricted age range from age 18 to 70 years. Independent variables are meteorological variables(Tmax, Tmean, Tmin, precipitation, humidity, DTR(Diurnal Temperature Range), Wind velocity), Pollen data(The count of grass, ragweed, wormwood, Japanese hop, alder, birch, hazelnut and oak) and air pollutants data(atmospheric concentration of NO2, CO, SO2, PM10, O3. Statistical researches including time-series analysis, case-crossover design were performed.

**(3) Result**

Tree and ragweed pollen were associated with asthma attack; the largest magnitudes of association was with the 5-day average(RRIQR¼ 1.15, 95%CI 1.10–1.20). Grass pollen was only minimally associated with the outcome. The associations with ozone and PM10 were strongest on the same day (lag0) of asthma attack(RRIQR¼ 1.05, 95%CI 1.03–1.07 and RRIQR¼ 1.03, 95%CI 1.01–1.05), respectively, with a decreasing lag effect.

**(4) Conclusion**

The different pollen types showed different associations with the outcome. High levels of tree pollen appear to be an important risk factor in asthma exacerbations.

Geographically, there are differences in association between their risk factors and asthma attacks.

## A652 Incidence of Emergency Department Visits and Hospitalizations for Asthma Exacerbations during the Lunar Month in Singapore

### Lydia Wong, Mohana Rajakulendran, Haripriya Santhanam, Lynette Shek, Tow Keang Lim

#### National University Health System, Singapore

##### **Correspondence:** Lydia Wong – National University Health System, Singapore

**Background:** There have been anecdotal observations that there is a higher incidence of emergency visits and hospitalizations for asthma exacerbations during the lunar months in Singapore. This is postulated to be due increased outdoor air pollution during these months secondary to cultural practices of incense and joss paper burning. Incense burning is known to release particulate matter into the environment. This increases the risk of acute irritative symptoms as well as the prevalence of chronic respiratory symptoms^1,2^.

**Methods:** Data of emergency visits and hospitalizations for asthma exacerbations between January 2005 and December 2012 was collected from the Singapore National Asthma Programme database. This included data from the seven main government restructured hospitals in Singapore. Linear regression was performed using SPSS 22.0 to compare the significance of asthma exacerbations during the weeks of the lunar month compared to weeks of non-lunar months between 2005 and 2012.

**Results:** Results showed that there was a significant difference between lunar weeks and non-lunar weeks for the years 2005 and 2011. For 2005, the lunar weeks had significantly increased number of emergency visits and hospitalizations for asthma exacerbations (p= 0.028). However, in 2011 it was noted that the incidence was significantly reduced during the lunar weeks (p= 0.03). A combined analysis of all eight years also did not show any significant increase during the lunar weeks.

**Conclusion:** There is no significant increase in the incidence of emergency visits and hospitalizations for asthma exacerbations during the lunar months in Singapore.

**References**

1) Incense smoke: clinical, structural and molecular effects on airway disease. Ta-Chang Lin, Guha Krishnaswamy, David S Chi. Clinical and Molecular Allergy 2008, 6:3

2) Mechanism of asthmatic exacerbation by ambient air pollution particles. Ghio AJ. Expert Rev Respir Med. 2008 Feb;2(1):109-18.

## A653 The Prevalence of Positive Reaction for Skin Prick Test in Korean Farmers and Its Occupational Risk Factors

### Hogil Kim, Soo-Jin Lee, Ji-Hoon Lee, Soo Yong Roh, Soon-Chan Kwon

#### Hanyang University College of Medicine, South Korea

##### **Correspondence:** Hogil Kim – Hanyang University College of Medicine, South Korea

**Background:** Farmers are known to be exposed to a variety of antigens can cause allergic nature of the work environment. However, the industrial structure of Korea has changed with significantly reduced number of farmers evolved, as a result, out of the main concerns of the national public health policy and research is also rare. This study was performed to determine the status of their allergen sensitization rate and causes antigen of Korean domestic farmers.

**Methods:** A total of 1,143 people in rural area of Gyeonggi-do were enrolled in this study. Evaluations included a structured questionnaire and skin prick test with 15 inhalation allergens (including one each negative and positive controls). Statistical analysis was used as a multivariable logistic regression analysis to analyze the association of the positive prevalence and risk factors.

**Results:** Thirty people were excluded skin prick test results invalid, the proportion of positive reaction to at least one allergen rate is 18.1%. The most common sensitizing allergen is D.pteronyssinus(8.7%) and D.farinae(8.6%) and cockroaches, grass pollen mixture in order. Farmers who answered mainly cultivate flowers is the adjusted odds ratio of 5.63(95% CI 2.317-13.547) times more than rice and particularly high grass pollen mixture, cockroaches, mugwort, ragweed. In other risk factors, such as whether currently engaged in agriculture, pesticide use, indoor work we did not find statistically significant.

**Conclusions:** This study has its significance in that it can evaluate the health risks of occupational exposure of domestic farmers. But positive reaction to the skin prick test and presence of allergic disease is not equal, so it is necessary to interpret cautiously. We observed odds ratio appears high in the flower farmers and to show a different trend in the prevalence of crops and other inhalation allergens are thought to require further research in the future it.

## A654 Drug Allergy in Children: A Three-Years Experience at Dr. Kariadi Hospital Semarang Indonesia

### Ani Wistiani^1,2^, Galuh H^1^

#### ^1^Allergy and Immunology Division, Department of Child Health Faculty of Medicine Diponegoro University/dr. Kariadi Hospital, Semarang, Indonesia; ^2^Dr. Kariadi Hospital, Indonesia

##### **Correspondence:** Ani Wistiani – Dr. Kariadi Hospital, Indonesia

**Introduction**

Drug allergy (DA) is an immunologically mediated response to specific agent in a sensitized person. The clinical manifestations of DA are restricted to certain syndromes that are specifically accepted as allergic in nature, which may present as mild to life threatening reactions.

**Objective**

To determine the clinical features of DA.

**Methods**

Patient with clinical history of DA referred to Dr. Kariadi Hospital Semarang between January 2012 to May 2015, under the age of 18 years were retrospectively identified from medical records. Drug allergy was defined as one of drug-related cutaneous reaction pattern and systemic symptoms of an immunologically mediated drug hypersensitivity. Statistic analyses was fisher exact test with statistic significance p < 0.05.

**Results**

A total of 13 patients, 9 boys (69,2%) and 4 girls (30,8%) were identified. The mean age was 67±SD 54 months. The frequency of severe malnutrition and well-nourished were 3 (23,1%) and 10 (76.9%), respectively. All patients under 60 months of age with DA had neutrophil-lymphocyte count ratio (NLCR) below 6 (7 cases, 53,8%). The most common type was Fixed Drug Eruption (FDE) (23,07%). Other type were Steven-Johnson syndrome (SJS), erythema multiforme (EM) and drug-hypersensitive syndrome (DHS). Antibiotics accounted for more than 50% of all drug implicated in DA 8 cases (61,5%). Four (30,8%) had a previously history of allergy manifestation and only 2 (15,4%) patient with family history of allergy. Eosinophilia from blood smear examination was found in 6 cases of DA (46,2%). All patients with FDE showed normal test of liver function 3 (100%), while non FDE were 10 (40%) with p 0.067. There were no correlation between DA with skin exfoliation and anemia, and also with hypoalbuminaemia. Clinical features of skin exfoliation had no correlation with NLCR. All patient of DA except those with FDE received steroid therapy. Mortality in our study was 7,7%.

**Conclusion**

Fixed Drug Eruption was the most common type of DA found, with male predominantly affected and antibiotic was the drug most commonly involved.

## A655 The Identification of Morphology, Structure and Study of Seasonal Variation of Airborne Fraxinus Excelsior Pollen Grains in the Tehran

### Gholam Ali Kardar^1^, Maryam Sharifshoushtari^2^, Ahmad Majd^3^, Taher Nejadsattari^2^, Zahra Pourpak^1^, Mostafa Moin^1^

#### ^1^Immunology, Asthma and Allergy Research Institute, Children’s Medical Center, Tehran University of Medical Sciences, Iran, ^2^Science and Research Branch, Islamic Azad University, ^3^Islamic Azad University, North Tehran Branch

##### **Correspondence:** Gholam Ali Kardar – Immunology, Asthma and Allergy Research Institute, Children’s Medical Center, Tehran University of Medical Sciences, Iran

**Background:** European ash (*Fraxinus excelsior*) is a common cause of asthma, allergic rhinitis and allergic conjunctivitis. Therefore, it is essential to study the period and duration of the flowering in airborne in each region/area. In this research, was to evaluate the relation between ash pollen counts with meteorological and air pollution factors in Tehran for six years. **Methods:** At the First, the districts of Tehran were classified as northern, southern, eastern, western and central regions and the near of Tehran is the control zone. Pollen samples were collected from *F. excelsior* trees planted in the areas of Tehran and also in non-polluted area Situated outside the city. Scanning electron microscopy was used to examine and compare shapes and structures of non-polluted and polluted pollens. An attempt was made to pollen counts determined by the two methods (volumetric7-day spore trap and gravimetric durham method) during six years (the Jan2010 to jun2015). Airborne ash Pollens were collected on a slide for 24 hour period and the slides were stained and examined under microscope. The relationship between the ash pollen counts, and the meteorological parameters including the mean values of high/ low rates of temperature, sunshine hours, high/ low relative humidity, wind speed and raining was investigated. Also air pollution data, such as Co, NO2, SO2, O3, and PM10 and PM2.5 were all obtained from the five study areas in Tehran for finding their relation by grass pollens count. **Results:** Our microscopic examinations showed that air pollution considerably improved the fragility of pollen exine, causing numerous cracks in its surface and facilitating pollen content liberation. A pollen calendar of ash pollen types was identified in the six years study period areas of Tehran. Pollen production was higher in the south and center area and was lower in the other areas of Tehran. Major pollen seasons were recognized Jan to April. Highest pollen counts were obtained in March and lowest in the Jan. The ash pollen calendar and its association with meteorological and air pollution factors related mainly to daily temperature, sunshine hours, wind speed and Co, NO2 and PM2.5. **Conclusions:** The ash pollens seasonal variation that is specific for each area in each country can help allergic patients for decision in their life in every day and for physicians to attend allergic disease management. So we hope these data be use full for patients and physicians in Tehran (capital of Iran). Finally, the results showed pollutant can be a cause of extending the period of pollination.

## A656 Sublingual Immunotherapy in Elderly Rhinitis Patients Sensitized to House Dust Mites

### Ji Hye Kim^1^, Daehong Seo^2^, Young Min Ye^1^, Hae-Sim Park^1^, Jung-Won Park^3^, Jae-Hyun Lee^3^, Yoo Seob Shin^1^

#### ^1^Ajou University School of Medicine, South Korea, ^2^Chungmu Hospital, ^3^Division of Allergy and Immunology, Department of Internal Medicine, Yonsei University College of Medicine

##### **Correspondence:** Ji Hye Kim – Ajou University School of Medicine, South Korea

**Background/Aims:** Allergic rhinitis (AR) in the elderly patients is constantly increasing with the extension of life expectancy, but little is known about elderly AR. Immunotherapy in the elderly patients is a controversial issue since no proven evidence in the safety and efficacy of this treatment had been shown. In this study, we were to find the characteristics of elderly AR sensitized to house dust mite (HDM) and evaluate the safety and efficacy of sublingual allergen-specific immunotherapy.

**Methods**

Total 21 patients older than 60 years old with AR sensitized to HDM with A/H ratio of >3 on skin prick test and/or >0.35IU/L in ImmunoCAP to *D.farinae* and *D.pteronyssinus* were enrolled in this study. The patients were randomized to either medication group with LAIS® Mites Sublingual tablets (2/week) or observation group using a double-blind method in their first visit and their symptoms and adverse reactions were monitored for 3 months.

**Results**

Of 21 patients, 13 (61.9%) patients were in the medication group and 8(38.1%) patients were in the observation group. Regardless of the groups, the mean age was 67.2 years, the ratio of male to female patients was 11(52.4%) to 10 (47.6%). The mean of total IgE was 601.1IU/L and specific IgE to *D.farinae* and *D.pteronyssinus* were 4.8IU/L and 11.72IU/L, respectively. The baseline mean value of total rhino-conjunctivitis symptom score, which is a primary endpoint of this study, was 22.09, which tended to decrease during 3 months. Serious adverse events have not been reported, and the patients are well tolerated with LAIS®.

**Conclusions**

Sublingual immunotherapy with tablet form in elderly AR patients sensitized to HDM is worth trying and no safety issue has become a problem, but further investigation results are needed.

This study was supported by Lofarma SpA, Milano, Italy.

## A657 Healthy Ageing Research Center (HARC) As a Platform for Multidisciplinary Approaches to Respiratory Research in the Elderly

### Marek L. Kowalski, Aleksandra Wardzynska, Marcin Kurowski, Malgorzata/Ewa Pawelczyk, Adam Wysokinski, Iwona Kloszewska, Janina Grzegorczyk, Wojciech Piotrowski, Joanna Makowska

#### Medical University of Lodz, Poland

##### **Correspondence:** Marek L. Kowalski – Medical University of Lodz, Poland

**Background:** World population, over the age 65 (usually defined as the elderly) has been steadily growing, reflecting general trends of ageing societies. With advancing age, chronic diseases become increasingly prevalent and contribute to the loss of independence, frailty and increased risk of death. Understanding the role of environmental factors, specifically respiratory infections, in the pathogenesis of chronic respiratory diseases in the elderly may help in decreasing morbidity and extending the lifespan of this population.

**Methods:** Healthy Ageing Research Center (HARC) which is a multidisciplinary research platform established at the Medical University of Lodz, involves research groups with various basic biological (immunologists, biochemists, molecular biologists) and clinical (geriatricians, psychiatrists, cardiologists and pulmonologists) interests and devoted to conduct research addressing key issues related to pathogenesis of increased morbidity in the elderly population. Here we present the HARC platform as a vehicle for development of multidisciplinary research related to various aspects of respiratory infections in the elderly.

**Results:** Preliminary study involving elderly subjects (n=157; mean age 68.2) allowed to establish risk factors associated with frequent infections. In a multivariate analysis polytherapy (more than 5 prescription drugs) has been determined as one of the most important risk factors for frequent (defined as more than 3 per year) respiratory infections (OR=1.93 (CI95% 1.11-3.36). The effect of comorbidities as risk factors for respiratory infections, will be further analyzed in cross-sectional study involving 3000 subjects randomly selected from the local population of elderly subjects. In collaboration with psychiatrics the prevalence and spectrum of respiratory infections among patients with various psychiatric disorders is being studied.

Although viral infections are considered to be important triggers in asthma exacerbation in children and adults, it is difficult to extrapolate infection rates and specific pathogens from these studies to older adults with asthma. The study currently developed with microbiologist is aiming at assessment of the role of viral and bacterial triggers asthma exacerbation in the elderly patients. In parallel, in vitro model to assess the peripheral blood leukocytes immune response to rhinovirus infection and miRNA expression in the elderly patients with and without asthma has been introduced .

**Conclusions**: Collaboration within multidisciplinary research team ( HARC platform) has allowed for development of innovative approaches to study various aspects of respiratory infections in the elderly population.

Project is supported by the European Commission RegPot-2012-2013-1.

## A658 Estimation of Cases of Work-Related Asthma Using Capture-Recapture Methods

### Soon-Chan Kwon^1^, Jaechul Song^1^, Yong-Kyu Kim^2^

#### ^1^Hanyang University College of Medicine, South Korea; ^2^Daejeon Sun Hospital

##### **Correspondence:** Soon-Chan Kwon – Hanyang University College of Medicine, South Korea

**Background:** Capture-recapture models allow one to estimate the number of actual cases by assessing the probability of overlap between cases from multiple sources. The Korea Work-Related Asthma Surveillance (KOWAS) is a scheme designed to collect information on WRA cases from multiple reporting sources, which are the Korea Workers’ Compensation and Welfare Service, occupational physicians, allergy and chest physicians, and regional work-related disease surveillance systems. The purpose of this study is to estimate the number of actual cases of WRA using the capture-recapture analysis.

**Methods:** Capture-recapture analysis was used to obtain a nearly unbiased estimator (NUE) of the total number of WRA cases reported to KOWAS from 2004 to 2006. To do this, the 4 reporting sources were stratified into 2 categories as follows: (1) the workers’ compensation scheme (*i.e.* the Korea Workers’ Compensation and Welfare Service), and (2) the other 3 reporting sources (*i.e.* physicians’ reports). Capture-recapture analysis was performed on sex, regions and specific industries when the number of overlapping reports was ≥7. Overlapping reports, i.e. duplicate cases, were defined as individuals who had identical names, dates of birth, resident registration numbers, and employers found in both reporting categories.

**Results:** From 2004 to 2006, a total of 172 cases of WRA were reported by 4 reporting sources : 62 (48.8%) by workers’ compensation schemes, 31 (24.4%) by occupational physicians, 68 (53.5%) by allergy and chest physicians, and 50 (39.4%) by regional surveillance systems. The number of cases estimated by capture-recapture method was 186 (95% CI: 150.75-221.43). The completeness of worker’s compensation and physicians’ reports were 0.52 and 0.33 respectively. By industry, the reported cases and estimated cases of the manufacturing sector were 189 and 333 (95% CI : 262.62-404.27). By region, the reported cases and estimated cases in Gyeonggi and Incheon were 75 and 114 (95% CI : 83.75-143.69).

**Conclusions:** Capture-recapture models allow the estimation of the true number of cases and the assessment of the completeness of our surveillance data. And this method can be useful in a various public health areas.

## A659 Primary School Students’ Parents Reported ISAAC Questionnaire in a Low Income Area of Ankara

### Ilknur Bostanci, Zeynep Sengul Emeksiz, Aysegul Ertugrul, Serap Ozmen, Soner Sahin

#### Dr. Sami Ulus Obstetrics, Children’s Health and Diseases Training and Research Hospital, Turkey

##### **Correspondence:** Ilknur Bostanci – Dr. Sami Ulus Obstetrics, Children’s Health and Diseases Training and Research Hospital, Turkey

**BACKGROUND**

Asthma is one of the most important chronic diseases of childhood. Parents’ awareness about asthma symptoms and knowledge of the asthma triggers are necessary for every step of diagnosis and treatment.

**Methods**

In this study, 85 primary school students’ parents leaving in a low-income district of Ankara were included. A questionnaire prepared 38 questions about symptoms and a trigger of asthma based on ISAAC (International Study of Asthma and Allergies in Childhood) was applied to the parents. P value was 0.05.

**Results**

Primary school students’ mean age was 9.1±0.9 year (min-max 7-11). Wheezing attack was recognized by parents in 10 of 85 children (11.7%). 9of 85 children (10.5%) were diagnosed as asthma by a medical doctor previously. 55 of 85 children (64.7%) had at least one smoker in their house. 14 of 85 parents (16.7%) defined humidity and 15 of them (17.6%) defined mold in their house.

**Conclusion**

Our study showed that, 11.7% of primary school students had wheezing. Indoor triggers such as humidity, mold and cigarette smoke were reported by the students’ parents. So especially in low income areas it is very important to educate the parents about indoor triggers.

## A660 Usefulness of PC20 Adenosine Monophosphate in Diagnosis and Treatment in Bronchial Asthma

### Sang-Ha Kim, Myoung Kyu Lee, Won Yeon Lee, Suk Joong Yong, Seok Jeong Lee, Ye-Ryung Jung

#### Yonsei University Wonju College of Medicine, South Korea

##### **Correspondence:** Sang-Ha Kim – Yonsei University Wonju College of Medicine, South Korea

**Background:** Airway hyperresponsiveness and airway inflammation are the characteristic features in bronchial asthma. Airway hyperresponsiveness is usually measured with direct stimuli such as methacholine or histamine. Adenosine 5’-monophosphate, which acts indirectly via the secondary release of mediators, is another stimulus to measure brochial hyperresponsiveness. The aim of this study was to investigate the relationship among the provocative concentration of methacholine producing a 20% decline in FEV1 (PC20 methacholine), PC20 adenosine and inflammatory markers in newly diagnosed asthmatics and to compare the changes in each after inhaled corticosteroid treatment. **Methods:** We performed a prospective analysis of data from 20 patients with a diagnosis of persistent bronchial asthma (17-67 years, FEV1>60% predicted). Patients were assigned into two groups according to severity of the disease of either mild (mean FEV1 91.6±4.7%, n=12) or moderate(mean FEV1 62.9±2.9%, n=8). Methacholine and adenosine bronchial provocation test, sputum induction, blood sampling were performed before and after treatment with corticosteroid for 4weeks. **Results:** The sensitivity of methacholine and adenosine bronchial provocation test in diagnosis of bronchial asthma are 65% and 60%, respectively. Both PC20 methacholine and PC20 adenosine are improved after corticosteroid treatment. Devided into two groups according to severity, in mild group, improvement in PC20 methacholine (p<0.05) after corticosteroid treatment was found but not in PC20 adenosine. In moderate group, improvement in PC20 adenosine (p<0.05) after corticosteroid treatment was found but not in PC20 methacholine. Pre-treatment (pre-) MMP-9 was correlated with PC20 methacholine and PC20 AMP (p<0.05; p<0.05). Strong correlations between pre- IL-8 and pre- MMP-9 (Rho=0.655, p<0.05) and between pre- IL-8 and pre- TIMP-1 (Rho=0.815, p<0.001) was observed. We found that changes in PC20 methacholine and PC20 adenosine after treatment with inhaled corticosteroid are correlated with eosinophil percentage in blood and pre- TIMP-1 in sputum, respectively (Rho=0.488, p<0.05; Rho=0.464, <0.05). All inflammatory markers such as IL-5, IL-8, MMP-9, TIMP-1 and ECP decreased after treatment but no significance was found. **Conclusions:** Our findings show that PC20 adenosine and PC20 methacholine are useful markers to assess the efficacy of antiinflammatory therapy. In moderate persistent asthma, PC20 adenosine may be more valuable to evaluate the efficacy of treatment than PC20 methacholine.

## A661 S100 Calcium Binding Protein A9 in Sputum of Patients with Steroid Naive Asthma: Relation with Airway Obstruction and Nneutrophilc Inflammation

### Myung Shin Kim^1^, Jong-Sook Park^2^, An-Soo Jang^2^, Choon-Sik Park^3^

#### ^1^Soonchunhyang University Gumi Hospital, South Korea; ^2^Soonchunhyang University Bucheon Hospital; ^3^Soonchunhyang University Hospital

##### **Correspondence:** Myung Shin Kim–Soonchunhyang University Gumi Hospital, South Korea

***Background*:** We previously reported elevation of S100 calcium bindingprotein A9 (S100A9) protein in sputum of neutrophilic severe uncontrolled asthmatics in spite of high dose inhaled corticosteroid treatment compared with stable asthmatics. The aim of this study was to evaluate the relation of S100A9 protein with neutrophilic inflammation in the steroid naive - mild to severe asthmatics and lung function. ***Materials and Methods*:** Sputum were obtained from 132 never-smoking or ex-smoking asthmatics (<10 pack-years). S100A9 proteins were measured in inhaled or systemic corticosteroid naïve asthmatic (SNBA, n=103), inhaled and systemic steroid treated asthmatic (STBA, n=29) and normal controls (NC, n=35) using an enzyme-linked immunosorbent assay. Correlations between S100A9 levels and inflammatory cells, FEV1, and annual rate of exacerbation were analyzed. ***Results*:** The S100A9 levels were significantly higher in sputum of both SNBA and STNA than that of normal controls (P=0.008, and 0.001 respectively) and were comparable between SNBA and STNA (P=0.734). In bronchial asthma (BA), S100A9 levels were significantly correlated with the percentages of neutrophils (r=0.267, p=0.002) and those of eosinophils (r= -0.195, p=0.025) in sputum. The correlations were persistently observed in SNBA. When BA was divided into 4 groups: Neutrophil dominant (70% or more), eosinophil dominant (3% or more), co-dominant and pauci-granulocytic group. The S100A9 levels was the highest in the neutrophilic group (13.51 pg/ug) compared to the eosinophilic (9.06 pg/ug), co-dominant (5.96 pg/ug) and pauci-granulocytic (7.87 pg/ug) groups. S100A9 levels was significantly increased in the subject experiencing exacerbation compared to those not experiencing exacerbation. ***Conclusion*:** Our data suggest that S100A9 protein was elevated in sputum of asthma compared to normal control. The levels were correlated with percentage of neutrophil in sputum and were regardless of steroid treatment.

## A662 Risk Factor Asthma in Pediatric Pneumonia Patients

### Diah Asri Wulandari, Cissy Kartasasmita

#### Hasan Sadikin General Hospital, Indonesia

##### **Correspondence:** Diah Asri Wulandari – Hasan Sadikin General Hospital, Indonesia

**Abstract**

**Background**: asthma is a risk factor of pneumonia in children. Children with asthma are 40% more likely to have pneumonia, and need hospitalization especially children under five years old. Asthma in pediatric pneumonia has more severe clinical symptoms with increased need of mechanical ventilation and mortality.

**Objective:** To comprehend asthma risk and the profile of children with pneumonia in Hasan Sadikin General Hospital Bandung-Indonesia.

**Method:** Retrospective study on pediatric pneumonia with asthma as a comorbidity at Pediatric Ward Department Hasan Sadikin General Hospital from January to December 2012. We include all patient hospitalized with pneumonia and asthma using ICD 10 (J11-J18 and J 45).

**Results:** Eight hundred and seventy three children with pneumonia were admitted to the hospital. Seven hundred and thirty six patients (84.3%) had comorbidities, and thirty six patients (4.1%) had asthma. Twenty pneumonia patients with asthma (2.3%), 1 patient (0.1%) had asthma and congenital heart disease, and 15 patients (1.5%) had asthma and other comorbid such as cerebral palsy, anemia, malnutrition, diarrhea, and pulmonary tuberculosis. Median age was 42.5 months (11 to 144 months old), with 22 of them (61.1%) were under five years old, and 22 patients were male (61,1%). Six patients didn’t have history of allergy/asthma in their family, and 4 patients had one asthma exacerbation in last 4 weeks. Six patients had previous hospitalization with similar symptoms. One of them received previous controller medication. Laboratories finding revealed white blood cell 5500-24.500/mm^3^, only 1 bacteria was found from blood culture which was *Serratia marcescens*, and chest X-ray shows bilateral infiltrate. Eight patients (22.2%) had severe asthma exacerbation. Length of stay was 1 to 8 days. There was no patient admitted to pediatric intensive care unit or perished.

**Conclusion:** Asthma was still a risk factor for children to developing pneumonia that required hospitalization.

## A663 Prevalence and Risk Factors of Childhood Asthma and Allergic Disease at Exposed Area By Emission of Cement Padang Factory

### Finny Fitry Yani^1^, Rizanda Machmud^2^, Dhina Lydia Lestari^1^

#### ^1^Dr. M. Djamil Hospital, Indonesia; ^2^Faculty of Medicine Andalas University

##### **Correspondence:** Finny Fitry Yani – Dr. M. Djamil Hospital, Indonesia

**Background.** Prevalence of asthma in children is increasing, especially in the industrial city. Padang city has Padang cement factory which is located near from community where people surrounding can inhale the dust pollutant emission from cement production, including children. We want to know the prevalence and risk factor of asthma and allergic disease in this area.

**Methods.** During May-June 2015, we conduct a cross sectional study to children around the Cement Padang factory, based on distance of house from the factory, <5 km (exposed area) and > 10 km (no exposed area). Children age from 0-15 years were selected. We used ISAAC questionnaire to determine asthma and allergic disease. Data was taken from the parent, with informed consent, including asthma, allergic rhinitis and atopic dermatitis symptoms and some risk factors. Data was analyzed with chi-square test.

**Results.** This study found 90 children, 43 (47.8%) from exposed area, most of them were female 46 (51.1%). The most age group is 1-5^th^(48.8%). More than half (54%) have normal nutritional status, but 33.3% undernourish. Asthma prevalence and atopic dermatitis was found higher in no exposed area than exposed area (19.5% vs 13.9% and 39.1% vs 37.2%), but allergic rhinitis was higher in exposed area (60.4% vs 58.6%), no significance difference. Atopic history of the mother was higher in exposed area (34.8% vs 17.3%). Other inhaled pollutan like pet, cigarretes smoke, kapok mattress, vapor from gasoline were higher in no exposed area, but no significance difference.

**Conclusion.** In exposed area of Cement Padang factory, the prevalence of rhinitis allergy was higher than asthma and atopic dermatitis, but no significance difference.

## A664 Association Between Serum Level of 25-Hydroxyvitamin D with Atopic Dermatitis Occurrence and Severity in Children

### Rusdi Rusdi, Yurmalina Yurmalina, Eryati Darwin

#### Rsup Dr.M.Djamil/Faculty of Medicine Andalas University, Indonesia

##### **Correspondence:** Rusdi Rusdi – Rsup Dr.M.Djamil/Faculty of Medicine Andalas University, Indonesia

**Abstract**

**Background** Atopic dermatitis (AD) mostly frequent occurred in infant and children. Intrinsic and extrinsic factor are important role in AD. Epidermal barrier injury and dysregulatory of immune system could be induced by vitamin D. The aim of study was to reveal relation in between of serum level of 25-hydroxyvitamin D with atopic dermatitis occurrence and severity in children.

**Method** This study was cross sectional study to 21 AD patient and 21 control. Serum level of 25-hydroxyvitamin D was measured by enzyme-linked immunosorbent assay (ELISA) and evaluating AD severity by using Severity Scoring of Atopic Dermatitis SCORAD.

**Result** Acquired deficiency of 25-hydroxyvitamin D in 20 AD patient (95,2%), Otherwise control study was obtained 57,1%. Median rate 25-hydroxyvitamin D in AD patient is lower than control study 4,977 (0,009-23,672) ng/mL and 14,374 (0,256-91,711) ng/mL, p=0,002. Median rate 25-hydroxyvitamin D in mild, moderate and severe AD patients are 4,262 (0,009-23,672) ng/mL, 4,262 (0,009-23,672) ng/mL and 2,336 (0,058-9,780) ng/mL.

**Conclusion** serum level of 25-hydroxyvitamin D in AD patient is lower significantly than control study, though there is no relationship 25-hydroxyvitamin D with severity AD (p>0,05).

**Keyword**: Atopic dermatitis, 25-hydroxyvitamin D, severity atopic dermatitis

## A665 Thiol-Disulfide Balance in Children with Atopic Dermatitis

### Ilknur Bostanci^1^, Gulin Karacan^2^, Nazli Ercan^2^, Asuman Colak^2^, Murat Alisik^3^, Gulay Basarir^4^, Ozcan Erel^3^

#### ^1^Dr. Sami Ulus Obstetrics, Children’s Health and Diseases Training and Research Hospital, Turkey; ^2^Dr Sami Ulus Children Hospital; ^3^Ataturk Hospital; ^4^Mimar Sinan University

##### **Correspondence:** Ilknur Bostanci – Dr. Sami Ulus Obstetrics, Children’s Health and Diseases Training and Research Hospital, Turkey

**BACKGROUND:** Dynamic thiol disulphide homeostasis status has critical roles in antioxidant protection, detoxification, signal transduction, apoptosis, regulation of enzymatic activity and cellular signaling mechanisms and transcription factors.

In this study, we evaluated thiol disulphide level in infants with atopic dermatitis.

**METHOD:** Atopic dermatitis patients (31) and healthy children (30), under 2 years, were included. This study was prospective cross-sectional. Eosinophilia %, IgE levels and native thiol (SH), total thiol, disulfide (SS) SS / SH, SS / total SH, SH / total SH values were measured by the method described by Erel & Neseliogl (1). SPSS 15 (Statistical Package for Social Sciences) were analyzed using (p<0.05).

**RESULTS:** Atopic Dermatitis group of native thiol 322 587 ± 64.59 micro mol s - 1, total thiol 365 197 ± 66.19 micro mol s - 1 and disulfide 21:30 ± 8.81 micro mol L-1, the control group of native thiol 365 483 ± 63.77 micromol s - 1, total thiol 399 587 ± 65.12 micro mol s - 1 and disulfide levels were found to be 17:05 4.99μmol l- ± 1 (respectively p=0.012, 0.047, 0.025). A negative total thiol level between eosinophils (r = -0608) (p = 0.001) and duration of breastfeeding has been shown to be positively correlated (r = +0512) (p = 0.000).

**CONCLUSION:** Our study showed that the thiol-disulfide balance weakened in children with Atopic Dermatitis and is seen as part of the registration to excessive levels of oxidative balance. This study is the first in the literature. The results showed that it is important in ensuring the thiol- disulfide balance of breastfeeding. Furthermore, the thiol-disulfide balance in monitoring in the children with Atopic Dermatitis can be used as serological markers.

**KEYWORDS:** Atopic Dermatitis/Eczema; Atopic Dermatitis/Eczema: Pathophysiology; Clinical Research: Pediatric

**References**

1) Erel O, Neselioglu S: A novel and automated assay for thiol/disulphide homeostasis. Clinical Biochemistry 47 (2014) 326–332

## A666 Recurrent Mouth Ulsers Caused By Braces after Developing a Nickel Allergy in Children

### Ilknur Bostanci^1^, Yasemin Keskin^2^

#### ^1^Dr. Sami Ulus Obstetrics, Children’s Health and Diseases Training and Research Hospital, Turkey, ^2^Faculty of Dendistry, Ankara University

##### **Correspondence:** Ilknur Bostanci – Dr. Sami Ulus Obstetrics, Children’s Health and Diseases Training and Research Hospital, Turkey

6-year-old man was admitted because of recurrent ulcers in the mouth. Patients four months ago began using braces to the front dental. Nickel sensitivity was observed in the patch test. We emphasize the need to question the nickel allergy, before fitting the device to the mouth in children.

## A667 Anaphylactic Reaction to Famotidine with Pheniramine Hypersensitivity

### Ilkay Koca Kalkan

#### Kirikkale Yüksek Ihtisas Hospital, Turkey

**Background:** Antihistamines are widely used medications worldwide. They are indicated for the symptomatic treatment of allergic disorders (H_1_) and decreasing gastric acid production (H_2_). They are considered to have excellent safety. Extremely rare cases of famotidine-induced anaphylaxis have been documented and hypersensitivity reactions to alkylamine derivatives such as pheniramine are also extremely rare.

**Methods:** We report a 44 year old woman with no allergic background, who was admitted to ER complaining from swelling of the lips and tongue, tightening in the throat, dyspnea, generalized itching and urticaria; that started about an hour after taking medications composed of naproxen sodium and famotidine. Following her recovery, she was discharged with pheniramine. But her generalized itching and urticaria complains reoccurred 1-2 hours after taking a tablet. Then she remembered she had the same symptoms 4 years ago with the same tablet prescribed for her pruritus, but her family doctor refused to consider her symptoms in connection with pheniramine.

**Results:** The study was carried out 5 weeks after the last reaction. First oral provocation test (OPT) with naproxen was done and was negative. Then she had a negative skin prick test (SPT), but positive intradermal test (IDT) with famotidine at concentration of 1 mg/ml. Possible cross-reactivity with other H2-receptor antagonists was assessed. SPT for ranitidine and IDT with ranitidine (1 mg/ml) and nizatidine (1:1000 dilution) were positive. In order to find a safe alternative, skin tests and OPTs were done with PPIs (Pantoprazole, omeprazole, esomeprazole) and were all negative. Skin prick test and IDT with for pheniramine (1:1000 dilution) were positive. But OPT with chlorpheniramine, another alkylamine derivative, was negative.

**Conclusion:** To the best of our knowledge this is the first reported case of an anaphylactic reaction to famotidine with pheniramine hypersensitivity. The underlying mechanism of such a relationship isn’t well understood, but this could be also seen in the efficacy of H_2_ receptor antagonists in the treatment of urticaria. We also observed cross-reactivity with ranitidine and nizatidine, which have similar side chains to the ring structures. Type I hypersensitivity reaction may have been involved in this patient based on the clinical history, time till reaction onset and also skin test results were positive. Hypersensitivity to antihistamines seems to be very rare may be because they are undersuspected and underdiagnosed. Our case suggests hypersensitivity to antihistamines H1 and H2 can occur together, and it may be advisable to asses a possible cross-reactivity not only within the same antihistamine receptor group but also between groups.

## A668 Novel Transcriptomic and Immunoproteotomic Approaches in Identifying Cross-Reactive Allergens Between Crustacean and Molluscs

### Andreas/Ludwig Lopata, Kyall Zenger, Roni Nugraha, Sandip Kamath

#### James Cook University, Australia

##### **Correspondence:** Andreas/Ludwig Lopata – James Cook University, Australia

**Background:** Utilizing crude protein extracts is currently inadequate to identify allergenic proteins in shellfish. Currently, only three proteins – tropomyosin, arginine kinase, and paramyosin – have been fully identified and characterized as potential cross-reactive allergens between crustacean and mollusks. Bioinformatics analysis could provide a powerful and versatile tool for the in-depth molecular characterization of biological species with unknown allergenicity. Bioinformatics approaches have recently been used to identify putative allergens in rice, chickpea and Johnson grass pollen. Therefore this study was aimed to identify putative and cross-reactive allergens using *in-silico* analysis of the transcriptome from the mollusk Pacific oyster (*Crassostrea gigas*).

**Methods:** Several allergen sequences related to shellfish allergy including from Black tiger prawn, lobster and abalone were documented from different databases. TBLASTN analysis was performed on these sequences against the genomic database of Pacific oyster to retrieve putative and cross-reactive allergen sequence. The retrieved sequences were used to generate phylogenetic trees using the Neighbor joining method in Mega6 software, after association using the MUSCLE multiple alignment program. Following this, structure model of the proteins were built using Chimera 1.9 program. In addition whole protein extracts where analysed by mass spectroscopy as well as IgE binding protein from shellfish allergic patients.

**Result:** Based on amino acid sequence similarity, cross-reactive allergens and several putative allergen genes were identified after detailed *in silico*analysis of the genomic data of Pacific oyster. Some of the putative oyster allergens demonstrated the potential to cross-react to crustacean allergens, based on conserved domains and similar structural features. This method also revealed the presence of various isoforms of the oyster allergens. The phylogenetic trees of six different allergens demonstrate that the Pacific oyster clusters with the corresponding allergens from other mollusk, however is grouped distinct separately from crustacean allergens.

**Conclusion:** Bioinformatics approaches reveal several putative and cross reactive allergens that have not been fully elucidated using less sensitive immuno-chemical IgE-based methods. These findings are of importance for the development of specific allergy diagnostics for shellfish allergens. *In-silico* approaches, in combination with recombinant allergen generation, will be an eminent method for the comprehensive assesment of allergenicity and opens an additional path for efficient allergy diagnostics and immunotherapeutics.

## A669 Pollen Season and Climate Change in the Continental United States (CONUS)

### Leonard Bielory, Panos Georgopoulos, Yong Zhang, Wheat Mi, Ting Cai

#### Rutgers University, USA

##### **Correspondence:** Leonard Bielory – Rutgers University, USA

**Background:** Climate change (CC) is expected to alter allergenic pollen season pollination for trees, weeds and grasses and potentially increase occurrence of allergic airway disease.

**Objective:** We sought to examine the spatiotemporal patterns of change of weeds (ragweed, mugwort), trees (birch, oak), grasses in multiple climate regions, and their relationships with the changing climate in the contiguous US over time from the year 1994 to 2010.

**Methods:** Regional assessments (South-S, Southeast-SE, Southwest-SW, Central-C, West-W, Northeast-NE, Eastnorthcentral-ENC, Westnorthcentral-WNC and Northwest-NW) for land area coverage, pollen season timing, hourly pollen concentrations, due to CC were obtained using statistical analyses and deterministic simulation based on observed and simulated models across the CONUS.

**Results:** The allergenic pollen seasons of representative trees, weeds and grasses during the 2000s across the CONUS have been observed to start 3.0 (95% Confidence Interval [CI], 1.1-4.9) days earlier on average than in the 1990s. The average peak value and annual total of daily counted airborne pollen have increased by 42.4% (95% CI, 21.9%- 62.9%) and 46.0% (95% CI, 21.5%-70.5%), respectively. Changes of ragweed pollen season timing and levels were identified as functions of latitude, and associated with changes of Growing Degree Days, Frost Free Days and precipitation.

**Conclusion:** These changes are likely due to recent climate change and particularly the enhanced warming and precipitation at higher latitudes in the CONUS. The observed pollen season start date, season length and airborne pollen level could be correctly predicted using Bayesian and machine learning models based on the locally observed meteorological factors.

## A670 Differences of Change in Der p IgG4 and CD4+CD25+FoxP3+ Treg Cells Between Sublingual and Subcutaneous Immunotherapy with House Dust Mite in Chinese Patients with Allergic Rhinitis

### MO Xian^1^, Jing Li^2^, Mulin Feng^2^

#### ^1^State Key Laboratory of Respiratory Disease, the First Affiliated Hospital, Guangzhou Medical University, China; ^2^Guangzhou Medical University

##### **Correspondence:** MO Xian – State Key Laboratory of Respiratory Disease, the First Affiliated Hospital, Guangzhou Medical University, China

**Background** Specific subcutaneous immunotherapy (SCIT) with house dust mite (HDM) has been shown to improve clinical symptoms in patients with allergic rhinitis and asthma in China, while less data regarding efficacy, safety and immunological mechanisms of specific sublingual immunotherapy (SLIT) in allergic patients. The aim of our study was to evaluate the efficacy, immunological mechanisms of SLIT HDM compared to SCIT HDM in rhinitis patients with HDM during 1 year of treatment.

**Method** Recruitment took place from November 2009 to September 2010. The study enrolled 67 patients aged 5. ‘55 years old with moderate-severe HDM induced allergic rhinitis with or without asthma. They were randomized (2:2:1) in three groups (SLIT group, n=27; SCIT group, n=26; Placebo, n=14, following the same schedule as SLIT group). The allergic rhinitis and asthma symptom and medication score collected from patient diary cards (from baseline to 1 year) were used for assessment of efficacy. Visual analogue score (VAS) were collected and skin prick tests, total-specific IgE were performed at baseline and 12 months after treatment. In addition, patients underwent Der p IgG4 and CD4+CD25+FoxP3+ Treg cells measurements.

**Results** SCIT groups had a significant improvement for the change from baseline in mean total rhinitis symptom score compared with placebo after 1 year therapy (*P*=0.015), but between SLIT and placebo groups there were no significant difference(*P*=0.060). Both two groups had a significant improvement in mean rhinitis medication score compared with placebo (*P*<0.05), and the analysis of rhinitis VAS score change from baseline showed the same results. The mean asthma symptom or medication score did not show significant difference among the three groups. There was a trend toward up-regulation in the rate of CD4+CD25+FoxP3+ Treg cells from baseline to 1 year in both SCIT and SLIT subjects compared with Placebo group(*P*<0.05). The level of Der p IgG4 showed a significant increase in both SLIT and SCIT compared with placebo (*P*<0.05), furthermore, the mean level of Der p IgG4 of SCIT group was almost thirty times higher than SLIT group after 1 year therapy (*P*<0.05).

**Conclusion** Both SCIT and SLIT had the efficacy in patients with moderate-severe HDM sensitized allergic rhinitis after 1 year of treatment, and SCIT also afford significant therapeutic benefits with respect to rhinitis symptom score. Serum Der p IgG4 and CD4+CD25+ FoxP3+ Treg cells may play roles in modulate immunoresponse to specific immunotherapy, but their relationship between clinical efficacy needs further study.

